# Exploring Treatment by Covariate Interactions Using Subgroup Analysis and Meta-Regression in Cochrane Reviews: A Review of Recent Practice

**DOI:** 10.1371/journal.pone.0128804

**Published:** 2015-06-01

**Authors:** Sarah Donegan, Lisa Williams, Sofia Dias, Catrin Tudur-Smith, Nicky Welton

**Affiliations:** 1 Department of Biostatistics, University of Liverpool, Liverpool, United Kingdom; 2 School of Social and Community Medicine, University of Bristol, Bristol, United Kingdom; Aligarh Muslim University, INDIA

## Abstract

**Background:**

Treatment by covariate interactions can be explored in reviews using interaction analyses (e.g., subgroup analysis). Such analyses can provide information on how the covariate modifies the treatment effect and is an important methodological approach for personalising medicine. Guidance exists regarding how to apply such analyses but little is known about whether authors follow the guidance.

**Methods:**

Using published recommendations, we developed criteria to assess how well interaction analyses were designed, applied, interpreted, and reported. The Cochrane Database of Systematic Reviews was searched (8th August 2013). We applied the criteria to the most recently published review, with an accessible protocol, for each Cochrane Review Group. We excluded review updates, diagnostic test accuracy reviews, withdrawn reviews, and overviews of reviews. Data were summarised regarding reviews, covariates, and analyses.

**Results:**

Each of the 52 included reviews planned or did interaction analyses; 51 reviews (98%) planned analyses and 33 reviews (63%) applied analyses. The type of analysis planned and the type subsequently applied (e.g., sensitivity or subgroup analysis) was discrepant in 24 reviews (46%). No review reported how or why each covariate had been chosen; 22 reviews (42%) did state each covariate a priori in the protocol but no review identified each post-hoc covariate as such. Eleven reviews (21%) mentioned five covariates or less. One review reported planning to use a method to detect interactions (i.e., interaction test) for each covariate; another review reported applying the method for each covariate. Regarding interpretation, only one review reported whether an interaction was detected for each covariate and no review discussed the importance, or plausibility, of the results, or the possibility of confounding for each covariate.

**Conclusions:**

Interaction analyses in Cochrane Reviews can be substantially improved. The proposed criteria can be used to help guide the reporting and conduct of analyses.

## Introduction

Most systematic reviews use a statistical technique called meta-analysis to combine individual study results to obtain a single pooled treatment effect estimate on which to base conclusions. Methods to assess the homogeneity assumption (i.e. similarity of treatment effects from trials in the meta-analysis) that underlies meta-analysis are well established [1]. If methods detect heterogeneity, it may be inappropriate to draw conclusions from the pooled treatment effect estimate. Instead, possible causes of heterogeneity can be explored using interaction analysis (i.e. subgroup analysis or meta-regression techniques) [1]. If heterogeneity is reduced by including the interaction, then at least some of the heterogeneity has been explained by the interaction. In the extreme case, all variability will be explained by the interaction and valid inferences can be drawn from the results of the interaction analysis.

Furthermore, using a stratified (or personalised) medicine approach, clinicians may present results in a review stratified by patient group because the relative treatment effect is believed to be inconsistent across patients groups; such results are produced by interaction analyses. For example, stratifying the results by surgical procedure (e.g. amputation, breast surgery) in a review comparing drugs for the prevention of pain after surgery [2]. As such, interaction analyses provide valuable information regarding how the covariate modifies the treatment effect, for instance, they can answer the questions: is the same treatment effect applicable to all patients? Is the treatment effect for amputation greater than for breast surgery? Is ketamine more effective for amputation and gabapentin more effective for breast surgery?

Substantial methodological guidance exists regarding how to apply such analyses in reviews [1,3–10]. Gagnier *et al*. [3] summarised published advice for investigating clinical heterogeneity in reviews. Key recommendations were:

- planned investigations of clinical heterogeneity should be made explicit in the protocol of the review;
- clinical experts should be included on the review team;
- a set of clinical covariates should be chosen considering variables from the participant-level, intervention-level, outcome-level, research setting, or others unique to the research question;
- covariates should have a clear scientific rationale;
- there should be a sufficient number of trials per covariate;
- and results of any such investigations should be interpreted with caution.

Gagnier *et al*. [4] went on to develop recommendations for investigating clinical heterogeneity in reviews using a Delphi technique involving methodological experts. Recommendations were grouped under the following themes: review team, planning, rationale, types of clinical variables to consider, role of statistical heterogeneity, plotting and visual aids, dealing with outliers, number of investigations to perform and variables to explore, the use of aggregate data (AD) vs. individual patient data (IPD), the role of the best evidence syntheses, statistical methods, interpretation of findings, and reporting.

However, there is limited research detailing how review authors currently explore interactions in practice. Higgins *et al*. [11] collated published recommendations for dealing with heterogeneity in reviews, investigated how review authors address heterogeneity in 39 Cochrane Reviews (i.e. systematic reviews of human health interventions prepared by the Cochrane Collaboration), and assessed whether recommendations were followed in practice. As part of this research, Higgins *et al*. [11] reported whether covariates were pre-specified in the protocol, whether post-hoc covariates were identified, and the number of studies in the reviews. We update and build on this research investigating interaction analyses in greater depth.

In this article, we devise criteria to assess how well interaction analyses were designed, applied, interpreted, and reported; we review recently published Cochrane Reviews to establish how well such analyses are carried out; and make recommendations regarding how such analyses can be improved based on our findings. To our knowledge no previous research has explored the design, interpretation, and reporting of published analyses, or explored the application of interaction analyses so thoroughly. We hope that this review will help readers of reviews to detect any flaws in interaction analyses, and help review authors plan, carry out, report and interpret interaction analyses.

## Methods

### Devising assessment criteria

Criteria were compiled by reading recommendations given in the Cochrane Handbook [1], other methodological articles [3–10], and our own experience. Ideally, we would expect 100% of Cochrane Reviews to satisfy the criteria that were based on recommendations given in the Cochrane handbook (criterion 1, 2, 3, 4, 8, 13, 14, 17, 18, 19, 20 in Table 1) and most reviews would also satisfy the remaining criteria. The criteria were pre-piloted by SDo using a sample of ten reviews, and then the criteria were then refined to minimise subjectivity, ensure the review was achievable, and avoid the need for clinical expertise in a wide range of areas. The final criteria focused on six components: choosing covariates, considering covariate data, analysing interactions, detecting interactions, reporting interaction results, and interpreting interaction results (Table 1). For each criterion, reviews were classified as yes (representing good practice), no (representing alternative practice), or unclear.

**Table 1:** Summary of results of assessment of interactions.

	Number of reviews (%)		
Criterion	Yes	No	Not applicable
**Choosing covariates**			
(1) Was external evidence (e.g. other reviews or studies) reported to be used to choose each covariate?	0 (0)	52 (100)	-
(2) Was rationale given for choosing each covariate as a potential treatment effect modifying covariate?	0 (0)	52 (100)	-
(3) Was each covariate reported a priori (i.e. in the protocol)?	22 (42)	30 (58)	-
(4) Was each post-hoc chosen covariate (i.e. in the review but not in the protocol) labelled as such?	0 (0)	30 (100)	22
(5) Were a limited number of covariates (i.e. <6) reported?	11 (21)	41 (79)	-
**Considering covariate data**			
(6) Was missing covariate data reported to be sought and planned to be sought?	0 (0)	52 (100)	-
(7) For study-level covariates, were AD analyses reported to be planned and, if interaction analyses were applied, actually carried out?	50 (96)	2 (4)	0 (0)
(8) If AD analyses were reported to be performed, was there reported to be at least 10 trials in the analysis for each covariate (for the outcome)?	5 (15)	28 (85)	19
(9) For patient-level covariates, were IPD analyses reported to be planned and, if interaction analyses were applied, actually carried out?	0 (0)	6 (100)	6
(10) Was justification given for categorising each continuous covariate that was categorised?	0 (0)	41 (100)	11: 8 no continuous covariates; 2 unclear if covariates were categorical or continuous; 1 unclear if continuous covariates were categorised.
(11) Were the categories reported for each categorised covariate?	6 (12)	46 (88)	0 (0)
(12) Was justification given for the categories chosen for each categorised covariate?	0 (0)	52 (100)	0 (0)
**Analysing interactions**			
(13) Was interaction analysis reported to be planned?	51 (98)	1 (2)	-
**Detecting interactions**			
(14) Were methods to detect interactions reported to be planned and, if interaction analyses were applied, actually used for each covariate?	1 (2)	51 (98)	-
**Reporting interaction results**			
(15) Were results from interaction analysis reported for each covariate (for the outcome)?	30 (91)	3 (9)	19
(16) Were results from methods to detect interactions reported for each covariate (for the outcome)?	4 (12)	29 (88)	19
**Interpreting interaction results**			
(17) Was it reported whether or not an interaction was detected for each analysed covariate (for the outcome)?	1 (3)	32 (97)	19
(18) Was the importance of the interaction or lack of interaction discussed for each analysed covariate (for the outcome)?	0 (0)	33 (100)	19
(19) Was the plausibility of the interaction or lack of interaction discussed for each analysed covariate (for the outcome)?	0 (0)	33 (100)	19
(20) Was the possibility of confounding discussed for each analysed covariate (for the outcome)?	0 (0)	33 (100)	19
(21) Was the covariate distribution discussed for each analysed covariate (for the outcome)?	13 (39)	20 (61)	19

^(1)^ Yes: external evidence (i.e. other reviews or studies) was reported to have been used to choose each covariate in the methods of the protocol and/or review. No: no external evidence was reported to have been used for choosing each covariate reported in the methods of the protocol and/or review.

^(2)^ Yes: rationale given for choosing each covariate in the methods of the protocol and/or review. No: rationale not given for choosing each covariate in the methods of the protocol and/or review.

^(3)^ Yes: each covariate stated in the methods of the protocol. No: at least one covariate was not stated in the methods of the protocol but was reported in the methods or results of the review.

^(4)^ Yes: each covariate chosen post-hoc (i.e. in the review but not in the protocol) was identified as a post-hoc covariate in the methods or results of the review. No: at least one covariate chosen post-hoc was not identified as a post-hoc covariate in the methods or results of the review. NA: no covariate was chosen post-hoc.

^(5)^ Yes: less than six covariates reported in the methods and/or results of the protocol and/or review; No: six or more covariates reported in the methods and/or results of the protocol and/or review.

^(6)^ Yes: missing covariate data was reported to be planned to be sought in the methods of the protocol and reported to be sought in the methods of the review. No: missing covariate data was not reported to be sought in the methods of the review and/or planned to be sought in the methods of the protocol. (e.g. ‘data’ was reported to be sought or planned to be sought, or no method was reported in the methods in the protocol and/or review).

^(7)^ Yes: for reviews including study-level covariates (i.e. intervention, methodological, outcome-related, or other covariates), AD analyses were reported to be planned in the methods of the protocol and, if interaction analyses were carried out, reported to be carried out in the methods and/or results of the review. No: for reviews including study-level covariates, IPD analyses were reported to be planned in the methods of the protocol and/or carried out in the methods and/or results of the review. NA: no study-level covariates. We presumed AD analyses were planned/or done if there was no mention of IPD.

^(8)^ Yes: for reviews that carried out AD analyses for the outcome, the number of trials (as calculated using the method described in S3 Table) in the interaction analysis for each covariate is at least ten for the outcome. No: for reviews that carried out AD analyses for the outcome, the number of trials in the interaction analysis for each covariate is less than ten for the outcome. NA: no AD interaction analyses in the review for the outcome. We presumed AD analyses were done if there was no mention of IPD, and results from interaction analyses were reported or it was reported that interaction analyses were carried out.

^(9)^ Yes: for reviews including patient-level covariates (i.e. patient covariates), IPD analyses were reported to be planned in the methods of the protocol and, if interaction analyses were carried out, reported to be carried out in the methods and/or results of the review. No: for reviews including patient-level covariates, AD analyses were reported to be planned in the methods of the protocol and/or carried out in the methods and/or results of the review. NA: no study-level covariates. We presumed AD analyses were planned/or done if there was no mention of IPD. We presumed AD analyses were carried out if there was no mention of IPD, and results from interaction analyses were reported or it was reported that interaction analyses were carried out.

^(10)^ Yes: justification was given for categorising each continuous covariate in the methods and/or results of the protocol and/or review. No: no justification was given for categorising each continuous covariate in the methods and/or results of the protocol and/or review. NA: no continuous covariates. We presumed a continuous covariate was categorised when categories were reported in the methods and/or results of the protocol and/or review, or when subgroup or sensitivity analysis was reported in the methods and/or results of the protocol and/or review.

^(11)^ Yes: categories were reported for each categorised covariate in the methods and/or results of the protocol and/or review. No: categories were not reported for each categorised covariate in the methods and/or results of the protocol and/or review. NA: no categorical covariates or categorised continuous covariates.

^(12)^ Yes: justification was given for the categories chosen for each categorised covariate in the methods and/or results of the protocol and/or review. No: justification was not given for the categories chosen for each categorised covariate in the methods and/or results of the protocol and/or review. NA: no categorical covariates or categorised continuous covariates.

^(13)^ Yes: interaction analysis (i.e. stratification/subgroup analysis, sensitivity analysis, or meta-regression) was reported to be planned in the methods of the protocol. No: interaction analysis not reported to be planned in the methods of the protocol.

^(14)^ Yes: a methods to detect interaction (e.g. comparing the overlap of confidence intervals across subgroups, the test for subgroup differences, and/or I square statistic, based on the size and significance of regression coefficients in meta-regression and/or reduction in between trial variance) was reported to be planned in the methods of the protocol and, if interaction analyses were carried out, reported to be used in the methods of the review. No: methods to detect interactions not reported to be planned in the methods of the protocol, or if interaction analyses were carried out, not reported to be used in the methods of the review.

^(15)^ Yes: reported statistical results from the interaction analysis for each analysed covariate (for the outcome) in the results of the review. No: did not report statistical results from the interaction analysis for each analysed covariate (for the outcome) in the results of the review (e.g. non-statistical statement presented in the text). NA: no interaction analyses carried out in the review for the outcome. We presumed interaction analysis was carried out for a particular covariate only when results were presented in the review for that covariate or when the review specifically stated they carried out interaction analysis for that covariate for the outcome.

^(16)^ Yes: reported results from the method to detect interactions for each analysed covariate (for the outcome) in the results of the review. No: did not report results from the method to detect interactions for each analysed covariate (for the outcome) in the results of the review. NA: no interaction analyses carried out in the review for the outcome.

^(17)^ Yes: reported whether or not an interaction was detected for each analysed covariate (for the outcome) in the results of the review. No: not reported whether or not an interaction was detected for each analysed covariate (for the outcome) in the results of the review. NA: no interaction analyses carried out in the review for the outcome.

^(18)^ Yes: explicitly discussed the importance of the interaction or lack of interaction for each analysed covariate (for the outcome) in the results and/or discussion of the review. No: did not explicitly discuss the importance of the interaction or lack of interaction for each analysed covariate (for the outcome) in the results and/or discussion of the review. NA: no interaction analyses carried out in the review for the outcome.

^(19)^ Yes: explicitly discussed the plausibility of the interaction or lack of interaction for each analysed covariate (for the outcome) in the results and/or discussion of the review. No: did not explicitly discuss the plausibility of the interaction or lack of interaction for each analysed covariate (for the outcome) in the results and/or discussion of the review. NA: no interaction analyses carried out in the review for the outcome.

^(20)^ Yes: explicitly discussed the possibility of confounding for each analysed covariate (for the outcome) in the results and/or discussion of the review. No: did not explicitly discuss the possibility of confounding for each analysed covariate (for the outcome) in the results and/or discussion of the review. NA: no interaction analyses carried out in the review for the outcome.

^(21)^ Yes: explicitly discussed the covariate distribution for each analysed covariate (for the outcome) in the results and/or discussion of the review. No: did not explicitly discus the covariate distribution for each analysed covariate (for the outcome) in the results and/or discussion of the review. NA: no interaction analyses carried out in the review for the outcome.

For choosing covariates, good practice would be: reporting using external evidence to choose covariates; giving the rationale for choosing covariates; reporting covariates a priori; labelling any covariate chosen post-hoc as such; and limiting the number of covariates examined to less than six. For considering covariate data, good practice would be, reporting that missing covariate data was sought and planned to be sought; planning to analyse and analysing IPD when exploring patient-level covariates; planning to analyse and analysing AD for study-level covariates; applying AD analyses only when at least 10 trials contribute to the analysis [1]; providing justification for categorising continuous covariates; reporting the categories of categorised covariates and justifying the categories. For analysing interactions, reporting whether interaction analysis (i.e. subgroup analysis) was planned is good practice. For detecting interactions, reporting whether a method to detect interactions (i.e. to assess differences in the treatment effects from different subgroups, e.g. test for subgroup differences) was planned and used is good practice. For reporting interaction results, reporting statistical results from the interaction analysis (e.g. treatment effects) and results from the method to detect interactions (e.g. p-value from the test for subgroup differences) would indicate good practice. For interpreting interaction results, good practice would be reporting, whether or not an interaction was detected; the clinical importance and biological plausibility of the results; the possibility of confounding; and the covariate distribution.

### The review of Cochrane Reviews

#### Eligibility criteria

For the 52 Cochrane Review Groups (excluding the Cochrane Methodology Review Group), we included the most recently published review for which the protocol was accessible from the Cochrane Database of Systematic Reviews (CDSR), and there was only one version of the review published in the CDSR. We excluded withdrawn reviews, overview of reviews, and diagnostic test accuracy reviews. We included only the most recently published review from each review group because each group focuses on a specific topic area (e.g. Airways Group) therefore we could obtain a description of current practice across a wide range of clinical conditions, whilst keeping the task manageable. We included only Cochrane Reviews because the published protocol is usually easily accessible allowing us to assess how well interaction analyses were planned and to compare pre-specified and performed analyses.

#### Search strategy

CDSR was searched on 8^th^ August 2013.

#### Review selection

SDo and LW independently assessed the eligibility of reviews using an assessment form. The ‘New Reviews’ in the ‘Table of Contents’ and the ‘Archives’ of the CDSR were assessed for eligibility (for reviews published between Dec 2012-Aug 2013). If no eligible review had been published during this time period for a particular review group, a list of all reviews published by the group was accessed in CDSR and each review of that particular group was assessed for eligibility. The results of the independent assessments were compared and differences were resolved by discussion between authors.

#### Data extraction

Using a data extraction form, SDo extracted data from the review and the protocol. The form was pre-piloted using a sample of ten reviews. LW independently extracted data for a sample of 17 (33%) reviews and discrepancies were discussed between authors.

Information was extracted regarding: review characteristics; covariate characteristics; and the design, analysis, reporting, and interpretation of interactions. See S1–S3 Tables for the types of information that were extracted.

For each review, we extracted information with respect to the first primary outcome (or the first listed outcome where no primary outcome was specified) reported in the methods section of the review document. When the outcome was described as one outcome in the methods (e.g. ‘arm and neck pain’) but reported as multiple outcomes in the results (e.g. ‘arm pain’ and ‘neck pain’), data for each relevant outcome listed in the results were extracted.

For each review, we extracted information for each covariate for which interaction analysis (i.e. stratification/subgroup analysis, sensitivity analysis, or meta-regression) was planned and/or done as reported in the protocol and/or review. See S1 File for the definitions used when we extracted data. We extracted data regarding sensitivity analysis, even though we would not recommend this approach for exploring interactions, because review authors sometimes incorrectly use the terms sensitivity analysis and subgroup analysis interchangeably.

#### Data analysis and assessment

The characteristics of reviews and covariates were tabulated and summarised.

SDo classified the type of intervention (e.g. drugs), type of covariate (i.e. patient (e.g. gender), intervention (e.g. dose), methodological (e.g. blinding), outcome-related (e.g. outcome definition), or other (e.g. publication year)) as suggested by Gagnier *et al*. [4], and type of analysis according to the S1 File (determined based on the covariate categories reported, results presented, or the use of words such as ‘removal’, ‘excluding’, ‘comparing’).

SDo assessed the interaction analyses in each review using the devised criteria and an assessment form. The percentage of reviews was calculated for each classification for each criterion. As additional analyses, where possible for a particular criterion (e.g. for ‘was external evidence reported to be used to choose each covariate?’), we presented the percentage of covariates that ‘met’ a particular criterion in each review (e.g. the percentage of covariates for which external evidence was reported) and calculated summary statistics (i.e. median, IQR, and range) across the percentages.

## Results of the Review of Cochrane Reviews

### Description of reviews

The 52 reviews were published between May 2012-Aug 2013 (see S2 File for the references). Forty-six reviews (88%) included randomised controlled trials; six reviews (12%) also included non-randomised studies. The reviews varied greatly in terms of participants and focussed on the following intervention types: drugs (26 reviews, 50%); herbal medicines (two reviews, 4%); non-pharmacological (12 reviews, 23%); psychological, psychosocial or psychotherapy (three reviews, 6%); surgical (six reviews, 12%); techniques (two reviews, 4%); and timing of the intervention (one review, 2%). All but one review reported at least one primary outcome.

The number of trials that reported the outcome in each review was low overall (median 4 trials, IQR 1–6 trials); and the number of patients also appeared limited (median 394 patients, IQR 45–1,467 patients). Notably, 21 reviews (40%) included no trials or one trial.

One review (2%) planned to collect IPD but in the review, they reported that it was not possible; 51 reviews (98%) did not report planning to use IPD and presumably planned to use AD, of which, one review (2%) reported estimating a hazard ratio from IPD of one trial in the review.

S1 and S4 Tables provide further details.

### Description of covariates

Each review reported at least one covariate; 33 reviews (63%) analysed at least one covariate. The percentage of reported covariates that were analysed per review was generally low (median 18%, IQR 0–43%).

Forty-six reviews (88%) reported patient-level covariates and all 52 reviews reported study-level covariates (45 reviews (87%) reported intervention covariates; 19 reviews (37%) reported outcome-related covariates; 49 reviews (94%) reported methodological covariates; and four reviews (8%) reported other covariates). See Table 2 for the covariates.

**Table 2:** Summary of covariates.

Covariate summary	Number of reviews	Number of covariates
**Patient covariates**.		
**All patient covariates**.	**46**	**109**
Demographics.	20	38
Diagnostic criteria.	1	1
Disease characteristics.	38	64
‘Inclusion criteria’.	2	2
Patient characteristics.	1	1
Setting or country.	3	3
**Intervention covariates**.		
**All intervention covariates**.	**45**	**126**
Additional interventions and type of intervention or control.	1	1
Additional interventions.	8	11
Dose.	10	11
Duration of intervention.	11	11
Intervention intensity.	2	2
Previous intervention.	1	1
Route of administration.	5	5
Time to therapeutic range (good/bad quality).	1	1
Timing of intervention.	3	4
Type of intervention or control and dose.	2	2
Type of intervention or control.	38	77
**Outcome-related covariates**.		
**All outcome-related covariates**.	**19**	**24**
Adjusted treatment effect.	1	1
Discontinued the treatment because of toxicity.	1	1
Measurement scale.	1	1
Outcome definition.	1	1
Skewed data.	1	1
Studies that report other abstinence outcomes.	1	1
Studies which report changes in understanding or values or choices made at group level only.	1	1
Time point.	16	17
**Methodological covariates**.		
**All methodological covariates**.	**49**	**145**
A combination of quality indicators.	1	1
Allocation concealment.	18	18
Blinding.	19	20
Definition of follow up.	1	1
Funding.	2	2
‘Implied randomisation’.	1	1
Incomplete outcome data/follow up.	10	11
‘Intention to treat’.	8	8
Length of follow up.	6	6
Length of study.	1	1
Publication language.	2	2
Publication status.	11	11
Quality of diagnostic criteria.	1	2
Quality.	14	16
Random generation or concealment.	1	1
Random generation.	7	7
Randomisation.	2	2
Risk of bias.	19	20
‘Second randomisation’.	1	1
Trial design.	5	5
Trial size.	6	9
**Other covariates**.		
**All other covariates**.	**4**	**5**
Effect size.	1	2
Outlying results.	2	2
Year of publication.	1	1

Every review reported categorical covariates; 42 reviews (81%) reported continuous covariates; and six reviews (12%) reported covariates for which it was unclear whether covariates were continuous or categorical. Of the 42 reviews that reported continuous covariates, 41 reviews (98%) reported continuous covariates that were categorised; one review (2%) reported continuous covariates that were not categorised; and six reviews (14%) reported continuous covariates for which it was unclear whether the covariate was categorised.

S2 Table provides details of the covariates reported in each review; S3 Table displays further details of the analysed covariates; and S4 Table provides summary information.

### Assessment of interaction analyses

Each of the reviews planned, or did, interaction analyses, therefore all reviews were included in the assessment of choosing covariates, considering covariate data, analysing interactions, and detecting interactions. The 33 reviews (63%) that carried out interaction analyses were included in the assessment of reporting and interpreting interaction results.

A summary of the assessment results is provided in Table 1 and presented below. S5 Table displays the assessment results for each review. S6–S11 Tables displays the percentage of covariates in each review that met a particular criterion and summary statistics across reviews.

#### Choosing covariates

*Was external evidence (e.g. other reviews or studies) reported to be used to choose each covariate?*

No review reported using external evidence to choose each covariate. No review described how they chose any covariate.

*Was rationale given for choosing each covariate as a potential treatment effect modifying covariate?*

No review provided an explanation of why every covariate was a potential treatment effect modifying covariate. However, 16 reviews (31%) gave the rationale for at least one (but not all) of the covariates in the review. The percentage of covariates with rationale reported in each review was very low overall (median 0%, IQR 0–9%). See S12 Table for the reported rationale.

*Was each covariate reported a priori (i.e. in the protocol)?*

All but one review reported at least one covariate a priori but only 22 reviews (42%) reported all of the covariates a priori. The percentage of covariates reported a priori in each review was generally reasonably high (median 88%, IQR 80–100%).

*Was each post-hoc chosen covariate (i.e. in the review but not in the protocol) labelled as such?*

Thirty reviews reported at least one post-hoc covariate. None of these had labelled all of the covariates as ‘post-hoc’ selected but four reviews (13%) did so for at least one of the covariates. Generally, the percentage of covariates identified as ‘post-hoc’ in each review was low (median 13%, IQR 0–0%).

*Were a limited number of covariates (i.e. <6) reported?*

Eleven reviews (21%) reported less than six covariates in the methods or results of the protocol and/or review. The number of reported covariates per review was fairly high overall (median 8, IQR 6–11); whereas, the average number of analysed covariates per review was low (median 1, IQR 0–3).

#### Considering covariate data

*Was missing covariate data reported to be sought and planned to be sought?*

No review explicitly reported that missing covariate data was planned to be sought and was sought. One review (2%) had not explicitly reported planning to seek missing covariate data but did report that it was done.

However, of the 52 included reviews, 37 reviews (71%) reported that some type of missing data (not explicitly reporting covariate data) was planned to be sought and was sought; one review (2%) reported that some type of missing data was planned to be sought but specifically reported that missing covariate data was sought; seven reviews (13%) reported planning to retrieve data but did not report whether this was done; two reviews (4%) reported planning to retrieve data but reported this wasn’t done; one review (2%) reported that data was sought but did not report that it was planned; and four reviews (8%) did not report whether any missing data was sought or planned to be sought.

*For study-level covariates, were AD analyses reported to be planned and, if interaction analyses were applied, actually carried out?*

52 reviews reported study-level covariates, of which, 50 reviews (96%) reported planning to analyse AD, rather than IPD, and actually doing so if interaction analyses were applied. Of the remaining two reviews, one review did not report covariates in the protocol and another review planned to collect IPD but did AD analyses.

*If AD analyses were reported to be performed, was there reported to be at least 10 trials in the analysis for each covariate (for the outcome)?*

Thirty-three reviews actually carried out AD analyses. Five reviews (15%) included 10 or more trials in the interaction analysis for each analysed covariate. However, seven reviews (21%) did so for at least one covariate (but not all covariates).

The average number of trials included in covariate analyses per review seemed limited (median 4, IQR 3–5).

*For patient-level covariates, were IPD analyses reported to be planned and, if interaction analyses were applied, actually carried out?*

Of the 46 reviews that reported patient-level covariates, no review reported planning to analyse IPD, rather than AD, and actually doing so if interaction analyses were applied. Instead, AD analyses were planned and carried out.

*Was justification given for categorising each continuous covariate that was categorised?*

Forty-one reviews reported at least one continuous covariate that had been categorised, of which, no review justified why any covariate was categorised.

*Were the categories reported for each categorised covariate?*

Every review included at least one categorised covariate (i.e. a categorical covariate or a continuous covariate that was categorised). Fifty-one reviews (98%) reported the categories for at least one covariate in the review but only six reviews (12%) reported the categories for every covariate. The percentage of covariates with categories reported in each review was reasonably high overall (median 76%, IQR 60–100).

*Was justification given for the categories chosen for each categorised covariate?*

No review provided justification for the covariate categories for every covariate but seven reviews (13%) gave justification for at least one covariate in the review. Overall, the percentage of covariates with justification reported in each review was very low (median 0%, IQR 0–0%). See S13 Table for the justification.

#### Analysing interactions

*Was interaction analysis reported to be planned?*

Fifty-one reviews (98%) reported that interaction analysis was planned (of which 32 reviews carried out interaction analyses). Another review (2%) did not report planned interaction analyses but did apply interaction analysis in the review. Reasons given for not doing interaction analysis were: not reported; insufficient data; no meta-analysis applied; or studies did not provide the required data.

The reported planned types of interaction analysis included subgroup analysis (48 reviews, 94%), sensitivity analysis (46 reviews, 90%), meta-regression (seven reviews, 14%), or an unnamed analysis (i.e. reported stratifying the analyses in some way but did not report the terms ‘subgroup analysis’, ‘sensitivity analysis’ or ‘meta-regression’) (23 reviews, 45%) or ‘subgroup analysis or meta-regression’ (one review, 2%). The reported applied types of interaction analysis included subgroup analysis (19 reviews, 58%), sensitivity analysis (10 reviews, 30%), meta-regression (one review, 3%), or an unnamed analysis (24 reviews, 73%).

Of the 33 reviews that applied interaction analyses, there were discrepancies between the reported planned and applied type of analysis for a particular covariate in four reviews (12%): one review planned sensitivity analysis but applied an unnamed analysis (i.e. did not use the term ‘sensitivity analysis’ in the review); two reviews planned unnamed analyses (i.e. did not use the term ‘subgroup analysis’ in the protocol) but reported applying subgroup analyses; one review planned to apply either subgroup analysis or meta-regression but applied both. The reviews did not report the reason for the difference between protocol and review.

For the 51 reviews that planned interaction analyses, when we compared the type of interaction analysis reported in the protocol with the type we judged it to be based on the S1 File, in 29 reviews (56%) there was a discrepancy: six reviews reported sensitivity analysis rather than subgroup analysis; one review reported subgroup analyses rather than sensitivity analysis; one review reported subgroup analyses and meta-regression rather than sensitivity analysis and meta-regression; and 22 reviews reported unnamed analysis rather than subgroup analysis (i.e. appeared to be planning subgroup analysis but did not report the type of the analysis explicitly).

For the 33 reviews that applied interaction analyses, when we compared the type of interaction analysis reported in the review with the type we judged it to be based on the S1 File, in 27 reviews (52%) there was a discrepancy: four reviews reported sensitivity analysis rather than subgroup analysis; two reviews reported subgroup analyses rather than sensitivity analysis; one review reported subgroup and meta-regression but applied either subgroup analysis or meta-regression; 23 reviews reported unnamed analysis rather than subgroup analysis (i.e. applied subgroup analysis but did not explicitly report the type of the analysis); and one review reported unnamed analysis rather than sensitivity analysis.

#### Detecting interactions

*Were methods to detect interactions reported to be planned and, if interaction analyses were applied, actually used for each covariate?*

In one review (2%), methods to detect interactions were reported to be planned and, if interaction analyses were applied actually used, for every covariate. The review planned to use a method to detect interactions (i.e. the test of interaction) for each covariate but did not carry out interaction analysis.

In nine reviews (17%), methods to detect interactions were reported to be planned and used (when interaction analyses were applied) for at least one covariate. Each of the nine reviews planned to use methods to detect interactions for at least one covariate but did not carry out interaction analysis. On average, the percentage of covariates with methods reported in each review was very low. The reported methods were test for subgroup differences (three reviews), t-tests and chi-squared tests (one review), I-squared statistic (one review), overlap of confidence intervals and test for subgroup differences (one review), ‘visually (e.g. box plots), subgroup analyses, meta-regression’ (one review), changes in the results (one review), interaction tests or overlap of confidence intervals (one review).

Of note, two reviews (4%) did not plan any methods but, in the review, reported using a method to detect interactions (i.e. ‘pre-specified criteria, including a test for interaction’ (one review), ‘comparing results’ (one review)) for at least one analysed covariate.

#### Reporting interaction results

*Were results from interaction analysis reported for each covariate (for the outcome)?*

Every review reported statistical results for at least one analysed covariate but only 30 reviews (91%) reported statistical results for each analysed covariate. The remaining three reviews (9%) described results qualitatively, rather than quantitatively, for at least one analysed covariate.

Types of results included: trial group results (e.g. event rates, or mean, standard deviation and number of patients, incident rate and confidence interval, percentage); observed trial treatment effects and their confidence intervals or p-values; the pooled treatment effect and its confidence interval, z statistic, and p-value for subtotals and/or totals; number needed to benefit or harm and its confidence interval; regression coefficient and its confidence interval and p-value; p-value from ANOVA, and number of eyes.

*Were results from methods to detect interactions reported for each covariate (for the outcome)?*

Four reviews (12%) reported statistical results for each analysed covariate; however, 14 reviews (42%) reported results for at least one analysed covariate. The percentage of covariates with results reported from methods to detect interactions in each review was markedly low overall (median 0%, IQR 0–50%).

Types of reported results included: test for subgroup differences (chi-square statistic, p-value, I-square statistic), or the regression coefficient and p-value from meta-regression.

#### Interpreting interaction results

*Was it reported whether or not an interaction was detected for each analysed covariate (for the outcome)?*

One review (3%) reported whether or not an interaction was detected for each analysed covariate. Eleven reviews (33%) reported whether or not an interaction was detected for at least one analysed covariate. See S3 Table for the reported text. Overall, the percentage of analysed covariates that reported whether an interaction was detected or not in each review was low (median 0%, IQR 0–33%).

S11 Table shows our judgement of whether an interaction was detected using the test for subgroup differences (where presented) and also whether the review authors reported an interaction existed (where reported) for each analysed covariate. Out of the 94 analyses reported in the 33 reviews, results from the test and the review authors’ conclusions were presented for nine analyses in three reviews. We found no discrepancies between the test result and authors conclusions.

*Was the importance of the interaction or lack of interaction discussed for each analysed covariate (for the outcome)?*

No review explicitly discussed the importance of any interaction or lack of interaction for any analysed covariate in the results and/or discussion of the review.

*Was the plausibility of the interaction or lack of interaction discussed for each analysed covariate (for the outcome)?*

No review discussed the plausibility of the interaction or lack of interaction for each analysed covariate. Two reviews (6%) discussed the plausibility for one analysed covariate (see S11 Table for the reported text).

*Was the possibility of confounding discussed for each analysed covariate (for the outcome)?*

No review discussed the possibility of confounding for every analysed covariate but one review (3%) did this for one of the analysed covariates (see S11 Table).

*Was the covariate distribution discussed for each analysed covariate (for the outcome)?*

Thirteen reviews (39%) reported the covariate distribution for every analysed covariate; while 26 reviews (79%) reported the distribution for at least one analysed covariate. The percentage of analysed covariates with the distribution reported in each review was generally high (median 67%, IQR 25–100). See S3 Table for the reported text.

## Discussion

### Key findings and recommendations

Our review highlights the strengths and weaknesses of interaction analyses in Cochrane Reviews. We identified important flaws with respect to all aspects of interaction analyses but believe there to be particularly poor practice regarding the interpretation, detection, and design, of interaction analyses. Conversely, we did identify good practice, in particular, for analysing interactions and reporting interaction results.

Based on our findings we make key recommendations that could be used by review authors to improve future reviews, and by readers of reviews evaluating the findings of published reviews:

The method for choosing covariates, the chosen covariates, and the clinical rationale for choosing each covariate as a potential treatment effect modifying covariate should be reported in the methods section of the protocol and subsequent review. Interaction analyses should be planned a priori wherever possible and any covariate analyses undertaken post-hoc (i.e. in the review but not in the protocol) should be identified as such in the review. The number of covariates should be limited. In this review, no review reported how or why covariates were chosen; 42% of the reviews reported each covariate a priori; no review labelled each post-hoc covariate as such; and 21% of reviews mentioned less than six covariates.

It is important that attempts are made to obtain missing covariate data from the trial investigators; however, we found that only one review reported doing this. AD analyses should be applied for study-level covariates providing the covariate distribution sufficiently differs across trials and the number of included trials is not too limited. We found that 96% of reviews including study-level covariates reported AD analyses were planned and, if analyses were applied, done; and 15% of reviews did AD analyses including at least 10 trials for each covariate. IPD analyses are the gold standard for exploring patient-level covariates (providing that the covariate distribution differs within trials), yet we found that no review including patient-level covariates, reported IPD analyses were planned and, if analyses were applied, done. We recommend avoiding categorising continuous covariates when possible, but if continuous covariates are categorised, justification should be provided for doing so in the methods. In our review, no review that categorised continuous covariates, justified the categorisation for each covariate. When categorical covariates are explored, we believe the chosen categories should be reported in the methods, along with the justification for the categories when the chosen categories involve subjectivity (e.g. cut off of a continuous scale chosen by review authors). We found 12% of review including categorical covariates reported categories for each covariate and no review justified the categories for each covariate.

Interaction analysis methods (i.e. subgroup analysis or meta-regression) should be reported, along with methods to detect interactions ((i.e. to assess differences in the treatment effects from different subgroups, e.g. test for subgroup differences) in the methods. Overall, 98% of reviews reported that interaction analysis was planned; 2% of reviews reported planning and, if analyses were applied, using a method to detect interactions for each covariate. We would like to emphasise the importance of providing reasons for differences in the methodology between the protocol and review. We found that in 46% of reviews there were discrepancies between the interaction analysis method reported in the protocol and review. Furthermore, reasons should be given if interaction analyses are not possible (e.g. insufficient data in the trial report).

Statistical results from the interaction analysis (e.g. treatment group data summaries from trials, treatment effects and confidence intervals for trials and meta-analyses) and methods to detect interactions (e.g. p-value from test for subgroup differences) should be reported for each covariate. We found that 91% and 12% of reviews reported results from the interaction analysis and methods to detect interactions respectively for each covariate.

To help readers interpret the analyses, review authors should clearly report whether or not an interaction was detected; as well as discussing the plausibility and importance of the findings, the possibility of confounding, the covariate distribution, and any limitations of the interaction analyses. In this review, only 3% of reviews reported whether an interaction was detected for each covariate and no review discussed the importance, or plausibility, of the results, or the possibility of confounding for each covariate. Yet, 39% of reviews discussed the covariate distribution for each covariate.

### Limitations, further work, and generalizability

The main limitation of this work is that we have only included a sample of Cochrane Reviews. The devised criteria are certainly applicable to the different types of reviews, such as those published in journals and health technology assessments and, on the most part, we believe that the findings of this review also apply to different types of reviews. However, the development of a protocol and the support from methodologists and clinical experts provided by review groups in the development of protocol and review [1,6,7], may in itself improve the standard of analysis and reporting of interactions. Therefore, the findings in this review may present an optimistic view of overall current practice. Of course, other types of reviews may also develop and register a protocol (e.g. on PROSPERO [12]). As further work, it would be useful to review and compare different types of reviews.

We excluded review updates (i.e. those with multiple versions of the review published in the CDSR) because we were interested in comparing the planned methods in the protocol and the methods applied in the review. Review updates are published after the original version of the review, therefore: methods in the protocol are more likely to have become outdated; the author team involved in the review update is more likely to differ from the team involved at the protocol stage and therefore individuals may disagree with the planned methods; and the author team is less likely to refer back to the protocol for a review update. For that reason we believe that there may be greater differences between the planned methods in the protocol and applied methods in a review update.

After pre-piloting the criteria, we chose not to assess: whether the covariate distribution was adequate to detect an interaction if it existed (not done because clinical input was required); whether the review author reported that heterogeneity was reduced in the interaction analysis (required detection of heterogeneity); whether trials were independent when combined in meta-analysis or involved in the test for subgroup differences (difficult without closer inspection); whether phrasing of covariates and covariate categories differed between the protocol and review (subjective). Some of the issues may be worth addressing in future research.

The results presented here differ somewhat from those reported by Higgins *et al*. [11]. Higgins *et al*. [11] presented results from 28 published Cochrane Reviews sought from CDSR in 2001. We found 98% of reviews reported at least one covariate a priori and 42% reported all of the covariates a priori; they found 54% (15/28) of reviews reported at least one covariate a priori and 32% (9/28) of reviews reported all of the covariates a priori; therefore reviews seem more likely to predefine covariates now than in 2001. Furthermore, we found that 2% of reviews did not plan interaction analysis in the protocol and 37% of reviews did not do any analyses in the review; they found 46% (13/28) of reviews did not plan such analyses and 68% (19/28) of reviews did not do analyses. Therefore, review authors also appear to be more likely to plan and apply interaction analyses currently than in 2001. They found that the reasons for not doing planned analyses included insufficient information to classify studies into subgroups, too few studies, planned subgroup analyses more appropriate for dividing patients than studies, too much diversity in studies to consider any meta-analysis, no studies in a subgroup and lack of a significant treatment effect across all studies; we identified similar reasoning (i.e. insufficient data, no meta-analysis applied, or studies did not provide the required data). Our review could be repeated in the future to see whether Cochrane Reviews have improved over time.

### Conclusions

In conclusion, interaction analyses can be extremely valuable, not only to explore causes of heterogeneity, but also for personalised medicine. Far fewer reviews follow recommendations given in the literature and elsewhere than expected. We found that the design of interaction analyses, detection of interactions, and interpretation of interactions was particularly poor. The criteria proposed in this review could be used to more carefully consider the design, application, reporting, and interpretation of analyses.

## Supporting Information

S1 File Definitions of covariates and interaction analyses used in this review. (DOCX)

S2 File References for included reviews. (DOCX)

S1 Table Characteristics of reviews. (DOCX)

S2 Table Covariates mentioned and/or analysed. (DOCX)

S3 Table Analysed covariates in each review. (DOCX)

S4 Table Number of trials and patients, type of covariates, and data type of covariates, in the review. (DOCX)

S5 Table Results of assessment of interactions for each review. (DOCX)

S6 Table Choosing covariates: covariates reported with rationale, a priori, and labelled as post-hoc; and number of reported and analysed covariates. (DOCX)

S7 Table Considering covariate data: number of covariates with reported categories and justification for categories; and number of trials in analyses. (DOCX)

S8 Table Analysing interactions: The type of interaction analysis reported in the protocol and review, and whether we judged the analysis to be of that type. (DOCX)

S9 Table Detecting interactions: planned and used methods to detect interactions. (DOCX)

S10 Table Reporting interaction results: results reported from interaction analysis and from method to detect interactions. (DOCX)

S11 Table Interpreting covariates: Reporting of whether interaction was detected, results from the test for subgroup differences, and discussion of plausibility, confounding and the covariate distribution. (DOCX)

S12 Table Rationale for covariates (when reported). (DOCX)

S13 Table Justification for covariate categories (when given). (DOCX)

## References

[1] Higgins JPT, Green S (editors). Cochrane Handbook for Systematic Reviews of Interventions Version 5.1.0 [updated March 2011]. The Cochrane Collaboration, 2011 Available from www.cochrane-handbook.org/.

[2] Chaparro LE, Smith SA, Moore RA, Wiffen PJ, Gilron I. Pharmacotherapy for the prevention of chronic pain after surgery in adults. Cochrane Database of Systematic Reviews. 2013, Issue 7. Art. No.: CD008307. 10.1002/14651858.CD008307.pub2. 10.1002/14651858.CD008307.pub2 PMC6481826 23881791

[3] Gagnier J, Moher D, Boon H, Beyene J, Bombardier C. Investigating clinical heterogeneity in systematic reviews: a methodologic review of guidance in the literature. BMC Med Res Methodol.2012; 12: 111 10.1186/1471-2288-12-111 22846171 PMC3564789

[4] Gagnier J, Morgenstern H, Altman D, Berlin J, Chang S, McCulloch P, et al Consensus-based recommendations for investigating clinical heterogeneity in systematic reviews. BMC Med Res Methodol.2013; 13: 106 10.1186/1471-2288-13-106 24004523 PMC3847163

[5] Thompson SG, Higgins JPT. How should meta-regression analyses be undertaken and interpreted? Statistics in Medicine.2002; 21: 1559–1573. 12111920 10.1002/sim.1187

[6] Dias S, Sutton AJ, Welton NJ, Ades AE. Evidence synthesis for decision making 3: heterogeneity: subgroups, meta-regression, bias and bias-adjustment. Med Decis Making. 2013; 33(5);618–640. 10.1177/0272989X13485157 23804507 PMC3704206

[7] NICE. Guide to the methods of technology appraisal. 2013. Available: http://publications.nice.org/pmg9. 27905712

[8] Yusuf S, Wittes J, Probstfield J, Tyroler HA. Analysis and interpretation of treatment effects in subgroups of patients in randomized clinical trials. JAMA.1991; 266: 93–98. 2046134

[9] Oxman AD, Guyatt GH. A Consumer's Guide to Subgroup Analyses. Ann Intern Med.1992; 116: 78–84. 1530753 10.7326/0003-4819-116-1-78

[10] Centre for Reviews and Dissemination. Systematic reviews. CRD's guidance for undertaking reviews in healthcare. 2009. Available: http://www.york.ac.uk/inst/crd/SysRev/!SSL!/WebHelp/SysRev3.htm.

[11] Higgins J, Thompson S, Deeks J, Altman D. Statistical heterogeneity in systematic reviews of clinical trials: a critical appraisal of guidelines and practice. J Health Serv Res Policy.2002; 7: 51–61. 11822262 10.1258/1355819021927674

[12] Centre for Reviews and Dissemination. Welcome to PROSPERO. 2013 Nov 28 [cited 9 June 2014]. Available: http://www.crd.york.ac.uk/PROSPERO/.

