# Supplementary material for: Exploring Treatment by Covariate Interactions Using Subgroup Analysis and Meta-Regression in Cochrane Reviews: A Review of Recent Practice
Source: PLoS One. 2015 Jun 1;10(6):e0128804. doi: 10.1371/journal.pone.0128804 (PMC4452239; doi:10.1371/journal.pone.0128804)
Supplement: S1 File — (DOCX) [file pone.0128804.s001.docx]

**File S1: Definitions of covariates and interaction analyses used in this review.**

**Covariate:** A patient-level baseline characteristic (e.g. age, gender) or study characteristic (e.g. setting, dose) that may, or may not, affect the size, or direction, of the treatment effect.

**Meta-analysis model with treatment by covariate interaction: a** statistical meta-analysis model used to explore the relationship between study (e.g. **allocation concealment**) or patient characteristics (e.g. gender) and the treatment effect (e.g. odds ratio) by including a treatment by covariate interaction in the model**. Such models can accommodate AD (e.g. the observed treatment effects and their standard errors from the studies, and study-level covariates (e.g. proportion of male patients) or IPD (i.e. raw data obtained from the trial investigators consisting of patient specific outcomes, treatment allocations, and covariate information (e.g. gender)). Models that accommodate the observed treatment effects and their standard errors from the studies are called meta-regression models.**

**Subgroup/stratification analysis:** an analysis that groups the trials according to their covariate category (e.g. age group, gender, socioeconomic class). Different data from the same trial may be included in one or more group (e.g. when the analysis is grouped by dose and a multi-arm study compares multiple doses). An across-group meta-analytic ‘total’ result may be reported as well as the within-group meta-analytic ‘subtotal’ results. Within-group ‘subtotal’ results may be reported in a single forest plot or in multiple forest plots (e.g. with one plot per group). If the results text or tables are stratified (i.e. has different sub-headings for different covariate categories), we considered this to be a stratification/subgroup analysis, even when no meta-analysis was carried out for trials in each category.

**Sensitivity analysis:** either (1) a secondary analysis that excludes a group of trials with a particular covariate category (e.g. exclude trials of high risk of bias for blinding); or (2) a secondary analysis that uses a different method compared with the primary analysis (e.g. summarising data using the risk ratio in a secondary analysis and using the odds ratio in the primary analysis). In this review, we considered (1) to be an interaction analysis, but not (2) because such analyses are used to assess how robust the results are to assumptions made about the data or the methods used in the review.
