# Supplementary material for: Exploring Treatment by Covariate Interactions Using Subgroup Analysis and Meta-Regression in Cochrane Reviews: A Review of Recent Practice
Source: PLoS One. 2015 Jun 1;10(6):e0128804. doi: 10.1371/journal.pone.0128804 (PMC4452239; doi:10.1371/journal.pone.0128804)
Supplement: S2 File — (DOCX) [file pone.0128804.s002.docx]

**File S2: References for included reviews** [1-104].

1. Schoot RA, van Dalen EC, van Ommen CH, van de Wetering MD. Antibiotic and other lock treatments for tunnelled central venous catheter related infections in children with cancer (Protocol). . Cochrane Database of Systematic Reviews. 2011, issue 2. Art. No.: CD008975. doi: 10.1002/14651858.CD008975.

2. Schoot RA, van Dalen EC, van Ommen CH, van de Wetering MD. Antibiotic and other lock treatments for tunnelled central venous catheter-related infections in children with cancer. Cochrane Database of Systematic Reviews. 2013, issue 6. Art. No.: CD008975. doi: 10.1002/14651858.CD008975.pub2.

3. Cruciani M, Mengoli C, Serpelloni G, Parisi Saverio G, Malena M, Bosco O. Abacavir-based triple nucleoside regimens for maintenance therapy in patients with HIV. Cochrane Database of Systematic Reviews. 2013, issue 6. Art. No.: CD008270. doi: 10.1002/14651858.CD008270.pub2.

4. Cruciani M, Mengoli C, Serpelloni G, Parisi SG. Abacavir-based triple nucleoside regimens for maintenance therapy in patients with HIV (Protocol). Cochrane Database of Systematic Reviews 2010, issue 1. Art. No.: CD008270. doi: 10.1002/14651858.CD008270.

5. Showell MG, Brown J, Clarke J, Hart RJ. Antioxidants for female subfertility. Cochrane Database of Systematic Reviews. 2013, issue 8. Art. No.: CD007807. doi: 10.1002/14651858.CD007807.pub2.

6. Clarke J, Showell MG, Hart RJ, Agarwal A, Gupta S. Antioxidants for female subfertility (Protocol). Cochrane Database of Systematic Reviews. 2009, issue 2. Art. No.: CD007807. doi: 10.1002/14651858.CD007807.

7. Sampson S, Mansour M, Maayan N, Soares-Weiser K, Adams CE. Intermittent drug techniques for schizophrenia. Cochrane Database of Systematic Reviews. 2013, issue 7. Art. No.: CD006196. doi: 10.1002/14651858.CD006196.pub2.

8. Mansour M, Alomar A, Boobes K, Awf Mouchli M. Intermittent drug techniques for schizophrenia (Protocol) Cochrane Database of Systematic Reviews 2006, issue 4. Art. No.: CD006196. doi: 10.1002/14651858.CD006196.

9. Boselie TFM, Willems PC, van Mameren H, de Bie R, Benzel EC, van Santbrink H. Arthroplasty versus fusion in single-level cervical degenerative disc disease. Cochrane Database of Systematic Reviews. 2012, issue 9. Art. No.: CD009173. doi: 10.1002/14651858.CD009173.pub2.

10. Boselie AFM, van Santbrink H, van Mameren H, de Bie R, Benzel EC, Willems PC. Fusion versus arthroplasty in single level cervical degenerative disc disease (Protocol). . Cochrane Database of Systematic Reviews. 2011, issue 6. Art. No.: CD009173. doi: 10.1002/14651858.CD009173.

11. Sajid MS, Hutson KH, Rapisarda IF, Bonomi R. Fibrin glue instillation under skin flaps to prevent seroma related morbidity following breast and axillary surgery (Protocol). Cochrane Database of Systematic Reviews 2012, issue 1. Art. No.: CD009557. doi: 10.1002/14651858.CD009557.

12. Sajid MS, Hutson KH, Rapisarda IF, Bonomi R. Fibrin glue instillation under skin flaps to prevent seroma-related morbidity following breast and axillary surgery. Cochrane Database of Systematic Reviews. 2013, issue 5. Art. No.: CD009557. doi: 10.1002/14651858.CD009557.pub2.

13. Gillies D, O'Brien L, Rogers P, Meekings C. Psychological therapies for the prevention and treatment of post-traumatic stress disorder in children and adolescents (Protocol). Cochrane Database of Systematic Reviews 2007, issue 3. Art. No.: CD006726. doi: 10.1002/14651858.CD006726.

14. Gillies D, Taylor F, Gray C, O'Brien L, D'Abrew N. Psychological therapies for the treatment of post-traumatic stress disorder in children and adolescents. Cochrane Database of Systematic Reviews. 2012, issue 12. Art. No.: CD006726. doi: 10.1002/14651858.CD006726.pub2.

15. Lawrie TA, Bryant A, Cameron A, Gray E, Morrison J. Pegylated liposomal doxorubicin for relapsed epithelial ovarian cancer. Cochrane Database of Systematic Reviews. 2013, issue 7. Art. No.: CD006910. doi: 10.1002/14651858.CD006910.pub2.

16. Cameron A, Gray E, Williams C. Pegylated liposomal doxorubicin for relapsed ovarian cancer (Protocol). Cochrane Database of Systematic Reviews 2008, issue 1. Art. No.: CD006910. doi: 10.1002/14651858.CD006910.

17. Johnston BC, Thorlund K. Probiotics for the prevention of Clostridium difficile associated diarrhea in adults and children (Protocol). Cochrane Database of Systematic Reviews 2009, issue 1. Art. No.: CD006095. doi: 10.1002/14651858.CD006095.pub2.

18. Goldenberg JZ, Ma SSY, Saxton JD, Martzen MR, Vandvik Per O, Thorlund K, et al. Probiotics for the prevention of Clostridium difficile-associated diarrhea in adults and children. Cochrane Database of Systematic Reviews. 2013, issue 5. Art. No.: CD006095. doi: 10.1002/14651858.CD006095.pub3.

19. Deare JC, Zheng Z, Xue CCL, Liu JP, Shang J, Scott SW, et al. Acupuncture for treating fibromyalgia. Cochrane Database of Systematic Reviews. 2013, issue 5. Art. No.: CD007070. doi: 10.1002/14651858.CD007070.pub2.

20. Deare JC, Zheng Z, Xue CC, Liu JP, Shang J, Scott SW, et al. Acupuncture for treating fibromyalgia (Protocol). Cochrane Database of Systematic Reviews 2008, issue 2. Art. No.: CD007070. doi: 10.1002/14651858.CD007070.

21. Chaparro LE, Smith SA, Moore RA, Wiffen PJ, Gilron I. Pharmacotherapy for the prevention of chronic pain after surgery in adults. Cochrane Database of Systematic Reviews. 2013, issue 7. Art. No.: CD008307. doi: 10.1002/14651858.CD008307.pub2.

22. Gilron I, Moore RA, Wiffen PJ, McQuay HJ. Pharmacotherapy for the prevention of chronic pain after surgery in adults (Protocol). Cochrane Database of Systematic Reviews. 2010, issue 1. Art. No.:

CD008307. doi: 10.1002/14651858.CD008307.

23. Sharma SK, Sharma A, Kadhiravan T, Tharyan P. Isoniazid monotherapy versus other monotherapies or combination chemotherapy for preventing active tuberculosis in HIV-negative persons (Protocol). Cochrane Database of Systematic Reviews 2009, issue 1. Art. No.: CD007545. doi: 10.1002/14651858.CD007545.

24. Sharma SK, Sharma A, Kadhiravan T, Tharyan P. Rifamycins (rifampicin, rifabutin and rifapentine) compared to isoniazid for preventing tuberculosis in HIV-negative people at risk of active TB. Cochrane Database of Systematic Reviews. 2013, issue 7. Art. No.: CD007545. doi: 10.1002/14651858.CD007545.pub2.

25. Ziebell M, Wetterslev J, Tisell M, Gluud C, Juhler M. Flow-regulated versus differential pressure-regulated shunt valves for adult patients with normal pressure hydrocephalus (Protocol) Cochrane Database of Systematic Reviews. 2012, issue 3. Art. No.: CD009706. doi: 10.1002/14651858.CD009706.

26. Ziebell M, Wetterslev J, Tisell M, Gluud C, Juhler M. Flow-regulated versus differential pressure-regulated shunt valves for adult patients with normal pressure hydrocephalus. Cochrane Database of Systematic Reviews. 2013, issue 5. Art. No.: CD009706. doi: 10.1002/14651858.CD009706.pub2.

27. Parker B, Turner W. Psychoanalytic/psychodynamic psychotherapy for children and adolescents who have been sexually abused (Protocol) Cochrane Database of Systematic Reviews. 2009, issue 4. Art. No.: CD008162. doi: 10.1002/14651858.CD008162.

28. Parker B, Turner W. Psychoanalytic/psychodynamic psychotherapy for children and adolescents who have been sexually abused. Cochrane Database of Systematic Reviews. 2013, issue 7. Art. No.: CD008162. doi: 10.1002/14651858.CD008162.pub2.

29. Marigold R, Günther A, Tiwari D, Kwan J. Antiepileptic drugs for the primary and secondary prevention of seizures after subarachnoid haemorrhage (Protocol). Cochrane Database of Systematic Reviews 2010, issue 9. Art. No.: CD008710. doi: 10.1002/14651858.CD008710.

30. Marigold R, Günther A, Tiwari D, Kwan J. Antiepileptic drugs for the primary and secondary prevention of seizures after subarachnoid haemorrhage. Cochrane Database of Systematic Reviews. 2013, issue 6. Art. No.: CD008710. doi: 10.1002/14651858.CD008710.pub2.

31. Gan T, Tian L, Jin SJ, Wang Y. Medicinal herbs for cholelithiasis (Protocol). Cochrane Database of Systematic Reviews. 2004, issue 1. Art. No.: CD004547. doi: 10.1002/14651858.CD004547.

32. Gan T, Chen J, Jin SJ, Wang Y. Chinese medicinal herbs for cholelithiasis. Cochrane Database of Systematic Reviews. 2013, issue 6. Art. No.: CD004547. doi: 10.1002/14651858.CD004547.pub2.

33. Liu ZL, Wu Q, Liu JP, Li GQ, Bensoussan A, Kiat H. Chinese herbal medicines for hypertriglyceridemia (Protocol). Cochrane Database of Systematic Reviews. 2012, issue 1. Art. No.: CD009560. doi: 10.1002/14651858.CD009560.

34. Liu ZL, Li GQ, Bensoussan A, Kiat H, Chan K, Liu JP. Chinese herbal medicines for hypertriglyceridaemia. Cochrane Database of Systematic Reviews. 2013, issue 6. Art. No.: CD009560. doi: 10.1002/14651858.CD009560.pub2.

35. Trivedi A, Sinn JKH. Early versus late administration of amino acids in preterm infants receiving parenteral nutrition (Protocol). Cochrane Database of Systematic Reviews. 2010, issue 10. Art. No.: CD008771. doi: 10.1002/14651858.CD008771.

36. Trivedi A, Sinn JKH. Early versus late administration of amino acids in preterm infants receiving parenteral nutrition. Cochrane Database of Systematic Reviews. 2013, issue 7. Art. No.: CD008771. doi: 10.1002/14651858.CD008771.pub2.

37. Dashash M, Yeung A, Sbenati A, Blinkhorn A. Interventions for the restorative care of amelogenesis imperfecta in children and adolescents (Protocol). Cochrane Database of Systematic Reviews 2008, issue 2. Art. No.: CD007157. doi: 10.1002/14651858.CD007157.

38. Dashash M, Yeung CA, Jamous I, Blinkhorn A. Interventions for the restorative care of amelogenesis imperfecta in children and adolescents. Cochrane Database of Systematic Reviews. 2013, issue 6. Art. No.: CD007157. doi: 10.1002/14651858.CD007157.pub2.

39. Van Teeffelen S, Pajkrt E, Willekes C, Van Kuijk SMJ, Mol BWJ. Amnioinfusion for improving fetal outcomes after preterm prelabour rupture of membranes (Protocol). Cochrane Database of Systematic Reviews. 2012, issue 7. Art. No.: CD009952. doi: 10.1002/14651858.CD009952.

40. Van Teeffelen S, Pajkrt E, Willekes C, Van Kuijk SMJ, Mol BWJ. Transabdominal amnioinfusion for improving fetal outcomes after oligohydramnios secondary to preterm prelabour rupture of membranes before 26 weeks. Cochrane Database of Systematic Reviews. 2013, issue 8. Art. No.: CD009952. doi: 10.1002/14651858.CD009952.pub2.

41. Lee AL, Burge A, Jones AP, Rowe BH, Holland AE. Airway clearance techniques for bronchiectasis (Protocol). Cochrane Database of Systematic Reviews. 2010, issue 2. Art. No.: CD008351. doi: 10.1002/14651858.CD008351.

42. Lee AL, Burge A, Holland AE. Airway clearance techniques for bronchiectasis. Cochrane Database of Systematic Reviews. 2013, issue 5. Art. No.: CD008351. doi: 10.1002/14651858.CD008351.pub2.

43. Almeida MO, Silva BN, Andriolo RB, Atallah ÁN, Peccin MS. Conservative interventions for treating exercise-related musculotendinous, ligamentous and osseous groin pain (Protocol). . Cochrane Database of Systematic Reviews 2012, issue 1. Art. No.: CD009565. doi: 10.1002/14651858.CD009565.

44. Almeida MO, Silva BNG, Andriolo RB, Atallah ÁN, Peccin MS. Conservative interventions for treating exercise-related musculotendinous, ligamentous and osseous groin pain. Cochrane Database of Systematic Reviews. 2013, issue 6. Art. No.: CD009565. doi: 10.1002/14651858.CD009565.pub2.

45. Kinnersley P, Stephens BL, Elwyn GJ, Blazeby J, Kelly M, Savage K, et al. Interventions to promote informed consent for patients undergoing surgical and other invasive healthcare procedures (Protocol). . Cochrane Database of Systematic Reviews 2011, issue 11. Art. No.: CD009445. doi: 10.1002/14651858.CD009445.

46. Kinnersley P, Phillips K, Savage K, Kelly M J, Farrell E, Morgan B, et al. Interventions to promote informed consent for patients undergoing surgical and other invasive healthcare procedures. Cochrane Database of Systematic Reviews. 2013, issue 7. Art. No.: CD009445. doi: 10.1002/14651858.CD009445.pub2.

47. Fedorowicz Z, van Zuuren EJ, A. AH. Low-molecular-weight heparins for managing vaso-occlusive crises in people with sickle cell disease (Protocol). Cochrane Database of Systematic Reviews. 2012, issue 10. Art. No.: CD010155. doi: 10.1002/14651858.CD010155.

48. van Zuuren EJ, Fedorowicz Z. Low-molecular-weight heparins for managing vaso-occlusive crises in people with sickle cell disease. Cochrane Database of Systematic Reviews. 2013, issue 6. Art. No.: CD010155. doi: 10.1002/14651858.CD010155.pub2.

49. Tejani AM, Chan AHW, Kuo IF, Li J. Magnesium for alcohol withdrawal (Protocol). Cochrane Database of Systematic Reviews 2010, issue 2. Art. No.: CD008358. doi: 10.1002/14651858.CD008358.

50. Sarai M, Tejani AM, Chan AHW, Kuo IF, Li J. Magnesium for alcohol withdrawal. Cochrane Database of Systematic Reviews. 2013, issue 6. Art. No.: CD008358. doi: 10.1002/14651858.CD008358.pub2.

51. Rockers PC, Bärnighausen T. Interventions for hiring, retaining and training district health system managers in low- and middle-income countries (Protocol). Cochrane Database of Systematic Reviews. 2011, issue 3. Art. No.: CD009035. doi: 10.1002/14651858.CD009035.

52. Rockers PC, Bärnighausen T. Interventions for hiring, retaining and training district health systems managers in low- and middle-income countries. Cochrane Database of Systematic Reviews. 2013, issue 4. Art. No.: CD009035. doi: 10.1002/14651858.CD009035.pub2.

53. Gois PHF, Souza ERDM, Santos CUD. Pharmacotherapy for hyperuricemia in hypertensive patients (Protocol). . Cochrane Database of Systematic Reviews. 2010, issue 7. Art. No.: CD008652. doi: 10.1002/14651858.CD008652. .

54. Gois PHF, Souza ERDM. Pharmacotherapy for hyperuricemia in hypertensive patients. Cochrane Database of Systematic Reviews. 2013, issue 1. Art. No.: CD008652. doi: 10.1002/14651858.CD008652.pub2.

55. Wang Y, Zhishun L, Peng W. Acupuncture for stress urinary incontinence in adults (Protocol) Cochrane Database of Systematic Reviews. 2011, issue 10. Art. No.: CD009408. doi: 0.1002/14651858.CD009408.

56. Wang Y, Zhishun L, Peng W, Zhao J, Liu B. Acupuncture for stress urinary incontinence in adults. Cochrane Database of Systematic Reviews. 2013, issue 7. Art. No.: CD009408. doi: 10.1002/14651858.CD009408.pub2.

57. Berlowitz D, Tamplin J. Respiratory muscle training for cervical spinal cord injury (Protocol). Cochrane Database of Systematic Reviews. 2010, issue 5. Art. No.: CD008507. doi: 10.1002/14651858.CD008507.

58. Berlowitz DJ, Tamplin J. Respiratory muscle training for cervical spinal cord injury. Cochrane Database of Systematic Reviews. 2013, issue 7. Art. No.: CD008507. doi: 10.1002/14651858.CD008507.pub2.

59. He D, Han K, Gao X, Dong S, Chu L, Feng Z, et al. Laquinimod for multiple sclerosis (Protocol). Cochrane Database of Systematic Reviews 2013, issue 4. Art. No.: CD010475. doi: 10.1002/14651858.CD010475.

60. He D, Han K, Gao X, Dong S, Chu L, Feng Z, et al. Laquinimod for multiple sclerosis. Cochrane Database of Systematic Reviews. 2013, issue 8. Art. No.: CD010475. doi: 10.1002/14651858.CD010475.pub2.

61. Aboumarzouk OM, Nelson RL. Pregabalin for chronic prostatitis (Protocol). . Cochrane Database of Systematic Reviews 2011, issue 4. Art. No.: CD009063. doi: 10.1002/14651858.CD009063.

62. Aboumarzouk OM, Nelson RL. Pregabalin for chronic prostatitis. Cochrane Database of Systematic Reviews. 2012, issue 8. Art. No.: CD009063. doi: 10.1002/14651858.CD009063.pub2.

63. Pega F, Carter K, Blakely T, Lucas P. In-work tax credits for families and their impact on health status in adults (Protocol). Cochrane Database of Systematic Reviews 2012, issue 7. Art. No.: CD009963 doi: 10.1002/14651858.CD009963.

64. Pega F, Carter K, Blakely T, Lucas PJ. In-work tax credits for families and their impact on health status in adults. Cochrane Database of Systematic Reviews. 2013, issue 8. Art. No.: CD009963. doi: 10.1002/14651858.CD009963.pub2.

65. Basurto OX, Rigau CD, Urrútia G. Opioids for acute pancreatitis pain (Protocol). Cochrane Database of Systematic Reviews. 2011, issue 6. Art. No.: CD009179. doi: 10.1002/14651858.CD009179.

66. Basurto OX, Rigau CD, Urrútia G. Opioids for acute pancreatitis pain. Cochrane Database of Systematic Reviews. 2013, issue 7. Art. No.: CD009179. doi: 10.1002/14651858.CD009179.pub2.

67. Li S, Yue J, Dong BR, Yang M, Lin X, Wu T. Acetaminophen (paracetamol) for the common cold in adults (Protocol). Cochrane Database of Systematic Reviews. 2010, issue 11. Art. No.: CD008800. doi: 10.1002/14651858.CD008800.

68. Li S, Yue J, Dong Bi R, Yang M, Lin X, Wu T. Acetaminophen (paracetamol) for the common cold in adults. Cochrane Database of Systematic Reviews. 2013, issue 7. Art. No.: CD008800. doi: 10.1002/14651858.CD008800.pub2.

69. Leyngold I, Nanji AA, Chuck RS, Behrens A, Vedula SS, McDonnell PJ, et al. Perioperative antibiotics for prevention of acute endophthalmitis after cataract surgery. (Protocol). Cochrane Database of Systematic Reviews 2007, issue 1. Art. No.: CD006364. doi: 10.1002/14651858.CD006364.

70. Gower EW, Lindsley K, Nanji A A, Leyngold I, McDonnell PJ. Perioperative antibiotics for prevention of acute endophthalmitis after cataract surgery. Cochrane Database of Systematic Reviews. 2013, issue 7. Art. No.: CD006364. doi: 10.1002/14651858.CD006364.pub2.

71. Wakai A, McMahon G. Nitrates for acute heart failure (Protocol). Cochrane Database of Systematic Reviews. 2005, issue 1. Art. No.: CD005151. doi: 10.1002/14651858.CD005151.

72. Wakai A, McCabe A, Kidney R, Brooks SC, Seupaul RA, Diercks DB, et al. Nitrates for acute heart failure syndromes. Cochrane Database of Systematic Reviews. 2013, issue 8. Art. No.: CD005151. doi: 10.1002/14651858.CD005151.pub2.

73. Freak-Poli RLA, Cumpston M, Peeters A, Clemes SA. Workplace pedometer interventions for increasing physical activity (Protocol). Cochrane Database of Systematic Reviews. 2011, issue 7. Art. No.: CD009209. doi: 10.1002/14651858.CD009209.

74. Freak-Poli RLA, Cumpston M, Peeters A, Clemes SA. Workplace pedometer interventions for increasing physical activity. Cochrane Database of Systematic Reviews. 2013, issue 4. Art. No.: CD009209. doi: 10.1002/14651858.CD009209.pub2.

75. Mutua FM, M'Imunya MJ, Wiysonge CS. Genital ulcer disease treatment for reducing sexual transmission of HIV (Protocol). Cochrane Database of Systematic Reviews. 2009, issue 3. Art. No.: CD007933. doi: 10.1002/14651858.CD007933.

76. Mutua FM, M'Imunya JM, Wiysonge CS. Genital ulcer disease treatment for reducing sexual acquisition of HIV. Cochrane Database of Systematic Reviews. 2012, issue 8. Art. No.: CD007933. doi: 10.1002/14651858.CD007933.pub2.

77. O'Connor D, Daborn C. Rehabilitation treatments following carpal tunnel surgery (Protocol). Cochrane Database of Systematic Reviews. 2003, issue 2. Art. No.: CD004158. doi: 10.1002/14651858.CD004158.

78. Peters S, Page MJ, Coppieters MW, Ross M, Johnston V. Rehabilitation following carpal tunnel release. Cochrane Database of Systematic Reviews. 2013, issue 6. Art. No.: CD004158. doi: 10.1002/14651858.CD004158.pub2.

79. Lopez LM, Hilgenberg D, Chen M, Denison J, Stuart G. Behavioral interventions for improving contraceptive use among women living with HIV. Cochrane Database of Systematic Reviews. 2013, issue 1. Art. No.: CD010243. doi: 10.1002/14651858.CD010243.pub2.

80. Lopez LM, Chen M, Hilgenberg D, Denison J, Stuart G. Behavioral interventions for improving contraceptive use among women living with HIV (Protocol). Cochrane Database of Systematic Reviews. 2012, issue 11. Art. No.: CD010243. doi: 10.1002/14651858.CD010243.

81. Cavalheri V, Tahirah F, Nonoyama M, Jenkins S, Hill K. Exercise training undertaken by people within 12 months of lung resection for non-small cell lung cancer. Cochrane Database of Systematic Reviews. 2013, issue 7. Art. No.: CD009955. doi: 10.1002/14651858.CD009955.pub2.

82. Cavalheri V, Tahirah F, Nonoyama M, Jenkins S, Hill K. Exercise training undertaken within 12 months following lung resection for patients with non-small cell lung cancer (Protocol). . Cochrane Database of Systematic Reviews. 2012, issue 7. Art. No.: CD009955. doi: 10.1002/14651858.CD009955.

83. Penninga L, Penninga EI, Møller Christian H, Iversen M, Steinbrüchel DA, Gluud C. Tacrolimus versus cyclosporin as primary immunosuppression for lung transplant recipients. Cochrane Database of Systematic Reviews. 2013, issue 5. Art. No.: CD008817. doi: 10.1002/14651858.CD008817.pub2.

84. Penninga L, Penninga EI, Møller CH, Steinbrüchel DA, Gluud C. Tacrolimus versus cyclosporin as primary immunosuppression for lung transplant recipients (Protocol). Cochrane Database of Systematic Reviews. 2010, issue 11. Art. No.: CD008817. doi: 10.1002/14651858.CD008817.

85. Bellmunt-Montoya S, Escribano JM, Dilme J, Martinez-Zapata MJ. CHIVA method for the treatment of chronic venous insufficiency. Cochrane Database of Systematic Reviews. 2013, issue 7. Art. No.: CD009648. doi: 10.1002/14651858.CD009648.pub2.

86. Bellmunt-Montoya S, Escribano JM, Dilme J, Martinez-Zapata MJ. CHIVA method for the treatment of varicose veins (Protocol). Cochrane Database of Systematic Reviews

2012, issue 2. Art. No.: CD009648. doi: 10.1002/14651858.CD009648.

87. Cheng Y, Lu J, Xiong X, Wu S, Lin Y, Wu T, et al. Gases for establishing pneumoperitoneum during laparoscopic abdominal surgery. Cochrane Database of Systematic Reviews. 2013, issue 1. Art. No.: CD009569. doi: 10.1002/14651858.CD009569.pub2.

88. Lu J, Cheng Y, Xiong X, Wu S, Lin Y, Wu T, et al. Gases for establishing pneumoperitoneum during laparoscopic abdominal surgery (Protocol). . Cochrane Database of Systematic Reviews. 2012, issue 1. Art. No.: CD009569. doi: 10.1002/14651858.CD009569.

89. Semple C, Parahoo K, Norman A, McCaughan E, Humphris G, Mills M. Psychosocial interventions for patients with head and neck cancer. Cochrane Database of Systematic Reviews. 2013, issue 7. Art. No.: CD009441. doi: 10.1002/14651858.CD009441.pub2.

90. Semple C, Parahoo K, Mills M, Humphris G, McCaughan E, Bessell A. Psychosocial interventions for patients with head and neck cancer (Protocol). Cochrane Database of Systematic Reviews 2011, issue 11. Art. No.: CD009441. doi: 10.1002/14651858.CD009441.

91. Sanders RD, Nicholson A, Lewis SR, Smith AF, Alderson P. Perioperative statin therapy for improving outcomes during and after noncardiac vascular surgery. Cochrane Database of Systematic Reviews. 2013, issue 7. Art. No.: CD009971. doi: 10.1002/14651858.CD009971.pub2.

92. Sanders RD, Nicholson A, Lewis SR, Smith AF, Alderson P. Perioperative statin therapy for improving outcomes during and after noncardiac vascular surgery (Protocol). . Cochrane Database of Systematic Reviews. 2012, issue 7. Art. No.: CD009971. doi: 10.1002/14651858.CD009971.

93. Yue J, Dong BR, Yang M, Chen X, Wu T, Liu GJ. Linezolid versus vancomycin for skin and soft tissue infections. Cochrane Database of Systematic Reviews. 2013, issue 7. Art. No.: CD008056. doi: 10.1002/14651858.CD008056.pub2.

94. Yue J, Dong BR, Yang M, Chen X, Wu T, Liu GJ. Linezolid versus vancomycin for skin and soft tissue infections (Protocol). Cochrane Database of Systematic Reviews 2009, issue 4. Art. No.: CD008056. doi: 10.1002/14651858.CD008056.

95. Mocellin S, Lens MB, Pasquali S, Pilati P, Chiarion SV. Interferon alpha for the adjuvant treatment of cutaneous melanoma. Cochrane Database of Systematic Reviews. 2013, issue 6. Art. No.: CD008955. doi: 10.1002/14651858.CD008955.pub2.

96. Mocellin S, Lens M, Pasquali S, Pilati P. Interferon alpha for the adjuvant treatment of cutaneous melanoma (Protocol). Cochrane Database of Systematic Reviews 2011, issue 1. Art. No.: CD008955. doi: 10.1002/14651858.CD008955.

97. Bruins Slot KMH, Berge E. Factor Xa inhibitors versus vitamin K antagonists for preventing cerebral or systemic embolism in patients with atrial fibrillation. Cochrane Database of Systematic Reviews. 2013, issue 8. Art. No.: CD008980. doi: 10.1002/14651858.CD008980.pub2.

98. Bruins Slot KMH, Berge E. Factor Xa inhibitors versus vitamin K antagonists for preventing cerebral or systemic embolism in patients with atrial fibrillation (Protocol). Cochrane Database of Systematic Reviews. 2011, issue 2. Art. No.: CD008980. doi: 10.1002/14651858.CD008980.

99. Itchaki G, Gafter-Gvili A, Lahav M, Vidal L, Raanani P, Shpilberg O, et al. Anthracycline-containing regimens for treatment of follicular lymphoma in adults. Cochrane Database of Systematic Reviews. 2013, issue 7. Art. No.: CD008909. doi: 10.1002/14651858.CD008909.pub2.

100. Itchaki G, Gafter-Gvili A, Lahav M, Vidal L, Raanani P, Shpilberg O, et al. Anthracyclines-containing regimens for treatment of follicular lymphoma in adults (Protocol). Cochrane Database of Systematic Reviews 2010, issue 12. Art. No.: CD008909. doi: 10.1002/14651858.CD008909.

101. Stead LF, Lancaster T. Behavioural interventions as adjuncts to pharmacotherapy for smoking cessation. Cochrane Database of Systematic Reviews. 2012, issue 12. Art. No.: CD009670. doi: 10.1002/14651858.CD009670.pub2.

102. Stead LF, Lancaster T. Behavioural interventions as adjuncts to pharmacotherapy for smoking cessation (Protocol). Cochrane Database of Systematic Reviews. 2012, issue 2. Art. No.: CD009670. doi: 10.1002/14651858.CD009670. .

103. Trotti LM, Bhadriraju S, Becker LA. Iron for restless legs syndrome. Cochrane Database of Systematic Reviews. 2012, issue 5. Art. No.: CD007834. doi: 10.1002/14651858.CD007834.pub2.

104. Trotti LM, Bhadriraju S, Becker LA. Iron for restless legs syndrome (Protocol). Cochrane Database of Systematic Reviews. 2009, issue 2. Art. No.: CD007834. doi: 10.1002/14651858.CD007834.
