# Supplementary material for: Exploring Treatment by Covariate Interactions Using Subgroup Analysis and Meta-Regression in Cochrane Reviews: A Review of Recent Practice
Source: PLoS One. 2015 Jun 1;10(6):e0128804. doi: 10.1371/journal.pone.0128804 (PMC4452239; doi:10.1371/journal.pone.0128804)
Supplement: S1 Table — (DOCX) [file pone.0128804.s003.docx]

**Table S1: Characteristics of reviews.**

| **Review** | | | | **Eligibility criteria^1^** | | | | | **Analysis^2^** | |  |
| --- | --- | --- | --- | --- | --- | --- | --- | --- | --- | --- | --- |
| **Review:** Aboumarzouk 2012 [[1](#_ENREF_1),[2](#_ENREF_2)]  **Review title:** Pregabalin for chronic prostatitis  **Review Group: Cochrane Prostatic Diseases and Urologic Cancers Group.**  **Date (issue):** Aug 2012 (8). | | | | **Studies:** Randomized controlled trials.  **Patients:** Participants were patients who had chronic prostatitis/chronic pelvic pain syndrome Classes IIIA or IIIB.  **Intervention type:** Drugs.  **Interventions:** Pregabalin versus placebo, an analgesia, or any other method of symptom relief.  **Outcome:** Improvement in men’s overall symptoms; primary; dichotomous; risk ratio; NT=1; NP=324. | | | | | **Meta-analysis in protocol:** Meta-analysis (presume pairwise).  **Reason if meta-analysis not planned:** NA.  **Meta-analysis in review:** No meta-analysis applied.  **Reason if meta-analysis not done and was planned:** One trial.  **Reason if meta-analysis done when not planned:** NA.  **Analysis type in protocol:** Not reported (presume Frequentist).  **Analysis type in review:** Not reported (presume Frequentist).  **Reason for changing mind**: NA.  **Data type in protocol:** Not reported (presume aggregate).  **Reason for choice in protocol:** Not reported.  **Data type in review:** Not reported (presume aggregate).  **Reason for choice in review:** Not reported.  **Reason for changing mind**: NA.  **Software in protocol:** RevMan.  **Software in review:** RevMan.  **Reason for changing mind**: NA.  **Missing data sought in protocol:** ‘The authors of papers with missing data (studies, outcomes, summary data, individuals, measures of variance, or study level characteristics) will be contacted for the missing data.’  **Missing data sought in review:** ‘The authors of the included study were not contacted for clarification of data or additional information as this was not needed.’  **Reason for changing mind**: Not reported. | |  |
| **Review:** Almeida 2013 [[3](#_ENREF_3),[4](#_ENREF_4)]  **Review title:** Conservative interventions for treating exercise-related musculotendinous, ligamentous and osseous groin pain  **Review Group:** Cochrane Bone, Joint and Muscle Trauma Group.  **Date (issue):** Jun 2013 (6). | | | | **Studies:** Randomized controlled trials and quasi-randomized controlled trials.  **Patients:** People with a diagnosis of exercise-related groin pain caused by pubic bone-related dysfunction, enthesopathy or muscles strains.  **Intervention type:** Non-pharmacological.  **Interventions:** Conservative intervention for exercise-related groin pain (single or complex intervention) versus placebo intervention; one conservative intervention (single or complex intervention) versus another conservative intervention (single or complex intervention).  **Outcome:** Pain relief at rest, on palpation and during physical activity (visual analogue scale, numeric rating scale, and others); primary; continuous; mean difference; NT=1; NP=25. | | | | | **Meta-analysis in protocol:** Meta-analysis (presume pairwise).  **Reason if meta-analysis not planned:** NA.  **Meta-analysis in review:** No meta-analysis applied.  **Reason if meta-analysis not done and was planned:** One trial.  **Reason if meta-analysis done when not planned:** NA.  **Analysis type in protocol:** Not reported (presume Frequentist).  **Analysis type in review:** Not reported (presume Frequentist).  **Reason for changing mind**: NA.  **Data type in protocol:** Not reported (presume aggregate).  **Reason for choice in protocol:** Not reported.  **Data type in review:** Not reported (presume aggregate).  **Reason for choice in review:** Not reported.  **Reason for changing mind**: NA.  **Software in protocol:** RevMan.  **Software in review:** RevMan.  **Reason for changing mind**: NA.  **Missing data sought in protocol:** ‘When necessary, requests will be sent to trial authors for additional information or data.’  **Missing data sought in review:** ‘When necessary, requests were sent to trial authors for additional information or data.’  **Reason for changing mind**: NA. | |  |
| **Review:** Basurto Ona 2013 [[5](#_ENREF_5),[6](#_ENREF_6)]  **Review title:** Opioids for acute pancreatitis pain  **Review Group:** Cochrane Upper Gastrointestinal and Pancreatic Diseases Group.  **Date (issue):** Jul 2013 (7). | | | | **Studies:** Randomised clinical trials with a parallel design (excluded quasi-randomised clinical trials and cross over trials).  **Patients:** Men or women, of any age, with abdominal pain due to acute pancreatitis.  **Intervention type:** Drugs.  **Interventions:** Opioids used as an analgesic drug at any dose, drug-release formulation or route of administration versus any other type of analgesic drug treatment, including other opioids, at any dose, drug-release formulation or route of administration.  **Outcome:** Number of participants showing improvements in pain intensity as defined by the trialist; primary; dichotomous; risk ratio; NT=1; NP=16. | | | | | **Meta-analysis in protocol:** Meta-analysis (presume pairwise).  **Reason if meta-analysis not planned:** NA.  **Meta-analysis in review:** No meta-analysis applied.  **Reason if meta-analysis not done and was planned:** One trial.  **Reason if meta-analysis done when not planned:** NA.  **Analysis type in protocol:** Not reported (presume Frequentist).  **Analysis type in review:** Not reported (presume Frequentist).  **Reason for changing mind**: NA.  **Data type in protocol:** Not reported (presume aggregate).  **Reason for choice in protocol:** Not reported.  **Data type in review:** Not reported (presume aggregate).  **Reason for choice in review:** Not reported.  **Reason for changing mind**: NA.  **Software in protocol:** Not reported (presume RevMan).  **Software in review:** RevMan.  **Reason for changing mind**: Not reported.  **Missing data sought in protocol:** ‘We will resolve any inadequacies or discrepancies between the extracted data by discussion and, if required, by contacting the study authors for further details.’  **Missing data sought in review:** ‘We resolved any inadequacies or discrepancies between the extracted data by discussion and if necessary by contacting the study authors for further details.’  **Reason for changing mind**: NA. | |  |
| **Review:** Bellmunt-Montoya 2013 [[7](#_ENREF_7),[8](#_ENREF_8)]  **Review title:** CHIVA method for the treatment of chronic venous insufficiency  **Review Group:** Cochrane Peripheral Vascular Diseases Group.  **Date (issue):** Jul 2013 (7). | | | | **Studies:** Randomised controlled trials.  **Patients:** Men and women over 18 years of age with varicose veins at stage C2-C6.  **Intervention type:** Surgical.  **Interventions:** CHIVA method compared with other proceduresto treat varicose veins, such as drugs, sclerotherapy, compressivedressings and other surgical methods.  **Outcome:** Recurrence of varicose veins; primary; dichotomous risk ratio; NT=4; NP=768. | | | | | **Meta-analysis in protocol:** Meta-analysis (presume pairwise).  **Reason if meta-analysis not planned:** NA.  **Meta-analysis in review:** pairwise meta-analysis applied.  **Reason if meta-analysis not done and was planned**: NA.  **Reason if meta-analysis done when not planned:** NA.  **Analysis type in protocol:** Not reported (presume Frequentist).  **Analysis type in review:** Not reported (presume Frequentist).  **Reason for changing mind**: NA.  **Data type in protocol:** Not reported (presume aggregate).  **Reason for choice in protocol:** Not reported.  **Data type in review:** Not reported (presume aggregate).  **Reason for choice in review:** Not reported.  **Reason for changing mind**: NA.  **Software in protocol:** RevMan.  **Software in review:** RevMan.  **Reason for changing mind**: NA.  **Missing data sought in protocol: ‘**We will contact study authors if more information is needed, as well as to clarify any doubts that may arise regarding missing data.’  **Missing data sought in review:** ‘We contacted study authors to obtain additional information.’  **Reason for changing mind**: NA. | |  |
| **Review:** Berlowitz 2013 [[9](#_ENREF_9),[10](#_ENREF_10)]  **Review title:** Respiratory muscle training for cervical spinal cord injury  **Review Group:** [Cochrane Injuries Group.](http://onlinelibrary.wiley.com/o/cochrane/clabout/articles/INJ/frame.html)  **Date (issue):** Jul 2013 (7). | | | | **Studies:** Randomised controlled trials.  **Patients:** People with any level of acquired cervical spinal cord injury, both acute and chronic.  **Intervention type:** Non-pharmacological.  **Interventions:** Respiratory muscle training compared with a control group using an alternative intervention, placebo, usual care, or no intervention.  **Outcome:** Respiratory complications; primary; continuous; number of episodes in each group reported; NT=1; NP=14. | | | | | **Meta-analysis in protocol:** Meta-analysis (presume pairwise).  **Reason if meta-analysis not planned:** NA.  **Meta-analysis in review:** No meta-analysis applied.  **Reason if meta-analysis not done and was planned:** One trial.  **Reason if meta-analysis done when not planned:** NA.  **Analysis type in protocol:** Not reported (presume Frequentist).  **Analysis type in review:** Not reported (presume Frequentist).  **Reason for changing mind**: NA.  **Data type in protocol:** Not reported (presume aggregate).  **Reason for choice in protocol:** Not reported.  **Data type in review:** Not reported (presume aggregate).  **Reason for choice in review:** Not reported.  **Reason for changing mind**: NA.  **Software in protocol:** RevMan.  **Software in review:** RevMan.  **Reason for changing mind**: NA.  **Missing data sought in protocol:** ‘Whenever possible, we will contact the author of each included trial to verify the accuracy of the data and, if possible, to obtain further data or information.’  **Missing data sought in review:** ‘Whenever possible, we contacted the author of each included trial to verify the accuracy of the data and, if possible, to obtain further data or information.’  **Reason for changing mind**: NA. | |  |
| **Review:** Boselie 2012 [[11](#_ENREF_11),[12](#_ENREF_12)]  **Review title:** Arthroplasty versus fusion in single-level cervical degenerative disc disease  **Review Group:** Cochrane Back Group.  **Date (issue):** Sept 2012 (9). | | | | **Studies:** Randomised controlled trials. Excluding quasi-randomised studies.  **Patients:** Skeletally mature patients with symptomatic single level cervical degenerative disc disease of C3-C4, C4-C5, C5-C6, or C6-C7.  **Intervention type:** Surgical.  **Interventions:** Single level anterior cervical discectomy with fusion (either by plate, cage, autograft, allograft material, or a combination) compared to anterior cervical discectomy with the placement of an artificial cervical disc.  **Outcome:** (Arm and neck) pain expressed on a visual analogue or similar scale; primary; continuous; mean difference; NT=6; NP=1,346 (for arm), NP=1,347 (for neck). | | | | | **Meta-analysis in protocol:** Meta-analysis (presume pairwise).  **Reason if meta-analysis not planned:** NA.  **Meta-analysis in review:** pairwise meta-analysis applied.  **Reason if meta-analysis not done and was planned**: NA.  **Reason if meta-analysis done when not planned:** NA.  **Analysis type in protocol:** Not reported (presume Frequentist).  **Analysis type in review:** Not reported (presume Frequentist).  **Reason for changing mind**: NA.  **Data type in protocol:** Not reported (presume aggregate).  **Reason for choice in protocol:** Not reported.  **Data type in review:** Not reported (presume aggregate).  **Reason for choice in review:** Not reported.  **Reason for changing mind**: NA.  **Software in protocol:** RevMan.  **Software in review:** RevMan.  **Reason for changing mind**: NA.  **Missing data sought in protocol:** ‘If possible, we will contact the original investigators to request the missing information.’  **Missing data sought in review:** ‘If possible, we contacted the original investigators to request any missing information.’  **Reason for changing mind**: NA. | |  |
| **Review:** Bruins Slot 2013 [[13](#_ENREF_13),[14](#_ENREF_14)]  **Review title:** Factor Xa inhibitors versus vitamin K antagonists for preventing cerebral or systemic embolism in patients with atrial fibrillation  **Review Group:** [Cochrane Stroke Group.](http://onlinelibrary.wiley.com/o/cochrane/clabout/articles/STROKE/frame.html)  **Date (issue):** Aug 2013 (8). | | | | **Studies:** Randomised clinical trials.  **Patients:** People with atrial fibrillation who were eligible for treatment with anticoagulants in order to reduce the risk of cerebral and systemic embolism.  **Intervention type:** Drugs.  **Interventions:** Treatment with an oral or parenteral factor Xa inhibitor (e.g. antistasin, apixaban, betrixaban, darexaban, DU176b, edoxaban, eribaxaban, fondaparinux, idraparinux, otamixaban, razaxaban, rivaroxaban, yagin, YM150, LY517717, SSR126517E) versus oral vitamin K antagonists (warfarin and congeners) with the intensity of anticoagulation dose-adjusted using the International Normalised Ratio (INR).  **Outcome:** The composite endpoint of all strokes (both ischaemic and haemorrhagic) and other systemic embolic events; primary; dichotomous; odds ratio; NT=9; NP=40,777. | | | | | **Meta-analysis in protocol:** Meta-analysis (presume pairwise).  **Reason if meta-analysis not planned:** NA.  **Meta-analysis in review:** pairwise meta-analysis applied.  **Reason if meta-analysis not done and was planned**: NA.  **Reason if meta-analysis done when not planned:** NA.  **Analysis type in protocol:** Not reported (presume Frequentist).  **Analysis type in review:** Not reported (presume Frequentist).  **Reason for changing mind**: NA.  **Data type in protocol:** Not reported (presume aggregate).  **Reason for choice in protocol:** Not reported.  **Data type in review:** Not reported (presume aggregate).  **Reason for choice in review:** Not reported.  **Reason for changing mind**: NA.  **Software in protocol:** RevMan.  **Software in review:** RevMan.  **Reason for changing mind**: NA.  **Missing data sought in protocol:** ‘If any relevant data are missing from the available publications, we will make direct contact with the principal investigators concerned.’  **Missing data sought in review:** ‘If any relevant data were missing from the available publications, we directly contacted the principal investigators or sponsor concerned, or both.’  **Reason for changing mind**: NA. | |  |
| **Review:** Cavalheri 2013 [[15](#_ENREF_15),[16](#_ENREF_16)]  **Review title:** Exercise training undertaken by people within 12 months of lung resection for non-small cell lung cancer  **Review Group:** Cochrane Lung Cancer Group.  **Date (issue):** Jul 2013 (7). | | | | **Studies:** Randomised controlled trials.  **Patients:** Participants following lung resection for non-small cell lung cancer.  **Intervention type:** Non-pharmacological.  **Interventions:** Exercise training versus usual care with either no exercise training or only instructions pertaining to exercise training.  **Outcome:** Any measure of exercise capacity including VO2peak and the six-minute walk distance; primary; continuous; mean difference; NT=3; NP=139. | | | | | **Meta-analysis in protocol:** Meta-analysis (presume pairwise).  **Reason if meta-analysis not planned:** NA.  **Meta-analysis in review:** pairwise meta-analysis applied.  **Reason if meta-analysis not done and was planned**: NA.  **Reason if meta-analysis done when not planned:** NA.  **Analysis type in protocol:** Not reported (presume Frequentist).  **Analysis type in review:** Not reported (presume Frequentist).  **Reason for changing mind**: NA.  **Data type in protocol:** Not reported (presume aggregate).  **Reason for choice in protocol:** Not reported.  **Data type in review:** Not reported (presume aggregate).  **Reason for choice in review:** Not reported.  **Reason for changing mind**: NA.  **Software in protocol:** RevMan.  **Software in review:** RevMan.  **Reason for changing mind**: NA.  **Missing data sought in protocol:** ‘We will try to contact the authors of any studies with missing data.’  **Missing data sought in review:** ‘We contacted the authors of all included studies to obtain missing data.’  **Reason for changing mind**: NA. | |  |
| **Review:** Chaparro 2013 [[17](#_ENREF_17),[18](#_ENREF_18)]  **Review title:** Pharmacotherapy for the prevention of chronic pain after surgery in adults  **Review Group:** [Cochrane Pain, Palliative and Supportive Care Group.](http://onlinelibrary.wiley.com/o/cochrane/clabout/articles/SYMPT/frame.html)  **Date (issue):** Jul 2013 (7). | | | | **Studies:** Double-blind, placebo-controlled, randomized trials.  **Patients:** Participants of both genders, 18 years of age and older, undergoing planned surgical procedures involving tissue injury.  **Intervention type:** Drugs.  **Interventions:** Drugs administered immediately before, during or after the procedure by any dose, route or frequency.  **Outcome:** Proportion of participants reporting any pain at the anatomical site of the procedure or pain referred to the surgical site, or both (for example phantom limb pain, shoulder pain referred from the diaphragm etc.), three months or more after the procedure; primary; dichotomous; risk ratio; NT=26; NP=2,865. | | | | | **Meta-analysis in protocol:** Meta-analysis (presume pairwise).  **Reason if meta-analysis not planned:** NA.  **Meta-analysis in review:** pairwise meta-analysis applied.  **Reason if meta-analysis not done and was planned**: NA.  **Reason if meta-analysis done when not planned:** NA.  **Analysis type in protocol:** Not reported (presume Frequentist).  **Analysis type in review:** Not reported (presume Frequentist).  **Reason for changing mind**: NA.  **Data type in protocol:** Not reported (presume aggregate).  **Reason for choice in protocol:** Not reported.  **Data type in review:** Not reported (presume aggregate).  **Reason for choice in review:** Not reported.  **Reason for changing mind**: NA.  **Software in protocol:** RevMan.  **Software in review:** RevMan.  **Reason for changing mind**: NA.  **Missing data sought in protocol:** Not reported.  **Missing data sought in review:** Not reported.  **Reason for changing mind**: NA. | |  |
| **Review:** Cheng 2013 [[19](#_ENREF_19),[20](#_ENREF_20)]  **Review title:** Gases for establishing pneumoperitoneum during laparoscopic abdominal surgery  **Review Group:** Cochrane Colorectal Cancer Group.  **Date (issue):** Jan 2013 (1). | | | | **Studies:** Randomised controlled trials (excluding quasi-randomised trials).  **Patients:** Patients who have undergone laparoscopic abdominal surgery under general anaesthesia performed by general surgeons.  **Intervention type:** Surgical.  **Interventions:** Laparoscopic abdominal surgery performed under standard pressure pneumoperitoneum with cold gas insufflation. ‘We will analysis the following gases for establishing pneumoperitoneum: (1) Carbon dioxide versus nitrous oxide; (2) Carbon dioxide versus helium; (3) Carbon dioxide versus argon; (4) Carbon dioxide versus nitrogen; (5) Carbon dioxide versus any other gas; (6) Any other gas (except carbon dioxide) versus any other gas (except carbon dioxide).  **Outcome:** Complications: (i) Cardiopulmonary complications (e.g., arrhythmia, ischemia, atelectasis, hypoxemia, pneumothorax, pulmonary edema), (ii) Procedure-related general complications (surgical morbidity); primary; dichotomous; risk ratio; NT=5; NP=268. | | | | | **Meta-analysis in protocol:** Meta-analysis (presume pairwise).  **Reason if meta-analysis not planned:** NA.  **Meta-analysis in review:** pairwise meta-analysis applied.  **Reason if meta-analysis not done and was planned**: NA.  **Reason if meta-analysis done when not planned:** NA.  **Analysis type in protocol:** Not reported (presume Frequentist).  **Analysis type in review:** Not reported (presume Frequentist).  **Reason for changing mind**: NA.  **Data type in protocol:** Not reported (presume aggregate).  **Reason for choice in protocol:** Not reported.  **Data type in review:** Not reported (presume aggregate).  **Reason for choice in review:** Not reported.  **Reason for changing mind**: NA.  **Software in protocol:** RevMan.  **Software in review:** RevMan.  **Reason for changing mind**: NA.  **Missing data sought in protocol:** ‘We will contact the original investigators to request further information in case of missing data.’  **Missing data sought in review:** ‘We contacted the original investigators to request further information in case of missing data.’  **Reason for changing mind**: NA. | |  |
| **Review:** Cruciani 2013 [[21](#_ENREF_21),[22](#_ENREF_22)]  **Review title:**Abacavir-based triple nucleoside regimens for maintenance therapy in patients with HIV  **Review Group:** Cochrane HIV/AIDS Group.  **Date (issue):** Jun 2013 (6). | | | | **Studies:** Randomised, controlled trials.  **Patients:** Chronically HIV-infected adult patients treated with a protease inhibitor –containing regimen (PI or boosted PI), with undetectable viral load. Study populations include patients starting a first antiretroviral PI-containing regimen as well as antiretroviral-experienced participants on a stable PI-containing regimen.  **Intervention type:** Drugs.  **Interventions:** Patients on a protease inhibitor -containing regimen had three possibilities: (1) Continue the protease inhibitor regimen or switch to a simplification maintenance regimen, including (2) Switch to a NNRTI (efavirenz-EFV- or nevirapine-NVP) containing regimen, or (3) Switch to a triple-NRTI regimen (ABC-AZT-3TC (Trizivir®)).  **Outcome:** Proportion of patients discontinuing or switching antiretroviral therapy due to virologic failure; primary; dichotomous; risk ratio; NT=8; NP=1,587. | | | | | **Meta-analysis in protocol:** Meta-analysis (presume pairwise).  **Reason if meta-analysis not planned:** NA.  **Meta-analysis in review:** pairwise meta-analysis applied.  **Reason if meta-analysis not done and was planned**: NA.  **Reason if meta-analysis done when not planned:** NA.  **Analysis type in protocol:** Not reported (presume Frequentist).  **Analysis type in review:** Not reported (presume Frequentist).  **Reason for changing mind**: NA.  **Data type in protocol:** Not reported (presume aggregate).  **Reason for choice in protocol:** Not reported.  **Data type in review:** Not reported (presume aggregate).  **Reason for choice in review:** Not reported.  **Reason for changing mind**: NA.  **Software in protocol:** RevMan.  **Software in review:** RevMan.  **Reason for changing mind**: NA.  **Missing data sought in protocol:** ‘We will contact the first or corresponding author of each included study for missing data or complementary information.’  **Missing data sought in review:** ‘When additional data were needed, we contacted the corresponding author of each study by e-mail in order to access further information.’  **Reason for changing mind**: NA. | |  |
| **Review:** Dashash 2013 [[23](#_ENREF_23),[24](#_ENREF_24)]  **Review title:** Interventions for the restorative care of amelogenesis imperfecta in children and adolescents  **Review Group:** [Cochrane Oral Health Group.](http://onlinelibrary.wiley.com/o/cochrane/clabout/articles/ORAL/frame.html)  **Date (issue):** Jun 2013 (6). | | | | **Studies:** Randomised controlled trials.  **Patients:** All children and adolescents under 18, who had amelogenesis imperfecta and required restorative care.  **Intervention type:** Techniques.  **Interventions:** Permanent restorative materials and techniques used for restoring teeth affected by amelogenesis imperfecta.  **Outcome:** Patient satisfaction due to reduced dental sensitivity and improved aesthetics; primary; dichotomous or continuous; risk ratio (dichotomous) or means and standard deviations (continuous) planned (no data); NT=0; NP=0. | | | | | **Meta-analysis in protocol:** Meta-analysis (presume pairwise).  **Reason if meta-analysis not planned:** NA.  **Meta-analysis in review:** No meta-analysis applied.  **Reason if meta-analysis not done and was planned:** No data.  **Reason if meta-analysis done when not planned:** NA.  **Analysis type in protocol:** Not reported (presume Frequentist).  **Analysis type in review:** Not reported (presume Frequentist).  **Reason for changing mind**: NA.  **Data type in protocol:** Not reported (presume aggregate).  **Reason for choice in protocol:** Not reported.  **Data type in review:** Not reported (presume aggregate).  **Reason for choice in review:** Not reported.  **Reason for changing mind**: NA.  **Software in protocol:** RevMan.  **Software in review:** Not reported (presume RevMan).  **Reason for changing mind**: Not reported.  **Missing data sought in protocol:** Not reported.  **Missing data sought in review:** Not reported.  **Reason for changing mind**: NA. | |  |
| **Review:** Deare 2013 [[25](#_ENREF_25),[26](#_ENREF_26)]  **Review title:** Acupuncture for treating fibromyalgia  **Review Group:** [Cochrane Musculoskeletal Group.](http://onlinelibrary.wiley.com/o/cochrane/clabout/articles/MUSKEL/frame.html)  **Date (issue):** May 2013 (5). | | | **Studies:** Randomised and quasi-randomised controlled clinical studies  **Patients:** Participants with a diagnosis of fibromyalgia.  **Intervention type:** Non-pharmacological.  **Interventions:** Any type of invasive acupuncture for fibromyalgia.  **Outcome:** Pain; main; continuous; mean difference and standardised mean difference; NT=9; NP=473. | | | **Meta-analysis in protocol:** Meta-analysis (presume pairwise).  **Reason if meta-analysis not planned:** NA.  **Meta-analysis in review:** pairwise meta-analysis applied.  **Reason if meta-analysis not done and was planned**: NA.  **Reason if meta-analysis done when not planned:** NA.  **Analysis type in protocol:** Not reported (presume Frequentist).  **Analysis type in review:** Not reported (presume Frequentist).  **Reason for changing mind**: NA.  **Data type in protocol:** Not reported (presume aggregate).  **Reason for choice in protocol:** Not reported.  **Data type in review:** Aggregate data (analysed in RevMan). Acknowledged two authors (Harris 2008 and Itoh 2010 ) for providing raw data.  **Reason for choice in review:** Not reported.  **Reason for changing mind:** Not reported.  **Software in protocol:** Not reported (presume RevMan).  **Software in review:** Not reported (presume RevMan).  **Reason for changing mind**: NA.  **Missing data sought in protocol:** Not reported.  **Missing data sought in review:** ‘We contacted the authors of the included/excluded articles to obtain further information.’  **Reason for changing mind**: Not reported. | | | | |  |
| **Review:** Freak-Poli 2013 [[27](#_ENREF_27),[28](#_ENREF_28)]  **Review title:** Workplace pedometer interventions for increasing physical activity  **Review Group:** [Cochrane Occupational Safety and Health Group.](http://onlinelibrary.wiley.com/o/cochrane/clabout/articles/OCCHEALTH/frame.html)  **Date (issue):** Apr 2013 (4). | | | **Studies:** Individual and cluster-randomised controlled trials  **Patients:** Employed adults.  **Intervention type:** Non-pharmacological.  **Interventions:** Workplace health promotion interventions with a pedometer component. ‘We will include all comparator groups in the review, including any  intervention without a pedometer, or no intervention.’  **Outcome:** Physical activity; primary; continuous; mean difference; NT=4; NP=1,809. | | | **Meta-analysis in protocol:** Meta-analysis (presume pairwise).  **Reason if meta-analysis not planned:** NA.  **Meta-analysis in review:** No meta-analysis applied.  **Reason if meta-analysis not done and was planned:** ‘We could not combine the results in a single meta-analysis because the studies differed too greatly in how they measured physical activity’  **Reason if meta-analysis done when not planned:** NA.  **Analysis type in protocol:** Not reported (presume Frequentist).  **Analysis type in review:** Not reported (presume Frequentist).  **Reason for changing mind**: NA.  **Data type in protocol:** Not reported (presume aggregate).  **Reason for choice in protocol:** Not reported.  **Data type in review:** Not reported (presume aggregate).  **Reason for choice in review:** Not reported.  **Reason for changing mind**: NA.  **Software in protocol:** RevMan, Excel.  **Software in review:** RevMan.  **Reason for changing mind**: Not reported.  **Missing data sought in protocol:** ‘Where information is missing from the included studies, we will contact the study authors to provide additional information.’  **Missing data sought in review:** ‘Where information was missing from the included studies, we contacted the study authors to provide additional information.’  **Reason for changing mind**: NA. | | | | |  |
| **Review:** Gan, 2013 [[29](#_ENREF_29),[30](#_ENREF_30)]  **Review title:** Chinese medicinal herbs for cholelithiasis  **Review Group:** [Cochrane Hepato-Biliary Group.](http://onlinelibrary.wiley.com/o/cochrane/clabout/articles/LIVER/frame.html)  **Date (issue):** Jun 2013 (6). | | | **Studies:** Parallel-group randomised clinical trials and the first phase of randomised cross-over trials.  **Patients:** Patients with cholelithiasis.  **Intervention type:** Herbal medicines.  **Interventions:** Chinese medicinal herbs (a single medicinal herb, i.e., patent herbal medicines or a medicinal herbal compound, i.e., self-made herbal compounds) versus placebo or no treatment, ursodeoxycholic acid, or another Western medicine (a product made by chemical synthesis or extraction in a pharmaceutical factory), or surgery.  **Outcome:** Mortality; primary; dichotomous; risk ratio planned (no data); NT=0; NP=0. | | **Meta-analysis in protocol:** Meta-analysis (presume pairwise).  **Reason if meta-analysis not planned:** NA.  **Meta-analysis in review:** No meta-analysis applied.  **Reason if meta-analysis not done and was planned:** No data.  **Reason if meta-analysis done when not planned:** NA.  **Analysis type in protocol:** Not reported (presume Frequentist).  **Analysis type in review:** Not reported (presume Frequentist).  **Reason for changing mind**: NA.  **Data type in protocol:** Not reported (presume aggregate).  **Reason for choice in protocol:** Not reported.  **Data type in review:** Not reported (presume aggregate).  **Reason for choice in review:** Not reported.  **Reason for changing mind**: NA.  **Software in protocol:** Not reported (presume RevMan).  **Software in review:** RevMan.  **Reason for changing mind**: Not reported.  **Missing data sought in protocol:** ‘The authors of the trials will be approached to specify the data, had they not been reported sufficiently in the article:’  **Missing data sought in review:** ‘The authors of the trials were queried to specify any data not sufficiently reported in the publication.’  **Reason for changing mind**: NA. | | | | | |  |
| **Review:** Gillies 2012 [[31](#_ENREF_31),[32](#_ENREF_32)]  **Review title:** Psychological therapies for the treatment of post‐traumatic stress disorder in children and adolescents  **Review Group:** [Cochrane Depression, Anxiety and Neurosis Group.](http://onlinelibrary.wiley.com/o/cochrane/clabout/articles/DEPRESSN/frame.html)  **Date (issue):** Dec 2012 (12). | | | **Studies:** Parallel, cross-over and cluster-randomised and quasi-randomised controlled trials.  **Patients:** Children or adolescents, aged 3 to 18 years, diagnosed with posttraumatic stress disorder.  **Intervention type:** Psychological.  **Interventions:** All psychological therapies including but not restricted to: cognitive behavioural therapy, exposure-based therapy, psychodynamic therapy, narrative therapy, supportive counselling, family-based therapy and eye movement desensitisation and reprocessing. Controls: control (treatment as usual, waiting list controls or no treatment), Another psychological therapy, pharmacological therapy, other treatments.  **Outcome:** Improvement from a diagnosis of post-traumatic stress disorder determined by accepted clinical diagnostic criteria such as the International  Classification of Diseases (ICD-10) or Diagnostic and Statistical Manual  of Mental Disorders text revision (DSM-IV TR); primary; dichotomous; odds ratio; NT=9; NP=540. | | | | | **Meta-analysis in protocol:** Meta-analysis (presume pairwise).  **Reason if meta-analysis not planned:** NA.  **Meta-analysis in review:** pairwise meta-analysis applied.  **Reason if meta-analysis not done and was planned**: NA.  **Reason if meta-analysis done when not planned:** NA.  **Analysis type in protocol:** Not reported (presume Frequentist).  **Analysis type in review:** Not reported (presume Frequentist).  **Reason for changing mind**: NA.  **Data type in protocol:** Not reported (presume aggregate).  **Reason for choice in protocol:** Not reported.  **Data type in review:** Not reported (presume aggregate).  **Reason for choice in review:** Not reported.  **Reason for changing mind**: NA.  **Software in protocol:** Not reported (presume RevMan).  **Software in review:** Not reported (presume RevMan).  **Reason for changing mind**: NA.  **Missing data sought in protocol:** ‘Where further clarification or missing data are needed from study authors, all reasonable attempts to contact the authors will be made.’  **Missing data sought in review:** ‘Where further clarification or missing data were needed from study authors, we made all reasonable attempts to contact the authors.’  **Reason for changing mind**: NA. | | |  |
| **Review:** Gois 2013 [[33](#_ENREF_33),[34](#_ENREF_34)]  **Review title:** Pharmacotherapy for hyperuricemia in hypertensive patients  **Review Group:** [Cochrane Hypertension Group.](http://onlinelibrary.wiley.com/o/cochrane/clabout/articles/HTN/frame.html)  **Date (issue):** Jan 2013 (1). | | | **Studies:** Published and non-published clinical trials with randomised or quasi-randomised allocation, including parallel or crossover studies with an adequate washout period of at least two weeks before crossover. Excluded cluster randomised trials.  **Patients:** Participants with primary hypertension.  **Intervention type:** Drugs.  **Interventions:** Uric acid lowering agents: either a xanthine-oxidase inhibitor or an uricosuric agent (for a minimum duration of four weeks) versus placebo.  **Outcome:** Change in casual or ambulatory, systolic and/or diastolic blood pressure; primary; continuous; mean difference (presented mean change from baseline for each group); NT=1; NP=30. | | | | | **Meta-analysis in protocol:** Meta-analysis (presume pairwise).  **Reason if meta-analysis not planned:** NA.  **Meta-analysis in review:** No meta-analysis applied.  **Reason if meta-analysis not done and was planned:** One trial.  **Reason if meta-analysis done when not planned:** NA.  **Analysis type in protocol:** Not reported (presume Frequentist).  **Analysis type in review:** Not reported (presume Frequentist).  **Reason for changing mind**: NA.  **Data type in protocol:** Not reported (presume aggregate).  **Reason for choice in protocol:** Not reported.  **Data type in review:** Not reported (presume aggregate).  **Reason for choice in review:** Not reported.  **Reason for changing mind**: NA.  **Software in protocol:** RevMan.  **Software in review:** Not reported (presume RevMan).  **Reason for changing mind**: Not reported.  **Missing data sought in protocol:** ‘the study authors will be contacted in order to obtain additional information or data.’  **Missing data sought in review:** ‘It was not necessary to contact the authors of the included study for additional information or data.’  **Reason for changing mind**: Not reported. | | |  |
| **Review:** Goldenberg 2013 [[35](#_ENREF_35),[36](#_ENREF_36)]  **Review title:** Probiotics for the prevention of Clostridium difficile-associated diarrhea in adults and children  **Review Group:** [Cochrane Inflammatory Bowel Disease and Functional Bowel Disorders Group.](http://onlinelibrary.wiley.com/o/cochrane/clabout/articles/IBD/frame.html)  **Date (issue):** May 2013 (5). | | | **Studies:** Randomized controlled trials.  **Patients:** Patients receiving antibiotic therapy for any reason.  **Intervention type:** Non-pharmacological.  **Interventions:** Probiotics versus placebo, alternative prophylaxis, or no treatment for the prevention of *C. difficile-*associated diarrhea.  **Outcome:** Incidence of *C. difficile-*associated diarrhea; primary;  dichotomous; risk ratio; NT=23; NP=4,213. | | | | | **Meta-analysis in protocol:** Meta-analysis (presume pairwise).  **Reason if meta-analysis not planned:** NA.  **Meta-analysis in review:** pairwise meta-analysis applied.  **Reason if meta-analysis not done and was planned**: NA.  **Reason if meta-analysis done when not planned:** NA.  **Analysis type in protocol:** Not reported (presume Frequentist).  **Analysis type in review:** Not reported (presume Frequentist).  **Reason for changing mind**: NA.  **Data type in protocol:** Not reported (presume aggregate).  **Reason for choice in protocol:** Not reported.  **Data type in review:** Not reported (presume aggregate).  **Reason for choice in review:** Not reported.  **Reason for changing mind**: NA.  **Software in protocol:** RevMan.  **Software in review:** RevMan and R.  **Reason for changing mind**: Not reported.  **Missing data sought in protocol:** ‘If needed, further information will be sought via contact with principle authors’.  **Missing data sought in review:** ‘For articles published in abstract form only, further information was sought by contacting principal authors.’  **Reason for changing mind**: NA. | | |  |
| **Review:** Gower 2013 [[37](#_ENREF_37),[38](#_ENREF_38)]  **Review title:** Perioperative antibiotics for prevention of acute endophthalmitis after cataract surgery  **Review Group:** [Cochrane Eyes and Vision Group.](http://onlinelibrary.wiley.com/o/cochrane/clabout/articles/EYES/frame.html)  **Date (issue):** Jul 2013 (7). | | | **Studies:** Randomized controlled trials.  **Patients:** Adults undergoing cataract surgery with any procedure for lens opacities due to any origin.  **Intervention type:** Drugs**.**  **Interventions:** We included trials evaluating preoperative antibiotics, intraoperative (intracameral, subconjunctival or systemic) or postoperative antibiotic prophylaxis for acute endophthalmitis. Comparisons of interest included: 1. Any prophylaxis versus no prophylaxis; 2. Preoperative versus postoperative or intraoperative prophylaxis or combinations; 3. Specific antibiotics used in included trials; 4. Mode of perioperative antibiotic delivery.  **Outcome:** Endophthalmitis: both presumed and culture-proven endophthalmitis within six weeks after cataract surgery. Our primary analysis was based on six-week outcomes; however, we also evaluated data from weeks one, two and four; primary; dichotomous; risk ratio; NT=4; NP=100,876. | | | | | **Meta-analysis in protocol:** Meta-analysis (presume pairwise).  **Reason if meta-analysis not planned:** NA.  **Meta-analysis in review:** No meta-analysis applied.  **Reason if meta-analysis not done and was planned:** ‘Interventions differed between them. Given the heterogeneity of study designs and modes of antibiotic delivery, we decided against conducting meta-analysis.’  **Reason if meta-analysis done when not planned:** NA.  **Analysis type in protocol:** Not reported (presume Frequentist).  **Analysis type in review:** Not reported (presume Frequentist).  **Reason for changing mind**: NA.  **Data type in protocol:** Not reported (presume aggregate).  **Reason for choice in protocol:** Not reported.  **Data type in review:** Not reported (presume aggregate).  **Reason for choice in review:** Not reported.  **Reason for changing mind**: NA.  **Software in protocol:** RevMan.  **Software in review:** RevMan.  **Reason for changing mind**: NA.  **Missing data sought in protocol:** ‘Primary investigators will be contacted for information regarding missing data.’  **Missing data sought in review:** ‘In the event of missing or unclear data, we contacted the primary investigators for additional information.’  **Reason for changing mind**: NA. | | |  |
| **Review:** He 2013 [[39](#_ENREF_39),[40](#_ENREF_40)]  **Review title:** Laquinimod for multiple sclerosis  **Review Group:** [Cochrane Multiple Sclerosis and Rare Diseases of the Central Nervous System Group.](http://onlinelibrary.wiley.com/o/cochrane/clabout/articles/MS/frame.html)  **Date (issue):** Aug 2013 (8). | | | **Studies:** All randomised, double-blind, controlled, parallel group clinical trials with a length of follow-up longer than one year. Excluded quasi-randomised trials.  **Patients:** Patients with multiple sclerosis.  **Intervention type:** Drugs.  **Interventions:** Laquinimod, orally as monotherapy or combination therapy, versus placebo or approved disease-modifying drugs.  **Outcome:** The annualised rate of relapse at one year or later; primary; continuous; mean and standard deviation by group reported with p-value; NT=1; NP=1,106. | | | | | **Meta-analysis in protocol:** Meta-analysis (presume pairwise).  **Reason if meta-analysis not planned:** NA.  **Meta-analysis in review:** No meta-analysis applied.  **Reason if meta-analysis not done and was planned:** One trial.  **Reason if meta-analysis done when not planned:** NA.  **Analysis type in protocol:** Not reported (presume Frequentist).  **Analysis type in review:** Not reported (presume Frequentist).  **Reason for changing mind**: NA.  **Data type in protocol:** Not reported (presume aggregate).  **Reason for choice in protocol:** Not reported.  **Data type in review:** Not reported (presume aggregate).  **Reason for choice in review:** Not reported.  **Reason for changing mind**: NA.  **Software in protocol:** RevMan.  **Software in review:** Reported RevMan analysis software not used (presume RevMan used).  **Reason for changing mind**: Not reported.  **Missing data sought in protocol:** ‘We will contact principal investigators of included studies to obtain additional data or confirmation of methodological aspects of the study.’  **Missing data sought in review:** ‘Principal investigators of included studies were contacted so that they could provide additional data or confirmation of methodological aspects of the study.’  **Reason for changing mind**: NA. | | |  |
| **Review:** Itchaki 2013 [[41](#_ENREF_41),[42](#_ENREF_42)]  **Review title:** Anthracycline-containing regimens for treatment of follicular lymphoma in adults  **Review Group:** [Cochrane Haematological Malignancies Group.](http://onlinelibrary.wiley.com/o/cochrane/clabout/articles/HAEMATOL/frame.html)  **Date (issue):** Jul 2013 (7). | | | **Studies:** Randomized controlled  Trials.  **Patients:** Adult patients with follicular lymphoma.  **Intervention type:** Drugs.  **Interventions:** Anthracycline-containing regimens compared to non- anthracycline-containing regimens.  **Outcome:** Overall survival; primary; time to event; hazard ratio; NT=5; NP=949. | | | | | **Meta-analysis in protocol:** Meta-analysis (presume pairwise).  **Reason if meta-analysis not planned:** NA.  **Meta-analysis in review:** pairwise meta-analysis applied.  **Reason if meta-analysis not done and was planned**: NA.  **Reason if meta-analysis done when not planned:** NA.  **Analysis type in protocol:** Not reported (presume Frequentist).  **Analysis type in review:** Not reported (presume Frequentist).  **Reason for changing mind**: NA.  **Data type in protocol:** Not reported (presume aggregate).  **Reason for choice in protocol:** Not reported.  **Data type in review:** Not reported (presume aggregate).  **Reason for choice in review:** Not reported.  **Reason for changing mind**: NA.  **Software in protocol:** RevMan and Comprehensive Meta-analysis.  **Software in review:** RevMan.  **Reason for changing mind**: Not reported.  **Missing data sought in protocol:** ‘We will try to complement missing data regarding review-defined outcomes and risk of bias assessment by correspondence with trial authors.’  **Missing data sought in review:** ‘We tried to complement missing data regarding review-defined outcomes and risk of bias assessment by correspondence with trial authors. None of the authors replied or could complete missing data.’  **Reason for changing mind**: NA. | | |  |
| **Review:** Kinnersley 2013 [[43](#_ENREF_43),[44](#_ENREF_44)]  **Review title:** Interventions to promote informed consent for patients undergoing surgical and other invasive healthcare procedures  **Review Group:** Cochrane Consumers and Communication Group.  **Date (issue):** Jul 2013 (7). | | | **Studies:** Randomised controlled trials including cluster randomised trials.  **Patients:** Patients aged 16 years and over being asked to give consent for a surgical or other invasive healthcare treatment or procedure, either for themselves, or on behalf of a minor or someone else for whom they have responsibility.  **Intervention type:** Non-pharmacological.  **Interventions:** Interventions to promote informed consent for patients undergoing surgical and other invasive healthcare procedures versus usual care.  **Outcome:** Informed consent; primary; continuous; mean difference; NT=1; NP=97. | | | | | **Meta-analysis in protocol:** Meta-analysis (presume pairwise).  **Reason if meta-analysis not planned:** NA.  **Meta-analysis in review:** No meta-analysis applied.  **Reason if meta-analysis not done and was planned:** One trial.  **Reason if meta-analysis done when not planned:** NA.  **Analysis type in protocol:** Not reported (presume Frequentist).  **Analysis type in review:** Not reported (presume Frequentist).  **Reason for changing mind**: NA.  **Data type in protocol:** Not reported (presume aggregate).  **Reason for choice in protocol:** Not reported.  **Data type in review:** Not reported (presume aggregate).  **Reason for choice in review:** Not reported.  **Reason for changing mind**: NA.  **Software in protocol:** RevMan.  **Software in review:** RevMan.  **Reason for changing mind**: NA.  **Missing data sought in protocol:** ‘In the case of missing data we will try to contact the authors of the studies by email to obtain the relevant information.’  **Missing data sought in review:** ‘In cases of missing data we tried to contact the authors of the studies by email to obtain the relevant information.’  **Reason for changing mind**: NA. | | |  |
| **Review:** Lawrie 2013 [[45](#_ENREF_45),[46](#_ENREF_46)]  **Review title:** Pegylated liposomal doxorubicin for relapsed epithelial ovarian cancer  **Review Group:** [Cochrane Gynaecological Cancer Group.](http://onlinelibrary.wiley.com/o/cochrane/clabout/articles/GYNAECA/frame.html)  **Date (issue):** Jul 2013 (7). | | | **Studies:** Randomised controlled trials.  **Patients:** Women with relapsed epithelial ovarian cancer of any stage, including patients with both platinum-sensitive and platinum-resistant disease.  **Intervention type**: Drugs.  **Interventions:** (1) Pegylated liposomal doxorubicin in combination with platinum-based therapy versus platinum-based therapy with another agent; (2) other chemotherapy agent(s) versus pegylated liposomal doxorubicin; (3) pegylated liposomal doxorubicin plus other agent(s) versus pegylated liposomal doxorubicin alone or with placebo.  **Outcome:** Progression-free survival; primary; time to event; hazard ratio; NT=13; NP=4,625. | | | | | **Meta-analysis in protocol:** Meta-analysis (presume pairwise).  **Reason if meta-analysis not planned:** NA.  **Meta-analysis in review:** pairwise meta-analysis applied.  **Reason if meta-analysis not done and was planned**: NA.  **Reason if meta-analysis done when not planned:** NA.  **Analysis type in protocol:** Not reported (presume Frequentist).  **Analysis type in review:** Not reported (presume Frequentist).  **Reason for changing mind**: NA.  **Data type in protocol:** Not reported (presume aggregate).  **Reason for choice in protocol:** Not reported.  **Data type in review:** Aggregate data and individual patient data. For the primary outcome there was two studies, ‘We estimated HRs from the raw data of one study’.  **Reason for choice in review:** Not reported.  **Reason for changing mind:** Not reported.  **Software in protocol:** RevMan.  **Software in review:** RevMan.  **Reason for changing mind**: NA.  **Missing data sought in protocol:** ‘Where possible, missing data will be sought from the authors.’  **Missing data sought in review:** Not reported.  **Reason for changing mind**: Not reported. | | |  |
| **Review:** Lee 2013 [[47](#_ENREF_47),[48](#_ENREF_48)]  **Review title:** Airway clearance techniques for bronchiectasis  **Review Group:** Cochrane Airways Group.  **Date (issue):** May 2013 (5). | | | **Studies:** Randomised controlled trials (parallel or crossover).  **Patients:** Individuals with acute or stable bronchiectasis.  **Intervention type:** Technique.  **Interventions:** Prescribed regimen of an airway clearance techniques is compared to no intervention, sham intervention or coughing alone.  **Outcome:** Rate of, duration of, or time to acute exacerbation of bronchiectasis; primary; dichotomous and continuous; number in each group and p-value reported (for rate), mean differences or standardised mean differences planned (for continuous); NT=1; NP=20 (for rate); NT=0; NP=0 (for duration and time to). | | | | | **Meta-analysis in protocol:** Meta-analysis (presume pairwise).  **Reason if meta-analysis not planned:** NA.  **Meta-analysis in review:** No meta-analysis applied.  **Reason if meta-analysis not done and was planned:** One trial for ‘rate’, no trials for ‘duration of, or time to’.  **Reason if meta-analysis done when not planned:** NA.  **Analysis type in protocol:** Not reported (presume Frequentist).  **Analysis type in review:** Not reported (presume Frequentist).  **Reason for changing mind**: NA.  **Data type in protocol:** Not reported (presume aggregate).  **Reason for choice in protocol:** Not reported.  **Data type in review:** Not reported (presume aggregate).  **Reason for choice in review:** Not reported.  **Reason for changing mind**: NA.  **Software in protocol:** RevMan.  **Software in review:** RevMan.  **Reason for changing mind**: NA.  **Missing data sought in protocol:** ‘Authors of included studies will be asked to verify the data extracted for their study and to provide details of missing data where applicable.’  **Missing data sought in review:** ‘We contacted authors of included studies to verify the extracted data for their study where possible and to provide details of missing data where applicable.’  **Reason for changing mind**: NA. | | |  |
| **Review:** Li 2013 [[49](#_ENREF_49),[50](#_ENREF_50)]  **Review title:** Acetaminophen (paracetamol) for the common cold in adults  **Review Group:** Cochrane Acute Respiratory Infections Group.  **Date (issue):** Jul 2013 (7). | | | **Studies:** Randomised controlled trials.  **Patients:** Participants with the common cold, aged 12 years or older.  **Intervention type:** Drugs.  **Interventions:** Acetaminophen (alone or in combination with other ingredients) versus placebo or no treatment.  **Outcome:** Subjective symptom score; primary; continuous; mean change from baseline in each group (1 trial), p-value comparing the two groups (2 trials), narrative overview (1 trial); NT=4; NP=758. | | | | | **Meta-analysis in protocol:** Meta-analysis (presume pairwise).  **Reason if meta-analysis not planned:** NA.  **Meta-analysis in review:** No meta-analysis applied.  **Reason if meta-analysis not done and was planned:** ‘We did not pool data because of heterogeneity in study design, outcomes and time points.’  **Reason if meta-analysis done when not planned:** NA.  **Analysis type in protocol:** Not reported (presume Frequentist).  **Analysis type in review:** Not reported (presume Frequentist).  **Reason for changing mind**: NA.  **Data type in protocol:** Not reported (presume aggregate).  **Reason for choice in protocol:** Not reported.  **Data type in review:** Not reported (presume aggregate).  **Reason for choice in review:** Not reported.  **Reason for changing mind**: NA.  **Software in protocol:** RevMan.  **Software in review:** RevMan.  **Reason for changing mind**: NA.  **Missing data sought in protocol:** Not reported.  **Missing data sought in review:** Not reported.  **Reason for changing mind**: NA. | | |  |
| **Review:** Liu 2013 [[51](#_ENREF_51),[52](#_ENREF_52)]  **Review title:** Chinese herbal medicines for hypertriglyceridaemia  **Review Group:** [Cochrane Metabolic and Endocrine Disorders Group.](http://onlinelibrary.wiley.com/o/cochrane/clabout/articles/ENDOC/frame.html)  **Date (issue):** Jun 2013 (6). | | | **Studies:** Randomised controlled clinical trials with an adequate method of random sequence generation.  **Patients:** Adults (18 years or older) with hypertriglyceridaemia.  **Intervention type:** Herbal medicines.  **Intervention:** Chinese herbal medicines (including medicines from mixtures of herbs, single herbs, Chinese proprietary medicines, or a formula of herbs prescribed by a Chinese medicine practitioner). **Control:** No treatment, placebo, non-traditional Chinese active agents.  **Outcome:** Cardiovascular and cerebrovascular events; primary; dichotomous; risk ratio planned (no data); NT=0; NP=0. | | | | | **Meta-analysis in protocol:** Meta-analysis (presume pairwise).  **Reason if meta-analysis not planned:** NA.  **Meta-analysis in review:** No meta-analysis applied.  **Reason if meta-analysis not done and was planned:** No data.  **Reason if meta-analysis done when not planned:** NA.  **Analysis type in protocol:** Not reported (presume Frequentist).  **Analysis type in review:** Not reported (presume Frequentist).  **Reason for changing mind**: NA.  **Data type in protocol:** Not reported (presume aggregate).  **Reason for choice in protocol:** Not reported.  **Data type in review:** Not reported (presume aggregate).  **Reason for choice in review:** Not reported.  **Reason for changing mind**: NA.  **Software in protocol:** Not reported (presume RevMan).  **Software in review:** Not reported (presume RevMan).  **Reason for changing mind**: NA.  **Missing data sought in protocol:** ‘Thereafter, we will seek relevant missing information on the trial from the original author(s) of the article, if required.’  **Missing data sought in review:** ‘Thereafter, we planned to seek relevant missing information on the trial from the original author(s) of the article, if required.’  **Reason for changing mind**: NA. | | |  |
| **Review:** Lopez 2013 [[53](#_ENREF_53),[54](#_ENREF_54)]  **Review title:** Behavioral interventions for improving contraceptive use among women living with HIV  **Review Group:** [Cochrane Fertility Regulation Group.](http://onlinelibrary.wiley.com/o/cochrane/clabout/articles/FERTILREG/frame.html)  **Date (issue):** Jan 2013 (1). | **Studies:** Randomised controlled trials (individually randomized or cluster randomized) and non-randomized (observational) studies (had to be comparative).  **Patients:** Women who are HIV-positive and of reproductive age.  **Intervention type:** Non-pharmacological.  **Interventions:** Behavioural intervention for improving contraceptive use versus another behavioural intervention, usual care, or no intervention.  **Outcome:** Contraception use, e.g., choice, uptake, or initiation of a new method; improved use or continuation of current method; primary; dichotomous and count; odds ratio, rate ratio and risk difference; NT=7; NP= 10,311. | | | | **Meta-analysis in protocol:** Meta-analysis (presume pairwise).  **Reason if meta-analysis not planned:** NA.  **Meta-analysis in review:** No meta-analysis applied.  **Reason if meta-analysis not done and was planned:** ‘Given the diversity of design features with observational studies, we did not conduct meta-analysis for pooled estimates.’  **Reason if meta-analysis done when not planned:** NA.  **Analysis type in protocol:** Not reported (presume Frequentist).  **Analysis type in review:** Not reported (presume Frequentist).  **Reason for changing mind**: NA.  **Data type in protocol:** Not reported (presume aggregate).  **Reason for choice in protocol:** Not reported.  **Data type in review:** Not reported (presume aggregate).  **Reason for choice in review:** Not reported.  **Reason for changing mind**: NA.  **Software in protocol:** RevMan.  **Software in review:** RevMan.  **Reason for changing mind**: NA.  **Missing data sought in protocol:** ‘If reports are missing data needed for analysis, we will write to the study researchers. However, we will limit our data requests to studies less than 10 years old. Researchers are unlikely to have access to data for older studies.’  **Missing data sought in review:** ‘If reports were missing data needed for analysis, we wrote to the study researchers.’ ‘We limited our data requests to studies less than 10 years old. Researchers are unlikely to have access to data for older studies.’  **Reason for changing mind**: NA. | | | | | |  |
| **Review:** Marigold 2013 [[55](#_ENREF_55),[56](#_ENREF_56)]  **Review title:** Antiepileptic drugs for the primary and secondary prevention of seizures after subarachnoid haemorrhage  **Review Group:** [Cochrane Epilepsy Group.](http://onlinelibrary.wiley.com/o/cochrane/clabout/articles/EPILEPSY/frame.html)  **Date (issue):** Jun 2013 (6). | **Studies:** Randomised and quasi-randomised controlled trials.  **Patients:** Patients with a diagnosis of subarachnoid haemorrhage, regardless of whether they have or have not had post- subarachnoid haemorrhage seizures.  **Intervention type:** Drugs.  **Interventions:** At least one Antiepileptic drug (i.e. carbamazepine, clobazam, clonazepam, diazepam, ethosuximide, gabapentin, lamotrigine, levetiracetam, lorazepam, oxcarbazepine, phenytoin, phenobarbitone, primidone, sodiumvalproate, tiagabine, topiramate, vigabatrin and zonisamide) versus placebo or no treatment.  **Outcome:** Proportion of patients who experienced clinical seizures in the scheduled follow-up period; primary; dichotomous; odds ratio or risk ratio planned (no data); NT=0; NP=0. | | | | **Meta-analysis in protocol:** Meta-analysis (presume pairwise).  **Reason if meta-analysis not planned:** NA.  **Meta-analysis in review:** No meta-analysis applied.  **Reason if meta-analysis not done and was planned:** No data.  **Reason if meta-analysis done when not planned:** NA.  **Analysis type in protocol:** Not reported (presume Frequentist).  **Analysis type in review:** Not reported (presume Frequentist).  **Reason for changing mind**: NA.  **Data type in protocol:** Not reported (presume aggregate).  **Reason for choice in protocol:** Not reported.  **Data type in review:** Not reported (presume aggregate).  **Reason for choice in review:** Not reported.  **Reason for changing mind**: NA.  **Software in protocol:** Not reported (presume RevMan).  **Software in review:** Not reported (presume RevMan).  **Reason for changing mind**: NA.  **Missing data sought in protocol:** ‘we will attempt to contact the study investigators when additional outcome data are needed.’  **Missing data sought in review:** Not reported.  **Reason for changing mind:** Not reported. | | | | | |  |
| **Review:** Mocellin 2013 [[57](#_ENREF_57),[58](#_ENREF_58)]  **Review title:** Interferon alpha for the adjuvant treatment of cutaneous melanoma  **Review Group:** Cochrane Skin Group.  **Date (issue):** Jun 2013 (6). | | **Studies:** Randomised controlled trials.  **Patients:** People with high-risk skin melanoma, that is, those with regional lymph node metastasis undergoing radical lymph node dissection (American Joint Committee on Cancer stage III), or people without nodal disease but with primary tumour thickness greater than 1 mm (American Joint Committee on Cancer stage II).  **Intervention type:** Drugs.  **Interventions:** Adjuvant (i.e. postoperative) interferon (experimental arm) versus observation or any treatment other than interferon (control arm).  **Outcome:** Disease-free survival; primary; time to event; hazard ratio; NT=17; NP=10,345. | | | | | | **Meta-analysis in protocol:** Meta-analysis (presume pairwise).  **Reason if meta-analysis not planned:** NA.  **Meta-analysis in review:** pairwise meta-analysis applied.  **Reason if meta-analysis not done and was planned**: NA.  **Reason if meta-analysis done when not planned:** NA.  **Analysis type in protocol:** Not reported (presume Frequentist).  **Analysis type in review:** Not reported (presume Frequentist).  **Reason for changing mind**: NA.  **Data type in protocol:** Not reported (presume aggregate).  **Reason for choice in protocol:** Not reported.  **Data type in review:** Not reported (presume aggregate).  **Reason for choice in review:** Not reported.  **Reason for changing mind**: NA.  **Software in protocol:** RevMan.  **Software in review:** RevMan.  **Reason for changing mind**: NA.  **Missing data sought in protocol:** ‘We shall contact trial authors of included published studies whenever data essential for the meta-analysis is missing or unclear.’  **Missing data sought in review:** ‘We contacted trial authors of included studies whenever data essential for the meta-analysis were missing or unclear.’  **Reason for changing mind**: NA. | | |  |
| **Review:** Mutua 2012 [[59](#_ENREF_59),[60](#_ENREF_60)]  **Review title:** Genital ulcer disease treatment for reducing sexual acquisition of HIV Review Group: [**Cochrane Sexually Transmitted Infections Group.**](http://onlinelibrary.wiley.com/o/cochrane/clabout/articles/STI/sect0-meta.html) **Date (issue):** Aug 2012 (8). | | **Studies:** Randomized controlled trials in which the unit of randomization  was the individual.  **Patients:** Sexually active men and women with confirmed genital ulcer disease caused by a curable sexually transmitted infection, who were confirmed to be HIV-negative.  **Intervention type:** Drugs.  **Interventions:** Any treatment intervention aimed at curing genital ulcer disease compared with an alternative treatment, a placebo, or no treatment.  **Outcome:** Incidence of HIV infection; primary; dichotomous; risk ratio; NT=3; NP=173. | | | | | | **Meta-analysis in protocol:** Meta-analysis (presume pairwise).  **Reason if meta-analysis not planned:** NA.  **Meta-analysis in review:** No meta-analysis applied.  **Reason if meta-analysis not done and was planned:** One trial focussed on chancroid and the other on syphilis (results were stratified for each group). Each trial made a different comparison and results were stratified by comparison.  **Reason if meta-analysis done when not planned:** NA.  **Analysis type in protocol:** Not reported (presume Frequentist).  **Analysis type in review:** Not reported (presume Frequentist).  **Reason for changing mind**: NA.  **Data type in protocol:** Not reported (presume aggregate).  **Reason for choice in protocol:** Not reported.  **Data type in review:** Not reported (presume aggregate).  **Reason for choice in review:** Not reported.  **Reason for changing mind**: NA.  **Software in protocol:** RevMan.  **Software in review:** Not reported (presume RevMan).  **Reason for changing mind**: Not reported.  **Missing data sought in protocol:** ‘Where information is unclear or missing, we will contact the trial authors for clarification.’  **Missing data sought in review:** ‘All authors of eligible studies were contacted for additional data such as HIV status.’  **Reason for changing mind**: NA. | | |  |
| **Review:** Parker 2013 [[61](#_ENREF_61),[62](#_ENREF_62)]  **Review title:** Psychoanalytic/psychodynamic psychotherapy for children and adolescents who have been sexually abused  **Review Group:** [Cochrane Developmental, Psychosocial and Learning Problems Group.](http://onlinelibrary.wiley.com/o/cochrane/clabout/articles/BEHAV/frame.html)  **Date (issue):** Jul 2013 (7). | | **Studies:** Randomised trials or quasi-randomised trials.  **Patients:** Symptomatic children and adolescents up to age 18 years who have experienced sexual abuse at any time prior to the intervention.  **Intervention type:** Psychotherapy.  **Interventions:** Psychoanalytic or psychodynamic psychotherapy versus treatment as usual (eg treatment by a psychiatrist) or versus no treatment control/ waiting list control.  **Outcome:** Post traumatic stress disorder; primary; dichotomous; relative risk planned (no data); NT=0; NP=0. | | | | | | **Meta-analysis in protocol:** Meta-analysis (presume pairwise).  **Reason if meta-analysis not planned:** NA.  **Meta-analysis in review:** No meta-analysis applied.  **Reason if meta-analysis not done and was planned:** No data.  **Reason if meta-analysis done when not planned:** NA.  **Analysis type in protocol:** Not reported (presume Frequentist).  **Analysis type in review:** Not reported (presume Frequentist).  **Reason for changing mind**: NA.  **Data type in protocol:** Not reported (presume aggregate).  **Reason for choice in protocol:** Not reported.  **Data type in review:** Not reported (presume aggregate).  **Reason for choice in review:** Not reported.  **Reason for changing mind**: NA.  **Software in protocol:** RevMan.  **Software in review:** RevMan.  **Reason for changing mind**: NA.  **Missing data sought in protocol:** ‘if necessary, the authors of the included studies will be contacted to gather further information.’  **Missing data sought in review:** Not reported.  **Reason for changing mind:** Not reported. | | |  |
| **Review:** Pega 2013 [[63](#_ENREF_63),[64](#_ENREF_64)]  **Review title:** In-work tax credits for families and their impact on health status in adults  **Review Group:** [Cochrane Public Health Group.](http://onlinelibrary.wiley.com/o/cochrane/clabout/articles/PUBHLTH/frame.html)  **Date (issue):** Aug 2013 (8). | | **Studies:** Randomised and quasi-randomised controlled trials and cohort, controlled before-and-after and interrupted time series studies.  **Patients:** Working age adults.  **Intervention type:** Non-pharmacological.  **Interventions:** In-work tax credits for families.  **Outcome:** Self rated general health; primary; dichotomous; risk difference; NT=1; NP=127,209. | | | | | **Meta-analysis in protocol:** Meta-analysis (presume pairwise).  **Reason if meta-analysis not planned:** NA.  **Meta-analysis in review:** No meta-analysis applied.  **Reason if meta-analysis not done and was planned:** One trial.  **Reason if meta-analysis done when not planned:** NA.  **Analysis type in protocol:** Not reported (presume Frequentist).  **Analysis type in review:** Not reported (presume Frequentist).  **Reason for changing mind**: NA.  **Data type in protocol:** Not reported (presume aggregate).  **Reason for choice in protocol:** Not reported.  **Data type in review:** Not reported (presume aggregate).  **Reason for choice in review:** Not reported.  **Reason for changing mind**: NA.  **Software in protocol:** RevMan.  **Software in review:** RevMan.  **Reason for changing mind**: NA.  **Missing data sought in protocol:** ‘Any of the missing information required for this review will be requested from the principal study authors by e-mail or phone.’  **Missing data sought in review:** ‘We requested all relevant missing information on the study methods, outcomes and statistical measures required for this review from the principal study authors by e-mail. For the included studies, we requested detailed information on the following missing data: • individuals missing from the study due to survey nonresponse; • missing outcome, exposure and covariate data for each survey or at each survey wave; • risk ratio measures; and • subgroup analyses for all characteristics, for which we planned to present subgroup analyses (that is, ethnicity, family type, gender, income).’  **Reason for changing mind**: Not reported. | | | |  |
| **Review:** Penninga 2013 [[65](#_ENREF_65),[66](#_ENREF_66)]  **Review title:** Tacrolimus versus cyclosporin as primary immunosuppression for lung transplant recipients  **Review Group:** Cochrane Renal Group.  **Date (issue):** May 2013 (5). | | **Studies:** Randomised controlled trials.  **Patients:** Adult and paediatric patients after first-time single or double lung transplantation.  **Intervention type:** Drugs.  **Interventions:** Any dose and duration of administration of tacrolimus versus cyclosporin.  **Outcome:** Mortality; primary; dichotomous; risk ratio; NT=3; NP=413. | | | | | **Meta-analysis in protocol:** Meta-analysis (presume pairwise).  **Reason if meta-analysis not planned:** NA.  **Meta-analysis in review:** pairwise meta-analysis applied.  **Reason if meta-analysis not done and was planned**: NA.  **Reason if meta-analysis done when not planned:** NA.  **Analysis type in protocol:** Not reported (presume Frequentist).  **Analysis type in review:** Not reported (presume Frequentist).  **Reason for changing mind**: NA.  **Data type in protocol:** Not reported (presume aggregate).  **Reason for choice in protocol:** Not reported.  **Data type in review:** Not reported (presume aggregate).  **Reason for choice in review:** Not reported.  **Reason for changing mind**: NA.  **Software in protocol:** RevMan.  **Software in review:** RevMan.  **Reason for changing mind**: NA.  **Missing data sought in protocol:** ‘Any further information required from the original author will be requested by written correspondence and any relevant information obtained in this manner will be included in the review.’  **Missing data sought in review:** ‘Any further information required from the original authors was requested by written correspondence and any relevant information obtained in this manner was included in the review.’  **Reason for changing mind**: NA. | | | |  |
| **Review:** Peters 2013 [[67](#_ENREF_67),[68](#_ENREF_68)]  **Review title:** Rehabilitation following carpal tunnel release Review Group: Cochrane Neuromuscular Disease Group. **Date (issue):** Jun 2013 (6). | | **Studies:** Randomised or quasi-randomised clinical trials.  **Patients:** All participants with a diagnosis of carpal tunnel syndrome who underwent carpal tunnel release, either endoscopically or with any form of open technique.  **Intervention type:** Non-pharmacological.  **Interventions:** All postoperative rehabilitation treatments including: the provision of advice, exercise, use of a hand or wrist orthosis, scar management, oedema management, electrotherapy, desensitisation, ergonomic modification, work modification, exercise prescription and return-to-work interventions.  **Outcome:** Long-term change in self reported functional ability; primary; continuous; mean difference or no results were reported; NT=6; NP=394. | | | | | **Meta-analysis in protocol:** Meta-analysis (presume pairwise).  **Reason if meta-analysis not planned:** NA.  **Meta-analysis in review:** No meta-analysis applied.  **Reason if meta-analysis not done and was planned:** Each trial made a different comparison and results were stratified by comparison.  **Reason if meta-analysis done when not planned:** NA.  **Analysis type in protocol:** Not reported (presume Frequentist).  **Analysis type in review:** Not reported (presume Frequentist).  **Reason for changing mind**: NA.  **Data type in protocol:** Not reported (presume aggregate).  **Reason for choice in protocol:** Not reported.  **Data type in review:** Not reported (presume aggregate).  **Reason for choice in review:** Not reported.  **Reason for changing mind**: NA.  **Software in protocol:** RevMan.  **Software in review:** Not reported (presume RevMan).  **Reason for changing mind**: Not reported.  **Missing data sought in protocol:** ‘Missing data will be obtained from the authors wherever possible.’  **Missing data sought in review:** ‘One review author obtained missing data from the trial authors wherever possible.’  **Reason for changing mind**: NA. | | | |  |
| **Review:** Rockers 2013 [[69](#_ENREF_69),[70](#_ENREF_70)]  **Review title:** Interventions for hiring, retaining and training district health systems managers in low- and middle-income countries  **Review Group:** [Cochrane Effective Practice and Organisation of Care Group.](http://onlinelibrary.wiley.com/o/cochrane/clabout/articles/EPOC/frame.html)  **Date (issue):** Apr 2013 (4). | | **Studies:** Randomized controlled trials, quasi-randomized controlled trials, controlled before-and-after studies, interrupted time series analyses. For controlled before-and-after studies and cluster randomized studies, ‘we included only those studies that had at least two clusters per comparison group’. For interrupted time series studies ‘we included only those that had at least three waves of data before and three waves of data after the intervention.’  **Patients:** The primary unit in which outcomes are assessed is the district health systems manager in low- and middle-income countries.  **Intervention type:** Non-pharmacological.  **Interventions:** Interventions for hiring, retaining and training district health systems managers.  **Outcome:** Health systems outcomes, such as: population health outcomes, access to health care, utilization of health care, quality of health care, efficiency of health care, equity of health care; primary; dichotomous; risk difference; NT=1; NP=not reported (for population health outcomes, access to health care, utilization of health care), NT=0; NP=0 (for quality of health care, efficiency of health care, equity of health care). | | | | | **Meta-analysis in protocol:** Meta-analysis (presume pairwise).  **Reason if meta-analysis not planned:** NA.  **Meta-analysis in review:** No meta-analysis applied.  **Reason if meta-analysis not done and was planned:** One trial.  **Reason if meta-analysis done when not planned:** NA.  **Analysis type in protocol:** Not reported (presume Frequentist).  **Analysis type in review:** Not reported (presume Frequentist).  **Reason for changing mind**: NA.  **Data type in protocol:** Not reported (presume aggregate).  **Reason for choice in protocol:** Not reported.  **Data type in review:** Not reported (presume aggregate).  **Reason for choice in review:** Not reported.  **Reason for changing mind**: NA.  **Software in protocol:** Not reported (presume RevMan).  **Software in review:** Not reported (presume RevMan).  **Reason for changing mind**: NA.  **Missing data sought in protocol:** ‘We will attempt to obtain missing data from the investigators.’  **Missing data sought in review:** Not reported.  **Reason for changing mind:** Not reported. | | | |  |
| **Review:** Sajid 2012 [[71](#_ENREF_71),[72](#_ENREF_72)]  **Review title:** Fibrin glue instillation under skin flaps to prevent seroma-related morbidity following breast and axillary surgery  **Review Group:** Cochrane Breast Cancer Group.  **Date (issue):** May 2013 (5). | | **Studies:** Randomised controlled trials.  **Patients:** People with breast cancer undergoing simple mastectomy, modified radical mastectomy, breast-conserving surgery, oncoplastic breast surgery, lumpectomy, quadrantectomy, axillary sentinel node biopsy, axillary sampling, axillary dissection of any level, and immediate partial or total breast reconstruction.  **Intervention type:** Surgical.  **Interventions:** Fibrin glue underneath skin flaps versus no use of fibrin glue.  **Outcome:** Incidence of seroma, defined as the presence of fluid collection under the skin flap diagnosed by clinical and radiological assessment and requiring prolonged hospital stay, frequent clinic visits or aspiration(s), or a combination of these; primary; dichotomous; risk ratio; NT=18; NP=1,252. | | | | | **Meta-analysis in protocol:** Meta-analysis (presume pairwise).  **Reason if meta-analysis not planned:** NA.  **Meta-analysis in review:** pairwise meta-analysis applied.  **Reason if meta-analysis not done and was planned**: NA.  **Reason if meta-analysis done when not planned:** NA.  **Analysis type in protocol:** Not reported (presume Frequentist).  **Analysis type in review:** Not reported (presume Frequentist).  **Reason for changing mind**: NA.  **Data type in protocol:** Not reported (presume aggregate).  **Reason for choice in protocol:** Not reported.  **Data type in review:** Not reported (presume aggregate).  **Reason for choice in review:** Not reported.  **Reason for changing mind**: NA.  **Software in protocol:** RevMan, Excel.  **Software in review:** RevMan.  **Reason for changing mind**: Not reported.  **Missing data sought in protocol:** ‘We will contact the first author of a study via personal communication in order to retrieve missing data. If further information is required from any source, we will contact all relevant persons involved in the running of that published trial.’  **Missing data sought in review:** ‘We contacted the first author of a study via personal communication in order to retrieve missing data. If further information was required from any source, we contacted all relevant people involved in the running of that published trial.’  **Reason for changing mind**: NA. | | | |  |
| **Review:** Sampson 2013 [[73](#_ENREF_73),[74](#_ENREF_74)]  **Review title:** Intermittent drug techniques for schizophrenia  **Review Group:** [Cochrane Schizophrenia Group.](http://onlinelibrary.wiley.com/o/cochrane/clabout/articles/SCHIZ/frame.html)  **Date (issue):** Jul 2013 (7). | | **Studies:** Randomised controlled trials. Excluding quasi-randomised studies.  **Patients:** We included people with schizophrenia and other types of schizophrenia-like psychoses (schizophreniform and schizoaffective disorders diagnosed by any criteria).  **Intervention type:** Drugs.  **Interventions:** Any type of intermittent drug technique (including prodrome-based/early intervention, crisis intervention, gradually increased drug-free period, drug holiday) versus maintenance therapy, placebo. Any intermittent drug technique (high dose, low or moderate dose).  **Outcome:** Relapse; primary; dichotomous; risk ratio; NT=12; NP=1,327. | | | | | **Meta-analysis in protocol:** Meta-analysis (presume pairwise).  **Reason if meta-analysis not planned:** NA.  **Meta-analysis in review:** pairwise meta-analysis applied.  **Reason if meta-analysis not done and was planned**: NA.  **Reason if meta-analysis done when not planned:** NA.  **Analysis type in protocol:** Not reported (presume Frequentist).  **Analysis type in review:** Not reported (presume Frequentist).  **Reason for changing mind**: NA.  **Data type in protocol:** Not reported (presume aggregate).  **Reason for choice in protocol:** Not reported.  **Data type in review:** Not reported (presume aggregate).  **Reason for choice in review:** Not reported.  **Reason for changing mind**: NA.  **Software in protocol:** RevMan.  **Software in review:** Not reported (presume RevMan).  **Reason for changing mind**: Not reported.  **Missing data sought in protocol:** ‘To avoid the pitfall of applying parametric tests to non-parametric data, we applied the following standards to all data before inclusion: (a) standard deviations and means were reported in the paper or were obtainable from the authors;’  ‘We have contacted authors of studies reporting only change data for endpoint figures.’  ‘In subsequent versions of this review we will seek to contact first authors of studies to obtain intra-class correlation co-efficients of their clustered data and to adjust for this using accepted methods’.  **Missing data sought in review:** ‘We attempted to contact authors through an open-ended request in order to obtain missing information or for clarification whenever necessary.’  **Reason for changing mind**: Not reported. | | | |  |
| **Review:** Sanders 2013 [[75](#_ENREF_75),[76](#_ENREF_76)]  **Review title:** Perioperative statin therapy for improving outcomes during and after noncardiac vascular surgery  **Review Group:** Cochrane Anaesthesia Group.  **Date (issue):** Jul 2013 (7). | | **Studies:** Randomized controlled trials including quasi-randomized and cluster-randomized trials.  **Patients:** Adult participants who were scheduled for elective and emergency noncardiac arterial vascular surgery, including both open and endovascular procedures.  **Intervention type:** Drugs.  **Interventions:** In statin-naive populations, short-term statin therapy commenced before or on the day of surgery and continued at least 48 hours versus placebo, no treatment or standard care. In patients already receiving statin therapy, comparing different doses of statins.  **Outcome:** All-cause mortality within 30 days of surgery; primary; dichotomous; risk ratio; NT=4; NP=675. | | | | | **Meta-analysis in protocol:** Meta-analysis (presume pairwise).  **Reason if meta-analysis not planned:** NA.  **Meta-analysis in review:** pairwise meta-analysis applied.  **Reason if meta-analysis not done and was planned**: NA.  **Reason if meta-analysis done when not planned:** NA.  **Analysis type in protocol:** Not reported (presume Frequentist).  **Analysis type in review:** Not reported (presume Frequentist).  **Reason for changing mind**: NA.  **Data type in protocol:** Not reported (presume aggregate).  **Reason for choice in protocol:** Not reported.  **Data type in review:** Not reported (presume aggregate).  **Reason for choice in review:** Not reported.  **Reason for changing mind**: NA.  **Software in protocol:** RevMan.  **Software in review:** RevMan.  **Reason for changing mind**: NA.  **Missing data sought in protocol:** ‘If relevant information or data are not available in the paper, we will contact the lead author to request the additional details.’  **Missing data sought in review:** ‘If relevant information or data were not available in the paper, we contacted the lead author to request the additional details.’  **Reason for changing mind**: NA. | | | |  |
| **Review:** Sarai 2013 [[77](#_ENREF_77),[78](#_ENREF_78)]  **Review title:** Magnesium for alcohol withdrawal  **Review Group:** [Cochrane Drugs and Alcohol Group.](http://onlinelibrary.wiley.com/o/cochrane/clabout/articles/ADDICTN/frame.html)  **Date (issue):** Jun 2013 (6). | | **Studies:** Randomised controlled trials.  **Patients:** Hospitalised adults with a current history of alcohol dependence, at risk for or already in acute withdrawal.  **Intervention type:** Drugs.  **Interventions:** Magnesium in addition to standard of usual care versus placebo (usual standard of care).  **Outcome:** Number of participants with at least one seizure; primary; dichotomous; number of patients with seizures in each group; NT=1; NP=100. | | | | | **Meta-analysis in protocol:** Meta-analysis (presume pairwise).  **Reason if meta-analysis not planned:** NA.  **Meta-analysis in review:** No meta-analysis applied.  **Reason if meta-analysis not done and was planned:** One trial.  **Reason if meta-analysis done when not planned:** NA.  **Analysis type in protocol:** Not reported (presume Frequentist).  **Analysis type in review:** Not reported (presume Frequentist).  **Reason for changing mind**: NA.  **Data type in protocol:** Not reported (presume aggregate).  **Reason for choice in protocol:** Not reported.  **Data type in review:** Not reported (presume aggregate).  **Reason for choice in review:** Not reported.  **Reason for changing mind**: NA.  **Software in protocol:** RevMan.  **Software in review:** RevMan.  **Reason for changing mind**: NA.  **Missing data sought in protocol:** ‘In general if there is missing data, the authors of the study will be contacted for clarification.’  **Missing data sought in review:** ‘In general if there were missing data, we contacted the authors of the study for clarification’.  **Reason for changing mind**: NA. | | | |  |
| **Review:** Schoot 2013 [[79](#_ENREF_79),[80](#_ENREF_80)]  **Review title:** Antibiotic and other lock treatments for tunnelled central venous catheter-related infections in children with cancer  **Review Group:** Cochrane Childhood Cancer Group.  **Date (issue):** June 2013 (6). | | **Studies:** Randomized controlled trials or controlled clinical trials.  **Patients:** Children with cancer (0 to 18 years) with a central venous catheter -related infection.  **Intervention type:** Drugs.  **Interventions:** (1) Lock treatment (antibiotic or other) versus another lock treatment without systemic antibiotics; (2) Lock treatment (antibiotic or other) versus systemic antibiotics alone. (3) Lock treatment (antibiotic or other) versus another lock treatment with concomitant systemic antibiotics. (4) Lock treatment (antibiotic or other) with concomitant systemic antibiotics versus systemic antibiotics alone.  **Outcome:** The number of children cured of their central venous catheter -related infection; primary; dichotomous; risk ratio; NT=3; NP=132. | | | | | **Meta-analysis in protocol:** Meta-analysis (presume pairwise).  **Reason if meta-analysis not planned:** NA.  **Meta-analysis in review:** pairwise meta-analysis applied.  **Reason if meta-analysis not done and was planned**: NA.  **Reason if meta-analysis done when not planned:** NA.  **Analysis type in protocol:** Not reported (presume Frequentist).  **Analysis type in review:** Not reported (presume Frequentist).  **Reason for changing mind**: NA.  **Data type in protocol:** Not reported (presume aggregate).  **Reason for choice in protocol:** Not reported.  **Data type in review:** Not reported (presume aggregate).  **Reason for choice in review:** Not reported.  **Reason for changing mind**: NA.  **Software in protocol:** RevMan.  **Software in review:** RevMan.  **Reason for changing mind**: NA.  **Missing data sought in protocol:** ‘We will contact study authors for additional information where necessary.’  **Missing data sought in review:** ‘If necessary we would have contacted study authors for additional information.’  **Reason for changing mind**: NA. | | | |  |
| **Review:** Semple 2013 [[81](#_ENREF_81),[82](#_ENREF_82)]  **Review title:** Psychosocial interventions for patients with head and neck cancer  **Review Group:** Cochrane Ear, Nose and Throat Disorders Group.  **Date (issue):** Jul 2013 (7). | | **Studies:** Randomised controlled trials and quasi-randomised controlled trials.  **Patients:** Adults with any type or stage of head and neck cancer.  **Intervention type:** Psychosocial.  **Interventions:** Psychosocial interventions included: psychoeducational, psychotherapy  (individual), cognitive behavioural training, supportive and group interventions. Control groups included those who had not received the psychosocial intervention being tested or who had received ‘standard care’.  **Outcome:** Quality of life; primary; continuous; mean difference or no data extracted; NT=5; NP=297. | | | | | **Meta-analysis in protocol:** Meta-analysis (presume pairwise).  **Reason if meta-analysis not planned:** NA.  **Meta-analysis in review:** pairwise meta-analysis applied.  **Reason if meta-analysis not done and was planned**: NA.  **Reason if meta-analysis done when not planned:** NA.  **Analysis type in protocol:** Not reported (presume Frequentist).  **Analysis type in review:** Not reported (presume Frequentist).  **Reason for changing mind**: NA.  **Data type in protocol:** Not reported (presume aggregate).  **Reason for choice in protocol:** Not reported.  **Data type in review:** Not reported (presume aggregate).  **Reason for choice in review:** Not reported.  **Reason for changing mind**: NA.  **Software in protocol:** RevMan.  **Software in review:** RevMan.  **Reason for changing mind**: NA.  **Missing data sought in protocol:** ‘When missing data or study discrepancies are evident, where possible we will make contact with the original investigators for further information.’  **Missing data sought in review:** ‘When missing data or study discrepancies were evident, we made attempts (where possible) to contact the original investigators for further information.’  **Reason for changing mind**: NA. | | | |  |
| **Review:** Sharma 2013 [[83](#_ENREF_83),[84](#_ENREF_84)]  **Review title:** Rifamycins (rifampicin, rifabutin and rifapentine) compared to isoniazid for preventing tuberculosis in HIV-negative people at risk of active TB  **Review Group:**  [Cochrane Infectious Diseases Group.](http://onlinelibrary.wiley.com/o/cochrane/clabout/articles/SYMPT/frame.html)  **Date (issue):** Jul 2013 (7). | | **Studies:** Randomized controlled trials that randomized individuals or clusters of individuals. Quasi-Randomized controlled trials were excluded.  **Patients:** HIV-negative people at risk of developing active tuberculosis and without active tuberculosis at the time of enrolment.  **Intervention type:** Drugs.  **Interventions:** Treatment with rifampicin or rifamycin-containing drug combinations (any dose or duration) versus Isoniazid monotherapy for six to 12 months.  **Outcome:** Rates of active TB; primary; dichotomous; risk ratio; NT=6; NP=8,904. | | | | | **Meta-analysis in protocol:** Meta-analysis (presume pairwise).  **Reason if meta-analysis not planned:** NA.  **Meta-analysis in review:** pairwise meta-analysis applied.  **Reason if meta-analysis not done and was planned**: NA.  **Reason if meta-analysis done when not planned:** NA.  **Analysis type in protocol:** Not reported (presume Frequentist).  **Analysis type in review:** Not reported (presume Frequentist).  **Reason for changing mind**: NA.  **Data type in protocol:** Not reported (presume aggregate).  **Reason for choice in protocol:** Not reported.  **Data type in review:** Not reported (presume aggregate).  **Reason for choice in review:** Not reported.  **Reason for changing mind**: NA.  **Software in protocol:** Not reported (presume RevMan).  **Software in review:** RevMan.  **Reason for changing mind**: Not reported.  **Missing data sought in protocol:** ‘Where data are not clear or not presented by the author in the publication, we will attempt to contact the trial author for further details.’  **Missing data sought in review:** ‘We attempted to contact the contact author or senior author for further details when data were not clear or not presented in the publication.’  **Reason for changing mind**: NA. | | | |  |
| **Review:** Showell 2013 [[85](#_ENREF_85),[86](#_ENREF_86)]  **Review title:** Antioxidants for female subfertility  **Review Group:** [Cochrane Menstrual Disorders and Subfertility Group.](http://onlinelibrary.wiley.com/o/cochrane/clabout/articles/MENSTR/frame.html)  **Date (issue):** Aug 2013 (8). | | **Studies:** Randomised controlled trials. Cross-over trials (the first phase). Excluded quasi-randomised trials.  **Patients:** Subfertile women who had been referred to a fertility clinic and might or might not be undergoing assisted reproductive techniques such as in vitro fertilisation, intrauterine insemination or intracytoplasmic sperm injection.  **Intervention type:** Drugs.  **Interventions:** (1) Any type of oral antioxidant supplementation versus control placebo (plus or minus a co-intervention) or no treatment/standard treatment (standard treatment includes folic acid < 1 mg); (2) Individual or combined oral antioxidants versus any antioxidant (head-to-head trials); or (3) Pentoxifylline versus control (placebo or no treatment/standard treatment).  **Outcome:** Live birth rate per woman randomly assigned (defined as the delivery of one or more living infants); primary; dichotomous; odds ratio; NT=4; NP=293. | | | | | **Meta-analysis in protocol:** Meta-analysis (presume pairwise).  **Reason if meta-analysis not planned:** NA.  **Meta-analysis in review:** pairwise meta-analysis applied.  **Reason if meta-analysis not done and was planned**: NA.  **Reason if meta-analysis done when not planned:** NA.  **Analysis type in protocol:** Not reported (presume Frequentist).  **Analysis type in review:** Not reported (presume Frequentist).  **Reason for changing mind**: NA.  **Data type in protocol:** Not reported (presume aggregate).  **Reason for choice in protocol:** Not reported.  **Data type in review:** Not reported (presume aggregate).  **Reason for choice in review:** Not reported.  **Reason for changing mind**: NA.  **Software in protocol:** RevMan.  **Software in review:** RevMan.  **Reason for changing mind**: NA.  **Missing data sought in protocol:** ‘The review authors shall contact the lead authors of the trials where if data clarification is required this contact shall be made by email post and by telephone.’  **Missing data sought in review:** ‘In cases where trial data were missing, we first sought information from the original trial investigators.’  **Reason for changing mind**: NA. | | | |  |
| **Review:** Stead 2012 [[87](#_ENREF_87),[88](#_ENREF_88)]  **Review title:** Behavioural interventions as adjuncts to pharmacotherapy for smoking cessation  **Review Group:** [Cochrane Tobacco Addiction Group.](http://onlinelibrary.wiley.com/o/cochrane/clabout/articles/TOBACCO/frame.html)  **Date (issue):** Dec 2012 (12). | | **Studies:** Randomized or quasi-randomized controlled trials.  **Patients:** We included trials that recruited people who smoke in any setting, with the exception of trials which only recruited pregnant women or adolescents.  **Intervention type:** Drugs.  **Interventions:** Smoking cessation interventions where all participants had access to a smoking cessation pharmacotherapy (including nicotine replacement therapy, varenicline, bupropion and nortriptyline, or a combination or choice of these) and in which one or more intervention conditions received more intensive behavioural support than the control condition.  **Outcome:** Smoking cessation at the longest follow-up; primary; dichotomous; risk ratio; NT=38; NP=15,506. | | | | | **Meta-analysis in protocol:** Meta-analysis (presume pairwise).  **Reason if meta-analysis not planned:** NA.  **Meta-analysis in review:** pairwise meta-analysis applied.  **Reason if meta-analysis not done and was planned**: NA.  **Reason if meta-analysis done when not planned:** NA.  **Analysis type in protocol:** Not reported (presume Frequentist).  **Analysis type in review:** Not reported (presume Frequentist).  **Reason for changing mind**: NA.  **Data type in protocol:** Not reported (presume aggregate).  **Reason for choice in protocol:** Not reported.  **Data type in review:** Not reported (presume aggregate).  **Reason for choice in review:** Not reported.  **Reason for changing mind**: NA.  **Software in protocol:** Not reported (presume RevMan).  **Software in review:** Not reported (presume RevMan).  **Reason for changing mind**: NA.  **Missing data sought in protocol:** Not reported.  **Missing data sought in review:** Not reported.  **Reason for changing mind**: NA. | | | |  |
| **Review:** Trivedi 2013 [[89](#_ENREF_89),[90](#_ENREF_90)]  **Review title:** Early versus late administration of amino acids in preterm infants receiving parenteral nutrition  **Review Group:** [Cochrane Neonatal Group.](http://onlinelibrary.wiley.com/o/cochrane/clabout/articles/NEONATAL/frame.html)  **Date (issue):** Jul 2013 (7). | | **Studies:** Randomised, quasi-randomised and cluster-randomised trials.  **Patients:** All neonates born at less than 37 weeks of gestation.  **Intervention type:** Timing of intervention.  **Interventions:** Early versus late initiation of amino acids with or without any other parenteral nutrition intake.  **Outcome:** Weight gain in gm/kg/week during the first month of life; primary; continuous; mean difference planned (no data); NT=0; NP=0. | | | | | **Meta-analysis in protocol:** Meta-analysis (presume pairwise).  **Reason if meta-analysis not planned:** NA.  **Meta-analysis in review:** No meta-analysis applied.  **Reason if meta-analysis not done and was planned:** No data.  **Reason if meta-analysis done when not planned:** NA.  **Analysis type in protocol:** Not reported (presume Frequentist).  **Analysis type in review:** Not reported (presume Frequentist).  **Reason for changing mind**: NA.  **Data type in protocol:** Not reported (presume aggregate).  **Reason for choice in protocol:** Not reported.  **Data type in review:** Not reported (presume aggregate).  **Reason for choice in review:** Not reported.  **Reason for changing mind**: NA.  **Software in protocol:** Not reported (presume RevMan).  **Software in review:** Not reported (presume RevMan).  **Reason for changing mind**: NA.  **Missing data sought in protocol:** ‘We will contact the authors of studies included in the review for missing data.’  **Missing data sought in review:** Not reported.  **Reason for changing mind:** Not reported. | | | |  |
| **Review:** Trotti 2012 [[91](#_ENREF_91),[92](#_ENREF_92)]  **Review title:** Iron for restless legs syndrome Review Group: [**Cochrane Movement Disorders Group.**](http://onlinelibrary.wiley.com/o/cochrane/clabout/articles/MOVEMENT/sect0-meta.html) **Date (issue):** May 2012 (5). | | **Studies:** Controlled trials regardless of whether or not they were randomized or blinded. Included parallel and cross-over trials (but not the second phase of cross-over trials).  **Patients:** Adult patients with restless legs syndrome.  **Intervention type:** Drugs.  **Interventions:** Therapy with any dose or regimen of oral or parenteral iron-containing compounds compared with placebo, other drugs or no intervention.  **Outcome:** Restlessness or unpleasant sensations as experienced subjectively by the patient; primary; continuous; mean difference; NT=5; NP=164. | | | | | | **Meta-analysis in protocol:** Meta-analysis (presume pairwise).  **Reason if meta-analysis not planned:** NA.  **Meta-analysis in review:** pairwise meta-analysis applied.  **Reason if meta-analysis not done and was planned**: NA.  **Reason if meta-analysis done when not planned:** NA.  **Analysis type in protocol:** Not reported (presume Frequentist).  **Analysis type in review:** Not reported (presume Frequentist).  **Reason for changing mind**: NA.  **Data type in protocol:** Not reported (presume aggregate).  **Reason for choice in protocol:** Not reported.  **Data type in review:** Not reported (presume aggregate).  **Reason for choice in review:** Not reported.  **Reason for changing mind**: NA.  **Software in protocol:** Not reported (presume RevMan).  **Software in review:** Not reported (presume RevMan).  **Reason for changing mind**: NA.  **Missing data sought in protocol:** ‘We will contact study authors to obtain unpublished information, including outcome data not explicitly stated in the published papers.’  **Missing data sought in review:** ‘We contacted study authors to obtain unpublished information, including outcome data not explicitly stated in the published papers.’  **Reason for changing mind**: NA. | | |  |
| **Review:** Van Teeffelen 2013 [[93](#_ENREF_93),[94](#_ENREF_94)]  **Review title:** Transabdominal amnioinfusion for improving fetal outcomes after oligohydramnios secondary to preterm prelabour rupture of membranes before 26 weeks  **Review Group:** [Cochrane Pregnancy and Childbirth Group.](http://onlinelibrary.wiley.com/o/cochrane/clabout/articles/PREG/frame.html)  **Date (issue):** Aug 2013 (8). | | **Studies:** Randomised controlled trials.  **Patients:** Women with a pregnancy complicated by premature prelabour rupture of membranes before 26 weeks and subsequent oligohydramnios.    **Intervention type:** Surgical.  **Interventions:** Transabdominal amnioinfusion versus standard management.  **Outcome:** Perinatal mortality; primary; dichotomous; risk ratio planned (no data); NT=0; NP=0. | | | | | | **Meta-analysis in protocol:** Meta-analysis (presume pairwise).  **Reason if meta-analysis not planned:** NA.  **Meta-analysis in review:** No meta-analysis applied.  **Reason if meta-analysis not done and was planned:** No data.  **Reason if meta-analysis done when not planned:** NA.  **Analysis type in protocol:** Not reported (presume Frequentist).  **Analysis type in review:** Not reported (presume Frequentist).  **Reason for changing mind**: NA.  **Data type in protocol:** Not reported (presume aggregate).  **Reason for choice in protocol:** Not reported.  **Data type in review:** Not reported (presume aggregate).  **Reason for choice in review:** Not reported.  **Reason for changing mind**: NA.  **Software in protocol:** RevMan.  **Software in review:** RevMan.  **Reason for changing mind**: NA.  **Missing data sought in protocol:** ‘When information regarding any of the above is unclear, we will attempt to contact authors of the original reports to provide further details.’  **Missing data sought in review:** Not reported.  **Reason for changing mind:** Not reported. | | | |
| **Review:** van Zuuren 2013 [[95](#_ENREF_95),[96](#_ENREF_96)]  **Review title:** Low-molecular-weight heparins for managing vaso-occlusive crises in people with sickle cell disease  **Review Group:** [Cochrane Cystic Fibrosis and Genetic Disorders Group.](http://onlinelibrary.wiley.com/o/cochrane/clabout/articles/CF/frame.html)  **Date (issue):** Jun 2013 (6). | | **Studies:** Randomised controlled clinical trials and controlled clinical trials.  **Patients:** People with homozygous sickle cell disease, Sickle cell-haemoglobin C disease, beta0− or beta+-thalassaemia.  **Intervention type:** Drugs.  **Interventions:** Any low-molecular-weight heparins administered subcutaneously compared with placebo or standard care for a period of up to two years.  **Outcome:** Pain i) Intensity (expressed as scores obtained through any validated patient-reported outcomes instrument, either generic or sickle cell disease specific) ii) Duration; primary; continuous; mean difference for duration, p-value for intensity; NT=1; NP=253 (same numbers for each outcome). | | | | | | **Meta-analysis in protocol:** Meta-analysis (presume pairwise).  **Reason if meta-analysis not planned:** NA.  **Meta-analysis in review:** No meta-analysis applied.  **Reason if meta-analysis not done and was planned:** One trial.  **Reason if meta-analysis done when not planned:** NA.  **Analysis type in protocol:** Not reported (presume Frequentist).  **Analysis type in review:** Not reported (presume Frequentist).  **Reason for changing mind**: NA.  **Data type in protocol:** Not reported (presume aggregate).  **Reason for choice in protocol:** Not reported.  **Data type in review:** Not reported (presume aggregate).  **Reason for choice in review:** Not reported.  **Reason for changing mind**: NA.  **Software in protocol:** RevMan.  **Software in review:** RevMan.  **Reason for changing mind**: NA.  **Missing data sought in protocol:** ‘The authors will contact principal investigators of included trials to supply missing data.’  **Missing data sought in review:** ‘The review authors contacted principal investigators of included trials to request missing data.’  **Reason for changing mind**: NA. | |  |  |
| **Review:** Wakai 2013 [[97](#_ENREF_97),[98](#_ENREF_98)]  **Review title:** Nitrates for acute heart failure syndromes  **Review Group:** [Cochrane Heart Group.](http://onlinelibrary.wiley.com/o/cochrane/clabout/articles/VASC/frame.html)  **Date (issue):** Aug 2013 (8). | | **Studies:** Randomised or quasi-randomised controlled trials.  **Patients:** Adult patients with acute heart failure syndromes.  **Intervention type:** Drugs**.**  **Interventions:** Administration of a nitrate (for example nitroglycerin or isosorbide dinitrate) compared with an alternative intervention (pharmacological agent like frusemide, hydralazine, prenalterol and nesiritide or non-pharmacological intervention such as non-invasive positive pressure ventilation) for acute heart failure.  **Outcome:** Rapidity with which symptoms (for example dyspnoea, fatigue, self reported patient satisfaction score, global clinical status) are relieved; primary; continuous; mean difference (1 trial) and no results for a second trial; NT=2; NP=558. | | | | | | **Meta-analysis in protocol:** Meta-analysis (presume pairwise).  **Reason if meta-analysis not planned:** NA.  **Meta-analysis in review:** No meta-analysis applied.  **Reason if meta-analysis not done and was planned:** ‘It was not possible to pool the results of these two trials because the comparator interventions were different and the rapidity of symptom relief was measured and reported in different ways in the two studies.’  **Reason if meta-analysis done when not planned:** NA.  **Analysis type in protocol:** Not reported (presume Frequentist).  **Analysis type in review:** Not reported (presume Frequentist).  **Reason for changing mind**: NA.  **Data type in protocol:** Individual patient data. ‘In case individual patient data can be obtained, the summary statistics for each study will be calculated by reanalysis of the raw data. This analysis is not possible with RevMan and will be done in a separate analysis program with assistance from a statistician. The summary statistics will then be input to RevMan and combined using the Peto odds ratio method (Deeks 2001).’  **Reason for choice in protocol:** Not reported.  **Data type in review:** Aggregate data.  **Reason for choice in review:** Not reported.  **Reason for changing mind:** ‘it was not possible to obtain the individual patient data from the trialists.’  **Software in protocol:** RevMan and a ‘separate analysis program’.  **Software in review:** RevMan.  **Reason for changing mind**: Not reported.  **Missing data sought in protocol:** ‘This problem will be handled by contacting the investigators, whenever possible, to ensure that no data are missing for their study.’  **Missing data sought in review:** ‘We handled this problem by contacting the investigators, whenever possible, to ensure that no data were missing for the studies.’  **Reason for changing mind**: NA. | |  |  |
| **Review:** Wang 2013 [[99](#_ENREF_99),[100](#_ENREF_100)]  **Review title:** Acupuncture for stress urinary incontinence in adults  **Review Group:** [Cochrane Incontinence Group.](http://onlinelibrary.wiley.com/o/cochrane/clabout/articles/INCONT/frame.html)  **Date (issue):** Jul 2013 (7). | | **Studies:** Randomised and quasi-randomised controlled trials.  **Patients:** Participants with a diagnosis of stress urinary incontinence.  **Intervention type:** Non-pharmacological.  **Interventions:** Acupuncture interventions without other treatment versus placebo, no treatment and any active treatment (i.e. conservative therapies, pharmacological therapies or surgery).  **Outcome:** Number of participants with incontinence; primary; dichotomous; risk ratio; NT=1; NP=60. | | | | | | **Meta-analysis in protocol:** Meta-analysis (presume pairwise).  **Reason if meta-analysis not planned:** NA.  **Meta-analysis in review:** No meta-analysis applied.  **Reason if meta-analysis not done and was planned:** One trial.  **Reason if meta-analysis done when not planned:** NA.  **Analysis type in protocol:** Not reported (presume Frequentist).  **Analysis type in review:** Not reported (presume Frequentist).  **Reason for changing mind**: NA.  **Data type in protocol:** Not reported (presume aggregate).  **Reason for choice in protocol:** Not reported.  **Data type in review:** Not reported (presume aggregate).  **Reason for choice in review:** Not reported.  **Reason for changing mind**: NA.  **Software in protocol:** RevMan.  **Software in review:** Not reported (presume RevMan).  **Reason for changing mind**: Not reported.  **Missing data sought in protocol:** ‘When data has been collected but not reported, we will seek clarification from the trialists.’  **Missing data sought in review:** ‘When data had been collected but not reported, we sought clarification from the trialists’.  **Reason for changing mind**: NA. | |  |  |
| **Review:** Yue 2013 [[101](#_ENREF_101),[102](#_ENREF_102)]  **Review title:** Linezolid versus vancomycin for skin and soft tissue infections  **Review Group:** [Cochrane Wounds Group.](http://onlinelibrary.wiley.com/o/cochrane/clabout/articles/WOUNDS/frame.html)  **Date (issue):** Jul 2013 (7). | | **Studies:** Randomised controlled trials.  **Patients:** People with skin and soft tissue infections.  **Intervention type:** Drugs.  **Interventions:** Any dose of linezolid or vancomycin, by any route. ‘We intended to present comparisons as follows: (1) Linezolid compared with vancomycin alone. (2) Linezolid plus co-interventions compared with vancomycin plus co-interventions.’  **Outcome:** 1. Clinical cure (resolution of symptoms and signs) and microbiological cure (eradication of bacteria on wound culture); primary; dichotomous; risk ratio; NT=9; NP=3,114 (for clinical cure); NT=9; NP=2,014 (for microbiological cure). | | | | | | **Meta-analysis in protocol:** Meta-analysis (presume pairwise).  **Reason if meta-analysis not planned:** NA.  **Meta-analysis in review:** pairwise meta-analysis applied.  **Reason if meta-analysis not done and was planned**: NA.  **Reason if meta-analysis done when not planned:** NA.  **Analysis type in protocol:** Not reported (presume Frequentist).  **Analysis type in review:** Not reported (presume Frequentist).  **Reason for changing mind**: NA.  **Data type in protocol:** Not reported (presume aggregate).  **Reason for choice in protocol:** Not reported.  **Data type in review:** Not reported (presume aggregate).  **Reason for choice in review:** Not reported.  **Reason for changing mind**: NA.  **Software in protocol:** RevMan.  **Software in review:** Not reported (presume RevMan).  **Reason for changing mind**: Not reported.  **Missing data sought in protocol:** ‘If data are missing from the trial reports, we will attempt to contact the trial authors to request these values.’  **Missing data sought in review:** ‘When data were missing from the trial reports, we attempted to contact the trial authors to request these values.’  **Reason for changing mind**: NA. | |  |  |
| **Review:** Ziebell 2013 [[103](#_ENREF_103),[104](#_ENREF_104)]  **Review title:** Flow-regulated versus differential pressure-regulated shunt valves for adult patients with normal pressure hydrocephalus  **Review Group:** [Cochrane Dementia and Cognitive Improvement Group.](http://onlinelibrary.wiley.com/o/cochrane/clabout/articles/DEMENTIA/frame.html)  **Date (issue):** May 2013 (5). | | **Studies:** Randomised clinical trials.  **Patients:** Surgical patients aged 18 years or older with normal pressure hydrocephalus (idiopathic and secondary).  **Intervention type:** Surgical.  **Interventions:** Surgical ventriculo-peritoneal shunt insertion. Flow-regulated valve type versus differential-pressure valve type.  **Outcome:** Death from any cause; primary; dichotomous; odds ratio or risk difference planned (no data); NT=0; NP=0. | | | | | | **Meta-analysis in protocol:** Meta-analysis (presume pairwise).  **Reason if meta-analysis not planned:** NA.  **Meta-analysis in review:** No meta-analysis applied.  **Reason if meta-analysis not done and was planned:** No data.  **Reason if meta-analysis done when not planned:** NA.  **Analysis type in protocol:** Not reported (presume Frequentist).  **Analysis type in review:** Not reported (presume Frequentist).  **Reason for changing mind**: NA.  **Data type in protocol:** Not reported (presume aggregate).  **Reason for choice in protocol:** Not reported.  **Data type in review:** Not reported (presume aggregate).  **Reason for choice in review:** Not reported.  **Reason for changing mind**: NA.  **Software in protocol:** RevMan, Stata 9 and Comprehensive Meta-analysis.  **Software in review:** Not reported (presume RevMan).  **Reason for changing mind**: Not reported.  **Missing data sought in protocol:** ‘We will approach all corresponding authors of the included trials for additional information relevant to the review’s outcomes measures and risk of bias components.’  **Missing data sought in review:** ‘We approached all corresponding authors of included trials for additional information relevant to the review’s outcomes measures and risk of bias components.’  **Reason for changing mind**: NA. | |  |  |

NT: Number of trials; NP: Number of patients; NA: not applicable.

**^1^**The type of studies, patients, interventions, and the outcome (i.e. the first primary outcome or the first listed outcome where no primary outcome was specified) reported in the methods of the review document. Information reported under the outcome heading is as follows: the outcome name, whether the outcome was a primary outcome, the type of outcome (e.g. continuous, categorical), the measure of effect (or other results if no measure of effect reported), and the number of trials and patients that reported the outcome.

^2^We presumed the type of data used in the analysis was AD unless IPD was specifically mentioned in the review. Likewise, we presumed the type of analysis was Frequentist (rather than Bayesian), the type of meta-analysis was pairwise (rather than network meta-analysis), and the software used was RevMan, unless otherwise stated.

**References**

1. Aboumarzouk OM, Nelson RL. Pregabalin for chronic prostatitis (Protocol). Cochrane Database of Systematic Reviews 2011, Issue 4. Art. No.: CD009063. doi: 10.1002/14651858.CD009063.

2. Aboumarzouk OM, Nelson RL. Pregabalin for chronic prostatitis. Cochrane Database of Systematic Reviews. 2012, Issue 8. Art. No.: CD009063. doi: 10.1002/14651858.CD009063.pub2.

3. Almeida MO, Silva BNG, Andriolo RB, Atallah ÁN, Peccin MS. Conservative interventions for treating exercise-related musculotendinous, ligamentous and osseous groin pain (Protocol). Cochrane Database of Systematic Reviews 2012, Issue 1. Art. No.: CD009565. doi: 10.1002/14651858.CD009565.

4. Almeida MO, Silva BNG, Andriolo RB, Atallah ÁN, Peccin MS. Conservative interventions for treating exercise-related musculotendinous, ligamentous and osseous groin pain. Cochrane Database of Systematic Reviews. 2013, Issue 6. Art. No.: CD009565. doi: 10.1002/14651858.CD009565.pub2.

5. Basurto OX, Rigau CD, Urrútia G. Opioids for acute pancreatitis pain (Protocol). Cochrane Database of Systematic Reviews. 2011, Issue 6. Art. No.: CD009179. doi: 10.1002/14651858.CD009179.

6. Basurto OX, Rigau CD, Urrútia G. Opioids for acute pancreatitis pain. Cochrane Database of Systematic Reviews. 2013, Issue 7. Art. No.: CD009179. doi: 10.1002/14651858.CD009179.pub2.

7. Bellmunt-Montoya S, Escribano JM, Dilme J, Martinez-Zapata MJ. CHIVA method for the treatment of chronic venous insufficiency. Cochrane Database of Systematic Reviews. 2013, Issue 7. Art. No.: CD009648. doi: 10.1002/14651858.CD009648.pub2.

8. Bellmunt-Montoya S, Escribano JM, Dilme J, Martinez-Zapata MJ. CHIVA method for the treatment of varicose veins (Protocol). Cochrane Database of Systematic Reviews

2012, Issue 2. Art. No.: CD009648. doi: 10.1002/14651858.CD009648.

9. Berlowitz D, Tamplin J. Respiratory muscle training for cervical spinal cord injury (Protocol). Cochrane Database of Systematic Reviews. 2010, Issue 5. Art. No.: CD008507. doi: 10.1002/14651858.CD008507.

10. Berlowitz DJ, Tamplin J. Respiratory muscle training for cervical spinal cord injury. Cochrane Database of Systematic Reviews. 2013, Issue 7. Art. No.: CD008507. doi: 10.1002/14651858.CD008507.pub2.

11. Boselie TFM, Willems PC, van Mameren H, de Bie R, Benzel EC, et al. Arthroplasty versus fusion in single-level cervical degenerative disc disease. Cochrane Database of Systematic Reviews. 2012, Issue 9. Art. No.: CD009173. doi: 10.1002/14651858.CD009173.pub2.

12. Boselie AFM, van Santbrink H, van Mameren H, de Bie R, Benzel EC, et al. Fusion versus arthroplasty in single level cervical degenerative disc disease (Protocol). Cochrane Database of Systematic Reviews. 2011, Issue 6. Art. No.: CD009173. doi: 10.1002/14651858.CD009173.

13. Bruins Slot KMH, Berge E. Factor Xa inhibitors versus vitamin K antagonists for preventing cerebral or systemic embolism in patients with atrial fibrillation. Cochrane Database of Systematic Reviews. 2013, Issue 8. Art. No.: CD008980. doi: 10.1002/14651858.CD008980.pub2.

14. Bruins Slot KMH, Berge E. Factor Xa inhibitors versus vitamin K antagonists for preventing cerebral or systemic embolism in patients with atrial fibrillation (Protocol). Cochrane Database of Systematic Reviews. 2011, Issue 2. Art. No.: CD008980. doi: 10.1002/14651858.CD008980.

15. Cavalheri V, Tahirah F, Nonoyama M, Jenkins S, Hill K. Exercise training undertaken by people within 12 months of lung resection for non-small cell lung cancer. Cochrane Database of Systematic Reviews. 2013, Issue 7. Art. No.: CD009955. doi: 10.1002/14651858.CD009955.pub2.

16. Cavalheri V, Tahirah F, Nonoyama M, Jenkins S, Hill K. Exercise training undertaken within 12 months following lung resection for patients with non-small cell lung cancer (Protocol). Cochrane Database of Systematic Reviews. 2012, Issue 7. Art. No.: CD009955. doi: 10.1002/14651858.CD009955.

17. Chaparro LE, Smith SA, Moore RA, Wiffen PJ, Gilron I. Pharmacotherapy for the prevention of chronic pain after surgery in adults. Cochrane Database of Systematic Reviews. 2013, Issue 7. Art. No.: CD008307. doi: 10.1002/14651858.CD008307.pub2.

18. Gilron I, Moore RA, Wiffen PJ, McQuay HJ. Pharmacotherapy for the prevention of chronic pain after surgery in adults (Protocol). Cochrane Database of Systematic Reviews. 2010, Issue 1. Art. No.: CD008307. doi: 10.1002/14651858.CD008307.

19. Cheng Y, Lu J, Xiong X, Wu S, Lin Y, et al. Gases for establishing pneumoperitoneum during laparoscopic abdominal surgery. Cochrane Database of Systematic Reviews. 2013, Issue 1. Art. No.: CD009569. doi: 10.1002/14651858.CD009569.pub2.

20. Lu J, Cheng Y, Xiong X, Wu S, Lin Y, et al. Gases for establishing pneumoperitoneum during laparoscopic abdominal surgery (Protocol). Cochrane Database of Systematic Reviews. 2012, Issue 1. Art. No.: CD009569. doi: 10.1002/14651858.CD009569.

21. Cruciani M, Mengoli C, Serpelloni G, Parisi SG, Malena M, et al. Abacavir-based triple nucleoside regimens for maintenance therapy in patients with HIV. Cochrane Database of Systematic Reviews. 2013, Issue 6. Art. No.: CD008270. doi: 10.1002/14651858.CD008270.pub2.

22. Cruciani M, Mengoli C, Serpelloni G, Parisi SG. Abacavir-based triple nucleoside regimens for maintenance therapy in patients with HIV (Protocol). Cochrane Database of Systematic Reviews 2010, Issue 1. Art. No.: CD008270. doi: 10.1002/14651858.CD008270.

23. Dashash M, Yeung A, Sbenati A, Blinkhorn A. Interventions for the restorative care of amelogenesis imperfecta in children and adolescents (Protocol). Cochrane Database of Systematic Reviews 2008, Issue 2. Art. No.: CD007157. doi: 10.1002/14651858.CD007157.

24. Dashash M, Yeung CA, Jamous I, Blinkhorn A. Interventions for the restorative care of amelogenesis imperfecta in children and adolescents. Cochrane Database of Systematic Reviews. 2013, Issue 6. Art. No.: CD007157. doi: 10.1002/14651858.CD007157.pub2.

25. Deare JC, Zheng Z, Xue CCL, Liu JP, Shang J, et al. Acupuncture for treating fibromyalgia. Cochrane Database of Systematic Reviews. 2013, Issue 5. Art. No.: CD007070. doi: 10.1002/14651858.CD007070.pub2.

26. Deare JC, Zheng Z, Xue CC, Liu JP, Shang J, et al. Acupuncture for treating fibromyalgia (Protocol). Cochrane Database of Systematic Reviews 2008, Issue 2. Art. No.: CD007070. doi: 10.1002/14651858.CD007070.

27. Freak-Poli RLA, Cumpston M, Peeters A, Clemes SA. Workplace pedometer interventions for increasing physical activity (Protocol). Cochrane Database of Systematic Reviews. 2011, Issue 7. Art. No.: CD009209. doi: 10.1002/14651858.CD009209.

28. Freak-Poli RLA, Cumpston M, Peeters A, Clemes SA. Workplace pedometer interventions for increasing physical activity. Cochrane Database of Systematic Reviews. 2013, Issue 4. Art. No.: CD009209. doi: 10.1002/14651858.CD009209.pub2.

29. Gan T, Tian L, Jin SJ, Wang Y. Medicinal herbs for cholelithiasis (Protocol). Cochrane Database of Systematic Reviews. 2004, Issue 1. Art. No.: CD004547. doi: 10.1002/14651858.CD004547.

30. Gan T, Chen J, Jin SJ, Wang Y. Chinese medicinal herbs for cholelithiasis. Cochrane Database of Systematic Reviews. 2013, Issue 6. Art. No.: CD004547. doi: 10.1002/14651858.CD004547.pub2.

31. Gillies D, O'Brien L, Rogers P, Meekings C. Psychological therapies for the prevention and treatment of post-traumatic stress disorder in children and adolescents (Protocol). Cochrane Database of Systematic Reviews 2007, Issue 3. Art. No.: CD006726. doi: 10.1002/14651858.CD006726.

32. Gillies D, Taylor F, Gray C, O'Brien L, D'Abrew N. Psychological therapies for the treatment of post-traumatic stress disorder in children and adolescents. Cochrane Database of Systematic Reviews. 2012, Issue 12. Art. No.: CD006726. doi: 10.1002/14651858.CD006726.pub2.

33. Gois PHF, Souza ERDM, Santos CUD. Pharmacotherapy for hyperuricemia in hypertensive patients (Protocol). Cochrane Database of Systematic Reviews. 2010, Issue 7. Art. No.: CD008652. doi: 10.1002/14651858.CD008652.

34. Gois PHF, Souza ERDM. Pharmacotherapy for hyperuricemia in hypertensive patients. Cochrane Database of Systematic Reviews. 2013, Issue 1. Art. No.: CD008652. doi: 10.1002/14651858.CD008652.pub2.

35. Johnston BC, Thorlund K. Probiotics for the prevention of Clostridium difficile associated diarrhea in adults and children (Protocol). Cochrane Database of Systematic Reviews 2009, Issue 1. Art. No.: CD006095. doi: 10.1002/14651858.CD006095.pub2.

36. Goldenberg JZ, Ma SSY, Saxton JD, Martzen MR, Vandvik PO, et al. Probiotics for the prevention of Clostridium difficile-associated diarrhea in adults and children. Cochrane Database of Systematic Reviews. 2013, Issue 5. Art. No.: CD006095. doi: 10.1002/14651858.CD006095.pub3.

37. Leyngold I, Nanji AA, Chuck RS, Behrens A, Vedula SS, et al. Perioperative antibiotics for prevention of acute endophthalmitis after cataract surgery. (Protocol). Cochrane Database of Systematic Reviews 2007, Issue 1. Art. No.: CD006364. doi: 10.1002/14651858.CD006364.

38. Gower EW, Lindsley K, Nanji AA, Leyngold I, McDonnell PJ. Perioperative antibiotics for prevention of acute endophthalmitis after cataract surgery. Cochrane Database of Systematic Reviews. 2013, Issue 7. Art. No.: CD006364. doi: 10.1002/14651858.CD006364.pub2.

39. He D, Han K, Gao X, Dong S, Chu L, et al. Laquinimod for multiple sclerosis (Protocol). Cochrane Database of Systematic Reviews 2013, Issue 4. Art. No.: CD010475. doi: 10.1002/14651858.CD010475.

40. He D, Han K, Gao X, Dong S, Chu L, et al. Laquinimod for multiple sclerosis. Cochrane Database of Systematic Reviews. 2013, Issue 8. Art. No.: CD010475. doi: 10.1002/14651858.CD010475.pub2.

41. Itchaki G, Gafter-Gvili A, Lahav M, Vidal L, Raanani P, et al. Anthracycline-containing regimens for treatment of follicular lymphoma in adults. Cochrane Database of Systematic Reviews. 2013, Issue 7. Art. No.: CD008909. doi: 10.1002/14651858.CD008909.pub2.

42. Itchaki G, Gafter-Gvili A, Lahav M, Vidal L, Raanani P, et al. Anthracyclines-containing regimens for treatment of follicular lymphoma in adults (Protocol). Cochrane Database of Systematic Reviews 2010, Issue 12. Art. No.: CD008909. doi: 10.1002/14651858.CD008909.

43. Kinnersley P, Stephens BL, Elwyn GJ, Blazeby J, Kelly M, et al. Interventions to promote informed consent for patients undergoing surgical and other invasive healthcare procedures (Protocol). Cochrane Database of Systematic Reviews 2011, Issue 11. Art. No.: CD009445. doi: 10.1002/14651858.CD009445.

44. Kinnersley P, Phillips K, Savage K, Kelly MJ, Farrell E, et al. Interventions to promote informed consent for patients undergoing surgical and other invasive healthcare procedures. Cochrane Database of Systematic Reviews. 2013, Issue 7. Art. No.: CD009445. doi: 10.1002/14651858.CD009445.pub2.

45. Lawrie TA, Bryant A, Cameron A, Gray E, Morrison J. Pegylated liposomal doxorubicin for relapsed epithelial ovarian cancer. Cochrane Database of Systematic Reviews. 2013, Issue 7. Art. No.: CD006910. doi: 10.1002/14651858.CD006910.pub2.

46. Cameron AGE, Williams C. Pegylated liposomal doxorubicin for relapsed ovarian cancer (Protocol). Cochrane Database of Systematic Reviews 2008, Issue 1. Art. No.: CD006910. doi: 10.1002/14651858.CD006910.

47. Lee AL, Burge A, Jones AP, Rowe BH, Holland AE. Airway clearance techniques for bronchiectasis (Protocol). Cochrane Database of Systematic Reviews. 2010, Issue 2. Art. No.: CD008351. doi: 10.1002/14651858.CD008351.

48. Lee AL, Burge A, Holland AE. Airway clearance techniques for bronchiectasis. Cochrane Database of Systematic Reviews. 2013, Issue 5. Art. No.: CD008351. doi: 10.1002/14651858.CD008351.pub2.

49. Li S, Yue J, Dong BR, Yang M, Lin X, et al. Acetaminophen (paracetamol) for the common cold in adults (Protocol). Cochrane Database of Systematic Reviews. 2010, Issue 11. Art. No.: CD008800. doi: 10.1002/14651858.CD008800.

50. Li S, Yue J, Dong BR, Yang M, Lin X, et al. Acetaminophen (paracetamol) for the common cold in adults. Cochrane Database of Systematic Reviews. 2013, Issue 7. Art. No.: CD008800. doi: 10.1002/14651858.CD008800.pub2.

51. Liu ZL, Wu Q, Liu JP, Li GQ, Bensoussan A, et al. Chinese herbal medicines for hypertriglyceridemia (Protocol). Cochrane Database of Systematic Reviews. 2012, Issue 1. Art. No.: CD009560. doi: 10.1002/14651858.CD009560.

52. Liu ZL, Li GQ, Bensoussan A, Kiat H, Chan K, et al. Chinese herbal medicines for hypertriglyceridaemia. Cochrane Database of Systematic Reviews. 2013, Issue 6. Art. No.: CD009560. doi: 10.1002/14651858.CD009560.pub2.

53. Lopez LM, Hilgenberg D, Chen M, Denison J, Stuart G. Behavioral interventions for improving contraceptive use among women living with HIV. Cochrane Database of Systematic Reviews. 2013, Issue 1. Art. No.: CD010243. doi: 10.1002/14651858.CD010243.pub2.

54. Lopez LM, Chen M, Hilgenberg D, Denison J, Stuart G. Behavioral interventions for improving contraceptive use among women living with HIV (Protocol). Cochrane Database of Systematic Reviews. 2012, Issue 11. Art. No.: CD010243. doi: 10.1002/14651858.CD010243.

55. Marigold R, Günther A, Tiwari D, Kwan J. Antiepileptic drugs for the primary and secondary prevention of seizures after subarachnoid haemorrhage (Protocol). Cochrane Database of Systematic Reviews 2010, Issue 9. Art. No.: CD008710. doi: 10.1002/14651858.CD008710.

56. Marigold R, Günther A, Tiwari D, Kwan J. Antiepileptic drugs for the primary and secondary prevention of seizures after subarachnoid haemorrhage. Cochrane Database of Systematic Reviews. 2013, Issue 6. Art. No.: CD008710. doi: 10.1002/14651858.CD008710.pub2.

57. Mocellin S, Lens Marko B, Pasquali S, Pilati P, Chiarion Sileni V. Interferon alpha for the adjuvant treatment of cutaneous melanoma. Cochrane Database of Systematic Reviews. 2013, Issue 6. Art. No.: CD008955. doi: 10.1002/14651858.CD008955.pub2.

58. Mocellin S, Lens M, Pasquali S, Pilati P. Interferon alpha for the adjuvant treatment of cutaneous melanoma (Protocol). Cochrane Database of Systematic Reviews 2011, Issue 1. Art. No.: CD008955. doi: 10.1002/14651858.CD008955.

59. Mutua FM, M'Imunya MJ, Wiysonge CS. Genital ulcer disease treatment for reducing sexual transmission of HIV (Protocol). Cochrane Database of Systematic Reviews. 2009, Issue 3. Art. No.: CD007933. doi: 10.1002/14651858.CD007933.

60. Mutua FM, M'Imunya JM, Wiysonge CS. Genital ulcer disease treatment for reducing sexual acquisition of HIV. Cochrane Database of Systematic Reviews. 2012, Issue 8. Art. No.: CD007933. doi: 10.1002/14651858.CD007933.pub2.

61. Parker B, Turner W. Psychoanalytic/psychodynamic psychotherapy for children and adolescents who have been sexually abused (Protocol) Cochrane Database of Systematic Reviews. 2009, Issue 4. Art. No.: CD008162. doi: 10.1002/14651858.CD008162.

62. Parker B, Turner W. Psychoanalytic/psychodynamic psychotherapy for children and adolescents who have been sexually abused. Cochrane Database of Systematic Reviews. 2013, Issue 7. Art. No.: CD008162. doi: 10.1002/14651858.CD008162.pub2.

63. Pega F, Carter K, Blakely T, Lucas P. In-work tax credits for families and their impact on health status in adults (Protocol). Cochrane Database of Systematic Reviews 2012, Issue 7. Art. No.: CD009963 doi: 10.1002/14651858.CD009963.

64. Pega F, Carter K, Blakely T, Lucas PJ. In-work tax credits for families and their impact on health status in adults. Cochrane Database of Systematic Reviews. 2013, Issue 8. Art. No.: CD009963. doi: 10.1002/14651858.CD009963.pub2.

65. Penninga L, Penninga EI, Møller CH, Iversen M, Steinbrüchel DA, et al. Tacrolimus versus cyclosporin as primary immunosuppression for lung transplant recipients. Cochrane Database of Systematic Reviews. 2013, Issue 5. Art. No.: CD008817. doi: 10.1002/14651858.CD008817.pub2.

66. Penninga L, Penninga EI, Møller CH, Steinbrüchel DA, Gluud C. Tacrolimus versus cyclosporin as primary immunosuppression for lung transplant recipients (Protocol). Cochrane Database of Systematic Reviews. 2010, Issue 11. Art. No.: CD008817. doi: 10.1002/14651858.CD008817.

67. O'Connor D, Daborn C. Rehabilitation treatments following carpal tunnel surgery (Protocol). Cochrane Database of Systematic Reviews. 2003, Issue 2. Art. No.: CD004158. doi: 10.1002/14651858.CD004158.

68. Peters S, Page MJ, Coppieters MW, Ross M, Johnston V. Rehabilitation following carpal tunnel release. Cochrane Database of Systematic Reviews. 2013, Issue 6. Art. No.: CD004158. doi: 10.1002/14651858.CD004158.pub2.

69. Rockers PC, Bärnighausen T. Interventions for hiring, retaining and training district health system managers in low- and middle-income countries (Protocol). Cochrane Database of Systematic Reviews. 2011, Issue 3. Art. No.: CD009035. doi: 10.1002/14651858.CD009035.

70. Rockers PC, Bärnighausen T. Interventions for hiring, retaining and training district health systems managers in low- and middle-income countries. Cochrane Database of Systematic Reviews. 2013, Issue 4. Art. No.: CD009035. doi: 10.1002/14651858.CD009035.pub2.

71. Sajid MS, Hutson KH, Rapisarda IF, Bonomi R. Fibrin glue instillation under skin flaps to prevent seroma related morbidity following breast and axillary surgery (Protocol). Cochrane Database of Systematic Reviews 2012, Issue 1. Art. No.: CD009557. doi: 10.1002/14651858.CD009557.

72. Sajid MS, Hutson KH, Rapisarda IF, Bonomi R. Fibrin glue instillation under skin flaps to prevent seroma-related morbidity following breast and axillary surgery. Cochrane Database of Systematic Reviews. 2013, Issue 5. Art. No.: CD009557. doi: 10.1002/14651858.CD009557.pub2.

73. Sampson S, Mansour M, Maayan N, Soares-Weiser K, Adams CE. Intermittent drug techniques for schizophrenia. Cochrane Database of Systematic Reviews. 2013, Issue 7. Art. No.: CD006196. doi: 10.1002/14651858.CD006196.pub2.

74. Mansour M, Alomar A, Boobes K, Awf Mouchli M. Intermittent drug techniques for schizophrenia (Protocol) Cochrane Database of Systematic Reviews 2006, Issue 4. Art. No.: CD006196. doi: 10.1002/14651858.CD006196.

75. Sanders RD, Nicholson A, Lewis S R, Smith AF, Alderson P. Perioperative statin therapy for improving outcomes during and after noncardiac vascular surgery. Cochrane Database of Systematic Reviews. 2013, Issue 7. Art. No.: CD009971. doi: 10.1002/14651858.CD009971.pub2.

76. Sanders RD, Nicholson A, Lewis SR, Smith AF, Alderson P. Perioperative statin therapy for improving outcomes during and after noncardiac vascular surgery (Protocol). Cochrane Database of Systematic Reviews. 2012, Issue 7. Art. No.: CD009971. doi: 10.1002/14651858.CD009971.

77. Tejani AM, Chan AHW, Kuo IF, Li J. Magnesium for alcohol withdrawal (Protocol). Cochrane Database of Systematic Reviews 2010, Issue 2. Art. No.: CD008358. doi: 10.1002/14651858.CD008358.

78. Sarai M, Tejani AM, Chan AHW, Kuo IF, Li J. Magnesium for alcohol withdrawal. Cochrane Database of Systematic Reviews. 2013, Issue 6. Art. No.: CD008358. doi: 10.1002/14651858.CD008358.pub2.

79. Schoot RA, van Dalen EC, van Ommen CH, van de Wetering MD. Antibiotic and other lock treatments for tunnelled central venous catheter related infections in children with cancer (Protocol). Cochrane Database of Systematic Reviews. 2011, Issue 2. Art. No.: CD008975. doi: 10.1002/14651858.CD008975.

80. Schoot RA, van Dalen EC, van Ommen CH, van de Wetering MD. Antibiotic and other lock treatments for tunnelled central venous catheter-related infections in children with cancer. Cochrane Database of Systematic Reviews. 2013, Issue 6. Art. No.: CD008975. doi: 10.1002/14651858.CD008975.pub2.

81. Semple C, Parahoo K, Norman A, McCaughan E, Humphris G, et al. Psychosocial interventions for patients with head and neck cancer. Cochrane Database of Systematic Reviews. 2013, Issue 7. Art. No.: CD009441. doi: 10.1002/14651858.CD009441.pub2.

82. Semple C, Parahoo K, Mills M, Humphris G, McCaughan E, et al. Psychosocial interventions for patients with head and neck cancer (Protocol). Cochrane Database of Systematic Reviews 2011, Issue 11. Art. No.: CD009441. doi: 10.1002/14651858.CD009441.

83. Sharma SK, Sharma A, Kadhiravan T, Tharyan P. Isoniazid monotherapy versus other monotherapies or combination chemotherapy for preventing active tuberculosis in HIV-negative persons (Protocol). Cochrane Database of Systematic Reviews 2009, Issue 1. Art. No.: CD007545. doi: 10.1002/14651858.CD007545.

84. Sharma SK, Sharma A, Kadhiravan T, Tharyan P. Rifamycins (rifampicin, rifabutin and rifapentine) compared to isoniazid for preventing tuberculosis in HIV-negative people at risk of active TB. Cochrane Database of Systematic Reviews. 2013, Issue 7. Art. No.: CD007545. doi: 10.1002/14651858.CD007545.pub2.

85. Showell MG, Brown J, Clarke J, Hart RJ. Antioxidants for female subfertility. Cochrane Database of Systematic Reviews. 2013, Issue 8. Art. No.: CD007807. doi: 10.1002/14651858.CD007807.pub2.

86. Clarke J, Showell MG, Hart RJ, Agarwal A, Gupta S. Antioxidants for female subfertility (Protocol). Cochrane Database of Systematic Reviews. 2009, Issue 2. Art. No.: CD007807. doi: 10.1002/14651858.CD007807.

87. Stead LF, Lancaster T. Behavioural interventions as adjuncts to pharmacotherapy for smoking cessation. Cochrane Database of Systematic Reviews. 2012, Issue 12. Art. No.: CD009670. doi: 10.1002/14651858.CD009670.pub2.

88. Stead LF, Lancaster T. Behavioural interventions as adjuncts to pharmacotherapy for smoking cessation (Protocol). Cochrane Database of Systematic Reviews. 2012, Issue 2. Art. No.: CD009670. doi: 10.1002/14651858.CD009670.

89. Trivedi A, Sinn JKH. Early versus late administration of amino acids in preterm infants receiving parenteral nutrition (Protocol). Cochrane Database of Systematic Reviews. 2010, Issue 10. Art. No.: CD008771. doi: 10.1002/14651858.CD008771.

90. Trivedi A, Sinn JKH. Early versus late administration of amino acids in preterm infants receiving parenteral nutrition. Cochrane Database of Systematic Reviews. 2013, Issue 7. Art. No.: CD008771. doi: 10.1002/14651858.CD008771.pub2.

91. Trotti LM, Bhadriraju S, Becker LA. Iron for restless legs syndrome. Cochrane Database of Systematic Reviews. 2012, Issue 5. Art. No.: CD007834. doi: 10.1002/14651858.CD007834.pub2.

92. Trotti LM, Bhadriraju S, Becker LA. Iron for restless legs syndrome (Protocol). Cochrane Database of Systematic Reviews. 2009, Issue 2. Art. No.: CD007834. doi: 10.1002/14651858.CD007834.

93. Van Teeffelen S, Pajkrt E, Willekes C, Van Kuijk SMJ, Mol BWJ. Amnioinfusion for improving fetal outcomes after preterm prelabour rupture of membranes (Protocol). Cochrane Database of Systematic Reviews. 2012, Issue 7. Art. No.: CD009952. doi: 10.1002/14651858.CD009952.

94. Van Teeffelen S, Pajkrt E, Willekes C, Van Kuijk SMJ, Mol BWJ. Transabdominal amnioinfusion for improving fetal outcomes after oligohydramnios secondary to preterm prelabour rupture of membranes before 26 weeks. Cochrane Database of Systematic Reviews. 2013, Issue 8. Art. No.: CD009952. doi: 10.1002/14651858.CD009952.pub2.

95. Fedorowicz Z, van Zuuren EJ, A. AH. Low-molecular-weight heparins for managing vaso-occlusive crises in people with sickle cell disease (Protocol). Cochrane Database of Systematic Reviews. 2012, Issue 10. Art. No.: CD010155. doi: 10.1002/14651858.CD010155.

96. van Zuuren EJ, Fedorowicz Z. Low-molecular-weight heparins for managing vaso-occlusive crises in people with sickle cell disease. Cochrane Database of Systematic Reviews. 2013, Issue 6. Art. No.: CD010155. doi: 10.1002/14651858.CD010155.pub2.

97. Wakai A, McMahon G. Nitrates for acute heart failure (Protocol). Cochrane Database of Systematic Reviews. 2005, Issue 1. Art. No.: CD005151. doi: 10.1002/14651858.CD005151.

98. Wakai A, McCabe A, Kidney R, Brooks SC, Seupaul RA, et al. Nitrates for acute heart failure syndromes. Cochrane Database of Systematic Reviews. 2013, Issue 8. Art. No.: CD005151. doi: 10.1002/14651858.CD005151.pub2.

99. Wang Y, Zhishun L, Peng W. Acupuncture for stress urinary incontinence in adults (Protocol) Cochrane Database of Systematic Reviews. 2011, Issue 10. Art. No.: CD009408. doi: 0.1002/14651858.CD009408.

100. Wang Y, Zhishun L, Peng W, Zhao J, Liu B. Acupuncture for stress urinary incontinence in adults. Cochrane Database of Systematic Reviews. 2013, Issue 7. Art. No.: CD009408. doi: 10.1002/14651858.CD009408.pub2.

101. Yue J, Dong Bi R, Yang M, Chen X, Wu T, et al. Linezolid versus vancomycin for skin and soft tissue infections. Cochrane Database of Systematic Reviews. 2013, Issue 7. Art. No.: CD008056. doi: 10.1002/14651858.CD008056.pub2.

102. Yue J, Dong BR, Yang M, Chen X, Wu T, et al. Linezolid versus vancomycin for skin and soft tissue infections (Protocol). Cochrane Database of Systematic Reviews 2009, Issue 4. Art. No.: CD008056. doi: 10.1002/14651858.CD008056.

103. Ziebell M, Wetterslev J, Tisell M, Gluud C, Juhler M. Flow-regulated versus differential pressure-regulated shunt valves for adult patients with normal pressure hydrocephalus (Protocol) Cochrane Database of Systematic Reviews. 2012, Issue 3. Art. No.: CD009706. doi: 10.1002/14651858.CD009706.

104. Ziebell M, Wetterslev J, Tisell M, Gluud C, Juhler M. Flow-regulated versus differential pressure-regulated shunt valves for adult patients with normal pressure hydrocephalus. Cochrane Database of Systematic Reviews. 2013, Issue 5. Art. No.: CD009706. doi: 10.1002/14651858.CD009706.pub2.
