# Supplementary material for: Exploring Treatment by Covariate Interactions Using Subgroup Analysis and Meta-Regression in Cochrane Reviews: A Review of Recent Practice
Source: PLoS One. 2015 Jun 1;10(6):e0128804. doi: 10.1371/journal.pone.0128804 (PMC4452239; doi:10.1371/journal.pone.0128804)
Supplement: S2 Table — (DOCX) [file pone.0128804.s004.docx]

**Table S2: Covariates mentioned and/or analysed.**

| **Review** | **Covariate^1^** | **Choosing covariates.** | | **Description/Choosing covariate data^2^** | **Analysing covariates^3^** | **Detecting interactions** |
| --- | --- | --- | --- | --- | --- | --- |
| Aboumarzouk 2012 | **Protocol:** ‘Any medication that interacts with pregabalin or other concurrent neuropathic analgesics’.  **Review methods:** Not reported.  **Review results:** Not analysed.  **Type:** Intervention.  **Covariate summary:** Type of intervention or control. | **Method to choose**  **covariate:** Not reported.  **Where method reported:** NA.  **Rationale for choosing the covariate:** Not reported.  **Where rationale reported:** NA. | **Where reported:** Protocol only.  **Reason not reported in review:** Not reported.  **Reason not reported in protocol:** NA.  **Labelled as ‘posthoc’ if not in protocol:** NA. | **Data type:** Categorical.  **Continuous covariate categorised:** NA.  **How determine it was categorised:** NA.  **Justification for categorising:** NA.  **Where justification reported:** NA.  **Categories reported:** Not reported.  **Where categories reported:** NA.  **Justification for categories:** Not reported.  **Where justification reported:** NA. | **Analysis type reported in protocol:** Subgroup analysis or meta-regression.  **Analysis type reported in review:** Not reported.  **Reason for changing mind:** Not reported.    **Analysis type in protocol according to glossary:** Subgroup analysis or meta-regression.  **How determined the analysis type:** Says ‘Subgroup analysis or meta-regression.’. No reason to dispute.  **Analysis type in review according to glossary:** Not reported.  **How determined the analysis type:** NA.  **Analysed:** No.  **Reasons given (in the review) for not doing interaction analysis if planned:** Not reported (presumably because one trial).  **Reasons given for carrying out interaction analysis if they weren’t planned:** NA.  **Scenario in protocol:** Not reported.  **Rationale for planned scenario:** NA.  **Scenario in review (if reported specifically):** Not reported.  **Rationale for done scenario:** NA.  **Reason for changing mind:** NA. | **Method to detect interactions reported in protocol:** Not reported.  **Method to detect interactions reported in review:** Not reported.  **Reason for changing mind:** NA. |
| Aboumarzouk 2012 | **Protocol:** ‘Comorbidities or painful conditions in other parts of the body, i. e. fibromyalgia, peripheral/Ischaemic vascular disease, anal fissures, etc.’  **Review methods:** Not reported.  **Review results:** Not analysed.  **Type:** Patient.  **Covariate summary:** Disease characteristics. | **Method to choose**  **covariate:** Not reported.  **Where method reported:** NA.  **Rationale for choosing the covariate:** Not reported.  **Where rationale reported:** NA. | **Where reported:** Protocol only.  **Reason not reported in review:** Not reported.  **Reason not reported in protocol:** NA.  **Labelled as ‘posthoc’ if not in protocol:** NA. | **Data type:** Categorical.  **Continuous covariate categorised:** NA.  **How determine it was categorised:** NA.  **Justification for categorising:** NA.  **Where justification reported:** NA.  **Categories reported:** Not reported.  **Where categories reported:** NA.  **Justification for categories:** Not reported.  **Where justification reported:** NA. | **Analysis type reported in protocol:** Subgroup analysis or meta-regression.  **Analysis type reported in review:** Not reported.  **Reason for changing mind:** Not reported.    **Analysis type in protocol according to glossary:** Subgroup analysis or meta-regression.  **How determined the analysis type:** Says ‘Subgroup analysis or meta-regression.’. No reason to dispute.  **Analysis type in review according to glossary:** Not reported.  **How determined the analysis type:** NA.  **Analysed:** No.  **Reasons given (in the review) for not doing interaction analysis if planned:** Not reported (presumably because one trial).  **Reasons given for carrying out interaction analysis if they weren’t planned:** NA.  **Scenario in protocol:** Not reported.  **Rationale for planned scenario:** NA.  **Scenario in review (if reported specifically):** Not reported.  **Rationale for done scenario:** NA.  **Reason for changing mind:** NA. | **Method to detect interactions reported in protocol:** Not reported.  **Method to detect interactions reported in review:** Not reported.  **Reason for changing mind:** NA. |
| Aboumarzouk 2012 | **Protocol:** ‘Different dosages of pregabalin’.  **Review methods:** Not reported.  **Review results:** Not analysed.  **Type:** Intervention.  **Covariate summary:** Dose. | **Method to choose**  **covariate:** Not reported.  **Where method reported:** NA.  **Rationale for choosing the covariate:** Not reported.  **Where rationale reported:** NA. | **Where reported:** Protocol only.  **Reason not reported in review:** Not reported.  **Reason not reported in protocol:** NA.  **Labelled as ‘posthoc’ if not in protocol:** NA. | **Data type:** Continuous.  **Continuous covariate categorised:** Unclear.  **How determine it was categorised:** NA.  **Justification for categorising:** NA..  **Where justification reported:** NA  **Categories reported:** Not reported.  **Where categories reported:** NA.  **Justification for categories:** Not reported.  **Where justification reported:** NA. | **Analysis type reported in protocol:** Subgroup analysis or meta-regression.  **Analysis type reported in review:** Not reported.  **Reason for changing mind:** Not reported.    **Analysis type in protocol according to glossary:** Subgroup analysis or meta-regression.  **How determined the analysis type:** Says ‘Subgroup analysis or meta-regression.’. No reason to dispute.  **Analysis type in review according to glossary:** Not reported.  **How determined the analysis type:** NA.  **Analysed:** No.  **Reasons given (in the review) for not doing interaction analysis if planned:** Not reported (presumably because one trial).  **Reasons given for carrying out interaction analysis if they weren’t planned:** NA.  **Scenario in protocol:** Not reported.  **Rationale for planned scenario:** NA.  **Scenario in review (if reported specifically):** Not reported.  **Rationale for done scenario:** NA.  **Reason for changing mind:** NA. | **Method to detect interactions reported in protocol:** Not reported.  **Method to detect interactions reported in review:** Not reported.  **Reason for changing mind:** NA. |
| Aboumarzouk 2012 | **Protocol:** ‘Different duration of treatment (treatment for weeks, months, or years)’.  **Review methods:** Not reported.  **Review results:** Not analysed.  **Type:** Intervention.  **Covariate summary:** Duration of intervention. | **Method to choose**  **covariate:** Not reported.  **Where method reported:** NA.  **Rationale for choosing the covariate:** Not reported.  **Where rationale reported:** NA. | **Where reported:** Protocol only.  **Reason not reported in review:** Not reported.  **Reason not reported in protocol:** NA.  **Labelled as ‘posthoc’ if not in protocol:** NA. | **Data type:** Continuous.  **Continuous covariate categorised:** Unclear.  **How determine it was categorised:** NA.  **Justification for categorising:** NA..  **Where justification reported:** NA  **Categories reported:** Not reported.  **Where categories reported:** NA.  **Justification for categories:** Not reported.  **Where justification reported:** NA. | **Analysis type reported in protocol:** Subgroup analysis or meta-regression.  **Analysis type reported in review:** Not reported.  **Reason for changing mind:** Not reported.    **Analysis type in protocol according to glossary:** Subgroup analysis or meta-regression.  **How determined the analysis type:** Says ‘Subgroup analysis or meta-regression.’. No reason to dispute.  **Analysis type in review according to glossary:** Not reported.  **How determined the analysis type:** NA.  **Analysed:** No.  **Reasons given (in the review) for not doing interaction analysis if planned:** Not reported (presumably because one trial).  **Reasons given for carrying out interaction analysis if they weren’t planned:** NA.  **Scenario in protocol:** Not reported.  **Rationale for planned scenario:** NA.  **Scenario in review (if reported specifically):** Not reported.  **Rationale for done scenario:** NA.  **Reason for changing mind:** NA. | **Method to detect interactions reported in protocol:** Not reported.  **Method to detect interactions reported in review:** Not reported.  **Reason for changing mind:** NA. |
| Aboumarzouk 2012 | **Protocol:** ‘Intervention - Pregabalin and different control’.  **Review methods:** Not reported.  **Review results:** Not analysed.  **Type:** Intervention.  **Covariate summary:** Type of intervention or control. | **Method to choose**  **covariate:** Not reported.  **Where method reported:** NA.  **Rationale for choosing the covariate:** Not reported.  **Where rationale reported:** NA. | **Where reported:** Protocol only.  **Reason not reported in review:** Not reported.  **Reason not reported in protocol:** NA.  **Labelled as ‘posthoc’ if not in protocol:** NA. | **Data type:** Categorical.  **Continuous covariate categorised:** NA.  **How determine it was categorised:** NA.  **Justification for categorising:** NA.  **Where justification reported:** NA.  **Categories reported:** Not reported.  **Where categories reported:** NA.  **Justification for categories:** Not reported.  **Where justification reported:** NA. | **Analysis type reported in protocol:** Subgroup analysis or meta-regression.  **Analysis type reported in review:** Not reported.  **Reason for changing mind:** Not reported.    **Analysis type in protocol according to glossary:** Subgroup analysis or meta-regression.  **How determined the analysis type:** Says ‘Subgroup analysis or meta-regression.’. No reason to dispute.  **Analysis type in review according to glossary:** Not reported.  **How determined the analysis type:** NA.  **Analysed:** No.  **Reasons given (in the review) for not doing interaction analysis if planned:** Not reported (presumably because one trial).  **Reasons given for carrying out interaction analysis if they weren’t planned:** NA.  **Scenario in protocol:** Not reported.  **Rationale for planned scenario:** NA.  **Scenario in review (if reported specifically):** Not reported.  **Rationale for done scenario:** NA.  **Reason for changing mind:** NA. | **Method to detect interactions reported in protocol:** Not reported.  **Method to detect interactions reported in review:** Not reported.  **Reason for changing mind:** NA. |
| Aboumarzouk 2012 | **Protocol:** ‘Study quality (i. e. analyses of the impact of studies with poor methodology on the final result)’.  **Review methods:** Not reported.  **Review results:** Not analysed.  **Type:** Methodological.  **Covariate summary:** Quality. | **Method to choose**  **covariate:** Not reported.  **Where method reported:** NA.  **Rationale for choosing the covariate:** Not reported.  **Where rationale reported:** NA. | **Where reported:** Protocol only.  **Reason not reported in review:** Not reported.  **Reason not reported in protocol:** NA.  **Labelled as ‘posthoc’ if not in protocol:** NA. | **Data type:** Categorical.  **Continuous covariate categorised:** NA.  **How determine it was categorised:** NA.  **Justification for categorising:** NA.  **Where justification reported:** NA.  **Categories reported:** Not reported.  **Where categories reported:** NA.  **Justification for categories:** Not reported.  **Where justification reported:** NA. | **Analysis type reported in protocol:** Sensitivity analysis.  **Analysis type reported in review:** Not reported.  **Reason for changing mind:** Not reported.    **Analysis type in protocol according to glossary:** Sensitivity analysis.  **How determined the analysis type:** Says ‘sensitivity analysis’. No reason to dispute.  **Analysis type in review according to glossary:** Not reported.  **How determined the analysis type:** NA.  **Analysed:** No.  **Reasons given (in the review) for not doing interaction analysis if planned:** Not reported (presumably because one trial).  **Reasons given for carrying out interaction analysis if they weren’t planned:** NA.  **Scenario in protocol:** Not reported.  **Rationale for planned scenario:** NA.  **Scenario in review (if reported specifically):** Not reported.  **Rationale for done scenario:** NA.  **Reason for changing mind:** NA. | **Method to detect interactions reported in protocol:** Not reported.  **Method to detect interactions reported in review:** Not reported.  **Reason for changing mind:** NA. |
| Almeida 2013 | **Protocol:** ‘Activity (recreational versus professional athletes)’.  **Review methods: ‘**Activity (recreational versus professional athletes)’.  **Review results:** Not analysed.  **Type:** Patient.  **Covariate summary:** Demographics. | **Method to choose**  **covariate:** Not reported.  **Where method reported:** NA.  **Rationale for choosing the covariate:** Not reported.  **Where rationale reported:** NA. | **Where reported:** Protocol and review.  **Reason not reported in review:** NA.  **Reason not reported in protocol:** NA.  **Labelled as ‘posthoc’ if not in protocol:** NA. | **Data type:** Categorical.  **Continuous covariate categorised:** NA.  **How determine it was categorised:** NA.  **Justification for categorising:** NA.  **Where justification reported:** NA.  **Categories reported:** Yes.  **Where categories reported:** Protocol and review.  **Justification for categories:** Not reported.  **Where justification reported:** NA. | **Analysis type reported in protocol:** Subgroup analysis.  **Analysis type reported in review:** Subgroup analysis.  **Reason for changing mind:** NA.    **Analysis type in protocol according to glossary:** Subgroup analysis.  **How determined the analysis type:** Says ‘Subgroup analysis’. No reason to dispute.  **Analysis type in review according to glossary:** Subgroup analysis.  **How determined the analysis type:** Says ‘Subgroup analysis’. No reason to dispute.  **Analysed:** No.  **Reasons given (in the review) for not doing interaction analysis if planned:** ‘If data had been available’  **Reasons given for carrying out interaction analysis if they weren’t planned:** NA.  **Scenario in protocol:** ‘  ‘If data are available to investigate heterogeneous results for specific  patient groups, we will consider performing subgroup analyses’  **Rationale for planned scenario:** Not reported.  **Scenario in review (if reported specifically):** ‘If data had been available, we planned to investigate heterogeneous  results for specific patient groups, performing subgroup analyses’  **Rationale for done scenario:** Not reported.  **Reason for changing mind:** NA. | **Method to detect interactions reported in protocol:** We will investigate whether the results of subgroups are significantly  different by inspecting the overlap of confidence intervals,  and performing the test for subgroup differences available in  RevMan.  **Method to detect interactions reported in review:** We planned to investigate  whether the results of subgroups were significantly different by  inspecting the overlap of confidence intervals and performing the  test for subgroup differences that is available in RevMan.  **Reason for changing mind:** NA. |
| Almeida 2013 | **Protocol:** ‘Duration of symptoms’.  **Review methods: ‘**Duration of symptoms’.  **Review results:** Not analysed.  **Type:** Patient.  **Covariate summary:** Disease characteristics. | **Method to choose**  **covariate:** Not reported.  **Where method reported:** NA.  **Rationale for choosing the covariate:** Not reported.  **Where rationale reported:** NA. | **Where reported:** Protocol and review.  **Reason not reported in review:** NA.  **Reason not reported in protocol:** NA.  **Labelled as ‘posthoc’ if not in protocol:** NA. | **Data type:** Continuous.  **Continuous covariate categorised:** Yes.  **How determine it was categorised:** Subgroup analysis planned.  **Justification for categorising:** Not reported.  **Where justification reported:** NA.  **Categories reported:** Not reported.  **Where categories reported:** NA.  **Justification for categories:** Not reported.  **Where justification reported:** NA. | **Analysis type reported in protocol:** Subgroup analysis.  **Analysis type reported in review:** Subgroup analysis.  **Reason for changing mind:** NA.    **Analysis type in protocol according to glossary:** Subgroup analysis.  **How determined the analysis type:** Says ‘Subgroup analysis’. No reason to dispute.  **Analysis type in review according to glossary:** Subgroup analysis.  **How determined the analysis type:** Says ‘Subgroup analysis’. No reason to dispute.  **Analysed:** No.  **Reasons given (in the review) for not doing interaction analysis if planned:** ‘If data had been available’  **Reasons given for carrying out interaction analysis if they weren’t planned:** NA.  **Scenario in protocol:** ‘  ‘If data are available to investigate heterogeneous results for specific  patient groups, we will consider performing subgroup analyses’  **Rationale for planned scenario:** Not reported.  **Scenario in review (if reported specifically):** ‘If data had been available, we planned to investigate heterogeneous  results for specific patient groups, performing subgroup analyses’  **Rationale for done scenario:** Not reported.  **Reason for changing mind:** NA. | **Method to detect interactions reported in protocol:** We will investigate whether the results of subgroups are significantly  different by inspecting the overlap of confidence intervals,  and performing the test for subgroup differences available in  RevMan.  **Method to detect interactions reported in review:** We planned to investigate  whether the results of subgroups were significantly different by  inspecting the overlap of confidence intervals and performing the  test for subgroup differences that is available in RevMan.  **Reason for changing mind:** NA. |
| Almeida 2013 | **Protocol:** ‘Gender’.  **Review methods: ‘**Gender’.  **Review results:** Not analysed.  **Type:** Patient.  **Covariate summary:** Demographics. | **Method to choose**  **covariate:** Not reported.  **Where method reported:** NA.  **Rationale for choosing the covariate:** Not reported.  **Where rationale reported:** NA. | **Where reported:** Protocol and review.  **Reason not reported in review:** NA.  **Reason not reported in protocol:** NA.  **Labelled as ‘posthoc’ if not in protocol:** NA. | **Data type:** Categorical.  **Continuous covariate categorised:** NA.  **How determine it was categorised:** NA.  **Justification for categorising:** NA.  **Where justification reported:** NA.  **Categories reported:** Not reported.  **Where categories reported:** NA.  **Justification for categories:** Not reported.  **Where justification reported:** NA. | **Analysis type reported in protocol:** Subgroup analysis.  **Analysis type reported in review:** Subgroup analysis.  **Reason for changing mind:** NA.    **Analysis type in protocol according to glossary:** Subgroup analysis.  **How determined the analysis type:** Says ‘Subgroup analysis’. No reason to dispute.  **Analysis type in review according to glossary:** Subgroup analysis.  **How determined the analysis type:** Says ‘Subgroup analysis’. No reason to dispute.  **Analysed:** No.  **Reasons given (in the review) for not doing interaction analysis if planned:** ‘If data had been available’  **Reasons given for carrying out interaction analysis if they weren’t planned:** NA.  **Scenario in protocol:** ‘  ‘If data are available to investigate heterogeneous results for specific  patient groups, we will consider performing subgroup analyses’  **Rationale for planned scenario:** Not reported.  **Scenario in review (if reported specifically):** ‘If data had been available, we planned to investigate heterogeneous  results for specific patient groups, performing subgroup analyses’  **Rationale for done scenario:** Not reported.  **Reason for changing mind:** NA. | **Method to detect interactions reported in protocol:** We will investigate whether the results of subgroups are significantly  different by inspecting the overlap of confidence intervals,  and performing the test for subgroup differences available in  RevMan.  **Method to detect interactions reported in review:** We planned to investigate  whether the results of subgroups were significantly different by  inspecting the overlap of confidence intervals and performing the  test for subgroup differences that is available in RevMan.  **Reason for changing mind:** NA. |
| Almeida 2013 | **Protocol:** ‘Our main comparisons will be conservative intervention versus no or placebo; one conservative intervention versus another conservative intervention’.  **Review methods:** ‘Our main comparisons were conservative intervention (single or complex intervention) versus placebo intervention; one conservative intervention (single or complex intervention) versus another conservative intervention (single or complex intervention)’.  **Review results:** ‘Exercise therapy versus conventional physiotherapy’; ‘Multi-modal therapy versus exercise therapy’.  **Type:** Intervention.  **Covariate summary:** Type of intervention or control. | **Method to choose**  **covariate:** Not reported.  **Where method reported:** NA.  **Rationale for choosing the covariate:** Not reported.  **Where rationale reported:** NA. | **Where reported:** Protocol and review.  **Reason not reported in review:** NA.  **Reason not reported in protocol:** NA.  **Labelled as ‘posthoc’ if not in protocol:** NA. | **Data type:** Categorical.  **Continuous covariate categorised:** NA.  **How determine it was categorised:** NA.  **Justification for categorising:** NA.  **Where justification reported:** NA.  **Categories reported:** Yes.  **Where categories reported:** Protocol and review.  **Justification for categories:** Not reported.  **Where justification reported:** NA. | **Analysis type reported in protocol:** Unnamed analysis.  **Analysis type reported in review:** Unnamed analysis.  **Reason for changing mind:** NA.    **Analysis type in protocol according to glossary:** Stratification/subgroup analysis.  **How determined the analysis type:** Categories given.  **Analysis type in review according to glossary:** Stratification/subgroup analysis.  **How determined the analysis type:** Categories given.  **Analysed:** Yes.  **Reasons given (in the review) for not doing interaction analysis if planned:** NA.  **Reasons given for carrying out interaction analysis if they weren’t planned:** NA.  **Scenario in protocol:** Not reported.  **Rationale for planned scenario:** NA.  **Scenario in review (if reported specifically):** Not reported.  **Rationale for done scenario:** NA.  **Reason for changing mind:** NA. | **Method to detect interactions reported in protocol:** Not reported.  **Method to detect interactions reported in review:** Not reported.  **Reason for changing mind:** NA. |
| Almeida 2013 | **Protocol:** Assessor blinding. We judged the following to be four covariates: ‘Where possible, we plan sensitivity analyses examining various aspects of study and review methodology, including the inclusion of studies at high risk of bias (specifically from lack of allocation concealment, assessor blinding, and incomplete outcome data), and inclusion of trials only reported in abstracts’  **Review methods:** Assessor blinding. We judged the following to be four covariates: ‘Where possible, we planned sensitivity analyses examining various aspects of study and review methodology, including the inclusion of studies at high risk of bias (specifically from lack of allocation concealment, assessor blinding, and incomplete outcome data), and inclusion of trials only reported in abstracts.’  **Review results:** Not analysed.  **Type:** Methodological.  **Covariate summary:** Blinding. | **Method to choose**  **covariate:** Not reported.  **Where method reported:** NA.  **Rationale for choosing the covariate:** Not reported.  **Where rationale reported:** NA. | **Where reported:** Protocol and review.  **Reason not reported in review:** NA.  **Reason not reported in protocol:** NA.  **Labelled as ‘posthoc’ if not in protocol:** NA. | **Data type:** Categorical.  **Continuous covariate categorised:** NA.  **How determine it was categorised:** NA.  **Justification for categorising:** NA.  **Where justification reported:** NA.  **Categories reported:** Yes.  **Where categories reported:** Protocol and review.  **Justification for categories:** Not reported.  **Where justification reported:** NA. | **Analysis type reported in protocol:** Sensitivity analysis.  **Analysis type reported in review:** Sensitivity analysis.  **Reason for changing mind:** NA.    **Analysis type in protocol according to glossary:** Sensitivity analysis.  **How determined the analysis type:** Categories given.  **Analysis type in review according to glossary:** Sensitivity analysis.  **How determined the analysis type:** Categories given.  **Analysed:** No.  **Reasons given (in the review) for not doing interaction analysis if planned:** Not reported (presumably because one trial).  **Reasons given for carrying out interaction analysis if they weren’t planned:** NA.  **Scenario in protocol:** ‘where possible’.  **Rationale for planned scenario:** Not reported.  **Scenario in review (if reported specifically):** ‘where possible’.  **Rationale for done scenario:** Not reported.  **Reason for changing mind:** NA. | **Method to detect interactions reported in protocol:** Not reported.  **Method to detect interactions reported in review:** Not reported.  **Reason for changing mind:** NA. |
| Almeida 2013 | **Protocol:** Inclusion of trials only reported in abstracts. We judged the following to be four covariates: ‘Where possible, we plan sensitivity analyses examining various aspects of study and review methodology, including the inclusion of studies at high risk of bias (specifically from lack of allocation concealment, assessor blinding, and incomplete outcome data), and inclusion of trials only reported in abstracts.’  **Review methods:** Inclusion of trials only reported in abstracts. We judged the following to be four covariates: ‘Where possible, we planned sensitivity analyses examining various aspects of study and review methodology, including the inclusion of studies at high risk of bias (specifically from lack of allocation concealment, assessor blinding, and incomplete outcome data), and inclusion of trials only reported in abstracts.’  **Review results:** Not analysed.  **Type:** Methodological.  **Covariate summary:** Publication status. | **Method to choose**  **covariate:** Not reported.  **Where method reported:** NA.  **Rationale for choosing the covariate:** Not reported.  **Where rationale reported:** NA. | **Where reported:** Protocol and review.  **Reason not reported in review:** NA.  **Reason not reported in protocol:** NA.  **Labelled as ‘posthoc’ if not in protocol:** NA. | **Data type:** Categorical.  **Continuous covariate categorised:** NA.  **How determine it was categorised:** NA.  **Justification for categorising:** NA.  **Where justification reported:** NA.  **Categories reported:** Yes.  **Where categories reported:** Protocol and review.  **Justification for categories:** Not reported.  **Where justification reported:** NA. | **Analysis type reported in protocol:** Sensitivity analysis.  **Analysis type reported in review:** Sensitivity analysis.  **Reason for changing mind:** NA.    **Analysis type in protocol according to glossary:** Sensitivity analysis.  **How determined the analysis type:** Categories given.  **Analysis type in review according to glossary:** Sensitivity analysis.  **How determined the analysis type:** Categories given.  **Analysed:** No.  **Reasons given (in the review) for not doing interaction analysis if planned:** Not reported (presumably because one trial).  **Reasons given for carrying out interaction analysis if they weren’t planned:** NA.  **Scenario in protocol:** ‘where possible’.  **Rationale for planned scenario:** Not reported.  **Scenario in review (if reported specifically):** ‘where possible’.  **Rationale for done scenario:** Not reported.  **Reason for changing mind:** NA. | **Method to detect interactions reported in protocol:** Not reported.  **Method to detect interactions reported in review:** Not reported.  **Reason for changing mind:** NA. |
| Almeida 2013 | **Protocol:** Incomplete outcome data. We judged the following to be four covariates: ‘Where possible, we plan sensitivity analyses examining various aspects of study and review methodology, including the inclusion of studies at high risk of bias (specifically from lack of allocation concealment, assessor blinding, and incomplete outcome data), and inclusion of trials only reported in abstracts.’  **Review methods:** Incomplete outcome data. We judged the following to be four covariates: ‘Where possible, we planned sensitivity analyses examining various aspects of study and review methodology, including the inclusion of studies at high risk of bias (specifically from lack of allocation concealment, assessor blinding, and incomplete outcome data), and inclusion of trials only reported in abstracts.’  **Review results:** Not analysed.  **Type:** Methodological.  **Covariate summary:** Incomplete outcome data/follow up. | **Method to choose**  **covariate:** Not reported.  **Where method reported:** NA.  **Rationale for choosing the covariate:** Not reported.  **Where rationale reported:** NA. | **Where reported:** Protocol and review.  **Reason not reported in review:** NA.  **Reason not reported in protocol:** NA.  **Labelled as ‘posthoc’ if not in protocol:** NA. | **Data type:** Categorical.  **Continuous covariate categorised:** NA.  **How determine it was categorised:** NA.  **Justification for categorising:** NA.  **Where justification reported:** NA.  **Categories reported:** Yes.  **Where categories reported:** Protocol and review.  **Justification for categories:** Not reported.  **Where justification reported:** NA. | **Analysis type reported in protocol:** Sensitivity analysis.  **Analysis type reported in review:** Sensitivity analysis.  **Reason for changing mind:** NA.    **Analysis type in protocol according to glossary:** Sensitivity analysis.  **How determined the analysis type:** Categories given.  **Analysis type in review according to glossary:** Sensitivity analysis.  **How determined the analysis type:** Categories given.  **Analysed:** No.  **Reasons given (in the review) for not doing interaction analysis if planned:** Not reported (presumably because one trial).  **Reasons given for carrying out interaction analysis if they weren’t planned:** NA.  **Scenario in protocol:** ‘where possible’.  **Rationale for planned scenario:** Not reported.  **Scenario in review (if reported specifically):** ‘where possible’.  **Rationale for done scenario:** Not reported.  **Reason for changing mind:** NA. | **Method to detect interactions reported in protocol:** Not reported.  **Method to detect interactions reported in review:** Not reported.  **Reason for changing mind:** NA. |
| Almeida 2013 | **Protocol:** Lack of allocation concealment. We judged the following to be four covariates: ‘Where possible, we plan sensitivity analyses examining various aspects of study and review methodology, including the inclusion of studies at high risk of bias (specifically from lack of allocation concealment, assessor blinding, and incomplete outcome data), and inclusion of trials only reported in abstracts.’  **Review methods:** Lack of allocation concealment. We judged the following to be four covariates: ‘Where possible, we planned sensitivity analyses examining various aspects of study and review methodology, including the inclusion of studies at high risk of bias (specifically from lack of allocation concealment, assessor blinding, and incomplete outcome data), and inclusion of trials only reported in abstracts.’  **Review results:** Not analysed.  **Type:** Methodological.  **Covariate summary:** Allocation concealment. | **Method to choose**  **covariate:** Not reported.  **Where method reported:** NA.  **Rationale for choosing the covariate:** Not reported.  **Where rationale reported:** NA. | **Where reported:** Protocol and review.  **Reason not reported in review:** NA.  **Reason not reported in protocol:** NA.  **Labelled as ‘posthoc’ if not in protocol:** NA. | **Data type:** Categorical.  **Continuous covariate categorised:** NA.  **How determine it was categorised:** NA.  **Justification for categorising:** NA.  **Where justification reported:** NA.  **Categories reported:** Yes.  **Where categories reported:** Protocol and review.  **Justification for categories:** Not reported.  **Where justification reported:** NA. | **Analysis type reported in protocol:** Sensitivity analysis.  **Analysis type reported in review:** Sensitivity analysis.  **Reason for changing mind:** NA.    **Analysis type in protocol according to glossary:** Sensitivity analysis.  **How determined the analysis type:** Categories given.  **Analysis type in review according to glossary:** Sensitivity analysis.  **How determined the analysis type:** Categories given.  **Analysed:** No.  **Reasons given (in the review) for not doing interaction analysis if planned:** Not reported (presumably because one trial).  **Reasons given for carrying out interaction analysis if they weren’t planned:** NA.  **Scenario in protocol:** ‘where possible’.  **Rationale for planned scenario:** Not reported.  **Scenario in review (if reported specifically):** ‘where possible’.  **Rationale for done scenario:** Not reported.  **Reason for changing mind:** NA. | **Method to detect interactions reported in protocol:** Not reported.  **Method to detect interactions reported in review:** Not reported.  **Reason for changing mind:** NA. |
| Basurto Ona 2013 | **Protocol:** ‘Disease aetiology (alcohol versus other causes)’.  **Review methods: ‘**Disease aetiology (alcohol versus other causes)’.  **Review results:** Not analysed.  **Type:** Patient.  **Covariate summary:** Disease characteristics. | **Method to choose**  **covariate:** Not reported.  **Where method reported:** NA.  **Rationale for choosing the covariate:** Not reported.  **Where rationale reported:** NA. | **Where reported:** Protocol and review.  **Reason not reported in review:** NA.  **Reason not reported in protocol:** NA.  **Labelled as ‘posthoc’ if not in protocol:** NA. | **Data type:** Categorical.  **Continuous covariate categorised:** NA.  **How determine it was categorised:** NA.  **Justification for categorising:** NA.  **Where justification reported:** NA.  **Categories reported:** Yes.  **Where categories reported:** Protocol and review.  **Justification for categories:** Not reported.  **Where justification reported:** NA. | **Analysis type reported in protocol:** Subgroup analysis.  **Analysis type reported in review:** Subgroup analysis..  **Reason for changing mind:** NA.    **Analysis type in protocol according to glossary:** Subgroup analysis.  **How determined the analysis type:** Categories given.  **Analysis type in review according to glossary:** Subgroup analysis.  **How determined the analysis type:** Categories given.  **Analysed:** No.  **Reasons given (in the review) for not doing interaction analysis if planned:** Not reported (presumably because one trial).  **Reasons given for carrying out interaction analysis if they weren’t planned:** NA.  **Scenario in protocol:** ‘If sufficient data exist’.  **Rationale for planned scenario:** Not reported.  **Scenario in review (if reported specifically):** ‘provided that sufficient data are available’.  **Rationale for done scenario:** Not reported.  **Reason for changing mind:** NA. | **Method to detect interactions reported in protocol:** Not reported.  **Method to detect interactions reported in review:** Not reported.  **Reason for changing mind:** NA. |
| Basurto Ona 2013 | **Protocol:** ‘Disease severity (severe versus less severe). Severe acute pancreatitis is defined as patients having any of the following criteria: organ failure, local complications, Ranson’s criteria > 3 or APACHE-II score ≥8.’  **Review methods: ‘**Disease severity (severe versus less severe). Severe acute pancreatitis is defined as patients having any of the following criteria: organ failure, local complications, Ranson’s criteria > 3 or APACHE-II score ≥8.’  **Review results:** Not analysed.  **Type:** Patient.  **Covariate summary:** Disease characteristics. | **Method to choose**  **covariate:** Not reported.  **Where method reported:** NA.  **Rationale for choosing the covariate:** Not reported.  **Where rationale reported:** NA. | **Where reported:** Protocol and review.  **Reason not reported in review:** NA.  **Reason not reported in protocol:** NA.  **Labelled as ‘posthoc’ if not in protocol:** NA. | **Data type:** Continuous.  **Continuous covariate categorised:** Yes.  **How determine it was categorised:** Categories reported.  **Justification for categorising:** Not reported.  **Where justification reported:** NA.  **Categories reported:** Yes.  **Where categories reported:** Protocol and review.  **Justification for categories:** Not reported.  **Where justification reported:** NA. | **Analysis type reported in protocol:** Subgroup analysis.  **Analysis type reported in review:** Subgroup analysis..  **Reason for changing mind:** NA.    **Analysis type in protocol according to glossary:** Subgroup analysis.  **How determined the analysis type:** Categories given.  **Analysis type in review according to glossary:** Subgroup analysis.  **How determined the analysis type:** Categories given.  **Analysed:** No.  **Reasons given (in the review) for not doing interaction analysis if planned:** Not reported (presumably because one trial).  **Reasons given for carrying out interaction analysis if they weren’t planned:** NA.  **Scenario in protocol:** ‘If sufficient data exist’.  **Rationale for planned scenario:** Not reported.  **Scenario in review (if reported specifically):** ‘provided that sufficient data are available’.  **Rationale for done scenario:** Not reported.  **Reason for changing mind:** NA. | **Method to detect interactions reported in protocol:** Not reported.  **Method to detect interactions reported in review:** Not reported.  **Reason for changing mind:** NA. |
| Basurto Ona 2013 | **Protocol:** ‘Opioid administration route (oral versus parenteral)’.  **Review methods: ‘**Opioid administration route (oral versus parenteral)’.  **Review results:** Not analysed.  **Type:** Intervention.  **Covariate summary:** Route of administration. | **Method to choose**  **covariate:** Not reported.  **Where method reported:** NA.  **Rationale for choosing the covariate:** Not reported.  **Where rationale reported:** NA. | **Where reported:** Protocol and review.  **Reason not reported in review:** NA.  **Reason not reported in protocol:** NA.  **Labelled as ‘posthoc’ if not in protocol:** NA. | **Data type:** Categorical.  **Continuous covariate categorised:** NA.  **How determine it was categorised:** NA.  **Justification for categorising:** NA.  **Where justification reported:** NA.  **Categories reported:** Yes.  **Where categories reported:** Protocol and review.  **Justification for categories:** Not reported.  **Where justification reported:** NA. | **Analysis type reported in protocol:** Subgroup analysis.  **Analysis type reported in review:** Subgroup analysis..  **Reason for changing mind:** NA.    **Analysis type in protocol according to glossary:** Subgroup analysis.  **How determined the analysis type:** Categories given.  **Analysis type in review according to glossary:** Subgroup analysis.  **How determined the analysis type:** Categories given.  **Analysed:** No.  **Reasons given (in the review) for not doing interaction analysis if planned:** Not reported (presumably because one trial).  **Reasons given for carrying out interaction analysis if they weren’t planned:** NA.  **Scenario in protocol:** ‘If sufficient data exist’.  **Rationale for planned scenario:** Not reported.  **Scenario in review (if reported specifically):** ‘provided that sufficient data are available’.  **Rationale for done scenario:** Not reported.  **Reason for changing mind:** NA. | **Method to detect interactions reported in protocol:** Not reported.  **Method to detect interactions reported in review:** Not reported.  **Reason for changing mind:** NA. |
| Basurto Ona 2013 | **Protocol:** ‘Opioid class (weak versus strong)’.  **Review methods: ‘**Opioid class (pure agonists, partial agonist, agonist-antagonists, antagonists)’.  **Review results:** Not analysed.  **Type:** Intervention.  **Covariate summary:** Type of intervention or control. | **Method to choose**  **covariate:** Not reported.  **Where method reported:** NA.  **Rationale for choosing the covariate:** Not reported.  **Where rationale reported:** NA. | **Where reported:** Protocol and review.  **Reason not reported in review:** NA.  **Reason not reported in protocol:** NA.  **Labelled as ‘posthoc’ if not in protocol:** NA. | **Data type:** Categorical.  **Continuous covariate categorised:** NA.  **How determine it was categorised:** NA.  **Justification for categorising:** NA.  **Where justification reported:** NA.  **Categories reported:** Yes.  **Where categories reported:** Protocol and review.  **Justification for categories:** Not reported.  **Where justification reported:** NA. | **Analysis type reported in protocol:** Subgroup analysis.  **Analysis type reported in review:** Subgroup analysis..  **Reason for changing mind:** NA.    **Analysis type in protocol according to glossary:** Subgroup analysis.  **How determined the analysis type:** Categories given.  **Analysis type in review according to glossary:** Subgroup analysis.  **How determined the analysis type:** Categories given.  **Analysed:** No.  **Reasons given (in the review) for not doing interaction analysis if planned:** Not reported (presumably because one trial).  **Reasons given for carrying out interaction analysis if they weren’t planned:** NA.  **Scenario in protocol:** ‘If sufficient data exist’.  **Rationale for planned scenario:** Not reported.  **Scenario in review (if reported specifically):** ‘provided that sufficient data are available’.  **Rationale for done scenario:** Not reported.  **Reason for changing mind:** NA. | **Method to detect interactions reported in protocol:** Not reported.  **Method to detect interactions reported in review:** Not reported.  **Reason for changing mind:** NA. |
| Basurto Ona 2013 | **Protocol:** Allocation concealment. We judged the following to be three covariates: ‘We will examine the effect on the primary outcome of excluding any study judged to be at a high risk of bias by three of the domains, sequence generation, allocation concealment and blinding.’  **Review methods:** Allocation concealment. We judged the following to be three covariates: ‘We will examine the effect on the primary outcome of excluding any study judged to be at a high risk of bias by three of the domains, i.e. sequence generation, allocation concealment and blinding.’  **Review results:** Not analysed.  **Type:** Methodological.  **Covariate summary:** Allocation concealment. | **Method to choose**  **covariate:** Not reported.  **Where method reported:** NA.  **Rationale for choosing the covariate:** Not reported.  **Where rationale reported:** NA. | **Where reported:** Protocol and review.  **Reason not reported in review:** NA.  **Reason not reported in protocol:** NA.  **Labelled as ‘posthoc’ if not in protocol:** NA. | **Data type:** Categorical.  **Continuous covariate categorised:** NA.  **How determine it was categorised:** NA.  **Justification for categorising:** NA.  **Where justification reported:** NA.  **Categories reported:** Yes.  **Where categories reported:** Protocol and review.  **Justification for categories:** Not reported.  **Where justification reported:** NA. | **Analysis type reported in protocol:** Sensitivity analysis.  **Analysis type reported in review:** Sensitivity analysis.  **Reason for changing mind:** NA.    **Analysis type in protocol according to glossary:** Sensitivity analysis.  **How determined the analysis type:** ‘excluding’.  **Analysis type in review according to glossary:** Sensitivity analysis.  **How determined the analysis type:** ‘excluding’.  **Analysed:** No.  **Reasons given (in the review) for not doing interaction analysis if planned:** Not reported (presumably because one trial).  **Reasons given for carrying out interaction analysis if they weren’t planned:** NA.  **Scenario in protocol:** ‘Provided there are sufficient trials’’. We will examine the effect on the primary outcome.’  **Rationale for planned scenario:** Not reported.  **Scenario in review (if reported specifically):** ‘We will examine the effect on the primary outcome’.  **Rationale for done scenario:** Not reported.  **Reason for changing mind:** NA. | **Method to detect interactions reported in protocol:** Not reported.  **Method to detect interactions reported in review:** Not reported.  **Reason for changing mind:** NA. |
| Basurto Ona 2013 | **Protocol:** Blinding. We judged the following to be three covariates: ‘We will examine the effect on the primary outcome of excluding any study judged to be at a high risk of bias by three of the domains, sequence generation, allocation concealment and blinding.’  **Review methods:** Blinding. We judged the following to be three covariates: ‘We will examine the effect on the primary outcome of excluding any study judged to be at a high risk of bias by three of the domains, i.e. sequence generation, allocation concealment and blinding.’  **Review results:** Not analysed.  **Type:** Methodological.  **Covariate summary:** Blinding. | **Method to choose**  **covariate:** Not reported.  **Where method reported:** NA.  **Rationale for choosing the covariate:** Not reported.  **Where rationale reported:** NA. | **Where reported:** Protocol and review.  **Reason not reported in review:** NA.  **Reason not reported in protocol:** NA.  **Labelled as ‘posthoc’ if not in protocol:** NA. | **Data type:** Categorical.  **Continuous covariate categorised:** NA.  **How determine it was categorised:** NA.  **Justification for categorising:** NA.  **Where justification reported:** NA.  **Categories reported:** Yes.  **Where categories reported:** Protocol and review.  **Justification for categories:** Not reported.  **Where justification reported:** NA. | **Analysis type reported in protocol:** Sensitivity analysis.  **Analysis type reported in review:** Sensitivity analysis.  **Reason for changing mind:** NA.    **Analysis type in protocol according to glossary:** Sensitivity analysis.  **How determined the analysis type:** ‘excluding’.  **Analysis type in review according to glossary:** Sensitivity analysis.  **How determined the analysis type:** ‘excluding’.  **Analysed:** No.  **Reasons given (in the review) for not doing interaction analysis if planned:** Not reported (presumably because one trial).  **Reasons given for carrying out interaction analysis if they weren’t planned:** NA.  **Scenario in protocol:** ‘Provided there are sufficient trials’’. We will examine the effect on the primary outcome.’  **Rationale for planned scenario:** Not reported.  **Scenario in review (if reported specifically):** ‘We will examine the effect on the primary outcome’.  **Rationale for done scenario:** Not reported.  **Reason for changing mind:** NA. | **Method to detect interactions reported in protocol:** Not reported.  **Method to detect interactions reported in review:** Not reported.  **Reason for changing mind:** NA. |
| Basurto Ona 2013 | **Protocol:** Not reported.  **Review methods:** Not reported.  **Review results: ‘**Buprenorphine versus pethidine’; ‘Morphine versus metamizole’; ‘Pentazocine versus procaine’; ‘Opioids versus no opioids’.  **Type:** Intervention.  **Covariate summary:** Type of intervention or control. | **Method to choose**  **covariate:** Not reported.  **Where method reported:** NA.  **Rationale for choosing the covariate:** Not reported.  **Where rationale reported:** NA. | **Where reported:** Review only.  **Reason not reported in review:** NA.  **Reason not reported in protocol:** Not reported.  **Labelled as ‘posthoc’ if not in protocol:** No. | **Data type:** Categorical.  **Continuous covariate categorised:** NA.  **How determine it was categorised:** NA.  **Justification for categorising:** NA.  **Where justification reported:** NA.  **Categories reported:** Yes.  **Where categories reported:** Review only.  **Justification for categories:** Not reported.  **Where justification reported:** NA. | **Analysis type reported in protocol:** Not reported.  **Analysis type reported in review:** Unnamed analysis.  **Reason for changing mind:** Not reported.    **Analysis type in protocol according to glossary:** Not reported.  **How determined the analysis type:** NA.  **Analysis type in review according to glossary:** stratification/subgroup analysis.  **How determined the analysis type:** Categories given.  **Analysed:** Yes.  **Reasons given (in the review) for not doing interaction analysis if planned:** NA.  **Reasons given for carrying out interaction analysis if they weren’t planned:** Not reported.  **Scenario in protocol:** Not reported.  **Rationale for planned scenario:** NA.  **Scenario in review (if reported specifically):** Not reported.  **Rationale for done scenario:** NA.  **Reason for changing mind:** NA. | **Method to detect interactions reported in protocol:** Not reported.  **Method to detect interactions reported in review:** Not reported.  **Reason for changing mind:** NA. |
| Basurto Ona 2013 | **Protocol:** Sequence generation. We judged the following to be three covariates: ‘We will examine the effect on the primary outcome of excluding any study judged to be at a high risk of bias by three of the domains, sequence generation, allocation concealment and blinding.’  **Review methods:** Sequence generation We judged the following to be three covariates: ‘We will examine the effect on the primary outcome of excluding any study judged to be at a high risk of bias by three of the domains, i.e. sequence generation, allocation concealment and blinding.’  **Review results:** Not analysed.  **Type:** Methodological.  **Covariate summary:** Random generation. | **Method to choose**  **covariate:** Not reported.  **Where method reported:** NA.  **Rationale for choosing the covariate:** Not reported.  **Where rationale reported:** NA. | **Where reported:** Protocol and review.  **Reason not reported in review:** NA.  **Reason not reported in protocol:** NA.  **Labelled as ‘posthoc’ if not in protocol:** NA. | **Data type:** Categorical.  **Continuous covariate categorised:** NA.  **How determine it was categorised:** NA.  **Justification for categorising:** NA.  **Where justification reported:** NA.  **Categories reported:** Yes.  **Where categories reported:** Protocol and review.  **Justification for categories:** Not reported.  **Where justification reported:** NA. | **Analysis type reported in protocol:** Sensitivity analysis.  **Analysis type reported in review:** Sensitivity analysis.  **Reason for changing mind:** NA.    **Analysis type in protocol according to glossary:** Sensitivity analysis.  **How determined the analysis type:** ‘excluding’.  **Analysis type in review according to glossary:** Sensitivity analysis.  **How determined the analysis type:** ‘excluding’.  **Analysed:** No.  **Reasons given (in the review) for not doing interaction analysis if planned:** Not reported (presumably because one trial).  **Reasons given for carrying out interaction analysis if they weren’t planned:** NA.  **Scenario in protocol:** ‘Provided there are sufficient trials’’. We will examine the effect on the primary outcome.’  **Rationale for planned scenario:** Not reported.  **Scenario in review (if reported specifically):** ‘We will examine the effect on the primary outcome’.  **Rationale for done scenario:** Not reported.  **Reason for changing mind:** NA. | **Method to detect interactions reported in protocol:** Not reported.  **Method to detect interactions reported in review:** Not reported.  **Reason for changing mind:** NA. |
| Bellmunt-Montoya 2013 | **Protocol:** ‘Comparing studies with high risk of bias versus low risk of bias’.  **Review methods: ‘**Comparing studies with high risk of bias versus those having low risk of bias’.  **Review results:** Not analysed.  **Type:** Methodological.  **Covariate summary:** Risk of bias. | **Method to choose**  **covariate:** Not reported.  **Where method reported:** NA.  **Rationale for choosing the covariate:** Not reported.  **Where rationale reported:** NA. | **Where reported:** Protocol and review.  **Reason not reported in review:** NA.  **Reason not reported in protocol:** NA.  **Labelled as ‘posthoc’ if not in protocol:** NA. | **Data type:** Categorical.  **Continuous covariate categorised:** NA.  **How determine it was categorised:** NA.  **Justification for categorising:** NA.  **Where justification reported:** NA.  **Categories reported:** Yes.  **Where categories reported:** Protocol and review.  **Justification for categories:** Not reported.  **Where justification reported:** NA. | **Analysis type reported in protocol:** Sensitivity analysis.  **Analysis type reported in review:** Sensitivity analysis.  **Reason for changing mind:** NA.    **Analysis type in protocol according to glossary:** Subgroup analysis.  **How determined the analysis type:** Categories given.  **Analysis type in review according to glossary:** Subgroup analysis.  **How determined the analysis type:** Categories given.  **Analysed:** No.  **Reasons given (in the review) for not doing interaction analysis if planned:** ‘We did not analyze sensitivity based on the level of risk of  bias because all studies had a high risk of bias. None of the studies  masked the interventions.’  **Reasons given for carrying out interaction analysis if they weren’t planned:** NA.  **Scenario in protocol:** ‘We will use sensitivity analysis to assess the strength of the results  and to explain possible heterogeneity between the studies.’  **Rationale for planned scenario:** Not reported.  **Scenario in review (if reported specifically):** ‘Not reported’.  **Rationale for done scenario:** Not reported.  **Reason for changing mind:** NA**.** | **Method to detect interactions reported in protocol:** Not reported.  **Method to detect interactions reported in review:** Not reported.  **Reason for changing mind:** NA. |
| Bellmunt-Montoya 2013 | **Protocol:** ‘Comparing unpublished studies (if there are any) versus published studies’.  **Review methods: ‘**Comparing any unpublished studies versus published studies’.  **Review results:** Not analysed.  **Type:** Methodological.  **Covariate summary:** Publication status. | **Method to choose**  **covariate:** Not reported.  **Where method reported:** NA.  **Rationale for choosing the covariate:** Not reported.  **Where rationale reported:** NA. | **Where reported:** Protocol and review.  **Reason not reported in review:** NA.  **Reason not reported in protocol:** NA.  **Labelled as ‘posthoc’ if not in protocol:** NA. | **Data type:** Categorical.  **Continuous covariate categorised:** NA.  **How determine it was categorised:** NA.  **Justification for categorising:** NA.  **Where justification reported:** NA.  **Categories reported:** Yes.  **Where categories reported:** Protocol and review.  **Justification for categories:** Not reported.  **Where justification reported:** NA. | **Analysis type reported in protocol:** Sensitivity analysis.  **Analysis type reported in review:** Sensitivity analysis.  **Reason for changing mind:** NA.    **Analysis type in protocol according to glossary:** Subgroup analysis.  **How determined the analysis type:** Categories given.  **Analysis type in review according to glossary:** Subgroup analysis.  **How determined the analysis type:** Categories given.  **Analysed:** No.  **Reasons given (in the review) for not doing interaction analysis if planned:** ‘The sensitivity analysis comparing published studies versus nonpublished was not performed because all three studies were published.’  **Reasons given for carrying out interaction analysis if they weren’t planned:** NA.  **Scenario in protocol:** ‘We will use sensitivity analysis to assess the strength of the results  and to explain possible heterogeneity between the studies.’  **Rationale for planned scenario:** Not reported.  **Scenario in review (if reported specifically):** ‘Not reported’.  **Rationale for done scenario:** Not reported.  **Reason for changing mind:** NA**.** | **Method to detect interactions reported in protocol:** Not reported.  **Method to detect interactions reported in review:** Not reported.  **Reason for changing mind:** NA. |
| Bellmunt-Montoya 2013 | **Protocol: ‘**Type of comparison assessed, i. e. CHIVA versus drugs, CHIVA versus leg compression, CHIVA versus any surgery (laser therapy, stripping, radiofrequency, etc. ).’  **Review methods:** ‘Type of comparison assessed: CHIVA versus drugs, compression dressings or other techniques.’  **Review results:** ‘Vein stripping versus the CHIVA method’; ‘compression dressing versus the CHIVA method’.  **Type:** Intervention.  **Covariate summary:** Type of intervention or control. | **Method to choose**  **covariate:** Not reported.  **Where method reported:** NA.  **Rationale for choosing the covariate:** Not reported.  **Where rationale reported:** NA. | **Where reported:** Protocol and review.  **Reason not reported in review:** NA.  **Reason not reported in protocol:** NA.  **Labelled as ‘posthoc’ if not in protocol:** NA. | **Data type:** Categorical.  **Continuous covariate categorised:** NA.  **How determine it was categorised:** NA.  **Justification for categorising:** NA.  **Where justification reported:** NA.  **Categories reported:** Yes.  **Where categories reported:** Protocol and review.  **Justification for categories:** Not reported.  **Where justification reported:** NA. | **Analysis type reported in protocol:** Subgroup analysis.  **Analysis type reported in review:** Subgroup analysis.  **Reason for changing mind:** NA.    **Analysis type in protocol according to glossary:** Subgroup analysis.  **How determined the analysis type:** Categories given.  **Analysis type in review according to glossary:** Subgroup analysis.  **How determined the analysis type:** Categories given.  **Analysed:** Yes.  **Reasons given (in the review) for not doing interaction analysis if planned:** NA.  **Reasons given for carrying out interaction analysis if they weren’t planned:** NA.  **Scenario in protocol:** ‘The authors anticipate the following as sources of clinical heterogeneity  and plan to conduct subgroup analyses comparing:’  **Rationale for planned scenario:** Not reported.  **Scenario in review (if reported specifically):** ‘We considered two sources of clinical heterogeneity to plan subgroup analysis if necessary.’  **Rationale for done scenario:** Not reported.  **Reason for changing mind:** NA**.** | **Method to detect interactions reported in protocol:** Not reported.  **Method to detect interactions reported in review:** Not reported.  **Reason for changing mind:** NA. |
| Bellmunt-Montoya 2013 | **Protocol: ‘**Type of procedure that was used to implement the CHIVA method, i. e. open surgery, endovascular surgery, sclerosis or laser technique.’  **Review methods:** ‘Type of procedure used to implement the CHIVA method: open surgery, sclerotherapy, laser, radiofrequency and any other.’  **Review results:** Not analysed.  **Type:** Intervention.  **Covariate summary:** Type of intervention or control. | **Method to choose**  **covariate:** Not reported.  **Where method reported:** NA.  **Rationale for choosing the covariate:** Not reported.  **Where rationale reported:** NA. | **Where reported:** Protocol and review.  **Reason not reported in review:** NA.  **Reason not reported in protocol:** NA.  **Labelled as ‘posthoc’ if not in protocol:** NA. | **Data type:** Categorical.  **Continuous covariate categorised:** NA.  **How determine it was categorised:** NA.  **Justification for categorising:** NA.  **Where justification reported:** NA.  **Categories reported:** Yes.  **Where categories reported:** Protocol and review.  **Justification for categories:** Not reported.  **Where justification reported:** NA. | **Analysis type reported in protocol:** Subgroup analysis.  **Analysis type reported in review:** Subgroup analysis.  **Reason for changing mind:** NA.    **Analysis type in protocol according to glossary:** Subgroup analysis.  **How determined the analysis type:** Categories given.  **Analysis type in review according to glossary:** Subgroup analysis.  **How determined the analysis type:** Categories given.  **Analysed:** No.  **Reasons given (in the review) for not doing interaction analysis if planned:** ‘All four included studies implemented the CHIVA method by  open surgery. Therefore, we did not need to perform a subgroup analysis according to the procedure used to implement the CHIVA  method.’  **Reasons given for carrying out interaction analysis if they weren’t planned:** NA.  **Scenario in protocol:** ‘The authors anticipate the following as sources of clinical heterogeneity  and plan to conduct subgroup analyses comparing:’  **Rationale for planned scenario:** Not reported.  **Scenario in review (if reported specifically):** ‘We considered two sources of clinical heterogeneity to plan subgroup analysis if necessary.’  **Rationale for done scenario:** Not reported.  **Reason for changing mind:** NA**.** | **Method to detect interactions reported in protocol:** Not reported.  **Method to detect interactions reported in review:** Not reported.  **Reason for changing mind:** NA. |
| Berlowitz 2013 | **Protocol:** ‘Acute (< 1 year) versus chronic cervical SCI participants’.  **Review methods: ‘**Acute (less than one year) versus chronic cervical SCI participants’.  **Review results:** Not analysed.  **Type:** Patient.  **Covariate summary:** Disease characteristics. | **Method to choose**  **covariate:** Not reported.  **Where method reported:** NA.  **Rationale for choosing the covariate:** Not reported.  **Where rationale reported:** NA. | **Where reported:** Protocol and review.  **Reason not reported in review:** NA.  **Reason not reported in protocol:** NA.  **Labelled as ‘posthoc’ if not in protocol:** NA. | **Data type:** Categorical.  **Continuous covariate categorised:** NA.  **How determine it was categorised:** NA.  **Justification for categorising:** NA.  **Where justification reported:** NA.  **Categories reported:** Yes.  **Where categories reported:** Protocol and review.  **Justification for categories:** Not reported.  **Where justification reported:** NA. | **Analysis type reported in protocol:** Subgroup analysis.  **Analysis type reported in review:** Subgroup analysis.  **Reason for changing mind:** NA.    **Analysis type in protocol according to glossary:** Subgroup analysis.  **How determined the analysis type:** Categories given.  **Analysis type in review according to glossary:** Subgroup analysis.  **How determined the analysis type:** Categories given.  **Analysed:** No.  **Reasons given (in the review) for not doing interaction analysis if planned:** Not reported. Presume because only one trial. In results: ‘We conducted subgroup analyses only on outcomes that had data from more than three trials’.  **Reasons given for carrying out interaction analysis if they weren’t planned:** NA.  **Scenario in protocol:** Not reported.  **Rationale for planned scenario:** NA.  **Scenario in review (if reported specifically):** Not reported.  **Rationale for done scenario:** NA.  **Reason for changing mind:** NA. | **Method to detect interactions reported in protocol:** Not reported.  **Method to detect interactions reported in review:** Not reported.  **Reason for changing mind:** NA. |
| Berlowitz 2013 | **Protocol:** Analyses performed on an intention-to-treat basis. We judged the following to be three covariates: ‘We will perform sensitivity analyses based on the methodological quality of the included studies. The analyses will examine the effect of excluding studies that either failed to report or did not include evidence of concealed allocation, blinding of outcome assessments and analyses performed on an intention-to-treat basis.’  **Review methods:** Analyses performed on an intention-to-treat basis. We judged the following to be three covariates: ‘We performed sensitivity analyses based on the methodological quality of the included studies. The analyses examined the effect of excluding studies that either failed to report or did not include evidence of concealed allocation, blinding of outcome assessments and analyses performed on an ITT basis.’  **Review results:** Not analysed.  **Type:** Methodological.  **Covariate summary:** Intention-to-treat. | **Method to choose**  **covariate:** Not reported.  **Where method reported:** NA.  **Rationale for choosing the covariate:** Not reported.  **Where rationale reported:** NA. | **Where reported:** Protocol and review.  **Reason not reported in review:** NA.  **Reason not reported in protocol:** NA.  **Labelled as ‘posthoc’ if not in protocol:** NA. | **Data type:** Categorical.  **Continuous covariate categorised:** NA.  **How determine it was categorised:** NA.  **Justification for categorising:** NA.  **Where justification reported:** NA.  **Categories reported:** Not reported.  **Where categories reported:** NA.  **Justification for categories:** Not reported.  **Where justification reported:** NA. | **Analysis type reported in protocol:** Sensitivity analysis.  **Analysis type reported in review:** Sensitivity analysis  **Reason for changing mind:** NA.    **Analysis type in protocol according to glossary:** Sensitivity analysis  **How determined the analysis type:** Says ‘sensitivity analyses’. No reason to dispute.  **Analysis type in review according to glossary:** Sensitivity analysis  **How determined the analysis type:** Says ‘sensitivity analyses’. No reason to dispute.  **Analysed:** No.  **Reasons given (in the review) for not doing interaction analysis if planned:** Not reported (presumably because one trial).  **Reasons given for carrying out interaction analysis if they weren’t planned:** NA.  **Scenario in protocol:** Not reported.  **Rationale for planned scenario:** NA.  **Scenario in review (if reported specifically):** Not reported.  **Rationale for done scenario:** NA.  **Reason for changing mind:** NA. | **Method to detect interactions reported in protocol:** Not reported.  **Method to detect interactions reported in review:** Not reported.  **Reason for changing mind:** NA. |
| Berlowitz 2013 | **Protocol:** Blinding of outcome assessments. We judged the following to be three covariates: ‘We will perform sensitivity analyses based on the methodological quality of the included studies. The analyses will examine the effect of excluding studies that either failed to report or did not include evidence of concealed allocation, blinding of outcome assessments and analyses performed on an intention-to-treat basis.’  **Review methods:** Blinding of outcome assessments. We judged the following to be three covariates: ‘We performed sensitivity analyses based on the methodological quality of the included studies. The analyses examined the effect of excluding studies that either failed to report or did not include evidence of concealed allocation, blinding of outcome assessments and analyses performed on an ITT basis.’  **Review results:** Not analysed.  **Type:** Methodological.  **Covariate summary:** Blinding. | **Method to choose**  **covariate:** Not reported.  **Where method reported:** NA.  **Rationale for choosing the covariate:** Not reported.  **Where rationale reported:** NA. | **Where reported:** Protocol and review.  **Reason not reported in review:** NA.  **Reason not reported in protocol:** NA.  **Labelled as ‘posthoc’ if not in protocol:** NA. | **Data type:** Categorical.  **Continuous covariate categorised:** NA.  **How determine it was categorised:** NA.  **Justification for categorising:** NA.  **Where justification reported:** NA.  **Categories reported:** Not reported.  **Where categories reported:** NA.  **Justification for categories:** Not reported.  **Where justification reported:** NA. | **Analysis type reported in protocol:** Sensitivity analysis.  **Analysis type reported in review:** Sensitivity analysis  **Reason for changing mind:** NA.    **Analysis type in protocol according to glossary:** Sensitivity analysis  **How determined the analysis type:** Says ‘sensitivity analyses’. No reason to dispute.  **Analysis type in review according to glossary:** Sensitivity analysis  **How determined the analysis type:** Says ‘sensitivity analyses’. No reason to dispute.  **Analysed:** No.  **Reasons given (in the review) for not doing interaction analysis if planned:** Not reported (presumably because one trial).  **Reasons given for carrying out interaction analysis if they weren’t planned:** NA.  **Scenario in protocol:** Not reported.  **Rationale for planned scenario:** NA.  **Scenario in review (if reported specifically):** Not reported.  **Rationale for done scenario:** NA.  **Reason for changing mind:** NA. | **Method to detect interactions reported in protocol:** Not reported.  **Method to detect interactions reported in review:** Not reported.  **Reason for changing mind:** NA. |
| Berlowitz 2013 | **Protocol:** Concealed allocation. We judged the following to be three covariates: ‘We will perform sensitivity analyses based on the methodological quality of the included studies. The analyses will examine the effect of excluding studies that either failed to report or did not include evidence of concealed allocation, blinding of outcome assessments and analyses performed on an intention-to-treat basis.’  **Review methods:** Concealed allocation. We judged the following to be three covariates: ‘We performed sensitivity analyses based on the methodological quality of the included studies. The analyses examined the effect of excluding studies that either failed to report or did not include evidence of concealed allocation, blinding of outcome assessments and analyses performed on an ITT basis.’  **Review results:** Not analysed.  **Type:** Methodological.  **Covariate summary:** Allocation concealment. | **Method to choose**  **covariate:** Not reported.  **Where method reported:** NA.  **Rationale for choosing the covariate:** Not reported.  **Where rationale reported:** NA. | **Where reported:** Protocol and review.  **Reason not reported in review:** NA.  **Reason not reported in protocol:** NA.  **Labelled as ‘posthoc’ if not in protocol:** NA. | **Data type:** Categorical.  **Continuous covariate categorised:** NA.  **How determine it was categorised:** NA.  **Justification for categorising:** NA.  **Where justification reported:** NA.  **Categories reported:** Not reported.  **Where categories reported:** NA.  **Justification for categories:** Not reported.  **Where justification reported:** NA. | **Analysis type reported in protocol:** Sensitivity analysis.  **Analysis type reported in review:** Sensitivity analysis  **Reason for changing mind:** NA.    **Analysis type in protocol according to glossary:** Sensitivity analysis  **How determined the analysis type:** Says ‘sensitivity analyses’. No reason to dispute.  **Analysis type in review according to glossary:** Sensitivity analysis  **How determined the analysis type:** Says ‘sensitivity analyses’. No reason to dispute.  **Analysed:** No.  **Reasons given (in the review) for not doing interaction analysis if planned:** Not reported. Presume because only one trial.  **Reasons given for carrying out interaction analysis if they weren’t planned:** NA.  **Scenario in protocol:** Not reported.  **Rationale for planned scenario:** NA.  **Scenario in review (if reported specifically):** Not reported.  **Rationale for done scenario:** NA.  **Reason for changing mind:** NA. | **Method to detect interactions reported in protocol:** Not reported.  **Method to detect interactions reported in review:** Not reported.  **Reason for changing mind:** NA. |
| Berlowitz 2013 | **Protocol:** Treatment duration. We judged the following to be two covariates: ‘Dose-response analyses will be conducted where possible to determine any relationship between treatment intensity or duration and an intervention effect’.  **Review methods:** Treatment duration. We judged the following to be two covariates: ‘We conducted dose-response analyses where possible to determine any relationship between treatment intensity or duration and an intervention effect.’  **Review results:** Not analysed.  **Type:** Intervention.  **Covariate summary:** Duration of intervention. | **Method to choose**  **covariate:** Not reported.  **Where method reported:** NA.  **Rationale for choosing the covariate:** Not reported.  **Where rationale reported:** NA. | **Where reported:** Protocol and review.  **Reason not reported in review:** NA.  **Reason not reported in protocol:** NA.  **Labelled as ‘posthoc’ if not in protocol:** NA. | **Data type:** Continuous.  **Continuous covariate categorised:** Yes.  **How determine it was categorised:** Subgroup analysis planned.  **Justification for categorising:** Not reported.  **Where justification reported:** NA.  **Categories reported:** Not reported.  **Where categories reported:** NA.  **Justification for categories:** Not reported.  **Where justification reported:** NA. | **Analysis type reported in protocol:** Subgroup analysis.  **Analysis type reported in review:** Subgroup analysis.  **Reason for changing mind:** NA.    **Analysis type in protocol according to glossary:** Subgroup analysis.  **How determined the analysis type:** Under heading of ‘subgroup analyses’. No reason to dispute.  **Analysis type in review according to glossary:** Subgroup analysis.  **How determined the analysis type:** Under heading of ‘subgroup analyses’. No reason to dispute.  **Analysed:** No.  **Reasons given (in the review) for not doing interaction analysis if planned:** Not reported. Presume because only one trial. In results: ‘We conducted subgroup analyses only on outcomes that had data from more than three trials’.  **Reasons given for carrying out interaction analysis if they weren’t planned:** NA.  **Scenario in protocol:** Not reported.  **Rationale for planned scenario:** NA.  **Scenario in review (if reported specifically):** Not reported.  **Rationale for done scenario:** NA.  **Reason for changing mind:** NA. | **Method to detect interactions reported in protocol:** Not reported.  **Method to detect interactions reported in review:** Not reported.  **Reason for changing mind:** NA. |
| Berlowitz 2013 | **Protocol:** Treatment intensity. We judged the following to be two covariates: ‘Dose-response analyses will be conducted where possible to determine any relationship between treatment intensity or duration and an intervention effect.’  **Review methods:** Treatment intensity. We judged the following to be two covariates: ‘We conducted dose-response analyses where possible to determine any relationship between treatment intensity or duration and an intervention effect.’  **Review results:** Not analysed.  **Type:** Intervention.  **Covariate summary:** Intervention intensity. | **Method to choose**  **covariate:** Not reported.  **Where method reported:** NA.  **Rationale for choosing the covariate:** Not reported.  **Where rationale reported:** NA. | **Where reported:** Protocol and review.  **Reason not reported in review:** NA.  **Reason not reported in protocol:** NA.  **Labelled as ‘posthoc’ if not in protocol:** NA. | **Data type:** Unclear.  **Continuous covariate categorised:** Unclear.  **How determine it was categorised:** NA.  **Justification for categorising:** Not reported.  **Where justification reported:** NA.  **Categories reported:** Not reported.  **Where categories reported:** NA.  **Justification for categories:** Not reported.  **Where justification reported:** NA. | **Analysis type reported in protocol:** Subgroup analysis.  **Analysis type reported in review:** Subgroup analysis.  **Reason for changing mind:** NA.    **Analysis type in protocol according to glossary:** Subgroup analysis.  **How determined the analysis type:** Under heading of ‘subgroup analyses’. No reason to dispute.  **Analysis type in review according to glossary:** Subgroup analysis.  **How determined the analysis type:** Under heading of ‘subgroup analyses’. No reason to dispute.  **Analysed:** No.  **Reasons given (in the review) for not doing interaction analysis if planned:** Not reported. Presume because only one trial. In results: ‘We conducted subgroup analyses only on outcomes that had data from more than three trials’.  **Reasons given for carrying out interaction analysis if they weren’t planned:** NA.  **Scenario in protocol:** Not reported.  **Rationale for planned scenario:** NA.  **Scenario in review (if reported specifically):** Not reported.  **Rationale for done scenario:** NA.  **Reason for changing mind:** NA. | **Method to detect interactions reported in protocol:** Not reported.  **Method to detect interactions reported in review:** Not reported.  **Reason for changing mind:** NA. |
| Boselie 2012 | **Protocol: ‘**Including studies with a high risk of bias.’ **‘**We will perform a sensitivity analysis to assess the influence of the number of risk of bias criteria that are met, including one that looks at the results when studies with a high risk of bias are excluded.’  **Review methods: ‘**Including studies with a high risk of bias.’  **Review results: ‘**Only including studies with an overall low risk of bias.’  **Type:** Methodological.  **Covariate summary:** Risk of bias. | **Method to choose**  **covariate:** Not reported.  **Where method reported:** NA.  **Rationale for choosing the covariate:** Not reported.  **Where rationale reported:** NA. | **Where reported:** Protocol and review.  **Reason not reported in review:** NA.  **Reason not reported in protocol:** NA.  **Labelled as ‘posthoc’ if not in protocol:** NA**.** | **Data type:** Categorical.  **Continuous covariate categorised:** NA.  **How determine it was categorised:** NA.  **Justification for categorising:** NA.  **Where justification reported:** NA.  **Categories reported:** Yes.  **Where categories reported:** Protocol and review.  **Justification for categories:** Not reported.  **Where justification reported:** NA. | **Analysis type reported in protocol:** Sensitivity analysis.  **Analysis type reported in review:** Sensitivity analysis.  **Reason for changing mind:** NA.    **Analysis type in protocol according to glossary:** Sensitivity analysis.  **How determined the analysis type:** Says ‘Sensitivity analysis’. No reason to dispute.  **Analysis type in review according to glossary:** Sensitivity analysis.  **How determined the analysis type:** Says ‘Sensitivity analysis’. No reason to dispute.  **Analysed:** Yes.  **Reasons given (in the review) for not doing interaction analysis if planned:** NA.  **Reasons given for carrying out interaction analysis if they weren’t planned:** NA.  **Scenario in protocol:** Not reported.  **Rationale for planned scenario:** NA.  **Scenario in review (if reported specifically):** Not reported.  **Rationale for done scenario:** Not reported.  **Reason for changing mind:** NA. | **Method to detect interactions reported in protocol:** Not reported.  **Method to detect interactions reported in review:** Not reported.  **Reason for changing mind:** NA. |
| Boselie 2012 | **Protocol:** ‘Presence of myelopathy.’  **Review methods:** Presence of myelopathy’  **Review results:** Not analysed.  **Type:** Patient.  **Covariate summary:** Disease characteristics. | **Method to choose**  **covariate:** Not reported.  **Where method reported:** NA.  **Rationale for choosing the covariate: ‘**In theory, we can expect different results from a patient suffering  solely from myelopathy and a patient suffering solely from  radiculopathy (e.g. VAS score for arm pain will usually decrease  postoperatively in the radiculopathy group, in contrast with a VAS  score for arm pain that will already be low in most patients in the  myelopathy group). It has also been postulated that arthroplasty  may maintain microtrauma to the spinal cord, although a study  comparing arthroplasty and fusion in myelopathy due to degenerative  disc disease showed similar results for both treatment modalities  (Riew 2008). This could potentially influence results, especially  when there are large differences in the percentage of patients  with myelopathy between study groups. Therefore we plan to do  a subgroup analysis to identify if the presence of myelopathy is a  confounding variable.’ In protocol.  ‘In theory, different results could be expected from a patient suffering  solely from myelopathy and a patient suffering solely from  radiculopathy (e.g. score for arm pain will usually decrease postoperatively  in the radiculopathy group, in contrast with a score  for arm pain that could already be low in most patients in the  myelopathy group). Another possible confounding factor is the  possibility of neurological deterioration in the presence of adequate  decompression that has been described in about 15% of patients  with a myelopathy. It has also been postulated that arthroplasty  may maintain microtrauma to the spinal cord, although a  study pooling two study populations comparing arthroplasty and  fusion inmyelopathy due to degenerative disc disease showed similar  results for both treatment modalities (Riew 2008). This could  potentially influence results, especially when there are large differences  in the percentage of patients with myelopathy between  study groups. Therefore, we planned to do a subgroup analysis to  identify if the presence of myelopathy was a confounding variable.  This could not be performed since none of the studies reported  results for the two groups separately.’ In review.  **Where rationale reported:** Protocol and review. | **Where reported:** Protocol and review.  **Reason not reported in review:** NA.  **Reason not reported in protocol:** NA.  **Labelled as ‘posthoc’ if not in protocol:** NA**.** | **Data type:** Categorical.  **Continuous covariate categorised:** NA.  **How determine it was categorised:** NA.  **Justification for categorising:** NA.  **Where justification reported:** NA.  **Categories reported:** No**.**  **Where categories reported:** NA.  **Justification for categories:** Not reported.  **Where justification reported:** NA. | **Analysis type reported in protocol:** Subgroup analysis.  **Analysis type reported in review:** Subgroup analysis.  **Reason for changing mind:** NA.    **Analysis type in protocol according to glossary:** Subgroup analysis.  **How determined the analysis type:** Says ‘Subgroup analysis’. No reason to dispute.  **Analysis type in review according to glossary:** Subgroup analysis.  **How determined the analysis type:** Says ‘Subgroup analysis’. No reason to dispute.  **Analysed:** No.  **Reasons given (in the review) for not doing interaction analysis if planned: ‘**This could not be performed since none of the studies reported  results for the two groups separately.’  **Reasons given for carrying out interaction analysis if they weren’t planned:** NA.  **Scenario in protocol:** Not reported.  **Rationale for planned scenario:** NA.  **Scenario in review (if reported specifically):** Not reported.  **Rationale for done scenario:** Not reported.  **Reason for changing mind:** NA. | **Method to detect interactions reported in protocol:** Not reported.  **Method to detect interactions reported in review:** Not reported.  **Reason for changing mind:** NA. |
| Boselie 2012 | **Protocol:** ‘Smoking’.  **Review methods: ‘**Smoking’.  **Review results:** Not analysed.  **Type:** Patient.  **Covariate summary:** Demographics. | **Method to choose**  **covariate:** Not reported.  **Where method reported:** NA.  **Rationale for choosing the covariate: ‘**A negative influence of smoking on fusion rates and patient reported  outcomes in spinal fusion surgery has been reported for  both lumbar and cervical spine. (Andersen 2001; Arnold 2010;  Glassman 2000) This negative influence has not been reported in  lumbar disc arthroplasty (Bertagnoli 2006). Therefore, we will do  a subgroup analysis to identify if smoking is a possible confounding  variable.’  ‘**Where rationale reported:** Protocol and review. | **Where reported:** Protocol and review.  **Reason not reported in review:** NA.  **Reason not reported in protocol:** NA.  **Labelled as ‘posthoc’ if not in protocol:** NA**.** | **Data type:** Categorical.  **Continuous covariate categorised:** NA.  **How determine it was categorised:** NA.  **Justification for categorising:** NA.  **Where justification reported:** NA.  **Categories reported:** No.  **Where categories reported:** NA.  **Justification for categories:** Not reported.  **Where justification reported:** NA. | **Analysis type reported in protocol:** Subgroup analysis.  **Analysis type reported in review:** Subgroup analysis.  **Reason for changing mind:** NA.    **Analysis type in protocol according to glossary:** Subgroup analysis.  **How determined the analysis type:** Says ‘Subgroup analysis’. No reason to dispute.  **Analysis type in review according to glossary:** Subgroup analysis.  **How determined the analysis type:** Says ‘Subgroup analysis’. No reason to dispute.  **Analysed:** No.  **Reasons given (in the review) for not doing interaction analysis if planned: ‘**In all of the studies smoking status was not  an exclusion criterion, and in none of the included studies were  results for smokers and non-smokers reported separately.This subgroup  analysis could therefore not be performed.’  **Reasons given for carrying out interaction analysis if they weren’t planned:** NA.  **Scenario in protocol:** Not reported.  **Rationale for planned scenario:** NA.  **Scenario in review (if reported specifically):** Not reported.  **Rationale for done scenario:** Not reported.  **Reason for changing mind:** NA. | **Method to detect interactions reported in protocol:** Not reported.  **Method to detect interactions reported in review:** Not reported.  **Reason for changing mind:** NA. |
| Boselie 2012 | **Protocol: ‘**We will collect all outcome measures at short-term follow-up (up to and including three months) and long-term follow-up (12 months or longer).’ ‘We will attribute different moments of follow-up to different time frames; either “short term” (up to and including three months after surgery), or “long term” (12 months after surgery or longer). If there is more than one follow-up moment within these time frames, the data for the one nearest to the cut-off point (i. e. three months or 12 months) will be extracted.’  **Review methods:** ‘We collected all outcome measures at short-term follow-up (up to and including three months) and medium-term follow-up (one to two years). At the moment of writing the protocol the authors were not aware of reports of studies with a follow-up longer than two years. Some abstracts and papers reporting results on follow-up longer than two years were published since then. To avoid introduction of heterogeneity because of large differences in the duration of follow-up we decided to extract one-year results when available, if these were not available we extracted two-year results. Owing to the very limited amount of studies reporting results after more than two years’ follow-up, these were not included as an additional time point in this review. Results on long-term follow-up (preferably five years or longer) will be included in a future update of this review.’ ‘We therefore attributed different periods of follow-up to different time frames; either ’three months’ (up to and including three months after surgery), or ’one to two years’ (between one and two years after surgery). If there was more than one follow-up period within these time frames, the data for the one nearest to the cutoff point (i. e. three months or one year) was extracted.’  **Review results:** ‘Baseline’; ‘3 months’; ‘1-2 years’.  **Type:** Outcome.  **Covariate summary:** Time point. | **Method to choose**  **covariate:** Not reported.  **Where method reported:** NA.  **Rationale for choosing the covariate:** Not reported.  **Where rationale reported:** NA. | **Where reported:** Protocol and review.  **Reason not reported in review:** NA.  **Reason not reported in protocol:** NA.  **Labelled as ‘posthoc’ if not in protocol:** NA**.** | **Data type:** Continuous.  **Continuous covariate categorised:** Yes.  **How determine it was categorised:** Categories given.  **Justification for categorising:** Not reported.  **Where justification reported:** NA.  **Categories reported:** Yes.  **Where categories reported:** Protocol and review.  **Justification for categories:** Not reported.  **Where justification reported:** NA. | **Analysis type reported in protocol:** Unnamed analysis.  **Analysis type reported in review:** Unnamed analysis.  **Reason for changing mind:** NA.    **Analysis type in protocol according to glossary:** Subgroup analysis.  **How determined the analysis type:** Categories given.  **Analysis type in review according to glossary:** Subgroup analysis.  **How determined the analysis type:** Categories given.  **Analysed:** Yes.  **Reasons given (in the review) for not doing interaction analysis if planned:** NA.  **Reasons given for carrying out interaction analysis if they weren’t planned:** NA.  **Scenario in protocol:** Not reported.  **Rationale for planned scenario:** NA.  **Scenario in review (if reported specifically):** Not reported.  **Rationale for done scenario:** Not reported.  **Reason for changing mind:** NA. | **Method to detect interactions reported in protocol:** Not reported.  **Method to detect interactions reported in review:** Not reported.  **Reason for changing mind:** NA. |
| Boselie 2012 | **Protocol: ‘**We will explore the impact of including studies with high levels of missing data (15%or greater of the participants lost to follow-up) in the overall assessment of treatment effect by using a sensitivity analysis.’  **Review methods: ‘**We explored the impact of including studies with high levels of missing data (15% or greater of the participants lost to follow-up) in the overall assessment of treatment effect by using a sensitivity analysis.’  **Review results: ‘**Excluding studies with >15% loss to follow-up’.  **Type:** Methodological.  **Covariate summary:** Incomplete outcome data/follow up. | **Method to choose**  **covariate:** Not reported.  **Where method reported:** NA.  **Rationale for choosing the covariate:** Not reported.  **Where rationale reported:** NA. | **Where reported:** Protocol and review.  **Reason not reported in review:** NA.  **Reason not reported in protocol:** NA.  **Labelled as ‘posthoc’ if not in protocol:** NA**.** | **Data type:** Continuous.  **Continuous covariate categorised:** Yes.  **How determine it was categorised:** Categories given.  **Justification for categorising:** Not reported.  **Where justification reported:** NA.  **Categories reported:** Yes.  **Where categories reported:** Protocol and review.  **Justification for categories:** Not reported.  **Where justification reported:** NA. | **Analysis type reported in protocol:** Sensitivity analysis.  **Analysis type reported in review:** Sensitivity analysis.  **Reason for changing mind:** NA.    **Analysis type in protocol according to glossary:** Sensitivity analysis.  **How determined the analysis type:** Says ‘Sensitivity analysis’. No reason to dispute.  **Analysis type in review according to glossary:** Sensitivity analysis.  **How determined the analysis type:** Says ‘Sensitivity analysis’. No reason to dispute.  **Analysed:** Yes.  **Reasons given (in the review) for not doing interaction analysis if planned:** NA.  **Reasons given for carrying out interaction analysis if they weren’t planned:** NA.  **Scenario in protocol:** Not reported.  **Rationale for planned scenario:** NA.  **Scenario in review (if reported specifically):** Not reported.  **Rationale for done scenario:** Not reported.  **Reason for changing mind:** NA. | **Method to detect interactions reported in protocol:** Not reported.  **Method to detect interactions reported in review:** Not reported.  **Reason for changing mind:** NA. |
| Boselie 2012 | **Protocol:** Not reported.  **Review methods:** ‘Investigated the possibility of a small study effect.’  **Review results:** ‘Small study effect’.  **Type:** Methodological.  **Covariate summary:** Trial size. | **Method to choose**  **covariate:** Not reported.  **Where method reported:** NA.  **Rationale for choosing the covariate:** Not reported.  **Where rationale reported:** NA. | **Where reported:** Review only.  **Reason not reported in review:** NA.  **Reason not reported in protocol:** Not reported.  **Labelled as ‘posthoc’ if not in protocol:** Yes**.** | **Data type:** Continuous.  **Continuous covariate categorised:** Yes.  **How determine it was categorised:** Categories given.  **Justification for categorising:** Not reported.  **Where justification reported:** NA.  **Categories reported:** Yes.  **Where categories reported:** Review only.  **Justification for categories:** ‘Small studies are more likely to remain unpublished, combined with the fact that it may be easier to publish a small study with strong (positive) results this may lead to an overestimation  of the treatment effect.’  **Where justification reported:** Review only. | **Analysis type reported in protocol:** Not reported.  **Analysis type reported in review:** Sensitivity analysis.  **Reason for changing mind:** Not reported.    **Analysis type in protocol according to glossary:** Not reported.  **How determined the analysis type:** NA.  **Analysis type in review according to glossary:** Sensitivity analysis.  **How determined the analysis type:** Categories given.  **Analysed:** Yes.  **Reasons given (in the review) for not doing interaction analysis if planned:** NA.  **Reasons given for carrying out interaction analysis if they weren’t planned:** Not reported.  **Scenario in protocol:** Not reported.  **Rationale for planned scenario:** NA.  **Scenario in review (if reported specifically):** Not reported.  **Rationale for done scenario:** NA.  **Reason for changing mind:** Not reported. | **Method to detect interactions reported in protocol:** Not reported.  **Method to detect interactions reported in review:** Not reported.  **Reason for changing mind:** NA. |
| Boselie 2012 | **Protocol:** Not reported.  **Review methods: ‘**Subgroup analyses for each type of disc prosthesis were not performed, the number of studies for each prosthesis was very low (in almost all cases only one study), which renders a comparison pointless. (reported in description of included studies)’.  **Review results:** Not analysed.  **Type:** Intervention.  **Covariate summary:** Type of intervention or control. | **Method to choose**  **covariate:** Not reported.  **Where method reported:** NA.  **Rationale for choosing the covariate:** Not reported.  **Where rationale reported:** NA. | **Where reported:** Review only.  **Reason not reported in review:** NA.  **Reason not reported in protocol:** Not reported.  **Labelled as ‘posthoc’ if not in protocol:** No**.** | **Data type:** Categorical.  **Continuous covariate categorised:** NA.  **How determine it was categorised:** NA.  **Justification for categorising:** NA.  **Where justification reported:** NA.  **Categories reported:** Not reported.  **Where categories reported:** NA.  **Justification for categories:** Not reported.  **Where justification reported:** NA. | **Analysis type reported in protocol:** Not reported.  **Analysis type reported in review:** Subgroup analysis.  **Reason for changing mind:** Not reported.    **Analysis type in protocol according to glossary:** Not reported.  **How determined the analysis type:** NA.  **Analysis type in review according to glossary:** Subgroup analysis.  **How determined the analysis type:** Says ‘subgroup analysis’. No reason to dispute.  **Analysed:** No.  **Reasons given (in the review) for not doing interaction analysis if planned:** ‘Subgroup analyses for each type of disc prosthesis were not performed,  the number of studies for each prosthesis was very low (in almost all cases only one study), which renders a comparison  pointless.’  **Reasons given for carrying out interaction analysis if they weren’t planned:** NA.  **Scenario in protocol:** Not reported.  **Rationale for planned scenario:** NA.  **Scenario in review (if reported specifically):** Not reported.  **Rationale for done scenario:** NA.  **Reason for changing mind:** Not reported. | **Method to detect interactions reported in protocol:** Not reported.  **Method to detect interactions reported in review:** Not reported.  **Reason for changing mind:** NA. |
| Bruins Slot 2013 | **Protocol:** ‘ Blinded versus open-label studies’  **Review methods:** ‘Excluding fully open-label trials’.  **Review results:** Not analysed.  **Type:** Methodological.  **Covariate summary:** Blinding. | **Method to choose**  **covariate:** Not reported.  **Where method reported:** NA.  **Rationale for choosing the covariate:** Not reported.  **Where rationale reported:** NA. | **Where reported:** Protocol and review.  **Reason not reported in review:** NA.  **Reason not reported in protocol:** NA.  **Labelled as ‘posthoc’ if not in protocol:** NA. | **Data type:** Categorical.  **Continuous covariate categorised:** NA.  **How determine it was categorised:** NA.  **Justification for categorising:** NA.  **Where justification reported:** NA.  **Categories reported:** Yes.  **Where categories reported:** Protocol and review.  **Justification for categories:** Not reported.  **Where justification reported:** NA. | **Analysis type reported in protocol:** Subgroup analysis.  **Analysis type reported in review: S**ubgroup analysis.  **Reason for changing mind:** NA.    **Analysis type in protocol according to glossary:** Subgroup analysis.  **How determined the analysis type:** Categories given.  **Analysis type in review according to glossary:** Sensitivity analysis.  **How determined the analysis type:** ‘Excluding’  **Analysed:** No.  **Reasons given (in the review) for not doing interaction analysis if planned:** Not reported.  **Reasons given for carrying out interaction analysis if they weren’t planned:** NA.  **Scenario in protocol: ‘**If we find evidence of heterogeneity that cannot be explained by  study quality we intend to conduct the following sensitivity analysis:’  **Rationale for planned scenario:** Not reported.  **Scenario in review (if reported specifically): ‘**In the case of any evidence of heterogeneity that could not be  explained by study quality, we intended to conduct a sensitivity  analysis’  **Rationale for done scenario:** Not reported.  **Reason for changing mind:** NA. | **Method to detect interactions reported in protocol:** Not reported.  **Method to detect interactions reported in review:** Not reported.  **Reason for changing mind:** NA. |
| Bruins Slot 2013 | **Protocol:** ‘Baseline stroke risk factors (assessed by the CHADS2 score)’.  **Review methods: ‘**Baseline stroke risk factors (assessed by the CHADS2 score)’.  **Review results:** ‘Baseline CHADS2 score.’ ‘CHADS2-score 0-1’; ‘CHADS2-score 2’; ‘CHADS2-score ≥ 3’).  **Type:** Patient.  **Covariate summary:** Disease characteristics. | **Method to choose**  **covariate:** Not reported.  **Where method reported:** NA.  **Rationale for choosing the covariate:** Not reported.  **Where rationale reported:** NA. | **Where reported:** Protocol and review.  **Reason not reported in review:** NA.  **Reason not reported in protocol:** NA.  **Labelled as ‘posthoc’ if not in protocol:** NA. | **Data type:** Continuous.  **Continuous covariate categorised:** Yes.  **How determine it was categorised:** categories given.  **Justification for categorising:** Not reported.  **Where justification reported:** NA.  **Categories reported:** Yes.  **Where categories reported:** Review only.  **Justification for categories:** Not reported.  **Where justification reported:** NA. | **Analysis type reported in protocol:** Subgroup analysis.  **Analysis type reported in review: S**ubgroup analysis.  **Reason for changing mind:** NA.    **Analysis type in protocol according to glossary: S**ubgroup analysis.  **How determined the analysis type:** Says ‘**S**ubgroup analysis.’ No reason to dispute.  **Analysis type in review according to glossary: S**ubgroup analysis.  **How determined the analysis type:** ‘**S**ubgroup analysis.’ No reason to dispute.  **Analysed: yes**  **Reasons given (in the review) for not doing interaction analysis if planned:** NA.  **Reasons given for carrying out interaction analysis if they weren’t planned:** NA.  **Scenario in protocol:** ‘Where possible,’  **Rationale for planned scenario:** Not reported.  **Scenario in review (if reported specifically):** ‘Where possible,’  **Rationale for done scenario:** Not reported.  **Reason for changing mind:** Not reported. | **Method to detect interactions reported in protocol:** Not reported.  **Method to detect interactions reported in review:** Not reported.  **Reason for changing mind:** NA. |
| Bruins Slot 2013 | **Protocol:** ‘Patients aged less than 75 years versus patients aged 75 years or over’.  **Review methods:** ‘Age less than 75 years versus age 75 years or over’.  **Review results:** ‘Age’. ‘Age < 75 years’; ‘Age ≥ 75 years’.  **Type:** Patient.  **Covariate summary:** Demographics. | **Method to choose**  **covariate:** Not reported.  **Where method reported:** NA.  **Rationale for choosing the covariate:** Not reported.  **Where rationale reported:** NA. | **Where reported:** Protocol and review.  **Reason not reported in review:** NA.  **Reason not reported in protocol:** NA.  **Labelled as ‘posthoc’ if not in protocol:** NA. | **Data type:** Continuous.  **Continuous covariate categorised:** Yes.  **How determine it was categorised:** Categories given.  **Justification for categorising:** Not reported.  **Where justification reported:** NA.  **Categories reported:** Yes.  **Where categories reported:** Protocol and review.  **Justification for categories:** Not reported.  **Where justification reported:** NA. | **Analysis type reported in protocol:** Subgroup analysis.  **Analysis type reported in review: S**ubgroup analysis.  **Reason for changing mind:** NA.    **Analysis type in protocol according to glossary: S**ubgroup analysis.  **How determined the analysis type:** Categories given.  **Analysis type in review according to glossary: S**ubgroup analysis.  **How determined the analysis type:** Categories given.  **Analysed: yes**  **Reasons given (in the review) for not doing interaction analysis if planned:** NA.  **Reasons given for carrying out interaction analysis if they weren’t planned:** NA.  **Scenario in protocol:** ‘Where possible,’  **Rationale for planned scenario:** Not reported.  **Scenario in review (if reported specifically):** ‘Where possible,’  **Rationale for done scenario:** Not reported.  **Reason for changing mind:** Not reported. | **Method to detect interactions reported in protocol:** Not reported.  **Method to detect interactions reported in review:** Not reported.  **Reason for changing mind:** NA. |
| Bruins Slot 2013 | **Protocol: ‘**Patients who have had a previous stroke versus those who have not’.  **Review methods: ‘**Previous stroke versus no previous stroke.’  **Review results:** ‘Previous stroke or TIA’. ‘Previous stroke or TIA’; ‘No previous stroke or TIA’.  **Type:** Patient.  **Covariate summary:** Disease characteristics. | **Method to choose**  **covariate:** Not reported.  **Where method reported:** NA.  **Rationale for choosing the covariate:** Not reported.  **Where rationale reported:** NA. | **Where reported:** Protocol and review.  **Reason not reported in review:** NA.  **Reason not reported in protocol:** NA.  **Labelled as ‘posthoc’ if not in protocol:** NA. | **Data type:** Categorical.  **Continuous covariate categorised:** NA.  **How determine it was categorised:** NA.  **Justification for categorising:** NA.  **Where justification reported:** NA.  **Categories reported:** Yes.  **Where categories reported:** Protocol and review.  **Justification for categories:** Not reported.  **Where justification reported:** NA. | **Analysis type reported in protocol:** Subgroup analysis.  **Analysis type reported in review: S**ubgroup analysis.  **Reason for changing mind:** NA.    **Analysis type in protocol according to glossary: S**ubgroup analysis.  **How determined the analysis type:** Categories given.  **Analysis type in review according to glossary: S**ubgroup analysis.  **How determined the analysis type:** Categories given.  **Analysed: yes**.  **Reasons given (in the review) for not doing interaction analysis if planned:** NA.  **Reasons given for carrying out interaction analysis if they weren’t planned:** NA.  **Scenario in protocol:** ‘Where possible,’  **Rationale for planned scenario:** Not reported.  **Scenario in review (if reported specifically):** ‘Where possible,’  **Rationale for done scenario:** Not reported.  **Reason for changing mind:** Not reported. | **Method to detect interactions reported in protocol:** Not reported.  **Method to detect interactions reported in review:** Not reported.  **Reason for changing mind:** NA. |
| Bruins Slot 2013 | **Protocol:** ‘Patients who have received vitamin K antagonist treatment with a sufficient and clinically relevant time-in-therapeutic range (i. e. equal to or greater than 60% of INR measurements within the therapeutic range 2. 0 to 3. 0 versus less than 60%) (Connolly 2008; ESC 2010).’  **Review methods:** ‘Participants who received VKA treatment with time-in-therapeutic range (TTR) equal to or greater than 60% (’good quality’) versus less than 60% (’poor quality’) (Connolly 2008; ESC 2010)’.  **Review results:** ‘Quality of anticoagulation with VKA (TTR) (Good quality, Bad quality)’.  **Type:** Intervention.  **Covariate summary:** Time to therapeutic range (good/bad quality). | **Method to choose**  **covariate:** Not reported.  **Where method reported:** NA.  **Rationale for choosing the covariate:** Not reported.  **Where rationale reported:** NA. | **Where reported:** Protocol and review.  **Reason not reported in review:** NA.  **Reason not reported in protocol:** NA.  **Labelled as ‘posthoc’ if not in protocol:** NA. | **Data type:** Categorical.  **Continuous covariate categorised:** NA.  **How determine it was categorised:** NA.  **Justification for categorising:** NA.  **Where justification reported:** NA.  **Categories reported:** Yes.  **Where categories reported:** Protocol and review.  **Justification for categories:** Not reported.  **Where justification reported:** NA. | **Analysis type reported in protocol:** Subgroup analysis.  **Analysis type reported in review: S**ubgroup analysis.  **Reason for changing mind:** NA.    **Analysis type in protocol according to glossary: S**ubgroup analysis.  **How determined the analysis type:** Categories given.  **Analysis type in review according to glossary: S**ubgroup analysis.  **How determined the analysis type:** Categories given.  **Analysed: yes**.  **Reasons given (in the review) for not doing interaction analysis if planned:** NA.  **Reasons given for carrying out interaction analysis if they weren’t planned:** NA.  **Scenario in protocol:** ‘Where possible,’  **Rationale for planned scenario:** Not reported.  **Scenario in review (if reported specifically):** ‘Where possible,’  **Rationale for done scenario:** Not reported.  **Reason for changing mind:** Not reported. | **Method to detect interactions reported in protocol:** Not reported.  **Method to detect interactions reported in review:** Not reported.  **Reason for changing mind:** NA. |
| Bruins Slot 2013 | **Protocol:** ‘Patients who received concomitant antiplatelet therapy (i. e. aspirin) versus those who did not.’  **Review methods: ‘**Patients who received concomitant antiplatelet therapy (i. e. aspirin) versus those who did not’.  **Review results:** ‘Concomitant antiplatelet use’. ‘Concomitant antiplatelet use’; ‘No concomitant antiplatelet use’.  **Type:** Intervention.  **Covariate summary:** Additional interventions. | **Method to choose**  **covariate:** Not reported.  **Where method reported:** NA.  **Rationale for choosing the covariate:** Not reported.  **Where rationale reported:** NA. | **Where reported:** Protocol and review.  **Reason not reported in review:** NA.  **Reason not reported in protocol:** NA.  **Labelled as ‘posthoc’ if not in protocol:** NA. | **Data type:** Categorical.  **Continuous covariate categorised:** NA.  **How determine it was categorised:** NA.  **Justification for categorising:** NA.  **Where justification reported:** NA.  **Categories reported:** Yes.  **Where categories reported:** Protocol and review.  **Justification for categories:** Not reported.  **Where justification reported:** NA. | **Analysis type reported in protocol:** Subgroup analysis.  **Analysis type reported in review: S**ubgroup analysis.  **Reason for changing mind:** NA.    **Analysis type in protocol according to glossary: S**ubgroup analysis.  **How determined the analysis type:** Categories given.  **Analysis type in review according to glossary: S**ubgroup analysis.  **How determined the analysis type:** Categories given.  **Analysed: yes**  **Reasons given (in the review) for not doing interaction analysis if planned:** NA.  **Reasons given for carrying out interaction analysis if they weren’t planned:** NA.  **Scenario in protocol:** ‘Where possible,’  **Rationale for planned scenario:** Not reported.  **Scenario in review (if reported specifically):** ‘Where possible,’  **Rationale for done scenario:** Not reported.  **Reason for changing mind:** Not reported. | **Method to detect interactions reported in protocol:** Not reported.  **Method to detect interactions reported in review:** Not reported.  **Reason for changing mind:** NA. |
| Bruins Slot 2013 | **Protocol:** ‘Race’.  **Review methods: ‘**Race’.  **Review results: ‘**Race’. ‘Asian patients’; ‘White patients’; ‘Black patients’; ‘Other races’.  **Type:** Patient.  **Covariate summary:** Demographics. | **Method to choose**  **covariate:** Not reported.  **Where method reported:** NA.  **Rationale for choosing the covariate:** Not reported.  **Where rationale reported:** NA. | **Where reported:** Protocol and review.  **Reason not reported in review:** NA.  **Reason not reported in protocol:** NA.  **Labelled as ‘posthoc’ if not in protocol:** NA. | **Data type:** Categorical.  **Continuous covariate categorised:** NA.  **How determine it was categorised:** NA.  **Justification for categorising:** NA.  **Where justification reported:** NA.  **Categories reported:** Yes.  **Where categories reported:** Review only.  **Justification for categories:** Not reported.  **Where justification reported:** NA. | **Analysis type reported in protocol:** Subgroup analysis.  **Analysis type reported in review: S**ubgroup analysis.  **Reason for changing mind:** NA.    **Analysis type in protocol according to glossary: S**ubgroup analysis.  **How determined the analysis type:** Says ‘**S**ubgroup analysis.’ No reason to dispute.  **Analysis type in review according to glossary: S**ubgroup analysis.  **How determined the analysis type:** ‘**S**ubgroup analysis.’ No reason to dispute.  **Analysed: yes**  **Reasons given (in the review) for not doing interaction analysis if planned:** NA.  **Reasons given for carrying out interaction analysis if they weren’t planned:** NA.  **Scenario in protocol:** ‘Where possible,’  **Rationale for planned scenario:** Not reported.  **Scenario in review (if reported specifically):** ‘Where possible,’  **Rationale for done scenario:** Not reported.  **Reason for changing mind:** Not reported. | **Method to detect interactions reported in protocol:** Not reported.  **Method to detect interactions reported in review:** Not reported.  **Reason for changing mind:** NA. |
| Bruins Slot 2013 | **Protocol:** ‘Sex’.  **Review methods: ‘**Sex’.  **Review results:** ‘Sex. ‘Female’; ‘Male’.  **Type:** Patient.  **Covariate summary:** Demographics. | **Method to choose**  **covariate:** Not reported.  **Where method reported:** NA.  **Rationale for choosing the covariate:** Not reported.  **Where rationale reported:** NA. | **Where reported:** Protocol and review.  **Reason not reported in review:** NA.  **Reason not reported in protocol:** NA.  **Labelled as ‘posthoc’ if not in protocol:** NA. | **Data type:** Categorical.  **Continuous covariate categorised:** NA.  **How determine it was categorised:** NA.  **Justification for categorising:** NA.  **Where justification reported:** NA.  **Categories reported:** Yes.  **Where categories reported:** Review only.  **Justification for categories:** Not reported.  **Where justification reported:** NA. | **Analysis type reported in protocol:** Subgroup analysis.  **Analysis type reported in review: S**ubgroup analysis.  **Reason for changing mind:** NA.    **Analysis type in protocol according to glossary: S**ubgroup analysis.  **How determined the analysis type:** Says ‘**S**ubgroup analysis.’ No reason to dispute.  **Analysis type in review according to glossary: S**ubgroup analysis.  **How determined the analysis type:** ‘**S**ubgroup analysis.’ No reason to dispute.  **Analysed: yes**  **Reasons given (in the review) for not doing interaction analysis if planned:** NA.  **Reasons given for carrying out interaction analysis if they weren’t planned:** NA.  **Scenario in protocol:** ‘Where possible,’  **Rationale for planned scenario:** Not reported.  **Scenario in review (if reported specifically):** ‘Where possible,’  **Rationale for done scenario:** Not reported.  **Reason for changing mind:** Not reported. | **Method to detect interactions reported in protocol:** Not reported.  **Method to detect interactions reported in review:** Not reported.  **Reason for changing mind:** NA. |
| Bruins Slot 2013 | **Protocol:** ‘Trial quality’.  **Review methods: ‘**Study quality’.  **Review results:** Not analysed.  **Type:** Methodological.  **Covariate summary:** Quality. | **Method to choose**  **covariate:** Not reported.  **Where method reported:** NA.  **Rationale for choosing the covariate:** Not reported.  **Where rationale reported:** NA. | **Where reported:** Protocol and review.  **Reason not reported in review:** NA.  **Reason not reported in protocol:** NA.  **Labelled as ‘posthoc’ if not in protocol:** NA. | **Data type:** Categorical.  **Continuous covariate categorised:** NA.  **How determine it was categorised:** NA.  **Justification for categorising:** NA.  **Where justification reported:** NA.  **Categories reported:** Not reported.  **Where categories reported:** NA.  **Justification for categories:** Not reported.  **Where justification reported:** NA. | **Analysis type reported in protocol:** Subgroup analysis.  **Analysis type reported in review:** Not reported.  **Reason for changing mind:** Not reported.    **Analysis type in protocol according to glossary: S**ubgroup analysis.  **How determined the analysis type:** Says ‘stratifying’.  **Analysis type in review according to glossary:** Not reported.  **How determined the analysis type:** NA.  **Analysed:** No.  **Reasons given (in the review) for not doing interaction analysis if planned:** Not reported.  **Reasons given for carrying out interaction analysis if they weren’t planned:** NA.  **Scenario in protocol:** ‘If we find substantial heterogeneity on efficacy analysis, our intention  is to explore heterogeneity by stratifying for trial quality’  **Rationale for planned scenario:** Not reported.  **Scenario in review (if reported specifically):** Not reported.  **Rationale for done scenario:** NA.  **Reason for changing mind:** Not reported. | **Method to detect interactions reported in protocol:** Not reported.  **Method to detect interactions reported in review:** Not reported.  **Reason for changing mind:** NA. |
| Bruins Slot 2013 | **Protocol: ‘**Vitamin K antagonist treatment-experienced patients versus treatment-naïve patients’.  **Review methods: ‘**Vitamin K antagonist treatment-experienced patients versus treatment-naïve patients’.  **Review results:** ‘Previous VKA use’. ‘VKA naive’; ‘VKA experienced’.  **Type:** Patient.  **Covariate summary:** Disease characteristics. | **Method to choose**  **covariate:** Not reported.  **Where method reported:** NA.  **Rationale for choosing the covariate:** Not reported.  **Where rationale reported:** NA. | **Where reported:** Protocol and review.  **Reason not reported in review:** NA.  **Reason not reported in protocol:** NA.  **Labelled as ‘posthoc’ if not in protocol:** NA. | **Data type:** Categorical.  **Continuous covariate categorised:** NA.  **How determine it was categorised:** NA.  **Justification for categorising:** NA.  **Where justification reported:** NA.  **Categories reported:** Yes.  **Where categories reported:** Protocol and review.  **Justification for categories:** Not reported.  **Where justification reported:** NA. | **Analysis type reported in protocol:** Subgroup analysis.  **Analysis type reported in review: S**ubgroup analysis.  **Reason for changing mind:** NA.    **Analysis type in protocol according to glossary: S**ubgroup analysis.  **How determined the analysis type:** Categories given.  **Analysis type in review according to glossary: S**ubgroup analysis.  **How determined the analysis type:** Categories given.  **Analysed: yes**.  **Reasons given (in the review) for not doing interaction analysis if planned:** NA.  **Reasons given for carrying out interaction analysis if they weren’t planned:** NA.  **Scenario in protocol:** ‘Where possible,’  **Rationale for planned scenario:** Not reported.  **Scenario in review (if reported specifically):** ‘Where possible,’  **Rationale for done scenario:** Not reported.  **Reason for changing mind:** Not reported. | **Method to detect interactions reported in protocol:** Not reported.  **Method to detect interactions reported in review:** Not reported.  **Reason for changing mind:** NA. |
| Bruins Slot 2013 | **Protocol:** Administration route of factor Xa inhibitor. We judged the following to be two covariates: ‘Where possible, we will do subgroup analyses for: administration route and dose of factor Xa inhibitor’.  **Review methods:** Administration route of factor Xa inhibitor. We judged the following to be two covariates: ‘Where possible, we performed subgroup analyses for: administration route and dose of factor Xa inhibitor’.  **Review results:** ‘Route of administration’. ‘Oral administration’; ‘Parenteral administration’.  **Type:** Intervention.  **Covariate summary:** Route of administration. | **Method to choose**  **covariate:** Not reported.  **Where method reported:** NA.  **Rationale for choosing the covariate:** Not reported.  **Where rationale reported:** NA. | **Where reported:** Protocol and review.  **Reason not reported in review:** NA.  **Reason not reported in protocol:** NA.  **Labelled as ‘posthoc’ if not in protocol:** NA. | **Data type:** Categorical.  **Continuous covariate categorised:** NA.  **How determine it was categorised:** NA.  **Justification for categorising:** NA.  **Where justification reported:** NA.  **Categories reported:** Yes.  **Where categories reported:** Review only.  **Justification for categories:** Not reported.  **Where justification reported:** NA. | **Analysis type reported in protocol:** Subgroup analysis.  **Analysis type reported in review: S**ubgroup analysis.  **Reason for changing mind:** NA.    **Analysis type in protocol according to glossary: S**ubgroup analysis.  **How determined the analysis type:** Says ‘**S**ubgroup analysis.’ No reason to dispute.  **Analysis type in review according to glossary: S**ubgroup analysis.  **How determined the analysis type:** ‘**S**ubgroup analysis.’ No reason to dispute.  **Analysed: Yes**.  **Reasons given (in the review) for not doing interaction analysis if planned:** NA.  **Reasons given for carrying out interaction analysis if they weren’t planned:** NA.  **Scenario in protocol:** ‘Where possible,’  **Rationale for planned scenario:** Not reported.  **Scenario in review (if reported specifically):** ‘Where possible,’  **Rationale for done scenario:** Not reported.  **Reason for changing mind:** Not reported. | **Method to detect interactions reported in protocol:** Not reported.  **Method to detect interactions reported in review:** Not reported.  **Reason for changing mind:** NA. |
| Bruins Slot 2013 | **Protocol:** Dose of factor Xa inhibitor. We judged the following to be two covariates: ‘Where possible, we will do subgroup analyses for: administration route and dose of factor Xa inhibitor’.  **Review methods:** Dose of factor Xa inhibitor. We judged the following to be two covariates: ‘Where possible, we performed subgroup analyses for: administration route and dose of factor Xa inhibitor’.  **Review results:** ‘Dose of Factor Xa inhibitor’. ‘Apixaban 2. 5mg twice daily’; ‘Apixaban 5 mg twice daily’; ‘Edoxaban 30mg once daily’; ‘Edoxaban 60mg once daily’; ‘Edoxaban 30mg twice daily’; ‘Edoxaban 60mg twice daily’; ‘Rivaroxaban 10mg once daily’; ‘Rivaroxaban 15mg once daily’; ‘Darexaban 30mg once daily’; ‘Darexaban 60mg once daily’; ‘Darexaban 120mg once daily’; ‘Darexaban 240mg’; ‘Idraparinux 1,5mg once weekly’; ‘Idraparinux 2,5mg once weekly’; ‘Betrixaban 40 mg’; ‘Betrixaban 60 mg’; ‘Betrixaban 80 mg’.  **Type:** Intervention.  **Covariate summary:** Dose. | **Method to choose**  **covariate:** Not reported.  **Where method reported:** NA.  **Rationale for choosing the covariate:** Not reported.  **Where rationale reported:** NA. | **Where reported:** Protocol and review.  **Reason not reported in review:** NA.  **Reason not reported in protocol:** NA.  **Labelled as ‘posthoc’ if not in protocol:** NA. | **Data type:** Continuous.  **Continuous covariate categorised:** Yes.  **How determine it was categorised:** categories given.  **Justification for categorising:** Not reported.  **Where justification reported:** NA.  **Categories reported:** Yes.  **Where categories reported:** Review only.  **Justification for categories:** Not reported.  **Where justification reported:** NA. | **Analysis type reported in protocol:** Subgroup analysis.  **Analysis type reported in review: S**ubgroup analysis.  **Reason for changing mind:** NA.    **Analysis type in protocol according to glossary: S**ubgroup analysis.  **How determined the analysis type:** Says ‘**S**ubgroup analysis.’ No reason to dispute.  **Analysis type in review according to glossary: S**ubgroup analysis.  **How determined the analysis type:** ‘**S**ubgroup analysis.’ No reason to dispute.  **Analysed: yes**.  **Reasons given (in the review) for not doing interaction analysis if planned:** NA.  **Reasons given for carrying out interaction analysis if they weren’t planned:** NA.  **Scenario in protocol:** ‘Where possible,’  **Rationale for planned scenario:** Not reported.  **Scenario in review (if reported specifically):** ‘Where possible,’  **Rationale for done scenario:** Not reported.  **Reason for changing mind:** Not reported. | **Method to detect interactions reported in protocol:** Not reported.  **Method to detect interactions reported in review:** Not reported.  **Reason for changing mind:** NA. |
| Bruins Slot 2013 | **Protocol:** Not reported.  **Review methods:** Not reported.  **Review results:** Different factor Xa inhibitors. ‘Apixaban versus VKA’; ‘Darexaban versus VKA’; ‘Edoxaban versus VKA’; ‘Idraparinux versus VKA’; ‘Rivaroxaban versus VKA’; ‘Betrixaban versus VKA’).  **Type:** Intervention.  **Covariate summary:** Type of intervention or control. | **Method to choose**  **covariate:** Not reported.  **Where method reported:** NA.  **Rationale for choosing the covariate:** Not reported.  **Where rationale reported:** NA. | **Where reported:** Review only.  **Reason not reported in review:** NA.  **Reason not reported in protocol:** Not reported.  **Labelled as ‘posthoc’ if not in protocol:** Not reported. | **Data type:** Categorical.  **Continuous covariate categorised:** NA.  **How determine it was categorised:** NA.  **Justification for categorising:** NA.  **Where justification reported:** NA.  **Categories reported:** Yes.  **Where categories reported:** Review only.  **Justification for categories:** Not reported.  **Where justification reported:** NA. | **Analysis type reported in protocol:** Not reported.  **Analysis type reported in review:** Unnamed analysis.  **Reason for changing mind:** Not reported.    **Analysis type in protocol according to glossary:** Not reported.  **How determined the analysis type:** NA.  **Analysis type in review according to glossary:** Subgroup analysis.  **How determined the analysis type:** Results presented.  **Analysed: yes**.  **Reasons given (in the review) for not doing interaction analysis if planned:** NA.  **Reasons given for carrying out interaction analysis if they weren’t planned:** Not reported.  **Scenario in protocol:** Not reported.  **Rationale for planned scenario:** NA.  **Scenario in review (if reported specifically):** Not reported.  **Rationale for done scenario:** NA.  **Reason for changing mind:** Not reported. | **Method to detect interactions reported in protocol:** Not reported.  **Method to detect interactions reported in review:** Not reported.  **Reason for changing mind:** NA. |
| Cavalheri 2013 | **Protocol:** ‘Supervised versus non-supervised exercise training’.  **Review methods:** Not reported.  **Review results:** Not analysed.  **Type:** Intervention.  **Covariate summary:** Type of intervention or control. | **Method to choose**  **covariate:** Not reported.  **Where method reported:** NA.  **Rationale for choosing the covariate:** Not reported.  **Where rationale reported:** NA. | **Where reported:** Protocol only.  **Reason not reported in review:** Not reported.  **Reason not reported in protocol:** NA.  **Labelled as ‘posthoc’ if not in protocol:** NA**.** | **Data type:** Categorical.  **Continuous covariate categorised:** NA.  **How determine it was categorised:** NA.  **Justification for categorising:** NA.  **Where justification reported:** NA.  **Categories reported:** Yes.  **Where categories reported:** Protocol only.  **Justification for categories:** Not reported.  **Where justification reported:** NA. | **Analysis type reported in protocol:** Subgroup analysis.  **Analysis type reported in review:** Not reported.  **Reason for changing mind:** Not reported.    **Analysis type in protocol according to glossary:** Subgroup analysis.  **How determined the analysis type:** Categories given.  **Analysis type in review according to glossary:** Not reported.  **How determined the analysis type:** NA.  **Analysed:** No.  **Reasons given (in the review) for not doing interaction analysis if planned:** ‘We did not perform subgroup analysis due to the small number of studies included  in the meta-analyses as well as their small sample sizes.’  **Reasons given for carrying out interaction analysis if they weren’t planned:** NA.  **Scenario in protocol:** ‘Subgroup analysis will  be performed to investigate whether clinical diversity andmethodological  diversity are potential causes of heterogeneity.’  **Rationale for planned scenario:** Not reported.  **Scenario in review (if reported specifically):** Not reported.  **Rationale for done scenario:** NA.  **Reason for changing mind:** NA**.** | **Method to detect interactions reported in protocol:** ‘Heterogeneity between subgroups will be performed using the  test of interaction between subgroups, in order to assess whether  treatment effects differ between exercise training regimens and also  to determinewhether the use of adjuvant chemotherapy affects the  impact of exercise training.’  **Method to detect interactions reported in review:** Not reported.  **Reason for changing mind:** Not reported. |
| Cavalheri 2013 | **Protocol:** ‘The use of chemotherapy treatment versus no chemotherapy treatment’.  **Review methods:** Not reported.  **Review results:** Not analysed.  **Type:** Intervention.  **Covariate summary:** Additional interventions. | **Method to choose**  **covariate:** Not reported.  **Where method reported:** NA.  **Rationale for choosing the covariate:** Not reported.  **Where rationale reported:** NA. | **Where reported:** Protocol only.  **Reason not reported in review:** Not reported.  **Reason not reported in protocol:** NA.  **Labelled as ‘posthoc’ if not in protocol:** NA**.** | **Data type:** Categorical.  **Continuous covariate categorised:** NA.  **How determine it was categorised:** NA.  **Justification for categorising:** NA.  **Where justification reported:** NA.  **Categories reported:** Yes.  **Where categories reported:** Protocol only.  **Justification for categories:** Not reported.  **Where justification reported:** NA. | **Analysis type reported in protocol:** Subgroup analysis.  **Analysis type reported in review:** Not reported.  **Reason for changing mind:** Not reported.    **Analysis type in protocol according to glossary:** Subgroup analysis.  **How determined the analysis type:** Categories given.  **Analysis type in review according to glossary:** Not reported.  **How determined the analysis type:** NA.  **Analysed:** No.  **Reasons given (in the review) for not doing interaction analysis if planned:** ‘We did not perform subgroup analysis due to the small number of studies included  in the meta-analyses as well as their small sample sizes.’  **Reasons given for carrying out interaction analysis if they weren’t planned:** NA.  **Scenario in protocol:** ‘Subgroup analysis will  be performed to investigate whether clinical diversity andmethodological  diversity are potential causes of heterogeneity.’  **Rationale for planned scenario:** Not reported.  **Scenario in review (if reported specifically):** Not reported.  **Rationale for done scenario:** NA.  **Reason for changing mind:** NA**.** | **Method to detect interactions reported in protocol:** ‘Heterogeneity between subgroups will be performed using the  test of interaction between subgroups, in order to assess whether  treatment effects differ between exercise training regimens and also  to determinewhether the use of adjuvant chemotherapy affects the  impact of exercise training.’  **Method to detect interactions reported in review:** Not reported.  **Reason for changing mind:** Not reported. |
| Cavalheri 2013 | **Protocol:** A combination. We judged the following to be four covariates: ‘using quality indicators such as concealed allocation, assessor blinding, or intention-to-treat analysis, or a combination.’  **Review methods:** Not reported.  **Review results:** Not analysed.  **Type:** Methodological.  **Covariate summary:** A combination of quality indicators. | **Method to choose**  **covariate:** Not reported.  **Where method reported:** NA.  **Rationale for choosing the covariate:** Not reported.  **Where rationale reported:** NA. | **Where reported:** Protocol only.  **Reason not reported in review:** Not reported.  **Reason not reported in protocol:** NA.  **Labelled as ‘posthoc’ if not in protocol:** NA**.** | **Data type:** Categorical.  **Continuous covariate categorised:** NA.  **How determine it was categorised:** NA.  **Justification for categorising:** NA.  **Where justification reported:** NA.  **Categories reported:** Not reported.  **Where categories reported:** NA.  **Justification for categories:** Not reported.  **Where justification reported:** NA. | **Analysis type reported in protocol:** Sensitivty analysis.  **Analysis type reported in review:** Not reported.  **Reason for changing mind:** Not reported.    **Analysis type in protocol according to glossary:** Sensitivty analysis.  **How determined the analysis type:** Says ‘Sensitivty analysis.’ No reason to dispute.  **Analysis type in review according to glossary:** Not reported.  **How determined the analysis type:** NA.  **Analysed:** No.  **Reasons given (in the review) for not doing interaction analysis if planned:** Not reported.  **Reasons given for carrying out interaction analysis if they weren’t planned:** NA.  **Scenario in protocol:** ‘If there is significant heterogeneity among the studies a sensitivity  analyses.’  **Rationale for planned scenario:** Not reported.  **Scenario in review (if reported specifically):** Not reported.  **Rationale for done scenario:** NA.  **Reason for changing mind:** NA**.** | **Method to detect interactions reported in protocol:** Not reported.  **Method to detect interactions reported in review:** Not reported.  **Reason for changing mind:** NA. |
| Cavalheri 2013 | **Protocol:** Assessor blinding . We judged the following to be four covariates: ‘using quality indicators such as concealed allocation, assessor blinding, or intention-to-treat analysis, or a combination.’  **Review methods:** Not reported.  **Review results:** Not analysed.  **Type:** Methodological.  **Covariate summary:** Blinding. | **Method to choose**  **covariate:** Not reported.  **Where method reported:** NA.  **Rationale for choosing the covariate:** Not reported.  **Where rationale reported:** NA. | **Where reported:** Protocol only.  **Reason not reported in review:** Not reported.  **Reason not reported in protocol:** NA.  **Labelled as ‘posthoc’ if not in protocol:** NA**.** | **Data type:** Categorical.  **Continuous covariate categorised:** NA.  **How determine it was categorised:** NA.  **Justification for categorising:** NA.  **Where justification reported:** NA.  **Categories reported:** Not reported.  **Where categories reported:** NA.  **Justification for categories:** Not reported.  **Where justification reported:** NA. | **Analysis type reported in protocol:** Sensitivty analysis.  **Analysis type reported in review:** Not reported.  **Reason for changing mind:** Not reported.    **Analysis type in protocol according to glossary:** Sensitivty analysis.  **How determined the analysis type:** Says ‘Sensitivty analysis.’ No reason to dispute.  **Analysis type in review according to glossary:** Not reported.  **How determined the analysis type:** NA.  **Analysed:** No.  **Reasons given (in the review) for not doing interaction analysis if planned:** Not reported.  **Reasons given for carrying out interaction analysis if they weren’t planned:** NA.  **Scenario in protocol:** ‘If there is significant heterogeneity among the studies a sensitivity  analyses.’  **Rationale for planned scenario:** Not reported.  **Scenario in review (if reported specifically):** Not reported.  **Rationale for done scenario:** NA.  **Reason for changing mind:** NA**.** | **Method to detect interactions reported in protocol:** Not reported.  **Method to detect interactions reported in review:** Not reported.  **Reason for changing mind:** NA. |
| Cavalheri 2013 | **Protocol:** Concealed allocation. We judged the following to be four covariates: ‘using quality indicators such as concealed allocation, assessor blinding, or intention-to-treat analysis, or a combination.’  **Review methods:** Not reported.  **Review results:** Not analysed.  **Type:** Methodological.  **Covariate summary:** Allocation concealment. | **Method to choose**  **covariate:** Not reported.  **Where method reported:** NA.  **Rationale for choosing the covariate:** Not reported.  **Where rationale reported:** NA. | **Where reported:** Protocol only.  **Reason not reported in review:** Not reported.  **Reason not reported in protocol:** NA.  **Labelled as ‘posthoc’ if not in protocol:** NA**.** | **Data type:** Categorical.  **Continuous covariate categorised:** NA.  **How determine it was categorised:** NA.  **Justification for categorising:** NA.  **Where justification reported:** NA.  **Categories reported:** Not reported.  **Where categories reported:** NA.  **Justification for categories:** Not reported.  **Where justification reported:** NA. | **Analysis type reported in protocol:** Sensitivty analysis.  **Analysis type reported in review:** Not reported.  **Reason for changing mind:** Not reported.    **Analysis type in protocol according to glossary:** Sensitivty analysis.  **How determined the analysis type:** Says ‘Sensitivty analysis.’ No reason to dispute.  **Analysis type in review according to glossary:** Not reported.  **How determined the analysis type:** NA.  **Analysed:** No.  **Reasons given (in the review) for not doing interaction analysis if planned:** Not reported.  **Reasons given for carrying out interaction analysis if they weren’t planned:** NA.  **Scenario in protocol:** ‘If there is significant heterogeneity among the studies a sensitivity  analyses.’  **Rationale for planned scenario:** Not reported.  **Scenario in review (if reported specifically):** Not reported.  **Rationale for done scenario:** NA.  **Reason for changing mind:** NA**.** | **Method to detect interactions reported in protocol:** Not reported.  **Method to detect interactions reported in review:** Not reported.  **Reason for changing mind:** NA. |
| Cavalheri 2013 | **Protocol:** Intention-to-treat analysis. We judged the following to be four covariates: ‘using quality indicators such as concealed allocation, assessor blinding, or intention-to-treat analysis, or a combination.’  **Review methods:** Not reported.  **Review results:** Not analysed.  **Type:** Methodological.  **Covariate summary:** Intention to treat. | **Method to choose**  **covariate:** Not reported.  **Where method reported:** NA.  **Rationale for choosing the covariate:** Not reported.  **Where rationale reported:** NA. | **Where reported:** Protocol only.  **Reason not reported in review:** Not reported.  **Reason not reported in protocol:** NA.  **Labelled as ‘posthoc’ if not in protocol:** NA**.** | **Data type:** Categorical.  **Continuous covariate categorised:** NA.  **How determine it was categorised:** NA.  **Justification for categorising:** NA.  **Where justification reported:** NA.  **Categories reported:** Not reported.  **Where categories reported:** NA.  **Justification for categories:** Not reported.  **Where justification reported:** NA. | **Analysis type reported in protocol:** Sensitivty analysis.  **Analysis type reported in review:** Not reported.  **Reason for changing mind:** Not reported.    **Analysis type in protocol according to glossary:** Sensitivty analysis.  **How determined the analysis type:** Says ‘Sensitivty analysis.’ No reason to dispute.  **Analysis type in review according to glossary:** Not reported.  **How determined the analysis type:** NA.  **Analysed:** No.  **Reasons given (in the review) for not doing interaction analysis if planned:** Not reported.  **Reasons given for carrying out interaction analysis if they weren’t planned:** NA.  **Scenario in protocol:** ‘If there is significant heterogeneity among the studies a sensitivity  analyses.’  **Rationale for planned scenario:** Not reported.  **Scenario in review (if reported specifically):** Not reported.  **Rationale for done scenario:** NA.  **Reason for changing mind:** NA**.** | **Method to detect interactions reported in protocol:** Not reported.  **Method to detect interactions reported in review:** Not reported.  **Reason for changing mind:** NA. |
| Cavalheri 2013 | **Protocol:** Minimal duration of the programme (e. g. 20 sessions or more versus less than 20 sessions of exercise).  **Review methods:** Not reported.  **Review results:** Not analysed.  **Type:** Intervention.  **Covariate summary:** Duration of intervention. | **Method to choose**  **covariate:** Not reported.  **Where method reported:** NA.  **Rationale for choosing the covariate:** Not reported.  **Where rationale reported:** NA. | **Where reported:** Protocol only.  **Reason not reported in review:** Not reported.  **Reason not reported in protocol:** NA.  **Labelled as ‘posthoc’ if not in protocol:** NA**.** | **Data type:** Continuous.  **Continuous covariate categorised:** Yes.  **How determine it was categorised:** Categories given.  **Justification for categorising:** Not reported.  **Where justification reported:** NA.  **Categories reported:** Yes.  **Where categories reported:** Protocol only.  **Justification for categories:** Not reported.  **Where justification reported:** NA. | **Analysis type reported in protocol:** Subgroup analysis.  **Analysis type reported in review:** Not reported.  **Reason for changing mind:** Not reported.    **Analysis type in protocol according to glossary:** Subgroup analysis.  **How determined the analysis type:** Categories given.  **Analysis type in review according to glossary:** Not reported.  **How determined the analysis type:** NA.  **Analysed:** No.  **Reasons given (in the review) for not doing interaction analysis if planned:** ‘We did not perform subgroup analysis due to the small number of studies included  in the meta-analyses as well as their small sample sizes.’  **Reasons given for carrying out interaction analysis if they weren’t planned:** NA.  **Scenario in protocol:** ‘Subgroup analysis will  be performed to investigate whether clinical diversity andmethodological  diversity are potential causes of heterogeneity.’  **Rationale for planned scenario:** Not reported.  **Scenario in review (if reported specifically):** Not reported.  **Rationale for done scenario:** NA.  **Reason for changing mind:** NA**.** | **Method to detect interactions reported in protocol:** ‘Heterogeneity between subgroups will be performed using the  test of interaction between subgroups, in order to assess whether  treatment effects differ between exercise training regimens and also  to determinewhether the use of adjuvant chemotherapy affects the  impact of exercise training.’  **Method to detect interactions reported in review:** Not reported.  **Reason for changing mind:** Not reported. |
| Chaparro 2013 | **Protocol:** ‘duration of intervention**’.** We judged the following to be two covariates: ‘Studies will be combined if they evaluate the same study drug(s) at roughly similar doses and durations of treatment (e. g. a study evaluating a single preoperative drug dose would not be compared to another study evaluating several weeks of treatment with the same drug).’ We judged the following to be four covariates: ‘Sensitivity analyses will be used to evaluate the robustness of a particular result by repeating primary analyses without studies considered to be outliers with respect to study quality, drug dose/duration or pain measurement scales.’  **Review methods:** ‘duration of intervention**’.** We judged the following to be two covariates: ‘Studies were combined if they evaluated the same study drug(s) at roughly similar doses and durations of treatment (for example a study evaluating a single preoperative drug dose would not be compared to another study evaluating several weeks of treatment with the same drug).’ We judged the following to be four covariates: ‘Sensitivity analyses were used to evaluate the robustness of a particular result by repeating primary analyses without any studies considered to be outliers with respect to study quality, drug dose and duration, or pain measurement scales.’  **Review results:** Not analysed.  **Type:** Intervention.  **Covariate summary:** Duration of intervention. | **Method to choose**  **covariate:** Not reported.  **Where method reported:** NA.  **Rationale for choosing the covariate:** Not reported.  **Where rationale reported:** NA. | **Where reported:** Protocol and review.  **Reason not reported in review:** NA.  **Reason not reported in protocol:** NA.  **Labelled as ‘posthoc’ if not in protocol:** NA. | **Data type:** Continuous.  **Continuous covariate categorised:** Yes.  **How determine it was categorised:** Sensitivity analysis planned.  **Justification for categorising:** Not reported.  **Where justification reported:** NA.  **Categories reported:** Not reported.  **Where categories reported:** NA.  **Justification for categories:** Not reported.  **Where justification reported:** NA. | **Analysis type reported in protocol:** Subgroup analysis and sensitivity analysis.  **Analysis type reported in review:** Subgroup analysis and sensitivity analysis.  **Reason for changing mind:** NA.    **Analysis type in protocol according to glossary:** Subgroup analysis and sensitivity analysis.  **How determined the analysis type:** Says ‘sensitivity analysis’ and ‘subgroup analysis’. No reason to dispute.  **Analysis type in review according to glossary:** Subgroup analysis and sensitivity analysis.  **How determined the analysis type:** Says ‘sensitivity analysis’ and ‘subgroup analysis’. No reason to dispute.  **Analysed:** No.  **Reasons given (in the review) for not doing interaction analysis if planned:** Not reported.  **Reasons given for carrying out interaction analysis if they weren’t planned:** NA.  **Scenario in protocol:** Not reported.  **Rationale for planned scenario:** NA.  **Scenario in review (if reported specifically):** Not reported.  **Rationale for done scenario:** NA..  **Reason for changing mind:** NA. | **Method to detect interactions reported in protocol:** Not reported.  **Method to detect interactions reported in review:** Not reported.  **Reason for changing mind:** NA. |
| Chaparro 2013 | **Protocol:** ‘Study participants with respect to preoperative pain at the surgical site.’  **Review methods:** ‘Study participants with respect to preoperative pain at the surgical site.’  **Review results:** Not analysed.  **Type:** Patient.  **Covariate summary:** Disease characteristics. | **Method to choose**  **covariate:** Not reported.  **Where method reported:** NA.  **Rationale for choosing the covariate:** Not reported.  **Where rationale reported:** NA. | **Where reported:** Protocol and review.  **Reason not reported in review:** NA.  **Reason not reported in protocol:** NA.  **Labelled as ‘posthoc’ if not in protocol:** NA. | **Data type:** Unclear.  **Continuous covariate categorised:** Not reported.  **How determine it was categorised:** NA.  **Justification for categorising:** Not reported.  **Where justification reported:** NA.  **Categories reported:** Not reported.  **Where categories reported:** NA.  **Justification for categories:** Not reported.  **Where justification reported:** NA. | **Analysis type reported in protocol:** Subgroup analysis.  **Analysis type reported in review:** Subgroup analysis.  **Reason for changing mind:** NA.    **Analysis type in protocol according to glossary:** Subgroup analysis.  **How determined the analysis type:** Says ‘Subgroup analysis.. No reason to dispute.  **Analysis type in review according to glossary:** Subgroup analysis.  **How determined the analysis type:** Says ‘Subgroup analysis’. No reason to dispute.  **Analysed:** No.  **Reasons given (in the review) for not doing interaction analysis if planned:** Not reported.  **Reasons given for carrying out interaction analysis if they weren’t planned:** NA.  **Scenario in protocol:** Not reported.  **Rationale for planned scenario:** NA.  **Scenario in review (if reported specifically):** Not reported.  **Rationale for done scenario:** NA..  **Reason for changing mind:** NA. | **Method to detect interactions reported in protocol:** Not reported.  **Method to detect interactions reported in review:** Not reported.  **Reason for changing mind:** NA. |
| Chaparro 2013 | **Protocol:** ‘Surgical procedures.’  **Review methods: ‘**Surgical procedures’.  **Review results: ‘**Abdominal and/or pelvic surgery’; ‘Amputation’; ‘Breast Surgery’; ‘Hemorrhoidectomy’; ‘Orthopedic Surgery’; ‘Thoracotomy’; ‘Caesarean section’; ‘Cardiac Surgery’; ‘Total hip arthroplasty’; ‘Total Knee Arthroplasty’; ‘Spine Surgery’; ‘Thyroidectomy’).  **Type:** Intervention.  **Covariate summary:** Type of intervention or control. | **Method to choose**  **covariate:** Not reported.  **Where method reported:** NA.  **Rationale for choosing the covariate:** Not reported.  **Where rationale reported:** NA. | **Where reported:** Protocol and review.  **Reason not reported in review:** NA.  **Reason not reported in protocol:** NA.  **Labelled as ‘posthoc’ if not in protocol:** NA. | **Data type:** Categorical.  **Continuous covariate categorised:** NA.  **How determine it was categorised:** NA.  **Justification for categorising:** NA.  **Where justification reported:** NA.  **Categories reported:** Yes.  **Where categories reported:** Review only.  **Justification for categories:** Not reported.  **Where justification reported:** NA. | **Analysis type reported in protocol:** Subgroup analysis.  **Analysis type reported in review:** Subgroup analysis.  **Reason for changing mind:** NA.    **Analysis type in protocol according to glossary:** Subgroup analysis.  **How determined the analysis type:** Says ‘Subgroup analysis.. No reason to dispute.  **Analysis type in review according to glossary:** Subgroup analysis.  **How determined the analysis type:** results reported.  **Analysed:** Yes.  **Reasons given (in the review) for not doing interaction analysis if planned:** NA.  **Reasons given for carrying out interaction analysis if they weren’t planned:** NA.  **Scenario in protocol:** Not reported.  **Rationale for planned scenario:** NA.  **Scenario in review (if reported specifically):** Not reported.  **Rationale for done scenario:** NA..  **Reason for changing mind:** NA. | **Method to detect interactions reported in protocol:** Not reported.  **Method to detect interactions reported in review:** Not reported.  **Reason for changing mind:** NA. |
| Chaparro 2013 | **Protocol:** ‘The primary comparison of interest will be between study drug(s) and placebo. Comparisons of study drug(s) and any other active treatment comparators will also be made.’  **Review methods: ‘**The primary comparison of interest was between study drug(s) and placebo. Comparisons of study drug(s) and any other active treatment comparators were also to be made.’  **Review results: ‘**Comparison 1. Ketamine versus placebo comparisons’; ‘Comparison 2. Gabapentin versus placebo’; ‘Comparison 3. Pregabalin versus placebo’; ‘Comparison 4. Venlafaxine versus placebo comparisons’ (in plot) and ‘other drugs’ (in text).  **Type:** Intervention  **Covariate summary:** Type of intervention or control. | **Method to choose**  **covariate:** Not reported.  **Where method reported:** NA.  **Rationale for choosing the covariate:** Not reported.  **Where rationale reported:** NA. | **Where reported:** Protocol and review.  **Reason not reported in review:** NA.  **Reason not reported in protocol:** NA.  **Labelled as ‘posthoc’ if not in protocol:** NA. | **Data type:** Categorical.  **Continuous covariate categorised:** NA.  **How determine it was categorised:** NA.  **Justification for categorising:** NA.  **Where justification reported:** NA.  **Categories reported:** Yes.  **Where categories reported:** Protocol and review.  **Justification for categories:** Not reported.  **Where justification reported:** NA. | **Analysis type reported in protocol:** Unnamed analysis.  **Analysis type reported in review:** Unnamed analysis.  **Reason for changing mind:** NA.    **Analysis type in protocol according to glossary:** Stratification/subgroup analysis.  **How determined the analysis type:** Categories given..  **Analysis type in review according to glossary:** Stratification/subgroup analysis.  **How determined the analysis type:** Categories given..  **Analysed:** Yes.  **Reasons given (in the review) for not doing interaction analysis if planned:** NA.  **Reasons given for carrying out interaction analysis if they weren’t planned:** NA.  **Scenario in protocol:** Not reported.  **Rationale for planned scenario:** NA.  **Scenario in review (if reported specifically):** Not reported.  **Rationale for done scenario:** NA..  **Reason for changing mind:** NA. | **Method to detect interactions reported in protocol:** Not reported.  **Method to detect interactions reported in review:** Not reported.  **Reason for changing mind:** NA. |
| Chaparro 2013 | **Protocol:** ‘Timing of intervention’.  **Review methods:** ‘Timing of intervention’.  **Review results:** Not analysed.  **Type:** Intervention  **Covariate summary:** Timing of intervention. | **Method to choose**  **covariate:** Not reported.  **Where method reported:** NA.  **Rationale for choosing the covariate:** Not reported.  **Where rationale reported:** NA. | **Where reported:** Protocol and review.  **Reason not reported in review:** NA.  **Reason not reported in protocol:** NA.  **Labelled as ‘posthoc’ if not in protocol:** NA. | **Data type:** Continuous.  **Continuous covariate categorised:** Yes.  **How determine it was categorised:** Subgroup analysis planned.  **Justification for categorising:** Not reported.  **Where justification reported:** NA.  **Categories reported:** Not reported.  **Where categories reported:** NA.  **Justification for categories:** Not reported.  **Where justification reported:** NA. | **Analysis type reported in protocol:** Subgroup analysis.  **Analysis type reported in review:** Subgroup analysis.  **Reason for changing mind:** NA.    **Analysis type in protocol according to glossary:** Subgroup analysis.  **How determined the analysis type:** Says ‘Subgroup analysis.. No reason to dispute.  **Analysis type in review according to glossary:** Subgroup analysis.  **How determined the analysis type:** Says ‘Subgroup analysis’. No reason to dispute.  **Analysed:** No.  **Reasons given (in the review) for not doing interaction analysis if planned:** Not reported.  **Reasons given for carrying out interaction analysis if they weren’t planned:** NA.  **Scenario in protocol:** Not reported.  **Rationale for planned scenario:** NA.  **Scenario in review (if reported specifically):** Not reported.  **Rationale for done scenario:** NA..  **Reason for changing mind:** NA. | **Method to detect interactions reported in protocol:** Not reported.  **Method to detect interactions reported in review:** Not reported.  **Reason for changing mind:** NA. |
| Chaparro 2013 | **Protocol:** Dose. We judged the following to be two covariates: ‘Studies will be combined if they evaluate the same study drug(s) at roughly similar doses and durations of treatment (e. g. a study evaluating a single preoperative drug dose would not be compared to another study evaluating several weeks of treatment with the same drug).’ We judged the following to be four covariates: ‘Sensitivity analyses will be used to evaluate the robustness of a particular result by repeating primary analyses without studies considered to be outliers with respect to study quality, drug dose/duration or pain measurement scales.’  **Review methods:** Dose. We judged the following to be two covariates: ‘Studies were combined if they evaluated the same study drug(s) at roughly similar doses and durations of treatment (for example a study evaluating a single preoperative drug dose would not be compared to another study evaluating several weeks of treatment with the same drug).’ We judged the following to be four covariates: ‘Sensitivity analyses were used to evaluate the robustness of a particular result by repeating primary analyses without any studies considered to be outliers with respect to study quality, drug dose and duration, or pain measurement scales.’  **Review results:** Not analysed.  **Type:** Intervention.  **Covariate summary:** Dose. | **Method to choose**  **covariate:** Not reported.  **Where method reported:** NA.  **Rationale for choosing the covariate:** Not reported.  **Where rationale reported:** NA. | **Where reported:** Protocol and review.  **Reason not reported in review:** NA.  **Reason not reported in protocol:** NA.  **Labelled as ‘posthoc’ if not in protocol:** NA. | **Data type:** Continuous.  **Continuous covariate categorised:** Yes.  **How determine it was categorised:** Sensitivity analysis planned.  **Justification for categorising:** Not reported.  **Where justification reported:** NA.  **Categories reported:** Not reported.  **Where categories reported:** NA.  **Justification for categories:** Not reported.  **Where justification reported:** NA. | **Analysis type reported in protocol:** Sensitivity analysis.  **Analysis type reported in review:** Sensitivity analysis.  **Reason for changing mind:** NA.    **Analysis type in protocol according to glossary:** Sensitivity analysis.  **How determined the analysis type:** Says ‘sensitivity analysis’. No reason to dispute.  **Analysis type in review according to glossary:** Sensitivity analysis.  **How determined the analysis type:** Says ‘sensitivity analysis’. No reason to dispute.  **Analysed:** No.  **Reasons given (in the review) for not doing interaction analysis if planned:** Not reported.  **Reasons given for carrying out interaction analysis if they weren’t planned:** NA.  **Scenario in protocol:** Not reported.  **Rationale for planned scenario:** NA.  **Scenario in review (if reported specifically):** Not reported.  **Rationale for done scenario:** NA..  **Reason for changing mind:** NA. | **Method to detect interactions reported in protocol:** Not reported.  **Method to detect interactions reported in review:** Not reported.  **Reason for changing mind:** NA. |
| Chaparro 2013 | **Protocol:** Not reported.  **Review methods:** Not reported.  **Review results: ‘**Drug administration >24 hours’; ‘Drug administration ≤ 24 hours’.  **Type:** Intervention  **Covariate summary:** Timing of intervention. | **Method to choose**  **covariate:** Not reported.  **Where method reported:** NA.  **Rationale for choosing the covariate:** Not reported.  **Where rationale reported:** NA. | **Where reported:** Review only.  **Reason not reported in review:** NA.  **Reason not reported in protocol:** Not reported.  **Labelled as ‘posthoc’ if not in protocol:** No. | **Data type:** Continuous.  **Continuous covariate categorised:** Yes.  **How determine it was categorised:** Categories given.  **Justification for categorising:** Not reported.  **Where justification reported:** NA.  **Categories reported:** Yes.  **Where categories reported:** Review only.  **Justification for categories:** Not reported.  **Where justification reported:** NA. | **Analysis type reported in protocol:** Not reported.  **Analysis type reported in review:** Unnamed analysis.  **Reason for changing mind:** Not reported.    **Analysis type in protocol according to glossary:** Not reported.  **How determined the analysis type:** NA.  **Analysis type in review according to glossary:** Subgroup analysis.  **How determined the analysis type:** Results reported.  **Analysed:** Yes.  **Reasons given (in the review) for not doing interaction analysis if planned:** NA.  **Reasons given for carrying out interaction analysis if they weren’t planned:** Not reported.  **Scenario in protocol:** Not reported.  **Rationale for planned scenario:** NA.  **Scenario in review (if reported specifically):** Not reported.  **Rationale for done scenario:** NA..  **Reason for changing mind:** NA. | **Method to detect interactions reported in protocol:** Not reported.  **Method to detect interactions reported in review:** Not reported.  **Reason for changing mind:** NA. |
| Chaparro 2013 | **Protocol:** Not reported.  **Review methods:** Not reported.  **Review results:** Pain at 3 months, 4 months, 6 months.  **Type:** Outcome.  **Covariate summary:** Time point. | **Method to choose**  **covariate:** Not reported.  **Where method reported:** NA.  **Rationale for choosing the covariate:** Not reported.  **Where rationale reported:** NA. | **Where reported:** Review only.  **Reason not reported in review:** NA.  **Reason not reported in protocol:** Not reported.  **Labelled as ‘posthoc’ if not in protocol:** No. | **Data type:** Continuous.  **Continuous covariate categorised:** Yes.  **How determine it was categorised:** Categories given.  **Justification for categorising:** Not reported.  **Where justification reported:** NA.  **Categories reported:** Yes.  **Where categories reported:** Review only.  **Justification for categories:** Not reported.  **Where justification reported:** NA. | **Analysis type reported in protocol:** Not reported.  **Analysis type reported in review:** Unnamed analysis.  **Reason for changing mind:** Not reported.    **Analysis type in protocol according to glossary:** Not reported.  **How determined the analysis type:** NA.  **Analysis type in review according to glossary:** Subgroup analysis.  **How determined the analysis type:** Results reported.  **Analysed:** Yes.  **Reasons given (in the review) for not doing interaction analysis if planned:** NA.  **Reasons given for carrying out interaction analysis if they weren’t planned:** Not reported.  **Scenario in protocol:** Not reported.  **Rationale for planned scenario:** NA.  **Scenario in review (if reported specifically):** Not reported.  **Rationale for done scenario:** NA..  **Reason for changing mind:** NA. | **Method to detect interactions reported in protocol:** Not reported.  **Method to detect interactions reported in review:** Not reported.  **Reason for changing mind:** NA. |
| Chaparro 2013 | **Protocol:** Pain measurement scales .We judged the following to be four covariates: ‘Sensitivity analyses will be used to evaluate the robustness of a particular result by repeating primary analyses without studies considered to be outliers with respect to study quality, drug dose/duration or pain measurement scales.’  **Review methods:** Pain measurement scales We judged the following to be four covariates: ‘Sensitivity analyses were used to evaluate the robustness of a particular result by repeating primary analyses without any studies considered to be outliers with respect to study quality, drug dose and duration, or pain measurement scales.’  **Review results:** Not analysed.  **Type:** Outcome.  **Covariate summary:** Measurement scale. | **Method to choose**  **covariate:** Not reported.  **Where method reported:** NA.  **Rationale for choosing the covariate:** Not reported.  **Where rationale reported:** NA. | **Where reported:** Protocol and review.  **Reason not reported in review:** NA.  **Reason not reported in protocol:** NA.  **Labelled as ‘posthoc’ if not in protocol:** NA. | **Data type:** Unclear.  **Continuous covariate categorised:** Not reported.  **How determine it was categorised:** NA.  **Justification for categorising:** Not reported.  **Where justification reported:** NA.  **Categories reported:** Not reported.  **Where categories reported:** NA.  **Justification for categories:** Not reported.  **Where justification reported:** NA. | **Analysis type reported in protocol:** Sensitivity analysis.  **Analysis type reported in review:** Sensitivity analysis.  **Reason for changing mind:** NA.    **Analysis type in protocol according to glossary:** Sensitivity analysis.  **How determined the analysis type:** Says ‘sensitivity analysis’. No reason to dispute.  **Analysis type in review according to glossary:** Sensitivity analysis.  **How determined the analysis type:** Says ‘sensitivity analysis’. No reason to dispute.  **Analysed:** No.  **Reasons given (in the review) for not doing interaction analysis if planned:** Not reported.  **Reasons given for carrying out interaction analysis if they weren’t planned:** NA.  **Scenario in protocol:** Not reported.  **Rationale for planned scenario:** NA.  **Scenario in review (if reported specifically):** Not reported.  **Rationale for done scenario:** NA..  **Reason for changing mind:** NA. | **Method to detect interactions reported in protocol:** Not reported.  **Method to detect interactions reported in review:** Not reported.  **Reason for changing mind:** NA. |
| Chaparro 2013 | **Protocol:** Study quality. We judged the following to be four covariates: ‘Sensitivity analyses will be used to evaluate the robustness of a particular result by repeating primary analyses without studies considered to be outliers with respect to study quality, drug dose/duration or pain measurement scales.’  **Review methods:** Study quality. We judged the following to be four covariates: ‘Sensitivity analyses were used to evaluate the robustness of a particular result by repeating primary analyses without any studies considered to be outliers with respect to study quality, drug dose and duration, or pain measurement scales.’  **Review results:** Not analysed.  **Type:** Methodological.  **Covariate summary:** Quality. | **Method to choose**  **covariate:** Not reported.  **Where method reported:** NA.  **Rationale for choosing the covariate:** Not reported.  **Where rationale reported:** NA. | **Where reported:** Protocol and review.  **Reason not reported in review:** NA.  **Reason not reported in protocol:** NA.  **Labelled as ‘posthoc’ if not in protocol:** NA. | **Data type:** Categorical.  **Continuous covariate categorised:** NA.  **How determine it was categorised:** NA.  **Justification for categorising:** NA.  **Where justification reported:** NA.  **Categories reported:** Not reported.  **Where categories reported:** NA.  **Justification for categories:** Not reported.  **Where justification reported:** NA. | **Analysis type reported in protocol:** Sensitivity analysis.  **Analysis type reported in review:** Sensitivity analysis.  **Reason for changing mind:** NA.    **Analysis type in protocol according to glossary:** Sensitivity analysis.  **How determined the analysis type:** Says ‘sensitivity analysis’. No reason to dispute.  **Analysis type in review according to glossary:** Sensitivity analysis.  **How determined the analysis type:** Says ‘sensitivity analysis’. No reason to dispute.  **Analysed:** No.  **Reasons given (in the review) for not doing interaction analysis if planned:** Not reported.  **Reasons given for carrying out interaction analysis if they weren’t planned:** NA.  **Scenario in protocol:** Not reported.  **Rationale for planned scenario:** NA.  **Scenario in review (if reported specifically):** Not reported.  **Rationale for done scenario:** NA..  **Reason for changing mind:** NA. | **Method to detect interactions reported in protocol:** Not reported.  **Method to detect interactions reported in review:** Not reported.  **Reason for changing mind:** NA. |
| Cheng 2013 | **Protocol:** ‘Excluding non-English literatures’.  **Review methods: ‘**Excluding non-English literatures’.  **Review results:** Not analysed.  **Type:** Methodological.  **Covariate summary:** Publication language. | **Method to choose**  **covariate:** Not reported.  **Where method reported:** NA.  **Rationale for choosing the covariate:** Not reported.  **Where rationale reported:** NA. | **Where reported:** Protocol and review.  **Reason not reported in review:** NA.  **Reason not reported in protocol:** NA.  **Labelled as ‘posthoc’ if not in protocol:** NA. | **Data type:** Categorical.  **Continuous covariate categorised:** NA.  **How determine it was categorised:** NA.  **Justification for categorising:** NA.  **Where justification reported:** NA.  **Categories reported:** Yes.  **Where categories reported:** Protocol and review.  **Justification for categories:** Not reported.  **Where justification reported:** NA. | **Analysis type reported in protocol:** Sensitivity analysis.  **Analysis type reported in review:** Sensitivity analysis.  **Reason for changing mind:** NA.    **Analysis type in protocol according to glossary:** Sensitivity analysis.  **How determined the analysis type:** Categories given.  **Analysis type in review according to glossary:** Sensitivity analysis.  **How determined the analysis type:** Categories given.  **Analysed:** No.  **Reasons given (in the review) for not doing interaction analysis if planned:** ‘We  did not perform the other planned sensitivity analyses because  none of the trials was of low risk of bias and because none was  published in non-English literatures.’  **Reasons given for carrying out interaction analysis if they weren’t planned:** NA.  **Scenario in protocol:** Not reported.  **Rationale for planned scenario:** NA..  **Scenario in review (if reported specifically):** Not reported.  **Rationale for done scenario:** NA.  **Reason for changing mind:** NA**.** | **Method to detect interactions reported in protocol: ‘**If the results do not change, they are considered to have low sensitivity.  If the results change, they are considered to have high sensitivity.’.  **Method to detect interactions reported in review:** ‘If the results did not change, they were considered to have low  sensitivity. If the results changed, they were considered to have  high sensitivity.’  **Reason for changing mind:** NA. |
| Cheng 2013 | **Protocol:** ‘Excluding RCTs with low quality’.  **Review methods: ‘**Excluding randomised controlled trials with low quality’.  **Review results:** Not analysed.  **Type:** Methodological.  **Covariate summary:** Quality. | **Method to choose**  **covariate:** Not reported.  **Where method reported:** NA.  **Rationale for choosing the covariate:** Not reported.  **Where rationale reported:** NA. | **Where reported:** Protocol and review.  **Reason not reported in review:** NA.  **Reason not reported in protocol:** NA.  **Labelled as ‘posthoc’ if not in protocol:** NA. | **Data type:** Categorical.  **Continuous covariate categorised:** NA.  **How determine it was categorised:** NA.  **Justification for categorising:** NA.  **Where justification reported:** NA.  **Categories reported:** Yes.  **Where categories reported:** Protocol and review.  **Justification for categories:** Not reported.  **Where justification reported:** NA. | **Analysis type reported in protocol:** Sensitivity analysis.  **Analysis type reported in review:** Sensitivity analysis.  **Reason for changing mind:** NA.    **Analysis type in protocol according to glossary:** Sensitivity analysis.  **How determined the analysis type:** Categories given.  **Analysis type in review according to glossary:** Sensitivity analysis.  **How determined the analysis type:** Categories given.  **Analysed:** No.  **Reasons given (in the review) for not doing interaction analysis if planned:** ‘We  did not perform the other planned sensitivity analyses because  none of the trials was of low risk of bias and because none was  published in non-English literatures.’  **Reasons given for carrying out interaction analysis if they weren’t planned:** NA.  **Scenario in protocol:** Not reported.  **Rationale for planned scenario:** NA..  **Scenario in review (if reported specifically):** Not reported.  **Rationale for done scenario:** NA.  **Reason for changing mind:** NA**.** | **Method to detect interactions reported in protocol: ‘**If the results do not change, they are considered to have low sensitivity.  If the results change, they are considered to have high sensitivity.’.  **Method to detect interactions reported in review:** ‘If the results did not change, they were considered to have low  sensitivity. If the results changed, they were considered to have  high sensitivity.’  **Reason for changing mind:** NA. |
| Cheng 2013 | **Protocol:** ‘High risk patients (e. g. patients with cardiopulmonary disease; American Society of Anesthesiologists (ASA) III  or IV) versus low risk patients (e. g. patients without cardiopulmonary disease; American Society of Anesthesiologists(ASA) I Or II).’  **Review methods: ‘**High anaesthetic risk patients (e. g. patients with cardiopulmonary disease; American Society of Anesthesiologists (ASA) iii or iv) versus low risk patients (e. g. patients without cardiopulmonary disease; American Society of Anesthesiologists (ASA) I or ii).’  **Review results:** Not analysed.  **Type:** Patient.  **Covariate summary:** Disease characteristics. | **Method to choose**  **covariate:** Not reported.  **Where method reported:** NA.  **Rationale for choosing the covariate:** Not reported.  **Where rationale reported:** NA. | **Where reported:** Protocol and review.  **Reason not reported in review:** NA.  **Reason not reported in protocol:** NA.  **Labelled as ‘posthoc’ if not in protocol:** NA. | **Data type:** Categorical.  **Continuous covariate categorised:** NA.  **How determine it was categorised:** NA.  **Justification for categorising:** NA.  **Where justification reported:** NA.  **Categories reported:** Yes.  **Where categories reported:** Protocol and review.  **Justification for categories:** Not reported.  **Where justification reported:** NA. | **Analysis type reported in protocol:** Subgroup analysis.  **Analysis type reported in review:** Subgroup analysis.  **Reason for changing mind:** NA.    **Analysis type in protocol according to glossary:** Subgroup analysis.  **How determined the analysis type:** Categories given.  **Analysis type in review according to glossary:** Subgroup analysis.  **How determined the analysis type:** Categories given.  **Analysed:** No.  **Reasons given (in the review) for not doing interaction analysis if planned:** ‘None of the other planned subgroup analyses was  performed because of the few trials included under each outcome.’  **Reasons given for carrying out interaction analysis if they weren’t planned:** NA.  **Scenario in protocol:** ‘If there is a significant heterogeneity among the RCTs, we plan  the following subgroup analyses:’  **Rationale for planned scenario:** Not reported.  **Scenario in review (if reported specifically):** Not reported.  **Rationale for done scenario:** NA.  **Reason for changing mind:** NA**.** | **Method to detect interactions reported in protocol:** Not reported.  **Method to detect interactions reported in review:** Not reported.  **Reason for changing mind:** NA. |
| Cheng 2013 | **Protocol:** ‘The type of operation (laparoscopic surgery of stomach, gallbladder, liver, pancreas, spleen, intestine, kidney, etc).’  **Review methods:** Not reported.  **Review results:** Not analysed.  **Type:** Intervention.  **Covariate summary:** Type of intervention or control. | **Method to choose**  **covariate:** Not reported.  **Where method reported:** NA.  **Rationale for choosing the covariate:** Not reported.  **Where rationale reported:** NA. | **Where reported:** Protocol only.  **Reason not reported in review:** Not reported.  **Reason not reported in protocol:** NA.  **Labelled as ‘posthoc’ if not in protocol:** NA. | **Data type:** Categorical.  **Continuous covariate categorised:** NA.  **How determine it was categorised:** NA.  **Justification for categorising:** NA.  **Where justification reported:** NA.  **Categories reported:** Yes.  **Where categories reported:** Protocol only.  **Justification for categories:** Not reported.  **Where justification reported:** NA. | **Analysis type reported in protocol:** Subgroup analysis.  **Analysis type reported in review:** Not reported.  **Reason for changing mind:** Not reported.    **Analysis type in protocol according to glossary:** Subgroup analysis.  **How determined the analysis type:** Categories given.  **Analysis type in review according to glossary:** Not reported.  **How determined the analysis type:** NA.  **Analysed:** No.  **Reasons given (in the review) for not doing interaction analysis if planned:** ‘None of the other planned subgroup analyses was  performed because of the few trials included under each outcome.’  **Reasons given for carrying out interaction analysis if they weren’t planned:** NA.  **Scenario in protocol:** ‘If there is a significant heterogeneity among the RCTs, we plan  the following subgroup analyses:’  **Rationale for planned scenario:** Not reported.  **Scenario in review (if reported specifically):** Not reported.  **Rationale for done scenario:** NA.  **Reason for changing mind:** NA**.** | **Method to detect interactions reported in protocol:** Not reported.  **Method to detect interactions reported in review:** Not reported.  **Reason for changing mind:** NA. |
| Cheng 2013 | **Protocol:** ‘Trials with low risk of bias versus trials with high risk of bias.’  **Review methods: ‘**Trials with low risk of bias versus trials with high risk of bias.’  **Review results:** Not analysed.  **Type:** Methodological.  **Covariate summary:** Risk of bias. | **Method to choose**  **covariate:** Not reported.  **Where method reported:** NA.  **Rationale for choosing the covariate:** Not reported.  **Where rationale reported:** NA. | **Where reported:** Protocol and review.  **Reason not reported in review:** NA.  **Reason not reported in protocol:** NA.  **Labelled as ‘posthoc’ if not in protocol:** NA. | **Data type:** Categorical.  **Continuous covariate categorised:** NA.  **How determine it was categorised:** NA.  **Justification for categorising:** NA.  **Where justification reported:** NA.  **Categories reported:** Yes.  **Where categories reported:** Protocol and review.  **Justification for categories:** Not reported.  **Where justification reported:** NA. | **Analysis type reported in protocol:** Subgroup analysis.  **Analysis type reported in review:** Subgroup analysis.  **Reason for changing mind:** NA.    **Analysis type in protocol according to glossary:** Subgroup analysis.  **How determined the analysis type:** Categories given.  **Analysis type in review according to glossary:** Subgroup analysis.  **How determined the analysis type:** Categories given.  **Analysed:** No.  **Reasons given (in the review) for not doing interaction analysis if planned:** ‘None of the trials was of low risk of  bias. Thus, we did not perform a subgroup analysis stratified by  the risk of bias.’  **Reasons given for carrying out interaction analysis if they weren’t planned:** NA.  **Scenario in protocol:** ‘If there is a significant heterogeneity among the RCTs, we plan  the following subgroup analyses:’  **Rationale for planned scenario:** Not reported.  **Scenario in review (if reported specifically):** Not reported.  **Rationale for done scenario:** NA.  **Reason for changing mind:** NA**.** | **Method to detect interactions reported in protocol:** Not reported.  **Method to detect interactions reported in review:** Not reported.  **Reason for changing mind:** NA. |
| Cheng 2013 | **Protocol:** ‘We will analysis the following gases for establishing pneumoperitoneum: 1. Carbon dioxide versus nitrous oxide.  2. Carbon dioxide versus helium. 3. Carbon dioxide versus argon. 4. Carbon dioxide versus nitrogen. 5. Carbon dioxide versus any other gas. 6. Any other gas (except carbon dioxide) versus any other gas (except carbon dioxide).’  **Review methods: ‘**We will planned to assess the following gases for establishing pneumoperitoneum: 1. Carbon dioxide versus nitrous oxide. 2. Carbon dioxide versus helium. 3. Carbon dioxide versus argon. 4. Carbon dioxide versus nitrogen. 5. Carbon dioxide versus any other gas. 6. Any other gas (except carbon dioxide) versus any other gas (except carbon dioxide).  **Review results:** ‘Carbon dioxide pneumoperitoneum versus nitrous oxide pneumoperitoneum’; ‘Carbon dioxide pneumoperitoneum versus helium Pneumoperitoneum.’  **Type:** Intervention.  **Covariate summary:** Type of intervention or control. | **Method to choose**  **covariate:** Not reported.  **Where method reported:** NA.  **Rationale for choosing the covariate:** Not reported.  **Where rationale reported:** NA. | **Where reported:** Protocol and review.  **Reason not reported in review:** NA.  **Reason not reported in protocol:** NA.  **Labelled as ‘posthoc’ if not in protocol:** NA. | **Data type:** Categorical.  **Continuous covariate categorised:** NA.  **How determine it was categorised:** NA.  **Justification for categorising:** NA.  **Where justification reported:** NA.  **Categories reported:** Yes.  **Where categories reported:** Protocol and review.  **Justification for categories:** Not reported.  **Where justification reported:** NA. | **Analysis type reported in protocol:** Unnamed analysis.  **Analysis type reported in review:** unnamed analysis.  **Reason for changing mind:** NA.    **Analysis type in protocol according to glossary:** Stratification/subgroup analysis.  **How determined the analysis type:** Categories given.  **Analysis type in review according to glossary:** Stratification/subgroup analysis.  **How determined the analysis type:** Categories given.  **Analysed:** Yes.  **Reasons given (in the review) for not doing interaction analysis if planned:** NA.  **Reasons given for carrying out interaction analysis if they weren’t planned:** NA.  **Scenario in protocol:** ‘If there is a significant heterogeneity among the RCTs, we plan  the following subgroup analyses:’  **Rationale for planned scenario:** Not reported.  **Scenario in review (if reported specifically):** Not reported.  **Rationale for done scenario:** NA.  **Reason for changing mind:** NA**.** | **Method to detect interactions reported in protocol:** Not reported.  **Method to detect interactions reported in review:** Not reported.  **Reason for changing mind:** NA. |
| Cheng 2013 | **Protocol:** Large sample size. We judged the following to be two covariates: ‘Excluding RCTs with either small or large sample sizes.’  **Review methods:** Large sample size. We judged the following to be two covariates: ‘Excluding randomised controlled trials with either small or large sample sizes.’  **Review results:** Not analysed.  **Type:** Methodological.  **Covariate summary:** Trial size. | **Method to choose**  **covariate:** Not reported.  **Where method reported:** NA.  **Rationale for choosing the covariate:** Not reported.  **Where rationale reported:** NA. | **Where reported:** Protocol and review.  **Reason not reported in review:** NA.  **Reason not reported in protocol:** NA.  **Labelled as ‘posthoc’ if not in protocol:** NA. | **Data type:** Continuous.  **Continuous covariate categorised:** Yes.  **How determine it was categorised:** Categories given.  **Justification for categorising:** Not reported.  **Where justification reported:** NA.  **Categories reported:** Yes.  **Where categories reported:** Protocol and review.  **Justification for categories:** Not reported.  **Where justification reported:** NA. | **Analysis type reported in protocol:** Sensitivity analysis.  **Analysis type reported in review:** Sensitivity analysis.  **Reason for changing mind:** NA.    **Analysis type in protocol according to glossary:** Sensitivity analysis.  **How determined the analysis type:** Categories given.  **Analysis type in review according to glossary:** Sensitivity analysis.  **How determined the analysis type:** Categories given.  **Analysed:** No.  **Reasons given (in the review) for not doing interaction analysis if planned:** ‘We  did not perform the other planned sensitivity analyses because  none of the trials was of low risk of bias and because none was  published in non-English literatures.’  **Reasons given for carrying out interaction analysis if they weren’t planned:** NA.  **Scenario in protocol:** Not reported.  **Rationale for planned scenario:** NA..  **Scenario in review (if reported specifically):** Not reported.  **Rationale for done scenario:** NA.  **Reason for changing mind:** NA**.** | **Method to detect interactions reported in protocol: ‘**If the results do not change, they are considered to have low sensitivity.  If the results change, they are considered to have high sensitivity.’.  **Method to detect interactions reported in review:** ‘If the results did not change, they were considered to have low  sensitivity. If the results changed, they were considered to have  high sensitivity.’  **Reason for changing mind:** NA. |
| Cheng 2013 | **Protocol:** Not reported.  **Review methods: ‘**Abdominal surgery versus pelvic surgery’.  **Review results:** Not analysed.  **Type:** Intervention.  **Covariate summary:** Type of intervention or control. | **Method to choose**  **covariate:** Not reported.  **Where method reported:** NA.  **Rationale for choosing the covariate:** Not reported.  **Where rationale reported:** NA. | **Where reported:** Review only.  **Reason not reported in review:** NA.  **Reason not reported in protocol:** Not reported.  **Labelled as ‘posthoc’ if not in protocol:** Not reported. | **Data type:** Categorical.  **Continuous covariate categorised:** NA.  **How determine it was categorised:** NA.  **Justification for categorising:** NA.  **Where justification reported:** NA.  **Categories reported:** Yes.  **Where categories reported:** Review only.  **Justification for categories:** Not reported.  **Where justification reported:** NA. | **Analysis type reported in protocol:** Not reported.  **Analysis type reported in review:** Subgroup analysis.  **Reason for changing mind:** Not reported.    **Analysis type in protocol according to glossary:** Not reported.  **How determined the analysis type:** NA.  **Analysis type in review according to glossary:** Subgroup analysis.  **How determined the analysis type:** Categories given.  **Analysed:** No.  **Reasons given (in the review) for not doing interaction analysis if planned:** Not reported.  **Reasons given for carrying out interaction analysis if they weren’t planned:** NA.  **Scenario in protocol:** Not reported.  **Rationale for planned scenario:** NA.  **Scenario in review (if reported specifically):** Not reported.  **Rationale for done scenario:** NA.  **Reason for changing mind:** NA**.** | **Method to detect interactions reported in protocol:** Not reported.  **Method to detect interactions reported in review:** Not reported.  **Reason for changing mind:** NA. |
| Cheng 2013 | **Protocol:** Not reported.  **Review methods: ‘**Elective procedure versus emergency procedure’.  **Review results:** Not analysed.  **Type:** Intervention.  **Covariate summary:** Type of intervention or control. | **Method to choose**  **covariate:** Not reported.  **Where method reported:** NA.  **Rationale for choosing the covariate:** Not reported.  **Where rationale reported:** NA. | **Where reported:** Review only.  **Reason not reported in review:** NA.  **Reason not reported in protocol:** Not reported.  **Labelled as ‘posthoc’ if not in protocol:** Not reported. | **Data type:** Categorical.  **Continuous covariate categorised:** NA.  **How determine it was categorised:** NA.  **Justification for categorising:** NA.  **Where justification reported:** NA.  **Categories reported:** Yes.  **Where categories reported:** Review only.  **Justification for categories:** Not reported.  **Where justification reported:** NA. | **Analysis type reported in protocol:** Not reported.  **Analysis type reported in review:** Subgroup analysis.  **Reason for changing mind:** Not reported.    **Analysis type in protocol according to glossary:** Not reported.  **How determined the analysis type:** NA.  **Analysis type in review according to glossary:** Subgroup analysis.  **How determined the analysis type:** Categories given.  **Analysed:** No.  **Reasons given (in the review) for not doing interaction analysis if planned:** ‘None of the other planned subgroup analyses was  performed because of the few trials included under each outcome.’  **Reasons given for carrying out interaction analysis if they weren’t planned:** NA.  **Scenario in protocol:** Not reported.  **Rationale for planned scenario:** NA.  **Scenario in review (if reported specifically):** Not reported.  **Rationale for done scenario:** NA.  **Reason for changing mind:** NA**.** | **Method to detect interactions reported in protocol:** Not reported.  **Method to detect interactions reported in review:** Not reported.  **Reason for changing mind:** NA. |
| Cheng 2013 | **Protocol:** Small sample size. We judged the following to be two covariates: ‘Excluding RCTs with either small or large sample sizes.’  **Review methods:** Small sample size. We judged the following to be two covariates: ‘Excluding randomised controlled trials with either small or large sample sizes.’  **Review results:** Not analysed.  **Type:** Methodological.  **Covariate summary:** Trial size. | **Method to choose**  **covariate:** Not reported.  **Where method reported:** NA.  **Rationale for choosing the covariate:** Not reported.  **Where rationale reported:** NA. | **Where reported:** Protocol and review.  **Reason not reported in review:** NA.  **Reason not reported in protocol:** NA.  **Labelled as ‘posthoc’ if not in protocol:** NA. | **Data type:** Continuous.  **Continuous covariate categorised:** Yes.  **How determine it was categorised:** Categories given.  **Justification for categorising:** Not reported.  **Where justification reported:** NA.  **Categories reported:** Yes.  **Where categories reported:** Protocol and review.  **Justification for categories:** Not reported.  **Where justification reported:** NA. | **Analysis type reported in protocol:** Sensitivity analysis.  **Analysis type reported in review:** Sensitivity analysis.  **Reason for changing mind:** NA.    **Analysis type in protocol according to glossary:** Sensitivity analysis.  **How determined the analysis type:** Categories given.  **Analysis type in review according to glossary:** Sensitivity analysis.  **How determined the analysis type:** Categories given.  **Analysed:** No.  **Reasons given (in the review) for not doing interaction analysis if planned:** ‘We  did not perform the other planned sensitivity analyses because  none of the trials was of low risk of bias and because none was  published in non-English literatures.’  **Reasons given for carrying out interaction analysis if they weren’t planned:** NA.  **Scenario in protocol:** Not reported.  **Rationale for planned scenario:** NA..  **Scenario in review (if reported specifically):** Not reported.  **Rationale for done scenario:** NA.  **Reason for changing mind:** NA**.** | **Method to detect interactions reported in protocol: ‘**If the results do not change, they are considered to have low sensitivity.  If the results change, they are considered to have high sensitivity.’.  **Method to detect interactions reported in review:** ‘If the results did not change, they were considered to have low  sensitivity. If the results changed, they were considered to have  high sensitivity.’  **Reason for changing mind:** NA. |
| Cruciani 2013 | **Protocol:** ‘Exclusion of studies with relevant mutations at baseline’.  **Review methods**: Not reported.  **Review results:** Not analysed.  **Type:** Patient.  **Covariate summary:** Disease characteristics. | **Method to choose**  **covariate:** Not reported.  **Where method reported:** NA.  **Rationale for choosing the covariate:** Not reported.  **Where rationale reported:** NA. | **Where reported:** Protocol only.  **Reason not reported in review:** Not reported.  **Reason not reported in protocol:** NA.  **Labelled as ‘posthoc’ if not in protocol:** NA**.** | **Data type:** Categorical.  **Continuous covariate categorised:** NA.  **How determine it was categorised:** NA.  **Justification for categorising:** NA.  **Where justification reported:** NA.  **Categories reported:** Not reported.  **Where categories reported:** NA.  **Justification for categories:** Not reported.  **Where justification reported:** NA. | **Analysis type reported in protocol:** Subgroup analysis.  **Analysis type reported in review:** Not reported.  **Reason for changing mind:** Not reported.    **Analysis type in protocol according to glossary:** Subgroup analysis.  **How determined the analysis type:** Says ‘subgroup analysis’. No reason to dispute.  **Analysis type in review according to glossary:** Not reported.  **How determined the analysis type:** NA.  **Analysed:** No.  **Reasons given (in the review) for not doing interaction analysis if planned:** Not reported.  **Reasons given for carrying out interaction analysis if they weren’t planned:** NA.  **Scenario in protocol:** Not reported.  **Rationale for planned scenario:** NA.  **Scenario in review (if reported specifically):** Not reported.  **Rationale for done scenario:** NA.  **Reason for changing mind:** NA. | **Method to detect interactions reported in protocol:** Not reported.  **Method to detect interactions reported in review:** Not reported.  **Reason for changing mind:** NA. |
| Cruciani 2013 | **Protocol:** ‘If data are available on outcome measures at two or more periods, they will be analyzed according to the different period of time.’  **Review methods:** ‘If data were available on outcome measures at two or more periods, they will be analyzed according to the different period of time.’  **Review results:** Not analysed.  **Type:** Outcome.  **Covariate summary:** Time point. | **Method to choose**  **covariate:** Not reported.  **Where method reported:** NA.  **Rationale for choosing the covariate:** Not reported.  **Where rationale reported:** NA. | **Where reported:** Protocol and review.  **Reason not reported in review:** NA.  **Reason not reported in protocol:** NA.  **Labelled as ‘posthoc’ if not in protocol:** NA**.** | **Data type:** Continuous.  **Continuous covariate categorised:** Unclear.  **How determine it was categorised:** NA.  **Justification for categorising:** NA.  **Where justification reported:** NA.  **Categories reported:** Not reported.  **Where categories reported:** NA.  **Justification for categories:** Not reported.  **Where justification reported:** NA. | **Analysis type reported in protocol:** Unnamed analysis.  **Analysis type reported in review:** Unnamed analysis.  **Reason for changing mind:** NA.    **Analysis type in protocol according to glossary:** Unclear.  **How determined the analysis type:** No categories given  **Analysis type in review according to glossary:** Unclear.  **How determined the analysis type:** No categories given.  **Analysed:** No.  **Reasons given (in the review) for not doing interaction analysis if planned:** Not reported.  **Reasons given for carrying out interaction analysis if they weren’t planned:** NA.  **Scenario in protocol:** ‘f data are available on outcome measures at two or more periods,’  **Rationale for planned scenario:** NA.  **Scenario in review (if reported specifically):** ‘f data are available on outcome measures at two or more periods,’  **Rationale for done scenario:** NA.  **Reason for changing mind:** Not reported. | **Method to detect interactions reported in protocol:** Not reported.  **Method to detect interactions reported in review:** Not reported.  **Reason for changing mind:** NA. |
| Cruciani 2013 | **Protocol:** ‘The adequacy of allocation concealment’.  **Review methods: ‘**The adequacy of allocation concealment’.  **Review results: ‘**The adequacy of allocation concealment’.  **Type:** Methodological.  **Covariate summary:** Allocation concealment. | **Method to choose**  **covariate:** Not reported.  **Where method reported:** NA.  **Rationale for choosing the covariate:** Not reported.  **Where rationale reported:** NA. | **Where reported:** Protocol and review.  **Reason not reported in review:** NA.  **Reason not reported in protocol:** NA.  **Labelled as ‘posthoc’ if not in protocol:** NA**.** | **Data type:** Categorical.  **Continuous covariate categorised:** NA.  **How determine it was categorised:** NA.  **Justification for categorising:** NA.  **Where justification reported:** NA.  **Categories reported:** Yes.  **Where categories reported:** Protocol and review.  **Justification for categories:** Not reported.  **Where justification reported:** NA. | **Analysis type reported in protocol:** Sensitivity analysis.  **Analysis type reported in review:** Sensitivity analysis.  **Reason for changing mind:** NA.    **Analysis type in protocol according to glossary:** Sensitivity analysis.  **How determined the analysis type:** Says ‘sensitivity analysis.’ No reason to dispute.  **Analysis type in review according to glossary:** Subgroup analysis.  **How determined the analysis type:** Results presented.  **Analysed:** Yes.  **Reasons given (in the review) for not doing interaction analysis if planned:** NA.  **Reasons given for carrying out interaction analysis if they weren’t planned:** NA.  **Scenario in protocol:** ‘Data permitting’.  **Rationale for planned scenario:** NA.  **Scenario in review (if reported specifically):** Not reported.  **Rationale for done scenario:** NA.  **Reason for changing mind:** Not reported. | **Method to detect interactions reported in protocol:** Not reported.  **Method to detect interactions reported in review:** Not reported.  **Reason for changing mind:** NA. |
| Cruciani 2013 | **Protocol: ‘**The type of drugs in the simplification regimen (e. g. nevirapine, efavirenz)’.  **Review methods:** ‘Type of drugs in the control group (e. g. nevirapine, efavirenz)’.  **Review results:** ‘Type NNRTI drug in the simplication regimen’ (in text). ‘ABC vs Nevirapine’ and ‘ABC vs Efavirenz’ (in plot).  **Type:** Intervention.  **Covariate summary:** Type of intervention or control. | **Method to choose**  **covariate:** Not reported.  **Where method reported:** NA.  **Rationale for choosing the covariate:** Not reported.  **Where rationale reported:** NA. | **Where reported:** Protocol and review.  **Reason not reported in review:** NA.  **Reason not reported in protocol:** NA.  **Labelled as ‘posthoc’ if not in protocol:** NA**.** | **Data type:** Categorical.  **Continuous covariate categorised:** NA.  **How determine it was categorised:** NA.  **Justification for categorising:** NA.  **Where justification reported:** NA.  **Categories reported:** Yes.  **Where categories reported:** Protocol and review.  **Justification for categories:** Not reported.  **Where justification reported:** NA. | **Analysis type reported in protocol:** Subgroup analysis.  **Analysis type reported in review:** Subgroup analysis.  **Reason for changing mind:** NA.    **Analysis type in protocol according to glossary:** Subgroup analysis.  **How determined the analysis type:** Categories given.  **Analysis type in review according to glossary:** Subgroup analysis.  **How determined the analysis type:** Categories given.  **Analysed:** Yes.  **Reasons given (in the review) for not doing interaction analysis if planned:** NA.  **Reasons given for carrying out interaction analysis if they weren’t planned:** NA.  **Scenario in protocol:** Not reported.  **Rationale for planned scenario:** NA.  **Scenario in review (if reported specifically):** Not reported.  **Rationale for done scenario:** NA.  **Reason for changing mind:** NA. | **Method to detect interactions reported in protocol:** Not reported.  **Method to detect interactions reported in review:** Not reported.  **Reason for changing mind:** NA. |
| Cruciani 2013 | **Protocol:** ‘Threshold of undetectable viral load’.  **Review methods:** Not reported.  **Review results:** Not analysed.  **Type:** Patient.  **Covariate summary:** Disease characteristics. | **Method to choose**  **covariate:** Not reported.  **Where method reported:** NA.  **Rationale for choosing the covariate:** Not reported.  **Where rationale reported:** NA. | **Where reported:** Protocol only.  **Reason not reported in review:** Not reported.  **Reason not reported in protocol:** NA.  **Labelled as ‘posthoc’ if not in protocol:** NA**.** | **Data type:** Continuous.  **Continuous covariate categorised:** Yes.  **How determine it was categorised:** Subgroup analysis planned.  **Justification for categorising:** Not reported.  **Where justification reported:** NA.  **Categories reported:** Not reported.  **Where categories reported:** NA.  **Justification for categories:** Not reported.  **Where justification reported:** NA. | **Analysis type reported in protocol:** Subgroup analysis.  **Analysis type reported in review:** Not reported.  **Reason for changing mind:** Not reported.    **Analysis type in protocol according to glossary:** Subgroup analysis.  **How determined the analysis type:** Says ‘subgroup analysis’. No reason to dispute.  **Analysis type in review according to glossary:** Not reported.  **How determined the analysis type:** NA.  **Analysed:** No.  **Reasons given (in the review) for not doing interaction analysis if planned:** Not reported.  **Reasons given for carrying out interaction analysis if they weren’t planned:** NA.  **Scenario in protocol:** Not reported.  **Rationale for planned scenario:** NA.  **Scenario in review (if reported specifically):** Not reported.  **Rationale for done scenario:** NA.  **Reason for changing mind:** NA. | **Method to detect interactions reported in protocol:** Not reported.  **Method to detect interactions reported in review:** Not reported.  **Reason for changing mind:** NA. |
| Cruciani 2013 | **Protocol:** ‘Timing of simplification to nucleoside therapy (e. g. simplification in patients after a first antiretroviral PI-containing regimen and in antiretroviral-experienced participants).’  **Review methods: ‘**Timing of simplification to nucleoside therapy (e. g. simplification in patients after a first antiretroviral PI-containing regimen and in antiretroviral-experienced participants).’  **Review results: ‘**Timing of simplification to nucleoside therapy’.  **Type:** Intervention.  **Covariate summary:** Timing of intervention. | **Method to choose**  **covariate:** Not reported.  **Where method reported:** NA.  **Rationale for choosing the covariate:** Not reported.  **Where rationale reported:** NA. | **Where reported:** Protocol and review.  **Reason not reported in review:** NA.  **Reason not reported in protocol:** NA.  **Labelled as ‘posthoc’ if not in protocol:** NA**.** | **Data type:** Categorical.  **Continuous covariate categorised:** NA.  **How determine it was categorised:** NA.  **Justification for categorising:** NA.  **Where justification reported:** NA.  **Categories reported:** Yes.  **Where categories reported:** Protocol and review.  **Justification for categories:** Not reported.  **Where justification reported:** NA. | **Analysis type reported in protocol:** Subgroup analysis.  **Analysis type reported in review:** Subgroup analysis.  **Reason for changing mind:** NA.    **Analysis type in protocol according to glossary:** Subgroup analysis.  **How determined the analysis type:** Categories given.  **Analysis type in review according to glossary:** Subgroup analysis.  **How determined the analysis type:** Categories given.  **Analysed:** Yes.  **Reasons given (in the review) for not doing interaction analysis if planned:** NA.  **Reasons given for carrying out interaction analysis if they weren’t planned:** NA.  **Scenario in protocol:** Not reported.  **Rationale for planned scenario:** NA.  **Scenario in review (if reported specifically):** Not reported.  **Rationale for done scenario:** NA.  **Reason for changing mind:** NA. | **Method to detect interactions reported in protocol:** Not reported.  **Method to detect interactions reported in review:** Not reported.  **Reason for changing mind:** NA. |
| Cruciani 2013 | **Protocol:** Not reported.  **Review methods:** Not reported.  **Review results:** ‘ABC vs PI’; ‘ABC vs NNRTI’**.**  **Type:** Intervention.  **Covariate summary:** Type of intervention or control. | **Method to choose**  **covariate:** Not reported.  **Where method reported:** NA.  **Rationale for choosing the covariate:** Not reported.  **Where rationale reported:** NA. | **Where reported:** Review only.  **Reason not reported in review:** NA.  **Reason not reported in protocol:** Not reported.  **Labelled as ‘posthoc’ if not in protocol:** No**.** | **Data type:** Categorical.  **Continuous covariate categorised:** NA.  **How determine it was categorised:** NA.  **Justification for categorising:** NA.  **Where justification reported:** NA.  **Categories reported:** Yes.  **Where categories reported:** Review only.  **Justification for categories:** Not reported.  **Where justification reported:** NA. | **Analysis type reported in protocol:** Not reported.  **Analysis type reported in review:** Unnamed analysis.  **Reason for changing mind:** Not reported.    **Analysis type in protocol according to glossary:** Not reported.  **How determined the analysis type:** NA.  **Analysis type in review according to glossary:** Subgroup analysis.  **How determined the analysis type:** Categories given.  **Analysed:** Yes.  **Reasons given (in the review) for not doing interaction analysis if planned:** NA.  **Reasons given for carrying out interaction analysis if they weren’t planned:** Not reported.  **Scenario in protocol:** Not reported.  **Rationale for planned scenario:** NA.  **Scenario in review (if reported specifically):** Not reported.  **Rationale for done scenario:** NA.  **Reason for changing mind:** NA.. | **Method to detect interactions reported in protocol:** Not reported.  **Method to detect interactions reported in review:** Not reported.  **Reason for changing mind:** NA. |
| Dashash 2013 | **Protocol:** ‘Age of participants’.  **Review methods: ‘**Age of participants’.  **Review results:** Not analysed.  **Type:** Patient.  **Covariate summary:** Demographics. | **Method to choose**  **covariate:** Not reported.  **Where method reported:** NA.  **Rationale for choosing the covariate:** Not reported.  **Where rationale reported:** NA. | **Where reported:** Protocol and review.  **Reason not reported in review:** NA.  **Reason not reported in protocol:** NA.  **Labelled as ‘posthoc’ if not in protocol:** NA. | **Data type:** Continuous.  **Continuous covariate categorised:** Yes.  **How determine it was categorised:** Subgroup analysis planned.  **Justification for categorising:** No reported.  **Where justification reported:** NA.  **Categories reported:** Not reported.  **Where categories reported:** NA.  **Justification for categories:** Not reported.  **Where justification reported:** NA. | **Analysis type reported in protocol:** Subgroup analysis.  **Analysis type reported in review:** Subgroup analysis.  **Reason for changing mind:** NA.    **Analysis type in protocol according to glossary:** Subgroup analysis.  **How determined the analysis type:** ‘subgroup analysis’ no reason to dispute this.  **Analysis type in review according to glossary:** Subgroup analysis.  **How determined the analysis type:** ‘subgroup analysis’ no reason to dispute this.  **Analysed:** No.  **Reasons given (in the review) for not doing interaction analysis if planned:** Not reported (presumably because no trials).  **Reasons given for carrying out interaction analysis if they weren’t planned:** NA.  **Scenario in protocol: ‘**A planned subgroup analysis will be utilised to investigate some  potential factors for heterogeneity, which may affect outcomes.’  **Rationale for planned scenario:** Not reported.  **Scenario in review (if reported specifically): ‘**A planned subgroup analysis would be utilised to investigate some potential factors for heterogeneity, which may affect outcomes.’  **Rationale for done scenario:** Not reported.  **Reason for changing mind:** NA. | **Method to detect interactions reported in protocol:** Not reported.  **Method to detect interactions reported in review:** Not reported.  **Reason for changing mind:** NA. |
| Dashash 2013 | **Protocol:** ‘Difference in techniques applied before restorations (acid-etch technique, dentine adhesive systems)’.  **Review methods: ‘**Difference in techniques applied before restorations (acid-etch technique, dentine adhesive systems)’.  **Review results:** Not analysed.  **Type:** Intervention.  **Covariate summary:** Previous interventions. | **Method to choose**  **covariate:** Not reported.  **Where method reported:** NA.  **Rationale for choosing the covariate:** Not reported.  **Where rationale reported:** NA. | **Where reported:** Protocol and review.  **Reason not reported in review:** NA.  **Reason not reported in protocol:** NA.  **Labelled as ‘posthoc’ if not in protocol:** NA. | **Data type:** Categorical.  **Continuous covariate categorised:** NA.  **How determine it was categorised:** NA.  **Justification for categorising:** NA.  **Where justification reported:** NA.  **Categories reported:** Yes.  **Where categories reported:** Protocol and review.  **Justification for categories:** Not reported.  **Where justification reported:** NA. | **Analysis type reported in protocol:** Subgroup analysis.  **Analysis type reported in review:** Subgroup analysis.  **Reason for changing mind:** NA.    **Analysis type in protocol according to glossary:** Subgroup analysis.  **How determined the analysis type:** categories given.  **Analysis type in review according to glossary:** Subgroup analysis.  **How determined the analysis type:** categories given.  **Analysed:** No.  **Reasons given (in the review) for not doing interaction analysis if planned:** Not reported (presumably because no trials).  **Reasons given for carrying out interaction analysis if they weren’t planned:** NA.  **Scenario in protocol: ‘**A planned subgroup analysis will be utilised to investigate some  potential factors for heterogeneity, which may affect outcomes.’  **Rationale for planned scenario:** Not reported.  **Scenario in review (if reported specifically): ‘**A planned subgroup analysis would be utilised to investigate some potential factors for heterogeneity, which may affect outcomes.’  **Rationale for done scenario:** Not reported.  **Reason for changing mind:** NA. | **Method to detect interactions reported in protocol:** Not reported.  **Method to detect interactions reported in review:** Not reported.  **Reason for changing mind:** NA. |
| Dashash 2013 | **Protocol:** ‘Including unpublished literature on the review’s findings’.  **Review methods:** ‘Including unpublished literature on the review’s findings’.  **Review results:** Not analysed.  **Type:** Methodological.  **Covariate summary:** Publication status. | **Method to choose**  **covariate:** Not reported.  **Where method reported:** NA.  **Rationale for choosing the covariate:** Not reported.  **Where rationale reported:** NA. | **Where reported:** Protocol and review.  **Reason not reported in review:** NA.  **Reason not reported in protocol:** NA.  **Labelled as ‘posthoc’ if not in protocol:** NA. | **Data type:** Categorical.  **Continuous covariate categorised:** NA.  **How determine it was categorised:** NA.  **Justification for categorising:** NA.  **Where justification reported:** NA.  **Categories reported:** Yes.  **Where categories reported:** Protocol and review.  **Justification for categories:** Not reported.  **Where justification reported:** NA. | **Analysis type reported in protocol:** Sensitivity analysis.  **Analysis type reported in review:** Sensitivity analysis.  **Reason for changing mind:** NA.    **Analysis type in protocol according to glossary:** Sensitivity analysis.  **How determined the analysis type:** categories given.  **Analysis type in review according to glossary:** Sensitivity analysis.  **How determined the analysis type:** categories given.  **Analysed:** No.  **Reasons given (in the review) for not doing interaction analysis if planned:** Not reported (presumably because no trials).  **Reasons given for carrying out interaction analysis if they weren’t planned:** NA.  **Scenario in protocol: ‘**if the data allow.’  **Rationale for planned scenario:** Not reported.  **Scenario in review (if reported specifically): ‘**if data allowed.’  **Rationale for done scenario:** Not reported.  **Reason for changing mind:** Not reported. | **Method to detect interactions reported in protocol:** Not reported.  **Method to detect interactions reported in review:** Not reported.  **Reason for changing mind:** NA. |
| Dashash 2013 | **Protocol:** ‘Location of restoration (anterior or posterior)’.  **Review methods: ‘**Location of restoration (anterior or posterior)’.  **Review results:** Not analysed.  **Type:** Patient.  **Covariate summary:** Disease characteristics. | **Method to choose**  **covariate:** Not reported.  **Where method reported:** NA.  **Rationale for choosing the covariate:** Not reported.  **Where rationale reported:** NA. | **Where reported:** Protocol and review.  **Reason not reported in review:** NA.  **Reason not reported in protocol:** NA.  **Labelled as ‘posthoc’ if not in protocol:** NA. | **Data type:** Categorical.  **Continuous covariate categorised:** NA.  **How determine it was categorised:** NA.  **Justification for categorising:** NA.  **Where justification reported:** NA.  **Categories reported:** Yes.  **Where categories reported:** Protocol and review.  **Justification for categories:** Not reported.  **Where justification reported:** NA. | **Analysis type reported in protocol:** Subgroup analysis.  **Analysis type reported in review:** Subgroup analysis.  **Reason for changing mind:** NA.    **Analysis type in protocol according to glossary:** Subgroup analysis.  **How determined the analysis type:** categories given.  **Analysis type in review according to glossary:** Subgroup analysis.  **How determined the analysis type:** categories given.  **Analysed:** No.  **Reasons given (in the review) for not doing interaction analysis if planned:** Not reported (presumably because no trials).  **Reasons given for carrying out interaction analysis if they weren’t planned:** NA.  **Scenario in protocol: ‘**A planned subgroup analysis will be utilised to investigate some  potential factors for heterogeneity, which may affect outcomes.’  **Rationale for planned scenario:** Not reported.  **Scenario in review (if reported specifically): ‘**A planned subgroup analysis would be utilised to investigate some potential factors for heterogeneity, which may affect outcomes.’  **Rationale for done scenario:** Not reported.  **Reason for changing mind:** NA. | **Method to detect interactions reported in protocol:** Not reported.  **Method to detect interactions reported in review:** Not reported.  **Reason for changing mind:** NA. |
| Dashash 2013 | **Protocol:** ‘Type of amelogenesis imperfecta (hypoplastic, hypomaturated, hypocalcified, and hypomaturated hypoplastic enamel with taurodontism)’.  **Review methods: ‘**Type of amelogenesis imperfecta (hypoplastic, hypomaturated, hypocalcified, and hypomaturated hypoplastic enamel with taurodontism)’.  **Review results:** Not analysed.  **Type:** Patient.  **Covariate summary:** Disease characteristics. | **Method to choose**  **covariate:** Not reported.  **Where method reported:** NA.  **Rationale for choosing the covariate:** Not reported.  **Where rationale reported:** NA. | **Where reported:** Protocol and review.  **Reason not reported in review:** NA.  **Reason not reported in protocol:** NA.  **Labelled as ‘posthoc’ if not in protocol:** NA. | **Data type:** Categorical.  **Continuous covariate categorised:** NA.  **How determine it was categorised:** NA.  **Justification for categorising:** NA.  **Where justification reported:** NA.  **Categories reported:** Yes.  **Where categories reported:** Protocol and review.  **Justification for categories:** Not reported.  **Where justification reported:** NA. | **Analysis type reported in protocol:** Subgroup analysis.  **Analysis type reported in review:** Subgroup analysis.  **Reason for changing mind:** NA.    **Analysis type in protocol according to glossary:** Subgroup analysis.  **How determined the analysis type:** categories given.  **Analysis type in review according to glossary:** Subgroup analysis.  **How determined the analysis type:** categories given.  **Analysed:** No.  **Reasons given (in the review) for not doing interaction analysis if planned:** Not reported (presumably because no trials).  **Reasons given for carrying out interaction analysis if they weren’t planned:** NA.  **Scenario in protocol: ‘**A planned subgroup analysis will be utilised to investigate some  potential factors for heterogeneity, which may affect outcomes.’  **Rationale for planned scenario:** Not reported.  **Scenario in review (if reported specifically): ‘**A planned subgroup analysis would be utilised to investigate some potential factors for heterogeneity, which may affect outcomes.’  **Rationale for done scenario:** Not reported.  **Reason for changing mind:** NA. | **Method to detect interactions reported in protocol:** Not reported.  **Method to detect interactions reported in review:** Not reported.  **Reason for changing mind:** NA. |
| Dashash 2013 | **Protocol:** Allocation concealment. We judged the following to be three covariates: ‘Sensitivity analysis will be undertaken to examine the effect of randomisation, allocation concealment and blind outcome assessment on the overall estimates of effect.’  **Review methods:** Allocation concealment. We judged the following to be three covariates: ‘Sensitivity analysis was planned to be undertaken to examine the effect of randomisation, allocation concealment and blind outcome assessment on the overall estimates of effect ’.  **Review results:** Not analysed.  **Type:** Methodological.  **Covariate summary:** Allocation concealment. | **Method to choose**  **covariate:** Not reported.  **Where method reported:** NA.  **Rationale for choosing the covariate:** Not reported.  **Where rationale reported:** NA. | **Where reported:** Protocol and review.  **Reason not reported in review:** NA.  **Reason not reported in protocol:** NA.  **Labelled as ‘posthoc’ if not in protocol:** NA. | **Data type:** Categorical.  **Continuous covariate categorised:** NA.  **How determine it was categorised:** NA.  **Justification for categorising:** NA.  **Where justification reported:** NA.  **Categories reported:** Not reported.  **Where categories reported:** NA.  **Justification for categories:** Not reported.  **Where justification reported:** NA. | **Analysis type reported in protocol:** Sensitivity analysis.  **Analysis type reported in review:** Sensitivity analysis.  **Reason for changing mind:** NA.    **Analysis type in protocol according to glossary:** Sensitivity analysis.  **How determined the analysis type:** ‘sensitivity analysis’ no reason to dispute this.  **Analysis type in review according to glossary:** Sensitivity analysis.  **How determined the analysis type:** ‘sensitivity analysis’ no reason to dispute this.  **Analysed:** No.  **Reasons given (in the review) for not doing interaction analysis if planned:** Not reported (presumably because no trials).  **Reasons given for carrying out interaction analysis if they weren’t planned:** NA.  **Scenario in protocol:** Not reported.  **Rationale for planned scenario:** Not reported.  **Scenario in review (if reported specifically): ‘**if sufficient number of trials had been included in the review.’  **Rationale for done scenario:** Not reported.  **Reason for changing mind:** Not reported. | **Method to detect interactions reported in protocol:** Not reported.  **Method to detect interactions reported in review:** Not reported.  **Reason for changing mind:** NA. |
| Dashash 2013 | **Protocol:** Blind outcome assessment. We judged the following to be three covariates: ‘Sensitivity analysis will be undertaken to examine the effect of randomisation, allocation concealment and blind outcome assessment on the overall estimates of effect.’  **Review methods:** Blind outcome assessment. We judged the following to be three covariates: ‘Sensitivity analysis was planned to be undertaken to examine the effect of randomisation, allocation concealment and blind outcome assessment on the overall estimates of effect ’.  **Review results:** Not analysed.  **Type:** Methodological.  **Covariate summary:** Blinding. | **Method to choose**  **covariate:** Not reported.  **Where method reported:** NA.  **Rationale for choosing the covariate:** Not reported.  **Where rationale reported:** NA. | **Where reported:** Protocol and review.  **Reason not reported in review:** NA.  **Reason not reported in protocol:** NA.  **Labelled as ‘posthoc’ if not in protocol:** NA. | **Data type:** Categorical.  **Continuous covariate categorised:** NA.  **How determine it was categorised:** NA.  **Justification for categorising:** NA.  **Where justification reported:** NA.  **Categories reported:** Not reported.  **Where categories reported:** NA.  **Justification for categories:** Not reported.  **Where justification reported:** NA. | **Analysis type reported in protocol:** Sensitivity analysis.  **Analysis type reported in review:** Sensitivity analysis.  **Reason for changing mind:** NA.    **Analysis type in protocol according to glossary:** Sensitivity analysis.  **How determined the analysis type:** ‘sensitivity analysis’ no reason to dispute this.  **Analysis type in review according to glossary:** Sensitivity analysis.  **How determined the analysis type:** ‘sensitivity analysis’ no reason to dispute this.  **Analysed:** No.  **Reasons given (in the review) for not doing interaction analysis if planned:** Not reported (presumably because no trials).  **Reasons given for carrying out interaction analysis if they weren’t planned:** NA.  **Scenario in protocol:** Not reported.  **Rationale for planned scenario:** Not reported.  **Scenario in review (if reported specifically): ‘**if sufficient number of trials had been included in the review.’  **Rationale for done scenario:** Not reported.  **Reason for changing mind:** Not reported. | **Method to detect interactions reported in protocol:** Not reported.  **Method to detect interactions reported in review:** Not reported.  **Reason for changing mind:** NA. |
| Dashash 2013 | **Protocol:** Randomisation. We judged the following to be three covariates: ‘Sensitivity analysis will be undertaken to examine the effect of randomisation, allocation concealment and blind outcome assessment on the overall estimates of effect.’  **Review methods:** Randomisation. We judged the following to be three covariates: ‘Sensitivity analysis was planned to be undertaken to examine the effect of randomisation, allocation concealment and blind outcome assessment on the overall estimates of effect ’.  **Review results:** Not analysed.  **Type:** Methodological.  **Covariate summary:** Randomisation. | **Method to choose**  **covariate:** Not reported.  **Where method reported:** NA.  **Rationale for choosing the covariate:** Not reported.  **Where rationale reported:** NA. | **Where reported:** Protocol and review.  **Reason not reported in review:** NA.  **Reason not reported in protocol:** NA.  **Labelled as ‘posthoc’ if not in protocol:** NA. | **Data type:** Categorical.  **Continuous covariate categorised:** NA.  **How determine it was categorised:** NA.  **Justification for categorising:** NA.  **Where justification reported:** NA.  **Categories reported:** Not reported.  **Where categories reported:** NA.  **Justification for categories:** Not reported.  **Where justification reported:** NA. | **Analysis type reported in protocol:** Sensitivity analysis.  **Analysis type reported in review:** Sensitivity analysis.  **Reason for changing mind:** NA.    **Analysis type in protocol according to glossary:** Sensitivity analysis.  **How determined the analysis type:** ‘sensitivity analysis’ no reason to dispute this.  **Analysis type in review according to glossary:** Sensitivity analysis.  **How determined the analysis type:** ‘sensitivity analysis’ no reason to dispute this.  **Analysed:** No.  **Reasons given (in the review) for not doing interaction analysis if planned:** Not reported (presumably because no trials).  **Reasons given for carrying out interaction analysis if they weren’t planned:** NA.  **Scenario in protocol:** Not reported.  **Rationale for planned scenario:** Not reported.  **Scenario in review (if reported specifically): ‘**if sufficient number of trials had been included in the review.’  **Rationale for done scenario:** Not reported.  **Reason for changing mind:** Not reported. | **Method to detect interactions reported in protocol:** Not reported.  **Method to detect interactions reported in review:** Not reported.  **Reason for changing mind:** NA. |
| Deare 2013 | **Protocol:** Not reported.  **Review methods: ‘**Different forms of sham/placebo acupuncture. We judged the following to be three covariates: ‘We planned subgroup analyses to assess the effect of different types of acupuncture: 1) manual acupuncture versus electro-acupuncture; 2) shallow needling versus deep needling; 3) different forms of sham/ placebo acupuncture.’  **Review results:** Not analysed.  **Type:** Intervention.  **Covariate summary:** Type of intervention or control. | **Method to choose**  **covariate:** Not reported.  **Where method reported:** NA.  **Rationale for choosing the covariate:** Not reported.  **Where rationale reported:** NA. | **Where reported:** Review only.  **Reason not reported in review:** NA.  **Reason not reported in protocol:** Not reported.  **Labelled as ‘posthoc’ if not in protocol:** No. | **Data type:** Categorical.  **Continuous covariate categorised:** NA.  **How determine it was categorised:** NA.  **Justification for categorising:** NA.  **Where justification reported:** NA.  **Categories reported:** Not reported.  **Where categories reported:** NA.  **Justification for categories:** Not reported.  **Where justification reported:** NA. | **Analysis type reported in protocol:** Not reported.  **Analysis type reported in review:** Subgroup analysis.  **Reason for changing mind:** Not reported.    **Analysis type in protocol according to glossary:** Not reported.  **How determined the analysis type:** NA.  **Analysis type in review according to glossary:** Subgroup analysis.  **How determined the analysis type:** Says ‘subgroup analysis’. No reason to dispute.  **Analysed:** No.  **Reasons given (in the review) for not doing interaction analysis if planned:** Not reported.  **Reasons given for carrying out interaction analysis if they weren’t planned:** NA.  **Scenario in protocol:** Not reported.  **Rationale for planned scenario:** NA.  **Scenario in review (if reported specifically):** ‘When there were sufficient appropriate data’  **Rationale for done scenario:** Not reported.  **Reason for changing mind:** Not reported. | **Method to detect interactions reported in protocol:** Not reported.  **Method to detect interactions reported in review:** Not reported.  **Reason for changing mind:** NA. |
| Deare 2013 | **Protocol:** Not reported.  **Review methods: ‘**Manual acupuncture versus electro-acupuncture’. We judged the following to be three covariates: ‘we planned subgroup analyses to assess the effect of different types of acupuncture: 1) manual acupuncture versus electro-acupuncture; 2) shallow needling versus deep needling; 3) different forms of sham/ placebo acupuncture.’  **Review results:** ‘Electro-acupuncture’; ‘manual acupuncture’.  **Type:** Intervention.  **Covariate summary:** Type of intervention or control. | **Method to choose**  **covariate:** Not reported.  **Where method reported:** NA.  **Rationale for choosing the covariate:** Not reported.  **Where rationale reported:** NA. | **Where reported:** Review only.  **Reason not reported in review:** NA.  **Reason not reported in protocol:** Not reported.  **Labelled as ‘posthoc’ if not in protocol:** No. | **Data type:** Categorical.  **Continuous covariate categorised:** NA.  **How determine it was categorised:** NA.  **Justification for categorising:** NA.  **Where justification reported:** NA.  **Categories reported:** Yes.  **Where categories reported:** Review only.  **Justification for categories:** Not reported.  **Where justification reported:** NA. | **Analysis type reported in protocol:** Not reported.  **Analysis type reported in review:** Subgroup analysis.  **Reason for changing mind:** Not reported.    **Analysis type in protocol according to glossary:** Not reported.  **How determined the analysis type:** NA.  **Analysis type in review according to glossary:** Subgroup analysis.  **How determined the analysis type:** Categories given.  **Analysed:** Yes.  **Reasons given (in the review) for not doing interaction analysis if planned:** NA  **Reasons given for carrying out interaction analysis if they weren’t planned:** Not reported.  **Scenario in protocol:** Not reported.  **Rationale for planned scenario:** NA.  **Scenario in review (if reported specifically):** ‘When there were sufficient appropriate data’  **Rationale for done scenario:** Not reported.  **Reason for changing mind:** Not reported. | **Method to detect interactions reported in protocol:** Not reported.  **Method to detect interactions reported in review:** Not reported.  **Reason for changing mind:** NA. |
| Deare 2013 | **Protocol:** Not reported.  **Review methods: ‘**Shallow needling versus deep needling’. We judged the following to be three covariates: ‘We planned subgroup analyses to assess the effect of different types of acupuncture: 1) manual acupuncture versus electro-acupuncture; 2) shallow needling versus deep needling; 3) different forms of sham/ placebo acupuncture.’  **Review results:** Not analysed.  **Type:** Intervention.  **Covariate summary:** Type of intervention or control. | **Method to choose**  **covariate:** Not reported.  **Where method reported:** NA.  **Rationale for choosing the covariate:** Not reported.  **Where rationale reported:** NA. | **Where reported:** Review only.  **Reason not reported in review:** NA.  **Reason not reported in protocol:** Not reported.  **Labelled as ‘posthoc’ if not in protocol:** No. | **Data type:** Categorical.  **Continuous covariate categorised:** NA.  **How determine it was categorised:** NA.  **Justification for categorising:** NA.  **Where justification reported:** NA.  **Categories reported:** Yes.  **Where categories reported:** Review only.  **Justification for categories:** Not reported.  **Where justification reported:** NA. | **Analysis type reported in protocol:** Not reported.  **Analysis type reported in review:** Subgroup analysis.  **Reason for changing mind:** Not reported.    **Analysis type in protocol according to glossary:** Not reported.  **How determined the analysis type:** NA.  **Analysis type in review according to glossary:** Subgroup analysis.  **How determined the analysis type:** Categories given.  **Analysed:** No.  **Reasons given (in the review) for not doing interaction analysis if planned:** Not reported.  **Reasons given for carrying out interaction analysis if they weren’t planned:** NA.  **Scenario in protocol:** Not reported.  **Rationale for planned scenario:** NA.  **Scenario in review (if reported specifically):** ‘When there were sufficient appropriate data’  **Rationale for done scenario:** Not reported.  **Reason for changing mind:** Not reported. | **Method to detect interactions reported in protocol:** Not reported.  **Method to detect interactions reported in review:** Not reported.  **Reason for changing mind:** NA. |
| Deare 2013 | **Protocol:** ‘The following comparisons will be made: 1. Real acupuncture versus non-acupuncture treatment (e. g. wait list or drug therapy). 2. Real acupuncture versus placebo or sham acupuncture. 3. Real acupuncture versus standard or usual care (e. g. physiotherapy  or exercise). 4. A particular style of acupuncture versus another.’  **Review methods: ‘**We examined the following comparisons: 1. Acupuncture versus no acupuncture (e. g. wait list) 2. Acupuncture versus placebo or sham acupuncture 3. Acupuncture versus standard/usual care (e. g. cognitive behavioural therapy (CBT) and/or exercise and/or pharmacotherapy) 4. Acupuncture as an adjunct therapy to standard/usual care (evaluating additional effect) 5. A particular style of acupuncture versus another (e. g. deep needling with stimulation versus deep needling without stimulation)’.  **Review results:** Comparison 1. Acupuncture versus non-acupuncture treatment; Comparison 2. Acupuncture versus placebo or sham acupuncture; Comparison 3. Acupuncture versus medication; Comparison 4. Acupuncture as an adjunct therapy; Comparison 5. Deep needling with stimulation (T/S) versus deep; needling without stimulation (T/O).  **Type:** Intervention.  **Covariate summary:** Type of intervention or control. | **Method to choose**  **covariate:** Not reported.  **Where method reported:** NA.  **Rationale for choosing the covariate:** Not reported.  **Where rationale reported:** NA. | **Where reported:** Protocol and review.  **Reason not reported in review:** NA.  **Reason not reported in protocol:** NA.  **Labelled as ‘posthoc’ if not in protocol:** NA. | **Data type:** Categorical.  **Continuous covariate categorised:** NA.  **How determine it was categorised:** NA.  **Justification for categorising:** NA.  **Where justification reported:** NA.  **Categories reported:** Yes.  **Where categories reported:** Protocol and review.  **Justification for categories:** Not reported.  **Where justification reported:** NA. | **Analysis type reported in protocol:** Unnamed analysis.  **Analysis type reported in review:** Unnamed analysis.  **Reason for changing mind:** NA.    **Analysis type in protocol according to glossary:** Stratification/subgroup analysis.  **How determined the analysis type:** Categories given.  **Analysis type in review according to glossary:** Stratification/subgroup analysis.  **How determined the analysis type:** Categories given  **Analysed:** Yes.  **Reasons given (in the review) for not doing interaction analysis if planned:** NA.  **Reasons given for carrying out interaction analysis if they weren’t planned:** NA.  **Scenario in protocol:** Not reported.  **Rationale for planned scenario:** NA.  **Scenario in review (if reported specifically):** Not reported.  **Rationale for done scenario:** NA.  **Reason for changing mind:** Not reported. | **Method to detect interactions reported in protocol:** Not reported.  **Method to detect interactions reported in review:** Not reported.  **Reason for changing mind:** NA. |
| Deare 2013 | **Protocol:** Allocation concealment. We judged the following to be two covariates: ‘If possible, a sensitivity analysis will be conducted to examine whether aspects of methodological quality influence the effect size For example, does inadequate concealment of allocation or failure to blind outcome assessors affect the robustness of the results?’  **Review methods:** Allocation concealment. We judged the following to be two covariates: ‘We also planned to conduct sensitivity analyses to examine whether aspects of methodological quality influence the effect size For example, does inadequate concealment of allocation or failure to blind outcome assessors affect the robustness of the results?’  **Review results:** Not analysed.  **Type:** Methodological.  **Covariate summary:** Allocation concealment. | **Method to choose**  **covariate:** Not reported.  **Where method reported:** NA.  **Rationale for choosing the covariate:** Not reported.  **Where rationale reported:** NA. | **Where reported:** Protocol and review.  **Reason not reported in review:** NA.  **Reason not reported in protocol:** NA.  **Labelled as ‘posthoc’ if not in protocol:** NA. | **Data type:** Categorical.  **Continuous covariate categorised:** NA.  **How determine it was categorised:** NA.  **Justification for categorising:** NA.  **Where justification reported:** NA.  **Categories reported:** Yes.  **Where categories reported:** Protocol and review.  **Justification for categories:** Not reported.  **Where justification reported:** NA. | **Analysis type reported in protocol:** Sensitivity analysis.  **Analysis type reported in review:** Sensitivity analysis.  **Reason for changing mind:** NA.    **Analysis type in protocol according to glossary:** Sensitivity analysis.  **How determined the analysis type:** Categories given.  **Analysis type in review according to glossary:** Sensitivity analysis.  **How determined the analysis type:** Categories given.  **Analysed:** No.  **Reasons given (in the review) for not doing interaction analysis if planned:** ‘We could not conduct planned sensitivity analyses due to a lack  of trials with and without adequate concealment of allocation;  or with and without blinded outcome assessor under one comparison.  For instance, under the comparison of real and sham  acupuncture, all six studies were at low risk of selection bias with  adequate concealment of treatment allocation, using no treatment  as the control, and at low risk of detection bias with outcome  assessors being blinded (Assefi 2005; Deluze 1992; Harris 2005;  Harris 2008; Harris 2009; Itoh 2010; Martin 2006). Two studies  were at higher risk of selection bias with unclear or inadequate  allocation concealment and had a high risk of detection bias with  inadequate or unclear blinding of outcome assessor (Guo 2005;  Targino 2008). They were, however, under different comparison  categories and contained only one study in each. Itoh 2010 had a  moderate risk of bias as a non-acupuncture treatment control was  used.’  **Reasons given for carrying out interaction analysis if they weren’t planned:** NA.  **Scenario in protocol:** ‘if possible’.  **Rationale for planned scenario:** Not reported.  **Scenario in review (if reported specifically):** Not reported.  **Rationale for done scenario:** NA.  **Reason for changing mind:** Not reported. | **Method to detect interactions reported in protocol:** Not reported.  **Method to detect interactions reported in review:** Not reported.  **Reason for changing mind:** NA. |
| Deare 2013 | **Protocol:** Blinding. We judged the following to be two covariates: ‘If possible, a sensitivity analysis will be conducted to examine whether aspects of methodological quality influence the effect size For example, does inadequate concealment of allocation or failure to blind outcome assessors affect the robustness of the results?’  **Review methods:** Blinding. We judged the following to be two covariates: ‘We also planned to conduct sensitivity analyses to examine whether aspects of methodological quality influence the effect size For example, does inadequate concealment of allocation or failure to blind outcome assessors affect the robustness of the results?’  **Review results:** Not analysed.  **Type:** Methodological.  **Covariate summary:** Blinding. | **Method to choose**  **covariate:** Not reported.  **Where method reported:** NA.  **Rationale for choosing the covariate:** Not reported.  **Where rationale reported:** NA. | **Where reported:** Protocol and review.  **Reason not reported in review:** NA.  **Reason not reported in protocol:** NA.  **Labelled as ‘posthoc’ if not in protocol:** NA. | **Data type:** Categorical.  **Continuous covariate categorised:** NA.  **How determine it was categorised:** NA.  **Justification for categorising:** NA.  **Where justification reported:** NA.  **Categories reported:** Yes.  **Where categories reported:** Protocol and review.  **Justification for categories:** Not reported.  **Where justification reported:** NA. | **Analysis type reported in protocol:** Sensitivity analysis.  **Analysis type reported in review:** Sensitivity analysis.  **Reason for changing mind:** NA.    **Analysis type in protocol according to glossary:** Sensitivity analysis.  **How determined the analysis type:** Categories given.  **Analysis type in review according to glossary:** Sensitivity analysis.  **How determined the analysis type:** Categories given.  **Analysed:** No.  **Reasons given (in the review) for not doing interaction analysis if planned:** ‘We could not conduct planned sensitivity analyses due to a lack  of trials with and without adequate concealment of allocation;  or with and without blinded outcome assessor under one comparison.  For instance, under the comparison of real and sham  acupuncture, all six studies were at low risk of selection bias with  adequate concealment of treatment allocation, using no treatment  as the control, and at low risk of detection bias with outcome  assessors being blinded (Assefi 2005; Deluze 1992; Harris 2005;  Harris 2008; Harris 2009; Itoh 2010; Martin 2006). Two studies  were at higher risk of selection bias with unclear or inadequate  allocation concealment and had a high risk of detection bias with  inadequate or unclear blinding of outcome assessor (Guo 2005;  Targino 2008). They were, however, under different comparison  categories and contained only one study in each. Itoh 2010 had a  moderate risk of bias as a non-acupuncture treatment control was  used.’  **Reasons given for carrying out interaction analysis if they weren’t planned:** NA.  **Scenario in protocol:** ‘if possible’.  **Rationale for planned scenario:** Not reported.  **Scenario in review (if reported specifically):** Not reported.  **Rationale for done scenario:** NA.  **Reason for changing mind:** Not reported. | **Method to detect interactions reported in protocol:** Not reported.  **Method to detect interactions reported in review:** Not reported.  **Reason for changing mind:** NA. |
| Deare 2013 | **Protocol:** Not reported.  **Review methods:** Not reported.  **Review results:** Time point: one month after treatment; seven months after treatment.  **Type:** Outcome.  **Covariate summary:** Time point. | **Method to choose**  **covariate:** Not reported.  **Where method reported:** NA.  **Rationale for choosing the covariate:** Not reported.  **Where rationale reported:** NA. | **Where reported:** Review only.  **Reason not reported in review:** NA.  **Reason not reported in protocol:** Not reported.  **Labelled as ‘posthoc’ if not in protocol:** No. | **Data type:** Categorical.  **Continuous covariate categorised:** NA.  **How determine it was categorised:** NA.  **Justification for categorising:** NA.  **Where justification reported:** NA.  **Categories reported:** Yes.  **Where categories reported:** Review only.  **Justification for categories:** Not reported.  **Where justification reported:** NA. | **Analysis type reported in protocol:** Not reported.  **Analysis type reported in review:** Unnamed analysis.  **Reason for changing mind:** Not reported.    **Analysis type in protocol according to glossary:** Not reported.  **How determined the analysis type:** NA.  **Analysis type in review according to glossary:** Stratification/subgroup analysis.  **How determined the analysis type:** Categories given  **Analysed:** Yes.  **Reasons given (in the review) for not doing interaction analysis if planned:** NA.  **Reasons given for carrying out interaction analysis if they weren’t planned:** Not reported.  **Scenario in protocol:** Not reported.  **Rationale for planned scenario:** NA.  **Scenario in review (if reported specifically):** Not reported.  **Rationale for done scenario:** NA.  **Reason for changing mind:** Not reported. | **Method to detect interactions reported in protocol:** Not reported.  **Method to detect interactions reported in review:** Not reported.  **Reason for changing mind:** NA. |
| Deare 2013 | **Protocol:** Not reported.  **Review methods**: Not reported.  **Review results: ‘**Sham non-invasive acupuncture (not breaking skin) vs sham invasive acupuncture (breaking skin)’. ‘We also undertook a subgroup analyses of studies using sham acupuncture without breaking the skin (Assefi 2005;Harris 2008;Harris 2009;Martin 2006) versus studies using breaking-skin sham interventions (Assefi 2005; Deluze 1992; Harris 2005).’  **Type:** Intervention.  **Covariate summary:** Type of intervention or control. | **Method to choose**  **covariate:** Not reported.  **Where method reported:** NA.  **Rationale for choosing the covariate:** Not reported.  **Where rationale reported:** NA. | **Where reported:** Review only.  **Reason not reported in review:** NA.  **Reason not reported in protocol:** Not reported.  **Labelled as ‘posthoc’ if not in protocol:** No. | **Data type:** Categorical.  **Continuous covariate categorised:** NA.  **How determine it was categorised:** NA.  **Justification for categorising:** NA.  **Where justification reported:** NA.  **Categories reported:** Yes.  **Where categories reported:** Review only.  **Justification for categories:** Not reported.  **Where justification reported:** NA. | **Analysis type reported in protocol:** Not reported.  **Analysis type reported in review:** Unnamed analysis.  **Reason for changing mind:** Not reported.    **Analysis type in protocol according to glossary:** Not reported.  **How determined the analysis type:** NA.  **Analysis type in review according to glossary:** Stratification/subgroup analysis.  **How determined the analysis type:** Categories given  **Analysed:** Yes.  **Reasons given (in the review) for not doing interaction analysis if planned:** NA.  **Reasons given for carrying out interaction analysis if they weren’t planned:** Not reported.  **Scenario in protocol:** Not reported.  **Rationale for planned scenario:** NA.  **Scenario in review (if reported specifically):** Not reported.  **Rationale for done scenario:** NA.  **Reason for changing mind:** Not reported. | **Method to detect interactions reported in protocol:** Not reported.  **Method to detect interactions reported in review:** Not reported.  **Reason for changing mind:** NA. |
| Freak-Poli 2013 | **Protocol:** ‘Age (as the probability of maintaining good health diminishes as an individual gets older (AIHW 2008), there may  be differing motivations for participation in pedometer-based workplace health programmes depending on age)’.  **Review methods: ‘**Age (as the probability of maintaining good health diminishes as an individual gets older (AIHW 2008), there may be differing motivations for participation in pedometer-based workplace health programmes depending on age)’.  **Review results:** Not analysed.  **Type:** Patient.  **Covariate summary:** Demographics. | **Method to choose**  **covariate:** Not reported.  **Where method reported:** NA.  **Rationale for choosing the covariate:** ‘as the probability of maintaining good health  diminishes as an individual gets older (AIHW 2008), there may  be differing motivations for participation in pedometer-based  workplace health programmes depending on age’.  **Where rationale reported:** Protocol and review. | **Where reported:** Protocol and review.  **Reason not reported in review:** NA.  **Reason not reported in protocol:** NA.  **Labelled as ‘posthoc’ if not in protocol:** NA | **Data type:** Continuous.  **Continuous covariate categorised:** Yes.  **How determine it was categorised:** Subgroup analysis planned.  **Justification for categorising:** Not reported.  **Where justification reported:** NA.  **Categories reported:** Not reported.  **Where categories reported:** NA.  **Justification for categories:** Not reported.  **Where justification reported:** NA. | **Analysis type reported in protocol:** Subgroup analysis.  **Analysis type reported in review:** Subgroup analysis.  **Reason for changing mind:** NA.    **Analysis type in protocol according to glossary:** Subgroup analysis.  **How determined the analysis type:** Says ‘subgroup analysis’. No reason to dispute.  **Analysis type in review according to glossary:** Subgroup analysis.  **How determined the analysis type:** Says ‘subgroup analysis’. No reason to dispute.  **Analysed:** No.  **Reasons given (in the review) for not doing interaction analysis if planned:** Not reported.  **Reasons given for carrying out interaction analysis if they weren’t planned:** NA.  **Scenario in protocol:** ‘If more than two trials are available that report data in each category,  we will explore the following participant characteristics using  subgroup analyses.’.  **Rationale for planned scenario:** Not reported.  **Scenario in review (if reported specifically):** ‘If more than two trials were available that reported data in each category, we aimed to explore the following participant characteristics  using subgroup analyses:’  **Rationale for done scenario:** NA.  **Reason for changing mind:** NA**.** | **Method to detect interactions reported in protocol:** Not reported.  **Method to detect interactions reported in review:** Not reported.  **Reason for changing mind:** NA. |
| Freak-Poli 2013 | **Protocol:** ‘Duration: are short duration interventions (less than or equal to three months) more effective than longer duration interventions (more than three months)?’ ‘We will analyse the following categories separately: short term and long term interventions.’  **Review methods: ‘**Duration: are short duration interventions (less than or equal to three months) more effective than longer duration interventions (more than three months)?’ ‘We aimed to analyse the following categories separately: Short-term and long-term interventions.’  **Review results:** Not analysed.  **Type:** Intervention.  **Covariate summary:** Duration of intervention. | **Method to choose**  **covariate:** Not reported.  **Where method reported:** NA.  **Rationale for choosing the covariate:** Not reported.  **Where rationale reported:** NA. | **Where reported:** Protocol and review.  **Reason not reported in review:** NA.  **Reason not reported in protocol:** NA.  **Labelled as ‘posthoc’ if not in protocol:** NA | **Data type:** Continuous.  **Continuous covariate categorised:** Yes.  **How determine it was categorised:** Categories given.  **Justification for categorising:** Not reported.  **Where justification reported:** NA.  **Categories reported:** Yes.  **Where categories reported:** Protocol and review.  **Justification for categories:** Not reported.  **Where justification reported:** NA. | **Analysis type reported in protocol:** Subgroup analysis.  **Analysis type reported in review:** Subgroup analysis.  **Reason for changing mind:** NA.    **Analysis type in protocol according to glossary:** Subgroup analysis.  **How determined the analysis type:** Categories given.  **Analysis type in review according to glossary:** Subgroup analysis.  **How determined the analysis type:** Categories given.  **Analysed:** No.  **Reasons given (in the review) for not doing interaction analysis if planned:** Not reported.  **Reasons given for carrying out interaction analysis if they weren’t planned:** NA.  **Scenario in protocol:** ‘If more than two trials are available that report data in each category,  we will explore the following intervention characteristics..’.  **Rationale for planned scenario:** Not reported.  **Scenario in review (if reported specifically):** ‘If more than two trials were available that reported data in each category, we aimed to explore the following intervention characteristics.’  **Rationale for done scenario:** NA.  **Reason for changing mind:** NA**.** | **Method to detect interactions reported in protocol:** Not reported.  **Method to detect interactions reported in review:** Not reported.  **Reason for changing mind:** NA. |
| Freak-Poli 2013 | **Protocol:** ‘Education status (completion of tertiary education)’.  **Review methods: ‘**Education status (completion of tertiary education)’.  **Review results:** Not analysed.  **Type:** Patient.  **Covariate summary:** Demographics. | **Method to choose**  **covariate:** Not reported.  **Where method reported:** NA.  **Rationale for choosing the covariate:** Not reported.  **Where rationale reported:** NA. | **Where reported:** Protocol and review.  **Reason not reported in review:** NA.  **Reason not reported in protocol:** NA.  **Labelled as ‘posthoc’ if not in protocol:** NA. | **Data type:** Categorical.  **Continuous covariate categorised:** NA.  **How determine it was categorised:** NA.  **Justification for categorising:** NA.  **Where justification reported:** NA.  **Categories reported:** Not reported.  **Where categories reported:** NA.  **Justification for categories:** Not reported.  **Where justification reported:** NA. | **Analysis type reported in protocol:** Subgroup analysis.  **Analysis type reported in review:** Subgroup analysis.  **Reason for changing mind:** NA.    **Analysis type in protocol according to glossary:** Subgroup analysis.  **How determined the analysis type:** Categories given.  **Analysis type in review according to glossary:** Subgroup analysis.  **How determined the analysis type:** Categories given.  **Analysed:** No.  **Reasons given (in the review) for not doing interaction analysis if planned:** Not reported.  **Reasons given for carrying out interaction analysis if they weren’t planned:** NA.  **Scenario in protocol:** ‘If more than two trials are available that report data in each category,  we will explore the following participant characteristics using  subgroup analyses.’.  **Rationale for planned scenario:** Not reported.  **Scenario in review (if reported specifically):** ‘If more than two trials were available that reported data in each category, we aimed to explore the following participant characteristics  using subgroup analyses:’  **Rationale for done scenario:** NA.  **Reason for changing mind:** NA**.** | **Method to detect interactions reported in protocol:** Not reported.  **Method to detect interactions reported in review:** Not reported.  **Reason for changing mind:** NA. |
| Freak-Poli 2013 | **Protocol:** ‘Eligibility of participants: Are interventions targeting high-risk employees more effective than interventions recruiting all employees?’  **Review methods: ‘**Eligibility of participants: Are interventions targeting high-risk employees more effective than interventions recruiting all employees?’  **Review results:** Not analysed.  **Type:** Patient.  **Covariate summary:** Disease characteristics. | **Method to choose**  **covariate:** Not reported.  **Where method reported:** NA.  **Rationale for choosing the covariate:** Not reported.  **Where rationale reported:** NA. | **Where reported:** Protocol and review.  **Reason not reported in review:** NA.  **Reason not reported in protocol:** NA.  **Labelled as ‘posthoc’ if not in protocol:** NA | **Data type:** Categorical.  **Continuous covariate categorised:** NA.  **How determine it was categorised:** NA.  **Justification for categorising:** NA.  **Where justification reported:** NA.  **Categories reported:** Yes.  **Where categories reported:** Protocol and review.  **Justification for categories:** Not reported.  **Where justification reported:** NA. | **Analysis type reported in protocol:** Subgroup analysis.  **Analysis type reported in review:** Subgroup analysis.  **Reason for changing mind:** NA.    **Analysis type in protocol according to glossary:** Subgroup analysis.  **How determined the analysis type:** Categories given.  **Analysis type in review according to glossary:** Subgroup analysis.  **How determined the analysis type:** Categories given.  **Analysed:** No.  **Reasons given (in the review) for not doing interaction analysis if planned:** Not reported.  **Reasons given for carrying out interaction analysis if they weren’t planned:** NA.  **Scenario in protocol:** ‘If more than two trials are available that report data in each category,  we will explore the following intervention characteristics..’.  **Rationale for planned scenario:** Not reported.  **Scenario in review (if reported specifically):** ‘If more than two trials were available that reported data in each category, we aimed to explore the following intervention characteristics.’  **Rationale for done scenario:** NA.  **Reason for changing mind:** NA**.** | **Method to detect interactions reported in protocol:** Not reported.  **Method to detect interactions reported in review:** Not reported.  **Reason for changing mind:** NA. |
| Freak-Poli 2013 | **Protocol:** ‘Eligibility of participants: Are interventions targeting sedentary/office employees more successful than interventions targeting active/manual employees?’  **Review methods: ‘**Eligibility of participants: Are interventions targeting sedentary/office employees more successful than interventions targeting active/manual employees?’  **Review results:** Not analysed.  **Type:** Patient.  **Covariate summary:** Demographics. | **Method to choose**  **covariate:** Not reported.  **Where method reported:** NA.  **Rationale for choosing the covariate:** Not reported.  **Where rationale reported:** NA. | **Where reported:** Protocol and review.  **Reason not reported in review:** NA.  **Reason not reported in protocol:** NA.  **Labelled as ‘posthoc’ if not in protocol:** NA. | **Data type:** Categorical.  **Continuous covariate categorised:** NA.  **How determine it was categorised:** NA.  **Justification for categorising:** NA.  **Where justification reported:** NA.  **Categories reported:** Yes.  **Where categories reported:** Protocol and review.  **Justification for categories:** Not reported.  **Where justification reported:** NA. | **Analysis type reported in protocol:** Subgroup analysis.  **Analysis type reported in review:** Subgroup analysis.  **Reason for changing mind:** NA.    **Analysis type in protocol according to glossary:** Subgroup analysis.  **How determined the analysis type:** Categories given.  **Analysis type in review according to glossary:** Subgroup analysis.  **How determined the analysis type:** Categories given.  **Analysed:** No.  **Reasons given (in the review) for not doing interaction analysis if planned:** Not reported.  **Reasons given for carrying out interaction analysis if they weren’t planned:** NA.  **Scenario in protocol:** ‘If more than two trials are available that report data in each category,  we will explore the following intervention characteristics..’.  **Rationale for planned scenario:** Not reported.  **Scenario in review (if reported specifically):** ‘If more than two trials were available that reported data in each category, we aimed to explore the following intervention characteristics.’  **Rationale for done scenario:** NA.  **Reason for changing mind:** NA**.** | **Method to detect interactions reported in protocol:** Not reported.  **Method to detect interactions reported in review:** Not reported.  **Reason for changing mind:** NA. |
| Freak-Poli 2013 | **Protocol:** ‘Provider: are interventions with an external programme provider more effective than interventions undertaken internally within the workplace?’  **Review methods: ‘**Provider: are interventions with an external programme provider more effective than interventions undertaken internally within the workplace?’  **Review results:** Not analysed.  **Type:** Intervention.  **Covariate summary:** Type of intervention or control. | **Method to choose**  **covariate:** Not reported.  **Where method reported:** NA.  **Rationale for choosing the covariate:** Not reported.  **Where rationale reported:** NA. | **Where reported:** Protocol and review.  **Reason not reported in review:** NA.  **Reason not reported in protocol:** NA.  **Labelled as ‘posthoc’ if not in protocol:** NA. | **Data type:** Categorical.  **Continuous covariate categorised:** NA.  **How determine it was categorised:** NA.  **Justification for categorising:** NA.  **Where justification reported:** NA.  **Categories reported:** Yes.  **Where categories reported:** Protocol and review.  **Justification for categories:** Not reported.  **Where justification reported:** NA. | **Analysis type reported in protocol:** Subgroup analysis.  **Analysis type reported in review:** Subgroup analysis.  **Reason for changing mind:** NA.    **Analysis type in protocol according to glossary:** Subgroup analysis.  **How determined the analysis type:** Categories given.  **Analysis type in review according to glossary:** Subgroup analysis.  **How determined the analysis type:** Categories given.  **Analysed:** No.  **Reasons given (in the review) for not doing interaction analysis if planned:** Not reported.  **Reasons given for carrying out interaction analysis if they weren’t planned:** NA.  **Scenario in protocol:** ‘If more than two trials are available that report data in each category,  we will explore the following intervention characteristics..’.  **Rationale for planned scenario:** Not reported.  **Scenario in review (if reported specifically):** ‘If more than two trials were available that reported data in each category, we aimed to explore the following intervention characteristics.’  **Rationale for done scenario:** NA.  **Reason for changing mind:** NA**.** | **Method to detect interactions reported in protocol:** Not reported.  **Method to detect interactions reported in review:** Not reported.  **Reason for changing mind:** NA. |
| Freak-Poli 2013 | **Protocol: ‘**Sex’.  **Review methods:** ’Gender’.  **Review results:** Not analysed.  **Type:** Patient.  **Covariate summary:** Demographics. | **Method to choose**  **covariate:** Not reported.  **Where method reported:** NA.  **Rationale for choosing the covariate:** Not reported.  **Where rationale reported:** NA. | **Where reported:** Protocol and review.  **Reason not reported in review:** NA.  **Reason not reported in protocol:** NA.  **Labelled as ‘posthoc’ if not in protocol:** NA. | **Data type:** Categorical.  **Continuous covariate categorised:** NA.  **How determine it was categorised:** NA.  **Justification for categorising:** NA.  **Where justification reported:** NA.  **Categories reported:** Not reported.  **Where categories reported:** NA.  **Justification for categories:** Not reported.  **Where justification reported:** NA. | **Analysis type reported in protocol:** Subgroup analysis.  **Analysis type reported in review:** Subgroup analysis.  **Reason for changing mind:** NA.    **Analysis type in protocol according to glossary:** Subgroup analysis.  **How determined the analysis type:** Says ‘subgroup analysis’. No reason to dispute.  **Analysis type in review according to glossary:** Subgroup analysis.  **How determined the analysis type:** Says ‘subgroup analysis’. No reason to dispute.  **Analysed:** No.  **Reasons given (in the review) for not doing interaction analysis if planned:** Not reported.  **Reasons given for carrying out interaction analysis if they weren’t planned:** NA.  **Scenario in protocol:** ‘If more than two trials are available that report data in each category,  we will explore the following participant characteristics using  subgroup analyses.’.  **Rationale for planned scenario:** Not reported.  **Scenario in review (if reported specifically):** ‘If more than two trials were available that reported data in each category, we aimed to explore the following participant characteristics  using subgroup analyses:’  **Rationale for done scenario:** NA.  **Reason for changing mind:** NA**.** | **Method to detect interactions reported in protocol:** Not reported.  **Method to detect interactions reported in review:** Not reported.  **Reason for changing mind:** NA. |
| Freak-Poli 2013 | **Protocol:** ‘Step diary: are interventions that utilise a step diary (i. e. daily or weekly record of steps) more effective in changing physical activity than non-diary interventions?’  **Review methods: ‘**Step diary: are interventions that utilise a step diary (i. e. daily or weekly record of steps) more effective in changing physical activity than non-diary interventions?’  **Review results:** Not analysed.  **Type:** Intervention.  **Covariate summary:** Type of intervention or control. | **Method to choose**  **covariate:** Not reported.  **Where method reported:** NA.  **Rationale for choosing the covariate:** Not reported.  **Where rationale reported:** NA. | **Where reported:** Protocol and review.  **Reason not reported in review:** NA.  **Reason not reported in protocol:** NA.  **Labelled as ‘posthoc’ if not in protocol:** NA. | **Data type:** Categorical.  **Continuous covariate categorised:** NA.  **How determine it was categorised:** NA.  **Justification for categorising:** NA.  **Where justification reported:** NA.  **Categories reported:** Yes.  **Where categories reported:** Protocol and review.  **Justification for categories:** Not reported.  **Where justification reported:** NA. | **Analysis type reported in protocol:** Subgroup analysis.  **Analysis type reported in review:** Subgroup analysis.  **Reason for changing mind:** NA.    **Analysis type in protocol according to glossary:** Subgroup analysis.  **How determined the analysis type:** Categories given.  **Analysis type in review according to glossary:** Subgroup analysis.  **How determined the analysis type:** Categories given.  **Analysed:** No.  **Reasons given (in the review) for not doing interaction analysis if planned:** Not reported.  **Reasons given for carrying out interaction analysis if they weren’t planned:** NA.  **Scenario in protocol:** ‘If more than two trials are available that report data in each category,  we will explore the following intervention characteristics..’.  **Rationale for planned scenario:** Not reported.  **Scenario in review (if reported specifically):** ‘If more than two trials were available that reported data in each category, we aimed to explore the following intervention characteristics.’  **Rationale for done scenario:** NA.  **Reason for changing mind:** NA**.** | **Method to detect interactions reported in protocol:** Not reported.  **Method to detect interactions reported in review:** Not reported.  **Reason for changing mind:** NA. |
| Freak-Poli 2013 | **Protocol:** ‘Step goal: are interventions that utilise a daily step goal (i. e. 10,000 steps per day) more effective than non-step goal defined interventions?’  **Review methods: ‘**Step goal: are interventions that utilise a daily step goal (i. e. 10,000 steps per day) more effective than non-step goal defined interventions?’  **Review results:** Not analysed.  **Type:** Intervention.  **Covariate summary:** Type of intervention or control. | **Method to choose**  **covariate:** Not reported.  **Where method reported:** NA.  **Rationale for choosing the covariate:** Not reported.  **Where rationale reported:** NA. | **Where reported:** Protocol and review.  **Reason not reported in review:** NA.  **Reason not reported in protocol:** NA.  **Labelled as ‘posthoc’ if not in protocol:** NA | **Data type:** Categorical.  **Continuous covariate categorised:** NA.  **How determine it was categorised:** NA.  **Justification for categorising:** NA.  **Where justification reported:** NA.  **Categories reported:** Yes.  **Where categories reported:** Protocol and review.  **Justification for categories:** Not reported.  **Where justification reported:** NA. | **Analysis type reported in protocol:** Subgroup analysis.  **Analysis type reported in review:** Subgroup analysis.  **Reason for changing mind:** NA.    **Analysis type in protocol according to glossary:** Subgroup analysis.  **How determined the analysis type:** Categories given.  **Analysis type in review according to glossary:** Subgroup analysis.  **How determined the analysis type:** Categories given.  **Analysed:** No.  **Reasons given (in the review) for not doing interaction analysis if planned:** Not reported.  **Reasons given for carrying out interaction analysis if they weren’t planned:** NA.  **Scenario in protocol:** ‘If more than two trials are available that report data in each category,  we will explore the following intervention characteristics..’.  **Rationale for planned scenario:** Not reported.  **Scenario in review (if reported specifically):** ‘If more than two trials were available that reported data in each category, we aimed to explore the following intervention characteristics.’  **Rationale for done scenario:** NA.  **Reason for changing mind:** NA**.** | **Method to detect interactions reported in protocol:** Not reported.  **Method to detect interactions reported in review:** Not reported.  **Reason for changing mind:** NA. |
| Freak-Poli 2013 | **Protocol: ‘**The primary measurement time point of interest is at long-term follow up. We will categorise follow-up time as short-term (less than one month), medium-term (more than a month but less than one year) and long-term (more than or equal to one year).’  **Review methods: ‘**We categorised follow-up time as short-term (less than one month), medium-term (more than a month but less than one year) and long-term (equal to or more than one year)’.  **Review results:** Not analysed.  **Type:** Outcome.  **Covariate summary:** Time point. | **Method to choose**  **covariate:** Not reported.  **Where method reported:** NA.  **Rationale for choosing the covariate:** Not reported.  **Where rationale reported:** NA. | **Where reported:** Protocol and review.  **Reason not reported in review:** NA.  **Reason not reported in protocol:** NA.  **Labelled as ‘posthoc’ if not in protocol:** NA | **Data type:** Continuous.  **Continuous covariate categorised:** Yes.  **How determine it was categorised:** Categories given.  **Justification for categorising:** Not reported.  **Where justification reported:** NA.  **Categories reported:** Yes.  **Where categories reported:** Protocol and review.  **Justification for categories:** Not reported.  **Where justification reported:** NA. | **Analysis type reported in protocol:** Unnamed analysis.  **Analysis type reported in review:** Unnamed analysis.  **Reason for changing mind:** NA.    **Analysis type in protocol according to glossary:** Stratification/subgroup analysis.  **How determined the analysis type:** Categories given.  **Analysis type in review according to glossary:** Stratification/subgroup analysis.  **How determined the analysis type:** Categories given.  **Analysed:** No.  **Reasons given (in the review) for not doing interaction analysis if planned:** Not reported.  **Reasons given for carrying out interaction analysis if they weren’t planned:** NA.  **Scenario in protocol:** Not reported.  **Rationale for planned scenario:** NA.  **Scenario in review (if reported specifically):** Not reported.  **Rationale for done scenario:** NA.  **Reason for changing mind:** NA**.** | **Method to detect interactions reported in protocol:** Not reported.  **Method to detect interactions reported in review:** Not reported.  **Reason for changing mind:** NA. |
| Freak-Poli 2013 | **Protocol:** ‘We will analyse the following categories separately: Studies comparing pedometer interventions to no intervention, similar components without a pedometer, larger scale health promotion interventions and other active interventions.’  **Review methods:** ‘We aimed to analyse the following categories separately: Studies comparing pedometer interventions to no intervention, similar components without a pedometer, larger scale health promotion interventions and other active interventions.’  **Review results:** ‘Pedometer programme versus ‘no intervention’ control’; ‘Pedometer programme versus alternative programme without pedometer’.  **Type:** Intervention.  **Covariate summary:** Type of intervention or control. | **Method to choose**  **covariate:** Not reported.  **Where method reported:** NA.  **Rationale for choosing the covariate:** Not reported.  **Where rationale reported:** NA. | **Where reported:** Protocol and review.  **Reason not reported in review:** NA.  **Reason not reported in protocol:** NA.  **Labelled as ‘posthoc’ if not in protocol:** NA**.** | **Data type:** Categorical.  **Continuous covariate categorised:** NA.  **How determine it was categorised:** NA.  **Justification for categorising:** NA.  **Where justification reported:** NA.  **Categories reported:** Yes.  **Where categories reported:** Protocol and review.  **Justification for categories:** Not reported.  **Where justification reported:** NA. | **Analysis type reported in protocol:** Unnamed analysis.  **Analysis type reported in review:** Unnamed analysis.  **Reason for changing mind:** NA.    **Analysis type in protocol according to glossary:** Stratification/subgroup analysis.  **How determined the analysis type:** Categories given.  **Analysis type in review according to glossary:** Stratification/subgroup analysis.  **How determined the analysis type:** Categories given.  **Analysed:** Yes.  **Reasons given (in the review) for not doing interaction analysis if planned:** NA.  **Reasons given for carrying out interaction analysis if they weren’t planned:** NA.  **Scenario in protocol:** Not reported.  **Rationale for planned scenario:** NA.  **Scenario in review (if reported specifically):** Not reported.  **Rationale for done scenario:** NA.  **Reason for changing mind:** NA**.** | **Method to detect interactions reported in protocol:** Not reported.  **Method to detect interactions reported in review:** Not reported.  **Reason for changing mind:** NA. |
| Freak-Poli 2013 | **Protocol:** ‘We will analyse the following categories separately: Studies of pedometers alone, pedometer-focused interventions with supporting components to increase motivation (e. g. step goals, diaries, teams, rewards) and broader health promotion interventions that incorporated pedometers as one of many components.’ ‘We will include interventions in which the pedometer is the sole component of the intervention, interventions in which the pedometer is the main focus of the intervention but is supported by components to increase motivation (e. g. step goals, diaries, teams, rewards) and broader health promotion interventions that incorporate pedometers as one of many components. We will explore the modifying effects of additional components if possible through subgroup analysis.’  **Review methods: ‘**We aimed to analyse the following categories separately: Studies of pedometers alone, pedometer-focused interventions with supporting components to increase motivation (e.g. step goals, diaries, teams, rewards) and broader health promotion interventions that incorporated pedometers as one of many components.’ ‘We included studies in which the pedometer was the sole component of the intervention; interventions in which the pedometer was the main focus of the intervention but was supported by other intervention components like step goals, diaries, teams, or rewards to increase motivation; and broader health promotion interventions that incorporated pedometers as one of many components. We aimed to explore the modifying effects of additional intervention components through subgroup analysis.’  **Review results:** Not analysed.  **Type:** Intervention.  **Covariate summary:** Type of intervention or control. | **Method to choose**  **covariate:** Not reported.  **Where method reported:** NA.  **Rationale for choosing the covariate:** Not reported.  **Where rationale reported:** NA. | **Where reported:** Protocol and review.  **Reason not reported in review:** NA.  **Reason not reported in protocol:** NA.  **Labelled as ‘posthoc’ if not in protocol:** NA | **Data type:** Categorical.  **Continuous covariate categorised:** NA.  **How determine it was categorised:** NA.  **Justification for categorising:** NA.  **Where justification reported:** NA.  **Categories reported:** Yes.  **Where categories reported:** Protocol and review.  **Justification for categories:** Not reported.  **Where justification reported:** NA. | **Analysis type reported in protocol:** Unnamed analysis.  **Analysis type reported in review:** Unnamed analysis.  **Reason for changing mind:** NA.    **Analysis type in protocol according to glossary:** Stratification/subgroup analysis.  **How determined the analysis type:** Categories given.  **Analysis type in review according to glossary:** Stratification/subgroup analysis.  **How determined the analysis type:** Categories given.  **Analysed:** No.  **Reasons given (in the review) for not doing interaction analysis if planned:** Not reported.  **Reasons given for carrying out interaction analysis if they weren’t planned:** NA.  **Scenario in protocol:** Not reported.  **Rationale for planned scenario:** NA.  **Scenario in review (if reported specifically):** Not reported.  **Rationale for done scenario:** NA.  **Reason for changing mind:** NA**.** | **Method to detect interactions reported in protocol:** Not reported.  **Method to detect interactions reported in review:** Not reported.  **Reason for changing mind:** NA. |
| Freak-Poli 2013 | **Protocol:** ‘We will carry out a sensitivity analysis for studies with low risk of bias, defined as meeting at least three of the following criteria: random sequence generation; allocation concealment; incomplete outcome data; selective outcome reporting; and other sources of bias.’  **Review methods: ‘**We aimed to carry out a sensitivity analysis for studies with low risk of bias, defined as meeting at least three of the following criteria: random sequence generation; allocation concealment; incomplete outcome data; selective outcome reporting; and other sources of bias.’  **Review results:** Not analysed.  **Type:** Methodological.  **Covariate summary:** Risk of bias. | **Method to choose**  **covariate:** Not reported.  **Where method reported:** NA.  **Rationale for choosing the covariate:** Not reported.  **Where rationale reported:** NA. | **Where reported:** Protocol and review.  **Reason not reported in review:** NA.  **Reason not reported in protocol:** NA.  **Labelled as ‘posthoc’ if not in protocol:** NA | **Data type:** Categorical.  **Continuous covariate categorised:** NA.  **How determine it was categorised:** NA.  **Justification for categorising:** NA.  **Where justification reported:** NA.  **Categories reported:** Yes.  **Where categories reported:** Protocol and review.  **Justification for categories:** Not reported.  **Where justification reported:** NA. | **Analysis type reported in protocol:** Subgroup analysis.  **Analysis type reported in review:** Subgroup analysis.  **Reason for changing mind:** NA.    **Analysis type in protocol according to glossary:** Subgroup analysis.  **How determined the analysis type:** Categories given.  **Analysis type in review according to glossary:** Subgroup analysis.  **How determined the analysis type:** Categories given.  **Analysed:** No.  **Reasons given (in the review) for not doing interaction analysis if planned:** Not reported.  **Reasons given for carrying out interaction analysis if they weren’t planned:** NA.  **Scenario in protocol:** ‘If more than two trials are available that report data in each category,  we will explore the following intervention characteristics..’.  **Rationale for planned scenario:** Not reported.  **Scenario in review (if reported specifically):** ‘If more than two trials were available that reported data in each category, we aimed to explore the following intervention characteristics.’  **Rationale for done scenario:** NA.  **Reason for changing mind:** NA**.** | **Method to detect interactions reported in protocol:** Not reported.  **Method to detect interactions reported in review:** Not reported.  **Reason for changing mind:** NA. |
| Gan 2013 | **Protocol:** ‘Disease severity at entry’.  **Review methods: ‘**Disease severity at entry’.  **Review results:** Not analysed.  **Type:** Patient.  **Covariate summary:** Disease characteristics. | **Method to choose**  **covariate:** Not reported.  **Where method reported:** NA.  **Rationale for choosing the covariate:** Not reported.  **Where rationale reported:** NA. | **Where reported:** Protocol and review.  **Reason not reported in review:** NA.  **Reason not reported in protocol:** NA.  **Labelled as ‘posthoc’ if not in protocol:** NA. | **Data type:** Continuous.  **Continuous covariate categorised:** Yes.  **How determine it was categorised:** Subgroup analysis planned.  **Justification for categorising:** No reported.  **Where justification reported:** NA.  **Categories reported:** Not reported.  **Where categories reported:** NA.  **Justification for categories:** Not reported.  **Where justification reported:** NA. | **Analysis type reported in protocol:** Subgroup analysis.  **Analysis type reported in review:** Subgroup analysis.  **Reason for changing mind:** NA.  **Analysis type in protocol according to glossary:** Subgroup analysis.  **How determined the analysis type:** ‘subgroup analysis’ no reason to dispute this.  **Analysis type in review according to glossary:** Subgroup analysis.  **How determined the analysis type:** ‘subgroup analysis’ no reason to dispute this.  **Analysed:** No.  **Reasons given (in the review) for not doing interaction analysis if planned:** Not reported (presumably because no trials).  **Reasons given for carrying out interaction analysis if they weren’t planned:** NA.  **Scenario in protocol:** Not reported.  **Rationale for planned scenario:** NA.  **Scenario in review (if reported specifically):** (if P value <0.1).  **Rationale for done scenario:** Not reported.  **Reason for changing mind:** Not reported. | **Method to detect interactions reported in protocol:** Not reported.  **Method to detect interactions reported in review:** Not reported.  **Reason for changing mind:** NA. |
| Gan 2013 | **Protocol:** Allocation concealment. We judged the following to be four covariates: ‘We will compare subgroups of trial with and without adequate methodological components (generation of allocation sequence, allocation concealment, blinding, follow-up) regarding the primary outcome measures.’  **Review methods:** ‘We planned to compare the trials with low risk of bias versus those with high risk of bias for the primary outcome measures.’  **Review results:** Not analysed.  **Type:** Methodological.  **Covariate summary:** Allocation concealment. | **Method to choose**  **covariate:** Not reported.  **Where method reported:** NA.  **Rationale for choosing the covariate:** Not reported.  **Where rationale reported:** NA. | **Where reported:** Protocol and review.  **Reason not reported in review:** NA.  **Reason not reported in protocol:** NA.  **Labelled as ‘posthoc’ if not in protocol:** NA. | **Data type:** Categorical.  **Continuous covariate categorised:** NA.  **How determine it was categorised:** NA.  **Justification for categorising:** NA.  **Where justification reported:** NA.  **Categories reported:** Yes.  **Where categories reported:** Protocol and review.  **Justification for categories:** Not reported.  **Where justification reported:** NA. | **Analysis type reported in protocol:** Subgroup analysis.  **Analysis type reported in review:** Subgroup analysis.  **Reason for changing mind:** NA.    **Analysis type in protocol according to glossary:** Subgroup analysis.  **How determined the analysis type:** Categories given.  **Analysis type in review according to glossary:** Subgroup analysis.  **How determined the analysis type:** Categories given.  **Analysed:** No.  **Reasons given (in the review) for not doing interaction analysis if planned:** Not reported (presumably because no trials).  **Reasons given for carrying out interaction analysis if they weren’t planned:** NA.  **Scenario in protocol:** ‘regarding the primary outcome measures’.  **Rationale for planned scenario:** Not reported.  **Scenario in review (if reported specifically):** ‘for the primary outcome measures’.  **Rationale for done scenario:** Not reported.  **Reason for changing mind:** NA. | **Method to detect interactions reported in protocol:** Not reported.  **Method to detect interactions reported in review:** Not reported.  **Reason for changing mind:** NA. |
| Gan 2013 | **Protocol:** Blinding. We judged the following to be four covariates: ‘We will compare subgroups of trial with and without adequate methodological components (generation of allocation sequence, allocation concealment, blinding, follow-up) regarding the primary outcome measures.’  **Review methods:** ‘We planned to compare the trials with low risk of bias versus those with high risk of bias for the primary outcome measures.’  **Review results:** Not analysed.  **Type:** Methodological.  **Covariate summary:** Blinding. | **Method to choose**  **covariate:** Not reported.  **Where method reported:** NA.  **Rationale for choosing the covariate:** Not reported.  **Where rationale reported:** NA. | **Where reported:** Protocol and review.  **Reason not reported in review:** NA.  **Reason not reported in protocol:** NA.  **Labelled as ‘posthoc’ if not in protocol:** NA. | **Data type:** Categorical.  **Continuous covariate categorised:** NA.  **How determine it was categorised:** NA.  **Justification for categorising:** NA.  **Where justification reported:** NA.  **Categories reported:** Yes.  **Where categories reported:** Protocol and review.  **Justification for categories:** Not reported.  **Where justification reported:** NA. | **Analysis type reported in protocol:** Subgroup analysis.  **Analysis type reported in review:** Subgroup analysis.  **Reason for changing mind:** NA.    **Analysis type in protocol according to glossary:** Subgroup analysis.  **How determined the analysis type:** Categories given.  **Analysis type in review according to glossary:** Subgroup analysis.  **How determined the analysis type:** Categories given.  **Analysed:** No.  **Reasons given (in the review) for not doing interaction analysis if planned:** Not reported (presumably because no trials).  **Reasons given for carrying out interaction analysis if they weren’t planned:** NA.  **Scenario in protocol:** ‘regarding the primary outcome measures’.  **Rationale for planned scenario:** Not reported.  **Scenario in review (if reported specifically):** ‘for the primary outcome measures’.  **Rationale for done scenario:** Not reported.  **Reason for changing mind:** NA. | **Method to detect interactions reported in protocol:** Not reported.  **Method to detect interactions reported in review:** Not reported.  **Reason for changing mind:** NA. |
| Gan 2013 | **Protocol:** Dose. We judged the following to be two covariates: ‘dose and duration of experimental interventions.’  **Review methods:** Dose. We judged the following to be two covariates: ‘dose and duration of experimental interventions.’  **Review results:** Not analysed.  **Type:** Intervention.  **Covariate summary:** Dose. | **Method to choose**  **covariate:** Not reported.  **Where method reported:** NA.  **Rationale for choosing the covariate:** Not reported.  **Where rationale reported:** NA. | **Where reported:** Protocol and review.  **Reason not reported in review:** NA.  **Reason not reported in protocol:** NA.  **Labelled as ‘posthoc’ if not in protocol:** NA. | **Data type:** Continuous.  **Continuous covariate categorised:** Yes.  **How determine it was categorised:** Subgroup analysis planned.  **Justification for categorising:** No reported.  **Where justification reported:** NA.  **Categories reported:** Not reported.  **Where categories reported:** NA.  **Justification for categories:** Not reported.  **Where justification reported:** NA. | **Analysis type reported in protocol:** Subgroup analysis.  **Analysis type reported in review:** Subgroup analysis.  **Reason for changing mind:** NA.  **Analysis type in protocol according to glossary:** Subgroup analysis.  **How determined the analysis type:** ‘subgroup analysis’ no reason to dispute this.  **Analysis type in review according to glossary:** Subgroup analysis.  **How determined the analysis type:** ‘subgroup analysis’ no reason to dispute this.  **Analysed:** No.  **Reasons given (in the review) for not doing interaction analysis if planned:** Not reported (presumably because no trials).  **Reasons given for carrying out interaction analysis if they weren’t planned:** NA.  **Scenario in protocol:** Not reported.  **Rationale for planned scenario:** NA.  **Scenario in review (if reported specifically):** (if P value <0.1).  **Rationale for done scenario:** Not reported.  **Reason for changing mind:** Not reported. | **Method to detect interactions reported in protocol:** Not reported.  **Method to detect interactions reported in review:** Not reported.  **Reason for changing mind:** NA. |
| Gan 2013 | **Protocol:** Duration. We judged the following to be two covariates: ‘dose and duration of experimental interventions.’  **Review methods:** Duration. We judged the following to be two covariates: ‘dose and duration of experimental interventions.’  **Review results:** Not analysed.  **Type:** Intervention.  **Covariate summary:** Duration of intervention. | **Method to choose**  **covariate:** Not reported.  **Where method reported:** NA.  **Rationale for choosing the covariate:** Not reported.  **Where rationale reported:** NA. | **Where reported:** Protocol and review.  **Reason not reported in review:** NA.  **Reason not reported in protocol:** NA.  **Labelled as ‘posthoc’ if not in protocol:** NA. | **Data type:** Continuous.  **Continuous covariate categorised:** Yes.  **How determine it was categorised:** Subgroup analysis planned.  **Justification for categorising:** No reported.  **Where justification reported:** NA.  **Categories reported:** Not reported.  **Where categories reported:** NA.  **Justification for categories:** Not reported.  **Where justification reported:** NA. | **Analysis type reported in protocol:** Subgroup analysis.  **Analysis type reported in review:** Subgroup analysis.  **Reason for changing mind:** NA.  **Analysis type in protocol according to glossary:** Subgroup analysis.  **How determined the analysis type:** ‘subgroup analysis’ no reason to dispute this.  **Analysis type in review according to glossary:** Subgroup analysis.  **How determined the analysis type:** ‘subgroup analysis’ no reason to dispute this.  **Analysed:** No.  **Reasons given (in the review) for not doing interaction analysis if planned:** Not reported (presumably because no trials).  **Reasons given for carrying out interaction analysis if they weren’t planned:** NA.  **Scenario in protocol:** Not reported.  **Rationale for planned scenario:** NA.  **Scenario in review (if reported specifically):** (if P value <0.1).  **Rationale for done scenario:** Not reported.  **Reason for changing mind:** Not reported. | **Method to detect interactions reported in protocol:** Not reported.  **Method to detect interactions reported in review:** Not reported.  **Reason for changing mind:** NA. |
| Gan 2013 | **Protocol:** Follow-up. We judged the following to be four covariates: ‘We will compare subgroups of trial with and without adequate methodological components (generation of allocation sequence, allocation concealment, blinding, follow-up) regarding the primary outcome measures.’  **Review methods:** ‘We planned to compare the trials with low risk of bias versus those with high risk of bias for the primary outcome measures.’  **Review results:** Not analysed.  **Type:** Methodological.  **Covariate summary:** Incomplete outcome data/follow up. | **Method to choose**  **covariate:** Not reported.  **Where method reported:** NA.  **Rationale for choosing the covariate:** Not reported.  **Where rationale reported:** NA. | **Where reported:** Protocol and review.  **Reason not reported in review:** NA.  **Reason not reported in protocol:** NA.  **Labelled as ‘posthoc’ if not in protocol:** NA. | **Data type:** Categorical.  **Continuous covariate categorised:** NA.  **How determine it was categorised:** NA.  **Justification for categorising:** NA.  **Where justification reported:** NA.  **Categories reported:** Yes.  **Where categories reported:** Protocol and review.  **Justification for categories:** Not reported.  **Where justification reported:** NA. | **Analysis type reported in protocol:** Subgroup analysis.  **Analysis type reported in review:** Subgroup analysis.  **Reason for changing mind:** NA.    **Analysis type in protocol according to glossary:** Subgroup analysis.  **How determined the analysis type:** Categories given.  **Analysis type in review according to glossary:** Subgroup analysis.  **How determined the analysis type:** Categories given.  **Analysed:** No.  **Reasons given (in the review) for not doing interaction analysis if planned:** Not reported (presumably because no trials).  **Reasons given for carrying out interaction analysis if they weren’t planned:** NA.  **Scenario in protocol:** ‘regarding the primary outcome measures’.  **Rationale for planned scenario:** Not reported.  **Scenario in review (if reported specifically):** ‘for the primary outcome measures’.  **Rationale for done scenario:** Not reported.  **Reason for changing mind:** NA. | **Method to detect interactions reported in protocol:** Not reported.  **Method to detect interactions reported in review:** Not reported.  **Reason for changing mind:** NA. |
| Gan 2013 | **Protocol:** Generation of allocation sequence. We judged the following to be four covariates: ‘We will compare subgroups of trial with and without adequate methodological components (generation of allocation sequence, allocation concealment, blinding, follow-up) regarding the primary outcome measures.’  **Review methods:** ‘We planned to compare the trials with low risk of bias versus those with high risk of bias for the primary outcome measures.’  **Review results:** Not analysed.  **Type:** Methodological.  **Covariate summary:** Random generation. | **Method to choose**  **covariate:** Not reported.  **Where method reported:** NA.  **Rationale for choosing the covariate:** Not reported.  **Where rationale reported:** NA. | **Where reported:** Protocol and review.  **Reason not reported in review:** NA.  **Reason not reported in protocol:** NA.  **Labelled as ‘posthoc’ if not in protocol:** NA. | **Data type:** Categorical.  **Continuous covariate categorised:** NA.  **How determine it was categorised:** NA.  **Justification for categorising:** NA.  **Where justification reported:** NA.  **Categories reported:** Yes.  **Where categories reported:** Protocol and review.  **Justification for categories:** Not reported.  **Where justification reported:** NA. | **Analysis type reported in protocol:** Subgroup analysis.  **Analysis type reported in review:** Subgroup analysis.  **Reason for changing mind:** NA.    **Analysis type in protocol according to glossary:** Subgroup analysis.  **How determined the analysis type:** Categories given.  **Analysis type in review according to glossary:** Subgroup analysis.  **How determined the analysis type:** Categories given.  **Analysed:** No.  **Reasons given (in the review) for not doing interaction analysis if planned:** Not reported (presumably because no trials).  **Reasons given for carrying out interaction analysis if they weren’t planned:** NA.  **Scenario in protocol:** ‘regarding the primary outcome measures’.  **Rationale for planned scenario:** Not reported.  **Scenario in review (if reported specifically):** ‘for the primary outcome measures’.  **Rationale for done scenario:** Not reported.  **Reason for changing mind:** NA. | **Method to detect interactions reported in protocol:** Not reported.  **Method to detect interactions reported in review:** Not reported.  **Reason for changing mind:** NA. |
| Gan 2013 | **Protocol:** Not reported.  **Review methods:** ‘The trials comparing herbs versus herbs were to be analysed separately from trials comparing herbs versus no treatment (with or without placebo).’  **Review results:** Not analysed.  **Type:** Intervention.  **Covariate summary:** Type of intervention or control. | **Method to choose**  **covariate:** Not reported.  **Where method reported:** NA.  **Rationale for choosing the covariate:** Not reported.  **Where rationale reported:** NA. | **Where reported:** Review only.  **Reason not reported in review:** NA.  **Reason not reported in protocol:** Not reported.  **Labelled as ‘posthoc’ if not in protocol:** No. | **Data type:** Categorical.  **Continuous covariate categorised:** NA.  **How determine it was categorised:** NA.  **Justification for categorising:** NA.  **Where justification reported:** NA.  **Categories reported:** Yes.  **Where categories reported:** Review only.  **Justification for categories:** Not reported.  **Where justification reported:** NA. | **Analysis type reported in protocol:** Not reported.  **Analysis type reported in review:** Unnamed analysis.  **Reason for changing mind:** Not reported.  **Analysis type in protocol according to glossary:** Not reported.  **How determined the analysis type:** NA.  **Analysis type in review according to glossary:** Subgroup analysis.  **How determined the analysis type:** Categories given.  **Analysed:** No.  **Reasons given (in the review) for not doing interaction analysis if planned:** Not reported (presumably because no trials).  **Reasons given for carrying out interaction analysis if they weren’t planned:** NA.  **Scenario in protocol:** Not reported.  **Rationale for planned scenario:** NA.  **Scenario in review (if reported specifically):** Not reported.  **Rationale for done scenario:** Not reported.  **Reason for changing mind:** NA. | **Method to detect interactions reported in protocol:** Not reported.  **Method to detect interactions reported in review:** Not reported.  **Reason for changing mind:** NA. |
| Gillies 2012 | **Protocol:** ‘Age group of participants: 0-5, 5-12, and 13-18 years’.  **Review methods:** Not reported.  **Review results:** Not analysed.  **Type:** Patient.  **Covariate summary:** Demographics. | **Method to choose**  **covariate:** Not reported.  **Where method reported:** NA.  **Rationale for choosing the covariate:** Not reported.  **Where rationale reported:** NA. | **Where reported:** Protocol only.  **Reason not reported in review:** Not reported.  **Reason not reported in protocol:** NA.  **Labelled as ‘posthoc’ if not in protocol:** NA. | **Data type:** Continuous.  **Continuous covariate categorised:** Yes.  **How determine it was categorised:** Categories given.  **Justification for categorising:** Not reported.  **Where justification reported:** NA.  **Categories reported:** Yes.  **Where categories reported:** Protocol only.  **Justification for categories:** Not reported.  **Where justification reported:** NA. | **Analysis type reported in protocol:** Subgroup analysis.  **Analysis type reported in review:** Not reported.  **Reason for changing mind:** Not reported.    **Analysis type in protocol according to glossary:** Subgroup analysis.  **How determined the analysis type:** Categories given.  **Analysis type in review according to glossary:** Not reported.  **How determined the analysis type:** NA.  **Analysed:** No.  **Reasons given (in the review) for not doing interaction analysis if planned:** Not reported.  **Reasons given for carrying out interaction analysis if they weren’t planned:** NA.  **Scenario in protocol:** ‘if data are available’  **Rationale for planned scenario:** Not reported.  **Scenario in review (if reported specifically):** Not reported.  **Rationale for done scenario:** NA.  **Reason for changing mind:** NA. | **Method to detect interactions reported in protocol:** Not reported.  **Method to detect interactions reported in review:** Not reported.  **Reason for changing mind:** NA. |
| Gillies 2012 | **Protocol:** ‘Data will be analysed as short-term (up to one month following completion of the therapy), medium-term (one month to one year following completion) and long-term (greater than one year).’  **Review methods:** ‘We analysed data as short-term (up to one month following completion of the therapy), medium-term (one month to one year following completion) and long-term (one year or more).’  **Review results:** ‘short’; ‘medium’; ‘long term’.  **Type:** Outcome.  **Covariate summary:** Time point. | **Method to choose**  **covariate:** Not reported.  **Where method reported:** NA.  **Rationale for choosing the covariate:** Not reported.  **Where rationale reported:** NA. | **Where reported:** Protocol and review.  **Reason not reported in review:** NA.  **Reason not reported in protocol:** NA.  **Labelled as ‘posthoc’ if not in protocol:** NA. | **Data type:** Continuous.  **Continuous covariate categorised:** Yes.  **How determine it was categorised:** Categories given.  **Justification for categorising:** Not reported.  **Where justification reported:** NA.  **Categories reported:** Yes.  **Where categories reported:** Protocol and review.  **Justification for categories:** Not reported.  **Where justification reported:** NA. | **Analysis type reported in protocol:** Unnamed analysis.  **Analysis type reported in review:** Unnamed analysis.  **Reason for changing mind:** NA.    **Analysis type in protocol according to glossary:** Stratifcation/subgroup analysis.  **How determined the analysis type:** Categories given.  **Analysis type in review according to glossary:** Stratifcation/subgroup analysis.  **How determined the analysis type:** Categories given.  **Analysed:** Yes.  **Reasons given (in the review) for not doing interaction analysis if planned:** NA.  **Reasons given for carrying out interaction analysis if they weren’t planned:** NA.  **Scenario in protocol:** Not reported.  **Rationale for planned scenario:** NA.  **Scenario in review (if reported specifically):** Not reported.  **Rationale for done scenario:** NA.  **Reason for changing mind:** NA. | **Method to detect interactions reported in protocol:** Not reported.  **Method to detect interactions reported in review:** Not reported.  **Reason for changing mind:** NA. |
| Gillies 2012 | **Protocol:** ‘Gender of participants’.  **Review methods:** Not reported.  **Review results:** Not analysed.  **Type:** Patient.  **Covariate summary:** Demographics. | **Method to choose**  **covariate:** Not reported.  **Where method reported:** NA.  **Rationale for choosing the covariate:** Not reported.  **Where rationale reported:** NA. | **Where reported:** Protocol only.  **Reason not reported in review:** Not reported.  **Reason not reported in protocol:** NA.  **Labelled as ‘posthoc’ if not in protocol:** NA. | **Data type:** Categorical.  **Continuous covariate categorised:** NA.  **How determine it was categorised:** NA.  **Justification for categorising:** NA.  **Where justification reported:** NA.  **Categories reported:** Not reported.  **Where categories reported:** NA.  **Justification for categories:** Not reported.  **Where justification reported:** NA. | **Analysis type reported in protocol:** Subgroup analysis.  **Analysis type reported in review:** Not reported.  **Reason for changing mind:** Not reported.    **Analysis type in protocol according to glossary:** Subgroup analysis.  **How determined the analysis type:** Says ‘Subgroup analysis’. No reason to dispute.  **Analysis type in review according to glossary:** Not reported.  **How determined the analysis type:** NA.  **Analysed:** No.  **Reasons given (in the review) for not doing interaction analysis if planned:** Not reported.  **Reasons given for carrying out interaction analysis if they weren’t planned:** NA.  **Scenario in protocol:** ‘if data are available’  **Rationale for planned scenario:** Not reported.  **Scenario in review (if reported specifically):** Not reported.  **Rationale for done scenario:** NA.  **Reason for changing mind:** NA. | **Method to detect interactions reported in protocol:** Not reported.  **Method to detect interactions reported in review:** Not reported.  **Reason for changing mind:** NA. |
| Gillies 2012 | **Protocol: ‘**The three main comparisons will be: 1. Psychological therapies vs no treatment 2. Psychological therapies vs pharmacological therapies 3. Psychological therapies vs other treatments Major categories of psychological therapies will include CBT (including behavioural and cognitive based therapies), exposure based therapy, psychodynamic therapy, supportive therapy (which will include non-directive counselling), debriefing (including psychological first aid), family-based therapy and EMDR.’  **Review methods: ‘**Main comparisons**.** 1. Psychological therapies versus a control 2. Psychological therapies versus other psychological therapies 3. Psychological therapies versus pharmacological therapies 4. Psychological therapies versus other treatments. The majority of interventions employed a variety of psychotherapeutic elements, so we categorised interventions on the primary description of the intervention, the primary objectives of the therapy, or both. However, as it became apparent to us during the review that some of the psychological therapies described in included studies were not based on any clear theoretical domain, we made the decision to include these under the heading of ’Other psychological therapies’. We organised data under the following categories of psychological therapies. 1. Cognitive behavioural therapy (CBT) 2. Behavioural therapy (BT) (including exposure-based therapy and narrative therapy) 3. Psychodynamic psychotherapy 4. Supportive counselling 5. EMDR 6. Interpersonal therapy (IPT) 7. Other psychological therapy’.  **Review results: ‘**Comparison 1. All psychological therapies versus control’; ‘Comparison 2. CBT versus control’; ‘Comparison 3. Narrative versus control’; ‘Comparison 4. EMDR versus control’; ‘Comparison 5. Other psychological therapies versus control’; ‘Comparison 6. CBT versus supportive counselling’; ‘Comparison 7. Narrative versus supportive counselling’; ‘Comparison 8. Narrative versus meditation/relaxation’; ‘Comparison 9. Exposure versus interpersonal therapy’.  **Type:** Intervention.  **Covariate summary:** Type of intervention or control. | **Method to choose**  **covariate:** Not reported.  **Where method reported:** NA.  **Rationale for choosing the covariate:** Not reported.  **Where rationale reported:** NA. | **Where reported:** Protocol and review.  **Reason not reported in review:** NA.  **Reason not reported in protocol:** NA.  **Labelled as ‘posthoc’ if not in protocol:** NA. | **Data type:** Categorical.  **Continuous covariate categorised:** NA.  **How determine it was categorised:** NA.  **Justification for categorising:** NA.  **Where justification reported:** NA.  **Categories reported:** Yes.  **Where categories reported:** Protocol and review.  **Justification for categories:** Not reported.  **Where justification reported:** NA. | **Analysis type reported in protocol:** Unnamed analysis.  **Analysis type reported in review:** Unnamed analysis.  **Reason for changing mind:** NA.    **Analysis type in protocol according to glossary:** Stratifcation/subgroup analysis.  **How determined the analysis type:** Categories given.  **Analysis type in review according to glossary:** Stratifcation/subgroup analysis.  **How determined the analysis type:** Categories given.  **Analysed:** Yes.  **Reasons given (in the review) for not doing interaction analysis if planned:** NA.  **Reasons given for carrying out interaction analysis if they weren’t planned:** NA.  **Scenario in protocol:** Not reported.  **Rationale for planned scenario:** NA.  **Scenario in review (if reported specifically):** Not reported.  **Rationale for done scenario:** NA.  **Reason for changing mind:** NA. | **Method to detect interactions reported in protocol:** Not reported.  **Method to detect interactions reported in review:** Not reported.  **Reason for changing mind:** NA. |
| Gillies 2012 | **Protocol: ‘**The type of trauma: natural or man-made disaster, physical or sexual abuse, war, violence, and life-threatening injury or illness.’  **Review methods:** ‘Type of trauma: sexual abuse, civil or social violence, physical trauma, natural disasters.’  **Review results:** ‘Sexual abuse’; ‘Civil/social violence’; ‘Physical trauma’.  **Type:** Patient.  **Covariate summary:** Disease characteristics. | **Method to choose**  **covariate:** Not reported.  **Where method reported:** NA.  **Rationale for choosing the covariate:** Not reported.  **Where rationale reported:** NA. | **Where reported:** Protocol and review.  **Reason not reported in review:** NA.  **Reason not reported in protocol:** NA.  **Labelled as ‘posthoc’ if not in protocol:** NA. | **Data type:** Categorical.  **Continuous covariate categorised:** NA.  **How determine it was categorised:** NA.  **Justification for categorising:** NA.  **Where justification reported:** NA.  **Categories reported:** Yes.  **Where categories reported:** Protocol and review.  **Justification for categories:** Not reported.  **Where justification reported:** NA. | **Analysis type reported in protocol:** Subgroup analysis.  **Analysis type reported in review:** Subgroup analysis.  **Reason for changing mind:** NA.    **Analysis type in protocol according to glossary:** Subgroup analysis.  **How determined the analysis type:** Categories given.  **Analysis type in review according to glossary:** Subgroup analysis.  **How determined the analysis type:** Categories given.  **Analysed:** Yes.  **Reasons given (in the review) for not doing interaction analysis if planned:** NA.  **Reasons given for carrying out interaction analysis if they weren’t planned:** NA.  **Scenario in protocol:** ‘if data are available’  **Rationale for planned scenario:** Not reported.  **Scenario in review (if reported specifically):** Not reported.  **Rationale for done scenario:** NA.  **Reason for changing mind:** NA. | **Method to detect interactions reported in protocol:** Not reported.  **Method to detect interactions reported in review:** Not reported.  **Reason for changing mind:** NA. |
| Gillies 2012 | **Protocol:** Allocation concealment. We judged the following to be two covariates: ‘Sensitivity analyses based on allocation concealment and blinding of outcome measurement will be done as these factors are most associated with a bias in effect size (Moher 1998).’  **Review methods:** Allocation concealment. We judged the following to be two covariates: ‘We carried out sensitivity analyses based on allocation concealment and blinding of outcome measurement as these factors are most associated with a bias in effect size’. Allocation concealment: low versus unclear risk (no high risk studies were identified).’  **Review results:** Not analysed.  **Type:** Methodological.  **Covariate summary:** Allocation concealment. | **Method to choose**  **covariate:** Not reported.  **Where method reported:** NA.  **Rationale for choosing the covariate:** ‘Sensitivity analyses based on allocation concealment and blinding  of outcome measurement will be done as these factors are most  associated with a bias in effect size (Moher 1998).’  **Where rationale reported:** Protocol and review. | **Where reported:** Protocol and review.  **Reason not reported in review:** NA.  **Reason not reported in protocol:** NA.  **Labelled as ‘posthoc’ if not in protocol:** NA. | **Data type:** Categorical.  **Continuous covariate categorised:** NA.  **How determine it was categorised:** NA.  **Justification for categorising:** NA.  **Where justification reported:** NA.  **Categories reported:** Yes.  **Where categories reported:** Review only.  **Justification for categories:** Not reported.  **Where justification reported:** NA. | **Analysis type reported in protocol:** Sensitivity analysis.  **Analysis type reported in review:** Sensitivity analysis.  **Reason for changing mind:** NA.    **Analysis type in protocol according to glossary:** Sensitivity analysis.  **How determined the analysis type:** Says ‘Sensitivity analysis’. No reason to dispute.  **Analysis type in review according to glossary:** Subgroup analysis.  **How determined the analysis type:** Categories given.  **Analysed:** No.  **Reasons given (in the review) for not doing interaction analysis if planned:** ‘As most studies did not report allocation concealment a sensitivity  analysis could only be conducted for PTSD symptom’.  **Reasons given for carrying out interaction analysis if they weren’t planned:** NA.  **Scenario in protocol:** Not reported.  **Rationale for planned scenario:** NA.  **Scenario in review (if reported specifically):** Not reported.  **Rationale for done scenario:** NA.  **Reason for changing mind:** NA. | **Method to detect interactions reported in protocol:** Not reported.  **Method to detect interactions reported in review:** Not reported.  **Reason for changing mind:** NA. |
| Gillies 2012 | **Protocol:** Blinding. We judged the following to be two covariates: ‘Sensitivity analyses based on allocation concealment and blinding of outcome measurement will be done as these factors are most associated with a bias in effect size (Moher 1998).’  **Review methods:** Blinding. We judged the following to be two covariates: ‘We carried out sensitivity analyses based on allocation concealment and blinding of outcome measurement as these factors are most associated with a bias in effect size’. Blinding: low versus unclear versus high risk.’  **Review results:** ‘low’; ‘unclear’; ‘high’.  **Type:** Methodological.  **Covariate summary:** Blinding. | **Method to choose**  **covariate:** Not reported.  **Where method reported:** NA.  **Rationale for choosing the covariate:** ‘Sensitivity analyses based on allocation concealment and blinding  of outcome measurement will be done as these factors are most  associated with a bias in effect size (Moher 1998).’  **Where rationale reported:** Protocol and review. | **Where reported:** Protocol and review.  **Reason not reported in review:** NA.  **Reason not reported in protocol:** NA.  **Labelled as ‘posthoc’ if not in protocol:** NA. | **Data type:** Categorical.  **Continuous covariate categorised:** NA.  **How determine it was categorised:** NA.  **Justification for categorising:** NA.  **Where justification reported:** NA.  **Categories reported:** Yes.  **Where categories reported:** Review only.  **Justification for categories:** Not reported.  **Where justification reported:** NA. | **Analysis type reported in protocol:** Sensitivity analysis.  **Analysis type reported in review:** Sensitivity analysis.  **Reason for changing mind:** NA.    **Analysis type in protocol according to glossary:** Sensitivity analysis.  **How determined the analysis type:** Says ‘Sensitivity analysis’. No reason to dispute.  **Analysis type in review according to glossary:** Subgroup analysis.  **How determined the analysis type:** Categories given.  **Analysed:** Yes.  **Reasons given (in the review) for not doing interaction analysis if planned:** NA.  **Reasons given for carrying out interaction analysis if they weren’t planned:** NA.  **Scenario in protocol:** Not reported.  **Rationale for planned scenario:** NA.  **Scenario in review (if reported specifically):** Not reported.  **Rationale for done scenario:** NA.  **Reason for changing mind:** NA. | **Method to detect interactions reported in protocol:** Not reported.  **Method to detect interactions reported in review:** Not reported.  **Reason for changing mind:** NA. |
| Gillies 2012 | **Protocol:** Individual or group therapy. We judged the following to be two covariates: ‘Therapies will also be separated into trauma-focused and non-trauma-focused and individual or group therapy where these categories are applicable.’ We judged the following to be two covariates: ‘The type of therapy: group versus individual therapy and single versus multiple therapy sessions.’  **Review methods:** ‘It was also proposed that subgroup analysis based on whether therapies were individual, parent, family or group-based would be done but insufficient data were available.’  **Review results:** Not analysed.  **Type:** Intervention.  **Covariate summary:** Type of intervention or control. | **Method to choose**  **covariate:** Not reported.  **Where method reported:** NA.  **Rationale for choosing the covariate:** Not reported.  **Where rationale reported:** NA. | **Where reported:** Protocol and review.  **Reason not reported in review:** NA.  **Reason not reported in protocol:** NA.  **Labelled as ‘posthoc’ if not in protocol:** NA. | **Data type:** Categorical.  **Continuous covariate categorised:** NA.  **How determine it was categorised:** NA.  **Justification for categorising:** NA.  **Where justification reported:** NA.  **Categories reported:** Yes..  **Where categories reported:** Protocol and review.  **Justification for categories:** Not reported.  **Where justification reported:** NA. | **Analysis type reported in protocol:** Subgroup analysis.  **Analysis type reported in review:** Subgroup analysis.  **Reason for changing mind:** NA.    **Analysis type in protocol according to glossary:** Subgroup analysis.  **How determined the analysis type:** Categories given.  **Analysis type in review according to glossary:** Subgroup analysis.  **How determined the analysis type:** Categories given.  **Analysed:** No.  **Reasons given (in the review) for not doing interaction analysis if planned:** ‘It was also proposed that subgroup analysis based on whether  therapies were individual, parent, family or group-based would be  done but insufficient data were available.’ In review  **Reasons given for carrying out interaction analysis if they weren’t planned:** NA.  **Scenario in protocol:** ‘if data are available’  **Rationale for planned scenario:** Not reported.  **Scenario in review (if reported specifically):** Not reported.  **Rationale for done scenario:** NA.  **Reason for changing mind:** NA. | **Method to detect interactions reported in protocol:** Not reported.  **Method to detect interactions reported in review:** Not reported.  **Reason for changing mind:** NA. |
| Gillies 2012 | **Protocol:** Not reported.  **Review methods: ‘**Type of therapy: CBT, narrative therapy, supportive counselling, interpersonal therapy, EMDR.’  **Review results:** ‘CBT’ versus ‘narrative’.  **Type:** Intervention.  **Covariate summary:** Type of intervention or control. | **Method to choose**  **covariate:** Not reported.  **Where method reported:** NA.  **Rationale for choosing the covariate:** Not reported.  **Where rationale reported:** NA. | **Where reported:** Review only.  **Reason not reported in review:** NA.  **Reason not reported in protocol:** Not reported.  **Labelled as ‘posthoc’ if not in protocol:** No. | **Data type:** Categorical.  **Continuous covariate categorised:** NA.  **How determine it was categorised:** NA.  **Justification for categorising:** NA.  **Where justification reported:** NA.  **Categories reported:** Yes..  **Where categories reported:** Review only.  **Justification for categories:** Not reported.  **Where justification reported:** NA. | **Analysis type reported in protocol:** Not reported.  **Analysis type reported in review:** Subgroup analysis.  **Reason for changing mind:** Not reported.    **Analysis type in protocol according to glossary:** Not reported.  **How determined the analysis type:** NA.  **Analysis type in review according to glossary:** Subgroup analysis.  **How determined the analysis type:** Categories given.  **Analysed:** Yes.  **Reasons given (in the review) for not doing interaction analysis if planned:** NA.  **Reasons given for carrying out interaction analysis if they weren’t planned:** Not reported.  **Scenario in protocol:** Not reported.  **Rationale for planned scenario:** NA.  **Scenario in review (if reported specifically):** Not reported.  **Rationale for done scenario:** NA.  **Reason for changing mind:** NA. | **Method to detect interactions reported in protocol:** Not reported.  **Method to detect interactions reported in review:** Not reported.  **Reason for changing mind:** NA. |
| Gillies 2012 | **Protocol:** Not reported.  **Review methods: ‘**Interpersonal versus non-interpersonal trauma’.  **Review results:** Not analysed.  **Type:** Patient.  **Covariate summary:** Disease characteristics. | **Method to choose**  **covariate:** Not reported.  **Where method reported:** NA.  **Rationale for choosing the covariate:** Not reported.  **Where rationale reported:** NA. | **Where reported:** Review only.  **Reason not reported in review:** NA.  **Reason not reported in protocol:** Not reported.  **Labelled as ‘posthoc’ if not in protocol:** Yes. | **Data type:** Categorical.  **Continuous covariate categorised:** NA.  **How determine it was categorised:** NA.  **Justification for categorising:** NA.  **Where justification reported:** NA.  **Categories reported:** Yes.  **Where categories reported:** Review only.  **Justification for categories:** Not reported.  **Where justification reported:** NA. | **Analysis type reported in protocol:** Not reported.  **Analysis type reported in review:** Subgroup analysis.  **Reason for changing mind:** Not reported.    **Analysis type in protocol according to glossary:** Not reported.  **How determined the analysis type:** NA.  **Analysis type in review according to glossary:** Subgroup analysis.  **How determined the analysis type:** Categories given.  **Analysed:** No.  **Reasons given (in the review) for not doing interaction analysis if planned:’** but insufficient information was provided  in each study to be able to conduct this analysis.’  **Reasons given for carrying out interaction analysis if they weren’t planned:** NA.  **Scenario in protocol:** Not reported.  **Rationale for planned scenario:** NA.  **Scenario in review (if reported specifically):** Not reported.  **Rationale for done scenario:** NA.  **Reason for changing mind:** NA. | **Method to detect interactions reported in protocol:** Not reported.  **Method to detect interactions reported in review:** Not reported.  **Reason for changing mind:** NA. |
| Gillies 2012 | **Protocol:** Single versus multiple therapy sessions. We judged the following to be two covariates: ‘The type of therapy: group versus individual therapy and single versus multiple therapy sessions.’  **Review methods:** Not reported.  **Review results:** Not analysed.  **Type:** Intervention.  **Covariate summary:** Type of intervention or control. | **Method to choose**  **covariate:** Not reported.  **Where method reported:** NA.  **Rationale for choosing the covariate:** Not reported.  **Where rationale reported:** NA. | **Where reported:** Protocol only.  **Reason not reported in review:** Not reported.  **Reason not reported in protocol:** NA.  **Labelled as ‘posthoc’ if not in protocol:** NA. | **Data type:** Continuous.  **Continuous covariate categorised:** Yes.  **How determine it was categorised:** Categories given.  **Justification for categorising:** Not reported.  **Where justification reported:** NA.  **Categories reported:** Yes..  **Where categories reported:** Protocol only.  **Justification for categories:** Not reported.  **Where justification reported:** NA. | **Analysis type reported in protocol:** Subgroup analysis.  **Analysis type reported in review:** Not reported.  **Reason for changing mind:** Not reported.    **Analysis type in protocol according to glossary:** Subgroup analysis.  **How determined the analysis type:** Categories given.  **Analysis type in review according to glossary:** Not reported.  **How determined the analysis type:** NA.  **Analysed:** No.  **Reasons given (in the review) for not doing interaction analysis if planned:** Not reported.  **Reasons given for carrying out interaction analysis if they weren’t planned:** NA.  **Scenario in protocol:** ‘if data are available’  **Rationale for planned scenario:** Not reported.  **Scenario in review (if reported specifically):** Not reported.  **Rationale for done scenario:** NA.  **Reason for changing mind:** NA. | **Method to detect interactions reported in protocol:** Not reported.  **Method to detect interactions reported in review:** Not reported.  **Reason for changing mind:** NA. |
| Gillies 2012 | **Protocol:** Trauma-focused and non-trauma-focused. We judged the following to be two covariates: ‘Therapies will also be separated into trauma-focused and non-trauma-focused and individual or group therapy where these categories are applicable.’  **Review methods:** Not reported.  **Review results:** Not analysed.  **Type:** Intervention.  **Covariate summary:** Type of intervention or control. | **Method to choose**  **covariate:** Not reported.  **Where method reported:** NA.  **Rationale for choosing the covariate:** Not reported.  **Where rationale reported:** NA. | **Where reported:** Protocol only.  **Reason not reported in review:** Not reported.  **Reason not reported in protocol:** NA.  **Labelled as ‘posthoc’ if not in protocol:** NA. | **Data type:** Categorical.  **Continuous covariate categorised:** NA.  **How determine it was categorised:** NA.  **Justification for categorising:** NA.  **Where justification reported:** NA.  **Categories reported:** Yes.  **Where categories reported:** Protocol only.  **Justification for categories:** Not reported.  **Where justification reported:** NA. | **Analysis type reported in protocol:** Unnamed analysis.  **Analysis type reported in review:** Not reported.  **Reason for changing mind:** Not reported..    **Analysis type in protocol according to glossary:** Stratifcation/subgroup analysis.  **How determined the analysis type:** Categories given.  **Analysis type in review according to glossary:** Not reported.  **How determined the analysis type:** NA.  **Analysed:** No.  **Reasons given (in the review) for not doing interaction analysis if planned:** Not reported.  **Reasons given for carrying out interaction analysis if they weren’t planned:** NA.  **Scenario in protocol:** Not reported.  **Rationale for planned scenario:** NA.  **Scenario in review (if reported specifically):** Not reported.  **Rationale for done scenario:** NA.  **Reason for changing mind:** NA. | **Method to detect interactions reported in protocol:** Not reported.  **Method to detect interactions reported in review:** Not reported.  **Reason for changing mind:** NA. |
| Gois 2013 | **Protocol:** ‘Age’.  **Review methods:** Not reported.  **Review results:** Not analysed.  **Type:** Patient.  **Covariate summary:** Demographics. | **Method to choose**  **covariate:** Not reported.  **Where method reported:** NA.  **Rationale for choosing the covariate:** Not reported.  **Where rationale reported:** NA. | **Where reported:** Protocol only.  **Reason not reported in review:** Not reported.  **Reason not reported in protocol:** NA.  **Labelled as ‘posthoc’ if not in protocol:** NA. | **Data type:** Continuous.  **Continuous covariate categorised:** Yes.  **How determine it was categorised:** Subgroup analysis planned.  **Justification for categorising:** Not reported.  **Where justification reported:** NA.  **Categories reported:** Not reported.  **Where categories reported:** NA.  **Justification for categories:** Not reported.  **Where justification reported:** NA. | **Analysis type reported in protocol:** Subgroup analysis.  **Analysis type reported in review:** Subgroup analysis.  **Reason for changing mind:** NA.    **Analysis type in protocol according to glossary:** Subgroup analysis.  **How determined the analysis type:** Says ‘Subgroup analysis’. No reason to dispute.  **Analysis type in review according to glossary:** Subgroup analysis.  **How determined the analysis type:** Says ‘Subgroup analysis’. No reason to dispute.  **Analysed:** No.  **Reasons given (in the review) for not doing interaction analysis if planned: ‘**Data were not available to perform subgroup analyses in this review.’  **Reasons given for carrying out interaction analysis if they weren’t planned:** NA.  **Scenario in protocol:** ‘ if data are available’.  **Rationale for planned scenario:** Not reported.  **Scenario in review (if reported specifically):** Not reported.  **Rationale for done scenario:** NA.  **Reason for changing mind:** NA. | **Method to detect interactions reported in protocol:** Not reported.  **Method to detect interactions reported in review:** Not reported.  **Reason for changing mind:** NA. |
| Gois 2013 | **Protocol:** ‘Analysis of all clinical trials and comparison with analysis of double blind studies only.’  **Review methods:** Not reported.  **Review results:** Not analysed.  **Type:** Methodological.  **Covariate summary:** Blinding. | **Method to choose**  **covariate:** Not reported.  **Where method reported:** NA.  **Rationale for choosing the covariate:** Not reported.  **Where rationale reported:** NA. | **Where reported:** Protocol only.  **Reason not reported in review:** Not reported.  **Reason not reported in protocol:** NA.  **Labelled as ‘posthoc’ if not in protocol:** NA. | **Data type:** Categorical.  **Continuous covariate categorised:** NA.  **How determine it was categorised:** NA.  **Justification for categorising:** NA.  **Where justification reported:** NA.  **Categories reported:** Yes.  **Where categories reported:** Protocol only.  **Justification for categories:** Not reported.  **Where justification reported:** NA. | **Analysis type reported in protocol:** Sensitivity analysis.  **Analysis type reported in review:** Sensitivity analysis.  **Reason for changing mind:** NA.    **Analysis type in protocol according to glossary:** Sensitivity analysis.  **How determined the analysis type:** Categories reported.  **Analysis type in review according to glossary:** Sensitivity analysis.  **How determined the analysis type:** Categories reported.  **Analysed:** No.  **Reasons given (in the review) for not doing interaction analysis if planned: ‘**Data were not available to perform sensitivity analysis in this review.’  **Reasons given for carrying out interaction analysis if they weren’t planned:** NA.  **Scenario in protocol:** Not reported.  **Rationale for planned scenario:** NA.  **Scenario in review (if reported specifically):** Not reported.  **Rationale for done scenario:** NA.  **Reason for changing mind:** NA. | **Method to detect interactions reported in protocol:** Not reported.  **Method to detect interactions reported in review:** Not reported.  **Reason for changing mind:** NA. |
| Gois 2013 | **Protocol:** ‘Analysis of all clinical trials with randomised allocation and comparison with analysis of studies with adequate allocation concealment only.’  **Review methods:** Not reported.  **Review results:** Not analysed.  **Type:** Methodological.  **Covariate summary:** Random generation. | **Method to choose**  **covariate:** Not reported.  **Where method reported:** NA.  **Rationale for choosing the covariate:** Not reported.  **Where rationale reported:** NA. | **Where reported:** Protocol only.  **Reason not reported in review:** Not reported.  **Reason not reported in protocol:** NA.  **Labelled as ‘posthoc’ if not in protocol:** NA. | **Data type:** Categorical.  **Continuous covariate categorised:** NA.  **How determine it was categorised:** NA.  **Justification for categorising:** NA.  **Where justification reported:** NA.  **Categories reported:** Yes.  **Where categories reported:** Protocol only.  **Justification for categories:** Not reported.  **Where justification reported:** NA. | **Analysis type reported in protocol:** Sensitivity analysis.  **Analysis type reported in review:** Sensitivity analysis.  **Reason for changing mind:** NA.    **Analysis type in protocol according to glossary:** Sensitivity analysis.  **How determined the analysis type:** Categories reported.  **Analysis type in review according to glossary:** Sensitivity analysis.  **How determined the analysis type:** Categories reported.  **Analysed:** No.  **Reasons given (in the review) for not doing interaction analysis if planned: ‘**Data were not available to perform sensitivity analysis in this review.’  **Reasons given for carrying out interaction analysis if they weren’t planned:** NA.  **Scenario in protocol:** Not reported.  **Rationale for planned scenario:** NA.  **Scenario in review (if reported specifically):** Not reported.  **Rationale for done scenario:** NA.  **Reason for changing mind:** NA. | **Method to detect interactions reported in protocol:** Not reported.  **Method to detect interactions reported in review:** Not reported.  **Reason for changing mind:** NA. |
| Gois 2013 | **Protocol:** ‘Each uric acid-lowering drug will be analysed separately’.  **Review methods:** Not reported.  **Review results:** Not analysed.  **Type:** Intervention.  **Covariate summary:** Type of intervention or control. | **Method to choose**  **covariate:** Not reported.  **Where method reported:** NA.  **Rationale for choosing the covariate:** Not reported.  **Where rationale reported:** NA. | **Where reported:** Protocol only.  **Reason not reported in review:** Not reported.  **Reason not reported in protocol:** NA.  **Labelled as ‘posthoc’ if not in protocol:** NA. | **Data type:** Categorical.  **Continuous covariate categorised:** NA.  **How determine it was categorised:** NA.  **Justification for categorising:** NA.  **Where justification reported:** NA.  **Categories reported:** Not reported.  **Where categories reported:** NA.  **Justification for categories:** Not reported.  **Where justification reported:** NA. | **Analysis type reported in protocol:** Unnamed analysis.  **Analysis type reported in review:** Not reported.  **Reason for changing mind:** Not reported    **Analysis type in protocol according to glossary:** Stratification/subgroup analysis.  **How determined the analysis type:** Categories reported.  **Analysis type in review according to glossary:** Not reported.  **How determined the analysis type:** NA.  **Analysed:** No.  **Reasons given (in the review) for not doing interaction analysis if planned:** Not reported (presumably because one trial).  **Reasons given for carrying out interaction analysis if they weren’t planned:** NA.  **Scenario in protocol:** Not reported.  **Rationale for planned scenario:** NA.  **Scenario in review (if reported specifically):** Not reported.  **Rationale for done scenario:** NA.  **Reason for changing mind:** NA. | **Method to detect interactions reported in protocol:** Not reported.  **Method to detect interactions reported in review:** Not reported.  **Reason for changing mind:** NA. |
| Gois 2013 | **Protocol:** ‘Ethnicity’.  **Review methods:** Not reported.  **Review results:** Not analysed.  **Type:** Patient.  **Covariate summary:** Demographics. | **Method to choose**  **covariate:** Not reported.  **Where method reported:** NA.  **Rationale for choosing the covariate:** Not reported.  **Where rationale reported:** NA. | **Where reported:** Protocol only.  **Reason not reported in review:** Not reported.  **Reason not reported in protocol:** NA.  **Labelled as ‘posthoc’ if not in protocol:** NA. | **Data type:** Categorical.  **Continuous covariate categorised:** NA.  **How determine it was categorised:** NA.  **Justification for categorising:** NA.  **Where justification reported:** NA.  **Categories reported:** Not reported.  **Where categories reported:** NA.  **Justification for categories:** Not reported.  **Where justification reported:** NA. | **Analysis type reported in protocol:** Subgroup analysis.  **Analysis type reported in review:** Subgroup analysis.  **Reason for changing mind:** NA.    **Analysis type in protocol according to glossary:** Subgroup analysis.  **How determined the analysis type:** Says ‘Subgroup analysis’. No reason to dispute.  **Analysis type in review according to glossary:** Subgroup analysis.  **How determined the analysis type:** Says ‘Subgroup analysis’. No reason to dispute.  **Analysed:** No.  **Reasons given (in the review) for not doing interaction analysis if planned: ‘**Data were not available to perform subgroup analyses in this review.’  **Reasons given for carrying out interaction analysis if they weren’t planned:** NA.  **Scenario in protocol:** ‘ if data are available’.  **Rationale for planned scenario:** Not reported.  **Scenario in review (if reported specifically):** Not reported.  **Rationale for done scenario:** NA.  **Reason for changing mind:** NA. | **Method to detect interactions reported in protocol:** Not reported.  **Method to detect interactions reported in review:** Not reported.  **Reason for changing mind:** NA. |
| Gois 2013 | **Protocol:** ‘Exclusion of non-published studies.’  **Review methods:** Not reported.  **Review results:** Not analysed.  **Type:** Methodological.  **Covariate summary:** Publication status. | **Method to choose**  **covariate:** Not reported.  **Where method reported:** NA.  **Rationale for choosing the covariate:** Not reported.  **Where rationale reported:** NA. | **Where reported:** Protocol only.  **Reason not reported in review:** Not reported.  **Reason not reported in protocol:** NA.  **Labelled as ‘posthoc’ if not in protocol:** NA. | **Data type:** Categorical.  **Continuous covariate categorised:** NA.  **How determine it was categorised:** NA.  **Justification for categorising:** NA.  **Where justification reported:** NA.  **Categories reported:** Yes.  **Where categories reported:** Protocol only.  **Justification for categories:** Not reported.  **Where justification reported:** NA. | **Analysis type reported in protocol:** Sensitivity analysis.  **Analysis type reported in review:** Sensitivity analysis.  **Reason for changing mind:** NA.    **Analysis type in protocol according to glossary:** Sensitivity analysis.  **How determined the analysis type:** Categories reported.  **Analysis type in review according to glossary:** Sensitivity analysis.  **How determined the analysis type:** Categories reported.  **Analysed:** No.  **Reasons given (in the review) for not doing interaction analysis if planned: ‘**Data were not available to perform sensitivity analysis in this review.’  **Reasons given for carrying out interaction analysis if they weren’t planned:** NA.  **Scenario in protocol:** Not reported.  **Rationale for planned scenario:** NA.  **Scenario in review (if reported specifically):** Not reported.  **Rationale for done scenario:** NA.  **Reason for changing mind:** NA. | **Method to detect interactions reported in protocol:** Not reported.  **Method to detect interactions reported in review:** Not reported.  **Reason for changing mind:** NA. |
| Gois 2013 | **Protocol:** ‘Exclusion of studies with poor methodological quality (according to the Cochrane collaboration´ s tool).’  **Review methods:** Not reported.  **Review results:** Not analysed.  **Type:** Methodological.  **Covariate summary:** Quality. | **Method to choose**  **covariate:** Not reported.  **Where method reported:** NA.  **Rationale for choosing the covariate:** Not reported.  **Where rationale reported:** NA. | **Where reported:** Protocol only.  **Reason not reported in review:** Not reported.  **Reason not reported in protocol:** NA.  **Labelled as ‘posthoc’ if not in protocol:** NA. | **Data type:** Categorical.  **Continuous covariate categorised:** NA.  **How determine it was categorised:** NA.  **Justification for categorising:** NA.  **Where justification reported:** NA.  **Categories reported:** Yes.  **Where categories reported:** Protocol only.  **Justification for categories:** Not reported.  **Where justification reported:** NA. | **Analysis type reported in protocol:** Sensitivity analysis.  **Analysis type reported in review:** Sensitivity analysis.  **Reason for changing mind:** NA.    **Analysis type in protocol according to glossary:** Sensitivity analysis.  **How determined the analysis type:** Categories reported.  **Analysis type in review according to glossary:** Sensitivity analysis.  **How determined the analysis type:** Categories reported.  **Analysed:** No.  **Reasons given (in the review) for not doing interaction analysis if planned: ‘**Data were not available to perform sensitivity analysis in this review.’  **Reasons given for carrying out interaction analysis if they weren’t planned:** NA.  **Scenario in protocol:** Not reported.  **Rationale for planned scenario:** NA.  **Scenario in review (if reported specifically):** Not reported.  **Rationale for done scenario:** NA.  **Reason for changing mind:** NA. | **Method to detect interactions reported in protocol:** Not reported.  **Method to detect interactions reported in review:** Not reported.  **Reason for changing mind:** NA. |
| Gois 2013 | **Protocol:** ‘Gender’.  **Review methods:** Not reported.  **Review results:** Not analysed.  **Type:** Patient.  **Covariate summary:** Demographics. | **Method to choose**  **covariate:** Not reported.  **Where method reported:** NA.  **Rationale for choosing the covariate:** Not reported.  **Where rationale reported:** NA. | **Where reported:** Protocol only.  **Reason not reported in review:** Not reported.  **Reason not reported in protocol:** NA.  **Labelled as ‘posthoc’ if not in protocol:** NA. | **Data type:** Categorical.  **Continuous covariate categorised:** NA.  **How determine it was categorised:** NA.  **Justification for categorising:** NA.  **Where justification reported:** NA.  **Categories reported:** Not reported.  **Where categories reported:** NA.  **Justification for categories:** Not reported.  **Where justification reported:** NA. | **Analysis type reported in protocol:** Subgroup analysis.  **Analysis type reported in review:** Subgroup analysis.  **Reason for changing mind:** NA.    **Analysis type in protocol according to glossary:** Subgroup analysis.  **How determined the analysis type:** Says ‘Subgroup analysis’. No reason to dispute.  **Analysis type in review according to glossary:** Subgroup analysis.  **How determined the analysis type:** Says ‘Subgroup analysis’. No reason to dispute.  **Analysed:** No.  **Reasons given (in the review) for not doing interaction analysis if planned: ‘**Data were not available to perform subgroup analyses in this review.’  **Reasons given for carrying out interaction analysis if they weren’t planned:** NA.  **Scenario in protocol:** ‘ if data are available’.  **Rationale for planned scenario:** Not reported.  **Scenario in review (if reported specifically):** Not reported.  **Rationale for done scenario:** NA.  **Reason for changing mind:** NA. | **Method to detect interactions reported in protocol:** Not reported.  **Method to detect interactions reported in review:** Not reported.  **Reason for changing mind:** NA. |
| Gois 2013 | **Protocol:** ‘Inclusion or exclusion of studies that presented certain ambiguities in the inclusion criteria.’  **Review methods:** Not reported.  **Review results:** Not analysed.  **Type:** Patient.  **Covariate summary:** Inclusion criteria. | **Method to choose**  **covariate:** Not reported.  **Where method reported:** NA.  **Rationale for choosing the covariate:** Not reported.  **Where rationale reported:** NA. | **Where reported:** Protocol only.  **Reason not reported in review:** Not reported.  **Reason not reported in protocol:** NA.  **Labelled as ‘posthoc’ if not in protocol:** NA. | **Data type:** Categorical.  **Continuous covariate categorised:** NA.  **How determine it was categorised:** NA.  **Justification for categorising:** NA.  **Where justification reported:** NA.  **Categories reported:** Yes.  **Where categories reported:** Protocol only.  **Justification for categories:** Not reported.  **Where justification reported:** NA. | **Analysis type reported in protocol:** Sensitivity analysis.  **Analysis type reported in review:** Sensitivity analysis.  **Reason for changing mind:** NA.    **Analysis type in protocol according to glossary:** Sensitivity analysis.  **How determined the analysis type:** Categories reported.  **Analysis type in review according to glossary:** Sensitivity analysis.  **How determined the analysis type:** Categories reported.  **Analysed:** No.  **Reasons given (in the review) for not doing interaction analysis if planned: ‘**Data were not available to perform sensitivity analysis in this review.’  **Reasons given for carrying out interaction analysis if they weren’t planned:** NA.  **Scenario in protocol:** Not reported.  **Rationale for planned scenario:** NA.  **Scenario in review (if reported specifically):** Not reported.  **Rationale for done scenario:** NA.  **Reason for changing mind:** NA. | **Method to detect interactions reported in protocol:** Not reported.  **Method to detect interactions reported in review:** Not reported.  **Reason for changing mind:** NA. |
| Gois 2013 | **Protocol:** ‘Quality of trial’.  **Review methods:** Not reported.  **Review results:** Not analysed.  **Type:** Methodological.  **Covariate summary:** Quality. | **Method to choose**  **covariate:** Not reported.  **Where method reported:** NA.  **Rationale for choosing the covariate:** Not reported.  **Where rationale reported:** NA. | **Where reported:** Protocol only.  **Reason not reported in review:** Not reported.  **Reason not reported in protocol:** NA.  **Labelled as ‘posthoc’ if not in protocol:** NA. | **Data type:** Categorical.  **Continuous covariate categorised:** NA.  **How determine it was categorised:** NA.  **Justification for categorising:** NA.  **Where justification reported:** NA.  **Categories reported:** Not reported.  **Where categories reported:** NA.  **Justification for categories:** Not reported.  **Where justification reported:** NA. | **Analysis type reported in protocol:** Subgroup analysis.  **Analysis type reported in review:** Subgroup analysis.  **Reason for changing mind:** NA.    **Analysis type in protocol according to glossary:** Subgroup analysis.  **How determined the analysis type:** Says ‘Subgroup analysis’. No reason to dispute.  **Analysis type in review according to glossary:** Subgroup analysis.  **How determined the analysis type:** Says ‘Subgroup analysis’. No reason to dispute.  **Analysed:** No.  **Reasons given (in the review) for not doing interaction analysis if planned: ‘**Data were not available to perform subgroup analyses in this review.’  **Reasons given for carrying out interaction analysis if they weren’t planned:** NA.  **Scenario in protocol:** ‘ if data are available’.  **Rationale for planned scenario:** Not reported.  **Scenario in review (if reported specifically):** Not reported.  **Rationale for done scenario:** NA.  **Reason for changing mind:** NA. | **Method to detect interactions reported in protocol:** Not reported.  **Method to detect interactions reported in review:** Not reported.  **Reason for changing mind:** NA. |
| Gois 2013 | **Protocol:** ‘Severity of hypertension at baseline’.  **Review methods:** Not reported.  **Review results:** Not analysed.  **Type:** Patient.  **Covariate summary:** Disease characteristics. | **Method to choose**  **covariate:** Not reported.  **Where method reported:** NA.  **Rationale for choosing the covariate:** Not reported.  **Where rationale reported:** NA. | **Where reported:** Protocol only.  **Reason not reported in review:** Not reported.  **Reason not reported in protocol:** NA.  **Labelled as ‘posthoc’ if not in protocol:** NA. | **Data type:** Continuous.  **Continuous covariate categorised:** Yes.  **How determine it was categorised:** Subgroup analysis planned.  **Justification for categorising:** Not reported.  **Where justification reported:** NA.  **Categories reported:** Not reported.  **Where categories reported:** NA.  **Justification for categories:** Not reported.  **Where justification reported:** NA. | **Analysis type reported in protocol:** Subgroup analysis.  **Analysis type reported in review:** Subgroup analysis.  **Reason for changing mind:** NA.    **Analysis type in protocol according to glossary:** Subgroup analysis.  **How determined the analysis type:** Says ‘Subgroup analysis’. No reason to dispute.  **Analysis type in review according to glossary:** Subgroup analysis.  **How determined the analysis type:** Says ‘Subgroup analysis’. No reason to dispute.  **Analysed:** No.  **Reasons given (in the review) for not doing interaction analysis if planned: ‘**Data were not available to perform subgroup analyses in this review.’  **Reasons given for carrying out interaction analysis if they weren’t planned:** NA.  **Scenario in protocol:** ‘ if data are available’.  **Rationale for planned scenario:** Not reported.  **Scenario in review (if reported specifically):** Not reported.  **Rationale for done scenario:** NA.  **Reason for changing mind:** NA. | **Method to detect interactions reported in protocol:** Not reported.  **Method to detect interactions reported in review:** Not reported.  **Reason for changing mind:** NA. |
| Goldenberg 2013 | **Protocol:** ‘Dose of probiotic.’  **Review methods:** ‘Dosage of probiotic’.  **Review results:** Not analysed.  **Type:** Intervention.  **Covariate summary:** Dose. | **Method to choose**  **covariate:** Not reported.  **Where method reported:** NA.  **Rationale for choosing the covariate:** ‘with an expected larger effect in trials  administering an increased dose (Johnston 2006; Johnston 2011).  **‘**  **Where rationale reported:** Review only. | **Where reported:** Protocol and review.  **Reason not reported in review:** NA.  **Reason not reported in protocol:** NA.  **Labelled as ‘posthoc’ if not in protocol:** NA. | **Data type:** Continuous.  **Continuous covariate categorised:** Unclear.  **How determine it was categorised:** Meta-regression planned.  **Justification for categorising:** NA.  **Where justification reported:** NA.  **Categories reported:** Not reported.  **Where categories reported:** NA.  **Justification for categories:** Not reported.  **Where justification reported:** NA. | **Analysis type reported in protocol:** Meta-regression.  **Analysis type reported in review:** Meta-regression and subgroup analysis.  **Reason for changing mind:** Not reported.    **Analysis type in protocol according to glossary:** Meta-regression.  **How determined the analysis type:** Says ‘Meta-regression’. No reason to dispute.  **Analysis type in review according to glossary:** Meta-regression and subgroup analysis.  **How determined the analysis type:** Says ‘Meta-regression and subgroup analysis’. No reason to dispute.  **Analysed:** No.  **Reasons given (in the review) for not doing interaction analysis if planned:** Not reported.  **Reasons given for carrying out interaction analysis if they weren’t planned:** NA.  **Scenario in protocol: ‘**Sources of heterogeneity will  be explored through meta-regression for meta-analyses including  a sufficient number of trials (k > 10).’  **Rationale for planned scenario:** Not reported.  **Scenario in review (if reported specifically):** Not reported.  **Rationale for done scenario:** NA.  **Reason for changing mind:** Not reported. | **Method to detect interactions reported in protocol:** Not reported.  **Method to detect interactions reported in review: ‘**To evaluate the credibility of our  subgroup analyses we used pre-specified criteria, including a test  for interaction (Sun 2010). For continuous variables such as probiotic  dose, we used random-effects meta-regression (Thompson  2002).’  **Reason for changing mind:** Not reported. |
| Goldenberg 2013 | **Protocol:** ‘Probiotic stain’.  **Review methods:** Not reported.  **Review results:** Not analysed.  **Type:** Intervention.  **Covariate summary:** Type of intervention or control. | **Method to choose**  **covariate:** Not reported.  **Where method reported:** NA.  **Rationale for choosing the covariate:** Not reported.  **Where rationale reported:** NA. | **Where reported:** Protocol only.  **Reason not reported in review:** Not reported.  **Reason not reported in protocol:** NA.  **Labelled as ‘posthoc’ if not in protocol:** NA. | **Data type:** Categorical.  **Continuous covariate categorised:** NA.  **How determine it was categorised:** NA.  **Justification for categorising:** NA.  **Where justification reported:** NA.  **Categories reported:** Not reported.  **Where categories reported:** NA.  **Justification for categories:** Not reported.  **Where justification reported:** NA. | **Analysis type reported in protocol:** Meta-regression.  **Analysis type reported in review:** Not reported.  **Reason for changing mind:** Not reported.    **Analysis type in protocol according to glossary:** Meta-regression.  **How determined the analysis type:** Says ‘Meta-regression’. No reason to dispute.  **Analysis type in review according to glossary:** Not reported.  **How determined the analysis type:** NA.  **Analysed:** No.  **Reasons given (in the review) for not doing interaction analysis if planned:** Not reported.  **Reasons given for carrying out interaction analysis if they weren’t planned:** NA.  **Scenario in protocol: ‘**Sources of heterogeneity will  be explored through meta-regression for meta-analyses including  a sufficient number of trials (k > 10).’  **Rationale for planned scenario:** Not reported.  **Scenario in review (if reported specifically):** Not reported.  **Rationale for done scenario:** NA.  **Reason for changing mind:** Not reported. | **Method to detect interactions reported in protocol:** Not reported.  **Method to detect interactions reported in review: ‘**To evaluate the credibility of our  subgroup analyses we used pre-specified criteria, including a test  for interaction (Sun 2010).’  **Reason for changing mind:** Not reported. |
| Goldenberg 2013 | **Protocol:** Not reported.  **Review methods: ‘**Adult versus pediatric population’.  **Review results:** ‘Adult studies’ versus ‘pediatric studies’.  **Type:** Patient.  **Covariate summary:** Demographics. | **Method to choose**  **covariate:** Not reported.  **Where method reported:** NA.  **Rationale for choosing the covariate:** with a postulated larger effect  in adults for CDAD and children for AAD (Hempel 2012);  **Where rationale reported:** Review only. | **Where reported:** Review only.  **Reason not reported in review:** NA.  **Reason not reported in protocol:** Not reported.  **Labelled as ‘posthoc’ if not in protocol:** No. ‘Apriori’. | **Data type:** Continuous.  **Continuous covariate categorised:** Yes.  **How determine it was categorised:** Categories given.  **Justification for categorising:** Not reported.  **Where justification reported:** NA.  **Categories reported:** Yes**.**  **Where categories reported:** Review only.  **Justification for categories: ‘**Adult versus pediatric population, with a postulated larger effect in adults for CDAD and children for AAD (Hempel 2012).’  **Where justification reported:** Review only. | **Analysis type reported in protocol:** Not reported.  **Analysis type reported in review:** Subgroup analysis.  **Reason for changing mind:** Not reported.    **Analysis type in protocol according to glossary:** Not reported.  **How determined the analysis type:** NA.  **Analysis type in review according to glossary:** subgroup analysis.  **How determined the analysis type:** Results reported.  **Analysed:** Yes.  **Reasons given (in the review) for not doing interaction analysis if planned:** NA.  **Reasons given for carrying out interaction analysis if they weren’t planned:** Not reported.  **Scenario in protocol:** Not reported.  **Rationale for planned scenario:** NA.  **Scenario in review (if reported specifically):** Not reported.  **Rationale for done scenario:** NA.  **Reason for changing mind:** Not reported. | **Method to detect interactions reported in protocol:** Not reported.  **Method to detect interactions reported in review: ‘**To evaluate the credibility of our  subgroup analyses we used pre-specified criteria, including a test  for interaction (Sun 2010).’  **Reason for changing mind:** Not reported. |
| Goldenberg 2013 | **Protocol:** Not reported.  **Review methods:** ‘Inpatients versus outpatients’.  **Review results: ‘**Inpatient’; ‘outpatient’; ‘mixed’.  **Type:** Patient.  **Covariate summary:** Setting. | **Method to choose**  **covariate:** Not reported.  **Where method reported:** NA.  **Rationale for choosing the covariate:** with a postulated  larger effect among inpatients  **Where rationale reported:** Review only. | **Where reported:** Review only.  **Reason not reported in review:** NA.  **Reason not reported in protocol:** Post hoc, on the recommendation of a peer reviewer.  **Labelled as ‘posthoc’ if not in protocol:** Yes. | **Data type:** Categorical.  **Continuous covariate categorised:** NA.  **How determine it was categorised:** NA.  **Justification for categorising:** NA.  **Where justification reported:** NA.  **Categories reported:** Yes.  **Where categories reported:** Review only.  **Justification for categories: ‘**Inpatients versus outpatients, with a postulated larger effect among inpatients’  **Where justification reported:** Review only. | **Analysis type reported in protocol:** Not reported.  **Analysis type reported in review:** Subgroup analysis.  **Reason for changing mind:** Not reported.    **Analysis type in protocol according to glossary:** Not reported.  **How determined the analysis type:** NA.  **Analysis type in review according to glossary:** subgroup analysis.  **How determined the analysis type:** Results reported.  **Analysed:** Yes.  **Reasons given (in the review) for not doing interaction analysis if planned:** NA..  **Reasons given for carrying out interaction analysis if they weren’t planned:** Post hoc, on the recommendation of a peer reviewer.  **Scenario in protocol:** Not reported.  **Rationale for planned scenario:** NA.  **Scenario in review (if reported specifically):** Not reported.  **Rationale for done scenario:** NA.  **Reason for changing mind:** Not reported. | **Method to detect interactions reported in protocol:** Not reported.  **Method to detect interactions reported in review: ‘**To evaluate the credibility of our  subgroup analyses we used pre-specified criteria, including a test  for interaction (Sun 2010).’  **Reason for changing mind:** Not reported. |
| Goldenberg 2013 | **Protocol:** Not reported.  **Review methods: ‘**Probiotic species’.  **Review results: ‘**Lactobacillus GG; S. boulardii; L. acidophilus + L. Casei’; ‘L. acidophilus + B. Bifidum L. Acidophilus’; ‘L. acidophilus + L. bulgaricus + B. bifidum + S. Thermophiles’; ‘B. breve + B. Longum +B. infantis + L. acidophilus +L. plantarum + L. paracasei +L. bulgaricus + S. Thermophiles’; ‘L. casei + L. bulgaris + S. Thermophiles’; ‘L. Plantarum’; ‘Lactobacillus GG + L. acidophilus + B. Animalis’.  ‘Lactobacillus GG’ versus ‘ S. boulardii’.  ‘Lactobacillus GG’ versus ‘L. acidophilus + L. Casei’.  **Type:** Intervention.  **Covariate summary:** Type of intervention or control. | **Method to choose**  **covariate:** Not reported.  **Where method reported:** NA.  **Rationale for choosing the covariate:** ‘with a larger effect  expected in trials of S. boulardii or L. rhamnosus (Johnston  2011).  **‘**  **Where rationale reported:** Review only. | **Where reported:** Review only.  **Reason not reported in review:** NA.  **Reason not reported in protocol:** Not reported.  **Labelled as ‘posthoc’ if not in protocol:** No. ‘Apriori’. | **Data type:** Categorical.  **Continuous covariate categorised:** NA.  **How determine it was categorised:** NA.  **Justification for categorising:** NA.  **Where justification reported:** NA.  **Categories reported:** Yes.  **Where categories reported:** Review only.  **Justification for categories:** Not reported.  **Where justification reported:** NA. | **Analysis type reported in protocol:** Not reported.  **Analysis type reported in review:** Subgroup analysis.  **Reason for changing mind:** Not reported.    **Analysis type in protocol according to glossary:** Not reported.  **How determined the analysis type:** NA.  **Analysis type in review according to glossary:** subgroup analysis.  **How determined the analysis type:** Results reported.  **Analysed:** Yes.  **Reasons given (in the review) for not doing interaction analysis if planned:** NA..  **Reasons given for carrying out interaction analysis if they weren’t planned:** Not reported.  **Scenario in protocol:** Not reported.  **Rationale for planned scenario:** NA.  **Scenario in review (if reported specifically):** Not reported.  **Rationale for done scenario:** NA.  **Reason for changing mind:** Not reported. | **Method to detect interactions reported in protocol:** Not reported.  **Method to detect interactions reported in review: ‘**To evaluate the credibility of our  subgroup analyses we used pre-specified criteria, including a test  for interaction (Sun 2010).’  **Reason for changing mind:** Not reported. |
| Goldenberg 2013 | **Protocol:** Not reported.  **Review methods: ‘**The risk of bias’.  **Review results:** Risk of bias. ‘Low risk of bias’; ‘high or unclear risk of bias’.  **Type:** Methodological.  **Covariate summary:** Risk of bias. | **Method to choose**  **covariate:** Not reported.  **Where method reported:** NA.  **Rationale for choosing the covariate:** with an expected larger effect in trials at high or  unclear risk of bias versus trials at low risk of bias (Higgins 2011).  **Where rationale reported:** Review only. | **Where reported:** Review only.  **Reason not reported in review:** NA.  **Reason not reported in protocol:** Not reported.  **Labelled as ‘posthoc’ if not in protocol:** No. ‘Apriori’. | **Data type:** Categorical.  **Continuous covariate categorised:** NA.  **How determine it was categorised:** NA.  **Justification for categorising:** NA.  **Where justification reported:** NA.  **Categories reported:** Yes.  **Where categories reported:** Review only.  **Justification for categories: ‘**The risk of bias, with an expected larger effect in trials at high or unclear risk of bias versus trials at low risk of bias (Higgins 2011).’  **Where justification reported:** Review only. | **Analysis type reported in protocol:** Not reported.  **Analysis type reported in review:** Subgroup analysis.  **Reason for changing mind:** Not reported.    **Analysis type in protocol according to glossary:** Not reported.  **How determined the analysis type:** NA.  **Analysis type in review according to glossary:** subgroup analysis.  **How determined the analysis type:** Results reported.  **Analysed:** Yes.  **Reasons given (in the review) for not doing interaction analysis if planned:** NA..  **Reasons given for carrying out interaction analysis if they weren’t planned:** Not reported.  **Scenario in protocol:** Not reported.  **Rationale for planned scenario:** NA.  **Scenario in review (if reported specifically):** Not reported.  **Rationale for done scenario:** NA.  **Reason for changing mind:** Not reported. | **Method to detect interactions reported in protocol:** Not reported.  **Method to detect interactions reported in review: ‘**To evaluate the credibility of our  subgroup analyses we used pre-specified criteria, including a test  for interaction (Sun 2010).’  **Reason for changing mind:** Not reported. |
| Gower 2013 | **Protocol: ‘**Comparisons of interest will be: (1) any prophylaxis versus no prophylaxis; (2) preoperative versus postoperative or intraoperative prophylaxis or combinations; (3) comparisons of specific antibiotics used in included trials; (4) mode of delivery.’  **Review methods: ‘**Comparisons of interest included: 1. Any prophylaxis versus no prophylaxis; 2. Preoperative versus postoperative or intraoperative prophylaxis or combinations; 3. Specific antibiotics used in included trials; 4. Mode of perioperative antibiotic delivery.’  **Review results:** ‘Perioperative prophylaxis versus no prophylaxis’; ‘Comparisons of specific antibiotics or combinations of antibiotics’; ‘Mode of antibiotic delivery’ (in table).  **Type:** Intervention.  **Covariate summary:** Type of intervention or control. | **Method to choose**  **covariate:** Not reported.  **Where method reported:** NA.  **Rationale for choosing the covariate:** Not reported.  **Where rationale reported:** NA. | **Where reported:** Protocol and review.  **Reason not reported in review:** NA.  **Reason not reported in protocol:** NA.  **Labelled as ‘posthoc’ if not in protocol:** NA. | **Data type:** Categorical.  **Continuous covariate categorised:** NA.  **How determine it was categorised:** NA.  **Justification for categorising:** NA.  **Where justification reported:** NA.  **Categories reported:** Yes.  **Where categories reported:** Protocol and review.  **Justification for categories:** Not reported.  **Where justification reported:** NA. | **Analysis type reported in protocol:** Unnamed analysis.  **Analysis type reported in review:** Unnamed analysis.  **Reason for changing mind:** NA.    **Analysis type in protocol according to glossary:** Stratification/subgroup analysis.  **How determined the analysis type:** Categories given.  **Analysis type in review according to glossary:** Stratification/subgroup analysis.  **How determined the analysis type:** Categories given.  **Analysed:** Yes.  **Reasons given (in the review) for not doing interaction analysis if planned:** NA.  **Reasons given for carrying out interaction analysis if they weren’t planned:** NA.  **Scenario in protocol:** Not reported.  **Rationale for planned scenario:** NA.  **Scenario in review (if reported specifically):** Not reported.  **Rationale for done scenario:** NA.  **Reason for changing mind:** NA. | **Method to detect interactions reported in protocol:** Not reported.  **Method to detect interactions reported in review:** Not reported.  **Reason for changing mind:** NA. |
| Gower 2013 | **Protocol:** ‘Duration of follow-up’.  **Review methods:** Not reported.  **Review results:** Not analysed.  **Type:** Methodological.  **Covariate summary:** Length of follow up. | **Method to choose**  **covariate:** Not reported.  **Where method reported:** NA.  **Rationale for choosing the covariate:** Not reported.  **Where rationale reported:** NA. | **Where reported:** Protocol only.  **Reason not reported in review:** Not reported.  **Reason not reported in protocol:** NA.  **Labelled as ‘posthoc’ if not in protocol:** NA | **Data type:** Continuous.  **Continuous covariate categorised:** Yes.  **How determine it was categorised:** Subgroup analysis planned.  **Justification for categorising:** Not reported.  **Where justification reported:** NA.  **Categories reported:** Not reported.  **Where categories reported:** NA.  **Justification for categories:** Not reported.  **Where justification reported:** NA. | **Analysis type reported in protocol:** Subgroup analysis.  **Analysis type reported in review:** Not reported.  **Reason for changing mind:** Not reported.  **Analysis type in protocol according to glossary:** Subgroup analysis.  **How determined the analysis type:** Says ‘Subgroup analysis.’ No reason to dispute.  **Analysis type in review according to glossary:** Not reported.  **How determined the analysis type:** NA.  **Analysed:** No.  **Reasons given (in the review) for not doing interaction analysis if planned:** Not reported (presumably because no meta-analysis).  **Reasons given for carrying out interaction analysis if they weren’t planned:** NA.  **Scenario in protocol:** ‘Where substantial heterogeneity exists, sub group analysis will be performed’.  **Rationale for planned scenario:** Not reported.  **Scenario in review (if reported specifically):** Not reported.  **Rationale for done scenario:** NA.  **Reason for changing mind:** NA. | **Method to detect interactions reported in protocol:** Not reported.  **Method to detect interactions reported in review:** Not reported.  **Reason for changing mind:** NA. |
| Gower 2013 | **Protocol:** ‘Exclusion of studies with lower methodological quality.’  **Review methods:** Not reported.  **Review results:** Not analysed.  **Type:** Methodological.  **Covariate summary:** Quality. | **Method to choose**  **covariate:** Not reported.  **Where method reported:** NA.  **Rationale for choosing the covariate:** Not reported.  **Where rationale reported:** NA. | **Where reported:** Protocol only.  **Reason not reported in review:** Not reported.  **Reason not reported in protocol:** NA.  **Labelled as ‘posthoc’ if not in protocol:** NA | **Data type:** Categorical.  **Continuous covariate categorised:** NA.  **How determine it was categorised:** NA.  **Justification for categorising:** NA.  **Where justification reported:** NA.  **Categories reported:** Yes.  **Where categories reported:** Protocol only.  **Justification for categories:** Not reported.  **Where justification reported:** NA. | **Analysis type reported in protocol:** Sensitivity analysis.  **Analysis type reported in review:** Not reported.  **Reason for changing mind:** not reported.    **Analysis type in protocol according to glossary:** Sensitivity analysis.  **How determined the analysis type:**’ exclusion’.  **Analysis type in review according to glossary:** Not reported.  **How determined the analysis type:** NA.  **Analysed:** No.  **Reasons given (in the review) for not doing interaction analysis if planned:** Not reported (presumably because no meta-analysis).  **Reasons given for carrying out interaction analysis if they weren’t planned:** NA.  **Scenario in protocol:** Not reported.  **Rationale for planned scenario:** NA.  **Scenario in review (if reported specifically):** Not reported.  **Rationale for done scenario:** NA.  **Reason for changing mind:** NA. | **Method to detect interactions reported in protocol:** Not reported.  **Method to detect interactions reported in review:** Not reported.  **Reason for changing mind:** NA. |
| Gower 2013 | **Protocol:** ‘Exclusion of unpublished studies.’  **Review methods:** Not reported.  **Review results:** Not analysed.  **Type:** Methodological.  **Covariate summary:** Publication status. | **Method to choose**  **covariate:** Not reported.  **Where method reported:** NA.  **Rationale for choosing the covariate:** Not reported.  **Where rationale reported:** NA. | **Where reported:** Protocol only.  **Reason not reported in review:** Not reported.  **Reason not reported in protocol:** NA.  **Labelled as ‘posthoc’ if not in protocol:** NA | **Data type:** Categorical.  **Continuous covariate categorised:** NA.  **How determine it was categorised:** NA.  **Justification for categorising:** NA.  **Where justification reported:** NA.  **Categories reported:** Yes.  **Where categories reported:** Protocol only.  **Justification for categories:** Not reported.  **Where justification reported:** NA. | **Analysis type reported in protocol:** Sensitivity analysis.  **Analysis type reported in review:** Not reported.  **Reason for changing mind:** Not reported.    **Analysis type in protocol according to glossary:** Sensitivity analysis.  **How determined the analysis type:**’ exclusion’.  **Analysis type in review according to glossary:** Not reported.  **How determined the analysis type:** NA.  **Analysed:** No.  **Reasons given (in the review) for not doing interaction analysis if planned:** Not reported (presumably because no meta-analysis).  **Reasons given for carrying out interaction analysis if they weren’t planned:** NA.  **Scenario in protocol:** Not reported.  **Rationale for planned scenario:** NA.  **Scenario in review (if reported specifically):** Not reported.  **Rationale for done scenario:** NA.  **Reason for changing mind:** NA. | **Method to detect interactions reported in protocol:** Not reported.  **Method to detect interactions reported in review:** Not reported.  **Reason for changing mind:** NA. |
| Gower 2013 | **Protocol:** ‘Losses to follow-up.’  **Review methods:** Not reported.  **Review results:** Not analysed.  **Type:** Methodological.  **Covariate summary:** Incomplete outcome data/follow up. | **Method to choose**  **covariate:** Not reported.  **Where method reported:** NA.  **Rationale for choosing the covariate:** Not reported.  **Where rationale reported:** NA. | **Where reported:** Protocol only.  **Reason not reported in review:** Not reported.  **Reason not reported in protocol:** NA.  **Labelled as ‘posthoc’ if not in protocol:** NA | **Data type:** Continuous.  **Continuous covariate categorised:** Yes.  **How determine it was categorised:** Subgroup analysis planned.  **Justification for categorising:** Not reported.  **Where justification reported:** NA.  **Categories reported:** Not reported.  **Where categories reported:** NA.  **Justification for categories:** Not reported.  **Where justification reported:** NA. | **Analysis type reported in protocol:** Subgroup analysis.  **Analysis type reported in review:** Not reported.  **Reason for changing mind:** Not reported.    **Analysis type in protocol according to glossary:** Subgroup analysis.  **How determined the analysis type:** Says ‘Subgroup analysis.’ No reason to dispute.  **Analysis type in review according to glossary:** Not reported.  **How determined the analysis type:** NA.  **Analysed:** No.  **Reasons given (in the review) for not doing interaction analysis if planned:** Not reported (presumably because no meta-analysis).  **Reasons given for carrying out interaction analysis if they weren’t planned:** NA.  **Scenario in protocol:** ‘Where substantial heterogeneity exists, sub group analysis will be performed’.  **Rationale for planned scenario:** Not reported.  **Scenario in review (if reported specifically):** Not reported.  **Rationale for done scenario:** NA.  **Reason for changing mind:** NA. | **Method to detect interactions reported in protocol:** Not reported.  **Method to detect interactions reported in review:** Not reported.  **Reason for changing mind:** NA. |
| Gower 2013 | **Protocol:** ‘Participant characteristics.’  **Review methods:** Not reported.  **Review results:** Not analysed.  **Type:** Patient.  **Covariate summary:** Patient characteristics. | **Method to choose**  **covariate:** Not reported.  **Where method reported:** NA.  **Rationale for choosing the covariate:** Not reported.  **Where rationale reported:** NA. | **Where reported:** Protocol only.  **Reason not reported in review:** Not reported.  **Reason not reported in protocol:** NA.  **Labelled as ‘posthoc’ if not in protocol:** NA | **Data type:** Unclear.  **Continuous covariate categorised:** Unclear.  **How determine it was categorised:** NA.  **Justification for categorising:** NA.  **Where justification reported:** NA.  **Categories reported:** Not reported.  **Where categories reported:** NA.  **Justification for categories:** Not reported.  **Where justification reported:** NA. | **Analysis type reported in protocol:** Subgroup analysis.  **Analysis type reported in review:** Not reported.  **Reason for changing mind:** Not reported.    **Analysis type in protocol according to glossary:** Subgroup analysis.  **How determined the analysis type:** Says ‘Subgroup analysis.’ No reason to dispute.  **Analysis type in review according to glossary:** Not reported.  **How determined the analysis type:** NA.  **Analysed:** No.  **Reasons given (in the review) for not doing interaction analysis if planned:** Not reported (presumably because no meta-analysis).  **Reasons given for carrying out interaction analysis if they weren’t planned:** NA.  **Scenario in protocol:** ‘Where substantial heterogeneity exists, sub group analysis will be performed’.  **Rationale for planned scenario:** Not reported.  **Scenario in review (if reported specifically):** Not reported.  **Rationale for done scenario:** NA.  **Reason for changing mind:** NA. | **Method to detect interactions reported in protocol:** Not reported.  **Method to detect interactions reported in review:** Not reported.  **Reason for changing mind:** NA. |
| Gower 2013 | **Protocol:** ‘Routes of administration of prophylactic measures.’  **Review methods:** Not reported.  **Review results:** Not analysed.  **Type:** Intervention.  **Covariate summary:** Route of administration. | **Method to choose**  **covariate:** Not reported.  **Where method reported:** NA.  **Rationale for choosing the covariate:** Not reported.  **Where rationale reported:** NA. | **Where reported:** Protocol only.  **Reason not reported in review:** Not reported.  **Reason not reported in protocol:** NA.  **Labelled as ‘posthoc’ if not in protocol:** NA | **Data type:** Categorical.  **Continuous covariate categorised:** NA.  **How determine it was categorised:** NA.  **Justification for categorising:** NA.  **Where justification reported:** NA.  **Categories reported:** Not reported.  **Where categories reported:** NA.  **Justification for categories:** Not reported.  **Where justification reported:** NA. | **Analysis type reported in protocol:** Subgroup analysis.  **Analysis type reported in review:** Not reported.  **Reason for changing mind:** Not reported.    **Analysis type in protocol according to glossary:** Subgroup analysis.  **How determined the analysis type:** Says ‘Subgroup analysis.’ No reason to dispute.  **Analysis type in review according to glossary:** Not reported.  **How determined the analysis type:** NA.  **Analysed:** No.  **Reasons given (in the review) for not doing interaction analysis if planned:** Not reported (presumably because no meta-analysis).  **Reasons given for carrying out interaction analysis if they weren’t planned:** NA.  **Scenario in protocol:** ‘Where substantial heterogeneity exists, sub group analysis will be performed’.  **Rationale for planned scenario:** Not reported.  **Scenario in review (if reported specifically):** Not reported.  **Rationale for done scenario:** NA.  **Reason for changing mind:** NA. | **Method to detect interactions reported in protocol:** Not reported.  **Method to detect interactions reported in review:** Not reported.  **Reason for changing mind:** NA. |
| Gower 2013 | **Protocol:** ‘Study quality’.  **Review methods:** Not reported.  **Review results:** Not analysed.  **Type:** Methodological.  **Covariate summary:** Quality. | **Method to choose**  **covariate:** Not reported.  **Where method reported:** NA.  **Rationale for choosing the covariate:** Not reported.  **Where rationale reported:** NA. | **Where reported:** Protocol only.  **Reason not reported in review:** Not reported.  **Reason not reported in protocol:** NA.  **Labelled as ‘posthoc’ if not in protocol:** NA. | **Data type:** Categorical.  **Continuous covariate categorised:** NA.  **How determine it was categorised:** NA.  **Justification for categorising:** NA.  **Where justification reported:** NA.  **Categories reported:** Not reported.  **Where categories reported:** NA.  **Justification for categories:** Not reported.  **Where justification reported:** NA. | **Analysis type reported in protocol:** Subgroup analysis.  **Analysis type reported in review:** Not reported.  **Reason for changing mind:** Not reported.    **Analysis type in protocol according to glossary:** Subgroup analysis.  **How determined the analysis type:** Says ‘Subgroup analysis.’ No reason to dispute.  **Analysis type in review according to glossary:** Not reported.  **How determined the analysis type:** NA.  **Analysed:** No.  **Reasons given (in the review) for not doing interaction analysis if planned:** Not reported (presumably because no meta-analysis).  **Reasons given for carrying out interaction analysis if they weren’t planned:** NA.  **Scenario in protocol:** ‘Where substantial heterogeneity exists, sub group analysis will be performed’.  **Rationale for planned scenario:** Not reported.  **Scenario in review (if reported specifically):** Not reported.  **Rationale for done scenario:** NA.  **Reason for changing mind:** NA. | **Method to detect interactions reported in protocol:** Not reported.  **Method to detect interactions reported in review:** Not reported.  **Reason for changing mind:** NA. |
| Gower 2013 | **Protocol:** ‘Type of prophylactic intervention’.  **Review methods:** Not reported.  **Review results:** Not analysed.  **Type:** Intervention.  **Covariate summary:** Type of intervention or control. | **Method to choose**  **covariate:** Not reported.  **Where method reported:** NA.  **Rationale for choosing the covariate:** Not reported.  **Where rationale reported:** NA. | **Where reported:** Protocol only.  **Reason not reported in review:** Not reported.  **Reason not reported in protocol:** NA.  **Labelled as ‘posthoc’ if not in protocol:** NA | **Data type:** Categorical.  **Continuous covariate categorised:** NA.  **How determine it was categorised:** NA.  **Justification for categorising:** NA.  **Where justification reported:** NA.  **Categories reported:** Not reported.  **Where categories reported:** NA.  **Justification for categories:** Not reported.  **Where justification reported:** NA. | **Analysis type reported in protocol:** Subgroup analysis.  **Analysis type reported in review:** Not reported.  **Reason for changing mind:** Not reported.    **Analysis type in protocol according to glossary:** Subgroup analysis.  **How determined the analysis type:** Says ‘Subgroup analysis.’ No reason to dispute.  **Analysis type in review according to glossary:** Not reported.  **How determined the analysis type:** NA.  **Analysed:** No.  **Reasons given (in the review) for not doing interaction analysis if planned:** Not reported (presumably because no meta-analysis).  **Reasons given for carrying out interaction analysis if they weren’t planned:** NA.  **Scenario in protocol:** ‘Where substantial heterogeneity exists, sub group analysis will be performed’.  **Rationale for planned scenario:** Not reported.  **Scenario in review (if reported specifically):** Not reported.  **Rationale for done scenario:** NA.  **Reason for changing mind:** NA. | **Method to detect interactions reported in protocol:** Not reported.  **Method to detect interactions reported in review:** Not reported.  **Reason for changing mind:** NA. |
| Gower 2013 | **Protocol:** Not reported.  **Review methods:** Not reported.  **Review results:** ‘Chloramphenicol-suphadimidine drops alone or with periocular penicillin’; ‘Subconjunctival versus retrobulbar antibiotic Injection’; ‘Irrigation with balanced salt solution (BSS) alone versus BSS with antibiotics’; ‘Perioperative prophylaxis with intracameral and/or topical antibiotics’.  **Type:** Intervention.  **Covariate summary:** Type of intervention or control. | **Method to choose**  **covariate:** Not reported.  **Where method reported:** NA.  **Rationale for choosing the covariate:** Not reported.  **Where rationale reported:** NA. | **Where reported:** Review only.  **Reason not reported in review:** NA.  **Reason not reported in protocol:** Not reported.  **Labelled as ‘posthoc’ if not in protocol:** No. | **Data type:** Categorical.  **Continuous covariate categorised:** NA.  **How determine it was categorised:** NA.  **Justification for categorising:** NA.  **Where justification reported:** NA.  **Categories reported:** Yes.  **Where categories reported:** Review only.  **Justification for categories:** Not reported.  **Where justification reported:** NA. | **Analysis type reported in protocol:** Unnamed analysis.  **Analysis type reported in review:** Unnamed analysis.  **Reason for changing mind:** NA.    **Analysis type in protocol according to glossary:** Stratification/subgroup analysis.  **How determined the analysis type:** Categories given.  **Analysis type in review according to glossary:** Stratification/subgroup analysis.  **How determined the analysis type:** Categories given.  **Analysed:** Yes.  **Reasons given (in the review) for not doing interaction analysis if planned:** NA.  **Reasons given for carrying out interaction analysis if they weren’t planned:** NA.  **Scenario in protocol:** Not reported.  **Rationale for planned scenario:** NA.  **Scenario in review (if reported specifically):** Not reported.  **Rationale for done scenario:** NA.  **Reason for changing mind:** NA. | **Method to detect interactions reported in protocol:** Not reported.  **Method to detect interactions reported in review:** Not reported.  **Reason for changing mind:** NA. |
| He 2013 | **Protocol:** ‘Baseline EDSS scores (e. g. ≤ 3.5, between 3. 5 and 6).’  **Review methods:** ‘Baseline EDSS scores (e. g. ≤ 3. 5, between 3. 5 and 6).’  **Review results:** Not analysed.  **Type:** Patient.  **Covariate summary:** Disease characteristics. | **Method to choose**  **covariate:** Not reported.  **Where method reported:** NA.  **Rationale for choosing the covariate:** Not reported.  **Where rationale reported:** NA. | **Where reported:** Protocol and review.  **Reason not reported in review:** NA.  **Reason not reported in protocol:** NA.  **Labelled as ‘posthoc’ if not in protocol:** NA. | **Data type:** Continuous.  **Continuous covariate categorised:** Yes.  **How determine it was categorised:** Categories given.  **Justification for categorising:** Not reported.  **Where justification reported:** NA.  **Categories reported:** Yes.  **Where categories reported:** Protocol and review.  **Justification for categories:** Not reported.  **Where justification reported:** NA. | **Analysis type reported in protocol:** Subgroup analysis.  **Analysis type reported in review:** Subgroup analysis.  **Reason for changing mind:** NA.    **Analysis type in protocol according to glossary:** Subgroup analysis.  **How determined the analysis type:** Categories given.  **Analysis type in review according to glossary:** Subgroup analysis.  **How determined the analysis type:** Categories given.  **Analysed:** No.  **Reasons given (in the review) for not doing interaction analysis if planned: ‘**We could not carry out subgroup analysis because of the paucity of data.’  **Reasons given for carrying out interaction analysis if they weren’t planned:** NA.  **Scenario in protocol:** ‘if possible’.  **Rationale for planned scenario:** Not reported.  **Scenario in review (if reported specifically):** ‘In future updates and if further data become available we  intend to undertake subgroup analyses’.  **Rationale for done scenario:** Not reported.  **Reason for changing mind:** NA. | **Method to detect interactions reported in protocol:** Not reported.  **Method to detect interactions reported in review:** Not reported.  **Reason for changing mind:** NA. |
| He 2013 | **Protocol:** ‘Different duration of MS (e. g. 5 years, more than 5 years)’.  **Review methods: ‘**Different duration of MS (e. g. 5 years, more than 5 years)’.  **Review results:** Not analysed.  **Type:** Patient.  **Covariate summary:** Disease characteristics. | **Method to choose**  **covariate:** Not reported.  **Where method reported:** NA.  **Rationale for choosing the covariate:** Not reported.  **Where rationale reported:** NA. | **Where reported:** Protocol and review.  **Reason not reported in review:** NA.  **Reason not reported in protocol:** NA.  **Labelled as ‘posthoc’ if not in protocol:** NA. | **Data type:** Continuous.  **Continuous covariate categorised:** Yes.  **How determine it was categorised:** Categories given.  **Justification for categorising:** Not reported.  **Where justification reported:** NA.  **Categories reported:** Yes.  **Where categories reported:** Protocol and review.  **Justification for categories:** Not reported.  **Where justification reported:** NA. | **Analysis type reported in protocol:** Subgroup analysis.  **Analysis type reported in review:** Subgroup analysis.  **Reason for changing mind:** NA.    **Analysis type in protocol according to glossary:** Subgroup analysis.  **How determined the analysis type:** Categories given.  **Analysis type in review according to glossary:** Subgroup analysis.  **How determined the analysis type:** Categories given.  **Analysed:** No.  **Reasons given (in the review) for not doing interaction analysis if planned: ‘**We could not carry out subgroup analysis because of the paucity of data.’  **Reasons given for carrying out interaction analysis if they weren’t planned:** NA.  **Scenario in protocol:** ‘if possible’.  **Rationale for planned scenario:** Not reported.  **Scenario in review (if reported specifically):** ‘In future updates and if further data become available we  intend to undertake subgroup analyses’.  **Rationale for done scenario:** Not reported.  **Reason for changing mind:** NA. | **Method to detect interactions reported in protocol:** Not reported.  **Method to detect interactions reported in review:** Not reported.  **Reason for changing mind:** NA. |
| He 2013 | **Protocol:** ‘Different MS patients (e. g. patients with RRMS or patients with progressive MS).’  **Review methods:** ‘Different MS patients (e. g. patients with RRMS or patients with progressive MS).’  **Review results:** Not analysed.  **Type:** Patient.  **Covariate summary:** Disease characteristics. | **Method to choose**  **covariate:** Not reported.  **Where method reported:** NA.  **Rationale for choosing the covariate:** Not reported.  **Where rationale reported:** NA. | **Where reported:** Protocol and review.  **Reason not reported in review:** NA.  **Reason not reported in protocol:** NA.  **Labelled as ‘posthoc’ if not in protocol:** NA. | **Data type:** Categorical.  **Continuous covariate categorised:** NA.  **How determine it was categorised:** NA.  **Justification for categorising:** NA.  **Where justification reported:** NA.  **Categories reported:** Yes.  **Where categories reported:** Protocol and review.  **Justification for categories:** Not reported.  **Where justification reported:** NA. | **Analysis type reported in protocol:** Subgroup analysis.  **Analysis type reported in review:** Subgroup analysis.  **Reason for changing mind:** NA.    **Analysis type in protocol according to glossary:** Subgroup analysis.  **How determined the analysis type:** Categories given.  **Analysis type in review according to glossary:** Subgroup analysis.  **How determined the analysis type:** Categories given.  **Analysed:** No.  **Reasons given (in the review) for not doing interaction analysis if planned: ‘**We could not carry out subgroup analysis because of the paucity of data.’  **Reasons given for carrying out interaction analysis if they weren’t planned:** NA.  **Scenario in protocol:** ‘if possible’.  **Rationale for planned scenario:** Not reported.  **Scenario in review (if reported specifically):** ‘In future updates and if further data become available we  intend to undertake subgroup analyses’.  **Rationale for done scenario:** Not reported.  **Reason for changing mind:** NA. | **Method to detect interactions reported in protocol:** Not reported.  **Method to detect interactions reported in review:** Not reported.  **Reason for changing mind:** NA. |
| He 2013 | **Protocol:** ‘Different therapies (e. g. monotherapy, combined IFN-beta therapy or combined glatiramer acetate therapy)’.  **Review methods:** ‘Different therapies (e. g. monotherapy, combined interferon (IFN)-beta therapy)’.  **Review results:** Not analysed.  **Type:** Intervention.  **Covariate summary:** Type of intervention or control. | **Method to choose**  **covariate:** Not reported.  **Where method reported:** NA.  **Rationale for choosing the covariate:** Not reported.  **Where rationale reported:** NA. | **Where reported:** Protocol and review.  **Reason not reported in review:** NA.  **Reason not reported in protocol:** NA.  **Labelled as ‘posthoc’ if not in protocol:** NA. | **Data type:** Categorical.  **Continuous covariate categorised:** NA.  **How determine it was categorised:** NA.  **Justification for categorising:** NA.  **Where justification reported:** NA.  **Categories reported:** Yes.  **Where categories reported:** Protocol and review.  **Justification for categories:** Not reported.  **Where justification reported:** NA. | **Analysis type reported in protocol:** Subgroup analysis.  **Analysis type reported in review:** Subgroup analysis.  **Reason for changing mind:** NA.    **Analysis type in protocol according to glossary:** Subgroup analysis.  **How determined the analysis type:** Categories given.  **Analysis type in review according to glossary:** Subgroup analysis.  **How determined the analysis type:** Categories given.  **Analysed:** No.  **Reasons given (in the review) for not doing interaction analysis if planned: ‘**We could not carry out subgroup analysis because of the paucity of data.’  **Reasons given for carrying out interaction analysis if they weren’t planned:** NA.  **Scenario in protocol:** ‘if possible’.  **Rationale for planned scenario:** Not reported.  **Scenario in review (if reported specifically):** ‘In future updates and if further data become available we  intend to undertake subgroup analyses’.  **Rationale for done scenario:** Not reported.  **Reason for changing mind:** NA. | **Method to detect interactions reported in protocol:** Not reported.  **Method to detect interactions reported in review:** Not reported.  **Reason for changing mind:** NA. |
| He 2013 | **Protocol:** ‘Dosage level (e. g. 0. 3mg/day, 0. 6mg/day or 1. 2 mg/day)’.  **Review methods:** ‘Dosage level (e. g. 0. 1 mg/day, 0. 3 mg/day, 0. 6 mg/day or 1. 2 mg/day)’.  **Review results:** Not analysed.  **Type:** Intervention.  **Covariate summary:** Dose. | **Method to choose**  **covariate:** Not reported.  **Where method reported:** NA.  **Rationale for choosing the covariate:** Not reported.  **Where rationale reported:** NA. | **Where reported:** Protocol and review.  **Reason not reported in review:** NA.  **Reason not reported in protocol:** NA.  **Labelled as ‘posthoc’ if not in protocol:** NA. | **Data type:** Continuous.  **Continuous covariate categorised:** Yes.  **How determine it was categorised:** Categories given.  **Justification for categorising:** Not reported.  **Where justification reported:** NA.  **Categories reported:** Yes.  **Where categories reported:** Protocol and review.  **Justification for categories:** Not reported.  **Where justification reported:** NA. | **Analysis type reported in protocol:** Subgroup analysis.  **Analysis type reported in review:** Subgroup analysis.  **Reason for changing mind:** NA.    **Analysis type in protocol according to glossary:** Subgroup analysis.  **How determined the analysis type:** Categories given.  **Analysis type in review according to glossary:** Subgroup analysis.  **How determined the analysis type:** Categories given.  **Analysed:** No.  **Reasons given (in the review) for not doing interaction analysis if planned: ‘**We could not carry out subgroup analysis because of the paucity of data.’  **Reasons given for carrying out interaction analysis if they weren’t planned:** NA.  **Scenario in protocol:** ‘if possible’.  **Rationale for planned scenario:** Not reported.  **Scenario in review (if reported specifically):** ‘In future updates and if further data become available we  intend to undertake subgroup analyses’.  **Rationale for done scenario:** Not reported.  **Reason for changing mind:** NA. | **Method to detect interactions reported in protocol:** Not reported.  **Method to detect interactions reported in review:** Not reported.  **Reason for changing mind:** NA. |
| He 2013 | **Protocol:** ‘Including trials at high risk of bias’.  **Review methods: ‘**Including trials at high risk of bias’.  **Review results:** Not analysed.  **Type:** Methodological.  **Covariate summary:** Risk of bias. | **Method to choose**  **covariate:** Not reported.  **Where method reported:** NA.  **Rationale for choosing the covariate:** Not reported.  **Where rationale reported:** NA. | **Where reported:** Protocol and review.  **Reason not reported in review:** NA.  **Reason not reported in protocol:** NA.  **Labelled as ‘posthoc’ if not in protocol:** NA. | **Data type:** Categorical.  **Continuous covariate categorised:** NA.  **How determine it was categorised:** NA.  **Justification for categorising:** NA.  **Where justification reported:** NA.  **Categories reported:** Yes.  **Where categories reported:** Protocol and review.  **Justification for categories:** Not reported.  **Where justification reported:** NA. | **Analysis type reported in protocol:** Sensitivity analysis.  **Analysis type reported in review:** Sensitivity analysis.  **Reason for changing mind:** NA.    **Analysis type in protocol according to glossary:** Sensitivity analysis.  **How determined the analysis type:** Categories given.  **Analysis type in review according to glossary:** Sensitivity analysis.  **How determined the analysis type:** Categories given.  **Analysed:** No.  **Reasons given (in the review) for not doing interaction analysis if planned: ‘**If a sufficient number of studies had been included, we would have undertaken sensitivity analyses to assess the robustness of our review results.’  **Reasons given for carrying out interaction analysis if they weren’t planned:** NA.  **Scenario in protocol:** ‘Where possible’.  **Rationale for planned scenario:** Not reported.  **Scenario in review (if reported specifically):** ‘If a sufficient number of studies had been included, we would have undertaken sensitivity analyses to assess the robustness of our review results. Where possible, we will conduct sensitivity analyses.’  **Rationale for done scenario:** Not reported.  **Reason for changing mind:** NA. | **Method to detect interactions reported in protocol:** Not reported.  **Method to detect interactions reported in review:** Not reported.  **Reason for changing mind:** NA. |
| He 2013 | **Protocol:** ‘The effects of analysing by intention to treat’.  **Review methods: ‘**The effects of analysing by intention to treat’.  **Review results:** Not analysed.  **Type:** Methodological.  **Covariate summary:** Intention to treat. | **Method to choose**  **covariate:** Not reported.  **Where method reported:** NA.  **Rationale for choosing the covariate:** Not reported.  **Where rationale reported:** NA. | **Where reported:** Protocol and review.  **Reason not reported in review:** NA.  **Reason not reported in protocol:** NA.  **Labelled as ‘posthoc’ if not in protocol:** NA. | **Data type:** Categorical.  **Continuous covariate categorised:** NA.  **How determine it was categorised:** NA.  **Justification for categorising:** NA.  **Where justification reported:** NA.  **Categories reported:** Yes.  **Where categories reported:** Protocol and review.  **Justification for categories:** Not reported.  **Where justification reported:** NA. | **Analysis type reported in protocol:** Sensitivity analysis.  **Analysis type reported in review:** Sensitivity analysis.  **Reason for changing mind:** NA.    **Analysis type in protocol according to glossary:** Sensitivity analysis.  **How determined the analysis type:** Categories given.  **Analysis type in review according to glossary:** Sensitivity analysis.  **How determined the analysis type:** Categories given.  **Analysed:** No.  **Reasons given (in the review) for not doing interaction analysis if planned: ‘**If a sufficient number of studies had been included, we would have undertaken sensitivity analyses to assess the robustness of our review results.’  **Reasons given for carrying out interaction analysis if they weren’t planned:** NA.  **Scenario in protocol:** ‘Where possible’.  **Rationale for planned scenario:** Not reported.  **Scenario in review (if reported specifically):** ‘If a sufficient number of studies had been included, we would have undertaken sensitivity analyses to assess the robustness of our review results. Where possible, we will conduct sensitivity analyses.’  **Rationale for done scenario:** Not reported.  **Reason for changing mind:** NA. | **Method to detect interactions reported in protocol:** Not reported.  **Method to detect interactions reported in review:** Not reported.  **Reason for changing mind:** NA. |
| He 2013 | **Protocol:** ‘We will assess the following primary outcomes, measured in the treatment phase and at the completion of follow-up versus baseline.’ ‘Duration of follow-up (e. g. 1 year, between 1 and 2 years, more than 2 years)’.  **Review methods:** ‘We will assess the following primary outcomes, measured in the treatment phase and at the completion of follow-up versus baseline.’ ‘Duration of follow-up (e. g. 1 year, between 1 and 2 years, more than 2 years)’.  **Review results:** Not analysed.  **Type:** Methodological.  **Covariate summary:** Length of follow up. | **Method to choose**  **covariate:** Not reported.  **Where method reported:** NA.  **Rationale for choosing the covariate:** Not reported.  **Where rationale reported:** NA. | **Where reported:** Protocol and review.  **Reason not reported in review:** NA.  **Reason not reported in protocol:** NA.  **Labelled as ‘posthoc’ if not in protocol:** NA. | **Data type:** Continuous.  **Continuous covariate categorised:** Yes.  **How determine it was categorised:** Categories given.  **Justification for categorising:** Not reported.  **Where justification reported:** NA.  **Categories reported:** Yes.  **Where categories reported:** Protocol and review.  **Justification for categories:** Not reported.  **Where justification reported:** NA. | **Analysis type reported in protocol:** Subgroup analysis.  **Analysis type reported in review:** Subgroup analysis.  **Reason for changing mind:** NA.    **Analysis type in protocol according to glossary:** Subgroup analysis.  **How determined the analysis type:** Categories given.  **Analysis type in review according to glossary:** Subgroup analysis.  **How determined the analysis type:** Categories given.  **Analysed:** No.  **Reasons given (in the review) for not doing interaction analysis if planned: ‘**We could not carry out subgroup analysis because of the paucity of data.’  **Reasons given for carrying out interaction analysis if they weren’t planned:** NA.  **Scenario in protocol:** ‘if possible’.  **Rationale for planned scenario:** Not reported.  **Scenario in review (if reported specifically):** ‘In future updates and if further data become available we  intend to undertake subgroup analyses’.  **Rationale for done scenario:** Not reported.  **Reason for changing mind:** NA. | **Method to detect interactions reported in protocol:** Not reported.  **Method to detect interactions reported in review:** Not reported.  **Reason for changing mind:** NA. |
| **Itchaki 2013** | **Protocol: ‘**Age over 60 years’. ‘Patients’ mean age’.  **Review methods: ‘**Age over 60 years’.  **Review results:** Not analysed.  **Type:** Patient.  **Covariate summary:** Demographics. | **Method to choose**  **covariate:** Not reported.  **Where method reported:** NA.  **Rationale for choosing the covariate:** Not reported.  **Where rationale reported:** NA. | **Where reported:** Protocol and review.  **Reason not reported in review:** NA.  **Reason not reported in protocol:** NA.  **Labelled as ‘posthoc’ if not in protocol:** NA. | **Data type:** Continuous.  **Continuous covariate categorised:** Yes for subgroup analysis, no for meta-regression.  **How determine it was categorised:** Subgroup analysis planned.  **Justification for categorising:** Not reported.  **Where justification reported:** NA.  **Categories reported:** Not reported.  **Where categories reported:** NA.  **Justification for categories:** Not reported.  **Where justification reported:** NA. | **Analysis type reported in protocol:** Subgroup analysis and meta-regression.  **Analysis type reported in review:** Subgroup analysis.  **Reason for changing mind:** Not reported.    **Analysis type in protocol according to glossary:** Sensitivity analysis and meta-regression.  **How determined the analysis type:** Categories given.  **Analysis type in review according to glossary:** Sensitivity analysis  **How determined the analysis type:** Categories given.  **Analysed:** No.  **Reasons given (in the review) for not doing interaction analysis if planned:** ‘However, the paucity of trials and lack of data in included studies  allowed for subgroup analyses of the last count alone.’  **Reasons given for carrying out interaction analysis if they weren’t planned:** NA.  **Scenario in protocol: ‘**We will investigate heterogeneity in each analysis  based on the following pre-planned subgroup analyses’ ‘if enough trials will be available’ (for meta-regression).  **Rationale for planned scenario:** protocol.  **Scenario in review (if reported specifically): ‘**We set to investigate heterogeneity in each analysis  based on the following preplanned subgroup analyses’  **Rationale for done scenario:** Not reported.  **Reason for changing mind:** NA. | **Method to detect interactions reported in protocol:** Not reported.  **Method to detect interactions reported in review:** Not reported.  **Reason for changing mind:** NA. |
| **Itchaki 2013** | **Protocol:** ‘Allocation concealment’.  **Review methods: ‘**Allocation concealment’.  **Review results: ‘**Adequate’ versus ‘unclear’.  **Type:** Methodological.  **Covariate summary:** Allocation concealment. | **Method to choose**  **covariate:** Not reported.  **Where method reported:** NA.  **Rationale for choosing the covariate:**  ‘Allocation concealment and blinding (see above), which have been shown to affect subjective outcomes most strongly (Wood 2008).’ in protocol. ‘Allocation concealment (see above), which has been shown to affect subjective outcomes most strongly (Wood 2008).’ in review.  **Where rationale reported:** Protocol and review. | **Where reported:** Protocol and review.  **Reason not reported in review:** NA.  **Reason not reported in protocol:** NA.  **Labelled as ‘posthoc’ if not in protocol:** NA. | **Data type:** Categorical.  **Continuous covariate categorised:** NA.  **How determine it was categorised:** NA.  **Justification for categorising:** NA.  **Where justification reported:** NA.  **Categories reported:** Yes.  **Where categories reported:** Review only.  **Justification for categories:** Not reported.  **Where justification reported:** NA. | **Analysis type reported in protocol:** Sensitivity analysis.  **Analysis type reported in review:** Sensitivity analysis.  **Reason for changing mind:** NA,    **Analysis type in protocol according to glossary:** Sensitivity analysis.  **How determined the analysis type:** Says ‘Sensitivity analysis’. No reason to dispute.  **Analysis type in review according to glossary:** Subgroup analysis.  **How determined the analysis type:** Results presented.  **Analysed:** Yes.  **Reasons given (in the review) for not doing interaction analysis if planned:** NA.  **Reasons given for carrying out interaction analysis if they weren’t planned:** NA.  **Scenario in protocol:** Not reported.  **Rationale for planned scenario:** NA.  **Scenario in review (if reported specifically):** Not reported.  **Rationale for done scenario:** NA.  **Reason for changing mind:** NA. | **Method to detect interactions reported in protocol:** Not reported.  **Method to detect interactions reported in review:** Not reported.  **Reason for changing mind:** NA. |
| **Itchaki 2013** | **Protocol:** ‘Blinding’.  **Review methods: ‘**Blinding’.  **Review results:** Not analysed.  **Type:** Methodological.  **Covariate summary:** Blinding. | **Method to choose**  **covariate:** Not reported.  **Where method reported:** NA.  **Rationale for choosing the covariate:** ‘Allocation concealment and blinding (see above), which have been shown to affect subjective outcomes most strongly  (Wood 2008).’  **Where rationale reported:** Protocol only. | **Where reported:** Protocol and review.  **Reason not reported in review:** NA.  **Reason not reported in protocol:** NA.  **Labelled as ‘posthoc’ if not in protocol:** NA. | **Data type:** Categorical.  **Continuous covariate categorised:** NA.  **How determine it was categorised:** NA.  **Justification for categorising:** NA.  **Where justification reported:** NA.  **Categories reported:** Not reported.  **Where categories reported:** NA.  **Justification for categories:** Not reported.  **Where justification reported:** NA. | **Analysis type reported in protocol:** Sensitivity analysis.  **Analysis type reported in review:** Sensitivity analysis.  **Reason for changing mind:** NA.    **Analysis type in protocol according to glossary:** Sensitivity analysis.  **How determined the analysis type:** Says ‘Sensitivity analysis’. No reason to dispute.  **Analysis type in review according to glossary:** Sensitivity analysis.  **How determined the analysis type:** Says ‘Sensitivity analysis’. No reason to dispute.  **Analysed:** No.  **Reasons given (in the review) for not doing interaction analysis if planned:** ‘Given the paucity of included trials, we were able to fulfil only  the first two counts, and did not undertake any further sensitivity  analyses.’  **Reasons given for carrying out interaction analysis if they weren’t planned:** NA.  **Scenario in protocol:** Not reported.  **Rationale for planned scenario:** NA.  **Scenario in review (if reported specifically):** Not reported.  **Rationale for done scenario:** NA.  **Reason for changing mind:** NA. | **Method to detect interactions reported in protocol:** Not reported.  **Method to detect interactions reported in review:** Not reported.  **Reason for changing mind:** NA. |
| **Itchaki 2013** | **Protocol:** ‘Different anthracyclines’.  **Review methods: ‘**Different anthracyclines’.  **Review results: ‘**Doxorubicin’ versus ‘Idarubicin’.  **Type:** Intervention.  **Covariate summary:** Type of intervention or control. | **Method to choose**  **covariate:** Not reported.  **Where method reported:** NA.  **Rationale for choosing the covariate:** Not reported.  **Where rationale reported:** NA. | **Where reported:** Protocol and review.  **Reason not reported in review:** NA.  **Reason not reported in protocol:** NA.  **Labelled as ‘posthoc’ if not in protocol:** NA. | **Data type:** Categorical.  **Continuous covariate categorised:** NA.  **How determine it was categorised:** NA.  **Justification for categorising:** NA.  **Where justification reported:** NA.  **Categories reported:** Yes.  **Where categories reported:** Review only.  **Justification for categories:** Not reported.  **Where justification reported:** NA. | **Analysis type reported in protocol:** Subgroup analysis.  **Analysis type reported in review:** Subgroup analysis.  **Reason for changing mind:** NA.    **Analysis type in protocol according to glossary:** Subgroup analysis.  **How determined the analysis type:** Categories given.  **Analysis type in review according to glossary:** Subgroup analysis.  **How determined the analysis type:** Categories given.  **Analysed:** Yes.  **Reasons given (in the review) for not doing interaction analysis if planned:** NA.  **Reasons given for carrying out interaction analysis if they weren’t planned:** NA.  **Scenario in protocol: ‘**We will investigate heterogeneity in each analysis  based on the following pre-planned subgroup analyses’  **Rationale for planned scenario:** protocol.  **Scenario in review (if reported specifically): ‘**We set to investigate heterogeneity in each analysis  based on the following preplanned subgroup analyses’  **Rationale for done scenario:** Not reported.  **Reason for changing mind:** NA. | **Method to detect interactions reported in protocol:** Not reported.  **Method to detect interactions reported in review:** Not reported.  **Reason for changing mind:** NA. |
| **Itchaki 2013** | **Protocol:** ‘Exclude studies in which the percentage of patients with FL is less than 70% of evaluated patients.’  **Review methods:** Not reported.  **Review results:** Included Zinzani 2000, a trial that included 50% FL.  **Type:** Patient.  **Covariate summary:** Disease characteristics. | **Method to choose**  **covariate:** Not reported.  **Where method reported:** NA.  **Rationale for choosing the covariate:** Not reported.  **Where rationale reported:** NA. | **Where reported:** Protocol only.  **Reason not reported in review:** Not reported.  **Reason not reported in protocol:** NA.  **Labelled as ‘posthoc’ if not in protocol:** NA. | **Data type:** Categorical.  **Continuous covariate categorised:** NA.  **How determine it was categorised:** NA.  **Justification for categorising:** NA.  **Where justification reported:** NA.  **Categories reported:** Yes.  **Where categories reported:** Protocol only.  **Justification for categories:** Not reported.  **Where justification reported:** NA. | **Analysis type reported in protocol:** Sensitivity analysis.  **Analysis type reported in review:** Unnamed analysis.  **Reason for changing mind:** Not reported.    **Analysis type in protocol according to glossary:** Sensitivity analysis.  **How determined the analysis type:** Categories given.  **Analysis type in review according to glossary:** Sensitivity analysis.  **How determined the analysis type:** Results reported.  **Analysed:** Yes.  **Reasons given (in the review) for not doing interaction analysis if planned:** NA.  **Reasons given for carrying out interaction analysis if they weren’t planned:** NA.  **Scenario in protocol:** Not reported.  **Rationale for planned scenario:** NA.  **Scenario in review (if reported specifically):** Not reported.  **Rationale for done scenario:** NA.  **Reason for changing mind:** NA. | **Method to detect interactions reported in protocol:** Not reported.  **Method to detect interactions reported in review:** Not reported.  **Reason for changing mind:** NA. |
| **Itchaki 2013** | **Protocol: ‘**First-line versus relapse or refractory’. ‘Percentage of patients with relapsed or refractory disease’.  **Review methods: ‘**First-line versus relapse or refractory’.  **Review results:** Not analysed.  **Type:** Patient.  **Covariate summary:** Disease characteristics. | **Method to choose**  **covariate:** Not reported.  **Where method reported:** NA.  **Rationale for choosing the covariate:** Not reported.  **Where rationale reported:** NA. | **Where reported:** Protocol and review.  **Reason not reported in review:** NA.  **Reason not reported in protocol:** NA.  **Labelled as ‘posthoc’ if not in protocol:** NA. | **Data type:** Categorical.  **Continuous covariate categorised:** NA.  **How determine it was categorised:** NA.  **Justification for categorising:** NA.  **Where justification reported:** NA.  **Categories reported:** Yes.  **Where categories reported:** Protocol and review.  **Justification for categories:** Not reported.  **Where justification reported:** NA. | **Analysis type reported in protocol:** Subgroup analysis and meta-regression.  **Analysis type reported in review:** Subgroup analysis.  **Reason for changing mind:** Not reported.    **Analysis type in protocol according to glossary:** Subgroup analysis and meta-regression.  **How determined the analysis type:** Categories given.  **Analysis type in review according to glossary:** Subgroup analysis.  **How determined the analysis type:** Categories given.  **Analysed:** No.  **Reasons given (in the review) for not doing interaction analysis if planned:** ‘However, the paucity of trials and lack of data in included studies  allowed for subgroup analyses of the last count alone.’  **Reasons given for carrying out interaction analysis if they weren’t planned:** NA.  **Scenario in protocol: ‘**We will investigate heterogeneity in each analysis  based on the following pre-planned subgroup analyses’ ‘if enough trials will be available’ (for meta-regression).  **Rationale for planned scenario:** protocol.  **Scenario in review (if reported specifically): ‘**We set to investigate heterogeneity in each analysis  based on the following preplanned subgroup analyses’  **Rationale for done scenario:** Not reported.  **Reason for changing mind:** NA. | **Method to detect interactions reported in protocol:** Not reported.  **Method to detect interactions reported in review:** Not reported.  **Reason for changing mind:** NA. |
| **Itchaki 2013** | **Protocol: ‘**FL grade 3’. ‘The percentage of patients with FL grade 3 in the trial’.  **Review methods: ‘**FL grade 3’.  **Review results:** Not analysed.  **Type:** Patient.  **Covariate summary:** Disease characteristics. | **Method to choose**  **covariate:** Not reported.  **Where method reported:** NA.  **Rationale for choosing the covariate:** Not reported.  **Where rationale reported:** NA. | **Where reported:** Protocol and review.  **Reason not reported in review:** NA.  **Reason not reported in protocol:** NA.  **Labelled as ‘posthoc’ if not in protocol:** NA. | **Data type:** Categorical.  **Continuous covariate categorised:** NA.  **How determine it was categorised:** NA.  **Justification for categorising:** NA.  **Where justification reported:** NA.  **Categories reported:** Not reported.  **Where categories reported:** NA.  **Justification for categories:** Not reported.  **Where justification reported:** NA. | **Analysis type reported in protocol:** Subgroup analysis and meta-regression.  **Analysis type reported in review:** Subgroup analysis.  **Reason for changing mind:** Not reported.    **Analysis type in protocol according to glossary:** Sensitivity analysis and meta-regression.  **How determined the analysis type:** Categories given.  **Analysis type in review according to glossary:** Sensitivity analysis  **How determined the analysis type:** Categories given.  **Analysed:** No.  **Reasons given (in the review) for not doing interaction analysis if planned:** ‘However, the paucity of trials and lack of data in included studies  allowed for subgroup analyses of the last count alone.’’ There were not sufficient outcome data for subgroup analysis according  to grade of FL or age of patients.’  **Reasons given for carrying out interaction analysis if they weren’t planned:** NA.  **Scenario in protocol: ‘**We will investigate heterogeneity in each analysis  based on the following pre-planned subgroup analyses’ ‘if enough trials will be available’ (for meta-regression).  **Rationale for planned scenario:** protocol.  **Scenario in review (if reported specifically): ‘**We set to investigate heterogeneity in each analysis  based on the following preplanned subgroup analyses’  **Rationale for done scenario:** Not reported.  **Reason for changing mind:** NA. | **Method to detect interactions reported in protocol:** Not reported.  **Method to detect interactions reported in review:** Not reported.  **Reason for changing mind:** NA. |
| **Itchaki 2013** | **Protocol:** ‘Regimens with or without monoclonal antibodies’.  **Review methods: ‘**Regimens with or without monoclonal antibodies’.  **Review results:** Not analysed.  **Type:** Intervention.  **Covariate summary:** Additional interventions. | **Method to choose**  **covariate:** Not reported.  **Where method reported:** NA.  **Rationale for choosing the covariate:** Not reported.  **Where rationale reported:** NA. | **Where reported:** Protocol and review.  **Reason not reported in review:** NA.  **Reason not reported in protocol:** NA.  **Labelled as ‘posthoc’ if not in protocol:** NA. | **Data type:** Categorical.  **Continuous covariate categorised:** NA.  **How determine it was categorised:** NA.  **Justification for categorising:** NA.  **Where justification reported:** NA.  **Categories reported:** Yes.  **Where categories reported:** Protocol and review.  **Justification for categories:** Not reported.  **Where justification reported:** NA. | **Analysis type reported in protocol:** Subgroup analysis.  **Analysis type reported in review:** Subgroup analysis.  **Reason for changing mind:** NA.    **Analysis type in protocol according to glossary:** Subgroup analysis.  **How determined the analysis type:** Categories given.  **Analysis type in review according to glossary:** Subgroup analysis.  **How determined the analysis type:** Categories given.  **Analysed:** No.  **Reasons given (in the review) for not doing interaction analysis if planned:** ‘However, the paucity of trials and lack of data in included studies  allowed for subgroup analyses of the last count alone.’ ‘Only Federico 2013 had included  monoclonal antibodies (rituximab) in study arms. Its results were  consistent with those of the other studies.’  **Reasons given for carrying out interaction analysis if they weren’t planned:** NA.  **Scenario in protocol: ‘**We will investigate heterogeneity in each analysis  based on the following pre-planned subgroup analyses’  **Rationale for planned scenario:** protocol.  **Scenario in review (if reported specifically): ‘**We set to investigate heterogeneity in each analysis  based on the following preplanned subgroup analyses’  **Rationale for done scenario:** Not reported.  **Reason for changing mind:** NA. | **Method to detect interactions reported in protocol:** Not reported.  **Method to detect interactions reported in review:** Not reported.  **Reason for changing mind:** NA. |
| **Itchaki 2013** | **Protocol: ‘**We will separate all analyses for trials in which the only difference between study arms is the addition of an anthracycline (“same” comparison) and trials where different chemotherapy regimens are used in the ACR and non-ACR arms.’ **‘**As detailed above, we will conduct a separate analysis for studies assessing directly the efficacy of anthracyclines by comparing the same chemotherapy regimens with or without the addition of an anthracycline.’  **Review methods:** ‘We separated trials according to the similarity of chemotherapeutic  regimens, other than anthracyclines, between study arms. Trials with the same chemotherapy, where the main difference was the addition of an anthracycline, were summed together.’ ‘As detailed above, we conducted a separate analysis for studies assessing directly the efficacy of anthracyclines by comparing the same chemotherapy regimens with or without the addition of an anthracycline.’  **Review results:** ‘Studies comparing same chemotherapy’; ‘Studies comparing different chemotherapies’ (in text). ‘Anthracycline versus no anthracycline different chemotherapy’; ‘Anthracycline versus no anthracycline same chemotherapy’ (in plot).  **Type:** Intervention.  **Covariate summary:** Additional drugs. | **Method to choose**  **covariate:** Not reported.  **Where method reported:** NA.  **Rationale for choosing the covariate:** Not reported.  **Where rationale reported:** NA. | **Where reported:** Protocol and review.  **Reason not reported in review:** NA.  **Reason not reported in protocol:** NA.  **Labelled as ‘posthoc’ if not in protocol:** NA. | **Data type:** Categorical.  **Continuous covariate categorised:** NA.  **How determine it was categorised:** NA.  **Justification for categorising:** NA.  **Where justification reported:** NA.  **Categories reported:** Yes.  **Where categories reported:** Protocol and review.  **Justification for categories:** Not reported.  **Where justification reported:** NA. | **Analysis type reported in protocol:** Unnamed analysis.  **Analysis type reported in review:** Unnamed analysis.  **Reason for changing mind:** NA.    **Analysis type in protocol according to glossary:** Subgroup analysis.  **How determined the analysis type:** Categories given.  **Analysis type in review according to glossary:** Subgroup analysis.  **How determined the analysis type:** Categories given.  **Analysed:** Yes.  **Reasons given (in the review) for not doing interaction analysis if planned:** NA.  **Reasons given for carrying out interaction analysis if they weren’t planned:** NA.  **Scenario in protocol:** Not reported.  **Rationale for planned scenario:** NA.  **Scenario in review (if reported specifically):** Not reported.  **Rationale for done scenario:** NA.  **Reason for changing mind:** NA. | **Method to detect interactions reported in protocol:** Not reported.  **Method to detect interactions reported in review:** Not reported.  **Reason for changing mind:** NA. |
| **Itchaki 2013** | **Protocol:** ‘We will separate initial treatment from second-line treatment.’  **Review methods:** Not reported.  **Review results:** Not analysed.  **Type:** Intervention.  **Covariate summary:** Type of intervention or control | **Method to choose**  **covariate:** Not reported.  **Where method reported:** NA.  **Rationale for choosing the covariate:** Not reported.  **Where rationale reported:** NA. | **Where reported:** Protocol only.  **Reason not reported in review:** Not reported.  **Reason not reported in protocol:** NA.  **Labelled as ‘posthoc’ if not in protocol:** NA. | **Data type:** Categorical.  **Continuous covariate categorised:** NA.  **How determine it was categorised:** NA.  **Justification for categorising:** NA.  **Where justification reported:** NA.  **Categories reported:** Yes.  **Where categories reported:** Protocol only.  **Justification for categories:** Not reported.  **Where justification reported:** NA. | **Analysis type reported in protocol:** Unnamed analysis.  **Analysis type reported in review:** Not reported.  **Reason for changing mind:** Not reported.    **Analysis type in protocol according to glossary:** Subgroup analysis.  **How determined the analysis type:** Categories given.  **Analysis type in review according to glossary:** Not reported  **How determined the analysis type:** NA.  **Analysed:** No.  **Reasons given (in the review) for not doing interaction analysis if planned:** ‘Also, all patients included  had first-line therapy.’  **Reasons given for carrying out interaction analysis if they weren’t planned:** NA.  **Scenario in protocol:** Not reported.  **Rationale for planned scenario:** NA.  **Scenario in review (if reported specifically):** Not reported.  **Rationale for done scenario:** NA.  **Reason for changing mind:** NA. | **Method to detect interactions reported in protocol:** Not reported.  **Method to detect interactions reported in review:** Not reported.  **Reason for changing mind:** NA. |
| **Itchaki 2013** | **Protocol:** Not reported.  **Review methods: ‘**Publication status: published in a peer-reviewed journal.’  **Review results:** Not analysed.  **Type:** Methodological.  **Covariate summary:** Publication. | **Method to choose**  **covariate:** Not reported.  **Where method reported:** NA.  **Rationale for choosing the covariate:** Not reported.  **Where rationale reported:** NA. | **Where reported:** Review only.  **Reason not reported in review:** NA.  **Reason not reported in protocol:** Not reported.  **Labelled as ‘posthoc’ if not in protocol:** No. | **Data type:** Categorical.  **Continuous covariate categorised:** NA.  **How determine it was categorised:** NA.  **Justification for categorising:** NA.  **Where justification reported:** NA.  **Categories reported:** Yes.  **Where categories reported:** Review only.  **Justification for categories:** Not reported.  **Where justification reported:** NA. | **Analysis type reported in protocol:** Not reported.  **Analysis type reported in review:** Sensitivity analysis.  **Reason for changing mind:** Not reported.    **Analysis type in protocol according to glossary:** Not reported.  **How determined the analysis type:** NA.  **Analysis type in review according to glossary:** Sensitivity analysis.  **How determined the analysis type:** Says ‘Sensitivity analysis’. No reason to dispute.  **Analysed:** No.  **Reasons given (in the review) for not doing interaction analysis if planned:** ‘Given the paucity of included trials, we were able to fulfil only  the first two counts, and did not undertake any further sensitivity  analyses.’ ‘All studies were published as peer-reviewed journal articles.’  **Reasons given for carrying out interaction analysis if they weren’t planned:** NA.  **Scenario in protocol:** Not reported.  **Rationale for planned scenario:** NA.  **Scenario in review (if reported specifically):** Not reported.  **Rationale for done scenario:** NA.  **Reason for changing mind:** NA. | **Method to detect interactions reported in protocol:** Not reported.  **Method to detect interactions reported in review:** Not reported.  **Reason for changing mind:** NA. |
| **Itchaki 2013** | **Protocol:** Not reported.  **Review methods: ‘**Trials that encompassed a second randomization.’  **Review results:** Not analysed.  **Type:** Methodological.  **Covariate summary:** Second randomisation | **Method to choose**  **covariate:** Not reported.  **Where method reported:** NA.  **Rationale for choosing the covariate:** Not reported.  **Where rationale reported:** NA. | **Where reported:** Review only.  **Reason not reported in review:** NA.  **Reason not reported in protocol:** Not reported.  **Labelled as ‘posthoc’ if not in protocol:** No. | **Data type:** Categorical.  **Continuous covariate categorised:** NA.  **How determine it was categorised:** NA.  **Justification for categorising:** NA.  **Where justification reported:** NA.  **Categories reported:** Yes.  **Where categories reported:** Review only.  **Justification for categories:** Not reported.  **Where justification reported:** NA. | **Analysis type reported in protocol:** Not reported.  **Analysis type reported in review:** Sensitivity analysis.  **Reason for changing mind:** Not reported.    **Analysis type in protocol according to glossary:** Not reported.  **How determined the analysis type:** NA.  **Analysis type in review according to glossary:** Sensitivity analysis.  **How determined the analysis type:** Says ‘Sensitivity analysis’. No reason to dispute.  **Analysed:** No.  **Reasons given (in the review) for not doing interaction analysis if planned:** ‘Given the paucity of included trials, we were able to fulfil only  the first two counts, and did not undertake any further sensitivity  analyses.’ ‘Asmentioned above, three studies proceeded to a second randomization  (Jones 1983; Lepage 1990; Taylor 2006), and only these  trials reported OS.’  **Reasons given for carrying out interaction analysis if they weren’t planned:** NA.  **Scenario in protocol:** Not reported.  **Rationale for planned scenario:** NA.  **Scenario in review (if reported specifically):** Not reported.  **Rationale for done scenario:** NA.  **Reason for changing mind:** NA. | **Method to detect interactions reported in protocol:** Not reported.  **Method to detect interactions reported in review:** Not reported.  **Reason for changing mind:** NA. |
| Kinnersley 2013 | **Protocol:** ‘Age of patients (young 16 to 35; middle aged 36 to 60; older 61 to 80, elderly 81+)’.  **Review methods: ‘**Age of patients (young 16 to 35; middle aged 36 to 60 years; older 61 to 80, elderly over 80 years)’.  **Review results:** Not analysed.  **Type:** Patient.  **Covariate summary:** Demographics. | **Method to choose**  **covariate:** Not reported.  **Where method reported:** NA.  **Rationale for choosing the covariate:** ‘Several features of the interaction between patient and clinician  may affect communication, the interaction, and thus the opportunity  to achieve informed consent for procedures.We plan to make  the following comparisons:’.  **Where rationale reported:** Protocol and review. | **Where reported:** Protocol and review.  **Reason not reported in review:** NA.  **Reason not reported in protocol:** NA.  **Labelled as ‘posthoc’ if not in protocol:** NA. | **Data type:** Continuous.  **Continuous covariate categorised:** Yes.  **How determine it was categorised:** Categories given.  **Justification for categorising:** Not reported.  **Where justification reported:** NA.  **Categories reported:** Yes.  **Where categories reported:** Protocol and review.  **Justification for categories:** Not reported.  **Where justification reported:** NA. | **Analysis type reported in protocol:** Visually, subgroup analysis, meta-regression.  **Analysis type reported in review:** Subgroup analysis.  **Reason for changing mind:** Not reported.    **Analysis type in protocol according to glossary:** subgroup analysis, meta-regression.  **How determined the analysis type:** Says’ subgroup analysis, meta-regression.’. No reason to dispute.  **Analysis type in review according to glossary:** Subgroup analysis.  **How determined the analysis type:** ‘For the subgroup analyses we divided the studies into the subgroups  of interest and conducted meta-analyses where possible.’  **Analysed:** No.  **Reasons given (in the review) for not doing interaction analysis if planned:** Not reported.  **Reasons given for carrying out interaction analysis if they weren’t planned:** NA.  **Scenario in protocol:** Meta-regression only if the ‘number of trials permits’.  **Rationale for planned scenario:** Not reported.  **Scenario in review (if reported specifically):** Not reported.  **Rationale for done scenario:** NA.  **Reason for changing mind:** NA. | **Method to detect interactions reported in protocol:** ‘Initially, we will investigate these potential differences visually (for  example by using box plots), and then by subgroup analyses or,  if the number of trials permits, by using random-effects metaregression.  The latter will be preferential because it will allow us  to estimate the relative change and confidence intervals.’  **Method to detect interactions reported in review:** Not reported.  **Reason for changing mind:** Not reported. |
| Kinnersley 2013 | **Protocol:** ‘Comparisons will be between interventions and usual care (controls), and between different interventions if trials include multiple interventions.’  **Review methods:** ‘Comparisons were made between interventions and usual care (controls).’  **Review results:** Not analysed.  **Type:** Intervention.  **Covariate summary:** Type of intervention or control. | **Method to choose**  **covariate:** Not reported.  **Where method reported:** NA.  **Rationale for choosing the covariate:** Not reported.  **Where rationale reported:** NA. | **Where reported:** Protocol and review.  **Reason not reported in review:** NA.  **Reason not reported in protocol:** NA.  **Labelled as ‘posthoc’ if not in protocol:** NA. | **Data type:** Categorical.  **Continuous covariate categorised:** NA.  **How determine it was categorised:** NA.  **Justification for categorising:** NA.  **Where justification reported:** NA.  **Categories reported:** Not reported.  **Where categories reported:** NA.  **Justification for categories:** Not reported.  **Where justification reported:** NA. | **Analysis type reported in protocol:** Unnamed analysis.  **Analysis type reported in review:** Unnamed analysis.  **Reason for changing mind:** NA.    **Analysis type in protocol according to glossary:** Stratification/subgroup analysis.  **How determined the analysis type:** Categories given.  **Analysis type in review according to glossary:** Stratification/subgroup analysis.  **How determined the analysis type:** Categories given.  **Analysed:** No.  **Reasons given (in the review) for not doing interaction analysis if planned:** Not reported (presumably because one trial).  **Reasons given for carrying out interaction analysis if they weren’t planned:** NA.  **Scenario in protocol:** Not reported.  **Rationale for planned scenario:** NA.  **Scenario in review (if reported specifically):** Not reported.  **Rationale for done scenario:** NA.  **Reason for changing mind:** NA. | **Method to detect interactions reported in protocol:** Not reported.  **Method to detect interactions reported in review:** Not reported.  **Reason for changing mind:** NA. |
| Kinnersley 2013 | **Protocol:** ‘Face to face interventions versus distant interventions (for example web-based)’.  **Review methods: ‘**Face to face interventions versus distant interventions (for example web-based)’.  **Review results:** Not analysed.  **Type:** Intervention.  **Covariate summary:** Type of intervention or control. | **Method to choose**  **covariate:** Not reported.  **Where method reported:** NA.  **Rationale for choosing the covariate:** ‘Several features of the interaction between patient and clinician  may affect communication, the interaction, and thus the opportunity  to achieve informed consent for procedures. e plan to make  the following comparisons:’.  **Where rationale reported:** Protocol and review. | **Where reported:** Protocol and review.  **Reason not reported in review:** NA.  **Reason not reported in protocol:** NA.  **Labelled as ‘posthoc’ if not in protocol:** NA. | **Data type:** Categorical.  **Continuous covariate categorised:** NA.  **How determine it was categorised:** NA.  **Justification for categorising:** NA.  **Where justification reported:** NA.  **Categories reported:** Yes.  **Where categories reported:** Protocol and review.  **Justification for categories:** Not reported.  **Where justification reported:** NA. | **Analysis type reported in protocol:** Visually, subgroup analysis, meta-regression.  **Analysis type reported in review:** Subgroup analysis.  **Reason for changing mind:** Not reported.    **Analysis type in protocol according to glossary:** subgroup analysis, meta-regression.  **How determined the analysis type:** Says’ subgroup analysis, meta-regression.’. No reason to dispute.  **Analysis type in review according to glossary:** Subgroup analysis.  **How determined the analysis type:** ‘For the subgroup analyses we divided the studies into the subgroups  of interest and conducted meta-analyses where possible.’  **Analysed:** No.  **Reasons given (in the review) for not doing interaction analysis if planned:** Not reported.  **Reasons given for carrying out interaction analysis if they weren’t planned:** NA.  **Scenario in protocol:** Meta-regression only if the ‘number of trials permits’.  **Rationale for planned scenario:** Not reported.  **Scenario in review (if reported specifically):** Not reported.  **Rationale for done scenario:** NA.  **Reason for changing mind:** NA. | **Method to detect interactions reported in protocol:** ‘Initially, we will investigate these potential differences visually (for  example by using box plots), and then by subgroup analyses or,  if the number of trials permits, by using random-effects meta-regression.  The latter will be preferential because it will allow us  to estimate the relative change and confidence intervals.’  **Method to detect interactions reported in review:** Not reported.  **Reason for changing mind:** Not reported. |
| Kinnersley 2013 | **Protocol:** ‘For the primary outcome (informed consent/choice), we will use reports of individual informed consent (achieved or not achieved) for meta-analysis. If there are studies which report changes in understanding or values or choices made at group level only we will report these separately and consider if it is appropriate to include them in a meta-analysis.’  **Review methods:** ‘For the primary outcome (informed consent/choice), we aimed to identify reports of individual informed consent (achieved or not achieved) for meta-analysis. If there are studies which report changes in understanding or values or choices made at group level only we will report these separately and consider if it is appropriate to include them in a meta-analysis.’  **Review results:** Not analysed.  **Type:** Outcome.  **Covariate summary:** Studies which report changes in understanding or values or choices made at group level only. | **Method to choose**  **covariate:** Not reported.  **Where method reported:** NA.  **Rationale for choosing the covariate:** Not reported.  **Where rationale reported:** NA. | **Where reported:** Protocol and review.  **Reason not reported in review:** NA.  **Reason not reported in protocol:** NA.  **Labelled as ‘posthoc’ if not in protocol:** NA. | **Data type:** Categorical.  **Continuous covariate categorised:** NA.  **How determine it was categorised:** NA.  **Justification for categorising:** NA.  **Where justification reported:** NA.  **Categories reported:** Yes.  **Where categories reported:** Protocol and review.  **Justification for categories:** Not reported.  **Where justification reported:** NA. | **Analysis type reported in protocol:** Unnamed analysis.  **Analysis type reported in review:** Unnamed analysis.  **Reason for changing mind:** NA.    **Analysis type in protocol according to glossary:** Stratification/subgroup analysis.  **How determined the analysis type:** Categories given.  **Analysis type in review according to glossary:** Stratification/subgroup analysis.  **How determined the analysis type:** Categories given.  **Analysed:** No.  **Reasons given (in the review) for not doing interaction analysis if planned:** Not reported (presumably because one trial).  **Reasons given for carrying out interaction analysis if they weren’t planned:** NA.  **Scenario in protocol:** ‘. If there are studies which report changes in understanding or values or choices made at group level only’  **Rationale for planned scenario:** Not reported.  **Scenario in review (if reported specifically):** ‘If there were studies which reported changes in understanding,’  **Rationale for done scenario:** Not reported.  **Reason for changing mind:** NA. | **Method to detect interactions reported in protocol:** Not reported.  **Method to detect interactions reported in review:** Not reported.  **Reason for changing mind:** NA. |
| Kinnersley 2013 | **Protocol:** ‘Interventions targeted at a condition. We judged the following to be two covariates: Interventions targeted at a specific procedure (i. e. whether to undergo, for example, an operation such as knee replacement for osteoarthritis) or at a condition more generally (but for which at least one option may be surgical (e. g. a decision aid addressing menorrhagia)).’  **Review methods: ‘**Interventions targeted at a condition. We judged the following to be two covariates: Interventions targeted at a specific procedure (i. e. whether to undergo, for example, an operation such as knee replacement for osteoarthritis) or at a condition more generally (but for which at least one option may be surgical (e. g. a decision aid addressing menorrhagia)).’  **Review results:** Not analysed.  **Type:** Intervention.  **Covariate summary:** Type of intervention or control. | **Method to choose**  **covariate:** Not reported.  **Where method reported:** NA.  **Rationale for choosing the covariate:** ‘Several features of the interaction between patient and clinician  may affect communication, the interaction, and thus the opportunity  to achieve informed consent for procedures.We plan to make  the following comparisons:’.  **Where rationale reported:** Protocol and review. | **Where reported:** Protocol and review.  **Reason not reported in review:** NA.  **Reason not reported in protocol:** NA.  **Labelled as ‘posthoc’ if not in protocol:** NA. | **Data type:** Categorical.  **Continuous covariate categorised:** NA.  **How determine it was categorised:** NA.  **Justification for categorising:** NA.  **Where justification reported:** NA.  **Categories reported:** Yes.  **Where categories reported:** Protocol and review.  **Justification for categories:** Not reported.  **Where justification reported:** NA. | **Analysis type reported in protocol:** Visually, subgroup analysis, meta-regression.  **Analysis type reported in review:** Subgroup analysis.  **Reason for changing mind:** Not reported.    **Analysis type in protocol according to glossary:** subgroup analysis, meta-regression.  **How determined the analysis type:** Says’ subgroup analysis, meta-regression.’. No reason to dispute.  **Analysis type in review according to glossary:** Subgroup analysis.  **How determined the analysis type:** ‘For the subgroup analyses we divided the studies into the subgroups  of interest and conducted meta-analyses where possible.’  **Analysed:** No.  **Reasons given (in the review) for not doing interaction analysis if planned:** Not reported.  **Reasons given for carrying out interaction analysis if they weren’t planned:** NA.  **Scenario in protocol:** Meta-regression only if the ‘number of trials permits’.  **Rationale for planned scenario:** Not reported.  **Scenario in review (if reported specifically):** Not reported.  **Rationale for done scenario:** NA.  **Reason for changing mind:** NA. | **Method to detect interactions reported in protocol:** ‘Initially, we will investigate these potential differences visually (for  example by using box plots), and then by subgroup analyses or,  if the number of trials permits, by using random-effects metaregression.  The latter will be preferential because it will allow us  to estimate the relative change and confidence intervals.’  **Method to detect interactions reported in review:** Not reported.  **Reason for changing mind:** Not reported. |
| Kinnersley 2013 | **Protocol:** ‘Interventions targeted at a specific procedure. We judged the following to be two covariates: Interventions targeted at a specific procedure (i. e. whether to undergo, for example, an operation such as knee replacement for osteoarthritis) or at a condition more generally (but for which at least one option may be surgical (e. g. a decision aid addressing menorrhagia)).’  **Review methods: ‘**Interventions targeted at a specific procedure. We judged the following to be two covariates: Interventions targeted at a specific procedure (i. e. whether to undergo, for example, an operation such as knee replacement for osteoarthritis) or at a condition more generally (but for which at least one option may be surgical (e. g. a decision aid addressing menorrhagia)).’  **Review results:** Not analysed.  **Type:** Intervention.  **Covariate summary:** Type of intervention or control. | **Method to choose**  **covariate:** Not reported.  **Where method reported:** NA.  **Rationale for choosing the covariate:** ‘Several features of the interaction between patient and clinician  may affect communication, the interaction, and thus the opportunity  to achieve informed consent for procedures.We plan to make  the following comparisons:’.  **Where rationale reported:** Protocol and review. | **Where reported:** Protocol and review.  **Reason not reported in review:** NA.  **Reason not reported in protocol:** NA.  **Labelled as ‘posthoc’ if not in protocol:** NA. | **Data type:** Categorical.  **Continuous covariate categorised:** NA.  **How determine it was categorised:** NA.  **Justification for categorising:** NA.  **Where justification reported:** NA.  **Categories reported:** Yes.  **Where categories reported:** Protocol and review.  **Justification for categories:** Not reported.  **Where justification reported:** NA. | **Analysis type reported in protocol:** Visually, subgroup analysis, meta-regression.  **Analysis type reported in review:** Subgroup analysis.  **Reason for changing mind:** Not reported.    **Analysis type in protocol according to glossary:** subgroup analysis, meta-regression.  **How determined the analysis type:** Says’ subgroup analysis, meta-regression.’. No reason to dispute.  **Analysis type in review according to glossary:** Subgroup analysis.  **How determined the analysis type:** ‘For the subgroup analyses we divided the studies into the subgroups  of interest and conducted meta-analyses where possible.’  **Analysed:** No.  **Reasons given (in the review) for not doing interaction analysis if planned:** Not reported.  **Reasons given for carrying out interaction analysis if they weren’t planned:** NA.  **Scenario in protocol:** Meta-regression only if the ‘number of trials permits’.  **Rationale for planned scenario:** Not reported.  **Scenario in review (if reported specifically):** Not reported.  **Rationale for done scenario:** NA.  **Reason for changing mind:** NA. | **Method to detect interactions reported in protocol:** ‘Initially, we will investigate these potential differences visually (for  example by using box plots), and then by subgroup analyses or,  if the number of trials permits, by using random-effects metaregression.  The latter will be preferential because it will allow us  to estimate the relative change and confidence intervals.’  **Method to detect interactions reported in review:** Not reported.  **Reason for changing mind:** Not reported. |
| Kinnersley 2013 | **Protocol:** ‘Interventions targeted at clinicians versus those targeted at patients or at organisational change.’  **Review methods: ‘**Interventions targeted at clinicians versus those targeted at patients or at organisational change.’  **Review results:** Not analysed.  **Type:** Intervention.  **Covariate summary:** Type of intervention or control. | **Method to choose**  **covariate:** Not reported.  **Where method reported:** NA.  **Rationale for choosing the covariate:** ‘Several features of the interaction between patient and clinician  may affect communication, the interaction, and thus the opportunity  to achieve informed consent for procedures. We plan to make  the following comparisons:’.  **Where rationale reported:** Protocol and review. | **Where reported:** Protocol and review.  **Reason not reported in review:** NA.  **Reason not reported in protocol:** NA.  **Labelled as ‘posthoc’ if not in protocol:** NA. | **Data type:** Categorical.  **Continuous covariate categorised:** NA.  **How determine it was categorised:** NA.  **Justification for categorising:** NA.  **Where justification reported:** NA.  **Categories reported:** Yes.  **Where categories reported:** Protocol and review.  **Justification for categories:** Not reported.  **Where justification reported:** NA. | **Analysis type reported in protocol:** Visually, subgroup analysis, meta-regression.  **Analysis type reported in review:** Subgroup analysis.  **Reason for changing mind:** Not reported.    **Analysis type in protocol according to glossary:** subgroup analysis, meta-regression.  **How determined the analysis type:** Says’ subgroup analysis, meta-regression.’. No reason to dispute.  **Analysis type in review according to glossary:** Subgroup analysis.  **How determined the analysis type:** ‘For the subgroup analyses we divided the studies into the subgroups  of interest and conducted meta-analyses where possible.’  **Analysed:** No.  **Reasons given (in the review) for not doing interaction analysis if planned:** Not reported.  **Reasons given for carrying out interaction analysis if they weren’t planned:** NA.  **Scenario in protocol:** Meta-regression only if the ‘number of trials permits’.  **Rationale for planned scenario:** Not reported.  **Scenario in review (if reported specifically):** Not reported.  **Rationale for done scenario:** NA.  **Reason for changing mind:** NA. | **Method to detect interactions reported in protocol:** ‘Initially, we will investigate these potential differences visually (for  example by using box plots), and then by subgroup analyses or,  if the number of trials permits, by using random-effects metaregression.  The latter will be preferential because it will allow us  to estimate the relative change and confidence intervals.’  **Method to detect interactions reported in review:** Not reported.  **Reason for changing mind:** Not reported. |
| Kinnersley 2013 | **Protocol:** Allocation concealment. We judged the following to be three covariates: ‘We will conduct a sensitivity analysis based on the risk of bias identified in the studies. Studies identified as being of greatest risk of bias, specifically regarding allocation concealment and blinding of intervention and outcome assessment will be systematically removed from the analysis and we will report the effects on the effect estimate.’  **Review methods:** Not reported.  **Review results:** Not analysed.  **Type:** Methodological.  **Covariate summary:** Allocation concealment. | **Method to choose**  **covariate:** Not reported.  **Where method reported:** NA.  **Rationale for choosing the covariate:** Not reported.  **Where rationale reported:** NA. | **Where reported:** Protocol only.  **Reason not reported in review:** Not reported.  **Reason not reported in protocol:** NA.  **Labelled as ‘posthoc’ if not in protocol:** NA. | **Data type:** Categorical.  **Continuous covariate categorised:** NA.  **How determine it was categorised:** NA.  **Justification for categorising:** NA.  **Where justification reported:** NA.  **Categories reported:** Yes.  **Where categories reported:** Protocol only.  **Justification for categories:** Not reported.  **Where justification reported:** NA. | **Analysis type reported in protocol:** Sensitivity analysis.  **Analysis type reported in review:** Not reported.  **Reason for changing mind:** Not reported.    **Analysis type in protocol according to glossary:** Sensitivity analysis.  **How determined the analysis type:** Categories given.  **Analysis type in review according to glossary:** Not reported.  **How determined the analysis type:** NA.  **Analysed:** No.  **Reasons given (in the review) for not doing interaction analysis if planned:** Not reported (presumably because one trial).  **Reasons given for carrying out interaction analysis if they weren’t planned:** NA.  **Scenario in protocol:** Not reported.  **Rationale for planned scenario:** NA.  **Scenario in review (if reported specifically):** Not reported.  **Rationale for done scenario:** NA.  **Reason for changing mind:** NA. | **Method to detect interactions reported in protocol:** Not reported.  **Method to detect interactions reported in review:** Not reported.  **Reason for changing mind:** NA. |
| Kinnersley 2013 | **Protocol:** Blinding of intervention. We judged the following to be three covariates: ‘We will conduct a sensitivity analysis based on the risk of bias identified in the studies. Studies identified as being of greatest risk of bias, specifically regarding allocation concealment and blinding of intervention and outcome assessment will be systematically removed from the analysis and we will report the effects on the effect estimate.’  **Review methods:** Not reported.  **Review results:** Not analysed.  **Type:** Methodological.  **Covariate summary:** Blinding. | **Method to choose**  **covariate:** Not reported.  **Where method reported:** NA.  **Rationale for choosing the covariate:** Not reported.  **Where rationale reported:** NA. | **Where reported:** Protocol only.  **Reason not reported in review:** Not reported.  **Reason not reported in protocol:** NA.  **Labelled as ‘posthoc’ if not in protocol:** NA. | **Data type:** Categorical.  **Continuous covariate categorised:** NA.  **How determine it was categorised:** NA.  **Justification for categorising:** NA.  **Where justification reported:** NA.  **Categories reported:** Yes.  **Where categories reported:** Protocol only.  **Justification for categories:** Not reported.  **Where justification reported:** NA. | **Analysis type reported in protocol:** Sensitivity analysis.  **Analysis type reported in review:** Not reported.  **Reason for changing mind:** Not reported.    **Analysis type in protocol according to glossary:** Sensitivity analysis.  **How determined the analysis type:** Categories given.  **Analysis type in review according to glossary:** Not reported.  **How determined the analysis type:** NA.  **Analysed:** No.  **Reasons given (in the review) for not doing interaction analysis if planned:** Not reported (presumably because one trial).  **Reasons given for carrying out interaction analysis if they weren’t planned:** NA.  **Scenario in protocol:** Not reported.  **Rationale for planned scenario:** NA.  **Scenario in review (if reported specifically):** Not reported.  **Rationale for done scenario:** NA.  **Reason for changing mind:** NA. | **Method to detect interactions reported in protocol:** Not reported.  **Method to detect interactions reported in review:** Not reported.  **Reason for changing mind:** NA. |
| Kinnersley 2013 | **Protocol:** Blinding of outcome assessment. We judged the following to be three covariates: ‘We will conduct a sensitivity analysis based on the risk of bias identified in the studies. Studies identified as being of greatest risk of bias, specifically regarding allocation concealment and blinding of intervention and outcome assessment will be systematically removed from the analysis and we will report the effects on the effect estimate.’  **Review methods:** Blinding of outcome assessment We judged the following to be three covariates: ‘Studies identified as being of greatest risk of bias, specifically regarding randomisation, attrition and blinded outcome assessment were systematically removed from the analysis and we report the effects on the effect estimate.’  **Review results:** Not analysed.  **Type:** Methodological.  **Covariate summary:** Blinding. | **Method to choose**  **covariate:** Not reported.  **Where method reported:** NA.  **Rationale for choosing the covariate:** Not reported.  **Where rationale reported:** NA. | **Where reported:** Protocol and review.  **Reason not reported in review:** NA.  **Reason not reported in protocol:** NA.  **Labelled as ‘posthoc’ if not in protocol:** NA. | **Data type:** Categorical.  **Continuous covariate categorised:** NA.  **How determine it was categorised:** NA.  **Justification for categorising:** NA.  **Where justification reported:** NA.  **Categories reported:** Yes.  **Where categories reported:** Protocol and review.  **Justification for categories:** Not reported.  **Where justification reported:** NA. | **Analysis type reported in protocol:** Sensitivity analysis.  **Analysis type reported in review:** Sensitivity analysis.  **Reason for changing mind:** NA.    **Analysis type in protocol according to glossary:** Sensitivity analysis.  **How determined the analysis type:** Categories given.  **Analysis type in review according to glossary:** Sensitivity analysis.  **How determined the analysis type:** Categories given.  **Analysed:** No.  **Reasons given (in the review) for not doing interaction analysis if planned:** Not reported (presumably because one trial).  **Reasons given for carrying out interaction analysis if they weren’t planned:** NA.  **Scenario in protocol:** Not reported.  **Rationale for planned scenario:** NA.  **Scenario in review (if reported specifically):** Not reported.  **Rationale for done scenario:** NA.  **Reason for changing mind:** NA. | **Method to detect interactions reported in protocol:** Not reported.  **Method to detect interactions reported in review:** Not reported.  **Reason for changing mind:** NA. |
| Kinnersley 2013 | **Protocol:** Not reported.  **Review methods:** Attrition. We judged the following to be three covariates: ‘Studies identified as being of greatest risk ofbias, specifically regarding randomisation, attrition and blinded outcome assessment were systematically removed from the analysis and we report the effects on the effect estimate.’  **Review results:** Not analysed.  **Type:** Methodological.  **Covariate summary:** Incomplete outcome data/follow up. | **Method to choose**  **covariate:** Not reported.  **Where method reported:** NA.  **Rationale for choosing the covariate:** Not reported.  **Where rationale reported:** NA. | **Where reported:** Review only.  **Reason not reported in review:** NA.  **Reason not reported in protocol:** Not reported.  **Labelled as ‘posthoc’ if not in protocol:** No. | **Data type:** Categorical.  **Continuous covariate categorised:** NA.  **How determine it was categorised:** NA.  **Justification for categorising:** NA.  **Where justification reported:** NA.  **Categories reported:** Yes.  **Where categories reported:** Review only.  **Justification for categories:** Not reported.  **Where justification reported:** NA. | **Analysis type reported in protocol:** Not reported.  **Analysis type reported in review:** Sensitivity analysis.  **Reason for changing mind:** Not reported.    **Analysis type in protocol according to glossary:** Not reported.  **How determined the analysis type:** NA.  **Analysis type in review according to glossary:** Sensitivity analysis.  **How determined the analysis type:** Categories given.  **Analysed:** No.  **Reasons given (in the review) for not doing interaction analysis if planned:** Not reported (presumably because one trial).  **Reasons given for carrying out interaction analysis if they weren’t planned:** NA.  **Scenario in protocol:** Not reported.  **Rationale for planned scenario:** NA.  **Scenario in review (if reported specifically):** Not reported.  **Rationale for done scenario:** NA.  **Reason for changing mind:** NA. | **Method to detect interactions reported in protocol:** Not reported.  **Method to detect interactions reported in review:** Not reported.  **Reason for changing mind:** NA. |
| Kinnersley 2013 | **Protocol:** Not reported.  **Review methods:** Randomisation. We judged the following to be three covariates: ‘Studies identified as being of greatest risk of bias, specifically regarding randomisation, attrition and blinded outcome assessment were systematically removed from the analysis and we report the effects on the effect estimate.’  **Review results:** Not analysed.  **Type:** Methodological.  **Covariate summary:** Randomisation. | **Method to choose**  **covariate:** Not reported.  **Where method reported:** NA.  **Rationale for choosing the covariate:** Not reported.  **Where rationale reported:** NA. | **Where reported:** Review only.  **Reason not reported in review:** NA.  **Reason not reported in protocol:** Not reported.  **Labelled as ‘posthoc’ if not in protocol:** No. | **Data type:** Categorical.  **Continuous covariate categorised:** NA.  **How determine it was categorised:** NA.  **Justification for categorising:** NA.  **Where justification reported:** NA.  **Categories reported:** Yes.  **Where categories reported:** Review only.  **Justification for categories:** Not reported.  **Where justification reported:** NA. | **Analysis type reported in protocol:** Not reported.  **Analysis type reported in review:** Sensitivity analysis.  **Reason for changing mind:** Not reported.    **Analysis type in protocol according to glossary:** Not reported.  **How determined the analysis type:** NA.  **Analysis type in review according to glossary:** Sensitivity analysis.  **How determined the analysis type:** Categories given.  **Analysed:** No.  **Reasons given (in the review) for not doing interaction analysis if planned:** Not reported (presumably because one trial).  **Reasons given for carrying out interaction analysis if they weren’t planned:** NA.  **Scenario in protocol:** Not reported.  **Rationale for planned scenario:** NA.  **Scenario in review (if reported specifically):** Not reported.  **Rationale for done scenario:** NA.  **Reason for changing mind:** NA. | **Method to detect interactions reported in protocol:** Not reported.  **Method to detect interactions reported in review:** Not reported.  **Reason for changing mind:** NA. |
| Lawrie 2013 | **Protocol: ‘**A sensitivity analysis will be performed, re analysing the data excluding those studies classified as grade C (as above).’  **Review methods:** ‘We had planned to perform sensitivity analyses for survival outcomes by excluding trials which were at a high risk of bias.’  **Review results:** Not analysed.  **Type:** Methodological.  **Covariate summary:** Risk of bias. | **Method to choose**  **covariate:** Not reported.  **Where method reported:** NA.  **Rationale for choosing the covariate:** Not reported.  **Where rationale reported:** NA. | **Where reported:** Protocol and review.  **Reason not reported in review:** NA.  **Reason not reported in protocol:** NA.  **Labelled as ‘posthoc’ if not in protocol:** NA. | **Data type:** Categorical.  **Continuous covariate categorised:** NA.  **How determine it was categorised:** NA.  **Justification for categorising:** NA.  **Where justification reported:** NA.  **Categories reported:** Yes.  **Where categories reported:** Protocol and review.  **Justification for categories:** Not reported.  **Where justification reported:** NA. | **Analysis type reported in protocol:** Sensitivity analysis.  **Analysis type reported in review:** Sensitivity analysis.  **Reason for changing mind:** NA.    **Analysis type in protocol according to glossary:** Sensitivity analysis.  **How determined the analysis type:** Categories given.  **Analysis type in review according to glossary:** Sensitivity analysis.  **How determined the analysis type:** Categories given.  **Analysed:** No.  **Reasons given (in the review) for not doing interaction analysis if planned: ‘**However,  most of the studies at a high risk of bias had no useable data  and so could not be included in meta-analyses. Therefore, sensitivity  analyses were not performed.’  **Reasons given for carrying out interaction analysis if they weren’t planned:** NA.  **Scenario in protocol:** Not reported.  **Rationale for planned scenario:** NA.  **Scenario in review (if reported specifically):** Not reported.  **Rationale for done scenario:** NA.  **Reason for changing mind:** NA. | **Method to detect interactions reported in protocol:** Not reported.  **Method to detect interactions reported in review:** Not reported.  **Reason for changing mind:** NA. |
| Lawrie 2013 | **Protocol:** ‘The RCTs will be grouped by comparison, i. e. Pegylated liposomal doxorubicin versus best supportive care, single agent pegylated liposomal doxorubicin versus another single agent or pegylated liposomal doxorubicin plus another drug versus the same drug alone or in combination.’  **Review methods:** ‘The RCTs were grouped by Types of interventions. Where the types of interventions differed within a comparison, e. g. Other drugs versus PLD, we subgrouped data by the comparator drug and did not combine subgroup data’ ‘Types of intterventions: 1. PLD in combination with platinum-based therapy versus platinum-based therapy with another agent, e.g. PLD plus carboplatin versus paclitaxel (PAC) plus carboplatin. 2. Other chemotherapy agent(s) versus PLD, e.g. topotecan (TOP) versus PLD. 3. PLD plus other agent(s) versus PLD alone or with placebo, e.g. trabectedin (TBD) plus PLD versus PLD.’  **Review results:** ‘Comparison 1. PLD/carboplatin vs carboplatin ± other drug/s: PLD/carbo vs carbo only’; ‘Comparison 1. PLD/carboplatin vs carboplatin ± other drug/s: PLD/carbo vs PAC/carbo’; ‘Comparison 2. Other drug vs PLD: GEM vs PLD’; ‘Comparison 2. Other drug vs PLD: TOP vs PLD’; ‘Comparison 2. Other drug vs PLD: OLA vs PLD’; ‘Comparison 2. Other drug vs PLD: PAT vs PLD’; ‘Comparison 3. PLD + other drug vs PLD TBD/PLD vs PLD’; ‘Comparison 3. PLD + other drug vs PLD:CAN/PLD vs PLD’; ‘Comparison 3. PLD + other drug vs PLD: EC145/PLD vs PLD’.  **Type:** Intervention.  **Covariate summary:** Type of intervention or control. | **Method to choose**  **covariate:** Not reported.  **Where method reported:** NA.  **Rationale for choosing the covariate:** Not reported.  **Where rationale reported:** NA. | **Where reported:** Protocol and review.  **Reason not reported in review:** NA.  **Reason not reported in protocol:** NA.  **Labelled as ‘posthoc’ if not in protocol:** NA. | **Data type:** Categorical.  **Continuous covariate categorised:** NA.  **How determine it was categorised:** NA.  **Justification for categorising:** NA.  **Where justification reported:** NA.  **Categories reported:** Yes.  **Where categories reported:** Protocol and review.  **Justification for categories:** Not reported.  **Where justification reported:** NA. | **Analysis type reported in protocol:** Unnamed analysis.  **Analysis type reported in review:** Subgroup analysis.  **Reason for changing mind:** Not reported.    **Analysis type in protocol according to glossary:** Subgroup analysis.  **How determined the analysis type:** Categories given.  **Analysis type in review according to glossary:** Subgroup analysis.  **How determined the analysis type:** Categories given.  **Analysed:** Yes.  **Reasons given (in the review) for not doing interaction analysis if planned:** NA.  **Reasons given for carrying out interaction analysis if they weren’t planned:** NA.  **Scenario in protocol:** Not reported.  **Rationale for planned scenario:** NA.  **Scenario in review (if reported specifically):** Not reported.  **Rationale for done scenario:** NA.  **Reason for changing mind:** NA. | **Method to detect interactions reported in protocol:** Not reported.  **Method to detect interactions reported in review:** Not reported.  **Reason for changing mind:** NA. |
| Lawrie 2013 | **Protocol:** ‘The RCTs will be grouped by comparison….’. ‘Each group will then also be subdivided into platinum sensitive and platinum resistant. These groups will be considered separately.’  **Review methods: ‘**We had planned to subgroup survival outcomes by platinum sensitivity.’  **Review results:** Not analysed.  **Type:** Patient.  **Covariate summary:** Disease characteristics. | **Method to choose**  **covariate:** Not reported.  **Where method reported:** NA.  **Rationale for choosing the covariate:** Not reported.  **Where rationale reported:** NA. | **Where reported:** Protocol and review.  **Reason not reported in review:** NA.  **Reason not reported in protocol:** NA.  **Labelled as ‘posthoc’ if not in protocol:** NA. | **Data type:** Categorical.  **Continuous covariate categorised:** NA.  **How determine it was categorised:** NA.  **Justification for categorising:** NA.  **Where justification reported:** NA.  **Categories reported:** Yes.  **Where categories reported:** Protocol and review.  **Justification for categories:** Not reported.  **Where justification reported:** NA. | **Analysis type reported in protocol:** Unnamed analysis.  **Analysis type reported in review:** Subgroup analysis.  **Reason for changing mind:** Not reported.    **Analysis type in protocol according to glossary:** Stratification/subgroup analysis.  **How determined the analysis type:** Categories given.  **Analysis type in review according to glossary:** Stratification/subgroup analysis.  **How determined the analysis type:** Says ‘subgroup analysis’. No reason to dispute.  **Analysed:** No.  **Reasons given (in the review) for not doing interaction analysis if planned:** We had planned to subgroup  survival outcomes by platinum sensitivity, however, this was not  possible due to insufficient data.’  **Reasons given for carrying out interaction analysis if they weren’t planned:** NA.  **Scenario in protocol:** Not reported.  **Rationale for planned scenario:** NA.  **Scenario in review (if reported specifically):** Not reported.  **Rationale for done scenario:** NA.  **Reason for changing mind:** NA. | **Method to detect interactions reported in protocol:** Not reported.  **Method to detect interactions reported in review:** Not reported.  **Reason for changing mind:** NA. |
| Lee 2013 | **Protocol:** ‘Data from studies of acute bronchiectasis will be analysed separately to data from studies of stable bronchiectasis.’  **Review methods:** ‘We intended to analyse data from studies of acute bronchiectasis separately to data from studies of stable bronchiectasis.’  **Review results:** Not analysed.  **Type:** Patient.  **Covariate summary:** Disease characteristics. | **Method to choose**  **covariate:** Not reported.  **Where method reported:** NA.  **Rationale for choosing the covariate:** Not reported.  **Where rationale reported:** NA. | **Where reported:** Protocol and review.  **Reason not reported in review:** NA.  **Reason not reported in protocol:** NA.  **Labelled as ‘posthoc’ if not in protocol:** NA. | **Data type:** Categorical.  **Continuous covariate categorised:** NA.  **How determine it was categorised:** NA.  **Justification for categorising:** NA.  **Where justification reported:** NA.  **Categories reported:** Yes.  **Where categories reported:** Protocol and review.  **Justification for categories:** Not reported.  **Where justification reported:** NA. | **Analysis type reported in protocol:** Unnamed analysis.  **Analysis type reported in review:** Unnamed analysis.  **Reason for changing mind:** NA.    **Analysis type in protocol according to glossary:** Subgroup analysis.  **How determined the analysis type:** Categories given.  **Analysis type in review according to glossary:** Subgroup analysis.  **How determined the analysis type:** Categories given.  **Analysed:** No.  **Reasons given (in the review) for not doing interaction analysis if planned:** ‘however, no studies pertaining to people with an acute exacerbation of bronchiectasis were identified.’  **Reasons given for carrying out interaction analysis if they weren’t planned:** NA.  **Scenario in protocol:** Not reported.  **Rationale for planned scenario:** NA.  **Scenario in review (if reported specifically):** Not reported.  **Rationale for done scenario:** NA.  **Reason for changing mind:** NA. | **Method to detect interactions reported in protocol:** Not reported.  **Method to detect interactions reported in review:** Not reported.  **Reason for changing mind:** NA. |
| Lee 2013 | **Protocol:** ‘PEP devices and ‘conventional’ ACTs: due to a significant difference in the proposed physiological rationale underpinning their action.’  **Review methods:** ‘PEP devices: ACTs that use PEP may have differing physiological effects and outcomes compared to ACTs not using PEP.’  **Review results:** Not analysed.  **Type:** Intervention.  **Covariate summary:** Type of intervention or control. | **Method to choose**  **covariate:** Not reported.  **Where method reported:** NA.  **Rationale for choosing the covariate:** ‘due to a significant difference in the proposed physiological rationale underpinning their action.’  ‘ACTs that use PEP may have differing physiological effects and outcomes compared to ACTs not using PEP.’  **Where rationale reported:** Protocol and review. | **Where reported:** Protocol and review.  **Reason not reported in review:** NA.  **Reason not reported in protocol:** NA.  **Labelled as ‘posthoc’ if not in protocol:** NA. | **Data type:** Categorical.  **Continuous covariate categorised:** NA.  **How determine it was categorised:** NA.  **Justification for categorising:** NA.  **Where justification reported:** NA.  **Categories reported:** Yes.  **Where categories reported:** Protocol and review.  **Justification for categories:** ‘due to a significant difference in the proposed physiological rationale underpinning their action.’ ‘ACTs that use PEP may have differing physiological effects and outcomes compared to ACTs not using PEP.’  **Where justification reported:** Protocol and review. | **Analysis type reported in protocol:** Subgroup analysis.  **Analysis type reported in review:** Subgroup analysis.  **Reason for changing mind:** NA.    **Analysis type in protocol according to glossary:** Subgroup analysis.  **How determined the analysis type:** Categories given.  **Analysis type in review according to glossary:** Subgroup analysis.  **How determined the analysis type:** Categories given.  **Analysed:** No.  **Reasons given (in the review) for not doing interaction analysis if planned:** Not reported (presumably because one trial).  **Reasons given for carrying out interaction analysis if they weren’t planned:** NA.  **Scenario in protocol:** Not reported.  **Rationale for planned scenario:** NA.  **Scenario in review (if reported specifically):** Not reported.  **Rationale for done scenario:** NA.  **Reason for changing mind:** NA. | **Method to detect interactions reported in protocol:** Not reported.  **Method to detect interactions reported in review:** Not reported.  **Reason for changing mind:** NA. |
| Lee 2013 | **Protocol:** Allocation concealment. We judged the following to be three covariates: ‘A sensitivity analysis will be performed to analyse the effects of allocation concealment, assessor blinding and use of intention to treat analysis on results’.  **Review methods:** Allocation concealment. We judged the following to be three covariates: ‘We planned to perform a sensitivity analysis to analyse the effects of allocation concealment, assessor blinding and use of intention to treat analysis on results’.  **Review results:** Not analysed.  **Type:** Methodological.  **Covariate summary:** Allocation concealment. | **Method to choose**  **covariate:** Not reported.  **Where method reported:** NA.  **Rationale for choosing the covariate:** Not reported.  **Where rationale reported:** NA. | **Where reported:** Protocol and review.  **Reason not reported in review:** NA.  **Reason not reported in protocol:** NA.  **Labelled as ‘posthoc’ if not in protocol:** NA. | **Data type:** Categorical.  **Continuous covariate categorised:** NA.  **How determine it was categorised:** NA.  **Justification for categorising:** NA.  **Where justification reported:** NA.  **Categories reported:** Not reported.  **Where categories reported:** NA.  **Justification for categories:** Not reported.  **Where justification reported:** NA. | **Analysis type reported in protocol:** Sensitivity analysis.  **Analysis type reported in review:** Sensitivity analysis.  **Reason for changing mind:** NA.    **Analysis type in protocol according to glossary:** Sensitivity analysis.  **How determined the analysis type: ‘**Sensitivity analysis’. No reason to dispute.  **Analysis type in review according to glossary:** Sensitivity analysis.  **How determined the analysis type:** ‘Sensitivity analysis’. No reason to dispute.  **Analysed:** No.  **Reasons given (in the review) for not doing interaction analysis if planned: ‘**However, insufficient numbers of included studies precluded this analysis.’  **Reasons given for carrying out interaction analysis if they weren’t planned:** NA.  **Scenario in protocol:** Not reported.  **Rationale for planned scenario:** NA.  **Scenario in review (if reported specifically):** Not reported.  **Rationale for done scenario:** NA.  **Reason for changing mind:** NA. | **Method to detect interactions reported in protocol:** Not reported.  **Method to detect interactions reported in review:** Not reported.  **Reason for changing mind:** NA. |
| Lee 2013 | **Protocol:** Assessor blinding. We judged the following to be three covariates: ‘A sensitivity analysis will be performed to analyse the effects of allocation concealment, assessor blinding and use of intention to treat analysis on results’.  **Review methods:** Assessor blinding. We judged the following to be three covariates: ‘We planned to perform a sensitivity analysis to analyse the effects of allocation concealment, assessor blinding and use of intention to treat analysis on results’.  **Review results:** Not analysed.  **Type:** Methodological.  **Covariate summary:** Blinding. | **Method to choose**  **covariate:** Not reported.  **Where method reported:** NA.  **Rationale for choosing the covariate:** Not reported.  **Where rationale reported:** NA. | **Where reported:** Protocol and review.  **Reason not reported in review:** NA.  **Reason not reported in protocol:** NA.  **Labelled as ‘posthoc’ if not in protocol:** NA. | **Data type:** Categorical.  **Continuous covariate categorised:** NA.  **How determine it was categorised:** NA.  **Justification for categorising:** NA.  **Where justification reported:** NA.  **Categories reported:** Not reported.  **Where categories reported:** NA.  **Justification for categories:** Not reported.  **Where justification reported:** NA. | **Analysis type reported in protocol:** Sensitivity analysis.  **Analysis type reported in review:** Sensitivity analysis.  **Reason for changing mind:** NA.    **Analysis type in protocol according to glossary:** Sensitivity analysis.  **How determined the analysis type: ‘**Sensitivity analysis’. No reason to dispute.  **Analysis type in review according to glossary:** Sensitivity analysis.  **How determined the analysis type:** ‘Sensitivity analysis’. No reason to dispute.  **Analysed:** No.  **Reasons given (in the review) for not doing interaction analysis if planned: ‘**However, insufficient numbers of included studies precluded this analysis.’  **Reasons given for carrying out interaction analysis if they weren’t planned:** NA.  **Scenario in protocol:** Not reported.  **Rationale for planned scenario:** NA.  **Scenario in review (if reported specifically):** Not reported.  **Rationale for done scenario:** NA.  **Reason for changing mind:** NA. | **Method to detect interactions reported in protocol:** Not reported.  **Method to detect interactions reported in review:** Not reported.  **Reason for changing mind:** NA. |
| Lee 2013 | **Protocol:** Not reported.  **Review methods: ‘**Studies were reported according to the duration of post-intervention follow-up, as follows: ’immediate’ (within 24 hours) and ’long term’ (greater than 24 hours)’.  **Review results:** Not analysed.  **Type:** Methodological.  **Covariate summary:** Length of follow up. | **Method to choose**  **covariate:** Not reported.  **Where method reported:** NA.  **Rationale for choosing the covariate:** Not reported.  **Where rationale reported:** NA. | **Where reported:** Review only.  **Reason not reported in review:** NA.  **Reason not reported in protocol:** Not reported.  **Labelled as ‘posthoc’ if not in protocol:** No. | **Data type:** Continuous.  **Continuous covariate categorised:** Yes.  **How determine it was categorised:** Categorises reported.  **Justification for categorising:** Not reported.  **Where justification reported:** NA.  **Categories reported:** Yes.  **Where categories reported:** Review only.  **Justification for categories:** Not reported.  **Where justification reported:** NA. | **Analysis type reported in protocol:** Not reported.  **Analysis type reported in review:** Unnamed analysis.  **Reason for changing mind:** Not reported.    **Analysis type in protocol according to glossary:** Not reported.  **How determined the analysis type:** NA.  **Analysis type in review according to glossary:** Subgroup analysis.  **How determined the analysis type:** Categories given.  **Analysed:** No.  **Reasons given (in the review) for not doing interaction analysis if planned:** Not reported (presumably because one trial).  **Reasons given for carrying out interaction analysis if they weren’t planned:** NA.  **Scenario in protocol:** Not reported.  **Rationale for planned scenario:** NA.  **Scenario in review (if reported specifically):** Not reported.  **Rationale for done scenario:** NA.  **Reason for changing mind:** NA. | **Method to detect interactions reported in protocol:** Not reported.  **Method to detect interactions reported in review:** Not reported.  **Reason for changing mind:** NA. |
| Lee 2013 | **Protocol:** Use of intention to treat analysis. We judged the following to be three covariates: ‘A sensitivity analysis will be performed to analyse the effects of allocation concealment, assessor blinding and use of intention to treat analysis on results’.  **Review methods:** Use of intention to treat analysis. We judged the following to be three covariates: ‘We planned to perform a sensitivity analysis to analyse the effects of allocation concealment, assessor blinding and use of intention to treat analysis on results’.  **Review results:** Not analysed.  **Type:** Methodological.  **Covariate summary:** Intention to treat. | **Method to choose**  **covariate:** Not reported.  **Where method reported:** NA.  **Rationale for choosing the covariate:** Not reported.  **Where rationale reported:** NA. | **Where reported:** Protocol and review.  **Reason not reported in review:** NA.  **Reason not reported in protocol:** NA.  **Labelled as ‘posthoc’ if not in protocol:** NA. | **Data type:** Categorical.  **Continuous covariate categorised:** NA.  **How determine it was categorised:** NA.  **Justification for categorising:** NA.  **Where justification reported:** NA.  **Categories reported:** Not reported.  **Where categories reported:** NA.  **Justification for categories:** Not reported.  **Where justification reported:** NA. | **Analysis type reported in protocol:** Sensitivity analysis.  **Analysis type reported in review:** Sensitivity analysis.  **Reason for changing mind:** NA.    **Analysis type in protocol according to glossary:** Sensitivity analysis.  **How determined the analysis type: ‘**Sensitivity analysis’. No reason to dispute.  **Analysis type in review according to glossary:** Sensitivity analysis.  **How determined the analysis type:** ‘Sensitivity analysis’. No reason to dispute.  **Analysed:** No.  **Reasons given (in the review) for not doing interaction analysis if planned: ‘**However, insufficient numbers of included studies precluded this analysis.’  **Reasons given for carrying out interaction analysis if they weren’t planned:** NA.  **Scenario in protocol:** Not reported.  **Rationale for planned scenario:** NA.  **Scenario in review (if reported specifically):** Not reported.  **Rationale for done scenario:** NA.  **Reason for changing mind:** NA. | **Method to detect interactions reported in protocol:** Not reported.  **Method to detect interactions reported in review:** Not reported.  **Reason for changing mind:** NA. |
| Li 2013 | **Protocol:** ‘Age’.  **Review methods: ‘**Age’.  **Review results:** Not analysed.  **Type:** Patient.  **Covariate summary:** Demographics. | **Method to choose**  **covariate:** Not reported.  **Where method reported:** NA.  **Rationale for choosing the covariate:** Not reported.  **Where rationale reported:** NA. | **Where reported:** Protocol and review.  **Reason not reported in review:** NA.  **Reason not reported in protocol:** NA.  **Labelled as ‘posthoc’ if not in protocol:** NA. | **Data type:** Continuous.  **Continuous covariate categorised:** Yes.  **How determine it was categorised:** Subgroup analysis planned.  **Justification for categorising:** Not reported.  **Where justification reported:** NA.  **Categories reported:** Not reported.  **Where categories reported:** NA.  **Justification for categories:** Not reported.  **Where justification reported:** NA. | **Analysis type reported in protocol:** Subgroup analysis.  **Analysis type reported in review:** Subgroup analysis..  **Reason for changing mind:** NA.    **Analysis type in protocol according to glossary:** Subgroup analysis.  **How determined the analysis type:** Says ‘Subgroup analysis.’ No reason to dispute.  **Analysis type in review according to glossary:** Subgroup analysis.  **How determined the analysis type:** Says ‘Subgroup analysis.’ No reason to dispute.  **Analysed:** No.  **Reasons given (in the review) for not doing interaction analysis if planned:** Not reported (presumably because no meta-analysis).  **Reasons given for carrying out interaction analysis if they weren’t planned:** NA.  **Scenario in protocol:** ‘We will consider subgroup analysis depending on number and  characteristics of studies’.  **Rationale for planned scenario:** Not reported.  **Scenario in review (if reported specifically):** ‘In the  future, if we identify suitable studies, we will consider subgroup analysis depending on numbers and characteristics of studies.’  **Rationale for done scenario:** Not reported.  **Reason for changing mind:** NA. | **Method to detect interactions reported in protocol:** Not reported.  **Method to detect interactions reported in review:** Not reported.  **Reason for changing mind:** NA. |
| Li 2013 | **Protocol:** ‘Baseline symptom severity’.  **Review methods: ‘**Baseline symptom severity’.  **Review results:** Not analysed.  **Type:** Patient.  **Covariate summary:** Disease characteristics. | **Method to choose**  **covariate:** Not reported.  **Where method reported:** NA.  **Rationale for choosing the covariate:** Not reported.  **Where rationale reported:** NA. | **Where reported:** Protocol and review.  **Reason not reported in review:** NA.  **Reason not reported in protocol:** NA.  **Labelled as ‘posthoc’ if not in protocol:** NA. | **Data type:** Continuous.  **Continuous covariate categorised:** Yes.  **How determine it was categorised:** Subgroup analysis planned.  **Justification for categorising:** Not reported.  **Where justification reported:** NA.  **Categories reported:** Not reported.  **Where categories reported:** NA.  **Justification for categories:** Not reported.  **Where justification reported:** NA. | **Analysis type reported in protocol:** Subgroup analysis.  **Analysis type reported in review:** Subgroup analysis..  **Reason for changing mind:** NA.    **Analysis type in protocol according to glossary:** Subgroup analysis.  **How determined the analysis type:** Says ‘Subgroup analysis.’ No reason to dispute.  **Analysis type in review according to glossary:** Subgroup analysis.  **How determined the analysis type:** Says ‘Subgroup analysis.’ No reason to dispute.  **Analysed:** No.  **Reasons given (in the review) for not doing interaction analysis if planned:** Not reported (presumably because no meta-analysis).  **Reasons given for carrying out interaction analysis if they weren’t planned:** NA.  **Scenario in protocol:** ‘We will consider subgroup analysis depending on number and  characteristics of studies’.  **Rationale for planned scenario:** Not reported.  **Scenario in review (if reported specifically):** ‘In the  future, if we identify suitable studies, we will consider subgroup analysis depending on numbers and characteristics of studies.’  **Rationale for done scenario:** Not reported.  **Reason for changing mind:** NA. | **Method to detect interactions reported in protocol:** Not reported.  **Method to detect interactions reported in review:** Not reported.  **Reason for changing mind:** NA. |
| Li 2013 | **Protocol:** ‘Exclusion of studies in which outcome evaluation was not blinded’.  **Review methods: ‘**Exclusion of studies in which outcome evaluation was not blinded’.  **Review results:** Not analysed.  **Type:** Methodological.  **Covariate summary:** Blinding. | **Method to choose**  **covariate:** Not reported.  **Where method reported:** NA.  **Rationale for choosing the covariate:** Not reported.  **Where rationale reported:** NA. | **Where reported:** Protocol and review.  **Reason not reported in review:** NA.  **Reason not reported in protocol:** NA.  **Labelled as ‘posthoc’ if not in protocol:** NA. | **Data type:** Categorical.  **Continuous covariate categorised:** NA.  **How determine it was categorised:** NA.  **Justification for categorising:** NA.  **Where justification reported:** NA.  **Categories reported:** Yes.  **Where categories reported:** Protocol and review.  **Justification for categories:** Not reported.  **Where justification reported:** NA. | **Analysis type reported in protocol:** Sensitivity analysis.  **Analysis type reported in review:** Sensitivity analysis.  **Reason for changing mind:** NA.    **Analysis type in protocol according to glossary:** Sensitivity analysis.  **How determined the analysis type:** ‘Exclusion’.  **Analysis type in review according to glossary:** Sensitivity analysis.  **How determined the analysis type:** ‘Exclusion’.  **Analysed:** No.  **Reasons given (in the review) for not doing interaction analysis if planned:** Not reported (presumably because no meta-analysis).  **Reasons given for carrying out interaction analysis if they weren’t planned:** NA.  **Scenario in protocol:** ‘If a sufficient number of trials are found, we will carry out sensitivity  analysis to assess the robustness of the results as follows’.  **Rationale for planned scenario:** Not reported.  **Scenario in review (if reported specifically):** ‘If a sufficient number of trials are found in future updates, we will carry out a sensitivity  analysis to assess the robustness of the results as follows.’  **Rationale for done scenario:** Not reported.  **Reason for changing mind:** NA. | **Method to detect interactions reported in protocol:** Not reported.  **Method to detect interactions reported in review:** Not reported.  **Reason for changing mind:** NA. |
| Li 2013 | **Protocol:** ‘Exclusion of studies with inadequate concealment of allocation’.  **Review methods: ‘**Exclusion of studies with inadequate concealment of allocation’.  **Review results:** Not analysed.  **Type:** Methodological.  **Covariate summary:** Allocation concealment. | **Method to choose**  **covariate:** Not reported.  **Where method reported:** NA.  **Rationale for choosing the covariate:** Not reported.  **Where rationale reported:** NA. | **Where reported:** Protocol and review.  **Reason not reported in review:** NA.  **Reason not reported in protocol:** NA.  **Labelled as ‘posthoc’ if not in protocol:** NA. | **Data type:** Categorical.  **Continuous covariate categorised:** NA.  **How determine it was categorised:** NA.  **Justification for categorising:** NA.  **Where justification reported:** NA.  **Categories reported:** Yes.  **Where categories reported:** Protocol and review.  **Justification for categories:** Not reported.  **Where justification reported:** NA. | **Analysis type reported in protocol:** Sensitivity analysis.  **Analysis type reported in review:** Sensitivity analysis.  **Reason for changing mind:** NA.    **Analysis type in protocol according to glossary:** Sensitivity analysis.  **How determined the analysis type:** ‘Exclusion’.  **Analysis type in review according to glossary:** Sensitivity analysis.  **How determined the analysis type:** ‘Exclusion’.  **Analysed:** No.  **Reasons given (in the review) for not doing interaction analysis if planned:** Not reported (presumably because no meta-analysis).  **Reasons given for carrying out interaction analysis if they weren’t planned:** NA.  **Scenario in protocol:** ‘If a sufficient number of trials are found, we will carry out sensitivity  analysis to assess the robustness of the results as follows’.  **Rationale for planned scenario:** Not reported.  **Scenario in review (if reported specifically):** ‘If a sufficient number of trials are found in future updates, we will carry out a sensitivity  analysis to assess the robustness of the results as follows.’  **Rationale for done scenario:** Not reported.  **Reason for changing mind:** NA. | **Method to detect interactions reported in protocol:** Not reported.  **Method to detect interactions reported in review:** Not reported.  **Reason for changing mind:** NA. |
| Li 2013 | **Protocol:** ‘Gender’.  **Review methods: ‘**Gender’.  **Review results:** Not analysed.  **Type:** Patient.  **Covariate summary:** Demographics. | **Method to choose**  **covariate:** Not reported.  **Where method reported:** NA.  **Rationale for choosing the covariate:** Not reported.  **Where rationale reported:** NA. | **Where reported:** Protocol and review.  **Reason not reported in review:** NA.  **Reason not reported in protocol:** NA.  **Labelled as ‘posthoc’ if not in protocol:** NA. | **Data type:** Categorical.  **Continuous covariate categorised:** NA.  **How determine it was categorised:** NA.  **Justification for categorising:** NA.  **Where justification reported:** NA.  **Categories reported:** Not reported.  **Where categories reported:** NA.  **Justification for categories:** Not reported.  **Where justification reported:** NA. | **Analysis type reported in protocol:** Subgroup analysis.  **Analysis type reported in review:** Subgroup analysis..  **Reason for changing mind:** NA.    **Analysis type in protocol according to glossary:** Subgroup analysis.  **How determined the analysis type:** Says ‘Subgroup analysis.’ No reason to dispute.  **Analysis type in review according to glossary:** Subgroup analysis.  **How determined the analysis type:** Says ‘Subgroup analysis.’ No reason to dispute.  **Analysed:** No.  **Reasons given (in the review) for not doing interaction analysis if planned:** Not reported (presumably because no meta-analysis).  **Reasons given for carrying out interaction analysis if they weren’t planned:** NA.  **Scenario in protocol:** ‘We will consider subgroup analysis depending on number and  characteristics of studies’.  **Rationale for planned scenario:** Not reported.  **Scenario in review (if reported specifically):** ‘In the  future, if we identify suitable studies, we will consider subgroup analysis depending on numbers and characteristics of studies.’  **Rationale for done scenario:** Not reported.  **Reason for changing mind:** NA. | **Method to detect interactions reported in protocol:** Not reported.  **Method to detect interactions reported in review:** Not reported.  **Reason for changing mind:** NA. |
| Li 2013 | **Protocol:** ‘Time from symptom onset to enrolment in the study’.  **Review methods: ‘**Time from symptom onset to enrolment in the study’.  **Review results:** Not analysed.  **Type:** Patient.  **Covariate summary:** Disease characteristics. | **Method to choose**  **covariate:** Not reported.  **Where method reported:** NA.  **Rationale for choosing the covariate:** Not reported.  **Where rationale reported:** NA. | **Where reported:** Protocol and review.  **Reason not reported in review:** NA.  **Reason not reported in protocol:** NA.  **Labelled as ‘posthoc’ if not in protocol:** NA. | **Data type:** Continuous.  **Continuous covariate categorised:** Yes.  **How determine it was categorised:** Subgroup analysis is planned.  **Justification for categorising:** Not reported.  **Where justification reported:** NA.  **Categories reported:** Not reported.  **Where categories reported:** NA.  **Justification for categories:** Not reported.  **Where justification reported:** NA. | **Analysis type reported in protocol:** Subgroup analysis.  **Analysis type reported in review:** Subgroup analysis..  **Reason for changing mind:** NA.    **Analysis type in protocol according to glossary:** Subgroup analysis.  **How determined the analysis type:** Says ‘Subgroup analysis.’ No reason to dispute.  **Analysis type in review according to glossary:** Subgroup analysis.  **How determined the analysis type:** Says ‘Subgroup analysis.’ No reason to dispute.  **Analysed:** No.  **Reasons given (in the review) for not doing interaction analysis if planned:** Not reported (presumably because no meta-analysis).  **Reasons given for carrying out interaction analysis if they weren’t planned:** NA.  **Scenario in protocol:** ‘We will consider subgroup analysis depending on number and  characteristics of studies’.  **Rationale for planned scenario:** Not reported.  **Scenario in review (if reported specifically):** ‘In the  future, if we identify suitable studies, we will consider subgroup analysis depending on numbers and characteristics of studies.’  **Rationale for done scenario:** Not reported.  **Reason for changing mind:** NA. | **Method to detect interactions reported in protocol:** Not reported.  **Method to detect interactions reported in review:** Not reported.  **Reason for changing mind:** NA. |
| Liu 2013 | **Protocol:** ‘Chinese herbal medicines includes medicines from mixtures of herbs, single herbs, Chinese proprietary medicines, or a compound of herbs that is prescribed by a Chinese medicine practitioner. We will group each category separately when conducting comparative analyses.’  **‘**formulation of herbs (extract, single herb, or mixture of herbs).’  **Review methods:** ‘Chinese herbal medicines (including medicines from mixtures of herbs, single herbs, Chinese proprietary medicines, or a formula of herbs prescribed by a Chinese medicine practitioner; we planned to group each category separately when conducting comparative analyses.’ **‘**formulation of herbs (extract, single herb, or mixture of herbs).’  **Review results:** Not analysed.  **Type:** Intervention.  **Covariate summary:** Type of intervention or control. | **Method to choose**  **covariate:** Not reported.  **Where method reported:** NA.  **Rationale for choosing the covariate:** Not reported.  **Where rationale reported:** NA. | **Where reported:** Protocol and review.  **Reason not reported in review:** NA.  **Reason not reported in protocol:** NA.  **Labelled as ‘posthoc’ if not in protocol:** NA. | **Data type:** Categorical.  **Continuous covariate categorised:** NA.  **How determine it was categorised:** NA.  **Justification for categorising:** NA.  **Where justification reported:** NA.  **Categories reported:** Yes.  **Where categories reported:** Protocol and review.  **Justification for categories:** Not reported.  **Where justification reported:** NA. | **Analysis type reported in protocol:** Subgroup analysis.  **Analysis type reported in review:** Subgroup analysis.  **Reason for changing mind:** NA.    **Analysis type in protocol according to glossary:** Subgroup analysis.  **How determined the analysis type:** Categories given.  **Analysis type in review according to glossary:** Subgroup analysis.  **How determined the analysis type:** Categories given.  **Analysed:** No.  **Reasons given (in the review) for not doing interaction analysis if planned:** Not reported (presumably because no trials).  **Reasons given for carrying out interaction analysis if they weren’t planned:** NA.  **Scenario in protocol: ‘**We will mainly carry out subgroup analyses of the primary outcome  parameter(s) (see above) and investigate interaction. If a sufficient number of randomized trials are identified, we will perform subgroup analyse’  **Rationale for planned scenario:** Not reported.  **Scenario in review (if reported specifically): ‘**We planned to carry out subgroup analyses of the primary outcome parameter(s) (see above) and investigate interaction. If a sufficient number of randomised trials were identified, we would have performed subgroup analyses’  **Rationale for done scenario:** Not reported.  **Reason for changing mind:** NA. | **Method to detect interactions reported in protocol:** Not reported.  **Method to detect interactions reported in review:** Not reported.  **Reason for changing mind:** NA. |
| Liu 2013 | **Protocol:** ‘Restricting the analysis taking account risk of bias’.  **Review methods: ‘**Restricting the analysis taking account risk of bias’.  **Review results:** Not analysed.  **Type:** Methodological.  **Covariate summary:** Risk of bias. | **Method to choose**  **covariate:** Not reported.  **Where method reported:** NA.  **Rationale for choosing the covariate:** Not reported.  **Where rationale reported:** NA. | **Where reported:** Protocol and review.  **Reason not reported in review:** NA.  **Reason not reported in protocol:** NA.  **Labelled as ‘posthoc’ if not in protocol:** NA. | **Data type:** Categorical.  **Continuous covariate categorised:** NA.  **How determine it was categorised:** NA.  **Justification for categorising:** NA.  **Where justification reported:** NA.  **Categories reported:** Not reported.  **Where categories reported:** NA.  **Justification for categories:** Not reported.  **Where justification reported:** NA. | **Analysis type reported in protocol:** Sensitivity analysis.  **Analysis type reported in review:** Sensitivity analysis.  **Reason for changing mind:** NA.    **Analysis type in protocol according to glossary:** Sensitivity analysis.  **How determined the analysis type:** Categories given.  **Analysis type in review according to glossary:** Sensitivity analysis.  **How determined the analysis type:** Categories given.  **Analysed:** No.  **Reasons given (in the review) for not doing interaction analysis if planned:** Not reported (presumably because no trials).  **Reasons given for carrying out interaction analysis if they weren’t planned:** NA.  **Scenario in protocol:** Not reported.  **Rationale for planned scenario:** NA.  **Scenario in review (if reported specifically):** Not reported.  **Rationale for done scenario:** NA.  **Reason for changing mind:** NA. | **Method to detect interactions reported in protocol:** Not reported.  **Method to detect interactions reported in review:** Not reported.  **Reason for changing mind:** NA. |
| Liu 2013 | **Protocol:** ‘Timing of outcome measurement. The minimum treatment duration will be four weeks. We will explore short-term (less than six months) and long-term (more than six months) duration in a subgroup analysis.’ ‘Treatment duration (no more than six months and more than six months).’  **Review methods:** ‘Timing of outcome measurement. The minimum treatment duration will be four weeks. We wanted to explore short-term (less than six months) and long-term (more than six months) duration in a subgroup analysis.’ ‘Treatment duration (no more than six months and more than six months).’  **Review results:** Not analysed.  **Type:** Outcome.  **Covariate summary:** Time point. | **Method to choose**  **covariate:** Not reported.  **Where method reported:** NA.  **Rationale for choosing the covariate:** Not reported.  **Where rationale reported:** NA. | **Where reported:** Protocol and review.  **Reason not reported in review:** NA.  **Reason not reported in protocol:** NA.  **Labelled as ‘posthoc’ if not in protocol:** NA. | **Data type:** Continuous.  **Continuous covariate categorised:** Yes.  **How determine it was categorised:** Categories given.  **Justification for categorising:** No reported.  **Where justification reported:** NA.  **Categories reported:** Yes.  **Where categories reported:** Protocol and review.  **Justification for categories:** Not reported.  **Where justification reported:** NA. | **Analysis type reported in protocol:** Subgroup analysis.  **Analysis type reported in review:** Subgroup analysis.  **Reason for changing mind:** NA.    **Analysis type in protocol according to glossary:** Subgroup analysis.  **How determined the analysis type:** Categories given.  **Analysis type in review according to glossary:** Subgroup analysis.  **How determined the analysis type:** Categories given.  **Analysed:** No.  **Reasons given (in the review) for not doing interaction analysis if planned:** Not reported (presumably because no trials).  **Reasons given for carrying out interaction analysis if they weren’t planned:** NA.  **Scenario in protocol: ‘**We will mainly carry out subgroup analyses of the primary outcome  parameter(s) (see above) and investigate interaction. If a sufficient number of randomized trials are identified, we will perform subgroup analyse’  **Rationale for planned scenario:** Not reported.  **Scenario in review (if reported specifically): ‘**We planned to carry out subgroup analyses of the primary outcome parameter(s) (see above) and investigate interaction. If a sufficient number of randomised trials were identified, we would have performed subgroup analyses’  **Rationale for done scenario:** Not reported.  **Reason for changing mind:** NA. | **Method to detect interactions reported in protocol:** Not reported.  **Method to detect interactions reported in review:** Not reported.  **Reason for changing mind:** NA. |
| Liu 2013 | **Protocol:** Country. We judged the following to be five covariates: ‘restricting the analysis to studies using the following filters: diagnostic criteria, language of publication, source of funding (industry versus other), country, trials with significant skewed distributions of participants in groups (Pocock 1990).’  **Review methods:** Country. We judged the following to be five covariates: ‘restricting the analysis to studies using the following filters: diagnostic criteria, language of publication, source of funding (industry versus other), country, trials with significant skewed distributions of participants in groups (Pocock 1990).’  **Review results:** Not analysed.  **Type:** Patient.  **Covariate summary:** Country. | **Method to choose**  **covariate:** Not reported.  **Where method reported:** NA.  **Rationale for choosing the covariate:** Not reported.  **Where rationale reported:** NA. | **Where reported:** Protocol and review.  **Reason not reported in review:** NA.  **Reason not reported in protocol:** NA.  **Labelled as ‘posthoc’ if not in protocol:** NA. | **Data type:** Categorical.  **Continuous covariate categorised:** NA.  **How determine it was categorised:** NA.  **Justification for categorising:** NA.  **Where justification reported:** NA.  **Categories reported:** Not reported.  **Where categories reported:** NA.  **Justification for categories:** Not reported.  **Where justification reported:** NA. | **Analysis type reported in protocol:** Sensitivity analysis.  **Analysis type reported in review:** Sensitivity analysis.  **Reason for changing mind:** NA.    **Analysis type in protocol according to glossary:** Sensitivity analysis.  **How determined the analysis type:** Categories given.  **Analysis type in review according to glossary:** Sensitivity analysis.  **How determined the analysis type:** Categories given.  **Analysed:** No.  **Reasons given (in the review) for not doing interaction analysis if planned:** Not reported (presumably because no trials).  **Reasons given for carrying out interaction analysis if they weren’t planned:** NA.  **Scenario in protocol:** Not reported.  **Rationale for planned scenario:** NA.  **Scenario in review (if reported specifically):** Not reported.  **Rationale for done scenario:** NA.  **Reason for changing mind:** NA. | **Method to detect interactions reported in protocol:** Not reported.  **Method to detect interactions reported in review:** Not reported.  **Reason for changing mind:** NA. |
| Liu 2013 | **Protocol:** Diagnostic criteria. We judged the following to be five covariates: ‘restricting the analysis to studies using the following filters: diagnostic criteria, language of publication, source of funding (industry versus other), country, trials with significant skewed distributions of participants in groups (Pocock 1990).’  **Review methods:** Diagnostic criteria. We judged the following to be five covariates: ‘restricting the analysis to studies using the following filters: diagnostic criteria, language of publication, source of funding (industry versus other), country, trials with significant skewed distributions of participants in groups (Pocock 1990).’  **Review results:** Not analysed.  **Type:** Patient.  **Covariate summary:** Diagnostic criteria. | **Method to choose**  **covariate:** Not reported.  **Where method reported:** NA.  **Rationale for choosing the covariate:** Not reported.  **Where rationale reported:** NA. | **Where reported:** Protocol and review.  **Reason not reported in review:** NA.  **Reason not reported in protocol:** NA.  **Labelled as ‘posthoc’ if not in protocol:** NA. | **Data type:** Categorical.  **Continuous covariate categorised:** NA.  **How determine it was categorised:** NA.  **Justification for categorising:** NA.  **Where justification reported:** NA.  **Categories reported:** Not reported.  **Where categories reported:** NA.  **Justification for categories:** Not reported.  **Where justification reported:** NA. | **Analysis type reported in protocol:** Sensitivity analysis.  **Analysis type reported in review:** Sensitivity analysis.  **Reason for changing mind:** NA.    **Analysis type in protocol according to glossary:** Sensitivity analysis.  **How determined the analysis type:** Categories given.  **Analysis type in review according to glossary:** Sensitivity analysis.  **How determined the analysis type:** Categories given.  **Analysed:** No.  **Reasons given (in the review) for not doing interaction analysis if planned:** Not reported (presumably because no trials).  **Reasons given for carrying out interaction analysis if they weren’t planned:** NA.  **Scenario in protocol:** Not reported.  **Rationale for planned scenario:** NA.  **Scenario in review (if reported specifically):** Not reported.  **Rationale for done scenario:** NA.  **Reason for changing mind:** NA. | **Method to detect interactions reported in protocol:** Not reported.  **Method to detect interactions reported in review:** Not reported.  **Reason for changing mind:** NA. |
| Liu 2013 | **Protocol:** Language of publication. We judged the following to be five covariates: ‘restricting the analysis to studies using the following filters: diagnostic criteria, language of publication, source of funding (industry versus other), country, trials with significant skewed distributions of participants in groups (Pocock 1990).’  **Review methods:** Language of publication. We judged the following to be five covariates: ‘restricting the analysis to studies using the following filters: diagnostic criteria, language of publication, source of funding (industry versus other), country, trials with significant skewed distributions of participants in groups (Pocock 1990).’  **Review results:** Not analysed.  **Type:** Methodological.  **Covariate summary:** Publication language. | **Method to choose**  **covariate:** Not reported.  **Where method reported:** NA.  **Rationale for choosing the covariate:** Not reported.  **Where rationale reported:** NA. | **Where reported:** Protocol and review.  **Reason not reported in review:** NA.  **Reason not reported in protocol:** NA.  **Labelled as ‘posthoc’ if not in protocol:** NA. | **Data type:** Categorical.  **Continuous covariate categorised:** NA.  **How determine it was categorised:** NA.  **Justification for categorising:** NA.  **Where justification reported:** NA.  **Categories reported:** Not reported.  **Where categories reported:** NA.  **Justification for categories:** Not reported.  **Where justification reported:** NA. | **Analysis type reported in protocol:** Sensitivity analysis.  **Analysis type reported in review:** Sensitivity analysis.  **Reason for changing mind:** NA.    **Analysis type in protocol according to glossary:** Sensitivity analysis.  **How determined the analysis type:** Categories given.  **Analysis type in review according to glossary:** Sensitivity analysis.  **How determined the analysis type:** Categories given.  **Analysed:** No.  **Reasons given (in the review) for not doing interaction analysis if planned:** Not reported (presumably because no trials).  **Reasons given for carrying out interaction analysis if they weren’t planned:** NA.  **Scenario in protocol:** Not reported.  **Rationale for planned scenario:** NA.  **Scenario in review (if reported specifically):** Not reported.  **Rationale for done scenario:** NA.  **Reason for changing mind:** NA. | **Method to detect interactions reported in protocol:** Not reported.  **Method to detect interactions reported in review:** Not reported.  **Reason for changing mind:** NA. |
| Liu 2013 | **Protocol:** Restricting the analysis to published studies’.  **Review methods:** Restricting the analysis to published studies’.  **Review results:** Not analysed.  **Type:** Methodological.  **Covariate summary:** Publication status. | **Method to choose**  **covariate:** Not reported.  **Where method reported:** NA.  **Rationale for choosing the covariate:** Not reported.  **Where rationale reported:** NA. | **Where reported:** Protocol and review.  **Reason not reported in review:** NA.  **Reason not reported in protocol:** NA.  **Labelled as ‘posthoc’ if not in protocol:** NA. | **Data type:** Categorical.  **Continuous covariate categorised:** NA.  **How determine it was categorised:** NA.  **Justification for categorising:** NA.  **Where justification reported:** NA.  **Categories reported:** Yes.  **Where categories reported:** Protocol and review.  **Justification for categories:** Not reported.  **Where justification reported:** NA. | **Analysis type reported in protocol:** Sensitivity analysis.  **Analysis type reported in review:** Sensitivity analysis.  **Reason for changing mind:** NA.    **Analysis type in protocol according to glossary:** Sensitivity analysis.  **How determined the analysis type:** Categories given.  **Analysis type in review according to glossary:** Sensitivity analysis.  **How determined the analysis type:** Categories given.  **Analysed:** No.  **Reasons given (in the review) for not doing interaction analysis if planned:** Not reported (presumably because no trials).  **Reasons given for carrying out interaction analysis if they weren’t planned:** NA.  **Scenario in protocol:** Not reported.  **Rationale for planned scenario:** NA.  **Scenario in review (if reported specifically):** Not reported.  **Rationale for done scenario:** NA.  **Reason for changing mind:** NA. | **Method to detect interactions reported in protocol:** Not reported.  **Method to detect interactions reported in review:** Not reported.  **Reason for changing mind:** NA. |
| Liu 2013 | **Protocol:** Restricting the analysis to very large studies. We judged the following to be two covariates: ‘restricting the analysis to very long or large studies to establish how much they dominate the results.’  **Review methods:** Restricting the analysis to very large studies. We judged the following to be two covariates: ‘restricting the analysis to very long or large studies to establish how much they dominate the results.’  **Review results:** Not analysed.  **Type:** Methodological.  **Covariate summary:** Trial size. | **Method to choose**  **covariate:** Not reported.  **Where method reported:** NA.  **Rationale for choosing the covariate:** Not reported.  **Where rationale reported:** NA. | **Where reported:** Protocol and review.  **Reason not reported in review:** NA.  **Reason not reported in protocol:** NA.  **Labelled as ‘posthoc’ if not in protocol:** NA. | **Data type:** Continuous.  **Continuous covariate categorised:** Yes.  **How determine it was categorised:** Categories given.  **Justification for categorising:** No reported.  **Where justification reported:** NA.  **Categories reported:** Yes.  **Where categories reported:** Protocol and review.  **Justification for categories:** Not reported.  **Where justification reported:** NA. | **Analysis type reported in protocol:** Sensitivity analysis.  **Analysis type reported in review:** Sensitivity analysis.  **Reason for changing mind:** NA.    **Analysis type in protocol according to glossary:** Sensitivity analysis.  **How determined the analysis type:** Categories given.  **Analysis type in review according to glossary:** Sensitivity analysis.  **How determined the analysis type:** Categories given.  **Analysed:** No.  **Reasons given (in the review) for not doing interaction analysis if planned:** Not reported (presumably because no trials).  **Reasons given for carrying out interaction analysis if they weren’t planned:** NA.  **Scenario in protocol:** Not reported.  **Rationale for planned scenario:** NA.  **Scenario in review (if reported specifically):** Not reported.  **Rationale for done scenario:** NA.  **Reason for changing mind:** NA. | **Method to detect interactions reported in protocol:** Not reported.  **Method to detect interactions reported in review:** Not reported.  **Reason for changing mind:** NA. |
| Liu 2013 | **Protocol:** Restricting the analysis to very long studies. We judged the following to be two covariates: ‘restricting the analysis to very long or large studies to establish how much they dominate the results.’  **Review methods:** Restricting the analysis to very long studies. We judged the following to be two covariates: ‘restricting the analysis to very long or large studies to establish how much they dominate the results.’  **Review results:** Not analysed.  **Type:** Methodological.  **Covariate summary:** Length of study. | **Method to choose**  **covariate:** Not reported.  **Where method reported:** NA.  **Rationale for choosing the covariate:** Not reported.  **Where rationale reported:** NA. | **Where reported:** Protocol and review.  **Reason not reported in review:** NA.  **Reason not reported in protocol:** NA.  **Labelled as ‘posthoc’ if not in protocol:** NA. | **Data type:** Continuous.  **Continuous covariate categorised:** Yes.  **How determine it was categorised:** Categories given.  **Justification for categorising:** No reported.  **Where justification reported:** NA.  **Categories reported:** Yes.  **Where categories reported:** Protocol and review.  **Justification for categories:** Not reported.  **Where justification reported:** NA. | **Analysis type reported in protocol:** Sensitivity analysis.  **Analysis type reported in review:** Sensitivity analysis.  **Reason for changing mind:** NA.    **Analysis type in protocol according to glossary:** Sensitivity analysis.  **How determined the analysis type:** Categories given.  **Analysis type in review according to glossary:** Sensitivity analysis.  **How determined the analysis type:** Categories given.  **Analysed:** No.  **Reasons given (in the review) for not doing interaction analysis if planned:** Not reported (presumably because no trials).  **Reasons given for carrying out interaction analysis if they weren’t planned:** NA.  **Scenario in protocol:** Not reported.  **Rationale for planned scenario:** NA.  **Scenario in review (if reported specifically):** Not reported.  **Rationale for done scenario:** NA.  **Reason for changing mind:** NA. | **Method to detect interactions reported in protocol:** Not reported.  **Method to detect interactions reported in review:** Not reported.  **Reason for changing mind:** NA. |
| Liu 2013 | **Protocol:** Source of funding (industry versus other). We judged the following to be five covariates: ‘restricting the analysis to studies using the following filters: diagnostic criteria, language of publication, source of funding (industry versus other), country, trials with significant skewed distributions of participants in groups (Pocock 1990).’  **Review methods:** Source of funding (industry versus other). We judged the following to be five covariates: ‘restricting the analysis to studies using the following filters: diagnostic criteria, language of publication, source of funding (industry versus other), country, trials with significant skewed distributions of participants in groups (Pocock 1990).’  **Review results:** Not analysed.  **Type:** Methodological.  **Covariate summary:** Funding. | **Method to choose**  **covariate:** Not reported.  **Where method reported:** NA.  **Rationale for choosing the covariate:** Not reported.  **Where rationale reported:** NA. | **Where reported:** Protocol and review.  **Reason not reported in review:** NA.  **Reason not reported in protocol:** NA.  **Labelled as ‘posthoc’ if not in protocol:** NA. | **Data type:** Categorical.  **Continuous covariate categorised:** NA.  **How determine it was categorised:** NA.  **Justification for categorising:** NA.  **Where justification reported:** NA.  **Categories reported:** Yes.  **Where categories reported:** Protocol and review.  **Justification for categories:** Not reported.  **Where justification reported:** NA. | **Analysis type reported in protocol:** Sensitivity analysis.  **Analysis type reported in review:** Sensitivity analysis.  **Reason for changing mind:** NA.    **Analysis type in protocol according to glossary:** Sensitivity analysis.  **How determined the analysis type:** Categories given.  **Analysis type in review according to glossary:** Sensitivity analysis.  **How determined the analysis type:** Categories given.  **Analysed:** No.  **Reasons given (in the review) for not doing interaction analysis if planned:** Not reported (presumably because no trials).  **Reasons given for carrying out interaction analysis if they weren’t planned:** NA.  **Scenario in protocol:** Not reported.  **Rationale for planned scenario:** NA.  **Scenario in review (if reported specifically):** Not reported.  **Rationale for done scenario:** NA.  **Reason for changing mind:** NA. | **Method to detect interactions reported in protocol:** Not reported.  **Method to detect interactions reported in review:** Not reported.  **Reason for changing mind:** NA. |
| Liu 2013 | **Protocol:** Trials with significant skewed distributions of participants in groups. We judged the following to be five covariates: ‘restricting the analysis to studies using the following filters: diagnostic criteria, language of publication, source of funding (industry versus other), country, trials with significant skewed distributions of participants in groups (Pocock 1990).’  **Review methods:** Trials with significant skewed distributions of participants in groups. We judged the following to be five covariates: ‘restricting the analysis to studies using the following filters: diagnostic criteria, language of publication, source of funding (industry versus other), country, trials with significant skewed distributions of participants in groups (Pocock 1990).’  **Review results:** Not analysed.  **Type:** Outcome.  **Covariate summary:** Skewed data. | **Method to choose**  **covariate:** Not reported.  **Where method reported:** NA.  **Rationale for choosing the covariate:** Not reported.  **Where rationale reported:** NA. | **Where reported:** Protocol and review.  **Reason not reported in review:** NA.  **Reason not reported in protocol:** NA.  **Labelled as ‘posthoc’ if not in protocol:** NA. | **Data type:** Categorical.  **Continuous covariate categorised:** NA.  **How determine it was categorised:** NA.  **Justification for categorising:** NA.  **Where justification reported:** NA.  **Categories reported:** Yes.  **Where categories reported:** Protocol and review.  **Justification for categories:** Not reported.  **Where justification reported:** NA. | **Analysis type reported in protocol:** Sensitivity analysis.  **Analysis type reported in review:** Sensitivity analysis.  **Reason for changing mind:** NA.    **Analysis type in protocol according to glossary:** Sensitivity analysis.  **How determined the analysis type:** Categories given.  **Analysis type in review according to glossary:** Sensitivity analysis.  **How determined the analysis type:** Categories given.  **Analysed:** No.  **Reasons given (in the review) for not doing interaction analysis if planned:** Not reported (presumably because no trials).  **Reasons given for carrying out interaction analysis if they weren’t planned:** NA.  **Scenario in protocol:** Not reported.  **Rationale for planned scenario:** NA.  **Scenario in review (if reported specifically):** Not reported.  **Rationale for done scenario:** NA.  **Reason for changing mind:** NA. | **Method to detect interactions reported in protocol:** Not reported.  **Method to detect interactions reported in review:** Not reported.  **Reason for changing mind:** NA. |
| Lopez 2013 | **Protocol:** Not reported.  **Review methods:** Not reported.  **Review results:** Time point. ‘Use of contraceptives (non-condom) per visit’; ‘New use of modern contraceptives (excluding condoms)’; ‘New use of condoms’; ‘Reported new use of contraceptives per 100 women-years’; ‘Use of modern contraceptive method at 12 to 14 months’; ‘Reported differences in use of modern contraceptives (baseline to follow-up)’; ‘Use of hormonal contraceptives at 1 year (Oral contraceptives)’; ‘Use of hormonal contraceptives at 1 year (Injectable or oral contraceptives)’; ‘Discontinued chosen hormonal contraceptive by 1 year’; ‘Use of condoms or spermicide at 1 year (Reported use of condoms at 1 year)’; ‘Use of condoms or spermicide at 1 year (Recorded using condoms at 1 year)’; ‘Use of condoms or spermicide at 1 year (Recorded using spermicide at 1 year)’; ‘Use of hormonal contraceptives after intervention (pill)’; ‘Use of hormonal contraceptives after intervention (Injected )’; ‘Use of hormonal contraceptives after intervention (Implant)’; ‘Use of modern contraceptive (injectable, IUD, pills, condoms) Use at month 6’; ‘Use of modern contraceptive (injectable, IUD, pills, condoms) Use at month 12’; ‘Use of modern contraceptive (injectable, IUD, pills, condoms) Use at month 24’; ‘Use of modern contraceptives (Reported hazard ratios from multivariate Cox models)’; ‘Reported percentages for consistent condom use (at 3 months, 6 months, 18 months)’.  **Type:** Outcome.  **Covariate summary:** Time point. | **Method to choose**  **covariate:** Not reported.  **Where method reported:** NA.  **Rationale for choosing the covariate:** Not reported.  **Where rationale reported:** NA. | **Where reported:** Review only.  **Reason not reported in review:** NA.  **Reason not reported in protocol:** Not reported.  **Labelled as ‘posthoc’ if not in protocol:** No**.** | **Data type:** Continuous.  **Continuous covariate categorised:** Yes.  **How determine it was categorised:** Categories given.  **Justification for categorising:** Not reported.  **Where justification reported:** NA.  **Categories reported:** Yes.  **Where categories reported:** Review only.  **Justification for categories:** Not reported.  **Where justification reported:** NA. | **Analysis type reported in protocol:** Not reported.  **Analysis type reported in review:** Unnamed analysis.  **Reason for changing mind:** Not reported.    **Analysis type in protocol according to glossary:** Not reported.  **How determined the analysis type:** NA.  **Analysis type in review according to glossary:** Stratification/subgroup analysis.  **How determined the analysis type:** Categories given.  **Analysed:** Yes.  **Reasons given (in the review) for not doing interaction analysis if planned:** NA.  **Reasons given for carrying out interaction analysis if they weren’t planned:** Not reported.  **Scenario in protocol:** Not reported.  **Rationale for planned scenario:** NA.  **Scenario in review (if reported specifically):** Not reported.  **Rationale for done scenario:** NA.  **Reason for changing mind:** NA**.** | **Method to detect interactions reported in protocol:** Not reported.  **Method to detect interactions reported in review:** Not reported.  **Reason for changing mind:** NA. |
| Lopez 2013 | **Protocol:** Not reported.  **Review methods:** Not reported.  **Review results: ‘**Comparison. Family planning intervention versus usual care’; ‘Integrated family planning versus routine care’; ‘Integrated family planning services: enhanced versus basic’; ‘Family planning counseling (HIV+ women) versus comparison (HIV- women)’; ‘Informational video + discussion: HIV+ versus HIV- women’; ‘Family planning counseling and free contraceptives: HIV+ versus HIV- women’ (in plot). ‘Integrated family planning services versus usual services or basic integration’; ‘HIV-positive versus HIV-negative women’.  **Type:** Intervention.  **Covariate summary:** Type of intervention or control. | **Method to choose**  **covariate:** Not reported.  **Where method reported:** NA.  **Rationale for choosing the covariate:** Not reported.  **Where rationale reported:** NA. | **Where reported:** Review only.  **Reason not reported in review:** NA.  **Reason not reported in protocol:** Not reported.  **Labelled as ‘posthoc’ if not in protocol:** No**.** | **Data type:** Categorical.  **Continuous covariate categorised:** NA.  **How determine it was categorised:** NA.  **Justification for categorising:** NA.  **Where justification reported:** NA.  **Categories reported:** Yes.  **Where categories reported:** Review only.  **Justification for categories:** Not reported.  **Where justification reported:** NA. | **Analysis type reported in protocol:** Not reported.  **Analysis type reported in review:** Unnamed analysis.  **Reason for changing mind:** Not reported.    **Analysis type in protocol according to glossary:** Not reported.  **How determined the analysis type:** NA.  **Analysis type in review according to glossary:** Stratification/subgroup analysis.  **How determined the analysis type:** Categories given.  **Analysed:** Yes.  **Reasons given (in the review) for not doing interaction analysis if planned:** NA.  **Reasons given for carrying out interaction analysis if they weren’t planned:** Not reported.  **Scenario in protocol:** Not reported.  **Rationale for planned scenario:** NA.  **Scenario in review (if reported specifically):** Not reported.  **Rationale for done scenario:** NA.  **Reason for changing mind:** NA**.** | **Method to detect interactions reported in protocol:** Not reported.  **Method to detect interactions reported in review:** Not reported.  **Reason for changing mind:** NA. |
| Marigold 2013 | **Protocol:** ‘Children or adults with clinically overt generalised or focal seizures will be included, regardless of whether EEG monitoring was used to confirm the diagnosis. For studies that included patients with non-convulsive seizures diagnosed only by EEG (Bearden 2008), the results for these patients will be extracted and analysed separately.’  **Review methods:** ‘Children or adults with clinically overt generalised or focal seizures were included, regardless of whether EEG monitoring was used to confirm the diagnosis. For studies that included patients with non-convulsive seizures diagnosed only by EEG (Bearden 2008), the results for these patients were extracted and analysed separately.’  **Review results:** Not analysed.  **Type:** Patient.  **Covariate summary:** Disease characteristics. | **Method to choose**  **covariate:** Not reported.  **Where method reported:** NA.  **Rationale for choosing the covariate:** Not reported.  **Where rationale reported:** NA. | **Where reported:** Protocol and review.  **Reason not reported in review:** NA.  **Reason not reported in protocol:** NA.  **Labelled as ‘posthoc’ if not in protocol:** NA. | **Data type:** Categorical.  **Continuous covariate categorised:** NA.  **How determine it was categorised:** NA.  **Justification for categorising:** NA.  **Where justification reported:** NA.  **Categories reported:** Yes.  **Where categories reported:** Protocol and review.  **Justification for categories:** Not reported.  **Where justification reported:** NA. | **Analysis type reported in protocol:** Unnamed analysis.  **Analysis type reported in review:** Unnamed analysis.  **Reason for changing mind:** NA.  **Analysis type in protocol according to glossary:** Subgroup analysis.  **How determined the analysis type:** Categories given.  **Analysis type in review according to glossary:** Subgroup analysis.  **How determined the analysis type:** Categories given.  **Analysed:** No.  **Reasons given (in the review) for not doing interaction analysis if planned:** Not reported (presumably because no trials).  **Reasons given for carrying out interaction analysis if they weren’t planned:** NA.  **Scenario in protocol:** None reported.  **Rationale for planned scenario:** NA.  **Scenario in review (if reported specifically):** None reported.  **Rationale for done scenario:** NA.  **Reason for changing mind:** NA. | **Method to detect interactions reported in protocol:** Not reported.  **Method to detect interactions reported in review:** Not reported.  **Reason for changing mind:** NA. |
| Marigold 2013 | **Protocol:** ‘Ideally, patients with a history of epilepsy should be excluded. In cases where studies have recruited a mixture of patients with and without a history of epilepsy, attempts will be made to extract only the results related to those patients without a history of epilepsy. If this is not possible, despite contacting the investigators, a sensitivity analysis will be conducted to determine the effects of including and excluding these studies.’  **Review methods:** Not reported.  **Review results:** Not analysed.  **Type:** Patient.  **Covariate summary:** Disease characteristics. | **Method to choose**  **covariate:** Not reported.  **Where method reported:** NA.  **Rationale for choosing the covariate:** Not reported.  **Where rationale reported:** NA. | **Where reported:** Protocol only.  **Reason not reported in review:** Not reported.  **Reason not reported in protocol:** NA.  **Labelled as ‘posthoc’ if not in protocol:** NA. | **Data type:** Categorical.  **Continuous covariate categorised:** NA.  **How determine it was categorised:** NA.  **Justification for categorising:** NA.  **Where justification reported:** NA.  **Categories reported:** Yes.  **Where categories reported:** Protocol only.  **Justification for categories:** Not reported.  **Where justification reported:** NA. | **Analysis type reported in protocol:** Sensitivity analysis.  **Analysis type reported in review:** Not reported.  **Reason for changing mind:** Not reported.  **Analysis type in protocol according to glossary:** Sensitivity analysis.  **How determined the analysis type:** ‘including and excluding’.  **Analysis type in review according to glossary:** Not reported.  **How determined the analysis type:** NA.  **Analysed:** No.  **Reasons given (in the review) for not doing interaction analysis if planned:** Not reported (presumably because no trials).  **Reasons given for carrying out interaction analysis if they weren’t planned:** NA.  **Scenario in protocol:** ‘Ideally, patients with a history of epilepsy should be excluded. In cases where studies have recruited a mixture of patients with and without a history of epilepsy, attempts will be made to extract only the results related to those patients without a history of epilepsy. If this is not possible, despite contacting the investigators, a sensitivity analysis will be conducted’  **Rationale for planned scenario:** Not reported.  **Scenario in review (if reported specifically):** Not reported.  **Rationale for done scenario:**NA.  **Reason for changing mind:** NA. | **Method to detect interactions reported in protocol:** Not reported.  **Method to detect interactions reported in review:** Not reported.  **Reason for changing mind:** NA. |
| Marigold 2013 | **Protocol:** ‘In cases where studies recruited patients who had prior neurosurgical procedures (e. g. aneurysmal clipping or coil embolisation) for SAH, the results for these patients will be extracted and analysed separately from those of patients who had not received neurosurgery.’  **Review methods:** ‘In cases where studies recruited patients who had prior neurosurgical procedures (e. g. aneurysmal clipping or coil embolisation) for SAH, the results for these patients will be extracted and analysed separately from those of patients who had not received neurosurgery.’  **Review results:** Not analysed.  **Type:** Patient.  **Covariate summary:** Disease characteristics. | **Method to choose**  **covariate:** Not reported.  **Where method reported:** NA.  **Rationale for choosing the covariate:** Not reported.  **Where rationale reported:** NA. | **Where reported:** Protocol and review.  **Reason not reported in review:** NA.  **Reason not reported in protocol:** NA.  **Labelled as ‘posthoc’ if not in protocol:** NA. | **Data type:** Categorical.  **Continuous covariate categorised:** NA.  **How determine it was categorised:** NA.  **Justification for categorising:** NA.  **Where justification reported:** NA.  **Categories reported:** Yes.  **Where categories reported:** Protocol and review.  **Justification for categories:** Not reported.  **Where justification reported:** NA. | **Analysis type reported in protocol:** Unnamed analysis.  **Analysis type reported in review:** Unnamed analysis.  **Reason for changing mind:** NA.  **Analysis type in protocol according to glossary:** Subgroup analysis.  **How determined the analysis type:** Categories given.  **Analysis type in review according to glossary:** Subgroup analysis.  **How determined the analysis type:** Categories given.  **Analysed:** No.  **Reasons given (in the review) for not doing interaction analysis if planned:** Not reported (presumably because no trials).  **Reasons given for carrying out interaction analysis if they weren’t planned:** NA.  **Scenario in protocol:** None reported.  **Rationale for planned scenario:** NA.  **Scenario in review (if reported specifically):** None reported.  **Rationale for done scenario:** NA.  **Reason for changing mind:** NA. | **Method to detect interactions reported in protocol:** Not reported.  **Method to detect interactions reported in review:** Not reported.  **Reason for changing mind:** NA. |
| Marigold 2013 | **Protocol:** ‘Methodological quality’.  **Review methods: ‘**Methodological quality’.  **Review results:** Not analysed.  **Type:** Methodological.  **Covariate summary:** Quality. | **Method to choose**  **covariate:** Not reported.  **Where method reported:** NA.  **Rationale for choosing the covariate:** Not reported.  **Where rationale reported:** NA. | **Where reported:** Protocol and review.  **Reason not reported in review:** NA.  **Reason not reported in protocol:** NA.  **Labelled as ‘posthoc’ if not in protocol:** NA. | **Data type:** Categorical.  **Continuous covariate categorised:** NA.  **How determine it was categorised:** NA.  **Justification for categorising:** NA.  **Where justification reported:** NA.  **Categories reported:** Not reported.  **Where categories reported:** NA.  **Justification for categories:** Not reported.  **Where justification reported:** NA. | **Analysis type reported in protocol:** Sensitivity analysis.  **Analysis type reported in review:** Sensitivity analysis.  **Reason for changing mind:** NA.  **Analysis type in protocol according to glossary:** Sensitivity analysis.  **How determined the analysis type:** ‘Sensitivity analysis.’ No reason to dispute.  **Analysis type in review according to glossary:** Sensitivity analysis.  **How determined the analysis type:** ‘Sensitivity analysis.’ No reason to dispute.  **Analysed:** No.  **Reasons given (in the review) for not doing interaction analysis if planned:** Not reported (presumably because no trials).  **Reasons given for carrying out interaction analysis if they weren’t planned:** NA.  **Scenario in protocol:** None reported.  **Rationale for planned scenario:** NA.  **Scenario in review (if reported specifically):** None reported.  **Rationale for done scenario:** NA.  **Reason for changing mind:** NA. | **Method to detect interactions reported in protocol:** Not reported.  **Method to detect interactions reported in review:** Not reported.  **Reason for changing mind:** NA. |
| Marigold 2013 | **Protocol:** Excluding the study with the largest sample size. We judged the following to be two covariates: ‘Excluding the study with the smallest or largest sample size.’  **Review methods:** Excluding the study with the largest sample size. We judged the following to be two covariates: ‘Excluding the study with the smallest or largest sample size.’  **Review results:** Not analysed.  **Type:** Methodological.  **Covariate summary:** Trial size. | **Method to choose**  **covariate:** Not reported.  **Where method reported:** NA.  **Rationale for choosing the covariate:** Not reported.  **Where rationale reported:** NA. | **Where reported:** Protocol and review.  **Reason not reported in review:** NA.  **Reason not reported in protocol:** NA.  **Labelled as ‘posthoc’ if not in protocol:** NA. | **Data type:** Continuous.  **Continuous covariate categorised:** Yes.  **How determine it was categorised:** Categories given.  **Justification for categorising:** No reported.  **Where justification reported:** NA.  **Categories reported:** Yes.  **Where categories reported:** Protocol and review.  **Justification for categories:** Not reported.  **Where justification reported:** NA. | **Analysis type reported in protocol:** Sensitivity analysis.  **Analysis type reported in review:** Sensitivity analysis.  **Reason for changing mind:** NA.  **Analysis type in protocol according to glossary:** Sensitivity analysis.  **How determined the analysis type:** ‘Excluding’  **Analysis type in review according to glossary:** Sensitivity analysis.  **How determined the analysis type:** ‘Excluding’  **Analysed:** No.  **Reasons given (in the review) for not doing interaction analysis if planned:** Not reported (presumably because no trials).  **Reasons given for carrying out interaction analysis if they weren’t planned:** NA.  **Scenario in protocol:** None reported.  **Rationale for planned scenario:** NA.  **Scenario in review (if reported specifically):** None reported.  **Rationale for done scenario:** NA.  **Reason for changing mind:** NA. | **Method to detect interactions reported in protocol:** Not reported.  **Method to detect interactions reported in review:** Not reported.  **Reason for changing mind:** NA. |
| Marigold 2013 | **Protocol:** Excluding the study with the smallest sample size. We judged the following to be two covariates: ‘Excluding the study with the smallest or largest sample size.’  **Review methods:** Excluding the study with the smallest sample size. We judged the following to be two covariates: ‘Excluding the study with the smallest or largest sample size.’  **Review results:** Not analysed.  **Type:** Methodological.  **Covariate summary:** Trial size. | **Method to choose**  **covariate:** Not reported.  **Where method reported:** NA.  **Rationale for choosing the covariate:** Not reported.  **Where rationale reported:** NA. | **Where reported:** Protocol and review.  **Reason not reported in review:** NA.  **Reason not reported in protocol:** NA.  **Labelled as ‘posthoc’ if not in protocol:** NA. | **Data type:** Continuous.  **Continuous covariate categorised:** Yes.  **How determine it was categorised:** Categories given.  **Justification for categorising:** No reported.  **Where justification reported:** NA.  **Categories reported:** Yes.  **Where categories reported:** Protocol and review.  **Justification for categories:** Not reported.  **Where justification reported:** NA. | **Analysis type reported in protocol:** Sensitivity analysis.  **Analysis type reported in review:** Sensitivity analysis.  **Reason for changing mind:** NA.  **Analysis type in protocol according to glossary:** Sensitivity analysis.  **How determined the analysis type:** ‘Excluding’  **Analysis type in review according to glossary:** Sensitivity analysis.  **How determined the analysis type:** ‘Excluding’  **Analysed:** No.  **Reasons given (in the review) for not doing interaction analysis if planned:** Not reported (presumably because no trials).  **Reasons given for carrying out interaction analysis if they weren’t planned:** NA.  **Scenario in protocol:** None reported.  **Rationale for planned scenario:** NA.  **Scenario in review (if reported specifically):** None reported.  **Rationale for done scenario:** NA.  **Reason for changing mind:** NA. | **Method to detect interactions reported in protocol:** Not reported.  **Method to detect interactions reported in review:** Not reported.  **Reason for changing mind:** NA. |
| Marigold 2013 | **Protocol:** Removing the study with the largest treatment effects. We judged the following to be two covariates: ‘removing the study with the smallest or largest treatment effects.’  **Review methods:** Removing the study with the largest treatment effects. We judged the following to be two covariates: ‘removing the study with the smallest or largest treatment effects.’  **Review results:** Not analysed.  **Type:** Other.  **Covariate summary:** Effect size. | **Method to choose**  **covariate:** Not reported.  **Where method reported:** NA.  **Rationale for choosing the covariate:** Not reported.  **Where rationale reported:** NA. | **Where reported:** Protocol and review.  **Reason not reported in review:** NA.  **Reason not reported in protocol:** NA.  **Labelled as ‘posthoc’ if not in protocol:** NA. | **Data type:** Continuous.  **Continuous covariate categorised:** Yes.  **How determine it was categorised:** Categories given.  **Justification for categorising:** No reported.  **Where justification reported:** NA.  **Categories reported:** Yes.  **Where categories reported:** Protocol and review.  **Justification for categories:** Not reported.  **Where justification reported:** NA. | **Analysis type reported in protocol:** Sensitivity analysis.  **Analysis type reported in review:** Sensitivity analysis.  **Reason for changing mind:** NA.  **Analysis type in protocol according to glossary:** Sensitivity analysis.  **How determined the analysis type:** ‘removing’.  **Analysis type in review according to glossary:** Sensitivity analysis.  **How determined the analysis type:** ‘removing’.  **Analysed:** No.  **Reasons given (in the review) for not doing interaction analysis if planned:** Not reported (presumably because no trials).  **Reasons given for carrying out interaction analysis if they weren’t planned:** NA.  **Scenario in protocol:** None reported.  **Rationale for planned scenario:** NA.  **Scenario in review (if reported specifically):** None reported.  **Rationale for done scenario:** NA.  **Reason for changing mind:** NA. | **Method to detect interactions reported in protocol:** Not reported.  **Method to detect interactions reported in review:** Not reported.  **Reason for changing mind:** NA. |
| Marigold 2013 | **Protocol:** Removing the study with the smallest treatment effects. We judged the following to be two covariates: ‘removing the study with the smallest or largest treatment effects.’  **Review methods:** Removing the study with the smallest treatment effects. We judged the following to be two covariates: ‘removing the study with the smallest or largest treatment effects.’  **Review results:** Not analysed.  **Type:** Other.  **Covariate summary:** Effect size. | **Method to choose**  **covariate:** Not reported.  **Where method reported:** NA.  **Rationale for choosing the covariate:** Not reported.  **Where rationale reported:** NA. | **Where reported:** Protocol and review.  **Reason not reported in review:** NA.  **Reason not reported in protocol:** NA.  **Labelled as ‘posthoc’ if not in protocol:** NA. | **Data type:** Continuous.  **Continuous covariate categorised:** Yes.  **How determine it was categorised:** Categories given.  **Justification for categorising:** No reported.  **Where justification reported:** NA.  **Categories reported:** Yes.  **Where categories reported:** Protocol and review.  **Justification for categories:** Not reported.  **Where justification reported:** NA. | **Analysis type reported in protocol:** Sensitivity analysis.  **Analysis type reported in review:** Sensitivity analysis.  **Reason for changing mind:** NA.  **Analysis type in protocol according to glossary:** Sensitivity analysis.  **How determined the analysis type:** ‘removing’  **Analysis type in review according to glossary:** Sensitivity analysis.  **How determined the analysis type:** ‘removing’  **Analysed:** No.  **Reasons given (in the review) for not doing interaction analysis if planned:** Not reported (presumably because no trials).  **Reasons given for carrying out interaction analysis if they weren’t planned:** NA.  **Scenario in protocol:** None reported.  **Rationale for planned scenario:** NA.  **Scenario in review (if reported specifically):** None reported.  **Rationale for done scenario:** NA.  **Reason for changing mind:** NA. | **Method to detect interactions reported in protocol:** Not reported.  **Method to detect interactions reported in review:** Not reported.  **Reason for changing mind:** NA. |
| Mocellin 2013 | **Protocol:** ‘Hazard ratio adjusted at multivariate analysis or not’.  **Review methods:** Not reported.  **Review results:** Not analysed.  **Type:** Outcome.  **Covariate summary:** Adjusted treatment effect. | **Method to choose**  **covariate:** Not reported.  **Where method reported:** NA.  **Rationale for choosing the covariate:** Not reported.  **Where rationale reported:** NA. | **Where reported:** Protocol only.  **Reason not reported in review:** Not reported.  **Reason not reported in protocol:** NA.  **Labelled as ‘posthoc’ if not in protocol:** NA. | **Data type:** Categorical.  **Continuous covariate categorised:** NA.  **How determine it was categorised:** NA.  **Justification for categorising:** NA.  **Where justification reported:** NA.  **Categories reported:** Yes  **Where categories reported:** Protocol only.  **Justification for categories:** Not reported.  **Where justification reported:** NA. | **Analysis type reported in protocol:** Subgroup analysis.  **Analysis type reported in review:** Not reported.  **Reason for changing mind:** Not reported.    **Analysis type in protocol according to glossary:** Subgroup analysis.  **How determined the analysis type:** Says’ subgroup analysis’. No reason to dispute.  **Analysis type in review according to glossary:** Not reported.  **How determined the analysis type:** NA.  **Analysed:** No.  **Reasons given (in the review) for not doing interaction analysis if planned:** Not reported.  **Reasons given for carrying out interaction analysis if they weren’t planned:** NA.  **Scenario in protocol:** ‘We will performsubgroup  analysis andmeta-regression irrespective of the levels  of heterogeneity we detect by the I² statistic.’  **Rationale for planned scenario:** Not reported.  **Scenario in review (if reported specifically):** Not reported.  **Rationale for done scenario:** Not reported.  **Reason for changing mind:** NA. | **Method to detect interactions reported in protocol:** Not reported.  **Method to detect interactions reported in review:** Not reported.  **Reason for changing mind:** NA. |
| Mocellin 2013 | **Protocol:** ‘Interferon dosage’.  **Review methods: ‘**Interferon dosage’.  **Review results: ‘**Interferon dosage’. ‘High’; ‘low’; ‘Intermediate’.  **Type:** Intervention.  **Covariate summary:** Dose. | **Method to choose**  **Covariate:** Not reported.  **Where method reported:** NA.  **Rationale for choosing the covariate:** Not reported.  **Where rationale reported:** NA. | **Where reported:** Protocol and review.  **Reason not reported in review:** NA.  **Reason not reported in protocol:** NA.  **Labelled as ‘posthoc’ if not in protocol:** NA. | **Data type:** Continuous.  **Continuous covariate categorised:** Yes.  **How determine it was categorised:** Categories given.  **Justification for categorising:** Not reported.  **Where justification reported:** NA.  **Categories reported:** Yes.  **Where categories reported:** Review only.  **Justification for categories:** Not reported.  **Where justification reported:** NA. | **Analysis type reported in protocol:** Subgroup analysis.  **Analysis type reported in review:** Subgroup analysis and meta-regression.  **Reason for changing mind:** Not reported.    **Analysis type in protocol according to glossary:** Subgroup analysis.  **How determined the analysis type:** Says’ subgroup analysis’. No reason to dispute.  **Analysis type in review according to glossary:** Subgroup analysis.  **How determined the analysis type:** Results Categories given.  **Analysed:** Yes.  **Reasons given (in the review) for not doing interaction analysis if planned:** NA.  **Reasons given for carrying out interaction analysis if they weren’t planned:** NA.  **Scenario in protocol:** ‘We will performsubgroup  analysis andmeta-regression irrespective of the levels  of heterogeneity we detect by the I² statistic.’  **Rationale for planned scenario:** Not reported.  **Scenario in review (if reported specifically):** ‘although we found no significant heterogeneity, we used  subgroup analysis and metaregression to investigate whether some  study design featuresmight have affected trial outcomes.’  **Rationale for done scenario:** Not reported.  **Reason for changing mind:** NA. | **Method to detect interactions reported in protocol:** Not reported.  **Method to detect interactions reported in review:** Not reported.  **Reason for changing mind:** NA. |
| Mocellin 2013 | **Protocol:** ‘Length of follow-up’.  **Review methods:** Not reported.  **Review results:** Not analysed.  **Type:** Methodological.  **Covariate summary:** Length of follow-up. | **Method to choose**  **covariate:** Not reported.  **Where method reported:** NA.  **Rationale for choosing the covariate:** Not reported.  **Where rationale reported:** NA. | **Where reported:** Protocol only.  **Reason not reported in review:** Not reported.  **Reason not reported in protocol:** NA.  **Labelled as ‘posthoc’ if not in protocol:** NA. | **Data type:** Continuous.  **Continuous covariate categorised:** Unclear  **How determine it was categorised:** NA.  **Justification for categorising:** Not reported.  **Where justification reported:** NA  **Categories reported:** Not reported.  **Where categories reported:** NA.  **Justification for categories:** Not reported.  **Where justification reported:** NA. | **Analysis type reported in protocol:** Meta-regression.  **Analysis type reported in review:** Not reported.  **Reason for changing mind:** Not reported.    **Analysis type in protocol according to glossary:** Meta-regression.  **How determined the analysis type:** Says’ meta-regression’. No reason to dispute.  **Analysis type in review according to glossary:** Not reported.  **How determined the analysis type:** NA.  **Analysed:** No.  **Reasons given (in the review) for not doing interaction analysis if planned:** Not reported.  **Reasons given for carrying out interaction analysis if they weren’t planned:** NA.  **Scenario in protocol:** ‘We will performsubgroup  analysis andmeta-regression irrespective of the levels  of heterogeneity we detect by the I² statistic.’  **Rationale for planned scenario:** Not reported.  **Scenario in review (if reported specifically):** Not reported.  **Rationale for done scenario:** Not reported.  **Reason for changing mind:** NA. | **Method to detect interactions reported in protocol:** Not reported.  **Method to detect interactions reported in review:** Not reported.  **Reason for changing mind:** NA. |
| Mocellin 2013 | **Protocol:** ‘Participants’ TNM stage’.  **Review methods: ‘**Participants’ AJCC TNM stage (studies have been conducted with the 5th and 6th edition of the TNM staging manual)’.  **Review results: ‘**TNM stage’. ‘Stage II’; ‘stage III’; ‘stage II-II)’.  **Type:** Patient.  **Covariate summary:** Disease characteristics. | **Method to choose**  **covariate:** Not reported.  **Where method reported:** NA.  **Rationale for choosing the covariate:** Not reported.  **Where rationale reported:** NA. | **Where reported:** Protocol and review.  **Reason not reported in review:** NA.  **Reason not reported in protocol:** NA.  **Labelled as ‘posthoc’ if not in protocol:** NA. | **Data type:** Categorical.  **Continuous covariate categorised:** NA.  **How determine it was categorised:** NA.  **Justification for categorising:** NA.  **Where justification reported:** NA.  **Categories reported:** Yes.  **Where categories reported:** Review only.  **Justification for categories:** Not reported.  **Where justification reported:** NA. | **Analysis type reported in protocol:** Subgroup analysis.  **Analysis type reported in review:** Subgroup analysis and meta-regression.  **Reason for changing mind:** Not reported.    **Analysis type in protocol according to glossary:** Subgroup analysis.  **How determined the analysis type:** Says’ subgroup analysis’. No reason to dispute.  **Analysis type in review according to glossary:** Subgroup analysis.  **How determined the analysis type:** Results reported.  **Analysed:** Yes.  **Reasons given (in the review) for not doing interaction analysis if planned:** NA.  **Reasons given for carrying out interaction analysis if they weren’t planned:** NA.  **Scenario in protocol:** ‘We will performsubgroup  analysis andmeta-regression irrespective of the levels  of heterogeneity we detect by the I² statistic.’  **Rationale for planned scenario:** Not reported.  **Scenario in review (if reported specifically):** ‘although we found no significant heterogeneity, we used  subgroup analysis and metaregression to investigate whether some  study design featuresmight have affected trial outcomes.’  **Rationale for done scenario:** Not reported.  **Reason for changing mind:** NA. | **Method to detect interactions reported in protocol:** Not reported.  **Method to detect interactions reported in review:** Not reported.  **Reason for changing mind:** NA. |
| Mocellin 2013 | **Protocol:** ‘Percentage of cross-over (participants allocated to observation arm that required interferon in case of relapse)’.  **Review methods:** Not reported.  **Review results:** Not analysed.  **Type:** Patient.  **Covariate summary:** Disease characteristics. | **Method to choose**  **covariate:** Not reported.  **Where method reported:** NA.  **Rationale for choosing the covariate:** Not reported.  **Where rationale reported:** NA. | **Where reported:** Protocol only.  **Reason not reported in review:** Not reported.  **Reason not reported in protocol:** NA.  **Labelled as ‘posthoc’ if not in protocol:** NA. | **Data type:** Categorical.  **Continuous covariate categorised:** NA.  **How determine it was categorised:** NA.  **Justification for categorising:** NA.  **Where justification reported:** NA.  **Categories reported:** Not reported.  **Where categories reported:** NA.  **Justification for categories:** Not reported.  **Where justification reported:** NA. | **Analysis type reported in protocol:** Meta-regression.  **Analysis type reported in review:** Not reported.  **Reason for changing mind:** Not reported.    **Analysis type in protocol according to glossary:** Meta-regression.  **How determined the analysis type:** Says’ meta-regression’. No reason to dispute.  **Analysis type in review according to glossary:** Not reported.  **How determined the analysis type:** NA.  **Analysed:** No.  **Reasons given (in the review) for not doing interaction analysis if planned:** Not reported.  **Reasons given for carrying out interaction analysis if they weren’t planned:** NA.  **Scenario in protocol:** ‘We will performsubgroup  analysis andmeta-regression irrespective of the levels  of heterogeneity we detect by the I² statistic.’  **Rationale for planned scenario:** Not reported.  **Scenario in review (if reported specifically):** Not reported.  **Rationale for done scenario:** Not reported.  **Reason for changing mind:** NA. | **Method to detect interactions reported in protocol:** Not reported.  **Method to detect interactions reported in review:** Not reported.  **Reason for changing mind:** NA. |
| Mocellin 2013 | **Protocol:** ‘Percentage of participants who discontinued the treatment because of toxicity (if this information is unavailable, we will consider the percentage of participants who had interferon dose reduction or delay in treatment because of toxicity)’.  **Review methods:** Not reported.  **Review results:** Not analysed.  **Type:** Outcome.  **Covariate summary:** Discontinued the treatment because of toxicity. | **Method to choose**  **covariate:** Not reported.  **Where method reported:** NA.  **Rationale for choosing the covariate:** Not reported.  **Where rationale reported:** NA. | **Where reported:** Protocol only.  **Reason not reported in review:** Not reported.  **Reason not reported in protocol:** NA.  **Labelled as ‘posthoc’ if not in protocol:** NA. | **Data type:** Categorical.  **Continuous covariate categorised:** NA.  **How determine it was categorised:** NA.  **Justification for categorising:** NA.  **Where justification reported:** NA.  **Categories reported:** Not reported.  **Where categories reported:** NA.  **Justification for categories:** Not reported.  **Where justification reported:** NA. | **Analysis type reported in protocol:** Meta-regression.  **Analysis type reported in review:** Not reported.  **Reason for changing mind:** Not reported.    **Analysis type in protocol according to glossary:** Meta-regression.  **How determined the analysis type:** Says’ meta-regression’. No reason to dispute.  **Analysis type in review according to glossary:** Not reported.  **How determined the analysis type:** NA.  **Analysed:** No.  **Reasons given (in the review) for not doing interaction analysis if planned:** Not reported.  **Reasons given for carrying out interaction analysis if they weren’t planned:** NA.  **Scenario in protocol:** ‘We will performsubgroup  analysis andmeta-regression irrespective of the levels  of heterogeneity we detect by the I² statistic.’  **Rationale for planned scenario:** Not reported.  **Scenario in review (if reported specifically):** Not reported.  **Rationale for done scenario:** Not reported.  **Reason for changing mind:** NA. | **Method to detect interactions reported in protocol:** Not reported.  **Method to detect interactions reported in review:** Not reported.  **Reason for changing mind:** NA. |
| Mocellin 2013 | **Protocol:** ‘The presence of poor quality randomised design’.  **Review methods:** Not reported.  **Review results:** Not analysed.  **Type:** Methodological.  **Covariate summary:** Quality. | **Method to choose**  **covariate:** Not reported.  **Where method reported:** NA.  **Rationale for choosing the covariate:** Not reported.  **Where rationale reported:** NA. | **Where reported:** Protocol only.  **Reason not reported in review:** Not reported.  **Reason not reported in protocol:** NA.  **Labelled as ‘posthoc’ if not in protocol:** NA. | **Data type:** Categorical.  **Continuous covariate categorised:** NA.  **How determine it was categorised:** NA.  **Justification for categorising:** NA.  **Where justification reported:** NA.  **Categories reported:** Yes.  **Where categories reported:** Protocol only.  **Justification for categories:** Not reported.  **Where justification reported:** NA. | **Analysis type reported in protocol:** Sensitivity analysis.  **Analysis type reported in review:** Not reported.  **Reason for changing mind:** Not reported.    **Analysis type in protocol according to glossary:** Sensitivity analysis.  **How determined the analysis type:** Says’ Sensitivity analysis.’. No reason to dispute.  **Analysis type in review according to glossary:** Not reported.  **How determined the analysis type:** NA.  **Analysed:** No.  **Reasons given (in the review) for not doing interaction analysis if planned:** Not reported.  **Reasons given for carrying out interaction analysis if they weren’t planned:** NA.  **Scenario in protocol:** ‘We will performsubgroup  analysis andmeta-regression irrespective of the levels  of heterogeneity we detect by the I² statistic.’  **Rationale for planned scenario:** Not reported.  **Scenario in review (if reported specifically):** Not reported.  **Rationale for done scenario:** Not reported.  **Reason for changing mind:** NA. | **Method to detect interactions reported in protocol:** Not reported.  **Method to detect interactions reported in review:** Not reported.  **Reason for changing mind:** NA. |
| Mocellin 2013 | **Protocol: ‘**Treatment duration’.  **Review methods: ‘**Treatment duration’.  **Review results: ‘**Treatment duration’.  **Type:** Intervention.  **Covariate summary:** Duration of intervention. | **Method to choose**  **covariate:** Not reported.  **Where method reported:** NA.  **Rationale for choosing the covariate:** Not reported.  **Where rationale reported:** NA. | **Where reported:** Protocol and review.  **Reason not reported in review:** NA.  **Reason not reported in protocol:** NA.  **Labelled as ‘posthoc’ if not in protocol:** NA. | **Data type:** Continuous.  **Continuous covariate categorised:** No.  **How determine it was categorised:** From meta-regression results.  **Justification for categorising:** NA  **Where justification reported:** NA  **Categories reported:** NA  **Where categories reported:** NA  **Justification for categories:** NA  **Where justification reported:** NA | **Analysis type reported in protocol:** Subgroup analysis and meta-regressions.  **Analysis type reported in review:** Subgroup analysis and meta-regression.  **Reason for changing mind:** NA.    **Analysis type in protocol according to glossary:** Subgroup analysis and meta-regression.  **How determined the analysis type:** Says’Subgroup analysis’ and ‘meta-regression’. No reason to dispute.  **Analysis type in review according to glossary:** Meta-regression.  **How determined the analysis type:** Results reported for meta-regression.  **Analysed:** Yes.  **Reasons given (in the review) for not doing interaction analysis if planned:** NA.  **Reasons given for carrying out interaction analysis if they weren’t planned:** NA.  **Scenario in protocol:** ‘We will performsubgroup  analysis andmeta-regression irrespective of the levels  of heterogeneity we detect by the I² statistic.’  **Rationale for planned scenario:** Not reported.  **Scenario in review (if reported specifically):** ‘although we found no significant heterogeneity, we used  subgroup analysis and metaregression to investigate whether some  study design featuresmight have affected trial outcomes.’  **Rationale for done scenario:** Not reported.  **Reason for changing mind:** NA. | **Method to detect interactions reported in protocol:** Not reported.  **Method to detect interactions reported in review:** Not reported.  **Reason for changing mind:** NA. |
| Mocellin 2013 | **Protocol:** ‘Type of comparator’.  **Review methods: ‘**Type of comparator’.  **Review results:** Not analysed.  **Type:** Intervention.  **Covariate summary:** Type of intervention or control. | **Method to choose**  **covariate:** Not reported.  **Where method reported:** NA.  **Rationale for choosing the covariate:** Not reported.  **Where rationale reported:** NA. | **Where reported:** Protocol and review.  **Reason not reported in review:** NA.  **Reason not reported in protocol:** NA.  **Labelled as ‘posthoc’ if not in protocol:** NA. | **Data type:** Categorical.  **Continuous covariate categorised:** NA.  **How determine it was categorised:** NA.  **Justification for categorising:** NA.  **Where justification reported:** NA.  **Categories reported: Yes.**  **Where categories reported:** Review only.  **Justification for categories:** Not reported.  **Where justification reported:** NA. | **Analysis type reported in protocol:** Subgroup analysis.  **Analysis type reported in review:** Subgroup analysis and meta-regression.  **Reason for changing mind:** Not reported.    **Analysis type in protocol according to glossary:** Subgroup analysis.  **How determined the analysis type:** Says’ subgroup analysis’. No reason to dispute.  **Analysis type in review according to glossary:** Subgroup analysis and meta-regression.  **How determined the analysis type:** Says’ subgroup analysis and meta-regression’. No reason to dispute.  **Analysed:** No.  **Reasons given (in the review) for not doing interaction analysis if planned:** Not reported.  **Reasons given for carrying out interaction analysis if they weren’t planned:** NA.  **Scenario in protocol:** ‘We will performsubgroup  analysis andmeta-regression irrespective of the levels  of heterogeneity we detect by the I² statistic.’  **Rationale for planned scenario:** Not reported.  **Scenario in review (if reported specifically):** ‘although we found no significant heterogeneity, we used  subgroup analysis and metaregression to investigate whether some  study design featuresmight have affected trial outcomes.’  **Rationale for done scenario:** Not reported.  **Reason for changing mind:** NA. | **Method to detect interactions reported in protocol:** Not reported.  **Method to detect interactions reported in review:** Not reported.  **Reason for changing mind:** NA. |
| Mocellin 2013 | **Protocol:** ‘Type of interferon alpha used’.  **Review methods: ‘**Type of interferon alpha used’.  **Review results:** Not analysed.  **Type:** Intervention.  **Covariate summary:** Type of intervention or control. | **Method to choose**  **covariate:** Not reported.  **Where method reported:** NA.  **Rationale for choosing the covariate:** Not reported.  **Where rationale reported:** NA. | **Where reported:** Protocol and review.  **Reason not reported in review:** NA.  **Reason not reported in protocol:** NA.  **Labelled as ‘posthoc’ if not in protocol:** NA. | **Data type:** Categorical.  **Continuous covariate categorised:** NA.  **How determine it was categorised:** NA.  **Justification for categorising:** NA.  **Where justification reported:** NA.  **Categories reported:** Not reported.  **Where categories reported:** NA.  **Justification for categories:** Not reported.  **Where justification reported:** NA. | **Analysis type reported in protocol:** Subgroup analysis.  **Analysis type reported in review:** Subgroup analysis and meta-regression.  **Reason for changing mind:** Not reported.    **Analysis type in protocol according to glossary:** Subgroup analysis.  **How determined the analysis type:** Says’ subgroup analysis’. No reason to dispute.  **Analysis type in review according to glossary:** Subgroup analysis and meta-regression.  **How determined the analysis type:** Says’ subgroup analysis and meta-regression’. No reason to dispute.  **Analysed:** No.  **Reasons given (in the review) for not doing interaction analysis if planned:** Not reported.  **Reasons given for carrying out interaction analysis if they weren’t planned:** NA.  **Scenario in protocol:** ‘We will performsubgroup  analysis andmeta-regression irrespective of the levels  of heterogeneity we detect by the I² statistic.’  **Rationale for planned scenario:** Not reported.  **Scenario in review (if reported specifically):** ‘although we found no significant heterogeneity, we used  subgroup analysis and metaregression to investigate whether some  study design featuresmight have affected trial outcomes.’  **Rationale for done scenario:** Not reported.  **Reason for changing mind:** NA. | **Method to detect interactions reported in protocol:** Not reported.  **Method to detect interactions reported in review:** Not reported.  **Reason for changing mind:** NA. |
| Mocellin 2013 | **Protocol:** ‘Year of publication’.  **Review methods:** ‘Year of publication’.  **Review results: ‘**Publication year’.  **Type:** Other.  **Covariate summary:** Year of publication. | **Method to choose**  **covariate:** Not reported.  **Where method reported:** NA.  **Rationale for choosing the covariate:** Not reported.  **Where rationale reported:** NA. | **Where reported:** Protocol and review.  **Reason not reported in review:** NA.  **Reason not reported in protocol:** NA.  **Labelled as ‘posthoc’ if not in protocol:** NA. | **Data type:** Continuous.  **Continuous covariate categorised:** No.  **How determine it was categorised:** From meta-regression results.  **Justification for categorising:** NA  **Where justification reported:** NA  **Categories reported:** NA  **Where categories reported:** NA  **Justification for categories:** NA  **Where justification reported:** NA | **Analysis type reported in protocol:** Meta-regression.  **Analysis type reported in review:** Subgroup analysis and meta-regression.  **Reason for changing mind:** Not reported.    **Analysis type in protocol according to glossary:** Meta-regression.  **How determined the analysis type:** Says’ meta-regression’. No reason to dispute.  **Analysis type in review according to glossary:** Meta-regression.  **How determined the analysis type:** Results presented.  **Analysed:** Yes.  **Reasons given (in the review) for not doing interaction analysis if planned:** NA.  **Reasons given for carrying out interaction analysis if they weren’t planned:** NA.  **Scenario in protocol:** ‘We will performsubgroup  analysis andmeta-regression irrespective of the levels  of heterogeneity we detect by the I² statistic.’  **Rationale for planned scenario:** Not reported.  **Scenario in review (if reported specifically):** ‘although we found no significant heterogeneity, we used  subgroup analysis and metaregression to investigate whether some  study design featuresmight have affected trial outcomes.’  **Rationale for done scenario:** Not reported.  **Reason for changing mind:** NA. | **Method to detect interactions reported in protocol:** Not reported.  **Method to detect interactions reported in review:** Not reported.  **Reason for changing mind:** NA. |
| Mocellin 2013 | **Protocol:** Not reported.  **Review methods:** Sample size. We judged the following to be two covariates: ‘We employed the exclusion of randomised studies for specific reasons (e. g. differences in control arms or sample size) as a further type of sensitivity analysis.’ **‘**In order to assess the impact of “small” studies, we also performed a sensitivity analysis (see Sensitivity analysis) by excluding these trials. To this aim, we defined “small” as any study with a sample size smaller than 400 based on the following parameters of an ideal trial: A) minimum hazard ratio (HR) = 1. 5; alpha level of significance = 5%; statistical power = 80%; median survival in controls = 60 months; accrual time = 36 months; follow-up time = 48 months; randomisation ratio = 1:1’.  **Review results: ‘**Exclusion of RCTs enrolling fewer than 400 participants’.  **Type:** Methodological.  **Covariate summary:** Trial size. | **Method to choose**  **covariate:** Not reported.  **Where method reported:** NA.  **Rationale for choosing the covariate:** Not reported.  **Where rationale reported:** NA. | **Where reported:** Review only.  **Reason not reported in review:** NA.  **Reason not reported in protocol:** Not reported.  **Labelled as ‘posthoc’ if not in protocol:** Not reported. | **Data type:** Continuous.  **Continuous covariate categorised:** Yes.  **How determine it was categorised:** Categories reported.  **Justification for categorising:** Not reported.  **Where justification reported:** NA.  **Categories reported:** Yes.  **Where categories reported:** Review only.  **Justification for categories:** Not reported.  **Where justification reported:** NA. | **Analysis type reported in protocol:** Not reported.  **Analysis type reported in review:** Sensitivity analysis.  **Reason for changing mind:** Not reported.    **Analysis type in protocol according to glossary:** Not reported.  **How determined the analysis type:** NA.  **Analysis type in review according to glossary:** Sensitivity analysis.  **How determined the analysis type:** ‘exclusion’.  **Analysed:** Yes.  **Reasons given (in the review) for not doing interaction analysis if planned:** NA.  **Reasons given for carrying out interaction analysis if they weren’t planned:** Not reported.  **Scenario in protocol:** Not reported.  **Rationale for planned scenario:** NA.  **Scenario in review (if reported specifically):** Not reported.  **Rationale for done scenario:** Not reported.  **Reason for changing mind:** NA. | **Method to detect interactions reported in protocol:** Not reported.  **Method to detect interactions reported in review:** Not reported.  **Reason for changing mind:** NA. |
| Mocellin 2013 | **Protocol:** Not reported.  **Review methods: ‘**Differences in control arms. We judged the following to be two analyses: ‘We employed the exclusion of randomised studies for specific reasons (e. g. differences in control arms or sample size) as a further type of sensitivity analysis.’  **Review results:** ‘Exclusion of two studies with an active control arm’.  **Type:** Intervention.  **Covariate summary:** Type of intervention or control. | **Method to choose**  **covariate:** Not reported.  **Where method reported:** NA.  **Rationale for choosing the covariate:** Not reported.  **Where rationale reported:** NA. | **Where reported:** Review only.  **Reason not reported in review:** NA.  **Reason not reported in protocol:** Not reported.  **Labelled as ‘posthoc’ if not in protocol:** Not reported. | **Data type:** Categorical.  **Continuous covariate categorised:** NA.  **How determine it was categorised:** NA.  **Justification for categorising:** NA.  **Where justification reported:** NA.  **Categories reported:** Yes.  **Where categories reported:** Review only.  **Justification for categories:** Not reported.  **Where justification reported:** NA. | **Analysis type reported in protocol:** Not reported.  **Analysis type reported in review:** Sensitivity analysis.  **Reason for changing mind:** Not reported.    **Analysis type in protocol according to glossary:** Not reported.  **How determined the analysis type:** NA.  **Analysis type in review according to glossary:** Sensitivity analysis.  **How determined the analysis type:** ‘exclusion’.  **Analysed:** Yes.  **Reasons given (in the review) for not doing interaction analysis if planned:** NA.  **Reasons given for carrying out interaction analysis if they weren’t planned:** Not reported.  **Scenario in protocol:** Not reported.  **Rationale for planned scenario:** NA.  **Scenario in review (if reported specifically):** Not reported.  **Rationale for done scenario:** Not reported.  **Reason for changing mind:** NA. | **Method to detect interactions reported in protocol:** Not reported.  **Method to detect interactions reported in review:** Not reported.  **Reason for changing mind:** NA. |
| Mocellin 2013 | **Protocol:** Percentage of enrolled participants with lymph node metastatic disease.  **Review methods:** Not reported.  **Review results:** Not analysed.  **Type:** Patient.  **Covariate summary:** Disease characteristics. | **Method to choose**  **covariate:** Not reported.  **Where method reported:** NA.  **Rationale for choosing the covariate:** Not reported.  **Where rationale reported:** NA. | **Where reported:** Protocol only.  **Reason not reported in review:** Not reported.  **Reason not reported in protocol:** NA.  **Labelled as ‘posthoc’ if not in protocol:** NA. | **Data type:** Categorical.  **Continuous covariate categorised:** NA.  **How determine it was categorised:** NA.  **Justification for categorising:** NA.  **Where justification reported:** NA.  **Categories reported:** Not reported.  **Where categories reported:** NA.  **Justification for categories:** Not reported.  **Where justification reported:** NA. | **Analysis type reported in protocol:** Meta-regression.  **Analysis type reported in review:** Not reported.  **Reason for changing mind:** Not reported.    **Analysis type in protocol according to glossary:** Meta-regression.  **How determined the analysis type:** Says’ meta-regression’. No reason to dispute.  **Analysis type in review according to glossary:** Not reported.  **How determined the analysis type:** NA.  **Analysed:** No.  **Reasons given (in the review) for not doing interaction analysis if planned:** Not reported.  **Reasons given for carrying out interaction analysis if they weren’t planned:** NA.  **Scenario in protocol:** ‘We will performsubgroup  analysis andmeta-regression irrespective of the levels  of heterogeneity we detect by the I² statistic.’  **Rationale for planned scenario:** Not reported.  **Scenario in review (if reported specifically):** Not reported.  **Rationale for done scenario:** Not reported.  **Reason for changing mind:** NA. | **Method to detect interactions reported in protocol:** Not reported.  **Method to detect interactions reported in review:** Not reported.  **Reason for changing mind:** NA. |
| Mutua 2012 | **Protocol:** ‘Comparisons will be made for different groups of intervention data. This will include treatment versus placebo ± other preventive measure(s), treatment versus no treatment ± other preventive measure(s), treatment versus placebo versus no treatment ± other preventive measure(s).’  **Review methods:** Not reported.  **Review results:** Not analysed.  **Type:** Intervention.  **Covariate summary:** Additional interventions and type of intervention or control. | **Method to choose**  **covariate:** Not reported.  **Where method reported:** NA.  **Rationale for choosing the covariate:** Not reported.  **Where rationale reported:** NA. | **Where reported:** Protocol only.  **Reason not reported in review:** Not reported.  **Reason not reported in protocol:** NA.  **Labelled as ‘posthoc’ if not in protocol:** NA**.** | **Data type:** Categorical.  **Continuous covariate categorised:** NA.  **How determine it was categorised:** NA.  **Justification for categorising:** NA.  **Where justification reported:** NA.  **Categories reported:** Yes.  **Where categories reported:** Protocol only.  **Justification for categories:** Not reported.  **Where justification reported:** NA. | **Analysis type reported in protocol:** Unnamed analysis.  **Analysis type reported in review:** Not reported.  **Reason for changing mind:** Not reported.    **Analysis type in protocol according to glossary:** Stratification/subgroup analysis.  **How determined the analysis type:** Categories given.  **Analysis type in review according to glossary:** Stratification/subgroup analysis.  **How determined the analysis type:** Categories given.  **Analysed:** No.  **Reasons given (in the review) for not doing interaction analysis if planned:** Not reported.  **Reasons given for carrying out interaction analysis if they weren’t planned:** NA.  **Scenario in protocol:** Not reported.  **Rationale for planned scenario:** NA.  **Scenario in review (if reported specifically):** Not reported.  **Rationale for done scenario:** NA.  **Reason for changing mind:** NA**.** | **Method to detect interactions reported in protocol:** Not reported.  **Method to detect interactions reported in review:** Not reported.  **Reason for changing mind:** NA. |
| Mutua 2012 | **Protocol:** ‘Sex of study participants’.  **Review methods: ‘**Sex of study participants’.  **Review results:** Not analysed.  **Type:** Patient.  **Covariate summary:** Demographics. | **Method to choose**  **covariate:** Not reported.  **Where method reported:** NA.  **Rationale for choosing the covariate:** Not reported.  **Where rationale reported:** NA. | **Where reported:** Protocol and review.  **Reason not reported in review:** NA.  **Reason not reported in protocol:** NA.  **Labelled as ‘posthoc’ if not in protocol:** NA**.** | **Data type:** Categorical.  **Continuous covariate categorised:** NA.  **How determine it was categorised:** NA.  **Justification for categorising:** NA.  **Where justification reported:** NA.  **Categories reported:** Not reported.  **Where categories reported:** NA.  **Justification for categories:** Not reported.  **Where justification reported:** NA. | **Analysis type reported in protocol:** Subgroup analysis.  **Analysis type reported in review:** Subgroup analysis.  **Reason for changing mind:** NA.    **Analysis type in protocol according to glossary:** Subgroup analysis.  **How determined the analysis type:** Says ‘Subgroup analysis.’ No reason to dispute.  **Analysis type in review according to glossary:** Subgroup analysis.  **How determined the analysis type:** Says ‘Subgroup analysis.’ No reason to dispute.  **Analysed:** No.  **Reasons given (in the review) for not doing interaction analysis if planned:** Not reported.  **Reasons given for carrying out interaction analysis if they weren’t planned:** NA.  **Scenario in protocol:** ‘Where significant statistical heterogeneity is found, possible causes will be explored by sub-group analyses;’  **Rationale for planned scenario:** Not reported.  **Scenario in review (if reported specifically):** ‘If we had performed a meta-analysis and found significant statistical heterogeneity, we would have been explored possible causes of the inconsistency in study results using subgroup analyses’.  **Rationale for done scenario:** Not reported.  **Reason for changing mind:** NA**.** | **Method to detect interactions reported in protocol:** Not reported.  **Method to detect interactions reported in review:** Not reported.  **Reason for changing mind:** NA. |
| Mutua 2012 | **Protocol: ‘**Study quality (high versus moderate/low risk of bias)’.  **Review methods: ‘**Risk of bias (high/unclear versus low risk of bias)’.  **Review results:** Not analysed.  **Type:** Methodological.  **Covariate summary:** Quality. | **Method to choose**  **covariate:** Not reported.  **Where method reported:** NA.  **Rationale for choosing the covariate:** Not reported.  **Where rationale reported:** NA. | **Where reported:** Protocol and review.  **Reason not reported in review:** NA.  **Reason not reported in protocol:** NA.  **Labelled as ‘posthoc’ if not in protocol:** NA**.** | **Data type:** Categorical.  **Continuous covariate categorised:** NA.  **How determine it was categorised:** NA.  **Justification for categorising:** NA.  **Where justification reported:** NA.  **Categories reported:** Yes.  **Where categories reported:** Protocol and review.  **Justification for categories:** Not reported.  **Where justification reported:** NA. | **Analysis type reported in protocol:** Sensitivity analysis.  **Analysis type reported in review:** Sensitivity analysis..  **Reason for changing mind:** NA.    **Analysis type in protocol according to glossary:** Subgroup analysis.  **How determined the analysis type:** Categories given.  **Analysis type in review according to glossary:** Subgroup analysis.  **How determined the analysis type:** Categories given.  **Analysed:** No.  **Reasons given (in the review) for not doing interaction analysis if planned:** Not reported.  **Reasons given for carrying out interaction analysis if they weren’t planned:** NA.  **Scenario in protocol:** ‘If there are sufficient trials, we will conduct a sensitivity analysis’  **Rationale for planned scenario:** Not reported.  **Scenario in review (if reported specifically):** ‘If we had obtained sufficient trials, we would have conducted a sensitivity analysis’.  **Rationale for done scenario:** Not reported.  **Reason for changing mind:** NA**.** | **Method to detect interactions reported in protocol:** Not reported.  **Method to detect interactions reported in review:** Not reported.  **Reason for changing mind:** NA. |
| Mutua 2012 | **Protocol:** ‘Type of interventions (treatment versus treatment plus other preventive measures, e. g. condom, diaphragm,etc).’  **Review methods: ‘**Type of intervention’.  **Review results:** Not analysed.  **Type:** Intervention.  **Covariate summary:** Type of intervention or control. | **Method to choose**  **covariate:** Not reported.  **Where method reported:** NA.  **Rationale for choosing the covariate:** Not reported.  **Where rationale reported:** NA. | **Where reported:** Protocol and review.  **Reason not reported in review:** NA.  **Reason not reported in protocol:** NA.  **Labelled as ‘posthoc’ if not in protocol:** NA**.** | **Data type:** Categorical.  **Continuous covariate categorised:** NA.  **How determine it was categorised:** NA.  **Justification for categorising:** NA.  **Where justification reported:** NA.  **Categories reported:** Yes.  **Where categories reported:** Protocol only.  **Justification for categories:** Not reported.  **Where justification reported:** NA. | **Analysis type reported in protocol:** Subgroup analysis.  **Analysis type reported in review:** Subgroup analysis.  **Reason for changing mind:** NA.    **Analysis type in protocol according to glossary:** Subgroup analysis.  **How determined the analysis type:** Says ‘Subgroup analysis.’ No reason to dispute.  **Analysis type in review according to glossary:** Subgroup analysis.  **How determined the analysis type:** Says ‘Subgroup analysis.’ No reason to dispute.  **Analysed:** No.  **Reasons given (in the review) for not doing interaction analysis if planned:** Not reported.  **Reasons given for carrying out interaction analysis if they weren’t planned:** NA.  **Scenario in protocol:** ‘Where significant statistical heterogeneity is found, possible causes will be explored by sub-group analyses;’  **Rationale for planned scenario:** Not reported.  **Scenario in review (if reported specifically):** ‘If we had performed a meta-analysis and found significant statistical heterogeneity, we would have been explored possible causes of the inconsistency in study results using subgroup analyses’.  **Rationale for done scenario:** Not reported.  **Reason for changing mind:** NA**.** | **Method to detect interactions reported in protocol:** Not reported.  **Method to detect interactions reported in review:** Not reported.  **Reason for changing mind:** NA. |
| Mutua 2012 | **Protocol:** ‘Type of sexual relationship (heterosexual or homosexual)’.  **Review methods: ‘**Type of sexual relationship (heterosexual or homosexual)’.  **Review results:** Not analysed.  **Type:** Patient.  **Covariate summary:** Demographics. | **Method to choose**  **covariate:** Not reported.  **Where method reported:** NA.  **Rationale for choosing the covariate:** Not reported.  **Where rationale reported:** NA. | **Where reported:** Protocol and review.  **Reason not reported in review:** NA.  **Reason not reported in protocol:** NA.  **Labelled as ‘posthoc’ if not in protocol:** NA**.** | **Data type:** Categorical.  **Continuous covariate categorised:** NA.  **How determine it was categorised:** NA.  **Justification for categorising:** NA.  **Where justification reported:** NA.  **Categories reported:** Yes.  **Where categories reported:** Protocol and review.  **Justification for categories:** Not reported.  **Where justification reported:** NA. | **Analysis type reported in protocol:** Subgroup analysis.  **Analysis type reported in review:** Subgroup analysis.  **Reason for changing mind:** NA.    **Analysis type in protocol according to glossary:** Subgroup analysis.  **How determined the analysis type:** Categories given.  **Analysis type in review according to glossary:** Subgroup analysis.  **How determined the analysis type:** Categories given.  **Analysed:** No.  **Reasons given (in the review) for not doing interaction analysis if planned:** Not reported.  **Reasons given for carrying out interaction analysis if they weren’t planned:** NA.  **Scenario in protocol:** ‘Where significant statistical heterogeneity is found, possible causes will be explored by sub-group analyses;’  **Rationale for planned scenario:** Not reported.  **Scenario in review (if reported specifically):** ‘If we had performed a meta-analysis and found significant statistical heterogeneity, we would have been explored possible causes of the inconsistency in study results using subgroup analyses’.  **Rationale for done scenario:** Not reported.  **Reason for changing mind:** NA**.** | **Method to detect interactions reported in protocol:** Not reported.  **Method to detect interactions reported in review:** Not reported.  **Reason for changing mind:** NA. |
| Mutua 2012 | **Protocol:** Not reported.  **Review methods:** Not reported.  **Review results:** ‘Penicillin vs single-dose azithromycin’; ‘Penicillin vs two-dose azithromycin’; ‘Fleroxacin 400 mg vs fleroxacin 200 mg’; ‘ Fleroxacin 400 mg vs TMP-SMZ’.  **Type:** Intervention.  **Covariate summary:** Type of intervention or control and dose. | **Method to choose**  **covariate:** Not reported.  **Where method reported:** NA.  **Rationale for choosing the covariate:** Not reported.  **Where rationale reported:** NA. | **Where reported:** Review only.  **Reason not reported in review:** NA.  **Reason not reported in protocol:** Not reported.  **Labelled as ‘posthoc’ if not in protocol:** Not reported**.** | **Data type:** Categorical.  **Continuous covariate categorised:** NA.  **How determine it was categorised:** NA.  **Justification for categorising:** NA.  **Where justification reported:** NA.  **Categories reported:** Yes.  **Where categories reported:** Review only.  **Justification for categories:** Not reported.  **Where justification reported:** NA. | **Analysis type reported in protocol:** Unnamed analysis.  **Analysis type reported in review:** Unnamed analysis.  **Reason for changing mind:** NA.    **Analysis type in protocol according to glossary:** Stratification/subgroup analysis.  **How determined the analysis type:** Categories given.  **Analysis type in review according to glossary:** Stratification/subgroup analysis.  **How determined the analysis type:** Categories given.  **Analysed:** Yes.  **Reasons given (in the review) for not doing interaction analysis if planned:** NA.  **Reasons given for carrying out interaction analysis if they weren’t planned:** NA.  **Scenario in protocol:** Not reported.  **Rationale for planned scenario:** NA.  **Scenario in review (if reported specifically):** Not reported.  **Rationale for done scenario:** NA.  **Reason for changing mind:** NA**.** | **Method to detect interactions reported in protocol:** Not reported.  **Method to detect interactions reported in review:** Not reported.  **Reason for changing mind:** NA. |
| Mutua 2012 | **Protocol:** Not reported.  **Review methods: ‘**Concomitant STI’.  **Review results:** Not analysed.  **Type:** Patient.  **Covariate summary:** Disease characteristics. | **Method to choose**  **covariate:** Not reported.  **Where method reported:** NA.  **Rationale for choosing the covariate:** Not reported.  **Where rationale reported:** NA. | **Where reported:** Review only.  **Reason not reported in review:** NA.  **Reason not reported in protocol:** Not reported.  **Labelled as ‘posthoc’ if not in protocol:** Not reported**.** | **Data type:** Categorical.  **Continuous covariate categorised:** NA.  **How determine it was categorised:** NA.  **Justification for categorising:** NA.  **Where justification reported:** NA.  **Categories reported:** Not reported.  **Where categories reported:** NA.  **Justification for categories:** Not reported.  **Where justification reported:** NA. | **Analysis type reported in protocol:** Not reported.  **Analysis type reported in review:** Subgroup analysis.  **Reason for changing mind:** Not reported.    **Analysis type in protocol according to glossary:** Not reported.  **How determined the analysis type:** NA.  **Analysis type in review according to glossary:** Subgroup analysis.  **How determined the analysis type:** Says ‘Subgroup analysis.’ No reason to dispute.  **Analysed:** No.  **Reasons given (in the review) for not doing interaction analysis if planned:** Not reported.  **Reasons given for carrying out interaction analysis if they weren’t planned:** NA.  **Scenario in protocol:** Not reported.  **Rationale for planned scenario:** NA.  **Scenario in review (if reported specifically):** ‘If we had performed a meta-analysis and found significant statistical heterogeneity, we would have been explored possible causes of the inconsistency in study results using subgroup analyses’.  **Rationale for done scenario:** Not reported.  **Reason for changing mind:** NA**.** | **Method to detect interactions reported in protocol:** Not reported.  **Method to detect interactions reported in review:** Not reported.  **Reason for changing mind:** NA. |
| Mutua 2012 | **Protocol:** Not reported.  **Review methods: ‘**Underlying cause of the ulcer’.  **Review results:** ‘Treatment of syphilis’; ‘treatment of chancroid’.  **Type:** Patient.  **Covariate summary:** Disease characteristics. | **Method to choose**  **covariate:** Not reported.  **Where method reported:** NA.  **Rationale for choosing the covariate:** Not reported.  **Where rationale reported:** NA. | **Where reported:** Review only.  **Reason not reported in review:** NA.  **Reason not reported in protocol:** Not reported.  **Labelled as ‘posthoc’ if not in protocol:** Not reported**.** | **Data type:** Categorical.  **Continuous covariate categorised:** NA.  **How determine it was categorised:** NA.  **Justification for categorising:** NA.  **Where justification reported:** NA.  **Categories reported:** Yes.  **Where categories reported:** Review only.  **Justification for categories:** Not reported.  **Where justification reported:** NA. | **Analysis type reported in protocol:** Not reported.  **Analysis type reported in review:** Subgroup analysis.  **Reason for changing mind:** Not reported.    **Analysis type in protocol according to glossary:** Not reported.  **How determined the analysis type:** NA.  **Analysis type in review according to glossary:** Subgroup analysis.  **How determined the analysis type:** Says ‘Subgroup analysis.’ No reason to dispute.  **Analysed:** Yes.  **Reasons given (in the review) for not doing interaction analysis if planned:** NA.  **Reasons given for carrying out interaction analysis if they weren’t planned:** Not reported.  **Scenario in protocol:** Not reported.  **Rationale for planned scenario:** NA.  **Scenario in review (if reported specifically):** ‘If we had performed a meta-analysis and found significant statistical heterogeneity, we would have been explored possible causes of the inconsistency in study results using subgroup analyses’.  **Rationale for done scenario:** Not reported.  **Reason for changing mind:** NA**.** | **Method to detect interactions reported in protocol:** Not reported.  **Method to detect interactions reported in review:** Not reported.  **Reason for changing mind:** NA. |
| Parker 2013 | **Protocol:** ‘Child (under 13 years) and adolescent (13-18 years) participants’.  **Review methods: ‘**Child (under 13 years) and adolescent (13-18 years) participants’.  **Review results:** Not analysed.  **Type:** Patient.  **Covariate summary:** Demographics. | **Method to choose**  **covariate:** Not reported.  **Where method reported:** NA.  **Rationale for choosing the covariate:** Not reported.  **Where rationale reported:** NA. | **Where reported:** Protocol and review.  **Reason not reported in review:** NA.  **Reason not reported in protocol:** NA.  **Labelled as ‘posthoc’ if not in protocol:** NA. | **Data type:** Continuous.  **Continuous covariate categorised:** Yes.  **How determine it was categorised:** Categories given.  **Justification for categorising:** No reported.  **Where justification reported:** NA.  **Categories reported:** Yes.  **Where categories reported:** Protocol and review.  **Justification for categories:** Not reported.  **Where justification reported:** NA. | **Analysis type reported in protocol:** Subgroup analysis.  **Analysis type reported in review:** Subgroup analysis.  **Reason for changing mind:** NA.  **Analysis type in protocol according to glossary:** Subgroup analysis.  **How determined the analysis type:** Categories given.  **Analysis type in review according to glossary:** Subgroup analysis.  **How determined the analysis type:** Categories given.  **Analysed:** No.  **Reasons given (in the review) for not doing interaction analysis if planned:** Not reported (presumably because no trials).  **Reasons given for carrying out interaction analysis if they weren’t planned:** NA.  **Scenario in protocol: ‘**Further investigation of the causes of heterogeneity will be conducted  using subgroup analyses. The following subgroup analyses  will be conducted with all outcomes that have a sufficient number  of studies (normally greater than 10) to see if there are any differences  in response between:’  **Rationale for planned scenario:** Not reported  **Scenario in review (if reported specifically): ‘**In future updates,wewill conduct the following subgroup analyses  for all outcomes that have a sufficient number of studies (normally  greater than 10) to see if there are any differences in response  between:’  **Rationale for done scenario:** Not reported  **Reason for changing mind:** NA. | **Method to detect interactions reported in protocol:** Not reported.  **Method to detect interactions reported in review:** Not reported.  **Reason for changing mind: NA.** |
| Parker 2013 | **Protocol:** ‘Male and female participants’.  **Review methods: ‘**Male and female participants’.  **Review results:** Not analysed.  **Type:** Patient.  **Covariate summary:** Demographics. | **Method to choose**  **covariate:** Not reported.  **Where method reported:** NA.  **Rationale for choosing the covariate:** Not reported.  **Where rationale reported:** NA. | **Where reported:** Protocol and review.  **Reason not reported in review:** NA.  **Reason not reported in protocol:** NA.  **Labelled as ‘posthoc’ if not in protocol:** NA. | **Data type:** Categorical.  **Continuous covariate categorised:** NA.  **How determine it was categorised:** NA.  **Justification for categorising:** NA.  **Where justification reported:** NA.  **Categories reported:** Yes.  **Where categories reported:** Protocol and review.  **Justification for categories:** Not reported.  **Where justification reported:** NA. | **Analysis type reported in protocol:** Subgroup analysis.  **Analysis type reported in review:** Subgroup analysis.  **Reason for changing mind:** NA.  **Analysis type in protocol according to glossary:** Subgroup analysis.  **How determined the analysis type:** Categories given.  **Analysis type in review according to glossary:** Subgroup analysis.  **How determined the analysis type:** Categories given.  **Analysed:** No.  **Reasons given (in the review) for not doing interaction analysis if planned:** Not reported (presumably because no trials).  **Reasons given for carrying out interaction analysis if they weren’t planned:** NA.  **Scenario in protocol: ‘**Further investigation of the causes of heterogeneity will be conducted  using subgroup analyses. The following subgroup analyses  will be conducted with all outcomes that have a sufficient number  of studies (normally greater than 10) to see if there are any differences  in response between:’  **Rationale for planned scenario:** Not reported  **Scenario in review (if reported specifically): ‘**In future updates,wewill conduct the following subgroup analyses  for all outcomes that have a sufficient number of studies (normally  greater than 10) to see if there are any differences in response  between:’  **Rationale for done scenario:** Not reported  **Reason for changing mind:** NA. | **Method to detect interactions reported in protocol:** Not reported.  **Method to detect interactions reported in review:** Not reported.  **Reason for changing mind: NA.** |
| Parker 2013 | **Protocol:** ‘Outcomes will be assessed immediately post intervention, for the short-term (up to one year post-intervention) and long term (over one year post-intervention).’ ‘Multiple time points. Studies of the effectiveness of psychotherapeutic interventions sometimes measure outcomes at multiple time points post-intervention. Ideally time points for assessing the impacts of treatments would be taken from randomisation, but given the likely variability in the duration of interventions between studies we have chosen to group measurements into those taken immediately post intervention, those at short-term follow-up (up to one year post intervention) and those at long-term follow-up (the final measure, greater than one year post-intervention).’  **Review methods:** ‘Outcomes were planned to be assessed immediately post intervention, for the short-term (up to one year post-intervention) and long term (over one year post-intervention).’ ‘Multiple time points. Studies of the effectiveness of psychotherapeutic interventions sometimes measure outcomes at multiple time points post-intervention. Ideally time points for assessing the impacts of treatments would be taken from randomisation, but given the likely variability in the duration of interventions between studies we have chosen to group measurements into those taken immediately post intervention, those at short-term follow-up (up to one year post intervention) and those at long-term follow-up (the final measure, greater than one year post-intervention).’  **Review results:** Not analysed.  **Type:** Outcome.  **Covariate summary:** Time point. | **Method to choose**  **covariate:** Not reported.  **Where method reported:** NA.  **Rationale for choosing the covariate: ‘**‘Studies of the effectiveness of psychotherapeutic interventions sometimes measure outcomes at multiple time points post-intervention. Ideally time points for assessing the impacts of treatments would be taken from randomisation, but given the likely variability in the duration of interventions between studies we have chosen to group measurements....’  **Where rationale reported:** Protocol and review. | **Where reported:** Protocol and review.  **Reason not reported in review:** NA.  **Reason not reported in protocol:** NA.  **Labelled as ‘posthoc’ if not in protocol:** NA. | **Data type:** Continuous.  **Continuous covariate categorised:** Yes.  **How determine it was categorised:** Categories given.  **Justification for categorising:** No reported.  **Where justification reported:** NA.  **Categories reported:** Yes.  **Where categories reported:** Protocol and review.  **Justification for categories:** Not reported.  **Where justification reported:** NA. | **Analysis type reported in protocol:** Unnamed.  **Analysis type reported in review:** Unnamed.  **Reason for changing mind:** NA.  **Analysis type in protocol according to glossary:** Subgroup analysis.  **How determined the analysis type:** Categories given. Meta-regression not mentioned.  **Analysis type in review according to glossary:** Subgroup analysis.  **How determined the analysis type:** Categories given. Meta-regression not mentioned.  **Analysed:** No.  **Reasons given (in the review) for not doing interaction analysis if planned:** Not reported (presumably because no trials).  **Reasons given for carrying out interaction analysis if they weren’t planned:** NA.  **Scenario in protocol:** Not reported.  **Rationale for planned scenario:** NA.  **Scenario in review (if reported specifically):** Not reported.  **Rationale for done scenario:**NA.  **Reason for changing mind:** NA. | **Method to detect interactions reported in protocol:** Not reported.  **Method to detect interactions reported in review:** Not reported.  **Reason for changing mind:** NA. |
| Parker 2013 | **Protocol:** ‘Studies of group treatment versus studies of individual treatment’.  **Review methods: ‘**Studies of group treatment versus studies of individual treatment’.  **Review results:** Not analysed.  **Type:** Intervention.  **Covariate summary:** Type of intervention or control. | **Method to choose**  **covariate:** Not reported.  **Where method reported:** NA.  **Rationale for choosing the covariate:** Not reported.  **Where rationale reported:** NA. | **Where reported:** Protocol and review.  **Reason not reported in review:** NA.  **Reason not reported in protocol:** NA.  **Labelled as ‘posthoc’ if not in protocol:** NA. | **Data type:** Categorical.  **Continuous covariate categorised:** NA.  **How determine it was categorised:** NA.  **Justification for categorising:** NA.  **Where justification reported:** NA.  **Categories reported:** Yes.  **Where categories reported:** Protocol and review.  **Justification for categories:** Not reported.  **Where justification reported:** NA. | **Analysis type reported in protocol:** Subgroup analysis.  **Analysis type reported in review:** Subgroup analysis.  **Reason for changing mind:** NA.  **Analysis type in protocol according to glossary:** Subgroup analysis.  **How determined the analysis type:** Categories given.  **Analysis type in review according to glossary:** Subgroup analysis.  **How determined the analysis type:** Categories given.  **Analysed:** No.  **Reasons given (in the review) for not doing interaction analysis if planned:** Not reported (presumably because no trials).  **Reasons given for carrying out interaction analysis if they weren’t planned:** NA.  **Scenario in protocol: ‘**Further investigation of the causes of heterogeneity will be conducted  using subgroup analyses. The following subgroup analyses  will be conducted with all outcomes that have a sufficient number  of studies (normally greater than 10) to see if there are any differences  in response between:’  **Rationale for planned scenario:** Not reported  **Scenario in review (if reported specifically): ‘**In future updates,wewill conduct the following subgroup analyses  for all outcomes that have a sufficient number of studies (normally  greater than 10) to see if there are any differences in response  between:’  **Rationale for done scenario:** Not reported  **Reason for changing mind:** NA. | **Method to detect interactions reported in protocol:** Not reported.  **Method to detect interactions reported in review:** Not reported.  **Reason for changing mind: NA.** |
| Parker 2013 | **Protocol:** ‘Studies where psychodynamic/psychoanalytic psychotherapy is delivered as an adjunctive treatment versus studies of psychodynamic/psychoanalytic psychotherapy alone.’ ‘Multiple interventions per participant.’If the participants in some trials receive psychoanalytic or psychodynamic psychotherapy plus treatment as usual (eg treatment by a psychiatrist), those studies will be meta-analysed separately, with the psychoanalytic or psychodynamic psychotherapy plus treatment as usual arm compared to treatment as usual alone. The treatment effects of these studies will be reported separately and discussion of these results will consider the extent to which additional treatments may have influenced outcomes.’  **Review methods: ‘**Studies where psychodynamic/psychoanalytic psychotherapy is delivered as an adjunctive treatment versus studies of psychodynamic/psychoanalytic psychotherapy alone.’ ‘Multiple interventions per participant.’If the participants in some trials receive psychoanalytic or psychodynamic psychotherapy plus treatment as usual (eg treatment by a psychiatrist), we will meta-analyse those studies separately, with the psychoanalytic or psychodynamic psychotherapy plus treatment as usual arm compared to treatment as usual alone. We will report the treatment effects of these studies and consider the extent to which additional treatments may have influenced outcomes’.  **Review results:** Not analysed.  **Type:** Intervention.  **Covariate summary:** Additional interventions. | **Method to choose**  **covariate:** Not reported.  **Where method reported:** NA.  **Rationale for choosing the covariate:** Not reported.  **Where rationale reported:** NA. | **Where reported:** Protocol and review.  **Reason not reported in review:** NA.  **Reason not reported in protocol:** NA.  **Labelled as ‘posthoc’ if not in protocol:** NA. | **Data type:** Categorical.  **Continuous covariate categorised:** NA.  **How determine it was categorised:** NA.  **Justification for categorising:** NA.  **Where justification reported:** NA.  **Categories reported:** Yes.  **Where categories reported:** Protocol and review.  **Justification for categories:** Not reported.  **Where justification reported:** NA. | **Analysis type reported in protocol:** Subgroup analysis.  **Analysis type reported in review:** Subgroup analysis.  **Reason for changing mind:** NA.  **Analysis type in protocol according to glossary:** Subgroup analysis.  **How determined the analysis type:** Categories given.  **Analysis type in review according to glossary:** Subgroup analysis.  **How determined the analysis type:** Categories given.  **Analysed:** No.  **Reasons given (in the review) for not doing interaction analysis if planned:** Not reported (presumably because no trials).  **Reasons given for carrying out interaction analysis if they weren’t planned:** NA.  **Scenario in protocol: ‘**Further investigation of the causes of heterogeneity will be conducted  using subgroup analyses. The following subgroup analyses  will be conducted with all outcomes that have a sufficient number  of studies (normally greater than 10) to see if there are any differences  in response between:’  **Rationale for planned scenario:** Not reported  **Scenario in review (if reported specifically): ‘**In future updates,wewill conduct the following subgroup analyses  for all outcomes that have a sufficient number of studies (normally  greater than 10) to see if there are any differences in response  between:’  **Rationale for done scenario:** Not reported  **Reason for changing mind:** NA. | **Method to detect interactions reported in protocol:** Not reported.  **Method to detect interactions reported in review:** Not reported.  **Reason for changing mind: NA.** |
| Parker 2013 | **Protocol:** ‘Studies with the intervention lasting an average of 25 or fewer sessions versus studies with the intervention lasting an average of more than 25 sessions.’  **Review methods: ‘**Studies with the intervention lasting an average of 25 or fewer sessions versus studies with the intervention lasting an average of more than 25 sessions.’  **Review results:** Not analysed.  **Type:** Intervention.  **Covariate summary:** Duration of intervention. | **Method to choose**  **covariate:** Not reported.  **Where method reported:** NA.  **Rationale for choosing the covariate:** Not reported.  **Where rationale reported:** NA. | **Where reported:** Protocol and review.  **Reason not reported in review:** NA.  **Reason not reported in protocol:** NA.  **Labelled as ‘posthoc’ if not in protocol:** NA. | **Data type:** Continuous.  **Continuous covariate categorised:** Yes.  **How determine it was categorised:** Categories given.  **Justification for categorising:** No reported.  **Where justification reported:** NA.  **Categories reported:** Yes.  **Where categories reported:** Protocol and review.  **Justification for categories:** Not reported.  **Where justification reported:** NA. | **Analysis type reported in protocol:** Subgroup analysis.  **Analysis type reported in review:** Subgroup analysis.  **Reason for changing mind:** NA.  **Analysis type in protocol according to glossary:** Subgroup analysis.  **How determined the analysis type:** Categories given.  **Analysis type in review according to glossary:** Subgroup analysis.  **How determined the analysis type:** Categories given.  **Analysed:** No.  **Reasons given (in the review) for not doing interaction analysis if planned:** Not reported (presumably because no trials).  **Reasons given for carrying out interaction analysis if they weren’t planned:** NA.  **Scenario in protocol: ‘**Further investigation of the causes of heterogeneity will be conducted  using subgroup analyses. The following subgroup analyses  will be conducted with all outcomes that have a sufficient number  of studies (normally greater than 10) to see if there are any differences  in response between:’  **Rationale for planned scenario:** Not reported  **Scenario in review (if reported specifically): ‘**In future updates,wewill conduct the following subgroup analyses  for all outcomes that have a sufficient number of studies (normally  greater than 10) to see if there are any differences in response  between:’  **Rationale for done scenario:** Not reported  **Reason for changing mind:** NA. | **Method to detect interactions reported in protocol:** Not reported.  **Method to detect interactions reported in review:** Not reported.  **Reason for changing mind: NA.** |
| Parker 2013 | **Protocol:** ‘The removal of studies with quasi-randomisation’.  **Review methods: ‘**The removal of studies with quasi-randomisation’.  **Review results:** Not analysed.  **Type:** Methodological.  **Covariate summary:** Random generation. | **Method to choose**  **covariate:** Not reported.  **Where method reported:** NA.  **Rationale for choosing the covariate:** Not reported.  **Where rationale reported:** NA. | **Where reported:** Protocol and review.  **Reason not reported in review:** NA.  **Reason not reported in protocol:** NA.  **Labelled as ‘posthoc’ if not in protocol:** NA. | **Data type:** Categorical.  **Continuous covariate categorised:** NA.  **How determine it was categorised:** NA.  **Justification for categorising:** NA.  **Where justification reported:** NA.  **Categories reported:** Yes.  **Where categories reported:** Protocol and review.  **Justification for categories:** Not reported.  **Where justification reported:** NA. | **Analysis type reported in protocol:** Sensitivity analysis.  **Analysis type reported in review:** Sensitivity analysis.  **Reason for changing mind:** NA.  **Analysis type in protocol according to glossary:** Sensitivity analysis.  **How determined the analysis type: ‘**removal’  **Analysis type in review according to glossary:** Sensitivity analysis.  **How determined the analysis type: ‘**removal’  **Analysed:** No.  **Reasons given (in the review) for not doing interaction analysis if planned:** Not reported (presumably because no trials).  **Reasons given for carrying out interaction analysis if they weren’t planned:** NA.  **Scenario in protocol:** ‘If the methodologies or analyses of the studies may have affected  the robustness of the results of the review, sensitivity analyses will be undertaken’  **Rationale for planned scenario:** Not reported.  **Scenario in review (if reported specifically): ‘**If the methodologies or analyses of the studies may have affected the robustness of the results of the review, we will undertake sensitivity analyses’  **Rationale for done scenario:** Not reported.  **Reason for changing mind:** NA. | **Method to detect interactions reported in protocol:** Not reported.  **Method to detect interactions reported in review:** Not reported.  **Reason for changing mind:** NA. |
| Parker 2013 | **Protocol:** Differential attrition. We judged the following to be four covariates: ‘The removal of studies with inconsistencies in the definition, measurement, and/or reporting of results (for example, differential attrition, dropouts, lack of intention-to-treat analysis, outcome measures not taken at consistent time point for all participants).’ ‘When attrition between groups differs within studies, use sensitivity analyses will be used to determine the extent to which these studies may bias the results of the meta-analyses.’  **Review methods:** Differential attrition. We judged the following to be four covariates: ‘The removal of studies with inconsistencies in the definition, measurement, and/or reporting of results (for example, differential attrition, dropouts, lack of intention-to-treat analysis, outcome measures not taken at consistent time point for all participants).’ ‘When attrition between groups differs within studies, we will use sensitivity analyses to determine the extent to which these studies may bias the results of the meta-analyses.’  **Review results:** Not analysed.  **Type:** Methodological.  **Covariate summary:** Incomplete outcome data/follow up. | **Method to choose**  **covariate:** Not reported.  **Where method reported:** NA.  **Rationale for choosing the covariate:** Not reported.  **Where rationale reported:** NA. | **Where reported:** Protocol and review.  **Reason not reported in review:** NA.  **Reason not reported in protocol:** NA.  **Labelled as ‘posthoc’ if not in protocol:** NA. | **Data type:** Continuous.  **Continuous covariate categorised:** Yes.  **How determine it was categorised:** Sensitivity analysis planned.  **Justification for categorising:** No reported.  **Where justification reported:** NA.  **Categories reported:** Not reported.  **Where categories reported:** NA.  **Justification for categories:** Not reported.  **Where justification reported:** NA. | **Analysis type reported in protocol:** Sensitivity analysis.  **Analysis type reported in review:** Sensitivity analysis.  **Reason for changing mind:** NA.  **Analysis type in protocol according to glossary:** Sensitivity analysis.  **How determined the analysis type: ‘**removal’  **Analysis type in review according to glossary:** Sensitivity analysis.  **How determined the analysis type: ‘**removal’  **Analysed:** No.  **Reasons given (in the review) for not doing interaction analysis if planned:** Not reported (presumably because no trials).  **Reasons given for carrying out interaction analysis if they weren’t planned:** NA.  **Scenario in protocol:** ‘If the methodologies or analyses of the studies may have affected  the robustness of the results of the review, sensitivity analyses will be undertaken’  **Rationale for planned scenario:** Not reported.  **Scenario in review (if reported specifically): ‘**If the methodologies or analyses of the studies may have affected the robustness of the results of the review, we will undertake sensitivity analyses’  **Rationale for done scenario:** Not reported.  **Reason for changing mind:** NA. | **Method to detect interactions reported in protocol:** Not reported.  **Method to detect interactions reported in review:** Not reported.  **Reason for changing mind:** NA. |
| Parker 2013 | **Protocol:** Dropouts. We judged the following to be four covariates: ‘The removal of studies with inconsistencies in the definition, measurement, and/or reporting of results (for example, differential attrition, dropouts, lack of intention-to-treat analysis, outcome measures not taken at consistent time point for all participants).’  **Review methods:** Dropouts. We judged the following to be four covariates: ‘The removal of studies with inconsistencies in the definition, measurement, and/or reporting of results (for example, differential attrition, dropouts, lack of intention-to-treat analysis, outcome measures not taken at consistent time point for all participants).’  **Review results:** Not analysed.  **Type:** Methodological.  **Covariate summary:** Incomplete outcome data/follow up. | **Method to choose**  **covariate:** Not reported.  **Where method reported:** NA.  **Rationale for choosing the covariate:** Not reported.  **Where rationale reported:** NA. | **Where reported:** Protocol and review.  **Reason not reported in review:** NA.  **Reason not reported in protocol:** NA.  **Labelled as ‘posthoc’ if not in protocol:** NA. | **Data type:** Continuous.  **Continuous covariate categorised:** Yes.  **How determine it was categorised:** Sensitivity analysis planned.  **Justification for categorising:** No reported.  **Where justification reported:** NA.  **Categories reported:** Not reported.  **Where categories reported:** NA.  **Justification for categories:** Not reported.  **Where justification reported:** NA. | **Analysis type reported in protocol:** Sensitivity analysis.  **Analysis type reported in review:** Sensitivity analysis.  **Reason for changing mind:** NA.  **Analysis type in protocol according to glossary:** Sensitivity analysis.  **How determined the analysis type: ‘**removal’  **Analysis type in review according to glossary:** Sensitivity analysis.  **How determined the analysis type: ‘**removal’  **Analysed:** No.  **Reasons given (in the review) for not doing interaction analysis if planned:** Not reported (presumably because no trials).  **Reasons given for carrying out interaction analysis if they weren’t planned:** NA.  **Scenario in protocol:** ‘If the methodologies or analyses of the studies may have affected  the robustness of the results of the review, sensitivity analyses will be undertaken’  **Rationale for planned scenario:** Not reported.  **Scenario in review (if reported specifically): ‘**If the methodologies or analyses of the studies may have affected the robustness of the results of the review, we will undertake sensitivity analyses’  **Rationale for done scenario:** Not reported.  **Reason for changing mind:** NA. | **Method to detect interactions reported in protocol:** Not reported.  **Method to detect interactions reported in review:** Not reported.  **Reason for changing mind:** NA. |
| Parker 2013 | **Protocol:** Lack of intention-to-treat analysis. We judged the following to be four covariates: ‘The removal of studies with inconsistencies in the definition, measurement, and/or reporting of results (for example, differential attrition, dropouts, lack of intention-to-treat analysis, outcome measures not taken at consistent time point for all participants).’  **Review methods:** Lack of intention-to-treat analysis. We judged the following to be four covariates: ‘The removal of studies with inconsistencies in the definition, measurement, and/or reporting of results (for example, differential attrition, dropouts, lack of intention-to-treat analysis, outcome measures not taken at consistent time point for all participants).’  **Review results:** Not analysed.  **Type:** Methodological.  **Covariate summary:** Intention to treat. | **Method to choose**  **covariate:** Not reported.  **Where method reported:** NA.  **Rationale for choosing the covariate:** Not reported.  **Where rationale reported:** NA. | **Where reported:** Protocol and review.  **Reason not reported in review:** NA.  **Reason not reported in protocol:** NA.  **Labelled as ‘posthoc’ if not in protocol:** NA. | **Data type:** Categorical.  **Continuous covariate categorised:** NA.  **How determine it was categorised:** NA.  **Justification for categorising:** NA.  **Where justification reported:** NA.  **Categories reported:** Yes.  **Where categories reported:** Protocol and review.  **Justification for categories:** Not reported.  **Where justification reported:** NA. | **Analysis type reported in protocol:** Sensitivity analysis.  **Analysis type reported in review:** Sensitivity analysis.  **Reason for changing mind:** NA.  **Analysis type in protocol according to glossary:** Sensitivity analysis.  **How determined the analysis type: ‘**removal’  **Analysis type in review according to glossary:** Sensitivity analysis.  **How determined the analysis type: ‘**removal’  **Analysed:** No.  **Reasons given (in the review) for not doing interaction analysis if planned:** Not reported (presumably because no trials).  **Reasons given for carrying out interaction analysis if they weren’t planned:** NA.  **Scenario in protocol:** ‘If the methodologies or analyses of the studies may have affected  the robustness of the results of the review, sensitivity analyses will be undertaken’  **Rationale for planned scenario:** Not reported.  **Scenario in review (if reported specifically): ‘**If the methodologies or analyses of the studies may have affected the robustness of the results of the review, we will undertake sensitivity analyses’  **Rationale for done scenario:** Not reported.  **Reason for changing mind:** NA. | **Method to detect interactions reported in protocol:** Not reported.  **Method to detect interactions reported in review:** Not reported.  **Reason for changing mind:** NA. |
| Parker 2013 | **Protocol:** Outcome measures not taken at consistent time point for all participants. We judged the following to be four covariates: ‘The removal of studies with inconsistencies in the definition, measurement, and/or reporting of results (for example, differential attrition, dropouts, lack of intention-to-treat analysis, outcome measures not taken at consistent time point for all participants).’  **Review methods:** Outcome measures not taken at consistent time point for all participants. We judged the following to be four covariates: ‘The removal of studies with inconsistencies in the definition, measurement, and/or reporting of results (for example, differential attrition, dropouts, lack of intention-to-treat analysis, outcome measures not taken at consistent time point for all participants).’  **Review results:** Not analysed.  **Type:** Outcome.  **Covariate summary:** Time point. | **Method to choose**  **covariate:** Not reported.  **Where method reported:** NA.  **Rationale for choosing the covariate:** Not reported.  **Where rationale reported:** NA. | **Where reported:** Protocol and review.  **Reason not reported in review:** NA.  **Reason not reported in protocol:** NA.  **Labelled as ‘posthoc’ if not in protocol:** NA. | **Data type:** Continuous.  **Continuous covariate categorised:** Yes.  **How determine it was categorised:** Sensitivity analysis planned.  **Justification for categorising:** No reported.  **Where justification reported:** NA.  **Categories reported:** Not reported.  **Where categories reported:** NA.  **Justification for categories:** Not reported.  **Where justification reported:** NA. | **Analysis type reported in protocol:** Sensitivity analysis.  **Analysis type reported in review:** Sensitivity analysis.  **Reason for changing mind:** NA.  **Analysis type in protocol according to glossary:** Sensitivity analysis.  **How determined the analysis type: ‘**removal’  **Analysis type in review according to glossary:** Sensitivity analysis.  **How determined the analysis type: ‘**removal’  **Analysed:** No.  **Reasons given (in the review) for not doing interaction analysis if planned:** Not reported (presumably because no trials).  **Reasons given for carrying out interaction analysis if they weren’t planned:** NA.  **Scenario in protocol:** ‘If the methodologies or analyses of the studies may have affected  the robustness of the results of the review, sensitivity analyses will be undertaken’  **Rationale for planned scenario:** Not reported.  **Scenario in review (if reported specifically): ‘**If the methodologies or analyses of the studies may have affected the robustness of the results of the review, we will undertake sensitivity analyses’  **Rationale for done scenario:** Not reported.  **Reason for changing mind:** NA. | **Method to detect interactions reported in protocol:** Not reported.  **Method to detect interactions reported in review:** Not reported.  **Reason for changing mind:** NA. |
| Pega 2013 | **Protocol:** ‘Family type (one-parent family, two-parent family).’  **Review methods: ‘**Family type (one-parent family, two-parent family).’  **Review results:** Not analysed.  **Type:** Patient.  **Covariate summary:** Demographics. | **Method to choose**  **covariate:** Not reported.  **Where method reported:** NA.  **Rationale for choosing the covariate:** The rationale  for conducting these subgroup analyses is that the impact of  the intervention might significantly differ by populations defined  by these key dimensions. For example, the subgroup analyses by  ethnicity will enable an assessment of difference in treatment effect  size between different ethnic groups, such as Indigenous versus  non-indigenous groups, which will provide an important equity  perspective..  **Where rationale reported:** Protocol only. | **Where reported:** Protocol and review.  **Reason not reported in review:** NA.  **Reason not reported in protocol:** NA.  **Labelled as ‘posthoc’ if not in protocol:** NA. | **Data type:** Categorical.  **Continuous covariate categorised:** NA.  **How determine it was categorised:** NA.  **Justification for categorising:** NA.  **Where justification reported:** NA.  **Categories reported:** Yes.  **Where categories reported:** Protocol and review.  **Justification for categories:** Not reported.  **Where justification reported:** NA. | **Analysis type reported in protocol:** Subgroup analysis.  **Analysis type reported in review:** Subgroup analysis..  **Reason for changing mind:** NA.    **Analysis type in protocol according to glossary:** Subgroup analysis.  **How determined the analysis type:** Says ‘Subgroup analysis.’. No reason to dispute. Categories given.  **Analysis type in review according to glossary:** Subgroup analysis.  **How determined the analysis type:** Says ‘Subgroup analysis.’. No reason to dispute. Categories given.  **Analysed:** No.  **Reasons given (in the review) for not doing interaction analysis if planned:** ‘However, the small number of studies included in the review prohibited meaningful subgroup analyses (Higgins 2011).’  **Reasons given for carrying out interaction analysis if they weren’t planned:** NA.  **Scenario in protocol:** ‘If appropriate data are available, subgroup analyses on the primary  analyses (whether meta-analyses or narrative syntheses) will  be conducted’  **Rationale for planned scenario:** Not reported.  **Scenario in review (if reported specifically):** Not reported.  **Rationale for done scenario:** NA.  **Reason for changing mind:** NA. | **Method to detect interactions reported in protocol:** If the number of studies included in a subgroup analysis  is sufficiently large to make statistical testing meaningful, ttests  and X2-tests will be used to test for differences in treatment  effects between subgroups.  **Method to detect interactions reported in review:** Not reported.  **Reason for changing mind:** Not reported. |
| Pega 2013 | **Protocol:** ‘Gender (female, male)’.  **Review methods: ‘**Gender (female, male)’.  **Review results:** Not analysed.  **Type:** Patient.  **Covariate summary:** Demographics. | **Method to choose**  **covariate:** Not reported.  **Where method reported:** NA.  **Rationale for choosing the covariate:** The rationale  for conducting these subgroup analyses is that the impact of  the intervention might significantly differ by populations defined  by these key dimensions. For example, the subgroup analyses by  ethnicity will enable an assessment of difference in treatment effect  size between different ethnic groups, such as Indigenous versus  non-indigenous groups, which will provide an important equity  perspective..  **Where rationale reported:** Protocol only. | **Where reported:** Protocol and review.  **Reason not reported in review:** NA.  **Reason not reported in protocol:** NA.  **Labelled as ‘posthoc’ if not in protocol:** NA. | **Data type:** Categorical.  **Continuous covariate categorised:** NA.  **How determine it was categorised:** NA.  **Justification for categorising:** NA.  **Where justification reported:** NA.  **Categories reported:** Yes.  **Where categories reported:** Protocol and review.  **Justification for categories:** Not reported.  **Where justification reported:** NA. | **Analysis type reported in protocol:** Subgroup analysis.  **Analysis type reported in review:** Subgroup analysis..  **Reason for changing mind:** NA.    **Analysis type in protocol according to glossary:** Subgroup analysis.  **How determined the analysis type:** Says ‘Subgroup analysis.’. No reason to dispute. Categories given.  **Analysis type in review according to glossary:** Subgroup analysis.  **How determined the analysis type:** Says ‘Subgroup analysis.’. No reason to dispute. Categories given.  **Analysed:** No.  **Reasons given (in the review) for not doing interaction analysis if planned:** ‘However, the small number of studies included in the review prohibited meaningful subgroup analyses (Higgins 2011).’  **Reasons given for carrying out interaction analysis if they weren’t planned:** NA.  **Scenario in protocol:** ‘If appropriate data are available, subgroup analyses on the primary  analyses (whether meta-analyses or narrative syntheses) will  be conducted’  **Rationale for planned scenario:** Not reported.  **Scenario in review (if reported specifically):** Not reported.  **Rationale for done scenario:** NA.  **Reason for changing mind:** NA. | **Method to detect interactions reported in protocol:** If the number of studies included in a subgroup analysis  is sufficiently large to make statistical testing meaningful, ttests  and X2-tests will be used to test for differences in treatment  effects between subgroups.  **Method to detect interactions reported in review:** Not reported.  **Reason for changing mind:** Not reported. |
| Pega 2013 | **Protocol:** ‘If parallel group and cross-over studies have been included in the same meta-analysis, we will conduct a sensitivity analysis that will exclusively include parallel group trials.’  **Review methods:** Not reported.  **Review results:** Not analysed.  **Type:** Methodological.  **Covariate summary:** Trial design. | **Method to choose**  **covariate:** Not reported.  **Where method reported:** NA.  **Rationale for choosing the covariate:** Not reported.  **Where rationale reported:** NA. | **Where reported:** Protocol only.  **Reason not reported in review:** Not reported.  **Reason not reported in protocol:** NA.  **Labelled as ‘posthoc’ if not in protocol:** NA**.** | **Data type:** Categorical.  **Continuous covariate categorised:** NA.  **How determine it was categorised:** NA.  **Justification for categorising:** NA.  **Where justification reported:** NA.  **Categories reported:** Yes.  **Where categories reported:** Protocol only.  **Justification for categories:** Not reported.  **Where justification reported:** NA. | **Analysis type reported in protocol:** Sensitivity analysis.  **Analysis type reported in review:** Sensitivity analysis.  **Reason for changing mind:** NA.    **Analysis type in protocol according to glossary:** Sensitivity analysis.  **How determined the analysis type:** Says ‘Sensitivity analysis’. No reason to dispute.  **Analysis type in review according to glossary:** Sensitivity analysis.  **How determined the analysis type:** Says ‘Sensitivity analysis’. No reason to dispute.  **Analysed:** No.  **Reasons given (in the review) for not doing interaction analysis if planned:** ‘Since no meta-analysis was conducted, sensitivity analyses were also not performed.’  **Reasons given for carrying out interaction analysis if they weren’t planned:** NA.  **Scenario in protocol:** ‘If meta-analyses have been conducted’  **Rationale for planned scenario:** Not reported.  **Scenario in review (if reported specifically):** Not reported.  **Rationale for done scenario:** NA.  **Reason for changing mind:** NA. | **Method to detect interactions reported in protocol:** Not reported.  **Method to detect interactions reported in review:** Not reported.  **Reason for changing mind:** NA. |
| Pega 2013 | **Protocol:** ‘Include only high-quality studies with a low risk of bias.’  **Review methods:** Not reported.  **Review results:** Not analysed.  **Type:** Methodological.  **Covariate summary:** Quality. | **Method to choose**  **covariate:** Not reported.  **Where method reported:** NA.  **Rationale for choosing the covariate:** Not reported.  **Where rationale reported:** NA. | **Where reported:** Protocol only.  **Reason not reported in review:** Not reported.  **Reason not reported in protocol:** NA.  **Labelled as ‘posthoc’ if not in protocol:** NA**.** | **Data type:** Categorical.  **Continuous covariate categorised:** NA.  **How determine it was categorised:** NA.  **Justification for categorising:** NA.  **Where justification reported:** NA.  **Categories reported:** Yes.  **Where categories reported:** Protocol only.  **Justification for categories:** Not reported.  **Where justification reported:** NA. | **Analysis type reported in protocol:** Sensitivity analysis.  **Analysis type reported in review:** Sensitivity analysis.  **Reason for changing mind:** NA.    **Analysis type in protocol according to glossary:** Sensitivity analysis.  **How determined the analysis type:** Says ‘Sensitivity analysis’. No reason to dispute.  **Analysis type in review according to glossary:** Sensitivity analysis.  **How determined the analysis type:** Says ‘Sensitivity analysis’. No reason to dispute.  **Analysed:** No.  **Reasons given (in the review) for not doing interaction analysis if planned:** ‘Since no meta-analysis was conducted, sensitivity analyses were also not performed.’  **Reasons given for carrying out interaction analysis if they weren’t planned:** NA.  **Scenario in protocol:** ‘If meta-analyses have been conducted’  **Rationale for planned scenario:** Not reported.  **Scenario in review (if reported specifically):** Not reported.  **Rationale for done scenario:** NA.  **Reason for changing mind:** NA. | **Method to detect interactions reported in protocol:** Not reported.  **Method to detect interactions reported in review:** Not reported.  **Reason for changing mind:** NA. |
| Pega 2013 | **Protocol:** ‘Income (for example, after-tax personal income or family income)’.  **Review methods: ‘**Income (for example, after-tax personal income or family income)’.  **Review results:** Not analysed.  **Type:** Patient.  **Covariate summary:** Demographics. | **Method to choose**  **covariate:** Not reported.  **Where method reported:** NA.  **Rationale for choosing the covariate:** The rationale  for conducting these subgroup analyses is that the impact of  the intervention might significantly differ by populations defined  by these key dimensions. For example, the subgroup analyses by  ethnicity will enable an assessment of difference in treatment effect  size between different ethnic groups, such as Indigenous versus  non-indigenous groups, which will provide an important equity  perspective..  **Where rationale reported:** Protocol only. | **Where reported:** Protocol and review.  **Reason not reported in review:** NA.  **Reason not reported in protocol:** NA.  **Labelled as ‘posthoc’ if not in protocol:** NA. | **Data type:** Continuous.  **Continuous covariate categorised:** Yes.  **How determine it was categorised:** Subgroup analyses Planned.  **Justification for categorising:** Not reported.  **Where justification reported:** NA  **Categories reported:** Not reported.  **Where categories reported:** NA.  **Justification for categories:** Not reported.  **Where justification reported:** NA. | **Analysis type reported in protocol:** Subgroup analysis.  **Analysis type reported in review:** Subgroup analysis..  **Reason for changing mind:** NA.    **Analysis type in protocol according to glossary:** Subgroup analysis.  **How determined the analysis type:** Says ‘Subgroup analysis.’. No reason to dispute.  **Analysis type in review according to glossary:** Subgroup analysis.  **How determined the analysis type:** Says ‘Subgroup analysis.’. No reason to dispute.  **Analysed:** No.  **Reasons given (in the review) for not doing interaction analysis if planned:** ‘However, the small number of studies included in the review prohibited meaningful subgroup analyses (Higgins 2011).’  **Reasons given for carrying out interaction analysis if they weren’t planned:** NA.  **Scenario in protocol:** ‘If appropriate data are available, subgroup analyses on the primary  analyses (whether meta-analyses or narrative syntheses) will  be conducted’  **Rationale for planned scenario:** Not reported.  **Scenario in review (if reported specifically):** Not reported.  **Rationale for done scenario:** NA.  **Reason for changing mind:** NA. | **Method to detect interactions reported in protocol:** If the number of studies included in a subgroup analysis  is sufficiently large to make statistical testing meaningful, ttests  and X2-tests will be used to test for differences in treatment  effects between subgroups.  **Method to detect interactions reported in review:** Not reported.  **Reason for changing mind:** Not reported. |
| Pega 2013 | **Protocol:** ‘Participant ethnicity’.  **Review methods: ‘**Ethnicity’.  **Review results:** Not analysed.  **Type:** Patient.  **Covariate summary:** Demographics. | **Method to choose**  **covariate:** Not reported.  **Where method reported:** NA.  **Rationale for choosing the covariate:** The rationale  for conducting these subgroup analyses is that the impact of  the intervention might significantly differ by populations defined  by these key dimensions. For example, the subgroup analyses by  ethnicity will enable an assessment of difference in treatment effect  size between different ethnic groups, such as Indigenous versus  non-indigenous groups, which will provide an important equity  perspective..  **Where rationale reported:** Protocol only. | **Where reported:** Protocol and review.  **Reason not reported in review:** NA.  **Reason not reported in protocol:** NA.  **Labelled as ‘posthoc’ if not in protocol:** NA. | **Data type:** Categorical.  **Continuous covariate categorised:** NA.  **How determine it was categorised:** NA.  **Justification for categorising:** NA.  **Where justification reported:** NA.  **Categories reported:** Not reported.  **Where categories reported:** NA.  **Justification for categories:** Not reported.  **Where justification reported:** NA. | **Analysis type reported in protocol:** Subgroup analysis.  **Analysis type reported in review:** Subgroup analysis..  **Reason for changing mind:** NA.    **Analysis type in protocol according to glossary:** Subgroup analysis.  **How determined the analysis type:** Says ‘Subgroup analysis.’. No reason to dispute.  **Analysis type in review according to glossary:** Subgroup analysis.  **How determined the analysis type:** Says ‘Subgroup analysis.’. No reason to dispute.  **Analysed:** No.  **Reasons given (in the review) for not doing interaction analysis if planned:** ‘However, the small number of studies included in the review prohibited meaningful subgroup analyses (Higgins 2011).’  **Reasons given for carrying out interaction analysis if they weren’t planned:** NA.  **Scenario in protocol:** ‘If appropriate data are available, subgroup analyses on the primary  analyses (whether meta-analyses or narrative syntheses) will  be conducted’  **Rationale for planned scenario:** Not reported.  **Scenario in review (if reported specifically):** Not reported.  **Rationale for done scenario:** NA.  **Reason for changing mind:** NA. | **Method to detect interactions reported in protocol:** If the number of studies included in a subgroup analysis  is sufficiently large to make statistical testing meaningful, ttests  and X2-tests will be used to test for differences in treatment  effects between subgroups.  **Method to detect interactions reported in review:** Not reported.  **Reason for changing mind:** Not reported. |
| Penninga 2013 | **Protocol:** ‘Adult compared to both adult and paediatric studies, as differences in immunology in paediatric patients might be  expected (Aurora 2009; Christie 2009).’  **Review methods:** ‘Adult compared with paediatric studies. This was planned because immunological differences in paediatric patients might be expected (Aurora 2009; Christie 2009).’  **Review results:** Not analysed.  **Type:** Patient.  **Covariate summary:** Demographics. | **Method to choose**  **covariate:** Not reported.  **Where method reported:** NA.  **Rationale for choosing the covariate:** ‘differences in immunology in paediatric patients might be  expected (Aurora 2009; Christie 2009).’  **Where rationale reported:** Protocol and review. | **Where reported:** Protocol and review.  **Reason not reported in review:** NA.  **Reason not reported in protocol:** NA.  **Labelled as ‘posthoc’ if not in protocol:** NA. | **Data type:** Continuous.  **Continuous covariate categorised:** Yes.  **How determine it was categorised:** Categories given.  **Justification for categorising:** Not reported.  **Where justification reported:** NA.  **Categories reported:** Yes.  **Where categories reported:** Protocol and review.  **Justification for categories:** ‘differences in immunology in paediatric patients might be expected (Aurora 2009; Christie 2009).’  **Where justification reported:** Protocol and review. | **Analysis type reported in protocol:** Subgroup analysis.  **Analysis type reported in review:** Subgroup analysis.  **Reason for changing mind:** NA.    **Analysis type in protocol according to glossary:** Subgroup analysis.  **How determined the analysis type:** Categories given.  **Analysis type in review according to glossary:** Subgroup analysis.  **How determined the analysis type:** Categories given.  **Analysed:** No.  **Reasons given (in the review) for not doing interaction analysis if planned:** ‘we were unable to perform subgroup analyses  comparing adult and paediatric studies because all participants  in the included studies were adults.’  **Reasons given for carrying out interaction analysis if they weren’t planned:** NA.  **Scenario in protocol:** Not reported.  **Rationale for planned scenario:** NA..  **Scenario in review (if reported specifically):** Not reported.  **Rationale for done scenario:** NA.  **Reason for changing mind:** NA**.** | **Method to detect interactions reported in protocol: ‘**Test of interaction will be performed to evaluate the difference  between the two estimates (Altman 2003).’  **Method to detect interactions reported in review:** Tests for subgroup differences were performed to evaluate differences  between estimates.  **Reason for changing mind:** NA. |
| Penninga 2013 | **Protocol:** ‘C2-monitoring (2 hours post-dose monitoring) of cyclosporin compared to cyclosporin dosing based on of trough levels, as better outcome has been reported for C2-monitoring of cyclosporin (Iversen 2009).’  **Review methods: ‘**Tacrolimus versus two hours post-dose monitoring of cyclosporin (C2-monitoring) compared with tacrolimus versus cyclosporin dosing based on trough levels (C0-monitoring). This was planned to investigate reports of better outcomes for two hours post-dose monitoring of cyclosporin (Iversen 2009a)’.  **Review results:** Not analysed.  **Type:** Intervention.  **Covariate summary:** Type of intervention or control. | **Method to choose**  **covariate:** Not reported.  **Where method reported:** NA.  **Rationale for choosing the covariate:** ‘as better outcome has been reported for C2-monitoring of  cyclosporin (Iversen 2009).’  **Where rationale reported:** Protocol and review. | **Where reported:** Protocol and review.  **Reason not reported in review:** NA.  **Reason not reported in protocol:** NA.  **Labelled as ‘posthoc’ if not in protocol:** NA. | **Data type:** Categorical.  **Continuous covariate categorised:** NA.  **How determine it was categorised:** NA.  **Justification for categorising:** NA.  **Where justification reported:** NA.  **Categories reported:** Yes.  **Where categories reported:** Protocol and review.  **Justification for categories: ‘**as better outcome has been reported for C2-monitoring of cyclosporin (Iversen 2009).’  **Where justification reported:** Protocol and review. | **Analysis type reported in protocol:** Subgroup analysis.  **Analysis type reported in review:** Subgroup analysis.  **Reason for changing mind:** NA.    **Analysis type in protocol according to glossary:** Subgroup analysis.  **How determined the analysis type:** Categories given.  **Analysis type in review according to glossary:** Subgroup analysis.  **How determined the analysis type:** Categories given.  **Analysed:** No.  **Reasons given (in the review) for not doing interaction analysis if planned:** ‘we were unable to performsubgroup analysis of C2-  monitoring (2 hours post-dose monitoring) of cyclosporin compared  with cyclosporin dosing based on of trough levels (C0): all  included studies applied cyclosporin dosing based on trough levels  (C0).’  **Reasons given for carrying out interaction analysis if they weren’t planned:** NA.  **Scenario in protocol:** Not reported.  **Rationale for planned scenario:** NA..  **Scenario in review (if reported specifically):** Not reported.  **Rationale for done scenario:** NA.  **Reason for changing mind:** NA**.** | **Method to detect interactions reported in protocol: ‘**Test of interaction will be performed to evaluate the difference  between the two estimates (Altman 2003).’  **Method to detect interactions reported in review:** Tests for subgroup differences were performed to evaluate differences  between estimates.  **Reason for changing mind:** NA. |
| Penninga 2013 | **Protocol:** ‘Oil-based cyclosporin compared to micro emulsion cyclosporin studies, as differences in absorption and oral bioavailability of the two formulas has been described (Lee 1998; Cantarovich 2004; Kahan 2004; Penninga 2010).’  **Review methods:** ‘Tacrolimus versus oil-based cyclosporin compared with tacrolimus versus microemulsion cyclosporin studies. This subgroup analysis was planned because differences in absorption and oral bioavailability of the two formulae have been described (Cantarovich 2004; Kahan 2004; Lee 1998; Penninga 2010a).’  **Review results:** Not analysed.  **Type:** Intervention.  **Covariate summary:** Type of intervention or control. | **Method to choose**  **covariate:** Not reported.  **Where method reported:** NA.  **Rationale for choosing the covariate:** ‘differences in absorption and oral  bioavailability of the two formulas has been described (Lee 1998;  Cantarovich 2004; Kahan 2004; Penninga 2010).’  **Where rationale reported:** Protocol and review. | **Where reported:** Protocol and review.  **Reason not reported in review:** NA.  **Reason not reported in protocol:** NA.  **Labelled as ‘posthoc’ if not in protocol:** NA. | **Data type:** Categorical.  **Continuous covariate categorised:** NA.  **How determine it was categorised:** NA.  **Justification for categorising:** NA.  **Where justification reported:** NA.  **Categories reported:** Yes.  **Where categories reported:** Protocol and review.  **Justification for categories:** ‘differences in absorption and oral bioavailability of the two formulas has been described (Lee 1998; Cantarovich 2004; Kahan 2004; Penninga 2010).’  **Where justification reported:** Protocol and review. | **Analysis type reported in protocol:** Subgroup analysis.  **Analysis type reported in review:** Subgroup analysis.  **Reason for changing mind:** NA.    **Analysis type in protocol according to glossary:** Subgroup analysis.  **How determined the analysis type:** Categories given.  **Analysis type in review according to glossary:** Subgroup analysis.  **How determined the analysis type:** Categories given.  **Analysed:** No.  **Reasons given (in the review) for not doing interaction analysis if planned:** ‘We were unable to perform subgroup analysis on tacrolimus versus  oil-based cyclosporin compared with tacrolimus versus microemulsion  cyclosporin studies: the included studies used microemulsion  or oral solution cyclosporin only.’  **Reasons given for carrying out interaction analysis if they weren’t planned:** NA.  **Scenario in protocol:** Not reported.  **Rationale for planned scenario:** NA..  **Scenario in review (if reported specifically):** Not reported.  **Rationale for done scenario:** NA.  **Reason for changing mind:** NA**.** | **Method to detect interactions reported in protocol: ‘**Test of interaction will be performed to evaluate the difference  between the two estimates (Altman 2003).’  **Method to detect interactions reported in review:** Tests for subgroup differences were performed to evaluate differences  between estimates.  **Reason for changing mind:** NA. |
| Penninga 2013 | **Protocol: ‘**Single compared to double lung transplant patients, as differences might be expected (Christie 2009).’  **Review methods:** ‘Single compared with double lung transplant patients. Subgroup analysis was planned because we anticipated differences between these populations (Christie 2009).’  **Review results:** Not analysed.  **Type:** Patient.  **Covariate summary:** Disease characteristics. | **Method to choose**  **covariate:** Not reported.  **Where method reported:** NA.  **Rationale for choosing the covariate:** ‘we anticipated differences  between these populations (Christie 2009).’  **Where rationale reported:** Protocol and review. | **Where reported:** Protocol and review.  **Reason not reported in review:** NA.  **Reason not reported in protocol:** NA.  **Labelled as ‘posthoc’ if not in protocol:** NA. | **Data type:** Categorical.  **Continuous covariate categorised:** NA.  **How determine it was categorised:** NA.  **Justification for categorising:** NA.  **Where justification reported:** NA.  **Categories reported:** Yes.  **Where categories reported:** Protocol and review.  **Justification for categories:** ‘we anticipated differences  between these populations (Christie 2009).’  **Where justification reported:** Protocol and review. | **Analysis type reported in protocol:** Subgroup analysis.  **Analysis type reported in review:** Subgroup analysis.  **Reason for changing mind:** NA.    **Analysis type in protocol according to glossary:** Subgroup analysis.  **How determined the analysis type:** Categories given.  **Analysis type in review according to glossary:** Subgroup analysis.  **How determined the analysis type:** Categories given.  **Analysed:** No.  **Reasons given (in the review) for not doing interaction analysis if planned:** ‘None of the studies reported  separately on results for recipients of single lung transplants compared  with double lung transplants, and hence, were also unable  to perform this subgroup analysis.’  **Reasons given for carrying out interaction analysis if they weren’t planned:** NA.  **Scenario in protocol:** Not reported.  **Rationale for planned scenario:** NA..  **Scenario in review (if reported specifically):** Not reported.  **Rationale for done scenario:** NA.  **Reason for changing mind:** NA**.** | **Method to detect interactions reported in protocol: ‘**Test of interaction will be performed to evaluate the difference  between the two estimates (Altman 2003).’  **Method to detect interactions reported in review:** Tests for subgroup differences were performed to evaluate differences  between estimates.  **Reason for changing mind:** NA. |
| Penninga 2013 | **Protocol:** ‘Studies with low risk of bias compared to studies with high risk of bias’.  **Review methods: ‘**Studies with low risk of bias compared to studies with high risk of bias’.  **Review results:** Not analysed.  **Type:** Methodological.  **Covariate summary:** Risk of bias. | **Method to choose**  **covariate:** Not reported.  **Where method reported:** NA.  **Rationale for choosing the covariate:** Not reported.  **Where rationale reported:** NA. | **Where reported:** Protocol and review.  **Reason not reported in review:** NA.  **Reason not reported in protocol:** NA.  **Labelled as ‘posthoc’ if not in protocol:** NA. | **Data type:** Categorical.  **Continuous covariate categorised:** NA.  **How determine it was categorised:** NA.  **Justification for categorising:** NA.  **Where justification reported:** NA.  **Categories reported:** Yes.  **Where categories reported:** Protocol and review.  **Justification for categories:** Not reported.  **Where justification reported:** NA. | **Analysis type reported in protocol:** Subgroup analysis.  **Analysis type reported in review:** Subgroup analysis.  **Reason for changing mind:** NA.    **Analysis type in protocol according to glossary:** Subgroup analysis.  **How determined the analysis type:** Categories given.  **Analysis type in review according to glossary:** Subgroup analysis.  **How determined the analysis type:** Categories given.  **Analysed:** No.  **Reasons given (in the review) for not doing interaction analysis if planned:** ‘We were unable to perform subgroup analyses of studies assessed  to be at low risk of bias compared to those at high risk of bias.  All included studies in this review were assessed to be at high risk  of bias.’  **Reasons given for carrying out interaction analysis if they weren’t planned:** NA.  **Scenario in protocol:** Not reported.  **Rationale for planned scenario:** NA..  **Scenario in review (if reported specifically):** Not reported.  **Rationale for done scenario:** NA.  **Reason for changing mind:** NA**.** | **Method to detect interactions reported in protocol: ‘**Test of interaction will be performed to evaluate the difference  between the two estimates (Altman 2003).’  **Method to detect interactions reported in review:** Tests for subgroup differences were performed to evaluate differences  between estimates.  **Reason for changing mind:** NA. |
| Peters 2013 | **Protocol:** ‘Other concomitant conditions. Subgroup analyses will be performed where participants with other concomitant conditions, such as metabolic and physiological conditions (rheumatic diseases, pregnancy, hypothyroidism), infections, neuropathies (for example, associated with diabetes mellitus or alcoholism), or space occupying conditions (tumours, fracture callus), are distinguished.’  **Review methods:** Not reported.  **Review results:** Not analysed.  **Type:** Patient.  **Covariate summary:** Disease characteristics. | **Method to choose**  **covariate:** Not reported.  **Where method reported:** NA.  **Rationale for choosing the covariate:** Not reported.  **Where rationale reported:** NA. | **Where reported:** Protocol only.  **Reason not reported in review:** Subgroup analyses were  amended to reflect current surgical interventions. ’Timing of rehabilitation’ and ’other concomitant conditions’ subgroup analyses were  removed from the review.  **Reason not reported in protocol:** NA.  **Labelled as ‘posthoc’ if not in protocol:** NA**.** | **Data type:** Categorical.  **Continuous covariate categorised:** NA.  **How determine it was categorised:** NA.  **Justification for categorising:** NA.  **Where justification reported:** NA.  **Categories reported:** Yes.  **Where categories reported:** Protocol only.  **Justification for categories:** Not reported.  **Where justification reported:** NA. | **Analysis type reported in protocol:** Subgroup analysis.  **Analysis type reported in review:** Not reported.  **Reason for changing mind:** Not reported.    **Analysis type in protocol according to glossary:** Subgroup analysis.  **How determined the analysis type:** Categories given.  **Analysis type in review according to glossary:** Not reported.  **How determined the analysis type:** NA.  **Analysed:** No.  **Reasons given (in the review) for not doing interaction analysis if planned:** ‘We could not perform any subgroup analyses in this review. Clinical  heterogeneity of interventions and outcomes or paucity of  the specified subgroups being distinguished by trialists meant that  these analyses were not possible.’ Subgroup analyses were  amended to reflect current surgical interventions. ’Timing of rehabilitation’ and ’other concomitant conditions’ subgroup analyses were  removed from the review.  **Reasons given for carrying out interaction analysis if they weren’t planned:** NA.  **Scenario in protocol:** Not reported.  **Rationale for planned scenario:** NA.  **Scenario in review (if reported specifically):** Not reported.  **Rationale for done scenario:** NA.  **Reason for changing mind:** NA**.** | **Method to detect interactions reported in protocol:** Not reported.  **Method to detect interactions reported in review:** Not reported.  **Reason for changing mind:** NA. |
| Peters 2013 | **Protocol:** ‘Severity of participants’ carpal tunnel symptoms. Subgroup analyses will be performed where participants with early (E), intermediate (I) and advanced (A) symptoms are distinguished. Early CTS is defined where intermittent symptoms, no motor impairment and normal electrophysiological tests are present. Intermediate CTS is defined where constant symptoms with abnormal electrophysiological tests are present, with or without motor impairment. Advanced symptoms are characterised by severe loss of sensory and motor functions, the presence of thenar muscle atrophy and weakness, and abnormal electrophysiological and sensory tests.’  **Review methods:** ‘Severity of CTS symptoms (Szabo 1994): • early CTS, defined as intermittent symptoms, no motor impairment and normal electrophysiological tests; • intermediate CTS, defined as constant symptoms with abnormal electrophysiological tests, with or without motor impairment; • advanced CTS, defined as severe loss of sensory and motor function, the presence of thenar muscle atrophy and weakness, and abnormal electrophysiological and sensory tests.’  **Review results:** Not analysed.  **Type:** Patient.  **Covariate summary:** Disease characteristics. | **Method to choose**  **covariate:** Not reported.  **Where method reported:** NA.  **Rationale for choosing the covariate:** Not reported.  **Where rationale reported:** NA. | **Where reported:** Protocol and review.  **Reason not reported in review:** NA.  **Reason not reported in protocol:** NA.  **Labelled as ‘posthoc’ if not in protocol:** NA**.** | **Data type:** Categorical.  **Continuous covariate categorised:** NA.  **How determine it was categorised:** NA.  **Justification for categorising:** NA.  **Where justification reported:** NA.  **Categories reported:** Yes.  **Where categories reported:** Protocol and review.  **Justification for categories:** Not reported.  **Where justification reported:** NA. | **Analysis type reported in protocol:** Subgroup analysis.  **Analysis type reported in review:** Subgroup analysis.  **Reason for changing mind:** NA.    **Analysis type in protocol according to glossary:** Subgroup analysis.  **How determined the analysis type:** Categories given.  **Analysis type in review according to glossary:** Subgroup analysis.  **How determined the analysis type:** Categories given.  **Analysed:** No.  **Reasons given (in the review) for not doing interaction analysis if planned:** ‘We could not perform any subgroup analyses in this review. Clinical  heterogeneity of interventions and outcomes or paucity of  the specified subgroups being distinguished by trialists meant that  these analyses were not possible.’  **Reasons given for carrying out interaction analysis if they weren’t planned:** NA.  **Scenario in protocol:** Not reported.  **Rationale for planned scenario:** NA.  **Scenario in review (if reported specifically):** Not reported.  **Rationale for done scenario:** NA.  **Reason for changing mind:** NA**.** | **Method to detect interactions reported in protocol:** Not reported.  **Method to detect interactions reported in review:** Not reported.  **Reason for changing mind:** NA. |
| Peters 2013 | **Protocol:** ‘Timing of rehabilitation. Subgroup analyses will be performed where the timing of provision of postoperative rehabilitation is described. Participants provided with rehabilitation starting within one week of surgery (A); between eight days and three weeks (B); and more than three weeks after surgery (C) will be distinguished.’  **Review methods:** Not reported.  **Review results:** Not analysed.  **Type:** Intervention.  **Covariate summary:** Timing of intervention. | **Method to choose**  **covariate:** Not reported.  **Where method reported:** NA.  **Rationale for choosing the covariate:** Not reported.  **Where rationale reported:** NA. | **Where reported:** Protocol only.  **Reason not reported in review: ‘**Subgroup analyses were  amended to reflect current surgical interventions. ’Timing of rehabilitation’ and ’other concomitant conditions’ subgroup analyses were  removed from the review.’  **Reason not reported in protocol:** NA.  **Labelled as ‘posthoc’ if not in protocol:** NA**.** | **Data type:** Continuous.  **Continuous covariate categorised:** Yes.  **How determine it was categorised:** Categories given.  **Justification for categorising:** Not reported.  **Where justification reported:** NA.  **Categories reported:** Yes.  **Where categories reported:** Protocol only.  **Justification for categories:** Not reported.  **Where justification reported:** NA. | **Analysis type reported in protocol:** Subgroup analysis.  **Analysis type reported in review:** Not reported.  **Reason for changing mind:** Not reported.    **Analysis type in protocol according to glossary:** Subgroup analysis.  **How determined the analysis type:** Categories given.  **Analysis type in review according to glossary:** Not reported.  **How determined the analysis type:** NA.  **Analysed:** No.  **Reasons given (in the review) for not doing interaction analysis if planned:** ‘We could not perform any subgroup analyses in this review. Clinical  heterogeneity of interventions and outcomes or paucity of  the specified subgroups being distinguished by trialists meant that  these analyses were not possible.’ ‘Subgroup analyses were  amended to reflect current surgical interventions. ’Timing of rehabilitation’ and ’other concomitant conditions’ subgroup analyses were  removed from the review.’  **Reasons given for carrying out interaction analysis if they weren’t planned:** NA.  **Scenario in protocol:** Not reported.  **Rationale for planned scenario:** NA.  **Scenario in review (if reported specifically):** Not reported.  **Rationale for done scenario:** NA.  **Reason for changing mind:** NA**.** | **Method to detect interactions reported in protocol:** Not reported.  **Method to detect interactions reported in review:** Not reported.  **Reason for changing mind:** NA. |
| Peters 2013 | **Protocol:** ‘Type of surgical intervention. Subgroup analyses will be performed where participants undergoing endoscopic (E), open (O), and other (O+) surgical intervention are distinguished.’  **Review methods:** ‘Type of surgical intervention: • endoscopic (either single portal or double portal); • open; • minimal-incision open; • another carpal tunnel surgery(including secondary procedures such as epineurotomy, internal neurolysis, synovectomy or reconstruction of the transverse carpal ligament).’  **Review results:** Not analysed.  **Type:** Intervention.  **Covariate summary:** Type of intervention or control. | **Method to choose**  **covariate:** Not reported.  **Where method reported:** NA.  **Rationale for choosing the covariate:** Not reported.  **Where rationale reported:** NA. | **Where reported:** Protocol and review.  **Reason not reported in review:** NA.  **Reason not reported in protocol:** NA.  **Labelled as ‘posthoc’ if not in protocol:** NA**.** | **Data type:** Categorical.  **Continuous covariate categorised:** NA.  **How determine it was categorised:** NA.  **Justification for categorising:** NA.  **Where justification reported:** NA.  **Categories reported:** Yes.  **Where categories reported:** Protocol and review.  **Justification for categories:** Not reported.  **Where justification reported:** NA. | **Analysis type reported in protocol:** Subgroup analysis.  **Analysis type reported in review:** Subgroup analysis.  **Reason for changing mind:** NA.    **Analysis type in protocol according to glossary:** Subgroup analysis.  **How determined the analysis type:** Categories given.  **Analysis type in review according to glossary:** Subgroup analysis.  **How determined the analysis type:** Categories given.  **Analysed:** No.  **Reasons given (in the review) for not doing interaction analysis if planned:** ‘We could not perform any subgroup analyses in this review. Clinical  heterogeneity of interventions and outcomes or paucity of  the specified subgroups being distinguished by trialists meant that  these analyses were not possible.  .’  **Reasons given for carrying out interaction analysis if they weren’t planned:** NA.  **Scenario in protocol:** Not reported.  **Rationale for planned scenario:** NA.  **Scenario in review (if reported specifically):** Not reported.  **Rationale for done scenario:** NA.  **Reason for changing mind:** NA**.** | **Method to detect interactions reported in protocol:** Not reported.  **Method to detect interactions reported in review:** Not reported.  **Reason for changing mind:** NA. |
| Peters 2013 | **Protocol:** Allocation concealment. We judged the following to be three covariates: **‘**Methodological quality of trials. Sensitivity analyses will be performed when (i) allocation concealment is rated as inadequate, not used or unclear (and attempts to clarify with authors fail) (A); (ii) blinding of outcome assessment is not done or is rated as unclear (and attempts to clarify with authors fail) (B); and (iii) intention-to-treat analysis is not performed or is unclear (and attempts to clarify with authors fail) (C). These quality criteria have been shown to influence estimates of treatment effect (Juni 2001). Sensitivity analyses will be performed in which A, B or C are excluded.’  **Review methods:** We judged the following to be three covariates: ‘We used predetermined sensitivity analyses to assess the effect of excluding studies when (a) allocation concealment was rated as inadequate, not used or unclear (and attempts to clarify with authors failed); (b) blinding of outcome assessment was not done (reason) or was rated as unclear (and attempts to clarify with authors failed); and (c) intention-to-treat analysis was not performed or was unclear (and attempts to clarify with authors failed). These quality criteria have been shown to influence estimates of treatment effects (Jüni 2001).’  **Review results:** Not analysed.  **Type:** Methodological.  **Covariate summary:** Allocation concealment. | **Method to choose**  **covariate:** Not reported.  **Where method reported:** NA.  **Rationale for choosing the covariate:** ‘These  quality criteria have been shown to influence estimates of treatment effect (Juni 2001).’  **Where rationale reported:** Protocol and review. | **Where reported:** Protocol and review.  **Reason not reported in review:** NA.  **Reason not reported in protocol:** NA.  **Labelled as ‘posthoc’ if not in protocol:** NA**.** | **Data type:** Categorical.  **Continuous covariate categorised:** NA.  **How determine it was categorised:** NA.  **Justification for categorising:** NA.  **Where justification reported:** NA.  **Categories reported:** Yes.  **Where categories reported:** Protocol and review.  **Justification for categories:** Not reported.  **Where justification reported:** NA. | **Analysis type reported in protocol:** Sensitivity analysis.  **Analysis type reported in review:** Sensitivity analysis.  **Reason for changing mind:** NA.    **Analysis type in protocol according to glossary:** Sensitivity analysis.  **How determined the analysis type:** Categories given.  **Analysis type in review according to glossary:** Sensitivity analysis.  **How determined the analysis type:** Categories given.  **Analysed:** No.  **Reasons given (in the review) for not doing interaction analysis if planned:** ‘Furthermore, sensitivity analyses were not performed as there were no meta-analyses.’  **Reasons given for carrying out interaction analysis if they weren’t planned:** NA.  **Scenario in protocol:** Not reported.  **Rationale for planned scenario:** NA.  **Scenario in review (if reported specifically):** Not reported.  **Rationale for done scenario:** NA.  **Reason for changing mind:** NA**.** | **Method to detect interactions reported in protocol:** Not reported.  **Method to detect interactions reported in review:** Not reported.  **Reason for changing mind:** NA. |
| Peters 2013 | **Protocol:** Blinding of outcome assessment. We judged the following to be three covariates: **‘**Methodological quality of trials. Sensitivity analyses will be performed when (i) allocation concealment is rated as inadequate, not used or unclear (and attempts to clarify with authors fail) (A); (ii) blinding of outcome assessment is not done or is rated as unclear (and attempts to clarify with authors fail) (B); and (iii) intention-to-treat analysis is not performed or is unclear (and attempts to clarify with authors fail) (C). These quality criteria have been shown to influence estimates of treatment effect (Juni 2001). Sensitivity analyses will be performed in which A, B or C are excluded.’  **Review methods:** We judged the following to be three covariates: ‘We used predetermined sensitivity analyses to assess the effect of excluding studies when (a) allocation concealment was rated as inadequate, not used or unclear (and attempts to clarify with authors failed); (b) blinding of outcome assessment was not done (reason) or was rated as unclear (and attempts to clarify with authors failed); and (c) intention-to-treat analysis was not performed or was unclear (and attempts to clarify with authors failed). These quality criteria have been shown to influence estimates of treatment effects (Jüni 2001).’  **Review results:** Not analysed.  **Type:** Methodological.  **Covariate summary:** Blinding. | **Method to choose**  **covariate:** Not reported.  **Where method reported:** NA.  **Rationale for choosing the covariate:** ‘These  quality criteria have been shown to influence estimates of treatment effect (Juni 2001).’  **Where rationale reported:** Protocol and review. | **Where reported:** Protocol and review.  **Reason not reported in review:** NA.  **Reason not reported in protocol:** NA.  **Labelled as ‘posthoc’ if not in protocol:** NA**.** | **Data type:** Categorical.  **Continuous covariate categorised:** NA.  **How determine it was categorised:** NA.  **Justification for categorising:** NA.  **Where justification reported:** NA.  **Categories reported:** Yes.  **Where categories reported:** Protocol and review.  **Justification for categories:** Not reported.  **Where justification reported:** NA. | **Analysis type reported in protocol:** Sensitivity analysis.  **Analysis type reported in review:** Sensitivity analysis.  **Reason for changing mind:** NA.    **Analysis type in protocol according to glossary:** Sensitivity analysis.  **How determined the analysis type:** Categories given.  **Analysis type in review according to glossary:** Sensitivity analysis.  **How determined the analysis type:** Categories given.  **Analysed:** No.  **Reasons given (in the review) for not doing interaction analysis if planned:** ‘Furthermore, sensitivity analyses  were not performed as there were no meta-analyses.’  **Reasons given for carrying out interaction analysis if they weren’t planned:** NA.  **Scenario in protocol:** Not reported.  **Rationale for planned scenario:** NA.  **Scenario in review (if reported specifically):** Not reported.  **Rationale for done scenario:** NA.  **Reason for changing mind:** NA**.** | **Method to detect interactions reported in protocol:** Not reported.  **Method to detect interactions reported in review:** Not reported.  **Reason for changing mind:** NA. |
| Peters 2013 | **Protocol:** Intention-to-treat analysis. We judged the following to be three covariates: **‘**Methodological quality of trials.Sensitivity analyses will be performed when (i) allocation concealment is rated as inadequate, not used or unclear (and attempts to clarify with authors fail) (A); (ii) blinding of outcome assessment is not done or is rated as unclear (and attempts to clarify with authors fail) (B); and (iii) intention-to-treat analysis is not performed or is unclear (and attempts to clarify with authors fail) (C). These quality criteria have been shown to influence estimates of treatment effect (Juni 2001). Sensitivity analyses will be performed in which A, B or C are excluded.’  **Review methods:** We judged the following to be three covariates: ‘We used predetermined sensitivity analyses to assess the effect of excluding studies when (a) allocation concealment was rated as inadequate, not used or unclear (and attempts to clarify with authors failed); (b) blinding of outcome assessment was not done (reason) or was rated as unclear (and attempts to clarify with authors failed); and (c) intention-to-treat analysis was not performed or was unclear (and attempts to clarify with authors failed). These quality criteria have been shown to influence estimates of treatment effects (Jüni 2001).’  **Review results:** Not analysed.  **Type:** Methodological.  **Covariate summary:** Intention to treat. | **Method to choose**  **covariate:** Not reported.  **Where method reported:** NA.  **Rationale for choosing the covariate:** ‘These  quality criteria have been shown to influence estimates of treatment effect (Juni 2001).’  **Where rationale reported:** Protocol and review. | **Where reported:** Protocol and review.  **Reason not reported in review:** NA.  **Reason not reported in protocol:** NA.  **Labelled as ‘posthoc’ if not in protocol:** NA**.** | **Data type:** Categorical.  **Continuous covariate categorised:** NA.  **How determine it was categorised:** NA.  **Justification for categorising:** NA.  **Where justification reported:** NA.  **Categories reported:** Yes.  **Where categories reported:** Protocol and review.  **Justification for categories:** Not reported.  **Where justification reported:** NA. | **Analysis type reported in protocol:** Sensitivity analysis.  **Analysis type reported in review:** Sensitivity analysis.  **Reason for changing mind:** NA.    **Analysis type in protocol according to glossary:** Sensitivity analysis.  **How determined the analysis type:** Categories given.  **Analysis type in review according to glossary:** Sensitivity analysis.  **How determined the analysis type:** Categories given.  **Analysed:** No.  **Reasons given (in the review) for not doing interaction analysis if planned:** ‘Furthermore, sensitivity analyses were not performed as there were no meta-analyses.’  **Reasons given for carrying out interaction analysis if they weren’t planned:** NA.  **Scenario in protocol:** Not reported.  **Rationale for planned scenario:** NA.  **Scenario in review (if reported specifically):** Not reported.  **Rationale for done scenario:** NA.  **Reason for changing mind:** NA**.** | **Method to detect interactions reported in protocol:** Not reported.  **Method to detect interactions reported in review:** Not reported.  **Reason for changing mind:** NA. |
| Peters 2013 | **Protocol:** Not reported.  **Review methods:** Not reported.  **Review results:** Comparison. In plot: ‘1. Low-level laser versus placebo’; ‘2. Immobilisation (wrist splint) versus mobilisation (bulky dressing)’; ‘3. Immobilisation (plaster of Paris splint) versus bulky dressing and mobilisation’; ‘4. Specialised home elevation device versus standard sling’; ‘5. Controlled cold therapy versus ice therapy’; ‘6. Bulky dressing plus splint versus light dressing’; ‘7. Contrast bath plus exercise versus contrast bath’; ‘8. Contrast bath plus exercises versus exercise’; ‘9. Contrast bath versus exercise’; ‘10. Arnica versus placebo’; ‘11. High dose arnica oral tablets versus placebo’; ‘12. Low dose arnica tablets versus placebo’; ‘13. High dose versus low dose oral arnica tablets’; ‘14. Multimodal hand therapy versus normal activities/exercise’; ‘15. Desensitisation therapy (as part of multiple interventions) versus no treatment’; ‘16. Short duration dressing versus extended duration dressing. In text: Low-level laser therapy (single intervention) versus “sham” therapy (placebo)’; ‘Immobilisation (single intervention) versus bulky dressing (single intervention)’; ‘Immobilisation (single intervention) versus mobilisation (multiple interventions)’; ‘Elevation (single intervention) versus standard care (control)’; ‘Electrical stimulation (single intervention) versus no treatment (control)’; ‘Controlled cold therapy and narcotic use (multiple interventions) versus ice therapy plus narcotic use (multiple interventions)’; ‘Bulky dressing and splint (single intervention) versus light dressing (single intervention)’; ‘Contrast baths plus exercise (multiple interventions) versus contrast baths (single intervention) versus exercise (single intervention)’; ‘Arnica (single intervention) versus “sham” therapy (placebo)’; ‘High dose arnica (single intervention) versus low dose arnica (single intervention) versus “sham” therapy (placebo)’; ‘Electrical stimulation versus decimer wave therapy versus combined therapy versus control’; ‘Multimodal hand therapy (multiple interventions) versus no formal therapy (control)’; ‘Desensitisation therapy (as part of multiple interventions) versus standard treatment (control)’; ‘Multimodal therapy (multiple interventions) versus progressive patient-directed home exercise program (single intervention)’; ‘Short duration postoperative dressing (single intervention) versus extended duration postoperative dressing (single intervention)’.  **Type:** Intervention.  **Covariate summary:** Type of intervention or control. | **Method to choose**  **covariate:** Not reported.  **Where method reported:** NA.  **Rationale for choosing the covariate:** Not reported.  **Where rationale reported:** NA. | **Where reported:** Review only.  **Reason not reported in review:** NA.  **Reason not reported in protocol:** Not reported.  **Labelled as ‘posthoc’ if not in protocol:** No**.** | **Data type:** Categorical.  **Continuous covariate categorised:** NA.  **How determine it was categorised:** NA.  **Justification for categorising:** NA.  **Where justification reported:** NA.  **Categories reported:** Yes.  **Where categories reported:** Review only.  **Justification for categories:** Not reported.  **Where justification reported:** NA. | **Analysis type reported in protocol:** Not reported.  **Analysis type reported in review:** Unnamed analysis.  **Reason for changing mind:** Not reported.    **Analysis type in protocol according to glossary:** Not reported.  **How determined the analysis type:** NA.  **Analysis type in review according to glossary:** Stratification/subgroup analysis.  **How determined the analysis type:** Categories given.  **Analysed:** Yes.  **Reasons given (in the review) for not doing interaction analysis if planned:** NA.  **Reasons given for carrying out interaction analysis if they weren’t planned:** Not reported.  **Scenario in protocol:** Not reported.  **Rationale for planned scenario:** NA.  **Scenario in review (if reported specifically):** Not reported.  **Rationale for done scenario:** NA.  **Reason for changing mind:** NA**.** | **Method to detect interactions reported in protocol:** Not reported.  **Method to detect interactions reported in review:** Not reported.  **Reason for changing mind:** NA. |
| Peters 2013 | **Protocol:** Not reported.  **Review methods:** Not reported.  **Review results:** Time point. 3, 6, 12 months.  **Type:** Outcome.  **Covariate summary:** Time point. | **Method to choose**  **covariate:** Not reported.  **Where method reported:** NA.  **Rationale for choosing the covariate:** Not reported.  **Where rationale reported:** NA. | **Where reported:** Review only.  **Reason not reported in review:** NA.  **Reason not reported in protocol:** Not reported.  **Labelled as ‘posthoc’ if not in protocol:** No**.** | **Data type:** Continuous.  **Continuous covariate categorised:** Yes.  **How determine it was categorised:** Categories given.  **Justification for categorising:** Not reported.  **Where justification reported:** NA.  **Categories reported:** Yes.  **Where categories reported:** Review only.  **Justification for categories:** Not reported.  **Where justification reported:** NA. | **Analysis type reported in protocol:** Not reported.  **Analysis type reported in review:** Unnamed analysis.  **Reason for changing mind:** Not reported.    **Analysis type in protocol according to glossary:** Not reported.  **How determined the analysis type:** NA.  **Analysis type in review according to glossary:** Stratification/subgroup analysis.  **How determined the analysis type:** Categories given.  **Analysed:** Yes.  **Reasons given (in the review) for not doing interaction analysis if planned:** NA.  **Reasons given for carrying out interaction analysis if they weren’t planned:** Not reported.  **Scenario in protocol:** Not reported.  **Rationale for planned scenario:** NA.  **Scenario in review (if reported specifically):** Not reported.  **Rationale for done scenario:** NA.  **Reason for changing mind:** NA**.** | **Method to detect interactions reported in protocol:** Not reported.  **Method to detect interactions reported in review:** Not reported.  **Reason for changing mind:** NA. |
| Peters 2013 | **Protocol:** Quality of diagnostic criteria (B and C are excluded). We judged the following to be two covariates: ‘Trials will be classified into high (A), moderate (B) and low (C) quality, according to criteria proposed by Rempel and colleagues (Rempel 1998). Sensitivity analyses will be performed in which C is excluded and when B and C are excluded.’  **Review methods:** Not reported.  **Review results:** Not analysed.  **Type:** Methodological.  **Covariate summary:** Quality of diagnostic criteria. | **Method to choose**  **covariate:** Not reported.  **Where method reported:** NA.  **Rationale for choosing the covariate:** Not reported.  **Where rationale reported:** NA. | **Where reported:** Protocol only.  **Reason not reported in review:** Not reported.  **Reason not reported in protocol:** NA.  **Labelled as ‘posthoc’ if not in protocol:** NA**.** | **Data type:** Categorical.  **Continuous covariate categorised:** NA.  **How determine it was categorised:** NA.  **Justification for categorising:** NA.  **Where justification reported:** NA.  **Categories reported:** Yes.  **Where categories reported:** Protocol only.  **Justification for categories:** Not reported.  **Where justification reported:** NA. | **Analysis type reported in protocol:** Sensitivity analysis.  **Analysis type reported in review:** Not reported.  **Reason for changing mind:** Not reported.    **Analysis type in protocol according to glossary:** Sensitivity analysis.  **How determined the analysis type:** Categories given.  **Analysis type in review according to glossary:** Not reported  **How determined the analysis type:** NA.  **Analysed:** No.  **Reasons given (in the review) for not doing interaction analysis if planned:** ‘Furthermore, sensitivity analyses were not performed as there were no meta-analyses.’  **Reasons given for carrying out interaction analysis if they weren’t planned:** NA.  **Scenario in protocol:** Not reported.  **Rationale for planned scenario:** NA.  **Scenario in review (if reported specifically):** Not reported.  **Rationale for done scenario:** NA.  **Reason for changing mind:** NA**.** | **Method to detect interactions reported in protocol:** Not reported.  **Method to detect interactions reported in review:** Not reported.  **Reason for changing mind:** NA. |
| Peters 2013 | **Protocol:** Quality of diagnostic criteria (C is excluded). We judged the following to be two covariates: ‘Trials will be classified into high (A), moderate (B) and low (C) quality, according to criteria proposed by Rempel and colleagues (Rempel 1998). Sensitivity analyses will be performed in which C is excluded and when B and C are excluded.’  **Review methods:** Not reported.  **Review results:** Not analysed.  **Type:** Methodological.  **Covariate summary:** Quality of diagnostic criteria. | **Method to choose**  **covariate:** Not reported.  **Where method reported:** NA.  **Rationale for choosing the covariate:** Not reported.  **Where rationale reported:** NA. | **Where reported:** Protocol only.  **Reason not reported in review:** Not reported.  **Reason not reported in protocol:** NA.  **Labelled as ‘posthoc’ if not in protocol:** NA**.** | **Data type:** Categorical.  **Continuous covariate categorised:** NA.  **How determine it was categorised:** NA.  **Justification for categorising:** NA.  **Where justification reported:** NA.  **Categories reported:** Yes.  **Where categories reported:** Protocol only.  **Justification for categories:** Not reported.  **Where justification reported:** NA. | **Analysis type reported in protocol:** Sensitivity analysis.  **Analysis type reported in review:** Not reported.  **Reason for changing mind:** Not reported.    **Analysis type in protocol according to glossary:** Sensitivity analysis.  **How determined the analysis type:** Categories given.  **Analysis type in review according to glossary:** Not reported  **How determined the analysis type:** NA.  **Analysed:** No.  **Reasons given (in the review) for not doing interaction analysis if planned:** ‘Furthermore, sensitivity analyses were not performed as there were no meta-analyses.’  **Reasons given for carrying out interaction analysis if they weren’t planned:** NA.  **Scenario in protocol:** Not reported.  **Rationale for planned scenario:** NA.  **Scenario in review (if reported specifically):** Not reported.  **Rationale for done scenario:** NA.  **Reason for changing mind:** NA**.** | **Method to detect interactions reported in protocol:** Not reported.  **Method to detect interactions reported in review:** Not reported.  **Reason for changing mind:** NA. |
| Rockers 2013 | **Protocol:** ‘We will also investigate the effects of including and excluding studies where there is some ambiguity as to whether they meet the inclusion criteria.’  **Review methods:** Not reported.  **Review results:** Not analysed.  **Type:** Patient.  **Covariate summary:** Inclusion criteria. | **Method to choose**  **covariate:** Not reported.  **Where method reported:** NA.  **Rationale for choosing the covariate:** Not reported.  **Where rationale reported:** NA. | **Where reported:** Protocol only.  **Reason not reported in review:** Not reported.  **Reason not reported in protocol:** NA.  **Labelled as ‘posthoc’ if not in protocol:** NA. | **Data type:** Categorical.  **Continuous covariate categorised:** NA.  **How determine it was categorised:** NA.  **Justification for categorising:** NA.  **Where justification reported:** NA.  **Categories reported:** Yes.  **Where categories reported:** Protocol only  **Justification for categories:** Not reported.  **Where justification reported:** NA. | **Analysis type reported in protocol:** Sensitivity analysis.  **Analysis type reported in review:** Not reported.  **Reason for changing mind:** Not reported.    **Analysis type in protocol according to glossary:** Sensitivity analysis.  **How determined the analysis type:** ‘including and excluding’.  **Analysis type in review according to glossary:** Not reported.  **How determined the analysis type:** NA.  **Analysed:** No.  **Reasons given (in the review) for not doing interaction analysis if planned: ‘**We did not conduct meta-analysis or subgroup analysis, because we could not pool study results due to the varied nature of the interventions included in this review.’  **Reasons given for carrying out interaction analysis if they weren’t planned:** NA.  **Scenario in protocol:** ‘If included studies allow for a meta-analysis, we will conduct sensitivity analyses on all pooled effect estimates.’  **Rationale for planned scenario:** Not reported.  **Scenario in review (if reported specifically):** Not reported.  **Rationale for done scenario:** NA.  **Reason for changing mind:** Not reported. | **Method to detect interactions reported in protocol:** Not reported.  **Method to detect interactions reported in review:** Not reported.  **Reason for changing mind:** NA. |
| Rockers 2013 | **Protocol:** Not reported.  **Review methods:** Not reported.  **Review results:** ‘Hiring and retaining’; ‘training’.  **Type:** Intervention.  **Covariate summary:** Type of intervention or control. | **Method to choose**  **covariate:** Not reported.  **Where method reported:** NA.  **Rationale for choosing the covariate:** Not reported.  **Where rationale reported:** NA. | **Where reported:** Review only.  **Reason not reported in review:** NA.  **Reason not reported in protocol:** Not reported.  **Labelled as ‘posthoc’ if not in protocol:** No. | **Data type:** Categorical.  **Continuous covariate categorised:** NA.  **How determine it was categorised:** NA.  **Justification for categorising:** NA.  **Where justification reported:** NA.  **Categories reported:** Yes.  **Where categories reported:** Review only.  **Justification for categories:** Not reported.  **Where justification reported:** NA. | **Analysis type reported in protocol:** Not reported.  **Analysis type reported in review:** Unnamed analysis.  **Reason for changing mind:** Not reported.    **Analysis type in protocol according to glossary:** Not reported.  **How determined the analysis type:** NA.  **Analysis type in review according to glossary:** Stratification/subgroup analysis.  **How determined the analysis type:** Results text stratified.  **Analysed:** Yes.  **Reasons given (in the review) for not doing interaction analysis if planned:** NA.  **Reasons given for carrying out interaction analysis if they weren’t planned:** Not reported.  **Scenario in protocol:** Not reported.  **Rationale for planned scenario:** NA.  **Scenario in review (if reported specifically):** Not reported.  **Rationale for done scenario:** NA.  **Reason for changing mind:** Not reported. | **Method to detect interactions reported in protocol:** Not reported.  **Method to detect interactions reported in review:** Not reported.  **Reason for changing mind:** NA. |
| Sajid, 2012 | **Protocol:** ‘Repeating the analysis each time excluding unpublished studies.’  **Review methods:** Not reported.  **Review results:** Not analysed.  **Type:** Methodological.  **Covariate summary:** Publication status. | **Method to choose**  **covariate:** Not reported.  **Where method reported:** NA.  **Rationale for choosing the covariate:** Not reported.  **Where rationale reported:** NA. | **Where reported:** Protocol only.  **Reason not reported in review:** Not reported.  **Reason not reported in protocol:** NA.  **Labelled as ‘posthoc’ if not in protocol:** NA. | **Data type:** Categorical.  **Continuous covariate categorised:** NA.  **How determine it was categorised:** NA.  **Justification for categorising:** NA.  **Where justification reported:** NA.  **Categories reported:** Yes.  **Where categories reported:** Protocol only.  **Justification for categories:** Not reported.  **Where justification reported:** NA. | **Analysis type reported in protocol:** Sensitivity analysis.  **Analysis type reported in review:** Not reported.  **Reason for changing mind:** Not reported.    **Analysis type in protocol according to glossary:** Sensitivity analysis.  **How determined the analysis type:** Categories given.  **Analysis type in review according to glossary:** Not reported.  **How determined the analysis type:** NA.  **Analysed:** No.  **Reasons given (in the review) for not doing interaction analysis if planned:** ‘In addition, there were no unpublished  studies. Therefore, authors were unable to perform subgroup analyses on both groups.’  **Reasons given for carrying out interaction analysis if they weren’t planned:** NA.  **Scenario in protocol:** ‘if adequate data are available’  **Rationale for planned scenario:** Not reported.  **Scenario in review (if reported specifically):** Not reported.  **Rationale for done scenario:** Not reported.  **Reason for changing mind:** Not reported. | **Method to detect interactions reported in protocol:** Not reported.  **Method to detect interactions reported in review:** Not reported.  **Reason for changing mind:** NA. |
| Sajid, 2012 | **Protocol:** ‘Repeating the analysis excluding studies with high risk of bias.’  **Review methods: ‘**Repeating the analysis excluding studies with high risk of bias.’  **Review results:** Not analysed.  **Type:** Methodological.  **Covariate summary:** Risk of bias. | **Method to choose**  **covariate:** Not reported.  **Where method reported:** NA.  **Rationale for choosing the covariate:** Not reported.  **Where rationale reported:** NA. | **Where reported:** Protocol and review.  **Reason not reported in review:** NA.  **Reason not reported in protocol:** NA.  **Labelled as ‘posthoc’ if not in protocol:** NA. | **Data type:** Categorical.  **Continuous covariate categorised:** NA.  **How determine it was categorised:** NA.  **Justification for categorising:** NA.  **Where justification reported:** NA.  **Categories reported:** Yes.  **Where categories reported:** Protocol and review.  **Justification for categories:** Not reported.  **Where justification reported:** NA. | **Analysis type reported in protocol:** Sensitivity analysis.  **Analysis type reported in review:** Sensitivity analysis.  **Reason for changing mind:** NA.    **Analysis type in protocol according to glossary:** Sensitivity analysis.  **How determined the analysis type:** Categories given.  **Analysis type in review according to glossary:** Sensitivity analysis.  **How determined the analysis type:** Categories given.  **Analysed:** No.  **Reasons given (in the review) for not doing interaction analysis if planned:** Not reported.  **Reasons given for carrying out interaction analysis if they weren’t planned:** NA.  **Scenario in protocol:** ‘if adequate data are available’  **Rationale for planned scenario:** Not reported.  **Scenario in review (if reported specifically):** ‘when adequate data were available’  **Rationale for done scenario:** Not reported.  **Reason for changing mind:** NA. | **Method to detect interactions reported in protocol:** Not reported.  **Method to detect interactions reported in review:** Not reported.  **Reason for changing mind:** NA. |
| Sajid, 2012 | **Protocol: ‘**We will also attempt to analyse any difference between various groups of patients who have undergone simple mastectomy, mastectomy and axillary dissection, axillary  surgery only and redo breast or axillary surgery.’  **Review methods:** ‘We performed the subgroup analysis on trials in breast surgery and breast plus axillary surgery to find out if there was any difference depending upon the site of surgery  for breast cancer.’  **Review results:** ‘All trials analysis’; ‘Subgroup analysis of trials on mastectomy’; ‘Subgroup analysis of trials on mastectomy plus axillary surgery’.  **Type:** Patient.  **Covariate summary:** Disease characteristics. | **Method to choose**  **covariate:** Not reported.  **Where method reported:** NA.  **Rationale for choosing the covariate:** Not reported.  **Where rationale reported:** NA. | **Where reported:** Protocol and review.  **Reason not reported in review:** NA.  **Reason not reported in protocol:** NA.  **Labelled as ‘posthoc’ if not in protocol:** NA. | **Data type:** Categorical.  **Continuous covariate categorised:** NA.  **How determine it was categorised:** NA.  **Justification for categorising:** NA.  **Where justification reported:** NA.  **Categories reported:** Yes.  **Where categories reported:** Protocol and review.  **Justification for categories:** Not reported.  **Where justification reported:** NA. | **Analysis type reported in protocol:** Subgroup analysis.  **Analysis type reported in review:** Subgroup analysis.  **Reason for changing mind:** NA.    **Analysis type in protocol according to glossary:** Subgroup analysis.  **How determined the analysis type:** Categories given.  **Analysis type in review according to glossary:** Subgroup analysis  **How determined the analysis type:** Categories given.  **Analysed:** Yes.  **Reasons given (in the review) for not doing interaction analysis if planned:** NA.  **Reasons given for carrying out interaction analysis if they weren’t planned:** NA.  **Scenario in protocol:** Not reported.  **Rationale for planned scenario:** NA.  **Scenario in review (if reported specifically):** Not reported.  **Rationale for done scenario:** NA.  **Reason for changing mind:** Not reported. | **Method to detect interactions reported in protocol:** Not reported.  **Method to detect interactions reported in review:** Not reported.  **Reason for changing mind:** NA. |
| Sajid, 2012 | **Protocol:** ‘We will attempt to study any difference between trials with short term follow-up (a year or less than one year) and long-term follow up (more than one year).’  **Review methods:** ‘Duration of follow up.’  **Review results:** Not analysed.  **Type:** Methodological.  **Covariate summary:** Length of follow up. | **Method to choose**  **covariate:** Not reported.  **Where method reported:** NA.  **Rationale for choosing the covariate:** Not reported.  **Where rationale reported:** NA. | **Where reported:** Protocol and review.  **Reason not reported in review:** NA.  **Reason not reported in protocol:** NA.  **Labelled as ‘posthoc’ if not in protocol:** NA. | **Data type:** Continuous.  **Continuous covariate categorised:** Yes.  **How determine it was categorised:** Categories given.  **Justification for categorising:** Not reported.  **Where justification reported:** NA.  **Categories reported:** Yes.  **Where categories reported:** Protocol only.  **Justification for categories:** Not reported.  **Where justification reported:** NA. | **Analysis type reported in protocol:** Subgroup analysis.  **Analysis type reported in review:** Subgroup analysis.  **Reason for changing mind:** NA.    **Analysis type in protocol according to glossary:** Subgroup analysis.  **How determined the analysis type:** Categories given.  **Analysis type in review according to glossary:** Subgroup analysis.  **How determined the analysis type:** Says ‘subgroup analysis’. No reason to dispute.  **Analysed:** No.  **Reasons given (in the review) for not doing interaction analysis if planned:** Based on the duration of follow-up there were insufficient data to  perform subgroup analyses.  **Reasons given for carrying out interaction analysis if they weren’t planned:** NA.  **Scenario in protocol:** Not reported.  **Rationale for planned scenario:** NA.  **Scenario in review (if reported specifically):** Not reported.  **Rationale for done scenario:** NA.  **Reason for changing mind:** Not reported. | **Method to detect interactions reported in protocol:** Not reported.  **Method to detect interactions reported in review:** Not reported.  **Reason for changing mind:** NA. |
| Sampson 2013 | **Protocol: ‘**Outcomes were grouped into the short term (up to 12 weeks), medium term (13 to 26 weeks) and long term (over 26 weeks).’  **Review methods:** ‘Data were sub-grouped according to duration of follow-up (short term, medium term or long term).’  **Review results: ‘**short term (≤12 weeks)’; ‘medium term (13-25 weeks)’; ‘long term (≥26 weeks)’.  **Type:** Outcome.  **Covariate summary:** Time point. | **Method to choose**  **covariate:** Not reported.  **Where method reported:** NA.  **Rationale for choosing the covariate:** Not reported.  **Where rationale reported:** NA. | **Where reported:** Protocol and review.  **Reason not reported in review:** NA.  **Reason not reported in protocol:** NA.  **Labelled as ‘posthoc’ if not in protocol:** NA**.** | **Data type:** Continuous.  **Continuous covariate categorised:** Yes.  **How determine it was categorised:** Categories given.  **Justification for categorising:** Not reported.  **Where justification reported:** NA.  **Categories reported:** Yes  **Where categories reported:** Protocol and review.  **Justification for categories:** Not reported.  **Where justification reported:** NA. | **Analysis type reported in protocol:** Unnamed analysis.  **Analysis type reported in review:** Subgroup analysis.  **Reason for changing mind:** Not reported.    **Analysis type in protocol according to glossary:** Subgroup analysis.  **How determined the analysis type:** Categories given.  **Analysis type in review according to glossary:** Subgroup analysis.  **How determined the analysis type:** Categories given.  **Analysed:** Yes.  **Reasons given (in the review) for not doing interaction analysis if planned:** NA.  **Reasons given for carrying out interaction analysis if they weren’t planned:** NA.  **Scenario in protocol:** Not reported.  **Rationale for planned scenario:** NA.  **Scenario in review (if reported specifically):** Not reported.  **Rationale for done scenario:** NA.  **Reason for changing mind:** NA. | **Method to detect interactions reported in protocol:** Not reported.  **Method to detect interactions reported in review:** Not reported.  **Reason for changing mind:** NA. |
| Sampson 2013 | **Protocol:** ‘We carried out a subgroup analysis to compare results between different interventions, as defined in ’Types of interventions’. 1.’Prodrome-based intervention’ defined as treatment given on the early signs of relapse. 2.’Crisis intervention’ defined as treatment given only in case of full relapse and discontinued again after re-stabilization. 3.’Gradually increased drug-free period’ defined as increasing the cessation period of the treatment constantly. 4.’Drug Holidays’ defined as stopping the medication for fixed periods, and then reintroducing it (repeating thismore than once). 5. Maintenance therapy, as defined by the treating physician.’  **Review methods:** Not reported.  **Review results:** ‘Comparison 1. Any intermittent drug technique versus maintenance therapy’; ‘Comparison 2. Intermittent (early-based) versus maintenance therapy’; ‘Comparison 3. Intermittent (crisis intervention) versus maintenance therapy’; ‘Comparison 4. Intermittent (gradually increased drug-free periods) versus maintenance therapy’; ‘Comparison 5. Intermittent (drug holiday) versus maintenance therapy’; ‘Comparison 6. Any intermittent drug technique versus placebo’; ‘Comparison 7. Any intermittent drug technique (specific drug) versus maintenance therapy (specific drug): intermittent fluphenazine decanoate (low dose) vs maintained fluphenazine decanoate (moderate dose) -short term (≤12 weeks)’; ‘Comparison 7. Any intermittent drug technique (specific drug) versus maintenance therapy (specific drug)): intermittent haloperidol equivalents (low dose) vs maintained haloperidol equivalents (low dose) – short term (≤12 weeks)’; ‘Comparison 7. Any intermittent drug technique (specific drug) versus maintenance therapy (specific drug)): various intermittent typical antipsychotics (moderate dose) vs maintained typical antipsychotics (moderate dose) - short term (≤12 weeks)’; ‘Comparison 7. Any intermittent drug technique (specific drug) versus maintenance therapy (specific drug)): intermittent haloperidol equivalents (low dose) vs maintained haloperidol equivalents (low dose) -medium term (13-25 weeks)’; ‘Comparison 7. Any intermittent drug technique (specific drug) versus maintenance therapy (specific drug)): atypical antipsychotics (dosage unclear) vs maintained atypical/typical antipsychotics (dosage unclear) – medium term (13-25 weeks)’; ‘Comparison 7. Any intermittent drug technique (specific drug) versus maintenance therapy (specific drug)): various intermittent typical antipsychotics (low dose) vs maintained typical antipsychotics (low dose) -medium term (13-25 weeks)’; ‘Comparison 7. Any intermittent drug technique (specific drug) versus maintenance therapy (specific drug)): various intermittent typical antipsychotics (moderate dose) vs maintained typical antipsychotics (moderate dose) – medium term (13-25 weeks)’; ‘Comparison 7. Any intermittent drug technique (specific drug) versus maintenance therapy (specific drug)): intermittent chlorpromazine equivalents (low dose) vs maintained chlorpromazine equivalents (low dose) - long term (≥26 weeks)’; ‘Comparison 7. Any intermittent drug technique (specific drug) versus maintenance therapy (specific drug)): intermittent clozapine (low dose) vs maintained clozapine (low dose) long term (≥26 weeks)’; ‘Comparison 7. Any intermittent drug technique (specific drug) versus maintenance therapy (specific drug)): intermittent haloperidol equivalents (low dose) vs maintained haloperidol equivalents (low dose) – long term (≥26 weeks)’; ‘Comparison 7. Any intermittent drug technique (specific drug) versus maintenance therapy (specific drug)): intermittent pimozide (high dose) vs fluphenazine decanoate (low dose) – long term (≥26 weeks)’; ‘Comparison 7. Any intermittent drug technique (specific drug) versus maintenance therapy (specific drug)): various intermittent typical antipsychotics (low dose) vs maintained typical antipsychotics - long term (≥26 weeks)’.  **Type:** Intervention.  **Covariate summary:** Type of intervention or control. | **Method to choose**  **covariate:** Not reported.  **Where method reported:** NA.  **Rationale for choosing the covariate:** Not reported.  **Where rationale reported:** NA. | **Where reported:** Protocol and review.  **Reason not reported in review:** NA.  **Reason not reported in protocol:** NA.  **Labelled as ‘posthoc’ if not in protocol:** NA**.** | **Data type:** Categorical.  **Continuous covariate categorised:** NA.  **How determine it was categorised:** NA.  **Justification for categorising:** NA.  **Where justification reported:** NA.  **Categories reported:** Yes.  **Where categories reported:** Protocol and review.  **Justification for categories:** Not reported.  **Where justification reported:** NA. | **Analysis type reported in protocol:** Subgroup analysis.  **Analysis type reported in review:** Subgroup analysis..  **Reason for changing mind:** NA.    **Analysis type in protocol according to glossary:** Subgroup analysis.  **How determined the analysis type:** Categories given.  **Analysis type in review according to glossary:** Subgroup analysis.  **How determined the analysis type:** Categories given.  **Analysed:** Yes.  **Reasons given (in the review) for not doing interaction analysis if planned:** NA.  **Reasons given for carrying out interaction analysis if they weren’t planned:** NA.  **Scenario in protocol:** Not reported.  **Rationale for planned scenario:** NA.  **Scenario in review (if reported specifically):** Not reported.  **Rationale for done scenario:** NA.  **Reason for changing mind:** NA. | **Method to detect interactions reported in protocol:** Not reported.  **Method to detect interactions reported in review:** Not reported.  **Reason for changing mind:** NA. |
| Sampson 2013 | **Protocol:** ‘Where a trial was described as ’double-blind’, but it was only implied that the study was randomised, we included these trials in a sensitivity analysis.’  **Review methods: ‘**Implication of randomisation. We included trials in a sensitivity analysis if they were described in some way as to imply randomisation.’  **Review results: ‘**When included studies that implied randomisation or provided no further details regarding randomisation techniques were removed from the meta-analysis’.  **Type:** Methodological.  **Covariate summary:** Implied randomisation. | **Method to choose**  **covariate:** Not reported.  **Where method reported:** NA.  **Rationale for choosing the covariate:** Not reported.  **Where rationale reported:** NA. | **Where reported:** Protocol and review.  **Reason not reported in review:** NA.  **Reason not reported in protocol:** NA.  **Labelled as ‘posthoc’ if not in protocol:** NA**.** | **Data type:** Categorical.  **Continuous covariate categorised:** NA.  **How determine it was categorised:** NA.  **Justification for categorising:** NA.  **Where justification reported:** NA.  **Categories reported:** Yes.  **Where categories reported:** Protocol and review.  **Justification for categories:** Not reported.  **Where justification reported:** NA. | **Analysis type reported in protocol:** Sensitivity analysis.  **Analysis type reported in review:** Sensitivity analysis.  **Reason for changing mind:** NA.    **Analysis type in protocol according to glossary:** Sensitivity analysis.  **How determined the analysis type:** Categories given.  **Analysis type in review according to glossary:** Sensitivity analysis.  **How determined the analysis type:** Categories given.  **Analysed:** Yes.  **Reasons given (in the review) for not doing interaction analysis if planned:** NA.  **Reasons given for carrying out interaction analysis if they weren’t planned:** NA.  **Scenario in protocol:** Not reported.  **Rationale for planned scenario:** NA.  **Scenario in review (if reported specifically):** Not reported.  **Rationale for done scenario:** NA.  **Reason for changing mind:** NA. | **Method to detect interactions reported in protocol:** Not reported.  **Method to detect interactions reported in review:** Not reported.  **Reason for changing mind:** NA. |
| Sampson 2013 | **Protocol:** Not reported.  **Review methods:** Risk of bias. ‘We analysed the effects of excluding trials that were judged to be at high risk of bias across one or more of the domains of randomisation (implied as randomised with no further details available) allocation concealment, blinding and outcome reporting for the meta-analysis of the primary outcome.’  **Review results:** Not analysed.  **Type:** Methodological.  **Covariate summary:** Risk of bias. | **Method to choose**  **covariate:** Not reported.  **Where method reported:** NA.  **Rationale for choosing the covariate:** Not reported.  **Where rationale reported:** NA. | **Where reported:** Review only.  **Reason not reported in review:** NA.  **Reason not reported in protocol:** Not reported.  **Labelled as ‘posthoc’ if not in protocol:** No**.** | **Data type:** Categorical.  **Continuous covariate categorised:** NA.  **How determine it was categorised:** NA.  **Justification for categorising:** NA.  **Where justification reported:** NA.  **Categories reported:** Yes.  **Where categories reported:** Review only.  **Justification for categories:** Not reported.  **Where justification reported:** NA. | **Analysis type reported in protocol:** Not reported.  **Analysis type reported in review:** Sensitivity analysis.  **Reason for changing mind:** Not reported.    **Analysis type in protocol according to glossary:** Not reported.  **How determined the analysis type:** NA.  **Analysis type in review according to glossary:** Sensitivity analysis.  **How determined the analysis type:** Categories given.  **Analysed:** No.  **Reasons given (in the review) for not doing interaction analysis if planned:** ‘However, all included studies were  rated as ’high’ on risk of bias on one or more of the domains,  therefore, the effects of excluding each study would have resulted  in no data to compare.’’ All of the included studies were judged to be at a high risk of bias across one or more of the domains of randomisation, allocation concealment, blinding and outcome reporting, which makes it difficult to draw meaningful conclusions from a sensitivity analyses, as we would need substantial power to show real differences with confidence.‘  **Reasons given for carrying out interaction analysis if they weren’t planned:** NA.  **Scenario in protocol:** Not reported.  **Rationale for planned scenario:** NA.  **Scenario in review (if reported specifically):** Not reported.  **Rationale for done scenario:** NA.  **Reason for changing mind:** NA. | **Method to detect interactions reported in protocol:** Not reported.  **Method to detect interactions reported in review:** If the exclusion of trials at  high risk of bias did not substantially alter the direction of effect  or the precision of the effect estimates, then data from these trials  were included in the analysis.  **Reason for changing mind:** Not reported. |
| Sanders 2013 | **Protocol: ‘**Co-interventions or standard care should be equivalent in all randomized groups. We will include trials where other features of care differ between intervention groups but will consider these differences in the risk of bias assessment and perform sensitivity analyses to assess their impact on the results of the review.’  **Review methods: ‘**Co-interventions or standard care should have been equivalent in all randomly assigned groups. We included trials where other features of care differ between intervention groups but considered these differences in the risk of bias assessment and performed sensitivity analyses to assess their impact on the results of the review.’  **Review results:** Not analysed.  **Type:** Methodological.  **Covariate summary:** Risk of bias. | **Method to choose**  **covariate:** Not reported.  **Where method reported:** NA.  **Rationale for choosing the covariate:** Not reported.  **Where rationale reported:** NA. | **Where reported:** Protocol and review.  **Reason not reported in review:** NA.  **Reason not reported in protocol:** NA.  **Labelled as ‘posthoc’ if not in protocol:** NA. | **Data type:** Categorical.  **Continuous covariate categorised:** NA.  **How determine it was categorised:** NA.  **Justification for categorising:** NA.  **Where justification reported:** NA.  **Categories reported:** Not reported.  **Where categories reported:** NA.  **Justification for categories:** Not reported.  **Where justification reported:** NA. | **Analysis type reported in protocol:** Sensitivity analysis.  **Analysis type reported in review:** Sensitivity analysis.  **Reason for changing mind:** NA.    **Analysis type in protocol according to glossary:** Sensitivity analysis.  **How determined the analysis type:** Says ‘Sensitivity analysis.’. No reason to dispute.  **Analysis type in review according to glossary:** Sensitivity analysis.  **How determined the analysis type:** Says ‘Sensitivity analysis.’. No reason to dispute.  **Analysed:** No.  **Reasons given (in the review) for not doing interaction analysis if planned:** Not reported.  **Reasons given for carrying out interaction analysis if they weren’t planned:** NA.  **Scenario in protocol:** Not reported.  **Rationale for planned scenario:** NA.  **Scenario in review (if reported specifically):** Not reported.  **Rationale for done scenario:** NA.  **Reason for changing mind:** NA. | **Method to detect interactions reported in protocol:** Not reported.  **Method to detect interactions reported in review:** Not reported.  **Reason for changing mind:** NA. |
| Sanders 2013 | **Protocol: ‘**Comparison groups will be one of: Placebo; or No treatment or standard care.’  **Review methods: ‘**Comparison groups were one of: Placebo; or No treatment or standard care.’ ‘In this patient group, the comparison groups will be a different dose of statin’.  **Review results:** ‘Comparison of statin with no treatment/placebo’; ‘comparison of high-dose with low-dose statin’.  **Type:** Intervention.  **Covariate summary:** Type of intervention or control and dose. | **Method to choose**  **covariate:** Not reported.  **Where method reported:** NA.  **Rationale for choosing the covariate:** Not reported.  **Where rationale reported:** NA. | **Where reported:** Protocol and review.  **Reason not reported in review:** NA.  **Reason not reported in protocol:** NA.  **Labelled as ‘posthoc’ if not in protocol:** NA. | **Data type:** Categorical.  **Continuous covariate categorised:** NA.  **How determine it was categorised:** NA.  **Justification for categorising:** NA.  **Where justification reported:** NA.  **Categories reported:** Yes.  **Where categories reported:** Protocol and review.  **Justification for categories:** Not reported.  **Where justification reported:** NA. | **Analysis type reported in protocol:** Unnamed analysis.  **Analysis type reported in review:** Unnamed analysis.  **Reason for changing mind:** NA.    **Analysis type in protocol according to glossary:** Stratification/subgroup analysis.  **How determined the analysis type:** Categories given.  **Analysis type in review according to glossary:** Stratification/subgroup analysis.  **How determined the analysis type:** Categories given.  **Analysed:** Yes.  **Reasons given (in the review) for not doing interaction analysis if planned:** NA.  **Reasons given for carrying out interaction analysis if they weren’t planned:** NA.  **Scenario in protocol:** Not reported.  **Rationale for planned scenario:** NA.  **Scenario in review (if reported specifically):** Not reported.  **Rationale for done scenario:** NA.  **Reason for changing mind:** NA. | **Method to detect interactions reported in protocol:** Not reported.  **Method to detect interactions reported in review:** Not reported.  **Reason for changing mind:** NA. |
| Sanders 2013 | **Protocol:** ‘Emergency or elective surgery’.  **Review methods: ‘**Emergency or elective surgery’.  **Review results:** Not analysed.  **Type:** Intervention.  **Covariate summary:** Type of intervention or control. | **Method to choose**  **covariate:** Not reported.  **Where method reported:** NA.  **Rationale for choosing the covariate:** Not reported.  **Where rationale reported:** NA. | **Where reported:** Protocol and review.  **Reason not reported in review:** NA.  **Reason not reported in protocol:** NA.  **Labelled as ‘posthoc’ if not in protocol:** NA. | **Data type:** Categorical.  **Continuous covariate categorised:** NA.  **How determine it was categorised:** NA.  **Justification for categorising:** NA.  **Where justification reported:** NA.  **Categories reported:** Yes.  **Where categories reported:** Protocol and review.  **Justification for categories:** Not reported.  **Where justification reported:** NA. | **Analysis type reported in protocol:** Subgroup analysis.  **Analysis type reported in review:** Subgroup analysis.  **Reason for changing mind:** NA.    **Analysis type in protocol according to glossary:** Subgroup analysis.  **How determined the analysis type:** Categories given.  **Analysis type in review according to glossary:** Subgroup analysis.  **How determined the analysis type:** Categories given.  **Analysed:** No.  **Reasons given (in the review) for not doing interaction analysis if planned:** ‘We were unable to carry out planned sensitivity analyses on type  of intervention, elective or emergency surgery or duration of statin  use because of low numbers of studies.’  **Reasons given for carrying out interaction analysis if they weren’t planned:** NA.  **Scenario in protocol: ‘**If data are sufficient we will investigate the following subgroups,  which may account for heterogeneity between studies:’  **Rationale for planned scenario:** Not reported.  **Scenario in review (if reported specifically): ‘**If data had been sufficient we planned to investigate the following  subgroups, which could account for heterogeneity between studies:’  **Rationale for done scenario:** Not reported.  **Reason for changing mind:** NA. | **Method to detect interactions reported in protocol: ‘**We will assess differences in effect size between subgroups in  Revman, using I2 estimates (Section 9.6.3.1 ofHiggins 2011).We  will combine results in smaller groups if appropriate.’  **Method to detect interactions reported in review: ‘**We planned to assess differences in effect size between subgroups  in RevMan, using I2 estimates (Section 9.6.3.1 of Higgins 2011)  and to combine results in smaller groups if appropriate.’  **Reason for changing mind:** NA. |
| Sanders 2013 | **Protocol:** ‘Risk of bias’.  **Review methods:** risk of bias.  **Review results:** Not analysed.  **Type:** Methodological.  **Covariate summary:** Risk of bias. | **Method to choose**  **covariate:** Not reported.  **Where method reported:** NA.  **Rationale for choosing the covariate:** Not reported.  **Where rationale reported:** NA. | **Where reported:** Protocol and review.  **Reason not reported in review:** NA.  **Reason not reported in protocol:** NA.  **Labelled as ‘posthoc’ if not in protocol:** NA. | **Data type:** Categorical.  **Continuous covariate categorised:** NA.  **How determine it was categorised:** NA.  **Justification for categorising:** NA.  **Where justification reported:** NA.  **Categories reported:** Not reported.  **Where categories reported:** NA.  **Justification for categories:** Not reported.  **Where justification reported:** NA. | **Analysis type reported in protocol:** Sensitivity analysis.  **Analysis type reported in review:** Sensitivity analysis.  **Reason for changing mind:** NA.    **Analysis type in protocol according to glossary:** Sensitivity analysis.  **How determined the analysis type:** Says ‘Sensitivity analysis.’. No reason to dispute.  **Analysis type in review according to glossary:** Sensitivity analysis.  **How determined the analysis type:** Says ‘Sensitivity analysis.’. No reason to dispute.  **Analysed:** No.  **Reasons given (in the review) for not doing interaction analysis if planned:** Not reported.  **Reasons given for carrying out interaction analysis if they weren’t planned:** NA.  **Scenario in protocol: ‘**In addition to the sensitivity analyses outlined above, to describe  the impact of missing data we will also undertake analyses to explore  the contribution of…’  **Rationale for planned scenario:** Not reported.  **Scenario in review (if reported specifically): ‘**If sufficient studies had been found, we aimed to undertake analyses to explore the contributions of:’  **Rationale for done scenario:** Not reported.  **Reason for changing mind:** NA. | **Method to detect interactions reported in protocol:** Not reported.  **Method to detect interactions reported in review:** Not reported.  **Reason for changing mind:** NA. |
| Sanders 2013 | **Protocol:** ‘Timing of outcome measurement’.  **Review methods: ‘**Timing of outcome measurement’.  **Review results:** Not analysed.  **Type:** Outcome.  **Covariate summary:** Time point. | **Method to choose**  **covariate:** Not reported.  **Where method reported:** NA.  **Rationale for choosing the covariate:** Not reported.  **Where rationale reported:** NA. | **Where reported:** Protocol and review.  **Reason not reported in review:** NA.  **Reason not reported in protocol:** NA.  **Labelled as ‘posthoc’ if not in protocol:** NA. | **Data type:** Continuous.  **Continuous covariate categorised:** Yes.  **How determine it was categorised:** Subgroup analysis planned.  **Justification for categorising:** Not reported.  **Where justification reported:** NA.  **Categories reported:** Not reported.  **Where categories reported:** NA.  **Justification for categories:** Not reported.  **Where justification reported:** NA. | **Analysis type reported in protocol:** Subgroup analysis.  **Analysis type reported in review:** Subgroup analysis.  **Reason for changing mind:** NA.    **Analysis type in protocol according to glossary:** Subgroup analysis.  **How determined the analysis type:** Says ‘subgroup analysis’. No reason to dispute.  **Analysis type in review according to glossary:** Subgroup analysis.  **How determined the analysis type:** Says ‘subgroup analysis’. No reason to dispute.  **Analysed:** No.  **Reasons given (in the review) for not doing interaction analysis if planned:** Not reported.  **Reasons given for carrying out interaction analysis if they weren’t planned:** NA.  **Scenario in protocol: ‘**If data are sufficient we will investigate the following subgroups,  which may account for heterogeneity between studies:’  **Rationale for planned scenario:** Not reported.  **Scenario in review (if reported specifically): ‘**If data had been sufficient we planned to investigate the following  subgroups, which could account for heterogeneity between studies:’  **Rationale for done scenario:** Not reported.  **Reason for changing mind:** NA. | **Method to detect interactions reported in protocol: ‘**We will assess differences in effect size between subgroups in  Revman, using I2 estimates (Section 9.6.3.1 ofHiggins 2011).We  will combine results in smaller groups if appropriate.’  **Method to detect interactions reported in review: ‘**We planned to assess differences in effect size between subgroups  in RevMan, using I2 estimates (Section 9.6.3.1 of Higgins 2011)  and to combine results in smaller groups if appropriate.’  **Reason for changing mind:** NA. |
| Sanders 2013 | **Protocol:** ‘Type of surgical intervention’.  **Review methods: ‘**Type of surgical intervention’.  **Review results:** Not analysed.  **Type:** Intervention.  **Covariate summary:** Type of intervention or control. | **Method to choose**  **covariate:** Not reported.  **Where method reported:** NA.  **Rationale for choosing the covariate:** Not reported.  **Where rationale reported:** NA. | **Where reported:** Protocol and review.  **Reason not reported in review:** NA.  **Reason not reported in protocol:** NA.  **Labelled as ‘posthoc’ if not in protocol:** NA. | **Data type:** Categorical.  **Continuous covariate categorised:** NA.  **How determine it was categorised:** NA.  **Justification for categorising:** NA.  **Where justification reported:** NA.  **Categories reported:** Not reported.  **Where categories reported:** NA.  **Justification for categories:** Not reported.  **Where justification reported:** NA. | **Analysis type reported in protocol:** Subgroup analysis.  **Analysis type reported in review:** Subgroup analysis.  **Reason for changing mind:** NA.    **Analysis type in protocol according to glossary:** Subgroup analysis.  **How determined the analysis type:** Says ‘subgroup analysis’. No reason to dispute.  **Analysis type in review according to glossary:** Subgroup analysis.  **How determined the analysis type:** Says ‘subgroup analysis’. No reason to dispute.  **Analysed:** No.  **Reasons given (in the review) for not doing interaction analysis if planned:** ‘We were unable to carry out planned sensitivity analyses on type  of intervention, elective or emergency surgery or duration of statin  use because of low numbers of studies.’  **Reasons given for carrying out interaction analysis if they weren’t planned:** NA.  **Scenario in protocol: ‘**If data are sufficient we will investigate the following subgroups,  which may account for heterogeneity between studies:’  **Rationale for planned scenario:** Not reported.  **Scenario in review (if reported specifically): ‘**If data had been sufficient we planned to investigate the following  subgroups, which could account for heterogeneity between studies:’  **Rationale for done scenario:** Not reported.  **Reason for changing mind:** NA. | **Method to detect interactions reported in protocol: ‘**We will assess differences in effect size between subgroups in  Revman, using I2 estimates (Section 9.6.3.1 ofHiggins 2011).We  will combine results in smaller groups if appropriate.’  **Method to detect interactions reported in review: ‘**We planned to assess differences in effect size between subgroups  in RevMan, using I2 estimates (Section 9.6.3.1 of Higgins 2011)  and to combine results in smaller groups if appropriate.’  **Reason for changing mind:** NA. |
| Sanders 2013 | **Protocol:** ‘Unpublished studies’.  **Review methods:** unpublished studies.  **Review results:** Not analysed.  **Type:** Methodological.  **Covariate summary:** Publication status. | **Method to choose**  **covariate:** Not reported.  **Where method reported:** NA.  **Rationale for choosing the covariate:** Not reported.  **Where rationale reported:** NA. | **Where reported:** Protocol and review.  **Reason not reported in review:** NA.  **Reason not reported in protocol:** NA.  **Labelled as ‘posthoc’ if not in protocol:** NA. | **Data type:** Categorical.  **Continuous covariate categorised:** NA.  **How determine it was categorised:** NA.  **Justification for categorising:** NA.  **Where justification reported:** NA.  **Categories reported:** Yes.  **Where categories reported:** Protocol and review.  **Justification for categories:** Not reported.  **Where justification reported:** NA. | **Analysis type reported in protocol:** Sensitivity analysis.  **Analysis type reported in review:** Sensitivity analysis.  **Reason for changing mind:** NA.    **Analysis type in protocol according to glossary:** Sensitivity analysis.  **How determined the analysis type:** Categories given.  **Analysis type in review according to glossary:** Sensitivity analysis.  **How determined the analysis type:** Categories given.  **Analysed:** No.  **Reasons given (in the review) for not doing interaction analysis if planned:** Not reported.  **Reasons given for carrying out interaction analysis if they weren’t planned:** NA.  **Scenario in protocol: ‘**In addition to the sensitivity analyses outlined above, to describe  the impact of missing data we will also undertake analyses to explore  the contribution of…’  **Rationale for planned scenario:** Not reported.  **Scenario in review (if reported specifically): ‘**If sufficient studies had been found, we aimed to undertake analyses to explore the contributions of:’  **Rationale for done scenario:** Not reported.  **Reason for changing mind:** NA. | **Method to detect interactions reported in protocol:** Not reported.  **Method to detect interactions reported in review:** Not reported.  **Reason for changing mind:** NA. |
| Sanders 2013 | **Protocol:** Dosage. We judged the following to be two covariates: ‘Dosage or duration of statin therapy’  **Review methods:** Dosage. We judged the following to be two covariates: ‘Dosage or duration of statin therapy’  **Review results:** Not analysed.  **Type:** Intervention.  **Covariate summary:** Dose. | **Method to choose**  **covariate:** Not reported.  **Where method reported:** NA.  **Rationale for choosing the covariate:** Not reported.  **Where rationale reported:** NA. | **Where reported:** Protocol and review.  **Reason not reported in review:** NA.  **Reason not reported in protocol:** NA.  **Labelled as ‘posthoc’ if not in protocol:** NA. | **Data type:** Continuous.  **Continuous covariate categorised:** Unclear.  **How determine it was categorised:** Subgroup analysis or meta-regression planned.  **Justification for categorising:** NA.  **Where justification reported:** NA.  **Categories reported:** Not reported.  **Where categories reported:** NA.  **Justification for categories:** Not reported.  **Where justification reported:** NA. | **Analysis type reported in protocol:** Subgroup analysis and meta-regression.  **Analysis type reported in review:** Subgroup analysis and meta-regression.  **Reason for changing mind:** NA.    **Analysis type in protocol according to glossary:** Subgroup analysis and meta-regression.  **How determined the analysis type:** Says ‘Subgroup analysis and meta-regression.’. No reason to dispute.  **Analysis type in review according to glossary:** Subgroup analysis and meta-regression.  **How determined the analysis type:** Says ‘Subgroup analysis and meta-regression.’. No reason to dispute  **Analysed:** No.  **Reasons given (in the review) for not doing interaction analysis if planned:** Not reported.  **Reasons given for carrying out interaction analysis if they weren’t planned:** NA.  **Scenario in protocol: ‘**Important heterogeneity  (Chi2 P < 0.1 and I2 > 50%) will be investigated, where  possible, by subgroup analyses and meta-regression,:’  **Rationale for planned scenario:** Not reported.  **Scenario in review (if reported specifically): ‘**Important heterogeneity (Chi2 P < 0.1 and I2 >  50%) would have been investigated, where possible, by subgroup  analyses and meta-regression,:’  **Rationale for done scenario:** Not reported.  **Reason for changing mind:** NA. | **Method to detect interactions reported in protocol:** Not reported.  **Method to detect interactions reported in review:** Not reported.  **Reason for changing mind:** NA. |
| Sanders 2013 | **Protocol:** Duration of statin therapy. We judged the following to be two covariates: ‘Dosage or duration of statin therapy’. ‘Duration of statin use.’  **Review methods:** Duration of statin therapy. We judged the following to be two covariates: ‘Dosage or duration of statin therapy’. ‘Duration of statin use.’  **Review results:** Not analysed.  **Type:** Intervention.  **Covariate summary:** Duration of intervention. | **Method to choose**  **covariate:** Not reported.  **Where method reported:** NA.  **Rationale for choosing the covariate:** Not reported.  **Where rationale reported:** NA. | **Where reported:** Protocol and review.  **Reason not reported in review:** NA.  **Reason not reported in protocol:** NA.  **Labelled as ‘posthoc’ if not in protocol:** NA. | **Data type:** Continuous.  **Continuous covariate categorised:** Unclear.  **How determine it was categorised:** Subgroup analysis or meta-regression planned.  **Justification for categorising:** NA.  **Where justification reported:** NA.  **Categories reported:** Not reported.  **Where categories reported:** NA.  **Justification for categories:** Not reported.  **Where justification reported:** NA. | **Analysis type reported in protocol:** Subgroup analysis and meta-regression.  **Analysis type reported in review:** Subgroup analysis and meta-regression.  **Reason for changing mind:** NA.    **Analysis type in protocol according to glossary:** Subgroup analysis and meta-regression.  **How determined the analysis type:** Says ‘Subgroup analysis and meta-regression.’. No reason to dispute.  **Analysis type in review according to glossary:** Subgroup analysis and meta-regression.  **How determined the analysis type:** Says ‘Subgroup analysis and meta-regression.’. No reason to dispute  **Analysed:** No.  **Reasons given (in the review) for not doing interaction analysis if planned:** ‘‘We were unable to carry out planned sensitivity analyses on type  of intervention, elective or emergency surgery or duration of statin  use because of low numbers of studies.’  **Reasons given for carrying out interaction analysis if they weren’t planned:** NA.  **Scenario in protocol: ‘**Important heterogeneity  (Chi2 P < 0.1 and I2 > 50%) will be investigated, where  possible, by subgroup analyses and meta-regression,:’  **‘**If data are sufficient we will investigate the following subgroups,  which may account for heterogeneity between studies:’  **Rationale for planned scenario:** Not reported.  **Scenario in review (if reported specifically): ‘**Important heterogeneity (Chi2 P < 0.1 and I2 >  50%) would have been investigated, where possible, by subgroup  analyses and meta-regression,:’  **‘**If data had been sufficient we planned to investigate the following  subgroups, which could account for heterogeneity between studies:’ | **Method to detect interactions reported in protocol: ‘**We will assess differences in effect size between subgroups in  Revman, using I2 estimates (Section 9.6.3.1 ofHiggins 2011).We  will combine results in smaller groups if appropriate.’ (for subgroup analysis).  **Method to detect interactions reported in review: ‘**We planned to assess differences in effect size between subgroups  in RevMan, using I2 estimates (Section 9.6.3.1 of Higgins 2011)  and to combine results in smaller groups if appropriate.’ (for subgroup analysis).  **Reason for changing mind:** NA. |
| Sanders 2013 | **Protocol:** Not reported.  **Review methods:** Not reported.  **Review results:** ‘We excluded the DECREASE III study because of concerns about validity, but we ran sensitivity analyses on its impact on our results’.  **Type:** Other.  **Covariate summary:** Outlying results. | **Method to choose**  **covariate:** Not reported.  **Where method reported:** NA.  **Rationale for choosing the covariate:** Not reported.  **Where rationale reported:** NA. | **Where reported:** Review only.  **Reason not reported in review:** NA.  **Reason not reported in protocol:** Not reported.  **Labelled as ‘posthoc’ if not in protocol:** Not reported. | **Data type:** Categorical.  **Continuous covariate categorised:** NA.  **How determine it was categorised:** NA.  **Justification for categorising:** NA.  **Where justification reported:** NA.  **Categories reported:** Yes.  **Where categories reported:** Review only.  **Justification for categories:** ‘because of concerns about  validity’.  **Where justification reported:** Review only. | **Analysis type reported in protocol:** Not reported.  **Analysis type reported in review:** Sensitivity analysis.  **Reason for changing mind:** Not reported.    **Analysis type in protocol according to glossary:** Not reported.  **How determined the analysis type:** NA.  **Analysis type in review according to glossary:** Sensitivity analysis.  **How determined the analysis type:** Categories given.  **Analysed:** Yes.  **Reasons given (in the review) for not doing interaction analysis if planned:** NA.  **Reasons given for carrying out interaction analysis if they weren’t planned:** Not reported.  **Scenario in protocol:** Not reported.  **Rationale for planned scenario:** NA.  **Scenario in review (if reported specifically):** Not reported.  **Rationale for done scenario:** NA.  **Reason for changing mind:** NA. | **Method to detect interactions reported in protocol:** Not reported.  **Method to detect interactions reported in review:** Not reported.  **Reason for changing mind:** NA. |
| Sanders 2013 | **Protocol:** Not reported.  **Review methods: ‘**Risk of missing outcome data’.  **Review results:** Not analysed.  **Type:** Methodological.  **Covariate summary:** Incomplete outcome data/follow up. | **Method to choose**  **covariate:** Not reported.  **Where method reported:** NA.  **Rationale for choosing the covariate:** Not reported.  **Where rationale reported:** NA. | **Where reported:** Review only.  **Reason not reported in review:** NA.  **Reason not reported in protocol:** Not reported.  **Labelled as ‘posthoc’ if not in protocol:** Not reported. | **Data type:** Categorical.  **Continuous covariate categorised:** NA.  **How determine it was categorised:** NA.  **Justification for categorising:** NA.  **Where justification reported:** NA.  **Categories reported:** Not reported.  **Where categories reported:** NA.  **Justification for categories:** Not reported.  **Where justification reported:** NA. | **Analysis type reported in protocol:** Not reported.  **Analysis type reported in review:** Sensitivity analysis.  **Reason for changing mind:** Not reported.    **Analysis type in protocol according to glossary:** Not reported.  **How determined the analysis type:** NA.  **Analysis type in review according to glossary:** Sensitivity analysis.  **How determined the analysis type:** Says ‘Sensitivity analysis.’. No reason to dispute.  **Analysed:** No.  **Reasons given (in the review) for not doing interaction analysis if planned:** Not reported.  **Reasons given for carrying out interaction analysis if they weren’t planned:** NA.  **Scenario in protocol: ‘**In addition to the sensitivity analyses outlined above, to describe  the impact of missing data we will also undertake analyses to explore  the contribution of…’  **Rationale for planned scenario:** Not reported.  **Scenario in review (if reported specifically): ‘**If sufficient studies had been found, we aimed to undertake analyses to explore the contributions of:’  **Rationale for done scenario:** Not reported.  **Reason for changing mind:** NA. | **Method to detect interactions reported in protocol:** Not reported.  **Method to detect interactions reported in review:** Not reported.  **Reason for changing mind:** NA. |
| Sarai 2013 | **Protocol:** ‘Dose of magnesium’.  **Review methods: ‘**Different doses of magnesium’.  **Review results:** Not analysed.  **Type:** Intervention.  **Covariate summary:** Dose. | **Method to choose**  **covariate:** Not reported.  **Where method reported:** NA.  **Rationale for choosing the covariate:** Not reported.  **Where rationale reported:** NA. | **Where reported:** Protocol and review.  **Reason not reported in review:** NA.  **Reason not reported in protocol:** NA.  **Labelled as ‘posthoc’ if not in protocol:** NA. | **Data type:** Continuous.  **Continuous covariate categorised:** Yes.  **How determine it was categorised:** Sensitivity analysis planned.  **Justification for categorising:** Not reported.  **Where justification reported:** NA.  **Categories reported:** Not reported.  **Where categories reported:** NA.  **Justification for categories:** Not reported.  **Where justification reported:** NA. | **Analysis type reported in protocol:** Sensitivity analyses.  **Analysis type reported in review:** Sensitivity analyses  **Reason for changing mind:** NA.    **Analysis type in protocol according to glossary:** Subgroup analyses.  **How determined the analysis type:** Categories given.  **Analysis type in review according to glossary:** Subgroup analyses.  **How determined the analysis type:** Categories given.  **Analysed:** No.  **Reasons given (in the review) for not doing interaction analysis if planned:** ‘Our meta-analysis was not amenable to the planned subgroup and sensitivity analyses, as we had too few included studies to explore these variations.’  **Reasons given for carrying out interaction analysis if they weren’t planned:** NA.  **Scenario in protocol:** Not reported.  **Rationale for planned scenario:** NA.  **Scenario in review (if reported specifically):** Not reported.  **Rationale for done scenario:** NA.  **Reason for changing mind:** NA. | **Method to detect interactions reported in protocol:** Not reported.  **Method to detect interactions reported in review:** Not reported.  **Reason for changing mind:** NA. |
| Sarai 2013 | **Protocol:** ‘Number of doses of magnesium’.  **Review methods: ‘**Number of doses of magnesium’.  **Review results:** Not analysed.  **Type:** Intervention.  **Covariate summary:** Dose. | **Method to choose**  **covariate:** Not reported.  **Where method reported:** NA.  **Rationale for choosing the covariate:** Not reported.  **Where rationale reported:** NA. | **Where reported:** Protocol and review.  **Reason not reported in review:** NA.  **Reason not reported in protocol:** NA.  **Labelled as ‘posthoc’ if not in protocol:** NA. | **Data type:** Continuous.  **Continuous covariate categorised:** Yes.  **How determine it was categorised:** Sensitivity analysis planned.  **Justification for categorising:** Not reported.  **Where justification reported:** NA.  **Categories reported:** Not reported.  **Where categories reported:** NA.  **Justification for categories:** Not reported.  **Where justification reported:** NA. | **Analysis type reported in protocol:** Sensitivity analyses.  **Analysis type reported in review:** Sensitivity analyses  **Reason for changing mind:** NA.    **Analysis type in protocol according to glossary:** Subgroup analyses.  **How determined the analysis type:** Categories given.  **Analysis type in review according to glossary:** Subgroup analyses.  **How determined the analysis type:** Categories given.  **Analysed:** No.  **Reasons given (in the review) for not doing interaction analysis if planned:** ‘Our meta-analysis was not amenable to the planned subgroup and sensitivity analyses, as we had too few included studies to explore these variations.’  **Reasons given for carrying out interaction analysis if they weren’t planned:** NA.  **Scenario in protocol:** Not reported.  **Rationale for planned scenario:** NA.  **Scenario in review (if reported specifically):** Not reported.  **Rationale for done scenario:** NA.  **Reason for changing mind:** NA. | **Method to detect interactions reported in protocol:** Not reported.  **Method to detect interactions reported in review:** Not reported.  **Reason for changing mind:** NA. |
| Sarai 2013 | **Protocol:** ‘Patients who had low serum magnesium levels versus patients that had normal/elevated serum magnesium levels.’  **Review methods: ‘**Participants who had low serum magnesium levels versus patients that had normal/elevated serum magnesium levels.’  **Review results:** Not analysed.  **Type:** Patient.  **Covariate summary:** Disease characteristics. | **Method to choose**  **covariate:** Not reported.  **Where method reported:** NA.  **Rationale for choosing the covariate:** Not reported.  **Where rationale reported:** NA. | **Where reported:** Protocol and review.  **Reason not reported in review:** NA.  **Reason not reported in protocol:** NA.  **Labelled as ‘posthoc’ if not in protocol:** NA. | **Data type:** Continuous.  **Continuous covariate categorised:** Yes.  **How determine it was categorised:** Categories given.  **Justification for categorising:** Not reported.  **Where justification reported:** NA.  **Categories reported:** Yes.  **Where categories reported:** Protocol and review.  **Justification for categories:** Not reported.  **Where justification reported:** NA. | **Analysis type reported in protocol:** Subgroup analyses.  **Analysis type reported in review:** Subgroup analyses.  **Reason for changing mind:** NA.    **Analysis type in protocol according to glossary:** Subgroup analyses.  **How determined the analysis type:** Categories given.  **Analysis type in review according to glossary:** Subgroup analyses.  **How determined the analysis type:** Categories given.  **Analysed:** No.  **Reasons given (in the review) for not doing interaction analysis if planned:** ‘Our meta-analysis was not amenable to the planned subgroup and sensitivity analyses, as we had too few included studies to explore these variations.’  **Reasons given for carrying out interaction analysis if they weren’t planned:** NA.  **Scenario in protocol:** Not reported.  **Rationale for planned scenario:** NA.  **Scenario in review (if reported specifically):** Not reported.  **Rationale for done scenario:** NA.  **Reason for changing mind:** NA. | **Method to detect interactions reported in protocol:** Not reported.  **Method to detect interactions reported in review:** Not reported.  **Reason for changing mind:** NA. |
| Sarai 2013 | **Protocol:** ‘Patients with severe comorbid diseases versus mild moderate comorbid diseases, as measure with appropriate, validated scales.’  **Review methods: ‘**Participants with severe comorbid diseases versus mild moderate comorbid diseases, as measure with appropriate, validated scales.’  **Review results:** Not analysed.  **Type:** Patient.  **Covariate summary:** Disease characteristics. | **Method to choose**  **covariate:** Not reported.  **Where method reported:** NA.  **Rationale for choosing the covariate:** Not reported.  **Where rationale reported:** NA. | **Where reported:** Protocol and review.  **Reason not reported in review:** NA.  **Reason not reported in protocol:** NA.  **Labelled as ‘posthoc’ if not in protocol:** NA. | **Data type:** Categorical.  **Continuous covariate categorised:** NA.  **How determine it was categorised:** NA.  **Justification for categorising:** NA.  **Where justification reported:** NA.  **Categories reported:** Yes.  **Where categories reported:** Protocol and review.  **Justification for categories:** Not reported.  **Where justification reported:** NA. | **Analysis type reported in protocol:** Subgroup analyses.  **Analysis type reported in review:** Subgroup analyses.  **Reason for changing mind:** NA.    **Analysis type in protocol according to glossary:** Subgroup analyses.  **How determined the analysis type:** Categories given.  **Analysis type in review according to glossary:** Subgroup analyses.  **How determined the analysis type:** Categories given.  **Analysed:** No.  **Reasons given (in the review) for not doing interaction analysis if planned:** ‘Our meta-analysis was not amenable to the planned subgroup and sensitivity analyses, as we had too few included studies to explore these variations.’  **Reasons given for carrying out interaction analysis if they weren’t planned:** NA.  **Scenario in protocol:** Not reported.  **Rationale for planned scenario:** NA.  **Scenario in review (if reported specifically):** Not reported.  **Rationale for done scenario:** NA.  **Reason for changing mind:** NA. | **Method to detect interactions reported in protocol:** Not reported.  **Method to detect interactions reported in review:** Not reported.  **Reason for changing mind:** NA. |
| Sarai 2013 | **Protocol:** ‘Previous co-morbidities specifically cardiovascular disease and renal dysfunction.’  **Review methods: ‘**Previous co-morbidities specifically cardiovascular disease and renal dysfunction.’  **Review results:** Not analysed.  **Type:** Patient.  **Covariate summary:** Disease characteristics. | **Method to choose**  **covariate:** Not reported.  **Where method reported:** NA.  **Rationale for choosing the covariate:** Not reported.  **Where rationale reported:** NA. | **Where reported:** Protocol and review.  **Reason not reported in review:** NA.  **Reason not reported in protocol:** NA.  **Labelled as ‘posthoc’ if not in protocol:** NA. | **Data type:** Categorical.  **Continuous covariate categorised:** NA.  **How determine it was categorised:** NA.  **Justification for categorising:** NA.  **Where justification reported:** NA.  **Categories reported:** Yes.  **Where categories reported:** Protocol and review.  **Justification for categories:** Not reported.  **Where justification reported:** NA. | **Analysis type reported in protocol:** Subgroup analyses.  **Analysis type reported in review:** Subgroup analyses.  **Reason for changing mind:** NA.    **Analysis type in protocol according to glossary:** Subgroup analyses.  **How determined the analysis type:** Categories given.  **Analysis type in review according to glossary:** Subgroup analyses.  **How determined the analysis type:** Categories given.  **Analysed:** No.  **Reasons given (in the review) for not doing interaction analysis if planned:** ‘Our meta-analysis was not amenable to the planned subgroup and sensitivity analyses, as we had too few included studies to explore these variations.’  **Reasons given for carrying out interaction analysis if they weren’t planned:** NA.  **Scenario in protocol:** Not reported.  **Rationale for planned scenario:** NA.  **Scenario in review (if reported specifically):** Not reported.  **Rationale for done scenario:** NA.  **Reason for changing mind:** NA. | **Method to detect interactions reported in protocol:** Not reported.  **Method to detect interactions reported in review:** Not reported.  **Reason for changing mind:** NA. |
| Sarai 2013 | **Protocol: ‘**Route of administration’  **Review methods: ‘**Different routes of administration’.  **Review results:** Not analysed.  **Type:** Intervention.  **Covariate summary:** Route of administration. | **Method to choose**  **covariate:** Not reported.  **Where method reported:** NA.  **Rationale for choosing the covariate:** Not reported.  **Where rationale reported:** NA. | **Where reported:** Protocol and review.  **Reason not reported in review:** NA.  **Reason not reported in protocol:** NA.  **Labelled as ‘posthoc’ if not in protocol:** NA. | **Data type:** Categorical.  **Continuous covariate categorised:** NA.  **How determine it was categorised:** NA.  **Justification for categorising:** NA.  **Where justification reported:** NA.  **Categories reported:** Not reported.  **Where categories reported:** NA.  **Justification for categories:** Not reported.  **Where justification reported:** NA. | **Analysis type reported in protocol:** Sensitivity analyses.  **Analysis type reported in review:** Sensitivity analyses  **Reason for changing mind:** NA.    **Analysis type in protocol according to glossary:** Subgroup analyses.  **How determined the analysis type:** Categories given.  **Analysis type in review according to glossary:** Subgroup analyses.  **How determined the analysis type:** Categories given.  **Analysed:** No.  **Reasons given (in the review) for not doing interaction analysis if planned:** ‘Our meta-analysis was not amenable to the planned subgroup and sensitivity analyses, as we had too few included studies to explore these variations.’  **Reasons given for carrying out interaction analysis if they weren’t planned:** NA.  **Scenario in protocol:** Not reported.  **Rationale for planned scenario:** NA.  **Scenario in review (if reported specifically):** Not reported.  **Rationale for done scenario:** NA.  **Reason for changing mind:** NA. | **Method to detect interactions reported in protocol:** Not reported.  **Method to detect interactions reported in review:** Not reported.  **Reason for changing mind:** NA. |
| Sarai 2013 | **Protocol:** ‘Studies that enrolled patients experiencing AWS versus studies that enrolled patients who are at risk of AWS.’  **Review methods: ‘**Studies that enrolled patients experiencing AWS versus studies that enrolled patients who are at risk of AWS’.  **Review results:** Not analysed.  **Type:** Patient.  **Covariate summary:** Disease characteristics. | **Method to choose**  **covariate:** Not reported.  **Where method reported:** NA.  **Rationale for choosing the covariate:** Not reported.  **Where rationale reported:** NA. | **Where reported:** Protocol and review.  **Reason not reported in review:** NA.  **Reason not reported in protocol:** NA.  **Labelled as ‘posthoc’ if not in protocol:** NA. | **Data type:** Categorical.  **Continuous covariate categorised:** NA.  **How determine it was categorised:** NA.  **Justification for categorising:** NA.  **Where justification reported:** NA.  **Categories reported:** Yes.  **Where categories reported:** Protocol and review.  **Justification for categories:** Not reported.  **Where justification reported:** NA. | **Analysis type reported in protocol:** Subgroup analyses.  **Analysis type reported in review:** Subgroup analyses.  **Reason for changing mind:** NA.    **Analysis type in protocol according to glossary:** Subgroup analyses.  **How determined the analysis type:** Categories given.  **Analysis type in review according to glossary:** Subgroup analyses.  **How determined the analysis type:** Categories given.  **Analysed:** No.  **Reasons given (in the review) for not doing interaction analysis if planned:** ‘Our meta-analysis was not amenable to the planned subgroup and sensitivity analyses, as we had too few included studies to explore these variations.’  **Reasons given for carrying out interaction analysis if they weren’t planned:** NA.  **Scenario in protocol:** Not reported.  **Rationale for planned scenario:** NA.  **Scenario in review (if reported specifically):** Not reported.  **Rationale for done scenario:** NA.  **Reason for changing mind:** NA. | **Method to detect interactions reported in protocol:** Not reported.  **Method to detect interactions reported in review:** Not reported.  **Reason for changing mind:** NA. |
| Sarai 2013 | **Protocol:** ‘Trials preformed without intention-to-treat analysis compared to those with an intention-to-treat analysis’.  **Review methods: ‘**Trials preformed with intention-to-treat analysis compared to those without’.  **Review results:** Not analysed.  **Type:** Methodological.  **Covariate summary:** Intention to treat. | **Method to choose**  **covariate:** Not reported.  **Where method reported:** NA.  **Rationale for choosing the covariate:** Not reported.  **Where rationale reported:** NA. | **Where reported:** Protocol and review.  **Reason not reported in review:** NA.  **Reason not reported in protocol:** NA.  **Labelled as ‘posthoc’ if not in protocol:** NA. | **Data type:** Categorical.  **Continuous covariate categorised:** NA.  **How determine it was categorised:** NA.  **Justification for categorising:** NA.  **Where justification reported:** NA.  **Categories reported:** Yes.  **Where categories reported:** Protocol and review.  **Justification for categories:** Not reported.  **Where justification reported:** NA. | **Analysis type reported in protocol:** Sensitivity analyses.  **Analysis type reported in review:** Sensitivity analyses  **Reason for changing mind:** NA.    **Analysis type in protocol according to glossary:** Subgroup analyses.  **How determined the analysis type:** Categories given.  **Analysis type in review according to glossary:** Subgroup analyses.  **How determined the analysis type:** Categories given.  **Analysed:** No.  **Reasons given (in the review) for not doing interaction analysis if planned:** ‘Our meta-analysis was not amenable to the planned subgroup and sensitivity analyses, as we had too few included studies to explore these variations.’  **Reasons given for carrying out interaction analysis if they weren’t planned:** NA.  **Scenario in protocol:** Not reported.  **Rationale for planned scenario:** NA.  **Scenario in review (if reported specifically):** Not reported.  **Rationale for done scenario:** NA.  **Reason for changing mind:** NA. | **Method to detect interactions reported in protocol:** Not reported.  **Method to detect interactions reported in review:** Not reported.  **Reason for changing mind:** NA. |
| Sarai 2013 | **Protocol:** ‘Trials without proper randomisation or concealment of allocation compared to those without these characteristics’.  **Review methods: ‘**Trials with acceptable randomisation or concealment of allocation compared to those without’.  **Review results:** Not analysed.  **Type:** Methodological.  **Covariate summary:** Random generation or concealment. | **Method to choose**  **covariate:** Not reported.  **Where method reported:** NA.  **Rationale for choosing the covariate:** Not reported.  **Where rationale reported:** NA. | **Where reported:** Protocol and review.  **Reason not reported in review:** NA.  **Reason not reported in protocol:** NA.  **Labelled as ‘posthoc’ if not in protocol:** NA. | **Data type:** Categorical.  **Continuous covariate categorised:** NA.  **How determine it was categorised:** NA.  **Justification for categorising:** NA.  **Where justification reported:** NA.  **Categories reported:** Yes.  **Where categories reported:** Protocol and review.  **Justification for categories:** Not reported.  **Where justification reported:** NA. | **Analysis type reported in protocol:** Sensitivity analyses.  **Analysis type reported in review:** Sensitivity analyses  **Reason for changing mind:** NA.    **Analysis type in protocol according to glossary:** Subgroup analyses.  **How determined the analysis type:** Categories given.  **Analysis type in review according to glossary:** Subgroup analyses.  **How determined the analysis type:** Categories given.  **Analysed:** No.  **Reasons given (in the review) for not doing interaction analysis if planned:** ‘Our meta-analysis was not amenable to the planned subgroup and sensitivity analyses, as we had too few included studies to explore these variations.’  **Reasons given for carrying out interaction analysis if they weren’t planned:** NA.  **Scenario in protocol:** Not reported.  **Rationale for planned scenario:** NA.  **Scenario in review (if reported specifically):** Not reported.  **Rationale for done scenario:** NA.  **Reason for changing mind:** NA. | **Method to detect interactions reported in protocol:** Not reported.  **Method to detect interactions reported in review:** Not reported.  **Reason for changing mind:** NA. |
| Sarai 2013 | **Protocol:** ‘Unblinded versus blinded trials’.  **Review methods: ‘**Unblinded versus blinded trials’.  **Review results:** Not analysed.  **Type:** Methodological.  **Covariate summary:** Blinding. | **Method to choose**  **covariate:** Not reported.  **Where method reported:** NA.  **Rationale for choosing the covariate:** Not reported.  **Where rationale reported:** NA. | **Where reported:** Protocol and review.  **Reason not reported in review:** NA.  **Reason not reported in protocol:** NA.  **Labelled as ‘posthoc’ if not in protocol:** NA. | **Data type:** Categorical.  **Continuous covariate categorised:** NA.  **How determine it was categorised:** NA.  **Justification for categorising:** NA.  **Where justification reported:** NA.  **Categories reported:** Yes.  **Where categories reported:** Protocol and review.  **Justification for categories:** Not reported.  **Where justification reported:** NA. | **Analysis type reported in protocol:** Sensitivity analyses.  **Analysis type reported in review:** Sensitivity analyses  **Reason for changing mind:** NA.    **Analysis type in protocol according to glossary:** Subgroup analyses.  **How determined the analysis type:** Categories given.  **Analysis type in review according to glossary:** Subgroup analyses.  **How determined the analysis type:** Categories given.  **Analysed:** No.  **Reasons given (in the review) for not doing interaction analysis if planned:** ‘Our meta-analysis was not amenable to the planned subgroup and sensitivity analyses, as we had too few included studies to explore these variations.’  **Reasons given for carrying out interaction analysis if they weren’t planned:** NA.  **Scenario in protocol:** Not reported.  **Rationale for planned scenario:** NA.  **Scenario in review (if reported specifically):** Not reported.  **Rationale for done scenario:** NA.  **Reason for changing mind:** NA. | **Method to detect interactions reported in protocol:** Not reported.  **Method to detect interactions reported in review:** Not reported.  **Reason for changing mind:** NA. |
| Schoot 2013 | **Protocol:** ‘If possible, we will look at patients with haematological and solid tumours separately’  **Review methods:** ‘We planned to look at participants with haematological and solid tumours separately, but the included studies did not provide the required data so this was not feasible.’  **Review results:** Not analysed.  **Type:** Patient.  **Covariate summary:** Disease characteristics. | **Method to choose**  **covariate:** Not reported.  **Where method reported:** NA.  **Rationale for choosing the covariate:** Not reported.  **Where rationale reported:** NA. | **Where reported:** Protocol and review.  **Reason not reported in review:** NA.  **Reason not reported in protocol:** NA.  **Labelled as ‘posthoc’ if not in protocol:** NA**.** | **Data type:** Categorical.  **Continuous covariate categorised:** NA.  **How determine it was categorised:** NA.  **Justification for categorising:** NA.  **Where justification reported:** NA..  **Categories reported:** Yes.  **Where categories reported:** Protocol and review.  **Justification for categories:** Not reported..  **Where justification reported:** NA. | **Analysis type reported in protocol:** Subgroup analysis.  **Analysis type reported in review:** Subgroup analysis.  **Reason for changing mind:** NA.    **Analysis type in protocol according to glossary:** Subgroup analysis.  **How determined the analysis type:** In subgroup section of methods. Categories given.  **Analysis type in review according to glossary:** Subgroup analysis.  **How determined the analysis type:** In subgroup section of methods. Categories given.  **Analysed:** No.  **Reasons given (in the review) for not doing interaction analysis if planned:** ‘the included studies did not provide the required data so this was not feasible.’  **Reasons given for carrying out interaction analysis if they weren’t planned:** NA.  **Scenario in protocol:** ‘If possible’  **Rationale for planned scenario:** Not reported.  **Scenario in review (if reported specifically):** Not reported.  **Rationale for done scenario:** NA.  **Reason for changing mind:** Not reported. | **Method to detect interactions reported in protocol:** Not reported.  **Method to detect interactions reported in review:** Not reported.  **Reason for changing mind:** NA. |
| Schoot 2013 | **Protocol:** ‘Risk of bias criteria’.  **Review methods:** ‘We performed a sensitivity analysis based on the risk of bias criteria (i. e. excluding studies with a high risk of bias and studies for which the risk of bias was unclear) and compared the results of studies with a low risk of bias with those of all available studies, for all analyses that included more than one study.  **Review results: ‘**Risk of bias criteria’.  **Type:** Methodological.  **Covariate summary:** Risk of bias. | **Method to choose**  **covariate:** Not reported.  **Where method reported:** NA.  **Rationale for choosing the covariate:** Not reported.  **Where rationale reported:** NA. | **Where reported:** Protocol and review.  **Reason not reported in review:** NA.  **Reason not reported in protocol:** NA.  **Labelled as ‘posthoc’ if not in protocol:** NA**.** | **Data type:** Categorical.  **Continuous covariate categorised:** NA.  **How determine it was categorised:** NA.  **Justification for categorising:** NA.  **Where justification reported:** NA.  **Categories reported:** Yes.  **Where categories reported:** Review only.  **Justification for categories:** Not reported.  **Where justification reported:** NA. | **Analysis type reported in protocol:** Sensitivity analysis.  **Analysis type reported in review:** Sensitivity analysis.  **Reason for changing mind:** NA.    **Analysis type in protocol according to glossary:** Sensitivity analysis.  **How determined the analysis type:** Says ‘Sensitivity analysis.’ No reason to dispute.  **Analysis type in review according to glossary:** Sensitivity analysis.  **How determined the analysis type:** Categories given.  **Analysed:** Yes  **Reasons given (in the review) for not doing interaction analysis if planned:** NA.  **Reasons given for carrying out interaction analysis if they weren’t planned:** NA.  **Scenario in protocol:** Not reported.  **Rationale for planned scenario:** NA.  **Scenario in review (if reported specifically):** Not reported.  **Rationale for done scenario:** NA.  **Reason for changing mind:** Not reported. | **Method to detect interactions reported in protocol:** Not reported.  **Method to detect interactions reported in review:** ‘compared the results of studies  with a low risk of bias with those of all available studies,’  **Reason for changing mind:** Not reported.. |
| Schoot 2013 | **Protocol:** Not reported.  **Review methods: ‘**RCTs and CCTs were analysed separately’.  **Review results: ‘**RCTs’ versus ‘CCTs’.  **Type:** Methodological.  **Covariate summary:** Trial design. | **Method to choose**  **covariate:** Not reported.  **Where method reported:** NA.  **Rationale for choosing the covariate:** Not reported.  **Where rationale reported:** NA. | **Where reported:** Review only.  **Reason not reported in review:** NA.  **Reason not reported in protocol:** Not reported.  **Labelled as ‘posthoc’ if not in protocol:** No. | **Data type:** Categorical.  **Continuous covariate categorised:** NA.  **How determine it was categorised:** NA.  **Justification for categorising:** NA.  **Where justification reported:** NA.  **Categories reported:** Yes.  **Where categories reported:** Review only.  **Justification for categories:** Not reported.  **Where justification reported:** NA. | **Analysis type reported in protocol:** Unnamed analysis.  **Analysis type reported in review:** Unnamed analysis.  **Reason for changing mind:** NA.    **Analysis type in protocol according to glossary:** Stratification/subgroup analysis.  **How determined the analysis type:** Categories given.  **Analysis type in review according to glossary:** Stratification/subgroup analysis.  **How determined the analysis type:** Categories given.  **Analysed:** Yes.  **Reasons given (in the review) for not doing interaction analysis if planned:** NA  **Reasons given for carrying out interaction analysis if they weren’t planned:** NA.  **Scenario in protocol:** Not reported.  **Rationale for planned scenario:** NA.  **Scenario in review (if reported specifically):** Not reported.  **Rationale for done scenario:** NA.  **Reason for changing mind:** Not reported. | **Method to detect interactions reported in protocol:** Not reported.  **Method to detect interactions reported in review:** Not reported.  **Reason for changing mind:** NA. |
| Semple 2013 | **Protocol: ‘**Duration (short-term (i. e. less than three months), longer term (i. e. more than three months))’.  **Review methods:** ‘Duration: short-term (i. e. less than three months), medium-term (i. e. three to six months) or long-term (more than six months)’.  **Review results:** ‘Medium term (3-6 months)’; ‘end of intervention’.  **Type:** Outcome.  **Covariate summary:** Time point. | **Method to choose**  **covariate:** Not reported.  **Where method reported:** NA.  **Rationale for choosing the covariate:** Not reported.  **Where rationale reported:** NA. | **Where reported:** Protocol and review.  **Reason not reported in review:** NA.  **Reason not reported in protocol:** NA.  **Labelled as ‘posthoc’ if not in protocol:** NA. | **Data type:** Continuous.  **Continuous covariate categorised:** Yes.  **How determine it was categorised:** Categories given.  **Justification for categorising:** Not reported.  **Where justification reported:** NA.  **Categories reported:** Yes.  **Where categories reported:** Protocol and review.  **Justification for categories:** Not reported.  **Where justification reported:** NA. | **Analysis type reported in protocol:** Subgroup analysis.  **Analysis type reported in review:** Subgroup analysis.  **Reason for changing mind:** NA.    **Analysis type in protocol according to glossary:** Subgroup analysis.  **How determined the analysis type:** Categories given.  **Analysis type in review according to glossary:** Subgroup analysis.  **How determined the analysis type:** Categories given.  **Analysed:** Yes.  **Reasons given (in the review) for not doing interaction analysis if planned:** NA.  **Reasons given for carrying out interaction analysis if they weren’t planned:** NA.  **Scenario in protocol:** ‘We will conduct the following subgroup analyses where data are  available:’  **Rationale for planned scenario:** Not reported.  **Scenario in review (if reported specifically): ‘**It was our intention to conduct the following subgroup analyses where data were available.’  **Rationale for done scenario:** Not reported.  **Reason for changing mind:** NA**.** | **Method to detect interactions reported in protocol:** Not reported.  **Method to detect interactions reported in review:** Not reported.  **Reason for changing mind:** NA. |
| Semple 2013 | **Protocol:** ‘Healthcare professional delivering intervention (nurse, psychologist, counsellor, allied health professional)’.  **Review methods: ‘**Healthcare professional delivering intervention (nurse, psychologist, counsellor, allied health professional).’  **Review results:** Not analysed.  **Type:** Intervention.  **Covariate summary:** Type of intervention or control. | **Method to choose**  **covariate:** Not reported.  **Where method reported:** NA.  **Rationale for choosing the covariate:** Not reported.  **Where rationale reported:** NA. | **Where reported:** Protocol and review.  **Reason not reported in review:** NA.  **Reason not reported in protocol:** NA.  **Labelled as ‘posthoc’ if not in protocol:** NA. | **Data type:** Categorical.  **Continuous covariate categorised:** NA.  **How determine it was categorised:** NA.  **Justification for categorising:** NA.  **Where justification reported:** NA.  **Categories reported:** Yes.  **Where categories reported:** Protocol and review.  **Justification for categories:** Not reported.  **Where justification reported:** NA. | **Analysis type reported in protocol:** Subgroup analysis.  **Analysis type reported in review:** Subgroup analysis.  **Reason for changing mind:** NA.    **Analysis type in protocol according to glossary:** Subgroup analysis.  **How determined the analysis type:** Categories given.  **Analysis type in review according to glossary:** Subgroup analysis.  **How determined the analysis type:** Categories given.  **Analysed:** No.  **Reasons given (in the review) for not doing interaction analysis if planned:** ‘When the data had been extracted it was clear that insufficient data were available to conduct these subgroup analyses.’ Due to the small number of studies and the methodological quality of the available studies, we did not perform subgroup analysis.  **Reasons given for carrying out interaction analysis if they weren’t planned:** NA.  **Scenario in protocol:** ‘We will conduct the following subgroup analyses where data are  available:’  **Rationale for planned scenario:** Not reported.  **Scenario in review (if reported specifically): ‘**It was our intention to conduct the following subgroup analyses where data were available.’  **Rationale for done scenario:** Not reported.  **Reason for changing mind:** NA**.** | **Method to detect interactions reported in protocol:** Not reported.  **Method to detect interactions reported in review:** Not reported.  **Reason for changing mind:** NA. |
| Semple 2013 | **Protocol:** ‘If high-risk studies are identified by the ‘Risk of bias’ tool we will perform a sensitivity analysis, including only those studies with suspected low risk of bias.’  **Review methods: ‘**When high-risk studies were identified by the ‘Risk of bias’ tool we will perform a sensitivity analysis, including only those studies with suspected low risk of bias’.  **Review results:** Not analysed.  **Type:** Methodological.  **Covariate summary:** Risk of bias. | **Method to choose**  **covariate:** Not reported.  **Where method reported:** NA.  **Rationale for choosing the covariate:** Not reported.  **Where rationale reported:** NA. | **Where reported:** Protocol and review.  **Reason not reported in review:** NA.  **Reason not reported in protocol:** NA.  **Labelled as ‘posthoc’ if not in protocol:** NA. | **Data type:** Categorical.  **Continuous covariate categorised:** NA.  **How determine it was categorised:** NA.  **Justification for categorising:** NA.  **Where justification reported:** NA.  **Categories reported:** Yes.  **Where categories reported:** Protocol and review.  **Justification for categories:** Not reported.  **Where justification reported:** NA. | **Analysis type reported in protocol:** Sensitivity analysis.  **Analysis type reported in review:** Sensitivity analysis.  **Reason for changing mind:** NA.    **Analysis type in protocol according to glossary:** Sensitivity analysis.  **How determined the analysis type:** Categories given.  **Analysis type in review according to glossary:** Sensitivity analysis.  **How determined the analysis type:** Categories given.  **Analysed:** No.  **Reasons given (in the review) for not doing interaction analysis if planned:** Not reported.  **Reasons given for carrying out interaction analysis if they weren’t planned:** NA.  **Scenario in protocol:** ‘If high-risk studies are identified by the ‘Risk of bias’ tool we will  perform a sensitivity analysis,’  **Rationale for planned scenario:** Not reported.  **Scenario in review (if reported specifically): ‘**When higher-risk studies were identified by the ’Risk of bias’ tool we conducted a sensitivity analysis,’ Due to the small number of studies and the methodological quality of the available studies, we did not perform subgroup analysis.  **Rationale for done scenario:** Not reported.  **Reason for changing mind:** NA**.** | **Method to detect interactions reported in protocol:** Not reported.  **Method to detect interactions reported in review:** Not reported.  **Reason for changing mind:** NA. |
| Semple 2013 | **Protocol: ‘**Method of delivery (individual, group, telephone, computer-assisted).’  **Review methods: ‘**Method of delivery (individual, group, telephone).’  **Review results:** Not analysed.  **Type:** Intervention.  **Covariate summary:** Type of intervention or control. | **Method to choose**  **covariate:** Not reported.  **Where method reported:** NA.  **Rationale for choosing the covariate:** Not reported.  **Where rationale reported:** NA. | **Where reported:** Protocol and review.  **Reason not reported in review:** NA.  **Reason not reported in protocol:** NA.  **Labelled as ‘posthoc’ if not in protocol:** NA. | **Data type:** Categorical.  **Continuous covariate categorised:** NA.  **How determine it was categorised:** NA.  **Justification for categorising:** NA.  **Where justification reported:** NA.  **Categories reported:** Yes.  **Where categories reported:** Protocol and review.  **Justification for categories:** Not reported.  **Where justification reported:** NA. | **Analysis type reported in protocol:** Subgroup analysis.  **Analysis type reported in review:** Subgroup analysis.  **Reason for changing mind:** NA.    **Analysis type in protocol according to glossary:** Subgroup analysis.  **How determined the analysis type:** Categories given.  **Analysis type in review according to glossary:** Subgroup analysis.  **How determined the analysis type:** Categories given.  **Analysed:** No.  **Reasons given (in the review) for not doing interaction analysis if planned:** ‘When the data had been extracted it was clear that insufficient data were available to conduct these subgroup analyses.’ Due to the small number of studies and the methodological quality of the available studies, we did not perform subgroup analysis.  **Reasons given for carrying out interaction analysis if they weren’t planned:** NA.  **Scenario in protocol:** ‘We will conduct the following subgroup analyses where data are  available:’  **Rationale for planned scenario:** Not reported.  **Scenario in review (if reported specifically): ‘**It was our intention to conduct the following subgroup analyses where data were available.’  **Rationale for done scenario:** Not reported.  **Reason for changing mind:** NA**.** | **Method to detect interactions reported in protocol:** Not reported.  **Method to detect interactions reported in review:** Not reported.  **Reason for changing mind:** NA. |
| Semple 2013 | **Protocol:** ‘Stage in cancer journey (potentially curative, palliative)’.  **Review methods: ‘**Stage in cancer journey (potentially curative, palliative)’.  **Review results:** Not analysed.  **Type:** Patient.  **Covariate summary:** Disease characteristics. | **Method to choose**  **covariate:** Not reported.  **Where method reported:** NA.  **Rationale for choosing the covariate:** Not reported.  **Where rationale reported:** NA. | **Where reported:** Protocol and review.  **Reason not reported in review:** NA.  **Reason not reported in protocol:** NA.  **Labelled as ‘posthoc’ if not in protocol:** NA. | **Data type:** Categorical.  **Continuous covariate categorised:** NA.  **How determine it was categorised:** NA.  **Justification for categorising:** NA.  **Where justification reported:** NA.  **Categories reported:** Yes.  **Where categories reported:** Protocol and review.  **Justification for categories:** Not reported.  **Where justification reported:** NA. | **Analysis type reported in protocol:** Subgroup analysis.  **Analysis type reported in review:** Subgroup analysis.  **Reason for changing mind:** NA.    **Analysis type in protocol according to glossary:** Subgroup analysis.  **How determined the analysis type:** Categories given.  **Analysis type in review according to glossary:** Subgroup analysis.  **How determined the analysis type:** Categories given.  **Analysed:** No.  **Reasons given (in the review) for not doing interaction analysis if planned:** ‘When the data had been extracted it was clear that insufficient data were available to conduct these subgroup analyses.’ ‘Due to the small number of studies and the methodological quality of the available studies, we did not perform subgroup analysis.’  **Reasons given for carrying out interaction analysis if they weren’t planned:** NA.  **Scenario in protocol:** ‘We will conduct the following subgroup analyses where data are  available:’  **Rationale for planned scenario:** Not reported.  **Scenario in review (if reported specifically): ‘**It was our intention to conduct the following subgroup analyses where data were available.’  **Rationale for done scenario:** Not reported.  **Reason for changing mind:** NA**.** | **Method to detect interactions reported in protocol:** Not reported.  **Method to detect interactions reported in review:** Not reported.  **Reason for changing mind:** NA. |
| Semple 2013 | **Protocol:** ‘We will conduct a separate analysis on studies by type as described in the Background section (Fawzy 1995). (‘Fawzy 1995 reported on four main types of psychosocial interventions which include: psychoeducational, psychotherapy (individual), cognitive behavioural training and group interventions.’  **Review methods: ‘**Separate analysis on studies by type as described in the Background section (Fawzy 1995). (‘Fawzy 1995 reported on four main types of psychosocial interventions which include: psychoeducational, psychotherapy (individual), cognitive behavioural training and group interventions.’  **Review results:** Not analysed.  **Type:** Intervention.  **Covariate summary:** Type of intervention or control. | **Method to choose**  **covariate:** Not reported.  **Where method reported:** NA.  **Rationale for choosing the covariate:** Not reported.  **Where rationale reported:** NA. | **Where reported:** Protocol and review.  **Reason not reported in review:** NA.  **Reason not reported in protocol:** NA.  **Labelled as ‘posthoc’ if not in protocol:** NA. | **Data type:** Categorical.  **Continuous covariate categorised:** NA.  **How determine it was categorised:** NA.  **Justification for categorising:** NA.  **Where justification reported:** NA.  **Categories reported:** Yes.  **Where categories reported:** Protocol and review.  **Justification for categories:** Not reported.  **Where justification reported:** NA. | **Analysis type reported in protocol:** Subgroup analysis.  **Analysis type reported in review:** Subgroup analysis.  **Reason for changing mind:** NA.    **Analysis type in protocol according to glossary:** Subgroup analysis.  **How determined the analysis type:** Categories given.  **Analysis type in review according to glossary:** Subgroup analysis.  **How determined the analysis type:** Categories given.  **Analysed:** No.  **Reasons given (in the review) for not doing interaction analysis if planned:** ‘When the data had been extracted it was clear that insufficient data were available to conduct these subgroup analyses.’ Due to the small number of studies and the methodological quality of the available studies, we did not perform subgroup analysis.  **Reasons given for carrying out interaction analysis if they weren’t planned:** NA.  **Scenario in protocol:** ‘We will conduct the following subgroup analyses where data are  available:’  **Rationale for planned scenario:** Not reported.  **Scenario in review (if reported specifically): ‘**It was our intention to conduct the following subgroup analyses where data were available.’  **Rationale for done scenario:** Not reported.  **Reason for changing mind:** NA**.** | **Method to detect interactions reported in protocol:** Not reported.  **Method to detect interactions reported in review:** Not reported.  **Reason for changing mind:** NA. |
| Sharma 2013 | **Protocol:** ‘Participant age (children <18 years versus adults)’.  **Review methods: ‘**Participant age (children <18 years versus adults)’.  **Review results:** ‘Adults’; ‘Children’.  **Type:** Patient.  **Covariate summary:** Demographics. | **Method to choose**  **covariate:** Not reported.  **Where method reported:** NA.  **Rationale for choosing the covariate:** Not reported.  **Where rationale reported:** NA. | **Where reported:** Protocol and review.  **Reason not reported in review:** NA.  **Reason not reported in protocol:** NA.  **Labelled as ‘posthoc’ if not in protocol:** NA. | **Data type:** Continuous.  **Continuous covariate categorised:** Yes.  **How determine it was categorised:** Categories given.  **Justification for categorising:** Not reported.  **Where justification reported:** NA.  **Categories reported:** Yes.  **Where categories reported:** Protocol and review.  **Justification for categories:** Not reported.  **Where justification reported:** NA. | **Analysis type reported in protocol:** Subgroup analysis.  **Analysis type reported in review:** Subgroup analysis.  **Reason for changing mind:** NA.    **Analysis type in protocol according to glossary:** Subgroup analysis.  **How determined the analysis type:** Categories given.  **Analysis type in review according to glossary:** Subgroup analysis.  **How determined the analysis type:** Categories given.  **Analysed:** Yes.  **Reasons given (in the review) for not doing interaction analysis if planned:** NA.  **Reasons given for carrying out interaction analysis if they weren’t planned:** NA.  **Scenario in protocol:** ‘We will explore the following potential sources of heterogeneity in  the following subgroup analyses using the primary outcome measure:’  **Rationale for planned scenario:** Not reported.  **Scenario in review (if reported specifically):** When data were available, we explored potential sources of heterogeneity  in the following subgroup analyses for the primary outcome  measure:  **Rationale for done scenario:** Not reported.  **Reason for changing mind:** NA. | **Method to detect interactions reported in protocol:** Not reported.  **Method to detect interactions reported in review:** Not reported.  **Reason for changing mind:** NA. |
| Sharma 2013 | **Protocol: ‘**Presence of underlying systemic or pulmonary diseases (eg silicosis or chronic renal failure).’  **Review methods:** ‘Presence of underlying systemic or pulmonary diseases (eg silicosis or chronic renal failure on haemodialysis);’  **Review results:** Not analysed.  **Type:** Patient.  **Covariate summary:** Disease characteristics. | **Method to choose**  **covariate:** Not reported.  **Where method reported:** NA.  **Rationale for choosing the covariate:** Not reported.  **Where rationale reported:** NA. | **Where reported:** Protocol and review.  **Reason not reported in review:** NA.  **Reason not reported in protocol:** NA.  **Labelled as ‘posthoc’ if not in protocol:** NA. | **Data type:** Categorical.  **Continuous covariate categorised:** NA.  **How determine it was categorised:** NA.  **Justification for categorising:** NA.  **Where justification reported:** NA.  **Categories reported:** Yes.  **Where categories reported:** Protocol and review.  **Justification for categories:** Not reported.  **Where justification reported:** NA. | **Analysis type reported in protocol:** Subgroup analysis.  **Analysis type reported in review:** Subgroup analysis.  **Reason for changing mind:** NA.    **Analysis type in protocol according to glossary:** Subgroup analysis.  **How determined the analysis type:** Categories given.  **Analysis type in review according to glossary:** Subgroup analysis.  **How determined the analysis type:** Categories given.  **Analysed:** No.  **Reasons given (in the review) for not doing interaction analysis if planned:** Not reported.  **Reasons given for carrying out interaction analysis if they weren’t planned:** NA.  **Scenario in protocol:** ‘We will explore the following potential sources of heterogeneity in  the following subgroup analyses using the primary outcome measure:’  **Rationale for planned scenario:** Not reported.  **Scenario in review (if reported specifically):** When data were available, we explored potential sources of heterogeneity  in the following subgroup analyses for the primary outcome  measure:  **Rationale for done scenario:** Not reported.  **Reason for changing mind:** NA. | **Method to detect interactions reported in protocol:** Not reported.  **Method to detect interactions reported in review:** Not reported.  **Reason for changing mind:** NA. |
| Sharma 2013 | **Protocol: ‘**Sensitivity analysis to investigate the robustness of the results to the various components of the risk of bias.  **Review methods:** ‘Where there were sufficient data, we undertook sensitivity analyses to investigate the robustness of the results to the exclusion of trials at high risk of bias.’  **Review results:** Not analysed.  **Type:** Methodological.  **Covariate summary:** Risk of bias | **Method to choose**  **covariate:** Not reported.  **Where method reported:** NA.  **Rationale for choosing the covariate:** Not reported.  **Where rationale reported:** NA. | **Where reported:** Protocol and review.  **Reason not reported in review:** NA.  **Reason not reported in protocol:** NA.  **Labelled as ‘posthoc’ if not in protocol:** NA. | **Data type:** Categorical.  **Continuous covariate categorised:** NA.  **How determine it was categorised:** NA.  **Justification for categorising:** NA.  **Where justification reported:** NA.  **Categories reported:** Yes.  **Where categories reported:** Review only.  **Justification for categories:** Not reported.  **Where justification reported:** NA. | **Analysis type reported in protocol:** Sensitivity analysis.  **Analysis type reported in review:** Sensitivity analysis.  **Reason for changing mind:** NA.    **Analysis type in protocol according to glossary:** Sensitivity analysis.  **How determined the analysis type:** Says ‘Sensitivity analysis.’. No reason to dispute.  **Analysis type in review according to glossary:** Sensitivity analysis.  **How determined the analysis type:** Categories given.  **Analysed:** No.  **Reasons given (in the review) for not doing interaction analysis if planned:** Not reported.  **Reasons given for carrying out interaction analysis if they weren’t planned:** NA.  **Scenario in protocol:** ‘Provided there are sufficient trials,’  **Rationale for planned scenario:** Not reported.  **Scenario in review (if reported specifically):** ‘Where there were sufficient data,’  **Rationale for done scenario:** Not reported.  **Reason for changing mind:** NA. | **Method to detect interactions reported in protocol:** Not reported.  **Method to detect interactions reported in review:** Not reported.  **Reason for changing mind:** NA. |
| Sharma 2013 | **Protocol:** ‘Treatment duration’.  **Review methods: ‘**Treatment duration’.  **Review results:** Not analysed.  **Type:** Intervention.  **Covariate summary:** Duration of intervention. | **Method to choose**  **covariate:** Not reported.  **Where method reported:** NA.  **Rationale for choosing the covariate:** Not reported.  **Where rationale reported:** NA. | **Where reported:** Protocol and review.  **Reason not reported in review:** NA.  **Reason not reported in protocol:** NA.  **Labelled as ‘posthoc’ if not in protocol:** NA. | **Data type:** Continuous.  **Continuous covariate categorised:** Yes.  **How determine it was categorised:** Subgroup analysis planned.  **Justification for categorising:** Not reported.  **Where justification reported:** NA.  **Categories reported:** Not reported.  **Where categories reported:** NA.  **Justification for categories:** Not reported.  **Where justification reported:** NA. | **Analysis type reported in protocol:** Subgroup analysis.  **Analysis type reported in review:** Subgroup analysis.  **Reason for changing mind:** NA.    **Analysis type in protocol according to glossary:** Subgroup analysis.  **How determined the analysis type:** Says ‘subgroup analysis’. No reason to dispute.  **Analysis type in review according to glossary:** Subgroup analysis.  **How determined the analysis type:** Says ‘subgroup analysis’. No reason to dispute.  **Analysed:** No.  **Reasons given (in the review) for not doing interaction analysis if planned:** Not reported.  **Reasons given for carrying out interaction analysis if they weren’t planned:** NA.  **Scenario in protocol:** ‘We will explore the following potential sources of heterogeneity in  the following subgroup analyses using the primary outcome measure:’  **Rationale for planned scenario:** Not reported.  **Scenario in review (if reported specifically):** When data were available, we explored potential sources of heterogeneity  in the following subgroup analyses for the primary outcome  measure:  **Rationale for done scenario:** Not reported.  **Reason for changing mind:** NA. | **Method to detect interactions reported in protocol:** Not reported.  **Method to detect interactions reported in review:** Not reported.  **Reason for changing mind:** NA. |
| Sharma 2013 | **Protocol:** ‘We will stratify the analysis by trial design (individually or cluster randomized)’.  **Review methods:** Not reported.  **Review results:** Not analysed.  **Type:** Methodological.  **Covariate summary:** Trial design. | **Method to choose**  **covariate:** Not reported.  **Where method reported:** NA.  **Rationale for choosing the covariate:** Not reported.  **Where rationale reported:** NA. | **Where reported:** Protocol only.  **Reason not reported in review:** Not reported.  **Reason not reported in protocol:** NA.  **Labelled as ‘posthoc’ if not in protocol:** NA. | **Data type:** Categorical.  **Continuous covariate categorised:** NA.  **How determine it was categorised:** NA.  **Justification for categorising:** NA.  **Where justification reported:** NA.  **Categories reported:** Yes.  **Where categories reported:** Protocol only.  **Justification for categories:** Not reported.  **Where justification reported:** NA. | **Analysis type reported in protocol:** Unnamed analysis.  **Analysis type reported in review:** Not reported.  **Reason for changing mind:** Not reported.    **Analysis type in protocol according to glossary:** Subgroup analysis.  **How determined the analysis type:** Categories given.  **Analysis type in review according to glossary:** Not reported.  **How determined the analysis type:** NA.  **Analysed:** No.  **Reasons given (in the review) for not doing interaction analysis if planned:** Not reported.  **Reasons given for carrying out interaction analysis if they weren’t planned:** NA.  **Scenario in protocol:** Not reported.  **Rationale for planned scenario:** NA.  **Scenario in review (if reported specifically):** Not reported.  **Rationale for done scenario:** NA.  **Reason for changing mind:** NA. | **Method to detect interactions reported in protocol:** Not reported.  **Method to detect interactions reported in review:** Not reported.  **Reason for changing mind:** NA. |
| Sharma 2013 | **Protocol:** Not reported.  **Review methods:** Not reported.  **Review results: ‘**Comparison 1. Rifampicin versus INH: Rifampicin 3 months versus INH 6 months (in adults with silicosis): At 5 years’; ‘Comparison 1. Rifampicin versus INH: Rifampicin 4 months versus INH 6 months (in adult prisoners with LTBI): At 3 years’; ‘Comparison 1. Rifampicin versus INH: Rifampicin 4 months versus INH 6 months (in children): At 2 years’. ‘Comparison 2. Rifampicin plus INH versus INH): INH plus rifampicin 3 months versus INH 6months (in adults with silicosis)’; ‘Comparison 3. Rifampicin plus pyrazinamide versus INH): Rifampicin plus pyrazinamide 2 months versus INH 6 months (in adults)’; ‘Comparison 3. Rifampicin plus pyrazinamide versus INH): Rifampicin plus pyrazinamide 2 months versus INH 6 months (in children)’; ‘Comparison 4. Rifapentine plus INH weekly for 3 months versus INH daily for 9 months’ (in plot). ‘Rifampicin versus INH; Rifampicin plus INH versus INH alone’; ‘Rifampicin plus pyrazinamide versus INH’; ‘Rifapentine plus INH once a week (DOT) for three months versus daily INH daily (self administered) for nine months’ (in text).  **Type:** Intervention.  **Covariate summary:** Type of intervention or control. | **Method to choose**  **covariate:** Not reported.  **Where method reported:** NA.  **Rationale for choosing the covariate:** Not reported.  **Where rationale reported:** NA. | **Where reported:** Review only.  **Reason not reported in review:** NA.  **Reason not reported in protocol:** Not reported.  **Labelled as ‘posthoc’ if not in protocol:** No. | **Data type:** Categorical.  **Continuous covariate categorised:** NA.  **How determine it was categorised:** NA.  **Justification for categorising:** NA.  **Where justification reported:** NA.  **Categories reported:** Yes.  **Where categories reported:** Review only.  **Justification for categories:** Not reported.  **Where justification reported:** NA. | **Analysis type reported in protocol:** Not reported.  **Analysis type reported in review:** Unnamed analysis.  **Reason for changing mind:** Not reported.    **Analysis type in protocol according to glossary:** Not reported.  **How determined the analysis type:** NA.  **Analysis type in review according to glossary:** Subgroup analysis.  **How determined the analysis type:** Categories given.  **Analysed:** Yes.  **Reasons given (in the review) for not doing interaction analysis if planned:** NA.  **Reasons given for carrying out interaction analysis if they weren’t planned:** Not reported.  **Scenario in protocol:** Not reported.  **Rationale for planned scenario:** NA.  **Scenario in review (if reported specifically):** Not reported.  **Rationale for done scenario:** NA.  **Reason for changing mind:** NA. | **Method to detect interactions reported in protocol:** Not reported.  **Method to detect interactions reported in review:** Not reported.  **Reason for changing mind:** NA. |
| Sharma 2013 | **Protocol:** Not reported.  **Review methods:** Not reported.  **Review results:** Time point. 5 years, 3 years.  **Type:** Outcome.  **Covariate summary:** Time point. | **Method to choose**  **covariate:** Not reported.  **Where method reported:** NA.  **Rationale for choosing the covariate:** Not reported.  **Where rationale reported:** NA. | **Where reported:** Review only.  **Reason not reported in review:** NA.  **Reason not reported in protocol:** Not reported.  **Labelled as ‘posthoc’ if not in protocol:** No. | **Data type:** Continuous.  **Continuous covariate categorised:** Yes.  **How determine it was categorised:** Categories given.  **Justification for categorising:** Not reported.  **Where justification reported:** NA.  **Categories reported:** Yes  **Where categories reported:** Review only.  **Justification for categories:** Not reported.  **Where justification reported:** NA. | **Analysis type reported in protocol:** Not reported.  **Analysis type reported in review:** Unnamed analysis.  **Reason for changing mind:** Not reported.    **Analysis type in protocol according to glossary:** Not reported.  **How determined the analysis type:** NA.  **Analysis type in review according to glossary:** Subgroup analysis.  **How determined the analysis type:** Categories given.  **Analysed:** Yes.  **Reasons given (in the review) for not doing interaction analysis if planned:** NA.  **Reasons given for carrying out interaction analysis if they weren’t planned:** Not reported.  **Scenario in protocol:** Not reported.  **Rationale for planned scenario:** NA.  **Scenario in review (if reported specifically):** Not reported.  **Rationale for done scenario:** NA.  **Reason for changing mind:** NA. | **Method to detect interactions reported in protocol:** Not reported.  **Method to detect interactions reported in review:** Not reported.  **Reason for changing mind:** NA. |
| Showell 2013 | **Protocol:** ‘Individual versus combined oral antioxidant therapy.’  **Review methods:** Not reported.  **Review results:** Not analysed.  **Type:** Intervention.  **Covariate summary:** Type of intervention or control. | **Method to choose**  **covariate:** Not reported.  **Where method reported:** NA.  **Rationale for choosing the covariate:** Not reported.  **Where rationale reported:** NA. | **Where reported:** Protocol only.  **Reason not reported in review:** Not reported.  **Reason not reported in protocol:** NA.  **Labelled as ‘posthoc’ if not in protocol:** NA**.** | **Data type:** Categorical.  **Continuous covariate categorised:** NA.  **How determine it was categorised:** NA.  **Justification for categorising:** NA.  **Where justification reported:** NA.  **Categories reported:** Yes.  **Where categories reported:** Protocol only.  **Justification for categories:** Not reported.  **Where justification reported:** NA. | **Analysis type reported in protocol:** Subgroup analysis.  **Analysis type reported in review:** Not reported.  **Reason for changing mind:** Not reported.    **Analysis type in protocol according to glossary:** Subgroup analysis.  **How determined the analysis type:** Categories given.  **Analysis type in review according to glossary:** Not reported.  **How determined the analysis type:** NA.  **Analysed:** No.  **Reasons given (in the review) for not doing interaction analysis if planned:** Not reported.  **Reasons given for carrying out interaction analysis if they weren’t planned:** NA.  **Scenario in protocol:** Not reported.  **Rationale for planned scenario:** NA.  **Scenario in review (if reported specifically):** Not reported.  **Rationale for done scenario:** NA.  **Reason for changing mind:** NA. | **Method to detect interactions reported in protocol:** Not reported.  **Method to detect interactions reported in review:** Not reported.  **Reason for changing mind:** NA. |
| Showell 2013 | **Protocol:** ‘Poor prognostic factors (for example age).’  **Review methods:** Not reported.  **Review results:** Not analysed.  **Type:** Patient.  **Covariate summary:** Disease characteristics. | **Method to choose**  **covariate:** Not reported.  **Where method reported:** NA.  **Rationale for choosing the covariate:** Not reported.  **Where rationale reported:** NA. | **Where reported:** Protocol only.  **Reason not reported in review:** Not reported.  **Reason not reported in protocol:** NA.  **Labelled as ‘posthoc’ if not in protocol:** NA**.** | **Data type:** Unclear.  **Continuous covariate categorised:** NA.  **How determine it was categorised:** NA.  **Justification for categorising:** NA.  **Where justification reported:** NA.  **Categories reported:** Not reported.  **Where categories reported:** NA.  **Justification for categories:** Not reported.  **Where justification reported:** NA. | **Analysis type reported in protocol:** Subgroup analysis.  **Analysis type reported in review:** Not reported.  **Reason for changing mind:** Not reported.    **Analysis type in protocol according to glossary:** Subgroup analysis.  **How determined the analysis type:** Says ‘Subgroup analysis’. No reason to dispute.  **Analysis type in review according to glossary:** Not reported.  **How determined the analysis type:** NA.  **Analysed:** No.  **Reasons given (in the review) for not doing interaction analysis if planned:** Not reported.  **Reasons given for carrying out interaction analysis if they weren’t planned:** NA.  **Scenario in protocol:** Not reported.  **Rationale for planned scenario:** NA.  **Scenario in review (if reported specifically):** Not reported.  **Rationale for done scenario:** NA.  **Reason for changing mind:** NA. | **Method to detect interactions reported in protocol:** Not reported.  **Method to detect interactions reported in review:** Not reported.  **Reason for changing mind:** NA. |
| Showell 2013 | **Protocol:** ‘Poor responders for ART cycles (women with one previous failed cycle).’  **Review methods:** Not reported.  **Review results:** Not analysed.  **Type:** Patient.  **Covariate summary:** Disease characteristics. | **Method to choose**  **covariate:** Not reported.  **Where method reported:** NA.  **Rationale for choosing the covariate:** Not reported.  **Where rationale reported:** NA. | **Where reported:** Protocol only.  **Reason not reported in review:** Not reported.  **Reason not reported in protocol:** NA.  **Labelled as ‘posthoc’ if not in protocol:** NA**.** | **Data type:** Categorical.  **Continuous covariate categorised:** NA.  **How determine it was categorised:** NA.  **Justification for categorising:** NA.  **Where justification reported:** NA.  **Categories reported:** Not reported.  **Where categories reported:** NA.  **Justification for categories:** Not reported.  **Where justification reported:** NA. | **Analysis type reported in protocol:** Subgroup analysis.  **Analysis type reported in review:** Not reported.  **Reason for changing mind:** Not reported.    **Analysis type in protocol according to glossary:** Sensitivity analysis.  **How determined the analysis type:** Categories given.  **Analysis type in review according to glossary:** Not reported.  **How determined the analysis type:** NA.  **Analysed:** No.  **Reasons given (in the review) for not doing interaction analysis if planned:** Not reported.  **Reasons given for carrying out interaction analysis if they weren’t planned:** NA.  **Scenario in protocol:** Not reported.  **Rationale for planned scenario:** NA.  **Scenario in review (if reported specifically):** Not reported.  **Rationale for done scenario:** NA.  **Reason for changing mind:** NA. | **Method to detect interactions reported in protocol:** Not reported.  **Method to detect interactions reported in review:** Not reported.  **Reason for changing mind:** NA. |
| Showell 2013 | **Protocol:** ‘Study quality- studies that were allocated the lowest quality score.’  **Review methods:** Not reported.  **Review results:** Not analysed.  **Type:** Methodological.  **Covariate summary:** Quality. | **Method to choose**  **covariate:** Not reported.  **Where method reported:** NA.  **Rationale for choosing the covariate:** Not reported.  **Where rationale reported:** NA. | **Where reported:** Protocol only.  **Reason not reported in review:** Not reported.  **Reason not reported in protocol:** NA.  **Labelled as ‘posthoc’ if not in protocol:** NA**.** | **Data type:** Categorical.  **Continuous covariate categorised:** NA.  **How determine it was categorised:** NA.  **Justification for categorising:** NA.  **Where justification reported:** NA.  **Categories reported:** Yes.  **Where categories reported:** Protocol only.  **Justification for categories:** Not reported.  **Where justification reported:** NA. | **Analysis type reported in protocol:** Sensitivity analysis.  **Analysis type reported in review:** Not reported.  **Reason for changing mind:** Not reported.    **Analysis type in protocol according to glossary:** Sensitivity analysis.  **How determined the analysis type:** Categories given.  **Analysis type in review according to glossary:** Not reported.  **How determined the analysis type:** NA.  **Analysed:** No.  **Reasons given (in the review) for not doing interaction analysis if planned:** Not reported.  **Reasons given for carrying out interaction analysis if they weren’t planned:** NA.  **Scenario in protocol:** Not reported.  **Rationale for planned scenario:** NA.  **Scenario in review (if reported specifically):** Not reported.  **Rationale for done scenario:** NA.  **Reason for changing mind:** NA. | **Method to detect interactions reported in protocol:** Not reported.  **Method to detect interactions reported in review:** Not reported.  **Reason for changing mind:** NA. |
| Showell 2013 | **Protocol:** ‘Unpublished studies- these may not have been subjected to the peer review process and may have intrinsic bias issues.’  **Review methods:** Not reported.  **Review results:** Not analysed.  **Type:** Methodological.  **Covariate summary:** Publication status. | **Method to choose**  **covariate:** Not reported.  **Where method reported:** NA.  **Rationale for choosing the covariate:** Not reported.  **Where rationale reported:** NA. | **Where reported:** Protocol only.  **Reason not reported in review:** Not reported.  **Reason not reported in protocol:** NA.  **Labelled as ‘posthoc’ if not in protocol:** NA**.** | **Data type:** Categorical.  **Continuous covariate categorised:** NA.  **How determine it was categorised:** NA.  **Justification for categorising:** NA.  **Where justification reported:** NA.  **Categories reported:** Yes.  **Where categories reported:** Protocol only.  **Justification for categories:** Not reported.  **Where justification reported:** NA. | **Analysis type reported in protocol:** Sensitivity analysis.  **Analysis type reported in review:** Not reported.  **Reason for changing mind:** Not reported.    **Analysis type in protocol according to glossary:** Sensitivity analysis.  **How determined the analysis type:** Categories given.  **Analysis type in review according to glossary:** Not reported.  **How determined the analysis type:** NA.  **Analysed:** No.  **Reasons given (in the review) for not doing interaction analysis if planned:** Not reported.  **Reasons given for carrying out interaction analysis if they weren’t planned:** NA.  **Scenario in protocol:** Not reported.  **Rationale for planned scenario:** NA.  **Scenario in review (if reported specifically):** Not reported.  **Rationale for done scenario:** NA.  **Reason for changing mind:** NA. | **Method to detect interactions reported in protocol:** Not reported.  **Method to detect interactions reported in review:** Not reported.  **Reason for changing mind:** NA. |
| Showell 2013 | **Protocol:** Not reported.  **Review methods: ‘**The following subgroup analysess were carried out: Antioxidants versus control (placebo or no treatment/standard treatment); Antioxidants versus antioxidants or head-to-head stratification by type of antioxidant; and Pentoxifylline versus control (placebo or no treatment/standard treatment).’  **Review results: ‘**Comparison 1. Antioxidant(s) versus placebo or no treatment/standard treatment;’ ‘Comparison 2. Head to head antioxidants’; ‘Comparison 3. Pentoxifylline versus placebo or no treatment/standard care’.  **Type:** Intervention.  **Covariate summary:** Type of intervention or control. | **Method to choose**  **covariate:** Not reported.  **Where method reported:** NA.  **Rationale for choosing the covariate:** ‘‘On clinical advice, trials that used folic acid (standard treatment)  and those that included a co-intervention (a fertility drug such as  clomiphene citrate or metformin) in both arms were analysed in  the antioxidant versus placebo or no treatment/standard treatment  comparison and not in the head-to-head comparison, as the controls  were not considered to be active treatments. Pentoxifylline  trials were analysed as a separate comparison as it was not possible  to separate the antioxidant effects from the other medical effects  of the drug.’’  **Where rationale reported:** Review only.. | **Where reported:** Review only.  **Reason not reported in review:** NA.  **Reason not reported in protocol:** Not reported.  **Labelled as ‘posthoc’ if not in protocol:** No**.** | **Data type:** Categorical.  **Continuous covariate categorised:** NA.  **How determine it was categorised:** NA.  **Justification for categorising:** NA.  **Where justification reported:** NA.  **Categories reported:** Yes.  **Where categories reported:** Review only.  **Justification for categories:** Not reported.  **Where justification reported:** NA. | **Analysis type reported in protocol:** Not reported.  **Analysis type reported in review:** unnamed analysis.  **Reason for changing mind:** Not reported.    **Analysis type in protocol according to glossary:** Not reported.  **How determined the analysis type:** NA.  **Analysis type in review according to glossary:** Subgroup analysis.  **How determined the analysis type:** Categories given.  **Analysed:** Yes.  **Reasons given (in the review) for not doing interaction analysis if planned:** NA.  **Reasons given for carrying out interaction analysis if they weren’t planned:** Not reported.  **Scenario in protocol:** Not reported.  **Rationale for planned scenario:** NA.  **Scenario in review (if reported specifically):** Not reported.  **Rationale for done scenario:** NA.  **Reason for changing mind:** NA. | **Method to detect interactions reported in protocol:** Not reported.  **Method to detect interactions reported in review:** Not reported.  **Reason for changing mind:** NA. |
| Showell 2013 | **Protocol:** Not reported.  **Review methods: ‘**Type of antioxidant, whether individual or combined (three or more antioxidants combined).’  **Review results: ‘**Type of antioxidant (N-acetyl-cysteine , L-arginine , Myo-Inositol versus d-chiro-inositol1, Pentoxifylline plus vitamin E)’.  **Type:** Intervention.  **Covariate summary:** Type of intervention or control. | **Method to choose**  **covariate:** Not reported.  **Where method reported:** NA.  **Rationale for choosing the covariate:** Not reported.  **Where rationale reported:** NA. | **Where reported:** Review only.  **Reason not reported in review:** NA.  **Reason not reported in protocol:** Not reported.  **Labelled as ‘posthoc’ if not in protocol:** No**.** | **Data type:** Categorical.  **Continuous covariate categorised:** NA.  **How determine it was categorised:** NA.  **Justification for categorising:** NA.  **Where justification reported:** NA.  **Categories reported:** Yes.  **Where categories reported:** Review only.  **Justification for categories:** Not reported.  **Where justification reported:** NA. | **Analysis type reported in protocol:** Not reported.  **Analysis type reported in review:** Subgroup analysis.  **Reason for changing mind:** Not reported.    **Analysis type in protocol according to glossary:** Not reported.  **How determined the analysis type:** NA.  **Analysis type in review according to glossary:** Subgroup analysis.  **How determined the analysis type:** Categories given.  **Analysed:** Yes.  **Reasons given (in the review) for not doing interaction analysis if planned:** NA.  **Reasons given for carrying out interaction analysis if they weren’t planned:** Not reported.  **Scenario in protocol:** Not reported.  **Rationale for planned scenario:** NA.  **Scenario in review (if reported specifically):** Not reported.  **Rationale for done scenario:** NA.  **Reason for changing mind:** NA. | **Method to detect interactions reported in protocol:** Not reported.  **Method to detect interactions reported in review:** Not reported.  **Reason for changing mind:** NA. |
| Showell 2013 | **Protocol:** Not reported.  **Review methods: ‘**Trials that enrolled women with different indications for infertility (i. e. PCOS, endometriosis, unexplained infertility or poor responders).’  **Review results:** ‘Indications for subfertility (Polycystic ovary syndrome, Tubal subfertility, Varying indications)’.  **Type:** Patient.  **Covariate summary:** Disease characteristics. | **Method to choose**  **covariate:** Not reported.  **Where method reported:** NA.  **Rationale for choosing the covariate:** Not reported.  **Where rationale reported:** NA. | **Where reported:** Review only.  **Reason not reported in review:** NA.  **Reason not reported in protocol:** Not reported.  **Labelled as ‘posthoc’ if not in protocol:** No**.** | **Data type:** Categorical.  **Continuous covariate categorised:** NA.  **How determine it was categorised:** NA.  **Justification for categorising:** NA.  **Where justification reported:** NA.  **Categories reported:** Yes.  **Where categories reported:** Review only.  **Justification for categories:** Not reported.  **Where justification reported:** NA. | **Analysis type reported in protocol:** Not reported.  **Analysis type reported in review:** Subgroup analysis.  **Reason for changing mind:** Not reported.    **Analysis type in protocol according to glossary:** Not reported.  **How determined the analysis type:** NA.  **Analysis type in review according to glossary:** Subgroup analysis.  **How determined the analysis type:** Categories given.  **Analysed:** Yes.  **Reasons given (in the review) for not doing interaction analysis if planned:** NA.  **Reasons given for carrying out interaction analysis if they weren’t planned:** Not reported.  **Scenario in protocol:** Not reported.  **Rationale for planned scenario:** NA.  **Scenario in review (if reported specifically):** Not reported.  **Rationale for done scenario:** NA.  **Reason for changing mind:** NA. | **Method to detect interactions reported in protocol:** Not reported.  **Method to detect interactions reported in review:** Not reported.  **Reason for changing mind:** NA. |
| Showell 2013 | **Protocol:** Not reported.  **Review methods: ‘**Trials that enrolled women who were also undergoing IVF or ICSI.’  **Review results:** ‘IVF/ICSI’.  **Type:** Patient.  **Covariate summary:** Disease characteristics. | **Method to choose**  **covariate:** Not reported.  **Where method reported:** NA.  **Rationale for choosing the covariate:** Not reported.  **Where rationale reported:** NA. | **Where reported:** Review only.  **Reason not reported in review:** NA.  **Reason not reported in protocol:** Not reported.  **Labelled as ‘posthoc’ if not in protocol:** No**.** | **Data type:** Categorical.  **Continuous covariate categorised:** NA.  **How determine it was categorised:** NA.  **Justification for categorising:** NA.  **Where justification reported:** NA.  **Categories reported:** Yes.  **Where categories reported:** Review only.  **Justification for categories:** Not reported.  **Where justification reported:** NA. | **Analysis type reported in protocol:** Not reported.  **Analysis type reported in review:** Subgroup analysis.  **Reason for changing mind:** Not reported.    **Analysis type in protocol according to glossary:** Not reported.  **How determined the analysis type:** NA.  **Analysis type in review according to glossary:** Subgroup analysis.  **How determined the analysis type:** Categories given.  **Analysed:** Yes.  **Reasons given (in the review) for not doing interaction analysis if planned:** NA.  **Reasons given for carrying out interaction analysis if they weren’t planned:** Not reported.  **Scenario in protocol:** Not reported.  **Rationale for planned scenario:** NA.  **Scenario in review (if reported specifically):** Not reported.  **Rationale for done scenario:** NA.  **Reason for changing mind:** NA. | **Method to detect interactions reported in protocol:** Not reported.  **Method to detect interactions reported in review:** Not reported.  **Reason for changing mind:** NA. |
| Showell 2013 | **Protocol:** Not reported.  **Review methods:** ‘Excluding studies: that used antioxidants plus a fertility drug (a cointervention) versus placebo plus a fertility drug’.  **Review results:** Not analysed.  **Type:** Intervention.  **Covariate summary:** Additional interventions. | **Method to choose**  **covariate:** Not reported.  **Where method reported:** NA.  **Rationale for choosing the covariate:** Not reported.  **Where rationale reported:** NA. | **Where reported:** Review only.  **Reason not reported in review:** NA.  **Reason not reported in protocol:** Not reported.  **Labelled as ‘posthoc’ if not in protocol:** No**.** | **Data type:** Categorical.  **Continuous covariate categorised:** NA.  **How determine it was categorised:** NA.  **Justification for categorising:** NA.  **Where justification reported:** NA.  **Categories reported:** Yes.  **Where categories reported:** Review only.  **Justification for categories:** Not reported.  **Where justification reported:** NA. | **Analysis type reported in protocol:** Not reported.  **Analysis type reported in review:** Sensitivity analysis.  **Reason for changing mind:** Not reported.    **Analysis type in protocol according to glossary:** Not reported.  **How determined the analysis type:** NA.  **Analysis type in review according to glossary:** Subgroup analysis.  **How determined the analysis type:** Categories given.  **Analysed:** No.  **Reasons given (in the review) for not doing interaction analysis if planned:** Not reported.  **Reasons given for carrying out interaction analysis if they weren’t planned:** NA.  **Scenario in protocol:** Not reported.  **Rationale for planned scenario:** NA.  **Scenario in review (if reported specifically):** ‘Sensitivity analyses (using the random-effects model in RevMan  software)were performed on the primary outcomes if a high degree  of heterogeneity was noted (where the I2 statistic was ≥ 50%),  excluding studies:’  **Rationale for done scenario:** Not reported.  **Reason for changing mind:** NA. | **Method to detect interactions reported in protocol:** Not reported.  **Method to detect interactions reported in review:** Not reported.  **Reason for changing mind:** NA. |
| Showell 2013 | **Protocol:** Not reported.  **Review methods: ‘**Excluding studies: that used antioxidants plus folic acid versus standard treatment (folic acid < 1 mg).’  **Review results:** Not analysed.  **Type:** Intervention.  **Covariate summary:** Additional interventions. | **Method to choose**  **covariate:** Not reported.  **Where method reported:** NA.  **Rationale for choosing the covariate:** Not reported.  **Where rationale reported:** NA. | **Where reported:** Review only.  **Reason not reported in review:** NA.  **Reason not reported in protocol:** Not reported.  **Labelled as ‘posthoc’ if not in protocol:** No**.** | **Data type:** Categorical.  **Continuous covariate categorised:** NA.  **How determine it was categorised:** NA.  **Justification for categorising:** NA.  **Where justification reported:** NA.  **Categories reported:** Yes.  **Where categories reported:** Review only.  **Justification for categories:** Not reported.  **Where justification reported:** NA. | **Analysis type reported in protocol:** Not reported.  **Analysis type reported in review:** Sensitivity analysis.  **Reason for changing mind:** Not reported.    **Analysis type in protocol according to glossary:** Not reported.  **How determined the analysis type:** NA.  **Analysis type in review according to glossary:** Subgroup analysis.  **How determined the analysis type:** Categories given.  **Analysed:** No.  **Reasons given (in the review) for not doing interaction analysis if planned:** Not reported.  **Reasons given for carrying out interaction analysis if they weren’t planned:** NA.  **Scenario in protocol:** Not reported.  **Rationale for planned scenario:** NA.  **Scenario in review (if reported specifically):** ‘Sensitivity analyses (using the random-effects model in RevMan  software)were performed on the primary outcomes if a high degree  of heterogeneity was noted (where the I2 statistic was ≥ 50%),  excluding studies:’  **Rationale for done scenario:** Not reported.  **Reason for changing mind:** NA. | **Method to detect interactions reported in protocol:** Not reported.  **Method to detect interactions reported in review:** Not reported.  **Reason for changing mind:** NA. |
| Showell 2013 | **Protocol:** Not reported.  **Review methods: ‘**Excluding studies: with a high risk of bias.’  **Review results:** Not analysed.  **Type:** Methodological.  **Covariate summary:** Risk of bias. | **Method to choose**  **covariate:** Not reported.  **Where method reported:** NA.  **Rationale for choosing the covariate:** Not reported.  **Where rationale reported:** NA. | **Where reported:** Review only.  **Reason not reported in review:** NA.  **Reason not reported in protocol:** Not reported.  **Labelled as ‘posthoc’ if not in protocol:** No**.** | **Data type:** Categorical.  **Continuous covariate categorised:** NA.  **How determine it was categorised:** NA.  **Justification for categorising:** NA.  **Where justification reported:** NA.  **Categories reported:** Yes.  **Where categories reported:** Review only.  **Justification for categories:** Not reported.  **Where justification reported:** NA. | **Analysis type reported in protocol:** Not reported.  **Analysis type reported in review:** Sensitivity analysis.  **Reason for changing mind:** Not reported.    **Analysis type in protocol according to glossary:** Not reported.  **How determined the analysis type:** NA.  **Analysis type in review according to glossary:** Sensitivity analysis.  **How determined the analysis type:** Categories given.  **Analysed:** No.  **Reasons given (in the review) for not doing interaction analysis if planned:** Not reported.  **Reasons given for carrying out interaction analysis if they weren’t planned:** NA.  **Scenario in protocol:** Not reported.  **Rationale for planned scenario:** NA.  **Scenario in review (if reported specifically):** ‘Sensitivity analyses (using the random-effects model in RevMan  software)were performed on the primary outcomes if a high degree  of heterogeneity was noted (where the I2 statistic was ≥ 50%),  excluding studies:’  **Rationale for done scenario:** Not reported.  **Reason for changing mind:** NA. | **Method to detect interactions reported in protocol:** Not reported.  **Method to detect interactions reported in review:** Not reported.  **Reason for changing mind:** NA. |
| Stead 2012 | **Protocol:** ‘In addition we will note any other abstinence outcomes reported and conducted sensitivity analyses if the choice of outcome in a study might have altered the results of a meta-analysis.’  **Review methods: ‘**In addition we noted any other abstinence outcomes reported and conducted sensitivity analyses if the choice of outcome in a study might have altered the results of a meta-analysis.’  **Review results:** Not analysed.  **Type:** Outcome.  **Covariate summary:** Studies that report other abstinence outcomes. | **Method to choose**  **covariate:** Not reported.  **Where method reported:** NA.  **Rationale for choosing the covariate:** Not reported.  **Where rationale reported:** NA. | **Where reported:** Protocol and review.  **Reason not reported in review:** NA.  **Reason not reported in protocol:** NA.  **Labelled as ‘posthoc’ if not in protocol:** NA. | **Data type:** Unclear.  **Continuous covariate categorised:** NA.  **How determine it was categorised:** NA.  **Justification for categorising:** NA.  **Where justification reported:** NA.  **Categories reported:** Not reported.  **Where categories reported:** NA.  **Justification for categories:** Not reported.  **Where justification reported:** NA. | **Analysis type reported in protocol:** Sensitivity analysis.  **Analysis type reported in review:** Sensitivity analysis.  **Reason for changing mind:** NA.    **Analysis type in protocol according to glossary:** Sensitivity analysis.  **How determined the analysis type:** Says ‘sensitivity analysis’. No reason to dispute.  **Analysis type in review according to glossary:** Sensitivity analysis.  **How determined the analysis type:** Says ‘sensitivity analysis’. No reason to dispute.  **Analysed:** No.  **Reasons given (in the review) for not doing interaction analysis if planned:** Not reported.  **Reasons given for carrying out interaction analysis if they weren’t planned:** NA.  **Scenario in protocol:** ‘if the choice of outcome in a study might have altered  the results of a meta-analysis’  **Rationale for planned scenario:** Not reported.  **Scenario in review (if reported specifically):** ‘if the choice of outcome in a study might have altered  the results of a meta-analysis’  **Rationale for done scenario:** Not reported.  **Reason for changing mind:** NA. | **Method to detect interactions reported in protocol:** Not reported.  **Method to detect interactions reported in review:** Not reported.  **Reason for changing mind:** NA. |
| Stead 2012 | **Protocol:** ‘We will categorise the intensity of behavioural support in both intervention and control conditions based on two of the categories used in the US Guidelines (Fiore 2008); ‘Total amount of contact time’ (Categories: 1-3, 4-30, 31-90, 91-300, >300 minutes) and ‘Number of person to person sessions’ (Categories: 0*, 1-3*, 4-8, >8 [*guideline used 0-1, 2-3])’. ‘We will use the relative intensity of support (number or duration of contacts) as the main potential feature to explain any heterogeneity.’  **Review methods: ‘**We categorise the intensity of behavioural support in both intervention and control conditions based on two of the categories used in the US Guidelines (Fiore 2008): ‘Total amount of contact time’ (Categories: 0, 1 to 30*, 31 to 90, 91 to 300, > 300 minutes [*guideline categories 1 to 3 and 4-30 combined]) and ‘Number of person-to-person sessions’ (Categories: 0*, 1 to 3*, 4 to 8, > 8 [*guideline used 0 to 1, 2 to 3]).’ ‘We used the difference in intensity of support (number or duration of contacts) between intervention and control conditions as the main potential feature to explain any heterogeneity.’  **Review results:** Subgroups by ‘contrast in number of contacts between intervention & control’. ‘4 to 8 or > 8 contacts versus no contact’; ‘More than 8 contacts versus 1 to 3 contacts’; ‘4 to 8 contacts versus 1 to 3 contacts’; ‘More than 8 contacts versus 4-8 contacts’; ‘Intervention & control in same contact category’).  **Type:** Intervention.  **Covariate summary:** Intervention intensity. | **Method to choose**  **covariate:** Not reported.  **Where method reported:** NA.  **Rationale for choosing the covariate:** ‘We would expect trials testing larger differences in intensity, that is using longer, more frequent sessions in the intervention condition and brief contact in the control, to show larger treatment effects.’  **Where rationale reported:** Protocol and review. | **Where reported:** Protocol and review.  **Reason not reported in review:** NA.  **Reason not reported in protocol:** NA.  **Labelled as ‘posthoc’ if not in protocol:** NA. | **Data type:** Continuous.  **Continuous covariate categorised:** Yes.  **How determine it was categorised:** Subgroup analysis planned.  **Justification for categorising:** Not reported.  **Where justification reported:** NA.  **Categories reported:** Not reported.  **Where categories reported:** NA.  **Justification for categories:** Not reported.  **Where justification reported:** NA. | **Analysis type reported in protocol:** Subgroup analysis.  **Analysis type reported in review:** Subgroup analysis.  **Reason for changing mind:** NA.    **Analysis type in protocol according to glossary:** Subgroup analysis.  **How determined the analysis type:** Says ‘subgroup analysis.’ No reason to dispute.  **Analysis type in review according to glossary:** Subgroup analysis.  **How determined the analysis type:** Says ‘subgroup analysis.’ No reason to dispute.  **Analysed:** Yes.  **Reasons given (in the review) for not doing interaction analysis if planned:** NA.  **Reasons given for carrying out interaction analysis if they weren’t planned:** NA  **Scenario in protocol:** Not reported.  **Rationale for planned scenario:** NA.  **Scenario in review (if reported specifically):** Not reported.  **Rationale for done scenario:** NA.  **Reason for changing mind:** NA. | **Method to detect interactions reported in protocol:** Not reported.  **Method to detect interactions reported in review:** Not reported.  **Reason for changing mind:** NA. |
| Stead 2012 | **Protocol:** ‘We will consider whether the results are sensitive to the exclusion of trials judged to have a high risk of bias.’  **Review methods: ‘**Had we judged any trials to be at high risk of bias in any domain, we would have considered whether the results were sensitive to their exclusion.’  **Review results:** Not reported.  **Type:** Methodological.  **Covariate summary:** Risk of bias. | **Method to choose**  **covariate:** Not reported.  **Where method reported:** NA.  **Rationale for choosing the covariate:** Not reported.  **Where rationale reported:** NA. | **Where reported:** Protocol and review.  **Reason not reported in review:** NA.  **Reason not reported in protocol:** NA.  **Labelled as ‘posthoc’ if not in protocol:** NA. | **Data type:** Categorical.  **Continuous covariate categorised:** NA.  **How determine it was categorised:** NA.  **Justification for categorising:** NA.  **Where justification reported:** NA.  **Categories reported:** Yes.  **Where categories reported:** Protocol and review.  **Justification for categories:** Not reported.  **Where justification reported:** NA. | **Analysis type reported in protocol:** Sensitivity analysis.  **Analysis type reported in review:** Sensitivity analysis.  **Reason for changing mind:** NA.    **Analysis type in protocol according to glossary:** Sensitivity analysis.  **How determined the analysis type:** Categories given.  **Analysis type in review according to glossary:** Sensitivity analysis.  **How determined the analysis type:** Categories given.  **Analysed:** No.  **Reasons given (in the review) for not doing interaction analysis if planned:** Not reported.  **Reasons given for carrying out interaction analysis if they weren’t planned:** NA.  **Scenario in protocol:** Not reported.  **Rationale for planned scenario:** NA.  **Scenario in review (if reported specifically):** Not reported.  **Rationale for done scenario:** NA.  **Reason for changing mind:** NA. | **Method to detect interactions reported in protocol:** Not reported.  **Method to detect interactions reported in review:** Not reported.  **Reason for changing mind:** NA. |
| Stead 2012 | **Protocol:** Definition of follow up. We judged the following to be three covariates: ‘We will also consider whether the definition and duration of follow up, or the use of biochemical validation of cessation, has any impact on treatment effect.’  **Review methods:** Definition of follow up. We judged the following to be three covariates: ‘We also considered whether the definition and duration of follow-up, or the use of biochemical validation of cessation, had any impact on treatment effect.’  **Review results:** Not analysed.  **Type:** Methodological.  **Covariate summary:** Definition of follow up. | **Method to choose**  **covariate:** Not reported.  **Where method reported:** NA.  **Rationale for choosing the covariate:** Not reported.  **Where rationale reported:** NA. | **Where reported:** Protocol and review.  **Reason not reported in review:** NA.  **Reason not reported in protocol:** NA.  **Labelled as ‘posthoc’ if not in protocol:** NA. | **Data type:** Categorical.  **Continuous covariate categorised:** NA.  **How determine it was categorised:** NA.  **Justification for categorising:** NA.  **Where justification reported:** NA.  **Categories reported:** Not reported.  **Where categories reported:** NA.  **Justification for categories:** Not reported.  **Where justification reported:** NA. | **Analysis type reported in protocol:** Subgroup analysis.  **Analysis type reported in review:** Subgroup analysis.  **Reason for changing mind:** NA.    **Analysis type in protocol according to glossary:** Subgroup analysis.  **How determined the analysis type:** Says ‘subgroup analysis.’ No reason to dispute.  **Analysis type in review according to glossary:** Subgroup analysis.  **How determined the analysis type:** Says ‘subgroup analysis.’ No reason to dispute.  **Analysed:** No.  **Reasons given (in the review) for not doing interaction analysis if planned:** Not reported.  **Reasons given for carrying out interaction analysis if they weren’t planned:** NA.  **Scenario in protocol:** Not reported.  **Rationale for planned scenario:** NA.  **Scenario in review (if reported specifically):** Not reported.  **Rationale for done scenario:** NA.  **Reason for changing mind:** NA. | **Method to detect interactions reported in protocol:** Not reported.  **Method to detect interactions reported in review:** Not reported.  **Reason for changing mind:** NA. |
| Stead 2012 | **Protocol:** Duration of follow up. We judged the following to be three covariates: ‘We will also consider whether the definition and duration of follow up, or the use of biochemical validation of cessation, has any impact on treatment effect.’  **Review methods:** Duration of follow up. We judged the following to be three covariates: ‘We also considered whether the definition and duration of follow-up, or the use of biochemical validation of cessation, had any impact on treatment effect.’  **Review results:** Not analysed.  **Type:** Methodological.  **Covariate summary:** Length of follow up. | **Method to choose**  **covariate:** Not reported.  **Where method reported:** NA.  **Rationale for choosing the covariate:** Not reported.  **Where rationale reported:** NA. | **Where reported:** Protocol and review.  **Reason not reported in review:** NA.  **Reason not reported in protocol:** NA.  **Labelled as ‘posthoc’ if not in protocol:** NA. | **Data type:** Continuous.  **Continuous covariate categorised:** Yes.  **How determine it was categorised:** Subgroup analysis planned.  **Justification for categorising:** Not reported.  **Where justification reported:** NA.  **Categories reported:** Not reported.  **Where categories reported:** NA.  **Justification for categories:** Not reported.  **Where justification reported:** NA. | **Analysis type reported in protocol:** Subgroup analysis.  **Analysis type reported in review:** Subgroup analysis.  **Reason for changing mind:** NA.    **Analysis type in protocol according to glossary:** Subgroup analysis.  **How determined the analysis type:** Says ‘subgroup analysis.’ No reason to dispute.  **Analysis type in review according to glossary:** Subgroup analysis.  **How determined the analysis type:** Says ‘subgroup analysis.’ No reason to dispute.  **Analysed:** No.  **Reasons given (in the review) for not doing interaction analysis if planned:** Not reported.  **Reasons given for carrying out interaction analysis if they weren’t planned:** NA.  **Scenario in protocol:** Not reported.  **Rationale for planned scenario:** NA.  **Scenario in review (if reported specifically):** Not reported.  **Rationale for done scenario:** NA.  **Reason for changing mind:** NA. | **Method to detect interactions reported in protocol:** Not reported.  **Method to detect interactions reported in review:** Not reported.  **Reason for changing mind:** NA. |
| Stead 2012 | **Protocol:** Not reported.  **Review methods:** Not reported.  **Review results: ‘**Duration of contact in control condition’. ‘Brief intervention’ for control’; ‘Dose response’, ‘over 30 minutes contact for control’).  **Type:** Intervention  **Covariate summary:** Duration of intervention. | **Method to choose**  **covariate:** Not reported.  **Where method reported:** NA.  **Rationale for choosing the covariate:** Not reported.  **Where rationale reported:** NA. | **Where reported:** Review only.  **Reason not reported in review:** NA.  **Reason not reported in protocol:** ‘At the suggestion of a peer reviewer we did two additional subgroup  analyses.’  **Labelled as ‘posthoc’ if not in protocol:** Yes (‘not prespecified’). | **Data type:** Continuous.  **Continuous covariate categorised:** Yes.  **How determine it was categorised:** Categories given.  **Justification for categorising:** Not reported.  **Where justification reported:** NA.  **Categories reported:** Yes.  **Where categories reported:** Review only.  **Justification for categories:** Not reported.  **Where justification reported:** NA. | **Analysis type reported in protocol:** Not reported.  **Analysis type reported in review:** Subgroup analysis.  **Reason for changing mind:** Not reported.    **Analysis type in protocol according to glossary:** Not reported.  **How determined the analysis type:** NA.  **Analysis type in review according to glossary:** Subgroup analysis.  **How determined the analysis type:** Categories given.  **Analysed:** Yes.  **Reasons given (in the review) for not doing interaction analysis if planned:** NA.  **Reasons given for carrying out interaction analysis if they weren’t planned:** ‘At the suggestion of a peer reviewer we did two additional subgroup  analyses.’  **Scenario in protocol:** Not reported  **Rationale for planned scenario:** NA.  **Scenario in review (if reported specifically):** Not reported  **Rationale for done scenario:** NA.  **Reason for changing mind:** NA. | **Method to detect interactions reported in protocol:** Not reported.  **Method to detect interactions reported in review:** Not reported.  **Reason for changing mind:** NA. |
| Stead 2012 | **Protocol:** Not reported.  **Review methods:** Not reported.  **Review results:** ‘Modality of contact’. ‘All contact by telephone’; ‘No contact for control group’, ‘face-to-face intervention’; ‘Face-to-face contact for both intervention & control conditions’.  **Type:** Intervention.  **Covariate summary:** Type of intervention or control. | **Method to choose**  **covariate:** Not reported.  **Where method reported:** NA.  **Rationale for choosing the covariate:** Not reported.  **Where rationale reported:** NA. | **Where reported:** Review only.  **Reason not reported in review:** NA.  **Reason not reported in protocol:** ‘At the suggestion of a peer reviewer we did two additional subgroup  analyses.’  **Labelled as ‘posthoc’ if not in protocol:** Yes (‘not prespecified’). | **Data type:** Categorical.  **Continuous covariate categorised:** NA.  **How determine it was categorised:** NA.  **Justification for categorising:** NA.  **Where justification reported:** NA.  **Categories reported:** Yes.  **Where categories reported:** Review only.  **Justification for categories:** Not reported.  **Where justification reported:** NA. | **Analysis type reported in protocol:** Not reported.  **Analysis type reported in review:** Subgroup analysis.  **Reason for changing mind:** Not reported.    **Analysis type in protocol according to glossary:** Not reported.  **How determined the analysis type:** NA.  **Analysis type in review according to glossary:** Subgroup analysis.  **How determined the analysis type:** Categories given.  **Analysed:** Yes.  **Reasons given (in the review) for not doing interaction analysis if planned:** NA.  **Reasons given for carrying out interaction analysis if they weren’t planned:** ‘At the suggestion of a peer reviewer we did two additional subgroup  analyses.’  **Scenario in protocol:** Not reported  **Rationale for planned scenario:** NA.  **Scenario in review (if reported specifically):** Not reported  **Rationale for done scenario:** NA.  **Reason for changing mind:** NA. | **Method to detect interactions reported in protocol:** Not reported.  **Method to detect interactions reported in review:** Not reported.  **Reason for changing mind:** NA. |
| Stead 2012 | **Protocol:** Not reported.  **Review methods:** Not reported.  **Review results:** Sensitivity analysis including intermediate intensity conditions. Adjunct behavioural support versus pharmacotherapy alone.  **Type:** Intervention  **Covariate summary:** Type of intervention or control. | **Method to choose**  **covariate:** Not reported.  **Where method reported:** NA.  **Rationale for choosing the covariate:** Not reported.  **Where rationale reported:** NA. | **Where reported:** Review only.  **Reason not reported in review:** NA.  **Reason not reported in protocol:** Not reported.  **Labelled as ‘posthoc’ if not in protocol:** No. | **Data type:** Categorical.  **Continuous covariate categorised:** NA.  **How determine it was categorised:** NA.  **Justification for categorising:** NA.  **Where justification reported:** NA.  **Categories reported:** Yes.  **Where categories reported:** Review only.  **Justification for categories:** Not reported.  **Where justification reported:** NA. | **Analysis type reported in protocol:** Not reported.  **Analysis type reported in review:** Sensitivity analysis.  **Reason for changing mind:** Not reported.    **Analysis type in protocol according to glossary:** Not reported.  **How determined the analysis type:** NA.  **Analysis type in review according to glossary:** Subgroup analysis.  **How determined the analysis type:** Categories given.  **Analysed:** Yes.  **Reasons given (in the review) for not doing interaction analysis if planned:** NA.  **Reasons given for carrying out interaction analysis if they weren’t planned:** Not reported  **Scenario in protocol:** Not reported  **Rationale for planned scenario:** NA.  **Scenario in review (if reported specifically):** Not reported  **Rationale for done scenario:** NA.  **Reason for changing mind:** NA. | **Method to detect interactions reported in protocol:** Not reported.  **Method to detect interactions reported in review:** Not reported.  **Reason for changing mind:** NA. |
| Stead 2012 | **Protocol:** Not reported.  **Review methods:** Not reported.  **Review results:** Setting. ‘Recruited and treatment initiated in health care setting’; ‘Members of health care organisation’; ‘Community volunteers’).  **Type:** Patient.  **Covariate summary:** Setting. | **Method to choose**  **covariate:** Not reported.  **Where method reported:** NA.  **Rationale for choosing the covariate:** Not reported.  **Where rationale reported:** NA. | **Where reported:** Review only.  **Reason not reported in review:** NA.  **Reason not reported in protocol:** Not reported.  **Labelled as ‘posthoc’ if not in protocol:** No. | **Data type:** Categorical.  **Continuous covariate categorised:** NA.  **How determine it was categorised:** NA.  **Justification for categorising:** NA.  **Where justification reported:** NA.  **Categories reported:** Yes.  **Where categories reported:** Review only.  **Justification for categories:** Not reported.  **Where justification reported:** NA. | **Analysis type reported in protocol:** Not reported.  **Analysis type reported in review:** Subgroup analysis.  **Reason for changing mind:** Not reported.    **Analysis type in protocol according to glossary:** Not reported.  **How determined the analysis type:** NA.  **Analysis type in review according to glossary:** Subgroup analysis.  **How determined the analysis type:** Categories given.  **Analysed:** Yes.  **Reasons given (in the review) for not doing interaction analysis if planned:** NA.  **Reasons given for carrying out interaction analysis if they weren’t planned:** Not reported  **Scenario in protocol:** Not reported  **Rationale for planned scenario:** NA.  **Scenario in review (if reported specifically):** Not reported  **Rationale for done scenario:** NA.  **Reason for changing mind:** NA. | **Method to detect interactions reported in protocol:** Not reported.  **Method to detect interactions reported in review:** Not reported.  **Reason for changing mind:** NA. |
| Stead 2012 | **Protocol:** Not reported.  **Review methods:** Not reported.  **Review results:** Type of pharmacotherapy. ‘NRT’; ‘Bupropion’; ‘Nortriptyline’; ‘Varenicline’; ‘NRT & bupropion’; ‘Choice of pharmacotherapy’).  **Type:** Intervention  **Covariate summary:** Type of intervention or control. | **Method to choose**  **covariate:** Not reported.  **Where method reported:** NA.  **Rationale for choosing the covariate:** Not reported.  **Where rationale reported:** NA. | **Where reported:** Review only.  **Reason not reported in review:** NA.  **Reason not reported in protocol:** Not reported.  **Labelled as ‘posthoc’ if not in protocol:** No. | **Data type:** Categorical.  **Continuous covariate categorised:** NA.  **How determine it was categorised:** NA.  **Justification for categorising:** NA.  **Where justification reported:** NA.  **Categories reported:** Yes.  **Where categories reported:** Review only.  **Justification for categories:** Not reported.  **Where justification reported:** NA. | **Analysis type reported in protocol:** Not reported.  **Analysis type reported in review:** Subgroup analysis.  **Reason for changing mind:** Not reported.    **Analysis type in protocol according to glossary:** Not reported.  **How determined the analysis type:** NA.  **Analysis type in review according to glossary:** Subgroup analysis.  **How determined the analysis type:** Categories given.  **Analysed:** Yes.  **Reasons given (in the review) for not doing interaction analysis if planned:** NA.  **Reasons given for carrying out interaction analysis if they weren’t planned:** Not reported  **Scenario in protocol:** Not reported  **Rationale for planned scenario:** NA.  **Scenario in review (if reported specifically):** Not reported  **Rationale for done scenario:** NA.  **Reason for changing mind:** NA. | **Method to detect interactions reported in protocol:** Not reported.  **Method to detect interactions reported in review:** Not reported.  **Reason for changing mind:** NA. |
| Stead 2012 | **Protocol:** The use of biochemical validation of cessation. We judged the following to be three covariates: ‘We will also consider whether the definition and duration of follow up, or the use of biochemical validation of cessation, has any impact on treatment effect.’  **Review methods:** The use of biochemical validation of cessation. We judged the following to be three covariates: ‘We also considered whether the definition and duration of follow-up, or the use of biochemical validation of cessation, had any impact on treatment effect.’  **Review results:** By outcome definition. ‘12m validation PP outcomes only’; ‘12m validated sustained outcomes’; ‘Not 12m’; ‘No validation at all’).  **Type:** Outcome.  **Covariate summary:** Use of biochemical validation. | **Method to choose**  **covariate:** Not reported.  **Where method reported:** NA.  **Rationale for choosing the covariate:** Not reported.  **Where rationale reported:** NA. | **Where reported:** Protocol and review.  **Reason not reported in review:** NA.  **Reason not reported in protocol:** NA.  **Labelled as ‘posthoc’ if not in protocol:** NA. | **Data type:** Categorical.  **Continuous covariate categorised:** NA.  **How determine it was categorised:** NA.  **Justification for categorising:** NA.  **Where justification reported:** NA.  **Categories reported:** Not reported.  **Where categories reported:** NA.  **Justification for categories:** Not reported.  **Where justification reported:** NA. | **Analysis type reported in protocol:** Subgroup analysis.  **Analysis type reported in review:** Subgroup analysis.  **Reason for changing mind:** NA.    **Analysis type in protocol according to glossary:** Subgroup analysis.  **How determined the analysis type:** Says ‘subgroup analysis.’ No reason to dispute.  **Analysis type in review according to glossary:** Subgroup analysis.  **How determined the analysis type:** Says ‘subgroup analysis.’ No reason to dispute.  **Analysed:** Yes.  **Reasons given (in the review) for not doing interaction analysis if planned:** NA.  **Reasons given for carrying out interaction analysis if they weren’t planned:** NA  **Scenario in protocol:** Not reported.  **Rationale for planned scenario:** NA.  **Scenario in review (if reported specifically):** Not reported.  **Rationale for done scenario:** NA.  **Reason for changing mind:** NA. | **Method to detect interactions reported in protocol:** Not reported.  **Method to detect interactions reported in review:** Not reported.  **Reason for changing mind:** NA. |
| Trivedi 2013 | **Protocol:** ‘Birth weight: High (≥2 g/kg/day at commencement) or low (< 2 g/kg/day at commencement) intake.’  **Review methods:** ‘Birth weight: High (≥2 g/kg/day at commencement) or low (< 2 g/kg/day at commencement) intake.’  **Review results:** Not analysed.  **Type:** Patient.  **Covariate summary:** Demographics. | **Method to choose**  **covariate:** Not reported.  **Where method reported:** NA.  **Rationale for choosing the covariate:** Not reported.  **Where rationale reported:** NA. | **Where reported:** Protocol and review.  **Reason not reported in review:** NA.  **Reason not reported in protocol:** NA.  **Labelled as ‘posthoc’ if not in protocol:** NA. | **Data type:** Continuous.  **Continuous covariate categorised:** Yes.  **How determine it was categorised:** Categories given.  **Justification for categorising:** No reported.  **Where justification reported:** NA.  **Categories reported:** Yes.  **Where categories reported:** Protocol and review.  **Justification for categories:** Not reported.  **Where justification reported:** NA. | **Analysis type reported in protocol:** Subgroup analysis.  **Analysis type reported in review:** Subgroup analysis.  **Reason for changing mind:** NA.    **Analysis type in protocol according to glossary:** Subgroup analysis.  **How determined the analysis type:** Categories given.  **Analysis type in review according to glossary:** Subgroup analysis.  **How determined the analysis type:** Categories given.  **Analysed:** No.  **Reasons given (in the review) for not doing interaction analysis if planned:** Not reported (presumably because no trials).  **Reasons given for carrying out interaction analysis if they weren’t planned:** NA.  **Scenario in protocol:** ‘restricted to the primary outcome’. ‘If noted, we will explore the possible causes of statistical  heterogeneity (see subgroup analyses below).’  **Rationale for planned scenario:** Not reported.  **Scenario in review (if reported specifically):** Not reported.  **Rationale for done scenario:** NA.  **Reason for changing mind:** Not reported. | **Method to detect interactions reported in protocol:** Not reported.  **Method to detect interactions reported in review:** Not reported.  **Reason for changing mind:** NA. |
| Trivedi 2013 | **Protocol:** ‘Birth weight: studies enrolling low birth weight infants (less than 1500 grams) versus studies enrolling premature infants with birth weight of 1500 grams or more.’  **Review methods: ‘**Birth weight: premature low birth weight infants (less than 1500 grams) versus studies enrolling premature infants with birth weight of 1500 grams or more.’  **Review results:** Not analysed.  **Type:** Patient.  **Covariate summary:** Demographics. | **Method to choose**  **covariate:** Not reported.  **Where method reported:** NA.  **Rationale for choosing the covariate:** Not reported.  **Where rationale reported:** NA. | **Where reported:** Protocol and review.  **Reason not reported in review:** NA.  **Reason not reported in protocol:** NA.  **Labelled as ‘posthoc’ if not in protocol:** NA. | **Data type:** Continuous.  **Continuous covariate categorised:** Yes.  **How determine it was categorised:** Categories given.  **Justification for categorising:** No reported.  **Where justification reported:** NA.  **Categories reported:** Yes.  **Where categories reported:** Protocol and review.  **Justification for categories:** Not reported.  **Where justification reported:** NA. | **Analysis type reported in protocol:** Subgroup analysis.  **Analysis type reported in review:** Subgroup analysis.  **Reason for changing mind:** NA.    **Analysis type in protocol according to glossary:** Subgroup analysis.  **How determined the analysis type:** Categories given.  **Analysis type in review according to glossary:** Subgroup analysis.  **How determined the analysis type:** Categories given.  **Analysed:** No.  **Reasons given (in the review) for not doing interaction analysis if planned:** Not reported (presumably because no trials).  **Reasons given for carrying out interaction analysis if they weren’t planned:** NA.  **Scenario in protocol:** ‘restricted to the primary outcome’. ‘If noted, we will explore the possible causes of statistical  heterogeneity (see subgroup analyses below).’  **Rationale for planned scenario:** Not reported.  **Scenario in review (if reported specifically):** Not reported.  **Rationale for done scenario:** NA.  **Reason for changing mind:** Not reported. | **Method to detect interactions reported in protocol:** Not reported.  **Method to detect interactions reported in review:** Not reported.  **Reason for changing mind:** NA. |
| Trivedi 2013 | **Protocol:** ‘Birth weight: studies enrolling very low birth weight infants (less than 1000 grams) versus studies enrolling premature infants with birth weight of 1000 grams or more.’  **Review methods: ‘**Birth weight: premature very low birth weight infants (less than 1000 grams) versus studies enrolling premature infants with birth weight of 1000 grams or more.’  **Review results:** Not analysed.  **Type:** Patient.  **Covariate summary:** Demographics. | **Method to choose**  **covariate:** Not reported.  **Where method reported:** NA.  **Rationale for choosing the covariate:** Not reported.  **Where rationale reported:** NA. | **Where reported:** Protocol and review.  **Reason not reported in review:** NA.  **Reason not reported in protocol:** NA.  **Labelled as ‘posthoc’ if not in protocol:** NA. | **Data type:** Continuous.  **Continuous covariate categorised:** Yes.  **How determine it was categorised:** Categories given.  **Justification for categorising:** No reported.  **Where justification reported:** NA.  **Categories reported:** Yes.  **Where categories reported:** Protocol and review.  **Justification for categories:** Not reported.  **Where justification reported:** NA. | **Analysis type reported in protocol:** Subgroup analysis.  **Analysis type reported in review:** Subgroup analysis.  **Reason for changing mind:** NA.    **Analysis type in protocol according to glossary:** Subgroup analysis.  **How determined the analysis type:** Categories given.  **Analysis type in review according to glossary:** Subgroup analysis.  **How determined the analysis type:** Categories given.  **Analysed:** No.  **Reasons given (in the review) for not doing interaction analysis if planned:** Not reported (presumably because no trials).  **Reasons given for carrying out interaction analysis if they weren’t planned:** NA.  **Scenario in protocol:** ‘restricted to the primary outcome’. ‘If noted, we will explore the possible causes of statistical  heterogeneity (see subgroup analyses below).’  **Rationale for planned scenario:** Not reported.  **Scenario in review (if reported specifically):** Not reported.  **Rationale for done scenario:** NA.  **Reason for changing mind:** Not reported. | **Method to detect interactions reported in protocol:** Not reported.  **Method to detect interactions reported in review:** Not reported.  **Reason for changing mind:** NA. |
| Trivedi 2013 | **Protocol:** ‘Studies enrolling premature infants with a gestational age less than 30 weeks versus studies enrolling premature infants with a gestational age 30 or more weeks.’  **Review methods: ‘**Premature infants with a gestational age less than 30 weeks versus studies enrolling premature infants with a gestational age 30 or more weeks.’  **Review results:** Not analysed.  **Type:** Patient.  **Covariate summary:** Demographics. | **Method to choose**  **covariate:** Not reported.  **Where method reported:** NA.  **Rationale for choosing the covariate:** Not reported.  **Where rationale reported:** NA. | **Where reported:** Protocol and review.  **Reason not reported in review:** NA.  **Reason not reported in protocol:** NA.  **Labelled as ‘posthoc’ if not in protocol:** NA. | **Data type:** Continuous.  **Continuous covariate categorised:** Yes.  **How determine it was categorised:** Categories given.  **Justification for categorising:** No reported.  **Where justification reported:** NA.  **Categories reported:** Yes.  **Where categories reported:** Protocol and review.  **Justification for categories:** Not reported.  **Where justification reported:** NA. | **Analysis type reported in protocol:** Subgroup analysis.  **Analysis type reported in review:** Subgroup analysis.  **Reason for changing mind:** NA.    **Analysis type in protocol according to glossary:** Subgroup analysis.  **How determined the analysis type:** Categories given.  **Analysis type in review according to glossary:** Subgroup analysis.  **How determined the analysis type:** Categories given.  **Analysed:** No.  **Reasons given (in the review) for not doing interaction analysis if planned:** Not reported (presumably because no trials).  **Reasons given for carrying out interaction analysis if they weren’t planned:** NA.  **Scenario in protocol:** ‘restricted to the primary outcome’. ‘If noted, we will explore the possible causes of statistical  heterogeneity (see subgroup analyses below).’  **Rationale for planned scenario:** Not reported.  **Scenario in review (if reported specifically):** Not reported.  **Rationale for done scenario:** NA.  **Reason for changing mind:** Not reported. | **Method to detect interactions reported in protocol:** Not reported.  **Method to detect interactions reported in review:** Not reported.  **Reason for changing mind:** NA. |
| Trivedi 2013 | **Protocol:** ‘Studies initiating AA solutions in isolation versus studies starting AA solutions with other components of total parenteral nutrition’.  **Review methods:** ‘AA solution in isolation versus AA solution with glucose and/or lipids’.  **Review results:** Not analysed.  **Type:** Intervention.  **Covariate summary:** Additional interventions. | **Method to choose**  **covariate:** Not reported.  **Where method reported:** NA.  **Rationale for choosing the covariate:** Not reported.  **Where rationale reported:** NA. | **Where reported:** Protocol and review.  **Reason not reported in review:** NA.  **Reason not reported in protocol:** NA.  **Labelled as ‘posthoc’ if not in protocol:** NA. | **Data type:** Categorical.  **Continuous covariate categorised:** NA.  **How determine it was categorised:** NA.  **Justification for categorising:** NA.  **Where justification reported:** NA.  **Categories reported:** Yes.  **Where categories reported:** Protocol and review.  **Justification for categories:** Not reported.  **Where justification reported:** NA. | **Analysis type reported in protocol:** Subgroup analysis.  **Analysis type reported in review:** Subgroup analysis.  **Reason for changing mind:** NA.    **Analysis type in protocol according to glossary:** Subgroup analysis.  **How determined the analysis type:** Categories given.  **Analysis type in review according to glossary:** Subgroup analysis.  **How determined the analysis type:** Categories given.  **Analysed:** No.  **Reasons given (in the review) for not doing interaction analysis if planned:** Not reported (presumably because no trials).  **Reasons given for carrying out interaction analysis if they weren’t planned:** NA.  **Scenario in protocol:** ‘restricted to the primary outcome’. ‘If noted, we will explore the possible causes of statistical  heterogeneity (see subgroup analyses below).’  **Rationale for planned scenario:** Not reported.  **Scenario in review (if reported specifically):** Not reported.  **Rationale for done scenario:** NA.  **Reason for changing mind:** Not reported. | **Method to detect interactions reported in protocol:** Not reported.  **Method to detect interactions reported in review:** Not reported.  **Reason for changing mind:** NA. |
| Trivedi 2013 | **Protocol:** Not reported.  **Review methods:** ‘Premature infants born with intrauterine growth restriction versus premature infants born with gestational age-appropriate birth weight.’  **Review results:** Not analysed.  **Type:** Patient.  **Covariate summary:** Disease characteristics. | **Method to choose**  **covariate:** Not reported.  **Where method reported:** NA.  **Rationale for choosing the covariate:** Not reported.  **Where rationale reported:** NA. | **Where reported:** Review only.  **Reason not reported in review:** NA.  **Reason not reported in protocol:** ‘Additional subgroup analyses based on the presence or absence of of intra-uterine growth restriction was added as per suggestion of the peer reviewer.’  **Labelled as ‘posthoc’ if not in protocol:** No. | **Data type:** Categorical.  **Continuous covariate categorised:** NA.  **How determine it was categorised:** NA.  **Justification for categorising:** NA.  **Where justification reported:** NA.  **Categories reported:** Yes.  **Where categories reported:** Review only.  **Justification for categories:** Not reported.  **Where justification reported:** NA. | **Analysis type reported in protocol:** Not reported.  **Analysis type reported in review:** Subgroup analysis.  **Reason for changing mind:** Not reported.    **Analysis type in protocol according to glossary:** Not reported.  **How determined the analysis type:** NA.  **Analysis type in review according to glossary:** Subgroup analysis.  **How determined the analysis type:** Categories given.  **Analysed:** No.  **Reasons given (in the review) for not doing interaction analysis if planned:** Not reported (presumably because no trials).  **Reasons given for carrying out interaction analysis if they weren’t planned:** NA.  **Scenario in protocol:** Not reported.  **Rationale for planned scenario:** NA.  **Scenario in review (if reported specifically):** Not reported.  **Rationale for done scenario:** NA.  **Reason for changing mind:** NA. | **Method to detect interactions reported in protocol:** Not reported.  **Method to detect interactions reported in review:** Not reported.  **Reason for changing mind:** NA. |
| Trotti 2012 | **Protocol:** ‘Different types of patients (e. g. , pregnant women or patients with ESRD). We judged the following to be two covariates: ‘We will perform subgroup analyses of different types of patients (e. g. , pregnant women or patients with ESRD) and different formulations of iron (oral versus parenteral).’  **Review methods: ‘**Different types of patients (e. g. , pregnant women or patients with ESRD). We judged the following to be two covariates: ‘We prespecified subgroup analyses to examine the effects of different types of patients (e. g. , pregnant women or patients with ESRD) and different formulations of iron (oral versus parenteral).’  **Review results:** Patients with ESRD.  **Type:** Patient.  **Covariate summary:** Disease characteristics. | **Method to choose**  **covariate:** Not reported.  **Where method reported:** NA.  **Rationale for choosing the covariate:** Not reported.  **Where rationale reported:** NA. | **Where reported:** Protocol and review.  **Reason not reported in review:** NA.  **Reason not reported in protocol:** NA.  **Labelled as ‘posthoc’ if not in protocol:** NA**.** | **Data type:** Categorical.  **Continuous covariate categorised:** NA.  **How determine it was categorised:** NA.  **Justification for categorising:** NA.  **Where justification reported:** NA.  **Categories reported:** Yes.  **Where categories reported:** Protocol and review.  **Justification for categories:** Not reported.  **Where justification reported:** NA. | **Analysis type reported in protocol:** Subgroup analysis.  **Analysis type reported in review:** Subgroup analysis.  **Reason for changing mind:** NA.    **Analysis type in protocol according to glossary:** Subgroup analysis.  **How determined the analysis type:** Categories given.  **Analysis type in review according to glossary:** Sensitivity analysis.  **How determined the analysis type:** Categories given.  **Analysed:** Yes.  **Reasons given (in the review) for not doing interaction analysis if planned:** NA.  **Reasons given for carrying out interaction analysis if they weren’t planned:** NA.  **Scenario in protocol:** Not reported.  **Rationale for planned scenario:** NA.  **Scenario in review (if reported specifically):** Not reported.  **Rationale for done scenario:** NA.  **Reason for changing mind:** NA. | **Method to detect interactions reported in protocol:** Not reported.  **Method to detect interactions reported in review:** Not reported.  **Reason for changing mind:** NA. |
| Trotti 2012 | **Protocol:** Different formulations of iron (oral versus parenteral). We judged the following to be two covariates: ‘We will perform subgroup analyses of different types of patients (e. g. , pregnant women or patients with ESRD) and different formulations of iron (oral versus parenteral).’  **Review methods:** Different formulations of iron (oral versus parenteral). We judged the following to be two covariates: ‘We prespecified subgroup analyses to examine the effects of different types of patients (e. g. , pregnant women or patients with ESRD) and different formulations of iron (oral versus parenteral).’  **Review results:** IV iron.  **Type:** Intervention.  **Covariate summary:** Route of administration. | **Method to choose**  **covariate:** Not reported.  **Where method reported:** NA.  **Rationale for choosing the covariate:** Not reported.  **Where rationale reported:** NA. | **Where reported:** Protocol and review.  **Reason not reported in review:** NA.  **Reason not reported in protocol:** NA.  **Labelled as ‘posthoc’ if not in protocol:** NA**.** | **Data type:** Categorical.  **Continuous covariate categorised:** NA.  **How determine it was categorised:** NA.  **Justification for categorising:** NA.  **Where justification reported:** NA.  **Categories reported:** Yes.  **Where categories reported:** Protocol and review.  **Justification for categories:** Not reported.  **Where justification reported:** NA. | **Analysis type reported in protocol:** Subgroup analysis.  **Analysis type reported in review:** Subgroup analysis.  **Reason for changing mind:** NA.    **Analysis type in protocol according to glossary:** Subgroup analysis.  **How determined the analysis type:** Categories given.  **Analysis type in review according to glossary:** Sensitivity analysis.  **How determined the analysis type:** Categories reported.  **Analysed:** Yes.  **Reasons given (in the review) for not doing interaction analysis if planned:** NA.  **Reasons given for carrying out interaction analysis if they weren’t planned:** NA.  **Scenario in protocol:** Not reported.  **Rationale for planned scenario:** NA.  **Scenario in review (if reported specifically):** Not reported.  **Rationale for done scenario:** NA.  **Reason for changing mind:** NA. | **Method to detect interactions reported in protocol:** Not reported.  **Method to detect interactions reported in review:** Not reported.  **Reason for changing mind:** NA. |
| Trotti 2012 | **Protocol:** High- versus low quality trials. We judged the following to be two covariates: ‘We planned to conduct sensitivity analyses for high- versus low quality trials and for parallel group versus cross-over trial designs.’  **Review methods:** High- versus low quality trials. We judged the following to be two covariates: ‘We planned to conduct sensitivity analyses for high- versus low quality trials and for parallel group versus cross-over trial designs.’  **Review results:** Not analysed.  **Type:** Methodological.  **Covariate summary:** Quality. | **Method to choose**  **covariate:** Not reported.  **Where method reported:** NA.  **Rationale for choosing the covariate:** Not reported.  **Where rationale reported:** NA. | **Where reported:** Protocol and review.  **Reason not reported in review:** NA.  **Reason not reported in protocol:** NA.  **Labelled as ‘posthoc’ if not in protocol:** NA**.** | **Data type:** Categorical.  **Continuous covariate categorised:** NA.  **How determine it was categorised:** NA.  **Justification for categorising:** NA.  **Where justification reported:** NA.  **Categories reported:** Yes.  **Where categories reported:** Protocol and review.  **Justification for categories:** Not reported.  **Where justification reported:** NA. | **Analysis type reported in protocol:** Sensitivity analysis.  **Analysis type reported in review:** Sensitivity analysis.  **Reason for changing mind:** NA.    **Analysis type in protocol according to glossary:** Subgroup analysis.  **How determined the analysis type:** Categories given.  **Analysis type in review according to glossary:** Subgroup analysis.  **How determined the analysis type:** Categories given.  **Analysed:** No.  **Reasons given (in the review) for not doing interaction analysis if planned:** ‘Sufficient data were not available to conduct  sensitivity analyses for low versus high quality trials nor parallel  **Reasons given for carrying out interaction analysis if they weren’t planned:** NA.  **Scenario in protocol:** Not reported.  **Rationale for planned scenario:** NA.  **Scenario in review (if reported specifically):** Not reported.  **Rationale for done scenario:** NA.  **Reason for changing mind:** NA. | **Method to detect interactions reported in protocol:** Not reported.  **Method to detect interactions reported in review:** Not reported.  **Reason for changing mind:** NA. |
| Trotti 2012 | **Protocol:** Not reported.  **Review methods:** Not reported.  **Review results: ‘**Iron deficient’.  **Type:** Patient.  **Covariate summary:** Disease characteristics. | **Method to choose**  **covariate:** Not reported.  **Where method reported:** NA.  **Rationale for choosing the covariate:** Not reported.  **Where rationale reported:** NA. | **Where reported:** Review only.  **Reason not reported in review:** NA.  **Reason not reported in protocol:** Not reported.  **Labelled as ‘posthoc’ if not in protocol:** No. | **Data type:** Categorical.  **Continuous covariate categorised:** NA.  **How determine it was categorised:** NA.  **Justification for categorising:** NA.  **Where justification reported:** NA.  **Categories reported:** Yes.  **Where categories reported:** Review only.  **Justification for categories:** Not reported.  **Where justification reported:** NA. | **Analysis type reported in protocol:** Not reported.  **Analysis type reported in review:** Subgroup analysis.  **Reason for changing mind:** Not reported.    **Analysis type in protocol according to glossary:** Not reported.  **How determined the analysis type:** NA.  **Analysis type in review according to glossary:** Sensitivity analysis.  **How determined the analysis type:** Categories reported.  **Analysed:** Yes.  **Reasons given (in the review) for not doing interaction analysis if planned:** NA.  **Reasons given for carrying out interaction analysis if they weren’t planned:** Not reported.  **Scenario in protocol:** Not reported.  **Rationale for planned scenario:** NA.  **Scenario in review (if reported specifically):** Not reported.  **Rationale for done scenario:** NA.  **Reason for changing mind:** NA. | **Method to detect interactions reported in protocol:** Not reported.  **Method to detect interactions reported in review:** Not reported.  **Reason for changing mind:** NA. |
| Trotti 2012 | **Protocol:** Parallel group versus cross-over trial designs. We judged the following to be two covariates: ‘We planned to conduct sensitivity analyses for high- versus low quality trials and for parallel group versus cross-over trial designs.’  **Review methods:** Parallel group versus cross-over trial designs. We judged the following to be two covariates: ‘We planned to conduct sensitivity analyses for high- versus low quality trials and for parallel group versus cross-over trial designs.’  **Review results:** Not analysed.  **Type:** Methodological.  **Covariate summary:** Trial design. | **Method to choose**  **covariate:** Not reported.  **Where method reported:** NA.  **Rationale for choosing the covariate:** Not reported.  **Where rationale reported:** NA. | **Where reported:** Protocol and review.  **Reason not reported in review:** NA.  **Reason not reported in protocol:** NA.  **Labelled as ‘posthoc’ if not in protocol:** NA**.** | **Data type:** Categorical.  **Continuous covariate categorised:** NA.  **How determine it was categorised:** NA.  **Justification for categorising:** NA.  **Where justification reported:** NA.  **Categories reported:** Yes.  **Where categories reported:** Protocol and review.  **Justification for categories:** Not reported.  **Where justification reported:** NA. | **Analysis type reported in protocol:** Sensitivity analysis.  **Analysis type reported in review:** Sensitivity analysis.  **Reason for changing mind:** NA.    **Analysis type in protocol according to glossary:** Subgroup analysis.  **How determined the analysis type:** Categories given.  **Analysis type in review according to glossary:** Subgroup analysis.  **How determined the analysis type:** Categories given.  **Analysed:** No.  **Reasons given (in the review) for not doing interaction analysis if planned:** ‘Sufficient data were not available to conduct  sensitivity analyses for low versus high quality trials nor parallel  group versus crossover trials.’  **Reasons given for carrying out interaction analysis if they weren’t planned:** NA.  **Scenario in protocol:** Not reported.  **Rationale for planned scenario:** NA.  **Scenario in review (if reported specifically):** Not reported.  **Rationale for done scenario:** NA.  **Reason for changing mind:** NA. | **Method to detect interactions reported in protocol:** Not reported.  **Method to detect interactions reported in review:** Not reported.  **Reason for changing mind:** NA. |
| Van Teeffelen, 2013 | **Protocol:** ‘Compare the groups with retainment of amniotic fluid in the treatment group, versus the group with spontaneous re-accumulation in the standard care group, and the group which does not retain amniotic fluid with the group in the standard care group that shows no signs of spontaneous re-accumulation. This is possible if frequent ultrasound monitoring of amniotic fluid volume is done in both groups.’  **Review methods:** ‘Compare the groups with retainment of amniotic fluid in the treatment group, versus the group with spontaneous re-accumulation in the standard care group, and the group which does not retain amniotic fluid with the group in the standard care group that shows no signs of spontaneous re-accumulation. This is possible if frequent ultrasound monitoring of amniotic fluid volume is done in both groups.’  **Review results:** Not analysed.  **Type:** Patient.  **Covariate summary:** Disease characteristics. | **Method to choose**  **covariate:** not reported.  **Where method reported:** NA.  **Rationale for choosing the covariate:’** In the literature, the incidence of spontaneous re-accumulation  of amniotic fluid after PPROM has been reported as 25% (Hadi  1994).The incidence of retainment of transabdominally amnioinfused  fluid after PPROMhas been reported by two authors. Tan et  al. found in 27 amnioinfused patients retainment of fluid after 48  hours in only 4 cases (24%), whereas this was 30% in 36 patients  in a study by Vergani et al. (Tan 2003, Vergani 2004). Hypothetically,  the retainment of amnioinfused fluid could be partly caused  by nothing more than spontaneous re-accumulation. This could  be due to spontaneous resealing of the membrane defect, which  occurs anyway in some patients, with or without amnioinfusion.  In that case benefit from amnioinfusion would be small or even  absent.’  **Where rationale reported:** Protocol and review. | **Where reported:** Protocol and review.  **Reason not reported in review:** NA.  **Reason not reported in protocol:** NA.  **Labelled as ‘posthoc’ if not in protocol:** NA. | **Data type:** Categorical.  **Continuous covariate categorised:** NA.  **How determine it was categorised:** NA.  **Justification for categorising:** NA.  **Where justification reported:** NA.  **Categories reported:** Yes.  **Where categories reported:** Protocol and review.  **Justification for categories:** ‘In the literature, the incidence of spontaneous re-accumulation of amniotic fluid after PPROM has been reported as 25% (Hadi  1994).The incidence of retainment of transabdominally amnioinfused fluid after PPROMhas been reported by two authors. Tan et al. found in 27 amnioinfused patients retainment of fluid after 48 hours in only 4 cases (24%), whereas this was 30% in 36 patients in a study by Vergani et al. (Tan 2003, Vergani 2004). Hypothetically, the retainment of amnioinfused fluid could be partly caused by nothing more than spontaneous re-accumulation. This could be due to spontaneous resealing of the membrane defect, which occurs anyway in some patients, with or without amnioinfusion. In that case benefit from amnioinfusion would be small or even absent.’  **Where justification reported:** Protocol and review. | **Analysis type reported in protocol:** Subgroup analysis.  **Analysis type reported in review:** Subgroup analysis.  **Reason for changing mind:** NA.    **Analysis type in protocol according to glossary:** Subgroup analysis.  **How determined the analysis type:** Categories given.  **Analysis type in review according to glossary:** Subgroup analysis.  **How determined the analysis type:** Categories given.  **Analysed:** No.  **Reasons given (in the review) for not doing interaction analysis if planned:** Not reported (presumably because no trials).  **Reasons given for carrying out interaction analysis if they weren’t planned:** NA.  **Scenario in protocol: ‘**If we identify substantial heterogeneity, we will investigate it using  subgroup analyses.’ ‘We plan to carry out two subgroup analysis for the primary outcome.’ **‘**This subgroup analysis will only be performed if enough  data are available from the randomised trials.’  **Rationale for planned scenario:** Not reported.  **Scenario in review (if reported specifically): ‘**If we identify substantial heterogeneity, we will investigate it using subgroup analyses.’ ‘We plan to carry out two subgroup analysis for the primary outcome.’ **‘**This subgroup analysis will only be performed if enough  data are available from the randomised trials.’  **Rationale for done scenario:** Not reported.  **Reason for changing mind:** NA. | **Method to detect interactions reported in protocol: ‘**For fixed-effect inverse variance meta-analyses, we will assess differences  between subgroups by interaction tests. For random-effects  and fixed-effect meta-analyses using methods other than inverse  variance, we will assess differences between subgroups by inspection  of the subgroups’ confidence intervals; non-overlapping  confidence intervals indicate a statistically significant difference in  treatment effect between the subgroups.’  **Method to detect interactions reported in review: ‘**We will assess subgroup differences by interaction tests available within RevMan (RevMan 2011).We will report the results of subgroup  analyses quoting the _  2 statistic and P value, and the interaction test I² value.’  **Reason for changing mind:** Not reported. |
| Van Teeffelen, 2013 | **Protocol:** ‘Excluding studies that describe a loss to follow-up of over 25%’.  **Review methods: ‘**Excluding studies that describe a loss to follow-up of over 25%’.  **Review results:** Not analysed.  **Type:** Methodological.  **Covariate summary:** Incomplete outcome data/follow up. | **Method to choose**  **covariate:** Not reported.  **Where method reported:** NA.  **Rationale for choosing the covariate:** Not reported.  **Where rationale reported:** NA. | **Where reported:** Protocol and review.  **Reason not reported in review:** NA.  **Reason not reported in protocol:** NA.  **Labelled as ‘posthoc’ if not in protocol:** NA. | **Data type:** Continuous.  **Continuous covariate categorised:** Yes.  **How determine it was categorised:** Categories given.  **Justification for categorising:** No reported.  **Where justification reported:** NA.  **Categories reported:** Yes.  **Where categories reported:** Protocol and review.  **Justification for categories:** Not reported.  **Where justification reported:** NA. | **Analysis type reported in protocol:** Sensitivity analysis.  **Analysis type reported in review:** Sensitivity analysis.  **Reason for changing mind:** NA.    **Analysis type in protocol according to glossary:** Sensitivity analysis.  **How determined the analysis type:** ‘excluding’.  **Analysis type in review according to glossary:** Sensitivity analysis.  **How determined the analysis type:** ‘excluding’.  **Analysed:** No.  **Reasons given (in the review) for not doing interaction analysis if planned:** Not reported (presumably because no trials).  **Reasons given for carrying out interaction analysis if they weren’t planned:** NA.  **Scenario in protocol:** ‘if applicable...’  **Rationale for planned scenario:** Not reported.  **Scenario in review (if reported specifically):** ‘if applicable...’  **Rationale for done scenario:** Not reported.  **Reason for changing mind:** NA. | **Method to detect interactions reported in protocol:** Not reported.  **Method to detect interactions reported in review:** Not reported.  **Reason for changing mind:** NA. |
| Van Teeffelen, 2013 | **Protocol:** ‘Patients from the intervention group who retain amniotic fluid (‘successful amnioinfusion’) versus the standard care group and patients from the intervention group who do not retain amniotic fluid (‘unsuccessful’ amnioinfusion) versus the standard care group.’ ‘Succesful amnioinfusion as defined by study specific criteria for diagnosing oligohydramnios (timing of measurement, cut-off value used for ultrasound assessment of amount of fluid).’  **Review methods:** ‘Patients from the intervention group who retain amniotic fluid (‘successful amnioinfusion’) versus the standard care group and patients from the intervention group who do not retain amniotic fluid (‘unsuccessful’ amnioinfusion) versus the standard care group.’ ‘Succesful amnioinfusion as defined by study specific criteria for diagnosing oligohydramnios (timing of measurement, cut-off value used for ultrasound assessment of amount of fluid).’  **Review results:** Not analysed.  **Type:** Patient.  **Covariate summary:** Disease characteristics. | **Method to choose**  **covariate:** not reported.  **Where method reported:** NA  **Rationale for choosing the covariate: ‘**The reason for this subgroup analysis is to test if even without retainment of amniotic  fluid, amnioinfusion is beneficial (by means of dilution and flushing of contaminated material in the womb) compared with standard care.’  **Where rationale reported:** Protocol and review. | **Where reported:** Protocol and review.  **Reason not reported in review:** NA.  **Reason not reported in protocol:** NA.  **Labelled as ‘posthoc’ if not in protocol:** NA. | **Data type:** Categorical.  **Continuous covariate categorised:** NA.  **How determine it was categorised:** NA.  **Justification for categorising:** NA.  **Where justification reported:** NA.  **Categories reported:** Yes.  **Where categories reported:** Protocol and review.  **Justification for categories:** ‘The reason for this  subgroup analysis is to test if even without retainment of amniotic fluid, amnioinfusion is beneficial (by means of dilution and flushing of contaminated material in the womb) compared with standard care.’  **Where justification reported:** Protocol and review. | **Analysis type reported in protocol:** Subgroup analysis.  **Analysis type reported in review:** Subgroup analysis.  **Reason for changing mind:** NA.    **Analysis type in protocol according to glossary:** Subgroup analysis.  **How determined the analysis type:** Categories given.  **Analysis type in review according to glossary:** Subgroup analysis.  **How determined the analysis type:** Categories given.  **Analysed:** No.  **Reasons given (in the review) for not doing interaction analysis if planned:** Not reported (presumably because no trials).  **Reasons given for carrying out interaction analysis if they weren’t planned:** NA.  **Scenario in protocol: ‘**If we identify substantial heterogeneity, we will investigate it using  subgroup analyses.’ ‘We plan to carry out two subgroup analysis for the primary outcome.’  **Rationale for planned scenario:** Not reported.  **Scenario in review (if reported specifically): ‘**If we identify substantial heterogeneity, we will investigate it using subgroup analyses.’ ‘We plan to carry out two subgroup analysis for the primary outcome.’  **Rationale for done scenario:** Not reported.  **Reason for changing mind:** NA. | **Method to detect interactions reported in protocol: ‘**For fixed-effect inverse variance meta-analyses, we will assess differences  between subgroups by interaction tests. For random-effects  and fixed-effect meta-analyses using methods other than inverse  variance, we will assess differences between subgroups by inspection  of the subgroups’ confidence intervals; non-overlapping  confidence intervals indicate a statistically significant difference in  treatment effect between the subgroups.’  **Method to detect interactions reported in review: ‘**We will assess subgroup differences by interaction tests available within RevMan (RevMan 2011).We will report the results of subgroup  analyses quoting the _  2 statistic and P value, and the interaction test I² value.’  **Reason for changing mind:** Not reported. |
| Van Teeffelen, 2013 | **Protocol:** Allocation. We judged the following to be two covariates: ‘excluding studies at high risk of bias, (allocation and sequence generation)’.  **Review methods:** Allocation. We judged the following to be two covariates: ‘excluding studies at high risk of bias, (allocation and sequence generation)’.  **Review results:** Not analysed.  **Type:** Methodological.  **Covariate summary:** Allocation concealment. | **Method to choose**  **covariate:** Not reported.  **Where method reported:** NA.  **Rationale for choosing the covariate:** Not reported.  **Where rationale reported:** NA. | **Where reported:** Protocol and review.  **Reason not reported in review:** NA.  **Reason not reported in protocol:** NA.  **Labelled as ‘posthoc’ if not in protocol:** NA. | **Data type:** Categorical.  **Continuous covariate categorised:** NA.  **How determine it was categorised:** NA.  **Justification for categorising:** NA.  **Where justification reported:** NA.  **Categories reported:** Yes.  **Where categories reported:** Protocol and review.  **Justification for categories:** Not reported.  **Where justification reported:** NA. | **Analysis type reported in protocol:** Sensitivity analysis.  **Analysis type reported in review:** Sensitivity analysis.  **Reason for changing mind:** NA.    **Analysis type in protocol according to glossary:** Sensitivity analysis.  **How determined the analysis type:** ‘excluding’.  **Analysis type in review according to glossary:** Sensitivity analysis.  **How determined the analysis type:** ‘excluding’.  **Analysed:** No.  **Reasons given (in the review) for not doing interaction analysis if planned:** Not reported (presumably because no trials).  **Reasons given for carrying out interaction analysis if they weren’t planned:** NA.  **Scenario in protocol:** ‘if applicable...’  **Rationale for planned scenario:** Not reported.  **Scenario in review (if reported specifically):** ‘if applicable...’  **Rationale for done scenario:** Not reported.  **Reason for changing mind:** NA. | **Method to detect interactions reported in protocol:** Not reported.  **Method to detect interactions reported in review:** Not reported.  **Reason for changing mind:** NA. |
| Van Teeffelen, 2013 | **Protocol:** Sequence generation. We judged the following to be two covariates: ‘excluding studies at high risk of bias, (allocation and sequence generation)’.  **Review methods:** Sequence generation. We judged the following to be two covariates: ‘excluding studies at high risk of bias, (allocation and sequence generation)’.  **Review results:** Not analysed.  **Type:** Methodological.  **Covariate summary:** Random generation. | **Method to choose**  **covariate:** Not reported.  **Where method reported:** NA.  **Rationale for choosing the covariate:** Not reported.  **Where rationale reported:** NA. | **Where reported:** Protocol and review.  **Reason not reported in review:** NA.  **Reason not reported in protocol:** NA.  **Labelled as ‘posthoc’ if not in protocol:** NA. | **Data type:** Categorical.  **Continuous covariate categorised:** NA.  **How determine it was categorised:** NA.  **Justification for categorising:** NA.  **Where justification reported:** NA.  **Categories reported:** Yes.  **Where categories reported:** Protocol and review.  **Justification for categories:** Not reported.  **Where justification reported:** NA. | **Analysis type reported in protocol:** Sensitivity analysis.  **Analysis type reported in review:** Sensitivity analysis.  **Reason for changing mind:** NA.    **Analysis type in protocol according to glossary:** Sensitivity analysis.  **How determined the analysis type:** ‘excluding’.  **Analysis type in review according to glossary:** Sensitivity analysis.  **How determined the analysis type:** ‘excluding’.  **Analysed:** No.  **Reasons given (in the review) for not doing interaction analysis if planned:** Not reported (presumably because no trials).  **Reasons given for carrying out interaction analysis if they weren’t planned:** NA.  **Scenario in protocol:** ‘if applicable...’  **Rationale for planned scenario:** Not reported.  **Scenario in review (if reported specifically):** ‘if applicable...’  **Rationale for done scenario:** Not reported.  **Reason for changing mind:** NA. | **Method to detect interactions reported in protocol:** Not reported.  **Method to detect interactions reported in review:** Not reported.  **Reason for changing mind:** NA. |
| van Zuuren 2013 | **Protocol:** ‘Different dosing schedules’.  **Review methods: ‘**Different dosing schedules’.  **Review results:** Not analysed.  **Type:** Intervention.  **Covariate summary:** Dose. | **Method to choose**  **covariate:** Not reported.  **Where method reported:** NA.  **Rationale for choosing the covariate:** Not reported.  **Where rationale reported:** NA. | **Where reported:** Protocol and review.  **Reason not reported in review:** NA.  **Reason not reported in protocol:** NA.  **Labelled as ‘posthoc’ if not in protocol:** NA. | **Data type:** Continuous.  **Continuous covariate categorised:** Yes.  **How determine it was categorised:** Subgroup analysis planned.  **Justification for categorising:** Not reported.  **Where justification reported:** NA.  **Categories reported:** Not reported.  **Where categories reported:** NA.  **Justification for categories:** Not reported.  **Where justification reported:** NA. | **Analysis type reported in protocol:** Subgroup analyses.  **Analysis type reported in review:** Subgroup analyses.  **Reason for changing mind:** NA.    **Analysis type in protocol according to glossary:** Subgroup analyses.  **How determined the analysis type:** Says **‘**Subgroup analyses.’. Not reason to dispute.  **Analysis type in review according to glossary:** Subgroup analyses.  **How determined the analysis type:** Says **‘**Subgroup analyses.’. Not reason to dispute.  **Analysed:** No.  **Reasons given (in the review) for not doing interaction analysis if planned:** Not reported (presumably because one trial).  **Reasons given for carrying out interaction analysis if they weren’t planned:** NA.  **Scenario in protocol: ‘**If the authors include a sufficient number of trials and identify  moderate, substantial or considerable heterogeneity (see ’Assessment of heterogeneity’) they plan to carry out the following subgroup analyses:’  **Rationale for planned scenario:** Not reported.  **Scenario in review (if reported specifically): ‘**In future updates, if a sufficient number of trials are included and if the authors identify moderate, substantial or considerable heterogeneity (see ’Assessment of heterogeneity’), they plan to carry  out the following subgroup analyses based on:’  **Rationale for done scenario:** Not reported.  **Reason for changing mind:** NA. | **Method to detect interactions reported in protocol:** Not reported.  **Method to detect interactions reported in review:** Not reported.  **Reason for changing mind:** NA. |
| van Zuuren 2013 | **Protocol:** ‘Exclusion of CCTs’.  **Review methods: ‘**Exclusion of CCTs’.  **Review results:** Not analysed.  **Type:** Methodological.  **Covariate summary:** Trial design. | **Method to choose**  **covariate:** Not reported.  **Where method reported:** NA.  **Rationale for choosing the covariate:** Not reported.  **Where rationale reported:** NA. | **Where reported:** Protocol and review.  **Reason not reported in review:** NA.  **Reason not reported in protocol:** NA.  **Labelled as ‘posthoc’ if not in protocol:** NA. | **Data type:** Categorical.  **Continuous covariate categorised:** NA.  **How determine it was categorised:** NA.  **Justification for categorising:** NA.  **Where justification reported:** NA.  **Categories reported:** Yes.  **Where categories reported:** Protocol and review.  **Justification for categories:** Not reported.  **Where justification reported:** NA. | **Analysis type reported in protocol:** Sensitivity analyses.  **Analysis type reported in review:** Sensitivity analyses.  **Reason for changing mind:** NA.    **Analysis type in protocol according to glossary:** Sensitivity analyses.  **How determined the analysis type:** Categories given.  **Analysis type in review according to glossary:** Sensitivity analyses.  **How determined the analysis type:** Categories given.  **Analysed:** No.  **Reasons given (in the review) for not doing interaction analysis if planned:** Not reported (presumably because one trial).  **Reasons given for carrying out interaction analysis if they weren’t planned:** NA.  **Scenario in protocol: ‘**The authors will undertake sensitivity analyses to assess the robustness of their review results by repeating the analysis with the following adjustment:’  **Rationale for planned scenario:** Not reported.  **Scenario in review (if reported specifically): ‘**The review authors will undertake sensitivity analyses in future updates if a larger number of trials are included, to assess the  robustness of their review results by repeating the analysis with the following adjustments:’  **Rationale for done scenario:** Not reported.  **Reason for changing mind:** NA. | **Method to detect interactions reported in protocol:** Not reported.  **Method to detect interactions reported in review:** Not reported.  **Reason for changing mind:** NA. |
| van Zuuren 2013 | **Protocol:** ‘Exclusion of trials with an unclear or high risk of bias for allocation concealment’.  **Review methods: ‘**Exclusion of trials with an unclear or high risk of bias for allocation concealment’.  **Review results:** Not analysed.  **Type:** Methodological.  **Covariate summary:** Allocation concealment. | **Method to choose**  **covariate:** Not reported.  **Where method reported:** NA.  **Rationale for choosing the covariate:** Not reported.  **Where rationale reported:** NA. | **Where reported:** Protocol and review.  **Reason not reported in review:** NA.  **Reason not reported in protocol:** NA.  **Labelled as ‘posthoc’ if not in protocol:** NA. | **Data type:** Categorical.  **Continuous covariate categorised:** NA.  **How determine it was categorised:** NA.  **Justification for categorising:** NA.  **Where justification reported:** NA.  **Categories reported:** Yes.  **Where categories reported:** Protocol and review.  **Justification for categories:** Not reported.  **Where justification reported:** NA. | **Analysis type reported in protocol:** Sensitivity analyses.  **Analysis type reported in review:** Sensitivity analyses.  **Reason for changing mind:** NA.    **Analysis type in protocol according to glossary:** Sensitivity analyses.  **How determined the analysis type:** Categories given.  **Analysis type in review according to glossary:** Sensitivity analyses.  **How determined the analysis type:** Categories given.  **Analysed:** No.  **Reasons given (in the review) for not doing interaction analysis if planned:** Not reported (presumably because one trial).  **Reasons given for carrying out interaction analysis if they weren’t planned:** NA.  **Scenario in protocol: ‘**The authors will undertake sensitivity analyses to assess the robustness of their review results by repeating the analysis with the following adjustment:’  **Rationale for planned scenario:** Not reported.  **Scenario in review (if reported specifically): ‘**The review authors will undertake sensitivity analyses in future updates if a larger number of trials are included, to assess the  robustness of their review results by repeating the analysis with the following adjustments:’  **Rationale for done scenario:** Not reported.  **Reason for changing mind:** NA. | **Method to detect interactions reported in protocol:** Not reported.  **Method to detect interactions reported in review:** Not reported.  **Reason for changing mind:** NA. |
| van Zuuren 2013 | **Protocol:** ‘Exclusion of trials with an unclear or high risk of bias for blinding of outcome assessment’.  **Review methods: ‘**Exclusion of trials with an unclear or high risk of bias for blinding of outcome assessment’.  **Review results:** Not analysed.  **Type:** Methodological.  **Covariate summary:** Blinding. | **Method to choose**  **covariate:** Not reported.  **Where method reported:** NA.  **Rationale for choosing the covariate:** Not reported.  **Where rationale reported:** NA. | **Where reported:** Protocol and review.  **Reason not reported in review:** NA.  **Reason not reported in protocol:** NA.  **Labelled as ‘posthoc’ if not in protocol:** NA. | **Data type:** Categorical.  **Continuous covariate categorised:** NA.  **How determine it was categorised:** NA.  **Justification for categorising:** NA.  **Where justification reported:** NA.  **Categories reported:** Yes.  **Where categories reported:** Protocol and review.  **Justification for categories:** Not reported.  **Where justification reported:** NA. | **Analysis type reported in protocol:** Sensitivity analyses.  **Analysis type reported in review:** Sensitivity analyses.  **Reason for changing mind:** NA.    **Analysis type in protocol according to glossary:** Sensitivity analyses.  **How determined the analysis type:** Categories given.  **Analysis type in review according to glossary:** Sensitivity analyses.  **How determined the analysis type:** Categories given.  **Analysed:** No.  **Reasons given (in the review) for not doing interaction analysis if planned:** Not reported (presumably because one trial).  **Reasons given for carrying out interaction analysis if they weren’t planned:** NA.  **Scenario in protocol: ‘**The authors will undertake sensitivity analyses to assess the robustness of their review results by repeating the analysis with the following adjustment:’  **Rationale for planned scenario:** Not reported.  **Scenario in review (if reported specifically): ‘**The review authors will undertake sensitivity analyses in future updates if a larger number of trials are included, to assess the  robustness of their review results by repeating the analysis with the following adjustments:’  **Rationale for done scenario:** Not reported.  **Reason for changing mind:** NA. | **Method to detect interactions reported in protocol:** Not reported.  **Method to detect interactions reported in review:** Not reported.  **Reason for changing mind:** NA. |
| van Zuuren 2013 | **Protocol:** ‘Exclusion of trials with an unclear or high risk of bias for completeness of follow-up’.  **Review methods: ‘**Exclusion of trials with an unclear or high risk of bias for completeness of follow-up’.  **Review results:** Not analysed.  **Type:** Methodological.  **Covariate summary:** Incomplete outcome data/follow up. | **Method to choose**  **covariate:** Not reported.  **Where method reported:** NA.  **Rationale for choosing the covariate:** Not reported.  **Where rationale reported:** NA. | **Where reported:** Protocol and review.  **Reason not reported in review:** NA.  **Reason not reported in protocol:** NA.  **Labelled as ‘posthoc’ if not in protocol:** NA. | **Data type:** Categorical.  **Continuous covariate categorised:** NA.  **How determine it was categorised:** NA.  **Justification for categorising:** NA.  **Where justification reported:** NA.  **Categories reported:** Yes.  **Where categories reported:** Protocol and review.  **Justification for categories:** Not reported.  **Where justification reported:** NA. | **Analysis type reported in protocol:** Sensitivity analyses.  **Analysis type reported in review:** Sensitivity analyses.  **Reason for changing mind:** NA.    **Analysis type in protocol according to glossary:** Sensitivity analyses.  **How determined the analysis type:** Categories given.  **Analysis type in review according to glossary:** Sensitivity analyses.  **How determined the analysis type:** Categories given.  **Analysed:** No.  **Reasons given (in the review) for not doing interaction analysis if planned:** Not reported (presumably because one trial).  **Reasons given for carrying out interaction analysis if they weren’t planned:** NA.  **Scenario in protocol: ‘**The authors will undertake sensitivity analyses to assess the robustness of their review results by repeating the analysis with the following adjustment:’  **Rationale for planned scenario:** Not reported.  **Scenario in review (if reported specifically): ‘**The review authors will undertake sensitivity analyses in future updates if a larger number of trials are included, to assess the  robustness of their review results by repeating the analysis with the following adjustments:’  **Rationale for done scenario:** Not reported.  **Reason for changing mind:** NA. | **Method to detect interactions reported in protocol:** Not reported.  **Method to detect interactions reported in review:** Not reported.  **Reason for changing mind:** NA. |
| van Zuuren 2013 | **Protocol:** ‘Type of low-molecular-weight heparins’.  **Review methods: ‘**Type of low-molecular-weight heparins’.  **Review results:** Not analysed.  **Type:** Intervention.  **Covariate summary:** Type of intervention or control. | **Method to choose**  **covariate:** Not reported.  **Where method reported:** NA.  **Rationale for choosing the covariate:** Not reported.  **Where rationale reported:** NA. | **Where reported:** Protocol and review.  **Reason not reported in review:** NA.  **Reason not reported in protocol:** NA.  **Labelled as ‘posthoc’ if not in protocol:** NA. | **Data type:** Categorical.  **Continuous covariate categorised:** NA.  **How determine it was categorised:** NA.  **Justification for categorising:** NA.  **Where justification reported:** NA.  **Categories reported:** Not reported.  **Where categories reported:** NA.  **Justification for categories:** Not reported.  **Where justification reported:** NA. | **Analysis type reported in protocol:** Subgroup analyses.  **Analysis type reported in review:** Subgroup analyses.  **Reason for changing mind:** NA.    **Analysis type in protocol according to glossary:** Subgroup analyses.  **How determined the analysis type:** Says **‘**Subgroup analyses.’. Not reason to dispute.  **Analysis type in review according to glossary:** Subgroup analyses.  **How determined the analysis type:** Says **‘**Subgroup analyses.’. Not reason to dispute.  **Analysed:** No.  **Reasons given (in the review) for not doing interaction analysis if planned:** Not reported (presumably because one trial).  **Reasons given for carrying out interaction analysis if they weren’t planned:** NA.  **Scenario in protocol: ‘**If the authors include a sufficient number of trials and identify  moderate, substantial or considerable heterogeneity (see ’Assessment of heterogeneity’) they plan to carry out the following subgroup analyses:’  **Rationale for planned scenario:** Not reported.  **Scenario in review (if reported specifically): ‘**In future updates, if a sufficient number of trials are included and if the authors identify moderate, substantial or considerable heterogeneity (see ’Assessment of heterogeneity’), they plan to carry  out the following subgroup analyses based on:’  **Rationale for done scenario:** Not reported.  **Reason for changing mind:** NA. | **Method to detect interactions reported in protocol:** Not reported.  **Method to detect interactions reported in review:** Not reported.  **Reason for changing mind:** NA. |
| van Zuuren 2013 | **Protocol:** ‘Type of SCD: sickle cell anaemia; haemoglobin SC disease; sickle cell ß0 thalassaemia; sickle cell ß+ thalassaemia.’  **Review methods: ‘**Type of SCD: sickle cell anaemia; haemoglobin SC disease; sickle cell ß0 thalassaemia; sickle cell ß+ thalassaemia.’  **Review results:** Not analysed.  **Type:** Patient.  **Covariate summary:** Disease characteristics. | **Method to choose**  **covariate:** Not reported.  **Where method reported:** NA.  **Rationale for choosing the covariate:** Not reported.  **Where rationale reported:** NA. | **Where reported:** Protocol and review.  **Reason not reported in review:** NA.  **Reason not reported in protocol:** NA.  **Labelled as ‘posthoc’ if not in protocol:** NA. | **Data type:** Categorical.  **Continuous covariate categorised:** NA.  **How determine it was categorised:** NA.  **Justification for categorising:** NA.  **Where justification reported:** NA.  **Categories reported:** Yes.  **Where categories reported:** Protocol and review.  **Justification for categories:** Not reported.  **Where justification reported:** NA. | **Analysis type reported in protocol:** Subgroup analyses.  **Analysis type reported in review:** Subgroup analyses.  **Reason for changing mind:** NA.    **Analysis type in protocol according to glossary:** Subgroup analyses.  **How determined the analysis type:** Says **‘**Subgroup analyses.’. Not reason to dispute.  **Analysis type in review according to glossary:** Subgroup analyses.  **How determined the analysis type:** Says **‘**Subgroup analyses.’. Not reason to dispute.  **Analysed:** No.  **Reasons given (in the review) for not doing interaction analysis if planned:** Not reported (presumably because one trial).  **Reasons given for carrying out interaction analysis if they weren’t planned:** NA.  **Scenario in protocol: ‘**If the authors include a sufficient number of trials and identify  moderate, substantial or considerable heterogeneity (see ’Assessment of heterogeneity’) they plan to carry out the following subgroup analyses:’  **Rationale for planned scenario:** Not reported.  **Scenario in review (if reported specifically): ‘**In future updates, if a sufficient number of trials are included and if the authors identify moderate, substantial or considerable heterogeneity (see ’Assessment of heterogeneity’), they plan to carry  out the following subgroup analyses based on:’  **Rationale for done scenario:** Not reported.  **Reason for changing mind:** NA. | **Method to detect interactions reported in protocol:** Not reported.  **Method to detect interactions reported in review:** Not reported.  **Reason for changing mind:** NA. |
| van Zuuren 2013 | **Protocol:** Not reported.  **Review methods:** Not reported.  **Review results:** Outcome time point. Day 2 and 3, day 4.  **Type:** Outcome.  **Covariate summary:** Time point. | **Method to choose**  **covariate:** Not reported.  **Where method reported:** NA.  **Rationale for choosing the covariate:** Not reported.  **Where rationale reported:** NA. | **Where reported:** Review only.  **Reason not reported in review:** NA.  **Reason not reported in protocol:** Not reported.  **Labelled as ‘posthoc’ if not in protocol:** Not reported. | **Data type:** Categorical.  **Continuous covariate categorised:** NA.  **How determine it was categorised:** NA.  **Justification for categorising:** NA.  **Where justification reported:** NA.  **Categories reported:** Yes.  **Where categories reported:** Review only.  **Justification for categories:** Not reported.  **Where justification reported:** NA. | **Analysis type reported in protocol:** Not reported.  **Analysis type reported in review:** Unnamed analysis.  **Reason for changing mind:** Not reported.    **Analysis type in protocol according to glossary:** Not reported.  **How determined the analysis type:** NA.  **Analysis type in review according to glossary:** Stratification/subgroup analysis.  **How determined the analysis type:** Categories given.  **Analysed:** Yes.  **Reasons given (in the review) for not doing interaction analysis if planned:** NA.  **Reasons given for carrying out interaction analysis if they weren’t planned:** Not reported.  **Scenario in protocol:** Not reported.  **Rationale for planned scenario:** NA.  **Scenario in review (if reported specifically):** Not reported.  **Rationale for done scenario:** NA.  **Reason for changing mind:** NA. | **Method to detect interactions reported in protocol:** Not reported.  **Method to detect interactions reported in review:** Not reported.  **Reason for changing mind:** NA. |
| Wakai 2013 | **Protocol:** ‘Good quality studies versus poor quality studies’. A good quality study will be defined as one which has all the following domains: adequate allocation concealment, blinding of outcome assessment and data analysis performed according to the intention-to-treat principle. A poor quality study will be defined as one which lacks one or more of these key domains.’  **Review methods:** ‘Good quality studies versus poor quality studies’. A good quality study will be defined as one which has all the following domains: adequate allocation concealment, blinding of outcome assessment and data analysis performed according to the intention-to-treat principle. A poor quality study will be defined as one which lacks one or more of these key domains.’  **Review results:** Not analysed.  **Type:** Methodological.  **Covariate summary:** Quality. | **Method to choose**  **covariate:** Not reported.  **Where method reported:** NA.  **Rationale for choosing the covariate:** Not reported.  **Where rationale reported:** NA. | **Where reported:** Protocol and review.  **Reason not reported in review:** NA.  **Reason not reported in protocol:** NA.  **Labelled as ‘posthoc’ if not in protocol:** NA. | **Data type:** Categorical.  **Continuous covariate categorised:** NA.  **How determine it was categorised:** NA.  **Justification for categorising:** NA.  **Where justification reported:** NA.  **Categories reported:** Yes.  **Where categories reported:** Protocol and review.  **Justification for categories:** Not reported.  **Where justification reported:** NA. | **Analysis type reported in protocol:** Sensitivity analysis.  **Analysis type reported in review:** Sensitivity analysis.  **Reason for changing mind:** NA.    **Analysis type in protocol according to glossary:** Subgroup analysis.  **How determined the analysis type:** Categories given.  **Analysis type in review according to glossary:** Subgroup analysis.  **How determined the analysis type:** Categories given.  **Analysed:** No.  **Reasons given (in the review) for not doing interaction analysis if planned:** Not reported.  **Reasons given for carrying out interaction analysis if they weren’t planned:** NA.  **Scenario in protocol:** Not reported.  **Rationale for planned scenario:** NA.  **Scenario in review (if reported specifically):** Not reported.  **Rationale for done scenario:** NA.  **Reason for changing mind:** NA. | **Method to detect interactions reported in protocol:** Not reported.  **Method to detect interactions reported in review:** Not reported.  **Reason for changing mind:** NA. |
| Wakai 2013 | **Protocol:** ‘Randomised versus quasi randomised’.  **Review methods:** ‘Randomised versus quasi randomised’.  **Review results:** Not analysed.  **Type:** Methodological.  **Covariate summary:** Random generation. | **Method to choose**  **covariate:** Not reported.  **Where method reported:** NA.  **Rationale for choosing the covariate:** Not reported.  **Where rationale reported:** NA. | **Where reported:** Protocol and review.  **Reason not reported in review:** NA.  **Reason not reported in protocol:** NA.  **Labelled as ‘posthoc’ if not in protocol:** NA | **Data type:** Categorical.  **Continuous covariate categorised:** NA.  **How determine it was categorised:** NA.  **Justification for categorising:** NA.  **Where justification reported:** NA.  **Categories reported:** Yes.  **Where categories reported:** Protocol and review.  **Justification for categories:** Not reported.  **Where justification reported:** NA. | **Analysis type reported in protocol:** Sensitivity analysis.  **Analysis type reported in review:** Sensitivity analysis.  **Reason for changing mind:** NA.    **Analysis type in protocol according to glossary:** Subgroup analysis.  **How determined the analysis type:** Categories given.  **Analysis type in review according to glossary:** Subgroup analysis.  **How determined the analysis type:** Categories given.  **Analysed:** No.  **Reasons given (in the review) for not doing interaction analysis if planned:** Not reported.  **Reasons given for carrying out interaction analysis if they weren’t planned:** NA.  **Scenario in protocol:** Not reported.  **Rationale for planned scenario:** NA.  **Scenario in review (if reported specifically):** Not reported.  **Rationale for done scenario:** NA.  **Reason for changing mind:** NA. | **Method to detect interactions reported in protocol:** Not reported.  **Method to detect interactions reported in review:** Not reported.  **Reason for changing mind:** NA. |
| Wakai 2013 | **Protocol:** ‘Subgroup analysis will performed for the effects of nitrates on the subset of patients with acute heart failure complicating acute myocardial infarction.’  **Review methods:** ‘We performed subgroup analysis for the effects of nitrates on the subset of patients with AHFS complicating acute myocardial infarction.’  **Review results:** Not analysed.  **Type:** Patient.  **Covariate summary:** Disease characteristics. | **Method to choose**  **covariate:** Not reported.  **Where method reported:** NA.  **Rationale for choosing the covariate:** ‘The effect of nitrates on heart failure complicating acute myocardial  infarction is poorly defined. Subgroup analysis will performed  for the effects of nitrates on the subset of patients with  acute heart failure complicating acutemyocardial infarction.Heart  failure complicating myocardial infarction is associated with a 3-  4 fold increase in hospital mortality (Hellermann 2002; Spencer  1999; Spencer 2002; Steg 2004; Wu 2002). Depending on study  design, the incidence of heart failure after myocardial infarction  varies greatly from 3% to 53% (Ambrosioni 1995; Bueno 1995;  Hellermann 2002). Meanwhile, because of their haemodynamically  beneficial effects, nitrates are recommended in heart failure  (Hunt 2001) and acute coronary syndromes (Peacock 2004),  respectively. Intravenous nitrate vasodilators are well tolerated in  acutemyocardial infarction with clinically significant hypotension  occurs in less than 4% of patients (GISSI-3 1994), but this responds  to dose reduction and fluid replacement. However, two  megatrials have failed to demonstrate any mortality reduction attributable  to nitrate use in acute coronary syndromes (GISSI-3  1994; ISIS-4 1995). The primary reason for the use of nitrates  in acute coronary syndromes is a beneficial haemodynamic effect  and to decrease the pain of myocardial ischaemia (Peacock 2004).’  **Where rationale reported:** Protocol. | **Where reported:** Protocol and review.  **Reason not reported in review:** NA.  **Reason not reported in protocol:** NA.  **Labelled as ‘posthoc’ if not in protocol:** NA | **Data type:** Categorical.  **Continuous covariate categorised:** NA.  **How determine it was categorised:** NA.  **Justification for categorising:** NA.  **Where justification reported:** NA.  **Categories reported:** Not reported.  **Where categories reported:** NA.  **Justification for categories:** Not reported.  **Where justification reported:** NA. | **Analysis type reported in protocol:** Subgroup analysis.  **Analysis type reported in review:** Subgroup analysis.  **Reason for changing mind:** NA.    **Analysis type in protocol according to glossary:** Subgroup analysis.  **How determined the analysis type:** Says’Subgroup analysis.’ No reason to dispute.  **Analysis type in review according to glossary:** Subgroup analysis.  **How determined the analysis type:** Says’Subgroup analysis.’ No reason to dispute.  **Analysed:** No.  **Reasons given (in the review) for not doing interaction analysis if planned:** Not reported.  **Reasons given for carrying out interaction analysis if they weren’t planned:** NA.  **Scenario in protocol:** Not reported.  **Rationale for planned scenario:** NA.  **Scenario in review (if reported specifically):** Not reported.  **Rationale for done scenario:** NA.  **Reason for changing mind:** NA. | **Method to detect interactions reported in protocol:** Not reported.  **Method to detect interactions reported in review:** Not reported.  **Reason for changing mind:** NA. |
| Wakai 2013 | **Protocol:** Not reported.  **Review methods:** Not reported.  **Review results:** Time point: 30minutes, 60 minutes, 3 hours, 24 hours  **Type:** Outcome.  **Covariate summary:** Time point. | **Method to choose**  **covariate:** Not reported.  **Where method reported:** NA.  **Rationale for choosing the covariate:** Not reported.  **Where rationale reported:** NA. | **Where reported:** Review only.  **Reason not reported in review:** NA.  **Reason not reported in protocol:** Not reported.  **Labelled as ‘posthoc’ if not in protocol:** Not reported. | **Data type:** Categorical.  **Continuous covariate categorised:** NA.  **How determine it was categorised:** NA.  **Justification for categorising:** NA.  **Where justification reported:** NA.  **Categories reported:** Yes.  **Where categories reported:** Review only.  **Justification for categories:** Not reported.  **Where justification reported:** NA. | **Analysis type reported in protocol:** Not reported.  **Analysis type reported in review:** Unnamed analysis.  **Reason for changing mind:** Not reported.    **Analysis type in protocol according to glossary:** Not reported.  **How determined the analysis type:** NA.  **Analysis type in review according to glossary:** Stratification analysis.  **How determined the analysis type:** Categories given.  **Analysed:** Yes.  **Reasons given (in the review) for not doing interaction analysis if planned:** NA.  **Reasons given for carrying out interaction analysis if they weren’t planned:** Not reported.  **Scenario in protocol:** Not reported.  **Rationale for planned scenario:** NA.  **Scenario in review (if reported specifically):** Not reported.  **Rationale for done scenario:** NA.  **Reason for changing mind:** NA. | **Method to detect interactions reported in protocol:** Not reported.  **Method to detect interactions reported in review:** Not reported.  **Reason for changing mind:** NA. |
| Wang 2013 | **Protocol:** ‘Condition of individuals’.  **Review methods:** Not reported.  **Review results:** Not analysed.  **Type:** Patient.  **Covariate summary:** Disease characteristics. | **Method to choose**  **covariate:** Not reported.  **Where method reported:** NA.  **Rationale for choosing the covariate:** Not reported.  **Where rationale reported:** NA. | **Where reported:** Protocol only.  **Reason not reported in review:** Not reported.  **Reason not reported in protocol:** NA.  **Labelled as ‘posthoc’ if not in protocol:** NA. | **Data type:** Unclear.  **Continuous covariate categorised:** Unclear.  **How determine it was categorised:** NA.  **Justification for categorising:** NA.  **Where justification reported:** NA.  **Categories reported:** Not reported.  **Where categories reported:** NA.  **Justification for categories:** Not reported.  **Where justification reported:** NA. | **Analysis type reported in protocol:** Subgroup analysis.  **Analysis type reported in review:** Not reported.  **Reason for changing mind:** Not reported.    **Analysis type in protocol according to glossary:** Subgroup analysis.  **How determined the analysis type:** Says ‘Subgroup analysis.’ No reason to dispute.  **Analysis type in review according to glossary:** Not reported.  **How determined the analysis type:** NA.  **Analysed:** No.  **Reasons given (in the review) for not doing interaction analysis if planned:** Not reported (presumably because one trial).  **Reasons given for carrying out interaction analysis if they weren’t planned:** NA.  **Scenario in protocol:** ‘If significant heterogeneity is found,’  **Rationale for planned scenario:** Not reported.  **Scenario in review (if reported specifically):** Not reported.  **Rationale for done scenario:** NA.  **Reason for changing mind:** NA. | **Method to detect interactions reported in protocol:** Not reported.  **Method to detect interactions reported in review:** Not reported.  **Reason for changing mind:** NA. |
| Wang 2013 | **Protocol:** ‘Methodological quality’.  **Review methods:** Not reported.  **Review results:** Not analysed.  **Type:** Methodological.  **Covariate summary:** Quality. | **Method to choose**  **covariate:** Not reported.  **Where method reported:** NA.  **Rationale for choosing the covariate:** Not reported.  **Where rationale reported:** NA. | **Where reported:** Protocol only.  **Reason not reported in review:** Not reported.  **Reason not reported in protocol:** NA.  **Labelled as ‘posthoc’ if not in protocol:** NA. | **Data type:** Categorical.  **Continuous covariate categorised:** NA.  **How determine it was categorised:** NA.  **Justification for categorising:** NA.  **Where justification reported:** NA.  **Categories reported:** Not reported.  **Where categories reported:** NA.  **Justification for categories:** Not reported.  **Where justification reported:** NA. | **Analysis type reported in protocol:** Subgroup analysis.  **Analysis type reported in review:** Not reported.  **Reason for changing mind:** Not reported.    **Analysis type in protocol according to glossary:** Subgroup analysis.  **How determined the analysis type:** Says ‘Subgroup analysis.’ No reason to dispute.  **Analysis type in review according to glossary:** Not reported.  **How determined the analysis type:** NA.  **Analysed:** No.  **Reasons given (in the review) for not doing interaction analysis if planned:** Not reported (presumably because one trial).  **Reasons given for carrying out interaction analysis if they weren’t planned:** NA.  **Scenario in protocol:** ‘If significant heterogeneity is found,’  **Rationale for planned scenario:** Not reported.  **Scenario in review (if reported specifically):** Not reported.  **Rationale for done scenario:** NA.  **Reason for changing mind:** NA. | **Method to detect interactions reported in protocol:** Not reported.  **Method to detect interactions reported in review:** Not reported.  **Reason for changing mind:** NA. |
| Wang 2013 | **Protocol:** ‘Sex of patients’.  **Review methods:** Not reported.  **Review results:** Not analysed.  **Type:** Patient.  **Covariate summary:** Demographics. | **Method to choose**  **covariate:** Not reported.  **Where method reported:** NA.  **Rationale for choosing the covariate:** Not reported.  **Where rationale reported:** NA. | **Where reported:** Protocol only.  **Reason not reported in review:** Not reported.  **Reason not reported in protocol:** NA.  **Labelled as ‘posthoc’ if not in protocol:** NA. | **Data type:** Categorical.  **Continuous covariate categorised:** NA.  **How determine it was categorised:** NA.  **Justification for categorising:** NA.  **Where justification reported:** NA.  **Categories reported:** Not reported.  **Where categories reported:** NA.  **Justification for categories:** Not reported.  **Where justification reported:** NA. | **Analysis type reported in protocol:** Subgroup analysis.  **Analysis type reported in review:** Not reported.  **Reason for changing mind:** Not reported.    **Analysis type in protocol according to glossary:** Subgroup analysis.  **How determined the analysis type:** Says ‘Subgroup analysis.’ No reason to dispute.  **Analysis type in review according to glossary:** Not reported.  **How determined the analysis type:** NA.  **Analysed:** No.  **Reasons given (in the review) for not doing interaction analysis if planned:** Not reported (presumably because one trial).  **Reasons given for carrying out interaction analysis if they weren’t planned:** NA.  **Scenario in protocol:** Not reported.  **Rationale for planned scenario:** NA.  **Scenario in review (if reported specifically):** Not reported.  **Rationale for done scenario:** NA.  **Reason for changing mind:** NA. | **Method to detect interactions reported in protocol:** Not reported.  **Method to detect interactions reported in review:** Not reported.  **Reason for changing mind:** NA. |
| Wang 2013 | **Protocol:** ‘The following comparisons will be addressed: 1. Acupuncture versus placebo or no treatment,. 2. Acupuncture versus any other treatment.’  **Review methods: ‘**The following comparisons will be addressed: 1. Acupuncture versus placebo or no treatment. 2. Acupuncture versus any other treatment.’  **Review results:** ‘Acupuncture versus placebo or no treatment’; ‘Acupuncture versus active treatment’.  **Type:** Intervention.  **Covariate summary:** Type of intervention or control. | **Method to choose**  **covariate:** Not reported.  **Where method reported:** NA.  **Rationale for choosing the covariate:** Not reported.  **Where rationale reported:** NA. | **Where reported:** Protocol and review.  **Reason not reported in review:** NA.  **Reason not reported in protocol:** NA.  **Labelled as ‘posthoc’ if not in protocol:** NA. | **Data type:** Categorical.  **Continuous covariate categorised:** NA.  **How determine it was categorised:** NA.  **Justification for categorising:** NA.  **Where justification reported:** NA.  **Categories reported:** Yes.  **Where categories reported:** Protocol and review.  **Justification for categories:** Not reported.  **Where justification reported:** NA. | **Analysis type reported in protocol:** Unnamed analysis.  **Analysis type reported in review:** Unnamed analysis.  **Reason for changing mind:** NA.    **Analysis type in protocol according to glossary:** Stratification/subgroup analysis.  **How determined the analysis type:** Categories given.  **Analysis type in review according to glossary:** Stratification/subgroup analysis.  **How determined the analysis type:** Categories given.  **Analysed:** Yes.  **Reasons given (in the review) for not doing interaction analysis if planned:** NA.  **Reasons given for carrying out interaction analysis if they weren’t planned:** NA.  **Scenario in protocol:** Not reported.  **Rationale for planned scenario:** NA.  **Scenario in review (if reported specifically):** Not reported.  **Rationale for done scenario:** NA.  **Reason for changing mind:** NA. | **Method to detect interactions reported in protocol:** Not reported.  **Method to detect interactions reported in review:** Not reported.  **Reason for changing mind:** NA. |
| Wang 2013 | **Protocol:** Outlying results. We judged the following to be two covariates: ‘We will assess the study quality and consider inclusion of trials without high risk of bias or outlying results.’  **Review methods:** Not reported.  **Review results:** Not analysed.  **Type:** Other.  **Covariate summary:** Outlying results. | **Method to choose**  **covariate:** Not reported.  **Where method reported:** NA.  **Rationale for choosing the covariate:** Not reported.  **Where rationale reported:** NA. | **Where reported:** Protocol only.  **Reason not reported in review:** Not reported.  **Reason not reported in protocol:** NA.  **Labelled as ‘posthoc’ if not in protocol:** NA. | **Data type:** Categorical.  **Continuous covariate categorised:** NA.  **How determine it was categorised:** NA.  **Justification for categorising:** NA.  **Where justification reported:** NA.  **Categories reported:** Yes.  **Where categories reported:** Protocol only.  **Justification for categories:** Not reported.  **Where justification reported:** NA. | **Analysis type reported in protocol:** Sensitivity analysis.  **Analysis type reported in review:** Not reported.  **Reason for changing mind:** Not reported.    **Analysis type in protocol according to glossary:** Sensitivity analysis.  **How determined the analysis type:** Says ‘Sensitivity analysis..’ No reason to dispute.  **Analysis type in review according to glossary:** Not reported.  **How determined the analysis type:** NA.  **Analysed:** No.  **Reasons given (in the review) for not doing interaction analysis if planned:** Not reported (presumably because one trial).  **Reasons given for carrying out interaction analysis if they weren’t planned:** NA.  **Scenario in protocol:** ‘If there are an adequate number of studies, we will conduct a  sensitivity analysis,’  **Rationale for planned scenario:** Not reported.  **Scenario in review (if reported specifically):** Not reported.  **Rationale for done scenario:** NA.  **Reason for changing mind:** NA. | **Method to detect interactions reported in protocol:** Not reported.  **Method to detect interactions reported in review:** Not reported.  **Reason for changing mind:** NA. |
| Wang 2013 | **Protocol:** Risk of bias. We judged the following to be two covariates: ‘We will assess the study quality and consider inclusion of trials without high risk of bias or outlying results.’  **Review methods:** Not reported.  **Review results:** Not analysed.  **Type:** Methodological.  **Covariate summary:** Risk of bias. | **Method to choose**  **covariate:** Not reported.  **Where method reported:** NA.  **Rationale for choosing the covariate:** Not reported.  **Where rationale reported:** NA. | **Where reported:** Protocol only.  **Reason not reported in review:** Not reported.  **Reason not reported in protocol:** NA.  **Labelled as ‘posthoc’ if not in protocol:** NA. | **Data type:** Categorical.  **Continuous covariate categorised:** NA.  **How determine it was categorised:** NA.  **Justification for categorising:** NA.  **Where justification reported:** NA.  **Categories reported:** Yes.  **Where categories reported:** Protocol only.  **Justification for categories:** Not reported.  **Where justification reported:** NA. | **Analysis type reported in protocol:** Sensitivity analysis.  **Analysis type reported in review:** Not reported.  **Reason for changing mind:** Not reported.    **Analysis type in protocol according to glossary:** Sensitivity analysis.  **How determined the analysis type:** Says ‘Sensitivity analysis..’ No reason to dispute.  **Analysis type in review according to glossary:** Not reported.  **How determined the analysis type:** NA.  **Analysed:** No.  **Reasons given (in the review) for not doing interaction analysis if planned:** Not reported (presumably because one trial).  **Reasons given for carrying out interaction analysis if they weren’t planned:** NA.  **Scenario in protocol:** ‘If there are an adequate number of studies, we will conduct a  sensitivity analysis,’  **Rationale for planned scenario:** Not reported.  **Scenario in review (if reported specifically):** Not reported.  **Rationale for done scenario:** NA.  **Reason for changing mind:** NA. | **Method to detect interactions reported in protocol:** Not reported.  **Method to detect interactions reported in review:** Not reported.  **Reason for changing mind:** NA. |
| Yue 2013 | **Protocol:** ‘Children (<18 years) and adults (>18 years)’.  **Review methods: ‘**Children (<18 years) and adults (>18 years)’.  **Review results: ‘**Children (<18 years) and adults (>18 years)’ (in text), ‘Adults subgroup (≥18 years)’ (in plot).  **Type:** Patient.  **Covariate summary:** Demographics. | **Method to choose**  **covariate:** Not reported.  **Where method reported:** NA.  **Rationale for choosing the covariate:** Not reported.  **Where rationale reported:** NA. | **Where reported:** Protocol and review.  **Reason not reported in review:** NA.  **Reason not reported in protocol:** NA.  **Labelled as ‘posthoc’ if not in protocol:** NA. | **Data type:** Continuous.  **Continuous covariate categorised:** Yes.  **How determine it was categorised:** Categories given.  **Justification for categorising:** Not reported.  **Where justification reported:** NA.  **Categories reported:** Yes.  **Where categories reported:** Protocol and review.  **Justification for categories:** Not reported.  **Where justification reported:** NA. | **Analysis type reported in protocol:** Subgroup analysis.  **Analysis type reported in review:** Subgroup analysis.  **Reason for changing mind:** NA.    **Analysis type in protocol according to glossary:** Subgroup analysis.  **How determined the analysis type:** Categories given.  **Analysis type in review according to glossary:** Subgroup analysis.  **How determined the analysis type:** Categories given.  **Analysed:** Yes.  **Reasons given (in the review) for not doing interaction analysis if planned:** NA.  **Reasons given for carrying out interaction analysis if they weren’t planned:** NA.  **Scenario in protocol: ‘**To assess whether the treatment effect is modified by clinical and  demographic variables, we will undertake subgroup analysis’  **Rationale for planned scenario:** Not reported.  **Scenario in review (if reported specifically): ‘**To assess whether the treatment effect is modified by clinical and  demographic variables, we undertook subgroup analyses’  **Rationale for done scenario:** Not reported.  **Reason for changing mind:** NA. | **Method to detect interactions reported in protocol:** Not reported.  **Method to detect interactions reported in review:** Not reported.  **Reason for changing mind:** NA. |
| Yue 2013 | **Protocol:** ‘Exclusion of studies in which outcome evaluation was not blinded’.  **Review methods: ‘**Exclusion of studies in which outcome evaluation was not blinded’.  **Review results: ‘**Exclusion of studies in which outcome evaluation was not blinded’.  **Type:** Methodological.  **Covariate summary:** Blinding. | **Method to choose**  **covariate:** Not reported.  **Where method reported:** NA.  **Rationale for choosing the covariate:** Not reported.  **Where rationale reported:** NA. | **Where reported:** Protocol and review.  **Reason not reported in review:** NA.  **Reason not reported in protocol:** NA.  **Labelled as ‘posthoc’ if not in protocol:** NA. | **Data type:** Categorical.  **Continuous covariate categorised:** NA.  **How determine it was categorised:** NA.  **Justification for categorising:** NA.  **Where justification reported:** NA.  **Categories reported:** Yes.  **Where categories reported:** Protocol and review.  **Justification for categories:** Not reported.  **Where justification reported:** NA. | **Analysis type reported in protocol:** Sensitivity analysis.  **Analysis type reported in review:** Sensitivity analysis.  **Reason for changing mind:** NA.    **Analysis type in protocol according to glossary:** Sensitivity analysis..  **How determined the analysis type:** Categories given.  **Analysis type in review according to glossary:** Sensitivity analysis..  **How determined the analysis type:** Categories given.  **Analysed:** Yes.  **Reasons given (in the review) for not doing interaction analysis if planned:** NA.  **Reasons given for carrying out interaction analysis if they weren’t planned:** NA.  **Scenario in protocol: ‘**If a sufficient number of trials are found, sensitivity analysis will  be done to assess the robustness of the results’  **Rationale for planned scenario:** Not reported.  **Scenario in review (if reported specifically): ‘**If a sufficient number of trials were found, sensitivity analysis  would be done to assess the robustness of the results as follows:’  **Rationale for done scenario:** Not reported.  **Reason for changing mind:** NA. | **Method to detect interactions reported in protocol:** Not reported.  **Method to detect interactions reported in review:** Not reported.  **Reason for changing mind:** NA. |
| Yue 2013 | **Protocol:** ‘Exclusion of studies with inadequate concealment of allocation’.  **Review methods: ‘**Exclusion of studies with inadequate concealment of allocation’.  **Review results: ‘**Exclusion of studies with inadequate concealment of allocation’.  **Type:** Methodological.  **Covariate summary:** Allocation concealment. | **Method to choose**  **covariate:** Not reported.  **Where method reported:** NA.  **Rationale for choosing the covariate:** Not reported.  **Where rationale reported:** NA. | **Where reported:** Protocol and review.  **Reason not reported in review:** NA.  **Reason not reported in protocol:** NA.  **Labelled as ‘posthoc’ if not in protocol:** NA. | **Data type:** Categorical.  **Continuous covariate categorised:** NA.  **How determine it was categorised:** NA.  **Justification for categorising:** NA.  **Where justification reported:** NA.  **Categories reported:** Yes.  **Where categories reported:** Protocol and review.  **Justification for categories:** Not reported.  **Where justification reported:** NA. | **Analysis type reported in protocol:** Sensitivity analysis.  **Analysis type reported in review:** Sensitivity analysis.  **Reason for changing mind:** NA.    **Analysis type in protocol according to glossary:** Sensitivity analysis..  **How determined the analysis type:** Categories given.  **Analysis type in review according to glossary:** Sensitivity analysis..  **How determined the analysis type:** Categories given.  **Analysed:** No.  **Reasons given (in the review) for not doing interaction analysis if planned:** ‘Allocation concealment was unclear in all included RCTs and,  therefore, this analysis was not undertaken.’  **Reasons given for carrying out interaction analysis if they weren’t planned:** NA.  **Scenario in protocol: ‘**If a sufficient number of trials are found, sensitivity analysis will  be done to assess the robustness of the results’  **Rationale for planned scenario:** Not reported.  **Scenario in review (if reported specifically): ‘**If a sufficient number of trials were found, sensitivity analysis  would be done to assess the robustness of the results as follows:’  **Rationale for done scenario:** Not reported.  **Reason for changing mind:** NA. | **Method to detect interactions reported in protocol:** Not reported.  **Method to detect interactions reported in review:** Not reported.  **Reason for changing mind:** NA. |
| Yue 2013 | **Protocol: ‘**Uncomplicated SSTIs and complicated SSTIs’.  **Review methods: ‘**Uncomplicated SSTIs and complicated SSTIs’.  **Review results:** Not analysed.  **Type:** Patient.  **Covariate summary:** Disease characteristics. | **Method to choose**  **covariate:** Not reported.  **Where method reported:** NA.  **Rationale for choosing the covariate:** Not reported.  **Where rationale reported:** NA. | **Where reported:** Protocol and review.  **Reason not reported in review:** NA.  **Reason not reported in protocol:** NA.  **Labelled as ‘posthoc’ if not in protocol:** NA. | **Data type:** Categorical.  **Continuous covariate categorised:** NA.  **How determine it was categorised:** NA.  **Justification for categorising:** NA.  **Where justification reported:** NA.  **Categories reported:** Yes.  **Where categories reported:** Protocol and review.  **Justification for categories:** Not reported.  **Where justification reported:** NA. | **Analysis type reported in protocol:** Subgroup analysis.  **Analysis type reported in review:** Subgroup analysis.  **Reason for changing mind:** NA.    **Analysis type in protocol according to glossary:** Subgroup analysis.  **How determined the analysis type:** Categories given.  **Analysis type in review according to glossary:** Subgroup analysis.  **How determined the analysis type:** Categories given.  **Analysed:** No.  **Reasons given (in the review) for not doing interaction analysis if planned:** ‘No trial reported uncomplicated SSTIs. This meant that there  were insufficient data to permit this subgroup analysis.’  **Reasons given for carrying out interaction analysis if they weren’t planned:** NA.  **Scenario in protocol: ‘**To assess whether the treatment effect is modified by clinical and  demographic variables, we will undertake subgroup analysis’  **Rationale for planned scenario:** Not reported.  **Scenario in review (if reported specifically): ‘**To assess whether the treatment effect is modified by clinical and  demographic variables, we undertook subgroup analyses’  **Rationale for done scenario:** Not reported.  **Reason for changing mind:** NA. | **Method to detect interactions reported in protocol:** Not reported.  **Method to detect interactions reported in review:** Not reported.  **Reason for changing mind:** NA. |
| Yue 2013 | **Protocol:** ‘We intend to present comparisons as follows: 1. Linezolid compared with vancomycin alone. 2. Linezolid plus co-interventions compared with vancomycin plus co-interventions’.  **Review methods: ‘**We intend to present comparisons as follows: 1. Linezolid compared with vancomycin alone. 2. Linezolid plus co-interventions compared with vancomycin plus co-interventions’.  **Review results:** ‘Linezolid compared with vancomycin’.  **Type:** Intervention.  **Covariate summary:** Additional interventions. | **Method to choose**  **covariate:** Not reported.  **Where method reported:** NA.  **Rationale for choosing the covariate:** Not reported.  **Where rationale reported:** NA. | **Where reported:** Protocol and review.  **Reason not reported in review:** NA.  **Reason not reported in protocol:** NA.  **Labelled as ‘posthoc’ if not in protocol:** NA. | **Data type:** Categorical.  **Continuous covariate categorised:** NA.  **How determine it was categorised:** NA.  **Justification for categorising:** NA.  **Where justification reported:** NA.  **Categories reported:** Yes.  **Where categories reported:** Protocol and review.  **Justification for categories:** Not reported.  **Where justification reported:** NA. | **Analysis type reported in protocol:** Unnamed analysis.  **Analysis type reported in review:** Unnamed analysis.  **Reason for changing mind:** NA.    **Analysis type in protocol according to glossary:** Stratification/subgroup analysis.  **How determined the analysis type:** Categories given.  **Analysis type in review according to glossary:** Stratification/subgroup analysis.  **How determined the analysis type:** Categories given.  **Analysed:** Yes.  **Reasons given (in the review) for not doing interaction analysis if planned:** NA.  **Reasons given for carrying out interaction analysis if they weren’t planned:** NA.  **Scenario in protocol:** Not reported.  **Rationale for planned scenario:** NA.  **Scenario in review (if reported specifically):** Not reported.**Rationale for done scenario:** NA.  **Reason for changing mind:** NA. | **Method to detect interactions reported in protocol:** Not reported.  **Method to detect interactions reported in review:** Not reported.  **Reason for changing mind:** NA. |
| Yue 2013 | **Protocol:** Not reported.  **Review methods:** MRSA subset**.**  **Review results:** MRSA subset**.**  **Type:** Patient.  **Covariate summary:** Disease characteristics. | **Method to choose**  **covariate:** Not reported.  **Where method reported:** NA.  **Rationale for choosing the covariate:** ‘In the section ”Subgroup analysis and investigation of heterogeneity“, we added:”3. MRSA subset“, because MRSA is important  for SSTIs. The morbidity and treatment costs associated with MRSA-infected SSTIs are higher than for other pathogen infections, so we added this subgroup analysis.’  **Where rationale reported:** Review only. | **Where reported:** Review only.  **Reason not reported in review:** NA.  **Reason not reported in protocol:** Not reported.  **Labelled as ‘posthoc’ if not in protocol:** Not reported. | **Data type:** Categorical.  **Continuous covariate categorised:** NA.  **How determine it was categorised:** NA.  **Justification for categorising:** NA.  **Where justification reported:** NA.  **Categories reported:** Yes  **Where categories reported:** Review only.  **Justification for categories:** ‘The morbidity and treatment costs associated with MRSA-infected SSTIs are higher than for other pathogen infections, so we added this subgroup analysis.’  **Where justification reported:** Review only. | **Analysis type reported in protocol:** Not reported.  **Analysis type reported in review:** Subgroup analysis.  **Reason for changing mind:** Not reported.    **Analysis type in protocol according to glossary:** Not reported.  **How determined the analysis type:** NA.  **Analysis type in review according to glossary:** Sensitivity analysis.  **How determined the analysis type:** Results presented.  **Analysed:** Yes.  **Reasons given (in the review) for not doing interaction analysis if planned:** NA.  **Reasons given for carrying out interaction analysis if they weren’t planned:** ‘In the section ”Subgroup analysis and investigation of heterogeneity“, we added:”3. MRSA subset“, because MRSA is important for SSTIs. The morbidity and treatment costs associated with MRSA-infected SSTIs are higher than for other pathogen infections, so  we added this subgroup analysis.’  **Scenario in protocol:** Not reported.  **Rationale for planned scenario:** NA.  **Scenario in review (if reported specifically): ‘**To assess whether the treatment effect is modified by clinical and demographic variables, we undertook subgroup analyses’  **Rationale for done scenario:** Not reported.  **Reason for changing mind:** NA. | **Method to detect interactions reported in protocol:** Not reported.  **Method to detect interactions reported in review:** Not reported.  **Reason for changing mind:** NA. |
| Ziebell 2013 | **Protocol:** ‘Assessment of the benefits and harms of flow-regulated valve compared with differential pressure-regulated valves when excluding data from trials only published as abstracts.’  **Review methods:** Not reported.  **Review results:** Not analysed.  **Type:** Methodological.  **Covariate summary:** Publication status. | **Method to choose**  **covariate:** Not reported.  **Where method reported:** NA.  **Rationale for choosing the covariate:** Not reported.  **Where rationale reported:** NA. | **Where reported:** Protocol only.  **Reason not reported in review:** Not reported.  **Reason not reported in protocol:** NA.  **Labelled as ‘posthoc’ if not in protocol:** NA. | **Data type:** Categorical.  **Continuous covariate categorised:** NA.  **How determine it was categorised:** NA.  **Justification for categorising:** NA.  **Where justification reported:** NA.  **Categories reported:** Yes.  **Where categories reported:** Protocol only.  **Justification for categories:** Not reported.  **Where justification reported:** NA. | **Analysis type reported in protocol:** Sensitivity analysis.  **Analysis type reported in review:** Not reported.  **Reason for changing mind:** Not reported (presumably because no trials).  **Analysis type in protocol according to glossary:** Sensitivity analysis.  **How determined the analysis type:** Says ‘excluding’.  **Analysis type in review according to glossary:** Not reported.  **How determined the analysis type:** NA.  **Analysed:** No.  **Reasons given (in the review) for not doing interaction analysis if planned:** Not reported (presumably because no trials).  **Reasons given for carrying out interaction analysis if they weren’t planned:** NA.  **Scenario in protocol:** ‘in cases of I 2 > 0 (for the primary outcomes) we will seek to determine the cause of heterogeneity by performing meta-regression analyses and  relevant subgroup and sensitivity analyses.’  **Rationale for planned scenario:** Not reported.  **Scenario in review (if reported specifically):** Not reported.  **Rationale for done scenario:** NA.  **Reason for changing mind:** Not reported (presumably because no trials). | **Method to detect interactions reported in protocol:** Not reported.  **Method to detect interactions reported in review:** Not reported.  **Reason for changing mind: NA.** |
| Ziebell 2013 | **Protocol:** ‘Assessment of the benefits and harms of flow-regulated valve compared with differential pressure-regulated valves when excluding data from trials with commercial funding.’  **Review methods:** Not reported.  **Review results:** Not analysed.  **Type:** Methodological.  **Covariate summary:** Funding. | **Method to choose**  **covariate:** Not reported.  **Where method reported:** NA.  **Rationale for choosing the covariate:** Not reported.  **Where rationale reported:** NA. | **Where reported:** Protocol only.  **Reason not reported in review:** Not reported.  **Reason not reported in protocol:** NA.  **Labelled as ‘posthoc’ if not in protocol:** NA. | **Data type:** Categorical.  **Continuous covariate categorised:** NA.  **How determine it was categorised:** NA.  **Justification for categorising:** NA.  **Where justification reported:** NA.  **Categories reported:** Yes.  **Where categories reported:** Protocol only.  **Justification for categories:** Not reported.  **Where justification reported:** NA. | **Analysis type reported in protocol:** Sensitivity analysis.  **Analysis type reported in review:** Not reported.  **Reason for changing mind:** Not reported (presumably because no trials).  **Analysis type in protocol according to glossary:** Sensitivity analysis.  **How determined the analysis type:** Says ‘excluding’.  **Analysis type in review according to glossary:** Not reported.  **How determined the analysis type:** NA.  **Analysed:** No.  **Reasons given (in the review) for not doing interaction analysis if planned:** Not reported (presumably because no trials).  **Reasons given for carrying out interaction analysis if they weren’t planned:** NA.  **Scenario in protocol:** ‘in cases of I 2 > 0 (for the primary outcomes) we will seek to determine the cause of heterogeneity by performing meta-regression analyses and  relevant subgroup and sensitivity analyses.’  **Rationale for planned scenario:** Not reported.  **Scenario in review (if reported specifically):** Not reported.  **Rationale for done scenario:** NA.  **Reason for changing mind:** Not reported (presumably because no trials). | **Method to detect interactions reported in protocol:** Not reported.  **Method to detect interactions reported in review:** Not reported.  **Reason for changing mind: NA.** |
| Ziebell 2013 | **Protocol:** ‘Comparing estimates of the pooled intervention effect in trials with low risk of bias to estimates from trials with high risk of bias (i. e. , trials having at least one unclear or high risk of bias component).’  **Review methods:** Not reported.  **Review results:** Not analysed.  **Type:** Methodological.  **Covariate summary:** Risk of bias. | **Method to choose**  **covariate:** Not reported.  **Where method reported:** NA.  **Rationale for choosing the covariate:** Not reported.  **Where rationale reported:** NA. | **Where reported:** Protocol only.  **Reason not reported in review:** Not reported.  **Reason not reported in protocol:** NA.  **Labelled as ‘posthoc’ if not in protocol:** NA. | **Data type:** Categorical.  **Continuous covariate categorised:** NA.  **How determine it was categorised:** NA.  **Justification for categorising:** NA.  **Where justification reported:** NA.  **Categories reported:** Yes.  **Where categories reported:** Protocol only.  **Justification for categories:** Not reported.  **Where justification reported:** NA. | **Analysis type reported in protocol:** Sensitivity analysis.  **Analysis type reported in review:** Not reported.  **Reason for changing mind:** Not reported (presumably because no trials).  **Analysis type in protocol according to glossary:** Subgroup analysis.  **How determined the analysis type:** Categories given.  **Analysis type in review according to glossary:** Not reported.  **How determined the analysis type:** NA.  **Analysed:** No.  **Reasons given (in the review) for not doing interaction analysis if planned:** Not reported (presumably because no trials).  **Reasons given for carrying out interaction analysis if they weren’t planned:** NA.  **Scenario in protocol:** ‘in cases of I 2 > 0 (for the primary outcomes) we will seek to determine the cause of heterogeneity by performing meta-regression analyses and  relevant subgroup and sensitivity analyses.’  **Rationale for planned scenario:** Not reported.  **Scenario in review (if reported specifically):** Not reported.  **Rationale for done scenario:** NA.  **Reason for changing mind:** Not reported (presumably because no trials). | **Method to detect interactions reported in protocol:** Not reported.  **Method to detect interactions reported in review:** Not reported.  **Reason for changing mind: NA.** |
| Ziebell 2013 | **Protocol:** ‘Fraction of idiopathic normal pressure hydrocephalus in the trial population at baseline.’  **Review methods:** Not reported.  **Review results:** Not analysed.  **Type:** Patient.  **Covariate summary:** Disease characteristics. | **Method to choose**  **covariate:** Not reported.  **Where method reported:** NA.  **Rationale for choosing the covariate:** Not reported.  **Where rationale reported:** NA. | **Where reported:** Protocol only.  **Reason not reported in review:** Not reported.  **Reason not reported in protocol:** NA.  **Labelled as ‘posthoc’ if not in protocol:** NA. | **Data type:** Categorical.  **Continuous covariate categorised:** NA.  **How determine it was categorised:** NA.  **Justification for categorising:** NA.  **Where justification reported:** NA.  **Categories reported:** Not reported.  **Where categories reported:** NA.  **Justification for categories:** Not reported.  **Where justification reported:** NA. | **Analysis type reported in protocol:** Meta-regression.  **Analysis type reported in review:** Not reported.  **Reason for changing mind:** Not reported (presumably because no trials).  **Analysis type in protocol according to glossary:** meta-regression.  **How determined the analysis type:** Says meta-regression.  **Analysis type in review according to glossary:** Not reported.  **How determined the analysis type:** NA.  **Analysed:** No.  **Reasons given (in the review) for not doing interaction analysis if planned:** Not reported (presumably because no trials).  **Reasons given for carrying out interaction analysis if they weren’t planned:** NA.  **Scenario in protocol:** ‘in cases of I 2 > 0 (for the primary outcomes) we will seek to determine the cause of heterogeneity by performing meta-regression analyses and  relevant subgroup and sensitivity analyses.’  **Rationale for planned scenario:** Not reported.  **Scenario in review (if reported specifically):** Not reported.  **Rationale for done scenario:** NA.  **Reason for changing mind:** Not reported (presumably because no trials). | **Method to detect interactions reported in protocol:** Not reported.  **Method to detect interactions reported in review:** Not reported.  **Reason for changing mind: NA.** |
| Ziebell 2013 | **Protocol:** ‘Mean age of trial population at baseline’.  **Review methods:** Not reported.  **Review results:** Not analysed.  **Type:** Patient.  **Covariate summary:** Demographics. | **Method to choose**  **covariate:** Not reported.  **Where method reported:** NA.  **Rationale for choosing the covariate:** Not reported.  **Where rationale reported:** NA. | **Where reported:** Protocol only.  **Reason not reported in review:** Not reported.  **Reason not reported in protocol:** NA.  **Labelled as ‘posthoc’ if not in protocol:** NA. | **Data type:** Continuous.  **Continuous covariate categorised:** Unclear.  **How determine it was categorised:** Meta-regression planned..  **Justification for categorising:** NA  **Where justification reported:** NA.  **Categories reported:** NA.  **Where categories reported:** NA.  **Justification for categories:** NA.  **Where justification reported:** NA. | **Analysis type reported in protocol:** Meta-regression.  **Analysis type reported in review:** Not reported.  **Reason for changing mind:** Not reported (presumably because no trials).  **Analysis type in protocol according to glossary:** meta-regression.  **How determined the analysis type:** Says meta-regression.  **Analysis type in review according to glossary:** Not reported.  **How determined the analysis type:** NA.  **Analysed:** No.  **Reasons given (in the review) for not doing interaction analysis if planned:** Not reported (presumably because no trials).  **Reasons given for carrying out interaction analysis if they weren’t planned:** NA.  **Scenario in protocol:** ‘in cases of I 2 > 0 (for the primary outcomes) we will seek to determine the cause of heterogeneity by performing meta-regression analyses and  relevant subgroup and sensitivity analyses.’  **Rationale for planned scenario:** Not reported.  **Scenario in review (if reported specifically):** Not reported.  **Rationale for done scenario:** NA.  **Reason for changing mind:** Not reported (presumably because no trials). | **Method to detect interactions reported in protocol:** Not reported.  **Method to detect interactions reported in review:** Not reported.  **Reason for changing mind:** NA. |
| Ziebell 2013 | **Protocol:** ± adjustable valves. We judged the following to be two covariates: ‘We will subgroup patients with the differential pressure-regulated valves into ± anti-siphoning device (applied to compensate for gravity in upright position) and ± adjustable valves; that is, the possibility to externally regulate the pressure gradient, which should in theory decrease the need for surgical revisions.’  **Review methods:** Not reported.  **Review results:** Not analysed.  **Type:** Intervention.  **Covariate summary:** Additional interventions. | **Method to choose**  **covariate:** Not reported.  **Where method reported:** NA.  **Rationale for choosing the covariate:** Not reported.  **Where rationale reported:** NA. | **Where reported:** Protocol only.  **Reason not reported in review:** Not reported.  **Reason not reported in protocol:** NA.  **Labelled as ‘posthoc’ if not in protocol:** NA. | **Data type:** Categorical.  **Continuous covariate categorised:** NA.  **How determine it was categorised:** NA.  **Justification for categorising:** NA.  **Where justification reported:** NA.  **Categories reported:** Yes.  **Where categories reported:** Protocol only.  **Justification for categories:** Not reported.  **Where justification reported:** NA. | **Analysis type reported in protocol:** Subgroup analysis.  **Analysis type reported in review:** Not reported.  **Reason for changing mind:** Not reported (presumably because no trials).  **Analysis type in protocol according to glossary:** Subgroup analysis.  **How determined the analysis type:** Categories given and says ‘subgroups’.  **Analysis type in review according to glossary:** Not reported.  **How determined the analysis type:** NA.  **Analysed:** No.  **Reasons given (in the review) for not doing interaction analysis if planned:** Not reported (presumably because no trials).  **Reasons given for carrying out interaction analysis if they weren’t planned:** NA.  **Scenario in protocol:** ‘in cases of I 2 > 0 (for the primary outcomes) we will seek to determine the cause of heterogeneity by performing meta-regression analyses and  relevant subgroup and sensitivity analyses.’  **Rationale for planned scenario:** Not reported.  **Scenario in review (if reported specifically):** Not reported.  **Rationale for done scenario:** NA.  **Reason for changing mind:** Not reported (presumably because no trials). | **Method to detect interactions reported in protocol: ‘**We will compare intervention effects in subgroups using a test of  interaction (Altman 2003). We consider P < 0.05 to be indicative  of significant interaction between the flow-regulated valve effect  on one of the co-primary outcomes and the subgroup category  (Higgins 2008, Chapters 9.6.1 and 9.7).’  **Method to detect interactions reported in review:** Not reported.  **Reason for changing mind:** Not reported (presumably because no trials). |
| Ziebell 2013 | **Protocol:** ± anti-siphoning device. We judged the following to be two covariates: ‘We will subgroup patients with the differential pressure-regulated valves into ± anti-siphoning device (applied to compensate for gravity in upright position) and ± adjustable valves; that is, the possibility to externally regulate the pressure gradient, which should in theory decrease the need for surgical revisions.’  **Review methods:** Not reported.  **Review results:** Not analysed.  **Type:** Intervention.  **Covariate summary:** Additional interventions. | **Method to choose**  **covariate:** Not reported.  **Where method reported:** NA.  **Rationale for choosing the covariate:** Not reported.  **Where rationale reported:** NA. | **Where reported:** Protocol only.  **Reason not reported in review:** Not reported.  **Reason not reported in protocol:** NA.  **Labelled as ‘posthoc’ if not in protocol:** NA. | **Data type:** Categorical.  **Continuous covariate categorised:** NA.  **How determine it was categorised:** NA.  **Justification for categorising:** NA.  **Where justification reported:** NA.  **Categories reported:** Yes.  **Where categories reported:** Protocol only.  **Justification for categories:** Not reported.  **Where justification reported:** NA. | **Analysis type reported in protocol:** Subgroup analysis.  **Analysis type reported in review:** Not reported.  **Reason for changing mind:** Not reported (presumably because no trials).  **Analysis type in protocol according to glossary:** Subgroup analysis.  **How determined the analysis type:** Categories given and says ‘subgroups’.  **Analysis type in review according to glossary:** Not reported.  **How determined the analysis type:** NA.  **Analysed:** No.  **Reasons given (in the review) for not doing interaction analysis if planned:** Not reported (presumably because no trials).  **Reasons given for carrying out interaction analysis if they weren’t planned:** NA.  **Scenario in protocol:** ‘in cases of I 2 > 0 (for the primary outcomes) we will seek to determine the cause of heterogeneity by performing meta-regression analyses and  relevant subgroup and sensitivity analyses.’  **Rationale for planned scenario:** Not reported.  **Scenario in review (if reported specifically):** Not reported.  **Rationale for done scenario:** NA.  **Reason for changing mind:** Not reported (presumably because no trials). | **Method to detect interactions reported in protocol: ‘**We will compare intervention effects in subgroups using a test of  interaction (Altman 2003). We consider P < 0.05 to be indicative  of significant interaction between the flow-regulated valve effect  on one of the co-primary outcomes and the subgroup category  (Higgins 2008, Chapters 9.6.1 and 9.7).’  **Method to detect interactions reported in review:** Not reported.  **Reason for changing mind:** Not reported (presumably because no trials). |
| Ziebell 2013 | **Protocol:** Allocation concealment. We judged the following to be five covariates: ‘Comparing estimates of the pooled intervention effect in trials based on different components of risk of bias (random sequence generation, allocation concealment, blinding, follow up, intention to treat).’  **Review methods:** Not reported.  **Review results:** Not analysed.  **Type:** Methodological.  **Covariate summary:** Allocation concealment. | **Method to choose**  **covariate:** Not reported.  **Where method reported:** NA.  **Rationale for choosing the covariate:** Not reported.  **Where rationale reported:** NA. | **Where reported:** Protocol only.  **Reason not reported in review:** Not reported.  **Reason not reported in protocol:** NA.  **Labelled as ‘posthoc’ if not in protocol:** NA. | **Data type:** Categorical.  **Continuous covariate categorised:** NA.  **How determine it was categorised:** NA.  **Justification for categorising:** NA.  **Where justification reported:** NA.  **Categories reported:** Not reported.  **Where categories reported:** NA.  **Justification for categories:** Not reported.  **Where justification reported:** NA. | **Analysis type reported in protocol:** Sensitivity analysis.  **Analysis type reported in review:** Not reported.  **Reason for changing mind:** Not reported (presumably because no trials).  **Analysis type in protocol according to glossary:** Subgroup analysis.  **How determined the analysis type:** ‘comparing’.  **Analysis type in review according to glossary:** Not reported.  **How determined the analysis type:** NA.  **Analysed:** No.  **Reasons given (in the review) for not doing interaction analysis if planned:** Not reported (presumably because no trials).  **Reasons given for carrying out interaction analysis if they weren’t planned:** NA.  **Scenario in protocol:** ‘in cases of I 2 > 0 (for the primary outcomes) we will seek to determine the cause of heterogeneity by performing meta-regression analyses and  relevant subgroup and sensitivity analyses.’  **Rationale for planned scenario:** Not reported.  **Scenario in review (if reported specifically):** Not reported.  **Rationale for done scenario:** NA.  **Reason for changing mind:** Not reported (presumably because no trials). | **Method to detect interactions reported in protocol:** Not reported.  **Method to detect interactions reported in review:** Not reported.  **Reason for changing mind: NA.** |
| Ziebell 2013 | **Protocol:** Blinding. We judged the following to be five covariates: ‘Comparing estimates of the pooled intervention effect in trials based on different components of risk of bias (random sequence generation, allocation concealment, blinding, follow up, intention to treat).’  **Review methods:** Not reported.  **Review results:** Not analysed.  **Type:** Methodological.  **Covariate summary:** Blinding. | **Method to choose**  **covariate:** Not reported.  **Where method reported:** NA.  **Rationale for choosing the covariate:** Not reported.  **Where rationale reported:** NA. | **Where reported:** Protocol only.  **Reason not reported in review:** Not reported.  **Reason not reported in protocol:** NA.  **Labelled as ‘posthoc’ if not in protocol:** NA. | **Data type:** Categorical.  **Continuous covariate categorised:** NA.  **How determine it was categorised:** NA.  **Justification for categorising:** NA.  **Where justification reported:** NA.  **Categories reported:** Not reported.  **Where categories reported:** NA.  **Justification for categories:** Not reported.  **Where justification reported:** NA. | **Analysis type reported in protocol:** Sensitivity analysis.  **Analysis type reported in review:** Not reported.  **Reason for changing mind:** Not reported (presumably because no trials).  **Analysis type in protocol according to glossary:** Subgroup analysis.  **How determined the analysis type:** ‘comparing’.  **Analysis type in review according to glossary:** Not reported.  **How determined the analysis type:** NA.  **Analysed:** No.  **Reasons given (in the review) for not doing interaction analysis if planned:** Not reported (presumably because no trials).  **Reasons given for carrying out interaction analysis if they weren’t planned:** NA.  **Scenario in protocol:** ‘in cases of I 2 > 0 (for the primary outcomes) we will seek to determine the cause of heterogeneity by performing meta-regression analyses and  relevant subgroup and sensitivity analyses.’  **Rationale for planned scenario:** Not reported.  **Scenario in review (if reported specifically):** Not reported.  **Rationale for done scenario:** NA.  **Reason for changing mind:** Not reported (presumably because no trials). | **Method to detect interactions reported in protocol:** Not reported.  **Method to detect interactions reported in review:** Not reported.  **Reason for changing mind: NA.** |
| Ziebell 2013 | **Protocol:** Excluding the largest trial. We judged the following to be two covariates: ‘Assessment of the benefits and harms of flow-regulated valve compared with differential pressure-regulated valves by conducting sensitivity analyses excluding the smallest or the largest trial.’  **Review methods:** Not reported.  **Review results:** Not analysed.  **Type:** Methodological.  **Covariate summary:** Trial size. | **Method to choose**  **covariate:** Not reported.  **Where method reported:** NA.  **Rationale for choosing the covariate:** Not reported.  **Where rationale reported:** NA. | **Where reported:** Protocol only.  **Reason not reported in review:** Not reported.  **Reason not reported in protocol:** NA.  **Labelled as ‘posthoc’ if not in protocol:** NA. | **Data type:** Continuous.  **Continuous covariate categorised:** Yes.  **How determine it was categorised:** Categories given.  **Justification for categorising:** No reported.  **Where justification reported:** NA.  **Categories reported:** Yes.  **Where categories reported:** Protocol only.  **Justification for categories:** Not reported.  **Where justification reported:** NA. | **Analysis type reported in protocol:** Sensitivity analysis.  **Analysis type reported in review:** Not reported.  **Reason for changing mind:** Not reported (presumably because no trials).  **Analysis type in protocol according to glossary:** Sensitivity analysis.  **How determined the analysis type:** Says ‘excluding’ and ‘Sensitivity analysis’.  **Analysis type in review according to glossary:** Not reported.  **How determined the analysis type:** NA.  **Analysed:** No.  **Reasons given (in the review) for not doing interaction analysis if planned:** Not reported (presumably because no trials).  **Reasons given for carrying out interaction analysis if they weren’t planned:** NA.  **Scenario in protocol:** ‘in cases of I 2 > 0 (for the primary outcomes) we will seek to determine the cause of heterogeneity by performing meta-regression analyses and  relevant subgroup and sensitivity analyses.’  **Rationale for planned scenario:** Not reported.  **Scenario in review (if reported specifically):** Not reported.  **Rationale for done scenario:** NA.  **Reason for changing mind:** Not reported (presumably because no trials). | **Method to detect interactions reported in protocol:** Not reported.  **Method to detect interactions reported in review:** Not reported.  **Reason for changing mind: NA.** |
| Ziebell 2013 | **Protocol:** Excluding the smallest trial. We judged the following to be two covariates: ‘Assessment of the benefits and harms of flow-regulated valve compared with differential pressure-regulated valves by conducting sensitivity analyses excluding the smallest or the largest trial.’  **Review methods:** Not reported.  **Review results:** Not analysed.  **Type:** Methodological.  **Covariate summary:** Trial size. | **Method to choose**  **covariate:** Not reported.  **Where method reported:** NA.  **Rationale for choosing the covariate:** Not reported.  **Where rationale reported:** NA. | **Where reported:** Protocol only.  **Reason not reported in review:** Not reported.  **Reason not reported in protocol:** NA.  **Labelled as ‘posthoc’ if not in protocol:** NA. | **Data type:** Continuous.  **Continuous covariate categorised:** Yes.  **How determine it was categorised:** Categories given.  **Justification for categorising:** No reported.  **Where justification reported:** NA.  **Categories reported:** Yes.  **Where categories reported:** Protocol only.  **Justification for categories:** Not reported.  **Where justification reported:** NA. | **Analysis type reported in protocol:** Sensitivity analysis.  **Analysis type reported in review:** Not reported.  **Reason for changing mind:** Not reported (presumably because no trials).  **Analysis type in protocol according to glossary:** Sensitivity analysis.  **How determined the analysis type:** Says ‘excluding’ and ‘Sensitivity analysis’.  **Analysis type in review according to glossary:** Not reported.  **How determined the analysis type:** NA.  **Analysed:** No.  **Reasons given (in the review) for not doing interaction analysis if planned:** Not reported (presumably because no trials).  **Reasons given for carrying out interaction analysis if they weren’t planned:** NA.  **Scenario in protocol:** ‘in cases of I 2 > 0 (for the primary outcomes) we will seek to determine the cause of heterogeneity by performing meta-regression analyses and  relevant subgroup and sensitivity analyses.’  **Rationale for planned scenario:** Not reported.  **Scenario in review (if reported specifically):** Not reported.  **Rationale for done scenario:** NA.  **Reason for changing mind:** Not reported (presumably because no trials). | **Method to detect interactions reported in protocol:** Not reported.  **Method to detect interactions reported in review:** Not reported.  **Reason for changing mind: NA.** |
| Ziebell 2013 | **Protocol:** Follow up. We judged the following to be five covariates: ‘Comparing estimates of the pooled intervention effect in trials based on different components of risk of bias (random sequence generation, allocation concealment, blinding, follow up, intention to treat).’  **Review methods:** Not reported.  **Review results:** Not analysed.  **Type:** Methodological.  **Covariate summary:** Incomplete outcome data/follow up. | **Method to choose**  **covariate:** Not reported.  **Where method reported:** NA.  **Rationale for choosing the covariate:** Not reported.  **Where rationale reported:** NA. | **Where reported:** Protocol only.  **Reason not reported in review:** Not reported.  **Reason not reported in protocol:** NA.  **Labelled as ‘posthoc’ if not in protocol:** NA. | **Data type:** Categorical.  **Continuous covariate categorised:** NA.  **How determine it was categorised:** NA.  **Justification for categorising:** NA.  **Where justification reported:** NA.  **Categories reported:** Not reported.  **Where categories reported:** NA.  **Justification for categories:** Not reported.  **Where justification reported:** NA. | **Analysis type reported in protocol:** Sensitivity analysis.  **Analysis type reported in review:** Not reported.  **Reason for changing mind:** Not reported (presumably because no trials).  **Analysis type in protocol according to glossary:** Subgroup analysis.  **How determined the analysis type:** ‘comparing’.  **Analysis type in review according to glossary:** Not reported.  **How determined the analysis type:** NA.  **Analysed:** No.  **Reasons given (in the review) for not doing interaction analysis if planned:** Not reported (presumably because no trials).  **Reasons given for carrying out interaction analysis if they weren’t planned:** NA.  **Scenario in protocol:** ‘in cases of I 2 > 0 (for the primary outcomes) we will seek to determine the cause of heterogeneity by performing meta-regression analyses and  relevant subgroup and sensitivity analyses.’  **Rationale for planned scenario:** Not reported.  **Scenario in review (if reported specifically):** Not reported.  **Rationale for done scenario:** NA.  **Reason for changing mind:** Not reported (presumably because no trials). | **Method to detect interactions reported in protocol:** Not reported.  **Method to detect interactions reported in review:** Not reported.  **Reason for changing mind: NA.** |
| Ziebell 2013 | **Protocol:** Intention to treat. We judged the following to be five covariates: ‘Comparing estimates of the pooled intervention effect in trials based on different components of risk of bias (random sequence generation, allocation concealment, blinding, follow up, intention to treat).’  **Review methods:** Not reported.  **Review results:** Not analysed.  **Type:** Methodological.  **Covariate summary:** Intention to treat. | **Method to choose**  **covariate:** Not reported.  **Where method reported:** NA.  **Rationale for choosing the covariate:** Not reported.  **Where rationale reported:** NA. | **Where reported:** Protocol only.  **Reason not reported in review:** Not reported.  **Reason not reported in protocol:** NA.  **Labelled as ‘posthoc’ if not in protocol:** NA. | **Data type:** Categorical.  **Continuous covariate categorised:** NA.  **How determine it was categorised:** NA.  **Justification for categorising:** NA.  **Where justification reported:** NA.  **Categories reported:** Not reported.  **Where categories reported:** NA.  **Justification for categories:** Not reported.  **Where justification reported:** NA. | **Analysis type reported in protocol:** Sensitivity analysis.  **Analysis type reported in review:** Not reported.  **Reason for changing mind:** Not reported (presumably because no trials).  **Analysis type in protocol according to glossary:** Subgroup analysis.  **How determined the analysis type:** ‘comparing’.  **Analysis type in review according to glossary:** Not reported.  **How determined the analysis type:** NA.  **Analysed:** No.  **Reasons given (in the review) for not doing interaction analysis if planned:** Not reported (presumably because no trials).  **Reasons given for carrying out interaction analysis if they weren’t planned:** NA.  **Scenario in protocol:** ‘in cases of I 2 > 0 (for the primary outcomes) we will seek to determine the cause of heterogeneity by performing meta-regression analyses and  relevant subgroup and sensitivity analyses.’  **Rationale for planned scenario:** Not reported.  **Scenario in review (if reported specifically):** Not reported.  **Rationale for done scenario:** NA.  **Reason for changing mind:** Not reported (presumably because no trials). | **Method to detect interactions reported in protocol:** Not reported.  **Method to detect interactions reported in review:** Not reported.  **Reason for changing mind: NA.** |
| Ziebell 2013 | **Protocol:** Random sequence generation. We judged the following to be five covariates: ‘Comparing estimates of the pooled intervention effect in trials based on different components of risk of bias (random sequence generation, allocation concealment, blinding, follow up, intention to treat).’  **Review methods:** Not reported.  **Review results:** Not analysed.  **Type:** Methodological.  **Covariate summary:** Random generation. | **Method to choose**  **covariate:** Not reported.  **Where method reported:** NA.  **Rationale for choosing the covariate:** Not reported.  **Where rationale reported:** NA. | **Where reported:** Protocol only.  **Reason not reported in review:** Not reported.  **Reason not reported in protocol:** NA.  **Labelled as ‘posthoc’ if not in protocol:** NA. | **Data type:** Categorical.  **Continuous covariate categorised:** NA.  **How determine it was categorised:** NA.  **Justification for categorising:** NA.  **Where justification reported:** NA.  **Categories reported:** Not reported.  **Where categories reported:** NA.  **Justification for categories:** Not reported.  **Where justification reported:** NA. | **Analysis type reported in protocol:** Sensitivity analysis.  **Analysis type reported in review:** Not reported.  **Reason for changing mind:** Not reported (presumably because no trials).  **Analysis type in protocol according to glossary:** Subgroup analysis.  **How determined the analysis type:** ‘comparing’.  **Analysis type in review according to glossary:** Not reported.  **How determined the analysis type:** NA.  **Analysed:** No.  **Reasons given (in the review) for not doing interaction analysis if planned:** Not reported (presumably because no trials).  **Reasons given for carrying out interaction analysis if they weren’t planned:** NA.  **Scenario in protocol:** ‘in cases of I 2 > 0 (for the primary outcomes) we will seek to determine the cause of heterogeneity by performing meta-regression analyses and  relevant subgroup and sensitivity analyses.’  **Rationale for planned scenario:** Not reported.  **Scenario in review (if reported specifically):** Not reported.  **Rationale for done scenario:** NA.  **Reason for changing mind:** Not reported (presumably because no trials). | **Method to detect interactions reported in protocol:** Not reported.  **Method to detect interactions reported in review:** Not reported.  **Reason for changing mind: NA.** |

NA: not applicable.

^1^We presumed that methodological, intervention, outcome, and other covariates where study-level covariates and patient covariates were patient-level covariates.

^2^Continuous covariates were considered to be have been categorised when categories were given, or when subgroup or sensitivity analyses were reported to be planned or carried out.

^3^The method according to Glossary 1 was determined based on the covariate categories reported (i.e. one or more), results presented, or the use of words ‘removal’, ‘excluding’ ‘exclusion’, ‘including and excluding’, or ‘removing’, ‘comparing’. When it was reported that a particular type of interaction analysis (e.g. sensitivity analysis) was planned and there was no reason to suspect that a different type (e.g. subgroup analysis), was intended, we judged the method according to the Glossary 1 was the reported method (e.g. sensitivity analysis).

We presumed interaction analysis was carried out for a particular covariate only when results were presented in the review for that covariate or when the review specifically stated they carried out interaction analysis for that covariate for the outcome.
