# Supplementary material for: Exploring Treatment by Covariate Interactions Using Subgroup Analysis and Meta-Regression in Cochrane Reviews: A Review of Recent Practice
Source: PLoS One. 2015 Jun 1;10(6):e0128804. doi: 10.1371/journal.pone.0128804 (PMC4452239; doi:10.1371/journal.pone.0128804)
Supplement: S3 Table — (DOCX) [file pone.0128804.s005.docx]

**Table S3: Analysed covariates in each review.**

| **Review** | **Covariate** | **Outcome** | **Covariate distribution^1^** | **Reporting interaction results^2^** | **Interpreting interaction results^3^** |
| --- | --- | --- | --- | --- | --- |
| Almeida 2013 | **Protocol:** ‘Our main comparisons will be conservative intervention versus no or placebo; one conservative intervention versus another conservative intervention’.  **Review methods:** ‘Our main comparisons were conservative intervention (single or complex intervention) versus placebo intervention; one conservative intervention (single or complex intervention) versus another conservative intervention (single or complex intervention)’.  **Review results:** ‘Exercise therapy versus conventional physiotherapy’; ‘Multi-modal therapy versus exercise therapy’.  **Type:** Intervention.  **Covariate summary:** Type of intervention or control. | Pain relief at rest, on palpation and during physical activity (visual analogue scale, numeric rating scale, and others); primary; continuous; mean difference; NT=1; NP=25. | **Number of trials:** 1.  **Covariate distribution (across trials):**  NT=1, NP=25 (‘Multi-modal therapy versus exercise therapy’);  NT=0, NP=0 (‘Exercise therapy versus conventional physiotherapy’).  **Covariate distribution (within trials):** NA. | **Analysis type in review according to glossary 1:** Subgroup analysis.  **Reported results:** Mean, SD and N by group for trials. Mean difference, CI, p-value for trials.  **Where reported:** Results text and forest plot.  **Method to detect interactions reported in review:** Not reported.  **Reported results from methods to detect interactions:** Not reported.  **Where reported:** NA. | **Reported detected interaction:** Not reported.  **What reported:**  ‘Multi-modal therapy versus exercise therapy:  Primary outcomes:  Pain relief at rest, on palpation and during physical activity:  Weir 2011 also reported the maximum pain during sports (visual analogue score, 0 to 100: worst pain) in the 25 participants who had returned to full sports participation. While favouring exercise therapy, the difference between the two groups for this subgroup was not statistically significant (MD 15.10, 95% CI -7.29 to 37.49, P = 0.19) (*see* Analysis 2.2).’  **Where reported:** Results text.  **Interaction exists based on reported results:** Not reported.  **How determined:** NA.  **Reported importance of the interaction:** Not reported.  **What reported:** Not reported.  **Where reported:** NA  **Reported plausibility of the interaction:** Not reported.  **What reported:** Not reported.  **Where reported:** NA  **Reported confounding possibility:** Not reported.  **What reported:** Not reported.  **Where reported:** NA.  **Mentioned covariate distribution:**  Yes.  **What reported:** as above.  **Where reported:** Results text. |
| Basurto Ona 2013 | **Protocol:** Not reported.  **Review methods:** Not reported.  **Review results: ‘**Buprenorphine versus pethidine’; ‘Morphine versus metamizole’; ‘Pentazocine versus procaine’; ‘Opioids versus no opioids’.  **Type:** Intervention.  **Covariate summary:** Type of intervention or control. | Number of participants showing improvements in pain intensity as defined by the trialist; primary; dichotomous; risk ratio; NT=1; NP=16. | **Number of trials:** 1.  **Covariate distribution (across trials):**  NT=0, NP=0 (Buprenorphine versus pethidine);  NT=1, NP=16 (Morphine versus metamizole);  NT=0, NP=0 (Pentazocine versus procaine);  NT=0, NP=0 (Opioids versus no opioids).  **Covariate distribution (within trials):** NA. | **Analysis type in review according to glossary 1:** Subgroup analysis.  **Reported results:** Event rates, risk ratio, and CI for trials. Risk ratio, CI, z statistic, and p-value for meta-analytic subtotal.  **Where reported:** Results text and forest plot.  **Method to detect interactions reported in review:** Not reported.  **Reported results from methods to detect interactions:** Not reported.  **Where reported:** NA. | **Reported detected interaction:** Not reported.  **What reported:** ‘Number of participants showing improvements in pain intensity as defined by the trialist Only one RCT reported data for this primary outcome (Peiro 2008), showing non-significant differences in the number of participants with subcutaneous morphine compared with intravenously metamizole (RR 0.50, 95% CI 0.19 to 1.33; Analysis 2.1).’  **Where reported:** Results text.  **Interaction exists based on reported results:** Not reported.  **How determined:** NA.  **Reported importance of the interaction:** Not reported.  **What reported:** Not reported.  **Where reported:** NA  **Reported plausibility of the interaction:** Not reported.  **What reported:** Not reported.  **Where reported:** NA  **Reported confounding possibility:** Not reported.  **What reported:** Not reported.  **Where reported:** NA.  **Mentioned covariate distribution:** Yes.  **What reported:** as above.  **Where reported:** Results text. |
| Bellmunt-Montoya 2013 | **Protocol: ‘**Type of comparison assessed, i. e. CHIVA versus drugs, CHIVA versus leg compression, CHIVA versus any surgery (laser therapy, stripping, radiofrequency, etc. ).’  **Review methods:** ‘Type of comparison assessed: CHIVA versus drugs, compression dressings or other techniques.’  **Review results:** ‘Vein stripping versus the CHIVA method’; ‘compression dressing versus the CHIVA method’.  **Type:** Intervention.  **Covariate summary:** Type of intervention or control. | Recurrence of varicose veins; primary; dichotomous risk ratio; NT=4; NP=768. | **Number of trials:** 4.  **Covariate distribution (across trials):**  NT=3, NP=721 (‘vein stripping versus the CHIVA method’);  NT=1, NP=47 (‘compression dressing versus the CHIVA method’).  **Covariate distribution (within trials):** NA. | **Analysis type in review according to glossary 1:** Subgroup analysis.  **Reported results:** Event rates, risk ratio, and CI for trials. Risk ratio, CI, z statistic, and p-value for meta-analytic subtotals. NNTB and CI, or NNTH and CI (for subtotals).  **Where reported:** Results text and forest plot.  **Method to detect interactions reported in review:** Not reported.  **Reported results from methods to detect interactions:** Not reported.  **Where reported:** NA. | **Reported detected interaction:** Not reported.  **What reported:**  Comparison of vein stripping versus the CHIVA Method:  We identified three RCTs that compared vein stripping versus CHIVA (Carandina 2008; Iborra-Ortega 2006; Pares 2010).  Primary outcome:  Recurrence of varicose veins:  These three studies included 721 participants. The pooled result was significant and favored the CHIVA method (RR 0.63; 95% CI 0.51 to 0.78; I2 = 0%; NNTB 6; 95% CI 4 to 10) (Figure 4).  Comparison of compression dressing versus the CHIVA method:  One RCT included only participants with venous ulcers. This study compared compression with the CHIVA method in 47 people (Zamboni 2003). The unit of analysis was the ulcer and not the  individual, but the data were analyzed because, except for one person, all participants had only one ulcer. The result of the outcome “Recurrence of venous ulcer” significantly favored the CHIVA method (RR 0.23; 95% CI 0.06 to 0.96; NNTH 3; 95% CI 2 to 17). “Cure of venous ulcer” showed no significant differences (RR 1.04; 95% CI 0.93 to 1.17).  **Where reported:** Results text.  **Interaction exists based on reported results:** Not reported.  **How determined:** NA.  **Reported importance of the interaction:** Not reported.  **What reported:** Not reported.  **Where reported:** NA  **Reported plausibility of the interaction:** Not reported.  **What reported:** Not reported.  **Where reported:** NA  **Reported confounding possibility:** Not reported.  **What reported:** Not reported.  **Where reported:** NA.  **Mentioned covariate distribution:** Yes.  **What reported:** As above.  **Where reported:** Results text. |
| Boselie 2012 | **Protocol: ‘**Including studies with a high risk of bias.’ **‘**We will perform a sensitivity analysis to assess the influence of the number of risk of bias criteria that are met, including one that looks at the results when studies with a high risk of bias are excluded.’  **Review methods: ‘**Including studies with a high risk of bias.’  **Review results: ‘**Only including studies with an overall low risk of bias.’  **Type:** Methodological.  **Covariate summary:** Risk of bias. | (Arm and neck) pain expressed on a visual analogue or similar scale; primary; continuous; mean difference; NT=6; NP=1,346 (for arm), 1347 (for neck). | **Number of trials:** 3 (arm); 3 (neck).  **Covariate distribution (across trials):**  Arm:  NT=3, NP=1,203 (3months, overall low risk of bias);  NT=3, NP=1,180 (1-2 years, overall low risk of bias).  Neck:  NT=3, NP=1,204 (3months, overall low risk of bias);  NT=3, NP=1,179 **(**1-2 years, overall low risk of bias).  **Covariate distribution (within trials):** NA. | **Analysis type in review according to glossary 1:** Sensitivity analysis.  **Reported results:** Mean, SD and N by group for trials. Mean difference and CI for trials. Mean difference, CI, z-statistic, and p-value for meta-analytic subtotal.  **Where reported:** Results text and forest plot.  **Method to detect interactions reported in review:** Not reported.  **Reported results from methods to detect interactions:** Not reported.  **Where reported:** NA. | **Reported detected interaction:** Not reported.  **What reported:**  Sensitivity analysis 1: only including studies with an overall low risk of bias  There were five studies with an overall low risk of bias (Heller 2009; Kelly 2011; Marzluff 2010; McAfee 2010; Mummaneni 2007). There were four studies with an overall high risk of bias (Coric 2011; Nabhan 2007; Pettine 2010; Porchet 2004).  • Arm pain: significance was lost for results at three months and one to two years on arm pain (MD -2.03; 95% CI -4.66 to 0.60; MD -2.44; 95% CI -5.16 to 0.27, respectively) (Analysis 1.35; Analysis 1.36). Size and direction of the effect were in line with the main analysis. We therefore suspect the possibility of a type II error.  • Neck pain: significance was gained for results at three months, and maintained for results at one to two years on neck pain, both in favour of arthroplasty (MD -5.70; 95% CI -8.41 to -3.00; MD -4.06; 95% CI -6.92 to -1.19, respectively) (Analysis 1.37; Analysis 1.38). Size and direction of the effect were similar to the main analysis. Contrary to the main analysis a fixed-effect model could be used since heterogeneity was lost by excluding the studies with an overall high risk of bias, this was identical to results with the random-effects model.  **Where reported:** Results text.  **Interaction exists based on reported results:** Not reported.  **How determined:**NA.  **Reported importance of the interaction:** Not reported.  **What reported:** Not reported.  **Where reported:** NA  **Reported plausibility of the interaction:** Not reported.  **What reported:** Not reported.  **Where reported:** NA  **Reported confounding possibility:** Not reported.  **What reported:** Not reported.  **Where reported:** NA.  **Mentioned covariate distribution:** Not reported.  **What reported:** Not reported.  **Where reported:** NA. |
| Boselie 2012 | **Protocol: ‘**We will collect all outcome measures at short-term follow-up (up to and including three months) and long-term follow-up (12 months or longer).’ ‘We will attribute different moments of follow-up to different time frames; either “short term” (up to and including three months after surgery), or “long term” (12 months after surgery or longer). If there is more than one follow-up moment within these time frames, the data for the one nearest to the cut-off point (i. e. three months or 12 months) will be extracted.’  **Review methods:** ‘We collected all outcome measures at short-term follow-up (up to and including three months) and medium-term follow-up (one to two years). At the moment of writing the protocol the authors were not aware of reports of studies with a follow-up longer than two years. Some abstracts and papers reporting results on follow-up longer than two years were published since then. To avoid introduction of heterogeneity because of large differences in the duration of follow-up we decided to extract one-year results when available, if these were not available we extracted two-year results. Owing to the very limited amount of studies reporting results after more than two years’ follow-up, these were not included as an additional time point in this review. Results on long-term follow-up (preferably five years or longer) will be included in a future update of this review.’ ‘We therefore attributed different periods of follow-up to different time frames; either ’three months’ (up to and including three months after surgery), or ’one to two years’ (between one and two years after surgery). If there was more than one follow-up period within these time frames, the data for the one nearest to the cutoff point (i. e. three months or one year) was extracted.’  **Review results:** ‘Baseline’; ‘3 months’; ‘1-2 years’.  **Type:** Outcome.  **Covariate summary:** Time point. | (Arm and neck) pain expressed on a visual analogue or similar scale; primary; continuous; mean difference; NT=6; NP=1,346 (for arm), 1347 (for neck). | **Number of trials:** 6 (arm); 6 (neck).  **Covariate distribution (across trials):**  Arm:  NT=6, NP=1,447 (baseline);  NT=6, NP=1,346 (3months);  NT=6, NP=1,310 (1-2 years).  Neck:  NT=6, NP=1,446 (baseline);  NT=6, NP=1,347 (3months);  NT=6, NP=1,309 (1-2 years).  **Covariate distribution (within trials):** NA. | **Analysis type in review according to glossary 1:** Subgroup analysis.  **Reported results:** Mean, SD and N by group for trials. Mean difference and CI for trials. Mean difference, CI, Z statistic, and p-value for meta-analytic subtotal.  **Where reported:** Results text and forest plot.  **Method to detect interactions reported in review:** Not reported.  **Reported results from methods to detect interactions:** Not reported.  **Where reported:** NA. | **Reported detected interaction:** Not reported.  **What reported:**  **Arm pain**  For arm pain, six studies were identified (Heller 2009; Marzluff 2010; Mummaneni 2007; Nabhan 2007; Pettine 2010; Porchet 2004) with a total of 1346 (at three months) and 1310 (at one to two years) participants. All six of these were suitable for pooled analysis, three of which had an overall low risk of bias (Heller 2009; Marzluff 2010; Mummaneni 2007).  There was low-quality evidence (risk of bias, reporting) that there was a significant difference between arthroplasty and fusion at three months and one to two years, in favour of arthroplasty (MD -2.18; 95% CI -3.68 to -0.68; MD -1.54; 95% CI -2.86 to - 0.22, respectively) (Analysis 1.2; Analysis 1.3; Figure 3). Clinical relevance was low, since the pooled difference in effect size was small (< 10% of the scale).  **Neck pain**  For neck pain, six studies were identified (Heller 2009; Marzluff 2010; Mummaneni 2007; Nabhan 2007; Pettine 2010; Porchet 2004) with a total of 1347 (at three months) and 1309 (at one to two years) participants. All six of these were suitable for pooled analysis, three of which had an overall low risk of bias (Heller 2009; Marzluff 2010; Mummaneni 2007).  There was moderate-quality evidence (risk of bias) that there was no significant difference between arthroplasty and fusion at three months (MD -3.67; 95% CI -9.80 to 2.46) (Analysis 1.5). For this analysis a random effects model was used owing to a large amount of heterogeneity (Chi2 = 36.32 at degrees of freedom (df ) = 5, I2 = 86%). This was found to be caused by the extremely small SDs of one study (Nabhan 2007). There was moderatequality evidence (risk of bias) that therewas a significant difference between arthroplasty and fusion at one to two years, in favour of arthroplasty (MD -3.12; 95% CI -4.69 to -1.28) (Analysis 1.6). Clinical relevance was low, since the pooled difference in effect size was small (< 10% of the scale).  **Where reported:** Results text.  **Interaction exists based on reported results:** Not reported.  **How determined:**NA.  **Reported importance of the interaction:** Not reported.  **What reported:** Not reported.  **Where reported:** NA  **Reported plausibility of the interaction:** Not reported.  **What reported:** Not reported.  **Where reported:** NA  **Reported confounding possibility:** Not reported.  **What reported:** Not reported.  **Where reported:** NA.  **Mentioned covariate distribution:** Yes.  **What reported:** as above.  **Where reported:** Results text. |
| Boselie 2012 | **Protocol: ‘**We will explore the impact of including studies with high levels of missing data (15%or greater of the participants lost to follow-up) in the overall assessment of treatment effect by using a sensitivity analysis.’  **Review methods: ‘**We explored the impact of including studies with high levels of missing data (15% or greater of the participants lost to follow-up) in the overall assessment of treatment effect by using a sensitivity analysis.’  **Review results: ‘**Excluding studies with >15% loss to follow-up’.  **Type:** Methodological.  **Covariate summary:** Incomplete outcome data/follow up. | (Arm and neck) pain expressed on a visual analogue or similar scale; primary; continuous; mean difference; NT=6; NP=1,346 (for arm), 1347 (for neck). | **Number of trials:** 4 (arm); 4 (neck).  **Covariate distribution (across trials):**  Arm:  NT=4, NP=1,253 (3months, >15% loss to follow up);  NT=2, NP=696 (1-2 years, >15% loss to follow up).  Neck:  NT=4, NP=1,254 (3months, >15% loss to follow up);  NT=2, NP=695 (1-2 years, >15% loss to follow up).  **Covariate distribution (within trials):** NA. | **Analysis type in review according to glossary 1:** Sensitivity analysis.  **Reported results:** Mean, SD and N by group for trials. Mean difference and CI for trials. Mean difference, CI, z statistic, and p-value for meta-analytic subtotal.  **Where reported:** Results text and forest plot.  **Method to detect interactions reported in review:** Not reported.  **Reported results from methods to detect interactions:** Not reported.  **Where reported:** NA. | **Reported detected interaction:** Not reported.  **What reported:**  Sensitivity analysis 2: excluding studies with > 15% loss to follow-up  There were two studies with > 15% loss to follow-up at three months (Nabhan 2007; Pettine 2010). There were seven studies with < 15% loss to follow-up at three months (Coric 2011; Heller 2009; Kelly 2011; Marzluff 2010; McAfee 2010; Mummaneni 2007; Porchet 2004).  There were four studies with > 15%loss to follow-up at one to two years (Mummaneni 2007; Nabhan 2007; Pettine 2010; Porchet 2004). There were five studies with < 15% loss to follow up at one to two years (Coric 2011; Heller 2009; Kelly 2011; Marzluff 2010; McAfee 2010).  • Arm pain: significance lost for results at three months and one to two years. Direction of the effect was unchanged. Size of the effect was similar for results at three months (MD -2.13; 95% CI -4.73 to 0.46) and slightly larger for results at one to two years (MD -2.86; 95% CI -6.26 to 0.53) when compared to the main analysis (Analysis 1.52; Analysis 1.53). Therefore this is most likely because of a type II error.  • Neck pain: significance gained for results at three months and maintained for results at one to two years. Direction of the effect unchanged. Slightly larger effect size for both time points (MD -5.45; 95% CI -8.11 to -2.80; MD -4.03; 95% CI -7.77 to -0.28, respectively) (Analysis 1.54; Analysis 1.55).  **Where reported:** Results text.  **Interaction exists based on reported results:** Not reported.  **How determined:**NA.  **Reported importance of the interaction:** Not reported.  **What reported:** Not reported.  **Where reported:** NA  **Reported plausibility of the interaction:** Yes.  **What reported:** As above. ‘Therefore this is most likely because of a type II error.’  **Where reported:** Results text.  **Reported confounding possibility:** Not reported.  **What reported:** Not reported.  **Where reported:** NA.  **Mentioned covariate distribution:** Not reported.  **What reported:** Not reported.  **Where reported:** NA. |
| Boselie 2012 | **Protocol:** Not reported.  **Review methods:** ‘Investigated the possibility of a small study effect.’  **Review results:** ‘Small study effect’.  **Type:** Methodological.  **Covariate summary:** Trial size. | (Arm and neck) pain expressed on a visual analogue or similar scale; primary; continuous; mean difference; NT=6; NP=1,346 (for arm), 1347 (for neck). | **Number of trials:** 3 (arm); 3 (neck).  **Covariate distribution (across trials):**  Arm:  NT=3, NP=1,203 (3months, <100 participants);  NT=3, NP=1,180 (1-2 years, <100 participants).  Neck:  NT=3, NP=1,204 (3months, <100 participants);  NT=3, NP=1,179 (1-2 years, <100 participants).  **Covariate distribution (within trials):** NA. | **Analysis type in review according to glossary 1:** Sensitivity analysis.  **Reported results:** Mean, SD and N by group for trials. Mean difference and CI for trials. Mean difference, CI, z-statistic, and p-value for meta-analytic subtotal.  **Where reported:** Results text and forest plot.  **Method to detect interactions reported in review:** Not reported.  **Reported results from methods to detect interactions:** Not reported.  **Where reported:** NA. | **Reported detected interaction:** Not reported.  **What reported:**  Sensitivity analysis 6: investigating possibility of a small study effect  In the absence of a sufficient number of studies for a funnel plot,we excluded the smaller studies (< 100 participants) (Nabhan 2007; Pettine 2010; Porchet 2004).Thiswas performed for the outcomes in which both small and large studies were included.  • Arm pain: No change for baseline comparison. For results at three months and one to two years significance was lost (MD - 2.03; 95% CI -4.66 to 0.60; MD -2.44; 95% CI -5.16 to 0.27, respectively) (Analysis 1.85; Analysis 1.86). Similar size and direction of effect was shown with wider CIs.  • Neck pain: baseline comparison unchanged. Significance gained for results at three months, with slightly larger effect size and narrower CIs (MD -5.70; 95% CI -8.41 to -3.00) than in the original analysis (MD -3.67; 95% CI -9.80 to 2.46) (Analysis 1.87; Analysis 1.88). Results at one to two years were unchanged, with slightly broader CIs.  **Where reported:** Results text.  **Interaction exists based on reported results:** Not reported.  **How determined:**NA.  **Reported importance of the interaction:** Not reported.  **What reported:** Not reported.  **Where reported:** NA  **Reported plausibility of the interaction:** Not reported.  **What reported:** Not reported.  **Where reported:** NA  **Reported confounding possibility:** Not reported.  **What reported:** Not reported.  **Where reported:** NA.  **Mentioned covariate distribution:** Not reported.  **What reported:** Not reported.  **Where reported:** NA. |
| Bruins Slot 2013 | **Protocol:** ‘Baseline stroke risk factors (assessed by the CHADS2 score)’.  **Review methods: ‘**Baseline stroke risk factors (assessed by the CHADS2 score)’.  **Review results:** ‘Baseline CHADS2 score.’ ‘CHADS2-score 0-1’; ‘CHADS2-score 2’; ‘CHADS2-score ≥ 3’).  **Type:** Patient.  **Covariate summary:** Disease characteristics. | The composite endpoint of all strokes (both ischaemic and haemorrhagic) and other systemic embolic events; primary; dichotomous; odds ratio; NT=9; NP=40,777. | **Number of trials:** 3.  **Covariate distribution (across trials):**  NT=1, NP=1,878 **(**CHADS2-score 0-1);  NT=3, NP=3,517 **(**CHADS2-score 2);  NT=3, NP=14,622 **(**CHADS2-score ≥ 3).  **Covariate distribution (within trials):**  Not reported % CHADS2-score 0-1 (1 trials);  Not reported % CHADS2-score 2 (3 trials);  Not reported % CHADS2-score ≥ 3 (3 trials). | **Analysis type in review according to glossary 1:** Subgroup analysis.  **Reported results:** Event rates, OR, and CI for trials. OR, CI, z statistic, and p-value for meta-analytic total and subtotals.  **Where reported:** Forest plot.  **Method to detect interactions reported in review:** Not reported.  **Reported results from methods to detect interactions:** Test for subgroup differences (Chi-square statistic, p-value, and I square statistic).  **Where reported:** Forest plot. | **Reported detected interaction:** Not reported.  **What reported:**  Other pre-specified subgroup analyses  We also performed analyses for the primary efficacy and safety endpoint for the following subgroups: administration route (Analysis 2.1; Analysis 2.2); dose of factor Xa inhibitor (Analysis 3.1; Analysis 3.2); previous stroke (Analysis 4.1; Analysis 4.2); prior VKA treatment-experience (Analysis 6.1; Analysis 6.2); concomitant antiplatelet therapy (aspirin) (Analysis 7.1; Analysis 7.2); age less than 75 years (Analysis 8.1); race (Analysis 9.1; Analysis 9.2); sex (Analysis 10.1; Analysis 10.2); and baseline stroke risk factors (Analysis 11.1; Analysis 11.2). Most of the explored subgroups contained relatively few events in the experimental and control arms and the results of these subgroup analyses should be interpreted with caution.  **Where reported:** Results text.  **Interaction exists based on reported results:** No.  **How determined:** Test for subgroup differences: Chi2= 0.68, df= 2 (P= 0.71), I2=0.0%  **Reported importance of the interaction:** Not reported.  **What reported:** Not reported.  **Where reported:** NA  **Reported plausibility of the interaction:** Not reported.  **What reported:** Not reported.  **Where reported:** NA  **Reported confounding possibility:** Not reported.  **What reported:** Not reported.  **Where reported:** NA.  **Mentioned covariate distribution:** Not reported.  **What reported:** Not reported.  **Where reported:** NA. |
| Bruins Slot 2013 | **Protocol:** ‘Patients aged less than 75 years versus patients aged 75 years or over’.  **Review methods:** ‘Age less than 75 years versus age 75 years or over’.  **Review results:** ‘Age’. ‘Age < 75 years’; ‘Age ≥ 75 years’.  **Type:** Patient.  **Covariate summary:** Demographics. | The composite endpoint of all strokes (both ischaemic and haemorrhagic) and other systemic embolic events; primary; dichotomous; odds ratio; NT=9; NP=40,777. | **Number of trials:** 2.  **Covariate distribution (across trials):**  NT=2, NP=10,972 (Age < 75 years);  NT=2, NP=7,775 (Age ≥ 75 years).  **Covariate distribution (within trials):**  Not reported % Age < 75 years (2 trials);  Not reported % Age ≥ 75 years (2 trials). | **Analysis type in review according to glossary 1:** Subgroup analysis.  **Reported results:** Event rates, OR, and CI for trials. OR, CI, z statistic, and p-value for meta-analytic total and subtotals.  **Where reported:** Forest plot.  **Method to detect interactions reported in review:** Not reported.  **Reported results from methods to detect interactions:** Test for subgroup differences (Chi-square statistic, p-value, and I square statistic).  **Where reported:** Forest plot. | **Reported detected interaction:** Not reported.  **What reported:**  Other pre-specified subgroup analyses  We also performed analyses for the primary efficacy and safety endpoint for the following subgroups: administration route (Analysis 2.1; Analysis 2.2); dose of factor Xa inhibitor (Analysis 3.1; Analysis 3.2); previous stroke (Analysis 4.1; Analysis 4.2); prior VKA treatment-experience (Analysis 6.1; Analysis 6.2); concomitant antiplatelet therapy (aspirin) (Analysis 7.1; Analysis 7.2); age less than 75 years (Analysis 8.1); race (Analysis 9.1; Analysis 9.2); sex (Analysis 10.1; Analysis 10.2); and baseline stroke risk factors (Analysis 11.1; Analysis 11.2). Most of the explored subgroups contained relatively few events in the experimental and control arms and the results of these subgroup analyses should be interpreted with caution.  **Where reported:** Results text.  **Interaction exists based on reported results:** No.  **How determined:** Test for subgroup differences: Chi2= 1.43, df= 1 (P= 0.23), I2=30%  **Reported importance of the interaction:** Not reported.  **What reported:** Not reported.  **Where reported:** NA  **Reported plausibility of the interaction:** Not reported.  **What reported:** Not reported.  **Where reported:** NA  **Reported confounding possibility:** Not reported.  **What reported:** Not reported.  **Where reported:** NA.  **Mentioned covariate distribution:** Not reported.  **What reported:** Not reported.  **Where reported:** NA. |
| Bruins Slot 2013 | **Protocol: ‘**Patients who have had a previous stroke versus those who have not’.  **Review methods: ‘**Previous stroke versus no previous stroke.’  **Review results:** ‘Previous stroke or TIA’. ‘Previous stroke or TIA’; ‘No previous stroke or TIA’.  **Type:** Patient.  **Covariate summary:** Disease characteristics. | The composite endpoint of all strokes (both ischaemic and haemorrhagic) and other systemic embolic events; primary; dichotomous; odds ratio; NT=9; NP=40,777. | **Number of trials:** 3.  **Covariate distribution (across trials):**  NT=3, NP=5,340 (Previous stroke or TIA);  NT=3, NP=18,710 (No previous stroke or TIA).  **Covariate distribution (within trials):**  Not reported % previous stroke or TIA (3 trials);  Not reported % no previous stroke or TIA (3 trials). | **Analysis type in review according to glossary 1:** Subgroup analysis.  **Reported results:** Event rates, OR, and CI for trials. OR, CI, z statistic, and p-value for meta-analytic total and subtotals.  **Where reported:** Forest plot.  **Method to detect interactions reported in review:** Not reported.  **Reported results from methods to detect interactions:** Test for subgroup differences (Chi-square statistic, p-value, and I square statistic).  **Where reported:** Forest plot. | **Reported detected interaction:** Not reported.  **What reported:**  Other pre-specified subgroup analyses  We also performed analyses for the primary efficacy and safety endpoint for the following subgroups: administration route (Analysis 2.1; Analysis 2.2); dose of factor Xa inhibitor (Analysis 3.1; Analysis 3.2); previous stroke (Analysis 4.1; Analysis 4.2); prior VKA treatment-experience (Analysis 6.1; Analysis 6.2); concomitant antiplatelet therapy (aspirin) (Analysis 7.1; Analysis 7.2); age less than 75 years (Analysis 8.1); race (Analysis 9.1; Analysis 9.2); sex (Analysis 10.1; Analysis 10.2); and baseline stroke risk factors (Analysis 11.1; Analysis 11.2). Most of the explored subgroups contained relatively few events in the experimental and control arms and the results of these subgroup analyses should be interpreted with caution.  **Where reported:** Results text.  **Interaction exists based on reported results:** No.  **How determined:** Test for subgroup differences: Chi2= 0.76, df= 1 (P= 0.38), I2=0.0%  **Reported importance of the interaction:** Not reported.  **What reported:** Not reported.  **Where reported:** NA  **Reported plausibility of the interaction:** Not reported.  **What reported:** Not reported.  **Where reported:** NA  **Reported confounding possibility:** Not reported.  **What reported:** Not reported.  **Where reported:** NA.  **Mentioned covariate distribution:** Not reported.  **What reported:** Not reported.  **Where reported:** NA. |
| Bruins Slot 2013 | **Protocol:** ‘Patients who have received vitamin K antagonist treatment with a sufficient and clinically relevant time-in-therapeutic range (i. e. equal to or greater than 60% of INR measurements within the therapeutic range 2. 0 to 3. 0 versus less than 60%) (Connolly 2008; ESC 2010).’  **Review methods:** ‘Participants who received VKA treatment with time-in-therapeutic range (TTR) equal to or greater than 60% (’good quality’) versus less than 60% (’poor quality’) (Connolly 2008; ESC 2010)’.  **Review results:** ‘Quality of anticoagulation with VKA (TTR) (Good quality, Bad quality)’.  **Type:** Intervention.  **Covariate summary:** Time to therapeutic range (good/bad quality). | The composite endpoint of all strokes (both ischaemic and haemorrhagic) and other systemic embolic events; primary; dichotomous; odds ratio; NT=9; NP=40,777. | **Number of trials:** 1.  **Covariate distribution (across trials):**  NT=1, NP=6,977 (Good quality);  NT=1, NP=6,994 (Bad quality).  **Covariate distribution (within trials):** NA. | **Analysis type in review according to glossary 1:** Subgroup analysis.  **Reported results:** Event rates, OR, and CI for trials. OR, CI, z statistic, and p-value for meta-analytic total and subtotals.  **Where reported:** Results text and forest plot.  **Method to detect interactions reported in review:** Not reported.  **Reported results from methods to detect interactions:** Test for subgroup differences (Chi-square statistic, p-value, and I square statistic).  **Where reported:** Forest plot. | **Reported detected interaction:** Not reported.  **What reported:**  Quality of anticoagulation with warfarin  We intended to perform a subgroup analysis in participants who received VKA treatment with time-in-therapeutic range (TTR) equal to or greater than 60% versus less than 60%. Unfortunately, we had only sufficient raw data fromROCKET AF 2011 to perform this subgroup analysis for the primary efficacy endpoint (Analysis 5.1). The number of strokes and systemic embolic events in participants treated at centres with ’good quality’ warfarin administration (centre TTR > 58.5%) was lower in participants that were treated with rivaroxaban compared with warfarin, though the difference did not reach statistical significance (OR 0.78, 95% CI 0.60 to 1.02). The number of strokes and systemic embolic events in centres with ’poor quality’ warfarin administration (centre TTR < 58.5%) was also lower in participants treated with rivaroxaban, though again a statistically significant difference was not observed (OR 0.81, 95% CI 0.62 to 1.07).  Data presented in the publication of the final results of the J-ROCKET AF 2012 trial also indicated that there was a nonsignificant decrease in the number of strokes and systemic embolic events in participants treated with rivaroxaban regardless of the quality of warfarin administration assessed by centre TTR. Data from the ARISTOTLE 2011 trial also indicated a non-significant decrease in the number of strokes and systemic embolic events in participants treated with apixaban regardless of the quality of warfarin administration by centre TTR (Wallentin 2011). These findings might indicate that, at least for apixaban and rivaroxaban, the benefits of preventing stroke and other systemic embolic events compared with warfarin are more or less consistent regardless of the quality of warfarin administration. Still, local standards of care might well affect the benefits of treatment with factor Xa inhibitors, as was observed with the direct thrombin inhibitor dabigatranwhen studied for a similar indication (Wallentin 2010). This important issue clearly merits further investigation.  **Where reported:** Results text.  **Interaction exists based on reported results:** No.  **How determined:**  Test for subgroup differences: Chi2= 0.04, df= 1 (P= 0.84), I2=0.0%.  **Reported importance of the interaction:** Not reported.  **What reported:** Not reported.  **Where reported:** NA  **Reported plausibility of the interaction:** Not reported.  **What reported:** Not reported.  **Where reported:** NA  **Reported confounding possibility:** Not reported.  **What reported:** Not reported.  **Where reported:** NA.  **Mentioned covariate distribution:** Not reported.  **What reported:** Not reported.  **Where reported:** NA. |
| Bruins Slot 2013 | **Protocol:** ‘Patients who received concomitant antiplatelet therapy (i. e. aspirin) versus those who did not.’  **Review methods: ‘**Patients who received concomitant antiplatelet therapy (i. e. aspirin) versus those who did not’.  **Review results:** ‘Concomitant antiplatelet use’. ‘Concomitant antiplatelet use’; ‘No concomitant antiplatelet use’.  **Type:** Intervention.  **Covariate summary:** Additional interventions. | The composite endpoint of all strokes (both ischaemic and haemorrhagic) and other systemic embolic events; primary; dichotomous; odds ratio; NT=9; NP=40,777. | **Number of trials:** 2.  **Covariate distribution (across trials):**  NT=2, NP=5,647 (Concomitant antiplatelet use);  NT=2, NP=7,926 (No concomitant antiplatelet use).    **Covariate distribution (within trials):** NA. | **Analysis type in review according to glossary 1:** Subgroup analysis.  **Reported results:** Event rates, OR, and CI for trials. OR, CI, z statistic, and p-value for meta-analytic total and subtotals.  **Where reported:** Forest plot.  **Method to detect interactions reported in review:** Not reported.  **Reported results from methods to detect interactions:** Test for subgroup differences (Chi-square statistic, p-value, and I square statistic).  **Where reported:** Forest plot. | **Reported detected interaction:** Not reported.  **What reported:**  Other pre-specified subgroup analyses  We also performed analyses for the primary efficacy and safety endpoint for the following subgroups: administration route (Analysis 2.1; Analysis 2.2); dose of factor Xa inhibitor (Analysis 3.1; Analysis 3.2); previous stroke (Analysis 4.1; Analysis 4.2); prior VKA treatment-experience (Analysis 6.1; Analysis 6.2); concomitant antiplatelet therapy (aspirin) (Analysis 7.1; Analysis 7.2); age less than 75 years (Analysis 8.1); race (Analysis 9.1; Analysis 9.2); sex (Analysis 10.1; Analysis 10.2); and baseline stroke risk factors (Analysis 11.1; Analysis 11.2). Most of the explored subgroups contained relatively few events in the experimental and control arms and the results of these subgroup analyses should be interpreted with caution.  **Where reported:** Results text.  **Interaction exists based on reported results:** Yes.  **How determined:** Test for subgroup differences: Chi2= 12.07, df= 1 (P= 0.00), I2=92%  **Reported importance of the interaction:** Not reported.  **What reported:** Not reported.  **Where reported:** NA  **Reported plausibility of the interaction:** Not reported.  **What reported:** Not reported.  **Where reported:** NA  **Reported confounding possibility:** Not reported.  **What reported:** Not reported.  **Where reported:** NA.  **Mentioned covariate distribution:** Not reported.  **What reported:** Not reported.  **Where reported:** NA. |
| Bruins Slot 2013 | **Protocol:** ‘Race’.  **Review methods: ‘**Race’.  **Review results: ‘**Race’. ‘Asian patients’; ‘White patients’; ‘Black patients’; ‘Other races’.  **Type:** Patient.  **Covariate summary:** Demographics. | The composite endpoint of all strokes (both ischaemic and haemorrhagic) and other systemic embolic events; primary; dichotomous; odds ratio; NT=9; NP=40,777. | **Number of trials:** 5.  **Covariate distribution (across trials):**  NT=5, NP=3,538 (Asian patients);  NT=2, NP=16,271 (White patients);  NT=2, NP=208 (Black patients);  NT=2, NP=465 (Other races)  **Covariate distribution (within trials):**  100% Asian patients (3 trials);  Not reported % Asian patients (2 trials);  Not reported % white patients (2 trials);  Not reported % black patients (2 trials);  Not reported % other races (2 trials). | **Analysis type in review according to glossary 1:** Subgroup analysis.  **Reported results:** Event rates, OR, and CI for trials. OR, CI, z statistic, and p-value for meta-analytic total and subtotals.  **Where reported:** Forest plot.  **Method to detect interactions reported in review:** Not reported.  **Reported results from methods to detect interactions:** Test for subgroup differences (Chi-square statistic, p-value, and I square statistic).  **Where reported:** Forest plot. | **Reported detected interaction:** Not reported.  **What reported:**  Other pre-specified subgroup analyses  We also performed analyses for the primary efficacy and safety endpoint for the following subgroups: administration route (Analysis 2.1; Analysis 2.2); dose of factor Xa inhibitor (Analysis 3.1; Analysis 3.2); previous stroke (Analysis 4.1; Analysis 4.2); prior VKA treatment-experience (Analysis 6.1; Analysis 6.2); concomitant  antiplatelet therapy (aspirin) (Analysis 7.1; Analysis 7.2); age less than 75 years (Analysis 8.1); race (Analysis 9.1; Analysis 9.2); sex (Analysis 10.1; Analysis 10.2); and baseline stroke risk factors (Analysis 11.1; Analysis 11.2). Most of the explored subgroups contained relatively few events in the experimental and control arms and the results of these subgroup analyses should be interpreted with caution.  **Where reported:** Results text.  **Interaction exists based on reported results:** No.  **How determined:** Test for subgroup differences: Chi2= 2.82, df= 3 (P= 0.42), I2=0.0%  **Reported importance of the interaction:** Not reported.  **What reported:** Not reported.  **Where reported:** NA  **Reported plausibility of the interaction:** Not reported.  **What reported:** Not reported.  **Where reported:** NA  **Reported confounding possibility:** Not reported.  **What reported:** Not reported.  **Where reported:** NA.  **Mentioned covariate distribution:** Not reported.  **What reported:** Not reported.  **Where reported:** NA. |
| Bruins Slot 2013 | **Protocol:** ‘Sex’.  **Review methods: ‘**Sex’.  **Review results:** ‘Sex. ‘Female’; ‘Male’.  **Type:** Patient.  **Covariate summary:** Demographics. | The composite endpoint of all strokes (both ischaemic and haemorrhagic) and other systemic embolic events; primary; dichotomous; odds ratio; NT=9; NP=40,777. | **Number of trials:** 3.  **Covariate distribution (across trials):**  NT=3, NP=7,386 (Female);  NT=3, NP=12,634 (Male).  **Covariate distribution (within trials):**  Not reported % Female (3 trials);  Not reported % Male (3 trials). | **Analysis type in review according to glossary 1:** Subgroup analysis.  **Reported results:** Event rates, OR, and CI for trials. OR, CI, z statistic, and p-value for meta-analytic total and subtotals.  **Where reported:** Forest plot.  **Method to detect interactions reported in review:** Not reported.  **Reported results from methods to detect interactions:** Test for subgroup differences (Chi-square statistic, p-value, and I square statistic).  **Where reported:** Forest plot. | **Reported detected interaction:** Not reported.  **What reported:**  Other pre-specified subgroup analyses  We also performed analyses for the primary efficacy and safety endpoint for the following subgroups: administration route (Analysis 2.1; Analysis 2.2); dose of factor Xa inhibitor (Analysis 3.1; Analysis 3.2); previous stroke (Analysis 4.1; Analysis 4.2); prior VKA treatment-experience (Analysis 6.1; Analysis 6.2); concomitant antiplatelet therapy (aspirin) (Analysis 7.1; Analysis 7.2); age less than 75 years (Analysis 8.1); race (Analysis 9.1; Analysis 9.2); sex (Analysis 10.1; Analysis 10.2); and baseline stroke risk factors (Analysis 11.1; Analysis 11.2). Most of the explored subgroups contained relatively few events in the experimental and control arms and the results of these subgroup analyses should be interpreted with caution.  **Where reported:** Results text.  **Interaction exists based on reported results:** No.  **How determined:** Test for subgroup differences: Chi2= 0.11, df= 1 (P= 0.74), I2=0.0%  **Reported importance of the interaction:** Not reported.  **What reported:** Not reported.  **Where reported:** NA  **Reported plausibility of the interaction:** Not reported.  **What reported:** Not reported.  **Where reported:** NA  **Reported confounding possibility:** Not reported.  **What reported:** Not reported.  **Where reported:** NA.  **Mentioned covariate distribution:** Not reported.  **What reported:** Not reported.  **Where reported:** NA. |
| Bruins Slot 2013 | **Protocol: ‘**Vitamin K antagonist treatment-experienced patients versus treatment-naïve patients’.  **Review methods: ‘**Vitamin K antagonist treatment-experienced patients versus treatment-naïve patients’.  **Review results:** ‘Previous VKA use’. ‘VKA naive’; ‘VKA experienced’.  **Type:** Patient.  **Covariate summary:** Disease characteristics. | The composite endpoint of all strokes (both ischaemic and haemorrhagic) and other systemic embolic events; primary; dichotomous; odds ratio; NT=9; NP=40,777. | **Number of trials:** 3.  **Covariate distribution (across trials):**  NT=3, NP=6,545 (VKA naive);  NT=3, NP=13,476 (VKA experienced).  **Covariate distribution (within trials):**  Not reported % VKA naive (3 trials);  Not reported % VKA experienced (3 trials). | **Analysis type in review according to glossary 1:** Subgroup analysis.  **Reported results:** Event rates, OR, and CI for trials. OR, CI, z statistic, and p-value for meta-analytic total and subtotals.  **Where reported:** Forest plot.  **Method to detect interactions reported in review:** Not reported.  **Reported results from methods to detect interactions:** Test for subgroup differences (Chi-square statistic, p-value, and I square statistic).  **Where reported:** Forest plot. | **Reported detected interaction:** Not reported.  **What reported:**  Other pre-specified subgroup analyses  We also performed analyses for the primary efficacy and safety endpoint for the following subgroups: administration route (Analysis 2.1; Analysis 2.2); dose of factor Xa inhibitor (Analysis 3.1; Analysis 3.2); previous stroke (Analysis 4.1; Analysis 4.2); prior VKA treatment-experience (Analysis 6.1; Analysis 6.2); concomitant antiplatelet therapy (aspirin) (Analysis 7.1; Analysis 7.2); age less than 75 years (Analysis 8.1); race (Analysis 9.1; Analysis 9.2); sex (Analysis 10.1; Analysis 10.2); and baseline stroke risk factors (Analysis 11.1; Analysis 11.2). Most of the explored subgroups contained relatively few events in the experimental and control arms and the results of these subgroup analyses should be interpreted with caution.  **Where reported:** Results text.  **Interaction exists based on reported results:** No.  **How determined:** Test for subgroup differences: Chi2= 2.49, df= 1 (P= 0.11), I2=60%  **Reported importance of the interaction:** Not reported.  **What reported:** Not reported.  **Where reported:** NA  **Reported plausibility of the interaction:** Not reported.  **What reported:** Not reported.  **Where reported:** NA  **Reported confounding possibility:** Not reported.  **What reported:** Not reported.  **Where reported:** NA.  **Mentioned covariate distribution:** Not reported.  **What reported:** Not reported.  **Where reported:** NA. |
| Bruins Slot 2013 | **Protocol:** Administration route of factor Xa inhibitor. We judged the following to be two covariates: ‘Where possible, we will do subgroup analyses for: administration route and dose of factor Xa inhibitor’.  **Review methods:** Administration route of factor Xa inhibitor. We judged the following to be two covariates: ‘Where possible, we performed subgroup analyses for: administration route and dose of factor Xa inhibitor’.  **Review results:** ‘Route of administration’. ‘Oral administration’; ‘Parenteral administration’.  **Type:** Intervention.  **Covariate summary:** Route of administration. | The composite endpoint of all strokes (both ischaemic and haemorrhagic) and other systemic embolic events; primary; dichotomous; odds ratio; NT=9; NP=40,777. | **Number of trials:** 9.  **Covariate distribution (across trials):**  NT=8, NP=36,201 (Oral administration);  NT=1, NP=4,576 (Parenteral administration).  **Covariate distribution (within trials):** NA. | **Analysis type in review according to glossary 1:** Subgroup analysis.  **Reported results:** Event rates, OR, and CI for trials. OR, CI, z statistic, and p-value for meta-analytic total and subtotals.  **Where reported:** Forest plot.  **Method to detect interactions reported in review:** Not reported.  **Reported results from methods to detect interactions:** Test for subgroup differences (Chi-square statistic, p-value, and I square statistic).  **Where reported:** Forest plot. | **Reported detected interaction:** Not reported.  **What reported:**  Other pre-specified subgroup analyses  We also performed analyses for the primary efficacy and safety endpoint for the following subgroups: administration route (Analysis 2.1; Analysis 2.2); dose of factor Xa inhibitor (Analysis 3.1; Analysis 3.2); previous stroke (Analysis 4.1; Analysis 4.2); prior VKA treatment-experience (Analysis 6.1; Analysis 6.2); concomitant antiplatelet therapy (aspirin) (Analysis 7.1; Analysis 7.2); age less than 75 years (Analysis 8.1); race (Analysis 9.1; Analysis 9.2); sex (Analysis 10.1; Analysis 10.2); and baseline stroke risk factors (Analysis 11.1; Analysis 11.2). Most of the explored subgroups contained relatively few events in the experimental and control arms and the results of these subgroup analyses should be interpreted with caution.  **Where reported:** Results text.  **Interaction exists based on reported results:** No.  **How determined:** Test for subgroup differences: Chi2= 0.42, df= 1 (P= 0.52), I2=0.0%  **Reported importance of the interaction:** Not reported.  **What reported:** Not reported.  **Where reported:** NA  **Reported plausibility of the interaction:** Not reported.  **What reported:** Not reported.  **Where reported:** NA  **Reported confounding possibility:** Not reported.  **What reported:** Not reported.  **Where reported:** NA.  **Mentioned covariate distribution:** Not reported.  **What reported:** Not reported.  **Where reported:** NA. |
| Bruins Slot 2013 | **Protocol:** Dose of factor Xa inhibitor. We judged the following to be two covariates: ‘Where possible, we will do subgroup analyses for: administration route and dose of factor Xa inhibitor’.  **Review methods:** Dose of factor Xa inhibitor. We judged the following to be two covariates: ‘Where possible, we performed subgroup analyses for: administration route and dose of factor Xa inhibitor’.  **Review results:** ‘Dose of Factor Xa inhibitor’. ‘Apixaban 2. 5mg twice daily’; ‘Apixaban 5 mg twice daily’; ‘Edoxaban 30mg once daily’; ‘Edoxaban 60mg once daily’; ‘Edoxaban 30mg twice daily’; ‘Edoxaban 60mg twice daily’; ‘Rivaroxaban 10mg once daily’; ‘Rivaroxaban 15mg once daily’; ‘Darexaban 30mg once daily’; ‘Darexaban 60mg once daily’; ‘Darexaban 120mg once daily’; ‘Darexaban 240mg’; ‘Idraparinux 1,5mg once weekly’; ‘Idraparinux 2,5mg once weekly’; ‘Betrixaban 40 mg’; ‘Betrixaban 60 mg’; ‘Betrixaban 80 mg’.  **Type:** Intervention.  **Covariate summary:** Dose. | The composite endpoint of all strokes (both ischaemic and haemorrhagic) and other systemic embolic events; primary; dichotomous; odds ratio; NT=9; NP=40,777. | **Number of trials:** 7.  **Covariate distribution (across trials):**  NT=1, NP=148 (Apixaban 2.5mg twice daily);  NT=1, NP=148 (Apixaban 5 mg twice daily );  NT=2, NP=639 (Edoxaban 30mg once daily);  NT=2, NP=639 (Edoxaban 60mg once daily);  NT=1, NP=494 (Edoxaban 30mg twice daily);  NT=1, NP=430 (Edoxaban 60mg twice daily);  NT=1, NP=781 (Rivaroxaban 10mg once daily);  NT=1, NP=1,136 (Rivaroxaban 15mg once daily );  NT=1, NP=184 (Darexaban 30mg once daily);  NT=1, NP=187 (Darexaban 60mg once daily);  NT=1, NP=187 (Darexaban 120mg once daily);  NT=1, NP=172 (Darexaban 240mg);  NT=1, NP=68 ( Idraparinux 1,5mg once weekly);  NT=1, NP=4508 (Idraparinux 2,5mg once weekly);  NT=1, NP=254 (Betrixaban 40 mg);  NT=1, NP=254 (Betrixaban 60 mg);  NT=1, NP=254 (Betrixaban 80 mg).  **Covariate distribution (within trials):** NA. | **Analysis type in review according to glossary 1:** Subgroup analysis.  **Reported results:** Event rates, OR, and CI for trials. OR, CI, z statistic, and p-value for meta-analytic total and subtotals.  **Where reported:** Forest plot.  **Method to detect interactions reported in review:** Not reported.  **Reported results from methods to detect interactions:** Test for subgroup differences (Chi-square statistic, p-value, and I square statistic).  **Where reported:** Forest plot. | **Reported detected interaction:** Not reported.  **What reported:**  Other pre-specified subgroup analyses  We also performed analyses for the primary efficacy and safety endpoint for the following subgroups: administration route (Analysis 2.1; Analysis 2.2); dose of factor Xa inhibitor (Analysis 3.1; Analysis 3.2); previous stroke (Analysis 4.1; Analysis 4.2); prior VKA treatment-experience (Analysis 6.1; Analysis 6.2); concomitant antiplatelet therapy (aspirin) (Analysis 7.1; Analysis 7.2); age less than 75 years (Analysis 8.1); race (Analysis 9.1; Analysis 9.2); sex (Analysis 10.1; Analysis 10.2); and baseline stroke risk factors (Analysis 11.1; Analysis 11.2). Most of the explored subgroups contained relatively few events in the experimental and control arms and the results of these subgroup analyses should be interpreted with caution.  **Where reported:** Results text.  **Interaction exists based on reported results:** No.  **How determined:** Test for subgroup differences: Chi2= 9.28, df= 12 (P= 0.68), I2=0.0%  **Reported importance of the interaction:** Not reported.  **What reported:** Not reported.  **Where reported:** NA  **Reported plausibility of the interaction:** Not reported.  **What reported:** Not reported.  **Where reported:** NA  **Reported confounding possibility:** Not reported.  **What reported:** Not reported.  **Where reported:** NA.  **Mentioned covariate distribution:** Not reported.  **What reported:** Not reported.  **Where reported:** NA. |
| Bruins Slot 2013 | **Protocol:** Not reported.  **Review methods:** Not reported.  **Review results:** Different factor Xa inhibitors. ‘Apixaban versus VKA’; ‘Darexaban versus VKA’; ‘Edoxaban versus VKA’; ‘Idraparinux versus VKA’; ‘Rivaroxaban versus VKA’; ‘Betrixaban versus VKA’).  **Type:** Intervention.  **Covariate summary:** Type of intervention or control. | The composite endpoint of all strokes (both ischaemic and haemorrhagic) and other systemic embolic events; primary; dichotomous; odds ratio; NT=9; NP=40,777. | **Number of trials:** 9.  **Covariate distribution (across trials):**  NT=2, NP=18,423 (Apixaban versus VKA);  NT=1, NP=448 (Darexaban versus VKA);  NT=2, NP=1,377 (Edoxaban versus VKA);  NT=1, NP=4,576 (Idraparinux versus VKA);  NT=2, NP=15,445 (Rivaroxaban versus VKA);  NT=1, NP=508 (Betrixaban versus VKA.  **Covariate distribution (within trials):** NA. | **Analysis type in review according to glossary 1:** Subgroup analysis.  **Reported results:** Event rates, OR, and CI for trials. OR, CI, z statistic, and p-value for meta-analytic total and subtotals.  **Where reported:** Results text and forest plot.  **Method to detect interactions reported in review:** Not reported.  **Reported results from methods to detect interactions:** Test for subgroup differences (Chi-square statistic, p-value, and I square statistic).  **Where reported:** Forest plot. | **Reported detected interaction:** Not reported.  **What reported:**  The composite endpoint of all strokes (both ischaemic and haemorrhagic) and other systemic embolic events was reported in nine of the included studies (n = 40,777). Most data (approximately 90%) were available from studies that used the agents apixaban (ARISTOTLE 2011; ARISTOTLE-J 2011) and rivaroxaban (ROCKET AF 2011; J-ROCKET AF 2012). No data were available for one of the trials that studied darexaban (OPAL-2 2011). Treatment with a factor Xa inhibitor significantly decreased the number of strokes and other systemic embolic events compared with dose-adjusted warfarin in participants with AF (Analysis 1.1: OR 0.81, 95% CI 0.72 to 0.91). We observed no statistically significant heterogeneity (I² = 0%). Of note, the total number of non-central nervous system (CNS) systemic embolic events was very low (n = 66), contributing to approximately 5% of all outcomes of the composite endpoint. The primary outcome was thus mainly driven by the stroke component.  We also calculated the number needed to treat (NNT) for studies with follow-up periods of one year or more (ARISTOTLE 2011; ROCKET AF 2011; J-ROCKET AF 2012). The NNT for apixaban (ARISTOTLE 2011) was 304 per year (or 169 for a total treatment period of 1.8 years), indicating that 304 people needed to be treated with apixaban for one year to prevent onemore stroke or systemic embolic embolism compared with dose-adjusted warfarin. TheNNTs for rivaroxaban were 369 per year (194 for a total treatment period of 1.9 years) based on data from ROCKET AF 2011, and 81 per year (58 for a total treatment period of 1.4 years) based on data from the smaller J-ROCKET AF 2012 trial.  Different factor Xa inhibitors  A subgroup analysis of the different factor Xa inhibitors showed that only the agents apixaban (OR 0.78, 95% CI 0.65 to 0.93) and rivaroxaban (OR 0.85, 95%CI 0.72 to 1.00) significantly decreased the number of strokes and systemic embolic events compared with warfarin (Analysis 1.1). The agents idraparinux, edoxaban, darexaban and betrixaban did not show a statistically significant difference in the number of strokes and systemic embolic events compared with warfarin, but there was no evidence of heterogeneity between the risk estimates of these agents and those of apixaban or rivaroxaban (Analysis 1.1).  **Where reported:** Results text.  **Interaction exists based on reported results:** No.  **How determined:** Test for subgroup differences: Chi2= 2.09, df= 5 (P= 0.84), I2=0.0%  **Reported importance of the interaction:** Not reported.  **What reported:** Not reported.  **Where reported:** NA  **Reported plausibility of the interaction:** Not reported.  **What reported:** Not reported.  **Where reported:** NA  **Reported confounding possibility:** Not reported.  **What reported:** Not reported.  **Where reported:** NA.  **Mentioned covariate distribution:** Not reported.  **What reported:** Not reported.  **Where reported:** NA. |
| Chaparro 2013 | **Protocol:** ‘Surgical procedures.’  **Review methods: ‘**Surgical procedures’.  **Review results: ‘**Abdominal and/or pelvic surgery’; ‘Amputation’; ‘Breast Surgery’; ‘Hemorrhoidectomy’; ‘Orthopedic Surgery’; ‘Thoracotomy’; ‘Caesarean section’; ‘Cardiac Surgery’; ‘Total hip arthroplasty’; ‘Total Knee Arthroplasty’; ‘Spine Surgery’; ‘Thyroidectomy’).  **Type:** Intervention.  **Covariate summary:** Type of intervention or control. | Proportion of participants reporting any pain at the anatomical site of the procedure or pain referred to the surgical site, or both (for example phantom limb pain, shoulder pain referred from the diaphragm etc.), three months or more after the procedure; primary; dichotomous; risk ratio; NT=26; NP=2,865. | **Number of trials:**  5 (Comparison 1, 3 months , all studies);  3 (Comparison 1, 3 months , ≤24 hours);  2 (Comparison 1, 3 months , >24 hours);  8 (Comparison 1, 6 months , all studies);  4 (Comparison 1, 6 months , ≤24 hours);  4 (Comparison 1, 6 months , >24 hours);  5 (Comparison 2, 3 months, all studies);  2 (Comparison 2, 3 months, ≤24 hours);  3 (Comparison 2, 3 months, > 24 hours);  2 (Comparison 2, 6 months);  4 (Comparison 3, 3 months , all studies);  1 (Comparison 3, 3 months , ≤24 hours);  3 (Comparison 3, 3 months , >24 hours);  1 (Comparison 3, 6 months);  1 (Comparison 3, 12 months).  **Covariate distribution (across trials):**  Comparison 1. Ketamine versus placebo comparisons:  NT=5, NP=384 (Incidence of any pain at 3 months (all studies)):  NT=1, NP=30 (Breast Surgery);  NT=1, NP=72 (Hemorrhoidectomy);  NT=2, NP=238 (Orthopedic Surgery);  NT=1, NP=44 (Thoracotomy).  Comparison 1. Ketamine versus placebo comparisons:  NT=3, NP=249 ( Incidence of any pain at 3 months (drug administration≤24 hours)):  NT=1, NP=30 (Breast Surgery);  NT=1, NP=72 (Hemorrhoidectomy);  NT=1, NP=147 (Orthopedic Surgery).  Comparison 1. Ketamine versus placebo comparisons:  NT=2, NP=135 (Incidence of any pain at 3 months (drug administration >24 hours)):  NT=1, NP=91 (Orthopedic Surgery);  NT=1, NP=44 (Thoracotomy).  Comparison 1. Ketamine versus placebo comparisons:  NT=8, NP=516 ( Incidence of any pain at 6 months (all studies)):  NT=2, NP=164 (Abdominal and/or pelvic surgery);  NT=1, NP=32 (Amputation);  NT=1, NP=86 (Breast surgery);  NT=3, NP=190 (Orthopedic surgery);  NT=1, NP=44 (Thoracotomy).  Comparison 1. Ketamine versus placebo comparisons:  NT=4, NP=318 ( Incidence of any pain at 6 months (drug administration ≤24 hours)):  NT=2, NP=164 (Abdominal and/or pelvic surgery);  NT=2, NP=154 (Orthopedic Surgery).  Comparison 1. Ketamine versus placebo comparisons:  NT=4, NP=198 ( Incidence of any pain at 6 months (drug administration >24 hours)):  NT=1, NP=32 (Amputation);  NT=1, NP=86 (Breast Surgery);  NT=1, NP=36 (Orthopedic Surgery);  NT=1, NP=44 (Thoracotomy).  Comparison 2. Gabapentin versus placebo:  NT=5, NP=280 ( Incidence of any pain at 3 months (all studies)):  NT=1, NP=38 (Amputation (phantom limb pain));  NT=1, NP=46 (Breast Surgery);  NT=1, NP=36 (Caesarean section);  NT=1, NP=40 (Cardiac Surgery);  NT=1, NP=120 (Thoracotomy).  Comparison 2. Gabapentin versus placebo:  NT=2, NP=156 (Incidence of any pain at 3 months (drug administration ≤24 hours)):  NT=1, NP=36 (Caesarean section);  NT=1, NP=120 (Thoracotomy).  Comparison 2. Gabapentin versus placebo:  NT=3, NP=124 (Incidence of any pain at 3 months (drug administration > 24 hours)):  NT=1, NP=38 (Amputation (phantom limb pain));  NT=1, NP=46 (Breast surgery);  NT=1, NP=40 (Cardiac surgery).  Comparison 2. Gabapentin versus placebo:  NT=2, NP=116 (Incidence of any pain at 6 months):  NT=1, NP=34 (Amputation (phantom limb pain) );  NT=1, NP=82 (Total hip arthroplasty).  Comparison 3. Pregabalin versus placebo:  NT=4, NP=439 (Incidence of any pain at 3 months follow up (all studies)):  NT=1, NP=57 (Cardiac surgery);  NT=1, NP=228 (Total Knee Arthroplasty);  NT=1, NP=60 (Spine Surgery);  NT=1, NP=94 (Thyroidectomy).  Comparison 3. Pregabalin versus placebo:  NT=1, NP=94 (Incidence of any pain at 3 months (drug administration≤24 hours)):  NT=1, NP=94 (Thyroidectomy).  Comparison 3. Pregabalin versus placebo:  NT=3, NP=345 (Incidence of any pain at 3months (drug administration >24 hours)):  NT=1, NP=57 (Cardiac Surgery);  NT=1, NP=228 (Total knee arthroplasty);  NT=1, NP=60 (Spine surgery).  Comparison 3. Pregabalin versus placebo:  NT=1, NP=228 (Incidence of any pain at 6 Months):  NT=1, NP=228 (Total Knee Arthroplasty).  Comparison 3. Pregabalin versus placebo:  NT=1, NP=60 (Incidence of any pain at 12 months follow up):  NT=1, NP=60 (Major spinal surgery).  **Covariate distribution (within trials):** NA. | **Analysis type in review according to glossary 1:** Subgroup analysis.  **Reported results:** Event rates, risk ratio, and CI for trials. Risk ratio, CI, z statistic, and p-value for meta-analytic subtotals and totals.  **Where reported:** Results text and forest plot.  **Method to detect interactions reported in review:** Not reported.  **Reported results from methods to detect interactions:** Test for subgroup differences (Chi-square statistic, p-value, and I square statistic).  **Where reported:** Forest plot. | **Reported detected interaction:** Not reported.  **What reported:**  *Ketamine*  Fourteen studies evaluating the perioperative effectiveness of ketamine were identified (Chaparro 2010; Crousier 2008; De Kock 2001; Duale 2009; Dullenkopf 2009; Hayes 2004; Katz 2004a; Malek 2006; Perrin 2009; Remerand 2009; Sen 2009a; Spreng 2010; Suzuki 2006; Sveticic 2008).  The studies are described in Table 1 in terms of the number of perioperative periods where the participants were exposed to ketamine: 1. preincisional loading dose; 2. intraoperative infusion; and 3. postoperative infusion (see Table 1). Three studies, one in thoracotomy (Duale 2009), a second one in amputation (Hayes 2004) and another one in total hip arthroplasty (Remerand 2009), administered ketamine in each of the periods of perioperative treatment; seven clinical trials used an incisional loading dose plus intraoperative infusion (Chaparro 2010; Crousier 2008; De Kock 2001; Katz 2004a; Perrin 2009; Sen 2009a; Spreng 2010); two studies started with an intraoperative infusion and continued the treatment for two (Malek 2006) or three (Suzuki 2006) postoperative days; only two studies limited the administration of ketamine to one of the three potential periods of treatment, one study used two different preincisional loading doses (Dullenkopf 2009) and one study administered ketamine mixed in the patient-controlled analgesia (PCA) device with morphine (Sveticic 2008).  A total cumulative dose was also calculated for each ketamine trial (see Table 1). A cumulative dose > 1 mg/kg was reached by eight out of the 14 aforementioned studies (De Kock 2001;Duale 2009; Hayes 2004; Malek 2006; Perrin 2009; Remerand 2009; Suzuki 2006; Sveticic 2008).  Only two ketamine studies did not report the outcomes of interest for this review (Dullenkopf 2009; Sen 2009).  Ketamine three month postoperative pain outcome  Five studies reported the incidence of pain at three months: one study in patients undergoing thoracotomy (Suzuki 2006); one study in breast surgery (Crousier 2008); two studies in major orthopaedic procedures (Remerand 2009; Sveticic 2008); and one study using (S)-ketamine reported the incidence of pain at three months after inguinal herniorrhaphy (Spreng 2010). A sixth study in thoracotomy patients evaluated pain outcomes at four months after surgery (Duale 2009), which we chose to exclude from this meta-analysis given the time point difference of one month. The overall effectiveness risk ratio showed a non-significant effect for ketamine compared to placebo (odds ratio (OR) 0.74, 95% confidence interval (CI) 0.45 to 1.23). A subgroup analysis based on duration of treatment suggested a significant effect of ketamine compared to placebo (OR 0.37, 95% CI 0.14 to 0.98) for studies evaluating ketamine treatment for longer than 24 hours compared to studies of less than 24 hours of ketamine treatment that, together, resulted in a non-significant effect of ketamine compared to placebo (OR 0.82, 95% CI 0.4 to 1.7).  Ketamine four month postoperative pain outcome  Only one study reported the incidence of pain at four months (Duale 2009). The data reported showed no difference between groups and corresponded with the item ’ongoing pain’ from a validated tool called the Neuropathic Pain Symptom Inventory, reported by the authors.  Ketamine six month postoperative pain outcome  Eight trials reported the incidence of pain at six months: two trials in joint replacement surgery (Perrin 2009; Remerand 2009); one in amputation surgery (Hayes 2004); one in thoracotomy (Suzuki 2006); one inmixed orthopedic procedures (Sveticic 2008); one in rectal carcinoma resection (De Kock 2001); one in breast surgery Malek 2006; and one in radical prostatectomy (Katz 2004a).  Eight clinical trials reported the incidence of pain (any) at six months follow-up (De Kock 2001; Hayes 2004; Katz 2004a; Malek 2006; Perrin 2009; Remerand 2009; Suzuki 2006; Sveticic 2008); the overall effectiveness risk ratio showed a significant difference favouring ketamine compared to placebo (OR 0.50, 95% CI 0.33 to 0.76) (see Figure 4). A subgroup analysis based on duration of treatment failed to support a significant effect of ketamine compared to placebo (OR 0.58, 95% CI 0.31 to 1.09) for studies evaluating ketamine treatment for longer than 24 hours, whereas studies of less than 24 hours of ketamine treatment surprisingly did result in a significant effect of ketamine compared to placebo (OR 0.45, 95% CI 0.26 to 0.78).  Three studies reported the incidence of moderate to severe pain (Hayes 2004;Malek 2006; Remerand 2009); the overall effectiveness risk ratio showed a significant difference favouring ketamine compared to placebo (risk ratio (RR) 0.44, 95% CI 0.20 to 0.93). Two of the three trials administered ketamine during the perioperative periods (Hayes 2004; Remerand 2009); the other trial started an intraoperative infusion that continued for two days after surgery (Malek 2006). The number needed to treat for patients that should receive ketamine to avoid one case of moderate to severe pain at six months was 10.83 (95% CI 5.69 to 109).  Ketamine 12 month postoperative pain outcome  Two studies reported the incidence of pain at 12 months. One of the studies compared high and low doses of intravenous and epidural ketamine versus placebo in patients undergoing rectal carcinoma resection (De Kock 2001); 92/100 patients were successfully contacted and 3/17 patients in the control group reported persistent pain versus none (0/37) in the intravenous ketamine groups; one patient in each epidural ketamine group (2/ 38) reported chronic pain (De Kock 2001). The other study was performed in women undergoing augmentation mammaplasty (Chaparro 2010). Only 50/106 patients could be contacted by a telephone call. None of the participants reported moderate or severe pain, however 3/25 patients in the ketamine group versus 7/25 in the placebo group reported sensory abnormalities such as persistent hypoesthesia and burning-like sensation around the surgical scar (Chaparro 2010). These studies were classified as heterogenous and a meta-analysis was not performed.  *Gabapentin*  We found 10 gabapentin clinical trials with long-term pain outcomes (Amr 2010; Brogly 2008; Clarke 2009; Fassoulaki 2002; Kinney 2011; Moore 2011; Nikolajsen 2006; Sen 2009; Sen 2009a; Ucak 2011). Table 2 describes trial features including surgery, preoperative and postoperative dosages, cumulative dose, as well as duration of gabapentin administration. One study administered gabapentin for 30 days (Nikolajsen 2006) in patients undergoing amputation. Two studies administered gabapentin for 10 days in breast surgery (Amr 2010; Fassoulaki 2002); however one of these trials did not report the incidence of pain but rather the median pain intensity score (Amr 2010) and was excluded from the meta-analysis. A single trial evaluated gabapentin administration in the setting of cardiac surgery (Ucak 2011). Six trials administered a single dose of gabapentin: one for inguinal herniorrhaphy (1200mg single dose) (Sen 2009); one in the setting of abdominal hysterectomy (1200 mg single dose) (Sen 2009a); one arthroplasty study compared the administration of 600 mg of gabapentin in the preoperative period versus postoperative (Clarke 2009); one clinical trial reported the impact of gabapentin 600 mg after thoracotomy (Kinney 2011); another trial evaluated the long-term impact of 1200 mg of gabapentin after thyroidectomy (Brogly 2008); and finally, one trial was conducted in the setting of cesarean section (Moore 2011).  Gabapentin three month postoperative pain outcome  Four clinical trials reported the incidence of any pain at three months (Fassoulaki 2002; Kinney 2011; Moore 2011; Ucak 2011). In one study, which did not report the three month pain incidence in the publication, the authors provided these data upon our request (Nikolajsen 2006). Two studies were not included in the analysis given that they did not report the incidence of pain but rather a continuous pain score (Sen 2009; Sen 2009a). Together, none of the five studies with threemonth data demonstrated a significant difference over placebo (OR 0.97, 95% CI 0.59 to 1.59) (see Figure 5). Subgroup analyses based on duration of treatment failed to show any different results for studies evaluating less than 24 hours of gabapentin treatment compared to those evaluating longer than 24 hours of gabapentin treatment.  Gabapentin six month postoperative pain outcome  Six out of 10 gabapentin trials followed the patients at six months. However, only two studies reported the primary outcome of the review, incidence of any pain (Clarke 2009;Nikolajsen 2006); neither of the two studies could demonstrate a significant difference with gabapentin over placebo. Three studies were not included in the analysis given that they did not report incidence, but a continuous pain score, and had no dichotomous outcomes such as incidence of moderate-severe pain (Amr 2010; Sen 2009; Sen 2009a). One study (Brogly 2008) reported the incidence of pain based on the scoring of the DN2 questionnaire, so the study was excluded from any meta-analysis.  *Pregabalin*  We identified five pregabalin trials with long-term pain outcomes (Burke 2010; Buvanendran 2010; Gianesello 2012; Kim 2010; Pesonen 2011). Table 3 describes the main characteristics of these studies, all of which involved more than one dose of study drug. Study medication was administered for at least two days in 3/5 trials (Buvanendran 2010;Gianesello 2012; Pesonen 2011).The preoperative dose was 150 mg (Kim 2010; Pesonen 2011) or 300 mg (Burke 2010; Buvanendran 2010; Gianesello 2012). The longest analgesic scheme opted for tapering pregabalin over two weeks for total knee arthroplasty (Buvanendran 2010). Pregabalin was studied in the setting of three major procedures: spine surgery, knee replacement, and cardiac surgery. A single trial evaluated the effects of pregabalin following thyroidectomy (Kim 2010).  Pregabalin three month postoperative pain outcome  Five clinical trials reported three month follow-up data (Burke 2010; Buvanendran 2010; Gianesello 2012; Kim 2010; Pesonen 2011) and four of them reported the incidence of any pain (Buvanendran 2010; Gianesello 2012; Kim2010; Pesonen 2011). One study was excluded from the meta-analysis due to the lack of reporting on the incidence of pain but rather a continuous pain score (Burke 2010). Only one (Pesonen 2011) of these studies demonstrated a substantial benefit of pregabalin versus placebo leading to an overall significant effect of pregabalin over placebo (OR 0.60, 95% CI 0.39 to 0.93). However, assessment of heterogeneity yielded an I2 of 28.5% (see Figure 6). Only one study evaluated less than 24 hours of pregabalin treatment so we did not perform any subgroup analyses according to treatment duration.  Pregabalin six month postoperative pain outcome  Only one trial continued the follow-up for six months (Buvanendran 2010), in total knee arthroplasty. The authors were contacted and provided the outcome data upon request, which suggested a six month ’any’ pain incidence of 15/113 for pregabalin versus 29/115 for placebo and a ’moderate-to-severe’ pain incidence of 3/113 for pregabalin versus 13/115 for placebo.  Pregabalin 12 month postoperative pain outcome  One trial followed the patients for one year (Gianesello 2012) after major spine surgery. Based on our primary outcome, the study could not demonstrate a significant benefit of pregabalin over placebo.  **Where reported:** Results text.  **Interaction exists based on reported results:**  Yes for ‘Pregabalin versus placebo; Incidence of any pain at 3months (drug administration >24 hours)’. No for ‘Comparison 1. Ketamine versus placebo comparisons; Incidence of any pain at 3 months (all studies) ’, ‘Comparison 1. Ketamine versus placebo comparisons; Incidence of any pain at 3 months (drug administration≤24 hours) ’, ‘Comparison 1. Ketamine versus placebo comparisons; Incidence of any pain at 3 months (drug administration >24 hours’, ‘Comparison 1. Ketamine versus placebo comparisons; Incidence of any pain at 6 months (all studies) ’, ‘Comparison 1. Ketamine versus placebo comparisons; Incidence of any pain at 6 months (drug administration ≤ 24 hours) ’, ‘Comparison 1. Ketamine versus placebo comparisons; Incidence of any pain at 6 months (drug administration >24 hours) ’, ‘Comparison 2. Gabapentin versus placebo; Incidence of any pain at 3 months (all studies) ’, ‘Comparison 2. Gabapentin versus placebo; Incidence of any pain at 3 months (drug administration ≤ 24 hours’, ‘Comparison 2. Gabapentin versus placebo; Incidence of any pain at 3 months (drug administration > 24 hours) ’, ‘Comparison 2. Gabapentin versus placebo; Incidence of any pain at 6 months’, ‘Comparison 3. Pregabalin versus placebo; Incidence of any pain at 3 months follow up (all studies)’.  **How determined:**  Comparison 1. Ketamine versus placebo comparisons; Incidence of any pain at 3 months (all studies): Test for subgroup differences: Chi2= 4.49, df= 3 (P= 0.21), I2=33%  Comparison 1. Ketamine versus placebo comparisons; Incidence of any pain at 3 months (drug administration≤24 hours): Test for subgroup differences: Chi2= 0.76, df= 2 (P= 0.68), I2=0.0%  Comparison 1. Ketamine versus placebo comparisons; Incidence of any pain at 3 months (drug administration >24 hours: Test for subgroup differences: Chi2= 0.10, df= 1 (P= 0.76), I2=0.0%  Comparison 1. Ketamine versus placebo comparisons; Incidence of any pain at 6 months (all studies): Test for subgroup differences: Chi2= 2.49, df= 4 (P= 0.65), I2=0.0%  Comparison 1. Ketamine versus placebo comparisons; Incidence of any pain at 6 months (drug administration ≤ 24 hours): Test for subgroup differences: Chi2= 0.93, df= 1 (P= 0.34), I2=0.0%  Comparison 1. Ketamine versus placebo comparisons; Incidence of any pain at 6 months (drug administration >24 hours): Test for subgroup differences: Chi2= 1.00, df= 3 (P= 0.80), I2=0.0%  Comparison 2. Gabapentin versus placebo; Incidence of any pain at 3 months (all studies):  Test for subgroup differences: Chi2= 1.10, df= 4 (P= 0.89), I2=0.0%  Comparison 2. Gabapentin versus placebo; Incidence of any pain at 3 months (drug administration ≤ 24 hours: Test for subgroup differences: Chi2= 0.42, df= 1 (P= 0.52), I2=0.0%  Comparison 2. Gabapentin versus placebo; Incidence of any pain at 3 months (drug administration > 24 hours): Test for subgroup differences: Chi2= 0.52, df= 2 (P= 0.77), I2=0.0%  Comparison 2. Gabapentin versus placebo; Incidence of any pain at 6 months: Test for subgroup differences: Chi2= 0.01, df= 1 (P= 0.93), I2=0.0%  Comparison 3. Pregabalin versus placebo; Incidence of any pain at 3 months follow up (all studies): Test for subgroup differences: Chi2= 4.92, df= 3 (P= 0.18), I2=39%  Comparison 3. Pregabalin versus placebo; Incidence of any pain at 3months (drug administration >24 hours): Test for subgroup differences: Chi2= 4.92, df= 2 (P= 0.09), I2=59%  **Reported importance of the interaction:** Not reported.  **What reported:** Not reported.  **Where reported:** NA  **Reported plausibility of the interaction:** Not reported.  **What reported:** Not reported.  **Where reported:** NA  **Reported confounding possibility:** Not reported.  **What reported:** Not reported.  **Where reported:** NA.  **Mentioned covariate distribution:** Not reported.  **What reported:** Not reported.  **Where reported:** NA. |
| Chaparro 2013 | **Protocol:** ‘The primary comparison of interest will be between study drug(s) and placebo. Comparisons of study drug(s) and any other active treatment comparators will also be made.’  **Review methods: ‘**The primary comparison of interest was between study drug(s) and placebo. Comparisons of study drug(s) and any other active treatment comparators were also to be made.’  **Review results: ‘**Comparison 1. Ketamine versus placebo comparisons’; ‘Comparison 2. Gabapentin versus placebo’; ‘Comparison 3. Pregabalin versus placebo’; ‘Comparison 4. Venlafaxine versus placebo comparisons’ (in plot) and ‘other drugs’ (in text).  **Type:** Intervention  **Covariate summary:** Type of intervention or control. | Proportion of participants reporting any pain at the anatomical site of the procedure or pain referred to the surgical site, or both (for example phantom limb pain, shoulder pain referred from the diaphragm etc.), three months or more after the procedure; primary; dichotomous; risk ratio; NT=26; NP=2,865. | **Number of trials:** 22.  **Covariate distribution (across trials):**  NT=12, NP=779 (Comparison 1. Ketamine versus placebo comparisons);  NT=6, NP=348 (Comparison 2. Gabapentin versus placebo);  NT=4, NP=439 (Comparison 3. Pregabalin versus placebo);  NT=0, NP=0 (Comparison 4. Venlafaxine versus placebo comparisons).  In the text:  NT=12, NP=779 (Ketamine);  NT=6, NP=348 (Gabapentin);  NT=4, NP=439 (Pregabalin);  NT=1, NP=28 (Corticosteroids);  NT=1, NP=902 (Non-steroidal anti-inflammatory drugs (NSAIDs));  NT=2, NP=175 (Other drugs: Mexiletine);  NT=1, NP=26 (Other drugs: Lidocaine);  NT=1, NP=22 (Other drugs: Amantadine);  NT=0, NP=0 (Other drugs: Dextromethorphan);  NT=1, NP=19 (Other drugs: Memantine);  NT=0, NP=0 (Other drugs: Venlafaxine);  NT=0, NP=0 (Other drugs: Nitrous oxide);  NT=1, NP=6 (Other drugs: Fentanyl).  **Covariate distribution (within trials):** NA. | **Analysis type in review according to glossary 1:** Subgroup analysis.  **Reported results:** Event rates, risk ratio, and CI for trials. Risk ratio, CI and p-value for meta-analytic subtotals.  **Where reported:** Results text and forest plot.  **Method to detect interactions reported in review:** Not reported.  **Reported results from methods to detect interactions:** Not reported.  **Where reported:** NA. | **Reported detected interaction:** Not reported.  **What reported:**  *Ketamine*  Fourteen studies evaluating the perioperative effectiveness of ketamine were identified (Chaparro 2010; Crousier 2008; De Kock 2001; Duale 2009; Dullenkopf 2009; Hayes 2004; Katz 2004a; Malek 2006; Perrin 2009; Remerand 2009; Sen 2009a; Spreng 2010; Suzuki 2006; Sveticic 2008).  The studies are described in Table 1 in terms of the number of perioperative periods where the participants were exposed to ketamine: 1. preincisional loading dose; 2. intraoperative infusion; and 3. postoperative infusion (see Table 1). Three studies, one in thoracotomy (Duale 2009), a second one in amputation (Hayes 2004) and another one in total hip arthroplasty (Remerand 2009), administered ketamine in each of the periods of perioperative treatment; seven clinical trials used an incisional loading dose plus intraoperative infusion (Chaparro 2010; Crousier 2008; De Kock 2001; Katz 2004a; Perrin 2009; Sen 2009a; Spreng 2010); two studies started with an intraoperative infusion and continued the treatment for two (Malek 2006) or three (Suzuki 2006) postoperative days; only two studies limited the administration of ketamine to one of the three potential periods of treatment, one study used two different preincisional loading doses (Dullenkopf 2009) and one study administered ketamine mixed in the patient-controlled analgesia (PCA) device with morphine (Sveticic 2008).  A total cumulative dose was also calculated for each ketamine trial (see Table 1). A cumulative dose > 1 mg/kg was reached by eight out of the 14 aforementioned studies (De Kock 2001;Duale 2009; Hayes 2004; Malek 2006; Perrin 2009; Remerand 2009; Suzuki 2006; Sveticic 2008).  Only two ketamine studies did not report the outcomes of interest for this review (Dullenkopf 2009; Sen 2009).  Ketamine three month postoperative pain outcome  Five studies reported the incidence of pain at three months: one study in patients undergoing thoracotomy (Suzuki 2006); one study in breast surgery (Crousier 2008); two studies in major orthopaedic procedures (Remerand 2009; Sveticic 2008); and one study using (S)-ketamine reported the incidence of pain at three months after inguinal herniorrhaphy (Spreng 2010). A sixth study in thoracotomy patients evaluated pain outcomes at four months after surgery (Duale 2009), which we chose to exclude from this meta-analysis given the time point difference of one month. The overall effectiveness risk ratio showed a non-significant effect for ketamine compared to placebo (odds ratio (OR) 0.74, 95% confidence interval (CI) 0.45 to 1.23). A subgroup analysis based on duration of treatment suggested a significant effect of ketamine compared to placebo (OR 0.37, 95% CI 0.14 to 0.98) for studies evaluating ketamine treatment for longer than 24 hours compared to studies of less than 24 hours of ketamine treatment that, together, resulted in a non-significant effect of ketamine compared to placebo (OR 0.82, 95% CI 0.4 to 1.7).  Ketamine four month postoperative pain outcome  Only one study reported the incidence of pain at four months (Duale 2009). The data reported showed no difference between groups and corresponded with the item ’ongoing pain’ from a validated tool called the Neuropathic Pain Symptom Inventory, reported by the authors.  Ketamine six month postoperative pain outcome  Eight trials reported the incidence of pain at six months: two trials in joint replacement surgery (Perrin 2009; Remerand 2009); one in amputation surgery (Hayes 2004); one in thoracotomy (Suzuki 2006); one inmixed orthopedic procedures (Sveticic 2008); one in rectal carcinoma resection (De Kock 2001); one in breast surgery Malek 2006; and one in radical prostatectomy (Katz 2004a).  Eight clinical trials reported the incidence of pain (any) at six months follow-up (De Kock 2001; Hayes 2004; Katz 2004a; Malek 2006; Perrin 2009; Remerand 2009; Suzuki 2006; Sveticic 2008); the overall effectiveness risk ratio showed a significant difference favouring ketamine compared to placebo (OR 0.50, 95% CI 0.33 to 0.76) (see Figure 4). A subgroup analysis based on duration of treatment failed to support a significant effect of ketamine compared to placebo (OR 0.58, 95% CI 0.31 to 1.09) for studies evaluating ketamine treatment for longer than 24 hours, whereas studies of less than 24 hours of ketamine treatment surprisingly did result in a significant effect of ketamine compared to placebo (OR 0.45, 95% CI 0.26 to 0.78).  Three studies reported the incidence of moderate to severe pain (Hayes 2004;Malek 2006; Remerand 2009); the overall effectiveness risk ratio showed a significant difference favouring ketamine compared to placebo (risk ratio (RR) 0.44, 95% CI 0.20 to 0.93). Two of the three trials administered ketamine during the perioperative periods (Hayes 2004; Remerand 2009); the other trial started an intraoperative infusion that continued for two days after surgery (Malek 2006). The number needed to treat for patients that should receive ketamine to avoid one case of moderate to severe pain at six months was 10.83 (95% CI 5.69 to 109).  Ketamine 12 month postoperative pain outcome  Two studies reported the incidence of pain at 12 months. One of the studies compared high and low doses of intravenous and epidural ketamine versus placebo in patients undergoing rectal carcinoma resection (De Kock 2001); 92/100 patients were successfully contacted and 3/17 patients in the control group reported persistent pain versus none (0/37) in the intravenous ketamine groups; one patient in each epidural ketamine group (2/ 38) reported chronic pain (De Kock 2001). The other study was performed in women undergoing augmentation mammaplasty (Chaparro 2010). Only 50/106 patients could be contacted by a telephone call. None of the participants reported moderate or severe pain, however 3/25 patients in the ketamine group versus 7/25 in the placebo group reported sensory abnormalities such as persistent hypoesthesia and burning-like sensation around the surgical scar (Chaparro 2010). These studies were classified as heterogenous and a meta-analysis was not performed.  *Gabapentin*  We found 10 gabapentin clinical trials with long-term pain outcomes (Amr 2010; Brogly 2008; Clarke 2009; Fassoulaki 2002; Kinney 2011; Moore 2011; Nikolajsen 2006; Sen 2009; Sen 2009a; Ucak 2011). Table 2 describes trial features including surgery, preoperative and postoperative dosages, cumulative dose, as well as duration of gabapentin administration. One study administered gabapentin for 30 days (Nikolajsen 2006) in patients undergoing amputation. Two studies administered gabapentin for 10 days in breast surgery (Amr 2010; Fassoulaki 2002); however one of these trials did not report the incidence of pain but rather the median pain intensity score (Amr 2010) and was excluded from the meta-analysis. A single trial evaluated gabapentin administration in the setting of cardiac surgery (Ucak 2011). Six trials administered a single dose of gabapentin: one for inguinal herniorrhaphy (1200mg single dose) (Sen 2009); one in the setting of abdominal hysterectomy (1200 mg single dose) (Sen 2009a); one arthroplasty study compared the administration of 600 mg of gabapentin in the preoperative period versus postoperative (Clarke 2009); one clinical trial reported the impact of gabapentin 600 mg after thoracotomy (Kinney 2011); another trial evaluated the long-term impact of 1200 mg of gabapentin after thyroidectomy (Brogly 2008); and finally, one trial was conducted in the setting of cesarean section (Moore 2011).  Gabapentin three month postoperative pain outcome  Four clinical trials reported the incidence of any pain at three months (Fassoulaki 2002; Kinney 2011; Moore 2011; Ucak 2011). In one study, which did not report the three month pain incidence in the publication, the authors provided these data upon our request (Nikolajsen 2006). Two studies were not included in the analysis given that they did not report the incidence of pain but rather a continuous pain score (Sen 2009; Sen 2009a). Together, none of the five studies with threemonth data demonstrated a significant difference over placebo (OR 0.97, 95% CI 0.59 to 1.59) (see Figure 5). Subgroup analyses based on duration of treatment failed to show any different results for studies evaluating less than 24 hours of gabapentin treatment compared to those evaluating longer than 24 hours of gabapentin treatment.  Gabapentin six month postoperative pain outcome  Six out of 10 gabapentin trials followed the patients at six months. However, only two studies reported the primary outcome of the review, incidence of any pain (Clarke 2009;Nikolajsen 2006); neither of the two studies could demonstrate a significant difference with gabapentin over placebo. Three studies were not included in the analysis given that they did not report incidence, but a continuous pain score, and had no dichotomous outcomes such as incidence of moderate-severe pain (Amr 2010; Sen 2009; Sen 2009a). One study (Brogly 2008) reported the incidence of pain based on the scoring of the DN2 questionnaire, so the study was excluded from any meta-analysis.  *Pregabalin*  We identified five pregabalin trials with long-term pain outcomes (Burke 2010; Buvanendran 2010; Gianesello 2012; Kim 2010; Pesonen 2011). Table 3 describes the main characteristics of these studies, all of which involved more than one dose of study drug. Study medication was administered for at least two days in 3/5 trials (Buvanendran 2010;Gianesello 2012; Pesonen 2011).The preoperative dose was 150 mg (Kim 2010; Pesonen 2011) or 300 mg (Burke 2010; Buvanendran 2010; Gianesello 2012). The longest analgesic scheme opted for tapering pregabalin over two weeks for total knee arthroplasty (Buvanendran 2010). Pregabalin was studied in the setting of three major procedures: spine surgery, knee replacement, and cardiac surgery. A single trial evaluated the effects of pregabalin following thyroidectomy (Kim 2010).  Pregabalin three month postoperative pain outcome  Five clinical trials reported three month follow-up data (Burke 2010; Buvanendran 2010; Gianesello 2012; Kim 2010; Pesonen 2011) and four of them reported the incidence of any pain (Buvanendran 2010; Gianesello 2012; Kim2010; Pesonen 2011). One study was excluded from the meta-analysis due to the lack of reporting on the incidence of pain but rather a continuous pain score (Burke 2010). Only one (Pesonen 2011) of these studies demonstrated a substantial benefit of pregabalin versus placebo leading to an overall significant effect of pregabalin over placebo (OR 0.60, 95% CI 0.39 to 0.93). However, assessment of heterogeneity yielded an I2 of 28.5% (see Figure 6). Only one study evaluated less than 24 hours of pregabalin treatment so we did not perform any subgroup analyses according to treatment duration.  Pregabalin six month postoperative pain outcome  Only one trial continued the follow-up for six months (Buvanendran 2010), in total knee arthroplasty. The authors were contacted and provided the outcome data upon request, which suggested a six month ’any’ pain incidence of 15/113 for pregabalin versus 29/115 for placebo and a ’moderate-to-severe’ pain incidence of 3/113 for pregabalin versus 13/115 for placebo.  Pregabalin 12 month postoperative pain outcome  One trial followed the patients for one year (Gianesello 2012) after major spine surgery. Based on our primary outcome, the study could not demonstrate a significant benefit of pregabalin over placebo.  *Corticosteroids*  Three perioperative corticosteroid trials reported on long-term pain outcomes. One study evaluated the long-term analgesic effect of 40 mg of dexamethasone administered before total hip arthroplasty (Bergeron 2009). The study contacted, one year after surgery, 31/50 patients that were randomized. The study could not demonstrate any difference between groups for the main outcomes of pain intensity and function, measured by the Harris hip score. Another trial was developed to evaluate the long-term impact of a single preoperative dose of methylprednisolone (125 mg) for augmentation mammaplasty (Romundstad 2006). The study made contact with 175/219 randomized patients one year after surgery.This clinical trial showed no benefit in terms of pain scores, however it showed a significant difference favouring the steroid for the prevalence of hyperesthesia (Romundstad 2006). A third steroid trial was performed using a loading dose and four days of intravenous infusion of hydrocortisone after cardiac surgery (Weis 2006); the outcomes were measured at six months, when 28/36 patients were successfully contacted. The conclusions by the authors were that stress doses of hydrocortisone had a significant positive impact on chronic stress symptoms, including pain, and health-related quality of life. Heterogeneity between these trials, based on drug, follow-up timing and pain outcomes measured, precluded any possible meta-analysis.  *Non-steroidal anti-inflammatory drugs (NSAIDs)*  Three perioperative NSAID trials reported long-term pain outcomes. One of these trials tested ibuprofen 400 mg before breast mastectomy plus four additional doses after the procedure (Lakdja 1997). Outcome assessment was planned at six months, when 28/ 30 patients were contacted; the authors concluded that ibuprofen had no significant impact on the control of dysesthesia or postmastectomy pain syndrome (Lakdja 1997). A second ibuprofen trial was in patients undergoing total hip replacement (Fransen 2006); the primary outcomes were the changes in self-reported pain and function, measured using theWestern Ontario and Mc- Master University arthritis index. Patients were contacted six to 12 months after surgery; the study could not demonstrate a significant impact of ibuprofen on the primary outcome (Fransen 2006). A third study included parecoxib as the active control arm of the methylprednisolone trial mentioned before (Romundstad 2006); a single dose of 40 mg of parecoxib made no difference versus placebo one year after surgery in patients undergoing augmentation mammaplasty (Romundstad 2006). Heterogeneity between these trials, based on drug, follow-up timing and pain outcomes measured, precluded any possible meta-analysis.  *Other drugs*  A comprehensive summary of the clinical trials developed for the following drugs is presented at Table 4.  Mexiletine: two clinical trials evaluated the impact of mexiletine (orally administered local anesthetic) on persistent pain after surgery. One of these studies (Fassoulaki 2001) had four arms, comparing the use of a regional block combined with mexiletine versus the interventions alone versus placebo. For the interest of this review, the study followed the patients for three months after surgery; the incidence of chronic pain or the mean pain scores did not differ between the four groups. However, the patients randomized to the combination reported less ’decreased sensation or hypoesthesia’ at three months after surgery (Fassoulaki 2001). A second clinical trial (Fassoulaki 2002) used mexiletine (200 mg orally three times a day for 10 days) as an active control arm of a study that evaluated gabapentin for breast surgery (Fassoulaki 2002). As we mentioned before, the study followed the patients for three months after surgery. The incidence of chronic pain and the mean pain score had no statistical difference between groups. However, the authors reported a significant difference in burning sensation, equally favouring the administration of gabapentin or mexiletine over placebo (Fassoulaki 2002).  Lidocaine: one clinical trial (Grigoras 2012) evaluated the administration of intravenous lidocaine (bolus dose 1.5 mg/kg plus infusion 1.5 mg/kg/hour up to the first postoperative hour) in 36 patients that underwent breast cancer surgery. Long-term followups were planned at three months after surgery. All patients were successfully contacted at three months after surgery: 2/17 patients in the lidocaine group versus 9/19 patients in the placebo group reported persistent pain after surgery; 0/17 in the lidocaine group versus 8/19 in the placebo group reportedmovement-evoked pain; the area of hyperalgesia was significantly larger in the placebo group at three months after surgery.  Amantadine: one clinical trial (Eisenberg 2007) evaluated the administration of amantadine sulfate (100 mg twice daily for 14 days starting the day before surgery) in 22 patients that underwent breast cancer surgery. Long-term follow-ups were planned at three and six months after surgery, and 17/22 patients were contacted at six months after surgery. All patients (9/9) in the amantadine group reported persistent pain after surgery versus 6/8 in the placebo group. The Short Form of the McGill pain questionnaire (SF-MPQ) was used for the follow-ups and no differences in the number of pain descriptors were found between groups. This study excluded patients that reported preoperative pain and participants were allowed to take opioids, anti-inflammatories or paracetamol as required.  Dextromethorphan  Memantine: one study evaluated increasing doses of memantine (10 mg to 30 mg) administered during the first four postoperative weeks (Schley 2007) in patients undergoing traumatic amputation. All patients received a regional block for postoperative analgesia during one week. Patients were followed at one, six and 12months after surgery. Pain prevalence was significantly lower in the memantine group compared to the placebo group at six, but not 12, months after surgery (Schley 2007).  Venlafaxine  Nitrous oxide  Fentanyl: a single opioid trialwas identified that fulfilled the inclusion criteria for this review (Karanikolas 2011). This was a five arm study comparing standard regimens of preoperative, intraoperative and postoperative analgesia using PCA or epidural analgesia, or both, in 65 patients undergoing lower limb amputation. The four active groups were compared with intramuscular administration of meperidine; to keep the blinding, subcutaneous catheters plus second pumps were used across the study. A total of 56/65 randomized patients were contacted at six months after surgery. Based on the incidence of phantomlimb pain, the study concluded that optimized analgesia with an epidural opioid or PCA started two days before surgery and continued up to the second day after surgery was better than intermittent administration ofmeperidine (Karanikolas 2011).  **Where reported:** Results text.  **Interaction exists based on reported results:** Not reported.  **How determined:** NA.  **Reported importance of the interaction:** Not reported.  **What reported:** Not reported.  **Where reported:** NA  **Reported plausibility of the interaction:** Not reported.  **What reported:** Not reported.  **Where reported:** NA  **Reported confounding possibility:** Not reported.  **What reported:** Not reported.  **Where reported:** NA.  **Mentioned covariate distribution:** Not reported.  **What reported:** Not reported.  **Where reported:** NA. |
| Chaparro 2013 | **Protocol:** Not reported.  **Review methods:** Not reported.  **Review results: ‘**Drug administration >24 hours’; ‘Drug administration ≤ 24 hours’.  **Type:** Intervention  **Covariate summary:** Timing of intervention. | Proportion of participants reporting any pain at the anatomical site of the procedure or pain referred to the surgical site, or both (for example phantom limb pain, shoulder pain referred from the diaphragm etc.), three months or more after the procedure; primary; dichotomous; risk ratio; NT=26; NP=2,865. | **Number of trials:** 5 (comparison 1, 3 months); 6 (comparison 1, 6 months); 5 (comparison 2); 4 (comparison 3).  **Covariate distribution (across trials):**  NT=5, NP=384 (Comparison 1. Ketamine versus placebo comparisons):  NT=3, NP=249 ( Incidence of any pain at 3 months (drug administration≤ 24 hours));  NT=2, NP=135 (Incidence of any pain at 3 months (drug administration >24 hours)).  NT=6, NP=806 (Comparison 1. Ketamine versus placebo comparisons):  NT=4, NP=318 ( Incidence of any pain at 6 months (drug administration≤24 hours));  NT=4, NP=198 ( Incidence of any pain at 6 months (drug administration >24 hours)).  NT=5, NP=280 (Comparison 2. Gabapentin versus placebo):  NT=2, NP=156 (Incidence of any pain at 3 months (drug administration ≤ 24 hours));  NT=3, NP=124 (Incidence of any pain at 3 months (drug administration > 24 hours)).  NT=4, NP=439 (Comparison 3. Pregabalin versus placebo):  NT=1, NP=94 (Incidence of any pain at 3 months (drug administration≤ 24 hours));  NT=3, NP=345 (Incidence of any pain at 3months (drug administration >24 hours)).  **Covariate distribution (within trials):** NA. | **Analysis type in review according to glossary 1:** Subgroup analysis.  **Reported results:** Event rates, risk ratio, and CI for trials. Risk ratio, CI and p-value for meta-analytic subtotals.  **Where reported:** Results text and forest plot.  **Method to detect interactions reported in review:** Not reported.  **Reported results from methods to detect interactions:** Not reported.  **Where reported:** NA. | **Reported detected interaction:** Not reported.  **What reported:**  *Ketamine*  Fourteen studies evaluating the perioperative effectiveness of ketamine were identified (Chaparro 2010; Crousier 2008; De Kock 2001; Duale 2009; Dullenkopf 2009; Hayes 2004; Katz 2004a; Malek 2006; Perrin 2009; Remerand 2009; Sen 2009a; Spreng 2010; Suzuki 2006; Sveticic 2008).  The studies are described in Table 1 in terms of the number of perioperative periods where the participants were exposed to ketamine: 1. preincisional loading dose; 2. intraoperative infusion; and 3. postoperative infusion (see Table 1). Three studies, one in thoracotomy (Duale 2009), a second one in amputation (Hayes 2004) and another one in total hip arthroplasty (Remerand 2009), administered ketamine in each of the periods of perioperative treatment; seven clinical trials used an incisional loading dose plus intraoperative infusion (Chaparro 2010; Crousier 2008; De Kock 2001; Katz 2004a; Perrin 2009; Sen 2009a; Spreng 2010); two studies started with an intraoperative infusion and continued the treatment for two (Malek 2006) or three (Suzuki 2006) postoperative days; only two studies limited the administration of ketamine to one of the three potential periods of treatment, one study used two different preincisional loading doses (Dullenkopf 2009) and one study administered ketamine mixed in the patient-controlled analgesia (PCA) device with morphine (Sveticic 2008).  A total cumulative dose was also calculated for each ketamine trial (see Table 1). A cumulative dose > 1 mg/kg was reached by eight out of the 14 aforementioned studies (De Kock 2001;Duale 2009; Hayes 2004; Malek 2006; Perrin 2009; Remerand 2009; Suzuki 2006; Sveticic 2008).  Only two ketamine studies did not report the outcomes of interest for this review (Dullenkopf 2009; Sen 2009).  Ketamine three month postoperative pain outcome  Five studies reported the incidence of pain at three months: one study in patients undergoing thoracotomy (Suzuki 2006); one study in breast surgery (Crousier 2008); two studies in major orthopaedic procedures (Remerand 2009; Sveticic 2008); and one study using (S)-ketamine reported the incidence of pain at three months after inguinal herniorrhaphy (Spreng 2010). A sixth study in thoracotomy patients evaluated pain outcomes at four months after surgery (Duale 2009), which we chose to exclude from this meta-analysis given the time point difference of one month. The overall effectiveness risk ratio showed a non-significant effect for ketamine compared to placebo (odds ratio (OR) 0.74, 95% confidence interval (CI) 0.45 to 1.23). A subgroup analysis based on duration of treatment suggested a significant effect of ketamine compared to placebo (OR 0.37, 95% CI 0.14 to 0.98) for studies evaluating ketamine treatment for longer than 24 hours compared to studies of less than 24 hours of ketamine treatment that, together, resulted in a non-significant effect of ketamine compared to placebo (OR 0.82, 95% CI 0.4 to 1.7).  Ketamine four month postoperative pain outcome  Only one study reported the incidence of pain at four months (Duale 2009). The data reported showed no difference between groups and corresponded with the item ’ongoing pain’ from a validated tool called the Neuropathic Pain Symptom Inventory, reported by the authors.  Ketamine six month postoperative pain outcome  Eight trials reported the incidence of pain at six months: two trials in joint replacement surgery (Perrin 2009; Remerand 2009); one in amputation surgery (Hayes 2004); one in thoracotomy (Suzuki 2006); one inmixed orthopedic procedures (Sveticic 2008); one in rectal carcinoma resection (De Kock 2001); one in breast surgery Malek 2006; and one in radical prostatectomy (Katz 2004a).  Eight clinical trials reported the incidence of pain (any) at six months follow-up (De Kock 2001; Hayes 2004; Katz 2004a; Malek 2006; Perrin 2009; Remerand 2009; Suzuki 2006; Sveticic 2008); the overall effectiveness risk ratio showed a significant difference favouring ketamine compared to placebo (OR 0.50, 95% CI 0.33 to 0.76) (see Figure 4). A subgroup analysis based on duration of treatment failed to support a significant effect of ketamine compared to placebo (OR 0.58, 95% CI 0.31 to 1.09) for studies evaluating ketamine treatment for longer than 24 hours, whereas studies of less than 24 hours of ketamine treatment surprisingly did result in a significant effect of ketamine compared to placebo (OR 0.45, 95% CI 0.26 to 0.78).  Three studies reported the incidence of moderate to severe pain (Hayes 2004;Malek 2006; Remerand 2009); the overall effectiveness risk ratio showed a significant difference favouring ketamine compared to placebo (risk ratio (RR) 0.44, 95% CI 0.20 to 0.93). Two of the three trials administered ketamine during the perioperative periods (Hayes 2004; Remerand 2009); the other trial started an intraoperative infusion that continued for two days after surgery (Malek 2006). The number needed to treat for patients that should receive ketamine to avoid one case of moderate to severe pain at six months was 10.83 (95% CI 5.69 to 109).  Ketamine 12 month postoperative pain outcome  Two studies reported the incidence of pain at 12 months. One of the studies compared high and low doses of intravenous and epidural ketamine versus placebo in patients undergoing rectal carcinoma resection (De Kock 2001); 92/100 patients were successfully contacted and 3/17 patients in the control group reported persistent pain versus none (0/37) in the intravenous ketamine groups; one patient in each epidural ketamine group (2/ 38) reported chronic pain (De Kock 2001). The other study was performed in women undergoing augmentation mammaplasty (Chaparro 2010). Only 50/106 patients could be contacted by a telephone call. None of the participants reported moderate or severe pain, however 3/25 patients in the ketamine group versus 7/25 in the placebo group reported sensory abnormalities such as persistent hypoesthesia and burning-like sensation around the surgical scar (Chaparro 2010). These studies were classified as heterogenous and a meta-analysis was not performed.  *Gabapentin*  We found 10 gabapentin clinical trials with long-term pain outcomes (Amr 2010; Brogly 2008; Clarke 2009; Fassoulaki 2002; Kinney 2011; Moore 2011; Nikolajsen 2006; Sen 2009; Sen 2009a; Ucak 2011). Table 2 describes trial features including surgery, preoperative and postoperative dosages, cumulative dose, as well as duration of gabapentin administration. One study administered gabapentin for 30 days (Nikolajsen 2006) in patients undergoing amputation. Two studies administered gabapentin for 10 days in breast surgery (Amr 2010; Fassoulaki 2002); however one of these trials did not report the incidence of pain but rather the median pain intensity score (Amr 2010) and was excluded from the meta-analysis. A single trial evaluated gabapentin administration in the setting of cardiac surgery (Ucak 2011). Six trials administered a single dose of gabapentin: one for inguinal herniorrhaphy (1200mg single dose) (Sen 2009); one in the setting of abdominal hysterectomy (1200 mg single dose) (Sen 2009a); one arthroplasty study compared the administration of 600 mg of gabapentin in the preoperative period versus postoperative (Clarke 2009); one clinical trial reported the impact of gabapentin 600 mg after thoracotomy (Kinney 2011); another trial evaluated the long-term impact of 1200 mg of gabapentin after thyroidectomy (Brogly 2008); and finally, one trial was conducted in the setting of cesarean section (Moore 2011).  Gabapentin three month postoperative pain outcome  Four clinical trials reported the incidence of any pain at three months (Fassoulaki 2002; Kinney 2011; Moore 2011; Ucak 2011). In one study, which did not report the three month pain incidence in the publication, the authors provided these data upon our request (Nikolajsen 2006). Two studies were not included in the analysis given that they did not report the incidence of pain but rather a continuous pain score (Sen 2009; Sen 2009a). Together, none of the five studies with threemonth data demonstrated a significant difference over placebo (OR 0.97, 95% CI 0.59 to 1.59) (see Figure 5). Subgroup analyses based on duration of treatment failed to show any different results for studies evaluating less than 24 hours of gabapentin treatment compared to those evaluating longer than 24 hours of gabapentin treatment.  Gabapentin six month postoperative pain outcome  Six out of 10 gabapentin trials followed the patients at six months. However, only two studies reported the primary outcome of the review, incidence of any pain (Clarke 2009;Nikolajsen 2006); neither of the two studies could demonstrate a significant difference with gabapentin over placebo. Three studies were not included in the analysis given that they did not report incidence, but a continuous pain score, and had no dichotomous outcomes such as incidence of moderate-severe pain (Amr 2010; Sen 2009; Sen 2009a). One study (Brogly 2008) reported the incidence of pain based on the scoring of the DN2 questionnaire, so the study was excluded from any meta-analysis.  *Pregabalin*  We identified five pregabalin trials with long-term pain outcomes (Burke 2010; Buvanendran 2010; Gianesello 2012; Kim 2010; Pesonen 2011). Table 3 describes the main characteristics of these studies, all of which involved more than one dose of study drug. Study medication was administered for at least two days in 3/5 trials (Buvanendran 2010;Gianesello 2012; Pesonen 2011).The preoperative dose was 150 mg (Kim 2010; Pesonen 2011) or 300 mg (Burke 2010; Buvanendran 2010; Gianesello 2012). The longest analgesic scheme opted for tapering pregabalin over two weeks for total knee arthroplasty (Buvanendran 2010). Pregabalin was studied in the setting of three major procedures: spine surgery, knee replacement, and cardiac surgery. A single trial evaluated the effects of pregabalin following thyroidectomy (Kim 2010).  Pregabalin three month postoperative pain outcome  Five clinical trials reported three month follow-up data (Burke 2010; Buvanendran 2010; Gianesello 2012; Kim 2010; Pesonen 2011) and four of them reported the incidence of any pain (Buvanendran 2010; Gianesello 2012; Kim2010; Pesonen 2011). One study was excluded from the meta-analysis due to the lack of reporting on the incidence of pain but rather a continuous pain score (Burke 2010). Only one (Pesonen 2011) of these studies demonstrated a substantial benefit of pregabalin versus placebo leading to an overall significant effect of pregabalin over placebo (OR 0.60, 95% CI 0.39 to 0.93). However, assessment of heterogeneity yielded an I2 of 28.5% (see Figure 6). Only one study evaluated less than 24 hours of pregabalin treatment so we did not perform any subgroup analyses according to treatment duration.  Pregabalin six month postoperative pain outcome  Only one trial continued the follow-up for six months (Buvanendran 2010), in total knee arthroplasty. The authors were contacted and provided the outcome data upon request, which suggested a six month ’any’ pain incidence of 15/113 for pregabalin versus 29/115 for placebo and a ’moderate-to-severe’ pain incidence of 3/113 for pregabalin versus 13/115 for placebo.  Pregabalin 12 month postoperative pain outcome  One trial followed the patients for one year (Gianesello 2012) after major spine surgery. Based on our primary outcome, the study could not demonstrate a significant benefit of pregabalin over placebo.  **Where reported:** Results text.  **Interaction exists based on reported results:** Not reported.  **How determined:** NA.  **Reported importance of the interaction:** Not reported.  **What reported:** Not reported.  **Where reported:** NA  **Reported plausibility of the interaction:** Not reported.  **What reported:** Not reported.  **Where reported:** NA  **Reported confounding possibility:** Not reported.  **What reported:** Not reported.  **Where reported:** NA.  **Mentioned covariate distribution:** Not reported.  **What reported:** Not reported.  **Where reported:** NA. |
| Chaparro 2013 | **Protocol:** Not reported.  **Review methods:** Not reported.  **Review results:** Pain at 3 months, 4 months, 6 months.  **Type:** Outcome.  **Covariate summary:** Time point. | Proportion of participants reporting any pain at the anatomical site of the procedure or pain referred to the surgical site, or both (for example phantom limb pain, shoulder pain referred from the diaphragm etc.), three months or more after the procedure; primary; dichotomous; risk ratio; NT=26; NP=2,865. | **Number of trials:** 14 (comparison 1); 6 (comparison 2); 4 (comparison 3).  **Covariate distribution (across trials):**  NT=12, NP=779 (Comparison 1. Ketamine versus placebo comparisons):  NT=5, NP=384 (Incidence of any pain at 3 months (all studies));  NT=1, NP=69 (Incidence of any pain at 4 months);  NT=8, NP=516 (Incidence of any pain at 6 months (all studies));  NT=2, NP=104 (Incidence of any pain at 12 months).  NT=6, NP=348 (Comparison 2. Gabapentin versus placebo):  NT=5, NP=280 (Incidence of any pain at 3 months (all studies));  NT=2, NP=116 (Incidence of any pain at 6 months).  NT=4, NP=439 (Comparison 3. Pregabalin versus placebo):  NT=4, NP=439 (Incidence of any pain at 3 months follow up (all studies));  NT=1, NP=228 (Incidence of any pain at 6 Months);  NT=1, NP=60 (Incidence of any pain at 12 months follow up).  **Covariate distribution (within trials):** NA. | **Analysis type in review according to glossary 1:** Subgroup analysis.  **Reported results:** Event rates, risk ratio, and CI for trials. Risk ratio, CI, z statistic, and p-value for meta-analytic subtotals.  **Where reported:** Results text and forest plot.  **Method to detect interactions reported in review:** Not reported.  **Reported results from methods to detect interactions:** Not reported.  **Where reported:** NA. | **Reported detected interaction:** Not reported.  **What reported:**  *Ketamine*  Fourteen studies evaluating the perioperative effectiveness of ketamine were identified (Chaparro 2010; Crousier 2008; De Kock 2001; Duale 2009; Dullenkopf 2009; Hayes 2004; Katz 2004a; Malek 2006; Perrin 2009; Remerand 2009; Sen 2009a; Spreng 2010; Suzuki 2006; Sveticic 2008).  The studies are described in Table 1 in terms of the number of perioperative periods where the participants were exposed to ketamine: 1. preincisional loading dose; 2. intraoperative infusion; and 3. postoperative infusion (see Table 1). Three studies, one in thoracotomy (Duale 2009), a second one in amputation (Hayes 2004) and another one in total hip arthroplasty (Remerand 2009), administered ketamine in each of the periods of perioperative treatment; seven clinical trials used an incisional loading dose plus intraoperative infusion (Chaparro 2010; Crousier 2008; De Kock 2001; Katz 2004a; Perrin 2009; Sen 2009a; Spreng 2010); two studies started with an intraoperative infusion and continued the treatment for two (Malek 2006) or three (Suzuki 2006) postoperative days; only two studies limited the administration of ketamine to one of the three potential periods of treatment, one study used two different preincisional loading doses (Dullenkopf 2009) and one study administered ketamine mixed in the patient-controlled analgesia (PCA) device with morphine (Sveticic 2008).  A total cumulative dose was also calculated for each ketamine trial (see Table 1). A cumulative dose > 1 mg/kg was reached by eight out of the 14 aforementioned studies (De Kock 2001;Duale 2009; Hayes 2004; Malek 2006; Perrin 2009; Remerand 2009; Suzuki 2006; Sveticic 2008).  Only two ketamine studies did not report the outcomes of interest for this review (Dullenkopf 2009; Sen 2009).  Ketamine three month postoperative pain outcome  Five studies reported the incidence of pain at three months: one study in patients undergoing thoracotomy (Suzuki 2006); one study in breast surgery (Crousier 2008); two studies in major orthopaedic procedures (Remerand 2009; Sveticic 2008); and one study using (S)-ketamine reported the incidence of pain at three months after inguinal herniorrhaphy (Spreng 2010). A sixth study in thoracotomy patients evaluated pain outcomes at four months after surgery (Duale 2009), which we chose to exclude from this meta-analysis given the time point difference of one month. The overall effectiveness risk ratio showed a non-significant effect for ketamine compared to placebo (odds ratio (OR) 0.74, 95% confidence interval (CI) 0.45 to 1.23). A subgroup analysis based on duration of treatment suggested a significant effect of ketamine compared to placebo (OR 0.37, 95% CI 0.14 to 0.98) for studies evaluating ketamine treatment for longer than 24 hours compared to studies of less than 24 hours of ketamine treatment that, together, resulted in a non-significant effect of ketamine compared to placebo (OR 0.82, 95% CI 0.4 to 1.7).  Ketamine four month postoperative pain outcome  Only one study reported the incidence of pain at four months (Duale 2009). The data reported showed no difference between groups and corresponded with the item ’ongoing pain’ from a validated tool called the Neuropathic Pain Symptom Inventory, reported by the authors.  Ketamine six month postoperative pain outcome  Eight trials reported the incidence of pain at six months: two trials in joint replacement surgery (Perrin 2009; Remerand 2009); one in amputation surgery (Hayes 2004); one in thoracotomy (Suzuki 2006); one inmixed orthopedic procedures (Sveticic 2008); one in rectal carcinoma resection (De Kock 2001); one in breast surgery Malek 2006; and one in radical prostatectomy (Katz 2004a).  Eight clinical trials reported the incidence of pain (any) at six months follow-up (De Kock 2001; Hayes 2004; Katz 2004a; Malek 2006; Perrin 2009; Remerand 2009; Suzuki 2006; Sveticic 2008); the overall effectiveness risk ratio showed a significant difference favouring ketamine compared to placebo (OR 0.50, 95% CI 0.33 to 0.76) (see Figure 4). A subgroup analysis based on duration of treatment failed to support a significant effect of ketamine compared to placebo (OR 0.58, 95% CI 0.31 to 1.09) for studies evaluating ketamine treatment for longer than 24 hours, whereas studies of less than 24 hours of ketamine treatment surprisingly did result in a significant effect of ketamine compared to placebo (OR 0.45, 95% CI 0.26 to 0.78).  Three studies reported the incidence of moderate to severe pain (Hayes 2004;Malek 2006; Remerand 2009); the overall effectiveness risk ratio showed a significant difference favouring ketamine compared to placebo (risk ratio (RR) 0.44, 95% CI 0.20 to 0.93). Two of the three trials administered ketamine during the perioperative periods (Hayes 2004; Remerand 2009); the other trial started an intraoperative infusion that continued for two days after surgery (Malek 2006). The number needed to treat for patients that should receive ketamine to avoid one case of moderate to severe pain at six months was 10.83 (95% CI 5.69 to 109).  Ketamine 12 month postoperative pain outcome  Two studies reported the incidence of pain at 12 months. One of the studies compared high and low doses of intravenous and epidural ketamine versus placebo in patients undergoing rectal carcinoma resection (De Kock 2001); 92/100 patients were successfully contacted and 3/17 patients in the control group reported persistent pain versus none (0/37) in the intravenous ketamine groups; one patient in each epidural ketamine group (2/ 38) reported chronic pain (De Kock 2001). The other study was performed in women undergoing augmentation mammaplasty (Chaparro 2010). Only 50/106 patients could be contacted by a telephone call. None of the participants reported moderate or severe pain, however 3/25 patients in the ketamine group versus 7/25 in the placebo group reported sensory abnormalities such as persistent hypoesthesia and burning-like sensation around the surgical scar (Chaparro 2010). These studies were classified as heterogenous and a meta-analysis was not performed.  *Gabapentin*  We found 10 gabapentin clinical trials with long-term pain outcomes (Amr 2010; Brogly 2008; Clarke 2009; Fassoulaki 2002; Kinney 2011; Moore 2011; Nikolajsen 2006; Sen 2009; Sen 2009a; Ucak 2011). Table 2 describes trial features including surgery, preoperative and postoperative dosages, cumulative dose, as well as duration of gabapentin administration. One study administered gabapentin for 30 days (Nikolajsen 2006) in patients undergoing amputation. Two studies administered gabapentin for 10 days in breast surgery (Amr 2010; Fassoulaki 2002); however one of these trials did not report the incidence of pain but rather the median pain intensity score (Amr 2010) and was excluded from the meta-analysis. A single trial evaluated gabapentin administration in the setting of cardiac surgery (Ucak 2011). Six trials administered a single dose of gabapentin: one for inguinal herniorrhaphy (1200mg single dose) (Sen 2009); one in the setting of abdominal hysterectomy (1200 mg single dose) (Sen 2009a); one arthroplasty study compared the administration of 600 mg of gabapentin in the preoperative period versus postoperative (Clarke 2009); one clinical trial reported the impact of gabapentin 600 mg after thoracotomy (Kinney 2011); another trial evaluated the long-term impact of 1200 mg of gabapentin after thyroidectomy (Brogly 2008); and finally, one trial was conducted in the setting of cesarean section (Moore 2011).  Gabapentin three month postoperative pain outcome  Four clinical trials reported the incidence of any pain at three months (Fassoulaki 2002; Kinney 2011; Moore 2011; Ucak 2011). In one study, which did not report the three month pain incidence in the publication, the authors provided these data upon our request (Nikolajsen 2006). Two studies were not included in the analysis given that they did not report the incidence of pain but rather a continuous pain score (Sen 2009; Sen 2009a). Together, none of the five studies with threemonth data demonstrated a significant difference over placebo (OR 0.97, 95% CI 0.59 to 1.59) (see Figure 5). Subgroup analyses based on duration of treatment failed to show any different results for studies evaluating less than 24 hours of gabapentin treatment compared to those evaluating longer than 24 hours of gabapentin treatment.  Gabapentin six month postoperative pain outcome  Six out of 10 gabapentin trials followed the patients at six months. However, only two studies reported the primary outcome of the review, incidence of any pain (Clarke 2009;Nikolajsen 2006); neither of the two studies could demonstrate a significant difference with gabapentin over placebo. Three studies were not included in the analysis given that they did not report incidence, but a continuous pain score, and had no dichotomous outcomes such as incidence of moderate-severe pain (Amr 2010; Sen 2009; Sen 2009a). One study (Brogly 2008) reported the incidence of pain based on the scoring of the DN2 questionnaire, so the study was excluded from any meta-analysis.  *Pregabalin*  We identified five pregabalin trials with long-term pain outcomes (Burke 2010; Buvanendran 2010; Gianesello 2012; Kim 2010; Pesonen 2011). Table 3 describes the main characteristics of these studies, all of which involved more than one dose of study drug. Study medication was administered for at least two days in 3/5 trials (Buvanendran 2010;Gianesello 2012; Pesonen 2011).The preoperative dose was 150 mg (Kim 2010; Pesonen 2011) or 300 mg (Burke 2010; Buvanendran 2010; Gianesello 2012). The longest analgesic scheme opted for tapering pregabalin over two weeks for total knee arthroplasty (Buvanendran 2010). Pregabalin was studied in the setting of three major procedures: spine surgery, knee replacement, and cardiac surgery. A single trial evaluated the effects of pregabalin following thyroidectomy (Kim 2010).  Pregabalin three month postoperative pain outcome  Five clinical trials reported three month follow-up data (Burke 2010; Buvanendran 2010; Gianesello 2012; Kim 2010; Pesonen 2011) and four of them reported the incidence of any pain (Buvanendran 2010; Gianesello 2012; Kim2010; Pesonen 2011). One study was excluded from the meta-analysis due to the lack of reporting on the incidence of pain but rather a continuous pain score (Burke 2010). Only one (Pesonen 2011) of these studies demonstrated a substantial benefit of pregabalin versus placebo leading to an overall significant effect of pregabalin over placebo (OR 0.60, 95% CI 0.39 to 0.93). However, assessment of heterogeneity yielded an I2 of 28.5% (see Figure 6). Only one study evaluated less than 24 hours of pregabalin treatment so we did not perform any subgroup analyses according to treatment duration.  Pregabalin six month postoperative pain outcome  Only one trial continued the follow-up for six months (Buvanendran 2010), in total knee arthroplasty. The authors were contacted and provided the outcome data upon request, which suggested a six month ’any’ pain incidence of 15/113 for pregabalin versus 29/115 for placebo and a ’moderate-to-severe’ pain incidence of 3/113 for pregabalin versus 13/115 for placebo.  Pregabalin 12 month postoperative pain outcome  One trial followed the patients for one year (Gianesello 2012) after major spine surgery. Based on our primary outcome, the study could not demonstrate a significant benefit of pregabalin over placebo.  **Where reported:** Results text.  **Interaction exists based on reported results:** Not reported.  **How determined:** NA.  **Reported importance of the interaction:** Not reported.  **What reported:** Not reported.  **Where reported:** NA  **Reported plausibility of the interaction:** Not reported.  **What reported:** Not reported.  **Where reported:** NA  **Reported confounding possibility:** Not reported.  **What reported:** Not reported.  **Where reported:** NA.  **Mentioned covariate distribution:** Not reported.  **What reported:** Not reported.  **Where reported:** NA. |
| Cheng 2013 | **Protocol:** ‘We will analysis the following gases for establishing pneumoperitoneum: 1. Carbon dioxide versus nitrous oxide.  2. Carbon dioxide versus helium. 3. Carbon dioxide versus argon. 4. Carbon dioxide versus nitrogen. 5. Carbon dioxide versus any other gas. 6. Any other gas (except carbon dioxide) versus any other gas (except carbon dioxide).’  **Review methods: ‘**We will planned to assess the following gases for establishing pneumoperitoneum: 1. Carbon dioxide versus nitrous oxide. 2. Carbon dioxide versus helium. 3. Carbon dioxide versus argon. 4. Carbon dioxide versus nitrogen. 5. Carbon dioxide versus any other gas. 6. Any other gas (except carbon dioxide) versus any other gas (except carbon dioxide).  **Review results:** ‘Carbon dioxide pneumoperitoneum versus nitrous oxide pneumoperitoneum’; ‘Carbon dioxide pneumoperitoneum versus helium Pneumoperitoneum.’  **Type:** Intervention.  **Covariate summary:** Type of intervention or control. | Complications: (i) Cardiopulmonary complications (e.g., arrhythmia,  ischemia, atelectasis, hypoxemia, pneumothorax, pulmonary edema), (ii) Procedure-related general complications (surgical morbidity); primary; dichotomous; risk ratio; NT=5; NP=268. | **Number of trials:** 5.  **Covariate distribution (across trials):**  NT=2, NP=140 (‘Carbon dioxide pneumoperitoneum versus nitrousoxide pneumoperitoneum’);  NT=3, NP=128 (‘Carbon dioxide pneumoperitoneum versus helium Pneumoperitoneum’).  **Covariate distribution (within trials):** NA. | **Analysis type in review according to glossary 1:** Subgroup analysis.  **Reported results:** Event rates, risk ratio, and CI for trials. Risk ratio, CI, z statistic, and p-value for meta-analytic subtotals.  **Where reported:** Results text and forest plot.  **Method to detect interactions reported in review:** Not reported.  **Reported results from methods to detect interactions:** Not reported.  **Where reported:** NA. | **Reported detected interaction:** Not reported.  **What reported:**  ‘Carbon dioxide pneumoperitoneum versus nitrous oxide pneumoperitoneum:  Only three trials were included in this comparison (Aitola 1998; Lipscomb 1993; Tsereteli 2002).  Primary outcomes:  *Cardiopulmonary complications* (Analysis 1.1):  There were no significant differences in the cardiopulmonary complicationsbetween the groups (RR 0.50; 95% CI 0.10 to 2.61; P=0.41) (Figure 4).  Carbon dioxide pneumoperitoneum versus helium pneumoperitoneum:  Four trials were included in this comparison (Bongard 1993; Naude 1996; Neuhaus 2001; O’Boyle 2002).  Primary outcomes:  *Cardiopulmonary complications* (Analysis 2.1):  Therewere no significant differences in the cardiopulmonary complications between the groups (RR 0.69; 95% CI 0.16 to 2.88; P=0.61) (heterogeneity: I2=0%; P=0.51) (Figure 5).  **Where reported:** Results text.  **Interaction exists based on reported results:** Not reported.  **How determined:** NA.  **Reported importance of the interaction:** Not reported.  **What reported:** Not reported.  **Where reported:** NA  **Reported plausibility of the interaction:** Not reported.  **What reported:** Not reported.  **Where reported:** NA  **Reported confounding possibility:** Not reported.  **What reported:** Not reported.  **Where reported:** NA.  **Mentioned covariate distribution:** Not reported.  **What reported:** Not reported.  **Where reported:** NA. |
| Cruciani 2013 | **Protocol:** ‘The adequacy of allocation concealment’.  **Review methods: ‘**The adequacy of allocation concealment’.  **Review results: ‘**The adequacy of allocation concealment’.  **Type:** Methodological.  **Covariate summary:** Allocation concealment. | Proportion of patients discontinuing or switching antiretroviral therapy due to virologic failure; primary; dichotomous; risk ratio; NT=8; NP=1,587. | **Number of trials**: Not reported.  **Covariate distribution (across trials):**  NT=not reported, NP=not reported (‘unclear);  NT=not reported, NP=not reported (‘low).  (Not reported whether the sensivity analysis was grouped by comparisons so I have not guessitmated the numbers here).  **Covariate distribution (within trials):** NA. | **Analysis type in review according to glossary 1:** Subgroup analysis.  **Reported results:** Text.  **Where reported:** Results text.  **Method to detect interactions reported in review:** Not reported.  **Reported results from methods to detect interactions:** Not reported.  **Where reported:** NA. | **Reported detected interaction:** No interaction.  **What reported: ‘Sensitivity analysis**  We conducted a sensitivity analysis to determine howthe adequacy of allocation concealment affected the results of the review. We made a judgment of ’low risk of bias’ for this domain in 4 studies with central randomizations (Bonjoch 2005; Katlama 2003; NEFA Study; Opravil 2002) and of ’unclear risk’ of bias in the remaining 4 studies (Clumeck 2001; Maggiolo 2003; Keiser 2005; Sprenger 2010). Subgroup analyses stratifying the trials according to allocation concealment did not demonstrate significant differences on pooled estimates for the outcomes analysed between trials with adequate and with unclear allocation concealment.**’**  **Where reported:** Results text.  **Interaction exists based on reported results**: Not reported.  **How determined:** NA.  **Reported importance of the interaction:** Not reported.  **What reported:** Not reported.  **Where reported:** NA  **Reported plausibility of the interaction:** Not reported.  **What reported:** Not reported.  **Where reported:** NA  **Reported confounding possibility:** Not reported.  **What reported:** Not reported.  **Where reported:** NA.  **Mentioned covariate distribution:** Not reported.  **What reported:** Not reported.  **Where reported:** NA. |
| Cruciani 2013 | **Protocol: ‘**The type of drugs in the simplification regimen (e. g. nevirapine, efavirenz)’.  **Review methods:** ‘Type of drugs in the control group (e. g. nevirapine, efavirenz)’.  **Review results:** ‘Type NNRTI drug in the simplication regimen’ (in text). ‘ABC vs Nevirapine’ and ‘ABC vs Efavirenz’ (in plot).  **Type:** Intervention.  **Covariate summary:** Type of intervention or control. | Proportion of patients discontinuing or switching antiretroviral therapy due to virologic failure; primary; dichotomous; risk ratio; NT=8; NP=1,587. | **Number of trials:** 3.  **Covariate distribution (across trials):**  NT=2, NP=364 (ABC vs Nevirapine);  NT=2, NP=369 (ABC vs Efavirenz).  **Covariate distribution (within trials):** NA. | **Analysis type in review according to glossary 1:** Subgroup analysis.  **Reported results:** Event rates, risk ratio, and CI for trials. Risk ratio, CI, Z statistic, and p-value for meta-analytic subtotals.  **Where reported:** Results text and forest plot.  **Method to detect interactions reported in review:** Not reported.  **Reported results from methods to detect interactions:** Test for subgroup differences (Chi-square statistic, p-value, and I square statistic).  **Where reported:** Forest plot. | **Reported detected interaction:** Not reported.  **What reported:** We performed subgroup analyses according to the type NNRTI drugs in the simplification regimen (Analysis 2.1; Analysis 2.2; Analysis 2.3; additional figg.11-13). Three studies (Maggiolo 2003; NEFA Study; Bonjoch 2005) reported separately results for nevirapine and/or efavirenz. The results of the comparison between triple nucleoside combination and nevirapine or efavirenz containing ART for the outcomes overall failure, discontinuation for adverse events and virologic failure are shown on figures 9- 11. There were no significant difference between the participants on triple nucleoside combination and controls, with the exception of a higher number of virologic failure on ABC compared to efavirenz (RR 2.76, 95 % CI, 1.21-6.31).  **Where reported:** Results text.  **Interaction exists based on reported results:** Yes.  **How determined:** Test for subgroup differences: Chi2= 3.56, df= 1 (P= 0.06), I2=72%  **Reported importance of the interaction:** Not reported.  **What reported:** Not reported.  **Where reported:** NA  **Reported plausibility of the interaction:** Not reported.  **What reported:** Not reported.  **Where reported:** NA  **Reported confounding possibility:** Not reported.  **What reported:** Not reported.  **Where reported:** NA.  **Mentioned covariate distribution:** Not reported.  **What reported:** Not reported.  **Where reported:** NA. |
| Cruciani 2013 | **Protocol:** ‘Timing of simplification to nucleoside therapy (e. g. simplification in patients after a first antiretroviral PI-containing regimen and in antiretroviral-experienced participants).’  **Review methods: ‘**Timing of simplification to nucleoside therapy (e. g. simplification in patients after a first antiretroviral PI-containing regimen and in antiretroviral-experienced participants).’  **Review results: ‘**Timing of simplification to nucleoside therapy’.  **Type:** Intervention.  **Covariate summary:** Timing of intervention. | Proportion of patients discontinuing or switching antiretroviral therapy due to virologic failure; primary; dichotomous; risk ratio; NT=8; NP=1,587. | **Number of trials**: Not reported.  **Covariate distribution (across trials):**  NT=not reported, NP=not reported (naives patients with simplification after a first-line antiretroviral PI-containing regimen);  NT=not reported, NP=not reported (antiretroviral-experienced participants).  (Not reported whether the sensivity analysis was grouped by comparisons so I have not guessitmated the numbers here).  **Covariate distribution (within trials):** NA. | **Analysis type in review according to glossary 1:** Subgroup analysis.  **Reported results:** risk ratio, CI and p-value for meta-analytic subtotal (one subgroup).  **Where reported:** Results text.  **Method to detect interactions reported in review:** Not reported.  **Reported results from methods to detect interactions:** Not reported.  **Where reported:** NA. | **Reported detected interaction:** No interaction.  **What reported: ‘**A subgroup analysis according to timing of simplification to nucleoside  therapy was also performed, and studies in naives patients with simplification after a first-line antiretroviral PI-containing regimen (Bonjoch 2005; Sprenger 2010) analysed separately from those in antiretroviral-experienced participants (data not shown). This latter group included patients with sub-optimal previous treatment (e.g.,NRTImono- or dual therapy).Of note, subgroup analyses of studies with simplification after first-line therapy did not affect significantly the results of the meta-analysis, though for the outcome virologic outcome the meta-analytical estimates and the test for overall effect(RR 0.84; 95 % CI, 0.35/2.07; p=0.7) were not longer closed to the level of significance as in the overall comparison(1.3**;** 0.95/2.02; p=0.09)’  **Where reported:** Results text.  **Interaction exists based on reported results:** Not reported**.**.  **How determined:** NA.  **Reported importance of the interaction:** Not reported.  **What reported:** Not reported.  **Where reported:** NA  **Reported plausibility of the interaction:** Not reported.  **What reported:** Not reported.  **Where reported:** NA  **Reported confounding possibility:** Not reported.  **What reported:** Not reported.  **Where reported:** NA.  **Mentioned covariate distribution:** Not reported.  **What reported:** Not reported.  **Where reported:** NA. |
| Cruciani 2013 | **Protocol:** Not reported.  **Review methods:** Not reported.  **Review results:** ‘ABC vs PI’; ‘ABC vs NNRTI’**.**  **Type:** Intervention.  **Covariate summary:** Type of intervention or control. | Proportion of patients discontinuing or switching antiretroviral therapy due to virologic failure; primary; dichotomous; risk ratio; NT=8; NP=1,587. | **Number of trials:** 8.  **Covariate distribution (across trials):**  NT=6, NP=899 (ABC vs PI);  NT=3, NP=688 (ABC vs NNRTI).  **Covariate distribution (within trials):** NA. | **Analysis type in review according to glossary 1:** Subgroup analysis.  **Reported results:** Event rates, risk ratio, and CI for trials. Risk ratio, CI, Z statistic, and p-value for meta-analytic subtotals and totals.  **Where reported:** Results text and forest plot.  **Method to detect interactions reported in review:** Not reported.  **Reported results from methods to detect interactions:** Test for subgroup differences (Chi-square statistic, p-value, and I square statistic).  **Where reported:** Forest plot. | **Reported detected interaction:** Not reported.  **What reported: ‘**Eight trials with 1,587 participants reported on virologic failure. Triple nucleoside combination (689 participants) was compared to PI continuation (461 participants) or to NNRTI simplification (437 participants). A random effect model was applied (*I*2=18 %). Overall, there was no significant difference between the participants on triple nucleoside combination and controls (RR 1.39, 95% CI 0.95 to 2.02), either PI-based (RR 1.49, 95% CI  0.72 to 3.08) or NNRTI based (RR 1.32, 95% CI 0.89 to 1.97), though the test for overall effect (p=0.09) was closed to the level of significance, thus suggesting a weak evidence of higher incidence of virologic failure in the 3NRTI group compared to controls’  **Where reported:** Results text.  **Interaction exists based on reported results:** No.  **How determined:** Test for subgroup differences: Chi2= 0.08, df= 1 (P= 0.78), I2=0.0%  **Reported importance of the interaction:** Not reported.  **What reported:** Not reported.  **Where reported:** NA  **Reported plausibility of the interaction:** Not reported.  **What reported:** Not reported.  **Where reported:** NA  **Reported confounding possibility:** Not reported.  **What reported:** Not reported.  **Where reported:** NA.  **Mentioned covariate distribution:** Not reported.  **What reported:** Not reported.  **Where reported:** NA. |
| Deare 2013 | **Protocol:** Not reported.  **Review methods: ‘**Manual acupuncture versus electro-acupuncture’. We judged the following to be three covariates: ‘we planned subgroup analyses to assess the effect of different types of acupuncture: 1) manual acupuncture versus electro-acupuncture; 2) shallow needling versus deep needling; 3) different forms of sham/ placebo acupuncture.’  **Review results:** ‘Electro-acupuncture’; ‘manual acupuncture’.  **Type:** Intervention.  **Covariate summary:** Type of intervention or control. | Pain; main; continuous; mean difference and standardised mean difference; NT=9; NP=473. | **Number of trials:** 6 (month 1); 2 (month 7).  **Covariate distribution (across trials):**  NT=6, NP=286 (Acupuncture versus placebo or sham acupuncture).  NT=6, NP=286 (up to 1 month after treatment):  NT=2, NP=104 (electro-acupuncture );  NT=4, NP=182 (manual acupuncture).  NT=6, NP=286 (Acupuncture versus placebo or sham acupuncture).  NT=2, NP=145 (up to 7 months after treatment):  NT=1, NP=49 (electro-acupuncture );  NT=1, NP=96 (manual acupuncture).  **Covariate distribution (within trials):** NA. | **Analysis type in review according to glossary 1:** Subgroup analysis.  **Reported results:** Mean, SD and N by group for trials. Standardised mean difference and CI for trials. Standardised mean difference, CI, Z statistic, and p-value for meta-analytic total and subtotal.  **Where reported:** Results text and forest plot.  **Method to detect interactions reported in review:** Not reported.  **Reported results from methods to detect interactions4:** Test for subgroup differences (Chi-square statistic, p-value, and I square statistic).  **Where reported:** Forest plot and text. | **Reported detected interaction: I**nteraction.  **What reported:** Interaction at 1 month; no interaction at 7 months.  Pain subgroup analysis (electro- versus manual acupuncture):  Pooled subgroup analysis of two electro-acupuncture studies, including 104 participants (Deluze 1992; Martin 2006), indicated that real electro-acupuncture was statistically significantly better than sham electro-acupuncture in reducing pain (SMD -0.63; 95% CI -1.02 to -0.23, P = 0.002, about 13 points on a 100- point scale) (Analysis 2.1) up to one month after treatment, with low heterogeneity (I2 = 0%, P = 0.72). Subgroup analysis of four manual acupuncture studies, including 182 participants (Assefi 2005; Harris 2005; Harris 2008; Harris 2009) showed no group difference between real and sham manual acupuncture in reducing pain (SMD 0.14; 95% CI -0.17 to 0.45, P = 0.37, 2.8 points on a 100-point scale) (Analysis 2.1), with no heterogeneity (I2 = 0%, P = 0.57). There was a statistically significant subgroup difference between electro- and manual acupuncture (Chi2 = 8.94, P = 0.003).  Long-term effects of acupuncture:  Two studies (Assefi 2005; Martin 2006) measured long-term effects of acupuncture for up to seven months after the end of the treatment. There was no difference between real and sham acupuncture on any outcome measures, including pain (Analysis 2.2), global well-being (Analysis 2.6), sleep (Analysis 2.8), fatigue (Analysis 2.10), stiffness (Analysis 2.12) and mental well-being (Analysis 2.15). Subgroup comparison indicated that electroacupuncture was not statistically significantly better than manual acupuncture at improving any of the outcomes at seven months after treatment.  **Where reported:** Results text.  **Interaction exists based on reported results:** Yes for 1 month; no for 7 months.  **How determined:**  1 month: Test for subgroup differences: Chi2= 8.94, df= 1 (P= 0.00), I2=89%  7 month: Test for subgroup differences: Chi2= 1.25, df= 1 (P= 0.26), I2=20%  **Reported importance of the interaction:** Not reported.  **What reported:** Not reported.  **Where reported:** NA  **Reported plausibility of the interaction:** Not reported.  **What reported:** Not reported.  **Where reported:** NA  **Reported confounding possibility:** Not reported.  **What reported:** Not reported.  **Where reported:** NA.  **Mentioned covariate distribution:** Not reported.  **What reported:** Not reported.  **Where reported:** NA. |
| Deare 2013 | **Protocol:** Not reported.  **Review methods: ‘**Different forms of sham/placebo acupuncture. We judged the following to be three covariates: ‘We planned subgroup analyses to assess the effect of different types of acupuncture: 1) manual acupuncture versus electro-acupuncture; 2) shallow needling versus deep needling; 3) different forms of sham/ placebo acupuncture.’  **Review results:** Not analysed.  **Type:** Intervention.  **Covariate summary:** Type of intervention or control. | Pain; main; continuous; mean difference and standardised mean difference; NT=9; NP=473. | **Number of trials:** 6.  **Covariate distribution (across trials):**  NT=3, NP=170 (Sham breaking skin);  NT=4, NP=116 (Sham not breaking skin).  **Covariate distribution (within trials):** NA. | **Analysis type in review according to glossary 1:** Subgroup analysis.  **Reported results:** Mean, SD and N by group for trials. Standardised mean difference and CI for trials. Standardised mean difference, CI, z statistic and p-value for meta-analytic subtotals.  **Where reported:** Results text and forest plot.  **Method to detect interactions reported in review:** Not reported.  **Reported results from methods to detect interactions:** Test for subgroup differences (Chi-square statistic, p-value, and I square statistic).  **Where reported:** Forest plot. | **Reported detected interaction:** No interaction.  **What reported:**  ‘Sham non-invasive (not breaking skin) acupuncture versus sham invasive (breaking skin) acupuncture  Four studies (Assefi 2005;Harris 2008;Harris 2009;Martin 2006) with 116 participants using non-invasive sham acupuncture were compared with three studies (Assefi 2005; Harris 2005; Deluze 1992) with 170 participants using invasive sham interventions. There was no statistically significant difference between the two subgroups on pain rating (Chi2 = 0.40, P = 0.53, Analysis 2.3).’  **Where reported:** Results text.  **Interaction exists based on reported results**: No.  **How determined:** Test for subgroup differences: Chi2= 0.40, df= 1 (P= 0.53), I2=0.0%  **Reported importance of the interaction:** Not reported.  **Reported importance of the interaction:** Not reported.  **What reported:** Not reported.  **Where reported:** NA  **Reported plausibility of the interaction:** Not reported.  **What reported:** Not reported.  **Where reported:** NA  **Reported confounding possibility:** Not reported.  **What reported:** Not reported.  **Where reported:** NA.  **Mentioned covariate distribution:** Yes.  **What reported:** as aabove.  **Where reported:** Results text. |
| Deare 2013 | **Protocol:** ‘The following comparisons will be made: 1. Real acupuncture versus non-acupuncture treatment (e. g. wait list or drug therapy). 2. Real acupuncture versus placebo or sham acupuncture. 3. Real acupuncture versus standard or usual care (e. g. physiotherapy  or exercise). 4. A particular style of acupuncture versus another.’  **Review methods: ‘**We examined the following comparisons: 1. Acupuncture versus no acupuncture (e. g. wait list) 2. Acupuncture versus placebo or sham acupuncture 3. Acupuncture versus standard/usual care (e. g. cognitive behavioural therapy (CBT) and/or exercise and/or pharmacotherapy) 4. Acupuncture as an adjunct therapy to standard/usual care (evaluating additional effect) 5. A particular style of acupuncture versus another (e. g. deep needling with stimulation versus deep needling without stimulation)’.  **Review results:** Comparison 1. Acupuncture versus non-acupuncture treatment; Comparison 2. Acupuncture versus placebo or sham acupuncture; Comparison 3. Acupuncture versus medication; Comparison 4. Acupuncture as an adjunct therapy; Comparison 5. Deep needling with stimulation (T/S) versus deep; needling without stimulation (T/O).  **Type:** Intervention.  **Covariate summary:** Type of intervention or control. | Pain; main; continuous; mean difference and standardised mean difference; NT=9; NP=473. | **Number of trials:** 9.  **Covariate distribution (across trials):**  NT=1, NP=13 (Acupuncture versus non-acupuncture treatment);  NT=6, NP=286 (Acupuncture versus placebo or sham acupuncture);  NT=1, NP=38 (Acupuncture versus medication);  NT=1, NP=58 (Acupuncture as an adjunct therapy);  NT=1, NP=41 (Deep needling with stimulation (T/S) versus deep needling without stimulation (T/O)).  **Covariate distribution (within trials):** NA. | **Analysis type in review according to glossary 1:** Subgroup analysis.  **Reported results:** Mean, SD and N by group for trials. Mean difference or standardised mean difference and CI for trials. Mean difference or standardised mean difference, CI, Z statistic, and p-value for meta-analytic subtotals.  **Where reported:** Results text and forest plot.  **Method to detect interactions reported in review:** Not reported.  **Reported results from methods to detect interactions:** Not reported.  **Where reported:** NA. | **Reported detected interaction:** Not reported.  **What reported:**  ‘1) Real acupuncture versus non-acupuncture treatment  One study in this category (Itoh 2010) included 13 participants and compared electro-acupuncture plus trigger point acupuncture with no acupuncture treatment. This was a cross-over study, and we used data before cross-over for analysis, that is after five sessions of treatment at the end of week five of a 10-week treatment programme.  Main outcome measure 1: Pain  Pain severity was measured using a VAS (100 mm). It showed a statistically significant reduction in pain for those treated with real acupuncture compared with no acupuncture at the end of treatment (mean difference (MD) -22.40 points on a 100-point scale; 95% confidence interval (CI) -40.98 to -3.82, P = 0.02), favouring acupuncture (Analysis 1.1).  2) Real acupuncture versus placebo or sham acupuncture  Main outcome measure 1: Pain up to one month after treatment  Six studies totaling 286 participants were in this category (Assefi 2005; Deluze 1992; Harris 2005; Harris 2008; Harris 2009; Martin 2006).Measurement tools used included VAS (0 to 10 cm and 0 to 100 mm), numerical pain rating scale (NRS), Multidimensional Pain Inventory (MPI) and McGill Pain Questionnaire (SF-MPQ). Pooled analysis of the six studies showed no statistically significant difference between the groups in reducing pain (standardised mean difference (SMD) -0.14; 95% CI -0.53 to 0.25, P = 0.48; corresponding to a reduction of 2.8 points on a 100-point scale) (Analysis 2.1).Moderate heterogeneity was found (I2 = 54%, P = 0.05) and is likely due to the two forms of acupuncture, electro- and manual, employed in the different studies.  Long-term effects of acupuncture  Two studies (Assefi 2005; Martin 2006) measured long-term effects of acupuncture for up to seven months after the end of the treatment. There was no difference between real and sham acupuncture on any outcome measures, including pain (Analysis 2.2), global well-being (Analysis 2.6), sleep (Analysis 2.8), fatigue (Analysis 2.10), stiffness (Analysis 2.12) and mental well-being (Analysis 2.15). Subgroup comparison indicated that electroacupuncture was not statistically significantly better than manual acupuncture at improving any of the outcomes at seven months after treatment.  3) Real acupuncture versus standard or usual care (medication)  One study in this category (Guo 2005) included 38 participants and compared manual acupuncture withWestern medicine (amitriptyline).  Main outcome measure 1: Pain at up to one month after treatment  Pain severity was measured using a VAS. It showed a statistically significant group difference favouring cupuncture (MD -17.30 points on a 100-point scale; 95% CI -24.13 to -10.47, P <0.00001) (Analysis 3.1).  4) Real acupuncture as an adjunct therapy  One study in this category (Targino 2008) with 58 participants compared manual acupuncture plus standard therapy, which included tricyclic antidepressants and exercise, with standard therapy alone.  Main outcome measure 1: Pain at up to one month after treatment  Pain severity was measured using a VAS. It showed a statistically significant group difference favouring acupuncture (MD -3.00 points on a 10-point scale; 95% CI -3.90 to -2.10, P < 0.00001) (Analysis 4.1).  Long-term effect of acupuncture (follow-up at six months)  At the six-month follow-up, the acupuncture as an adjunct therapy group continued to be better than the standard therapy alone group for tender points (MD-2.00 number of tender points; 95% CI -3.51 to -0.49, P = 0.009) (Analysis 4.5) and mean pressure pain threshold (MD 0.60 kg/cm2; 95% CI 0.26 to 0.94, P =0.0005) (Analysis 4.7) but not pain (MD -0.50 points on a 10-point scale; 95% CI -1.49 to 0.49, P = 0.37) (Analysis 4.2).  5) A particular style of acupuncture versus another (deep invasive needling with stimulation (deqi) (T/S) versus deep invasive needling without stimulation (T/O))  Two different styles of acupuncture, deep needling using manual acupuncture on the point with stimulation to achieve *deqi* as in traditional Chinese acupuncture (T/S) versus deep needling on the point without stimulation (T/O), were compared in one study of 41 participants (Harris 2005).  Main outcome measure 1: Pain at the end of the treatment  Pain was measured using the NRS. It showed no statistically significant difference between the two interventions (MD 0.30 on a 10-point scale; 95% CI -18.34 to 18.94, P = 0.97) (Analysis 5.1).’  **Where reported:** Results text.  **Interaction exists based on reported results**: Not reported.  **How determined:** NA.  **Reported importance of the interaction:** Not reported.  **What reported:** Not reported.  **Where reported:** NA  **Reported plausibility of the interaction:** Not reported.  **What reported:** Not reported.  **Where reported:** NA  **Reported confounding possibility:** Not reported.  **What reported:** Not reported.  **Where reported:** NA.  **Mentioned covariate distribution:** Yes.  **What reported:** as above.  **Where reported:** Results text. |
| Deare 2013 | **Protocol:** Not reported.  **Review methods:** Not reported.  **Review results:** Time point: one month after treatment; seven months after treatment.  **Type:** Outcome.  **Covariate summary:** Time point. | Pain; main; continuous; mean difference and standardised mean difference; NT=9; NP=473. | **Number of trials:** 1 (comparison 1); 6 (comparison 2); 1 (comparison 3); 1 (comparison 4); 1 (comparison 5).  **Covariate distribution (across trials):**  NT=1, NP=13 (Comparison 1. Acupuncture versus non-acupuncture treatment):  NT=1, NP=13 (up to 1 month after treatment).  NT=6, NP=286 (Comparison 2. Acupuncture versus placebo or sham acupuncture):  NT=6, NP=286 (up to 1 month after treatment);  NT=2, NP=145 (up to 7 months after treatment).  NT=1, NP=38 (Comparison 3. Acupuncture versus medication):  NT=1, NP=38 (up to 1 month after treatment).  NT=1, NP=58 (Comparison 4. Acupuncture as an adjunct therapy):  NT=1, NP=58 (up to 1 month after treatment);  NT=1, NP=58 (up to 7 months after treatment).  NT=1, NP=41 (Comparison 5. Deep needling with stimulation (T/S) versus deep needling without stimulation (T/O)):  NT=1, NP=41 (up to 1 month after treatment).  **Covariate distribution (within trials):** NA. | **Analysis type in review according to glossary 1:** Subgroup analysis.  **Reported results:** Mean, SD and N by group for trials. Mean difference or standardised mean difference and CI for trials. Mean difference or standardised mean difference, CI, z statistic and p-value for meta-analytic subtotals.  **Where reported:** Results text and forest plot.  **Method to detect interactions reported in review:** Not reported.  **Reported results from methods to detect interactions:** Not reported.  **Where reported:** NA. | **Reported detected interaction:** Not reported.  **What reported:**  ‘1) Real acupuncture versus non-acupuncture treatment  One study in this category (Itoh 2010) included 13 participants and compared electro-acupuncture plus trigger point acupuncture with no acupuncture treatment. This was a cross-over study, and we used data before cross-over for analysis, that is after five sessions of treatment at the end of week five of a 10-week treatment programme.  Main outcome measure 1: Pain  Pain severity was measured using a VAS (100 mm). It showed a statistically significant reduction in pain for those treated with real acupuncture compared with no acupuncture at the end of treatment (mean difference (MD) -22.40 points on a 100-point scale; 95% confidence interval (CI) -40.98 to -3.82, P = 0.02), favouring acupuncture (Analysis 1.1).  2) Real acupuncture versus placebo or sham acupuncture  Main outcome measure 1: Pain up to one month after treatment  Six studies totaling 286 participants were in this category (Assefi 2005; Deluze 1992; Harris 2005; Harris 2008; Harris 2009; Martin 2006).Measurement tools used included VAS (0 to 10 cm and 0 to 100 mm), numerical pain rating scale (NRS), Multidimensional Pain Inventory (MPI) and McGill Pain Questionnaire (SF-MPQ). Pooled analysis of the six studies showed no statistically significant difference between the groups in reducing pain (standardised mean difference (SMD) -0.14; 95% CI -0.53 to 0.25, P = 0.48; corresponding to a reduction of 2.8 points on a 100-point scale) (Analysis 2.1).Moderate heterogeneitywas found (I2 = 54%, P = 0.05) and is likely due to the two forms of acupuncture, electro- and manual, employed in the different studies.  Long-term effects of acupuncture  Two studies (Assefi 2005; Martin 2006) measured long-term effects of acupuncture for up to seven months after the end of the treatment. There was no difference between real and sham acupuncture on any outcome measures, including pain (Analysis 2.2), global well-being (Analysis 2.6), sleep (Analysis 2.8), fatigue (Analysis 2.10), stiffness (Analysis 2.12) and mental well-being (Analysis 2.15). Subgroup comparison indicated that electroacupuncture was not statistically significantly better than manual acupuncture at improving any of the outcomes at seven months after treatment.  3) Real acupuncture versus standard or usual care (medication)  One study in this category (Guo 2005) included 38 participants and compared manual acupuncture withWestern medicine (amitriptyline).  Main outcome measure 1: Pain at up to one month after treatment  Pain severity was measured using a VAS. It showed a statistically significant group difference favouring cupuncture (MD -17.30 points on a 100-point scale; 95% CI -24.13 to -10.47, P <0.00001) (Analysis 3.1).  4) Real acupuncture as an adjunct therapy  One study in this category (Targino 2008) with 58 participants compared manual acupuncture plus standard therapy, which included tricyclic antidepressants and exercise, with standard therapy alone.  Main outcome measure 1: Pain at up to one month after treatment  Pain severity was measured using a VAS. It showed a statistically significant group difference favouring acupuncture (MD -3.00 points on a 10-point scale; 95% CI -3.90 to -2.10, P < 0.00001) (Analysis 4.1).  Long-term effect of acupuncture (follow-up at six months)  At the six-month follow-up, the acupuncture as an adjunct therapy group continued to be better than the standard therapy alone group for tender points (MD-2.00 number of tender points; 95% CI -3.51 to -0.49, P = 0.009) (Analysis 4.5) and mean pressure pain threshold (MD 0.60 kg/cm2; 95% CI 0.26 to 0.94, P =0.0005) (Analysis 4.7) but not pain (MD -0.50 points on a 10-point scale; 95% CI -1.49 to 0.49, P = 0.37) (Analysis 4.2).  5) A particular style of acupuncture versus another (deep invasive needling with stimulation (deqi) (T/S) versus deep invasive needling without stimulation (T/O))  Two different styles of acupuncture, deep needling using manual acupuncture on the point with stimulation to achieve *deqi* as in traditional Chinese acupuncture (T/S) versus deep needling on the point without stimulation (T/O), were compared in one study of 41 participants (Harris 2005).  Main outcome measure 1: Pain at the end of the treatment  Pain was measured using the NRS. It showed no statistically significant difference between the two interventions (MD 0.30 on a 10-point scale; 95% CI -18.34 to 18.94, P = 0.97) (Analysis 5.1).’  **Where reported:** Results text.  **Interaction exists based on reported results**: Not reported.  **How determined:** NA.  **Reported importance of the interaction:** Not reported.  **What reported:** Not reported.  **Where reported:** NA  **Reported plausibility of the interaction:** Not reported.  **What reported:** Not reported.  **Where reported:** NA  **Reported confounding possibility:** Not reported.  **What reported:** Not reported.  **Where reported:** NA.  **Mentioned covariate distribution:** Yes.  **What reported:** as above.  **Where reported:** Results text. |
| Freak-Poli 2013 | **Protocol:** ‘We will analyse the following categories separately: Studies comparing pedometer interventions to no intervention, similar components without a pedometer, larger scale health promotion interventions and other active interventions.’  **Review methods:** ‘We aimed to analyse the following categories separately: Studies comparing pedometer interventions to no intervention, similar components without a pedometer, larger scale health promotion interventions and other active interventions.’  **Review results:** ‘Pedometer programme versus ‘no intervention’ control’; ‘Pedometer programme versus alternative programme without pedometer’.  **Type:** Intervention.  **Covariate summary:** Type of intervention or control. | Physical activity; primary; continuous; mean difference; NT=4; NP=1,809. | **Number of trials:** 4.  **Covariate distribution (across trials):**  NT=3, NP=1,653 (‘Pedometer programme versus ‘no intervention’ control’);  NT=1, NP=156 (‘Pedometer programme versus alternative programme without pedometer’).  **Covariate distribution (within trials):** NA. | **Analysis type in review according to glossary 1:** Subgroup analysis.  **Reported results:** Mean difference, CI from trials.  **Where reported:** Results text.  **Method to detect interactions reported in review:** Not reported.  **Reported results from methods to detect interactions:** Not reported.  **Where reported:** NA. | **Reported detected interaction:** Not reported.  **What reported:**  Pedometer programme versus ’no intervention’  control  Primary outcome: Physical activity  The three studies in this group (Dishman 2009;Maruyama 2010; Morgan 2011) found inconsistent results for physical activity.We could not combine the results in a single meta-analysis because the studies differed too greatly in how they measured physical activity (see the ’Characteristics of included studies’ table for details of the measures used). Wheremultiplemeasures of physical activity were available from the same study, we report the measures which were most direct and comparable.  • Maruyama 2010 measured the change in the number of pedometer steps recorded by participants during the study, and found no significant difference between the pedometer programme and the control (mean difference (MD) 649 steps per day over one week, 95% confidence interval (CI) -630.75 to 1928.75).  • Dishman 2009 measured walking, moderate and vigorous physical activity using ’metabolic equivalent of task’ (METs) units, and found that, on average, those allocated to the pedometer programme were more vigorously active (MD 8.80 METs per week, 95% CI 3.95 to 13.65), more moderately active (2.70 METs, 95% CI 0.14 to 5.26) and walked more (3.60 METs, 95% CI 0.74 to 6.46) at the end of the programme compared to the control group. We re-analysed the data in this study to correctly account for clustering (Cochrane Handbook) using outcome-specific intraclass correlation coefficients (ICCs) provided by the study authors.  • Morgan 2011 measured overall physical activity during leisure time using METs, and found no statistically significant difference between the pedometer programme and the control (MD 0.30 METs, 95% CI -0.04 to 0.64). We obtained correct sample sizes through correspondence with the authors. These results are not necessarily inconsistent with each other, but we cannot conclusively say that each study has demonstrated a positive effect of pedometer programmes.  Pedometer programme versus alternative programme without pedometer  Talbot 2011 compared a multi-component programme including a pedometer with an alternative physical activity programme including similar components without a pedometer. At baseline, the control group performed higher levels of moderate and very high intensity physical activity, making it difficult to interpret any observed differences in physical activity. For example, it has been suggested that a healthier, more motivated group may be more likely to attain the programme goal, leading the study to overestimate the health benefits of the programme in this group (Freak-Poli 2011). However, a group that was healthier and more active at baseline would also have less room to improve, thereby leading the study to underestimate the general health benefits of participation.  Primary outcome: Physical activity Talbot 2011 found positive improvements in hard physical activity associated with the alternative physical activity programme that did not include a pedometer. The authors observed no differences between the pedometer programme and the alternative physical activity programme for total physical activity, very hard physical activity, moderate physical activity or pedometer steps immediately at the end of the programme. However, when baseline imbalances are considered, the pedometer programme may favour improvements in moderate activity and number of steps counted by the pedometer, although interpreting these results is not as straightforward as subtracting baseline from postintervention values, for the reasons outlined above.  • The authors found no statistically significant difference between the pedometer programme and the alternative physical activity programme in the number of steps counted by the pedometer (MD 224.00 steps per day over one week, 95% CI - 954.79 to 1402.79) at the end of the programme. However, at baseline the alternative programme group undertook 885 more steps per day over the week than the pedometer programme group.  • The authors found no statistically significant difference between the pedometer programme and the alternative physical activity programme in physical activity measured either as total (MD -25.70 kcal/kg/wk or METs, 95% CI -54.72 to 3.32; baseline MD -21.2), very hard (MD -7.70 METs, 95% CI - 18.40 to 3.00; baseline MD -8.4) or moderate physical activity (MD 1.40 METs, 95% CI -15.81 to 18.61; baseline MD -20.3) at the end of the programme. It is important to note that at baseline, those allocated to the alternative physical activity programme were undertaking 20.3 METs more moderate activity per week than those allocated to the pedometer programme*.* Hence, the pedometer programme might have increased moderate activity had there not been such a large baseline imbalance.  • On average, those allocated to the pedometer programme undertook 19.40 METs (95% CI 3.59 to 35.21) less hard physical activity at the end of the programme than those in the alternative physical activity programme. However at baseline, those in the pedometer programme were undertaking 11.99 hard activity METs more than those in the alternative programme. Hence, the magnitude of this result might have been smaller without such a baseline imbalance.  **Where reported:** Results text.  **Interaction exists based on reported results:** Not reported.  **How determined:** NA.  **Reported importance of the interaction:** Not reported.  **What reported:** Not reported.  **Where reported:** NA  **Reported plausibility of the interaction:** Not reported.  **What reported:** Not reported.  **Where reported:** NA  **Reported confounding possibility:** Not reported.  **What reported:** Not reported.  **Where reported:** NA.  **Mentioned covariate distribution:** Yes.  **What reported:** as above.  **Where reported:** Results text. |
| Gillies 2012 | **Protocol:** ‘Data will be analysed as short-term (up to one month following completion of the therapy), medium-term (one month to one year following completion) and long-term (greater than one year).’  **Review methods:** ‘We analysed data as short-term (up to one month following completion of the therapy), medium-term (one month to one year following completion) and long-term (one year or more).’  **Review results:** ‘short’; ‘medium’; ‘long term’.  **Type:** Outcome.  **Covariate summary:** Time point. | Improvement from a diagnosis of post-traumatic stress disorder determined by accepted clinical diagnostic criteria such as the International Classification of Diseases (ICD-10) or Diagnostic and Statistical Manual of Mental Disorders text revision (DSM-IV TR); primary; dichotomous; odds ratio; NT=9; NP=540. | **Number of trials:**  4 (All psychological therapies versus control);  2 (CBT versus control);  2 (Narrative versus control);  0 (EMDR versus controls);  0 (Other psychological therapies versus controls);  2 (CBT versus supportive counselling);  1 (Narrative versus supportive counselling);  2 (Narrative therapy versus other therapies (meditation/relaxation));  1 (Exposure versus interpersonal therapy).  **Covariate distribution (across trials):**  NT=4, NP=127 (All psychological therapies versus control):  NT=2, NP=49 (Short-term);  NT=2, NP=50 (Medium-term);  NT=1, NP=53 (Long-term ).  NT=2, NP=49 (CBT versus control):  NT=2, NP=49 (Short-term);  NT=1, NP=25 (Medium-term).  NT=2, NP=78 (Narrative versus control):  NT=1, NP=25 (Medium-term);  NT=1, NP=53 (Long-term ).  NT=0, NP=0 (EMDR versus controls).  NT=0, NP=0 (Other psychological therapies versus controls).  NT=2, NP=208 (CBT versus supportive counselling):  NT=2, NP=208 (Short-term).  NT=1, NP=48 (Narrative versus supportive counselling):  NT=1, NP=48 (Long-term ).  NT=2, NP=77 (Narrative therapy versus other therapies (meditation/relaxation)):  NT=1, NP=31 (Short-term);  NT=2, NP=76 (Medium-term).  NT=1, NP=30 (Exposure versus interpersonal therapy):  NT=1, NP=30 (Short-term);  NT=1, NP=27 (Medium-term).  **Covariate distribution (within trials):** NA. | **Analysis type in review according to glossary 1:** Subgroup analysis.  **Reported results:** Event rates, OR, and CI for trials. OR, CI, z statistics, and p-value for meta-analytic subtotals.  **Where reported:** Results text and forest plot.  **Method to detect interactions reported in review:** Not reported.  **Reported results from methods to detect interactions:** Not reported.  **Where reported:** NA. | **Reported detected interaction:** Not reported.  **What reported:**  All psychological therapies versus controls  1. Improvement  Over all studies that compared a psychological therapy to a control group, there was a significantly smaller proportion of children and adolescents who were still diagnosed with PTSD at the end of the study compared to the control group in the short term (two studies, n = 49, odds ratio (OR) 8.64, 95% confidence interval (CI) 2.01 to 37.14, I 2 = 0%, Analysis 1.1) and medium term (two studies, n = 50, OR 9.46, 95% CI 2.46 to 36.32, I 2 = 57%, Analysis 1.1) but there was no significant difference in the long term (one study, n = 53, OR 1.84, 95% CI 0.60 to 5.65, Analysis 1.1).  CBT versus controls  1. Improvement  There was significantly more improvement in the CBT groups over the two studies that reported this outcome in the short term (n = 49, OR 8.64, 95% CI 2.01 to 37.14, I 2 = 0%, Analysis 2.1) and one study (n = 25, OR 8.00, 95%CI 1.21 to 52.69) reporting medium-term data.  Narrative therapy versus controls  1. Improvement  There was a significant difference in improvement when narrative therapywas compared to a control in themediumterm(one study, n = 25, OR 11.25, 95% CI 1.65 to 76.85, Analysis 3.1) but not the long term (one study, n = 53, OR 1.84, 95% CI 0.60 to 5.65, Analysis 3.1).  EMDR versus controls  Apart from the two studies which reported PTSDtotal scores, data for all comparisons of EMDR to a control came from one study.  1. Improvement  No data were reported.  Other psychological therapies versus controls  Two studies compared other psychological therapies with a control but because these data could not be pooled all results were from one study only.  1. Improvement  No data were reported.  CBT versus supportive therapy  Two studies compared CBT to supportive therapy.  1. Improvement  The short-term improvement rate was significantly higher in the CBT group compared to the supportive therapy group (two studies, n = 208, OR 3.18, 95% CI 1.76 to 5.74, I 2 = 0%, Analysis 6.1).  Narrative therapy versus supportive counselling  One study compared narrative therapy to supportive counselling.  1. Improvement  There was no statistical difference between groups in improvement (short term: one study, n = 48, OR 1.95, 95% CI 0.60 to 6.29, Analysis 7.1).  Narrative therapy versus other therapies (meditation/relaxation)  1. Improvement  There was no difference in the proportion of children and adolescents still diagnosed with PTSD in the short (one study, n = 31, OR 1.50, 95% CI 0.32 to 7.14, Analysis 8.1) or medium term (two studies, n = 76, OR 1.09, 95% CI 0.41 to 2.88, I 2 = 0%, Analysis 8.1) when narrative therapy was compared to meditation relaxation.  Exposure versus interpersonal therapy  One study compared exposure to interpersonal therapy.  1. Improvement  There was significantly more short and medium-term improvement in the exposure therapy group (short term: n = 30, OR 7.43, 95% CI 1.23 to 45.01; medium term: n = 27, OR 9.60, 95% CI 1.48 to 62.16, Analysis 9.1).  **Where reported:** Results text.  **Interaction exists based on reported results**: Not reported.  **How determined:** NA.  **Reported importance of the interaction:** Not reported.  **What reported:** Not reported.  **Where reported:** NA  **Reported plausibility of the interaction:** Not reported.  **What reported:** Not reported.  **Where reported:** NA  **Reported confounding possibility:** Not reported.  **What reported:** Not reported.  **Where reported:** NA.  **Mentioned covariate distribution:** yes.  **What reported:** as above.  **Where reported:** Results text. |
| Gillies 2012 | **Protocol: ‘**The three main comparisons will be: 1. Psychological therapies vs no treatment 2. Psychological therapies vs pharmacological therapies 3. Psychological therapies vs other treatments Major categories of psychological therapies will include CBT (including behavioural and cognitive based therapies), exposure based therapy, psychodynamic therapy, supportive therapy (which will include non-directive counselling), debriefing (including psychological first aid), family-based therapy and EMDR.’  **Review methods: ‘**Main comparisons**.** 1. Psychological therapies versus a control 2. Psychological therapies versus other psychological therapies 3. Psychological therapies versus pharmacological therapies 4. Psychological therapies versus other treatments. The majority of interventions employed a variety of psychotherapeutic elements, so we categorised interventions on the primary description of the intervention, the primary objectives of the therapy, or both. However, as it became apparent to us during the review that some of the psychological therapies described in included studies were not based on any clear theoretical domain, we made the decision to include these under the heading of ’Other psychological therapies’. We organised data under the following categories of psychological therapies. 1. Cognitive behavioural therapy (CBT) 2. Behavioural therapy (BT) (including exposure-based therapy and narrative therapy) 3. Psychodynamic psychotherapy 4. Supportive counselling 5. EMDR 6. Interpersonal therapy (IPT) 7. Other psychological therapy’.  **Review results: ‘**Comparison 1. All psychological therapies versus control’; ‘Comparison 2. CBT versus control’; ‘Comparison 3. Narrative versus control’; ‘Comparison 4. EMDR versus control’; ‘Comparison 5. Other psychological therapies versus control’; ‘Comparison 6. CBT versus supportive counselling’; ‘Comparison 7. Narrative versus supportive counselling’; ‘Comparison 8. Narrative versus meditation/relaxation’; ‘Comparison 9. Exposure versus interpersonal therapy’.  **Type:** Intervention.  **Covariate summary:** Type of intervention or control. | Improvement from a diagnosis of post-traumatic stress disorder determined by accepted clinical diagnostic criteria such as the International Classification of Diseases (ICD-10) or Diagnostic and Statistical Manual of Mental Disorders text revision (DSM-IV TR); primary; dichotomous; odds ratio; NT=9; NP=540. | **Number of trials:** 9.  **Covariate distribution (across trials):**  NT=4, NP=127 (All psychological therapies versus control);  NT=2, NP=49 (CBT versus control);  NT=2, NP=78 (Narrative versus control);  NT=0, NP=0 (EMDR versus controls);  NT=0, NP=0 (Other psychological therapies versus controls);  NT=2, NP=208 (CBT versus supportive counselling);  NT=1, NP=48 (Narrative versus supportive counselling);  NT=2, NP=77 (Narrative therapy versus other therapies (meditation/relaxation));  NT=1, NP=30 (Exposure versus interpersonal therapy).  **Covariate distribution (within trials):** NA. | **Analysis type in review according to glossary 1:** Subgroup analysis.  **Reported results:** Event rates, OR, and CI for trials. OR, CI, z statistic, and p-value for meta-analytic subtotals.  **Where reported:** Results text and forest plot.  **Method to detect interactions reported in review:** Not reported.  **Reported results from methods to detect interactions:** Not reported.  **Where reported:** NA. | **Reported detected interaction:** Not reported.  **What reported:**  All psychological therapies versus controls  1. Improvement  Over all studies that compared a psychological therapy to a control group, there was a significantly smaller proportion of children and adolescents who were still diagnosed with PTSD at the end of the study compared to the control group in the short term (two studies, n = 49, odds ratio (OR) 8.64, 95% confidence interval (CI) 2.01 to 37.14, I 2 = 0%, Analysis 1.1) and medium term (two studies, n = 50, OR 9.46, 95% CI 2.46 to 36.32, I 2 = 57%, Analysis 1.1) but there was no significant difference in the long term (one study, n = 53, OR 1.84, 95% CI 0.60 to 5.65, Analysis 1.1).  CBT versus controls  1. Improvement  There was significantly more improvement in the CBT groups over the two studies that reported this outcome in the short term (n = 49, OR 8.64, 95% CI 2.01 to 37.14, I 2 = 0%, Analysis 2.1) and one study (n = 25, OR 8.00, 95%CI 1.21 to 52.69) reporting medium-term data.  Narrative therapy versus controls  1. Improvement  There was a significant difference in improvement when narrative therapywas compared to a control in themediumterm(one study, n = 25, OR 11.25, 95% CI 1.65 to 76.85, Analysis 3.1) but not the long term (one study, n = 53, OR 1.84, 95% CI 0.60 to 5.65, Analysis 3.1).  EMDR versus controls  Other psychological therapies versus controls  CBT versus supportive therapy  Two studies compared CBT to supportive therapy.  1. Improvement  The short-term improvement rate was significantly higher in the CBT group compared to the supportive therapy group (two studies, n = 208, OR 3.18, 95% CI 1.76 to 5.74, I 2 = 0%, Analysis 6.1).  Narrative therapy versus supportive counselling  One study compared narrative therapy to supportive counselling.  1. Improvement  There was no statistical difference between groups in improvement (short term: one study, n = 48, OR 1.95, 95% CI 0.60 to 6.29, Analysis 7.1).  Narrative therapy versus other therapies (meditation/relaxation)  1. Improvement  There was no difference in the proportion of children and adolescents still diagnosed with PTSD in the short (one study, n = 31, OR 1.50, 95% CI 0.32 to 7.14, Analysis 8.1) or medium term (two studies, n = 76, OR 1.09, 95% CI 0.41 to 2.88, I 2 = 0%, Analysis 8.1) when narrative therapy was compared to meditation relaxation.  Exposure versus interpersonal therapy  One study compared exposure to interpersonal therapy.  1. Improvement  There was significantly more short and medium-term improvement in the exposure therapy group (short term: n = 30, OR 7.43, 95% CI 1.23 to 45.01; medium term: n = 27, OR 9.60, 95% CI 1.48 to 62.16, Analysis 9.1).  **Where reported:** Results text.  **Interaction exists based on reported results:** Not reported**.**  **How determined:** NA.  **Reported importance of the interaction:** Not reported.  **What reported:** Not reported.  **Where reported:** NA  **Reported plausibility of the interaction:** Not reported.  **What reported:** Not reported.  **Where reported:** NA  **Reported confounding possibility:** Not reported.  **What reported:** Not reported.  **Where reported:** NA.  **Mentioned covariate distribution:** Yes.  **What reported:** as above.  **Where reported:** Results text. |
| Gillies 2012 | **Protocol: ‘**The type of trauma: natural or man-made disaster, physical or sexual abuse, war, violence, and life-threatening injury or illness.’  **Review methods:** ‘Type of trauma: sexual abuse, civil or social violence, physical trauma, natural disasters.’  **Review results:** ‘Sexual abuse’; ‘Civil/social violence’; ‘Physical trauma’.  **Type:** Patient.  **Covariate summary:** Disease characteristics. | Improvement from a diagnosis of post-traumatic stress disorder determined by accepted clinical diagnostic criteria such as the International Classification of Diseases (ICD-10) or Diagnostic and Statistical Manual of Mental Disorders text revision (DSM-IV TR); primary; dichotomous; odds ratio; NT=9; NP=540. | **Number of trials:** 4.  **Covariate distribution (across trials):**  NT=1, NP=25 (Sexual abuse);  NT=2, NP=78 (Civil/social violence);  NT=1, NP=24 (Physical trauma).  **Covariate distribution (within trials):**  100% Sexual abuse (1 trial);  100% former child soldiers (1 trial);  violent attacks against the parent or other family members at home 19/26, witnessing attacks outside the home 13/26, accidents 9/26, living in a place of war 9/26, seeing dead bodies 9/26, traumatic medical treatments 7/26, the death of a beloved person 7/26, earthquakes 5/26, other natural disasters 3/26, sexual abuse 2/26 (1 trial);  motor vehicle accident 21/24, interpersonal violence 12/24, witnessing violence 4/24 (1 trial). | **Analysis type in review according to glossary 1:** Subgroup analysis.  **Reported results:** Event rates, OR, and CI for trials. OR, CI, z statistic, and p-value for meta-analytic subtotals.  **Where reported:** Results text and forest plot.  **Method to detect interactions reported in review:** Not reported.  **Reported results from methods to detect interactions:** Test for subgroup differences (Chi-square statistic, p-value, and I square statistic).  **Where reported:** Results text and forest plot. | **Reported detected interaction:** No interaction.  **What reported:**  Type of trauma  We compared the type of trauma (sexual abuse, civil or social violence, physical trauma, natural disaster) in a subgroup analyses. There was no significant difference between subgroups in improvement (Chi2 = 0.88, df = 2, P = 0.65, I 2 = 0%, Analysis 11.1) or PTSD symptoms (Chi2 = 3.36, df = 3, P = 0.24, I 2 = 10.6%, Analysis 11.2).  **Where reported:** Results text.  **Interaction exists based on reported results**: No.  **How determined:** Test for subgroup differences: Chi2= 0.88, df= 2 (P= 0.65), I2=0.0%  **Reported importance of the interaction:** Not reported.  **What reported:** Not reported.  **Where reported:** NA  **Reported plausibility of the interaction:** Not reported.  **What reported:** Not reported.  **Where reported:** NA  **Reported confounding possibility:** Not reported.  **What reported:** Not reported.  **Where reported:** NA.  **Mentioned covariate distribution:** Not reported.  **What reported:** Not reported.  **Where reported:** NA. |
| Gillies 2012 | **Protocol:** Blinding. We judged the following to be two covariates: ‘Sensitivity analyses based on allocation concealment and blinding of outcome measurement will be done as these factors are most associated with a bias in effect size (Moher 1998).’  **Review methods:** Blinding. We judged the following to be two covariates: ‘We carried out sensitivity analyses based on allocation concealment and blinding of outcome measurement as these factors are most associated with a bias in effect size’. Blinding: low versus unclear versus high risk.’  **Review results:** ‘low’; ‘unclear’; ‘high’.  **Type:** Methodological.  **Covariate summary:** Blinding. | Improvement from a diagnosis of post-traumatic stress disorder determined by accepted clinical diagnostic criteria such as the International Classification of Diseases (ICD-10) or Diagnostic and Statistical Manual of Mental Disorders text revision (DSM-IV TR); primary; dichotomous; odds ratio; NT=9; NP=540. | **Number of trials:** 4.  **Covariate distribution (across trials):**  NT=2, NP=49 (low risk);  NT=1, NP=53 (unclear risk);  NT=1, NP=25 (high risk).  **Covariate distribution (within trials):** NA. | **Analysis type in review according to glossary 1:** Subgroup analysis.  **Reported results:** Event rates, OR, and CI for trials. OR, CI, z statistics, and p-value for meta-analytic subtotals.  **Where reported:** Results text and forest plot.  **Method to detect interactions reported in review:** Not reported.  **Reported results from methods to detect interactions:** Test for subgroup differences (Chi-square statistic, p-value, and I square statistic).  **Where reported:** Forest plot. | **Reported detected interaction:** No interaction.  **What reported:**  **Blinding**  There was no difference between studies with a low, high or unclear risk of detection bias in improvement (Chi2 = 4.36, df = 2, P = 0.11, I 2 = 54.1%, Analysis 13.1)’.  **Where reported:** Results text.  **Interaction exists based on reported results**: No.  **How determined:** Test for subgroup differences: Chi2= 4.36, df= 2 (P= 0.11), I2=54%  **Reported importance of the interaction:** Not reported.  **What reported:** Not reported.  **Where reported:** NA  **Reported plausibility of the interaction:** Not reported.  **What reported:** Not reported.  **Where reported:** NA  **Reported confounding possibility:** Not reported.  **What reported:** Not reported.  **Where reported:** NA.  **Mentioned covariate distribution:** Not reported.  **What reported:** Not reported.  **Where reported:** NA. |
| Gillies 2012 | **Protocol:** Not reported.  **Review methods: ‘**Type of therapy: CBT, narrative therapy, supportive counselling, interpersonal therapy, EMDR.’  **Review results:** ‘CBT’ versus ‘narrative’.  **Type:** Intervention.  **Covariate summary:** Type of intervention or control. | Improvement from a diagnosis of post-traumatic stress disorder determined by accepted clinical diagnostic criteria such as the International Classification of Diseases (ICD-10) or Diagnostic and Statistical Manual of Mental Disorders text revision (DSM-IV TR); primary; dichotomous; odds ratio; NT=9; NP=540. | **Number of trials:** 4.  **Covariate distribution (across trials):**  NT=2, NP=49 (CBT);  NT=2, NP=78 (Narrative).  **Covariate distribution (within trials):** NA. | **Analysis type in review according to glossary 1:** Subgroup analysis.  **Reported results:** Event rates, OR, and CI for trials. OR, CI, z statistic and p-value for meta-analytic subtotals.  **Where reported:** Results text and forest plot.  **Method to detect interactions reported in review:** Not reported.  **Reported results from methods to detect interactions:** Test for subgroup differences (Chi-square statistic, p-value, and I square statistic).  **Where reported:** Results text and forest plot. | **Reported detected interaction:** No interaction.  **What reported:**  Type of therapy  We carried out subgroup analyses of the different psychological therapies compared to controls for the primary outcomes of improvement and PTSD symptoms. Improvement was reported in studies which had used CBT and narrative therapies. There was no difference between these subgroups (Chi2 = 0.49, df = 1, P = 0.48, I 2 = 0%, Analysis 10.1). There was also no difference between CBT, narrative, EMDR and other psychological therapies subgroups for symptoms of PTSD (Chi2 = 4.03, df = 3, P = 0.26, I 2 = 25.5%, Analysis 10.2).  **Where reported:** Results text.  **Interaction exists based on reported results**: No.  **How determined:** Test for subgroup differences: Chi2= 0.49, df= 1 (P= 0.48), I2=0.0%  **Reported importance of the interaction:** Not reported.  **What reported:** Not reported.  **Where reported:** NA  **Reported plausibility of the interaction:** Not reported.  **What reported:** Not reported.  **Where reported:** NA  **Reported confounding possibility:** Not reported.  **What reported:** Not reported.  **Where reported:** NA.  **Mentioned covariate distribution:** Not reported.  **What reported:** Not reported.  **Where reported:** NA. |
| Goldenberg 2013 | **Protocol:** Not reported.  **Review methods: ‘**Probiotic species’.  **Review results: ‘**Lactobacillus GG; S. boulardii; L. acidophilus + L. Casei’; ‘L. acidophilus + B. Bifidum L. Acidophilus’; ‘L. acidophilus + L. bulgaricus + B. bifidum + S. Thermophiles’; ‘B. breve + B. Longum +B. infantis + L. acidophilus +L. plantarum + L. paracasei +L. bulgaricus + S. Thermophiles’; ‘L. casei + L. bulgaris + S. Thermophiles’; ‘L. Plantarum’; ‘Lactobacillus GG + L. acidophilus + B. Animalis’.  ‘Lactobacillus GG’ versus ‘ S. boulardii’.  ‘Lactobacillus GG’ versus ‘L. acidophilus + L. Casei’.  **Type:** Intervention.  **Covariate summary:** Type of intervention or control. | Incidence of *C. difficile-*associated diarrhea; primary;  dichotomous; risk ratio; NT=23; NP=4,213. | **Number of trials:**  NT=22 (All species);  NT=12 (LGG versus SB);  NT=8 (Species: LGG versus LA + LC).  **Covariate distribution (across trials):**  NT=22 (All species):  NT=5, NP=1,131 (Lactobacillus GG);  NT=7, NP=1,507 (S. boulardii);  NT=3, NP=781 (L. acidophilus + L. casei);  NT=1, NP=138 (L. acidophilus + B. Bifidum);  NT=1, NP=40 (L. acidophilus);  NT=1, NP=100 (L. acidophilus + L. bulgaricus + B. bifidum + S. thermophiles);  NT=1, NP=124 (B. breve + B. Longum +B. infantis + L. acidophilus +L. plantarum + L. paracasei +L. bulgaricus + S. thermophiles);  NT=1, NP=109 (L. casei + L. bulgaris + S.Thermophiles);  NT=1, NP=163 (L. plantarum);  NT=1, NP=63 (Lactobacillus GG + L.acidophilus + B. animalis).  NT=12 (LGG versus SB):  NT=5, NP=1,131 (Lactobacillus GG);  NT=7, NP=1,507 (boulardii).  NT=8 (Species: LGG versus LA + LC):  NT=5, NP=1,131 (Lactobacillus GG);  NT=3, NP=781 (L. acidophilus + L. casei).  **Covariate distribution (within trials):** NA. | **Analysis type in review according to glossary 1:** Subgroup analysis.  **Reported results:** Event rates, risk ratio, and CI for trials. Risk ratio, CI, z statistic, and p-value for meta-analytic subtotals and total.  **Where reported:** Results text and forest plot.  **Method to detect interactions reported in review: ‘**To evaluate the credibility of our  subgroup analyses we used pre-specified criteria, including a test  for interaction (Sun 2010).’  **Reported results from methods to detect interactions:** Test for subgroup differences (Chi-square statistic, p-value, and I square statistic).  **Where reported:** Results text and forest plot. | **Reported detected interaction:** Interaction.  **What reported:**  **‘**With two exceptions, no subgroup comparisons resulted in a statistically significant test of interaction. The exceptions were noted when comparing the *L. acidophilus* + *L. casei* subgroup (RR 0.21; 95% CI 0.11 to 0.42, I2 = 0%, n = 781) versus *Lactobacillus rhamnosus* subgroup (RR 0.63; 95% CI 0.30 to 1.33, I2 = 88%, n = 1031) for the CDAD outcome ........... Regarding the former, the test for interaction revealed statistically significant species related heterogeneity (P = 0.03). However, using 11 published criteria to evaluate the credibility of the subgroup analysis (Sun 2010), we consider the credibility of this subgroup effect as unlikely. That is, the subgroup effect is based on between study comparisons and is not consistent across studies, the direction of the subgroup effect was not pre-specified and we are unaware of any biological or direct evidence that suggests that *L. acidophilus* + *L. casei* is superior to *Lactobacillus rhamnosus*.’  **Where reported:** Results text.  **Interaction exists based on reported results:** Yes for LGG versus LA + LC. No for LGG versus SB and all species.  **How determined:**  All species: Test for subgroup differences: Chi2= 9.24, df= 8 (P= 0.32), I2=13%  LGG versus SB: Test for subgroup differences: Chi2= 0.32, df= 1 (P= 0.57), I2=0.0%  LGG versus LA + LC: Test for subgroup differences: Chi2= 4.54, df= 1 (P= 0.03), I2=78%  **Reported importance of the interaction:** Not reported.  **What reported:** Not reported.  **Where reported:** NA  **Reported plausibility of the interaction:** Yes.  **What reported:**  As above. ‘However, using 11 published criteria to evaluate the credibility of the subgroup analysis (Sun 2010), we consider the credibility of this subgroup effect as unlikely. That is, the subgroup effect is based on between study comparisons and is not consistent across studies, the direction of the subgroup effect was not pre-specified and we are unaware of any biological or direct evidence that suggests that *L. acidophilus* + *L. casei* is superior to *Lactobacillus rhamnosus*.’  **Where reported:** Results text.  **Reported confounding possibility:** Not reported.  **What reported:** Not reported.  **Where reported:** NA.  **Mentioned covariate distribution:** Not reported.  **What reported:** Not reported.  **Where reported:** NA. |
| Goldenberg 2013 | **Protocol:** Not reported.  **Review methods:** ‘Inpatients versus outpatients’.  **Review results: ‘**Inpatient’; ‘outpatient’; ‘mixed’.  **Type:** Patient.  **Covariate summary:** Setting. | Incidence of *C. difficile-*associated diarrhea; primary;  dichotomous; risk ratio; NT=23; NP=4,213. | **Number of trials:** 21.  **Covariate distribution (across trials):**  NT=14, NP=2,359 (inpatient);  NT=2, NP=462 (outpatient);  NT=5, NP=1,205 (mixed).  **Covariate distribution (within trials):**  100% inpatient (14 trials);  100% outpatient (1 trial);  Not reported (analysed as outpatient) (1 trial);  ‘mixed inpatient and outpatient’ (4 trials);  inpatients 5/119 outpatients 114/119 (1 trial). | **Analysis type in review according to glossary 1:** Subgroup analysis.  **Reported results:** Event rates, risk ratio, and CI for trials. Risk ratio, CI, z statistic, and p-value for meta-analytic subtotals and total.  **Where reported:** Results text and forest plot.  **Method to detect interactions reported in review: ‘**To evaluate the credibility of our  subgroup analyses we used pre-specified criteria, including a test  for interaction (Sun 2010).’  **Reported results from methods to detect interactions:** Test for subgroup differences (Chi-square statistic, p-value, and I square statistic).  **Where reported:** Results text and forest plot. | **Reported detected interaction:** No interaction.  **What reported:**  ‘Regarding patient population, one trial had exclusively outpatient data, 14 trials had inpatient data, five had mixed populations, and three were not specified. In *post hoc* analysis there was no statistical evidence of a subgroup difference (P= 0.64, I2=0%).’  **Where reported:** Results text.  **Interaction exists based on reported results:** No.  **How determined:** Test for subgroup differences: Chi2= 0.24, df= 2 (P= 0.89), I2=0.0%  **Reported importance of the interaction:** Not reported.  **What reported:** Not reported.  **Where reported:** NA  **Reported plausibility of the interaction:** Not reported.  **What reported:** Not reported.  **Where reported:** NA  **Reported confounding possibility:** Not reported.  **What reported:** Not reported.  **Where reported:** NA.  **Mentioned covariate distribution:** Yes.  **What reported:** as above.  **Where reported:** Results text. |
| Goldenberg 2013 | **Protocol:** Not reported.  **Review methods: ‘**The risk of bias’.  **Review results:** Risk of bias. ‘Low risk of bias’; ‘high or unclear risk of bias’.  **Type:** Methodological.  **Covariate summary:** Risk of bias. | Incidence of *C. difficile-*associated diarrhea; primary;  dichotomous; risk ratio; NT=23; NP=4,213. | **Number of trials:** 23.  **Covariate distribution (across trials):**  NT=7, NP=1,308 (low risk);  NT=16, NP=2,972 (High or unclear risk of bias unclear risk).  **Covariate distribution (within trials):** NA. | **Analysis type in review according to glossary 1: S**ubgroup analysis.  **Reported results:** Event rates, risk ratio, and CI for trials. Risk ratio, CI, z statistic, and p-value for meta-analytic subtotals and total.  **Where reported:** Results text and forest plot.  **Method to detect interactions reported in review: ‘**To evaluate the credibility of our  subgroup analyses we used pre-specified criteria, including a test  for interaction (Sun 2010).’  **Reported results from methods to detect interactions:** Test for subgroup differences (Chi-square statistic, p-value, and I square statistic).  **Where reported:** Results text and forest plot. | **Reported detected interaction:** No interaction.  **What reported:**  ‘Seven of the 23 studies were rated as having a low risk of bias, while 16 were rated as having a high or unclear risk of bias. The low risk of bias studies suggested a stronger pooled protective effect of probiotics (RR 0.27; 95% CI 0.16 to 0.46) than the high risk of bias studies (RR 0.45; 95% CI 0.28 to 0.72) although a test of interaction between low and high or unclear risk of bias studies was not statistically significant (P= 0.16).’  **Where reported:** Results text.  **Interaction exists based on reported results**: No.  **How determined:** Test for subgroup differences: Chi2= 1.99, df= 1 (P= 0.16), I2=50%  **Reported importance of the interaction:** Not reported.  **What reported:** Not reported.  **Where reported:** NA  **Reported plausibility of the interaction:** Not reported.  **What reported:** Not reported.  **Where reported:** NA  **Reported confounding possibility:** Not reported.  **What reported:** Not reported.  **Where reported:** NA.  **Mentioned covariate distribution:** Yes.  **What reported:** as above.  **Where reported:** Results text. |
| Goldenberg 2013 | **Protocol:** Not reported.  **Review methods: ‘**Adult versus pediatric population’.  **Review results:** ‘Adult studies’ versus ‘pediatric studies’.  **Type:** Patient.  **Covariate summary:** Demographics. | Incidence of *C. difficile-*associated diarrhea; primary;  dichotomous; risk ratio; NT=23; NP=4,213. | **Number of trials:** 22.  **Covariate distribution (across trials):**  NT=19, NP=3,551 (adult studies);  NT=3, NP= 605 (Pediatric studies).  **Covariate distribution (within trials):**  ‘Mixed population (15 to 81 years of age)’ (analysed as adult) (1 trial);  ‘17 to 81 yrs of age’ (1 trial);  ‘Adult population (elderly)’ (1 trial);  ‘Adult population (> 50 years of age)’ (1 trial);  ‘Pediatric population’ (3 trials);  ‘Adult population’ (14 trials);  not reported (analysed as adult) (1 trial). | **Analysis type in review according to glossary 1:** Subgroup analysis.  **Reported results:** Event rates, risk ratio, and CI for trials. Risk ratio, CI, z statistic, and p-value for meta-analytic subtotals and total.  **Where reported:** Results text and forest plot.  **Method to detect interactions reported in review: ‘**To evaluate the credibility of our  subgroup analyses we used pre-specified criteria, including a test  for interaction (Sun 2010).’  **Reported results from methods to detect interactions:** Test for subgroup differences (Chi-square statistic, p-value, and I square statistic).  **Where reported:** Results text and forest plot. | **Reported detected interaction:** Not reported.  **What reported:** Not reported.  **Where reported:** NA.  **Interaction exists based on reported results:** No.  **How determined:** Test for subgroup differences: Chi2= 0.07, df= 1 (P= 0.79), I2=0.0%  **Reported importance of the interaction:** Not reported.  **What reported:** Not reported.  **Where reported:** NA  **Reported plausibility of the interaction:** Not reported.  **What reported:** Not reported.  **Where reported:** NA  **Reported confounding possibility:** Not reported.  **What reported:** Not reported.  **Where reported:** NA.  **Mentioned covariate distribution:** Not reported.  **What reported:** Not reported.  **Where reported:** NA. |
| Gower 2013 | **Protocol: ‘**Comparisons of interest will be: (1) any prophylaxis versus no prophylaxis; (2) preoperative versus postoperative or intraoperative prophylaxis or combinations; (3) comparisons of specific antibiotics used in included trials; (4) mode of delivery.’  **Review methods: ‘**Comparisons of interest included: 1. Any prophylaxis versus no prophylaxis; 2. Preoperative versus postoperative or intraoperative prophylaxis or combinations; 3. Specific antibiotics used in included trials; 4. Mode of perioperative antibiotic delivery.’  **Review results:** ‘Perioperative prophylaxis versus no prophylaxis’; ‘Comparisons of specific antibiotics or combinations of antibiotics’; ‘Mode of antibiotic delivery’ (in table).  **Type:** Intervention.  **Covariate summary:** Type of intervention or control. | Endophthalmitis: both presumed and culture-proven endophthalmitis within six weeks after cataract surgery. Our primary analysis was based on six-week outcomes; however, we also evaluated data from weeks one, two and four; primary; dichotomous; risk ratio; NT=4; NP=100,876. | **Number of trials**: 4.  **Covariate distribution (across trials):**  NT=2, NP=17,243 (‘Perioperative prophylaxis versus no prophylaxis’);  NT=2, NP=23,221 (‘Comparisons of specific antibiotics or combinations of antibiotics’);  NT=1, NP=77,015 (‘Mode of antibiotic delivery’).  **Covariate distribution (within trials):** NA. | **Analysis type in review according to glossary 1:** Subgroup analysis.  **Reported results:** Number of eyes, event rates, risk ratio, and CI for trials.  **Where reported:** Results table.  **Method to detect interactions reported in review:** Not reported.  **Reported results from methods to detect interactions:** Not reported.  **Where reported:** NA. | **Reported detected interaction:** Not reported.  **What reported:** Not reported. (results text stratifed differently).  **Where reported:** NA.  **Interaction exists based on reported results:** Not reported.  **How determined:** NA.  **Reported importance of the interaction:** Not reported.  **What reported:** Not reported.  **Where reported:** NA  **Reported plausibility of the interaction:** Not reported.  **What reported:** Not reported.  **Where reported:** NA  **Reported confounding possibility:** Not reported.  **What reported:** Not reported.  **Where reported:** NA.  **Mentioned covariate distribution:** Not reported.  **What reported:** Not reported.  **Where reported:** NA. |
| Gower 2013 | **Protocol:** Not reported.  **Review methods:** Not reported.  **Review results:** ‘Chloramphenicol-suphadimidine drops alone or with periocular penicillin’; ‘Subconjunctival versus retrobulbar antibiotic Injection’; ‘Irrigation with balanced salt solution (BSS) alone versus BSS with antibiotics’; ‘Perioperative prophylaxis with intracameral and/or topical antibiotics’.  **Type:** Intervention.  **Covariate summary:** Type of intervention or control. | Endophthalmitis: both presumed and culture-proven endophthalmitis within six weeks after cataract surgery. Our primary analysis was based on six-week outcomes; however, we also evaluated data from weeks one, two and four; primary; dichotomous; risk ratio; NT=4; NP=100,876. | **Number of trials:** 4.  **Covariate distribution (across trials):**  NT=1, NP=6,618 (‘Chloramphenicol-suphadimidine drops alone or with periocular penicillin’);  NT=1, NP=77,015 (‘Subconjunctival versus retrobulbar antibiotic Injection’);  NT=1, NP=640 (‘Irrigation with balanced salt solution (BSS) alone versus BSS with antibiotics);  NT=1, NP=16,603 (‘Perioperative prophylaxis with intracameral and/or topical antibiotics’).  **Covariate distribution (within trials):** NA. | **Analysis type in review according to glossary 1:** Subgroup analysis.  **Reported results:** Number of eyes, event rates, risk ratio, and CI for trials.  **Where reported:** Results text and table.  **Method to detect interactions reported in review:** Not reported.  **Reported results from methods to detect interactions:** Not reported.  **Where reported:** NA. | **Reported detected interaction:** Not reported.  **What reported:**  Chloramphenicol-suphadimidine drops alone or with periocular penicillin:  In the Christy 1979 study of chloramphenicol-sulphadimidine drops with or without periocular penicillin injection, five (0.15%) of 3309 eyes that received combined prophylaxis (drops and injection at the time of surgery) had postoperative endophthalmitis at one week, compared with 15 (0.45%) of 3309 eyes that received the topical regimen alone (risk ratio (RR) 0.33, 95% confidence interval (CI) 0.12 to 0.92).  Subconjunctival versus retrobulbar antibiotic injection:  In Christy 1986, at one week after surgery, 38 (0.10%) of 39,752 eyes receiving subconjunctival injection had presumed postoperative endophthalmitis, compared with 42 (0.11%) of 37,263 eyes receiving retrobulbar antibiotic injection. The risk of postoperative endophthalmitis was similar between groups (RR 0.85, 95% CI 0.55 to 1.32).  Irrigation with balanced salt solution (BSS) alone versus BSS with antibiotics:  In Sobaci 2003, at six weeks none of the 322 eyes that received vancomycin and gentamycin in balanced salt solution (BSS) irrigating infusion fluid had postoperative endophthalmitis compared with two (0.62%) of 322 eyes that received BSS-only irrigating infusion fluid. This difference was not statistically significant (RR 0.20, 95% CI 0.01 to 4.15), but the analysis was based on a small number of cases and was not powered to detect a difference.  Perioperative prophylaxis with intracameral and/or topical antibiotics:  In ESCRS 2007, the risk of clinically-diagnosed (presumed) postoperative endophthalmitis at six weeks was significantly reduced for eyes that received intracameral cefuroxime injections, with or without topical levofloxacin, compared with no prophylaxis (neither injection nor topical levofloxacin) (RR 0.14, 95% CI 0.03 to 0.63with topical levofloxacin;RR0.21, 95%CI 0.06 to 0.74without topical drops) (Table 1). Similar results were observed when analyzing culture-proven cases of postoperative endophthalmitis. Topical levofloxacin alone did not significantly reduce the risk of postoperative endophthalmitis comparedwith no prophylaxis (RR 0.72, 95% CI 0.32 to 1.61 for presumed cases; RR 0.70, 95% CI 0.27 to 1.84 for culture-proven cases) (Table 1). Additionally, no statistically significant difference was evident between eyes treated with intracameral cefuroxime alone and eyes treated with topical levofloxacin alone. A statistically significant risk reduction was observed for eyes treated with combined intracameral cefuroxime and topical levofloxacin compared with eyes treated with topical levofloxacin alone for presumed cases of postoperative endophthalmitis (RR 0.20, 95% CI 0.04 to 0.91), but this difference was not statistically significant for culture-proven cases (RR 0.14, 95% CI 0.02 to 1.16).  **Where reported:** Results text.  **Interaction exists based on reported results:** Not reported.  **How determined:** NA.  **Reported importance of the interaction:** Not reported.  **What reported:** Not reported.  **Where reported:** NA  **Reported plausibility of the interaction:** Not reported.  **What reported:** Not reported.  **Where reported:** NA  **Reported confounding possibility:** Not reported.  **What reported:** Not reported.  **Where reported:** NA.  **Mentioned covariate distribution:** Yes.  **What reported:** as above.  **Where reported:** Results text. |
| **Itchaki 2013** | **Protocol:** ‘Allocation concealment’.  **Review methods: ‘**Allocation concealment’.  **Review results: ‘**Adequate’ versus ‘unclear’.  **Type:** Methodological.  **Covariate summary:** Allocation concealment. | Overall survival; primary; time to event; hazard ratio; NT=5; NP=949. | **Number of trials:** 3.  **Covariate distribution (across trials):**  NT=2, NP=265 **(**adequate);  NT=1, NP=199 **(**unclear).  **Covariate distribution (within trials):** NA. | **Analysis type in review according to glossary 1:** Subgroup analysis.  **Reported results:** Event rates, HR, and CI for trials. HR, CI, z statistic, and p-value for meta-analytic subtotals.  **Where reported:** Results text and forest plot.  **Method to detect interactions reported in review:** Not reported.  **Reported results from methods to detect interactions:** Test for subgroup differences (Chi-square statistic, p-value, and I square statistic).  **Where reported:** Forest plot. | **Reported detected interaction:** Not reported.  **What reported:**  ‘There was no evidence for OS benefit of anthracyclines in trials reporting adequate allocation concealment or in those with unclear allocation concealment (Analysis 2.1).’  **Where reported:** Results text.  **Interaction exists based on reported results:** No.  **How determined:** Test for subgroup differences: Chi2= 0.05, df= 1 (P= 0.82), I2=0.0%  **Reported importance of the interaction:** Not reported.  **What reported:** Not reported.  **Where reported:** NA  **Reported plausibility of the interaction:** Not reported.  **What reported:** Not reported.  **Where reported:** NA  **Reported confounding possibility:** Not reported.  **What reported:** Not reported.  **Where reported:** NA.  **Mentioned covariate distribution:** Not reported.  **What reported:** Not reported.  **Where reported:** NA. |
| **Itchaki 2013** | **Protocol:** ‘Different anthracyclines’.  **Review methods: ‘**Different anthracyclines’.  **Review results: ‘**Doxorubicin’ versus ‘Idarubicin’.  **Type:** Intervention.  **Covariate summary:** Type of intervention or control. | Overall survival; primary; time to event; hazard ratio; NT=5; NP=949. | **Number of trials:** 3.  **Covariate distribution (across trials):**  NT=2, NP=265 **(**Doxorubicin);  NT=1, NP=199 **(**Idarubicin).  **Covariate distribution (within trials):** NA. | **Analysis type in review according to glossary 1:** Subgroup analysis.  **Reported results:** Event rates, HR, and CI for trials. HR, CI, z statistic, and p-value for meta-analytic subtotals.  **Where reported:** Results text and forest plot.  **Method to detect interactions reported in review:** Not reported.  **Reported results from methods to detect interactions:** Test for subgroup differences (Chi-square statistic, p-value, and I square statistic).  **Where reported:** Forest plot. | **Reported detected interaction:** Not reported.  **What reported:** ‘We undertook subgroup analysis of the different anthracyclines used. Regarding OS, trials employing the same chemotherapies used either doxorubicin (Jones 1983; Lepage 1990) or idarubicin (Taylor 2006). There was no evidence of survival benefit to either type of anthracycline, as shown in Analysis 4.1.’  **Where reported:** Results text.  **Interaction exists based on reported results:** No.  **How determined:** Test for subgroup differences: Chi2= 0.05, df= 1 (P= 0.82), I2=0.0%  **Reported importance of the interaction:** Not reported.  **What reported:** Not reported.  **Where reported:** NA.  **Reported plausibility of the interaction:** Not reported.  **What reported:** Not reported.  **Where reported:** NA  **Reported confounding possibility:** Not reported.  **What reported:** Not reported.  **Where reported:** NA.  **Mentioned covariate distribution:** Yes.  **What reported:** As above.  **Where reported:** Results text. |
| **Itchaki 2013** | **Protocol:** ‘Exclude studies in which the percentage of patients with FL is less than 70% of evaluated patients.’  **Review methods:** Not reported.  **Review results:** Included Zinzani 2000, a trial that included 50% FL.  **Type:** Patient.  **Covariate summary:** Disease characteristics. | Overall survival; primary; time to event; hazard ratio; NT=5; NP=949. | **Number of trials:** 4.  **Covariate distribution (across trials):**  NT=4, NP=663 **(**Included Zinzani 2000).  **Covariate distribution (within trials):**  number with FL/number randomised: 102/208 (1 trial); 226/652 (1 trial); 101/113 (1 trial); 155/200 (1 trial). | **Analysis type in review according to glossary 1:** Sensitivity analysis.  **Reported results:** Event rates, HR, and CI for trials. HR, CI, z statistic, and p-value for meta-analytic subtotals.  **Where reported:** Results text and forest plot.  **Method to detect interactions reported in review:** Not reported.  **Reported results from methods to detect interactions:** Not reported.  **Where reported:** NA. | **Reported detected interaction:** No interaction.  **What reported: ‘**OS data were analyzed for 464 patients included in three studies (Jones 1983; Lepage 1990; Taylor 2006). The pooled HR for OS was 0.99 (95% CI 0.77 to 1.29; Figure 3), indicating that there was no advantage to ACR chemotherapy compared with other chemotherapy. There was no heterogeneity among trials (P = 0.37; I2 = 0%). Federico 2013 did not publish mortality data, as it was under-powered to assess survival. We did not include Zinzani 2000 in the analysis, since survival data were reported for the whole cohort, and not specifically for FL. However, adding it did not change the pooled results (HR 0.97; 95%CI 0.76 to 1.23, Analysis 1.2).’  **Where reported:** Results text.  **Interaction exists based on reported results**: Not reported.  **How determined:** NA.  **Reported importance of the interaction:** Not reported.  **What reported:** Not reported.  **Where reported:** NA  **Reported plausibility of the interaction:** Not reported.  **What reported:** Not reported.  **Where reported:** NA  **Reported confounding possibility:** Not reported.  **What reported:** Not reported.  **Where reported:** NA.  **Mentioned covariate distribution:** Yes.  **What reported:** as aabove.  **Where reported:** Results text. |
| **Itchaki 2013** | **Protocol: ‘**We will separate all analyses for trials in which the only difference between study arms is the addition of an anthracycline (“same” comparison) and trials where different chemotherapy regimens are used in the ACR and non-ACR arms.’ **‘**As detailed above, we will conduct a separate analysis for studies assessing directly the efficacy of anthracyclines by comparing the same chemotherapy regimens with or without the addition of an anthracycline.’  **Review methods:** ‘We separated trials according to the similarity of chemotherapeutic  regimens, other than anthracyclines, between study arms. Trials with the same chemotherapy, where the main difference was the addition of an anthracycline, were summed together.’ ‘As detailed above, we conducted a separate analysis for studies assessing directly the efficacy of anthracyclines by comparing the same chemotherapy regimens with or without the addition of an anthracycline.’  **Review results:** ‘Studies comparing same chemotherapy’; ‘Studies comparing different chemotherapies’ (in text). ‘Anthracycline versus no anthracycline different chemotherapy’; ‘Anthracycline versus no anthracycline same chemotherapy’ (in plot).  **Type:** Intervention.  **Covariate summary:** Additional drugs. | Overall survival; primary; time to event; hazard ratio; NT=5; NP=949. | **Number of trials:** 5.  **Covariate distribution (across trials):**  NT=3, NP=464 (‘Studies comparing same chemotherapy’ or ‘Anthracycline versus no anthracycline same chemotherapy’);  NT=2, NP=485 (‘Studies comparing different chemotherapies’ or‘Anthracycline versus no anthracycline different chemotherapy’).  **Covariate distribution (within trials):** NA. | **Analysis type in review according to glossary 1:** Subgroup analysis.  **Reported results:** Event rates, HR, and CI for trials. HR, CI, z statistic, and p-value for meta-analytic subtotals.  **Where reported:** Results text and forest plot.  **Method to detect interactions reported in review:** Not reported.  **Reported results from methods to detect interactions:** Not reported.  **Where reported:** NA. | **Reported detected interaction:** No interaction.  **What reported:**  Studies comparing same chemotherapy:  OS data were analyzed for 464 patients included in three studies (Jones 1983; Lepage 1990; Taylor 2006). The pooled HR for OS was 0.99 (95% CI 0.77 to 1.29; Figure 3), indicating that there was no advantage to ACR chemotherapy compared with other chemotherapy. There was no heterogeneity among trials (P = 0.37; I2 = 0%). Federico 2013 did not publish mortality data, as it was under-powered to assess survival. We did not include Zinzani 2000 in the analysis, since survival data were reported for the whole cohort, and not specifically for FL. However, adding it did not change the pooled results (HR 0.97; 95%CI 0.76 to 1.23, Analysis 1.2).  Studies comparing different chemotherapies:  Three trials used different regimens between study arms, and among trials themselves, and therefore could not be summed together. Peterson 2003 compared CHOP-B with continuous cyclophosphamide PO; Unterhalt 1996 compared prednimustinemitoxantrone with CVP regimen; and Kimby 1994 evaluated CHOP with chlorambucil-dexamethasone, both given PO (Characteristics of included studies). The effects of interventions are summarized in Additional Table 4, and graphically in Analysis 5.1; Analysis 5.2; Analysis 5.3; and Analysis 5.4. Consistent with pooled results presented for trials comparing the same chemotherapy, therewas no evidence of effect on survival, three-, and five-year mortality for the ACR arms. All trials showed a trend in favour of anthracyclines for disease control. Results for CR and OR were heterogeneous.  **Where reported:** Results text.  **Interaction exists based on reported results:** Not reported.  **How determined:** NA.  **Reported importance of the interaction:** Not reported.  **What reported:** Not reported.  **Where reported:** NA  **Reported plausibility of the interaction:** Not reported.  **What reported:** Not reported.  **Where reported:** NA  **Reported confounding possibility:** Not reported.  **What reported:** Not reported.  **Where reported:** NA.  **Mentioned covariate distribution:** Yes.  **What reported:** as above.  **Where reported:** Results text. |
| Lawrie 2013 | **Protocol:** ‘The RCTs will be grouped by comparison, i. e. Pegylated liposomal doxorubicin versus best supportive care, single agent pegylated liposomal doxorubicin versus another single agent or pegylated liposomal doxorubicin plus another drug versus the same drug alone or in combination.’  **Review methods:** ‘The RCTs were grouped by Types of interventions. Where the types of interventions differed within a comparison, e. g. Other drugs versus PLD, we subgrouped data by the comparator drug and did not combine subgroup data’ ‘Types of intterventions: 1. PLD in combination with platinum-based therapy versus platinum-based therapy with another agent, e.g. PLD plus carboplatin versus paclitaxel (PAC) plus carboplatin. 2. Other chemotherapy agent(s) versus PLD, e.g. topotecan (TOP) versus PLD. 3. PLD plus other agent(s) versus PLD alone or with placebo, e.g. trabectedin (TBD) plus PLD versus PLD.’  **Review results:** ‘Comparison 1. PLD/carboplatin vs carboplatin ± other drug/s: PLD/carbo vs carbo only’; ‘Comparison 1. PLD/carboplatin vs carboplatin ± other drug/s: PLD/carbo vs PAC/carbo’; ‘Comparison 2. Other drug vs PLD: GEM vs PLD’; ‘Comparison 2. Other drug vs PLD: TOP vs PLD’; ‘Comparison 2. Other drug vs PLD: OLA vs PLD’; ‘Comparison 2. Other drug vs PLD: PAT vs PLD’; ‘Comparison 3. PLD + other drug vs PLD TBD/PLD vs PLD’; ‘Comparison 3. PLD + other drug vs PLD:CAN/PLD vs PLD’; ‘Comparison 3. PLD + other drug vs PLD: EC145/PLD vs PLD’.  **Type:** Intervention.  **Covariate summary:** Type of intervention or control. | Progression-free survival; primary; time to event; hazard ratio; NT=13; NP=4,625. | **Number of trials: 10.**  **Covariate distribution (across trials):**  NT=3, NP=1,225 (Comparison 1. PLD/carboplatin vs carboplatin ± other drug/s):  NT=1, NP=61(PLD/carbo vs carbo only);  NT=2, NP=1,164 (PLD/carbo vs PAC/carbo).  NT=4, NP=1,522 (Comparison 2. Other drug vs PLD):  NT=1, NP=153 (GEM vs PLD);  NT=1, NP=481 (TOP vs PLD);  NT=1, NP=60 (OLA vs PLD);  NT=1, NP=828 (PAT vs PLD).  NT=3, NP=946 (Comparison 3. PLD + other drug vs PLD):  NT=1, NP=672 (TBD/PLD vs PLD);  NT=1, NP=125 (CAN/PLD vs PLD);  NT=1, NP=149 (EC145/PLD vs PLD).  **Covariate distribution (within trials):** NA. | **Analysis type in review according to glossary 1:** Subgroup analysis.  **Reported results:** HR, SE, CI and N for trials. HR, CI and p-value for meta-analytic subtotals.  **Where reported:** Results text and forest plot.  **Method to detect interactions reported in review:** Not reported.  **Reported results from methods to detect interactions:** For one comparison, test for subgroup differences (Chi-square statistic, p-value, and I square statistic).  **Where reported:** Forest plot. | **Reported detected interaction:** Not reported.  **What reported:**  A. PLD plus carboplatin versus carboplatin ± other drug/s:  *PLD/carbo* versus*carbo alone:* The PLD/carbo regimen resulted in a significantly longer PFS that the carbo alone regimen (one study, 61 participants; hazard ratio (HR) 0.52, 95% confidence interval (CI) 0.31 to 0.88; Analysis 1.1).  *PLD/carbo* versus*PAC/carbo:* The PLD/carbo regimen resulted in a significantly longer PFS than the PAC/carbo regimen (two studies, 1164 participants; HR 0.85, 95% CI 0.74 to 0.97; I²= 7%; P value 0.01; Analysis 1.1).’  B. Other drug(s) versus PLD:  Only two studies published HRs for PFS (Kaye 2012; Colombo 2012).We estimatedHRs fromthe raw data of one study (MITO- 3 2008) and from the published Kaplan-Meier curve of another (Gordon 2001). There was only one study per subgroup and we did not combine these data. There were no significant differences in PFS between treatment arms in the GEM versus PLD, TOP versus PLD, OLA versus PLD, or PAT versus PLD subgroups (Analysis 2.1).  C. PLD plus other drug/s versus PLD alone:  TBD/PLD versus PLD (one study, 672 participants): PFS was significantly longer in the combination arm compared with PLD alone (HR 0.79, 95%CI 0.65 to 0.96; P value 0.02; Analysis 3.1). EC145/PLD versus PLD (one study, 149 participants): PFS was significantly longer in the combination arm compared with PLD alone (HR 0.63, 95%CI 0.41 to 0.97; P value 0.04; Analysis 3.1).  **Where reported:** Results text.  **Interaction exists based on reported results:** Yes for comparison 1. Not reported for comparisons 2 and 3.  **How determined:** Comparison 1. PLD/carboplatin vs carboplatin ± other drug/s**:** Test for subgroup differences: Chi2= 3.10, df= 1 (P= 0.08), I2=68%.  **Reported importance of the interaction:** Not reported.  **What reported:** Not reported.  **Where reported:** NA  **Reported plausibility of the interaction:** Not reported.  **What reported:** Not reported.  **Where reported:** NA  **Reported confounding possibility:** Not reported.  **What reported:** Not reported.  **Where reported:** NA.  **Mentioned covariate distribution:** Yes.  **What reported:** as above.  **Where reported:** Results text. |
| Lopez 2013 | **Protocol:** Not reported.  **Review methods:** Not reported.  **Review results:** Time point. ‘Use of contraceptives (non-condom) per visit’; ‘New use of modern contraceptives (excluding condoms)’; ‘New use of condoms’; ‘Reported new use of contraceptives per 100 women-years’; ‘Use of modern contraceptive method at 12 to 14 months’; ‘Reported differences in use of modern contraceptives (baseline to follow-up)’; ‘Use of hormonal contraceptives at 1 year (Oral contraceptives)’; ‘Use of hormonal contraceptives at 1 year (Injectable or oral contraceptives)’; ‘Discontinued chosen hormonal contraceptive by 1 year’; ‘Use of condoms or spermicide at 1 year (Reported use of condoms at 1 year)’; ‘Use of condoms or spermicide at 1 year (Recorded using condoms at 1 year)’; ‘Use of condoms or spermicide at 1 year (Recorded using spermicide at 1 year)’; ‘Use of hormonal contraceptives after intervention (pill)’; ‘Use of hormonal contraceptives after intervention (Injected )’; ‘Use of hormonal contraceptives after intervention (Implant)’; ‘Use of modern contraceptive (injectable, IUD, pills, condoms) Use at month 6’; ‘Use of modern contraceptive (injectable, IUD, pills, condoms) Use at month 12’; ‘Use of modern contraceptive (injectable, IUD, pills, condoms) Use at month 24’; ‘Use of modern contraceptives (Reported hazard ratios from multivariate Cox models)’; ‘Reported percentages for consistent condom use (at 3 months, 6 months, 18 months)’.  **Type:** Outcome.  **Covariate summary:** Time point. | Contraception use, e.g., choice, uptake, or initiation of a new method; improved use or continuation of current method; primary; dichotomous and count; odds ratio, rate ratio and risk difference; NT=7; NP=10,311. | **Number of trials:** 1.  **Covariate distribution (across trials):**  NT=1, NP=899 (6. Family planning counseling and free contraceptives: HIV+ versus HIV- women):  NT=1, NP=899 ( Use of modern contraceptive (injectable, IUD, pills, condoms) Use at month 6);  NT=1, NP=851 ( Use of modern contraceptive (injectable, IUD, pills, condoms) Use at month 12);  NT=1, NP=751 ( Use of modern contraceptive (injectable, IUD, pills, condoms) Use at month 24).  **Covariate distribution (within trials):** NA. | **Analysis type in review according to glossary 1:** Subgroup analysis.  **Reported results:** Event rates, OR, and CI for trials. OR, CI, z-statistic,and p-value for meta-analytic subtotals.  **Where reported:** Results text and forest plot.  **Method to detect interactions reported in review:** Not reported.  **Reported results from methods to detect interactions:** Test for subgroup differences (Chi-square statistic, p-value, and I square statistic).  **Where reported:** Forest plot. | **Reported detected interaction:** Not reported.  **What reported:** ‘For unadjusted use of modern contraceptives, the HIV-positive and HIV-negative groups in Brou 2009 did not differ significantly at month 6 (Analysis 6.1). However, women in the HIV-negative group were more likely to be using modern contraceptives at month 12 (OR 0.62; 95% CI 0.46 to 0.84) and month 24 (OR 0.53; 95% CI 0.40 to 0.72) (Analysis 6.1). The subgroup analysis showed heterogeneity (I2 = 72.5%). The HIV-positive group lost proportionately more women than the HIV-negative group (nearly 20% versus 13%, respectively). Also, the HIV-negative group had proportionately more IUD users at the later times. Project staff members were not recommending the IUD for HIV positive women due to the risk of infection.’  **Where reported:** Results text.  **Interaction exists based on reported results:** Yes  **How determined:** Test for subgroup differences: Chi2 = 7.27, df = 2 (P = 0.03), I2 =73%  **Reported importance of the interaction:** Not reported.  **What reported:** Not reported.  **Where reported:** NA  **Reported plausibility of the interaction:** Not reported.  **What reported:** Not reported.  **Where reported:** NA  **Reported confounding possibility:** Not reported.  **What reported:** Not reported.  **Where reported:** NA.  **Mentioned covariate distribution:** Not reported.  **What reported:** Not reported.  **Where reported:** NA. |
| Lopez 2013 | **Protocol:** Not reported.  **Review methods:** Not reported.  **Review results: ‘**Comparison. Family planning intervention versus usual care’; ‘Integrated family planning versus routine care’; ‘Integrated family planning services: enhanced versus basic’; ‘Family planning counseling (HIV+ women) versus comparison (HIV- women)’; ‘Informational video + discussion: HIV+ versus HIV- women’; ‘Family planning counseling and free contraceptives: HIV+ versus HIV- women’ (in plot). ‘Integrated family planning services versus usual services or basic integration’; ‘HIV-positive versus HIV-negative women’.  **Type:** Intervention.  **Covariate summary:** Type of intervention or control. | Contraception use, e.g., choice, uptake, or initiation of a new method; improved use or continuation of current method; primary; dichotomous and count; odds ratio, rate ratio and risk difference; NT=7; NP=10,311. | **Number of trials:** 7.  **Covariate distribution (across trials):**  NT=1, NP=3,463 (1. Family planning intervention versus usual care);  NT=1, NP=4,031 (2. Integrated family planning versus routine care);  NT=1, NP=252 (3. Integrated family planning services: enhanced versus basic);  NT=2, NP=1,499 (4. Family planning counseling (HIV+ women) versus comparison (HIV- women));  NT=1, NP=502 (5. Informational video + discussion: HIV+ versus HIV- women;)  NT=1, NP=899 (6. Family planning counseling and free contraceptives: HIV+ versus HIV- women.)  Text is stratified by:  NT=3, NP=6,818 (1 ‘Integrated family planning services versus usual services or basic integration’);  NT=4, NP=3,087 (2 ‘HIV-positive versus HIV-negative women’).  **Covariate distribution (within trials):** NA. | **Analysis type in review according to glossary 1:** Subgroup analysis.  **Reported results:**  Event rates, OR, and CI for trials. OR, CI, z-statistic,and p-value for meta-analytic subtotals. Incident rate and CI by group and attributable risk and CI. % by group, or % by group and % difference and a p-value.  **Where reported:** Results text and forest plot.  **Method to detect interactions reported in review:** Not reported.  **Reported results from methods to detect interactions:** Not reported.  **Where reported:** NA. | **Reported detected interaction:** Not reported.  **What reported:**  Integrated family planning services versus usual services or basic integration:  In Ngure 2009, use of non-condom contraceptives was reported per visit rather than per woman. The time period was after the intervention began. Results are presented for the HIV-positive women. At the intervention site, use of non-condom contraceptives was more likely than at the comparison sites during the same time period (OR 6.40; 95% CI 5.37 to 7.62) (Analysis 1.1). The investigators reported the results as OR 6.4 (95% CI 4.6 to 8.9). Condom use (per sex act) was reportedly high during follow-up for both HIV-positive (88%) and HIV-negative (91%) women from all sites.  Mean follow-up time in Kosgei 2011 was approximately 11.4 months for the group receiving integrated family planning and 12 months for the group with routine care. All women were HIVpositive. The researchers focused on new use of modern contraceptives for pregnancy prevention. New use of modern contraceptives (excluding condoms) was less likely for the group receiving integrated services than for the group with routine care (OR 0.56; 95% CI 0.42 to 0.75) (Analysis 2.1), based on unadjusted analysis. The reported adjusted incidence difference was 3.8%, indicating significantly lower incidence for the integrated-services group compared to the routine-care group. Reported incident rates per 100 women-years were low: 4.8 (95%CI 3.7 to 6.0) for integrated services and 7.8 (95%CI 6.8 to 8.9) for routine care. The reported attributable risk for new use of modern contraceptives (excluding condoms) was -3.0 (95% CI -4.6 to -1.4) for integrated services versus routine care (Analysis 2.3). In contrast, new use of condoms was more likely for the integrated services group compared to those with routine services (OR 1.73; 95% CI 1.52 to 1.98) (Analysis 2.2). The reported attributable risk was 16.4 (95% CI 11.9 to 21.0) (Analysis 2.3).  Use of modern contraceptives was assessed at follow-up (12 to 14 months) in McCarraher 2011. All women were HIV-positive. Women at the sites with enhanced integrated services were more likely to use modern contraceptives at follow-up compared to women at sites with basic integrated services (OR 2.48; 95% CI 1.31 to 4.72) (Analysis 3.1), based on unadjusted comparisons. Differences from baseline to follow-up were reported for each group. The adjusted differences in use of modern contraceptives were 11.0% and 11.6% for the enhanced and basic groups, respectively (Analysis 3.2). The investigators found that the 0.6% difference between the groups was not statistically significant.  HIV-positive versus HIV-negative women:  The interventions were similar for Temmerman 1990 and Allen 1993, but we did not pool the data due to different outcome measures and time frames for assessment. At one year, the HIVpositive and HIV-negative groups were not significantly different for use of oral contraceptives in Temmerman 1990 (OR 1.04; 95% CI 0.31 to 3.53) (Analysis 4.1). For Allen 1993, the groups were not significantly different for use of hormonal contraceptives (OR 0.87; 95%CI 0.63 to 1.21) (Analysis 4.1). TheHIV-positive women in Allen 1993 were more likely to have discontinued their chosenmethod of contraception (OR 2.52; 95% CI 1.53 to 4.14) (Analysis 4.2).  For use of condoms in Temmerman 1990, the study groups did not differ significantly (OR 1.41; 95%CI 0.18 to 10.78) (Analysis 4.3). However, in a 1992 article, Allen 1993 showed the HIVpositive women were more likely to be using condoms (OR 2.82; 95% CI 2.18 to 3.65) and spermicide (OR 2.36; 95% CI 1.69 to 3.30) at one year (Analysis 4.3).  Many of the women in King 1995 had also been part of Allen 1993. The family planning intervention had been very limited in Allen 1993, and had only been provided to HIV-positive women. The mean follow-up time in King 1995 for use of hormonal contraceptives was 5.4 months. The HIV-positive and HIV-negative groups did not differ significantly for use of oral contraceptive pills (OR 0.97; 95% CI 0.52 to 1.84), injectables (OR 0.86; 95% CI 0.49 to 1.51), or implants (OR 3.49; 95% CI 0.78 to 15.63) (Analysis 5.1). The researchers examined new users but not by HIV status.  For unadjusted use of modern contraceptives, the HIV-positive andHIV-negative groups in Brou 2009 did not differ significantly at month 6 (Analysis 6.1). However, women in the HIV-negative group were more likely to be using modern contraceptives at month 12 (OR 0.62; 95% CI 0.46 to 0.84) and month 24 (OR 0.53; 95% CI 0.40 to 0.72) (Analysis 6.1). The subgroup analysis showed heterogeneity (I2 = 72.5%). The HIV-positive group lost proportionately more women than the HIV-negative group (nearly 20% versus 13%, respectively). Also, the HIV-negative group had proportionatelymore IUDusers at the later times. Project staff members were not recommending the IUD for HIVpositive women due to the risk of infection. The reported hazard ratio from adjusted analysis showed no significant difference between the groups for modern contraceptive use (reported HR 0.90; 95% CI 0.72 to 1.14) (Analysis 6.2). Also from reported analyses, the HIV-positive and HIV-negative groups did not differ significantly for consistent condom use at months 3, 12, or 18 (Analysis 6.3).  **Where reported:** reults text.  **Interaction exists based on reported results:** Not reported.  **How determined:** NA.  **Reported importance of the interaction:** Not reported.  **What reported:** Not reported.  **Where reported:** NA  **Reported plausibility of the interaction:** Not reported.  **What reported:** Not reported.  **Where reported:** NA  **Reported confounding possibility:** Not reported.  **What reported:** Not reported.  **Where reported:** NA.  **Mentioned covariate distribution:** Not reported.  **What reported:** Not reported.  **Where reported:** NA. |
| Mocellin 2013 | **Protocol:** ‘Interferon dosage’.  **Review methods: ‘**Interferon dosage’.  **Review results: ‘**Interferon dosage’. ‘High’; ‘low’; ‘Intermediate’.  **Type:** Intervention.  **Covariate summary:** Dose. | disease-free survival; primary; time to event; hazard ratio; NT=17; NP=10,345. | **Number of trials:** 17.  **Covariate distribution (across trials):**  NT=8, NP=5,220 (high);  NT=8, NP=3,793 (low);  NT=2, NP=2,243 (intermediate).  **Covariate distribution (within trials):** NA. | **Analysis type in review according to glossary 1:** Subgroup analysis.  **Reported results:** HR, CI and p-value for meta-analytic subtotals.  **Where reported:** Results text and table.  **Method to detect interactions reported in review:** Not reported.  **Reported results from methods to detect interactions:** Not reported.  **Where reported:** NA. | **Reported detected interaction:** No interaction.  **What reported:**  ‘Overall, we found that none of the assessable factors we considered (interferon dose, TNMstage, year of publication, and treatment duration) significantly affected the impact of interferon on participants’ DFS’  **Where reported:** Results text.  **Interaction exists based on reported results:** Not reported..  **How determined:** NA.  **Reported importance of the interaction:** Not reported.  **What reported:** Not reported.  **Where reported:** NA  **Reported plausibility of the interaction:** Not reported.  **What reported:** Not reported.  **Where reported:** NA  **Reported confounding possibility:** Not reported.  **What reported:** Not reported.  **Where reported:** NA.  **Mentioned covariate distribution:** Not reported.  **What reported:** Not reported.  **Where reported:** NA. |
| Mocellin 2013 | **Protocol:** ‘Participants’ TNM stage’.  **Review methods: ‘**Participants’ AJCC TNM stage (studies have been conducted with the 5th and 6th edition of the TNM staging manual)’.  **Review results: ‘**TNM stage’. ‘Stage II’; ‘stage III’; ‘stage II-II)’.  **Type:** Patient.  **Covariate summary:** Disease characteristics. | disease-free survival; primary; time to event; hazard ratio; NT=17; NP=10,345. | **Number of trials:** 17.  **Covariate distribution (across trials):**  NT=2, NP=780 (II);  NT=5, NP=4,065 (III);  NT=10, NP=5,803 (II-III).  **Covariate distribution (within trials):**  100% II (2 trials);  100% III (5 trial);  100% II-III (10 trials). | **Analysis type in review according to glossary 1:** Subgroup analysis.  **Reported results:** HR, CI and p-value for meta-analytic subtotals.  **Where reported:** Results text and table.  **Method to detect interactions reported in review:** Not reported.  **Reported results from methods to detect interactions:** Not reported.  **Where reported:** NA. | **Reported detected interaction:** No interaction.  **What reported:**  ‘Overall, we found that none of the assessable factors we considered (interferon dose, TNM stage, year of publication, and treatment duration) significantly affected the impact of interferon on participants’ DFS.’  **Where reported:** Results text.  **Interaction exists based on reported results**: Not reported.  **How determined:** NA.  **Reported importance of the interaction:** Not reported.  **What reported:** Not reported.  **Where reported:** NA  **Reported plausibility of the interaction:** Not reported.  **What reported:** Not reported.  **Where reported:** NA  **Reported confounding possibility:** Not reported.  **What reported:** Not reported.  **Where reported:** NA.  **Mentioned covariate distribution:** Not reported.  **What reported:** Not reported.  **Where reported:** NA. |
| Mocellin 2013 | **Protocol: ‘**Treatment duration’.  **Review methods: ‘**Treatment duration’.  **Review results: ‘**Treatment duration’.  **Type:** Intervention.  **Covariate summary:** Duration of intervention. | disease-free survival; primary; time to event; hazard ratio; NT=17; NP=10,345. | **Number of trials:** 17.  **Covariate distribution (across trials):**  From characteristics tables:  NT=1, NP=1,256 (5 years 8 weeks);  NT=1, NP=444 (3 years);  NT=2, NP=2,243 (16 months or 28 months);  NT=2, NP=1,074 (2 year);  NT=1, NP=487 (18 months);  NT=7,NP=3,636 (1 year);  NT=1, NP=94 (6 months);  NT=1, NP=264 (4 months);  NT=1, NP=1,150 (4 weeks).  **Covariate distribution (within trials):** NA. | **Analysis type in review according to glossary 1:** Meta-regression.  **Reported results:** Regression coefficient, CI, p-value.  **Where reported:** Table.  **Method to detect interactions reported in review:** Not reported.  **Reported results from methods to detect interactions:** Regression coefficient, and p-value.  **Where reported:** Table. | **Reported detected interaction:** No interaction.  **What reported:**  ‘Overall, we found that none of the assessable factors we considered (interferon dose, TNM stage, year of publication, and treatment duration) significantly affected the impact of interferon on participants’ DFS’  **Where reported:** Results text.  **Interaction exists based on reported results**: Not reported.  **How determined:** NA.  **Reported importance of the interaction:** Not reported.  **What reported:** Not reported.  **Where reported:** NA  **Reported plausibility of the interaction:** Not reported.  **What reported:** Not reported.  **Where reported:** NA  **Reported confounding possibility:** Not reported.  **What reported:** Not reported.  **Where reported:** NA.  **Mentioned covariate distribution:** Not reported.  **What reported:** Not reported.  **Where reported:** NA. |
| Mocellin 2013 | **Protocol:** ‘Year of publication’.  **Review methods:** ‘Year of publication’.  **Review results: ‘**Publication year’.  **Type:** Other.  **Covariate summary:** Year of publication. | disease-free survival; primary; time to event; hazard ratio; NT=17; NP=10,345. | **Number of trials:** 17.  **Covariate distribution (across trials):**  From characteristics tables:  NT=2, NP=2,005 (2011);  NT=3, NP=2,471 (2008);  NT=1, NP=1,388 (2005);  NT=2, NP=1,426 (2004);  NT=4, NP=1,419 (2001);  NT=1, NP=608 (2000);  NT=2, NP=780 (1998);  NT=1, NP=287 (1996);  NT=1, NP=264 (1995).  **Covariate distribution (within trials):** NA. | **Analysis type in review according to glossary 1:** Meta-regression.  **Reported results:** Regression coefficient, CI, p-value.  **Where reported:** Table.  **Method to detect interactions reported in review:** Not reported.  **Reported results from methods to detect interactions:** Regression coefficient and p-value.  **Where reported:** Table. | **Reported detected interaction:** No interaction.  **What reported:**  Overall, we found that none of the assessable factors we considered (interferon dose, TNM stage, year of publication, and treatment duration) significantly affected the impact of interferon on participants’ DFS  **Where reported:** Review text.  **Interaction exists based on reported results:** Not reported.  **How determined:** NA.  **Reported importance of the interaction:** Not reported.  **What reported:** Not reported.  **Where reported:** NA  **Reported plausibility of the interaction:** Not reported.  **What reported:** Not reported.  **Where reported:** NA  **Reported confounding possibility:** Not reported.  **What reported:** Not reported.  **Where reported:** NA.  **Mentioned covariate distribution:** Not reported.  **What reported:** Not reported.  **Where reported:** NA. |
| Mocellin 2013 | **Protocol:** Not reported.  **Review methods:** Sample size. We judged the following to be two covariates: ‘We employed the exclusion of randomised studies for specific reasons (e. g. differences in control arms or sample size) as a further type of sensitivity analysis.’ **‘**In order to assess the impact of “small” studies, we also performed a sensitivity analysis (see Sensitivity analysis) by excluding these trials. To this aim, we defined “small” as any study with a sample size smaller than 400 based on the following parameters of an ideal trial: A) minimum hazard ratio (HR) = 1. 5; alpha level of significance = 5%; statistical power = 80%; median survival in controls = 60 months; accrual time = 36 months; follow-up time = 48 months; randomisation ratio = 1:1’.  **Review results: ‘**Exclusion of RCTs enrolling fewer than 400 participants’.  **Type:** Methodological.  **Covariate summary:** Trial size. | disease-free survival; primary; time to event; hazard ratio; NT=17; NP=10,345. | **Number of trials:** 11.  **Covariate distribution (across trials):**  NT=11, NP=8,829 (after exclusion of RCTs enrolling fewer than 400 participants).  **Covariate distribution (within trials):** NA. | **Analysis type in review according to glossary 1:** Sensitivity analysis.  **Reported results:** HR, CI and p-value for meta-analytic subtotal.  **Where reported:** Results text.  **Method to detect interactions reported in review:** Not reported.  **Reported results from methods to detect interactions:** Not reported.  **Where reported:** NA. | **Reported detected interaction:** No interaction.  **What reported:**  ‘On the other hand, upon exclusion of RCTs enrolling fewer than 400 participants (Cameron 2001; Creagan 1995; Kirkwood 1996; Kirkwood 2001a; McMasters 2008; Pehamberger 1998), the meta-analysis yielded results very similar to those obtained including all RCTs (HR = 0.85; CI 0.80 to 0.90; Z-test P value < 0.0001; I² statistic = 20%; Q-test P value = 0.25).’  **Where reported:** Results text.  **Interaction exists based on reported results**: Not reported.  **How determined:** NA.  **Reported importance of the interaction:** Not reported.  **What reported:** Not reported.  **Where reported:** NA  **Reported plausibility of the interaction:** Not reported.  **What reported:** Not reported.  **Where reported:** NA  **Reported confounding possibility:** Not reported.  **What reported:** Not reported.  **Where reported:** NA.  **Mentioned covariate distribution:** Not reported.  **What reported:** Not reported.  **Where reported:** NA. |
| Mocellin 2013 | **Protocol:** Not reported.  **Review methods: ‘**Differences in control arms. We judged the following to be two analyses: ‘We employed the exclusion of randomised studies for specific reasons (e. g. differences in control arms or sample size) as a further type of sensitivity analysis.’  **Review results:** ‘Exclusion of two studies with an active control arm’.  **Type:** Intervention.  **Covariate summary:** Type of intervention or control. | disease-free survival; primary; time to event; hazard ratio; NT=17; NP=10,345. | **Number of trials:** 15.  **Covariate distribution (across trials):**  NT=15, NP=9,464 (observation is control).  **Covariate distribution (within trials):** NA. | **Analysis type in review according to glossary 1:** Sensitivity analysis.  **Reported results:** HR, CI and p-value for meta-analytic subtotal.  **Where reported:** Results text.  **Method to detect interactions reported in review:** Not reported.  **Reported results from methods to detect interactions:** Not reported.  **Where reported:** NA. | **Reported detected interaction:** No interaction.  **What reported:**  ‘Upon exclusion of the 2 RCTs with an active control arm (Kirkwood 2001; Kirkwood 2001a), the results were very similar to those obtained including all 17 RCTs (HR = 0.84; CI 0.79 to 0.89; Z-test P value < 0.0001; I² statistic = 2%, Q-test P value =0.43).’  **Where reported:** Review text.  **Interaction exists based on reported results:** Not reported.  **How determined:** NA.  **Reported importance of the interaction:** Not reported.  **What reported:** Not reported.  **Where reported:** NA  **Reported plausibility of the interaction:** Not reported.  **What reported:** Not reported.  **Where reported:** NA  **Reported confounding possibility:** Not reported.  **What reported:** Not reported.  **Where reported:** NA.  **Mentioned covariate distribution**: Yes.  **What reported:** as above.  **Where reported:** Results text. |
| Mutua 2012 | **Protocol:** Not reported.  **Review methods:** Not reported.  **Review results:** ‘Penicillin vs single-dose azithromycin’; ‘Penicillin vs two-dose azithromycin’; ‘Fleroxacin 400 mg vs fleroxacin 200 mg’; ‘ Fleroxacin 400 mg vs TMP-SMZ’.  **Type:** Intervention.  **Covariate summary:** Type of intervention or control and dose. | Incidence of HIV infection; primary; dichotomous; risk ratio; NT=3; NP=173. | **Number of trials:** 3.  **Covariate distribution (across trials):**  NT=1, NP=19 (Penicillin vs single-dose azithromycin);  NT=1, NP=22 (Penicillin vs two-dose azithromycin);  NT=1, NP=45 (Fleroxacin 400 mg vs fleroxacin 200 mg);  NT=1, NP=98 (Fleroxacin 400 mg vs TMP-SMZ).  **Covariate distribution (within trials):** NA. | **Analysis type in review according to glossary 1:** Subgroup analysis.  **Reported results:** Event rates, risk ratio, and CI for trials.  **Where reported:** Results text and forest plot.  **Method to detect interactions reported in review:** Not reported.  **Reported results from methods to detect interactions:** Not reported.  **Where reported:** NA. | **Reported detected interaction:** Not reported.  **What reported:**  ‘Treatment of syphilis  There were three treatment arms in the trial by Hook et al (Hook 2002), one that received benzathine penicillinGand the other two received azithromycin in two different doses. There were no HIV seroconversions by the end of the 12-month study period (Analysis 1.1; Analysis 1.2; Summary of findings for the main comparison).  Treatment of chancroid  Two trials focused on treatment of chancroid (MacDonald 1989; Plourde 1992).  In the MacDonald 1989 study, two of the 18 participants on 400 mg fleroxacin and one of the 27 participants receiving 200 mg fleroxacin tested HIV positive by the last follow-up visit on day  28 (RR 3.00; 95% CI 0.29 to 30.69; Analysis 2.1; Summary of findings 2).  ....  Of the 12 participants included in the final analyses in the Plourde 1992 study, one in the fleroxacin arm and three in the TMPSMZ arm seroconverted by the end of eight weeks. There were no significant differences in the rate of HIV seroconversion at eight to 12 weeks in the two treatment arms (RR 0.33; 95% CI 0.04 to 3.09; Analysis 2.2; Summary of findings 2).’  **Where reported:** Results text.  **Interaction exists based on reported results:** Not reported.  **How determined:** NA.  **Reported importance of the interaction:** Not reported.  **What reported:** Not reported.  **Where reported:** NA  **Reported plausibility of the interaction:** Not reported.  **What reported:** Not reported.  **Where reported:** NA  **Reported confounding possibility:** Not reported.  **What reported:** Not reported.  **Where reported:** NA.  **Mentioned covariate distribution:** Yes.  **What reported:** as above.  **Where reported:** Results text. |
| Mutua 2012 | **Protocol:** Not reported.  **Review methods: ‘**Underlying cause of the ulcer’.  **Review results:** ‘Treatment of syphilis’; ‘treatment of chancroid’.  **Type:** Patient.  **Covariate summary:** Disease characteristics. | Incidence of HIV infection; primary; dichotomous; risk ratio; NT=3; NP=173. | **Number of trials:** 3.  **Covariate distribution (across trials):**  NT=1, NP=30 (‘Treatment of syphilis’);  NT=2, NP=143 (‘treatment of chancroid’).  **Covariate distribution (within trials):**  syphilis (1 trial-100% patients);  chancroid (2 trial-100% patients). | **Analysis type in review according to glossary 1:** Subgroup analysis.  **Reported results:** Event rates, risk ratio, and CI for trials.  **Where reported:** Results text and forest plot.  **Method to detect interactions reported in review:** Not reported.  **Reported results from methods to detect interactions:** Not reported.  **Where reported:** NA. | **Reported detected interaction:** Not reported.  **What reported:**  ‘Treatment of syphilis  There were three treatment arms in the trial by Hook et al (Hook 2002), one that received benzathine penicillinGand the other two received azithromycin in two different doses. There were no HIV seroconversions by the end of the 12-month study period (Analysis 1.1; Analysis 1.2; Summary of findings for the main comparison).  Treatment of chancroid  Two trials focused on treatment of chancroid (MacDonald 1989; Plourde 1992).  In the MacDonald 1989 study, two of the 18 participants on 400 mg fleroxacin and one of the 27 participants receiving 200 mg fleroxacin tested HIV positive by the last follow-up visit on day  28 (RR 3.00; 95% CI 0.29 to 30.69; Analysis 2.1; Summary of findings 2).  ....  Of the 12 participants included in the final analyses in the Plourde 1992 study, one in the fleroxacin arm and three in the TMPSMZ arm seroconverted by the end of eight weeks. There were no significant differences in the rate of HIV seroconversion at eight to 12 weeks in the two treatment arms (RR 0.33; 95% CI 0.04 to 3.09; Analysis 2.2; Summary of findings 2).’  **Where reported:** Results text.  **Interaction exists based on reported results:** Not reported.  **How determined:** NA.  **Reported importance of the interaction:** Not reported.  **What reported:** Not reported.  **Where reported:** NA  **Reported plausibility of the interaction:** Not reported.  **What reported:** Not reported.  **Where reported:** NA  **Reported confounding possibility:** Not reported.  **What reported:** Not reported.  **Where reported:** NA.  **Mentioned covariate distribution:** Yes.  **What reported:** as above.  **Where reported:** Results text. |
| Peters 2013 | **Protocol:** Not reported.  **Review methods:** Not reported.  **Review results:** Comparison. In plot: ‘1. Low-level laser versus placebo’; ‘2. Immobilisation (wrist splint) versus mobilisation (bulky dressing)’; ‘3. Immobilisation (plaster of Paris splint) versus bulky dressing and mobilisation’; ‘4. Specialised home elevation device versus standard sling’; ‘5. Controlled cold therapy versus ice therapy’; ‘6. Bulky dressing plus splint versus light dressing’; ‘7. Contrast bath plus exercise versus contrast bath’; ‘8. Contrast bath plus exercises versus exercise’; ‘9. Contrast bath versus exercise’; ‘10. Arnica versus placebo’; ‘11. High dose arnica oral tablets versus placebo’; ‘12. Low dose arnica tablets versus placebo’; ‘13. High dose versus low dose oral arnica tablets’; ‘14. Multimodal hand therapy versus normal activities/exercise’; ‘15. Desensitisation therapy (as part of multiple interventions) versus no treatment’; ‘16. Short duration dressing versus extended duration dressing. In text: Low-level laser therapy (single intervention) versus “sham” therapy (placebo)’; ‘Immobilisation (single intervention) versus bulky dressing (single intervention)’; ‘Immobilisation (single intervention) versus mobilisation (multiple interventions)’; ‘Elevation (single intervention) versus standard care (control)’; ‘Electrical stimulation (single intervention) versus no treatment (control)’; ‘Controlled cold therapy and narcotic use (multiple interventions) versus ice therapy plus narcotic use (multiple interventions)’; ‘Bulky dressing and splint (single intervention) versus light dressing (single intervention)’; ‘Contrast baths plus exercise (multiple interventions) versus contrast baths (single intervention) versus exercise (single intervention)’; ‘Arnica (single intervention) versus “sham” therapy (placebo)’; ‘High dose arnica (single intervention) versus low dose arnica (single intervention) versus “sham” therapy (placebo)’; ‘Electrical stimulation versus decimer wave therapy versus combined therapy versus control’; ‘Multimodal hand therapy (multiple interventions) versus no formal therapy (control)’; ‘Desensitisation therapy (as part of multiple interventions) versus standard treatment (control)’; ‘Multimodal therapy (multiple interventions) versus progressive patient-directed home exercise program (single intervention)’; ‘Short duration postoperative dressing (single intervention) versus extended duration postoperative dressing (single intervention)’.  **Type:** Intervention.  **Covariate summary:** Type of intervention or control. | Long-term change in self reported functional ability; primary; continuous; mean difference or no results were reported; NT=6; NP=394. | **Number of trials:** 3.  **Covariate distribution (across trials):**  In analyses:  NT=0, NP=0 (1. Low-level laser versus placebo);  NT=0, NP=0 (2. Immobilisation (wrist splint) versus mobilisation (bulky dressing));  NT=1, NP=40 (3. Immobilisation (plaster of Paris splint) versus bulky dressing and mobilisation);  NT=0, NP=0 (4. Specialised home elevation device versus standard sling);  NT=0, NP=0 (5. Controlled cold therapy versus ice therapy);  NT=0, NP=0 (6. Bulky dressing plus splint versus light dressing);  NT=0, NP=0 (7. Contrast bath plus exercise versus contrast bath);  NT=0, NP=0 (8. Contrast bath plus exercises versus exercise);  NT=0, NP=0 (9. Contrast bath versus exercise);  NT=0, NP=0 (10. Arnica versus placebo);  NT=0, NP=0 (11. High dose arnica oral tablets versus placebo);  NT=0, NP=0 (12. Low dose arnica tablets versus placebo);  NT=0, NP=0 (13. High dose versus low dose oral arnica tablets);  NT=1, NP=150 (14. Multimodal hand therapy versus normal activities/exercise);  NT=1, NP=27 (15. Desensitisation therapy (as part of multiple interventions) versus no treatment);  NT=0, NP=0 (16. Short duration dressing versus extended duration dressing).  In the text:  NT=0, NP=0 (Low-level laser therapy (single intervention) versus “sham” therapy (placebo));  NT=0, NP=0 (Immobilisation (single intervention) versus bulky dressing (single intervention));  NT=1, NP=40 (Immobilisation (single intervention) versus mobilisation (multiple interventions));  NT=0, NP=0 (Elevation (single intervention) versus standard care (control));  NT=0, NP=0 (Electrical stimulation (single intervention) versus no treatment (control));  NT=0, NP=0 (Controlled cold therapy and narcotic use (multiple interventions) versus ice therapy plus narcotic use (multiple interventions));  NT=0, NP=0 (Bulky dressing and splint (single intervention) versus light dressing (single intervention));  NT=0, NP=0 (Contrast baths plus exercise (multiple interventions) versus contrast baths (single intervention) versus exercise (single intervention));  NT=0, NP=0 (Arnica (single intervention) versus “sham” therapy (placebo));  NT=0, NP=0 (High dose arnica (single intervention) versus low dose arnica (single intervention) versus “sham” therapy (placebo));  NT=0, NP=0 (Electrical stimulation versus decimer wave therapy versus combined therapy versus control);  NT=1, NP=150 (Multimodal hand therapy (multiple interventions) versus no formal therapy (control));  NT=1, NP=27 (Desensitisation therapy (as part of multiple interventions) versus standard treatment (control));  NT=0, NP=0 (Multimodal therapy (multiple interventions) versus progressive patient-directed home exercise program (single intervention));  NT=0, NP=0 (Short duration postoperative dressing (single intervention) versus extended duration postoperative dressing (single intervention).  **Covariate distribution (within trials):** NA. | **Analysis type in review according to glossary 1:** Subgroup analysis.  **Reported results:** Mean, SD and N by group for trials. Mean difference and CI for trials. Mean difference, CI, Z statistic, and p-value for meta-analytic subtotal.  **Where reported:** Results text and forest plot.  **Method to detect interactions reported in review:** Not reported.  **Reported results from methods to detect interactions:** Not reported.  **Where reported:** NA. | **Reported detected interaction:** Not reported.  **What reported:**  Immobilisation (single intervention) versus mobilisation (multiple interventions):  Four trials investigated immobilisation versus mobilisation ( Cebesoy 2007; Cook 1995; Finsen 1999; Martins 2006). One quasi-randomised trial (Cebesoy 2007) investigated immobilisation (using a plaster wrist orthosis worn until suture removal) and latemobilisation (bulky dressing worn for a three-week period and immediate early mobilisation exercises). Cook 1995 randomly allocated participants to immobilisation using a volar wrist orthosis for two weeks versus bulky dressing and advice to move the hand and wrist with no restrictions. One quasi-randomised trial (Finsen 1999) compared the use of a plaster wrist orthosis for two weeks versus light dressing and active mobilisation within comfort from 48 hours post surgery. Martins 2006 investigated the efficacy of immobilisation using a neutral wrist orthosis for two weeks post surgery versus no orthosis and unrestricted movement of the wrist and fingers. Cebesoy 2007, Cook 1995, Finsen 1999 and Martins 2006 examined the effects of immobilisation using a wrist orthosis when comparedwithmobilisation using activemovement of the affected limb commenced immediately postoperatively (Cebesoy 2007) or on the first (Cook 1995) or second (Finsen 1999; Martins 2006) postoperative day. The duration of orthotic use between trials was different: Cebesoy 2007 immobilised the wrists for ten days, Cook 1995 and Martins 2006 immobilised the affected wrists for two weeks, whilst another trial (Finsen 1999) immobilised the affected wrists for four weeks. However, in Finsen 1999 the trialists reported six deviations from protocol in which people were splinted for either a shorter or longer time.  Primary outcomes  1. Long-termchange in self reported functional ability as measured  on a continuous scale at three months or longer  • Reported in Cebesoy 2007  • Function was assessed using the Functional Severity Scale on the BCTQ preoperatively and three months post surgery in Cebesoy 2007. There was no statistically significant difference in function between participants receiving bulky dressing and early mobilisation compared to those receiving splint and late mobilisation at three months post surgery (Analysis 3.1).  Multimodal hand therapy (multiple interventions) versus no formal therapy (control)  Pomerance 2007 examined the effects of a formal programofmultimodal hand therapy (consisting of six 30 min sessions of nerve gliding, range of motion and strengthening and additional treatments, for example, massage, fluidotherapy with a qualified hand therapist). Treatments were provided over a two-week period starting at five to seven days post surgery. The control group received advice regarding tendon gliding exercises and scar massage prior to surgery but no formal therapy after surgery. A total of 150 participants were randomised. Outcomes included time to return to work, pain intensity, lateral pinch strength, grip strength, function using the DASH questionnaire, persistence of symptoms, wound dehiscence, and an economic evaluation of the intervention.  Primary outcomes  1. Long-termchange in self reported functional ability as measured on a continuous scale at three months or longer  • DASH was measured at five and a half months posttreatment (six months post surgery). There was no statistically significant difference between treatments (Analysis 14.1).  Desensitisation therapy (as part of multiple interventions) versus standard treatment (control)  The Powell 2003 unpublished study randomly allocated 29 participants to either a graduated desensitisation program for three months or a standard treatment control.Outcomesweremeasured at three and sixweeks, and threemonths post operation.Outcomes included scar sensitivity using a dolorimeter (pressure gauge), patient- reported scar sensitivity, functional status using the BCTQ Functional Status Scale, and grip strength.  Primary outcomes   - Long-termchange in self reported functional ability asmeasured on a continuous scale at three months or longer   • BCTQ Functional Status Scale was used to record functional ability at three months. There was no statistically significant benefit of graded desensitisation over standard treatment (Analysis 15.1)  **Where reported:** Results text.  **Interaction exists based on reported results:** Not reported.  **How determined:** NA.  **Reported importance of the interaction:** Not reported.  **What reported:** Not reported.  **Where reported:** NA  **Reported plausibility of the interaction:** Not reported.  **What reported:** Not reported.  **Where reported:** NA  **Reported confounding possibility:** Not reported.  **What reported:** Not reported.  **Where reported:** NA.  **Mentioned covariate distribution:** yes.  **What reported:** as above.  **Where reported:** Results text. |
| Peters 2013 | **Protocol:** Not reported.  **Review methods:** Not reported.  **Review results:** Time point. 3, 6, 12 months.  **Type:** Outcome.  **Covariate summary:** Time point. | Long-term change in self reported functional ability; primary; continuous; mean difference or no results were reported; NT=6; NP=394. | **Number of trials:** 3.  **Covariate distribution (across trials):**  In analyses:  NT=1, NP=40 (3. Immobilisation (plaster of Paris splint) versus bulky dressing and mobilisation):  Long-term improvment in functional ability (BCTQ Functional Status Score) at 3 months.  NT=1, NP=150 (14. Multimodal hand therapy versus normal activities/exercise):  Long-term improvment in functional ability (BCTQ Functional Status Score). DASH at 6 months post surgery.  NT=1, NP=27 (15. Desensitisation therapy (as part of multiple interventions) versus no treatment):  Long-term improvment in functional ability (BCTQ Functional Status Score) at 12 months post surgery.  **Covariate distribution (within trials):** NA. | **Analysis type in review according to glossary 1:** Subgroup analysis.  **Reported results:** Mean, SD and N by group for trials. Mean difference and CI for trials. Mean difference, CI, Z statistic, and p-value for meta-analytic subtotal.  **Where reported:** Results text and forest plot.  **Method to detect interactions reported in review:** Not reported.  **Reported results from methods to detect interactions:** Not reported.  **Where reported:** NA. | **Reported detected interaction:** Not reported.  **What reported:**  Immobilisation (single intervention) versus mobilisation (multiple interventions):  Four trials investigated immobilisation versus mobilisation ( Cebesoy 2007; Cook 1995; Finsen 1999; Martins 2006). One quasi-randomised trial (Cebesoy 2007) investigated immobilisation (using a plaster wrist orthosis worn until suture removal) and latemobilisation (bulky dressing worn for a three-week period and immediate early mobilisation exercises). Cook 1995 randomly allocated participants to immobilisation using a volar wrist orthosis for two weeks versus bulky dressing and advice to move the hand and wrist with no restrictions. One quasi-randomised trial (Finsen 1999) compared the use of a plaster wrist orthosis for two weeks versus light dressing and active mobilisation within comfort from 48 hours post surgery. Martins 2006 investigated the efficacy of immobilisation using a neutral wrist orthosis for two weeks post surgery versus no orthosis and unrestricted movement of the wrist and fingers. Cebesoy 2007, Cook 1995, Finsen 1999 and Martins 2006 examined the effects of immobilisation using a wrist orthosis when comparedwithmobilisation using activemovement of the affected limb commenced immediately postoperatively (Cebesoy 2007) or on the first (Cook 1995) or second (Finsen 1999; Martins 2006) postoperative day. The duration of orthotic use between trials was different: Cebesoy 2007 immobilised the wrists for ten days, Cook 1995 and Martins 2006 immobilised the affected wrists for two weeks, whilst another trial (Finsen 1999) immobilised the affected wrists for four weeks. However, in Finsen 1999 the trialists reported six deviations from protocol in which people were splinted for either a shorter or longer time.  Primary outcomes  1. Long-termchange in self reported functional ability as measured  on a continuous scale at three months or longer  • Reported in Cebesoy 2007  • Function was assessed using the Functional Severity Scale on the BCTQ preoperatively and three months post surgery in Cebesoy 2007. There was no statistically significant difference in function between participants receiving bulky dressing and early mobilisation compared to those receiving splint and late mobilisation at three months post surgery (Analysis 3.1).  Multimodal hand therapy (multiple interventions) versus no formal therapy (control)  Pomerance 2007 examined the effects of a formal programofmultimodal hand therapy (consisting of six 30 min sessions of nerve gliding, range of motion and strengthening and additional treatments, for example, massage, fluidotherapy with a qualified hand therapist). Treatments were provided over a two-week period starting at five to seven days post surgery. The control group received advice regarding tendon gliding exercises and scar massage prior to surgery but no formal therapy after surgery. A total of 150 participants were randomised. Outcomes included time to return to work, pain intensity, lateral pinch strength, grip strength, function using the DASH questionnaire, persistence of symptoms, wound dehiscence, and an economic evaluation of the intervention.  Primary outcomes  1. Long-termchange in self reported functional ability as measured on a continuous scale at three months or longer  • DASH was measured at five and a half months posttreatment (six months post surgery). There was no statistically significant difference between treatments (Analysis 14.1).  Desensitisation therapy (as part of multiple interventions) versus standard treatment (control)  The Powell 2003 unpublished study randomly allocated 29 participants to either a graduated desensitisation program for three months or a standard treatment control.Outcomesweremeasured at three and sixweeks, and threemonths post operation.Outcomes included scar sensitivity using a dolorimeter (pressure gauge), patient- reported scar sensitivity, functional status using the BCTQ Functional Status Scale, and grip strength.  Primary outcomes   - Long-termchange in self reported functional ability asmeasured on a continuous scale at three months or longer   • BCTQ Functional Status Scale was used to record functional ability at three months. There was no statistically significant benefit of graded desensitisation over standard treatment (Analysis 15.1)  **Where reported:** Results text.  **Interaction exists based on reported results:** Not reported.  **How determined:** NA.  **Reported importance of the interaction:** Not reported.  **What reported:** Not reported.  **Where reported:** NA  **Reported plausibility of the interaction:** Not reported.  **What reported:** Not reported.  **Where reported:** NA  **Reported confounding possibility:** Not reported.  **What reported:** Not reported.  **Where reported:** NA.  **Mentioned covariate distribution:** Yes.  **What reported:** as above.  **Where reported:** Results text. |
| Rockers 2013 | **Protocol:** Not reported.  **Review methods:** Not reported.  **Review results:** ‘Hiring and retaining’; ‘training’.  **Type:** Intervention.  **Covariate summary:** Type of intervention or control. | Health systems outcomes, such as: population health outcomes, access to health care, utilization of health care, quality of health care, efficiency of health care, equity of health care; primary; dichotomous; risk difference; NT=1; NP=not reported (for population health outcomes, access to health care, utilization of health care),  NT=0; NP=0 (for quality of health care, efficiency of health care, equity of health care). | **Number of trials:** 1 (for population health outcomes, access to health care, utilization of health care); 0 (for quality of health care, efficiency of health care, and equity of health care).  **Covariate distribution (across trials):**  For population health outcomes, access to health care, utilization of health care:  NT=1, NP=not reported (hiring);  NT=0, NP=0 (training).  No included trials for quality of health care, efficiency of health care, and equity of health care.  **Covariate distribution (within trials):** NA. | **Analysis type in review according to glossary 1:** Subgroup analysis.  **Reported results:** Risk difference and CI from trials (for access to health care, utilization of health care) or narrative results (for population health outcomes).  **Where reported:** Results text.  **Method to detect interactions reported in review:** Not reported.  **Reported results from methods to detect interactions:** Not reported.  **Where reported:** NA. | **Reported detected interaction:** Not reported.  **What reported:**  ‘Hiring: Bloom 2006 found that contracting-in district management had a statistically significant impact on various outcomes. In a TOT analysis at the level of the health facility, the authors found that contracting-in increased the probability that a health facilitywould be open 24 hours by 83% (95% confidence interval (CI): 61 to 105) and thatmedical equipment and supplies would be available. In an ITT analysis at the level of the individual patient, they found that contracting-in increased use of antenatal care by 28% (95% CI: 16 to 40) and use of public facilities by 14%(95%CI: 6 to 22). In a TOT analysis of the same outcomes, the authors found that contracting-in increased use of antenatal care by 36% (95% CI: 20 to 52) and use of public facilities by 18% (95% CI: 10 to 26). The authors did not find a significant effect of the contractingin treatment on population health outcomes. Finally, the authors found no effect of contracting-in on healthcare spending at the individual level.’  **Where reported:** Results text.  **Interaction exists based on reported results:** Not reported.  **How determined:** NA.  **Reported importance of the interaction:** Not reported.  **What reported:** Not reported.  **Where reported:** NA  **Reported plausibility of the interaction:** Not reported.  **What reported:** Not reported.  **Where reported:** NA  **Reported confounding possibility:** Not reported.  **What reported:** Not reported.  **Where reported:** NA.  **Mentioned covariate distribution:** Not reported.  **What reported:** Not reported.  **Where reported:** NA. |
| Sajid, 2012 | **Protocol: ‘**We will also attempt to analyse any difference between various groups of patients who have undergone simple mastectomy, mastectomy and axillary dissection, axillary  surgery only and redo breast or axillary surgery.’  **Review methods:** ‘We performed the subgroup analysis on trials in breast surgery and breast plus axillary surgery to find out if there was any difference depending upon the site of surgery  for breast cancer.’  **Review results:** ‘All trials analysis’; ‘Subgroup analysis of trials on mastectomy’; ‘Subgroup analysis of trials on mastectomy plus axillary surgery’.  **Type:** Patient.  **Covariate summary:** Disease characteristics. | Incidence of seroma, defined as the presence of fluid collection under the skin flap diagnosed by clinical and radiological assessment and requiring prolonged hospital stay, frequent clinic visits or aspiration(s), or a combination of these; primary; dichotomous; risk ratio; NT=18; NP=1,252. | **Number of trials:** 17.  **Covariate distribution (across trials):**  NT=10, NP=629 **(**mastectomy);  NT=7, NP=496 **(**mastectomy + axillary surgery).  **Covariate distribution (within trials):**  Not reported % mastectomy (10 trials);  Not reported % mastectomy + axillary surgery (7 trials ). | **Analysis type in review according to glossary 1:** Subgroup analysis.  **Reported results:** Event rates, risk ratio, and CI for trials. Risk ratio, CI, z statistic, and p-value for meta-analytic subtotals.  **Where reported:** Results text and forest plot.  **Method to detect interactions reported in review:** Not reported.  **Reported results from methods to detect interactions:** Not reported.  **Where reported:** NA. | **Reported detected interaction:** Not reported.  **What reported:**  Subgroup analysis of trials on mastectomy:  Ten RCTs encompassing 629 participants undergoing breast surgery for breast cancer were retrieved from the electronic databases (Cipolla 2010;Dinsmore 2000; El-Nakeeb 2009;Moore  1997; Moore 2001; Mustonen 2004; Tasinato 1993; Uden 1993; Ulusoy 2003; Vaxman 1995). There were 335 people in the FG group and 294 people in the NFG group.  Incidence of postoperative wound site seroma:  All 10 trials contributed to the combined calculation of this variable (Cipolla 2010; Dinsmore 2000; El-Nakeeb 2009; Moore 1997; Moore 2001; Mustonen 2004; Tasinato 1993; Uden 1993;  Ulusoy 2003; Vaxman 1995). There was no significant heterogeneity (I2 = 10%) among studies. Therefore, in the fixed-effect model, FG was statistically ineffective in reducing the incidence  of postoperative seroma in people undergoing breast and axillary surgery (RR 1.02; 95% CI 0.90 to 1.15, P value = 0.80; Analysis 2.1  Subgroup analysis of trials on mastectomy plus axillary surgery:  Seven RCTs encompassing 496 participants undergoing breast plus axillary surgery for breast cancer were retrieved from the electronic databases (Gilly 1998; Gioffrè Florio 1993; Jain 2004;  Johnson 2005; Ko 2009; Langer 2003; Segura-Castillo 2005). There were 224 people in the FG group and 272 people in the NFG group.  Incidence of postoperative wound site seroma:  Seven trials contributed to the combined calculation of this variable (Gilly 1998; Gioffrè Florio 1993; Jain 2004; Johnson 2005; Ko 2009; Langer 2003; Segura-Castillo 2005). There was no significant heterogeneity (I2 = 16%) among studies. Therefore, in the fixed-effect model, FG was statistically ineffective in reducing the incidence of postoperative seroma in participants undergoing breast and axillary surgery (RR 1.04; 95%CI 0.72 to 1.51; P value= 0.82; Analysis 3.1).  **Where reported:** Results text.  **Interaction exists based on reported results:** Not reported.  **How determined:** NA.  **Reported importance of the interaction:** Not reported.  **What reported:** Not reported.  **Where reported:** NA  **Reported plausibility of the interaction:** Not reported.  **What reported:** Not reported.  **Where reported:** NA  **Reported confounding possibility:** Not reported.  **What reported:** Not reported.  **Where reported:** NA.  **Mentioned covariate distribution:** Yes.  **What reported:** as above.  **Where reported:** Results text. |
| Sampson 2013 | **Protocol: ‘**Outcomes were grouped into the short term (up to 12 weeks), medium term (13 to 26 weeks) and long term (over 26 weeks).’  **Review methods:** ‘Data were sub-grouped according to duration of follow-up (short term, medium term or long term).’  **Review results: ‘**short term (≤12 weeks)’; ‘medium term (13-25 weeks)’; ‘long term (≥26 weeks)’.  **Type:** Outcome.  **Covariate summary:** Time point. | Relapse; primary; dichotomous; risk ratio; NT=12; NP=1,327. | **Number of trials:** 12 (Comparison 1. Any intermittent drug technique versus maintenance therapy);  2 (Comparison 2. Intermittent (early-based) versus maintenance therapy);  0 (Comparison 3. Intermittent (crisis intervention) versus maintenance therapy);  3 (Comparison 4. Intermittent (gradually increased drug-free periods) versus maintenance therapy);  6(Comparison 5. Intermittent (drug holiday) versus maintenance therapy);  2 (Comparison 6. Any intermittent drug technique versus placebo);  **Covariate distribution (across trials):**  NT=12, NP==1,113 (Comparison 1. Any intermittent drug technique versus maintenance therapy):  NT=4, NP=396 (short term (≤12 weeks));  NT=5, NP=774 ( medium term (13-25 weeks));  NT=7, NP=436( long term (≥26 weeks)).  NT=2, NP=155 (Comparison 2. Intermittent (early-based) versus maintenance therapy):  NT=2, NP=155 (long term (≥26 weeks)).  NT=0, NP=0 (Comparison 3. Intermittent (crisis intervention) versus maintenance therapy).  NT=3, NP=219 (Comparison 4. Intermittent (gradually increased drug-free periods) versus maintenance therapy):  NT=1, NP=128 (short term (≤12 weeks));  NT=1, NP=128 (medium term (13-25 weeks));  NT=3, NP=219(long term (≥26 weeks)).  NT=6, NP=365 (Comparison 5. Intermittent (drug holiday) versus maintenance therapy):  NT=3, NP=268 (short term (≤12 weeks));  NT=3, NP=272 (medium term (13-25 weeks));  NT=2, NP=62 (long term (≥26 weeks)).  NT=2, NP=290 (Comparison 6. Any intermittent drug technique versus placebo):  NT=1, NP=260 (short term (≤12 weeks));  NT=2, NP=290 (medium term (13-25 weeks)).  **Covariate distribution (within trials):** NA. | **Analysis type in review according to glossary 1:** Subgroup analysis.  **Reported results:** Event rates, risk ratio, and CI for trials. Risk ratio, CI, z statistic, and p-value for meta-analytic subtotals.  **Where reported:** Results text and forest plot.  **Method to detect interactions reported in review:** Not reported.  **Reported results from methods to detect interactions:** Test for subgroup differences (Chi-square statistic, p-value, and I square statistic).  **Where reported:** Forest plot. | **Reported detected interaction:** Not reported.  **What reported:**  All 17 included studies were included in this comparison, which included any type of intermittent drug therapy, with a total n = 2252. Twelve of the included studies reported data for the primary outcome of relapse (n = 1327) and six reported data for the second primary outcome of hospitalisation (n = 661). All the remaining studies reported for outcomes of either death, global state, general functioning, service outcomes, adverse effects, quality of life and leaving the study early, with the primary objective of evaluating the effects of intermittent drug techniques on these outcomes - participants had to be clinically stabilised to some extent to participate in the study.  **1.1 Relapse**  Data demonstrate that there was a significantly greater risk of relapse for participants receiving intermittent drug therapy; results were homogenous, clinically and statistically significant in favour of maintenance therapy at each short term (n = 396, 4 RCTs, risk ratio (RR) 1.68, 95% confidence interval (CI) 1.00 to 2.81), medium term (n = 774, 5 RCTs, RR 2.41, 95% CI 1.50 to 3.86) and long term (n = 436, 7 RCTs, RR 2.46, 95% CI 1.70 to 3.54, Analysis 1.1).  **Comparison 2: INTERMITTENT (EARLY-BASED) versus MAINTENANCE THERAPY**  Five studies compared intermittent (early-based) therapy with maintenance therapy (Carpenter 1987; Carpenter 1990*; Herz 1991*; Jolley 1989/1990; Schooler 1997, n = 626).  **2.1 Relapse**  Data from two RCTs showed that people receiving intermittent (early-based) treatmentweremore likely to relapse,with significant favour of maintenance therapy at long term(n = 155, 2 RCTs, RR 2.33, 95% CI 1.32 to 4.12, Analysis 2.1).  **Comparison 3: INTERMITTENT (CRISIS INTERVENTION) versus MAINTENANCE THERAPY**  **Comparison 4: INTERMITTENT (GRADUALLY INCREASED DRUG-FREE PERIODS) versus MAINTENANCE THERAPY**  Three studies provided data for this comparison (Gaebel 2011;Wiedemann 2001; Wunderink 2007, n = 260).  **4.1 Relapse**  Data from three RCTs showed that people receiving intermittent (gradually increased drug-free) treatment were more likely to relapse, with no significant difference at short (n = 128, 1 RCT) and medium term (n = 128, 1 RCT), but with significant favour of maintenance therapy at long term (n = 219, 3 RCTs, RR 2.76, 95%CI 1.63 to 4.67, Analysis 4.1).Heterogenitywas presentwith long-term data, but this was only slight (Chi² = 2.89, P = 0.24, I² = 31%), and results remain in data and analysis using a fixedeffect model.  **Comparison 5: INTERMITTENT (DRUG HOLIDAY) versus MAINTENANCE THERAPY**  Seven studies provided data for this comparison (Blackburn 1961;Caffey 1964; McCreadie 1980; McCreadie 1982; Olson 1962*;Remington 2011; Shenoy 1981, n = 627).  **5.1 Relapse**  Data demonstrate that there was a significantly greater risk of relapse for participants receiving intermittent drug therapy; results were homogenous, statistically significant in favour of maintenance therapy at medium term (n = 272, 3 RCTs, RR 2.15, 95% CI 1.25 to 3.68), however, results were equivocal by short term (n= 268, 3 RCTs, RR 1.59, 95% CI 0.94 to 2.70) and long term (n= 62, 2 RCTs, RR 1.70, 95% CI 0.54 to 5.38, Analysis 5.1).  **Comparison 6: ANY INTERMITTENT DRUG TECHNIQUE versus PLACEBO**  Three studies reported data for this comparison (Blackburn 1961;Caffey 1964; Olson 1962*, n = 498).  **6.1 Relapse**  Compared with placebo, there was significant favour for intermittent treatment at short term (n = 260, 1 RCT, RR 0.22, 95% CI 0.10 to 0.45) and medium term (n = 290, 2 RCTs, RR 0.37, 95% CI 0.24 to 0.58, Analysis 6.1), however, heterogeneity is present for the medium term results (Chi² = 2.59, P = 0.11, I² = 61%), and so caution should be employed in interpreting this data.  **Where reported:** Results text.  **Interaction exists based on reported results:** No.  **How determined:**  COMP 1: Test for subgroup differences: Chi2= 1.54, df= 2 (P= 0.46), I2=0.0%  COMP 4: Test for subgroup differences: Chi2= 0.10, df= 2 (P= 0.95), I2=0.0%  COMP 5: Test for subgroup differences: Chi2= 0.61, df= 2 (P= 0.74), I2=0.0%  COMP 6: Test for subgroup differences: Chi2= 1.54, df= 1 (P= 0.21), I2=35%  **Reported importance of the interaction:** Not reported.  **What reported:** Not reported.  **Where reported:** NA  **Reported plausibility of the interaction:** Not reported.  **What reported:** Not reported.  **Where reported:** NA  **Reported confounding possibility:** Not reported.  **What reported:** Not reported.  **Where reported:** NA.  **Mentioned covariate distribution:** Yes.  **What reported:** as above.  **Where reported:** Results text. |
| Sampson 2013 | **Protocol:** ‘We carried out a subgroup analysis to compare results between different interventions, as defined in ’Types of interventions’. 1.’Prodrome-based intervention’ defined as treatment given on the early signs of relapse. 2.’Crisis intervention’ defined as treatment given only in case of full relapse and discontinued again after re-stabilization. 3.’Gradually increased drug-free period’ defined as increasing the cessation period of the treatment constantly. 4.’Drug Holidays’ defined as stopping the medication for fixed periods, and then reintroducing it (repeating thismore than once). 5. Maintenance therapy, as defined by the treating physician.’  **Review methods:** Not reported.  **Review results:** ‘Comparison 1. Any intermittent drug technique versus maintenance therapy’; ‘Comparison 2. Intermittent (early-based) versus maintenance therapy’; ‘Comparison 3. Intermittent (crisis intervention) versus maintenance therapy’; ‘Comparison 4. Intermittent (gradually increased drug-free periods) versus maintenance therapy’; ‘Comparison 5. Intermittent (drug holiday) versus maintenance therapy’; ‘Comparison 6. Any intermittent drug technique versus placebo’; ‘Comparison 7. Any intermittent drug technique (specific drug) versus maintenance therapy (specific drug): intermittent fluphenazine decanoate (low dose) vs maintained fluphenazine decanoate (moderate dose) -short term (≤12 weeks)’; ‘Comparison 7. Any intermittent drug technique (specific drug) versus maintenance therapy (specific drug)): intermittent haloperidol equivalents (low dose) vs maintained haloperidol equivalents (low dose) – short term (≤12 weeks)’; ‘Comparison 7. Any intermittent drug technique (specific drug) versus maintenance therapy (specific drug)): various intermittent typical antipsychotics (moderate dose) vs maintained typical antipsychotics (moderate dose) - short term (≤12 weeks)’; ‘Comparison 7. Any intermittent drug technique (specific drug) versus maintenance therapy (specific drug)): intermittent haloperidol equivalents (low dose) vs maintained haloperidol equivalents (low dose) -medium term (13-25 weeks)’; ‘Comparison 7. Any intermittent drug technique (specific drug) versus maintenance therapy (specific drug)): atypical antipsychotics (dosage unclear) vs maintained atypical/typical antipsychotics (dosage unclear) – medium term (13-25 weeks)’; ‘Comparison 7. Any intermittent drug technique (specific drug) versus maintenance therapy (specific drug)): various intermittent typical antipsychotics (low dose) vs maintained typical antipsychotics (low dose) -medium term (13-25 weeks)’; ‘Comparison 7. Any intermittent drug technique (specific drug) versus maintenance therapy (specific drug)): various intermittent typical antipsychotics (moderate dose) vs maintained typical antipsychotics (moderate dose) – medium term (13-25 weeks)’; ‘Comparison 7. Any intermittent drug technique (specific drug) versus maintenance therapy (specific drug)): intermittent chlorpromazine equivalents (low dose) vs maintained chlorpromazine equivalents (low dose) - long term (≥26 weeks)’; ‘Comparison 7. Any intermittent drug technique (specific drug) versus maintenance therapy (specific drug)): intermittent clozapine (low dose) vs maintained clozapine (low dose) long term (≥26 weeks)’; ‘Comparison 7. Any intermittent drug technique (specific drug) versus maintenance therapy (specific drug)): intermittent haloperidol equivalents (low dose) vs maintained haloperidol equivalents (low dose) – long term (≥26 weeks)’; ‘Comparison 7. Any intermittent drug technique (specific drug) versus maintenance therapy (specific drug)): intermittent pimozide (high dose) vs fluphenazine decanoate (low dose) – long term (≥26 weeks)’; ‘Comparison 7. Any intermittent drug technique (specific drug) versus maintenance therapy (specific drug)): various intermittent typical antipsychotics (low dose) vs maintained typical antipsychotics - long term (≥26 weeks)’.  **Type:** Intervention.  **Covariate summary:** Type of intervention or control. | Relapse; primary; dichotomous; risk ratio; NT=12; NP=1,327. | **Number of trials:** 12.  **Covariate distribution (across trials):**  NT=12, NP==1,113 (Comparison 1. Any intermittent drug technique versus maintenance therapy);  NT=2, NP=155 (Comparison 2. Intermittent (early-based) versus maintenance therapy);  NT=0, NP=0 (Comparison 3. Intermittent (crisis intervention) versus maintenance therapy);  NT=3, NP=219 (Comparison 4. Intermittent (gradually increased drug-free periods) versus maintenance therapy);  NT=6, NP=365 (Comparison 5. Intermittent (drug holiday) versus maintenance therapy);  NT=2, NP=290 (Comparison 6. Any intermittent drug technique versus placebo);  NT=12, NP=1,111 (Comparison 7. Any intermittent drug technique (specific drug) versus maintenance therapy (specific drug)):  NT=1, NP=31 (intermittent fluphenazine decanoate (low dose) vs maintained fluphenazine decanoate (moderate dose) -short term (≤12 weeks));  NT=1, NP=128 (intermittent haloperidol equivalents (low dose) vs maintained haloperidol equivalents (low dose) – short term (≤12 weeks));  NT=1, NP=177 (various intermittent typical antipsychotics (moderate dose) vs maintained typical antipsychotics (moderate dose) - short term (≤12 weeks));  NT=1, NP=128 (intermittent haloperidol equivalents (low dose) vs maintained haloperidol equivalents (low dose) -medium term (13-25 weeks));  NT=1, NP=35 (atypical antipsychotics (dosage unclear) vs maintained atypical/typical antipsychotics (dosage unclear) – medium term (13-25 weeks));  NT=1, NP=60 (various intermittent typical antipsychotics (low dose) vs maintained typical antipsychotics (low dose) -medium term (13-25 weeks));  NT=2, NP=551 (various intermittent typical antipsychotics (moderate dose) vs maintained typical antipsychotics (moderate dose) – medium term (13-25 weeks));  NT=2, NP=148 (intermittent chlorpromazine equivalents (low dose) vs maintained chlorpromazine equivalents (low dose) - long term (≥26 weeks));  NT=1, NP=25 (intermittent clozapine (low dose) vs maintained clozapine (low dose) long term (≥26 weeks));  NT=3, NP=226 (intermittent haloperidol equivalents (low dose) vs maintained haloperidol equivalents (low dose) – long term (≥26 weeks));  NT=2, NP=62 (intermittent pimozide (high dose) vs fluphenazine decanoate (low dose) – long term (≥26 weeks));  NT=1, NP=26 (various intermittent typical antipsychotics (low dose) vs maintained typical antipsychotics - long term (≥26 weeks)).  **Covariate distribution (within trials):** NA. | **Analysis type in review according to glossary 1:** Subgroup analysis.  **Reported results:** Event rates, risk ratio, and CI for trials. Risk ratio, CI, z statistic, and p-value for meta-analytic subtotals.  **Where reported:** Results text and forest plot.  **Method to detect interactions reported in review:** Not reported.  **Reported results from methods to detect interactions:** For comparison 7, test for subgroup differences (Chi-square statistic, p-value, and I square statistic).  **Where reported:** Forest plot. | **Reported detected interaction:** Not reported.  **What reported:**  **Comparison 1. ANY INTERMITTENT DRUG TECHNIQUE versus MAINTENANCE THERAPY**  All 17 included studies were included in this comparison, which included any type of intermittent drug therapy, with a total n = 2252. Twelve of the included studies reported data for the primary outcome of relapse (n = 1327) and six reported data for the second primary outcome of hospitalisation (n = 661). All the remaining studies reported for outcomes of either death, global state, general functioning, service outcomes, adverse effects, quality of life and leaving the study early, with the primary objective of evaluating the effects of intermittent drug techniques on these outcomes - participants had to be clinically stabilised to some extent to participate in the study.  **1.1 Relapse**  Data demonstrate that there was a significantly greater risk of relapse for participants receiving intermittent drug therapy; results were homogenous, clinically and statistically significant in favour of maintenance therapy at each short term (n = 396, 4 RCTs, risk ratio (RR) 1.68, 95% confidence interval (CI) 1.00 to 2.81), medium term (n = 774, 5 RCTs, RR 2.41, 95% CI 1.50 to 3.86) and long term (n = 436, 7 RCTs, RR 2.46, 95% CI 1.70 to 3.54, Analysis 1.1).  **Comparison 2: INTERMITTENT (EARLY-BASED) versus MAINTENANCE THERAPY**  Five studies compared intermittent (early-based) therapy with maintenance therapy (Carpenter 1987; Carpenter 1990*; Herz 1991*; Jolley 1989/1990; Schooler 1997, n = 626).  **2.1 Relapse**  Data from two RCTs showed that people receiving intermittent (early-based) treatmentweremore likely to relapse,with significant favour of maintenance therapy at long term(n = 155, 2 RCTs, RR 2.33, 95% CI 1.32 to 4.12, Analysis 2.1).  **Comparison 3: INTERMITTENT (CRISIS INTERVENTION) versus MAINTENANCE THERAPY**  **Comparison 4: INTERMITTENT (GRADUALLY INCREASED DRUG-FREE PERIODS) versus MAINTENANCE THERAPY**  Three studies provided data for this comparison (Gaebel 2011;Wiedemann 2001; Wunderink 2007, n = 260).  **4.1 Relapse**  Data from three RCTs showed that people receiving intermittent (gradually increased drug-free) treatment were more likely to relapse, with no significant difference at short (n = 128, 1 RCT) and medium term (n = 128, 1 RCT), but with significant favour of maintenance therapy at long term (n = 219, 3 RCTs, RR 2.76, 95%CI 1.63 to 4.67, Analysis 4.1).Heterogenitywas presentwith long-term data, but this was only slight (Chi² = 2.89, P = 0.24, I² = 31%), and results remain in data and analysis using a fixedeffect model.  **Comparison 5: INTERMITTENT (DRUG HOLIDAY) versus MAINTENANCE THERAPY**  Seven studies provided data for this comparison (Blackburn 1961;Caffey 1964; McCreadie 1980; McCreadie 1982; Olson 1962*;Remington 2011; Shenoy 1981, n = 627).  **5.1 Relapse**  Data demonstrate that there was a significantly greater risk of relapse for participants receiving intermittent drug therapy; results were homogenous, statistically significant in favour of maintenance therapy at medium term (n = 272, 3 RCTs, RR 2.15, 95% CI 1.25 to 3.68), however, results were equivocal by short term (n= 268, 3 RCTs, RR 1.59, 95% CI 0.94 to 2.70) and long term (n= 62, 2 RCTs, RR 1.70, 95% CI 0.54 to 5.38, Analysis 5.1).  **Comparison 6: ANY INTERMITTENT DRUG TECHNIQUE versus PLACEBO**  Three studies reported data for this comparison (Blackburn 1961;Caffey 1964; Olson 1962*, n = 498).  **6.1 Relapse**  Compared with placebo, there was significant favour for intermittent treatment at short term (n = 260, 1 RCT, RR 0.22, 95% CI 0.10 to 0.45) and medium term (n = 290, 2 RCTs, RR 0.37, 95% CI 0.24 to 0.58, Analysis 6.1), however, heterogeneity is present for the medium term results (Chi² = 2.59, P = 0.11, I² = 61%), and so caution should be employed in interpreting this data.  **Comparison 7: ANY INTERMITTENT DRUG TECHNIQUE (SPECIFIC DRUG) versus MAINTENANCE THERAPY (SPECIFIC DRUG)**  We compared the differences between the various antipsychotics used in intermittent treatment compared with maintenance therapy at short term, medium and long term for our two primary outcomes of relapse and hospitalisation. Twelve studies provided data for the outcome of relapse (Blackburn 1961; Caffey 1964; Gaebel 2011; Herz 1991*; Jolley 1989/1990; McCreadie 1980; McCreadie 1982; Prien 1973; Remington 2011; Shenoy 1981; Wiedemann 2001; Wunderink 2007, n = 1327); however, some studies did not provide adequate information regarding the types of antipsychotics used, instead converting dosages into milligrams of chlorpromazine (Carpenter 1987; Carpenter 1990*; Herz 1991*; Wiedemann 2001) or haloperidol (Gaebel 2011; Jolley 1989/1990; Wunderink 2007) equivalents. Dosages were unclear in Remington 2011.  **7.1 Relapse**  Results demonstrated homogeneity, with favour of maintenance therapy with each specific drug comparison; some results were statistically significant, however, and demonstrate a higher risk of relapse for people receiving intermittent drugs in the following comparisons: various intermittent typical antipsychotics (moderate dose) versusmaintained typical antipsychotics (moderate dose) at medium term (n = 551, 2 RCTs, RR 3.75, 95% CI 1.42 to 9.94); intermittent chlorpromazine equivalents (low dose) versus maintained chlorpromazine equivalents (lowdose) by long term(n = 148, 2 RCTs, RR 2.62, 95% CI 1.30 to 5.28) and intermittent haloperidol equivalents (low dose) versus maintained haloperidol equivalents (low dose) by long term (n = 226, 3 RCTs, RR 2.53, 95% CI 1.60 to 4.01, Analysis 7.1).  **Where reported:** Results text.  **Interaction exists based on reported results:** No for comparison 7.  **How determined:** COMP 7: Test for subgroup differences: Chi2= 4.37, df= 10 (P= 0.93), I2=0.0%  **Reported importance of the interaction:** Not reported.  **What reported:** Not reported.  **Where reported:** NA  **Reported plausibility of the interaction:** Not reported.  **What reported:** Not reported.  **Where reported:** NA  **Reported confounding possibility:** Not reported.  **What reported:** Not reported.  **Where reported:** NA.  **Mentioned covariate distribution:** Not reported.  **What reported:** Not reported.  **Where reported:** NA. |
| Sampson 2013 | **Protocol:** ‘Where a trial was described as ’double-blind’, but it was only implied that the study was randomised, we included these trials in a sensitivity analysis.’  **Review methods: ‘**Implication of randomisation. We included trials in a sensitivity analysis if they were described in some way as to imply randomisation.’  **Review results: ‘**When included studies that implied randomisation or provided no further details regarding randomisation techniques were removed from the meta-analysis’.  **Type:** Methodological.  **Covariate summary:** Implied randomisation. | Relapse; primary; dichotomous; risk ratio; NT=12; NP=1,327. | **Number of trials:** 3.  **Covariate distribution (across trials):**  NT=3, NP=273 (Any intermittent drug technique versus maintenance therapy. When included studies that implied randomisation or provided no further details regarding randomisation techniques were removed from the meta-analysis).  **Covariate distribution (within trials):** NA. | **Analysis type in review according to glossary 1:** Sensitivity analysis.  **Reported results:** risk ratio and CI for meta-analytic totals.  **Where reported:** Results text.  **Method to detect interactions reported in review:** Not reported.  **Reported results from methods to detect interactions:** Not reported.  **Where reported:** NA. | **Reported detected interaction:** No interaction.  **What reported:**  **‘**‘When included studies that implied randomisation or provided no further details regarding randomisation techniques were removed from the meta-analysis, this did not substantially alter the direction of effect or the precision of the effect estimates, with a significantly greater risk of relapse still demonstrated for people receiving any intermittent therapy by long term (n= 273, 3 RCTs, RR 2.19, 95% CI 1.41 to 3.42),...’  **Where reported:** Results text.  **Interaction exists based on reported results:** Not reported.  **How determined:** NA.  **Reported importance of the interaction:** Not reported.  **What reported:** Not reported.  **Where reported:** NA  **Reported plausibility of the interaction:** Not reported.  **What reported:** Not reported.  **Where reported:** NA  **Reported confounding possibility:** Not reported.  **What reported:** Not reported.  **Where reported:** NA.  **Mentioned covariate distribution:** Yes.  **What reported:** as above.  **Where reported:** Results text. |
| Sanders 2013 | **Protocol: ‘**Comparison groups will be one of: Placebo; or No treatment or standard care.’  **Review methods: ‘**Comparison groups were one of: Placebo; or No treatment or standard care.’ ‘In this patient group, the comparison groups will be a different dose of statin’.  **Review results:** ‘Comparison of statin with no treatment/placebo’; ‘comparison of high-dose with low-dose statin’.  **Type:** Intervention.  **Covariate summary:** Type of intervention or control and dose. | All-cause mortality within 30 days of surgery; primary; dichotomous; risk ratio; NT=3; NP=178. | **Number of trials:** 3.  **Covariate distribution (across trials):**  NT=3, NP=178 (‘comparison of statin with no treatment/placebo’);  NT=0, NP=0 (‘comparison of high-dose with low-dose statin’) (no data presented).  **Covariate distribution (within trials):** NA. | **Analysis type in review according to glossary 1:** Subgroup analysis.  **Reported results:** Event rates, risk ratio, and CI for trials. Risk ratio, CI, z statistic, and p-value for meta-analytic subtotal (one group).  **Where reported:** Results text and forest plot.  **Method to detect interactions reported in review:** Not reported.  **Reported results from methods to detect interactions:** Not reported.  **Where reported:** NA. | **Reported detected interaction:** Not reported.  **What reported:**  Comparison of statin with no treatment/ placebo:  All-cause mortality within 30 days of surgery (Analysis 1.1); was reported in three studies with a total of 178 participants. 7/ 105 (6.7%) participants in the statin group died within 30 days of surgery, as did 10/73 (13.7%) participants in the control group. No perioperative deaths were reported in the STAR VaS study or the MAPS study, and the estimate from Durazzo 2004 shows a statistically non-significant decrease in risk (risk ratio (RR) 0.73, 95% confidence interval (CI) 0.31 to 1.75). Mortality rates were high in this study, approaching 20%, possibly reflecting the poor state of health of participants (Figure 4).  Comparison of high-dose with low-dose statin:  Only two studies compared outcomes in participants who had received high- and low-dose statin. The MAPS study reported all outcomes, but only one MI was reported in the low-dose (A10) group and one new atrial fibrillation in the high-dose (A80) group. In preliminary reports from the APVS study, only combined cardiovascular endpoints (cardiac death, non-fatal MI and stroke) were reported with 3/53 (5.7% incidence) in the A80 group and 7/53 (13.2%) in the A20 group. This gives a relative risk of 0.43 (95% CI 0.12 to 1.57). We do not have sufficient data to present effect estimates on any of our prespecified outcomes for this comparison.  **Where reported:** Results text.  **Interaction exists based on reported results:** Not reported.  **How determined:** NA.  **Reported importance of the interaction:** Not reported.  **What reported:** Not reported.  **Where reported:** NA  **Reported plausibility of the interaction:** Not reported.  **What reported:** Not reported.  **Where reported:** NA  **Reported confounding possibility:** Not reported.  **What reported:** Not reported.  **Where reported:** NA.  **Mentioned covariate distribution:** Yes.  **What reported:** as above.  **Where reported:** Results text. |
| Sanders 2013 | **Protocol:** Not reported.  **Review methods:** Not reported.  **Review results:** ‘We excluded the DECREASE III study because of concerns about validity, but we ran sensitivity analyses on its impact on our results’.  **Type:** Other.  **Covariate summary:** Outlying results. | All-cause mortality within 30 days of surgery; primary; dichotomous; risk ratio; NT=4; NP=675. | **Number of trials:** 4.  **Covariate distribution (across trials):**  NT=4, NP=675 (‘comparison of statin with no treatment/placebo’).  **Covariate distribution (within trials):** NA. | **Analysis type in review according to glossary 1:** Sensitivity analysis.  **Reported results:** risk ratio and CI for meta-analytic subtotal.  **Where reported:** Results text.  **Method to detect interactions reported in review:** Not reported.  **Reported results from methods to detect interactions:** Not reported.  **Where reported:** NA. | **Reported detected interaction:** No interaction.  **What reported:** ‘We excluded theDECREASE III study because of concerns about validity, but we ran sensitivity analyses on its impact on our results for comparison 1 statin versus placebo/no treatment. This trial consisted of 497 participants who were scheduled for AAA repair, distal aorto-iliac reconstruction, lower limb arterial reconstruction or carotid endarterectomy at Erasmus Medical Centre, Rotterdam, Netherlands. In these analyses, our achieved sample size was 356 in the statin group and 320 in the control group. Results from DECREASE were similar to those of included studies, and in these analyses, the pooled estimates were as follows: for all-cause mortality, RR 0.61 (95% CI, 0.32 to 1.17);’  **Where reported:** Results text.  **Interaction exists based on reported results:** Not reported.  **How determined:** NA.  **Reported importance of the interaction:** Not reported.  **What reported:** Not reported.  **Where reported:** NA  **Reported plausibility of the interaction:** Not reported.  **What reported:** Not reported.  **Where reported:** NA  **Reported confounding possibility:** Not reported.  **What reported:** Not reported.  **Where reported:** NA.  **Mentioned covariate distribution:** Not reported.  **What reported:** Not reported.  **Where reported:** NA. |
| Schoot 2013 | **Protocol:** ‘Risk of bias criteria’.  **Review methods:** ‘We performed a sensitivity analysis based on the risk of bias criteria (i. e. excluding studies with a high risk of bias and studies for which the risk of bias was unclear) and compared the results of studies with a low risk of bias with those of all available studies, for all analyses that included more than one study.  **Review results: ‘**Risk of bias criteria’.  **Type:** Methodological.  **Covariate summary:** Risk of bias. | The number of children cured of their central venous catheter -related infection; primary; dichotomous; risk ratio; NT=3; NP=92. | **Number of trials**: Not reported.  **Covariate distribution (across trials)**: Not reported.  (Not reported because it’s uinclear whether the sensitivty analysis also grouped by trial design etc).  **Covariate distribution (within trials):** NA. | **Analysis type in review according to glossary 1:** Sensitivity analysis.  **Reported results:** Text.  **Where reported:** Results text.  **Method to detect interactions reported in review:** ‘compared the results of studies  with a low risk of bias with those of all available studies’**.**  **Reported results from methods to detect interactions:** Not reported.  **Where reported:** NA. | **Reported detected interaction:** No interaction.  **What reported:**  ‘**Sensitivity analyses for the used risk of bias criteria**  The results of the sensitivity analyses were consistent among the trials and did not differ from the overall analyses.’  **Where reported:** Results text.  **Interaction exists based on reported results:** Not reported.  **How determined:** NA.  **Reported importance of the interaction:** Not reported.  **What reported:** Not reported.  **Where reported:** NA  **Reported plausibility of the interaction:** Not reported.  **What reported:** Not reported.  **Where reported:** NA  **Reported confounding possibility:** Not reported.  **What reported:** Not reported.  **Where reported:** NA.  **Mentioned covariate distribution:** Not reported.  **What reported:** Not reported.  **Where reported:** NA. |
| Schoot 2013 | **Protocol:** Not reported.  **Review methods: ‘**RCTs and CCTs were analysed separately’.  **Review results: ‘**RCTs’ versus ‘CCTs’.  **Type:** Methodological.  **Covariate summary:** Trial design. | The number of children cured of their central venous catheter -related infection; primary; dichotomous; risk ratio; NT=3; NP=132. | **Number of trials:** 3.  **Covariate distribution (across trials):**  NT=2, NP=104 **(**RCTs);  NT=1, NP=28 **(**CCTs).  **Covariate distribution (within trials):** NA. | **Analysis type in review according to glossary 1:** Subgroup analysis.  **Reported results:** Event rates, risk ratio, and CI for trials. Risk ratio, CI, z statistic, and p-value for meta-analytic subtotals.  **Where reported:** Results text and forest plot.  **Method to detect interactions reported in review:** Not reported.  **Reported results from methods to detect interactions:** Not reported.  **Where reported:** NA. | **Reported detected interaction:** Not reported.  **What reported:**  ‘Number of children cured from their CVC-related infection  We were able to extract data on the number of participants cured fromall three included studies (Atkinson1998;Dannenberg 2003; La Quaglia 1994). We performed meta-analysis on results from the two randomised controlled trials (RCTs) with a total of 104 participant children (Atkinson 1998; La Quaglia 1994) (Figure 3). Thirty-nine of 56 children (70%) randomised to urokinase lock and systemic antibiotics were cured fromtheir CVC-related infection, compared with 33 of the 48 children (69%) randomised to systemic antibiotics alone.We found no significant difference between urokinase lock treatment with concomitant systemic antibiotics and systemic antibiotics alone (risk ratio (RR) 1.02, 95%confidence Interval (CI) 0.79 to 1.32, P = 0.89). No heterogeneity was detected (I² = 0%).  Data extracted from the CCT (Dannenberg 2003) covering 28 children also showed no significant difference between those treated with ethanol locks and concomitant systemic antibiotics and those treated with systemic antibiotics alone (RR 1.06, 95% CI 0.66 to 1.70, P = 0.81) (see Figure 3; Analysis 1.1). Eleven children out of 15 (73%) randomised to ethanol lock and systemic antibiotics were cured of their CVC-related infection, compared with nine of the 13 (69%) randomised to systemic antibiotics alone.’  **Where reported:** Results text.  **Interaction exists based on reported results:** Not reported.  **How determined:** NA.  **Reported importance of the interaction:** Not reported.  **What reported:** Not reported.  **Where reported:** NA  **Reported plausibility of the interaction:** Not reported.  **What reported:** Not reported.  **Where reported:** NA  **Reported confounding possibility:** Not reported.  **What reported:** Not reported.  **Where reported:** NA.  **Mentioned covariate distribution:** Yes.  **What reported:** as above.  **Where reported:** Results text. |
| Semple 2013 | **Protocol: ‘**Duration (short-term (i. e. less than three months), longer term (i. e. more than three months))’.  **Review methods:** ‘Duration: short-term (i. e. less than three months), medium-term (i. e. three to six months) or long-term (more than six months)’.  **Review results:** ‘Medium term (3-6 months)’; ‘end of intervention’.  **Type:** Outcome.  **Covariate summary:** Time point. | Quality of life; primary; continuous; mean difference or no data extracted; NT=5; NP=297. | **Number of trials:** 5.  **Covariate distribution (across trials):**  NT=3, NP=138 (‘Medium term (3-6 months)’;  NT=5, NP=297 (‘end of intervention’).  **Covariate distribution (within trials):** NA. | **Analysis type in review according to glossary 1:** Subgroup analysis.  **Reported results:** Mean, SD and N by group for trials. Mean difference and CI for trials. Mean difference, CI, Z statistic, and p-value for meta-analytic subtotal.  **Where reported:** Results text and forest plot.  **Method to detect interactions reported in review:** Not reported.  **Reported results from methods to detect interactions:** Not reported.  **Where reported:** NA. | **Reported detected interaction:** Not reported.  **What reported:**  ‘Medium-term follow-up (three to six months postintervention):  ..... The analysis did not provide evidence for the effectiveness of psychosocial intervention to improve global quality of life (MD -2.57, 95% CI -13.54 to 8.40) (Analysis 1.5) at this time juncture.  End of intervention:  There was no difference in global quality of life between the two groups at this time point (Analysis 1.12).’  **Where reported:** Results text.  **Interaction exists based on reported results:** Not reported.  **How determined:** NA.  **Reported importance of the interaction:** Not reported.  **What reported:** Not reported.  **Where reported:** NA  **Reported plausibility of the interaction:** Not reported.  **What reported:** Not reported.  **Where reported:** NA  **Reported confounding possibility:** Not reported.  **What reported:** Not reported.  **Where reported:** NA.  **Mentioned covariate distribution:** Not reported.  **What reported:** Not reported.  **Where reported:** NA. |
| Sharma 2013 | **Protocol:** ‘Participant age (children <18 years versus adults)’.  **Review methods: ‘**Participant age (children <18 years versus adults)’.  **Review results:** ‘Adults’; ‘Children’.  **Type:** Patient.  **Covariate summary:** Demographics. | Rates of active tuberculosis; primary; dichotomous; risk ratio; NT=6; NP=8,904. | **Number of trials:** 5.  **Covariate distribution (across trials):**  NT=1, NP=332 (Rifampicin 3 months versus INH 6 months (in adults with silicosis): At 5 years).  NT=1, NP=373 (Rifampicin 4 months versus INH 6 months (in adult prisoners with LTBI): At 3 years).  NT=1, NP=100 (Rifampicin 4 months versus INH 6 months (in children): At 2 years).  NT=1, NP=328 (INH plus rifampicin 3months versus INH 6months (in adults with silicosis).  NT=2, NP=368 (Rifampicin plus pyrazinamide 2 months versus INH 6 months (in adults).  NT=1, NP=100 (Rifampicin plus pyrazinamide 2 months versus INH 6 months (in children).  **Covariate distribution (within trials):**  NT=1 (Rifampicin 3 months versus INH 6 months (in adults with silicosis): At 5 years):  Less than 65 years ( 1 trial).  NT=1 (Rifampicin 4 months versus INH 6 months (in adult prisoners with LTBI): At 3 years):  age>18 yrs (1 trial).  NT=1 (Rifampicin 4 months versus INH 6 months (in children): At 2 years):  <18 yrs (1 trial).  NT=1 (INH plus rifampicin 3months versus INH 6months (in adults with silicosis):  <65 years (1 trial).  NT=2 (Rifampicin plus pyrazinamide 2 months versus INH 6 months (in adults):  ‘adults’ (1 trial); ‘>1 year’ (1 trial).  NT=1 (Rifampicin plus pyrazinamide 2 months versus INH 6 months (in children):  <18 yrs (1 trial). | **Analysis type in review according to glossary 1:** Subgroup analysis.  **Reported results:** Event rates, risk ratio, and CI for trials. Risk ratio and CI, z statistic, p-value for meta-analytic subtotals.  **Where reported:** Results text and forest plot.  **Method to detect interactions reported in review:** Not reported.  **Reported results from methods to detect interactions:** For comparison 3, test for subgroup differences (Chi-square statistic, p-value, and I square statistic).  **Where reported:** Forest plot. | **Reported detected interaction:** Not reported.  **What reported:**  **1. Rifampicin versus INH**  Five trials provided data for this comparison. See Summary of findings for the main comparison for details of relative and absolute effects of the interventions linked to the overall quality of evidence for critically important and important outcomes.  **Active TB**  Three trials evaluated the development of TB but only one trial including adult Chinese men with silicosis and LTBI (HKCS 1992) reported that active TB developed over five years follow-up. The other two trials did not detect active TB over three years’ followup in prisoners with LTBI (Chan 2012), or over two years’ followup in children and adolescents at risk (Magdorf 1994). Rifampicin 600 mg/day given for three months did not differ significantly from INH 300 mg/day given for six months in proportions developing active TB (one trial, 332 participants, Analysis 1.1: subgroup 1.1.1). The cumulative percentage of active TB in those participants in this trial (HKCS 1992) evaluated over five years among those who completed their treatment without known interruption (rifampicin 142/165; INH123/167) also did not differ significantly (rifampicin 10%, INH 14%).  One arm of the four-arm HKCS 1992 trial randomized 159 participants to matching placebo for rifampicin and INH (not included in the quantitative synthesis in this review). Of the 159 participants randomized to placebo 36 (23%) developed active TB, compared to 12% in the rifampicin arm and 15% in the INH arm.The cumulative percentage of those developing active TB over the five years among 133 participants on placebo who completed their treatment without interruption was 27%.  **2. Rifampicin plus INH versus INH alone**  Two trials in adults evaluated the combination of rifampicin plus INH for three months versus INH given for six months (HKCS 1992) and for nine months (Martinez Alfaro 1998) (Summary of findings 2).  **Active TB**  Only one four-arm trial in silicosis patients reported this outcome (HKCS 1992). As with the comparison between rifampicin alone versus INH alone, the addition of INH 300 mg/day to rifampicin 600 mg/day for three months did not significantly reduce the risk of developing active TB when compared to INH 300 mg/ day given for six months (one trial, 328 participants, Analysis 2.1). However, analyses comparing the effects of INH plus rifampicin versus the placebo arm in the trial did reveal (as with rifampicin alone) significant reductions in the cumulative risk of active TB over five years of follow-up in 123/161 adults with silicosis who completed treatment with INH plus rifampicin with no known interruptions (16%) versus those who completed uninterrupted treatment with placebo (27%).  **3. Rifampicin plus pyrazinamide versus INH**  Four trials (Leung 2003; Magdorf 1994; Sanchez-Arcilla 2004; Tortajada 2005) evaluated rifampicin plus pyrazinamide given for two months versus INH given for six months (Summary of findings 3).  **Active TB**  Three trials reported this outcome. Tortajada 2005 did not detect any participant with TB during this trial that was stopped early for harms; hence comparative efficacy could not evaluated. The proportionswho developed activeTBover two to five years’ followup in adults with silicosis (Leung 2003) and in children (Magdorf 1994) did not significantly differ in those given rifampicin plus pyrazinamide compared to those given INHalone (two trials, 176 participants, Analysis 3.1).  **4. Rifapentine plus INH once a week (DOT) for three**  **months versus daily INH daily (self administered) for**  **nine months**  See Summary of findings 4 for details of relative and absolute effects of the interventions and the overall quality of evidence for critically important and important outcomes in Sterling 2011,  **Active TB**  This trial that was designed to demonstrate the non-inferiority of 12 doses of rifapentine plus INH DOT given weekly over three months compared to 270 doses of daily, self-administered INH over ninemonths.TBdeveloped in seven of 3986 people (0.2%) in the combination treatment arm versus 15 of 3745 people (0.4%) in the INHarmover 33months of follow-up after enrolment (one trial, 7731 participants, Analysis 4.1). Of those who took 100% of treatment doses, TB developed in five of 3376 subjects (0.1%) in the combination-therapy arm versus six of 2792 (0.2%) in the INH-only arm.  The combination-therapy was consistently non-inferior to the INH-only regimen in the primary analysis where the upper limit of the 95% CI of the difference was set at < 0.75%, and in sensitivity analysis when this was reduced to < 0.50%.  In this trial, close contacts of the first eligible person in a household were randomized by household, and other high-risk participants who were not part of a household were randomized individually. The risk of developing TB was similar when the results included only the first person randomized in a household, in sensitivity analysis done to adjust for the effects of clustering.The resultswere also similar after 24 months of follow-up after the last treatment. TB incidence rates did not differ disproportionately between the study sites in the US, Canada, Brazil, or Spain.  **Where reported:** Results text.  **Interaction exists based on reported results:** Not for comparison 3.  **How determined:**  Rifampicin plus pyrazinamide 2 months versus INH 6 months (in adults)  Rifampicin plus pyrazinamide 2 months versus INH 6 months (in children)  Test for subgroup differences: Chi2= 0.32, df= 1 (P= 0.57), I2=0.0%  **Reported importance of the interaction:** Not reported.  **What reported:** Not reported.  **Where reported:** NA  **Reported plausibility of the interaction:** Not reported.  **What reported:** Not reported.  **Where reported:** NA  **Reported confounding possibility:** Not reported.  **What reported:** Not reported.  **Where reported:** NA.  **Mentioned covariate distribution:** Not reported.  **What reported:** Not reported.  **Where reported:** NA. |
| Sharma 2013 | **Protocol:** Not reported.  **Review methods:** Not reported.  **Review results: ‘**Comparison 1. Rifampicin versus INH: Rifampicin 3 months versus INH 6 months (in adults with silicosis): At 5 years’; ‘Comparison 1. Rifampicin versus INH: Rifampicin 4 months versus INH 6 months (in adult prisoners with LTBI): At 3 years’; ‘Comparison 1. Rifampicin versus INH: Rifampicin 4 months versus INH 6 months (in children): At 2 years’. ‘Comparison 2. Rifampicin plus INH versus INH): INH plus rifampicin 3 months versus INH 6months (in adults with silicosis)’; ‘Comparison 3. Rifampicin plus pyrazinamide versus INH): Rifampicin plus pyrazinamide 2 months versus INH 6 months (in adults)’; ‘Comparison 3. Rifampicin plus pyrazinamide versus INH): Rifampicin plus pyrazinamide 2 months versus INH 6 months (in children)’; ‘Comparison 4. Rifapentine plus INH weekly for 3 months versus INH daily for 9 months’ (in plot). ‘Rifampicin versus INH; Rifampicin plus INH versus INH alone’; ‘Rifampicin plus pyrazinamide versus INH’; ‘Rifapentine plus INH once a week (DOT) for three months versus daily INH daily (self administered) for nine months’ (in text).  **Type:** Intervention.  **Covariate summary:** Type of intervention or control. | Rates of active tuberculosis; primary; dichotomous; risk ratio; NT=6; NP=8,904. | **Number of trials:** 6.  **Covariate distribution (across trials):**  NT=3, NP=805 (Rifampicin versus INH);  NT=1, NP=328 (Rifampicin plus INH versus INH);  NT=3, NP=468 (Rifampicin plus pyrazinamide versus INH);  NT=1, NP=7,731 (Rifapentine plus INH weekly for 3 months versus INH daily for 9 months).  **Covariate distribution (within trials):** NA. | **Analysis type in review according to glossary 1:** Subgroup analysis.  **Reported results:** Event rates, risk ratio, and CI for trials. Risk ratio, CI, z statistic, and p-value for meta-analytic subtotals.  **Where reported:** Results text and forest plot.  **Method to detect interactions reported in review:** Not reported.  **Reported results from methods to detect interactions:** Not reported.  **Where reported:** NA. | **Reported detected interaction:** Not reported.  **What reported:**  ‘**1. Rifampicin versus INH**  Five trials provided data for this comparison. See Summary of findings for the main comparison for details of relative and absolute effects of the interventions linked to the overall quality of evidence for critically important and important outcomes.  **Active TB**  Three trials evaluated the development of TB but only one trial including adult Chinese men with silicosis and LTBI (HKCS 1992) reported that active TB developed over five years follow-up. The other two trials did not detect active TB over three years’ followup in prisoners with LTBI (Chan 2012), or over two years’ followup in children and adolescents at risk (Magdorf 1994). Rifampicin 600 mg/day given for three months did not differ significantly from INH 300 mg/day given for six months in proportions developing active TB (one trial, 332 participants, Analysis 1.1: subgroup 1.1.1). The cumulative percentage of active TB in those participants in this trial (HKCS 1992) evaluated over five years among those who completed their treatment without known interruption (rifampicin 142/165; INH123/167) also did not differ significantly (rifampicin 10%, INH 14%).  One arm of the four-arm HKCS 1992 trial randomized 159 participants to matching placebo for rifampicin and INH (not included in the quantitative synthesis in this review). Of the 159 participants randomized to placebo 36 (23%) developed active TB, compared to 12% in the rifampicin arm and 15% in the INH arm.The cumulative percentage of those developing active TB over the five years among 133 participants on placebo who completed their treatment without interruption was 27%.  **2. Rifampicin plus INH versus INH alone**  Two trials in adults evaluated the combination of rifampicin plus INH for three months versus INH given for six months (HKCS 1992) and for nine months (Martinez Alfaro 1998) (Summary of findings 2).  **Active TB**  Only one four-arm trial in silicosis patients reported this outcome (HKCS 1992). As with the comparison between rifampicin alone versus INH alone, the addition of INH 300 mg/day to rifampicin 600 mg/day for three months did not significantly reduce the risk of developing active TB when compared to INH 300 mg/ day given for six months (one trial, 328 participants, Analysis 2.1). However, analyses comparing the effects of INH plus rifampicin versus the placebo arm in the trial did reveal (as with rifampicin alone) significant reductions in the cumulative risk of active TB over five years of follow-up in 123/161 adults with silicosis who completed treatment with INH plus rifampicin with no known interruptions (16%) versus those who completed uninterrupted treatment with placebo (27%).  **3. Rifampicin plus pyrazinamide versus INH**  Four trials (Leung 2003; Magdorf 1994; Sanchez-Arcilla 2004; Tortajada 2005) evaluated rifampicin plus pyrazinamide given for two months versus INH given for six months (Summary of findings 3).  **Active TB**  Three trials reported this outcome. Tortajada 2005 did not detect any participant with TB during this trial that was stopped early for harms; hence comparative efficacy could not evaluated. The proportionswho developed activeTBover two to five years’ followup in adults with silicosis (Leung 2003) and in children (Magdorf 1994) did not significantly differ in those given rifampicin plus pyrazinamide compared to those given INHalone (two trials, 176 participants, Analysis 3.1).  **4. Rifapentine plus INH once a week (DOT) for three**  **months versus daily INH daily (self administered) for**  **nine months**  See Summary of findings 4 for details of relative and absolute effects of the interventions and the overall quality of evidence for critically important and important outcomes in Sterling 2011,  **Active TB**  This trial that was designed to demonstrate the non-inferiority of 12 doses of rifapentine plus INH DOT given weekly over three months compared to 270 doses of daily, self-administered INH over ninemonths.TBdeveloped in seven of 3986 people (0.2%) in the combination treatment arm versus 15 of 3745 people (0.4%) in the INHarmover 33months of follow-up after enrolment (one trial, 7731 participants, Analysis 4.1). Of those who took 100% of treatment doses, TB developed in five of 3376 subjects (0.1%) in the combination-therapy arm versus six of 2792 (0.2%) in the INH-only arm.  The combination-therapy was consistently non-inferior to the INH-only regimen in the primary analysis where the upper limit of the 95% CI of the difference was set at < 0.75%, and in sensitivity analysis when this was reduced to < 0.50%.  In this trial, close contacts of the first eligible person in a household were randomized by household, and other high-risk participants who were not part of a household were randomized individually. The risk of developing TB was similar when the results included only the first person randomized in a household, in sensitivity analysis done to adjust for the effects of clustering.The resultswere also similar after 24 months of follow-up after the last treatment. TB incidence rates did not differ disproportionately between the study sites in the US, Canada, Brazil, or Spain. ‘  **Where reported:** Results text.  **Interaction exists based on reported results:** Not reported.  **How determined:** NA.  **Reported importance of the interaction:** Not reported.  **What reported:** Not reported.  **Where reported:** NA  **Reported plausibility of the interaction:** Not reported.  **What reported:** Not reported.  **Where reported:** NA  **Reported confounding possibility:** Not reported.  **What reported:** Not reported.  **Where reported:** NA.  **Mentioned covariate distribution:** Yes.  **What reported:** as above.  **Where reported:** Results text. |
| Sharma 2013 | **Protocol:** Not reported.  **Review methods:** Not reported.  **Review results:** Time point. 5 years, 3 years.  **Type:** Outcome.  **Covariate summary:** Time point. | Rates of active tuberculosis; primary; dichotomous; risk ratio; NT=6; NP=8,904. | **Number of trials:** 3.  **Covariate distribution (across trials):**  NT=1, NP=332 (Rifampicin 3 months versus INH 6 months (in adults with silicosis): At 5 years);  NT=1, NP=373 (Rifampicin 4 months versus INH 6 months (in adult prisoners with LTBI): At 3 years);  NT=1, NP=100 (Rifampicin 4 months versus INH 6 months (in children): At 2 years).  **Covariate distribution (within trials):** NA. | **Analysis type in review according to glossary 1:** Subgroup analysis.  **Reported results:** Event rates, risk ratio, and CI for trials. Risk ratio, CI, z statistic, and p-value for meta-analytic subtotals and total.  **Where reported:** Results text and forest plot.  **Method to detect interactions reported in review:** Not reported.  **Reported results from methods to detect interactions:** Not reported.  **Where reported:** NA. | **Reported detected interaction:** Not reported.  **What reported:**  ‘Three trials evaluated the development of TB but only one trial including adult Chinese men with silicosis and LTBI (HKCS 1992) reported that active TB developed over five years follow-up. The other two trials did not detect active TB over three years’ followup in prisoners with LTBI (Chan 2012), or over two years’ followup in children and adolescents at risk (Magdorf 1994). Rifampicin 600 mg/day given for three months did not differ significantly from INH 300 mg/day given for six months in proportions developing active TB (one trial, 332 participants, Analysis 1.1: subgroup 1.1.1). The cumulative percentage of active TB in those participants in this trial (HKCS 1992) evaluated over five years among those who completed their treatment without known interruption (rifampicin 142/165; INH123/167) also did not differ significantly (rifampicin 10%, INH 14%).  One arm of the four-arm HKCS 1992 trial randomized 159 participants to matching placebo for rifampicin and INH (not included in the quantitative synthesis in this review). Of the 159 participants randomized to placebo 36 (23%) developed active TB, compared to 12% in the rifampicin arm and 15% in the INH arm.The cumulative percentage of those developing active TB over the five years among 133 participants on placebo who completed their treatment without interruption was 27%.’  **Where reported:** Results text.  **Interaction exists based on reported results:** Not reported.  **How determined:** NA.  **Reported importance of the interaction:** Not reported.  **What reported:** Not reported.  **Where reported:** NA  **Reported plausibility of the interaction:** Not reported.  **What reported:** Not reported.  **Where reported:** NA  **Reported confounding possibility:** Not reported.  **What reported:** Not reported.  **Where reported:** NA.  **Mentioned covariate distribution:** Yes.  **What reported:** as above.  **Where reported:** Results text. |
| Showell 2013 | **Protocol:** Not reported.  **Review methods: ‘**Type of antioxidant, whether individual or combined (three or more antioxidants combined).’  **Review results: ‘**Type of antioxidant (N-acetyl-cysteine , L-arginine , Myo-Inositol versus d-chiro-inositol1, Pentoxifylline plus vitamin E)’.  **Type:** Intervention.  **Covariate summary:** Type of intervention or control. | Live birth rate per woman randomly assigned (defined as the delivery of one or more living infants); primary; dichotomous; odds ratio; NT=4; NP=293. | **Number of trials:** 2 (comparison 1); 1 (comparison 2); 1 (comparison 3).  **Covariate distribution (across trials):**  NT=2, NP=97 (Comparison 1. Antioxidant(s) versus placebo or no treatment/standard treatment):  NT=1, NP=60 (N-acetyl-cysteine);  NT=1, NP=37 (L-arginine).  NT=1, NP=84 (Comparison 2. Head to head antioxidants):  NT=1, NP=84 (Myo-Inositol versus d-chiro-inositol).  NT=1, NP=112 (Comparison 3. Pentoxifylline versus placebo or no treatment/standard care type of antioxidant):  NT=1, NP=112 (Pentoxifylline plus vitamin E).  **Covariate distribution (within trials):** NA. | **Analysis type in review according to glossary 1:** Subgroup analysis.  **Reported results:** Event rates, OR, and CI for trials. OR, CI, z statistic, and p-value for meta-analytic subtotals.  **Where reported:** Results text and forest plot.  **Method to detect interactions reported in review:** Not reported.  **Reported results from methods to detect interactions:** Not reported.  **Where reported:** NA. | **Reported detected interaction:** Not reported.  **What reported:**  1. Antioxidant supplement versus placebo, no treatment/standard treatment  1.2 Live birth; type of antioxidant  See Analysis 1.2.  Subtotals only were used for this analysis. Each comparison included only one trial.  1.2.1Nasr 2010; *N*-acetyl-cysteine versus placebo (OR 2.87, 95% CI 1.05 to 7.84, P = 0.04).  1.2.2 Battaglia 2002; compared L-arginine with placebo (OR  0.45, 95% CI 0.10 to 2.00, P = 0.30).  2. Head-to-head antioxidants  2.1 Live birth; type of antioxidant  See Analysis 2.1.  *2.1.1 Myo-inositol versus d-chiro-inositol*  Unfer 2011 reported on live birth (OR3.44, 95%CI 1.27 to 9.34, P = 0.02), measuring the effects of myo-inositol versus d-chiroinositol.  3. Pentoxifylline supplement versus placebo, no treatment/standard treatment  3.2 Live birth; type of antioxidant  See Analysis 3.2  *3.2.1 Pentoxifylline plus vitamin E versus no treatment.*  The type of antioxidant used in Aleyasin 2009 was pentoxifylline plus vitamin E versus no treatment.  **Where reported:** Results text.  **Interaction exists based on reported results:** Not reported**.**  **How determined:** NA.  **Reported importance of the interaction:** Not reported.  **What reported:** Not reported.  **Where reported:** NA  **Reported plausibility of the interaction:** Not reported.  **What reported:** Not reported.  **Where reported:** NA  **Reported confounding possibility:** Not reported.  **What reported:** Not reported.  **Where reported:** NA.  **Mentioned covariate distribution:** Yes.  **What reported:** as above.  **Where reported:** Results text. |
| Showell 2013 | **Protocol:** Not reported.  **Review methods: ‘**Trials that enrolled women with different indications for infertility (i. e. PCOS, endometriosis, unexplained infertility or poor responders).’  **Review results:** ‘Indications for subfertility (Polycystic ovary syndrome, Tubal subfertility, Varying indications)’.  **Type:** Patient.  **Covariate summary:** Disease characteristics. | Live birth rate per woman randomly assigned (defined as the delivery of one or more living infants); primary; dichotomous; odds ratio; NT=4; NP=293. | **Number of trials:** 2 (comparison 1); 1 (comparison 2); 1 (comparison 3).  **Covariate distribution (across trials):**  NT=2, NP=97 (Comparison 1. Antioxidant(s) versus placebo or no treatment/standard treatment):  NT=1, NP=60 (Polycystic ovary syndrome);  NT=1, NP=37 (Tubal subfertility).  NT=1, NP=84 (Comparison 2. Head to head antioxidants):  NT=1, NP=84 (Polycystic ovary syndrome).  NT=1, NP=112 (Comparison 3. Pentoxifylline versus placebo or no treatment/standard care type of antioxidant):  NT=1, NP=112 (Varying indications).  **Covariate distribution (within trials):**  Comparison 1: 100% polycystic ovary syndrome (1 trial); 100% tubal subfertility (1 trial).  Comparison 2: 100% polycystic ovary syndrome (1 trial).  Comparison 3: 100% varying indications (1 trial). | **Analysis type in review according to glossary 1:** Subgroup analysis.  **Reported results:** Event rates, OR, and CI for trials. OR, CI, z statistic, and p-value for meta-analytic subtotals.  **Where reported:** Results text and forest plot.  **Method to detect interactions reported in review:** Not reported.  **Reported results from methods to detect interactions:** Test for subgroup differences (Chi-square statistic, p-value, and I square statistic).  **Where reported:** Forest plot. | **Reported detected interaction:** Not reported.  1. Antioxidant supplement versus placebo, no treatment/standard treatment  1.3 Live birth rate; indications for subfertility  See Analysis 1.3.  Battaglia 2002 enrolled women with tubal subfertility undergoing IVF (OR 0.45, 95% CI 0.10 to 2.00, P = 0.30, 37 women), and Nasr 2010 enrolled women with PCOS (OR 2.87, 95% CI 1.05 to 7.84, P = 0.04, 60 women).  2. Head-to-head antioxidants  2.2 Live birth; indications for subfertility  See Analysis 2.2.  *2.2.1 Polycystic ovary syndrome*  Unfer 2011 enrolled women with PCOS.  3. Pentoxifylline supplement versus placebo, no treatment/standard treatment  3.3 Live birth; indications for subfertility  See Analysis 3.3  *3.3.1 Varying indications*  The trial Aleyasin 2009 enrolled women with varying causes of subfertility, and the cause of subfertility in 45% of these women was the male partner.  **Where reported:** Results text.  **Interaction exists based on reported results:** Yes for comparison 1.  **How determined:**  comp 1: Test for subgroup differences: Chi2= 4.06, df= 1 (P= 0.04), I2=75%.  **Reported importance of the interaction:** Not reported.  **What reported:** Not reported.  **Where reported:** NA  **Reported plausibility of the interaction:** Not reported.  **What reported:** Not reported.  **Where reported:** NA  **Reported confounding possibility:** Not reported.  **What reported:** Not reported.  **Where reported:** NA.  **Mentioned covariate distribution:** Yes.  **What reported:** as above.  **Where reported:** Results text. |
| Showell 2013 | **Protocol:** Not reported.  **Review methods: ‘**Trials that enrolled women who were also undergoing IVF or ICSI.’  **Review results:** ‘IVF/ICSI’.  **Type:** Patient.  **Covariate summary:** Disease characteristics. | Live birth rate per woman randomly assigned (defined as the delivery of one or more living infants); primary; dichotomous; odds ratio; NT=4; NP=293. | **Number of trials: Number of trials:** 1 (comparison 1); 1 (comparison 2); 1 (comparison 3).  **Covariate distribution (across trials):**  NT=1, NP=37 (Comparison 1. Antioxidant(s) versus placebo or no treatment/standard treatment):  NT=1, NP=37 (IVF/ICSI).  NT=1, NP=84 (Comparison 2. Head to head antioxidants):  NT=1, NP=84 (IVF/ICSI).  NT=1, NP=112 (Comparison 3. Pentoxifylline versus placebo or no treatment/standard care):  NT=1, NP=112 (IVF/ICSI).  **Covariate distribution (within trials):**  Comparison 1:100% IVF/ICSI (1 trial).  Comparison 2: 100% IVF/ICSI (1 trial).  Comparison 3: Not reported % IVF/ICSI (1 trial). | **Analysis type in review according to glossary 1:** Subgroup analysis.  **Reported results:** Event rates, OR, and CI for trials. OR, CI, z statistic, and p-value for meta-analytic subtotal (one group).  **Where reported:** Results text and forest plot.  **Method to detect interactions reported in review:** Not reported.  **Reported results from methods to detect interactions:** Not reported.  **Where reported:** NA. | **Reported detected interaction:** Not reported.  **What reported:**  1. Antioxidant supplement versus placebo, no treatment/standard treatment  1.4 Live birth; IVF/ICSI  See Analysis 1.4.  Only one trial (Battaglia 2002) compared antioxidants with placebo or no treatment in women having IVF/ICSI treatment and reported live birth (OR 0.45, 95% CI 0.10 to 2.00, P = 0.30, 1 RCT, 37 women) (Battaglia 2002).  2. Head-to-head antioxidants  2.3 Live birth; IVF/ICSI  See Analysis 2.3.  The women who were enrolled in Unfer 2011 were also undergoing ICSI.  3. Pentoxifylline supplement versus placebo, no treatment/standard treatment  3.4 Live Birth: IVF/ICSI  See Analysis 3.4  The women enrolled in Aleyasin 2009 were also undergoing IVF/ ICSI.  **Where reported:** Results text.  **Interaction exists based on reported results:** Not reported**.**  **How determined:** NA.  **Reported importance of the interaction:** Not reported.  **What reported:** Not reported.  **Where reported:** NA  **Reported plausibility of the interaction:** Not reported.  **What reported:** Not reported.  **Where reported:** NA  **Reported confounding possibility:** Not reported.  **What reported:** Not reported.  **Where reported:** NA.  **Mentioned covariate distribution:** Yes.  **What reported:** as above.  **Where reported:** Results text. |
| Showell 2013 | **Protocol:** Not reported.  **Review methods: ‘**The following subgroup analysess were carried out: Antioxidants versus control (placebo or no treatment/standard treatment); Antioxidants versus antioxidants or head-to-head stratification by type of antioxidant; and Pentoxifylline versus control (placebo or no treatment/standard treatment).’  **Review results: ‘**Comparison 1. Antioxidant(s) versus placebo or no treatment/standard treatment;’ ‘Comparison 2. Head to head antioxidants’; ‘Comparison 3. Pentoxifylline versus placebo or no treatment/standard care’.  **Type:** Intervention.  **Covariate summary:** Type of intervention or control. | Live birth rate per woman randomly assigned (defined as the delivery of one or more living infants); primary; dichotomous; odds ratio; NT=4; NP=293. | **Number of trials:** 2 (comparison 1); 1 (comparison 2); 1 (comparison 3).  **Covariate distribution (across trials):**  NT=2, NP=97 (Comparison 1. Antioxidant(s) versus placebo or no treatment/standard treatment):  NT=2, NP=97 (Placebo).  NT=1, NP=84 (Comparison 2. Head to head antioxidants).  NT=1, NP=112 (Comparison 3. Pentoxifylline versus placebo or no treatment/standard care):  NT=1, NP=112 (No treatment/standard treatment).  **Covariate distribution (within trials):** NA. | **Analysis type in review according to glossary 1:** Subgroup analysis.  **Reported results:** Event rates, OR, and CI for trials. OR, CI, z-statistic,and p-value for meta-analytic subtotal.  **Where reported:** Results text and forest plot.  **Method to detect interactions reported in review:** Not reported.  **Reported results from methods to detect interactions:** Not reported.  **Where reported:** NA. | **Reported detected interaction:** Not reported.  **What reported:**  1. Antioxidant supplement versus placebo, no treatment/standard treatment  Primary outcome: Live birth  1.1 Live birth; antioxidants versus placebo or no treatment/standard treatment  See Analysis 1.1.  Antioxidants were not associated with an increased live birth rate compared with placebo or no treatment (OR 1.60, 95% CI 0.70 to 3.69, P = 0.27, 2 RCTs, 97 women, I2 = 75%, very low-quality evidence). As the I2 statistic was greater than 50%, we repeated the analysis using a random-effects model, and here again, antioxidants were not associated with an increased live birth rate compared with placebo or no treatment (OR 1.25, 95% CI 0.19 to 8.26, P = 0.82, 2 RCTs, 97 women, I2 = 75%, very low-quality evidence) (Figure 5). This suggests that among subfertile women with an expected live birth rate of 37%, the rate among women using antioxidants would be between 10%and 83%(Summary of findings for the main comparison). Heterogeneity remained high, with an I2 statistic of 75%.  *1.1.1 Live birth; antioxidants versus placebo*  No evidence of a statistically significant difference in live birth was noted between antioxidant and placebo groups (OR 1.25, 95% CI 0.19 to 8.26, P = 0.82, 2 RCTs, 97 women, I2 = 75%). The high heterogeneity was possibly due to the differing populations. Battaglia 2002 enrolled women with tubal infertility undergoing IVF, and Nasr 2010 enrolled women with PCOS undergoing laparoscopic ovarian drilling. We could not perform a sensitivity analysis as only two trials were included in the placebo analysis.  2. Head-to-head antioxidants  Primary outcome: Live birth  Only one trial reported on live birth (Unfer 2011).  3. Pentoxifylline supplement versus placebo, no treatment/standard treatment  Primary outcome: Live birth  3.1 Live birth; pentoxifylline versus placebo or no treatment/standard treatment  See Analysis 3.1  *3.1.1 Live birth; pentoxifylline versus no treatment*  Only one trial (Aleyasin 2009) performed this comparison in the pentoxifylline versus no treatment subgroup (OR 1.53, 95% CI 0.68 to 3.44, P = 0.30, 112 women).  **Where reported:** Results text.  **Interaction exists based on reported results:** Not reported**.**  **How determined:** NA.  **Reported importance of the interaction:** Not reported.  **What reported:** Not reported.  **Where reported:** NA  **Reported plausibility of the interaction:** Not reported.  **What reported:** Not reported.  **Where reported:** NA  **Reported confounding possibility:** Not reported.  **What reported:** Not reported.  **Where reported:** NA.  **Mentioned covariate distribution:** Yes.  **What reported:** as above.  **Where reported:** Results text. |
| Stead 2012 | **Protocol:** ‘We will categorise the intensity of behavioural support in both intervention and control conditions based on two of the categories used in the US Guidelines (Fiore 2008); ‘Total amount of contact time’ (Categories: 1-3, 4-30, 31-90, 91-300, >300 minutes) and ‘Number of person to person sessions’ (Categories: 0*, 1-3*, 4-8, >8 [*guideline used 0-1, 2-3])’. ‘We will use the relative intensity of support (number or duration of contacts) as the main potential feature to explain any heterogeneity.’  **Review methods: ‘**We categorise the intensity of behavioural support in both intervention and control conditions based on two of the categories used in the US Guidelines (Fiore 2008): ‘Total amount of contact time’ (Categories: 0, 1 to 30*, 31 to 90, 91 to 300, > 300 minutes [*guideline categories 1 to 3 and 4-30 combined]) and ‘Number of person-to-person sessions’ (Categories: 0*, 1 to 3*, 4 to 8, > 8 [*guideline used 0 to 1, 2 to 3]).’ ‘We used the difference in intensity of support (number or duration of contacts) between intervention and control conditions as the main potential feature to explain any heterogeneity.’  **Review results:** Subgroups by ‘contrast in number of contacts between intervention & control’. ‘4 to 8 or > 8 contacts versus no contact’; ‘More than 8 contacts versus 1 to 3 contacts’; ‘4 to 8 contacts versus 1 to 3 contacts’; ‘More than 8 contacts versus 4-8 contacts’; ‘Intervention & control in same contact category’).  **Type:** Intervention.  **Covariate summary:** Intervention intensity. | smoking cessation at the longest follow-up; primary; dichotomous; risk ratio; NT=38; NP=15,506. | **Number of trials:** 38.  **Covariate distribution (across trials):**  NT=6, NP=3,762 (to 8 or > 8 contacts versus no contact);  NT=2, NP=609 (More than 8 contacts versus 1 to 3 contacts);  NT=12, NP=6,817 (4 to 8 contacts versus 1 to 3 contacts);  NT=9, NP=1,568 More than 8 contacts versus 4-8 contacts);  NT=9, NP=2,750 ( Intervention & control in same contact category).  **Covariate distribution (within trials):** NA. | **Analysis type in review according to glossary 1:** Subgroup analysis.  **Reported results:** Event rates, risk ratio, and CI for trials. Risk ratio, CI, z statistic, and p-value for meta-analytic subtotals and totals.  **Where reported:** Results text and forest plot.  **Method to detect interactions reported in review:** Not reported.  **Reported results from methods to detect interactions:** Test for subgroup differences (Chi-square statistic, p-value, and I square statistic).  **Where reported:** Forest plot. | **Reported detected interaction:** Not reported.  **What reported:**  Subgroups by difference in intensity Analysis 1.2 categorises trials based on number of contacts, with the subgroups with the largest contrast in intensities listed first and studies where the intensity of intervention and control fell into the same category shown last. There was little evidence of any dose response but the point estimate was highest for the subgroup in which controls did not have any personal contact (six trials, RR 1.25, 95% CI 1.08 to 1.45). All interventions in this subgroup of trials involved at least four contacts. Since there was little heterogeneity overall it was not surprising that subgroups did not consistently reduce heterogeneity. We did not repeat this approach for duration of intervention categories as inspection suggested that the number of studies falling into different categories was small and that further subgroup analysis could be misleading.  **Where reported:** Results text.  **Interaction exists based on reported results:** No.  **How determined:** Test for subgroup differences: Chi2= 4.43, df= 4 (P= 0.35), I2=10%.  **Reported importance of the interaction:** Not reported.  **What reported:** Not reported.  **Where reported:** NA  **Reported plausibility of the interaction:** Not reported.  **What reported:** Not reported.  **Where reported:** NA  **Reported confounding possibility:** Not reported.  **What reported:** Not reported.  **Where reported:** NA.  **Mentioned covariate distribution:** Not reported.  **What reported:** Not reported.  **Where reported:** NA. |
| Stead 2012 | **Protocol:** Not reported.  **Review methods:** Not reported.  **Review results:** Type of pharmacotherapy. ‘NRT’; ‘Bupropion’; ‘Nortriptyline’; ‘Varenicline’; ‘NRT & bupropion’; ‘Choice of pharmacotherapy’).  **Type:** Intervention  **Covariate summary:** Type of intervention or control. | smoking cessation at the longest follow-up; primary; dichotomous; risk ratio; NT=38; NP=15,506. | **Number of trials:** 38  **Covariate distribution (across trials):**  NT=27, NP=9,772 (NRT);  NT=4, NP=1,995 (Bupropion);  NT=2, NP=172 (Nortriptyline);  NT=1, NP=800 (Varenicline);  NT=2, NP=690 (NRT & bupropion);  NT=3, NP=2,077 (Choice of pharmacotherapy).  **Covariate distribution (within trials):** NA. | **Analysis type in review according to glossary 1:** Subgroup analysis.  **Reported results:** Event rates, risk ratio, and CI for trials. Risk ratio, CI, z statistic, and p-value for meta-analytic subtotals and totals.  **Where reported:** Results text and forest plot.  **Method to detect interactions reported in review:** Not reported.  **Reported results from methods to detect interactions:** Test for subgroup differences (Chi-square statistic, p-value, and I square statistic).  **Where reported:** Results text and forest plot. | **Reported detected interaction:** No interaction.  **What reported:**  There was no overall evidence of statistical heterogeneity (I² = 3%) or of differences between the subgroups defined by pharmacotherapy, so we pooled all 38 studies, including a total of over 15,000 participants. Hall 2002 contributes separate data to two subgroups in the primary meta-analysis. Twelve of the studies had point estimates below 1, that is, with higher quit rates in the less intensive condition, but all these had wide confidence intervals (CI). Only five studies detected benefits of intervention with confidence intervals that excluded 1. The estimated risk ratio (RR) was 1.16, with 95% CI 1.09 to 1.24. This suggests that increasing the intensity of behavioural support for people making a cessation attempt with the aid of pharmacotherapy typically leads to a relatively small increase in the proportion who are quit at 6 to 12 months (Figure 1, Analysis 1.1, Summary of findings for themain comparison).  The effect size was similar and also reached statistical significance in the subgroups using NRT (27 trials, RR 1.15, 95% CI 1.06 to 1.25); bupropion (four trials, RR 1.25 95% CI 1.08 to 1.44). Subgroup results were not significant for nortriptyline (two trials, RR 0.98, 95% CI 0.59 to 1.63), varenicline (one trial, RR 1.11, 95%CI 0.89 to 1.37), NRT and bupropion combined (two trials, RR 1.22, 95% CI 0.98 to 1.52) or a choice of pharmacotherapy (three trials, RR1.15, 95%CI 0.92 to 1.45).This is likely to reflect the smaller number of studies and lower precision rather than a true difference in effect. A test for differences between subgroups was not significant (p = 0.87).  **Where reported:** Results text.  **Interaction exists based on reported results:** No.  **How determined:** Test for subgroup differences: Chi2= 1.84, df= 5 (P= 0.87), I2=0.0%  **Reported importance of the interaction:** Not reported.  **What reported:** Not reported.  **Where reported:** NA  **Reported plausibility of the interaction:** Not reported.  **What reported:** Not reported.  **Where reported:** NA  **Reported confounding possibility:** Not reported.  **What reported:** Not reported.  **Where reported:** NA.  **Mentioned covariate distribution:** Yes.  **What reported:** as above.  **Where reported:** Results text. |
| Stead 2012 | **Protocol:** Not reported.  **Review methods:** Not reported.  **Review results: ‘**Duration of contact in control condition’. ‘Brief intervention’ for control’; ‘Dose response’, ‘over 30 minutes contact for control’).  **Type:** Intervention  **Covariate summary:** Duration of intervention. | smoking cessation at the longest follow-up; primary; dichotomous; risk ratio; NT=38; NP=15,506. | **Number of trials:** 38  **Covariate distribution (across trials):**  NT=20, NP=11,042 (’Brief intervention’ for control);  NT=18, NP=4,464 ( ’Dose response’, over 30 minutes contact for control)  **Covariate distribution (within trials):** NA. | **Analysis type in review according to glossary 1:** Subgroup analysis.  **Reported results:** Event rates, risk ratio, and CI for trials. Risk ratio, CI, z statistic, and p-value for meta-analytic subtotals and totals.  **Where reported:** Results text and forest plot.  **Method to detect interactions reported in review:** Not reported.  **Reported results from methods to detect interactions:** Test for subgroup differences (Chi-square statistic, p-value, and I square statistic).  **Where reported:** Forest plot. | **Reported detected interaction:** Not reported.  **What reported:**  At the suggestion of a peer reviewer we did two additional subgroup analyses. In Analysis 1.3 we categorised only by control group contact to investigate whether there was a difference between trials where the control could be categorised as a brief intervention (either no contact or up to 30 minutes) and trials which might be characterised as testing a dose-response for behavioural support, which we defined as being where the controls received more than 30 minutes of behavioural support. Twenty trials and about two thirds of the participants came in the first subgroup, and the estimate remained very close to the main analysis (RR 1.18, 95% CI 1.10 to 1.27). There were 18 trials in the ’doseresponse’ category; the point estimate was smaller and the confidence intervals included 1 (RR 1.11, 95% CI 0.99 to 1.25). This was not sensitive to excluding studies where the intervention and control fell into the same intensity category.  **Where reported:** Results text.  **Interaction exists based on reported results:** No.  **How determined:** Test for subgroup differences: Chi2= 0.74, df= 1 (P= 0.39), I2=0.0%.  **Reported importance of the interaction:** Not reported.  **What reported:** Not reported.  **Where reported:** NA  **Reported plausibility of the interaction:** Not reported.  **What reported:** Not reported.  **Where reported:** NA  **Reported confounding possibility:** Not reported.  **What reported:** Not reported.  **Where reported:** NA.  **Mentioned covariate distribution:** Yes.  **What reported:** as above.  **Where reported:** Results text. |
| Stead 2012 | **Protocol:** Not reported.  **Review methods:** Not reported.  **Review results:** ‘Modality of contact’. ‘All contact by telephone’; ‘No contact for control group’, ‘face-to-face intervention’; ‘Face-to-face contact for both intervention & control conditions’.  **Type:** Intervention.  **Covariate summary:** Type of intervention or control. | smoking cessation at the longest follow-up; primary; dichotomous; risk ratio; NT=38; NP=15,506. | **Number of trials:** 38  **Covariate distribution (across trials):**  NT=6, NP=5,311 (All contact by telephone);  NT=3, NP=2,364 (No contact for control group, face-to-face intervention);  NT=29, NP=7,831 (Face-to-face contact for both intervention & control conditions).  **Covariate distribution (within trials):** NA. | **Analysis type in review according to glossary 1:** Subgroup analysis.  **Reported results:** Event rates, risk ratio, and CI for trials. Risk ratio, CI, z statistic, and p-value for meta-analytic subtotals and totals.  **Where reported:** Results text and forest plot.  **Method to detect interactions reported in review:** Not reported.  **Reported results from methods to detect interactions:** Test for subgroup differences (Chi-square statistic, p-value, and I square statistic).  **Where reported:** Forest plot. | **Reported detected interaction:** Not reported.  **What reported:**  In Analysis 1.4, we categorised studies according to whether there was some face-to face contact as part of the intervention, or whether all support was given by telephone. In the subgroup of six studies using telephone counselling (which had some overlap with studies where there was no personal contact for the control), the point estimate was higher although the CIs were wide (RR 1.28, 95% CI 1.17 to 1.41). In the subgroup of 29 studies where both intervention and control conditions had face-to-face support, there was no evidence of benefit but the upper CI was still consistent with a possible small benefit (RR 1.09, 95% CI 0.99 to 1.19).  **Where reported:** Results text.  **Interaction exists based on reported results:** Yes.  **How determined:** Test for subgroup differences: Chi2= 6.64, df= 2 (P= 0.04), I2=70%.  **Reported importance of the interaction:** Not reported.  **What reported:** Not reported.  **Where reported:** NA  **Reported plausibility of the interaction:** Not reported.  **What reported:** Not reported.  **Where reported:** NA  **Reported confounding possibility:** Not reported.  **What reported:** Not reported.  **Where reported:** NA.  **Mentioned covariate distribution:** Yes.  **What reported:** as above.  **Where reported:** Results text. |
| Stead 2012 | **Protocol:** Not reported.  **Review methods:** Not reported.  **Review results:** Sensitivity analysis including intermediate intensity conditions. Adjunct behavioural support versus pharmacotherapy alone.  **Type:** Intervention  **Covariate summary:** Type of intervention or control. | smoking cessation at the longest follow-up; primary; dichotomous; risk ratio; NT=38; NP=15,506. | **Number of trials:** 38.  **Covariate distribution (across trials):**  NT=27, NP=11,430 (NRT);  NT=4, NP=1,995 (Bupropion );  NT=2, NP=172 (Nortriptyline );  NT=1, NP=1,202 (Varenicline);  NT=2, NP=690 (NRT & bupropion);  NT=3, NP=2,315 (Choice of pharmacotherapy).  **Covariate distribution (within trials):** NA. | **Analysis type in review according to glossary 1:** Subgroup analysis.  **Reported results:** Event rates, risk ratio, and CI for trials. Risk ratio, CI, z statistic, and p-value for meta-analytic subtotals and totals.  **Where reported:** Results text and forest plot.  **Method to detect interactions reported in review:** Not reported.  **Reported results from methods to detect interactions:** Test for subgroup differences (Chi-square statistic, p-value, and I square statistic).  **Where reported:** Forest plot. | **Reported detected interaction:** Not reported.  **What reported:**  Inclusion of medium intensity intervention from studies with  multiple intervention conditions  Seven studies (Jorenby 1995; Alterman 2001; Smith 2001; Fiore 2004; Hollis 2007; Ellerbeck 2009; Swan 2010) included an intervention condition intermediate in intensity between the highest intensity and the control. These arms were not included in the primary analysis in case they reduced the contrast between intervention and control. In a sensitivity analysis we added in these arms. This had almost no impact on the estimated effect (RR 1.15, 95%CI 1.08 to 1.22, Analysis 2.1), tending to support the finding that there is not a clear dose-response relationship with amount of support.  **Where reported:** Results text.  **Interaction exists based on reported results**: No.  **How determined:** Test for subgroup differences: Chi2= 2.25, df= 5 (P= 0.81), I2=0.0%.  **Reported importance of the interaction:** Not reported.  **What reported:** Not reported.  **Where reported:** NA  **Reported plausibility of the interaction:** Not reported.  **What reported:** Not reported.  **Where reported:** NA  **Reported confounding possibility:** Not reported.  **What reported:** Not reported.  **Where reported:** NA.  **Mentioned covariate distribution:** Not reported.  **What reported:** Not reported.  **Where reported:** NA. |
| Stead 2012 | **Protocol:** Not reported.  **Review methods:** Not reported.  **Review results:** Setting. ‘Recruited and treatment initiated in health care setting’; ‘Members of health care organisation’; ‘Community volunteers’).  **Type:** Patient.  **Covariate summary:** Setting. | smoking cessation at the longest follow-up; primary; dichotomous; risk ratio; NT=38; NP=15,506. | **Number of trials:** 38  **Covariate distribution (across trials):**  NT=12, NP=5,422 (Recruited and treatment initiated in health care setting);  NT=3, NP=2,833 (Members of health care organisation);  NT=23, NP=7,251 (Community volunteers).  **Covariate distribution (within trials):**  100% recruited and treatment initiated in health care setting (12 trials );  100% members of health care organisation (3 trials);  100% community volunteers (23 trials). | **Analysis type in review according to glossary 1:** Subgroup analysis.  **Reported results:** Event rates, risk ratio, and CI for trials. Risk ratio, CI, z statistic, and p-value for meta-analytic subtotals and totals.  **Where reported:** Forest plot.  **Method to detect interactions reported in review:** Not reported.  **Reported results from methods to detect interactions:** Test for subgroup differences (Chi-square statistic, p-value, and I square statistic).  **Where reported:** Forest plot. | **Reported detected interaction:** Not reported.  **What reported:** Not reported.  **Where reported:** NA.  **Interaction exists based on reported results:** No.  **How determined:** Test for subgroup differences: Chi2= 1.18, df= 2 (P= 0.55), I2=0.0%  **Reported importance of the interaction:** Not reported.  **What reported:** Not reported.  **Where reported:** NA  **Reported plausibility of the interaction:** Not reported.  **What reported:** Not reported.  **Where reported:** NA  **Reported confounding possibility:** Not reported.  **What reported:** Not reported.  **Where reported:** NA.  **Mentioned covariate distribution:** Not reported.  **What reported:** Not reported.  **Where reported:** NA. |
| Stead 2012 | **Protocol:** The use of biochemical validation of cessation. We judged the following to be three covariates: ‘We will also consider whether the definition and duration of follow up, or the use of biochemical validation of cessation, has any impact on treatment effect.’  **Review methods:** The use of biochemical validation of cessation. We judged the following to be three covariates: ‘We also considered whether the definition and duration of follow-up, or the use of biochemical validation of cessation, had any impact on treatment effect.’  **Review results:** By outcome definition. ‘12m validation PP outcomes only’; ‘12m validated sustained outcomes’; ‘Not 12m’; ‘No validation at all’).  **Type:** Outcome.  **Covariate summary:** Use of biochemical validation. | smoking cessation at the longest follow-up; primary; dichotomous; risk ratio; NT=38; NP=15,506. | **Number of trials:** 38.  **Covariate distribution (across trials):**  NT=14, NP=3,202 (12m validation PP outcomes only);  NT=7, NP=2,322 (12m validated sustained outcomes);  NT=9, NP=2,783 (Not 12m);  NT=8, NP=7,199 (No validation at all)  **Covariate distribution (within trials):** NA. | **Analysis type in review according to glossary 1:** Subgroup analysis.  **Reported results:** Event rates, risk ratio, and CI for trials. Risk ratio, CI, z statistic, and p-value for meta-analytic subtotals and totals.  **Where reported:** Results text and forest plot.  **Method to detect interactions reported in review:** Not reported.  **Reported results from methods to detect interactions:** Test for subgroup differences (Chi-square statistic, p-value, and I square statistic).  **Where reported:** Forest plot. | **Reported detected interaction:** Not reported.  **What reported:**  Definition of abstinence & use of validation  We considered whether the way in which abstinence was defined was related to the effect size, and also to absolute quit rates. We did not find any difference in relative effect between studies that reported point prevalence rather than sustained abstinence at 12 months (Analysis 2.2). Some studies that reported sustained outcomes also reported point prevalence rates, but substituting the less stringent definition did not change the overall findings. However, studies with point prevalence outcomes had, on average, higher quit rates in both intervention and control arms. A study comparing outcomes based on different abstinence definitions reported within studies found that, for pharmacotherapy studies, point prevalence and sustained abstinence outcomes were strongly related, with sustained abstinence averaging around 74% of point prevalence rates (Hughes 2010). In this review there was a bigger difference, and the average quit rate in studies that reported a sustained outcome was 50 to 60%of the average for studies reporting point prevalence.  Most of the studies used biochemical validation of self-reported cessation. When we excluded the six studies that did not, the estimated effect fell slightly and the confidence intervals no longer excluded 1 (RR 1.09, 95% CI 0.99 to 1.21), whilst the effect in the excluded subgroup without validation supported a benefit (RR 1.23, 95% CI 1.12 to 1.34). The importance of this is unclear; whilst it is possible that the absence of validation was a source of bias, it is also possible that these studies had genuinely larger effects; five involved telephone counselling, and two had no personal contact in the control, potentially leading to a larger contrast between intervention and control conditions.  **Where reported:** Results text.  **Interaction exists based on reported results:** Yes.  **How determined:** Test for subgroup differences: Chi2= 7.18, df= 3 (P= 0.07), I2=58%  **Reported importance of the interaction:** Not reported.  **What reported:** Not reported.  **Where reported:** NA  **Reported plausibility of the interaction:** Not reported.  **What reported:** Not reported.  **Where reported:** NA  **Reported confounding possibility:** Not reported.  **What reported:** Not reported.  **Where reported:** NA.  **Mentioned covariate distribution:** Not reported.  **What reported:** Not reported.  **Where reported:** NA. |
| Trotti 2012 | **Protocol:** ‘Different types of patients (e. g. , pregnant women or patients with ESRD). We judged the following to be two covariates: ‘We will perform subgroup analyses of different types of patients (e. g. , pregnant women or patients with ESRD) and different formulations of iron (oral versus parenteral).’  **Review methods: ‘**Different types of patients (e. g. , pregnant women or patients with ESRD). We judged the following to be two covariates: ‘We prespecified subgroup analyses to examine the effects of different types of patients (e. g. , pregnant women or patients with ESRD) and different formulations of iron (oral versus parenteral).’  **Review results:** Patients with ESRD.  **Type:** Patient.  **Covariate summary:** Disease characteristics. | restlessness or unpleasant sensations as experienced subjectively by the patient; primary; continuous; mean difference; NT=5; NP=164. | **Number of trials:** 1.  **Covariate distribution (across trials):**  NT=1, NP=25 **(**ESRD).  **Covariate distribution (within trials):**  100% ESRD (1 trial). | **Analysis type in review according to glossary 1:** Sensitivity analysis.  **Reported results:** Text.  **Where reported:** Results text.  **Method to detect interactions reported in review:** Not reported.  **Reported results from methods to detect interactions:** Not reported.  **Where reported:** NA. | **Reported detected interaction:** Not reported.  **What reported:**  ‘Subgroup analyses by patient type were possible for the subgroups of ESRD and baseline iron deficiency. Only one study explicitly included patients with ESRD (Sloand 2004), and this study  did show a significant improvement in RLS severity scores at 2 weeks.’  **Where reported:** Results text.  **Interaction exists based on reported results:** Not reported.  **How determined:** NA.  **Reported importance of the interaction:** Not reported.  **What reported:** Not reported.  **Where reported:** NA  **Reported plausibility of the interaction:** Not reported.  **What reported:** Not reported.  **Where reported:** NA  **Reported confounding possibility:** Not reported.  **Mentioned covariate distribution:** Yes.  **What reported:** as aabove.  **Where reported:** Results text. |
| Trotti 2012 | **Protocol:** Different formulations of iron (oral versus parenteral). We judged the following to be two covariates: ‘We will perform subgroup analyses of different types of patients (e. g. , pregnant women or patients with ESRD) and different formulations of iron (oral versus parenteral).’  **Review methods:** Different formulations of iron (oral versus parenteral). We judged the following to be two covariates: ‘We prespecified subgroup analyses to examine the effects of different types of patients (e. g. , pregnant women or patients with ESRD) and different formulations of iron (oral versus parenteral).’  **Review results:** IV iron.  **Type:** Intervention.  **Covariate summary:** Route of administration. | restlessness or unpleasant sensations as experienced subjectively by the patient; primary; continuous; mean difference; NT=5; NP=164. | **Number of trials:** 3.  **Covariate distribution (across trials):**  NT=3, NP=121 **(**IV iron).  **Covariate distribution (within trials):** NA. | **Analysis type in review according to glossary 1:** Sensitivity analysis.  **Reported results:** Mean, SD and N by group for trials. Mean difference and CI for trials. Mean difference, CI, z statistic, and p-value for meta-analytic subtotal.  **Where reported:** Forest plot.  **Method to detect interactions reported in review:** Not reported.  **Reported results from methods to detect interactions:** Not reported.  **Where reported:** NA. | **Reported detected interaction:** Not reported.  **What reported:**  ‘Considering type of iron delivery (oral versus intravenous), the one study to use both oral iron and a measure of RLS severity (Wang 2009) found a significant improvement in RLS severity with treatment, while intravenous iron was associated with significant improvements in two of three studies (Allen 2009, Sloand 2004).’  ‘There was moderate heterogeneity for our primary outcome measure (I2= 52%). Heterogenity decreased when only IV studies were considered together, suggesting that method of iron administration explained some of the observed heterogeneity.’  ‘The IV studies measured outcomes at 2-3 weeks and the oral studies at 12 weeks, so heterogeneity caused by difference in time of outcome assessment cannot be separated from difference in method of administration.’  **Where reported:** Results text.  **Interaction exists based on reported results**: Not reported.  **How determined:** NA.  **Reported importance of the interaction:** Not reported.  **What reported:** Not reported.  **Where reported:** NA  **Reported plausibility of the interaction:** Not reported.  **What reported:** Not reported.  **Where reported:** NA  **Reported confounding possibility:** Yes.  **What reported:** ‘The IV studies measured outcomes at 2-3 weeks and the oral studies at 12 weeks, so heterogeneity caused by difference in time of outcome assessment cannot be separated from difference in method of administration.’  **Where reported:** Results text.  **Mentioned covariate distribution:** Yes.  **What reported:** as above.  **Where reported:** Results text. |
| Trotti 2012 | **Protocol:** Not reported.  **Review methods:** Not reported.  **Review results: ‘**Iron deficient’.  **Type:** Patient.  **Covariate summary:** Disease characteristics. | restlessness or unpleasant sensations as experienced subjectively by the patient; primary; continuous; mean difference; NT=5; NP=164. | **Number of trials:** 2.  **Covariate distribution (across trials):**  NT=2, NP=78 **(**Iron deficient).  **Covariate distribution (within trials):** s-ferritin < 45 (1 trial); ferritin 15-75 (1 trial). | **Analysis type in review according to glossary 1:** Sensitivity analysis.  **Reported results:** Mean, SD and N by group for trials. Mean difference and CI for trials. Mean difference, CI, z statistic and p-value for meta-analytic subtotal.  **Where reported:** Forest plot.  **Method to detect interactions reported in review:** Not reported.  **Reported results from methods to detect interactions:** Not reported.  **Where reported:** NA. | **Reported detected interaction:** Not reported.  **What reported:**  ‘One study included only subjects who were iron deficient (as defined by a serum ferritin < 45) (Grote 2009), with an additional study limited to those with low and low-normal iron store  (as defined by serum ferritin levels between 15 and 75) (Wang 2009). Neither the Grote study alone nor both studies in combination found an improvement in IRLS scores in the treated group  compared to placebo for our specified time points, although the Grote study did find a significant improvement at another time point (albeit not their prespecified primary endpoint). Two studies  (Allen 2009, Davis 2000) reported that baseline iron stores were not different between subjects who responded to iron therapy and those who did not.’  ‘Considering only those studies in which subjects had low or low-normal iron status at baseline resulted in greater heterogeneity (I2= 77%), suggesting that baseline iron stores did not  account for observed heterogeneity.’  **Where reported:** Results text.  **Interaction exists based on reported results**: Not reported.  **How determined:** NA.  **Reported importance of the interaction:** Not reported.  **What reported:** Not reported.  **Where reported:** NA  **Reported plausibility of the interaction:** Not reported.  **What reported:** Not reported.  **Where reported:** NA  **Reported confounding possibility:** Not reported.  **What reported:** Not reported.  **Where reported:** NA.  **Mentioned covariate distribution:** Not reported.  **What reported:** Not reported.  **Where reported:** NA. |
| van Zuuren 2013 | **Protocol:** Not reported.  **Review methods:** Not reported.  **Review results:** Outcome time point. Day 2 and 3, day 4.  **Type:** Outcome.  **Covariate summary:** Time point. | Pain i) Intensity (expressed as scores obtained through any validated patient-reported outcomes instrument, either generic or SCD specific) ii) Duration; primary; continuous; mean difference for duration, p-value for intensity; NT=1; NP=253 (same numbers for each outcome). | **Number of trials:** 1.  **Covariate distribution (across trials):**  NT=1, NP=253 (‘day 2 and 3’);  NT=1, NP=253 (‘day 4’).  **Covariate distribution (within trials):** NA. | **Analysis type in review according to glossary 1:** Subgroup analysis.  **Reported results:** p-value ANOVA from one trial at 2 time points.  **Where reported:** Results text.  **Method to detect interactions reported in review:** Not reported.  **Reported results from methods to detect interactions:** Not reported.  **Where reported:** NA. | **Reported detected interaction:** Not reported.  **What reported:** ‘***a. Intensity (expressed as scores obtained through any validated patient-reported outcomes instrument, either generic or SCD specific)***  No precise datawere reported; therefore, thesewere estimated from the graph-plot in the report.The authors indicated that at days two and three, the pain severity score was lower in the tinzaparin group than in the placebo group (P < 0.01 (ANOVA)), in addition to that  at day 4 (P < 0.05 (ANOVA)), and thus that tinzaparin resulted in more rapid resolution of pain as measured by the numerical pain scale (NMS).’  **Where reported:** resultrs text.  **Interaction exists based on reported results:** Not reported.  **How determined:** NA.  **Reported importance of the interaction:** Not reported.  **What reported:** Not reported.  **Where reported:** NA  **Reported plausibility of the interaction:** Not reported.  **What reported:** Not reported.  **Where reported:** NA  **Reported confounding possibility:** Not reported.  **What reported:** Not reported.  **Where reported:** NA.  **Mentioned covariate distribution:** Yes.  **What reported:** as above.  **Where reported:** resultrs text. |
| Wakai 2013 | **Protocol:** Not reported.  **Review methods:** Not reported.  **Review results:** Time point: 30 minutes, 60 minutes, 3 hours, 24 hours  **Type:** Outcome.  **Covariate summary:** Time point. | Rapidity with which symptoms (for example dyspnoea, fatigue, self reported patient satisfaction score, global clinical status) are relieved; primary; continuous; mean difference (1 trial) and no results for a second trial; NT=2; NP=558. | **Number of trials:** 1.  **Covariate distribution (across trials):**  NT=1, NP=69 (30 minutes);  NT=1, NP=69 (60 minutes);  NT=1, NP=69 (3 hours);  NT=1, NP=69 (24 hours).  **Covariate distribution (within trials):** NA. | **Analysis type in review according to glossary 1:** Subgroup analysis.  **Reported results:** Mean difference, CI from trials.  **Where reported:** Results text.  **Method to detect interactions reported in review:** Not reported.  **Reported results from methods to detect interactions:** Not reported.  **Where reported:** NA. | **Reported detected interaction:** Not reported.  **What reported:**  ‘Rapidity of symptom relief  Two studies reported the rapidity of symptom relief, the primary outcome measure of this review. It was not possible to pool the results of these two trials because the comparator interventions were different and the rapidity of symptom relief was measured and reported in different ways in the two studies. Beltrame 1998 reported no significant difference in the dyspnoea score (0 to 3) between intravenous nitroglycerin/N-acetylcysteine and intravenous frusemide/morphine at 30 minutes (fixed-effect mean difference (MD) -0.30, 95%confidence interval (CI) -0.65 to 0.05), 60minutes (fixed-effect MD -0.20, 95% CI -0.65 to 0.25), three hours (fixed-effectMD0.20, 95%CI -0.27 to 0.67) and 24 hours (fixedeffect MD 0.00, 95% CI -0.31 to 0.31). VMAC 2002 reported the patient’s self evaluation of dyspnoea (all patients) and global clinical status at three, six and 24 hours, respectively, after the start of the study drug. Global clinical status was rated by the patient on a five-category scale (markedly better, better, no change, worse or markedly worse). Dyspnoea was rated by the patient on a threecategory scale (improved, no change or worse). However, the results were reported graphically and sufficient detail about the exact scores for these end points was not published (VMAC 2002).’ **Where reported:** Results text.  **Interaction exists based on reported results:** Not reported.  **How determined:** NA.  **Reported importance of the interaction:** Not reported.  **What reported:** Not reported.  **Where reported:** NA  **Reported plausibility of the interaction:** Not reported.  **What reported:** Not reported.  **Where reported:** NA  **Reported confounding possibility:** Not reported.  **What reported:** Not reported.  **Where reported:** NA.  **Mentioned covariate distribution:** Yes.  **What reported:** as above.  **Where reported:** Results text. |
| Wang 2013 | **Protocol:** ‘The following comparisons will be addressed: 1. Acupuncture versus placebo or no treatment,. 2. Acupuncture versus any other treatment.’  **Review methods: ‘**The following comparisons will be addressed: 1. Acupuncture versus placebo or no treatment. 2. Acupuncture versus any other treatment.’  **Review results:** ‘Acupuncture versus placebo or no treatment’; ‘Acupuncture versus active treatment’.  **Type:** Intervention.  **Covariate summary:** Type of intervention or control. | Number of participants with incontinence; primary; dichotomous; risk ratio; NT=1; NP=60. | **Number of trials:** 1.  **Covariate distribution (across trials):**  NT=1, NP=60 (‘Acupuncture versus active treatment’);  NT=0, NP=0 (‘Acupuncture versus placebo or no treatment’).  **Covariate distribution (within trials):** NA. | **Analysis type in review according to glossary 1:** Subgroup analysis.  **Reported results:** Event rates, risk ratio, and CI for trials.  **Where reported:** Results text and forest plot.  **Method to detect interactions reported in review:** Not reported.  **Reported results from methods to detect interactions:** Not reported.  **Where reported:** NA. | **Reported detected interaction:** Not reported.  **What reported:**  ‘Acupuncture versus placebo or no treatment: We found no trials.  Acupuncture versus active treatment: The one small included trial compared electroacupuncture versus a drug (midodrine) (Bi 2007). Acupuncture seemed to be better than the drug alone in terms of number of women improved (73% with acupuncture versus 33% with midodrine; RR 2.20, 95% CI 1.27 to 3.81; Analysis 2.1; Figure 2), but not for cure rates (13% with acupuncture versus 7% with midodrine; RR 2.00, 95% CI 0.40 to 10.11; Analysis 2.2; Figure 3).’  **Where reported:** Results text.  **Interaction exists based on reported results:** Not reported.  **How determined:** NA.  **Reported importance of the interaction:** Not reported.  **What reported:** Not reported.  **Where reported:** NA  **Reported plausibility of the interaction:** Not reported.  **What reported:** Not reported.  **Where reported:** NA  **Reported confounding possibility:** Not reported.  **What reported:** Not reported.  **Where reported:** NA.  **Mentioned covariate distribution:** Yes.  **What reported:** As above.  **Where reported:** Results text. |
| Yue 2013 | **Protocol:** ‘Children (<18 years) and adults (>18 years)’.  **Review methods: ‘**Children (<18 years) and adults (>18 years)’.  **Review results: ‘**Children (<18 years) and adults (>18 years)’ (in text), ‘Adults subgroup (≥18 years)’ (in plot).  **Type:** Patient.  **Covariate summary:** Demographics. | 1. Clinical cure (resolution of symptoms and signs) and microbiological cure (eradication of bacteria on wound culture); primary; dichotomous; risk ratio; NT=9; NP=3,114 (for clinical cure); NT=9; NP=2,014 (for microbiological cure). | **Number of trials:** 5 (clinical cure); 5 (microbiological cure).  **Covariate distribution (across trials):**  Clinical cure:  NT=1, NP=120 (‘children (<18 years)’);  NT=5, NP=2,402 (‘adults (>18 years)’).  Microbiological cure:  NT=1, NP=120 (‘children (<18 years)’);  NT=5, NP=1,458 (‘adults (>18 years)’).  **Covariate distribution (within trials):**  Average age per treatment group (for all trials that could have been included in the analysis):  49.7, 49.4 years (1 trial);  68.4, 67.5 years (1 trial);  56.3, 59.6 years (1 trial);  66, 76 years (1 trial);  52, 52 years (1 trial);  3.48, 3.03 years (1 trial);  47.2, 48.1 years (1 trial);  63.9, 59.8 years (1 trial);  53.7, 53.8 years (1 trial). | **Analysis type in review according to glossary 1:** Subgroup analysis.  **Reported results:** Event rates, risk ratio, and CI for trials. Risk ratio, CI, z-statistic, and p-value for meta-analytic subtotals.  **Where reported:** Results text and forest plot.  **Method to detect interactions reported in review:** Not reported.  **Reported results from methods to detect interactions:** Not reported.  **Where reported:** NA. | **Reported detected interaction:** Not reported.  **What reported:**  ‘There was only one study (120 participants) in children (Yogev 2003), though five RCTs (2402 participants) included adults (18 years or over) with SSTI (Itani 2010; Kohno 2007; Lin 2008; Sharpe 2005; Weigelt 2005). The remaining three RCTs (592 participants) included mixed populations (13 years and over) with SSTI (Jaksic 2006; Stevens 2002;Wilcox 2009). In children, there was no statistically significant difference for either clinical cure (RR 1.14; 95%CI 0.91 to 1.44) or microbiological cure (RR 1.08; 95% CI 0.90 to 1.31). In adults (18 years and over), there was a statistically significant difference in favour of linezolid for both clinical cure (RR 1.16; 95% CI 1.02 to 1.32; I2= 42%; Analysis 1.2) and microbiological cure (RR 1.17; 95% CI 1.02 to 1.34; I2= 61%; Analysis 2.2).’  **Where reported:** Results text.  **Interaction exists based on reported results**: Not reported.  **How determined:** NA.  **Reported importance of the interaction:** Not reported.  **What reported:** Not reported.  **Where reported:** NA  **Reported plausibility of the interaction:** Not reported.  **What reported:** Not reported.  **Where reported:** NA  **Reported confounding possibility:** Not reported.  **What reported:** Not reported.  **Where reported:** NA.  **Mentioned covariate distribution:** Yes.  **What reported:** as above.  **Where reported:** Results text. |
| Yue 2013 | **Protocol:** ‘Exclusion of studies in which outcome evaluation was not blinded’.  **Review methods: ‘**Exclusion of studies in which outcome evaluation was not blinded’.  **Review results: ‘**Exclusion of studies in which outcome evaluation was not blinded’.  **Type:** Methodological.  **Covariate summary:** Blinding. | 1. Clinical cure (resolution of symptoms and signs) and microbiological cure (eradication of bacteria on wound culture); primary; dichotomous; risk ratio; NT=9; NP=3,114 (for clinical cure); NT=9; NP=2,014 (for microbiological cure). | **Number of trials:** 2 (clinical cure); 2 (microbiological cure).  **Covariate distribution (across trials):**  Clinical cure:  NT=2, NP=109 (‘blinded design’).  Microbiological cure:  NT=2, NP=101 (‘blinded design’).  **Covariate distribution (within trials):** NA. | **Analysis type in review according to glossary 1:** Sensitivity analysis.  **Reported results:** risk ratio and CI for meta-analytic total.  **Where reported:** Results text.  **Method to detect interactions reported in review:** Not reported.  **Reported results from methods to detect interactions:** Not reported.  **Where reported:** NA. | **Reported detected interaction:** No interaction.  **What reported:**  ‘Only two of the nine included trials had a blinded design (Jaksic 2006; Lin 2008). After removal of the studies in which outcome evaluation was not blinded, there was no difference in treatment success for clinical cure for patients (RR 1.22, 95% CI 0.97 to 1.53), or for microbiological cure for patients (RR 1.08, 95% CI 0.85 to 1.38).’  **Where reported:** Results text.  **Interaction exists based on reported results**: Not reported.  **How determined:** NA.  **Reported importance of the interaction:** Not reported.  **What reported:** Not reported.  **Where reported:** NA  **Reported plausibility of the interaction:** Not reported.  **What reported:** Not reported.  **Where reported:** NA  **Reported confounding possibility:** Not reported.  **What reported:** Not reported.  **Where reported:** NA.  **Mentioned covariate distribution:** Yes.  **What reported:** as above.  **Where reported:** Results text. |
| Yue 2013 | **Protocol:** ‘We intend to present comparisons as follows: 1. Linezolid compared with vancomycin alone. 2. Linezolid plus co-interventions compared with vancomycin plus co-interventions’.  **Review methods: ‘**We intend to present comparisons as follows: 1. Linezolid compared with vancomycin alone. 2. Linezolid plus co-interventions compared with vancomycin plus co-interventions’.  **Review results:** ‘Linezolid compared with vancomycin’.  **Type:** Intervention.  **Covariate summary:** Additional interventions. | 1. Clinical cure (resolution of symptoms and signs) and microbiological cure (eradication of bacteria on wound culture); primary; dichotomous; risk ratio; NT=9; NP=3,114 (for clinical cure); NT=9; NP=2,014 (for microbiological cure). | **Number of trials:** 9 (clinical cure); 9 (microbiological cure).  **Covariate distribution (across trials):**  Clinical cure:  NT=9, NP=3,114 (‘Linezolid compared with vancomycin’).  Microbiological cure:  NT=9, NP=2,014 (‘Linezolid compared with vancomycin’).  **Covariate distribution (within trials):** NA. | **Analysis type in review according to glossary 1:** Subgroup analysis.  **Reported results:** Event rates, risk ratio, and CI for trials. Risk ratio, CI, z statistic, and p-value for meta-analytic subtotal.  **Where reported:** Results text and forest plot.  **Method to detect interactions reported in review:** Not reported.  **Reported results from methods to detect interactions:** Not reported.  **Where reported:** NA. | **Reported detected interaction:** Not reported.  **What reported:**  **Linezolid compared with vancomycin (nine RCTs)**  **Clinical cure**  Eight RCTs that reported outcomes in adult or mixed populations (Itani 2010; Jaksic 2006; Kohno 2007; Lin 2008; Sharpe 2005; Stevens 2002; Weigelt 2005; Wilcox 2009), and one RCT that reported outcomes in children (Yogev 2003),were included for this outcome. In total, 3114 participants with SSTIs were randomised in nine RCTs. We conducted ITT analysis for all randomised participants. We coded indeterminate outcomes and missing data as ”no cure“. Pooling of the nine trials demonstrated a statistically significant difference in cure rate of SSTIs in favour of linezolid (RR 1.09, 95% CI 1.03 to 1.16; I2 = 13%; Analysis 1.1). The NNT was 20.  **Microbiological cure**  The meta-analysis to evaluate the microbiological cure rate included 2014 SSTI participants from all nine trials with a positive culture at baseline.More SSTIs achieved microbiological cure when treated with linezolid than with vancomycin (RR 1.08, 95% CI 1.01 to 1.16; I2 = 42%; Analysis 2.1).The NNT was 20.  **Where reported:** Results text.  **Interaction exists based on reported results:** Not reported.  **How determined:** NA.  **Reported importance of the interaction:** Not reported.  **What reported:** Not reported.  **Where reported:** NA  **Reported plausibility of the interaction:** Not reported.  **What reported:** Not reported.  **Where reported:** NA  **Reported confounding possibility:** Not reported.  **What reported:** Not reported.  **Where reported:** NA.  **Mentioned covariate distribution**: Yes**.**  **What reported:** as above.  **Where reported:** Results text. |
| Yue 2013 | **Protocol:** Not reported.  **Review methods:** MRSA subset**.**  **Review results:** MRSA subset**.**  **Type:** Patient.  **Covariate summary:** Disease characteristics. | 1. Clinical cure (resolution of symptoms and signs) and microbiological cure (eradication of bacteria on wound culture); primary; dichotomous; risk ratio; NT=9; NP=3,114 (for clinical cure); NT=9; NP=2,014 (for microbiological cure). | **Number of trials:** 6 (clinical cure); 6 (microbiological cure).  **Covariate distribution (across trials):**  Clinical cure:  NT=6, NP=2,659 (‘MRSA’).  Microbiological cure:  NT=6, NP=1,289 (‘MRSA’).  **Covariate distribution (within trials):**  Clinical cure:  100% MRSA patients (3 trials); 48/151 (1 trial); 640/1052 (1 trial); not reported (1 trial).  Microbiological cure:  100% MRSA patients (3 trials); 48/151 (1 trial); 640/1052 (1 trial); not reported (1 trial) | **Analysis type in review according to glossary 1:** Sensitivity analysis.  **Reported results:** Event rates, risk ratio, and CI for trials. Risk ratio, CI, z statistic, and p-value for meta-analytic subtotal.  **Where reported:** Results text and forest plot.  **Method to detect interactions reported in review:** Not reported.  **Reported results from methods to detect interactions:** Not reported.  **Where reported:** NA. | **Reported detected interaction:** Not reported.  **What reported:**  ‘Five RCTs (2570 participants) enrolled people with MRSA infections (Itani 2010; Kohno 2007; Sharpe 2005; Stevens 2002; Weigelt 2005).One RCT enrolled people with Gram-positive bacterial infection, but reported data of MRSA as a subset (89 participants) (Wilcox 2009). Thus, six RCTs reported the clinical and microbiological cure rate of MRSA infections. Clinical cure was evaluated in the people who were suspected or proven to have MRSA infections (2659 participants), while microbiological cure rate was evaluated for the people who had a positive MRSA culture at baseline (1289 participants). The results showed that linezolid achieved both a significantly better clinical cure rate (RR 1.09, 95% CI 1.03 to 1.17; I2= 0%; Analysis 1.3), and microbiological cure rate (RR 1.17, 95%CI 1.04 to 1.32; I2= 46%; Analysis 2.3), than vancomycin’.  **Where reported:** Results text.  **Interaction exists based on reported results**: Not reported.  **How determined:** NA.  **Reported importance of the interaction:** Not reported.  **What reported:** Not reported.  **Where reported:** NA  **Reported plausibility of the interaction:** Not reported.  **What reported:** Not reported.  **Where reported:** NA  **Reported confounding possibility:** Not reported.  **What reported:** Not reported.  **Where reported:** NA.  **Mentioned covariate distribution:** Yes.  **What reported:** as above.  **Where reported:** Results text. |

^1^When the covariate was a second-level stratification in the analysis (e.g. for the covariate age, when the analyses where first stratified by comparison then by age within the comparison subgroups), to calculate the number of trials included in the interaction analysis, we extracted the number of trials in each first-level stratification (e.g. the number of trials for each comparison) and calculated the average number of trials across the first-level stratification (e.g. the average number of trials per comparison) to give the number of trials in the interaction analysis.

^2^Results were reported in the results text, forest plots, or tables.

^3^To determine whether an interaction exists based on reported results, we used the results from the test for subgroup differences (where reported).
