# Supplementary material for: Exploring Treatment by Covariate Interactions Using Subgroup Analysis and Meta-Regression in Cochrane Reviews: A Review of Recent Practice
Source: PLoS One. 2015 Jun 1;10(6):e0128804. doi: 10.1371/journal.pone.0128804 (PMC4452239; doi:10.1371/journal.pone.0128804)
Supplement: S4 Table — (DOCX) [file pone.0128804.s006.docx]

**Table S4: Number of trials and patients, type of covariates, and data type of covariates, in the review.**

| **Review** | **Number of trials^1^** | **Number of patients^1^** | **Number of covariates analysed /Number of covariates reported (%)** | **Number of patient covariates**  **/Number of covariates reported (%)** | **Number of intervention covariates**  **/Number of covariates reported (%)** | **Number of methodological covariates /Number of covariates reported (%)** | **Number of outcome-related covariates**  **/Number of covariates reported (%)** | **Number of other covariates /Number of covariates reported (%)** | **Number of categorical covariates /Number of covariates reported (%)** | **Number of continuous covariates /Number of covariates reported (%)** | **Number of covariates of unclear type**  **/Number of covariates reported (%)** | **Number of continuous covariates (unclear if categorised)/Number of covariates reported (%)** | **Number of continuous covariates (not categorised)/Number of covariates reported (%)** | **Number of continuous covariates (categorised)/Number of covariates reported (%)** | **Number of continuous covariates that were believed to be categorised because categories given/**  **Number of covariates reported (%)** | **Number of continuous covariates that were believed to be categorised because subgroup/sensitivity analyses planned or done/**  **Number of covariates reported (%)** |
| --- | --- | --- | --- | --- | --- | --- | --- | --- | --- | --- | --- | --- | --- | --- | --- | --- |
| Aboumarzouk 2012 | 1 | 324 | 0/6 (0) | 1/6 (17) | 4/6 (67) | 1/6 (17) | 0/6 (0) | 0/6 (0) | 4/6 (67) | 2/6 (33) | 0/6 (0) | 2/6 (33) | 0/6 (0) | 0/6 (0) | 0/6 (0) | 0/6 (0) |
| Almeida 2013 | 1 | 25 | 1/8 (13) | 3/8 (38) | 1/8 (13) | 4/8 (50) | 0/8 (0) | 0/8 (0) | 7/8 (88) | 1/8 (13) | 0/8 (0) | 0/8 (0) | 0/8 (0) | 1/8 (13) | 0/8 (0) | 1/8 (13) |
| Basurto Ona 2013 | 1 | 16 | 1/8 (13) | 2/8 (25) | 3/8 (38) | 3/8 (38) | 0/8 (0) | 0/8 (0) | 7/8 (88) | 1/8 (13) | 0/8 (0) | 0/8 (0) | 0/8 (0) | 1/8 (13) | 1/8 (13) | 0/8 (0) |
| Bellmunt-Montoya 2013 | 4 | 768 | 1/4 (25) | 0/4 (0) | 2/4 (50) | 2/4 (50) | 0/4 (0) | 0/4 (0) | 4/4 (100) | 0/4 (0) | 0/4 (0) | 0/4 (0) | 0/4 (0) | 0/4 (0) | 0/4 (0) | 0/4 (0) |
| Berlowitz 2013 | 1 | 14 | 0/6 (0) | 1/6 (17) | 2/6 (33) | 3/6 (50) | 0/6 (0) | 0/6 (0) | 4/6 (67) | 1/6 (17) | 1/6 (17) | 0/6 (0) | 0/6 (0) | 1/6 (17) | 0/6 (0) | 1/6 (17) |
| Boselie 2012 | 6 | 1,346 | 4**/**7 **(**57**)** | 2**/**7 (29) | 1**/**7 (14) | 3**/**7 (43) | 1**/**7 (14) | 0**/**7 (0) | 4**/**7 (57**)** | 3**/**7 (43**)** | 0**/**7 (0**)** | 0**/**7 (0) | 0**/**7 (0) | 3**/**7 (43) | 3**/**7 (43) | 0**/**7 (0) |
| Bruins Slot 2013 | 9 | 40,777 | 11/13 (85) | 6/13 (46) | 5/13 (38) | 2/13 (15) | 0/13 (0) | 0/13 (0) | 10/13 (77) | 3/13 (23) | 0/13 (0) | 0/13 (0) | 0/13 (0) | 3/13 (23) | 3/13 (23) | 0/13 (0) |
| Cavalheri, 2013 | 3 | 139 | 0**/**7 **(**0**)** | 0**/**7 (0) | 3**/**7 (43) | 4**/**7 (57) | 0**/**7 (0) | 0**/**7 (0) | 6**/**7 (86**)** | 1**/**7 (14**)** | 0**/**7 (0**)** | 0**/**7 (0) | 0**/**7 (0) | 1**/**7 (14) | 1**/**7 (14) | 0**/**7 (0) |
| Chaparro 2013 | 26 | 2,865 | 4**/**10 **(**40**)** | 1**/**10 (10) | 6**/**10 (60) | 1**/**10 (10) | 2**/**10 (20) | 0**/**10 (0) | 3**/**10 (30**)** | 5**/**10 (50**)** | 2**/**10 (20**)** | 0**/**10 (0) | 0**/**10 (0) | 5**/**10 (50) | 2**/**10 (20) | 3**/**10 (30) |
| Cheng 2013 | 5 | 268 | 1**/**10 **(**10**)** | 1**/**10 (10) | 4**/**10 (40) | 5**/**10 (50) | 0**/**10 (0) | 0**/**10 (0) | 8**/**10 (80**)** | 2**/**10 (20**)** | 0**/**10 (0**)** | 0**/**10 (0) | 0**/**10 (0) | 2**/**10 (20) | 2**/**10 (20) | 0**/**10 (0) |
| Cruciani 2013 | 8 | 1,587 | 4**/**7 **(**57**)** | 2**/**7 (29) | 3**/**7 (43) | 1**/**7 (14) | 1**/**7 (14) | 0**/**7 (0) | 5**/**7 (71**)** | 2**/**7 (29**)** | 0**/**7 (0**)** | 1**/**7 (14) | 0**/**7 (0) | 1**/**7 (14) | 0**/**7 (0) | 1**/**7 (14) |
| Dashash 2013 | 0 | 0 | 0/8 (0) | 3/8 (38) | 1/8 (13) | 4/8 (50) | 0/8 (0) | 0/8 (0) | 7/8 (88) | 1/8 (13) | 0/8 (0) | 0/8 (0) | 0/8 (0) | 1/8 (13) | 0/8 (0) | 1/8 (13) |
| Deare 2013 | 9 | 473 | 4/8 (50) | 0/8 (0) | 5/8 (63) | 2/8 (25) | 1/8 (13) | 0/8 (0) | 8/8 (100) | 0/8 (0) | 0/8 (0) | 0/8 (0) | 0/8 (0) | 0/8 (0) | 0/8 (0) | 0/8 (0) |
| Freak-Poli 2013 | 4 | 1,809 | 1**/**13 **(**8**)** | 5**/**13 (38) | 6**/**13 (46) | 1**/**13 (8) | 1**/**13 (8) | 0**/**13 (0) | 10**/**13 (77**)** | 3**/**13 (23**)** | 0**/**13 (0**)** | 0**/**13 (0) | 0**/**13 (0) | 3**/**13 (23) | 2**/**13 (15) | 1**/**13 (8) |
| Gan, 2013 | 0 | 0 | 0/8 (0) | 1/8 (13) | 3/8 (38) | 4/8 (50) | 0/8 (0) | 0/8 (0) | 5/8 (63) | 3/8 (38) | 0/8 (0) | 0/8 (0) | 0/8 (0) | 3/8 (38) | 0/8 (0) | 3/8 (38) |
| Gillies 2012 | 9 | 540 | 5**/**12 **(**42**)** | 4**/**12 (33) | 5**/**12 (42) | 2**/**12 (17) | 1**/**12 (8) | 0**/**12 (0) | 9**/**12 (75**)** | 3**/**12 (25**)** | 0**/**12 (0**)** | 0**/**12 (0) | 0**/**12 (0) | 3**/**12 (25) | 3**/**12 (25) | 0**/**12 (0) |
| Gois 2013 | 1 | 30 | 0/11 (0) | 5/11 (45) | 1/11 (9) | 5/11 (45) | 0/11 (0) | 0/11 (0) | 9/11 (82) | 2/11 (18) | 0/11 (0) | 0/11 (0) | 0/11 (0) | 2/11 (18) | 0/11 (0) | 2/11 (18) |
| Goldenberg 2013 | 23 | 4,213 | 4**/**6 **(**67**)** | 2**/**6 (33) | 3**/**6 (50) | 1**/**6 (17) | 0**/**6 (0) | 0**/**6 (0) | 4**/**6 (67**)** | 2**/**6 (33**)** | 0**/**6 (0**)** | 1**/**6 (17) | 0**/**6 (0) | 1**/**6 (17) | 1**/**6 (17) | 0**/**6 (0) |
| Gower 2013 | 4 | 100,876 | 2**/**10 **(**20**)** | 1**/**10 (10) | 4**/**10 (40) | 5**/**10 (50) | 0**/**10 (0) | 0**/**10 (0) | 7**/**10 (70**)** | 2**/**10 (20**)** | 1**/**10 (10**)** | 0**/**10 (0) | 0**/**10 (0) | 2**/**10 (20) | 0**/**10 (0) | 2**/**10 (20) |
| He 2013 | 1 | 1,106 | 0**/**8 **(**0**)** | 3**/**8 (38) | 2**/**8 (25) | 3**/**8 (38) | 0**/**8 (0) | 0**/**8 (0) | 4**/**8 (50**)** | 4**/**8 (50**)** | 0**/**8 (0**)** | 0**/**8 (0) | 0**/**8 (0) | 4**/**8 (50) | 4**/**8 (50) | 0**/**8 (0) |
| Itchaki 2013 | 5 | 949 | 4/12 (33) | 4/12 (33) | 4/12 (33) | 4/12 (33) | 0/12 (0) | 0/12 (0) | 11/12 (92) | 1/12 (8) | 0/12 (0) | 0/12 (0) | 0/12 (0) | 1/12 (8) | 0/12 (0) | 1/12 (8) |
| Kinnersley 2013 | 1 | 97 | 0/12 (0) | 1/12 (8) | 5/12 (42) | 5/12 (42) | 1/12 (8) | 0/12 (0) | 11/12 (92) | 1/12 (8) | 0/12 (0) | 0/12 (0) | 0/12 (0) | 1/12 (8) | 1/12 (8) | 0/12 (0) |
| Lawrie 2013 | 13 | 4,625 | 1**/**3 **(**33**)** | 1**/**3 (33) | 1**/**3 (33) | 1**/**3 (33) | 0**/**3 (0) | 0**/**3 (0) | 3**/**3 (100**)** | 0**/**3 (0**)** | 0**/**3 (0**)** | 0**/**3 (0) | 0**/**3 (0) | 0**/**3 (0) | 0**/**3 (0) | 0**/**3 (0) |
| Lee 2013 | 1 | 20 | 0**/**6 **(**0**)** | 1**/**6 (17) | 1**/**6 (17) | 4**/**6 (67) | 0**/**6 (0) | 0**/**6 (0) | 5**/**6 (83**)** | 1**/**6 (17**)** | 0**/**6 (0**)** | 0**/**6 (0) | 0**/**6 (0) | 1**/**6 (17) | 1**/**6 (17) | 0**/**6 (0) |
| Li 2013 | 4 | 758 | 0/6 (0) | 4/6 (67) | 0/6 (0) | 2/6 (33) | 0/6 (0) | 0/6 (0) | 3/6 (50) | 3/6 (50) | 0/6 (0) | 0/6 (0) | 0/6 (0) | 3/6 (50) | 0/6 (0) | 3/6 (50) |
| Liu 2013 | 0 | 0 | 0**/**11 **(**0**)** | 2**/**11 (18) | 1**/**11 (9) | 6**/**11 (55) | 2**/**11 (18) | 0**/**11 (0) | 8**/**11 (73**)** | 3**/**11 (27**)** | 0**/**11 (0**)** | 0**/**11 (0) | 0**/**11 (0) | 3**/**11 (27) | 3**/**11 (27) | 0**/**11 (0) |
| Lopez 2013 | 7 | 10,311 | 2/2 (100) | 0/2 (0) | 1/2 (50) | 0/2 (0) | 1/2 (50) | 0/2 (0) | 1/2 (50) | 1/2 (50) | 0/2 (0) | 0/2 (0) | 0/2 (0) | 1/2 (50) | 1/2 (50) | 0/2 (0) |
| Marigold 2013 | 0 | 0 | 0/8 (0) | 3/8 (38) | 0/8 (0) | 3/8 (38) | 0/8 (0) | 2/8 (25) | 4/8 (50) | 4/8 (50) | 0/8 (0) | 0/8 (0) | 0/8 (0) | 4/8 (50) | 4/8 (50) | 0/8 (0) |
| Mocellin 2013 | 17 | 10,345 | 6**/**14 **(**43**)** | 3**/**14 (21) | 5**/**14 (36) | 3**/**14 (21) | 2**/**14 (14) | 1**/**14 (7) | 9**/**14 (64**)** | 5**/**14 (36**)** | 0**/**14 (0**)** | 1**/**14 (7) | 2**/**14 (14) | 2**/**14 (14) | 2**/**14 (14) | 0**/**14 (0) |
| Mutua 2012 | 3 | 173 | 2/8 (25) | 4/8 (50) | 3/8 (38) | 1/8 (13) | 0/8 (0) | 0/8 (0) | 8/8 (100) | 0/8 (0) | 0/8 (0) | 0/8 (0) | 0/8 (0) | 0/8 (0) | 0/8 (0) | 0/8 (0) |
| Parker 2013 | 0 | 0 | 0/11 (0) | 2/11 (18) | 3/11 (27) | 4/11 (36) | 2/11 (18) | 0/11 (0) | 5/11 (45) | 6/11 (55) | 0/11 (0) | 0/11 (0) | 0/11 (0) | 6/11 (55) | 3/11 (27) | 3/11 (27) |
| Pega, 2013 | 1 | 127,209 | 0**/**6 **(**0**)** | 4**/**6 (67) | 0**/**6 (0) | 2**/**6 (33) | 0**/**6 (0) | 0**/**6 (0) | 5**/**6 (83**)** | 1**/**6 (17**)** | 0**/**6 (0**)** | 0**/**6 (0) | 0**/**6 (0) | 1**/**6 (17) | 0**/**6 (0) | 1**/**6 (17) |
| Penninga 2013 | 3 | 413 | 0/5 (0) | 2/5 (40) | 2/5 (40) | 1/5 (20) | 0/5 (0) | 0/5 (0) | 4/5 (80) | 1/5 (20) | 0/5 (0) | 0/5 (0) | 0/5 (0) | 1/5 (20) | 1/5 (20) | 0/5 (0) |
| Peters 2013 | 6 | 394 | 2/11 (18) | 2/11 (18) | 3/11 (27) | 5/11 (45) | 1/11 (9) | 0/11 (0) | 9/11 (82) | 2/11 (18) | 0/11 (0) | 0/11 (0) | 0/11 (0) | 2/11 (18) | 2/11 (18) | 0/11 (0) |
| Rockers 2013 | 1 | Not reported | 1**/**2 **(**50**)** | 1**/**2 (50) | 1**/**2 (50) | 0**/**2 (0) | 0**/**2 (0) | 0**/**2 (0) | 2**/**2 (100**)** | 0**/**2 (0**)** | 0**/**2 (0**)** | 0**/**2 (0) | 0**/**2 (0) | 0**/**2 (0) | 0**/**2 (0) | 0**/**2 (0) |
| Sajid, 2012 | 18 | 1,252 | 1**/**4 **(**25**)** | 1**/**4 (25) | 0**/**4 (0) | 3**/**4 (75) | 0**/**4 (0) | 0**/**4 (0) | 3**/**4 (75**)** | 1**/**4 (25**)** | 0**/**4 (0**)** | 0**/**4 (0) | 0**/**4 (0) | 1**/**4 (25) | 1**/**4 (25) | 0**/**4 (0) |
| Sampson 2013 | 12 | 1,327 | 3**/**4 **(**75**)** | 0**/**4 (0) | 1**/**4 (25) | 2**/**4 (50) | 1**/**4 (25) | 0**/**4 (0) | 3**/**4 (75**)** | 1**/**4 (25**)** | 0**/**4 (0**)** | 0**/**4 (0) | 0**/**4 (0) | 1**/**4 (25) | 1**/**4 (25) | 0**/**4 (0) |
| Sanders 2013 | 4 | 675 | 2/11 (18) | 0/11 (0) | 5/11 (45) | 4/11 (36) | 1/11 (9) | 1/11 (9) | 8/11 (73) | 3/11 (27) | 0/11 (0) | 2/11 (18) | 0/11 (0) | 1/11 (9) | 0/11 (0) | 1/11 (9) |
| Sarai 2013 | 1 | 100 | 0**/**10 **(**0**)** | 4**/**10 (40) | 3**/**10 (30) | 3**/**10 (30) | 0**/**10 (0) | 0**/**10 (0) | 7**/**10 (70**)** | 3**/**10 (30**)** | 0**/**10 (0**)** | 0**/**10 (0) | 0**/**10 (0) | 3**/**10 (30) | 1**/**10 (10) | 2**/**10 (20) |
| Schoot 2013 | 3 | 132 | 2/3 (67) | 1/3 (33) | 0/3 (0) | 2/3 (67) | 0/3 (0) | 0/3 (0) | 3/3 (100) | 0/3 (0) | 0/3 (0) | 0/3 (0) | 0/3 (0) | 0/3 (0) | 0/3 (0) | 0/3 (0) |
| Semple 2013 | 5 | 297 | 1/6 (17) | 1/6 (17) | 3/6 (50) | 1/6 (17) | 1/6 (17) | 0/6 (0) | 5/6 (83) | 1/6 (17) | 0/6 (0) | 0/6 (0) | 0/6 (0) | 1/6 (17) | 1/6 (17) | 0/6 (0) |
| Sharma 2013 | 6 | 8,904 | 3/7 (43) | 2/7 (29) | 2/7 (29) | 2/7 (29) | 1/7 (14) | 0/7 (0) | 4/7 (57) | 3/7 (43) | 0/7 (0) | 0/7 (0) | 0/7 (0) | 3/7 (43) | 2/7 (29) | 1/7 (14) |
| Showell 2013 | 4 | 293 | 4/12 (33) | 4/12 (33) | 5/12 (42) | 3/12 (25) | 0/12 (0) | 0/12 (0) | 11/12 (92) | 0/12 (0) | 1/12 (8) | 0/12 (0) | 0/12 (0) | 0/12 (0) | 0/12 (0) | 0/12 (0) |
| Stead 2012 | 38 | 15,506 | 7**/**11 **(**64**)** | 1**/**11 (9) | 5**/**11 (45) | 3**/**11 (27) | 2**/**11 (18) | 0**/**11 (0) | 7**/**11 (64**)** | 3**/**11 (27**)** | 1**/**11 (9**)** | 0**/**11 (0) | 0**/**11 (0) | 3**/**11 (27) | 1**/**11 (9) | 2**/**11 (18) |
| Trivedi 2013 | 0 | 0 | 0**/**6 **(**0**)** | 5**/**6 (83) | 1**/**6 (17) | 0**/**6 (0) | 0**/**6 (0) | 0**/**6 (0) | 2**/**6 (33**)** | 4**/**6 (67**)** | 0**/**6 (0**)** | 0**/**6 (0) | 0**/**6 (0) | 4**/**6 (67) | 4**/**6 (67) | 0**/**6 (0) |
| Trotti 2012 | 5 | 164 | 3/5 (60) | 2/5 (40) | 1/5 (20) | 2/5 (40) | 0/5 (0) | 0/5 (0) | 5/5 (100) | 0/5 (0) | 0/5 (0) | 0/5 (0) | 0/5 (0) | 0/5 (0) | 0/5 (0) | 0/5 (0) |
| Van Teeffelen, 2013 | 0 | 0 | 0**/**5 **(**0**)** | 2**/**5 (40) | 0**/**5 (0) | 3**/**5 (60) | 0**/**5 (0) | 0**/**5 (0) | 4**/**5 (80**)** | 1**/**5 (20**)** | 0**/**5 (0**)** | 0**/**5 (0) | 0**/**5 (0) | 1**/**5 (20) | 1**/**5 (20) | 0**/**5 (0) |
| van Zuuren 2013 | 1 | 253 | 1**/**8 **(**13**)** | 1**/**8 (13) | 2**/**8 (25) | 4**/**8 (50) | 1**/**8 (13) | 0**/**8 (0) | 7**/**8 (88**)** | 1**/**8 (13**)** | 0**/**8 (0**)** | 0**/**8 (0) | 0**/**8 (0) | 1**/**8 (13) | 0**/**8 (0) | 1**/**8 (13) |
| Wakai 2013 | 2 | 558 | 1**/**4 **(**25**)** | 1**/**4 (25) | 0**/**4 (0) | 2**/**4 (50) | 1**/**4 (25) | 0**/**4 (0) | 4**/**4 (100**)** | 0**/**4 (0**)** | 0**/**4 (0**)** | 0**/**4 (0) | 0**/**4 (0) | 0**/**4 (0) | 0**/**4 (0) | 0**/**4 (0) |
| Wang 2013 | 1 | 60 | 1**/**6 **(**17**)** | 2**/**6 (33) | 1**/**6 (17) | 2**/**6 (33) | 0**/**6 (0) | 1**/**6 (17) | 5**/**6 (83**)** | 0**/**6 (0**)** | 1**/**6 (17**)** | 0**/**6 (0) | 0**/**6 (0) | 0**/**6 (0) | 0**/**6 (0) | 0**/**6 (0) |
| Yue 2013 | 9 | 3,114 | 4**/**6 **(**67**)** | 3**/**6 (50) | 1**/**6 (17) | 2**/**6 (33) | 0**/**6 (0) | 0**/**6 (0) | 5**/**6 (83**)** | 1**/**6 (17**)** | 0**/**6 (0**)** | 0**/**6 (0) | 0**/**6 (0) | 1**/**6 (17) | 1**/**6 (17) | 0**/**6 (0) |
| Ziebell 2013 | 0 | 0 | 0/14 (0) | 2/14 (14) | 2/14 (14) | 10/14 (71) | 0/14 (0) | 0/14 (0) | 11/14 (79) | 3/14 (21) | 0/14 (0) | 1/14 (7) | 0/14 (0) | 2/14 (14) | 2/14 (14) | 0/14 (0) |
| Summed totals | - | - | 94/409 (23) | 109/409 (27) | 126/409 (31) | 145/409 (35) | 24/409 (6) | 5/409 (1) | 307/409 (75) | 95/409 (23) | 7/409 (2) | 8/409 (2) | 2/409 (0) | 85/409 (21) | 55/409 (13) | 30/409 (7) |
| Number of reviews with > 1 covariate in numerator | - | - | 33/52 (63) | 46/52 (88) | 45/52 (87) | 49/52 (94) | 19/52 (37) | 4/52 (8) | 52/52 (100) | 42/52 (81) | 6/52 (12) | 6/52 (12) | 1/52 (2) | 41/52 (79) | 29/52 (56) | 18/52 (35) |
| Median | 4 | 394 | 18 | 29 | 33 | 36 | 0 | 0 | 80 | 20 | 0 | 0 | 0 | 17 | 12 | 0 |
| IQR | 1-6 | 45-1,467 | 0-43 | 14-38 | 16-42 | 21-50 | 0-13 | 0-0 | 67-88 | 13-31 | 0-0 | 0-0 | 0-0 | 9-26 | 0-20 | 0-13 |
| Range | 0-38 | 0-127,209 | 0-100 | 0-83 | 0-67 | 0-75 | 0-50 | 0-25 | 30-100 | 0-67 | 0-20 | 0-33 | 0-14 | 0-67 | 0-67 | 0-50 |

IQR: inter-quartile range.

^1^When the outcome was described as one outcome in the methods but reported as multiple outcomes in the results, the number of trials and patients for the first outcome listed in the results was used here.
