# Supplementary material for: Exploring Treatment by Covariate Interactions Using Subgroup Analysis and Meta-Regression in Cochrane Reviews: A Review of Recent Practice
Source: PLoS One. 2015 Jun 1;10(6):e0128804. doi: 10.1371/journal.pone.0128804 (PMC4452239; doi:10.1371/journal.pone.0128804)
Supplement: S5 Table — (DOCX) [file pone.0128804.s007.docx]

**Table S5: Results of assessment of interactions for each review.**

| **Review** | **Choosing covariates** | | | | | **Considering covariate data** | | | | | | | **Analysing interactions** | **Detecting interactions** | **Reporting interaction results** | | **Interpreting interaction results** | | | | |
| --- | --- | --- | --- | --- | --- | --- | --- | --- | --- | --- | --- | --- | --- | --- | --- | --- | --- | --- | --- | --- | --- |
|  | 1. Was external evidence (e.g. other reviews or studies) reported to be used to choose each covariate? | 1. Was rationale given for choosing each covariate as a potential treatment effect modifying covariate? | 1. Was each covariate reported a priori (i.e. in the protocol)? | 1. Was each post-hoc chosen covariate (i.e. in the review but not in the protocol) labelled as such? | 1. Were a limited number of covariates (i.e. <6) reported? | 1. Was missing covariate data reported to be sought and planned to be sought? | 1. For study-level covariates, were AD analyses reported to be planned and, if interaction analyses were applied, actually carried out? | 1. If AD analyses were reported to be performed, was there reported to be at least 10 trials in the analysis for each covariate (for the outcome)? | 1. For patient-level covariates, were IPD analyses reported to be planned and, if interaction analyses were applied, actually carried out? | 1. Was justification given for categorising each continuous covariate that was categorised? | 1. Were the categories reported for each categorised covariate? | 1. Was justification given for the categories chosen for each categorised covariate? | 1. Was interaction analysis reported to be planned? | 1. Were methods to detect interactions reported to be planned and, if interaction analyses were applied, actually used for each covariate? | 1. Were results from interaction analysis reported for each covariate (for the outcome)? | 1. Were results from methods to detect interactions reported for each covariate (for the outcome)? | 1. Was it reported whether or not an interaction was detected for each analysed covariate (for the outcome)? | 1. Was the importance of the interaction or lack of interaction discussed for each analysed covariate (for the outcome)? | 1. Was the plausibility of the interaction or lack of interaction discussed for each analysed covariate (for the outcome)? | 1. Was the possibility of confounding discussed for each analysed covariate (for the outcome)? | 1. Was the covariate distribution discussed for each analysed covariate (for the outcome)? |
| Aboumarzouk 2012 | N | N | Y | NA | N | N | Y | NA | N | NA | N | N | Y | N | NA | NA | NA | NA | NA | NA | NA |
| Almeida 2013 | N | N | Y | NA | N | N | Y | N | N | N | N | N | Y | N | Y | N | N | N | N | N | Y |
| Basurto Ona 2013 | N | N | N | N | N | N | Y | N | N | N | N | N | Y | N | Y | N | N | N | N | N | Y |
| Bellmunt-Montoya 2013 | N | N | Y | NA | Y | N | Y | N | NA | NA | Y | N | Y | N | Y | N | N | N | N | N | Y |
| Berlowitz 2013 | N | N | Y | NA | N | N | Y | NA | N | N | N | N | Y | N | NA | NA | NA | NA | NA | NA | NA |
| Boselie 2012 | N | N | N | N | N | N | Y | N | N | N | N | N | Y | N | Y | N | N | N | N | N | N |
| Bruins Slot 2013 | N | N | N | N | N | N | Y | N | N | N | N | N | Y | N | Y | Y | N | N | N | N | N |
| Cavalheri, 2013 | N | N | Y | NA | N | N | Y | NA | NA | N | N | N | Y | N | NA | NA | NA | NA | NA | NA | NA |
| Chaparro 2013 | N | N | N | N | N | N | Y | N | N | N | N | N | Y | N | Y | N | N | N | N | N | N |
| Cheng 2013 | N | N | N | N | N | N | Y | N | N | N | N | N | Y | N | Y | N | N | N | N | N | N |
| Cruciani 2013 | N | N | N | N | N | N | Y | N | N | N | N | N | Y | N | N | N | N | N | N | N | N |
| Dashash 2013 | N | N | Y | NA | N | N | Y | NA | N | N | N | N | Y | N | NA | NA | NA | NA | NA | NA | NA |
| Deare 2013 | N | N | N | N | N | N | Y | N | NA | NA | N | N | Y | N | Y | N | N | N | N | N | N |
| Freak-Poli 2013 | N | N | Y | NA | N | N | Y | N | N | N | N | N | Y | N | Y | N | N | N | N | N | Y |
| Gan, 2013 | N | N | N | N | N | N | Y | NA | N | N | N | N | Y | N | NA | NA | NA | NA | NA | NA | NA |
| Gillies 2012 | N | N | N | N | N | N | Y | N | N | N | N | N | Y | N | Y | N | N | N | N | N | N |
| Gois 2013 | N | N | Y | NA | N | N | Y | NA | N | N | N | N | Y | N | NA | NA | NA | NA | NA | NA | NA |
| Goldenberg 2013 | N | N | N | N | N | N | Y | Y | N | N | N | N | Y | N | Y | Y | N | N | N | N | N |
| Gower 2013 | N | N | N | N | N | N | Y | N | N | N | N | N | Y | N | Y | N | N | N | N | N | N |
| He 2013 | N | N | Y | NA | N | N | Y | NA | N | N | Y | N | Y | N | NA | NA | NA | NA | NA | NA | NA |
| Itchaki 2013 | N | N | N | N | N | N | Y | N | N | N | N | N | Y | N | Y | N | N | N | N | N | N |
| Kinnersley 2013 | N | N | N | N | N | N | Y | NA | N | N | N | N | Y | N | NA | NA | NA | NA | NA | NA | NA |
| Lawrie 2013 | N | N | Y | NA | Y | N | Y | Y | N | NA | Y | N | Y | N | Y | Y | N | N | N | N | Y |
| Lee 2013 | N | N | N | N | N | N | Y | NA | N | N | N | N | Y | N | NA | NA | NA | NA | NA | NA | NA |
| Li 2013 | N | N | Y | NA | N | N | Y | NA | N | N | N | N | Y | N | NA | NA | NA | NA | NA | NA | NA |
| Liu 2013 | N | N | Y | NA | N | N | Y | NA | N | N | N | N | Y | N | NA | NA | NA | NA | NA | NA | NA |
| Lopez 2013 | N | N | N | N | Y | N | N | N | NA | N | N | N | N | N | Y | N | N | N | N | N | N |
| Marigold 2013 | N | N | Y | NA | N | N | Y | NA | N | N | N | N | Y | N | NA | NA | NA | NA | NA | NA | NA |
| Mocellin 2013 | N | N | N | N | N | N | Y | Y | N | N | N | N | Y | N | Y | N | Y | N | N | N | N |
| Mutua 2012 | N | N | N | N | N | N | Y | N | N | NA | N | N | Y | N | Y | N | N | N | N | N | Y |
| Parker 2013 | N | N | Y | NA | N | N | Y | NA | N | N | N | N | Y | N | NA | NA | NA | NA | NA | NA | NA |
| Pega, 2013 | N | N | Y | NA | N | N | Y | NA | N | N | N | N | Y | N | NA | NA | NA | NA | NA | NA | NA |
| Penninga 2013 | N | N | Y | NA | Y | N | Y | NA | N | N | Y | N | Y | Y | NA | NA | NA | NA | NA | NA | NA |
| Peters 2013 | N | N | N | N | N | N | Y | N | N | N | N | N | Y | N | Y | N | N | N | N | N | Y |
| Rockers 2013 | N | N | N | N | Y | N | Y | N | N | NA | N | N | Y | N | Y | N | N | N | N | N | N |
| Sajid, 2012 | N | N | Y | NA | Y | N | Y | Y | N | N | N | N | Y | N | Y | N | N | N | N | N | Y |
| Sampson 2013 | N | N | N | N | Y | N | Y | N | NA | N | N | N | Y | N | Y | N | N | N | N | N | N |
| Sanders 2013 | N | N | N | N | N | N | Y | N | NA | N | N | N | Y | N | Y | N | N | N | N | N | N |
| Sarai 2013 | N | N | Y | NA | N | N | Y | NA | N | N | N | N | Y | N | NA | NA | NA | NA | NA | NA | NA |
| Schoot 2013 | N | N | N | N | Y | N | Y | N | N | NA | N | N | Y | N | N | N | N | N | N | N | N |
| Semple 2013 | N | N | Y | NA | N | N | Y | N | N | N | Y | N | Y | N | Y | N | N | N | N | N | N |
| Sharma 2013 | N | N | N | N | N | N | Y | N | N | N | N | N | Y | N | Y | N | N | N | N | N | N |
| Showell 2013 | N | N | N | N | N | N | Y | N | N | NA | N | N | Y | N | Y | N | N | N | N | N | Y |
| Stead 2012 | N | N | N | N | N | N | Y | Y | N | N | N | N | Y | N | Y | Y | N | N | N | N | N |
| Trivedi 2013 | N | N | N | N | N | N | Y | NA | N | N | N | N | Y | N | NA | NA | NA | NA | NA | NA | NA |
| Trotti 2012 | N | N | N | N | Y | N | Y | N | N | NA | N | N | Y | N | N | N | N | N | N | N | N |
| Van Teeffelen, 2013 | N | N | Y | NA | Y | N | Y | NA | N | N | Y | N | Y | N | NA | NA | NA | NA | NA | NA | NA |
| van Zuuren 2013 | N | N | N | N | N | N | Y | N | N | N | N | N | Y | N | Y | N | N | N | N | N | Y |
| Wakai 2013 | N | N | N | N | Y | N | N | N | N | NA | N | N | Y | N | Y | N | N | N | N | N | Y |
| Wang 2013 | N | N | Y | NA | N | N | Y | N | N | NA | N | N | Y | N | Y | N | N | N | N | N | Y |
| Yue 2013 | N | N | N | N | N | N | Y | N | N | N | N | N | Y | N | Y | N | N | N | N | N | Y |
| Ziebell 2013 | N | N | Y | NA | N | N | Y | NA | N | N | N | N | Y | N | NA | NA | NA | NA | NA | NA | NA |

Y: Yes; N: no; NA: not applicable.
