# Supplementary material for: Exploring Treatment by Covariate Interactions Using Subgroup Analysis and Meta-Regression in Cochrane Reviews: A Review of Recent Practice
Source: PLoS One. 2015 Jun 1;10(6):e0128804. doi: 10.1371/journal.pone.0128804 (PMC4452239; doi:10.1371/journal.pone.0128804)
Supplement: S6 Table — (DOCX) [file pone.0128804.s008.docx]

**Table S6: Choosing covariates: covariates reported with rationale, a priori, and labelled as post-hoc; and number of reported and analysed covariates.**

| **Review** | **Number of covariates with rationale given**  **/Number of covariates reported (%)** | **Number of covariates chosen a priori**  **/Number of covariates reported (%)** | **Number of covariates in protocol and review**  **/Number of covariates reported (%)** | **Number of covariates in protocol only**  **/Number of covariates reported (%)** | **Number of post-hoc covariates labelled as such/**  **Number of post-hoc covariates (%)** | **Number of covariates reported** | **Number of covariates analysed** |
| --- | --- | --- | --- | --- | --- | --- | --- |
| Aboumarzouk 2012 | 0/6 (0) | 6/6 (100) | 0/6 (0) | 6/6 (100) | 0/0 (-) | 6 | 0 |
| Almeida 2013 | 0/8 (0) | 8/8 (100) | 8/8 (100) | 0/8 (0) | 0/0 (-) | 8 | 1 |
| Basurto Ona 2013 | 0/8 (0) | 7/8 (88) | 7/8 (88) | 0/8 (0) | 0/1 (0) | 8 | 1 |
| Bellmunt-Montoya 2013 | 0/4 (0) | 4/4 (100) | 4/4 (100) | 0/4 (0) | 0/0 (-) | 4 | 1 |
| Berlowitz 2013 | 0/6 (0) | 6/6 (100) | 6/6 (100) | 0/6 (0) | 0/0 (-) | 6 | 0 |
| Boselie 2012 | 2**/**7 (29**)** | 5**/**7 **(**71**)** | 5**/**7 **(**71**)** | 0**/**7 (0) | 1/2 (50) | 7 | 4 |
| Bruins Slot 2013 | 0/13 (0) | 12/13 (92) | 12/13 (92) | 0/13 (0) | 0/1 (0) | 13 | 11 |
| Cavalheri, 2013 | 0**/**7 **(**0**)** | 7**/**7 **(**100**)** | 0**/**7 **(**0**)** | 7**/**7 (100) | 0/0 (-) | 7 | 0 |
| Chaparro 2013 | 0**/**10 **(**0**)** | 8**/**10 **(**80**)** | 8**/**10 **(**80**)** | 0**/**10 (0) | 0/2 (0) | 10 | 4 |
| Cheng 2013 | 0**/**10 **(**0**)** | 8**/**10 **(**80**)** | 7**/**10 **(**70**)** | 1**/**10 (10) | 0/2 (0) | 10 | 1 |
| Cruciani 2013 | 0**/**7 **(**0**)** | 6**/**7 **(**86**)** | 4**/**7 **(**57**)** | 2**/**7 (29) | 0/1 (0) | 7 | 4 |
| Dashash 2013 | 0/8 (0) | 8/8 (100) | 8/8 (100) | 0/8 (0) | 0/0 (-) | 8 | 0 |
| Deare 2013 | 0/8 (0) | 3/8 (38) | 3/8 (38) | 0/8 (0) | 0/5 (0) | 8 | 4 |
| Freak-Poli 2013 | 1**/**13 **(**8**)** | 13**/**13 **(**100**)** | 13**/**13 **(**100**)** | 0**/**13 (0) | 0/0 (-) | 13 | 1 |
| Gan, 2013 | 0/8 (0) | 7/8 (88) | 7/8 (88) | 0/8 (0) | 0/1 (0) | 8 | 0 |
| Gillies 2012 | 2**/**12 **(**17**)** | 10**/**12 **(**83**)** | 6**/**12 **(**50**)** | 4**/**12 (33) | 1/2 (50) | 12 | 5 |
| Gois 2013 | 0/11 (0) | 11/11 (100) | 0/11 (0) | 11/11 (100) | 0/0 (-) | 11 | 0 |
| Goldenberg 2013 | 5**/**6 **(**83**)** | 2**/**6 **(**33**)** | 1**/**6 **(**17**)** | 1**/**6 (17) | 1/4 (25) | 6 | 4 |
| Gower 2013 | 0**/**10 **(**0**)** | 9**/**10 **(**90**)** | 1**/**10 **(**10**)** | 8**/**10 (80) | 0/1 (0) | 10 | 2 |
| He 2013 | 0**/**8 **(**0**)** | 8**/**8 **(**100**)** | 8**/**8 **(**100**)** | 0**/**8 (0) | 0/0 (-) | 8 | 0 |
| Itchaki 2013 | 2/12 (17) | 10/12 (83) | 8/12 (67) | 2/12 (17) | 0/2 (0) | 12 | 4 |
| Kinnersley 2013 | 5/12 (42) | 10/12 (83) | 8/12 (67) | 2/12 (17) | 0/2 (0) | 12 | 0 |
| Lawrie 2013 | 0**/**3 **(**0**)** | 3**/**3 **(**100**)** | 3**/**3 **(**100**)** | 0**/**3 (0) | 0/0 (-) | 3 | 1 |
| Lee 2013 | 1**/**6 (17**)** | 5**/**6 **(**83**)** | 5**/**6 **(**83**)** | 0**/**6 (0) | 0/1 (0) | 6 | 0 |
| Li 2013 | 0/6 (0) | 6/6 (100) | 6/6 (100) | 0/6 (0) | 0/0 (-) | 6 | 0 |
| Liu 2013 | 0**/**11 **(**0**)** | 11**/**11 **(**100**)** | 11**/**11 **(**100**)** | 0**/**11 (0) | 0/0 (-) | 11 | 0 |
| Lopez 2013 | 0/2 (0) | 0/2 (0) | 0/2 (0) | 0/2 (0) | 0/2 (0) | 2 | 2 |
| Marigold 2013 | 0/8 (0) | 8/8 (100) | 7/8 (88) | 1/8 (13) | 0/0 (-) | 8 | 0 |
| Mocellin 2013 | 0**/**14 **(**0**)** | 12**/**14 **(**86**)** | 6**/**14 **(**43**)** | 6**/**14 (43) | 0/2 (0) | 14 | 6 |
| Mutua 2012 | 0/8 (0) | 5/8 (63) | 4/8 (50) | 1/8 (13) | 0/3 (0) | 8 | 2 |
| Parker 2013 | 1/11 (9) | 11/11 (100) | 11/11 (100) | 0/11 (0) | 0/0 (-) | 11 | 0 |
| Pega, 2013 | 4**/**6 **(**67**)** | 6**/**6 **(**100**)** | 4**/**6 **(**67**)** | 2**/**6 (33) | 0/0 (-) | 6 | 0 |
| Penninga 2013 | 4/5 (80) | 5/5 (100) | 5/5 (100) | 0/5 (0) | 0/0 (-) | 5 | 0 |
| Peters 2013 | 3/11 (27) | 9/11 (82) | 5/11 (45) | 4/11 (36) | 0/2 (0) | 11 | 2 |
| Rockers 2013 | 0**/**2 **(**0**)** | 1**/**2 **(**50**)** | 0**/**2 **(**0**)** | 1**/**2 (50) | 0/1 (0) | 2 | 1 |
| Sajid, 2012 | 0**/**4 **(**0**)** | 4**/**4 **(**100**)** | 3**/**4 **(**75**)** | 1**/**4 (25) | 0/0 (-) | 4 | 1 |
| Sampson 2013 | 0**/**4 **(**0**)** | 3**/**4 **(**75**)** | 3**/**4 **(**75**)** | 0**/**4 (0) | 0/1 (0) | 4 | 3 |
| Sanders 2013 | 0/11 (0) | 9/11 (82) | 9/11 (82) | 0/11 (0) | 0/2 (0) | 11 | 2 |
| Sarai 2013 | 0**/**10 **(**0**)** | 10**/**10 **(**100**)** | 10**/**10 **(**100**)** | 0**/**10 (0) | 0/0 (-) | 10 | 0 |
| Schoot 2013 | 0/3 (0) | 2/3 (67) | 2/3 (67) | 0/3 (0) | 0/1 (0) | 3 | 2 |
| Semple 2013 | 0/6 (0) | 6/6 (100) | 6/6 (100) | 0/6 (0) | 0/0 (-) | 6 | 1 |
| Sharma 2013 | 0/7 (0) | 5/7 (71) | 4/7 (57) | 1/7 (14) | 0/2 (0) | 7 | 3 |
| Showell 2013 | 1/12 (8) | 5/12 (42) | 0/12 (0) | 5/12 (42) | 0/7 (0) | 12 | 4 |
| Stead 2012 | 1**/**11 **(**9**)** | 6**/**11 **(**55**)** | 6**/**11 **(**55**)** | 0**/**11 (0) | 2/5 (40) | 11 | 7 |
| Trivedi 2013 | 0**/**6 **(**0**)** | 5**/**6 **(**83**)** | 5**/**6 **(**83**)** | 0**/**6 (0) | 0/1 (0) | 6 | 0 |
| Trotti 2012 | 0/5 (0) | 4/5 (80) | 4/5 (80) | 0/5 (0) | 0/1 (0) | 5 | 3 |
| Van Teeffelen 2013 | 2**/**5 **(**40**)** | 5**/**5 **(**100**)** | 5**/**5 **(**100**)** | 0**/**5 (0) | 0/0 (-) | 5 | 0 |
| van Zuuren 2013 | 0**/**8 **(**0**)** | 7**/**8 **(**88**)** | 7**/**8 **(**88**)** | 0**/**8 (0) | 0/1 (0) | 8 | 1 |
| Wakai 2013 | 1**/**4 **(**25**)** | 3**/**4 **(**75**)** | 3**/**4 **(**75**)** | 0**/**4 (0) | 0/1 (0) | 4 | 1 |
| Wang 2013 | 0**/**6 **(**0**)** | 6**/**6 **(**100**)** | 1**/**6 **(**17**)** | 5**/**6 (83) | 0/0 (-) | 6 | 1 |
| Yue 2013 | 1**/**6 **(**17**)** | 5**/**6 **(**83**)** | 5**/**6 **(**83**)** | 0**/**6 (0) | 0/1 (0) | 6 | 4 |
| Ziebell 2013 | 0/14 (0) | 14/14 (100) | 0/14 (0) | 14/14 (100) | 0/0 (-) | 14 | 0 |
| Summed totals | 36/409 (9) | 349/409 (85) | 264/409 (65) | 85/409 (21) | 5/60 (8) | - | - |
| Number of reviews with > 1 covariate in numerator | 16/52 (31) | 51/52 (98) | 45/52 (87) | 21/52 (40) | 4/30 (13) | - | - |
| Median | 0 | 88 | 75 | 0 | 0 | 8 | 1 |
| IQR | 0-9 | 80-100 | 49-100 | 0-26 | 0-0 | 6-11 | 0-3 |
| Range | 0-83 | 0-100 | 0-100 | 0-100 | 0-50 | 2-14 | 0-11 |

IQR: inter-quartile range.
