# Supplementary material for: Exploring Treatment by Covariate Interactions Using Subgroup Analysis and Meta-Regression in Cochrane Reviews: A Review of Recent Practice
Source: PLoS One. 2015 Jun 1;10(6):e0128804. doi: 10.1371/journal.pone.0128804 (PMC4452239; doi:10.1371/journal.pone.0128804)
Supplement: S7 Table — (DOCX) [file pone.0128804.s009.docx]

**Table S7: Considering covariate data: number of covariates with reported categories and justification for categories, and number of trials in analyses.**

| **Review** | **Numbers of trials in the analysis for each covariate** | **Number of analyses with at least 10 trials**  **/Number of analyses (%)** | **Median number of trials in analyses** | **Number of covariates with categories reported /Number of categorised covariate (%)** | **Number of covariates with categories reported in protocol and review**  **/Number of categorised covariate (%)** | **Number of covariates with categories reported in protocol only**  **/Number of categorised covariate (%)** | **Number of covariates with categories reported in review only**  **/Number of categorised covariate (%)** | **Number of covariates with justification for covariate categories given**  **/Number of categorised covariate (%)** |
| --- | --- | --- | --- | --- | --- | --- | --- | --- |
| Aboumarzouk 2012 | - | - | - | 0/4 (0) | 0/4 (0) | 0/4 (0) | 0/4 (0) | 0/4 (0) |
| Almeida 2013 | 1 | 0/1 (0) | 1 | 6/8 (75) | 6/8 (75) | 0/8 (0) | 0/8 (0) | 0/8 (0) |
| Basurto Ona 2013 | 1 | 0/1 (0) | 1 | 8/8 (100) | 7/8 (88) | 0/8 (0) | 1/8 (13) | 0/8 (0) |
| Bellmunt-Montoya 2013 | 4 | 0/1 (0) | 4 | 4/4 (100) | 4/4 (100) | 0/4 (0) | 0/4 (0) | 0/4 (0) |
| Berlowitz 2013 | - | - | - | 1/5 (20) | 1/5 (20) | 0/5 (0) | 0/5 (0) | 0/5 (0) |
| Boselie 2012 | 3, 3, 4, 6 | 0/4 (0) | 3.5 | 4/7 (57) | 3/7 (43) | 0/7 (0) | 1/7 (14) | 1/7 (14) |
| Bruins Slot 2013 | 1, 2, 2, 3, 3, 3, 3, 5, 7, 9, 9 | 0/11 (0) | 3 | 12/13 (92) | 6/13 (46) | 0/13 (0) | 6/13 (46) | 0/13 (0) |
| Cavalheri, 2013 | - | - | - | 3/7 (43) | 0/7 (0) | 3/7 (43) | 0/7 (0) | 0/7 (0) |
| Chaparro 2013 | 3, 5, 8, 22 | 1/4 (25) | 6.5 | 4/8 (50) | 1/8 (13) | 0/8 (0) | 3/8 (38) | 0/8 (0) |
| Cheng 2013 | 5 | 0/1 (0) | 5 | 10/10 (100) | 7/10 (70) | 1/10 (10) | 2/10 (20) | 0/10 (0) |
| Cruciani 2013 | NR, NR, 3, 8 | 0/2 (0) | 5.5 | 4/6 (67) | 3/6 (50) | 0/6 (0) | 1/6 (17) | 0/6 (0) |
| Dashash 2013 | - | - | - | 4/8 (50) | 4/8 (50) | 0/8 (0) | 0/8 (0) | 0/8 (0) |
| Deare 2013 | 2, 4, 6, 9 | 0/4 (0) | 5 | 7/8 (88) | 3/8 (38) | 0/8 (0) | 4/8 (50) | 0/8 (0) |
| Freak-Poli 2013 | 4 | 0/1 (0) | 4 | 10/13 (77) | 10/13 (77) | 0/13 (0) | 0/13 (0) | 0/13 (0) |
| Gan, 2013 | - | - | - | 5/8 (63) | 4/8 (50) | 0/8 (0) | 1/8 (13) | 0/8 (0) |
| Gillies 2012 | 2, 4, 4, 4, 9 | 0/5 (0) | 4 | 11/12 (92) | 4/12 (33) | 3/12 (25) | 4/12 (33) | 0/12 (0) |
| Gois 2013 | - | - | - | 5/11 (45) | 0/11 (0) | 5/11 (45) | 0/11 (0) | 0/11 (0) |
| Goldenberg 2013 | 14, 21, 22, 23 | 4/4 (100) | 21.5 | 4/5 (80) | 0/5 (0) | 0/5 (0) | 4/5 (80) | 3/5 (60) |
| Gower 2013 | 4, 4 | 0/2 (0) | 4 | 4/9 (44) | 1/9 (11) | 2/9 (22) | 1/9 (11) | 0/9 (0) |
| He 2013 | - | - | - | 8/8 (100) | 8/8 (100) | 0/8 (0) | 0/8 (0) | 0/8 (0) |
| Itchaki 2013 | 3, 3, 4, 5 | 0/4 (0) | 3.5 | 9/12 (75) | 3/12 (25) | 2/12 (17) | 4/12 (33) | 0/12 (0) |
| Kinnersley 2013 | - | - | - | 11/12 (92) | 7/12 (58) | 2/12 (17) | 2/12 (17) | 0/12 (0) |
| Lawrie 2013 | 10 | 1/1 (100) | 10 | 3/3 (100) | 3/3 (100) | 0/3 (0) | 0/3 (0) | 0/3 (0) |
| Lee 2013 | - | - | - | 3/6 (50) | 2/6 (33) | 0/6 (0) | 1/6 (17) | 1/6 (17) |
| Li 2013 | - | - | - | 2/6 (33) | 2/6 (33) | 0/6 (0) | 0/6 (0) | 0/6 (0) |
| Liu 2013 | - | - | - | 7/11 (64) | 7/11 (64) | 0/11 (0) | 0/11 (0) | 0/11 (0) |
| Lopez 2013 | 1, 7 | 0/2 (0) | 4 | 2/2 (100) | 0/2 (0) | 0/2 (0) | 2/2 (100) | 0/2 (0) |
| Marigold 2013 | - | - | - | 7/8 (88) | 6/8 (75) | 1/8 (13) | 0/8 (0) | 0/8 (0) |
| Mocellin 2013 | 11, 15, 17, 17, 17, 17 | 6/6 (100) | 17 | 7/11 (64) | 0/11 (0) | 2/11 (18) | 5/11 (45) | 0/11 (0) |
| Mutua 2012 | 3, 3 | 0/2 (0) | 3 | 6/8 (75) | 2/8 (25) | 2/8 (25) | 2/8 (25) | 0/8 (0) |
| Parker 2013 | - | - | - | 8/11 (73) | 8/11 (73) | 0/11 (0) | 0/11 (0) | 0/11 (0) |
| Pega, 2013 | - | - | - | 4/6 (67) | 2/6 (33) | 2/6 (33) | 0/6 (0) | 0/6 (0) |
| Penninga 2013 | - | - | - | 5/5 (100) | 5/5 (100) | 0/5 (0) | 0/5 (0) | 4/5 (80) |
| Peters 2013 | 3, 3 | 0/2 (0) | 3 | 11/11 (100) | 5/11 (45) | 4/11 (36) | 2/11 (18) | 0/11 (0) |
| Rockers 2013 | 1 | 0/1 (0) | 1 | 2/2 (100) | 0/2 (0) | 1/2 (50) | 1/2 (50) | 0/2 (0) |
| Sajid, 2012 | 17 | 1/1 (100) | 17 | 4/4 (100) | 2/4 (50) | 2/4 (50) | 0/4 (0) | 0/4 (0) |
| Sampson 2013 | 3, 5, 12 | 1/3 (33) | 5 | 4/4 (100) | 3/4 (75) | 0/4 (0) | 1/4 (25) | 0/4 (0) |
| Sanders 2013 | 3, 4 | 0/2 (0) | 3.5 | 4/9 (44) | 3/9 (33) | 0/9 (0) | 1/9 (11) | 1/9 (11) |
| Sarai 2013 | - | - | - | 7/10 (70) | 7/10 (70) | 0/10 (0) | 0/10 (0) | 0/10 (0) |
| Schoot 2013 | NR, 3 | 0/1 (0) | 3 | 3/3 (100) | 1/3 (33) | 0/3 (0) | 2/3 (67) | 0/3 (0) |
| Semple 2013 | 5 | 0/1 (0) | 5 | 6/6 (100) | 6/6 (100) | 0/6 (0) | 0/6 (0) | 0/6 (0) |
| Sharma 2013 | 3, 5, 6 | 0/3 (0) | 5 | 6/7 (86) | 2/7 (29) | 1/7 (14) | 3/7 (43) | 0/7 (0) |
| Showell 2013 | 1, 1, 1, 1 | 0/4 (0) | 1 | 10/11 (91) | 0/11 (0) | 3/11 (27) | 7/11 (64) | 0/11 (0) |
| Stead 2012 | 38, 38, 38, 38, 38, 38, 38 | 7/7 (100) | 38 | 6/10 (60) | 1/10 (10) | 0/10 (0) | 5/10 (50) | 0/10 (0) |
| Trivedi 2013 | - | - | - | 6/6 (100) | 5/6 (83) | 0/6 (0) | 1/6 (17) | 0/6 (0) |
| Trotti 2012 | 1, 2, 3 | 0/3 (0) | 2 | 5/5 (100) | 4/5 (80) | 0/5 (0) | 1/5 (20) | 0/5 (0) |
| Van Teeffelen, 2013 | - | - | - | 5/5 (100) | 5/5 (100) | 0/5 (0) | 0/5 (0) | 2/5 (40) |
| van Zuuren 2013 | 1 | 0/1 (0) | 1 | 6/8 (75) | 5/8 (63) | 0/8 (0) | 1/8 (13) | 0/8 (0) |
| Wakai 2013 | 1 | 0/1 (0) | 1 | 3/4 (75) | 2/4 (50) | 0/4 (0) | 1/4 (25) | 0/4 (0) |
| Wang 2013 | 1 | 0/1 (0) | 1 | 3/5 (60) | 1/5 (20) | 2/5 (40) | 0/5 (0) | 0/5 (0) |
| Yue 2013 | 2, 5, 6, 9 | 0/4 (0) | 5.5 | 6/6 (100) | 5/6 (83) | 0/6 (0) | 1/6 (17) | 1/6 (17) |
| Ziebell 2013 | - | - | - | 7/13 (54) | 0/13 (0) | 7/13 (54) | 0/13 (0) | 0/13 (0) |
| Summed totals | - | 21/88 (24) | - | 292/392 (74) | 176/392 (45) | 45/392 (11) | 71/392 (18) | 13/392 (3) |
| Number of reviews with > 1 covariate in numerator | - | 7/33 (21) | - | 51/52 (98) | 43/52 (83) | 18/52 (35) | 30/52 (58) | 7/52 (13) |
| Median | 4 | 0 | 4* | 76 | 46 | 0 | 13 | 0 |
| IQR | 3-9 | 0-0 | 3-5* | 60-100 | 20-75 | 0-17 | 0-27 | 0-0 |
| Range | 1-38 | 0-100 | 1-38* | 0-100 | 0-100 | 0-54 | 0-100 | 0-80 |

NR: Number of trials not reported. IQR: inter-quartile range.

*Analyses for which the number of trials was not reported were excluded from the calculation.
