# Supplementary material for: Exploring Treatment by Covariate Interactions Using Subgroup Analysis and Meta-Regression in Cochrane Reviews: A Review of Recent Practice
Source: PLoS One. 2015 Jun 1;10(6):e0128804. doi: 10.1371/journal.pone.0128804 (PMC4452239; doi:10.1371/journal.pone.0128804)
Supplement: S8 Table — (DOCX) [file pone.0128804.s010.docx]

**Table S8: Analysing interactions: The type of interaction analysis reported in the protocol and review, and whether we judged the analysis to be of that type.**

| **Reported planned methods** | **SU+M** | **SU** | **SE** | **U** | **U** | **SU+M** | **SU or M** | **SU+SE** | **SU** | **SE** | **Visually +SU+M** | **M** | **NR** | **NR** | **NR** | **SU** | **SE** | **NR** | **NR** | **NR** | **SE** | **SE** | **SU** | **SU** | **M** | **SU+M** | **U** | **U** | **Any method planned /Number of covariates reported (%)** | **Any method used**  **/Number of covariates reported (%)** |
| --- | --- | --- | --- | --- | --- | --- | --- | --- | --- | --- | --- | --- | --- | --- | --- | --- | --- | --- | --- | --- | --- | --- | --- | --- | --- | --- | --- | --- | --- | --- |
| **Planned method according to Glossary 1** | **SE +M** | **SE** | **SU** | **Unclear** | **SU** | **SU+ M** | **SU or M** | **SU+SE** | **SU** | **SE** | **SU + M** | **M** | **-** | **-** | **-** | **SU** | **SE** | **-** | **-** | **-** | **SE** | **SE** | **SU** | **SU** | **M** | **SU+M** | **SU** | **SU** |  |  |
| **Reported used method** | **NA** | **NA** | **NA** | **NA** | **NA** | **NA** | **NA** | **NA** | **NA** | **NA** | **NA** | **NA** | **NA** | **SE** | **SU** | **SU** | **SE** | **SE** | **SU** | **U** | **U** | **SE** | **SU** | **SU +M** | **SU +M** | **SU +M** | **U** | **SU** |  |  |
| **Used according to Glossary 1** | **NA** | **NA** | **NA** | **NA** | **NA** | **NA** | **NA** | **NA** | **NA** | **NA** | **NA** | **NA** | **NA** | **SE** | **SU** | **SU** | **SE** | **SU** | **SE** | **SU** | **SE** | **SU** | **SE** | **SU** | **M** | **M** | **SU** | **SU** |  |  |
| Aboumarzouk 2012 | 0/6 (0) | 0/6 (0) | 0/6 (0) | 0/6 (0) | 0/6 (0) | 0/6 (0) | 5/6 (83) | 0/6 (0) | 0/6 (0) | 1/6 (17) | 0/6 (0) | 0/6 (0) | 0/6 (0) | 0/6 (0) | 0/6 (0) | 0/6 (0) | 0/6 (0) | 0/6 (0) | 0/6 (0) | 0/6 (0) | 0/6 (0) | 0/6 (0) | 0/6 (0) | 0/6 (0) | 0/6 (0) | 0/6 (0) | 0/6 (0) | 0/6 (0) | 6/6 (100) | 0/6 (0) |
| Almeida 2013 | 0/8 (0) | 0/8 (0) | 0/8 (0) | 0/8 (0) | 0/8 (0) | 0/8 (0) | 0/8 (0) | 0/8 (0) | 3/8 (38) | 4/8 (50) | 0/8 (0) | 0/8 (0) | 0/8 (0) | 0/8 (0) | 0/8 (0) | 0/8 (0) | 0/8 (0) | 0/8 (0) | 0/8 (0) | 0/8 (0) | 0/8 (0) | 0/8 (0) | 0/8 (0) | 0/8 (0) | 0/8 (0) | 0/8 (0) | 1/8 (13) | 0/8 (0) | 8/8 (100) | 1/8 (13) |
| Basurto Ona 2013 | 0/8 (0) | 0/8 (0) | 0/8 (0) | 0/8 (0) | 0/8 (0) | 0/8 (0) | 0/8 (0) | 0/8 (0) | 4/8 (50) | 3/8 (38) | 0/8 (0) | 0/8 (0) | 0/8 (0) | 0/8 (0) | 0/8 (0) | 0/8 (0) | 0/8 (0) | 0/8 (0) | 0/8 (0) | 1/8 (13) | 0/8 (0) | 0/8 (0) | 0/8 (0) | 0/8 (0) | 0/8 (0) | 0/8 (0) | 0/8 (0) | 0/8 (0) | 7/8 (88) | 1/8 (13) |
| Bellmunt-Montoya 2013 | 0/4 (0) | 0/4 (0) | 2/4 (50) | 0/4 (0) | 0/4 (0) | 0/4 (0) | 0/4 (0) | 0/4 (0) | 1/4 (25) | 0/4 (0) | 0/4 (0) | 0/4 (0) | 0/4 (0) | 0/4 (0) | 0/4 (0) | 1/4 (25) | 0/4 (0) | 0/4 (0) | 0/4 (0) | 0/4 (0) | 0/4 (0) | 0/4 (0) | 0/4 (0) | 0/4 (0) | 0/4 (0) | 0/4 (0) | 0/4 (0) | 0/4 (0) | 4/4 (100) | 1/4 (25) |
| Berlowitz 2013 | 0/6 (0) | 0/6 (0) | 0/6 (0) | 0/6 (0) | 0/6 (0) | 0/6 (0) | 0/6 (0) | 0/6 (0) | 3/6 (50) | 3/6 (50) | 0/6 (0) | 0/6 (0) | 0/6 (0) | 0/6 (0) | 0/6 (0) | 0/6 (0) | 0/6 (0) | 0/6 (0) | 0/6 (0) | 0/6 (0) | 0/6 (0) | 0/6 (0) | 0/6 (0) | 0/6 (0) | 0/6 (0) | 0/6 (0) | 0/6 (0) | 0/6 (0) | 6/6 (100) | 0/6 (0) |
| Boselie 2012 | 0/7 (0) | 0/7 (0) | 0/7 (0) | 0/7 (0) | 0/7 (0) | 0/7 (0) | 0/7 (0) | 0/7 (0) | 2/7 (29) | 0/7 (0) | 0/7 (0) | 0/7 (0) | 1/7 (14) | 1/7 (14) | 0/7 (0) | 0/7 (0) | 2/7 (29) | 0/7 (0) | 0/7 (0) | 0/7 (0) | 0/7 (0) | 0/7 (0) | 0/7 (0) | 0/7 (0) | 0/7 (0) | 0/7 (0) | 1/7 (14) | 0/7 (0) | 5/7 (71) | 4**/**7 **(**57**)** |
| Bruins Slot 2013 | 0/13 (0) | 0/13 (0) | 0/13 (0) | 0/13 (0) | 0/13 (0) | 0/13 (0) | 0/13 (0) | 0/13 (0) | 2/13 (15) | 0/13 (0) | 0/13 (0) | 0/13 (0) | 0/13 (0) | 0/13 (0) | 0/13 (0) | 10/13 (77) | 0/13 (0) | 0/13 (0) | 0/13 (0) | 1/13 (8) | 0/13 (0) | 0/13 (0) | 0/13 (0) | 0/13 (0) | 0/13 (0) | 0/13 (0) | 0/13 (0) | 0/13 (0) | 12/13 (92) | 11/13 (85) |
| Cavalheri, 2013 | 0**/**7 **(**0**)** | 0**/**7 **(**0**)** | 0**/**7 **(**0**)** | 0**/**7 **(**0**)** | 0**/**7 **(**0**)** | 0**/**7 **(**0**)** | 0**/**7 **(**0**)** | 0**/**7 **(**0**)** | 3/7 (43) | 4/7 (43) | 0**/**7 **(**0**)** | 0**/**7 **(**0**)** | 0**/**7 **(**0**)** | 0/7 (0) | 0**/**7 **(**0**)** | 0**/**7 **(**0**)** | 0**/**7 **(**0**)** | 0**/**7 **(**0**)** | 0**/**7 **(**0**)** | 0**/**7 **(**0**)** | 0**/**7 **(**0**)** | 0**/**7 **(**0**)** | 0**/**7 **(**0**)** | 0**/**7 **(**0**)** | 0**/**7 **(**0**)** | 0**/**7 **(**0**)** | 0**/**7 **(**0**)** | 0**/**7 **(**0**)** | 7/7 (100) | 0**/**7 **(**0**)** |
| Chaparro 2013 | 0/10 (0) | 0/10 (0) | 0/10 (0) | 0/10 (0) | 0/10 (0) | 0/10 (0) | 0/10 (0) | 1/10 (10) | 2/10 (20) | 3/10 (30) | 0/10 (0) | 0/10 (0) | 0/10 (0) | 0/10 (0) | 0/10 (0) | 1/10 (10) | 0/10 (0) | 0/10 (0) | 0/10 (0) | 2/10 (20) | 0/10 (0) | 0/10 (0) | 0/10 (0) | 0/10 (0) | 0/10 (0) | 0/10 (0) | 1/10 (10) | 0/10 (0) | 8/10 (80) | 4**/**10 **(**40**)** |
| Cheng 2013 | 0/10 (0) | 0/10 (0) | 0/10 (0) | 0/10 (0) | 0/10 (0) | 0/10 (0) | 0/10 (0) | 0/10 (0) | 3/10 (30) | 4/10 (40) | 0/10 (0) | 0/10 (0) | 2/10 (20) | 0/10 (0) | 0/10 (0) | 0/10 (0) | 0/10 (0) | 0/10 (0) | 0/10 (0) | 0/10 (0) | 0/10 (0) | 0/10 (0) | 0/10 (0) | 0/10 (0) | 0/10 (0) | 0/10 (0) | 1**/**10 **(**10**)** | 0/10 (0) | 8/10 (80) | 1**/**10 **(**10**)** |
| Cruciani 2013 | 0/7 (0) | 0/7 (0) | 0/7 (0) | 1/7 (14) | 0/7 (0) | 0/7 (0) | 0/7 (0) | 0/7 (0) | 2/7 (29) | 0/7 (0) | 0/7 (0) | 0/7 (0) | 0/7 (0) | 0/7 (0) | 0/7 (0) | 2/7 (29) | 0/7 (0) | 0/7 (0) | 0/7 (0) | 1/7 (14) | 0/7 (0) | 1/7 (14) | 0/7 (0) | 0/7 (0) | 0/7 (0) | 0/7 (0) | 0/7 (0) | 0/7 (0) | 6/7 (86) | 4**/**7 **(**57**)** |
| Dashash 2013 | 0/8 (0) | 0/8 (0) | 0/8 (0) | 0/8 (0) | 0/8 (0) | 0/8 (0) | 0/8 (0) | 0/8 (0) | 4/8 (50) | 4/8 (50) | 0/8 (0) | 0/8 (0) | 0/8 (0) | 0/8 (0) | 0/8 (0) | 0/8 (0) | 0/8 (0) | 0/8 (0) | 0/8 (0) | 0/8 (0) | 0/8 (0) | 0/8 (0) | 0/8 (0) | 0/8 (0) | 0/8 (0) | 0/8 (0) | 0/8 (0) | 0/8 (0) | 8/8 (100) | 0/8 (0) |
| Deare 2013 | 0/8 (0) | 0/8 (0) | 0/8 (0) | 0/8 (0) | 0/8 (0) | 0/8 (0) | 0/8 (0) | 0/8 (0) | 0/8 (0) | 2/8 (25) | 0/8 (0) | 0/8 (0) | 2/8 (25) | 0/8 (0) | 1/8 (13) | 0/8 (0) | 0/8 (0) | 0/8 (0) | 0/8 (0) | 2/8 (25) | 0/8 (0) | 0/8 (0) | 0/8 (0) | 0/8 (0) | 0/8 (0) | 0/8 (0) | 1/8 (13) | 0/8 (0) | 3/8 (38) | 4/8 (50) |
| Freak-Poli 2013 | 0/13 (0) | 0/13 (0) | 0/13 (0) | 0/13 (0) | 2/13 (15) | 0/13 (0) | 0/13 (0) | 0/13 (0) | 10/13 (77) | 0/13 (0) | 0/13 (0) | 0/13 (0) | 0/13 (0) | 0/13 (0) | 0/13 (0) | 0/13 (0) | 0/13 (0) | 0/13 (0) | 0/13 (0) | 0/13 (0) | 0/13 (0) | 0/13 (0) | 0/13 (0) | 0/13 (0) | 0/13 (0) | 0/13 (0) | 1**/**13 **(**8**)** | 0/13 (0) | 13/13 (100) | 1**/**13 **(**8**)** |
| Gan, 2013 | 0/8 (0) | 0/8 (0) | 0/8 (0) | 0/8 (0) | 0/8 (0) | 0/8 (0) | 0/8 (0) | 0/8 (0) | 7/8 (88) | 0/8 (0) | 0/8 (0) | 0/8 (0) | 1/8 (13) | 0/8 (0) | 0/8 (0) | 0/8 (0) | 0/8 (0) | 0/8 (0) | 0/8 (0) | 0/8 (0) | 0/8 (0) | 0/8 (0) | 0/8 (0) | 0/8 (0) | 0/8 (0) | 0/8 (0) | 0/8 (0) | 0/8 (0) | 7/8 (88) | 0/8 (0) |
| Gillies 2012 | 0/12 (0) | 0/12 (0) | 0/12 (0) | 0/12 (0) | 1/12 (8) | 0/12 (0) | 0/12 (0) | 0/12 (0) | 4/12 (33) | 1/12 (8) | 0/12 (0) | 0/12 (0) | 1/12 (8) | 0/12 (0) | 1/12 (8) | 1/12 (8) | 0/12 (0) | 0/12 (0) | 0/12 (0) | 0/12 (0) | 0/12 (0) | 1/12 (8) | 0/12 (0) | 0/12 (0) | 0/12 (0) | 0/12 (0) | 2/12 (17) | 0/12 (0) | 10/12 (83) | 5**/**12 **(**42**)** |
| Gois 2013 | 0/11 (0) | 0/11 (0) | 0/11 (0) | 0/11 (0) | 1/11 (9) | 0/11 (0) | 0/11 (0) | 0/11 (0) | 5/11 (45) | 5/11 (45) | 0/11 (0) | 0/11 (0) | 0/11 (0) | 0/11 (0) | 0/11 (0) | 0/11 (0) | 0/11 (0) | 0/11 (0) | 0/11 (0) | 0/11 (0) | 0/11 (0) | 0/11 (0) | 0/11 (0) | 0/11 (0) | 0/11 (0) | 0/11 (0) | 0/11 (0) | 0/11 (0) | 11/11 (100) | 0/11 (0) |
| Goldenberg 2013 | 0/6 (0) | 0/6 (0) | 0/6 (0) | 0/6 (0) | 0/6 (0) | 0/6 (0) | 0/6 (0) | 0/6 (0) | 0/6 (0) | 0/6 (0) | 0/6 (0) | 2/6 (33) | 0/6 (0) | 0/6 (0) | 4**/**6 **(**67**)** | 0/6 (0) | 0/6 (0) | 0/6 (0) | 0/6 (0) | 0/6 (0) | 0/6 (0) | 0/6 (0) | 0/6 (0) | 0/6 (0) | 0/6 (0) | 0/6 (0) | 0/6 (0) | 0/6 (0) | 2/6 (33) | 4**/**6 **(**67**)** |
| Gower 2013 | 0/10 (0) | 0/10 (0) | 0/10 (0) | 0/10 (0) | 0/10 (0) | 0/10 (0) | 0/10 (0) | 0/10 (0) | 6/10 (60) | 2/10 (20) | 0/10 (0) | 0/10 (0) | 0/10 (0) | 0/10 (0) | 0/10 (0) | 0/10 (0) | 0/10 (0) | 0/10 (0) | 0/10 (0) | 0/10 (0) | 0/10 (0) | 0/10 (0) | 0/10 (0) | 0/10 (0) | 0/10 (0) | 0/10 (0) | 2**/**10 **(**20**)** | 0/10 (0) | 10/10 (100) | 2**/**10 **(**20**)** |
| He 2013 | 0/8 (0) | 0/8 (0) | 0/8 (0) | 0/8 (0) | 0/8 (0) | 0/8 (0) | 0/8 (0) | 0/8 (0) | 6/8 (75) | 2/8 (25) | 0/8 (0) | 0/8 (0) | 0/8 (0) | 0/8 (0) | 0/8 (0) | 0/8 (0) | 0/8 (0) | 0/8 (0) | 0/8 (0) | 0/8 (0) | 0/8 (0) | 0/8 (0) | 0/8 (0) | 0/8 (0) | 0/8 (0) | 0/8 (0) | 0/8 (0) | 0/8 (0) | 8/8 (100) | 0**/**8 **(**0**)** |
| Itchaki 2013 | 2/12 (17) | 0/12 (0) | 0/12 (0) | 0/12 (0) | 1/12 (8) | 1/12 (8) | 0/12 (0) | 0/12 (0) | 1/12 (8) | 1/12 (8) | 0/12 (0) | 0/12 (0) | 2/12 (17) | 0/12 (0) | 0/12 (0) | 1/12 (8) | 0/12 (0) | 0/12 (0) | 0/12 (0) | 0/12 (0) | 1/12 (8) | 1/12 (8) | 0/12 (0) | 0/12 (0) | 0/12 (0) | 0/12 (0) | 1/12 (8) | 0/12 (0) | 10/12 (83) | 4/12 (33) |
| Kinnersley 2013 | 0/12 (0) | 0/12 (0) | 0/12 (0) | 0/12 (0) | 2/12 (17) | 0/12 (0) | 0/12 (0) | 0/12 (0) | 0/12 (0) | 3/12 (25) | 5/12 (42) | 0/12 (0) | 2/12 (17) | 0/12 (0) | 0/12 (0) | 0/12 (0) | 0/12 (0) | 0/12 (0) | 0/12 (0) | 0/12 (0) | 0/12 (0) | 0/12 (0) | 0/12 (0) | 0/12 (0) | 0/12 (0) | 0/12 (0) | 0/12 (0) | 0/12 (0) | 10/12 (83) | 0/12 (0) |
| Lawrie 2013 | 0/3 (0) | 0/3 (0) | 0/3 (0) | 0/3 (0) | 1/3 (33) | 0/3 (0) | 0/3 (0) | 0/3 (0) | 0/3 (0) | 1/3 (33) | 0/3 (0) | 0/3 (0) | 0/3 (0) | 0/3 (0) | 0/3 (0) | 0/3 (0) | 0/3 (0) | 0/3 (0) | 0/3 (0) | 0/3 (0) | 0/3 (0) | 0/3 (0) | 0/3 (0) | 0/3 (0) | 0/3 (0) | 0/3 (0) | 0/3 (0) | 1**/**3 **(**33**)** | 3/3 (100) | 1**/**3 **(**33**)** |
| Lee 2013 | 0/6 (0) | 0/6 (0) | 0/6 (0) | 0/6 (0) | 1/6 (17) | 0/6 (0) | 0/6 (0) | 0/6 (0) | 1/6 (17) | 3/6 (50) | 0/6 (0) | 0/6 (0) | 1/6 (17) | 0/6 (0) | 0/6 (0) | 0/6 (0) | 0/6 (0) | 0/6 (0) | 0/6 (0) | 0/6 (0) | 0/6 (0) | 0/6 (0) | 0/6 (0) | 0/6 (0) | 0/6 (0) | 0/6 (0) | 0/6 (0) | 0/6 (0) | 5/6 (83) | 0**/**6 **(**0**)** |
| Li 2013 | 0/6 (0) | 0/6 (0) | 0/6 (0) | 0/6 (0) | 0/6 (0) | 0/6 (0) | 0/6 (0) | 0/6 (0) | 4/6 (67) | 2/6 (33) | 0/6 (0) | 0/6 (0) | 0/6 (0) | 0/6 (0) | 0/6 (0) | 0/6 (0) | 0/6 (0) | 0/6 (0) | 0/6 (0) | 0/6 (0) | 0/6 (0) | 0/6 (0) | 0/6 (0) | 0/6 (0) | 0/6 (0) | 0/6 (0) | 0/6 (0) | 0/6 (0) | 6/6 (100) | 0/6 (0) |
| Liu 2013 | 0/11 (0) | 0/11 (0) | 0/11 (0) | 0/11 (0) | 0/11 (0) | 0/11 (0) | 0/11 (0) | 0/11 (0) | 2/11 (18) | 9/11 (82) | 0/11 (0) | 0/11 (0) | 0/11 (0) | 0/11 (0) | 0/11 (0) | 0/11 (0) | 0/11 (0) | 0/11 (0) | 0/11 (0) | 0/11 (0) | 0/11 (0) | 0/11 (0) | 0/11 (0) | 0/11 (0) | 0/11 (0) | 0/11 (0) | 0/11 (0) | 0/11 (0) | 11/11 (100) | 0**/**11 **(**0**)** |
| Lopez 2013 | 0/2 (0) | 0/2 (0) | 0/2 (0) | 0/2 (0) | 0/2 (0) | 0/2 (0) | 0/2 (0) | 0/2 (0) | 0/2 (0) | 0/2 (0) | 0/2 (0) | 0/2 (0) | 0/2 (0) | 0/2 (0) | 0/2 (0) | 0/2 (0) | 0/2 (0) | 0/2 (0) | 0/2 (0) | 2/2 (100) | 0/2 (0) | 0/2 (0) | 0/2 (0) | 0/2 (0) | 0/2 (0) | 0/2 (0) | 0/2 (0) | 0/2 (0) | 0/2 (0) | 2/2 (100) |
| Marigold 2013 | 0/8 (0) | 0/8 (0) | 0/8 (0) | 0/8 (0) | 2/8 (25) | 0/8 (0) | 0/8 (0) | 0/8 (0) | 0/8 (0) | 6/8 (75) | 0/8 (0) | 0/8 (0) | 0/8 (0) | 0/8 (0) | 0/8 (0) | 0/8 (0) | 0/8 (0) | 0/8 (0) | 0/8 (0) | 0/8 (0) | 0/8 (0) | 0/8 (0) | 0/8 (0) | 0/8 (0) | 0/8 (0) | 0/8 (0) | 0/8 (0) | 0/8 (0) | 8/8 (100) | 0/8 (0) |
| Mocellin 2013 | 0/14 (0) | 0/14 (0) | 0/14 (0) | 0/14 (0) | 0/14 (0) | 0/14 (0) | 0/14 (0) | 0/14 (0) | 3/14 (21) | 1/14 (7) | 0/14 (0) | 4/14 (29) | 0/14 (0) | 2/14 (14) | 0/14 (0) | 0/14 (0) | 0/14 (0) | 0/14 (0) | 0/14 (0) | 0/14 (0) | 0/14 (0) | 0/14 (0) | 0/14 (0) | 2/14 (14) | 1/14 (7) | 1/14 (7) | 0/14 (0) | 0/14 (0) | 12/14 (86) | 6**/**14 **(**43**)** |
| Mutua 2012 | 0/8 (0) | 0/8 (0) | 1/8 (13) | 0/8 (0) | 1/8 (13) | 0/8 (0) | 0/8 (0) | 0/8 (0) | 3/8 (38) | 0/8 (0) | 0/8 (0) | 0/8 (0) | 1/8 (13) | 0/8 (0) | 1/8 (13) | 0/8 (0) | 0/8 (0) | 0/8 (0) | 0/8 (0) | 0/8 (0) | 0/8 (0) | 0/8 (0) | 0/8 (0) | 0/8 (0) | 0/8 (0) | 0/8 (0) | 1/8 (13) | 0/8 (0) | 6/8 (75) | 2/8 (25) |
| Parker 2013 | 0/11 (0) | 0/11 (0) | 0/11 (0) | 0/11 (0) | 1/11 (9) | 0/11 (0) | 0/11 (0) | 0/11 (0) | 5/11 (45) | 5/11 (45) | 0/11 (0) | 0/11 (0) | 0/11 (0) | 0/11 (0) | 0/11 (0) | 0/11 (0) | 0/11 (0) | 0/11 (0) | 0/11 (0) | 0/11 (0) | 0/11 (0) | 0/11 (0) | 0/11 (0) | 0/11 (0) | 0/11 (0) | 0/11 (0) | 0/11 (0) | 0/11 (0) | 11/11 (100) | 0/11 (0) |
| Pega 2013 | 0/6 (0) | 0/6 (0) | 0/6 (0) | 0/6 (0) | 0/6 (0) | 0/6 (0) | 0/6 (0) | 0/6 (0) | 4/6 (67) | 2/6 (33) | 0/6 (0) | 0/6 (0) | 0/6 (0) | 0/6 (0) | 0/6 (0) | 0/6 (0) | 0/6 (0) | 0/6 (0) | 0/6 (0) | 0/6 (0) | 0/6 (0) | 0/6 (0) | 0/6 (0) | 0/6 (0) | 0/6 (0) | 0/6 (0) | 0/6 (0) | 0/6 (0) | 6/6 (100) | 0**/**6 **(**0**)** |
| Penninga 2013 | 0/5 (0) | 0/5 (0) | 0/5 (0) | 0/5 (0) | 0/5 (0) | 0/5 (0) | 0/5 (0) | 0/5 (0) | 5/5 (100) | 0/5 (0) | 0/5 (0) | 0/5 (0) | 0/5 (0) | 0**/**5 **(**0**)** | 0**/**5 **(**0**)** | 0**/**5 **(**0**)** | 0**/**5 **(**0**)** | 0**/**5 **(**0**)** | 0**/**5 **(**0**)** | 0**/**5 **(**0**)** | 0**/**5 **(**0**)** | 0**/**5 **(**0**)** | 0**/**5 **(**0**)** | 0**/**5 **(**0**)** | 0**/**5 **(**0**)** | 0**/**5 **(**0**)** | 0**/**5 **(**0**)** | 0**/**5 **(**0**)** | 5/5 (100) | 0/5 (0) |
| Peters 2013 | 0/11 (0) | 0/11 (0) | 0/11 (0) | 0/11 (0) | 0/11 (0) | 0/11 (0) | 0/11 (0) | 0/11 (0) | 4/11 (36) | 5/11 (45) | 0/11 (0) | 0/11 (0) | 0/11 (0) | 0/11 (0) | 0/11 (0) | 0/11 (0) | 0/11 (0) | 0/11 (0) | 0/11 (0) | 2/11 (18) | 0/11 (0) | 0/11 (0) | 0/11 (0) | 0/11 (0) | 0/11 (0) | 0/11 (0) | 0/11 (0) | 0/11 (0) | 9/11 (82) | 2/11 (18) |
| Rockers 2013 | 0/2 (0) | 0/2 (0) | 0/2 (0) | 0/2 (0) | 0/2 (0) | 0/2 (0) | 0/2 (0) | 0/2 (0) | 0/2 (0) | 1/2 (50) | 0/2 (0) | 0/2 (0) | 0/2 (0) | 0/2 (0) | 0/2 (0) | 0/2 (0) | 0/2 (0) | 0/2 (0) | 0/2 (0) | 1**/**2 **(**50**)** | 0/2 (0) | 0/2 (0) | 0/2 (0) | 0/2 (0) | 0/2 (0) | 0/2 (0) | 0/2 (0) | 0/2 (0) | 1/2 (50) | 1**/**2 **(**50**)** |
| Sajid, 2012 | 0/4 (0) | 0/4 (0) | 0/4 (0) | 0/4 (0) | 0/4 (0) | 0/4 (0) | 0/4 (0) | 0/4 (0) | 1/4 (25) | 2/4 (50) | 0/4 (0) | 0/4 (0) | 0/4 (0) | 0/4 (0) | 0/4 (0) | 1/4 (25) | 0/4 (0) | 0/4 (0) | 0/4 (0) | 0/4 (0) | 0/4 (0) | 0/4 (0) | 0/4 (0) | 0/4 (0) | 0/4 (0) | 0/4 (0) | 0/4 (0) | 0/4 (0) | 4/4 (100) | 1**/**4 **(**25**)** |
| Sampson 2013 | 0/4 (0) | 0/4 (0) | 0/4 (0) | 0/4 (0) | 0/4 (0) | 0/4 (0) | 0/4 (0) | 0/4 (0) | 0/4 (0) | 0/4 (0) | 0/4 (0) | 0/4 (0) | 1/4 (25) | 0/4 (0) | 0/4 (0) | 1/4 (25) | 1/4 (25) | 0/4 (0) | 0/4 (0) | 0/4 (0) | 0/4 (0) | 0/4 (0) | 0/4 (0) | 0/4 (0) | 0/4 (0) | 0/4 (0) | 0/4 (0) | 1/4 (25) | 3/4 (75) | 3**/**4 **(**75**)** |
| Sanders 2013 | 0/11 (0) | 0/11 (0) | 0/11 (0) | 0/11 (0) | 0/11 (0) | 2/11 (18) | 0/11 (0) | 0/11 (0) | 3/11 (27) | 3/11 (27) | 0/11 (0) | 0/11 (0) | 1/11 (9) | 1/11 (9) | 0/11 (0) | 0/11 (0) | 0/11 (0) | 0/11 (0) | 0/11 (0) | 0/11 (0) | 0/11 (0) | 0/11 (0) | 0/11 (0) | 0/11 (0) | 0/11 (0) | 0/11 (0) | 1/11 (9) | 0/11 (0) | 9/11 (82) | 2/11 (18) |
| Sarai 2013 | 0**/**10 **(**0**)** | 0**/**10 **(**0**)** | 6/10 (60) | 0**/**10 **(**0**)** | 0**/**10 **(**0**)** | 0**/**10 **(**0**)** | 0**/**10 **(**0**)** | 0**/**10 **(**0**)** | 4/10 (40) | 0**/**10 **(**0**)** | 0**/**10 **(**0**)** | 0**/**10 **(**0**)** | 0**/**10 **(**0**)** | 0**/**10 **(**0**)** | 0**/**10 **(**0**)** | 0**/**10 **(**0**)** | 0**/**10 **(**0**)** | 0**/**10 **(**0**)** | 0**/**10 **(**0**)** | 0**/**10 **(**0**)** | 0**/**10 **(**0**)** | 0**/**10 **(**0**)** | 0**/**10 **(**0**)** | 0**/**10 **(**0**)** | 0**/**10 **(**0**)** | 0**/**10 **(**0**)** | 0**/**10 **(**0**)** | 0**/**10 **(**0**)** | 10/10 (100) | 0**/**10 **(**0**)** |
| Schoot 2013 | 0/3 (0) | 0/3 (0) | 0/3 (0) | 0/3 (0) | 0/3 (0) | 0/3 (0) | 0/3 (0) | 0/3 (0) | 1/3 (33) | 0/3 (0) | 0/3 (0) | 0/3 (0) | 0/3 (0) | 0/3 (0) | 0/3 (0) | 0/3 (0) | 1/3 (33) | 0/3 (0) | 0/3 (0) | 0/3 (0) | 0/3 (0) | 0/3 (0) | 0/3 (0) | 0/3 (0) | 0/3 (0) | 0/3 (0) | 1/3 (33) | 0/3 (0) | 3/3 (100) | 2/3 (67) |
| Semple 2013 | 0/6 (0) | 0/6 (0) | 0/6 (0) | 0/6 (0) | 0/6 (0) | 0/6 (0) | 0/6 (0) | 0/6 (0) | 4/6 (67) | 1/6 (17) | 0/6 (0) | 0/6 (0) | 0/6 (0) | 0/6 (0) | 0/6 (0) | 1/6 (17) | 0/6 (0) | 0/6 (0) | 0/6 (0) | 0/6 (0) | 0/6 (0) | 0/6 (0) | 0/6 (0) | 0/6 (0) | 0/6 (0) | 0/6 (0) | 0/6 (0) | 0/6 (0) | 6/6 (100) | 1/6 (17) |
| Sharma 2013 | 0/7 (0) | 0/7 (0) | 0/7 (0) | 0/7 (0) | 1/7 (14) | 0/7 (0) | 0/7 (0) | 0/7 (0) | 2/7 (29) | 1/7 (14) | 0/7 (0) | 0/7 (0) | 0/7 (0) | 0/7 (0) | 0/7 (0) | 1/7 (14) | 0/7 (0) | 0/7 (0) | 0/7 (0) | 2/7 (29) | 0/7 (0) | 0/7 (0) | 0/7 (0) | 0/7 (0) | 0/7 (0) | 0/7 (0) | 0/7 (0) | 0/7 (0) | 5/7 (71) | 3/7 (43) |
| Showell 2013 | 0/12 (0) | 1/12 (8) | 0/12 (0) | 0/12 (0) | 0/12 (0) | 0/12 (0) | 0/12 (0) | 0/12 (0) | 2/12 (17) | 2/12 (17) | 0/12 (0) | 0/12 (0) | 3/12 (25) | 0/12 (0) | 3/12 (25) | 0/12 (0) | 0/12 (0) | 0/12 (0) | 0/12 (0) | 1/12 (8) | 0/12 (0) | 0/12 (0) | 0/12 (0) | 0/12 (0) | 0/12 (0) | 0/12 (0) | 0/12 (0) | 0/12 (0) | 5/12 (42) | 4/12 (33) |
| Stead 2012 | 0/11 (0) | 0/11 (0) | 0/11 (0) | 0/11 (0) | 0/11 (0) | 0/11 (0) | 0/11 (0) | 0/11 (0) | 2/11 (18) | 2/11 (18) | 0/11 (0) | 0/11 (0) | 0/11 (0) | 0/11 (0) | 4/11 (36) | 2/11 (18) | 0/11 (0) | 1/11 (9) | 0/11 (0) | 0/11 (0) | 0/11 (0) | 0/11 (0) | 0/11 (0) | 0/11 (0) | 0/11 (0) | 0/11 (0) | 0/11 (0) | 0/11 (0) | 6/11 (55) | 7**/**11 **(**64**)** |
| Trivedi 2013 | 0/6 (0) | 0/6 (0) | 0/6 (0) | 0/6 (0) | 0/6 (0) | 0/6 (0) | 0/6 (0) | 0/6 (0) | 5/6 (83) | 0/6 (0) | 0/6 (0) | 0/6 (0) | 1/6 (17) | 0/6 (0) | 0/6 (0) | 0/6 (0) | 0/6 (0) | 0/6 (0) | 0/6 (0) | 0/6 (0) | 0/6 (0) | 0/6 (0) | 0/6 (0) | 0/6 (0) | 0/6 (0) | 0/6 (0) | 0/6 (0) | 0/6 (0) | 5/6 (83) | 0**/**6 **(**0**)** |
| Trotti 2012 | 0/5 (0) | 0/5 (0) | 2/5 (40) | 0/5 (0) | 0/5 (0) | 0/5 (0) | 0/5 (0) | 0/5 (0) | 0/5 (0) | 0/5 (0) | 0/5 (0) | 0/5 (0) | 0/5 (0) | 0/5 (0) | 0/5 (0) | 0/5 (0) | 0/5 (0) | 0/5 (0) | 1/5 (20) | 0/5 (0) | 0/5 (0) | 0/5 (0) | 2/5 (40) | 0/5 (0) | 0/5 (0) | 0/5 (0) | 0/5 (0) | 0/5 (0) | 4/5 (80) | 3/5 (60) |
| Van Teeffelen, 2013 | 0**/**5 **(**0**)** | 0**/**5 **(**0**)** | 0**/**5 **(**0**)** | 0**/**5 **(**0**)** | 0**/**5 **(**0**)** | 0**/**5 **(**0**)** | 0**/**5 **(**0**)** | 0**/**5 **(**0**)** | 2/5 (40) | 3/5 (60) | 0**/**5 **(**0**)** | 0**/**5 **(**0**)** | 0**/**5 **(**0**)** | 0**/**5 **(**0**)** | 0**/**5 **(**0**)** | 0**/**5 **(**0**)** | 0**/**5 **(**0**)** | 0**/**5 **(**0**)** | 0**/**5 **(**0**)** | 0**/**5 **(**0**)** | 0**/**5 **(**0**)** | 0**/**5 **(**0**)** | 0**/**5 **(**0**)** | 0**/**5 **(**0**)** | 0**/**5 **(**0**)** | 0**/**5 **(**0**)** | 0**/**5 **(**0**)** | 0**/**5 **(**0**)** | 5/5 (100) | 0**/**5 **(**0**)** |
| van Zuuren 2013 | 0/8 (0) | 0/8 (0) | 0/8 (0) | 0/8 (0) | 0/8 (0) | 0/8 (0) | 0/8 (0) | 0/8 (0) | 3/8 (38) | 4/8 (50) | 0/8 (0) | 0/8 (0) | 0/8 (0) | 0/8 (0) | 0/8 (0) | 0/8 (0) | 0/8 (0) | 0/8 (0) | 0/8 (0) | 1/8 (13) | 0/8 (0) | 0/8 (0) | 0/8 (0) | 0/8 (0) | 0/8 (0) | 0/8 (0) | 0/8 (0) | 0/8 (0) | 7/8 (88) | 1**/**8 **(**13**)** |
| Wakai 2013 | 0/4 (0) | 0/4 (0) | 2/4 (50) | 0/4 (0) | 0/4 (0) | 0/4 (0) | 0/4 (0) | 0/4 (0) | 1/4 (25) | 0/4 (0) | 0/4 (0) | 0/4 (0) | 0/4 (0) | 0/4 (0) | 0/4 (0) | 0/4 (0) | 0/4 (0) | 0/4 (0) | 0/4 (0) | 1/4 (25) | 0/4 (0) | 0/4 (0) | 0/4 (0) | 0/4 (0) | 0/4 (0) | 0/4 (0) | 0/4 (0) | 0/4 (0) | 3/4 (75) | 1**/**4 **(**25**)** |
| Wang 2013 | 0/6 (0) | 0/6 (0) | 0/6 (0) | 0/6 (0) | 0/6 (0) | 0/6 (0) | 0/6 (0) | 0/6 (0) | 3/6 (50) | 2/6 (33) | 0/6 (0) | 0/6 (0) | 0/6 (0) | 0/6 (0) | 0/6 (0) | 0/6 (0) | 0/6 (0) | 0/6 (0) | 0/6 (0) | 0/6 (0) | 0/6 (0) | 0/6 (0) | 0/6 (0) | 0/6 (0) | 0/6 (0) | 0/6 (0) | 1/6 (17) | 0/6 (0) | 6/6 (100) | 1**/**6 **(**17**)** |
| Yue 2013 | 0/6 (0) | 0/6 (0) | 0/6 (0) | 0/6 (0) | 0/6 (0) | 0/6 (0) | 0/6 (0) | 0/6 (0) | 1/6 (17) | 1/6 (17) | 0/6 (0) | 0/6 (0) | 0/6 (0) | 0/6 (0) | 0/6 (0) | 1/6 (17) | 1/6 (17) | 0/6 (0) | 1/6 (17) | 0/6 (0) | 0/6 (0) | 0/6 (0) | 0/6 (0) | 0/6 (0) | 0/6 (0) | 0/6 (0) | 1/6 (17) | 0/6 (0) | 5/6 (83) | 4**/**6 **(**67**)** |
| Ziebell 2013 | 0/14 (0) | 0/14 (0) | 6/14 (43) | 0/14 (0) | 0/14 (0) | 0/14 (0) | 0/14 (0) | 0/14 (0) | 2/14 (14) | 4/14 (29) | 0/14 (0) | 2/14 (14) | 0/14 (0) | 0/14 (0) | 0/14 (0) | 0/14 (0) | 0/14 (0) | 0/14 (0) | 0/14 (0) | 0/14 (0) | 0/14 (0) | 0/14 (0) | 0/14 (0) | 0/14 (0) | 0/14 (0) | 0/14 (0) | 0/14 (0) | 0/14 (0) | 14/14 (100) | 0/14 (0) |
| Summed total | 2/409 (0) | 1/409 (0) | 19/409 (5) | 1/409 (0) | 14/409 (3) | 3/409 (1) | 5/409 (1) | 1/409 (0) | 135/409 (33) | 102/409 (25) | 5/409 (1) | 8/409 (2) | 19/409 (5) | 4/409 (1) | 14/409 (3) | 23/409 (6) | 5/409 (1) | 1/409 (0) | 2/409 (0) | 17/409 (4) | 1/409 (0) | 3/409 (1) | 2/409 (0) | 2/409 (0) | 1/409 (0) | 1/409 (0) | 16/409 (4) | 2/409 (0) | 352/409 (86) | 94/409 (23) |
| Number of reviews with > 1 covariate in numerator | 1/52 (2) | 1/52 (2) | 6/52 (12) | 1/52 (2) | 11/52 (21) | 2/52 (4) | 1/52 (2) | 1/52 (2) | 42/52 (81) | 36/52 (70) | 1/52 (2) | 3/52 (6) | 13/52 (25) | 3/52 (6) | 6/52 (12) | 12/52 (23) | 4/52 (8) | 1/52 (2) | 2/52 (4) | 12/52 (23) | 1/52 (2) | 3/52 (6) | 1/52 (2) | 1/52 (2) | 1/52 (2) | 1/52 (2) | 14/52 (27) | 2/52 (4) | 51/52 (98) | - |
| Median | 0 | 0 | 0 | 0 | 0 | 0 | 0 | 0 | 29 | 23 | 0 | 0 | 0 | 0 | 0 | 0 | 0 | 0 | 0 | 0 | 0 | 0 | 0 | 0 | 0 | 0 | 0 | 0 | 88 | 18 |
| IQR | 0-0 | 0-0 | 0-0 | 0-0 | 0-0 | 0-0 | 0-0 | 0-0 | 17-46 | 0-44 | 0-0 | 0-0 | 0-2 | 0-0 | 0-0 | 0-0 | 0-0 | 0-0 | 0-0 | 0-0 | 0-0 | 0-0 | 0-0 | 0-0 | 0-0 | 0-0 | 0-8 | 0-0 | 80-100 | 0-43 |
| Range | 0-17 | 0-8 | 0-60 | 0-14 | 0-33 | 0-18 | 0-83 | 0-10 | 0-100 | 0-82 | 0-42 | 0-33 | 0-25 | 0-14 | 0-67 | 0-77 | 0-33 | 0-9 | 0-20 | 0-100 | 0-8 | 0-14 | 0-40 | 0-14 | 0-7 | 0-7 | 0-33 | 0-33 | 0-100 | 0-100 |

M: meta-regression; NR: not reported; SE: sensitivity analysis; SU: subgroup analysis; U: unnamed analysis. IQR: inter-quartile range.
