# Supplementary material for: Exploring Treatment by Covariate Interactions Using Subgroup Analysis and Meta-Regression in Cochrane Reviews: A Review of Recent Practice
Source: PLoS One. 2015 Jun 1;10(6):e0128804. doi: 10.1371/journal.pone.0128804 (PMC4452239; doi:10.1371/journal.pone.0128804)
Supplement: S9 Table — (DOCX) [file pone.0128804.s011.docx]

**Table S9: Detecting interactions: planned and used methods to detect interactions.**

| **Review** | **Number of covariates with planned and used method (used if did analysis)**  **/Number of covariates reported (%)** | **Number of covariates with planned method (but did no analysis)**  **/Number of covariates reported (%)** | **Number of covariates that planned interaction tests or overlap of confidence intervals (but did no analysis)**  **/Number of covariates reported (%)** | **Number of covariates that planned visually (e.g. box plots), subgroup analyses, meta-regression (but did no analysis)**  **/Number of covariates reported (%)** | **Number of covariates that planned overlap of confidence intervals and test for subgroup differences (but did no analysis)**  **/Number of covariates reported (%)** | **Number of covariates that planned test for subgroup differences (but did no analysis)**  **/Number of covariates reported (%)** | **Number of covariates that planned t-tests and chi-squared tests (but did no analysis)**  **/Number of covariates reported (%)** | **Number of covariates that planned I-squared statistic (but did no analysis)**  **/Number of covariates reported (%)** | **Number of covariates that planned changes in the results (but did no analysis)**  **/Number of covariates reported (%)** | **Number of covariates without planned method (but did no analysis)**  **/Number of covariates reported (%)** | **Number of covariates that used any method (but no planned method)**  **/Number of analysed covariates (%)** | **Number of covariates that used ‘pre-specified criteria, including a test for interaction’ (but no planned method)**  **/Number of analysed covariates (%)** | **Number of covariates that used comparing results (but no planned method)**  **/Number of analysed covariates (%)** | **Number of covariates without planned method & used method not reported**  **/Number of analysed covariates (%)** |
| --- | --- | --- | --- | --- | --- | --- | --- | --- | --- | --- | --- | --- | --- | --- |
| Aboumarzouk 2012 | 0/6 (0) | 0/6 (0) | 0/6 (0) | 0/6 (0) | 0/6 (0) | 0/6 (0) | 0/6 (0) | 0/6 (0) | 0/6 (0) | 6/6 (100) | 0/0 (-) | 0/0 (-) | 0/0 (-) | 0/0 (-) |
| Almeida 2013 | 3/8 (38) | 3/8 (38) | 0/8 (0) | 0/8 (0) | 3 /8 (38) | 0/8 (0) | 0/8 (0) | 0/8 (0) | 0/8 (0) | 4/8 (50) | 0/1 (0) | 0/1 (0) | 0/1 (0) | 1/1 (100) |
| Basurto Ona 2013 | 0/8 (0) | 0/8 (0) | 0/8 (0) | 0/8 (0) | 0/8 (0) | 0/8 (0) | 0/8 (0) | 0/8 (0) | 0/8 (0) | 7/8 (88) | 0/1 (0) | 0/1 (0) | 0/1 (0) | 1/1 (100) |
| Bellmunt-Montoya 2013 | 0/4 (0) | 0/4 (0) | 0/4 (0) | 0/4 (0) | 0/4 (0) | 0/4 (0) | 0/4 (0) | 0/4 (0) | 0/4 (0) | 3/4 (75) | 0/1 (0) | 0/1 (0) | 0/1 (0) | 1/1 (100) |
| Berlowitz 2013 | 0/6 (0) | 0/6 (0) | 0/6 (0) | 0/6 (0) | 0/6 (0) | 0/6 (0) | 0/6 (0) | 0/6 (0) | 0/6 (0) | 6/6 (100) | 0/0 (-) | 0/0 (-) | 0/0 (-) | 0/0 (-) |
| Boselie 2012 | 0/7 (0) | 0/7 (0) | 0/7 (0) | 0/7 (0) | 0/7 (0) | 0/7 (0) | 0/7 (0) | 0/7 (0) | 0/7 (0) | 3/7 (43) | 0/4 (0) | 0/4 (0) | 0/4 (0) | 4/4 (100) |
| Bruins Slot 2013 | 0/13 (0) | 0/13 (0) | 0/13 (0) | 0/13 (0) | 0/13 (0) | 0/13 (0) | 0/13 (0) | 0/13 (0) | 0/13 (0) | 2/13 (15) | 0/11 (0) | 0/11 (0) | 0/11 (0) | 11/11 (100) |
| Cavalheri, 2013 | 3/7 (43) | 3/7 (43) | 0/7 (0) | 0/7 (0) | 0/7 (0) | 3/7 (43) | 0/7 (0) | 0/7 (0) | 0/7 (0) | 4/7 (57) | 0/0 (-) | 0/0 (-) | 0/0 (-) | 0/0 (-) |
| Chaparro 2013 | 0/10 (0) | 0/10 (0) | 0/10 (0) | 0/10 (0) | 0/10 (0) | 0/10 (0) | 0/10 (0) | 0/10 (0) | 0/10 (0) | 6/10 (60) | 0/4 (0) | 0/4 (0) | 0/4 (0) | 4/4 (100) |
| Cheng 2013 | 4/10 (40) | 4/10 (40) | 0/10 (0) | 0/10 (0) | 0/10 (0) | 0/10 (0) | 0/10 (0) | 0/10 (0) | 4/10 (40) | 5/10 (50) | 0/1 (0) | 0/1 (0) | 0/1 (0) | 1/1 (100) |
| Cruciani 2013 | 0/7 (0) | 0/7 (0) | 0/7 (0) | 0/7 (0) | 0/7 (0) | 0/7 (0) | 0/7 (0) | 0/7 (0) | 0/7 (0) | 3/7 (43) | 0/4 (0) | 0/4 (0) | 0/4 (0) | 4/4 (100) |
| Dashash 2013 | 0/8 (0) | 0/8 (0) | 0/8 (0) | 0/8 (0) | 0/8 (0) | 0/8 (0) | 0/8 (0) | 0/8 (0) | 0/8 (0) | 8/8 (100) | 0/0 (-) | 0/0 (-) | 0/0 (-) | 0/0 (-) |
| Deare 2013 | 0/8 (0) | 0/8 (0) | 0/8 (0) | 0/8 (0) | 0/8 (0) | 0/8 (0) | 0/8 (0) | 0/8 (0) | 0/8 (0) | 4/8 (50) | 0/4 (0) | 0/4 (0) | 0/4 (0) | 4/4 (100) |
| Freak-Poli 2013 | 0/13 (0) | 0/13 (0) | 0/13 (0) | 0/13 (0) | 0/13 (0) | 0/13 (0) | 0/13 (0) | 0/13 (0) | 0/13 (0) | 12/13 (92) | 0/1 (0) | 0/1 (0) | 0/1 (0) | 1/1 (100) |
| Gan, 2013 | 0/8 (0) | 0/8 (0) | 0/8 (0) | 0/8 (0) | 0/8 (0) | 0/8 (0) | 0/8 (0) | 0/8 (0) | 0/8 (0) | 8/8 (100) | 0/0 (-) | 0/0 (-) | 0/0 (-) | 0/0 (-) |
| Gillies 2012 | 0/12 (0) | 0/12 (0) | 0/12 (0) | 0/12 (0) | 0/12 (0) | 0/12 (0) | 0/12 (0) | 0/12 (0) | 0/12 (0) | 7/12 (58) | 0/5 (0) | 0/5 (0) | 0/5 (0) | 5/5 (100) |
| Gois 2013 | 0/11 (0) | 0/11 (0) | 0/11 (0) | 0/11 (0) | 0/11 (0) | 0/11 (0) | 0/11 (0) | 0/11 (0) | 0/11 (0) | 11/11 (100) | 0/0 (-) | 0/0 (-) | 0/0 (-) | 0/0 (-) |
| Goldenberg 2013 | 0/6 (0) | 0/6 (0) | 0/6 (0) | 0/6 (0) | 0/6 (0) | 0/6 (0) | 0/6 (0) | 0/6 (0) | 0/6 (0) | 2/6 (33) | 4/4 (100) | 4/4 (100) | 0/4 (0) | 0/4 (0) |
| Gower 2013 | 0/10 (0) | 0/10 (0) | 0/10 (0) | 0/10 (0) | 0/10 (0) | 0/10 (0) | 0/10 (0) | 0/10 (0) | 0/10 (0) | 8/10 (80) | 0/2 (0) | 0/2 (0) | 0/2 (0) | 2/2 (100) |
| He 2013 | 0/8 (0) | 0/8 (0) | 0/8 (0) | 0/8 (0) | 0/8 (0) | 0/8 (0) | 0/8 (0) | 0/8 (0) | 0/8 (0) | 8/8 (100) | 0/0 (-) | 0/0 (-) | 0/0 (-) | 0/0 (-) |
| Itchaki 2013 | 0/12 (0) | 0/12 (0) | 0/12 (0) | 0/12 (0) | 0/12 (0) | 0/12 (0) | 0/12 (0) | 0/12 (0) | 0/12 (0) | 8/12 (67) | 0/4 (0) | 0/4 (0) | 0/4 (0) | 4/4 (100) |
| Kinnersley 2013 | 5/12 (42) | 5/12 (42) | 0/12 (0) | 5/12 (42) | 0/12 (0) | 0/12 (0) | 0/12 (0) | 0/12 (0) | 0/12 (0) | 7/12 (58) | 0/0 (-) | 0/0 (-) | 0/0 (-) | 0/0 (-) |
| Lawrie 2013 | 0/3 (0) | 0/3 (0) | 0/3 (0) | 0/3 (0) | 0/3 (0) | 0/3 (0) | 0/3 (0) | 0/3 (0) | 0/3 (0) | 2/3 (67) | 0/1 (0) | 0/1 (0) | 0/1 (0) | 1/1 (100) |
| Lee 2013 | 0/6 (0) | 0/6 (0) | 0/6 (0) | 0/6 (0) | 0/6 (0) | 0/6 (0) | 0/6 (0) | 0/6 (0) | 0/6 (0) | 6/6 (100) | 0/0 (-) | 0/0 (-) | 0/0 (-) | 0/0 (-) |
| Li 2013 | 0/6 (0) | 0/6 (0) | 0/6 (0) | 0/6 (0) | 0/6 (0) | 0/6 (0) | 0/6 (0) | 0/6 (0) | 0/6 (0) | 6/6 (100) | 0/0 (-) | 0/0 (-) | 0/0 (-) | 0/0 (-) |
| Liu 2013 | 0/11 (0) | 0/11 (0) | 0/11 (0) | 0/11 (0) | 0/11 (0) | 0/11 (0) | 0/11 (0) | 0/11 (0) | 0/11 (0) | 11/11 (100) | 0/0 (-) | 0/0 (-) | 0/0 (-) | 0/0 (-) |
| Lopez 2013 | 0/2 (0) | 0/2 (0) | 0/2 (0) | 0/2 (0) | 0/2 (0) | 0/2 (0) | 0/2 (0) | 0/2 (0) | 0/2 (0) | 0/2 (0) | 0/2 (0) | 0/2 (0) | 0/2 (0) | 2/2 (100) |
| Marigold 2013 | 0/8 (0) | 0/8 (0) | 0/8 (0) | 0/8 (0) | 0/8 (0) | 0/8 (0) | 0/8 (0) | 0/8 (0) | 0/8 (0) | 8/8 (100) | 0/0 (-) | 0/0 (-) | 0/0 (-) | 0/0 (-) |
| Mocellin 2013 | 0/14 (0) | 0/14 (0) | 0/14 (0) | 0/14 (0) | 0/14 (0) | 0/14 (0) | 0/14 (0) | 0/14 (0) | 0/14 (0) | 8/14 (57) | 0/6 (0) | 0/6 (0) | 0/6 (0) | 6/6 (100) |
| Mutua 2012 | 0/8 (0) | 0/8 (0) | 0/8 (0) | 0/8 (0) | 0/8 (0) | 0/8 (0) | 0/8 (0) | 0/8 (0) | 0/8 (0) | 6/8 (75) | 0/2 (0) | 0/2 (0) | 0/2 (0) | 2/2 (100) |
| Parker 2013 | 0/11 (0) | 0/11 (0) | 0/11 (0) | 0/11 (0) | 0/11 (0) | 0/11 (0) | 0/11 (0) | 0/11 (0) | 0/11 (0) | 11/11 (100) | 0/0 (-) | 0/0 (-) | 0/0 (-) | 0/0 (-) |
| Pega, 2013 | 4/6 (67) | 4/6 (67) | 0/6 (0) | 0/6 (0) | 0/6 (0) | 0/6 (0) | 4/6 (67) | 0/6 (0) | 0/6 (0) | 2/6 (33) | 0/0 (-) | 0/0 (-) | 0/0 (-) | 0/0 (-) |
| Penninga 2013 | 5/5 (100) | 5/5 (100) | 0/5 (0) | 0/5 (0) | 0/5 (0) | 5/5 (100) | 0/5 (0) | 0/5 (0) | 0/5 (0) | 0/5 (0) | 0/0 (-) | 0/0 (-) | 0/0 (-) | 0/0 (-) |
| Peters 2013 | 0/11 (0) | 0/11 (0) | 0/11 (0) | 0/11 (0) | 0/11 (0) | 0/11 (0) | 0/11 (0) | 0/11 (0) | 0/11 (0) | 9/11 (82) | 0/2 (0) | 0/2 (0) | 0/2 (0) | 2/2 (100) |
| Rockers 2013 | 0/2 (0) | 0/2 (0) | 0/2 (0) | 0/2 (0) | 0/2 (0) | 0/2 (0) | 0/2 (0) | 0/2 (0) | 0/2 (0) | 1/2 (50) | 0/1 (0) | 0/1 (0) | 0/1 (0) | 1/1 (100) |
| Sajid, 2012 | 0/4 (0) | 0/4 (0) | 0/4 (0) | 0/4 (0) | 0/4 (0) | 0/4 (0) | 0/4 (0) | 0/4 (0) | 0/4 (0) | 3/4 (75) | 0/1 (0) | 0/1 (0) | 0/1 (0) | 1/1 (100) |
| Sampson 2013 | 0/4 (0) | 0/4 (0) | 0/4 (0) | 0/4 (0) | 0/4 (0) | 0/4 (0) | 0/4 (0) | 0/4 (0) | 0/4 (0) | 1/4 (25) | 0/3 (0) | 0/3 (0) | 0/3 (0) | 3/3 (100) |
| Sanders 2013 | 4/11 (36) | 4/11 (36) | 0/11 (0) | 0/11 (0) | 0/11 (0) | 0/11 (0) | 0/11 (0) | 4/11 (36) | 0/11 (0) | 5/11 (45) | 0/2 (0) | 0/2 (0) | 0/2 (0) | 2/2 (100) |
| Sarai 2013 | 0/10 (0) | 0/10 (0) | 0/10 (0) | 0/10 (0) | 0/10 (0) | 0/10 (0) | 0/10 (0) | 0/10 (0) | 0/10 (0) | 10/10 (100) | 0/0 (-) | 0/0 (-) | 0/0 (-) | 0/0 (-) |
| Schoot 2013 | 0/3 (0) | 0/3 (0) | 0/3 (0) | 0/3 (0) | 0/3 (0) | 0/3 (0) | 0/3 (0) | 0/3 (0) | 0/3 (0) | 1/3 (33) | 1/2 (50) | 0/2 (0) | 1/2 (50) | 1/2 (50) |
| Semple 2013 | 0/6 (0) | 0/6 (0) | 0/6 (0) | 0/6 (0) | 0/6 (0) | 0/6 (0) | 0/6 (0) | 0/6 (0) | 0/6 (0) | 5/6 (83) | 0/1 (0) | 0/1 (0) | 0/1 (0) | 1/1 (100) |
| Sharma 2013 | 0/7 (0) | 0/7 (0) | 0/7 (0) | 0/7 (0) | 0/7 (0) | 0/7 (0) | 0/7 (0) | 0/7 (0) | 0/7 (0) | 4/7 (57) | 0/3 (0) | 0/3 (0) | 0/3 (0) | 3/3 (100) |
| Showell 2013 | 0/12 (0) | 0/12 (0) | 0/12 (0) | 0/12 (0) | 0/12 (0) | 0/12 (0) | 0/12 (0) | 0/12 (0) | 0/12 (0) | 8/12 (67) | 0/4 (0) | 0/4 (0) | 0/4 (0) | 4/4 (100) |
| Stead 2012 | 0/11 (0) | 0/11 (0) | 0/11 (0) | 0/11 (0) | 0/11 (0) | 0/11 (0) | 0/11 (0) | 0/11 (0) | 0/11 (0) | 4/11 (36) | 0/7 (0) | 0/7 (0) | 0/7 (0) | 7/7 (100) |
| Trivedi 2013 | 0/6 (0) | 0/6 (0) | 0/6 (0) | 0/6 (0) | 0/6 (0) | 0/6 (0) | 0/6 (0) | 0/6 (0) | 0/6 (0) | 6/6 (100) | 0/0 (-) | 0/0 (-) | 0/0 (-) | 0/0 (-) |
| Trotti 2012 | 0/5 (0) | 0/5 (0) | 0/5 (0) | 0/5 (0) | 0/5 (0) | 0/5 (0) | 0/5 (0) | 0/5 (0) | 0/5 (0) | 2/5 (40) | 0/3 (0) | 0/3 (0) | 0/3 (0) | 3/3 (100) |
| Van Teeffelen, 2013 | 2/5 (40) | 2/5 (40) | 2/5 (40) | 0/5 (0) | 0/5 (0) | 0/5 (0) | 0/5 (0) | 0/5 (0) | 0/5 (0) | 3/5 (60) | 0/0 (-) | 0/0 (-) | 0/0 (-) | 0/0 (-) |
| van Zuuren 2013 | 0/8 (0) | 0/8 (0) | 0/8 (0) | 0/8 (0) | 0/8 (0) | 0/8 (0) | 0/8 (0) | 0/8 (0) | 0/8 (0) | 7/8 (88) | 0/1 (0) | 0/1 (0) | 0/1 (0) | 1/1 (100) |
| Wakai 2013 | 0/4 (0) | 0/4 (0) | 0/4 (0) | 0/4 (0) | 0/4 (0) | 0/4 (0) | 0/4 (0) | 0/4 (0) | 0/4 (0) | 3/4 (75) | 0/1 (0) | 0/1 (0) | 0/1 (0) | 1/1 (100) |
| Wang 2013 | 0/6 (0) | 0/6 (0) | 0/6 (0) | 0/6 (0) | 0/6 (0) | 0/6 (0) | 0/6 (0) | 0/6 (0) | 0/6 (0) | 5/6 (83) | 0/1 (0) | 0/1 (0) | 0/1 (0) | 1/1 (100) |
| Yue 2013 | 0/6 (0) | 0/6 (0) | 0/6 (0) | 0/6 (0) | 0/6 (0) | 0/6 (0) | 0/6 (0) | 0/6 (0) | 0/6 (0) | 2/6 (33) | 0/4 (0) | 0/4 (0) | 0/4 (0) | 4/4 (100) |
| Ziebell 2013 | 2/14 (14) | 2/14 (14) | 0/14 (0) | 0/14 (0) | 0/14 (0) | 2/14 (14) | 0/14 (0) | 0/14 (0) | 0/14 (0) | 12/14 (86) | 0/0 (-) | 0/0 (-) | 0/0 (-) | 0/0 (-) |
| Summed total | 32/409 (8) | 32/409 (8) | 2/409 (0) | 5/409 (1) | 3/409 (1) | 10/409 (2) | 4/409 (1) | 4/409 (1) | 4/409 (1) | 283/409 (69) | 5/94 (5) | 4/94 (4) | 1/94 (1) | 89/94 (95) |
| Number of reviews with > 1 covariate in numerator | 9/52 (17) | 9/52 (17) | 1/52 (2) | 1/52 (2) | 1/52 (2) | 3/52 (6) | 1/52 (2) | 1/52 (2) | 1/52 (2) | 50/52 (96) | 2/33 (6) | 1/33 (3) | 1/33 (3) | 32/33 (97) |
| Median | 0 | 0 | 0 | 0 | 0 | 0 | 0 | 0 | 0 | 67 | 0 | 0 | 0 | 100 |
| IQR | 0-0 | 0-0 | 0-0 | 0-0 | 0-0 | 0-0 | 0-0 | 0-0 | 0-0 | 49-94 | 0-0 | 0-0 | 0-0 | 100-100 |
| Range | 0-100 | 0-100 | 0-40 | 0-42 | 0-38 | 0-100 | 0-67 | 0-36 | 0-40 | 0-100 | 0-100 | 0-100 | 0-50 | 0-100 |

IQR: inter-quartile range.
