# Supplementary material for: Exploring Treatment by Covariate Interactions Using Subgroup Analysis and Meta-Regression in Cochrane Reviews: A Review of Recent Practice
Source: PLoS One. 2015 Jun 1;10(6):e0128804. doi: 10.1371/journal.pone.0128804 (PMC4452239; doi:10.1371/journal.pone.0128804)
Supplement: S10 Table — (DOCX) [file pone.0128804.s012.docx]

**Table S10: Reporting interaction results: results reported from interaction analysis and from method to detect interactions.**

| **Review** | **Number of covariates with statistical results reported**  **/Number of analysed covariates (%)** | **Number of covariates reporting trial group results, treatment effect & CI for trials. Treatment effect, CI, z statistic, & p-value for MA subtotals & totals /Number of analysed covariates (%)^1^** | **Number of covariates reporting trial group results, treatment effect, & CI for trials. Treatment effect & CI, z statistic, p-value for MA subtotals**  **/Number of analysed covariates (%)^1^** | **Number of covariates reporting event rates, OR, & CI for trials. OR, CI, z-statistic, & p-value for MA subtotals, or % by group with or without % difference & a p-value**  **/Number of analysed covariates (%)** | **Number of covariates reporting event rates, RR, & CI for trials. RR, CI, z statistic, p-value &NNTB or NNTH & CI for MA subtotals**  **/Number of analysed covariates (%)** | **Number of covariates reporting HR, SE, CI & N for trials. HR, CI & p-value for MA subtotals /Number of analysed covariates (%)** | **Number of covariates reporting number of eyes, event rates, RR, &CI for trials /Number of analysed covariates (%)** | **Number of covariates reporting mean, SD & N by group for trials. MD, CI, p-value for trials /Number of analysed covariates (%)** | **Number of covariates reporting event rates, RR, & CI for trials /Number of analysed covariates (%)** | **Number of covariates reporting MD& CI for trials /Number of analysed covariates (%)** | **Number of covariates reporting RD & CI for trials, or text /Number of analysed covariates (%)** | **Number of covariates reporting ANOVA P-value /Number of analysed covariates (%)** | **Number of covariates reporting regression coefficient, CI, & p-value /Number of analysed covariates (%)** | **Number of covariates reporting RR & CI for MA subtotals /Number of analysed covariates (%)** | **Number of covariates reporting RR & CI for MA total /Number of analysed covariates (%)** | **Number of covariates reporting HR, CI & p-value for MA subtotal /Number of analysed covariates (%)** | **Number of covariates with no statistical results (text) /Number of analysed covariates (%)** | **Number of covariate with statistical results reported from method to detect interactions**  **/Number of analysed covariates (%)** | **Number of covariate with results reported from test for subgroup differences /Number of analysed covariates (%)^2^** | **Number of covariate with reported regression coefficient & p-value /Number of analysed covariates (%)** |
| --- | --- | --- | --- | --- | --- | --- | --- | --- | --- | --- | --- | --- | --- | --- | --- | --- | --- | --- | --- | --- |
| Almeida 2013 | 1/1 (100) | 0/1 (0) | 0/1 (0) | 0/1 (0) | 0/1 (0) | 0/1 (0) | 0/1 (0) | 1/1 (100) | 0/1 (0) | 0/1 (0) | 0/1 (0) | 0/1 (0) | 0/1 (0) | 0/1 (0) | 0/1 (0) | 0/1 (0) | 0/1 (0) | 0/1 (0) | 0/1 (0) | 0/1 (0) |
| Basurto Ona 2013 | 1/1 (100) | 0/1 (0) | 1/1 (100) | 0/1 (0) | 0/1 (0) | 0/1 (0) | 0/1 (0) | 0/1 (0) | 0/1 (0) | 0/1 (0) | 0/1 (0) | 0/1 (0) | 0/1 (0) | 0/1 (0) | 0/1 (0) | 0/1 (0) | 0/1 (0) | 0/1 (0) | 0/1 (0) | 0/1 (0) |
| Bellmunt-Montoya 2013 | 1/1 (100) | 0/1 (0) | 0/1 (0) | 0/1 (0) | 1/1 (100) | 0/1 (0) | 0/1 (0) | 0/1 (0) | 0/1 (0) | 0/1 (0) | 0/1 (0) | 0/1 (0) | 0/1 (0) | 0/1 (0) | 0/1 (0) | 0/1 (0) | 0/1 (0) | 0/1 (0) | 0/1 (0) | 0/1 (0) |
| Boselie 2012 | 4/4 (100) | 0/4 (0) | 4/4 (100) | 0/4 (0) | 0/4 (0) | 0/4 (0) | 0/4 (0) | 0/4 (0) | 0/4 (0) | 0/4 (0) | 0/4 (0) | 0/4 (0) | 0/4 (0) | 0/4 (0) | 0/4 (0) | 0/4 (0) | 0/4 (0) | 0/4 (0) | 0/4 (0) | 0/4 (0) |
| Bruins Slot 2013 | 11/11 (100) | 11/11 (100) | 0/11 (0) | 0/11 (0) | 0/11 (0) | 0/11 (0) | 0/11 (0) | 0/11 (0) | 0/11 (0) | 0/11 (0) | 0/11 (0) | 0/11 (0) | 0/11 (0) | 0/11 (0) | 0/11 (0) | 0/11 (0) | 0/11 (0) | 11/11 (100) | 11/11 (100) | 0/11 (0) |
| Chaparro 2013 | 4/4 (100) | 1/4 (25) | 3/4 (75) | 0/4 (0) | 0/4 (0) | 0/4 (0) | 0/4 (0) | 0/4 (0) | 0/4 (0) | 0/4 (0) | 0/4 (0) | 0/4 (0) | 0/4 (0) | 0/4 (0) | 0/4 (0) | 0/4 (0) | 0/4 (0) | 1/4 (25) | 1/4 (25) | 0/4 (0) |
| Cheng 2013 | 1/1 (100) | 0/1 (0) | 1/1 (100) | 0/1 (0) | 0/1 (0) | 0/1 (0) | 0/1 (0) | 0/1 (0) | 0/1 (0) | 0/1 (0) | 0/1 (0) | 0/1 (0) | 0/1 (0) | 0/1 (0) | 0/1 (0) | 0/1 (0) | 0/1 (0) | 0/1 (0) | 0/1 (0) | 0/1 (0) |
| Cruciani 2013 | 3/4 (75) | 1/4 (25) | 1/4 (25) | 0/4 (0) | 0/4 (0) | 0/4 (0) | 0/4 (0) | 0/4 (0) | 0/4 (0) | 0/4 (0) | 0/4 (0) | 0/4 (0) | 0/4 (0) | 1/4 (25) | 0/4 (0) | 0/4 (0) | 1/4 (25) | 2/4 (50) | 2/4 (50) | 0/4 (0) |
| Deare 2013 | 4/4 (100) | 2/4 (50) | 2/4 (50) | 0/4 (0) | 0/4 (0) | 0/4 (0) | 0/4 (0) | 0/4 (0) | 0/4 (0) | 0/4 (0) | 0/4 (0) | 0/4 (0) | 0/4 (0) | 0/4 (0) | 0/4 (0) | 0/4 (0) | 0/4 (0) | 2/4 (50) | 2/4 (50) | 0/4 (0) |
| Freak-Poli 2013 | 1/1 (100) | 0/1 (0) | 0/1 (0) | 0/1 (0) | 0/1 (0) | 0/1 (0) | 0/1 (0) | 0/1 (0) | 0/1 (0) | 1/1 (100) | 0/1 (0) | 0/1 (0) | 0/1 (0) | 0/1 (0) | 0/1 (0) | 0/1 (0) | 0/1 (0) | 0/1 (0) | 0/1 (0) | 0/1 (0) |
| Gillies 2012 | 5/5 (100) | 0/5 (0) | 5/5 (100) | 0/5 (0) | 0/5 (0) | 0/5 (0) | 0/5 (0) | 0/5 (0) | 0/5 (0) | 0/5 (0) | 0/5 (0) | 0/5 (0) | 0/5 (0) | 0/5 (0) | 0/5 (0) | 0/5 (0) | 0/5 (0) | 3/5 (60) | 3/5 (60) | 0/5 (0) |
| Goldenberg 2013 | 4/4 (100) | 4/4 (100) | 0/4 (0) | 0/4 (0) | 0/4 (0) | 0/4 (0) | 0/4 (0) | 0/4 (0) | 0/4 (0) | 0/4 (0) | 0/4 (0) | 0/4 (0) | 0/4 (0) | 0/4 (0) | 0/4 (0) | 0/4 (0) | 0/4 (0) | 4/4 (100) | 4/4 (100) | 0/4 (0) |
| Gower 2013 | 2/2 (100) | 0/2 (0) | 0/2 (0) | 0/2 (0) | 0/2 (0) | 0/2 (0) | 2/2 (100) | 0/2 (0) | 0/2 (0) | 0/2 (0) | 0/2 (0) | 0/2 (0) | 0/2 (0) | 0/2 (0) | 0/2 (0) | 0/2 (0) | 0/2 (0) | 0/2 (0) | 0/2 (0) | 0/2 (0) |
| Itchaki 2013 | 4/4 (100) | 0/4 (0) | 4/4 (100) | 0/4 (0) | 0/4 (0) | 0/4 (0) | 0/4 (0) | 0/4 (0) | 0/4 (0) | 0/4 (0) | 0/4 (0) | 0/4 (0) | 0/4 (0) | 0/4 (0) | 0/4 (0) | 0/4 (0) | 0/4 (0) | 2/4 (50) | 2/4 (50) | 0/4 (0) |
| Lawrie 2013 | 1/1 (100) | 0/1 (0) | 0/1 (0) | 0/1 (0) | 0/1 (0) | 1/1 (100) | 0/1 (0) | 0/1 (0) | 0/1 (0) | 0/1 (0) | 0/1 (0) | 0/1 (0) | 0/1 (0) | 0/1 (0) | 0/1 (0) | 0/1 (0) | 0/1 (0) | 1/1 (100) | 1/1 (100) | 0/1 (0) |
| Lopez 2013 | 2/2 (100) | 0/2 (0) | 1/2 (50) | 1/2 (50) | 0/2 (0) | 0/2 (0) | 0/2 (0) | 0/2 (0) | 0/2 (0) | 0/2 (0) | 0/2 (0) | 0/2 (0) | 0/2 (0) | 0/2 (0) | 0/2 (0) | 0/2 (0) | 0/2 (0) | 1/2 (50) | 1/2 (50) | 0/2 (0) |
| Mocellin 2013 | 6/6 (100) | 0/6 (0) | 0/6 (0) | 0/6 (0) | 0/6 (0) | 0/6 (0) | 0/6 (0) | 0/6 (0) | 0/6 (0) | 0/6 (0) | 0/6 (0) | 0/6 (0) | 2/6 (33) | 0/6 (0) | 0/6 (0) | 4/6 (67) | 0/6 (0) | 2/6 (33) | 0/6 (0) | 2/6 (33) |
| Mutua 2012 | 2/2 (100) | 0/2 (0) | 0/2 (0) | 0/2 (0) | 0/2 (0) | 0/2 (0) | 0/2 (0) | 0/2 (0) | 2/2 (100) | 0/2 (0) | 0/2 (0) | 0/2 (0) | 0/2 (0) | 0/2 (0) | 0/2 (0) | 0/2 (0) | 0/2 (0) | 0/2 (0) | 0/2 (0) | 0/2 (0) |
| Peters 2013 | 2/2 (100) | 0/2 (0) | 2/2 (100) | 0/2 (0) | 0/2 (0) | 0/2 (0) | 0/2 (0) | 0/2 (0) | 0/2 (0) | 0/2 (0) | 0/2 (0) | 0/2 (0) | 0/2 (0) | 0/2 (0) | 0/2 (0) | 0/2 (0) | 0/2 (0) | 0/2 (0) | 0/2 (0) | 0/2 (0) |
| Rockers 2013 | 1/1 (100) | 0/1 (0) | 0/1 (0) | 0/1 (0) | 0/1 (0) | 0/1 (0) | 0/1 (0) | 0/1 (0) | 0/1 (0) | 0/1 (0) | 1/1 (100) | 0/1 (0) | 0/1 (0) | 0/1 (0) | 0/1 (0) | 0/1 (0) | 0/1 (0) | 0/1 (0) | 0/1 (0) | 0/1 (0) |
| Sajid, 2012 | 1/1 (100) | 0/1 (0) | 1/1 (100) | 0/1 (0) | 0/1 (0) | 0/1 (0) | 0/1 (0) | 0/1 (0) | 0/1 (0) | 0/1 (0) | 0/1 (0) | 0/1 (0) | 0/1 (0) | 0/1 (0) | 0/1 (0) | 0/1 (0) | 0/1 (0) | 0/1 (0) | 0/1 (0) | 0/1 (0) |
| Sampson 2013 | 3/3 (100) | 0/3 (0) | 2/3 (67) | 0/3 (0) | 0/3 (0) | 0/3 (0) | 0/3 (0) | 0/3 (0) | 0/3 (0) | 0/3 (0) | 0/3 (0) | 0/3 (0) | 0/3 (0) | 0/3 (0) | 1/3 (33) | 0/3 (0) | 0/3 (0) | 2/3 (67) | 2/3 (67) | 0/3 (0) |
| Sanders 2013 | 2/2 (100) | 0/2 (0) | 1/2 (50) | 0/2 (0) | 0/2 (0) | 0/2 (0) | 0/2 (0) | 0/2 (0) | 0/2 (0) | 0/2 (0) | 0/2 (0) | 0/2 (0) | 0/2 (0) | 1/2 (50) | 0/2 (0) | 0/2 (0) | 0/2 (0) | 0/2 (0) | 0/2 (0) | 0/2 (0) |
| Schoot 2013 | 1/2 (50) | 0/2 (0) | 1/2 (50) | 0/2 (0) | 0/2 (0) | 0/2 (0) | 0/2 (0) | 0/2 (0) | 0/2 (0) | 0/2 (0) | 0/2 (0) | 0/2 (0) | 0/2 (0) | 0/2 (0) | 0/2 (0) | 0/2 (0) | 1/2 (50) | 0/2 (0) | 0/2 (0) | 0/2 (0) |
| Semple 2013 | 1/1 (100) | 0/1 (0) | 1/1 (100) | 0/1 (0) | 0/1 (0) | 0/1 (0) | 0/1 (0) | 0/1 (0) | 0/1 (0) | 0/1 (0) | 0/1 (0) | 0/1 (0) | 0/1 (0) | 0/1 (0) | 0/1 (0) | 0/1 (0) | 0/1 (0) | 0/1 (0) | 0/1 (0) | 0/1 (0) |
| Sharma 2013 | 3/3 (100) | 1/3 (33) | 2/3 (67) | 0/3 (0) | 0/3 (0) | 0/3 (0) | 0/3 (0) | 0/3 (0) | 0/3 (0) | 0/3 (0) | 0/3 (0) | 0/3 (0) | 0/3 (0) | 0/3 (0) | 0/3 (0) | 0/3 (0) | 0/3 (0) | 1/3 (33) | 1/3 (33) | 0/3 (0) |
| Showell 2013 | 4/4 (100) | 0/4 (0) | 4/4 (100) | 0/4 (0) | 0/4 (0) | 0/4 (0) | 0/4 (0) | 0/4 (0) | 0/4 (0) | 0/4 (0) | 0/4 (0) | 0/4 (0) | 0/4 (0) | 0/4 (0) | 0/4 (0) | 0/4 (0) | 0/4 (0) | 1/4 (25) | 1/4 (25) | 0/4 (0) |
| Stead 2012 | 7/7 (100) | 7/7 (100) | 0/7 (0) | 0/7 (0) | 0/7 (0) | 0/7 (0) | 0/7 (0) | 0/7 (0) | 0/7 (0) | 0/7 (0) | 0/7 (0) | 0/7 (0) | 0/7 (0) | 0/7 (0) | 0/7 (0) | 0/7 (0) | 0/7 (0) | 7/7 (100) | 7/7 (100) | 0/7 (0) |
| Trotti 2012 | 2/3 (67) | 0/3 (0) | 2/3 (67) | 0/3 (0) | 0/3 (0) | 0/3 (0) | 0/3 (0) | 0/3 (0) | 0/3 (0) | 0/3 (0) | 0/3 (0) | 0/3 (0) | 0/3 (0) | 0/3 (0) | 0/3 (0) | 0/3 (0) | 1/3 (33) | 0/3 (0) | 0/3 (0) | 0/3 (0) |
| van Zuuren 2013 | 1/1 (100) | 0/1 (0) | 0/1 (0) | 0/1 (0) | 0/1 (0) | 0/1 (0) | 0/1 (0) | 0/1 (0) | 0/1 (0) | 0/1 (0) | 0/1 (0) | 1/1 (100) | 0/1 (0) | 0/1 (0) | 0/1 (0) | 0/1 (0) | 0/1 (0) | 0/1 (0) | 0/1 (0) | 0/1 (0) |
| Wakai 2013 | 1/1 (100) | 0/1 (0) | 0/1 (0) | 0/1 (0) | 0/1 (0) | 0/1 (0) | 0/1 (0) | 0/1 (0) | 0/1 (0) | 1/1 (100) | 0/1 (0) | 0/1 (0) | 0/1 (0) | 0/1 (0) | 0/1 (0) | 0/1 (0) | 0/1 (0) | 0/1 (0) | 0/1 (0) | 0/1 (0) |
| Wang 2013 | 1/1 (100) | 0/1 (0) | 0/1 (0) | 0/1 (0) | 0/1 (0) | 0/1 (0) | 0/1 (0) | 0/1 (0) | 1/1 (100) | 0/1 (0) | 0/1 (0) | 0/1 (0) | 0/1 (0) | 0/1 (0) | 0/1 (0) | 0/1 (0) | 0/1 (0) | 0/1 (0) | 0/1 (0) | 0/1 (0) |
| Yue 2013 | 4/4 (100) | 0/4 (0) | 3/4 (75) | 0/4 (0) | 0/4 (0) | 0/4 (0) | 0/4 (0) | 0/4 (0) | 0/4 (0) | 0/4 (0) | 0/4 (0) | 0/4 (0) | 0/4 (0) | 0/4 (0) | 1/4 (25) | 0/4 (0) | 0/4 (0) | 0/4 (0) | 0/4 (0) | 0/4 (0) |
| Summed total | 89/92 (97) | 27/92 (29) | 39/92 (42) | 1/92 (1) | 1/92 (1) | 1/92 (1) | 2/92 (2) | 1/92 (1) | 3/92 (3) | 2/92 (2) | 1/92 (1) | 1/92 (1) | 2/92 (2) | 2/92 (2) | 2/92 (2) | 4/92 (4) | 3/92 (3) | 40/94 (43) | 38/94 (40) | 2/94 (2) |
| Number of reviews with > 1 covariate in numerator | 33/33 (100) | 7/33 (21) | 19/33 (58) | 1/33 (3) | 1/33 (3) | 1/33 (3) | 1/33 (3) | 1/33 (3) | 2/33 (6) | 2/33 (6) | 1/33 (3) | 1/33 (3) | 1/33 (3) | 2/33 (6) | 2/33 (6) | 1/33 (3) | 3/33 (9) | 14/33 (42) | 13/33 (39) | 1/33 (3) |
| Median | 100 | 0 | 50 | 0 | 0 | 0 | 0 | 0 | 0 | 0 | 0 | 0 | 0 | 0 | 0 | 0 | 0 | 0 | 0 | 0 |
| IQR | 100-100 | 0-0 | 0-100 | 0-0 | 0-0 | 0-0 | 0-0 | 0-0 | 0-0 | 0-0 | 0-0 | 0-0 | 0-0 | 0-0 | 0-0 | 0-0 | 0-0 | 0-50 | 0-50 | 0-0 |
| Range | 50-100 | 0-100 | 0-100 | 0-50 | 0-100 | 0-100 | 0-100 | 0-100 | 0-100 | 0-100 | 0-100 | 0-100 | 0-33 | 0-50 | 0-33 | 0-67 | 0-50 | 0-100 | 0-100 | 0-33 |

CI: confidence interval; HR: hazard ratio; IQR: inter-quartile range; MA: meta-analysis; MD: mean difference; N: number of patients; NNTH: number needed to harm; NNTB: number needed to benefit; OR: odds ratio; RD: risk difference; RR: risk ratio; SD: standard deviation, SMD: standardised mean difference.

^1^Trial group results include event rates or mean, SD and N. Treatment effects include RR, OR, HR, MD, SMD.

^2^The test for subgroup differences involves presented a Chi-square statistic, p-value, and I square statistic.
