# Supplementary material for: Exploring Treatment by Covariate Interactions Using Subgroup Analysis and Meta-Regression in Cochrane Reviews: A Review of Recent Practice
Source: PLoS One. 2015 Jun 1;10(6):e0128804. doi: 10.1371/journal.pone.0128804 (PMC4452239; doi:10.1371/journal.pone.0128804)
Supplement: S11 Table — (DOCX) [file pone.0128804.s013.docx]

**Table S11: Interpreting covariates: Reporting of whether interaction was detected, results from the test for subgroup differences, and discussion of plausibility, confounding and the covariate distribution.**

| **Review** | **Number of covariates reporting whether an interaction was detected**  **/Number of analysed covariates (%)** | **Number of covariates reporting interaction detected**  **/Number of analysed covariates (%)** | **Number of covariates reporting no interaction detected**  **/Number of analysed covariates (%)** | **Number of covariates with no discrepancy between the reported interaction and the result from the test for subgroup differences**  **/Number of analysed covariates (%)** | **Number of covariates reporting there was an interaction & test detected interaction**  **/Number of analysed covariates (%)** | **Number of covariates reporting there was no interaction & test detected no interaction**  **/Number of analysed covariates (%)** | **Number of covariates with unclear discrepancy between the reported interaction and the result from the test for subgroup differences/Number of analysed covariates (%)** | **Number of covariates without reporting & test detected interaction**  **Number of analysed covariates (%)** | **Number of covariates without reporting & test detected no interaction**  **/Number of analysed covariates (%)** | **Number of covariates without reporting & no test result given**  **/Number of analysed covariates (%)** | **Number of covariates reporting there was no interaction but no test result given**  **/Number of analysed covariates (%)** | **Number of covariates with plausibility discussed**  **/Number of analysed covariates (%)** | **Number of covariates with confounding discussed**  **/Number of analysed covariates (%)** | **Number of covariates with the covariate distribution discussed**  **/Number of analysed covariates (%)** |
| --- | --- | --- | --- | --- | --- | --- | --- | --- | --- | --- | --- | --- | --- | --- |
| Almeida 2013 | 0/1 (0) | 0/1 (0) | 0/1 (0) | 0/1 (0) | 0/1 (0) | 0/1 (0) | 1/1 (100) | 0/1 (0) | 0/1 (0) | 1/1 (100) | 0/1 (0) | 0/1 (0) | 0/1 (0) | 1/1 (100) |
| Basurto Ona 2013 | 0/1 (0) | 0/1 (0) | 0/1 (0) | 0/1 (0) | 0/1 (0) | 0/1 (0) | 1/1 (100) | 0/1 (0) | 0/1 (0) | 1/1 (100) | 0/1 (0) | 0/1 (0) | 0/1 (0) | 1/1 (100) |
| Bellmunt-Montoya 2013 | 0/1 (0) | 0/1 (0) | 0/1 (0) | 0/1 (0) | 0/1 (0) | 0/1 (0) | 1/1 (100) | 0/1 (0) | 0/1 (0) | 1/1 (100) | 0/1 (0) | 0/1 (0) | 0/1 (0) | 1/1 (100) |
| Boselie 2012 | 0/4 (0) | 0/4 (0) | 0/4 (0) | 0/4 (0) | 0/4 (0) | 0/4 (0) | 4/4 (100) | 0/4 (0) | 0/4 (0) | 4/4 (100) | 0/4 (0) | 1/4 (25)* | 0/4 (0) | 1/4 (25) |
| Bruins Slot 2013 | 0/11 (0) | 1/11 (9) | 10/11 (91) | 0/11 (0) | 0/11 (0) | 0/11 (0) | 11/11 (100) | 1/11 (9) | 10/11 (91) | 0/11 (0) | 0/11 (0) | 0/11 (0) | 0/11 (0) | 0/11 (0) |
| Chaparro 2013 | 0/4 (0) | 1/4 (25) | 0/4 (0) | 0/4 (0) | 0/4 (0) | 0/4 (0) | 4/4 (100) | 1/4 (25) | 0/4 (0) | 3/4 (75) | 0/4 (0) | 0/4 (0) | 0/4 (0) | 0/4 (0) |
| Cheng 2013 | 0/1 (0) | 0/1 (0) | 0/1 (0) | 0/1 (0) | 0/1 (0) | 0/1 (0) | 1/1 (100) | 0/1 (0) | 0/1 (0) | 1/1 (100) | 0/1 (0) | 0/1 (0) | 0/1 (0) | 0/1 (0) |
| Cruciani 2013 | 2/4 (50) | 1/4 (25) | 1/4 (25) | 0/4 (0) | 0/4 (0) | 0/4 (0) | 4/4 (100) | 1/4 (25) | 1/4 (25) | 0/4 (0) | 2/4 (50) | 0/4 (0) | 0/4 (0) | 0/4 (0) |
| Deare 2013 | 2/4 (50) | 1/4 (25) | 1/4 (25) | 2/4 (50) | 1/4 (25) | 1/4 (25) | 2/4 (50) | 0/4 (0) | 0/4 (0) | 2/4 (50) | 0/4 (0) | 0/4 (0) | 0/4 (0) | 3/4 (75) |
| Freak-Poli 2013 | 0/1 (0) | 0/1 (0) | 0/1 (0) | 0/1 (0) | 0/1 (0) | 0/1 (0) | 1/1 (100) | 0/1 (0) | 0/1 (0) | 1/1 (100) | 0/1 (0) | 0/1 (0) | 0/1 (0) | 1/1 (100) |
| Gillies 2012 | 3/5 (60) | 0/5 (0) | 3/5 (60) | 3/5 (60) | 0/5 (0) | 3/5 (60) | 2/5 (40) | 0/5 (0) | 0/5 (0) | 2/5 (40) | 0/5 (0) | 0/5 (0) | 0/5 (0) | 2/5 (40) |
| Goldenberg 2013 | 3/4 (75) | 1/4 (25) | 3/4 (75) | 3/4 (75) | 1/4 (25) | 2/4 (50) | 1/4 (25) | 0/4 (0) | 1/4 (25) | 0/4 (0) | 0/4 (0) | 1/4 (25)** | 0/4 (0) | 2/4 (50) |
| Gower 2013 | 0/2 (0) | 0/2 (0) | 0/2 (0) | 0/2 (0) | 0/2 (0) | 0/2 (0) | 2/2 (100) | 0/2 (0) | 0/2 (0) | 2/2 (100) | 0/2 (0) | 0/2 (0) | 0/2 (0) | 1/2 (50) |
| Itchaki 2013 | 2/4 (50) | 0/4 (0) | 2/4 (50) | 0/4 (0) | 0/4 (0) | 0/4 (0) | 4/4 (100) | 0/4 (0) | 2/4 (50) | 0/4 (0) | 2/4 (50) | 0/4 (0) | 0/4 (0) | 3/4 (75) |
| Lawrie 2013 | 0/1 (0) | 1/1 (100) | 0/1 (0) | 0/1 (0) | 0/1 (0) | 0/1 (0) | 1/1 (100) | 1/1 (100) | 0/1 (0) | 0/1 (0) | 0/1 (0) | 0/1 (0) | 0/1 (0) | 1/1 (100) |
| Lopez 2013 | 0/2 (0) | 1/2 (50) | 0/2 (0) | 0/2 (0) | 0/2 (0) | 0/2 (0) | 2/2 (100) | 1/2 (50) | 0/2 (0) | 1/2 (50) | 0/2 (0) | 0/2 (0) | 0/2 (0) | 0/2 (0) |
| Mocellin 2013 | 6/6 (100) | 0/6 (0) | 0/6 (0) | 0/6 (0) | 0/6 (0) | 0/6 (0) | 6/6 (100) | 0/6 (0) | 0/6 (0) | 0/6 (0) | 6/6 (100) | 0/6 (0) | 0/6 (0) | 1/6 (17) |
| Mutua 2012 | 0/2 (0) | 0/2 (0) | 0/2 (0) | 0/2 (0) | 0/2 (0) | 0/2 (0) | 2/2 (100) | 0/2 (0) | 0/2 (0) | 2/2 (100) | 0/2 (0) | 0/2 (0) | 0/2 (0) | 2/2 (100) |
| Peters 2013 | 0/2 (0) | 0/2 (0) | 0/2 (0) | 0/2 (0) | 0/2 (0) | 0/2 (0) | 2/2 (100) | 0/2 (0) | 0/2 (0) | 2/2 (100) | 0/2 (0) | 0/2 (0) | 0/2 (0) | 2/2 (100) |
| Rockers 2013 | 0/1 (0) | 0/1 (0) | 0/1 (0) | 0/1 (0) | 0/1 (0) | 0/1 (0) | 1/1 (100) | 0/1 (0) | 0/1 (0) | 1/1 (100) | 0/1 (0) | 0/1 (0) | 0/1 (0) | 0/1 (0) |
| Sajid, 2012 | 0/1 (0) | 0/1 (0) | 0/1 (0) | 0/1 (0) | 0/1 (0) | 0/1 (0) | 1/1 (100) | 0/1 (0) | 0/1 (0) | 1/1 (100) | 0/1 (0) | 0/1 (0) | 0/1 (0) | 1/1 (100) |
| Sampson 2013 | 1/3 (33) | 0/3 (0) | 2/3 (67) | 0/3 (0) | 0/3 (0) | 0/3 (0) | 3/3 (100) | 0/3 (0) | 2/3 (67) | 0/3 (0) | 1/3 (33) | 0/3 (0) | 0/3 (0) | 2/3 (67) |
| Sanders 2013 | 1/2 (50) | 0/2 (0) | 0/2 (0) | 0/2 (0) | 0/2 (0) | 0/2 (0) | 2/2 (100) | 0/2 (0) | 0/2 (0) | 1/2 (50) | 1/2 (50) | 0/2 (0) | 0/2 (0) | 1/2 (50) |
| Schoot 2013 | 1/2 (50) | 0/2 (0) | 0/2 (0) | 0/2 (0) | 0/2 (0) | 0/2 (0) | 2/2 (100) | 0/2 (0) | 0/2 (0) | 1/2 (50) | 1/2 (50) | 0/2 (0) | 0/2 (0) | 1/2 (50) |
| Semple 2013 | 0/1 (0) | 0/1 (0) | 0/1 (0) | 0/1 (0) | 0/1 (0) | 0/1 (0) | 1/1 (100) | 0/1 (0) | 0/1 (0) | 1/1 (100) | 0/1 (0) | 0/1 (0) | 0/1 (0) | 0/1 (0) |
| Sharma 2013 | 0/3 (0) | 0/3 (0) | 1/3 (33) | 0/3 (0) | 0/3 (0) | 0/3 (0) | 3/3 (100) | 0/3 (0) | 1/3 (33) | 2/3 (67) | 0/3 (0) | 0/3 (0) | 0/3 (0) | 2/3 (67) |
| Showell 2013 | 0/4 (0) | 1/4 (25) | 0/4 (0) | 0/4 (0) | 0/4 (0) | 0/4 (0) | 4/4 (100) | 1/4 (25) | 0/4 (0) | 3/4 (75) | 0/4 (0) | 0/4 (0) | 0/4 (0) | 4/4 (100) |
| Stead 2012 | 1/7 (14) | 2/7 (29) | 5/7 (71) | 1/7 (14) | 0/7 (0) | 1/7 (14) | 6/7 (86) | 2/7 (29) | 4/7 (57) | 0/7 (0) | 0/7 (0) | 0/7 (0) | 0/7 (0) | 3/7 (43) |
| Trotti 2012 | 0/3 (0) | 0/3 (0) | 0/3 (0) | 0/3 (0) | 0/3 (0) | 0/3 (0) | 3/3 (100) | 0/3 (0) | 0/3 (0) | 3/3 (100) | 0/3 (0) | 0/3 (0) | 1/3 (33)*** | 2/3 (67) |
| van Zuuren 2013 | 0/1 (0) | 0/1 (0) | 0/1 (0) | 0/1 (0) | 0/1 (0) | 0/1 (0) | 1/1 (100) | 0/1 (0) | 0/1 (0) | 1/1 (100) | 0/1 (0) | 0/1 (0) | 0/1 (0) | 1/1 (100) |
| Wakai 2013 | 0/1 (0) | 0/1 (0) | 0/1 (0) | 0/1 (0) | 0/1 (0) | 0/1 (0) | 1/1 (100) | 0/1 (0) | 0/1 (0) | 1/1 (100) | 0/1 (0) | 0/1 (0) | 0/1 (0) | 1/1 (100) |
| Wang 2013 | 0/1 (0) | 0/1 (0) | 0/1 (0) | 0/1 (0) | 0/1 (0) | 0/1 (0) | 1/1 (100) | 0/1 (0) | 0/1 (0) | 1/1 (100) | 0/1 (0) | 0/1 (0) | 0/1 (0) | 1/1 (100) |
| Yue 2013 | 1/4 (25) | 0/4 (0) | 0/4 (0) | 0/4 (0) | 0/4 (0) | 0/4 (0) | 4/4 (100) | 0/4 (0) | 0/4 (0) | 3/4 (75) | 1/4 (25) | 0/4 (0) | 0/4 (0) | 4/4 (100) |
| Summed totals | 23/94 (24) | 10/94 (11) | 28/94 (30) | 9/94 (10) | 2/94 (2) | 7/94 (7) | 85/94 (91) | 8/94 (9) | 21/94 (22) | 42/94 (45) | 14/94 (15) | 2/94 (2) | 1/94 (1) | 45/94 (48) |
| Number of reviews with > 1 covariate in numerator | 11/33 (33) | 9/33 (27) | 9/33 (27) | 3/33 (9) | 2/33 (6) | 4/33 (12) | 33/33 (100) | 7/33 (21) | 7/33 (21) | 25/33 (76) | 7/33 (21) | 2/33 (6) | 1/33 (3) | 26/33 (79) |
| Median | 0 | 0 | 0 | 0 | 0 | 0 | 100 | 0 | 0 | 75 | 0 | 0 | 0 | 67 |
| IQR | 0-33 | 0-9 | 0-25 | 0-0 | 0-0 | 0-0 | 100-100 | 0-0 | 0-0 | 40-100 | 0-0 | 0-0 | 0-0 | 25-100 |
| Range | 0-100 | 0-100 | 0-91 | 0-75 | 0-25 | 0-60 | 25-100 | 0-100 | 0-91 | 0-100 | 0-100 | 0-25 | 0-33 | 0-100 |

IQR: inter-quartile range.

*‘Therefore this is most likely because of a type II error’ in the results text.

**‘However, using 11 published criteria to evaluate the credibility of the subgroup analysis (Sun 2010), we consider the credibility of this subgroup effect as unlikely. That is, the subgroup effect is based on between study comparisons and is not consistent across studies, the direction of the subgroup effect was not pre-specified and we are unaware of any biological or direct evidence that suggests that *L. acidophilus* + *L. casei* is superior to *Lactobacillus rhamnosus*’ in the results text.

***‘The IV studies measured outcomes at 2-3 weeks and the oral studies at 12 weeks, so heterogeneity caused by difference in time of outcome assessment cannot be separated from difference in method of administration’ in the results text.
