# Supplementary material for: Exploring Treatment by Covariate Interactions Using Subgroup Analysis and Meta-Regression in Cochrane Reviews: A Review of Recent Practice
Source: PLoS One. 2015 Jun 1;10(6):e0128804. doi: 10.1371/journal.pone.0128804 (PMC4452239; doi:10.1371/journal.pone.0128804)
Supplement: S12 Table — (DOCX) [file pone.0128804.s014.docx]

**Table S12: Rationale for covariates (when reported).**

| **Review** | **Covariate** | **Rationale for choosing the covariate** | **Where rationale reported** |
| --- | --- | --- | --- |
| Boselie 2012 | **Protocol:** ‘Presence of myelopathy.’  **Review methods:** Presence of myelopathy’  **Review results:** Not analysed.  **Type:** Patient.  **Covariate summary:** Disease characteristics. | ‘In theory, we can expect different results from a patient suffering solely from myelopathy and a patient suffering solely from radiculopathy (e.g. VAS score for arm pain will usually decrease postoperatively in the radiculopathy group, in contrast with a VAS score for arm pain that will already be low in most patients in the myelopathy group). It has also been postulated that arthroplasty may maintain microtrauma to the spinal cord, although a study comparing arthroplasty and fusion in myelopathy due to degenerative disc disease showed similar results for both treatment modalities (Riew 2008). This could potentially influence results, especially when there are large differences in the percentage of patients with myelopathy between study groups. Therefore we plan to do a subgroup analysis to identify if the presence of myelopathy is a confounding variable.’ in protocol.  ‘In theory, different results could be expected from a patient suffering solely from myelopathy and a patient suffering solely from radiculopathy (e.g. score for arm pain will usually decrease postoperatively in the radiculopathy group, in contrast with a score for arm pain that could already be low in most patients in the myelopathy group). Another possible confounding factor is the possibility of neurological deterioration in the presence of adequate decompression that has been described in about 15% of patients with a myelopathy. It has also been postulated that arthroplasty may maintain microtrauma to the spinal cord, although a study pooling two study populations comparing arthroplasty and fusion inmyelopathy due to degenerative disc disease showed similar results for both treatment modalities (Riew 2008). This could potentially influence results, especially when there are large differences in the percentage of patients with myelopathy between study groups. Therefore, we planned to do a subgroup analysis to identify if the presence of myelopathy was a confounding variable. This could not be performed since none of the studies reported results for the two groups separately.’ in review. | Protocol and review. |
| Boselie 2012 | **Protocol:** ‘Smoking’.  **Review methods: ‘**Smoking’.  **Review results:** Not analysed.  **Type:** Patient.  **Covariate summary:** Demographics. | ‘A negative influence of smoking on fusion rates and patient reported outcomes in spinal fusion surgery has been reported for both lumbar and cervical spine. (Andersen 2001; Arnold 2010; Glassman 2000) This negative influence has not been reported in lumbar disc arthroplasty (Bertagnoli 2006). Therefore, we will do a subgroup analysis to identify if smoking is a possible confounding variable.’ | Protocol and review. |
| Freak-Poli 2013 | **Protocol:** ‘Age (as the probability of maintaining good health diminishes as an individual gets older (AIHW 2008), there may  be differing motivations for participation in pedometer-based workplace health programmes depending on age)’.  **Review methods: ‘**Age (as the probability of maintaining good health diminishes as an individual gets older (AIHW 2008), there may be differing motivations for participation in pedometer-based workplace health programmes depending on age)’.  **Review results:** Not analysed.  **Type:** Patient.  **Covariate summary:** Demographics. | ‘as the probability of maintaining good health diminishes as an individual gets older (AIHW 2008), there may be differing motivations for participation in pedometer-based workplace health programmes depending on age’. | Protocol and review. |
| Gillies 2012 | **Protocol:** Allocation concealment. We judged the following to be two covariates: ‘Sensitivity analyses based on allocation concealment and blinding of outcome measurement will be done as these factors are most associated with a bias in effect size (Moher 1998).’  **Review methods:** Allocation concealment. We judged the following to be two covariates: ‘We carried out sensitivity analyses based on allocation concealment and blinding of outcome measurement as these factors are most associated with a bias in effect size’. Allocation concealment: low versus unclear risk (no high risk studies were identified).’  **Review results:** Not analysed.  **Type:** Methodological.  **Covariate summary:** Allocation concealment. | ‘Sensitivity analyses based on allocation concealment and blinding of outcome measurement will be done as these factors are most associated with a bias in effect size (Moher 1998).’ | Protocol and review. |
| Gillies 2012 | **Protocol:** Blinding. We judged the following to be two covariates: ‘Sensitivity analyses based on allocation concealment and blinding of outcome measurement will be done as these factors are most associated with a bias in effect size (Moher 1998).’  **Review methods:** Blinding. We judged the following to be two covariates: ‘We carried out sensitivity analyses based on allocation concealment and blinding of outcome measurement as these factors are most associated with a bias in effect size’. Blinding: low versus unclear versus high risk.’  **Review results:** ‘low’; ‘unclear’; ‘high’.  **Type:** Methodological.  **Covariate summary:** Blinding. | ‘Sensitivity analyses based on allocation concealment and blinding of outcome measurement will be done as these factors are most associated with a bias in effect size (Moher 1998).’ | Protocol and review. |
| Goldenberg 2013 | **Protocol:** ‘Dose of probiotic.’  **Review methods:** ‘Dosage of probiotic’.  **Review results:** Not analysed.  **Type:** Intervention.  **Covariate summary:** Dose. | ‘with an expected larger effect in trials administering an increased dose (Johnston 2006; Johnston 2011).’ | Review only. |
| Goldenberg 2013 | **Protocol:** Not reported.  **Review methods: ‘**Adult versus pediatric population’.  **Review results:** ‘Adult studies’ versus ‘pediatric studies’.  **Type:** Patient.  **Covariate summary:** Demographics. | ‘with a postulated larger effect in adults for CDAD and children for AAD (Hempel 2012);’ | Review only. |
| Goldenberg 2013 | **Protocol:** Not reported.  **Review methods:** ‘Inpatients versus outpatients’.  **Review results: ‘**Inpatient’; ‘outpatient’; ‘mixed’.  **Type:** Patient.  **Covariate summary:** Setting. | ‘with a postulated larger effect among inpatients’ | Review only. |
| Goldenberg 2013 | **Protocol:** Not reported.  **Review methods: ‘**Probiotic species’.  **Review results: ‘**Lactobacillus GG; S. boulardii; L. acidophilus + L. Casei’; ‘L. acidophilus + B. Bifidum L. Acidophilus’; ‘L. acidophilus + L. bulgaricus + B. bifidum + S. Thermophiles’; ‘B. breve + B. Longum +B. infantis + L. acidophilus +L. plantarum + L. paracasei +L. bulgaricus + S. Thermophiles’; ‘L. casei + L. bulgaris + S. Thermophiles’; ‘L. Plantarum’; ‘Lactobacillus GG + L. acidophilus + B. Animalis’.  ‘Lactobacillus GG’ versus ‘ S. boulardii’.  ‘Lactobacillus GG’ versus ‘L. acidophilus + L. Casei’.  **Type:** Intervention.  **Covariate summary:** Type of intervention or control. | ‘with a larger effect expected in trials of S. boulardii or L. rhamnosus (Johnston 2011).’ | Review only. |
| Goldenberg 2013 | **Protocol:** Not reported.  **Review methods: ‘**The risk of bias’.  **Review results:** Risk of bias. ‘Low risk of bias’; ‘high or unclear risk of bias’.  **Type:** Methodological.  **Covariate summary:** Risk of bias. | ‘with an expected larger effect in trials at high or unclear risk of bias versus trials at low risk of bias (Higgins 2011).’ | Review only. |
| **Itchaki 2013** | **Protocol:** ‘Allocation concealment’.  **Review methods: ‘**Allocation concealment’.  **Review results: ‘**Adequate’ versus ‘unclear’.  **Type:** Methodological.  **Covariate summary:** Allocation concealment. | ‘Allocation concealment and blinding (see above), which have been shown to affect subjective outcomes most strongly (Wood 2008).’ in protocol.  ‘Allocation concealment (see above), which has been shown to affect subjective outcomes most strongly (Wood 2008).’ in review. | Protocol and review. |
| **Itchaki 2013** | **Protocol:** ‘Blinding’.  **Review methods: ‘**Blinding’.  **Review results:** Not analysed.  **Type:** Methodological.  **Covariate summary:** Blinding. | ‘Allocation concealment and blinding (see above), which have been shown to affect subjective outcomes most strongly (Wood 2008).’ | Protocol only. |
| Kinnersley 2013 | **Protocol:** ‘Age of patients (young 16 to 35; middle aged 36 to 60; older 61 to 80, elderly 81+)’.  **Review methods: ‘**Age of patients (young 16 to 35; middle aged 36 to 60 years; older 61 to 80, elderly over 80 years)’.  **Review results:** Not analysed.  **Type:** Patient.  **Covariate summary:** Demographics. | ‘Several features of the interaction between patient and clinician may affect communication, the interaction, and thus the opportunity to achieve informed consent for procedures. We plan to make the following comparisons:’. | Protocol and review. |
| Kinnersley 2013 | **Protocol:** ‘Face to face interventions versus distant interventions (for example web-based)’.  **Review methods: ‘**Face to face interventions versus distant interventions (for example web-based)’.  **Review results:** Not analysed.  **Type:** Intervention.  **Covariate summary:** Type of intervention or control. | ‘Several features of the interaction between patient and clinician may affect communication, the interaction, and thus the opportunity to achieve informed consent for procedures. We plan to make the following comparisons:’. | Protocol and review. |
| Kinnersley 2013 | **Protocol:** ‘Interventions targeted at a condition. We judged the following to be two covariates: Interventions targeted at a specific procedure (i. e. whether to undergo, for example, an operation such as knee replacement for osteoarthritis) or at a condition more generally (but for which at least one option may be surgical (e. g. a decision aid addressing menorrhagia)).’  **Review methods: ‘**Interventions targeted at a condition. We judged the following to be two covariates: Interventions targeted at a specific procedure (i. e. whether to undergo, for example, an operation such as knee replacement for osteoarthritis) or at a condition more generally (but for which at least one option may be surgical (e. g. a decision aid addressing menorrhagia)).’  **Review results:** Not analysed.  **Type:** Intervention.  **Covariate summary:** Type of intervention or control. | ‘Several features of the interaction between patient and clinician may affect communication, the interaction, and thus the opportunity to achieve informed consent for procedures. We plan to make the following comparisons:’. | Protocol and review. |
| Kinnersley 2013 | **Protocol:** ‘Interventions targeted at a specific procedure. We judged the following to be two covariates: Interventions targeted at a specific procedure (i. e. whether to undergo, for example, an operation such as knee replacement for osteoarthritis) or at a condition more generally (but for which at least one option may be surgical (e. g. a decision aid addressing menorrhagia)).’  **Review methods: ‘**Interventions targeted at a specific procedure. We judged the following to be two covariates: Interventions targeted at a specific procedure (i. e. whether to undergo, for example, an operation such as knee replacement for osteoarthritis) or at a condition more generally (but for which at least one option may be surgical (e. g. a decision aid addressing menorrhagia)).’  **Review results:** Not analysed.  **Type:** Intervention.  **Covariate summary:** Type of intervention or control. | ‘Several features of the interaction between patient and clinician may affect communication, the interaction, and thus the opportunity to achieve informed consent for procedures. We plan to make the following comparisons:’. | Protocol and review. |
| Kinnersley 2013 | **Protocol:** ‘Interventions targeted at clinicians versus those targeted at patients or at organisational change.’  **Review methods: ‘**Interventions targeted at clinicians versus those targeted at patients or at organisational change.’  **Review results:** Not analysed.  **Type:** Intervention.  **Covariate summary:** Type of intervention or control. | ‘Several features of the interaction between patient and clinician may affect communication, the interaction, and thus the opportunity to achieve informed consent for procedures. We plan to make the following comparisons:’. | Protocol and review. |
| Lee 2013 | **Protocol:** ‘PEP devices and ‘conventional’ ACTs: due to a significant difference in the proposed physiological rationale underpinning their action.’  **Review methods:** ‘PEP devices: ACTs that use PEP may have differing physiological effects and outcomes compared to ACTs not using PEP.’  **Review results:** Not analysed.  **Type:** Intervention.  **Covariate summary:** Type of intervention or control. | ‘due to a significant difference in the proposed physiological rationale underpinning their action.’ ‘ACTs that use PEP may have differing physiological effects and outcomes compared to ACTs not using PEP.’ | Protocol and review. |
| Parker 2013 | **Protocol:** ‘Outcomes will be assessed immediately post intervention, for the short-term (up to one year post-intervention) and long term (over one year post-intervention).’ ‘Multiple time points. Studies of the effectiveness of psychotherapeutic interventions sometimes measure outcomes at multiple time points post-intervention. Ideally time points for assessing the impacts of treatments would be taken from randomisation, but given the likely variability in the duration of interventions between studies we have chosen to group measurements into those taken immediately post intervention, those at short-term follow-up (up to one year post intervention) and those at long-term follow-up (the final measure, greater than one year post-intervention).’  **Review methods:** ‘Outcomes were planned to be assessed immediately post intervention, for the short-term (up to one year post-intervention) and long term (over one year post-intervention).’ ‘Multiple time points. Studies of the effectiveness of psychotherapeutic interventions sometimes measure outcomes at multiple time points post-intervention. Ideally time points for assessing the impacts of treatments would be taken from randomisation, but given the likely variability in the duration of interventions between studies we have chosen to group measurements into those taken immediately post intervention, those at short-term follow-up (up to one year post intervention) and those at long-term follow-up (the final measure, greater than one year post-intervention).’  **Review results:** Not analysed.  **Type:** Outcome.  **Covariate summary:** Time point. | ‘Studies of the effectiveness of psychotherapeutic interventions sometimes measure outcomes at multiple time points post-intervention. Ideally time points for assessing the impacts of treatments would be taken from randomisation, but given the likely variability in the duration of interventions between studies we have chosen to group measurements....’ | Protocol and review. |
| Pega 2013 | **Protocol:** ‘Family type (one-parent family, two-parent family).’  **Review methods: ‘**Family type (one-parent family, two-parent family).’  **Review results:** Not analysed.  **Type:** Patient.  **Covariate summary:** Demographics. | ‘The rationale for conducting these subgroup analyses is that the impact of the intervention might significantly differ by populations defined by these key dimensions. For example, the subgroup analyses by ethnicity will enable an assessment of difference in treatment effect size between different ethnic groups, such as Indigenous versus non-indigenous groups, which will provide an important equity perspective.’ | Protocol only. |
| Pega 2013 | **Protocol:** ‘Gender (female, male)’.  **Review methods: ‘**Gender (female, male)’.  **Review results:** Not analysed.  **Type:** Patient.  **Covariate summary:** Demographics. | ‘The rationale for conducting these subgroup analyses is that the impact of the intervention might significantly differ by populations defined by these key dimensions. For example, the subgroup analyses by ethnicity will enable an assessment of difference in treatment effect size between different ethnic groups, such as Indigenous versus non-indigenous groups, which will provide an important equity perspective.’ | Protocol only. |
| Pega 2013 | **Protocol:** ‘Income (for example, after-tax personal income or family income)’.  **Review methods: ‘**Income (for example, after-tax personal income or family income)’.  **Review results:** Not analysed.  **Type:** Patient.  **Covariate summary:** Demographics. | ‘The rationale for conducting these subgroup analyses is that the impact of the intervention might significantly differ by populations defined by these key dimensions. For example, the subgroup analyses by ethnicity will enable an assessment of difference in treatment effect size between different ethnic groups, such as Indigenous versus non-indigenous groups, which will provide an important equity perspective.’ | Protocol only. |
| Pega 2013 | **Protocol:** ‘Participant ethnicity’.  **Review methods: ‘**Ethnicity’.  **Review results:** Not analysed.  **Type:** Patient.  **Covariate summary:** Demographics. | ‘The rationale for conducting these subgroup analyses is that the impact of the intervention might significantly differ by populations defined by these key dimensions. For example, the subgroup analyses by ethnicity will enable an assessment of difference in treatment effect size between different ethnic groups, such as Indigenous versus non-indigenous groups, which will provide an important equity perspective.’ | Protocol only. |
| Penninga 2013 | **Protocol:** ‘Adult compared to both adult and paediatric studies, as differences in immunology in paediatric patients might be  expected (Aurora 2009; Christie 2009).’  **Review methods:** ‘Adult compared with paediatric studies. This was planned because immunological differences in paediatric patients might be expected (Aurora 2009; Christie 2009).’  **Review results:** Not analysed.  **Type:** Patient.  **Covariate summary:** Demographics. | ‘differences in immunology in paediatric patients might be expected (Aurora 2009; Christie 2009).’ | Protocol and review. |
| Penninga 2013 | **Protocol:** ‘C2-monitoring (2 hours post-dose monitoring) of cyclosporin compared to cyclosporin dosing based on of trough levels, as better outcome has been reported for C2-monitoring of cyclosporin (Iversen 2009).’  **Review methods: ‘**Tacrolimus versus two hours post-dose monitoring of cyclosporin (C2-monitoring) compared with tacrolimus versus cyclosporin dosing based on trough levels (C0-monitoring). This was planned to investigate reports of better outcomes for two hours post-dose monitoring of cyclosporin (Iversen 2009a)’.  **Review results:** Not analysed.  **Type:** Intervention.  **Covariate summary:** Type of intervention or control. | ‘as better outcome has been reported for C2-monitoring of cyclosporin (Iversen 2009).’ | Protocol and review. |
| Penninga 2013 | **Protocol:** ‘Oil-based cyclosporin compared to micro emulsion cyclosporin studies, as differences in absorption and oral bioavailability of the two formulas has been described (Lee 1998; Cantarovich 2004; Kahan 2004; Penninga 2010).’  **Review methods:** ‘Tacrolimus versus oil-based cyclosporin compared with tacrolimus versus microemulsion cyclosporin studies. This subgroup analysis was planned because differences in absorption and oral bioavailability of the two formulae have been described (Cantarovich 2004; Kahan 2004; Lee 1998; Penninga 2010a).’  **Review results:** Not analysed.  **Type:** Intervention.  **Covariate summary:** Type of intervention or control. | ‘differences in absorption and oral bioavailability of the two formulas has been described (Lee 1998; Cantarovich 2004; Kahan 2004; Penninga 2010).’ | Protocol and review. |
| Penninga 2013 | **Protocol: ‘**Single compared to double lung transplant patients, as differences might be expected (Christie 2009).’  **Review methods:** ‘Single compared with double lung transplant patients. Subgroup analysis was planned because we anticipated differences between these populations (Christie 2009).’  **Review results:** Not analysed.  **Type:** Patient.  **Covariate summary:** Disease characteristics. | ‘we anticipated differences between these populations (Christie 2009).’ | Protocol and review. |
| Peters 2013 | **Protocol:** Allocation concealment. We judged the following to be three covariates: **‘**Methodological quality of trials. Sensitivity analyses will be performed when (i) allocation concealment is rated as inadequate, not used or unclear (and attempts to clarify with authors fail) (A); (ii) blinding of outcome assessment is not done or is rated as unclear (and attempts to clarify with authors fail) (B); and (iii) intention-to-treat analysis is not performed or is unclear (and attempts to clarify with authors fail) (C). These quality criteria have been shown to influence estimates of treatment effect (Juni 2001). Sensitivity analyses will be performed in which A, B or C are excluded.’  **Review methods:** We judged the following to be three covariates: ‘We used predetermined sensitivity analyses to assess the effect of excluding studies when (a) allocation concealment was rated as inadequate, not used or unclear (and attempts to clarify with authors failed); (b) blinding of outcome assessment was not done (reason) or was rated as unclear (and attempts to clarify with authors failed); and (c) intention-to-treat analysis was not performed or was unclear (and attempts to clarify with authors failed). These quality criteria have been shown to influence estimates of treatment effects (Jüni 2001).’  **Review results:** Not analysed.  **Type:** Methodological.  **Covariate summary:** Allocation concealment. | ‘These quality criteria have been shown to influence estimates of treatment effect (Juni 2001).’ | Protocol and review. |
| Peters 2013 | **Protocol:** Blinding of outcome assessment. We judged the following to be three covariates: **‘**Methodological quality of trials. Sensitivity analyses will be performed when (i) allocation concealment is rated as inadequate, not used or unclear (and attempts to clarify with authors fail) (A); (ii) blinding of outcome assessment is not done or is rated as unclear (and attempts to clarify with authors fail) (B); and (iii) intention-to-treat analysis is not performed or is unclear (and attempts to clarify with authors fail) (C). These quality criteria have been shown to influence estimates of treatment effect (Juni 2001). Sensitivity analyses will be performed in which A, B or C are excluded.’  **Review methods:** We judged the following to be three covariates: ‘We used predetermined sensitivity analyses to assess the effect of excluding studies when (a) allocation concealment was rated as inadequate, not used or unclear (and attempts to clarify with authors failed); (b) blinding of outcome assessment was not done (reason) or was rated as unclear (and attempts to clarify with authors failed); and (c) intention-to-treat analysis was not performed or was unclear (and attempts to clarify with authors failed). These quality criteria have been shown to influence estimates of treatment effects (Jüni 2001).’  **Review results:** Not analysed.  **Type:** Methodological.  **Covariate summary:** Blinding. | ‘These quality criteria have been shown to influence estimates of treatment effect (Juni 2001).’ | Protocol and review. |
| Peters 2013 | **Protocol:** Intention-to-treat analysis. We judged the following to be three covariates: **‘**Methodological quality of trials.Sensitivity analyses will be performed when (i) allocation concealment is rated as inadequate, not used or unclear (and attempts to clarify with authors fail) (A); (ii) blinding of outcome assessment is not done or is rated as unclear (and attempts to clarify with authors fail) (B); and (iii) intention-to-treat analysis is not performed or is unclear (and attempts to clarify with authors fail) (C). These quality criteria have been shown to influence estimates of treatment effect (Juni 2001). Sensitivity analyses will be performed in which A, B or C are excluded.’  **Review methods:** We judged the following to be three covariates: ‘We used predetermined sensitivity analyses to assess the effect of excluding studies when (a) allocation concealment was rated as inadequate, not used or unclear (and attempts to clarify with authors failed); (b) blinding of outcome assessment was not done (reason) or was rated as unclear (and attempts to clarify with authors failed); and (c) intention-to-treat analysis was not performed or was unclear (and attempts to clarify with authors failed). These quality criteria have been shown to influence estimates of treatment effects (Jüni 2001).’  **Review results:** Not analysed.  **Type:** Methodological.  **Covariate summary:** Intention to treat. | ‘These quality criteria have been shown to influence estimates of treatment effect (Juni 2001).’ | Protocol and review. |
| Showell 2013 | **Protocol:** Not reported.  **Review methods: ‘**The following subgroup analysess were carried out: Antioxidants versus control (placebo or no treatment/standard treatment); Antioxidants versus antioxidants or head-to-head stratification by type of antioxidant; and Pentoxifylline versus control (placebo or no treatment/standard treatment).’  **Review results: ‘**Comparison 1. Antioxidant(s) versus placebo or no treatment/standard treatment;’ ‘Comparison 2. Head to head antioxidants’; ‘Comparison 3. Pentoxifylline versus placebo or no treatment/standard care’.  **Type:** Intervention.  **Covariate summary:** Type of intervention or control. | ‘On clinical advice, trials that used folic acid (standard treatment) and those that included a co-intervention (a fertility drug such as clomiphene citrate or metformin) in both arms were analysed in the antioxidant versus placebo or no treatment/standard treatment comparison and not in the head-to-head comparison, as the controls were not considered to be active treatments. Pentoxifylline trials were analysed as a separate comparison as it was not possible to separate the antioxidant effects from the other medical effects of the drug.’ | Review only. |
| Stead 2012 | **Protocol:** ‘We will categorise the intensity of behavioural support in both intervention and control conditions based on two of the categories used in the US Guidelines (Fiore 2008); ‘Total amount of contact time’ (Categories: 1-3, 4-30, 31-90, 91-300, >300 minutes) and ‘Number of person to person sessions’ (Categories: 0*, 1-3*, 4-8, >8 [*guideline used 0-1, 2-3])’. ‘We will use the relative intensity of support (number or duration of contacts) as the main potential feature to explain any heterogeneity.’  **Review methods: ‘**We categorise the intensity of behavioural support in both intervention and control conditions based on two of the categories used in the US Guidelines (Fiore 2008): ‘Total amount of contact time’ (Categories: 0, 1 to 30*, 31 to 90, 91 to 300, > 300 minutes [*guideline categories 1 to 3 and 4-30 combined]) and ‘Number of person-to-person sessions’ (Categories: 0*, 1 to 3*, 4 to 8, > 8 [*guideline used 0 to 1, 2 to 3]).’ ‘We used the difference in intensity of support (number or duration of contacts) between intervention and control conditions as the main potential feature to explain any heterogeneity.’  **Review results:** Subgroups by ‘contrast in number of contacts between intervention & control’. ‘4 to 8 or > 8 contacts versus no contact’; ‘More than 8 contacts versus 1 to 3 contacts’; ‘4 to 8 contacts versus 1 to 3 contacts’; ‘More than 8 contacts versus 4-8 contacts’; ‘Intervention & control in same contact category’).  **Type:** Intervention.  **Covariate summary:** Intervention intensity. | ‘We would expect trials testing larger differences in intensity, that is using longer, more frequent sessions in the intervention condition and brief contact in the control, to show larger treatment effects.’ | Protocol and review. |
| Van Teeffelen, 2013 | **Protocol:** ‘Compare the groups with retainment of amniotic fluid in the treatment group, versus the group with spontaneous re-accumulation in the standard care group, and the group which does not retain amniotic fluid with the group in the standard care group that shows no signs of spontaneous re-accumulation. This is possible if frequent ultrasound monitoring of amniotic fluid volume is done in both groups.’  **Review methods:** ‘Compare the groups with retainment of amniotic fluid in the treatment group, versus the group with spontaneous re-accumulation in the standard care group, and the group which does not retain amniotic fluid with the group in the standard care group that shows no signs of spontaneous re-accumulation. This is possible if frequent ultrasound monitoring of amniotic fluid volume is done in both groups.’  **Review results:** Not analysed.  **Type:** Patient.  **Covariate summary:** Disease characteristics. | ‘In the literature, the incidence of spontaneous re-accumulation of amniotic fluid after PPROM has been reported as 25% (Hadi 1994).The incidence of retainment of transabdominally amnioinfused fluid after PPROMhas been reported by two authors. Tan et al. found in 27 amnioinfused patients retainment of fluid after 48 hours in only 4 cases (24%), whereas this was 30% in 36 patients in a study by Vergani et al. (Tan 2003, Vergani 2004). Hypothetically, the retainment of amnioinfused fluid could be partly caused by nothing more than spontaneous re-accumulation. This could be due to spontaneous resealing of the membrane defect, which occurs anyway in some patients, with or without amnioinfusion. In that case benefit from amnioinfusion would be small or even absent.’ | Protocol and review. |
| Van Teeffelen, 2013 | **Protocol:** ‘Patients from the intervention group who retain amniotic fluid (‘successful amnioinfusion’) versus the standard care group and patients from the intervention group who do not retain amniotic fluid (‘unsuccessful’ amnioinfusion) versus the standard care group.’ ‘Succesful amnioinfusion as defined by study specific criteria for diagnosing oligohydramnios (timing of measurement, cut-off value used for ultrasound assessment of amount of fluid).’  **Review methods:** ‘Patients from the intervention group who retain amniotic fluid (‘successful amnioinfusion’) versus the standard care group and patients from the intervention group who do not retain amniotic fluid (‘unsuccessful’ amnioinfusion) versus the standard care group.’ ‘Succesful amnioinfusion as defined by study specific criteria for diagnosing oligohydramnios (timing of measurement, cut-off value used for ultrasound assessment of amount of fluid).’  **Review results:** Not analysed.  **Type:** Patient.  **Covariate summary:** Disease characteristics. | ‘The reason for this subgroup analysis is to test if even without retainment of amniotic fluid, amnioinfusion is beneficial (by means of dilution and flushing of contaminated material in the womb) compared with standard care.’ | Protocol and review. |
| Wakai 2013 | **Protocol:** ‘Subgroup analysis will performed for the effects of nitrates on the subset of patients with acute heart failure complicating acute myocardial infarction.’  **Review methods:** ‘We performed subgroup analysis for the effects of nitrates on the subset of patients with AHFS complicating acute myocardial infarction.’  **Review results:** Not analysed.  **Type:** Patient.  **Covariate summary:** Disease characteristics. | ‘The effect of nitrates on heart failure complicating acute myocardial infarction is poorly defined. Subgroup analysis will performed for the effects of nitrates on the subset of patients with acute heart failure complicating acutemyocardial infarction.Heart failure complicating myocardial infarction is associated with a 3- 4 fold increase in hospital mortality (Hellermann 2002; Spencer 1999; Spencer 2002; Steg 2004; Wu 2002). Depending on study design, the incidence of heart failure after myocardial infarction varies greatly from 3% to 53% (Ambrosioni 1995; Bueno 1995; Hellermann 2002). Meanwhile, because of their haemodynamically beneficial effects, nitrates are recommended in heart failure (Hunt 2001) and acute coronary syndromes (Peacock 2004), respectively. Intravenous nitrate vasodilators are well tolerated in acutemyocardial infarction with clinically significant hypotension occurs in less than 4% of patients (GISSI-3 1994), but this responds to dose reduction and fluid replacement. However, two megatrials have failed to demonstrate any mortality reduction attributable to nitrate use in acute coronary syndromes (GISSI-3 1994; ISIS-4 1995). The primary reason for the use of nitrates in acute coronary syndromes is a beneficial haemodynamic effect and to decrease the pain of myocardial ischaemia (Peacock 2004).’ | Protocol only. |
| Yue 2013 | **Protocol:** Not reported.  **Review methods:** MRSA subset**.**  **Review results:** MRSA subset**.**  **Type:** Patient.  **Covariate summary:** Disease characteristics. | ‘In the section ‘Subgroup analysis and investigation of heterogeneity’, we added: ‘3. MRSA subset’, because MRSA is important for SSTIs. The morbidity and treatment costs associated with MRSA-infected SSTIs are higher than for other pathogen infections, so we added this subgroup analysis.’ | Review only. |

AAD: antibiotic-associated diarrhoea. ACT: airway clearance technique. AHFS: acute heart failure syndromes. *CDAD: Clostridium difficile*-associated diarrhoea. MRSA: methicillin-resistant *Staphylococcus aureus*. PEP: positive expiratory pressure. PPROM: preterm prelabour rupture of membranes. SSTI: skin and soft tissue infections. VAS: visual analogue scale.
