# Supplementary material for: Exploring Treatment by Covariate Interactions Using Subgroup Analysis and Meta-Regression in Cochrane Reviews: A Review of Recent Practice
Source: PLoS One. 2015 Jun 1;10(6):e0128804. doi: 10.1371/journal.pone.0128804 (PMC4452239; doi:10.1371/journal.pone.0128804)
Supplement: S13 Table — (DOCX) [file pone.0128804.s015.docx]

**Table S13: Justification for covariate categories (when given).**

| **Review** | **Covariate** | **Justification for categories** | **Where justification reported** |
| --- | --- | --- | --- |
| Boselie 2012 | **Protocol:** Not reported.  **Review methods:** ‘Investigated the possibility of a small study effect.’  **Review results:** ‘Small study effect’.  **Type:** Methodological.  **Covariate summary:** Trial size. | ‘Small studies are more likely to remain unpublished,combined with the fact that it may be easier to publish a small study with strong (positive) results this may lead to an overestimation of the treatment effect.’ | Review only. |
| Goldenberg 2013 | **Protocol:** Not reported.  **Review methods: ‘**Adult versus pediatric population’.  **Review results:** ‘Adult studies’ versus ‘pediatric studies’.  **Type:** Patient.  **Covariate summary:** Demographics. | **‘**Adult versus pediatric population, with a postulated larger effect in adults for CDAD and children for AAD (Hempel 2012).’ | Review only. |
| Goldenberg 2013 | **Protocol:** Not reported.  **Review methods:** ‘Inpatients versus outpatients’.  **Review results: ‘**Inpatient’; ‘outpatient’; ‘mixed’.  **Type:** Patient.  **Covariate summary:** Setting. | **‘**In patients versus outpatients, with a postulated larger effect among inpatients’ | Review only. |
| Goldenberg 2013 | **Protocol:** Not reported.  **Review methods: ‘**The risk of bias’.  **Review results:** Risk of bias. ‘Low risk of bias’; ‘high or unclear risk of bias’.  **Type:** Methodological.  **Covariate summary:** Risk of bias. | ‘The risk of bias, with an expected larger effect in trials at high or unclear risk of bias versus trials at low risk of bias (Higgins 2011).’ | Review only. |
| Lee 2013 | **Protocol:** ‘PEP devices and ‘conventional’ ACTs: due to a significant difference in the proposed physiological rationale underpinning their action.’  **Review methods:** ‘PEP devices: ACTs that use PEP may have differing physiological effects and outcomes compared to ACTs not using PEP.’  **Review results:** Not analysed.  **Type:** Intervention.  **Covariate summary:** Type of intervention or control. | ‘due to a significant difference in the proposed physiological rationale underpinning their action.’  ‘ACTs that use PEP may have differing physiological effects and outcomes compared to ACTs not using PEP.’ | Protocol and review. |
| Penninga 2013 | **Protocol:** ‘Adult compared to both adult and paediatric studies, as differences in immunology in paediatric patients might be  expected (Aurora 2009; Christie 2009).’  **Review methods:** ‘Adult compared with paediatric studies. This was planned because immunological differences in paediatric patients might be expected (Aurora 2009; Christie 2009).’  **Review results:** Not analysed.  **Type:** Patient.  **Covariate summary:** Demographics. | ‘differences in immunology in paediatric patients might be expected (Aurora 2009; Christie 2009).’ | Protocol and review. |
| Penninga 2013 | **Protocol:** ‘C2-monitoring (2 hours post-dose monitoring) of cyclosporin compared to cyclosporin dosing based on of trough levels, as better outcome has been reported for C2-monitoring of cyclosporin (Iversen 2009).’  **Review methods: ‘**Tacrolimus versus two hours post-dose monitoring of cyclosporin (C2-monitoring) compared with tacrolimus versus cyclosporin dosing based on trough levels (C0-monitoring). This was planned to investigate reports of better outcomes for two hours post-dose monitoring of cyclosporin (Iversen 2009a)’.  **Review results:** Not analysed.  **Type:** Intervention.  **Covariate summary:** Type of intervention or control. | **‘**as better outcome has been reported for C2-monitoring of cyclosporin (Iversen 2009).’ | Protocol and review. |
| Penninga 2013 | **Protocol:** ‘Oil-based cyclosporin compared to micro emulsion cyclosporin studies, as differences in absorption and oral bioavailability of the two formulas has been described (Lee 1998; Cantarovich 2004; Kahan 2004; Penninga 2010).’  **Review methods:** ‘Tacrolimus versus oil-based cyclosporin compared with tacrolimus versus microemulsion cyclosporin studies. This subgroup analysis was planned because differences in absorption and oral bioavailability of the two formulae have been described (Cantarovich 2004; Kahan 2004; Lee 1998; Penninga 2010a).’  **Review results:** Not analysed.  **Type:** Intervention.  **Covariate summary:** Type of intervention or control. | ‘differences in absorption and oral bioavailability of the two formulas has been described (Lee 1998;Cantarovich 2004; Kahan 2004; Penninga 2010).’ | Protocol and review. |
| Penninga 2013 | **Protocol: ‘**Single compared to double lung transplant patients, as differences might be expected (Christie 2009).’  **Review methods:** ‘Single compared with double lung transplant patients. Subgroup analysis was planned because we anticipated differences between these populations (Christie 2009).’  **Review results:** Not analysed.  **Type:** Patient.  **Covariate summary:** Disease characteristics. | ‘we anticipated differences between these populations (Christie 2009).’ | Protocol and review. |
| Sanders 2013 | **Protocol:** Not reported.  **Review methods:** Not reported.  **Review results:** ‘We excluded the DECREASE III study because of concerns about validity, but we ran sensitivity analyses on its impact on our results’.  **Type:** Other.  **Covariate summary:** Outlying results. | ‘because of concerns about validity’. | Review only. |
| Van Teeffelen, 2013 | **Protocol:** ‘Compare the groups with retainment of amniotic fluid in the treatment group, versus the group with spontaneous re-accumulation in the standard care group, and the group which does not retain amniotic fluid with the group in the standard care group that shows no signs of spontaneous re-accumulation. This is possible if frequent ultrasound monitoring of amniotic fluid volume is done in both groups.’  **Review methods:** ‘Compare the groups with retainment of amniotic fluid in the treatment group, versus the group with spontaneous re-accumulation in the standard care group, and the group which does not retain amniotic fluid with the group in the standard care group that shows no signs of spontaneous re-accumulation. This is possible if frequent ultrasound monitoring of amniotic fluid volume is done in both groups.’  **Review results:** Not analysed.  **Type:** Patient.  **Covariate summary:** Disease characteristics. | ‘In the literature, the incidence of spontaneous re-accumulation of amniotic fluid after PPROM has been reported as 25% (Hadi 1994).The incidence of retainment of transabdominally amnioinfused fluid after PPROMhas been reported by two authors. Tan et al. found in 27 amnioinfused patients retainment of fluid after 48 hours in only 4 cases (24%), whereas this was 30% in 36 patients in a study by Vergani et al. (Tan 2003, Vergani 2004). Hypothetically, the retainment of amnioinfused fluid could be partly caused by nothing more than spontaneous re-accumulation. This could be due to spontaneous resealing of the membrane defect, which occurs anyway in some patients, with or without amnioinfusion. In that case benefit from amnioinfusion would be small or even absent.’ | Protocol and review. |
| Van Teeffelen, 2013 | **Protocol:** ‘Patients from the intervention group who retain amniotic fluid (‘successful amnioinfusion’) versus the standard care group and patients from the intervention group who do not retain amniotic fluid (‘unsuccessful’ amnioinfusion) versus the standard care group.’ ‘Succesful amnioinfusion as defined by study specific criteria for diagnosing oligohydramnios (timing of measurement, cut-off value used for ultrasound assessment of amount of fluid).’  **Review methods:** ‘Patients from the intervention group who retain amniotic fluid (‘successful amnioinfusion’) versus the standard care group and patients from the intervention group who do not retain amniotic fluid (‘unsuccessful’ amnioinfusion) versus the standard care group.’ ‘Succesful amnioinfusion as defined by study specific criteria for diagnosing oligohydramnios (timing of measurement, cut-off value used for ultrasound assessment of amount of fluid).’  **Review results:** Not analysed.  **Type:** Patient.  **Covariate summary:** Disease characteristics. | ‘The reason for this subgroup analysis is to test if even without retainment of amniotic fluid, amnioinfusion is beneficial (by means of dilution and flushing of contaminated material in the womb) compared with standard care.’ | Protocol and review. |
| Yue 2013 | **Protocol:** Not reported.  **Review methods:** MRSA subset**.**  **Review results:** MRSA subset**.**  **Type:** Patient.  **Covariate summary:** Disease characteristics. | ‘The morbidity and treatment costs associated with MRSA-infected SSTIs are higher than for other pathogen infections, so  we added this subgroup analysis.’ | Review only. |

AAD: antibiotic-associated diarrhoea. ACT: airway clearance technique. *CDAD: Clostridium difficile*-associated diarrhoea. MRSA: methicillin-resistant *Staphylococcus aureus*. PEP: positive expiratory pressure. PPROM: preterm prelabour rupture of membranes. SSTI: skin and soft tissue infections.
